# Identification of senescence-related biomarker for aortic dissection based on bioinformatics and machine learning algorithms

**DOI:** 10.1097/MD.0000000000048873

**Published:** 2026-05-29

**Authors:** Jiabao Zhong, Hongli Luo

**Affiliations:** a Department of Cardiac Surgery, The First Hospital of China Medical University, Shenyang, Liaoning Province, China.

**Keywords:** aortic dissection, diagnosis and treatment, infiltrating immune cells, machine learning, senescence

## Abstract

Aortic dissection (AD) is a vascular surgical disease that seriously threatens human health. Due to a high misdiagnosis rate and unclear pathogenesis, it brings greater challenges to AD patients and vascular surgeons. This study aimed to explore sensitive diagnostic markers and potential therapeutic targets of AD from the perspective of cellular senescence, which has been our long-term concern. We downloaded the expression matrix of AD and control samples from the Gene Expression Omnibus database and obtained the senescence-related gene set. Differentially expressed genes (DEGs) in AD and control groups were analyzed by the “limma” package. Gene Ontology, Disease Ontology, and Kyoto Encyclopedia of Genes and Genomes enrichment analyses were carried out to demonstrate the enrichment function of DEGs. Two sensitive screening diagnostic marker algorithms, the least absolute shrinkage and selection operator and support vector machine–recursive feature elimination, were used to identify target genes in combination with senescence-related genes. Receiver operating characteristic curves for targeted genes were plotted to assess diagnostic efficacy. Gene set enrichment analysis was performed to preliminarily explore the pathways enriched in the 2 groups. CIBERSORT was used to look for differentially infiltrating immune cells, and Spearman analysis was carried out to explore the association between targeted genes and infiltrating immune cells. A total of 111 DEGs were identified, which were closely related to regulation of leukocyte migration, cellular transition metal ion homeostasis, transition metal ion homeostasis, and detoxification of copper ion from Gene Ontology and Kyoto Encyclopedia of Genes and Genomes analysis. Disease Ontology analysis found that these DEGs were mainly involved in acute myocardial infarction, vasculitis, and coronary artery disease. Hexokinase-3 (HK3) was identified as an overlapping gene of least absolute shrinkage and selection operator, support vector machine–recursive feature elimination, and senescence. HK3 showed good diagnostic efficacy (area under the receiver operating characteristic curve = 0.924 in the integrated cohort and 0.914 in the validation cohort). Correlation analysis results showed that HK3 was positively correlated with monocytes, neutrophils, and natural killer cells resting, while B cells naive, mast cells resting, and macrophages M1 were negatively correlated. HK3 was a potential biomarker for AD diagnosis and a target for precision therapy, and HK3 is also closely related to immune infiltration.

## 1. Introduction

Aortic dissection (AD) is the most catastrophic aortic disease, as the aortic intima ruptures and circulating blood components enter the aortic wall, and the intima and adventitia are separated and form the true and false lumen.^[1,2]^ AD is quite dangerous, often leading to rupture, massive bleeding, and insufficient organ blood supply.^[3]^ The in-hospital mortality rate of AD is as high as 20% to 22%,^[4]^ and the annual incidence rate is 3 to 5 per 100,000 people.^[5]^ The risk is highest among people between 50 and 70 years old, which means increasing age is an important factor related to the long-term mortality of AD.^[6]^ Although reports show that the diagnostic technology and treatment methods of AD are constantly improving and updating, the misdiagnosis and mortality rates are still high. Early identification and targeted therapy of AD are necessary and urgent.^[7,8]^

For the past few years, microarray technology and bioinformatics analysis have been jointly applied to screen a variety of new cardiovascular-related genes, which may be used as biomarkers for early diagnosis. At the same time, the concept of machine learning algorithms as the core has been gradually cited and popularized.^[9]^ In one study, the least absolute shrinkage and selection operator (LASSO) algorithm was used to find that SLC2A3, CLEC4D, GPR97, PLAUR, and BST1 had a good predictive effect on ST-segment elevation heart failure.^[10]^ In another study, 3 metabolism-related genes, AKR1C3, GLUL, and BST1, were identified by the support vector machine–recursive feature elimination (SVM-RFE) algorithm as a diagnostic and therapeutic biomarker for acute myocardial infarction.^[11]^ There was also a study that combined multiple algorithms to identify diagnostic markers of atherosclerosis.^[12]^ However, relevant research on exploring potential biological markers of AD based on machine learning is still insufficient.

Vascular wall homeostasis and remodeling are important links in the development and prognosis of AD. Vascular media structural changes, such as vascular smooth muscle cells (VSMCs) loss and elastic fiber breakage, play an important role in the pathogenesis of AD.^[5,13]^ Notably, cellular senescence has emerged as a key driver of VSMCs dysfunction and vascular wall degeneration – senescent VSMCs lose their proliferative and contractile capacities, secrete proinflammatory cytokines, and accelerate extracellular matrix degradation, all of which disrupt vascular homeostasis.^[14,15]^ Senescence-related genes, which regulate the senescence process through pathways such as p53 and janus kinase–signal transducer and activator of transcription (JAK–STAT),^[16,17]^ have been implicated in various cardiovascular diseases, including abdominal aortic aneurysm and atherosclerosis.^[14,18]^ Moreover, cellular senescence is also a major risk factor for cardiovascular health impairment, and anti-senescence treatments are also being actively explored.^[15]^ Therefore, we aimed to explore and analyze the specific markers and potential therapeutic targets of AD based on cellular senescence.

## 2. Materials and methods

### 2.1. Data download and processing

Publicly available transcriptome datasets related to AD, including GSE98770, GSE153434, and GSE147026, were downloaded from the Gene Expression Omnibus database (http://www.ncbi.nlm.nih.gov/geo/) and used as the discovery cohort. GSE52093 was used as an independent external validation dataset. Sample grouping (AD vs control) was defined strictly according to the original Gene Expression Omnibus series annotations and corresponding platform files. Only samples with explicit disease status and expression matrix information were included in the downstream analyses. No additional patient recruitment or reclassification was performed in this study. Detailed information for each dataset, including accession number, sample type, platform, and sample size, is provided in Supplementary File 1, Supplemental Digital Content.

Before dataset integration, probe identifiers were converted to gene symbols according to the corresponding platform annotation files. When multiple probes mapped to the same gene, the average expression value was used as the representative value. The 3 discovery datasets were then merged, and batch effects caused by different platforms or experimental sources were corrected using the “SVA” package in R (R Foundation for Statistical Computing, Vienna, Austria) . Principal component analysis before and after batch correction was used to visually assess the effectiveness of normalization and batch-effect removal. In addition, 279 senescence-related genes were downloaded from the CellAge database (https://genomics.senescence.info/cells/) and are listed in Supplementary File 2, Supplemental Digital Content.^[19]^

### 2.2. Difference analysis and enrichment analysis

Differentially expressed genes (DEGs) between AD and control samples were identified using the “limma” package in R (R Foundation for Statistical Computing). To improve analytical transparency and reproducibility, the thresholds were predefined as adjusted false discovery rate < 0.05 and |log2 fold change| > 2. Volcano plots and heatmaps were generated using the “ggplot2” and “pheatmap” packages (R Foundation for Statistical Computing), respectively. Functional annotation of DEGs was subsequently performed using the “clusterProfiler” package, including Gene Ontology (GO), Kyoto Encyclopedia of Genes and Genomes, and Disease Ontology enrichment analyses.

### 2.3. Candidate diagnostic biomarker screening

To reduce the risk of model instability associated with a single feature-selection strategy, 2 machine learning algorithms were applied in parallel to screen candidate diagnostic biomarkers. LASSO regression was implemented using the “glmnet” package (R Foundation for Statistical Computing), and SVM-RFE was used as a complementary supervised learning method.^[20,21]^

### 2.4. Gene set enrichment analysis (GSEA)

GSEA was performed to elucidate the biological significance of feature biomarkers,^[22]^ and the “c2.cp.kegg.v7.4.symbols” genome from the Molecular Signature Database (MSigDB, http://software.broadinstitute.org/gsea/msigdb/) was used as a reference set.

### 2.5. Diagnostic value of identified biomarkers

To assess the predictive power of the targeted genes, receiver operating characteristic (ROC) curves were drawn using matrices from 21 AD and 19 control samples in the integrated dataset. The area under the ROC curve (AUC) was analyzed to assess the diagnostic validity for distinguishing the 2 groups and further validated in GSE52093, containing 7 AD patients and 5 healthy controls.

### 2.6. Analysis of immune cell infiltration

CIBERSORT (https://cibersortx.stanford.edu/) was carried out to quantify the relative abundance of the 22 immune cell types in each dataset.^[23]^ Histograms and violin plots were plotted using the “corrplot” and “vioplot” packages to visualize the differences in immune cells in AD and control groups. In addition, Spearman correlation analysis was performed between the proportions of immune cells and the expression levels of targeted biomarkers.

The R packages used for data analysis are provided as supplemental material in Supplementary File 3, Supplemental Digital Content.

### 2.7. Statistical analysis

All statistical tests were implemented using RStudio software 4.1.1 (R Foundation for Statistical Computing). The Wilcoxon or Student *t* test was used to analyze differences between the AD and control groups. The Spearman correlation test was performed to determine correlations between variables. *P* values were corrected by the Benjamini test, and *P* < .05 was considered statistically significant.

## 3. Results

### 3.1. A number of 111 DEGs were identified and the enrichment result of DEGs

**Figure 1.**
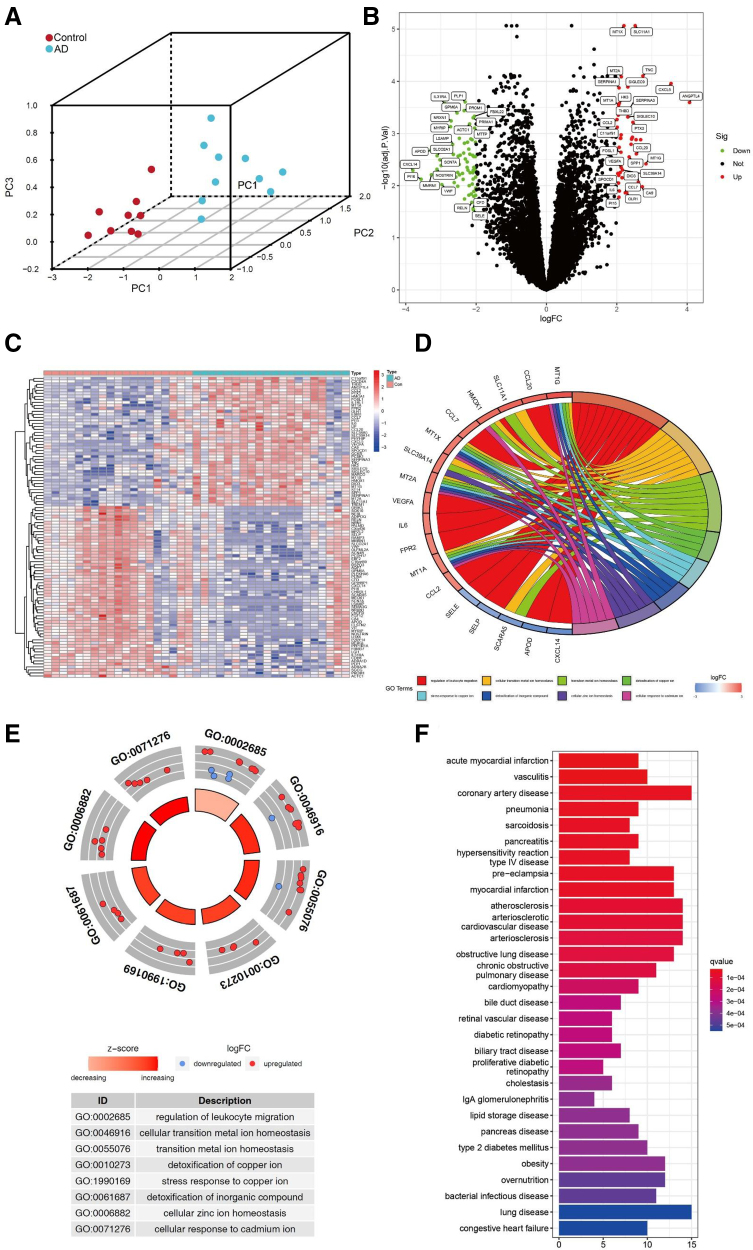
Screening and analysis of DEGs between AD tissues and control tissues. (A) PCA plots showed the batch effect correction result. (B, C) DEGs were visualized by volcano map and heatmap. (D–F) GO, KEGG, and DO analyses were performed for DEGs. AD = aortic dissection, DEGs = differentially expressed genes, DO = Disease Ontology, GO = Gene Ontology, KEGG = Kyoto Encyclopedia of Genes and Genomes, PCA = principal component analysis.

Three datasets, GSE98770, GSE153434, and GSE147026, were integrated (Supplementary File 4, Supplemental Digital Content), and the batch effect correction result is shown in Figure 1A. A total of 111 DEGs were identified (Supplementary File 5, Supplemental Digital Content), including 43 upregulated genes and 68 downregulated genes (Fig. 1B and C). Next, biological functions and enrichment pathways of DEGs were further analyzed (Supplementary File 6, Supplemental Digital Content). GO analysis found that these DEGs were closely related to regulation of leukocyte migration, cellular transition metal ion homeostasis, transition metal ion homeostasis, and detoxification of copper ions (Fig. 1D). Kyoto Encyclopedia of Genes and Genomes analysis showed a similar direction and confirmed the enrichment results of GO (Fig. 1E). Disease Ontology analysis showed that these DEGs were mainly involved in acute myocardial infarction, vasculitis, and coronary artery disease (Fig. 1F). These enrichment findings provided biological context for the transcriptomic differences observed between groups.

### 3.2. Identification of HK3 as diagnostic biomarker

**Figure 2.**
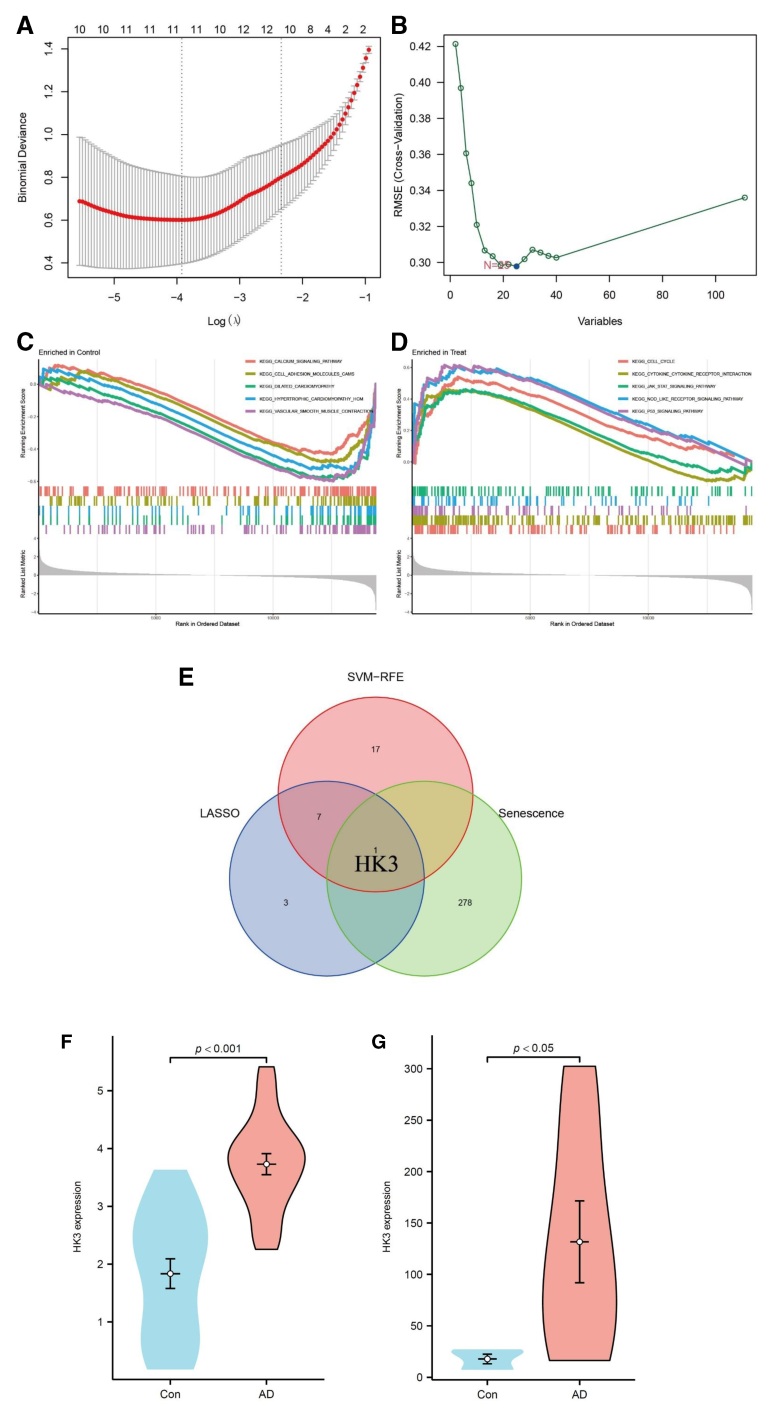
Identification and validation of diagnostic biomarkers. (A, B) LASSO and SVM-RFE algorithm were used to identify potential diagnostic biomarkers. (C, D) Pathways enriched by AD and control samples were analyzed through GSEA. (E) Venn diagram was used to show the overlapping genes between LASSO, SVM-RFE, and senescence-related genes. (F) Expression levels of HK3 gene in integrated dataset. (G) Expression levels of HK3 gene in validation dataset. AD = aortic dissection, GSEA = gene set enrichment analysis, HK3 = hexokinase-3, LASSO = least absolute shrinkage and selection operator, NK cells = natural killer cells, SVM-RFE = support vector machine–recursive feature elimination, RMSE = root mean square error.

Using LASSO and SVM-RFE, we identified 14 and 40 candidate genes, respectively, and then focused on the overlapping genes generated by the 2 algorithms (Fig. 2A and B). GSEA showed that the AD group was predominantly enriched in pathways such as cell cycle, cytokine–cytokine receptor interaction, JAK–STAT signaling, NOD-like receptor signaling, and p53 signaling, whereas pathways including calcium signaling, cell adhesion molecules, cardiomyopathy-related, and vascular smooth muscle contraction were mainly enriched in the control group (Supplementary File 7, Supplemental Digital Content; Fig. 2C and D). Because several of the AD-enriched pathways are closely linked to cellular senescence,^[16,17]^ senescence-related genes were further incorporated into the screening framework. By intersecting machine learning candidates with the senescence-related gene set, hexokinase-3 (HK3) was selected as the final candidate biomarker (Fig. 2E). Importantly, this stepwise strategy was intended to improve biological interpretability and reduce the chance of selecting purely statistical features without clear pathological relevance. In both the integrated dataset and the external validation dataset, HK3 expression was significantly higher in AD samples than in control samples (Fig. 2F and G).

### 3.3. Diagnostic efficiency of HK3 in AD

**Figure 3.**
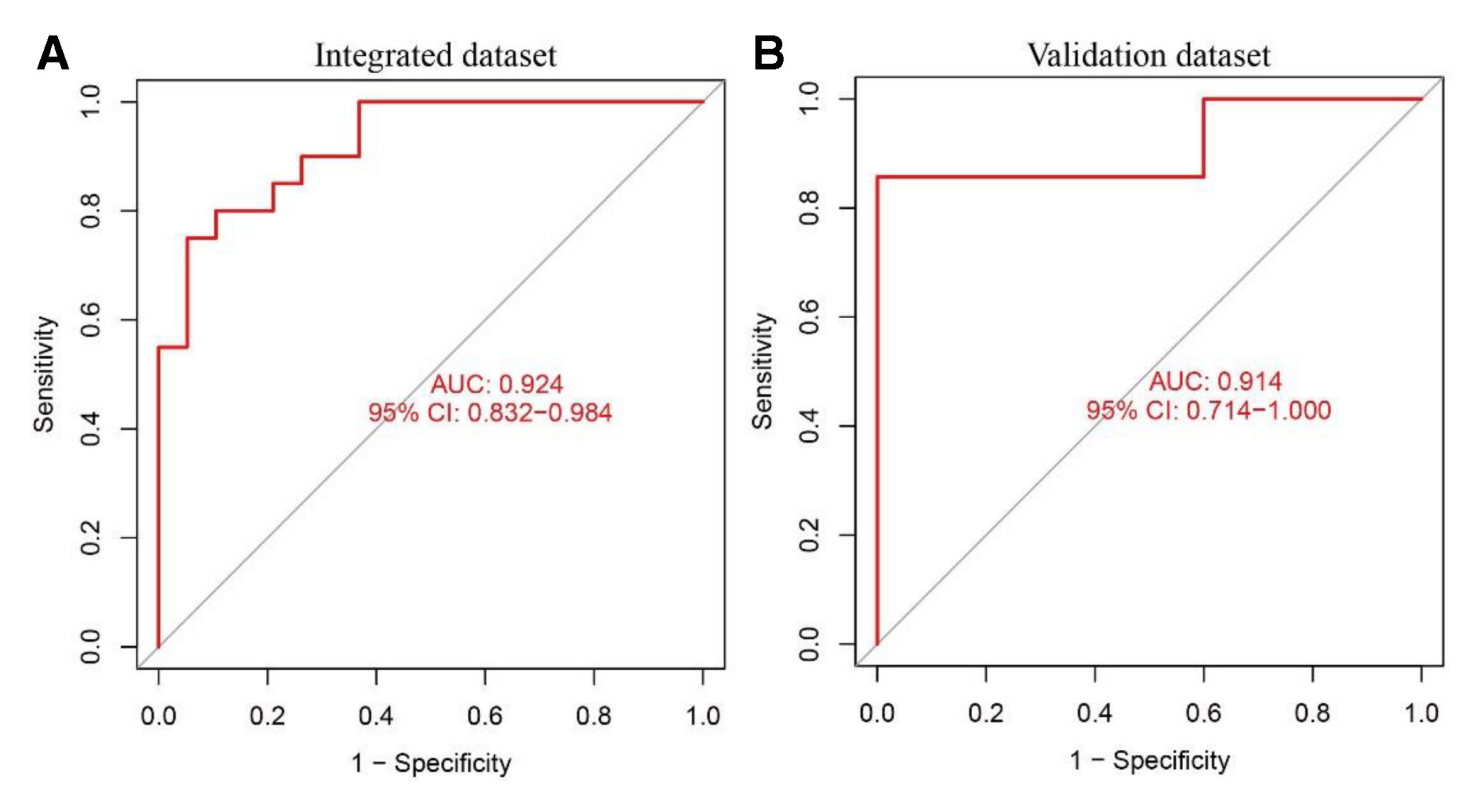
Evaluation of the diagnostic efficiency of HK3 in AD. (A, B) ROC curves for HK3 were drawn in the integration and validation datasets. AD = aortic dissection, AUC = area under the receiver operating characteristic curve, CI = confidence interval, HK3 = hexokinase-3.

As shown by the ROC curve (Fig. 3A), HK3 had good diagnostic value in distinguishing AD from the normal group, with an AUC of 0.924 (95% confidence interval: 0.832–0.984). Similarly, good capability was demonstrated in the validation dataset, with the AUC of the ROC curve for HK3 being 0.914 (Fig. 3B).

### 3.4. Correlation analysis between HK3 and infiltrating immune cells

**Figure 4.**
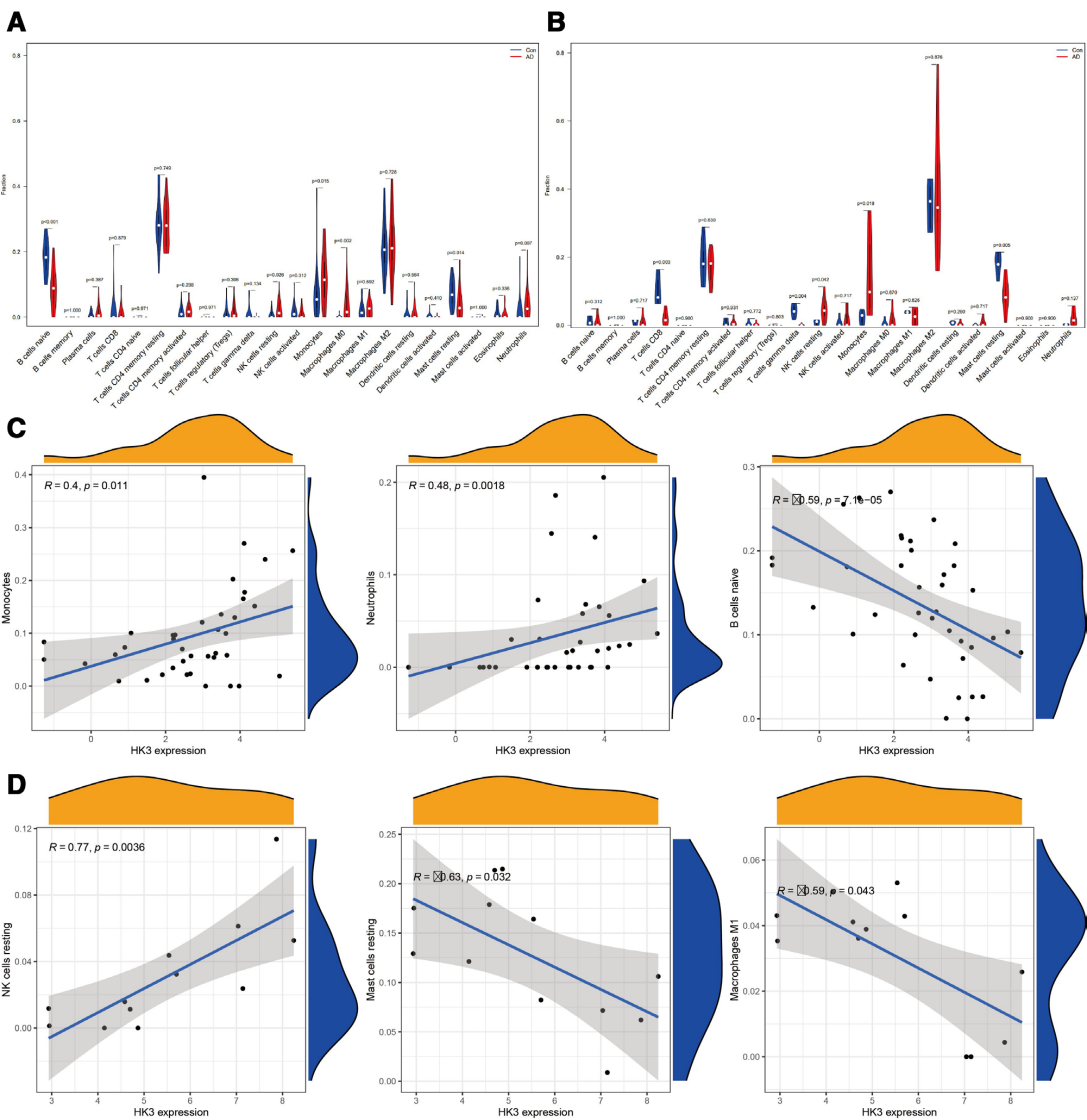
Correlation analysis between HK3 and infiltrating immune cells. (A, B) Composition of immune cell proportions in AD patients and control groups in the integration dataset and validation dataset. (C, D) Spearman correlation analysis was performed to analyze the correlation between HK3 and differentially expressed immune cells in the integration dataset and validation dataset. AD = aortic dissection, HK3 = hexokinase-3.

We analyzed the abundance of infiltrating immune cells in AD and control groups in the integrated dataset (Supplementary File 8, Supplemental Digital Content). The percentages of natural killer (NK) cells resting, monocytes, macrophages M0, and neutrophils in AD samples were prominently higher than those in control samples, while the numbers of B cells naive and mast cells resting in AD samples were remarkably lower than those in control samples (Fig. 4A). NK cells resting, monocytes, and mast cells resting in the GSE52093 dataset were consistent with the trend in the integrated dataset. Furthermore, it was also found that the proportion of T cells gamma delta was higher and the proportion of T cells CD8 was lower in AD tissues than in control tissues (Fig. 4B). Spearman correlation analysis was performed to analyze the relationship between HK3 expression levels and differentially expressed immune cells (Supplementary File 9, Supplemental Digital Content). As shown by the integrated dataset, HK3 was positively correlated with monocytes and neutrophils, while B cells naive were negatively correlated (Fig. 4C). The results of the validation dataset also manifested that HK3 was positively correlated with NK cells resting and negatively correlated with mast cells resting and macrophages M1 (Fig. 4D). These findings suggested a potential relationship between HK3 and the inflammatory immune microenvironment in AD, although the directionality and biological basis of these associations require experimental validation.

## 4. Discussion

mRNA and noncoding RNA are gradually being reported as diagnostic markers of AD. A study found that cyclin-dependent kinase 1 could be viewed as a promising diagnostic biomarker and therapeutic target for thoracic AD.^[24]^ The function of noncoding RNA in AD pathophysiology was described in detail in a review, and their potential as biomarkers and precise targets for AD was highlighted.^[25]^ In this study, we found that the AD group was significantly involved in senescence-related pathways, including cell cycle, cytokine receptor interaction, JAK–STAT signaling pathway, NOD-like receptor signaling pathway, and p53 signaling pathway. Using 2 highly efficient machine learning algorithms and intersection with senescence-related genes, we identified HK3 as a biomarker for AD. HK is one of the key enzymes involved in glycolysis, which contains 4 isoenzymes, namely HK1, HK2, HK3, and HK4, encoded by different genes.^[26]^ Meanwhile, HK3 was also a regulatory gene closely related to premature ovarian failure.^[27]^ Furthermore, one study identified HK3 as a promising target in the context of atherosclerosis.^[18]^ Another study suggested that HK3 could affect the clinical response to intravenous immunoglobulin therapy in acute Kawasaki disease, and it was also a good biomarker for accurate diagnosis.^[28]^ Our study found that both the integrated and validation cohorts showed that HK3 had a good diagnostic performance for AD patients.

Importantly, compared with previous bioinformatics studies in AD, our work has both similarities and distinctions. Liu et al focused on chromatin regulator-related genes and identified tumor protein 53, chromobox homolog 7, and janus kinase 2 as potential diagnostic biomarkers using integrated bioinformatics analysis and external validation.^[29]^ Their study supports the feasibility of biologically constrained biomarker discovery in AD, but its candidate selection was centered on epigenetic regulators. In contrast, our study started from a senescence-oriented framework, which may be more closely linked to vascular wall degeneration, inflammatory activation, and stress responses in AD. Ye et al investigated lactylation-associated biomarkers in AD and, by combining weighted gene co-expression network analysis with machine learning, identified phoshoglycerate kinase 1 and high mobility group protein A1 as candidate markers, followed by validation in human aortic tissues and an animal model.^[30]^ Compared with that study, our results similarly support the value of integrating pathway-focused gene sets with machine-learning approaches, but also indicate that the current conclusions regarding HK3 should be interpreted more cautiously, because our evidence is still mainly based on public transcriptomic datasets and peripheral validation rather than tissue-level and functional confirmation. Therefore, our data support HK3 as a promising candidate, rather than as a definitively established clinical marker for AD.

Currently, machine learning and artificial intelligence (AI) have advanced rapidly in cardiology and are increasingly being applied to disease detection, electrocardiogram (ECG)-based screening, risk prediction, and individualized clinical decision-making. Studies have shown that AI-enabled ECG models can identify individuals with possible coronary artery disease from routine 12-lead ECGs,^[31]^ detect occlusive myocardial infarction with high diagnostic performance,^[32]^ and uncover latent electrophysiological signatures of atrial fibrillation even during sinus rhythm, thereby supporting early screening and future risk prediction in high-risk populations.^[33]^ Meanwhile, review evidence has further highlighted the expanding role of AI in coronary artery disease and atrial fibrillation management.^[34]^ Machine-learning approaches based on ECG waveform features, including P, QRS, and T wave characteristics derived from treadmill exercise testing, have also shown promising value in predicting obstructive coronary artery disease.^[35]^ Moreover, recent studies on clinical prediction modeling have emphasized that appropriately developed and validated algorithms can provide highly accurate and clinically meaningful estimates for adverse cardiovascular events and perioperative myocardial injury.^[36]^ Systematic review evidence further suggests that machine-learning models demonstrate good overall performance in cardiovascular disease risk prediction, although rigorous external validation and careful assessment of generalizability remain essential before routine clinical implementation.^[37]^ Collectively, these advances support the rationale for applying machine-learning-based strategies in our study to screen diagnostic markers for AD, and they further suggest that biomarker discovery models integrating multi-omics data with algorithmic approaches may offer new opportunities for early auxiliary diagnosis, risk stratification, and more precise management of AD.

CIBERSORT is a method that can characterize the composition of immune cells from mRNA expression matrices of complex tissues, which will help to explore the correlation between AD lesions and infiltrating immune cells.^[20]^ Our results showed that, compared with the control samples, the AD samples contained more abundant NK cells resting, monocytes, macrophages M0 and neutrophils, while B cells naive and mast cells resting were relatively low. Our data implied that AD was closely related to immune infiltration and was consistent with single-cell RNA sequencing findings an increased proportion of NK cells and macrophages in AD tissues, whereas B cells were contradictory.^[38]^ A report found that macrophages, neutrophils, natural killer t cells, and natural regulatory T cells were involved in the occurrence and development of type A AD.^[39]^ In addition, Spearman correlation analysis was adopted to further clarify the association between HK3 and immune cells. Our results found that HK3 was positively correlated with monocytes and neutrophils, but negatively correlated with B cells naive. These data suggested that HK3 was also closely related to immune cell infiltration. Meanwhile, evidence has shown that HK3 expression was closely related to inflammatory activity and immune response in non-small cell lung cancer.^[40]^ HK3 also specifically stimulated the infiltration of mononuclear/macrophage cells presenting surface markers, regulated the immune checkpoint molecules programmed cell death protein 1 and cytotoxic T lymphocyte antigen 4 of exhausted T cells, inhibited immune response, assisted the escape of tumor cells, and promoted the invasive progression of clear cell renal cell carcinoma.^[41]^

Previous studies on biomarkers of AD mainly focused on the aspect of differential expression analysis, single-algorithmscreening, or specific pathways/molecular families. The novelty of this study lies in the following: it focuses on the pathological process of cellular senescence, which has a clear biological direction, rather than conducting candidate gene digging without direction; combining 2 machine learning algorithms, LASSO and SVM-RFE, for feature selection and then intersecting them with senescence-related genes, to improve the targeting and robustness of candidate diagnostic biomarker screening; and a further combination with immune cell infiltration analysis after the identification of HK3 to preliminarily establish a framework for the correlation between features and immune microenvironment changes in senescence-related genes.

Although the expression level of HK3 was analyzed from a public database, we cannot ignore that our study has some limitations. First, we combined multiple datasets to expand the sample size, but it is still insufficient. Second, it brings difficulties for further analysis due to insufficient clinical information in these samples.

## 5. Conclusion

In summary, this study identified HK3 as a targeted gene by combining senescence-related gene sets with 2 excellent computer algorithms. Meanwhile, HK3 was viewed as a potential biological marker for AD diagnosis and a target for precision therapy, and HK3 was also closely related to immune infiltration.

## Author contributions

**Conceptualization:** Jiabao Zhong, Hongli Luo.

**Data curation:** Jiabao Zhong.

**Formal analysis:** Jiabao Zhong.

**Visualization:** Jiabao Zhong.

**Investigation:** Hongli Luo.

**Validation:** Hongli Luo.

**Writing – original draft:** Jiabao Zhong.

**Writing – review & editing:** Hongli Luo.



















## References

[1] Lombardi JV Hughes GC Appoo JJ. Society for Vascular Surgery (SVS) and Society of Thoracic Surgeons (STS) reporting standards for type B aortic dissections. J Vasc Surg. 2020;71:723–47. 32001058 10.1016/j.jvs.2019.11.013

[2] Rombouts KB van Merrienboer TAR Ket JCF Bogunovic N van der Velden J Yeung KK. The role of vascular smooth muscle cells in the development of aortic aneurysms and dissections. Eur J Clin Invest. 2022;52:e13697. 34698377 10.1111/eci.13697 PMC9285394

[3] Kamman AV Yang B Kim KM Williams DM Michael Deeb G Patel HJ. Visceral malperfusion in aortic dissection: the Michigan experience. Semin Thorac Cardiovasc Surg. 2017;29:173–8. 28823323 10.1053/j.semtcvs.2016.10.002

[4] Evangelista A Isselbacher EM Bossone E. Insights from the International Registry of Acute Aortic Dissection: a 20-year experience of collaborative clinical research. Circulation. 2018;137:1846–60. 29685932 10.1161/CIRCULATIONAHA.117.031264

[5] Nienaber CA Clough RE Sakalihasan N. Aortic dissection. Nat Rev Dis Primers. 2016;2:16053. 27440162 10.1038/nrdp.2016.53

[6] Olsson C Thelin S Ståhle E Ekbom A Granath F. Thoracic aortic aneurysm and dissection: increasing prevalence and improved outcomes reported in a nationwide population-based study of more than 14,000 cases from 1987 to 2002. Circulation. 2006;114:2611–8. 17145990 10.1161/CIRCULATIONAHA.106.630400

[7] Nienaber CA Clough RE. Management of acute aortic dissection. Lancet. 2015;385:800–11. 25662791 10.1016/S0140-6736(14)61005-9

[8] Salmasi MY Al-Saadi N Hartley P. The risk of misdiagnosis in acute thoracic aortic dissection: a review of current guidelines. Heart. 2020;106:885–91. 32170039 10.1136/heartjnl-2019-316322

[9] Joshi A Rienks M Theofilatos K Mayr M. Systems biology in cardiovascular disease: a multiomics approach. Nat Rev Cardiol. 2021;18:313–30. 33340009 10.1038/s41569-020-00477-1

[10] Xu J Yang Y. Integrated gene expression profiling analysis reveals potential molecular mechanisms and candidate biomarkers for early risk stratification and prediction of STEMI and post-STEMI heart failure patients. Front Cardiovasc Med. 2021;8:736497. 34957234 10.3389/fcvm.2021.736497 PMC8702808

[11] Xie H Zha E Zhang Y. Identification of featured metabolism-related genes in patients with acute myocardial infarction. Dis Markers. 2020;2020:8880004. 33354250 10.1155/2020/8880004 PMC7737445

[12] Yang Y Yi X Cai Y Zhang Y Xu Z. Immune-associated gene signatures and subtypes to predict the progression of atherosclerotic plaques based on machine learning. Front Pharmacol. 2022;13:865624. 35559253 10.3389/fphar.2022.865624 PMC9086243

[13] Xia L Sun C Zhu H. Melatonin protects against thoracic aortic aneurysm and dissection through SIRT1-dependent regulation of oxidative stress and vascular smooth muscle cell loss. J Pineal Res. 2020;69:e12661. 32329099 10.1111/jpi.12661

[14] Ma D Zheng B Liu H-L. Klf5 down-regulation induces vascular senescence through eIF5a depletion and mitochondrial fission. PLoS Biol. 2020;18:e3000808. 32817651 10.1371/journal.pbio.3000808 PMC7462304

[15] Owens WA Walaszczyk A Spyridopoulos I Dookun E Richardson GD. Senescence and senolytics in cardiovascular disease: promise and potential pitfalls. Mech Ageing Dev. 2021;198:111540. 34237321 10.1016/j.mad.2021.111540 PMC8387860

[16] Rufini A Tucci P Celardo I Melino G. Senescence and aging: the critical roles of p53. Oncogene. 2013;32:5129–43. 23416979 10.1038/onc.2012.640

[17] Salama R Sadaie M Hoare M Narita M. Cellular senescence and its effector programs. Genes Dev. 2014;28:99–114. 24449267 10.1101/gad.235184.113 PMC3909793

[18] Ménégaut L Jalil A Pilot T. Regulation of glycolytic genes in human macrophages by oxysterols: a potential role for liver X receptors. Br J Pharmacol. 2021;178:3124–39. 33377180 10.1111/bph.15358

[19] Aging Atlas Consortium. Aging Atlas: a multi-omics database for aging biology. Nucleic Acids Res. 2021;49:D825–30. 33119753 10.1093/nar/gkaa894 PMC7779027

[20] Friedman J Hastie T Tibshirani R. Regularization paths for generalized linear models via coordinate descent. J Stat Softw. 2010;33:1–22. 20808728 PMC2929880

[21] Huang M-L Hung Y-H Lee WM Li RK Jiang B-R. SVM-RFE based feature selection and Taguchi parameters optimization for multiclass SVM classifier. ScientificWorldJournal. 2014;2014:795624. 25295306 10.1155/2014/795624 PMC4175386

[22] Subramanian A Tamayo P Mootha VK. Gene set enrichment analysis: a knowledge-based approach for interpreting genome-wide expression profiles. Proc Natl Acad Sci U S A. 2005;102:15545–50. 16199517 10.1073/pnas.0506580102 PMC1239896

[23] Newman AM Liu CL Green MR. Robust enumeration of cell subsets from tissue expression profiles. Nat Methods. 2015;12:453–7. 25822800 10.1038/nmeth.3337 PMC4739640

[24] Wang W Liu Q Wang Y. Verification of hub genes in the expression profile of aortic dissection. PLoS One. 2019;14:e0224922. 31751374 10.1371/journal.pone.0224922 PMC6872142

[25] Cheng M Yang Y Xin H. Non-coding RNAs in aortic dissection: from biomarkers to therapeutic targets. J Cell Mol Med. 2020;24:11622–37. 32885591 10.1111/jcmm.15802 PMC7578866

[26] Wilson JE. Isozymes of mammalian hexokinase: structure, subcellular localization and metabolic function. J Exp Biol. 2003;206:2049–57. 12756287 10.1242/jeb.00241

[27] Qin Y Sun M You L. ESR1, HK3 and BRSK1 gene variants are associated with both age at natural menopause and premature ovarian failure. Orphanet J Rare Dis. 2012;7:5. 22248077 10.1186/1750-1172-7-5 PMC3275465

[28] Geng Z Liu J Hu J. Crucial transcripts predict response to initial immunoglobulin treatment in acute Kawasaki disease. Sci Rep. 2020;10:17860. 33082496 10.1038/s41598-020-75039-z PMC7575539

[29] Liu C Zhou Y Zhao D. Identification and validation of differentially expressed chromatin regulators for diagnosis of aortic dissection using integrated bioinformatics analysis and machine-learning algorithms. Front Genet. 2022;13:950613. 36035141 10.3389/fgene.2022.950613 PMC9403720

[30] Ye J Fu Y Ke Y. Lactylation associated biomarkers and immune infiltration in aortic dissection. Sci Rep. 2025;15:21536. 40596702 10.1038/s41598-025-08613-y PMC12219385

[31] Kany S Friedman SF Al-Alusi M. Electrocardiogram-based artificial intelligence to identify coronary artery disease. JACC Adv. 2025;4:102041. 40749517 10.1016/j.jacadv.2025.102041 PMC12337187

[32] Al-Zaiti SS Martin-Gill C Zègre-Hemsey JK. Machine learning for ECG diagnosis and risk stratification of occlusion myocardial infarction. Nat Med. 2023;29:1804–13. 37386246 10.1038/s41591-023-02396-3 PMC10353937

[33] Attia ZI Noseworthy PA Lopez-Jimenez F. An artificial intelligence-enabled ECG algorithm for the identification of patients with atrial fibrillation during sinus rhythm: a retrospective analysis of outcome prediction. Lancet. 2019;394:861–7. 31378392 10.1016/S0140-6736(19)31721-0

[34] Hayiroğlu MI Altay S. The role of artificial intelligence in coronary artery disease and atrial fibrillation. Balkan Med J. 2023;40:151–2. 37025078 10.4274/balkanmedj.galenos.2023.06042023 PMC10175890

[35] Yilmaz A Hayiroğlu MI Salturk S. Machine learning approach on high risk treadmill exercise test to predict obstructive coronary artery disease by using P, QRS, and T waves’ features. Curr Probl Cardiol. 2023;48:101482. 36336117 10.1016/j.cpcardiol.2022.101482

[36] Cicek V Babaoglu M Saylik F. A new risk prediction model for the assessment of myocardial injury in elderly patients undergoing non-elective surgery. J Cardiovasc Dev Dis. 2024;12:6. 39852284 10.3390/jcdd12010006 PMC11765956

[37] Liu T Krentz A Lu L Curcin V. Machine learning based prediction models for cardiovascular disease risk using electronic health records data: systematic review and meta-analysis. Eur Heart J Digit Health. 2025;6:7–22. 39846062 10.1093/ehjdh/ztae080 PMC11750195

[38] Liu Y Zou L Tang H. Single-cell sequencing of immune cells in human aortic dissection tissue provides insights into immune cell heterogeneity. Front Cardiovasc Med. 2022;9:791875. 35433892 10.3389/fcvm.2022.791875 PMC9008490

[39] Chen F Han J Tang B. Patterns of immune infiltration and the key immune-related genes in acute type A aortic dissection in bioinformatics analyses. Int J Gen Med. 2021;14:2857–69. 34211294 10.2147/IJGM.S317405 PMC8242140

[40] Tuo Z Zheng X Zong Y. HK3 is correlated with immune infiltrates and predicts response to immunotherapy in non-small cell lung cancer. Clin Transl Med. 2020;10:319–30. 32508023 10.1002/ctm2.6 PMC7240846

[41] Xu W Liu W-R Xu Y. Hexokinase 3 dysfunction promotes tumorigenesis and immune escape by upregulating monocyte/macrophage infiltration into the clear cell renal cell carcinoma microenvironment. Int J Biol Sci. 2021;17:2205–22. 34239350 10.7150/ijbs.58295 PMC8241725

