# Supplementary material for: Identification of senescence-related biomarker for aortic dissection based on bioinformatics and machine learning algorithms
Source: Medicine (Baltimore). 2026 May 29;105(22):e48873. doi: 10.1097/MD.0000000000048873 (PMC13249447; doi:10.1097/MD.0000000000048873)
Supplement: Supplementary file 1 [file medi-105-e48873-s001.docx]

**Supplementary file 1 Table S1.** Baseline data of datasets from the GEO database.

| Data | GSE98770 | GSE153434 | GSE147026 | GSE52093 |
| --- | --- | --- | --- | --- |
| Cohort description | aortic dissection | aortic dissection | aortic dissection | acute aortic dissection |
| Control (n) | 5 | 10 | 4 | 5 |
| Aortic dissection(n) | 6 | 10 | 4 | 7 |
| Platform | GPL14550 | GPL20795 | GPL24676 | GPL10558 |
| Organism | Homo sapiens | Homo sapiens | Homo sapiens | Homo sapiens |
| Male (n) | 8 | 0 | 0 | 10 |
| Female (n) | 3 | 0 | 0 | 1 |
