# Supplementary material for: Identification of senescence-related biomarker for aortic dissection based on bioinformatics and machine learning algorithms
Source: Medicine (Baltimore). 2026 May 29;105(22):e48873. doi: 10.1097/MD.0000000000048873 (PMC13249447; doi:10.1097/MD.0000000000048873)
Supplement: Supplementary file 2 [file medi-105-e48873-s002.docx]

**Supplementary file 2 Table S2.** Senescence-related genes.

| ACLY |
| --- |
| AAK1 |
| ABI3 |
| ADCK5 |
| AKR1B1 |
| AGT |
| AKT1 |
| ALOX15B |
| AR |
| ARPC1B |
| ASF1A |
| ASPH |
| ATF7IP |
| ATM |
| AURKA |
| AXL |
| BAG3 |
| BHLHE40 |
| BCL6 |
| BLK |
| BLVRA |
| BMI1 |
| BRAF |
| BRD7 |
| BRCA1 |
| BTG3 |
| C11orf31 |
| CAV1 |
| CBX7 |
| CBX8 |
| CCND1 |
| CDK1 |
| CDK18 |
| CDK2AP1 |
| CDK6 |
| CDK4 |
| CDKN1A |
| CDKN1C |
| CDKN1B |
| CDKN2A |
| CDKN2AIP |
| CDKN2B |
| CENPA |
| CEBPB |
| CHEK1 |
| CKB |
| CPEB1 |
| CSNK1A1 |
| CTNNAL1 |
| CSNK2A1 |
| CXCL1 |
| DDB2 |
| CYR61 |
| DEK |
| DGCR8 |
| DHCR24 |
| DLX2 |
| DHX9 |
| DPY30 |
| DUSP3 |
| DUSP16 |
| E2F1 |
| EHF |
| ENDOG |
| EPHA3 |
| ERRFI1 |
| ETS1 |
| ETS2 |
| EWSR1 |
| FASTK |
| EZH2 |
| FBXO31 |
| FOXM1 |
| FOS |
| FOXO3 |
| FXR1 |
| G6PD |
| GAPDH |
| GKN1 |
| GATA4 |
| GNG11 |
| GLB1 |
| GRK6 |
| HDAC4 |
| HDAC1 |
| HEPACAM |
| HJURP |
| HIVEP1 |
| HK3 |
| HMGB1 |
| HRAS |
| HSPA5 |
| HSPB2 |
| ID1 |
| ID4 |
| IGFBP1 |
| IFNG |
| IGFBP3 |
| IGFBP6 |
| IGFBP5 |
| IL1A |
| IL8 |
| ING1 |
| ING2 |
| IRF3 |
| IRF5 |
| IRF7 |
| ITPK1 |
| ITGB4 |
| ITPKB |
| ITSN2 |
| KCNJ12 |
| KDM4A |
| KDM5B |
| KIAA1524 |
| KL |
| KSR2 |
| LATS1 |
| LEO1 |
| LGALS3 |
| LIMA1 |
| LIMK1 |
| MAGEA2 |
| MAGOH |
| MAD2L1 |
| MAGOHB |
| MAP2K1 |
| MAP2K3 |
| MAP2K2 |
| MAP2K6 |
| MAP3K6 |
| MAP2K7 |
| MAP4K1 |
| MAP3K7 |
| MAPK12 |
| MAPKAPK5 |
| 5-Mar |
| MAPK14 |
| MAST1 |
| MATK |
| MCL1 |
| MDH1 |
| MCRS1 |
| MECP2 |
| MOB3A |
| MMP9 |
| MORC3 |
| MORF4 |
| MXD4 |
| MVK |
| MYC |
| MYLK |
| NADK |
| NANOG |
| NDRG1 |
| NEK1 |
| NEK4 |
| NEK6 |
| NFE2L2 |
| NINJ1 |
| NOTCH3 |
| NOX4 |
| NR2E1 |
| NTN4 |
| NUAK1 |
| OTX2 |
| P3H1 |
| PATZ1 |
| PAK4 |
| PBRM1 |
| PCGF2 |
| PDCD10 |
| PDIK1L |
| PDZD2 |
| PDPK1 |
| PEBP1 |
| PEX19 |
| PIAS4 |
| PIK3R5 |
| PIK3C2A |
| PIM1 |
| PLA2R1 |
| PKM |
| PML |
| PNPT1 |
| PMVK |
| POT1 |
| POU5F1 |
| PPM1B |
| PPM1D |
| PRMT6 |
| PRKCH |
| PRKCD |
| PROX1 |
| PRPF19 |
| PSMB5 |
| PTRF |
| PTTG1 |
| PSMD14 |
| RAD21 |
| RAF1 |
| RB1 |
| RBP2 |
| RBX1 |
| RNASEL |
| RPS6KA6 |
| RSL1D1 |
| RUNX1 |
| RUVBL2 |
| SENP1 |
| SENP2 |
| SENP7 |
| SERPINE1 |
| SFN |
| SIK1 |
| SGK1 |
| SIN3B |
| SIRT1 |
| SIRT6 |
| SIX1 |
| SLC13A3 |
| SLC16A7 |
| SMARCA4 |
| SMG1 |
| SMARCB1 |
| SMURF2 |
| SNAI1 |
| SOCS1 |
| SOD1 |
| SORBS2 |
| SOX2 |
| SPIN1 |
| SOX5 |
| SP1 |
| SPOP |
| SRC |
| SREBF1 |
| SRSF1 |
| STAT5B |
| STK32C |
| STK40 |
| SUPT5H |
| SYK |
| TACC3 |
| TERC |
| TBX2 |
| TERF2 |
| TERT |
| TFAP4 |
| TFDP1 |
| TGFB1I1 |
| TLR3 |
| TMSB4X |
| TNFSF13 |
| TNFSF15 |
| TOP1 |
| TP63 |
| TPR |
| TP53 |
| TRIM28 |
| TRPM8 |
| TXN |
| TXNIP |
| UBTD1 |
| TYK2 |
| VENTX |
| USP1 |
| VEGFA |
| WNT16 |
| WNT2 |
| WRN |
| WT1 |
| XAF1 |
| WWP1 |
| YAP1 |
| YPEL3 |
| ZFP36 |
| ZMAT3 |
| ZNF148 |
