# Supplementary material for: Identification of senescence-related biomarker for aortic dissection based on bioinformatics and machine learning algorithms
Source: Medicine (Baltimore). 2026 May 29;105(22):e48873. doi: 10.1097/MD.0000000000048873 (PMC13249447; doi:10.1097/MD.0000000000048873)
Supplement: Supplementary file 3 [file medi-105-e48873-s003.pdf]

```
#install.packages("corrplot")


#引用包
library(corrplot)
inputFile="CIBERSORT-Results.txt"     #免疫细胞浸润的结果文件
setwd("C:\\biowolf\\Diagnostic\\19.barplot")     #设置工作目录

#读取免疫细胞浸润文件
rt=read.table(inputFile, header=T, sep="\t", check.names=F,
row.names=1)

#对样品分组
con=grepl("_con", rownames(rt), ignore.case=T)
treat=grepl("_treat", rownames(rt), ignore.case=T)
conData=rt[con,]
treatData=rt[treat,]
conNum=nrow(conData)
treatNum=nrow(treatData)
data=t(rbind(conData,treatData))

#绘制柱状图
pdf(file="barplot.pdf", width=16, height=9)
col=rainbow(nrow(data), s=0.7, v=0.7)
par(las=1,mar=c(8,5,4,16),mgp=c(3,0.1,0),cex.axis=1.5)
a1=barplot(data,col=col,yaxt="n",ylab="Relative
Percent",xaxt="n",cex.lab=1.8)
a2=axis(2,tick=F,labels=F)
axis(2,a2,paste0(a2*100,"%"))
par(srt=0,xpd=T)
rect(xleft = a1[1], ybottom = -0.01, xright = a1[conNum], ytop = -
0.06,col="green")
text(a1[conNum]/2,-0.035,"Con",cex=1.8)
rect(xleft = a1[conNum], ybottom = -0.01, xright =a1[length(a1)] ,
ytop = -0.06,col="red")
text((a1[length(a1)]+a1[conNum])/2,-0.035,"Treat",cex=1.8)
ytick2 = cumsum(data[,ncol(data)])
```

```
ytick1 = c(0,ytick2[-length(ytick2)])
legend(par('usr')[2]*0.98,par('usr')[4],legend=rownames(data),col=col,
pch=15,bty="n",cex=1.2)
dev.off()

#相关性分析
pdf("corHeatmap.pdf", width=12, height=12)
corrplot(corr=cor(rt),
         method = "color",
         order = "hclust",
         tl.col="black",
         addCoef.col = "black",
         number.cex = 0.8,
         col=colorRampPalette(c("blue", "white", "red"))(50),
         )
dev.off()
```

KEGG_N_GLYCAN_BIOSYNTHESIS http://www.gsea-msigdb.org/gsea/msigdb/cards/KEGG_N_GLYCAN_BIOSYNTHESIS ALG13 DOLPP1 RPN1 ALG14 MAN1B1 ALG3 B4GALT1 MGAT5 RPN2 STT3A MGAT3 DAD1 MGAT2 ALG12 TUSC3 MAN1C1 DPM2 DPM1 GANAB ALG1 MGAT4A ALG10B STT3B MAN1A2 ALG10 ALG11 ALG8 ALG2 DPAGT1 RFT1 DPM3 DDOST MGAT4B ALG6 MAN2A2 MAN1A1 MAN2A1 ST6GAL1 B4GALT3 ALG5 B4GALT2 MGAT5B ALG9 MOGS FUT8 MGAT1

KEGG_OTHER_GLYCAN_DEGRADATION http://www.gsea-msigdb.org/gsea/msigdb/cards/KEGG_OTHER_GLYCAN_DEGRADATION ENGASE GLB1 MANBA MAN2B1 GBA NEU4 NEU2 NEU1 FUCA1 FUCA2 AGA MAN2C1 MAN2B2 NEU3 HEXB HEXA

KEGG_O_GLYCAN_BIOSYNTHESIS http://www.gsea-msigdb.org/gsea/msigdb/cards/KEGG_O_GLYCAN_BIOSYNTHESIS GALNT4 GALNT15 GALNTL5 GALNT6 GALNT5 GALNT16 GALNTL6 GALNT13 GCNT3 GALNT10 ST6GALNAC1 GALNT9 GALNT7 GCNT4 GALNT11 GCNT1 GALNT8 GALNT14 B3GNT6 C1GALT1 GALNT18 GALNT12 GALNT2 GALNT3 GALNT17 GALNT1 ST3GAL2 ST3GAL1 B4GALT5 C1GALT1C1

KEGG_GLYCOSAMINOGLYCAN_DEGRADATION http://www.gsea-msigdb.org/gsea/msigdb/cards/KEGG_GLYCOSAMINOGLYCAN_DEGRADATION HS3ST3A1 HPSE HPSE2 GLB1 GUSB HYAL3 GNS HYAL4 HYAL1 HYAL2 SPAM1 HGSNAT GALNS IDUA HS3ST3B1 SGSH HEXB NAGLU HEXA IDS ARSB

KEGG_GLYCOSAMINOGLYCAN_BIOSYNTHESIS_KERATAN_SULFATE http://www.gsea-msigdb.org/gsea/msigdb/cards/KEGG_GLYCOSAMINOGLYCAN_BIOSYNTHESIS_KERATAN_SULFATE CHST2 B4GAT1 B3GNT2 CHST1 B4GALT1 B3GNT7 ST3GAL2 B4GALT3 ST3GAL1 B4GALT2 B4GALT4 CHST4 ST3GAL3 FUT8 CHST6

KEGG_GLYCEROLIPID_METABOLISM http://www.gsea-msigdb.org/gsea/msigdb/cards/KEGG_GLYCEROLIPID_METABOLISM MBOAT2 GPAM LIPG DGKZ DGKE DGKD DGKH MBOAT1 GK DGAT2 GK2 GLA ALDH7A1 AGK TKFC AGPAT3 AWAT2 AGPAT4 AKR1B1 PNLIPRP2 PNLIPRP1 PNLIP DGAT1 GLYCTK LPL ALDH1B1 DGKQ ALDH2 DGKB DGKG AKR1A1 DGKA PLPP3 LIPC DGKI LCLAT1 GPAT3 MGLL GPAT4 ALDH9A1 ALDH3A2 PNPLA3 PLPP1 PLPP2 AGPAT1 AGPAT2 CEL GPAT2 LIPF

KEGG_GLYCOSYLPHOSPHATIDYLINOSITOL_GPI_ANCHOR_BIOSYNTHESIS

http://www.gsea-msigdb.org/gsea/msigdb/cards/KEGG_GLYCOSYLPHOSPHATIDYLINOSITOL_GPI_ANCHOR_BIOSYNTHESIS PIGP PIGW PIGM PIGN PIGS PIGF DPM2 PIGQ PIGH PIGO PIGG PIGZ GPAA1 PIGU PGAP1 PIGT PIGK PIGY PIGL PIGV PIGB PIGX PIGA GPLD1 PIGC

KEGG_GLYCEROPHOSPHOLIPID_METABOLISM http://www.gsea-msigdb.org/gsea/msigdb/cards/KEGG_GLYCEROPHOSPHOLIPID_METABOLISM PEMT PLA2G15 GPD1 GPD1L LPGAT1 GPAM MBOAT2 MBOAT7 CHAT LPCAT1 PTDSS1 ACHE PLA2G3 DGKZ ETNK1 DGKE DGKD PCYT1A DGKH JMJD7-PLA2G4B CHPT1 MBOAT1 PLA2G6 PLA2G2E PLA2G10 LPCAT3 PLA2G2A ETNK2 PLA2G4A PLA2G5 PLA2G12B PLA2G4B PTDSS2 PLA2G2F PCYT2 CDS1 AGPAT3 AGPAT4 PGS1 LYPLA1 PLA2G1B ADPRM CDIPT DGKQ CDS2 DGKB DGKG PLPP3 DGKA DGKI PHOSPHO1 LCLAT1 TAZ GPAT3 GNPAT LPCAT4 PLA2G4E GPAT4 CHKA PLPP1 PLPP2 LYPLA2 PLA2G2C AGPAT1 PLA2G2D LPCAT2 PLA2G12A AGPAT2 PLD1 CHKB PISD PLD2 PCYT1B GPAT2 LCAT CRLS1 GPD2

KEGG_ETHER_LIPID_METABOLISM http://www.gsea-msigdb.org/gsea/msigdb/cards/KEGG_ETHER_LIPID_METABOLISM ENPP2 PAFAH1B1 PLPP3 LPCAT1 PLA2G3 ENPP6 LPCAT4 PLA2G4E JMJD7-PLA2G4B CHPT1 AGPS PLA2G6 PAFAH1B3 PLA2G2E PLA2G10 PAFAH2 PLA2G2A PLA2G4A PLA2G5 PLA2G12B PLA2G4B PLA2G7 PLA2G2F PLPP1 PLPP2 PLA2G2C PAFAH1B2 PLA2G2D LPCAT2 PLD1 PLA2G12A PLD2 PLA2G1B

KEGG_ARACHIDONIC_ACID_METABOLISM http://www.gsea-msigdb.org/gsea/msigdb/cards/KEGG_ARACHIDONIC_ACID_METABOLISMEPHX2 CYP2E1 ALOX5 CYP4A11 CYP2J2 GGT1 PLA2G3 AKR1C3 PTGIS ALOX12B CYP4F2 CYP2C18 PTGES2 ALOX15B ALOX15 JMJD7-PLA2G4B PLA2G6 PLA2G2E PLA2G10 PLA2G2A PLA2G4A PLA2G5 PTGS2 PLA2G12B PTGS1 PLA2G4B PLA2G2F ALOX12 CYP2C9 GPX7 GPX6 CYP2C19 CYP2C8 CBR1 CYP2B6 CBR3 CYP2U1 PLA2G1B LTA4H PTGES GPX5 GPX1 CYP4A22 GPX2 GPX3 GPX4 CYP4F3 PLA2G4E LTC4S GGT6 GGT7 GGT5 HPGDS PLA2G2C PTGDS PLA2G2D PLA2G12A TBXAS1

KEGG_LINOLEIC_ACID_METABOLISM http://www.gsea-msigdb.org/gsea/msigdb/cards/KEGG_LINOLEIC_ACID_METABOLISM CYP2E1 CYP3A5 CYP2J2 CYP3A4 PLA2G3 AKR1B10 CYP2C18 CYP1A2 ALOX15

PLA2G4E JMJD7-PLA2G4B PLA2G6 PLA2G2E PLA2G10 PLA2G2A PLA2G4A CYP3A43 PLA2G5 PLA2G12B PLA2G4B PLA2G2F PLA2G2C PLA2G2D CYP2C9 PLA2G12A CYP2C19 CYP2C8 CYP3A7 PLA2G1B

KEGG_ALPHA_LINOLENIC_ACID_METABOLISM http://www.gsea-msigdb.org/gsea/msigdb/cards/KEGG_ALPHA_LINOLENIC_ACID_METABOLISM JMJD7-PLA2G4B PLA2G6 PLA2G2E PLA2G10 PLA2G2A PLA2G4A PLA2G5 PLA2G12B PLA2G4B PLA2G2F FADS2 ACOX3 PLA2G2C PLA2G2D PLA2G3 PLA2G12A ACOX1 PLA2G4E PLA2G1B

KEGG_SPHINGOLIPID_METABOLISM http://www.gsea-msigdb.org/gsea/msigdb/cards/KEGG_SPHINGOLIPID_METABOLISM GAL3ST1 SGPP2 GLB1 GALC SGMS2 GBA SPHK2 NEU2 PLPP3 NEU1 ACER2 UGCG DEGS2 ARSA SPHK1 SGPL1 NEU3 SMPD4 SGMS1 ACER3 CERK SMPD2 ENPP7 DEGS1 SGPP1 NEU4 GLA ACER1 PLPP1 ASAH1 PLPP2 SMPD1 B4GALT6 SPTLC2 SPTLC1 SMPD3 KDSR UGT8 ASAH2

KEGG_GLYCOSPHINGOLIPID_BIOSYNTHESIS_LACTO_AND_NEOLACTO_SERIES http://www.gsea-msigdb.org/gsea/msigdb/cards/KEGG_GLYCOSPHINGOLIPID_BIOSYNTHESIS_LACTO_AND_NEOLACTO_SERIES B3GNT4 B3GNT2 ST3GAL6 B4GALT1 B3GNT5 FUT9 FUT7 FUT6 FUT5 FUT4 FUT3 FUT2 FUT1 B4GAT1 GCNT2 B3GNT3 B3GALT5 ABO B4GALT3 B4GALT2 ST3GAL4 B4GALT4 ST3GAL3 B3GALT2 B3GALT1 ST8SIA1

KEGG_GLYCOSPHINGOLIPID_BIOSYNTHESIS_GLOBO_SERIES http://www.gsea-msigdb.org/gsea/msigdb/cards/KEGG_GLYCOSPHINGOLIPID_BIOSYNTHESIS_GLOBO_SERIES A4GALT B3GALT5 GLA FUT9 ST3GAL2 GBGT1 ST3GAL1 FUT2 FUT1 HEXB NAGA ST8SIA1 B3GALNT1 HEXA

KEGG_GLYCOSPHINGOLIPID_BIOSYNTHESIS_GANGLIO_SERIES http://www.gsea-msigdb.org/gsea/msigdb/cards/KEGG_GLYCOSPHINGOLIPID_BIOSYNTHESIS_GANGLIO_SERIES ST6GALNAC4 ST6GALNAC3 ST6GALNAC6 B4GALNT1 GLB1 ST6GALNAC5 SLC33A1 ST3GAL5 ST3GAL2 ST3GAL1 HEXB ST8SIA1 B3GALT4 ST8SIA5 HEXA

KEGG_RIBOFLAVIN_METABOLISM http://www.gsea-msigdb.org/gsea/msigdb/cards/KEGG_RIBOFLAVIN_METABOLISM ACP4 ENPP3 ENPP1 ACP6 RFK ACP3 FLAD1 TYR MTMR7 MTMR6 PHPT1 MTMR1 ACP1 ACP2 ACP5 MTMR2

KEGG_NICOTINATE_AND_NICOTINAMIDE_METABOLISM http://www.gsea-msigdb.org/gsea/msigdb/cards/KEGG_NICOTINATE_AND_NICOTINAMIDE_METABOLISM NMNAT1 NT5C2 ENPP3 ENPP1 BST1 NNT AOX1 CD38 NT5M NADSYN1 NUDT12 NT5C1B NT5C3A NMRK1 NT5C1A QPRT NAMPT NT5C NMNAT3 PNP NMNAT2 NT5E NADK NNMT

KEGG_PANTOTHENATE_AND_COA_BIOSYNTHESIS http://www.gsea-msigdb.org/gsea/msigdb/cards/KEGG_PANTOTHENATE_AND_COA_BIOSYNTHESIS PPCS ENPP3 PANK3 PPCDC ENPP1 VNN2 VNN1 PANK1 PANK4 UPB1 DPYS COASY DPYD PANK2 BCAT1 BCAT2

KEGG_AMINOACYL_TRNA_BIOSYNTHESIS http://www.gsea-msigdb.org/gsea/msigdb/cards/KEGG_AMINOACYL_TRNA_BIOSYNTHESIS CARS2 DARS2 RARS1 SARS1 VARS2 YARS2 WARS1 AARS1 FARSA HARS1 SARS2 RARS2 NARS1 LARS2 FARSB AARS2 YARS1 NARS2 GARS1 IARS2 KARS1 WARS2 TARS3 LARS1 VARS1 PARS2 MARS2 CARS1 IARS1 TARS2 SEPSECS MTFMT TARS1 DARS1 HARS2 PSTK QARS1 EARS2 EPRS1 FARS2 MARS1

KEGG_BASAL_TRANSCRIPTION_FACTORS http://www.gsea-msigdb.org/gsea/msigdb/cards/KEGG_BASAL_TRANSCRIPTION_FACTORS TBPL2 TAF1L TAF2 TAF4 TAF7L TAF1 TAF9B GTF2A1L STON1 TBP GTF2A1 GTF2B GTF2A2 TAF11 TAF12 TAF5L TAF13 GTF2IRD1 TAF9 TAF10 GTF2H2 TAF7 GTF2H1 TAF6L GTF2F2 GTF2F1 GTF2E2 TAF4B TAF6 GTF2E1 TAF5 GTF2I GTF2H4 TBPL1 GTF2H3

KEGG_NON_HOMOLOGOUS_END_JOINING http://www.gsea-msigdb.org/gsea/msigdb/cards/KEGG_NON_HOMOLOGOUS_END_JOINING XRCC4 MRE11 POLL POLM NHEJ1 LIG4 FEN1 DNTT XRCC5 RAD50 XRCC6 PRKDC DCLRE1C

KEGG_SNARE_INTERACTIONS_IN_VESICULAR_TRANSPORT http://www.gsea-msigdb.org/gsea/msigdb/cards/KEGG_SNARE_INTERACTIONS_IN_VESICULAR_TRANSPORT STX12 STX2 VAMP5 GOSR2 SNAP29 STX7 STX6 STX1A STX11 STX16 VAMP1 VAMP3 BNIP1 STX3 VAMP2 STX10 VAMP7 SNAP47 SEC22B VAMP4 VAMP8 YKT6 SNAP23 BET1L STX18 GOSR1 SNAP25 USE1 STX5 STX4 STX19 VTI1B BET1 VTI1A TSNARE1 STX8 STX1B STX17

KEGG_LYSOSOME http://www.gsea-msigdb.org/gsea/msigdb/cards/KEGG_LYSOSOME PLA2G15 AP3B2 GGA1

SLC11A1 PPT1 MFSD8 CTSZ NAGPA IGF2R ARSG ATP6V0A1 CTSW ATP6V0B LGMN CTSS AP4S1 LAMP2 ATP6AP1 LAPTM5 AP1S3 CLTCL1 AP4B1 AP3B1 LAMP3 ATP6V0A4 CTSL ABCB9 AP1B1 CTSK SLC11A2 GNPTAB SMPD1 CTSH AP1G1 CTSG CTSE LAPTM4A GM2A LAMP1 CTSO GGA2 CTSV AP3M1 CTSC GBA AP3D1 CD164 ATP6V1H HGSNAT ABCA2 DNASE2B AGA AP3M2 ATP6V0D2 ARSA CTSD CTSB ARSB TCIRG1 GNS PPT2 DNASE2 SORT1 ASAH1 AP4E1 GALNS AP4M1 PSAPL1 IDUA IDS ATP6V0C GALC GNPTG NEU1 M6PR GUSB AP3S2 GLA GGA3 ATP6V0A2 FUCA1 NPC2 ATP6V0D1 ENTPD4 SGSH AP1M2 CD68 CD63 GLB1 CTSF MANBA MAN2B1 PSAP CTNS LIPA AP1S2 AP1M1 LAPTM4B NAPSA HEXB AP3S1 SUMF1 MCOLN1 AP1S1 HEXA NPC1 SLC17A5 SCARB2 HYAL1 CLN5 CTSA TPP1 CLTA CLTB CLN3 CLTC ACP2 GAA ACP5 NAGA NAGLU

KEGG_CARDIAC_MUSCLE_CONTRACTION http://www.gsea-msigdb.org/gsea/msigdb/cards/KEGG_CARDIAC_MUSCLE_CONTRACTION CACNA2D1 CACNB1 COX8A CACNB2 UQCR10 COX7C CACNB3 CACNB4 CACNG1 COX6CP3 FXYD2 CACNG8 RYR2 CACNG6 ATP2A2 CACNG7 TPM2 MT-CYB TPM1 ATP1B2 ATP1B3 ATP1A4 ATP1B1 MT-CO2 COX7B MT-CO1 COX7A1 COX7A2 COX8C COX6C MT-CO3 CACNA2D3 COX6B1 CACNA1D SLC8A1 COX5B SLC9A1 UQCR10P1 CACNA1C TNNI3 CACNA1F COX4I1 ATP1A1 CACNA2D2 CACNA1S COX7B2 ATP1A3 TNNC1 ATP1A2 UQCRB COX6A2 SLC9A6 TPM4 ACTC1 COX6A1 CACNA2D4 UQCRH UQCRFS1 UQCRC2 TPM3 UQCRC1 CACNG2 CACNG3 UQCR11 UQCRQ MYL3 TNNT2 MYL2 COX6B2 COX4I2 COX7A2L COX5A CACNG5 CACNG4 UQCRHL CYC1 MYH7 MYH6 ATP1B4

KEGG_RENIN_ANGIOTENSIN_SYSTEM http://www.gsea-msigdb.org/gsea/msigdb/cards/KEGG_RENIN_ANGIOTENSIN_SYSTEM LNPEP ACE2 REN ANPEP CMA1 CTSA CTSG THOP1 AGT AGTR1 ACE AGTR2 NLN ENPEP CPA3 MAS1 MME

KEGG_CIRCADIAN_RHYTHM_MAMMAL http://www.gsea-msigdb.org/gsea/msigdb/cards/KEGG_CIRCADIAN_RHYTHM_MAMMAL NPAS2 PER3 PER2 CSNK1D CRY1 BHLHE41 BHLHE40 NR1D1 CRY2 CSNK1E PER1 CLOCK ARNTL

KEGG_TASTE_TRANSDUCTION http://www.gsea-msigdb.org/gsea/msigdb/cards/KEGG_TASTE_TRANSDUCTION TAS2R60 GRM4

PLCB2 ADCY8 ADCY6 TAS2R42 TAS1R2 TAS1R1 TRPM5 ASIC2 PDE1A CACNA1A ITPR3 CACNA1B TAS2R5 ADCY4 GNAT3 GNG13 TAS2R3 TAS2R9 TAS1R3 TAS2R1 KCNB1 TAS2R16 TAS2R4 SCNN1G GNB3 PRKX GNG3 SCNN1B SCNN1A TAS2R43 TAS2R40 TAS2R41 TAS2R39 TAS2R14 PRKACA PRKACB PRKACG GNAS TAS2R38 GNB1 TAS2R20 TAS2R50 TAS2R45 TAS2R8 TAS2R46 TAS2R7 TAS2R13 TAS2R19 TAS2R10 TAS2R31

KEGG_PROXIMAL_TUBULE_BICARBONATE_RECLAMATION http://www.gsea-msigdb.org/gsea/msigdb/cards/KEGG_PROXIMAL_TUBULE_BICARBONATE_RECLAMATION SLC25A10 GLUD2 PCK2 CA2 CA4 PCK1 GLS2 FXYD2 MDH1 SLC38A3 ATP1A1 GLUD1 ATP1A3 SLC4A4 ATP1A2 GLS ATP1B2 ATP1B3 ATP1A4 ATP1B1 AQP1 ATP1B4 SLC9A3

KEGG_PATHOGENIC_ESCHERICHIA_COLI_INFECTION http://www.gsea-msigdb.org/gsea/msigdb/cards/KEGG_PATHOGENIC_ESCHERICHIA_COLI_INFECTION TUBA1A PRKCA KRT18 WAS CLDN1 TUBB4A TUBB4B TUBB3 RHOA CTTN ROCK1 TUBB2A WASL LY96 ABL1 TUBA1B ARPC4 CD14 ARPC5 ITGB1 ARPC1B ARPC3 ACTG1 ARPC2 NCL TUBB7P TUBA3D NCK1 YWHAQ TLR5 TUBB2B TUBAL3 CTNNB1 TUBA3C TUBB8 TUBA8 TUBA4A NCK2 TUBA1C ACTB ROCK2 TUBB1 HCLS1 EZR ARHGEF2 ARPC1A TLR4 TUBB6 ARPC5L TUBB YWHAZ FYN OCLN CDC42 CDH1 TUBA3E

KEGG_SYSTEMIC_LUPUS_ERYTHEMATOSUS http://www.gsea-msigdb.org/gsea/msigdb/cards/KEGG_SYSTEMIC_LUPUS_ERYTHEMATOSUS CD80 CD86 H2BU1 CD28 MACROH2A1 H3Y2 H2AC1 H2AC7 H2AW H2AC8 FCGR1A H2AC21 H3C14 H2AC19 C9 CTSG H3Y1 H4C9 FCGR3B FCGR2A C8A H3-4 C7 FCGR3A C8G FCGR2B C8B HLA-DRB4 HLA-DRB5 HLA-DRB3 C6 C5 ELANE H4-16 C4B C4A H4C15 HLA-DPA1 H2AB3 HLA-DPB1 HLA-DQA1 HLA-DQA2 HLA-DQB1 H4C14 H2BC3 H2AX H2AZ1 H2BC5 C2 H3-3B C1S C3 C1QB H2AC12 C1QA H3-3A C1R C1QC H2BC12 ACTN2 HLA-DRB1 ACTN3 HLA-DRA H2AC11 HLA-DOA HLA-DOB H3C7 TNF H4C4 FCGR2C H4C12 MACROH2A2 H4C6 H2AZ2 H4C3 H4C11 H4C2 HLA-DMB H4C8 HLA-DMA ACTN4 H4C13 H2BC11 ACTN1 H4C5 H4C7 H2BW1 H2BC21 GRIN2A GRIN2B RO60 TRIM21 H3C4 H3C1 H2BC18 H3C8 H3C11 H3C6 H3C3 H4C1 IL10 H3C2 SSB H3C10 H3C15 H3C12 H2BW2 H2AB1 H3-5 H2AC20 H2BC8 H2AB2 H2AJ SNRPD3 H2ACP1 IFNG H2BC13 H2BC10 H2BC9 H2BC17 H2BC4 H2BC14 H2BC15 H2BC6 H2BC7 CD40LG CD40 SNRPB H2AC13 H3C13 H2AC18 H2AC17 SNRPD1 H2AC4

H2AC6 H2AC16 H2AC14 H2BC1 H2AC15

KEGG_PRIMARY_IMMUNODEFICIENCY http://www.gsea-msigdb.org/gsea/msigdb/cards/KEGG_PRIMARY_IMMUNODEFICIENCY ZAP70 CD4 TNFRSF13C IKBKG IL2RG RFXAP TAP2 RFX5 CD8A CD8B TAP1 ICOS UNG IL7R ADA CD40LG CD40 AICDA BTK BLNK CD19 RAG2 RAG1 CD3D CD3E RFXANK AIRE CD79A TNFRSF13B JAK3 IGLL1 CIITA LCK PTPRC DCLRE1C

KEGG_HYPERTROPHIC_CARDIOMYOPATHY_HCM http://www.gsea-msigdb.org/gsea/msigdb/cards/KEGG_HYPERTROPHIC_CARDIOMYOPATHY_HCM CACNA2D1 CACNB1 CACNB2 CACNB3 CACNB4 CACNG1 TNF ITGA9 IGF1 RYR2 CACNG8 LMNA CACNG6 ATP2A2 CACNG7 ITGA4 TPM2 ITGA3 TPM1 ITGA2B ITGA7 ITGA5 EMD ITGA11 PRKAG2 DMD CACNA2D3 CACNA1D SLC8A1 CACNA1C TNNI3 CACNA1F ITGB3 CACNA2D2 CACNA1S ITGB4 PRKAG3 TNNC1 ITGB5 ITGB6 ITGB7 ITGAV ITGB1 TPM4 ACTC1 ACTG1 CACNA2D4 MYBPC3 TPM3 ITGA10 SGCD SGCG SGCA ITGA8 SGCB CACNG2 DAG1 CACNG3 LAMA2 TGFB2 MYL3 TNNT2 MYL2 TGFB1 PRKAB2 PRKAB1 PRKAA2 ITGB8 PRKAA1 TTN ACTB ITGA6 TGFB3 ITGA2 ITGA1 CACNG5 CACNG4 PRKAG1 DES MYH7 MYH6 ACE IL6

KEGG_ARRHYTHMOGENIC_RIGHT_VENTRICULAR_CARDIOMYOPATHY_ARVC http://www.gsea-msigdb.org/gsea/msigdb/cards/KEGG_ARRHYTHMOGENIC_RIGHT_VENTRICULAR_CARDIOMYOPATHY_ARVC CACNA2D1 CACNB1 CACNB2 CACNB3 CACNB4 CACNG1 ITGA9 CACNG8 RYR2 JUP LMNA CACNG6 ATP2A2 CACNG7 ITGA4 ITGA3 ITGA2B ITGA7 LEF1 ITGA5 EMD ACTN4 ITGA11 DSC2 DSG2 ACTN1 DMD CACNA2D3 CACNA1D SLC8A1 CACNA1C CACNA1F ITGB3 CACNA2D2 CACNA1S ITGB4 ITGB5 ITGB6 ITGB7 ITGAV ITGB1 TCF7 ACTG1 CACNA2D4 PKP2 CTNNA1 CDH2 TCF7L2 ITGA10 SGCD SGCG SGCA ITGA8 SGCB CACNG2 CTNNA2 DAG1 CACNG3 CTNNB1 LAMA2 GJA1 ITGB8 ACTB ITGA6 ITGA2 ITGA1 CACNG5 CACNG4 TCF7L1 DSP DES CTNNA3 ACTN2 ACTN3

KEGG_DILATED_CARDIOMYOPATHY http://www.gsea-msigdb.org/gsea/msigdb/cards/KEGG_DILATED_CARDIOMYOPATHY CACNA2D1 CACNB1 CACNB2 ADCY8 CACNB3 ADCY9 CACNB4 ADCY6 CACNG1 ADCY7 ADCY5 TNF ITGA9 IGF1 RYR2 CACNG8 CACNG6 LMNA CACNG7 ATP2A2 ADRB1 ITGA4 TPM2 ITGA3 TPM1 ITGA2B ITGA7 ITGA5

EMD ITGA11 DMD CACNA2D3 CACNA1D SLC8A1 CACNA1C TNNI3 CACNA1F ITGB3 CACNA2D2 CACNA1S ITGB4 ADCY4 TNNC1 ITGB5 ITGB6 ITGB7 ITGAV ITGB1 TPM4 ACTC1 ACTG1 CACNA2D4 MYBPC3 TPM3 ITGA10 SGCD SGCG SGCA ITGA8 SGCB CACNG2 DAG1 CACNG3 PRKX PLN LAMA2 TGFB2 MYL3 TNNT2 MYL2 TGFB1 ADCY3 ADCY2 ADCY1 ITGB8 TTN ACTB ITGA6 TGFB3 ITGA2 ITGA1 CACNG5 CACNG4 PRKACA PRKACB PRKACG GNAS DES MYH7 MYH6

KEGG_GLYCOLYSIS_GLUCONEOGENESIS http://www.gsea-msigdb.org/gsea/msigdb/cards/KEGG_GLYCOLYSIS_GLUCONEOGENESIS ACSS2 GCK PGK2 PGK1 PDHB PDHA1 PDHA2 PGM2 TPI1 ACSS1 FBP1 ADH1B HK2 ADH1C HK1 HK3 ADH4 PGAM2 ADH5 PGAM1 ADH1A ALDOC ALDH7A1 LDHAL6B PKLR LDHAL6A ENO1 PKM PFKP BPGM PCK2 PCK1 ALDH1B1 ALDH2 ALDH3A1 AKR1A1 FBP2 PFKM PFKL LDHC GAPDH ENO3 ENO2 PGAM4 ADH7 ADH6 LDHB ALDH1A3 ALDH3B1 ALDH3B2 ALDH9A1 ALDH3A2 GALM ALDOA DLD DLAT ALDOB G6PC2 LDHA G6PC PGM1 GPI

KEGG_CITRATE_CYCLE_TCA_CYCLE http://www.gsea-msigdb.org/gsea/msigdb/cards/KEGG_CITRATE_CYCLE_TCA_CYCLE IDH3B DLST PCK2 CS PDHB PCK1 PDHA1 PDHA2 SUCLG2P2 FH SDHD OGDH SDHB IDH3A SDHC IDH2 IDH1 ACO1 ACLY MDH2 DLD MDH1 DLAT OGDHL PC SDHA SUCLG1 SUCLA2 SUCLG2 IDH3G ACO2

KEGG_PENTOSE_PHOSPHATE_PATHWAY http://www.gsea-msigdb.org/gsea/msigdb/cards/KEGG_PENTOSE_PHOSPHATE_PATHWAY RPE RPIA PGM2 PGLS PRPS2 FBP2 PFKM PFKL TALDO1 TKT FBP1 TKTL2 PGD RBKS ALDOA ALDOC ALDOB H6PD RPEL1 PRPS1L1 PRPS1 DERA G6PD PGM1 TKTL1 PFKP GPI

KEGG_PENTOSE_AND_GLUCURONATE_INTERCONVERSIONS http://www.gsea-msigdb.org/gsea/msigdb/cards/KEGG_PENTOSE_AND_GLUCURONATE_INTERCONVERSIONS UGT1A10 UGT1A8 RPE UGT1A7 UGT1A6 UGT2B28 UGT1A5 CRYL1 UGDH UGT2A1 GUSB UGT1A9 DCXR RPEL1 DHDH UGT2B11 UGP2 XYLB UGT2B10 AKR1B1 UGT2B7 UGT2B4 UGT2A3 UGT1A4 UGT2B17 UGT1A1 UGT1A3 UGT2B15

KEGG_FRUCTOSE_AND_MANNOSE_METABOLISM http://www.gsea-msigdb.org/gsea/msigdb/cards/KEGG_FRUCTOSE_AND_MANNOSE_METABOLISM

MPI PMM2 PMM1 FBP2 PFKM GMDS PFKFB4 PFKL MTMR6 TPI1 PHPT1 PFKFB3 FCSK PFKFB2 MTMR1 PFKFB1 AKR1B10 FPGT KHK FBP1 MTMR2 HK2 HK3 HK1 ALDOA ALDOC ALDOB MTMR7 GFUS AKR1B1 SORD GMPPA PFKP GMPPB

KEGG_GALACTOSE_METABOLISM http://www.gsea-msigdb.org/gsea/msigdb/cards/KEGG_GALACTOSE_METABOLISM GCK GALK1 GLB1 GALE B4GALT1 PGM2 LALBA PFKM PFKL MGAM HK2 HK1 HK3 GALT G6PC2 GLA GANC LCT GALK2 G6PC UGP2 PGM1 AKR1B1 B4GALT2 GAA PFKP

KEGG_ASCORBATE_AND_ALDARATE_METABOLISM http://www.gsea-msigdb.org/gsea/msigdb/cards/KEGG_ASCORBATE_AND_ALDARATE_METABOLISM UGT1A10 UGT1A8 UGT1A7 UGT1A6 ALDH1B1 UGT2B28 ALDH2 UGT1A5 MIOX UGDH UGT2A1 ALDH9A1 ALDH3A2 UGT1A9 ALDH7A1 UGT2B11 UGT2B10 UGT2B7 UGT2B4 UGT2A3 UGT1A4 UGT1A1 UGT2B17 UGT1A3 UGT2B15

KEGG_FATTY_ACID_METABOLISM http://www.gsea-msigdb.org/gsea/msigdb/cards/KEGG_FATTY_ACID_METABOLISM CPT1A CPT1C ACADS ALDH1B1 ACADSB ACADL ALDH2 ACADM CYP4A11 ACAT2 ACADVL ACAT1 ACAA2 HADH HADHB HADHA CYP4A22 ADH7 ADH6 ACSL6 ADH1B ADH1C ADH4 ECHS1 ADH5 ALDH9A1 ALDH3A2 ACSL5 ADH1A EHHADH GCDH ALDH7A1 ACOX3 ACSL1 ACAA1 CPT2 CPT1B ACOX1 ECI2 ECI1 ACSL3 ACSL4

KEGG_STEROID_BIOSYNTHESIS http://www.gsea-msigdb.org/gsea/msigdb/cards/KEGG_STEROID_BIOSYNTHESIS SOAT1 LSS SQLE EBP CYP51A1 DHCR7 CYP27B1 DHCR24 HSD17B7 MSMO1 FDFT1 SC5D LIPA CEL TM7SF2 NSDHL SOAT2

KEGG_PRIMARY_BILE_ACID_BIOSYNTHESIS http://www.gsea-msigdb.org/gsea/msigdb/cards/KEGG_PRIMARY_BILE_ACID_BIOSYNTHESIS CYP46A1 SLC27A5 BAAT CYP7B1 AKR1C4 HSD17B4 SCP2 AKR1D1 ACOX2 HSD3B7 CYP27A1 AMACR CYP7A1 CYP8B1 CYP39A1 CH25H

KEGG_STEROID_HORMONE_BIOSYNTHESIS http://www.gsea-msigdb.org/gsea/msigdb/cards/KEGG_STEROID_HORMONE_BIOSYNTHESIS SRD5A3 AKR1C4 CYP3A5 HSD3B2 UGT2B28 HSD3B1 COMT SULT2B1 CYP3A4 CYP7A1 CYP11A1 AKR1C3 HSD11B2 SULT1E1 HSD11B1 HSD17B2 HSD17B3 CYP21A2 HSD17B1 CYP19A1 CYP17A1 CYP11B2 CYP11B1 HSD17B6 HSD17B7 UGT2B11 UGT2A3 UGT1A4 CYP3A7 UGT1A1 UGT1A3 UGT1A10 UGT1A8 UGT1A7 UGT1A6 UGT1A5 HSD17B8 AKR1C2 AKR1C1 CYP1A1

CYP1B1 STS UGT2A1 SRD5A2 SRD5A1 CYP3A43 CYP7B1 UGT1A9 AKR1D1 HSD17B12 UGT2B10 UGT2B7 UGT2B4 UGT2B17 UGT2B15

KEGG_OXIDATIVE_PHOSPHORYLATION http://www.gsea-msigdb.org/gsea/msigdb/cards/KEGG_OXIDATIVE_PHOSPHORYLATION ATP6V1G1 UQCR10 NDUFA5 NDUFA4 COX6CP3 PPA2 ATP5MF NDUFS7 MT-CYB ATP6V0A1 ATP6V1G2 ATP6V0B ATP5PO MT-CO2 MT-CO1 ATP6AP1 COX8C MT-CO3 COX5B COX4I1 ATP6V0A4 ATP12A MT-ATP6 MT-ATP8 ATP5PD NDUFA2 NDUFA3 NDUFA1 ATP5MC1P5 UQCRQ ATP6V1H ATP5F1D LHPP ATP6V0D2 COX7A2L ATP5MC3 ATP6V1C2 ATP5MC2 ATP5MC1 ATP5PB ATP5F1E COX5A TCIRG1 UQCRHL ATP4B ATP6V1D ATP4A ATP5F1A ATP5PF ATP5ME SDHA ATP6V1A ATP6V1E1 NDUFA4L2 ATP6V1B2 ATP6V1B1 ATP6V1C1 ATP6V0C COX10 COX8A COX7C SDHD SDHB SDHC ATP6V1G3 COX7B COX7A1 COX7A2 COX6C COX17 ATP5F1B COX6B1 ATP5F1C UQCR10P1 ATP6V1F COX7B2 ATP6V0A2 UQCRB ATP6V0D1 COX6A2 COX6A1 UQCRH UQCRFS1 UQCRC2 UQCRC1 NDUFB3 NDUFB1 NDUFB2 NDUFA10 NDUFAB1 ATP6V0E2 MT-ND6 NDUFA9 MT-ND5 NDUFA7 NDUFA8 UQCR11 NDUFA6 NDUFV3 ATP6V1E2 MT-ND4 MT-ND4L MT-ND2 COX6B2 PPA1 MT-ND3 COX4I2 MT-ND1 ATP5MG ATP6V0E1 NDUFA11 NDUFB10 NDUFS8 NDUFC1 NDUFC2 NDUFS1 NDUFV2 NDUFB6 NDUFS4 NDUFB7 NDUFV1 NDUFB8 NDUFS6 NDUFB9 NDUFS5 NDUFS2 NDUFB4 NDUFS3 NDUFB5 CYC1 COX11 COX15

KEGG_PURINE_METABOLISM http://www.gsea-msigdb.org/gsea/msigdb/cards/KEGG_PURINE_METABOLISM POLR2G NT5C2 POLR2H ENPP3 POLR2E POLR2F ENPP1 XDH POLR2I POLR2J POLE3 ADSS2 PRPS2 ADSL NME6 POLR1D PNPT1 POLR2K PDE11A POLD3 POLR2L AK1 NME1-NME2 AK2 AK4 POLA2 CANT1 AMPD1 AMPD3 AMPD2 NME4 GUCY2D NME1 ATIC NME2 DGUOK POLR3C NME3 POLR3G POLR3F PRUNE1 ADPRM PDE4B PDE4C PDE3B PDE4A NUDT2 GMPR2 PDE6C PDE6D PDE4D PDE6A PRIM1 PRIM2 AK5 GMPR NME5 PDE7B PDE5A POLD4 GUCY1A2 FHIT ADCY3 ADCY2 ADCY1 POLA1 PDE3A PDE2A PDE1C ADSS1 ADA ENTPD8 POLE2 PDE8A PDE9A PDE1B NUDT9 POLD1 GDA POLD2 POLE PDE6B PDE7A PRPS1 POLR1E PAICS NT5C1A POLR2B POLR3A PDE6H POLR2A PDE6G POLR2D DCK POLR2C ADCY8 ADCY9 ADCY6 AK7 ADCY7 ADCY5 GMPS ADCY10 POLR3K POLR1B PNP RRM2B PDE1A POLR2J2 HPRT1 GART PAPSS1 PAPSS2 NT5M ADCY4 IMPDH1 IMPDH2 POLR3H POLR2J3 POLR1A POLR3B GUCY1B1 PKLR GUCY2C ENTPD4 PKM GUCY1A1 GUK1

GUCY2F ADK ITPA POLR1C URAD POLE4 RRM1 POLR3D RRM2 NT5C3A PDE8B POLR3GL NT5E NPR2 NPR1 PDE10A ENTPD6 ENTPD2 ENTPD5 ENTPD3 NUDT5 ENTPD1 PFAS NT5C1B APRT PRPS1L1 PPAT NME7 NT5C ALLC POLR1H

KEGG_PYRIMIDINE_METABOLISM http://www.gsea-msigdb.org/gsea/msigdb/cards/KEGG_PYRIMIDINE_METABOLISM NT5C2 POLR2G POLR2H POLR2E POLR2F POLR2I POLR2J POLE3 TYMS TXNRD1 NME6 POLR1D PNPT1 POLR2K DHODH POLD3 POLR2L NME1-NME2 POLA2 UPRT CANT1 NME4 NME1 NME2 NME3 POLR3C POLR3G POLR3F NUDT2 PRIM1 PRIM2 NME5 POLD4 DTYMK CTPS1 POLA1 ENTPD8 POLE2 POLD1 POLD2 POLE CAD CMPK2 POLR1E NT5C1A POLR3A DCTD POLR2B POLR2A DCK POLR2D POLR2C UCK1 UPB1 TXNRD2 CMPK1 POLR3K RRM2B PNP POLR1B UPP2 CTPS2 POLR2J2 CDA NT5M DUT POLR3H UCKL1 POLR2J3 POLR1A POLR3B ENTPD4 ITPA POLR1C POLE4 RRM1 RRM2 POLR3D NT5C3A DPYS DPYD TYMP UMPS UCK2 POLR3GL TK1 NT5E TK2 UPP1 ENTPD6 ENTPD5 ENTPD3 ENTPD1 NT5C1B NT5C NME7 AK3 POLR1H

KEGG_ALANINE_ASPARTATE_AND_GLUTAMATE_METABOLISM http://www.gsea-msigdb.org/gsea/msigdb/cards/KEGG_ALANINE_ASPARTATE_AND_GLUTAMATE_METABOLISM GLUD2 GFPT2 AGXT2 CPS1 GLS2 ABAT ADSS2 ADSL GLUD1 GLS ASL IL4I1 GPT GFPT1 GPT2 DDO ADSS1 ACY3 GAD1 GAD2 GOT2 AGXT GLUL NIT2 GOT1 ALDH4A1 CAD ALDH5A1 ASS1 ASNS PPAT ASPA

KEGG_GLYCINE_SERINE_AND_THREONINE_METABOLISM http://www.gsea-msigdb.org/gsea/msigdb/cards/KEGG_GLYCINE_SERINE_AND_THREONINE_METABOLISM ALAS1 ALAS2 GLYCTK MAOB AGXT2 MAOA AOC2 GATM SDS AOC3 GNMT SHMT1 GCAT PHGDH DMGDH SHMT2 SRR PSAT1 BHMT GLDC DLD GAMT PSPH AGXT SARDH AMT CHDH CBS CTH PIPOX DAO

KEGG_CYSTEINE_AND_METHIONINE_METABOLISM http://www.gsea-msigdb.org/gsea/msigdb/cards/KEGG_CYSTEINE_AND_METHIONINE_METABOLISM AMD1 SRM ADI1 AHCY DNMT1 SDS TRDMT1 DNMT3A DNMT3B LDHC IL4I1 SMS LDHB ENOPH1 BHMT GOT2 CDO1 APIP GOT1 LDHAL6B TAT AHCYL1 MTAP MAT2B LDHA MTR CBS MAT1A AHCYL2 CTH MAT2A MPST LDHAL6A DNMT3L

KEGG_VALINE_LEUCINE_AND_ISOLEUCINE_DEGRADATION http://www.gsea-

msigdb.org/gsea/msigdb/cards/KEGG_VALINE_LEUCINE_AND_ISOLEUCINE_DEGRADATION AOX1 ALDH1B1 ACADS ACADSB ABAT ALDH2 ACADM ACAT2 HSD17B10 ACAT1 OXCT1 ACAA2 IL4I1 HADH MCEE HADHB DBT HADHA ALDH6A1 HMGCL IVD BCKDHB ACAD8 ECHS1 ALDH9A1 ALDH3A2 PCCB HIBCH DLD HMGCS2 EHHADH HMGCS1 PCCA ALDH7A1 HIBADH ACAA1 OXCT2 MMUT MCCC2 BCAT1 AUH BCAT2 BCKDHA MCCC1

KEGG_VALINE_LEUCINE_AND_ISOLEUCINE_BIOSYNTHESIS http://www.gsea-msigdb.org/gsea/msigdb/cards/KEGG_VALINE_LEUCINE_AND_ISOLEUCINE_BIOSYNTHESIS PDHB VARS2 IARS2 PDHA1 PDHA2 LARS1 LARS2 VARS1 BCAT1 BCAT2 IARS1

KEGG_LYSINE_DEGRADATION http://www.gsea-msigdb.org/gsea/msigdb/cards/KEGG_LYSINE_DEGRADATION SUV39H2 AASDHPPT DLST ALDH1B1 AASDH ALDH2 ACAT2 PLOD2 PLOD1 TMLHE EHMT2 ACAT1 KMT5A HADH SETMAR OGDH HADHA BBOX1 AASS ECHS1 ALDH9A1 ALDH3A2 KMT5B SUV39H1 ASH1L SETD1B DOT1L NSD2 EHHADH OGDHL SETD1A GCDH KMT5C SETD7 ALDH7A1 NSD3 EHMT1 SETDB1 AADAT SETD2 SETDB2 PLOD3 NSD1 PIPOX

KEGG_ARGININE_AND_PROLINE_METABOLISM http://www.gsea-msigdb.org/gsea/msigdb/cards/KEGG_ARGININE_AND_PROLINE_METABOLISM SRM AZIN2 GLUD2 GLS2 ARG1 ARG2 GLUD1 GLS ASL CKMT1A P4HA2 SMS CKB NOS3 NOS2 NOS1 GOT2 GLUL P4HA1 AOC1 ALDH7A1 LAP3 AGMAT GOT1 PYCR1 ASS1 ALDH18A1 SAT1 NAGS DAO AMD1 MAOB MAOA CPS1 P4HA3 ALDH1B1 GATM ALDH2 OTC PRODH2 OAT CKM ACY1 SAT2 ALDH9A1 ALDH3A2 CKMT1B PYCR3 GAMT PRODH ALDH4A1 CKMT2 PYCR2 ODC1

KEGG_HISTIDINE_METABOLISM http://www.gsea-msigdb.org/gsea/msigdb/cards/KEGG_HISTIDINE_METABOLISM CNDP1 MAOB MAOA ALDH1B1 ALDH2 METTL6 ALDH3A1 BUD23 HAL HNMT DDC ACY3 ALDH1A3 METTL2B ALDH3B1 ALDH3B2 ALDH9A1 ALDH3A2 HEMK1 LCMT2 UROC1 TRMT11 AOC1 ALDH7A1 FTCD AMDHD1 ASPA LCMT1 HDC

KEGG_TYROSINE_METABOLISM http://www.gsea-msigdb.org/gsea/msigdb/cards/KEGG_TYROSINE_METABOLISM MAOB MAOA HPD AOX1 AOC2 TYR HGD METTL6 ALDH3A1 COMT AOC3 BUD23 IL4I1 GSTZ1 DDC ADH7 DBH ADH6 METTL2B ADH1B ALDH1A3 ADH1C ALDH3B1 ADH4 ALDH3B2 ADH5 HEMK1 ADH1A GOT2 LCMT2

TRMT11 GOT1 TYRP1 TAT MIF NAA80 PNMT DCT LCMT1 TPO TH FAH

KEGG_PHENYLALANINE_METABOLISM http://www.gsea-msigdb.org/gsea/msigdb/cards/KEGG_PHENYLALANINE_METABOLISM ALDH1A3 ALDH3B1 ALDH3B2 MAOB MAOA HPD GOT2 AOC2 ALDH3A1 AOC3 PAH GOT1 PRDX6 TAT MIF NAA80 IL4I1 DDC

KEGG_TRYPTOPHAN_METABOLISM http://www.gsea-msigdb.org/gsea/msigdb/cards/KEGG_TRYPTOPHAN_METABOLISM MAOB MAOA IDO2 AOX1 ALDH1B1 AANAT ALDH2 WARS1 IDO1 CAT ACAT2 ACAT1 IL4I1 HADH OGDH TPH1 HADHA DDC AFMID CYP1A1 CYP1A2 CYP1B1 ASMT ECHS1 ALDH9A1 KMO ALDH3A2 WARS2 EHHADH OGDHL GCDH ALDH7A1 AOC1 INMT TDO2 HAAO KYNU AADAT TPH2 ACMSD

KEGG_BETA_ALANINE_METABOLISM http://www.gsea-msigdb.org/gsea/msigdb/cards/KEGG_BETA_ALANINE_METABOLISM CNDP1 SRM ECHS1 ALDH9A1 ALDH3A2 GAD1 GAD2 AOC2 ALDH1B1 HIBCH ABAT EHHADH ALDH2 ACADM UPB1 ALDH7A1 AOC3 DPYS DPYD MLYCD HADHA SMS

KEGG_TAURINE_AND_HYPOTAURINE_METABOLISM http://www.gsea-msigdb.org/gsea/msigdb/cards/KEGG_TAURINE_AND_HYPOTAURINE_METABOLISM GGT7 CSAD GGT5 GGT1 GAD1 BAAT GAD2 GGT6 CDO1 ADO

KEGG_SELENOAMINO_ACID_METABOLISM http://www.gsea-msigdb.org/gsea/msigdb/cards/KEGG_SELENOAMINO_ACID_METABOLISMMETTL6 AHCY BUD23 SEPHS1 GGT1 SEPHS2 METTL2B HEMK1 GGT6 LCMT2 PAPSS1 PAPSS2 TRMT11 MARS2 SCLY AHCYL1 GGT7 GGT5 MAT2B MAT1A LCMT1 CBS AHCYL2 CTH MAT2A MARS1

KEGG_GLUTATHIONE_METABOLISM http://www.gsea-msigdb.org/gsea/msigdb/cards/KEGG_GLUTATHIONE_METABOLISM SRM GGT1 GSTP1 GSTT2 GSTT1 GSTZ1 RRM2B SMS PGD GSTO1 GSTA5 MGST2 LAP3 MGST1 MGST3 GSTA3 GSTM1 GPX7 GSTA4 GPX6 GSTM4 GGCT GSTM3 GSTM2 GSTM5 GSTA1 GSTA2 GSR GSS RRM1 RRM2 GCLC GSTK1 GPX5 TXNDC12 GPX1 GPX2 GPX3 IDH2 GPX4 IDH1 OPLAH GCLM GGT6 ANPEP GGT7 GSTO2 GGT5 G6PD ODC1

KEGG_STARCH_AND_SUCROSE_METABOLISM http://www.gsea-msigdb.org/gsea/msigdb/cards/KEGG_STARCH_AND_SUCROSE_METABOLISM ENPP3 GCK ENPP1 AMY2B UGT2B28 PGM2 UXS1 UGDH MGAM GYS1 GYS2 HK2 HK3 HK1 GBA3 GUSB TREH AMY1A AMY1B AMY1C AMY2A UGT2B11 PYGB PYGM PYGL UGT2A3 UGT1A4 UGT1A1 UGT1A3 UGT1A10 UGT1A8 UGT1A7 UGT1A6 PGM2L1 AGL UGT1A5 SI UGT2A1 GBE1 UGT1A9 G6PC2 GANC G6PC UGP2 PGM1 UGT2B10 UGT2B7 UGT2B4 GAA GPI UGT2B17 UGT2B15

KEGG_AMINO_SUGAR_AND_NUCLEOTIDE_SUGAR_METABOLISM http://www.gsea-msigdb.org/gsea/msigdb/cards/KEGG_AMINO_SUGAR_AND_NUCLEOTIDE_SUGAR_METABOLISM MPI GCK GFPT2 GALK1 GALE PMM2 PGM2 PMM1 AMDHD2 GNPDA2 GMDS UXS1 GNPNAT1 NANS UGDH CYB5R3 GFPT1 FCSK NANP CYB5R1 FPGT HEXB HEXA HK2 HK1 HK3 GALT GNE NAGK CHIT1 UAP1 RENBP CHIA NPL UGP2 GALK2 GFUS PGM1 CMAS PGM3 GNPDA1 GMPPA GPI GMPPB

KEGG_GLYCOSAMINOGLYCAN_BIOSYNTHESIS_CHONDROITIN_SULFATE http://www.gsea-msigdb.org/gsea/msigdb/cards/KEGG_GLYCOSAMINOGLYCAN_BIOSYNTHESIS_CHONDROITIN_SULFATE DSE B3GALT6 CHPF XYLT1 CHSY3 XYLT2 CHST14 B3GAT1 CHSY1 CHST11 CHPF2 CHST15 B4GALT7 CHST7 UST CHST13 CSGALNACT2 B3GAT2 CSGALNACT1 CHST3 CHST12 B3GAT3

KEGG_GLYCOSAMINOGLYCAN_BIOSYNTHESIS_HEPARAN_SULFATE http://www.gsea-msigdb.org/gsea/msigdb/cards/KEGG_GLYCOSAMINOGLYCAN_BIOSYNTHESIS_HEPARAN_SULFATE HS3ST5 EXT2 EXT1 NDST3 XYLT1 XYLT2 B3GAT1 NDST1 GLCE HS3ST3B1 HS2ST1 HS6ST2 B3GAT3 HS3ST1 B3GALT6 HS3ST2 HS6ST1 HS3ST3A1 NDST2 NDST4 HS6ST3 B4GALT7 B3GAT2 EXTL1 EXTL2 EXTL3

KEGG_INOSITOL_PHOSPHATE_METABOLISM http://www.gsea-msigdb.org/gsea/msigdb/cards/KEGG_INOSITOL_PHOSPHATE_METABOLISM PLCB2 PLCB1 INPP1 IPMK PLCD1 PLCB3 PLCB4 MIOX TPI1 INPP5A INPP5B INPP4A INPPL1 SYNJ2 PIP4K2B PLCD3 PIKFYVE IMPA1 IMPA2 PIP5K1A PIP5K1B ISYNA1 INPP5J INPP5K SYNJ1 PLCD4 ITPKA ITPKB ITPK1 CDIPT INPP4B PLCE1 ALDH6A1 PIP5K1C PIK3C3 PIK3C2B PIK3C2G PIP4K2A PIK3C2A PTEN PIP4K2C MINPP1 IPPK PIK3CA PIK3CB PIK3CD PLCG1 PLCG2 PLCZ1 PI4KB PIK3CG INPP5E OCRL PI4KA

KEGG_PYRUVATE_METABOLISM http://www.gsea-msigdb.org/gsea/msigdb/cards/KEGG_PYRUVATE_METABOLISM ACSS2 PCK2 ME3 GRHPR PDHB PCK1 PDHA1 ALDH1B1 PDHA2 HAGH ALDH2 ACAT2 ACAT1 LDHC LDHD HAGHL ACSS1 LDHB ACYP2 ME1 ACYP1 ALDH9A1 ALDH3A2 ME2 DLD MDH2 DLAT MDH1 ALDH7A1 PC GLO1 LDHAL6B LDHA ACACB ACACA PKLR AKR1B1 LDHAL6A PKM ACOT12

KEGG_GLYOXYLATE_AND_DICARBOXYLATE_METABOLISM http://www.gsea-msigdb.org/gsea/msigdb/cards/KEGG_GLYOXYLATE_AND_DICARBOXYLATE_METABOLISM ACO1 GLYCTK MTHFD2 GRHPR CS HAO1 MDH2 MDH1 MTHFD2L PGP MTHFD1L MTHFD1 HAO2 AFMID HYI ACO2

KEGG_PROPANOATE_METABOLISM http://www.gsea-msigdb.org/gsea/msigdb/cards/KEGG_PROPANOATE_METABOLISM ACSS2 ALDH1B1 ABAT SUCLG2P2 ALDH2 ACADM ACAT2 ACAT1 LDHC MCEE HADHA ALDH6A1 ACSS1 LDHB ECHS1 ALDH9A1 ALDH3A2 PCCB HIBCH EHHADH PCCA ALDH7A1 LDHAL6B SUCLG1 MLYCD LDHA SUCLA2 ACACB MMUT SUCLG2 ACACA ACSS3 LDHAL6A

KEGG_BUTANOATE_METABOLISM http://www.gsea-msigdb.org/gsea/msigdb/cards/KEGG_BUTANOATE_METABOLISM AACS PDHB ALDH1B1 ACADS PDHA1 ABAT PDHA2 ALDH2 ACAT2 ACAT1 OXCT1 HADH BDH1 HADHA ACSM1 HMGCL AKR1B10 ECHS1 ALDH9A1 GAD1 ALDH3A2 ACSM3 GAD2 ACSM4 HMGCS2 EHHADH HMGCS1 ALDH7A1 ALDH5A1 BDH2 OXCT2 ACSM2A ACSM5 L2HGDH

KEGG_ONE_CARBON_POOL_BY_FOLATE http://www.gsea-msigdb.org/gsea/msigdb/cards/KEGG_ONE_CARBON_POOL_BY_FOLATE MTHFD2 GART TYMS FTCD ALDH1L1 MTHFS MTFMT AMT MTHFD2L DHFR SHMT1 MTHFD1L MTHFD1 MTR ATIC MTHFR SHMT2

KEGG_FOLATE_BIOSYNTHESIS http://www.gsea-msigdb.org/gsea/msigdb/cards/KEGG_FOLATE_BIOSYNTHESIS QDPR ALPP ALPG DHFR GGH GCH1 FPGS SPR ALPL PTS ALPI

KEGG_RETINOL_METABOLISM http://www.gsea-msigdb.org/gsea/msigdb/cards/KEGG_RETINOL_METABOLISM RPE65 CYP3A5 UGT2B28 CYP4A11 CYP3A4 RDH8 DHRS3 CYP2C18 ADH1B ADH1C ADH4 ADH5 DGAT2 ADH1A BCO1 RDH10 CYP26A1 UGT2B11 CYP2C9 AWAT2 CYP26C1 CYP2C19 CYP2C8 CYP2B6 UGT2A3 CYP2A13 UGT1A4 CYP3A7 UGT1A1 UGT1A3 CYP26B1 DHRS9 UGT1A10 DGAT1 UGT1A8 UGT1A7

UGT1A6 ALDH1A1 RETSAT RDH12 LRAT RDH11 UGT1A5 CYP2A6 CYP2A7 CYP4A22 DHRS4 CYP1A1 CYP1A2 ADH7 ADH6 UGT2A1 ALDH1A2 CYP3A43 DHRS4L2 RDH16 UGT1A9 RDH5 UGT2B10 UGT2B7 PNPLA4 UGT2B4 UGT2B17 UGT2B15

KEGG_PORPHYRIN_AND_CHLOROPHYLL_METABOLISM http://www.gsea-msigdb.org/gsea/msigdb/cards/KEGG_PORPHYRIN_AND_CHLOROPHYLL_METABOLISM ALAS1 COX10 UGT1A10 ALAS2 UGT1A8 UGT1A7 ALAD UGT1A6 CPOX BLVRA UGT2B28 BLVRB UGT1A5 UROS HCCS UGT2A1 GUSB FTMT UGT1A9 MMAB FECH HMOX1 HMOX2 UGT2B11 PPOX FTH1 UGT2B10 UGT2B7 UROD UGT2B4 COX15 HMBS CP UGT2A3 UGT1A4 EARS2 UGT2B17 UGT1A1 EPRS1 UGT2B15 UGT1A3

KEGG_TERPENOID_BACKBONE_BIOSYNTHESIS http://www.gsea-msigdb.org/gsea/msigdb/cards/KEGG_TERPENOID_BACKBONE_BIOSYNTHESIS GGPS1 PDSS1 HMGCS2 IDI2 HMGCS1 MVK ACAT2 ACAT1 FDPS PDSS2 PMVK DHDDS MVD HMGCR IDI1

KEGG_LIMONENE_AND_PINENE_DEGRADATION http://www.gsea-msigdb.org/gsea/msigdb/cards/KEGG_LIMONENE_AND_PINENE_DEGRADATION NAA80 ECHS1 ALDH9A1 ALDH3A2 HADHA ALDH1B1 EHHADH YOD1 ALDH2 ALDH7A1

KEGG_NITROGEN_METABOLISM http://www.gsea-msigdb.org/gsea/msigdb/cards/KEGG_NITROGEN_METABOLISM CA5A CA5B CA6 CA12 CA7 GLUD2 CA2 CPS1 CA3 CA4 GLS2 GLUL CA8 CA14 GLUD1 CA9 CA1 GLS AMT HAL ASNS CA13 CTH

KEGG_SULFUR_METABOLISM http://www.gsea-msigdb.org/gsea/msigdb/cards/KEGG_SULFUR_METABOLISM SULT1E1 PAPSS1 SULT1A4 SULT1A3 PAPSS2 CHST11 SULT1A1 SULT2B1 SUOX BPNT1 CHST13 SULT1A2 CHST12

KEGG_METABOLISM_OF_XENOBIOTICS_BY_CYTOCHROME_P450 http://www.gsea-msigdb.org/gsea/msigdb/cards/KEGG_METABOLISM_OF_XENOBIOTICS_BY_CYTOCHROME_P450 CYP2F1 CYP2E1 AKR1C4 EPHX1 CYP3A5 UGT2B28 CYP3A4 GSTP1 GSTT2 GSTT1 AKR1C3 GSTZ1 CYP2C18 ADH1B CYP2S1 ADH1C ADH4 ADH5 GSTO1 ADH1A GSTA5 MGST2 MGST1 MGST3 GSTA3 GSTM1 UGT2B11 CYP2C9 GSTA4 CYP2C19 GSTM4 CYP2C8 GSTM3 CYP2B6 GSTM2 UGT2A3 UGT1A4 UGT1A1 CYP3A7 UGT1A3 GSTM5 UGT1A10 UGT1A8 UGT1A7 GSTA1 UGT1A6 GSTA2 ALDH3A1 UGT1A5 GSTK1 AKR1C2 AKR1C1 CYP1A1 ADH7 CYP1A2 CYP1B1 ADH6 UGT2A1 ALDH1A3

ALDH3B1 ALDH3B2 CYP3A43 UGT1A9 DHDH GSTO2 UGT2B10 UGT2B7 UGT2B4 UGT2B17 UGT2B15

KEGG_DRUG_METABOLISM_CYTOCHROME_P450 http://www.gsea-msigdb.org/gsea/msigdb/cards/KEGG_DRUG_METABOLISM_CYTOCHROME_P450 CYP2E1 CYP3A5 UGT2B28 CYP3A4 GSTP1 GSTT2 GSTT1 GSTZ1 CYP2C18 CYP2D6 ADH1B FMO4 ADH1C ADH4 ADH5 GSTO1 ADH1A GSTA5 MGST2 MGST1 FMO1 FMO2 MGST3 FMO3 GSTA3 GSTM1 UGT2B11 CYP2C9 FMO5 GSTA4 CYP2C19 GSTM4 CYP2C8 GSTM3 CYP2B6 GSTM2 UGT2A3 CYP2A13 UGT1A4 UGT1A1 CYP3A7 UGT1A3 GSTM5 UGT1A10 UGT1A8 UGT1A7 GSTA1 UGT1A6 MAOB GSTA2 MAOA AOX1 ALDH3A1 UGT1A5 GSTK1 CYP2A6 CYP2A7 ADH7 CYP1A2 ADH6 UGT2A1 ALDH1A3 ALDH3B1 ALDH3B2 CYP3A43 UGT1A9 GSTO2 UGT2B10 UGT2B7 UGT2B4 UGT2B17 UGT2B15

KEGG_DRUG_METABOLISM_OTHER_ENZYMES http://www.gsea-msigdb.org/gsea/msigdb/cards/KEGG_DRUG_METABOLISM_OTHER_ENZYMES XDH CYP3A5 UGT2B28 UPB1 CYP3A4 GMPS UPP2 HPRT1 CDA GUSB IMPDH1 IMPDH2 CES2 UCKL1 UGT2B11 TPMT NAT1 CES5A CYP2A13 UGT2A3 UGT1A4 CYP3A7 UGT1A1 UGT1A3 UGT1A10 UGT1A8 UGT1A7 ITPA UGT1A6 UGT1A5 CYP2A6 CYP2A7 DPYS TYMP DPYD UMPS UCK2 TK1 TK2 UPP1 UGT2A1 CYP3A43 CES1 UGT1A9 NAT2 UGT2B10 UGT2B7 UGT2B4 UCK1 UGT2B17 UGT2B15

KEGG_BIOSYNTHESIS_OF_UNSATURATED_FATTY_ACIDS http://www.gsea-msigdb.org/gsea/msigdb/cards/KEGG_BIOSYNTHESIS_OF_UNSATURATED_FATTY_ACIDS ACOT4 TECR BAAT ELOVL5 ELOVL6 ACOT2 YOD1 ELOVL2 FADS1 FADS2 ACOX3 PECR SCD5 SCD HACD1 ACAA1 HADHA ACOT1 ACOT7 ACOX1 HSD17B12 HACD2

KEGG_ABC_TRANSPORTERS http://www.gsea-msigdb.org/gsea/msigdb/cards/KEGG_ABC_TRANSPORTERS ABCA7 ABCG4 ABCD1 ABCC12 ABCA1 TAP2 ABCA6 TAP1 ABCA5 ABCB8 ABCA10 ABCD4 ABCA3 ABCB11 ABCD3 ABCA2 ABCC2 ABCC11 ABCC6 ABCC3 ABCC9 ABCG2 ABCC4 ABCC5 ABCA12 ABCG1 ABCB4 CFTR ABCB1 ABCC1 ABCD2 ABCB6 ABCB7 ABCA8 ABCA4 ABCC8 ABCC10 ABCB10 ABCB9 ABCA13 ABCA9 ABCB5 ABCG5 ABCG8

KEGG_RIBOSOME http://www.gsea-msigdb.org/gsea/msigdb/cards/KEGG_RIBOSOME RPL35 RPL23 RPL3 RPL3L RPL31 RPL4 RPL5 RPL32 RPS10 RPL34 RPS11 RPS12

RPL36AL RPS13 RPL35A RPL37A RPS15 RPL37 RPL38 RPL14 RPS15A FAU RPSA RPS16 RPL27 RPS17 RPL26 RPL24 RPL29 RPL28 RPL27A UBA52 RPL30 RPL36 RPS20 RPS19 RPS18 RPS4X RSL24D1P11 RPS4Y1 RPS5 RPL22L1 RPL26L1 RPS6 RPLP2 RPL22 RPL23A RPL18 RPL18A RPL19 RPL21 RPS3A RPL10A RPS27L RPS3 RPS2 MRPL13 RPL17 RPL15 RPL36A RPL12 RPL13 RPL41 RPL10 RPL39 RPL11 RPL8 RPL9 RPL13A RPL7A RPS26 RPS27 RPS25 RPS29 RPLP1 RPS27A RPLP0 RPS28 RPS24 RPS9 RSL24D1 RPS23 RPS8 RPS21 RPS7 RPL10L RPL7 RPL6

KEGG_RNA_DEGRADATION http://www.gsea-msigdb.org/gsea/msigdb/cards/KEGG_RNA_DEGRADATION LSM5 MPHOSPH6 MTREX PATL1 EXOSC4 PNPT1 EXOSC2 SKIV2L XRN2 EXOSC5 TTC37 LSM6 EXOSC3 EXOSC1 EDC4 CNOT3 CNOT2 XRN1 PARN C1D PAPOLA DDX6 DCP1A ZCCHC7 ENO1 CNOT4 CNOT7 TENT4A EXOSC8 C1DP3 HSPA9 LSM1 CNOT6L EXOSC6 LSM7 LSM8 CNOT9 LSM4 CNOT1 CNOT10 EXOSC7 LSM3 DIS3 ENO3 ENO2 CNOT6 C1DP2 LSM2 DCP2 HSPD1 EXOSC9 EXOSC10 EDC3 DCPS PAPOLB DCP1B WDR61 CNOT8 PAPOLG

KEGG_RNA_POLYMERASEhttp://www.gsea-msigdb.org/gsea/msigdb/cards/KEGG_RNA_POLYMERASE POLR2G POLR2H POLR2E POLR2F POLR1C POLR2I POLR2J POLR3D POLR1D POLR3K POLR3GL POLR1B POLR2K POLR2J2 POLR2L POLR3H POLR1E POLR2J3 POLR1A POLR3C POLR2B POLR3A POLR3G POLR3B POLR2A POLR3F POLR2D POLR2C POLR1H

KEGG_DNA_REPLICATION http://www.gsea-msigdb.org/gsea/msigdb/cards/KEGG_DNA_REPLICATION DNA2 POLE4 POLE3 PRIM1 PRIM2 POLD4 RFC4 RFC5 RPA1 POLA1 RPA3 POLD3 RNASEH2B RPA2 PCNA RPA4 RNASEH1 RNASEH2C MCM4 POLE2 MCM3 MCM6 MCM5 POLA2 MCM7 POLD1 POLD2 RNASEH2A POLE RFC1 RFC3 RFC2 MCM2 FEN1 LIG1 SSBP1

KEGG_SPLICEOSOME http://www.gsea-msigdb.org/gsea/msigdb/cards/KEGG_SPLICEOSOME EFTUD2 LSM5 SF3A1 HNRNPA1L2 SF3B2 PRPF19 SNRNP27 HNRNPA3 LSM6 USP39 DDX42 NCBP1 SF3B1 MAGOH CDC40 SF3B3 SRSF9 ACIN1 TXNL4A TCERG1 U2AF2 RBM17 PRPF38B THOC1 PLRG1 CCDC12 MAGOHB HSPA8 LSM7 CHERP LSM8 PQBP1 RBMX LSM4 EIF4A3 SF3A3 DDX46

RBM25 PRPF40A THOC3 SNU13 PRPF40B LSM2 SRSF8 SF3A2 SMNDC1 BCAS2 NCBP2 PRPF8 HNRNPA1P60 ALYREF DDX39B CWC15 DDX23 PRPF18 CDC5L SF3B5 SNRNP40 PRPF31 SNW1 DHX15 WBP11 HNRNPU CTNNBL1 HNRNPK TRA2A SNRNP200 HSPA1L HSPA1B RBM22 HSPA2 HSPA1A ZMAT2 PRPF6 DHX16 U2AF1 PRPF38A HNRNPC DDX5 DHX8 HSPA6 DHX38 ISY1 XAB2 SNRPG SNRPE SNRPF SNRPD2 SNRPD3 SART1 SYF2 CRNKL1 U2SURP HNRNPA1 PRPF3 AQR PRPF4 LSM3 SF3B4 PPIH SLU7 BUD31 PHF5A SRSF10 SRSF6 SNRPA RBM8A SRSF7 SNRPA1 HNRNPM SNRPB TRA2B SNRPB2 PUF60 SRSF5 SNRNP70 PCBP1 PPIL1 SF3B6 SRSF4 SRSF3 SNRPD1 THOC2 SNRPC PPIE SRSF2 SRSF1

KEGG_PROTEASOME http://www.gsea-msigdb.org/gsea/msigdb/cards/KEGG_PROTEASOME PSMD11 PSMD12 PSMC1P4 PSMD13 PSMB11 PSMA1 PSMD4 POMP PSMD6 PSMD7 PSMA5 PSMD8 PSMA4 PSMA3 PSMA2 PSMF1 IFNG PSME4 PSME3 PSMB10 PSMB9 PSMD2 PSMD3 PSMC6 PSMD1 PSMC4 PSMB3 PSMC5 PSMD14 PSMB2 PSME1 PSMC3 PSMB5 PSME2 PSMB4 SEM1 PSMC1 PSMB7 PSMC2 PSMB6 PSMB8 PSMA6 PSMA7 PSMB1 PSMA8 PSMA6P4

KEGG_PROTEIN_EXPORT http://www.gsea-msigdb.org/gsea/msigdb/cards/KEGG_PROTEIN_EXPORT SRPRA IMMP1L SRP9P1 SRP9 SRP14 SEC61B SEC63 SRPRB SRP19 SRP54 SEC61G HSPA5 IMMP2L SEC61A2 OXA1L SEC11A SPCS1 SEC11C SPCS2 SEC62 SPCS3 SEC61A1 SRP72 SRP68

KEGG_PPAR_SIGNALING_PATHWAY http://www.gsea-msigdb.org/gsea/msigdb/cards/KEGG_PPAR_SIGNALING_PATHWAY SLC27A5 SLC27A4 SORBS1 ACADL CYP4A11 ACADM APOA2 CD36 FADS2 APOA1 CYP7A1 SLC27A1 SCD CYP8B1 UCP1 DBI NR1H3 ACSL6 ME1 MMP1 GK GK2 APOA5 RXRB RXRG PLTP PDPK1 RXRA ILK APOC3 ACOX2 ACOX3 CYP27A1 OLR1 CPT2 ACAA1 CPT1B ANGPTL4 SLC27A2 LPL FABP1 CPT1A PCK2 FABP4 CPT1C FABP2 PCK1 SCP2 PPARA AQP7 PLIN1 CYP4A22 FABP3 FABP5 FABP6 FABP7 ACSL5 ADIPOQ SLC27A6 HMGCS2 EHHADH UBC PPARG PPARD ACSL1 SCD5 ACOX1 ACSL3 ACSL4

KEGG_BASE_EXCISION_REPAIR http://www.gsea-msigdb.org/gsea/msigdb/cards/KEGG_BASE_EXCISION_REPAIR NEIL2 MPG SMUG1 XRCC1 POLE4 HMGB1 POLE3 POLD4 MBD4 OGG1 UNG

POLD3 PCNA NEIL1 POLE2 PARP4 PARP3 PARP2 POLB APEX1 POLL POLD1 POLD2 POLE NEIL3 TDG APEX2 LIG3 HMGB1P1 NTHL1 HMGB1P40 FEN1 LIG1 MUTYH PARP1

KEGG_NUCLEOTIDE_EXCISION_REPAIR http://www.gsea-msigdb.org/gsea/msigdb/cards/KEGG_NUCLEOTIDE_EXCISION_REPAIR MNAT1 POLE4 ERCC4 POLE3 ERCC3 ERCC6 ERCC5 GTF2H5 POLD4 ERCC2 RFC4 CETN2 RFC5 RPA1 RAD23B RBX1 DDB2 RPA3 POLD3 RPA2 RAD23A PCNA RPA4 DDB1 POLE2 ERCC1 POLD1 POLD2 POLE RFC1 RFC3 RFC2 XPC XPA GTF2H2 GTF2H1 CDK7 LIG1 CUL4A CUL4B ERCC8 CCNH GTF2H4 GTF2H3

KEGG_MISMATCH_REPAIR http://www.gsea-msigdb.org/gsea/msigdb/cards/KEGG_MISMATCH_REPAIR MLH3 POLD1 MLH1 POLD2 RFC1 MSH2 RFC3 RFC2 MSH3 POLD4 PMS2 RFC4 LIG1 RFC5 RPA1 MSH6 RPA3 POLD3 RPA2 PCNA SSBP1 RPA4 EXO1

KEGG_HOMOLOGOUS_RECOMBINATION http://www.gsea-msigdb.org/gsea/msigdb/cards/KEGG_HOMOLOGOUS_RECOMBINATION RAD54L XRCC3 RAD51B NBN RAD52 RAD51D XRCC2 POLD4 BLM EME1 RPA1 RAD51 POLD3 RAD50 RPA3 RPA2 RPA4 RAD51C MRE11 BRCA2 POLD1 POLD2 MUS81 TOP3A RAD54B SEM1 TOP3B SSBP1

KEGG_MAPK_SIGNALING_PATHWAY http://www.gsea-msigdb.org/gsea/msigdb/cards/KEGG_MAPK_SIGNALING_PATHWAY JUN MEF2C ELK4 ELK1 JUND GADD45B MAP3K20 STMN1 RRAS2 MAP3K5 MAP3K1 MAP3K3 MAP3K4 MAP3K7 MAP3K8 AKT1 AKT2 ARRB2 CD14 ARRB1 NRAS DUSP16 CHP2 RASGRP3 NFKB2 NFKB1 MYC NFATC4 MAPK14 FLNC FLNA KRAS FLNB PRKX TRAF6 TGFB2 DUSP1 DUSP2 TGFB1 TRAF2 BDNF TAB2 ECSIT TGFBR2 DUSP7 TGFBR1 DUSP5 DUSP6 DUSP3 TGFB3 PLA2G4E DUSP4 CACNG5 CACNG4 NF1 PLA2G12A NFATC2 RASGRP4 MAP3K2 MAX DUSP10 FGF9 FGF8 FGF7 FGF6 FGF5 FGF3 FGF4 FGF1 FGF2 PTPN5 FGF21 IL1R2 MAPK9 CACNA2D3 MAPK10 MAPK11 RASGRP2 PLA2G2A MAP2K2 PLA2G4A MAP2K3 MECOM PLA2G5 MAPK13 MAP2K1 FGFR2 MAP2K7 RASA2 MAPK8IP2 RASGRF1 MAP2K5 RASGRF2 FGFR4 MAP2K6 MAPK8IP3 MAP3K6 CASP3 MAP3K12 FGFR3 FGFR1 RASA1 FGF14 RPS6KA2 RPS6KA3 FGF17 FGF16 FGF10 GRB2 FGF11 FGF12 FGF13 PLA2G1B RPS6KA1 MAPKAPK3 IKBKG HRAS CACNG2 FGF23 CACNG3

MKNK2 FGF18 STK4 STK3 MAPK8IP1 MOS RAP1A MAPT RAP1B PPP3CB PPP3CA CACNA1H CACNA1G CACNA1I ATF4 TAB1 FOS TAOK2 RPS6KA6 PPP3R2 CACNG8 PPP5C PPP3CC CACNG6 PPP3R1 CACNG7 MAP2K4 ATF2 PDGFRB JMJD7-PLA2G4B MAP4K3 PLA2G6 PLA2G2E PLA2G10 MAP4K4 RPS6KA5 BRAF IKBKB PLA2G4B MAP3K11 CACNA2D2 IL1A PLA2G2F DAXX AKT3 GADD45G FGF20 RELB MAPKAPK5 MAPK12 RELA GNA12 HSPA8 HSPB1 PTPRR LAMTOR3 GADD45A NGF DDIT3 MAP3K14 TAOK1 PDGFA FGF22 PDGFB NLK PDGFRA PRKACA PRKACB PAK1 PRKACG CRK CDC25B CRKL MAP3K13 PLA2G2D CDC42 RASGRP1 CACNA2D1 CACNB1 SRF CACNB2 SOS2 CACNB3 CACNB4 CHUK CACNG1 PRKCB RAF1 PRKCA TNF PAK2 MKNK1 PLA2G3 PRKCG PTPN7 RAPGEF2 HSPA1L CACNA1A HSPA1B RAC2 HSPA2 CACNA1D CACNA1E RAC3 CACNA1B HSPA1A PLA2G12B CACNA1C DUSP8 CACNA1F CACNA1S MAP4K1 TNFRSF1A DUSP9 PPM1A PPM1B MAPK3 RPS6KA4 CACNA2D4 HSPA6 MAPK7 FGF19 RAC1 SOS1 MAPK1 DUSP14 MAP4K2 MAPK8 EGFR MAPKAPK2 EGF RRAS TAOK3 GNG12 NTF4 IL1B MRAS NTF3 IL1R1 TP53 FAS NR4A1 PLA2G2C FASLG NTRK2 NTRK1 CHP1

KEGG_ERBB_SIGNALING_PATHWAY http://www.gsea-msigdb.org/gsea/msigdb/cards/KEGG_ERBB_SIGNALING_PATHWAY JUN SOS2 PAK3 RAF1 PRKCB BTC SHC1 PRKCA ELK1 NRG1 PAK2 MTOR PAK4 MAP2K4 EIF4EBP1 BAD PRKCG NRG3 MAPK9 ERBB4 MAPK10 PTK2 ERBB2 ERBB3 MAP2K2 TGFA BRAF MAP2K1 MAP2K7 ABL1 NRG2 AKT1 ABL2 AKT2 SHC4 RPS6KB1 RPS6KB2 AKT3 NRAS GRB2 AREG STAT5B MAPK3 STAT5A PAK6 SOS1 MYC MAPK1 NCK1 PIK3R5 NRG4 HRAS MAPK8 EGFR GSK3B CBLB KRAS CBL SHC3 CDKN1B CDKN1A EGF EREG ARAF NCK2 PAK5 SRC PIK3R3 CAMK2A CAMK2B CAMK2D CAMK2G PAK1 CBLC CRK PIK3CA PIK3CB CRKL PIK3CD GAB1 PLCG1 PLCG2 SHC2 HBEGF PIK3CG PIK3R1 PIK3R2

KEGG_CALCIUM_SIGNALING_PATHWAY http://www.gsea-msigdb.org/gsea/msigdb/cards/KEGG_CALCIUM_SIGNALING_PATHWAY CALM2 PTGFR BST1 PTGER1 F2R CALM1 PTGER3 ATP2A1 OXTR PPP3R2 P2RX4 TACR3 P2RX5 PPP3CC ADRB3 ATP2A3 P2RX1 PPP3R1 ADRB2 ATP2A2 P2RX3 ADRB1 TACR1 TACR2 SPHK1 CCKAR CCKBR NTSR1 PDGFRB VDAC2P5 SLC8A1 ITPR3 SLC8A3 PLCD3 EDNRA P2RX7 CHP2 DRD1 DRD5 TRHR EDNRB PHKB PHKA2 PHKA1 GNA15 GNA11

VDAC1 VDAC2 CHRM1 VDAC3 CHRM2 PRKX PLN BDKRB1 MYLK
PLCE1 CHRM3 ADCY3 CALML5 LTB4R2 ADCY2 ADCY1 BDKRB2 PDE1C
CYSLTR1 GNA14 PDE1B CAMK4 CAMK2A CAMK2B PDGFRA CAMK2D
CAMK2G PRKACA PRKACB GNAQ SLC25A6 PRKACG GNAS SLC25A5
CALML3 SLC25A4 GNAL ATP2B2 ATP2B3 ATP2B4 PLCG1 PLCG2 PLCZ1
AGTR1 ATP2B1 CALM3 PTK2B TRPC1 SLC25A31 PLCB2 GRM5
ADCY8 RYR1 PLCB1 ADCY9 PLCD1 PRKCB ADCY7 PRKCA PLCB3
PLCB4 TNNC2 RYR2 RYR3 GRM1 PPID PHKG1 PHKG2 PRKCG
PDE1A ERBB4 CACNA1A GRIN1 GRIN2A ERBB2 ERBB3 CHRNA7 NOS3
CACNA1D GRIN2C CACNA1E GRIN2D CHRM5 CACNA1B NOS2 CACNA1C NOS1
MYLK2 CACNA1F CACNA1S ADCY4 TNNC1 PLCD4 P2RX2 ITPKA
ADORA2A ITPKB EGFR ADORA2B ITPR1 ITPR2 HTR4 SPHK2 HTR5A
HTR6 HTR7 HTR2A HTR2C HTR2B P2RX6 SLC8A2 AVPR1A
AVPR1B HRH2 ADRA1D ADRA1B ADRA1A GRPR CD38 PPP3CB PTAFR
PPP3CA CACNA1H CACNA1G CACNA1I CYSLTR2 LHCGR TBXA2R MYLK3 HRH1
CHP1 CALML6

KEGG_CYTOKINE_CYTOKINE_RECEPTOR_INTERACTION http://www.gsea-msigdb.org/gsea/msigdb/cards/KEGG_CYTOKINE_CYTOKINE_RECEPTOR_INTERACTION CCL26 TNFSF13 HGF CCL3L1 TNFSF12 TNFRSF8 TNFSF10 CCL2 TNFSF8
TNFSF9 CCL3 TNFSF14 IL21R CCL5 CCL4 CCL13 IL17RB CCL11
CCL8 CCL7 TNFRSF17 TNFSF11 IL23A CTF1 FLT1 CCL14
FLT3 CCL15 FLT3LG FLT4 IL22 CD27 CCL18 CCL17 CCL20
CCL19 CCL21 CCL23 CCL22 TPO CCL16 CSF2 CSF1R TNFRSF13C
IL13RA1 CSF1 PRLR PRL TNFRSF14 IFNE CCL24 CSF2RB
CSF2RA TGFB2 TGFB1 CX3CL1 IL23R XCL1 CXCL5 CXCL11 CXCL6
CCR2 CCL25 CSF3 TGFBR2 TGFBR1 MET TGFB3 CXCR3 CSF3R
CLCF1 IL17B CXCL12 CCR10 OSMR XCR1 IL20 INHBE
PLEKHO2 CXCL10 MPL CCL27 INHBB INHBC IFNLR1 INHBA KITLG
TNFRSF19 CXCR4 IL1R2 KIT CD70 TNFRSF11B LEPR CXCR6
RELT LEP IL19 LTA IL17A IL18 TNFRSF9 OSM GDF5 IL15RA
IL15 CXCL13 LTB TNFRSF25 LTBR IL22RA1 CD40LG CD40
PPBPP1 GHR IL18RAP CXCL9 IL17RA GH1 LIF GH2 LIFR IL18R1
TNFSF4 TNFRSF4 CX3CR1 CCL3L3 PDGFRB EDA CXCL14 IL1A EDAR
IL24 CNTF CNTFR TNFSF13B PDGFC VEGFA VEGFC VEGFB
CCR9 NGFR IL22RA2 PDGFA ACVRL1 PDGFB CCL4L2 ACVR2B
PDGFRA ACVR2A ACVR1B ACVR1 VEGFD TNFRSF13B IL25 TNFSF15
EPO IL9R CXCL1 TNF CXCR5 IFNA5 IFNA4 IFNA2 TNFRSF6B
IFNA1 CXCR2 CXCR1 IL9 CCL28 IL7R CXCL8 IL12RB1 IL12B

IL13 IL12RB2 IL11RA IL12A KDR TNFRSF1B IFNA17 TNFRSF1A IFNA21 TNFRSF18 IFNA6 CCR3 TNFRSF12A IFNA7 CCR4 IFNA8 CCR5 IFNA10 CCR6 IFNA13 CCR7 IFNA14 CCR8 IFNA16 IL26 CXCL16 IL10 EDA2R IL10RA IL10RB EPOR IL11 IL3RA IL3 IL2RG EGFR IL2RB CCR1 XCL2 IFNGR2 EGF CCL1 TNFRSF10A IFNG IFNGR1 TNFRSF10D IFNB1 TNFRSF11A TNFRSF10B IFNAR1 TNFRSF10C IFNAR2 IL1RAP IL1B AMHR2 IL1R1 AMH IL2RA IL20RB IL21 TNFSF18 IL2 IL20RA BMPR2 BMPR1A IL6R BMPR1B CRLF2 IL6ST IFNK IL7 PF4 CXCL2 CXCL3 BMP2 PF4V1 BMP7 IFNL3 IFNL1 TNFRSF21 FAS IFNL2 FASLG IFNW1 PPBP TSLP IL4 IL4R IL5 IL5RA IL6

KEGG_CHEMOKINE_SIGNALING_PATHWAY http://www.gsea-msigdb.org/gsea/msigdb/cards/KEGG_CHEMOKINE_SIGNALING_PATHWAYCCL26 STAT3 STAT1 STAT2 CCL3L1 CCL2 GNB4 CCL3 GRK3 GRK2 CCL5 CCL4 CX3CR1 CCL13 CCL11 CCL8 CCL7 CCL3L3 ROCK1 PTK2 BRAF CXCL14 VAV1 CCL14 CCL15 IKBKB AKT1 AKT2 JAK3 SHC4 DOCK2 JAK2 CCL18 GRK7 ARRB2 CCL17 ARRB1 CCL20 VAV2 CCL19 AKT3 NRAS CCL21 CCL23 TIAM1 CCL22 GNG13 STAT5B NFKB1 GRK1 NFKBIB NFKBIA CCL16 PIK3R5 RELA GNG7 GNG5 KRAS SHC3 FGR GNB3 GNB2 CCL24 PRKX GNG4 GNG3 GNAI1 GNG10 GNAI2 GNG11 GNGT1 CX3CL1 GNGT2 XCL1 CXCL5 ADCY3 CXCL11 GNG2 ADCY2 CXCL6 ADCY1 CCR9 CCR2 CCL25 PXN CXCR3 CSK CCL4L2 FOXO3 PRKACA BCAR1 PRKACB GNB5 PRKACG PAK1 GNAI3 CRK CXCL12 CCR10 GNB1 CRKL XCR1 PTK2B CDC42 PLCB2 SOS2 ADCY8 CXCL10 PLCB1 ADCY9 ADCY6 CHUK RAF1 PRKCB ADCY7 CXCL1 SHC1 PLCB3 PLCB4 ADCY5 CXCR5 CCL27 WAS PREX1 RHOA CXCR4 CXCR2 GNG8 CXCR1 CCL28 PRKCD HCK CXCL8 RAC2 WASL RASGRP2 MAP2K1 ADCY4 CCR3 CCR4 CXCR6 CCR5 PRKCZ CCR6 CCR7 CCR8 GRB2 MAPK3 CXCL16 RAC1 SOS1 MAPK1 IKBKG HRAS GSK3B CCR1 GSK3A XCL2 ITK CCL1 CXCL13 GNG12 ROCK2 RAP1A PIK3R3 RAP1B TIAM2 PF4 PPBPP1 CXCL2 CXCL3 PF4V1 GRK6 PIK3CA PIK3CB CXCL9 PIK3CD NCF1 ELMO1 SHC2 PPBP GRK4 PARD3 GRK5 PIK3CG PIK3R1 VAV3 LYN PIK3R2

KEGG_PHOSPHATIDYLINOSITOL_SIGNALING_SYSTEM http://www.gsea-msigdb.org/gsea/msigdb/cards/KEGG_PHOSPHATIDYLINOSITOL_SIGNALING_SYSTEM PLCB2 CALM2 INPP1 PLCB1 PLCD1 PRKCB PRKCA PLCB3 CALM1

PLCB4 DGKZ INPP5A DGKE INPP5B DGKD INPP4A PRKCG INPPL1 DGKH INPP5D SYNJ2 PIP4K2B ITPR3 PLCD3 PIKFYVE IMPA1 IMPA2 PIP5K1A PIP5K1B INPP5J CDS1 INPP5K SYNJ1 PLCD4 ITPKA PIK3R5 ITPKB ITPK1 CDIPT ITPR1 ITPR2 DGKQ CDS2 INPP4B DGKB DGKG DGKA DGKI PLCE1 CALML5 PIP5K1C PIK3C3 PIK3C2B PIK3C2G PIP4K2A PIK3C2A PTEN PIK3R3 PIP4K2C IPPK CALML3 PIK3CA PIK3CB PIK3CD PLCG1 PLCG2 PLCZ1 PI4KB CALM3 INPP5E PIK3CG PIK3R1 OCRL PIK3R2 CALML6 PI4KA

KEGG_NEUROACTIVE_LIGAND_RECEPTOR_INTERACTION http://www.gsea-msigdb.org/gsea/msigdb/cards/KEGG_NEUROACTIVE_LIGAND_RECEPTOR_INTERACTION PTGFR PTGER2 PTGER1 PTGER4 PTGER3 CALCRL TACR3 PTGIR ADRB3 ADRB2 HRH4 ADRA2C ADRB1 TACR1 ADRA2A ADRA2B TACR2 GPR35 THRB THRA PTH2R PTH1R PRSS3 PRSS2 NPY5R PRSS1 CGA NPY1R NPY2R RXFP1 MCHR1 LPAR4 DRD1 DRD2 DRD3 DRD4 DRD5 TRHR NMUR2 SSTR2 SSTR3 SSTR1 PRLR PRL C5AR1 UTS2R BDKRB1 BDKRB2 NPBWR2 NPBWR1 PRLHR CSH1 NPFFR2 TAAR6 NTSR2 APLNR NMUR1 GPR83 GABRQ PLG C3AR1 AGTR1 GALR1 AGTR2 SSTR5 SSTR4 ADCYAP1R1 TSHR GRIK1 TSHB GRIK2 GRIK3 GRIA4 GRID1 GRID2 MC1R TSPO S1PR5 MC3R MC2R GABRR2 GRIA3 TRPV1 GRIA2 GRIA1 MTNR1A LEPR MTNR1B LEP MC4R GABRD MC5R GABRE GABRG1 GABRG2 GABRG3 GABRP GABRR1 GABRB3 ADORA1 GABRB2 ADORA2A GABRB1 FPR3 FPR2 FPR1 ADORA2B HTR4 HTR5A HTR6 HTR7 HTR2A GALR3 HTR1F HTR2C OPRL1 GIPR HTR2B OPRM1 GALR2 OPRD1 OPRK1 GABRA5 GABRA6 AVPR2 GABRA3 GABRA4 AVPR1A GABRA1 AVPR1B GABRA2 HRH2 PTGDR GABBR1 ADRA1D ADRA1B ADRA1A GHR FSHB LPAR3 GHRHR PTAFR GHSR MLNR LHB FSHR LHCGR GH1 GH2 ADORA3 GRIN3A HRH1 GRIN3B MAS1 F2 F2R GCGR CRHR2 P2RY10 GLRA2 OXTR GLRA1 GLP1R GLP2R CRHR1 GPR50 P2RX4 P2RX5 P2RX1 GLRB P2RX3 NPY4R CCKAR BRS3 F2RL1 TAAR1 CCKBR MCHR2 F2RL2 NTSR1 NMBR TAAR9 LPAR6 P2RY4 P2RY6 S1PR1 P2RY11 LPAR1 S1PR3 RXFP2 EDNRA HTR1D P2RY1 HTR1B P2RX7 HTR1E CTSG P2RY2 HTR1A GZMA EDNRB CHRM1 CHRNA10 CHRM2 CHRM4 CHRM3 LTB4R2 TAAR8 CNR2 CNR1 CYSLTR1 CALCR GNRHR GABBR2 TAAR5 LPAR2 VIPR2 VIPR1 GPR156 TAAR2 GRM4 GRM5 GRM2 KISS1R GRM3 GRM8 CHRNG CHRNE GRM6 CHRND HRH3 GRM7 GRM1 CHRNA9 F2RL3 CHRNA6 CHRNA5 GRIK5 CHRNA4 GRIN1 GRIN2A CHRNA7 GRIN2B CHRNA1 GRIN2C CHRM5 GRIN2D CHRNA3

CHRNA2 NR3C1 S1PR2 LTB4R GRIK4 P2RY8 CHRNB1 CHRNB2 CHRNB3 CHRNB4 P2RX2 S1PR4 P2RY13 SCTR P2RX6 P2RY14 NPFFR1 GRPR GLRA3 CYSLTR2 TBXA2R PARD3 HCRTR1 HCRTR2

KEGG_CELL_CYCLE http://www.gsea-msigdb.org/gsea/msigdb/cards/KEGG_CELL_CYCLE CDC16 CDC7 CDC45 GADD45B DBF4 ANAPC1 CREBBP MDM2 ABL1 SMC1B SKP1P2 GADD45G ATM ATR ANAPC7 RBL2 ANAPC5 RBL1 MYC CDC14B SMC1A CDC14A SKP1 TGFB2 TGFB1 GADD45A STAG1 PLK1 RBX1 STAG2 TGFB3 MCM4 ORC6 CCND1 MAD1L1 MCM3 MCM6 MCM5 YWHAB CCNA2 MCM7 BUB1 CHEK1 WEE2 MCM2 PTTG1 CDC27 CDC25B CDC25C CDC25A CDC6 CDC20 BUB3 YWHAZ CCND2 YWHAH CCNB1 YWHAG YWHAE CCNE1 ORC3 CCND3 PTTG2 SFN E2F1 TFDP1 ZBTB17 CDK1 ESPL1 ANAPC10 RAD21 BUB1B ANAPC11 RB1 SKP2 CUL1 SMAD3 SMAD4 ANAPC2 TFDP2 PRKDC MAD2L1 ANAPC4 SMAD2 YWHAQ CHEK2 CDC23 EP300 GSK3B CDKN2A CDKN1C CDKN1B CDKN1A CDKN2D CCNA1 CDKN2B CDKN2C FZR1 SMC3 ANAPC13 PCNA TTK PKMYT1 CDK2 CDC26 E2F5 CDK4 WEE1 E2F4 E2F3 TP53 E2F2 ORC1 ORC2 CCNE2 CDK6 ORC4 CCNB2 CDK7 MAD2L2 ORC5 HDAC1 HDAC2 CCNH CCNB3

KEGG_OOCYTE_MEIOSIS http://www.gsea-msigdb.org/gsea/msigdb/cards/KEGG_OOCYTE_MEIOSIS CDC16 CALM2 CALM1 IGF1 RPS6KA6 PPP3R2 PPP3CC SLK PPP3R1 IGF1R ANAPC1 SPDYC ITPR3 SMC1B SKP1P2 CHP2 ANAPC7 ANAPC5 FBXO43 SMC1A REC8 MAPK12 BTRC PRKX SKP1 FBXW11 CALML5 ADCY3 ADCY2 PLK1 ADCY1 RBX1 STAG3 PGR YWHAB CAMK2A BUB1 CAMK2B CAMK2D CAMK2G PRKACA PRKACB PRKACG FBXO5 SGO1 PTTG1 CALML3 CDC27 CDC25C PLCZ1 CDC20 YWHAZ YWHAH CALM3 CCNB1 YWHAG YWHAE CCNE1 PTTG2 ADCY8 ADCY9 ADCY6 ADCY7 ADCY5 SPDYA CDK1 ESPL1 ANAPC10 INS ANAPC11 MAP2K1 ADCY4 RPS6KA2 RPS6KA3 CUL1 PPP1CC PPP1CB PPP1CA CPEB1 MAPK3 ANAPC2 MAD2L1 ANAPC4 MAPK1 RPS6KA1 YWHAQ CDC23 ITPR1 ITPR2 AURKA PPP2CB SMC3 PPP2CA PPP2R1A AR PPP2R1B ANAPC13 MOS PKMYT1 CDK2 CDC26 PPP3CB PPP3CA PPP2R5A CCNE2 CCNB2 PPP2R5E MAD2L2 PPP2R5D PPP2R5C PPP2R5B CHP1 CALML6

KEGG_P53_SIGNALING_PATHWAY http://www.gsea-msigdb.org/gsea/msigdb/cards/KEGG_P53_SIGNALING_PATHWAY CASP9 CASP8 SFN TSC2 IGF1 CDK1 GADD45B RCHY1 ZMAT3 IGFBP3 RRM2B

ADGRB1 TP73 SERPINB5 RPRM PPM1D BID MDM4 BAX MDM2 TP53AIP1 CASP3 TP53I3 PMAIP1 PIDD1 COP1 PERP GADD45G EI24 SESN2 ATM ATR SESN3 CHEK2 APAF1 CDKN2A RRM2 CDKN1A BBC3 GADD45A TNFRSF10B DDB2 GTSE1 SIAH1 CCND1 CD82 PTEN CDK2 CHEK1 SESN1 CDK4 SERPINE1 TP53 CCNE2 FAS CDK6 CYCS STEAP3 CCNB2 CCND2 SHISA5 CCNB1 CCNG1 CCNE1 CCNB3 THBS1 CCNG2 CCND3

KEGG_UBIQUITIN_MEDIATED_PROTEOLYSIS http://www.gsea-msigdb.org/gsea/msigdb/cards/KEGG_UBIQUITIN_MEDIATED_PROTEOLYSIS CDC16 KLHL13 PIAS3 UBA6 UBE2L6 BIRC3 HERC1 XIAP PML HERC2 PRPF19 ANAPC1 UBE2S UBE2O UBE2M UBE2Q2 MAP3K1 UBE4A BRCA1 MDM2 UBE2K UBE2D4 UBOX5 KLHL9 UBR5 UBE3A UBE2R2 COP1 TRIM37 FBXW8 UBE2E3 SKP1P2 ANAPC7 ANAPC5 UBE2QL1 PIAS1 SMURF1 STUB1 UBE2G2 BTRC UBE2G1 UBE2I SOCS1 UBE2H UBE2J2 SKP1 TRAF6 FBXW11 UBE2L3 NHLRC1 UBE2N UBE4B MGRN1 RBX1 DDB2 NEDD4L SOCS3 SIAH1 DDB1 DET1 RNF7 BIRC2 UBE2U UBE2Z UBA7 VHL UBA1 UBE2NL PIAS4 AIRE FBXO4 CBLC UBE2A CDC27 HUWE1 UBE2B CDC34 UBE2D1 UBA3 UBE2E1 UBE2E2 UBE2D2 CDC20 UBE2D3 ITCH UBE2Q1 ANAPC10 RCHY1 UBE2W WWP1 PPIL2 ELOC UBE3C UBE2J1 ANAPC11 ELOB SAE1 UBA2 RHOBTB2 CUL5 KEAP1 SYVN1 PRKN SKP2 PIAS2 CUL7 CUL1 FBXW7 CUL2 CUL3 UBE3B CUL4A CUL4B ANAPC2 FANCL ANAPC4 SMURF2 CDC23 CBLB CBL NEDD4 TRIM32 FZR1 TRIP12 UBE2F ANAPC13 FBXO2 HERC4 WWP2 UBE2C CDC26 HERC3 BIRC6 ERCC8 MID1

KEGG_REGULATION_OF_AUTOPHAGY http://www.gsea-msigdb.org/gsea/msigdb/cards/KEGG_REGULATION_OF_AUTOPHAGY ATG4A ULK3 BECN1 IFNA5 IFNA4 IFNA2 GABARAPL1 ATG12 IFNG IFNA1 ATG4B PRKAA2 PRKAA1 GABARAPL2 PIK3C3 INS ATG5 ULK1 PIK3R4 ATG7 IFNA17 IFNA21 IFNA6 IFNA7 IFNA8 IFNA10 IFNA13 IFNA14 ULK2 IFNA16 ATG4C ATG4D BECN2 ATG3 GABARAP

KEGG_ENDOCYTOSIS http://www.gsea-msigdb.org/gsea/msigdb/cards/KEGG_ENDOCYTOSIS F2R EPN3 IQSEC1 IQSEC3 GRK3 GRK2 ADRB3 ADRB2 EPN2 STAMBP ADRB1 IGF1R CLTCL1 HGS VPS37C MVB12B PIP4K2B RUFY1 SMAP2 HLA-G FLT1 STAM MDM2 PIKFYVE VPS37D PIP5K1A VPS28 CHMP2B MVB12A

PIP5K1B RAB11FIP1 ACAP1 ARRB2 AP2A2 GRK7 ARRB1 AP2B1 AP2A1 EEA1 GRK1 CSF1R SMURF1 SH3GLB1 VPS4A ARFGAP3 DNM2 HSPA8 VPS36 PARD6B RAB31 RAB11A TRAF6 CHMP4A PDCD6IP LDLR PIP5K1C NEDD4L ACAP3 CHMP5 ARAP2 IQSEC2 HLA-F MET HLA-E ARAP3 ASAP2 RAB11B RAB11FIP5 RBSN PDGFRA AGAP1 DNM3 AGAP2 ARFGAP1 ARAP1 CHMP4B CBLC ACAP2 VPS25 RAB22A EHD1 PLD1 PLD2 ITCH CDC42 SH3GLB2 PARD6A CHMP6 VTA1 RNF41 DNAJC6 ARF6 CHMP1B PSD2 USP8 PSD4 WWP1 HLA-C CXCR4 HLA-B PRKCI CXCR2 SH3GL3 CXCR1 TSG101 HLA-A ERBB4 HSPA1L HSPA1B ERBB3 HSPA2 RAB11FIP4 SH3GL1 EPS15 DNM1L HSPA1A TFRC SH3GL2 SMAP1 RAB11FIP2 FGFR2 KDR KIT FGFR4 GIT2 DNM1 FGFR3 LDLRAP1 RET CCR5 PRKCZ PSD3 ASAP3 HSPA6 RAB5C DAB2 SMURF2 HRAS CHMP3 VPS37A EGFR IL2RG VPS37B IL2RB CBLB CBL PSD NEDD4 EGF PARD6G SH3KBP1 ARFGAP2 RAB5B RAB4A EHD4 RAB5A AP2S1 EHD2 IL2RA EHD3 AP2M1 SNF8 SRC VPS4B STAM2 CHMP2A GIT1 GRK6 RABEP1 VPS45 RAB11FIP3 CLTA CLTB CLTC CHMP4C GRK4 PARD3 GRK5 ASAP1 NTRK1 EPN1

KEGG_PEROXISOME http://www.gsea-msigdb.org/gsea/msigdb/cards/KEGG_PEROXISOME EPHX2 XDH BAAT MVK CAT NUDT19 PMVK PEX16 HAO2 FAR2 PRDX5 HMGCL PHYH DDO ACSL6 AGPS SOD1 SOD2 MPV17L NOS2 HAO1 HSD17B4 CRAT PAOX ACOX2 PEX11G ACOX3 AMACR PEX5 MLYCD ACOT8 ACAA1 SLC27A2 SLC25A17 MPV17 PIPOX FAR1 DAO ABCD1 DECR2 SCP2 HACL1 GSTK1 PEX1 NUDT12 ABCD4 ABCD3 PEX11B PEX19 ECH1 IDH2 IDH1 DHRS4 GNPAT PEX2 PXMP2 PEX26 PEX3 PEX6 PXMP4 PEX10 ABCD2 PEX7 ACSL5 PEX13 PEX12 EHHADH PEX14 AGXT PRDX1 PECR ACSL1 PEX11A ACOX1 ECI2 CROT ACSL3 ACSL4

KEGG_MTOR_SIGNALING_PATHWAY http://www.gsea-msigdb.org/gsea/msigdb/cards/KEGG_MTOR_SIGNALING_PATHWAY TSC2 IGF1 RPS6KA6 MTOR EIF4B EIF4E EIF4EBP1 RICTOR INS PGF ULK1 RHEB BRAF HIF1A PDPK1 RPTOR AKT1 AKT2 RPS6KA2 RPS6KA3 RPS6KB1 MLST8 AKT3 RPS6KB2 TSC1 ULK2 CAB39 MAPK3 CAB39L RPS6 MAPK1 RPS6KA1 PIK3R5 ULK3 EIF4E1B STK11 EIF4E2 VEGFA PRKAA2 VEGFC PRKAA1 VEGFB STRADA PIK3R3 VEGFD DDIT4 PIK3CA PIK3CB PIK3CD PIK3CG PIK3R1 PIK3R2

KEGG_APOPTOSIS http://www.gsea-msigdb.org/gsea/msigdb/cards/KEGG_APOPTOSIS CASP10 CASP9 CASP8 CASP7 CHUK PRKAR2B TNF TNFSF10 BIRC3 XIAP PPP3R2 PPP3CC PPP3R1 MYD88 FADD CFLAR RIPK1 BAD IRAK4 BID BAX IKBKB CASP6 IL1A AKT1 CASP3 AKT2 TNFRSF1A AKT3 CHP2 ATM ENDOG NFKB1 NFKBIA CAPN2 PIK3R5 IKBKG CAPN1 IL3RA IL3 RELA ENDOD1 APAF1 PRKX CSF2RB TNFRSF10A TRAF2 TNFRSF10D NGF TNFRSF10B TNFRSF10C MAP3K14 IL1RAP IL1B IRAK2 IL1R1 IRAK1 TRADD PIK3R3 BCL2 BCL2L1 BIRC2 IRAK3 PRKACA PRKACB PRKACG PPP3CB TP53 PPP3CA PIK3CA PIK3CB FAS DFFA CYCS DFFB PIK3CD PRKAR1A FASLG PRKAR2A PRKAR1B EXOG PIK3CG AIFM1 NTRK1 PIK3R1 PIK3R2 CHP1

KEGG_VASCULAR_SMOOTH_MUSCLE_CONTRACTION http://www.gsea-msigdb.org/gsea/msigdb/cards/KEGG_VASCULAR_SMOOTH_MUSCLE_CONTRACTION CALM2 CALD1 CALM1 CYP4A11 CALCRL PTGIR PPP1R14A JMJD7-PLA2G4B ROCK1 PLA2G6 PLA2G2E IRAG1 PLA2G10 BRAF ITPR3 PLA2G4B MYL6B PLA2G2F EDNRA GNA12 GNA11 GNA13 PRKX GUCY1A2 MYLK ADCY3 MYL6 CALML5 ADCY2 ADCY1 PLA2G4E KCNMB3 PRKACA GNAQ PRKACB PRKACG GNAS CALML3 PLA2G2D PLA2G12A AGTR1 MYH11 CALM3 PLCB2 KCNMB2 ADCY8 PLCB1 ADCY9 PPP1R12B ADCY6 ADCY7 PRKCB RAF1 PLCB3 PRKCA ADCY5 PLCB4 MYL9 ARHGEF11 KCNMB1 KCNMA1 RHOA PLA2G3 PPP1R12A PRKCG PRKCH PRKCD PRKCE PLA2G2A MAP2K2 CACNA1D PLA2G4A PRKCQ PLA2G5 PLA2G12B MAP2K1 CACNA1C ARHGEF12 MYLK2 CACNA1F CACNA1S ADCY4 PPP1CC PPP1CA PPP1CB GUCY1B1 MAPK3 GUCY1A1 ACTG2 PRKG1 PLA2G1B MAPK1 ADORA2A RAMP3 RAMP1 ACTA2 RAMP2 ITPR1 ADORA2B ITPR2 ARAF CYP4A22 NPR2 AVPR1A AVPR1B ROCK2 NPR1 ADRA1D ADRA1B ADRA1A KCNMB4 PLA2G2C ARHGEF1 MYLK3 CALML6

KEGG_WNT_SIGNALING_PATHWAY http://www.gsea-msigdb.org/gsea/msigdb/cards/KEGG_WNT_SIGNALING_PATHWAY JUN LRP5 LRP6 PPP3R2 SFRP2 SFRP1 PPP3CC VANGL1 PPP3R1 FZD1 FZD4 APC2 FZD6 FZD7 SENP2 FZD8 LEF1 CREBBP FZD9 PRICKLE1 CTBP2 ROCK1 CTBP1 WNT9B WNT9A CTNNBIP1 DAAM2 TBL1XR1 MMP7 CER1 MAP3K7 VANGL2 WNT2B WNT11 WNT10B DKK2 SKP1P2 CHP2 AXIN1 AXIN2 DKK4 NFAT5 MYC SOX17 CSNK2A1 CSNK2A2 NFATC4 CSNK1A1 NFATC3 CSNK1E BTRC PRKX SKP1 FBXW11 RBX1 CSNK2B SIAH1 TBL1Y WNT5B CCND1 CAMK2A NLK

CAMK2B CAMK2D CAMK2G PRKACA APC PRKACB PRKACG WNT16 DAAM1 CHD8 FRAT1 CACYBP CCND2 NFATC2 NFATC1 CCND3 PLCB2 PLCB1 CSNK1A1L PRKCB PLCB3 PRKCA PLCB4 WIF1 PRICKLE2 PORCN RHOA FRAT2 PRKCG MAPK9 MAPK10 WNT3A DVL3 RAC2 DVL2 RAC3 FZD3 DKK1 CXXC4 DVL1 FOSL1 CUL1 WNT10A WNT4 SMAD3 TCF7 SMAD4 RAC1 TCF7L2 SMAD2 WNT1 MAPK8 EP300 WNT7A GSK3B WNT7B PSEN1 WNT8A WNT8B WNT2 WNT3 WNT5A WNT6 CTNNB1 PPP2CB PPP2CA PPP2R1A TBL1X PPP2R1B ROCK2 NKD1 FZD10 FZD5 NKD2 TCF7L1 RUVBL1 PPARD PPP3CB TP53 PPP3CA PPP2R5A PPP2R5E PPP2R5D PPP2R5C PPP2R5B FZD2 SFRP5 SFRP4 CHP1

KEGG_DORSO_VENTRAL_AXIS_FORMATION http://www.gsea-msigdb.org/gsea/msigdb/cards/KEGG_DORSO_VENTRAL_AXIS_FORMATION SOS2 NOTCH4 NOTCH3 NOTCH2 EGFR NOTCH1 KRAS FMN2 ETV7 PIWIL3 ETV6 PIWIL2 MAP2K1 PIWIL1 GRB2 SPIRE2 CPEB1 MAPK3 ETS1 ETS2 SPIRE1 SOS1 PIWIL4 MAPK1

KEGG_NOTCH_SIGNALING_PATHWAY http://www.gsea-msigdb.org/gsea/msigdb/cards/KEGG_NOTCH_SIGNALING_PATHWAY HES5 DTX3 NOTCH4 DTX3L NOTCH3 NOTCH2 EP300 HES1 NOTCH1 NUMB PSEN2 PSEN1 PTCRA SNW1 APH1A KAT2A ADAM17 RFNG RBPJ DTX1 CREBBP DTX2 MAML1 CTBP2 NCOR2 CTBP1 DVL3 JAG2 DVL2 NUMBL MAML2 KAT2B DLL4 PSENEN DLL3 DVL1 CIR1 DLL1 LFNG JAG1 MAML3 HDAC1 HDAC2 NCSTN DTX4 MFNG RBPJL

KEGG_HEDGEHOG_SIGNALING_PATHWAY http://www.gsea-msigdb.org/gsea/msigdb/cards/KEGG_HEDGEHOG_SIGNALING_PATHWAY CSNK1A1L HHIP PTCH2 GAS1 WNT3A ZIC2 WNT9B WNT9A LRP2 CSNK1G1 WNT2B WNT11 WNT10B IHH SMO WNT10A WNT4 CSNK1G3 SHH WNT1 CSNK1D RAB23 CSNK1A1 CSNK1G2 CSNK1E BMP8A GSK3B WNT7A BTRC WNT7B WNT8A WNT8B WNT2 WNT3 PRKX WNT5A WNT6 FBXW11 STK36 WNT5B GLI1 DHH PRKACA PRKACB SUFU BMP4 PRKACG BMP2 GLI2 BMP7 GLI3 PTCH1 BMP8B WNT16 BMP5 BMP6

KEGG_TGF_BETA_SIGNALING_PATHWAY http://www.gsea-msigdb.org/gsea/msigdb/cards/KEGG_TGF_BETA_SIGNALING_PATHWAY TFDP1 NOG TNF GDF7 INHBB INHBC COMP INHBA THBS4 RHOA CREBBP ROCK1 ID1 ID2 RPS6KB1 RPS6KB2 CUL1 SKP1P2 ID4 SMAD3

MAPK3 RBL2 SMAD4 RBL1 NODAL SMAD1 MYC SMAD2 MAPK1 SMURF2 SMURF1 EP300 BMP8A GDF5 SKP1 CHRD TGFB2 TGFB1 IFNG CDKN2B PPP2CB PPP2CA PPP2R1A ID3 SMAD5 RBX1 FST PITX2 PPP2R1B TGFBR2 AMHR2 LTBP1 LEFTY1 AMH TGFBR1 SMAD9 LEFTY2 SMAD7 ROCK2 TGFB3 SMAD6 BMPR2 GDF6 BMPR1A BMPR1B ACVRL1 ACVR2B ACVR2A ACVR1 BMP4 E2F5 BMP2 ACVR1C E2F4 SP1 BMP7 BMP8B ZFYVE9 BMP5 BMP6 ZFYVE16 THBS3 INHBE THBS2 DCN THBS1

KEGG_AXON_GUIDANCE http://www.gsea-msigdb.org/gsea/msigdb/cards/KEGG_AXON_GUIDANCE UNC5B PLXNB2 PPP3R2 PPP3CC PPP3R1 PAK4 NGEF SEMA4C SEMA4A PLXNC1 ROCK1 ABLIM3 PTK2 ROBO1 SLIT2 ROBO2 PLXNA1 ABL1 PLXNA2 PLXNB3 PLXNB1 SEMA3G ITGB1 SEMA3F NRAS ROBO3 CHP2 NFAT5 SEMA6D RHOD PAK6 PLXNA3 NCK1 NFATC4 NTNG1 SEMA6A NFATC3 KRAS SRGAP3 L1CAM CFL2 CFL1 GNAI1 UNC5A GNAI2 RND1 NCK2 MET SLIT1 SLIT3 PAK5 NTN1 PAK1 LRRC4C SEMA3C GNAI3 CXCL12 SEMA5A NFATC2 NFATC1 DCC CDC42 UNC5D SRGAP2 SEMA6C SEMA6B PAK3 SEMA3D EPHB4 EPHB6 SEMA4D SEMA4B PAK2 SEMA4F RHOA CXCR4 EPHB1 EPHA8 EPHB3 EPHB2 EFNA4 EFNA3 RAC2 EFNB1 EFNA5 EFNB3 RAC3 EFNB2 ARHGEF12 ABLIM2 SRGAP1 EFNA2 RASA1 EFNA1 DPYSL5 SEMA3A UNC5C NRP1 MAPK3 RAC1 MAPK1 SEMA5B HRAS SEMA3B GSK3B SEMA3E DPYSL2 LIMK2 LIMK1 ABLIM1 ROCK2 SEMA7A EPHA1 NTN4 EPHA5 EPHA7 EPHA3 EPHA2 EPHA4 SEMA4G PPP3CB FES PPP3CA CDK5 RGS3 FYN EPHA6 NTN3 CHP1

KEGG_VEGF_SIGNALING_PATHWAY http://www.gsea-msigdb.org/gsea/msigdb/cards/KEGG_VEGF_SIGNALING_PATHWAY CASP9 RAF1 PRKCB PRKCA PPP3R2 PPP3CC PPP3R1 SH2D2A PLA2G3 SPHK1 BAD PRKCG JMJD7-PLA2G4B PLA2G6 RAC2 PTK2 PLA2G2E PLA2G10 NOS3 MAPK11 MAP2K2 PLA2G2A RAC3 PLA2G4A PTGS2 PLA2G5 MAPK13 PLA2G12B MAP2K1 KDR PLA2G4B AKT1 PLA2G2F AKT2 NRAS AKT3 CHP2 NFAT5 MAPK3 RAC1 PLA2G1B MAPK1 MAPKAPK3 PIK3R5 HRAS NFATC4 MAPK14 MAPK12 NFATC3 MAPKAPK2 KRAS SPHK2 HSPB1 VEGFA PXN PLA2G4E SRC PIK3R3 PPP3CB PPP3CA PIK3CA PIK3CB PIK3CD PLA2G2C PLCG1 SHC2 PLA2G2D PLCG2 PLA2G12A NFATC2 NFATC1 PIK3CG PIK3R1 CDC42 PIK3R2 CHP1

KEGG_FOCAL_ADHESIONhttp://www.gsea-

msigdb.org/gsea/msigdb/cards/KEGG_FOCAL_ADHESION JUN ELK1 HGF PARVA FN1 TNN IGF1 BIRC3 XIAP COMP THBS4 IGF1R DIAPH1 ITGA11 PGF PARVG ROCK1 PTK2 MYL7 FLT1 FLT4 AKT1 RELN AKT2 LAMC2 MYL12A LAMB2 LAMB3 LAMC1 LAMA4 LAMA5 LAMB1 PAK6 PIK3R5 CAPN2 LAMB4 FLNC FLNA FLNB MYL2 MYLK MYL5 PIP5K1C MET MYL10 BIRC2 COL11A1 LAMC3 COL11A2 THBS3 THBS2 THBS1 VWF ZYX IBSP VTN PDGFD PPP1R12A BAD ACTN4 ACTN1 MAPK9 MAPK10 MAP2K1 RASGRF1 ILK RAPGEF1 GRB2 PPP1CC PPP1CB ACTG1 ITGA10 HRAS ITGA8 CTNNB1 MYL12B ACTB ROCK2 PTEN RAP1A PIK3R3 RAP1B TNC CAV2 CAV1 CAV3 COL5A3 TLN1 VAV3 COL6A2 COL6A3 COL5A2 COL6A1 LAMA1 ITGA9 CHAD PAK4 ITGA4 ITGA3 ITGA2B ITGA7 ITGA5 COL5A1 COL4A6 PDGFRB COL2A1 COL3A1 COL4A1 PARVB COL4A2 BRAF COL4A4 VAV1 PDPK1 ITGB3 ITGB4 VASP ITGB5 SHC4 ITGB6 DOCK1 ITGB7 ITGAV ITGB1 AKT3 VAV2 SPP1 COL1A1 COL1A2 TLN2 PDGFC VCL SHC3 VEGFA VEGFC ITGB8 VEGFB PXN PAK5 CCND1 PDGFA BCL2 PDGFB PDGFRA ARHGAP5 BCAR1 PAK1 VEGFD CRK CRKL CCND2 CDC42 ACTN2 CCND3 ACTN3 SOS2 PAK3 PRKCB RAF1 PRKCA SHC1 PAK2 MYL9 RHOA PRKCG MYLPF ERBB2 RAC2 RAC3 KDR MYLK2 PPP1CA MAPK3 ARHGAP35 RAC1 SOS1 MAPK1 MAPK8 EGFR GSK3B TNR EGF LAMA3 TNXB LAMA2 ITGA6 ITGA2 SRC ITGA1 PIK3CA PIK3CB PIK3CD SHC2 COL6A6 MYLK3 FYN PIK3CG PIK3R1 PIK3R2

KEGG_ECM_RECEPTOR_INTERACTION http://www.gsea-msigdb.org/gsea/msigdb/cards/KEGG_ECM_RECEPTOR_INTERACTION GP1BA COL6A2 COL6A3 GP1BB COL5A2 COL6A1 LAMA1 VWF HSPG2 TNN FN1 ITGA9 GP9 COMP IBSP CD36 CHAD GP5 VTN THBS4 ITGA4 ITGA3 ITGA2B ITGA7 ITGA5 COL5A1 COL4A6 ITGA11 SV2C COL2A1 COL3A1 COL4A1 AGRN COL4A2 COL4A4 ITGB3 ITGB4 RELN ITGB5 ITGB6 ITGB7 LAMC2 ITGAV ITGB1 LAMB2 SPP1 LAMB3 LAMC1 COL1A1 LAMA4 LAMA5 LAMB1 COL1A2 ITGA10 GP6 ITGA8 LAMB4 TNR CD47 SV2A CD44 DAG1 TNXB LAMA3 LAMA2 SDC3 ITGB8 ITGA6 ITGA2 ITGA1 SV2B TNC COL11A1 LAMC3 COL11A2 HMMR SDC2 SDC4 COL5A3 THBS3 COL6A6 THBS2 SDC1 THBS1

KEGG_CELL_ADHESION_MOLECULES_CAMS http://www.gsea-msigdb.org/gsea/msigdb/cards/KEGG_CELL_ADHESION_MOLECULES_CAMS CDH5

JAM3 CDH3 NLGN3 CDH4 CD80 NLGN1 CD86 CD28 CD274 PDCD1LG2 ITGA9 ITGAL NRCAM ITGAM CD34 CD276 ICOSLG CADM3 ITGA4 ICOS SIGLEC1 CADM1 HLA-G CLDN20 PECAM1 CD22 ITGB7 SELL VCAM1 ITGAV SELP SPN ITGB1 SELPLG ITGB2 CDH2 JAM2 CTLA4 HLA-DRB4 CLDN18 CD4 HLA-DRB5 CNTN1 NLGN2 HLA-DRB3 NRXN3 ALCAM SELE CD8A CD8B CD6 CLDN17 L1CAM ITGB8 MAG VCAN HLA-F NFASC HLA-E NRXN1 HLA-DPA1 HLA-DPB1 HLA-DQA1 HLA-DQA2 HLA-DQB1 NRXN2 CD2 CLDN16 CLDN23 MADCAM1 SDC2 SDC4 SDC1 OCLN PVR HLA-DRB1 NECTIN2 CDH1 HLA-DRA NECTIN1 HLA-DOA HLA-DOB CLDN10 ICAM2 ICAM3 CLDN8 CLDN2 CLDN6 CLDN5 CLDN1 ICAM1 NEO1 HLA-C HLA-B ESAM HLA-DMB HLA-DMA HLA-A F11R PDCD1 CLDN19 PTPRF CLDN15 CD226 CD99 CLDN22 CNTNAP2 MPZ MPZL1 PTPRC NECTIN3 ITGA8 NCAM2 NCAM1 CD58 NEGR1 CLDN11 SDC3 CLDN7 CLDN4 PTPRM CLDN3 ITGA6 CNTN2 CD40LG CNTNAP1 CD40 GLG1 CDH15 CLDN14 NLGN4X CLDN9

KEGG_ADHERENS_JUNCTION http://www.gsea-msigdb.org/gsea/msigdb/cards/KEGG_ADHERENS_JUNCTION AFDN SORBS1 WAS SNAI2 IGF1R RHOA CREBBP LEF1 ACTN4 PTPN6 ACTN1 SNAI1 ERBB2 WASF2 RAC2 PTPRJ WASL RAC3 PTPRF WASF1 MAP3K7 FGFR1 IQGAP1 SMAD3 MAPK3 TCF7 SMAD4 ACTG1 INSR WASF3 CTNNA1 PTPRB RAC1 TCF7L2 SMAD2 MAPK1 CSNK2A1 NECTIN3 CSNK2A2 EP300 EGFR VCL CTNNA2 CTNNB1 CTNND1 FARP2 CSNK2B TGFBR2 PTPRM NECTIN4 TGFBR1 ACTB TJP1 MET SSX2IP SRC NLK TCF7L1 YES1 FER BAIAP2 CTNNA3 LMO7 FYN ACP1 PTPN1 PARD3 CDC42 ACTN2 NECTIN2 CDH1 ACTN3 NECTIN1

KEGG_TIGHT_JUNCTIONhttp://www.gsea-msigdb.org/gsea/msigdb/cards/KEGG_TIGHT_JUNCTION PATJ JAM3 AFDN YBX3 PPP2R2D MAGI2 MPP5 TJP3 MYH13 MAP3K20 EPB41L3 MYH14 RRAS2 MYH1 CTTN MYH15 MYL7 CRB3 CLDN20 AKT1 AKT2 MYL12A AKT3 NRAS EXOC3 CTNNA1 JAM2 CSNK2A1 CLDN18 LLGL1 LLGL2 CSNK2A2 KRAS CLDN17 PARD6B MAGI1 GNAI1 GNAI2 MYL2 VAPA MYL5 CSNK2B SYMPK MYL10 IGSF5 ASH1L YES1 MAGI3 GNAI3 CLDN16 CLDN23 MYH3 MYH2 MYH4 MYH7 MYH6 MYH9 MYH8 MYH11 MYH10 OCLN CDC42 ACTN2 ACTN3 PARD6A PRKCB PRKCA CLDN10 CASK CLDN8 CLDN2 CGN CLDN6 MYL9 TJP2 CLDN5 CLDN1 RHOA MPDZ PRKCI ACTN4 PRKCG ACTN1 PRKCH

MYLPF PRKCD F11R PRKCE CLDN19 PRKCQ CLDN15 PRKCZ CLDN22 ACTG1 RAB13 HRAS CTNNA2 PARD6G TJAP1 SPTAN1 PPP2R2A CTNNB1 PPP2R2B RRAS CLDN11 PPP2CB PPP2CA PPP2R1A CLDN7 MYL12B PPP2R1B MRAS CLDN4 EXOC4 CLDN3 RAB3B ACTB TJP1 SRC PTEN MYH7B HCLS1 CDK4 AMOTL1 CLDN14 CLDN9 PPP2R2C CTNNA3 EPB41L2 EPB41L1 EPB41 PARD3

KEGG_GAP_JUNCTION http://www.gsea-msigdb.org/gsea/msigdb/cards/KEGG_GAP_JUNCTION PLCB2 GRM5 SOS2 ADCY8 PLCB1 ADCY9 ADCY6 TUBA1A RAF1 PRKCB ADCY7 PRKCA PLCB3 PLCB4 ADCY5 GRM1 CDK1 PDGFD TUBB4A ADRB1 TUBB4B TUBB3 PRKCG PDGFRB TUBB2A MAP2K2 ITPR3 MAP2K1 MAP2K5 LPAR1 ADCY4 TUBA1B NRAS GRB2 MAPK3 DRD1 GUCY1B1 DRD2 MAPK7 GUCY1A1 PRKG1 SOS1 TUBB7P PRKG2 MAPK1 TUBA3D HRAS GJD2 CSNK1D EGFR TUBB2B ITPR1 ITPR2 PDGFC TUBAL3 GNA11 KRAS EGF PRKX GJA1 GNAI1 HTR2A TUBA3C GNAI2 GUCY1A2 HTR2C HTR2B TUBB8 TUBA8 ADCY3 TUBA4A ADCY2 ADCY1 TUBA1C TJP1 TUBB1 SRC PDGFA PDGFB PDGFRA PRKACA PRKACB GNAQ PRKACG GNAS GNAI3 TUBB6 TUBB MAP3K2 TUBA3E

KEGG_COMPLEMENT_AND_COAGULATION_CASCADES http://www.gsea-msigdb.org/gsea/msigdb/cards/KEGG_COMPLEMENT_AND_COAGULATION_CASCADES F2 F2R VWF KNG1 FGB PLAT SERPIND1 MBL2 F3 F5 SERPINA1 PLAUR F7 PLAU F10 F9 TFPI F8 KLKB1 CR2 MASP1 C9 A2M CR1 F12 F13A1 F11 C8A F13B C7 C8G C8B CD59 SERPINA5 FGG CD55 C6 C5AR1 C5 BDKRB1 C4BPB C4BPA C4B C4A BDKRB2 CFH CFB CPB2 CD46 PROS1 SERPINF2 PROC FGA SERPINE1 PLG C2 SERPINC1 C1S C3AR1 C3 C1QB CFD C1QA C1R C1QC CFI SERPING1 MASP2 THBD

KEGG_ANTIGEN_PROCESSING_AND_PRESENTATION http://www.gsea-msigdb.org/gsea/msigdb/cards/KEGG_ANTIGEN_PROCESSING_AND_PRESENTATION HLA-DOA HLA-DOB KLRC3 KLRD1 KLRC1 KLRC2 RFXAP RFX5 IFNA5 IFNA4 IFNA2 IFNA1 LGMN PSME3 CTSS HLA-C HLA-B HLA-DMB HLA-DMA HLA-A HSPA1L HSPA1B HSPA2 KIR2DS5 HLA-G KIR3DL1 KIR3DL2 HSPA1A RFXANK CREB1 IFNA17 HSPA5 CD74 HSPA4 IFNA21 CTSL IFNA6 IFI30 IFNA7 IFNA8 IFNA10 IFNA13 CIITA IFNA14 IFNA16 KIR2DL5A HSPA6 HLA-DRB4 CD4 KIR2DL1 HLA-DRB5 LTA KIR3DL3 HLA-DRB3 TAP2 KIR2DS4 TAPBP CD8A CD8B TAP1

KIR2DS3 HSPA8 KIR2DL4 CANX KIR2DS1 KIR2DL2 KIR2DL3 KLRC4 NFYC HSP90AA1 NFYA NFYB HLA-F CTSB HLA-E CALR HLA-DPA1 HLA-DPB1 HLA-DQA1 PDIA3 HLA-DQA2 HLA-DQB1 PSME1 PSME2 HSP90AB1 B2M HLA-DRB1 HLA-DRA

KEGG_TOLL_LIKE_RECEPTOR_SIGNALING_PATHWAY http://www.gsea-msigdb.org/gsea/msigdb/cards/KEGG_TOLL_LIKE_RECEPTOR_SIGNALING_PATHWAY JUN CD80 CD86 STAT1 TLR9 CCL3 MYD88 CCL5 MAP2K4 CCL4 LBP RIPK1 TLR6 IKBKB LY96 MAP3K8 MAP3K7 AKT1 AKT2 CD14 CTSK AKT3 SPP1 IKBKE NFKB1 NFKBIA PIK3R5 TLR5 TIRAP MAPK14 MAPK12 RELA TRAF6 TRAF3 TOLLIP CXCL11 TAB2 TLR7 TLR8 CXCL10 CASP8 TICAM1 CHUK TNF IFNA5 IFNA4 IFNA2 FADD IFNA1 IRAK4 CXCL8 MAPK9 IL12B MAPK10 MAPK11 MAP2K2 MAP2K3 IL12A MAPK13 MAP2K1 MAP2K7 MAP2K6 IFNA17 IFNA21 IFNA6 IFNA7 IFNA8 IFNA10 IFNA13 IFNA14 IFNA16 MAPK3 RAC1 MAPK1 IRF3 IKBKG MAPK8 IFNB1 IFNAR1 IFNAR2 TICAM2 IL1B IRAK1 CD40 PIK3R3 TBK1 PIK3CA CXCL9 PIK3CB PIK3CD TLR3 TLR4 TAB1 TLR1 FOS TLR2 IRF5 PIK3CG IL6 PIK3R1 IRF7 PIK3R2

KEGG_NOD_LIKE_RECEPTOR_SIGNALING_PATHWAY http://www.gsea-msigdb.org/gsea/msigdb/cards/KEGG_NOD_LIKE_RECEPTOR_SIGNALING_PATHWAY TRIP6 CASP8 CHUK TAB3 CXCL1 RIPK2 TNF BIRC3 CCL2 XIAP CCL5 NOD1 CARD6 CCL13 CCL11 CCL8 CARD8 CCL7 MEFV CXCL8 MAPK9 MAPK10 MAPK11 ERBIN MAPK13 CASP5 IKBKB NOD2 MAP3K7 CASP1 CARD18 TNFAIP3 SUGT1 MAPK3 PSTPIP1 NFKB1 NFKBIB NFKBIA MAPK1 IKBKG CARD9 MAPK8 MAPK14 MAPK12 IL18 RELA PYDC1 TRAF6 HSP90AA1 PYCARD TAB2 NLRC4 IL1B HSP90B1 NLRP3 NLRP1 NAIP BIRC2 CXCL2 HSP90AB1 TAB1 IL6

KEGG_RIG_I_LIKE_RECEPTOR_SIGNALING_PATHWAY http://www.gsea-msigdb.org/gsea/msigdb/cards/KEGG_RIG_I_LIKE_RECEPTOR_SIGNALING_PATHWAY CASP10 CASP8 CXCL10 CHUK TRIM25 TNF IFNA5 IFNA4 IFNA2 ATG12 TBKBP1 FADD SIKE1 IFNA1 RIPK1 CXCL8 MAPK9 IL12B MAPK10 ISG15 MAPK11 MAP3K1 IL12A MAPK13 IKBKB MAP3K7 IFNA17 TKFC IFNA21 IFNA6 IFNA7 IFNA8 IFNA10 OTUD5 IFNA13 IFNA14 IFNA16 DDX3X IKBKE NFKB1 NFKBIB NFKBIA IRF3 IFIH1 IKBKG MAPK8 MAPK14 MAPK12 RELA DDX3Y IFNE TRAF6 TRAF3 TRAF2 IFNB1 TANK CYLD TRADD ATG5 DDX58 PIN1

MAVS IFNK DHX58 TBK1 STING1 IFNW1 AZI2 NLRX1 IRF7 RNF125

KEGG_CYTOSOLIC_DNA_SENSING_PATHWAY http://www.gsea-msigdb.org/gsea/msigdb/cards/KEGG_CYTOSOLIC_DNA_SENSING_PATHWAY

CXCL10 CHUK IFNA5 IFNA4 IFNA2 POLR1D CCL5 CCL4 IFNA1 RIPK1 POLR3K AIM2 IKBKB IFNA17 CASP1 IFNA21 IFNA6 POLR3H IFNA7 IFNA8 IFNA10 IFNA13 IFNA14 IFNA16 POLR3C IKBKE POLR3G POLR3B POLR3F NFKB1 NFKBIB NFKBIA IRF3 IKBKG TREX1 POLR1C IL18 RELA ZBP1 POLR3D RIPK3 IFNB1 IL33 PYCARD POLR3GL IL1B ADAR DDX58 MAVS CCL4L2 TBK1 STING1 POLR3A IL6 IRF7

KEGG_JAK_STAT_SIGNALING_PATHWAY http://www.gsea-msigdb.org/gsea/msigdb/cards/KEGG_JAK_STAT_SIGNALING_PATHWAY STAT3

STAT4 STAT1 STAT2 PIAS3 TYK2 IL21R CREBBP SOCS5 IL23A CTF1 STAM SPRY4 IL22 JAK1 AKT1 AKT2 JAK3 JAK2 AKT3 STAT5B STAT5A TPO STAT6 MYC IL24 SPRED1 PIK3R5 CSF2 IL13RA2 PIAS1 IL13RA1 CNTF PRLR CNTFR PRL SOCS1 IFNE CSF2RB CSF2RA IL23R CSF3 SOCS3 CCND1 IL22RA2 CSH1 BCL2L1 CSF3R PIAS4 CBLC CLCF1 OSMR IL20 CCND2 CCND3 SOS2 EPO PTPN11 IL9R MPL SPRED2 IFNA5 IFNA4 IFNA2 IFNLR1 SOCS2 IFNA1 CISH PTPN6 IL9 IL7R IL12RB1 IL12B IL13 IL12RB2 IL11RA IL12A IRF9 IFNA17 PIAS2 IFNA21 IFNA6 LEPR IFNA7 IFNA8 IFNA10 IFNA13 GRB2 IFNA14 LEP IFNA16 IL26 IL19 IL10 IL10RA SOS1 IL10RB EPOR IL11 IL3RA IL3 EP300 IL2RG IL2RB CBLB OSM CBL IL15RA IFNGR2 IL15 IFNG IFNGR1 IFNB1 IFNAR1 IFNAR2 IL21 IL20RB IL2RA IL22RA1 IL20RA IL2 IL6R PIK3R3 CRLF2 IFNK SPRY2 IL6ST SPRY1 IL7 STAM2 GHR IFNL3 IFNL1 SOCS4 PIK3CA PIK3CB PIM1 IFNL2 PIK3CD LIF GH1 GH2 IFNW1 LIFR TSLP IL4 IL4R SPRY3 IL5 PIK3CG SOCS7 IL5RA PIK3R1 IL6 PIK3R2

KEGG_HEMATOPOIETIC_CELL_LINEAGE http://www.gsea-msigdb.org/gsea/msigdb/cards/KEGG_HEMATOPOIETIC_CELL_LINEAGE GP1BA

GP1BB EPO IL9R CD33 TNF GP9 ITGAM CD34 CD36 GP5 ITGA4 ITGA3 KITLG ITGA2B FCGR1A ITGA5 FCER2 GYPA THPO IL1R2 IL7R MME IL11RA CD19 MS4A1 TFRC CD22 KIT FLT3 ITGB3 FLT3LG IL1A CR2 CD14 CR1 TPO EPOR IL11 CSF2 HLA-DRB4 CD4 CSF1R HLA-DRB5 CSF1 HLA-DRB3 IL3RA IL3 CD59 CD9

CD8A CD8B CD44 CD7 CD5 CSF2RA CD55 DNTT CSF3 IL1B IL1R1 IL2RA ITGA6 IL6R ITGA2 ITGA1 CSF3R IL7 CD37 CD2 CD3D CD3E CD38 CD3G CD1B ANPEP CD1C CD1D CD1E CD1A IL4 IL4R IL5 IL5RA IL6 HLA-DRB1 HLA-DRA

KEGG_NATURAL_KILLER_CELL_MEDIATED_CYTOTOXICITY http://www.gsea-msigdb.org/gsea/msigdb/cards/KEGG_NATURAL_KILLER_CELL_MEDIATED_CYTOTOXICITY ZAP70 TNFSF10 ITGAL PPP3R2 PPP3CC PPP3R1 FCER1G SH2D1B HLA-G BRAF VAV1 SHC4 PRF1 VAV2 NRAS CHP2 ITGB2 GZMB NFAT5 FCGR3B RAET1E ULBP3 FCGR3A CSF2 PIK3R5 NCR1 HCST NCR2 NFATC4 NFATC3 KRAS SHC3 CD247 RAET1L HLA-E PAK1 LCP2 KLRK1 PLCG1 PLCG2 PTK2B NFATC2 NFATC1 LCK SOS2 PTPN11 KLRC3 RAF1 PRKCB KLRD1 SHC1 KLRC1 PRKCA KLRC2 TNF ICAM2 IFNA5 IFNA4 IFNA2 ICAM1 IFNA1 RAET1G HLA-C HLA-B PTPN6 PRKCG HLA-A RAC2 SH3BP2 BID MAP2K2 KIR2DS5 RAC3 KIR3DL1 KIR3DL2 MAP2K1 IFNA17 CASP3 IFNA21 IFNA6 IFNA7 IFNA8 TYROBP IFNA10 IFNA13 IFNA14 GRB2 IFNA16 MAPK3 KIR2DL5A RAC1 SOS1 MAPK1 KIR2DL1 HRAS KIR2DS4 CD48 KIR2DS3 IFNGR2 LAT KIR2DL4 ULBP1 KIR2DS1 ULBP2 KIR2DL2 KIR2DL3 TNFRSF10A IFNG IFNGR1 TNFRSF10D SYK IFNB1 MICB ARAF TNFRSF10B IFNAR1 TNFRSF10C IFNAR2 MICA PIK3R3 NCR3 CD244 PPP3CB PPP3CA PIK3CA PIK3CB FAS PIK3CD SHC2 FASLG FYN PIK3CG VAV3 PIK3R1 SH2D1A PIK3R2 CHP1

KEGG_T_CELL_RECEPTOR_SIGNALING_PATHWAY http://www.gsea-msigdb.org/gsea/msigdb/cards/KEGG_T_CELL_RECEPTOR_SIGNALING_PATHWAY JUN ZAP70 MALT1 CD28 PDPK1 PPP3R2 PPP3CC PPP3R1 PAK4 ICOS VAV1 IKBKB MAP3K8 MAP3K7 AKT1 AKT2 NRAS AKT3 VAV2 CHP2 NFAT5 NFKB1 NFKBIB NFKBIA PAK6 NFKBIE NCK1 CTLA4 CSF2 PIK3R5 CD4 NFATC4 MAPK14 MAPK12 NFATC3 RELA KRAS CD8A CD8B CD247 NCK2 MAP3K14 PAK5 CD3D CD3E PAK1 CD3G CBLC LCP2 PLCG1 NFATC2 NFATC1 LCK CDC42 RASGRP1 SOS2 PAK3 CHUK RAF1 TNF CARD11 PAK2 RHOA PTPN6 PDCD1 MAPK9 MAPK11 MAP2K2 PRKCQ MAPK13 MAP2K1 MAP2K7 GRAP2 GRB2 MAPK3 IL10 PTPRC SOS1 MAPK1 HRAS IKBKG TEC GSK3B CBLB CBL ITK LAT IFNG IL2 CD40LG PIK3R3 CDK4 PPP3CB DLG1 PPP3CA PIK3CA PIK3CB PIK3CD BCL10 IL4 FYN IL5 FOS PIK3CG VAV3 PIK3R1 PIK3R2 CHP1

KEGG_B_CELL_RECEPTOR_SIGNALING_PATHWAY http://www.gsea-

msigdb.org/gsea/msigdb/cards/KEGG_B_CELL_RECEPTOR_SIGNALING_PATHWAY

JUN SOS2 CHUK RAF1 PRKCB MALT1 CARD11 PPP3R2 PPP3CC PPP3R1 PTPN6 INPP5D DAPP1 RAC2 MAP2K2 RAC3 CD19 MAP2K1 VAV1 CD22 CD72 IKBKB CD79A AKT1 CD81 CR2 AKT2 CD79B VAV2 AKT3 NRAS CHP2 GRB2 RASGRP3 NFAT5 MAPK3 NFKB1 NFKBIB RAC1 NFKBIA SOS1 FCGR2B NFKBIE MAPK1 PIK3R5 IKBKG HRAS IFITM1 NFATC4 RELA NFATC3 GSK3B KRAS SYK BTK PIK3R3 BLNK PIK3AP1 PPP3CB PPP3CA LILRB3 PIK3CA PIK3CB PIK3CD PLCG2 BCL10 NFATC2 FOS NFATC1 PIK3CG LYN VAV3 PIK3R1 PIK3R2 CHP1

KEGG_FC_EPSILON_RI_SIGNALING_PATHWAY http://www.gsea-msigdb.org/gsea/msigdb/cards/KEGG_FC_EPSILON_RI_SIGNALING_PATHWAY

SOS2 RAF1 PRKCB PRKCA PDPK1 TNF MAP2K4 PLA2G3 FCER1G MS4A2 FCER1A PRKCD INPP5D PRKCE JMJD7-PLA2G4B MAPK9 MAPK10 PLA2G6 RAC2 IL13 PLA2G2E PLA2G10 MAPK11 MAP2K2 PLA2G2A RAC3 MAP2K3 PLA2G4A MAPK13 PLA2G5 PLA2G12B MAP2K1 VAV1 MAP2K7 PLA2G4B MAP2K6 AKT1 PLA2G2F AKT2 VAV2 AKT3 NRAS GRB2 MAPK3 RAC1 SOS1 PLA2G1B MAPK1 CSF2 PIK3R5 HRAS MAPK8 IL3 MAPK14 MAPK12 GAB2 KRAS LAT SYK PLA2G4E BTK PIK3R3 PIK3CA PIK3CB LCP2 PIK3CD PLA2G2C PLCG1 PLA2G2D PLCG2 PLA2G12A IL4 FYN IL5 PIK3CG VAV3 LYN PIK3R1 PIK3R2

KEGG_FC_GAMMA_R_MEDIATED_PHAGOCYTOSIS http://www.gsea-msigdb.org/gsea/msigdb/cards/KEGG_FC_GAMMA_R_MEDIATED_PHAGOCYTOSIS

FCGR1A SPHK1 PIP4K2B WASF2 PLA2G6 VAV1 WASF1 PLA2G4B PIKFYVE VASP AKT1 AKT2 PIP5K1A AMPH DOCK2 PIP5K1B ARPC4 ARPC5 AKT3 VAV2 ARPC1B ARPC3 SCIN WASF3 FCGR2A FCGR3A FCGR2B PIK3R5 DNM2 CFL2 CFL1 PIP5K1C PLA2G4E ASAP2 DNM3 PAK1 CRK CRKL PLCG1 PLCG2 PLD1 ARPC5L PLD2 CDC42 PRKCB RAF1 PRKCA ARF6 WAS FCGR2C PRKCG PRKCD INPP5D HCK PRKCE RAC2 MYO10 WASL PLA2G4A DNM1L MAP2K1 DNM1 MARCKS RPS6KB1 RPS6KB2 ASAP3 MAPK3 PLA2G4F ARPC2 RAC1 PTPRC MAPK1 MARCKSL1 GSN GAB2 SPHK2 LAT PLPP3 LIMK2 LIMK1 SYK PLA2G4D PIK3R3 PIK3CA PLPP1 PIK3CB PLPP2 PIK3CD ARPC1A NCF1 PIK3CG ASAP1 LYN VAV3 PIK3R1 PIK3R2

KEGG_LEUKOCYTE_TRANSENDOTHELIAL_MIGRATION http://www.gsea-msigdb.org/gsea/msigdb/cards/KEGG_LEUKOCYTE_TRANSENDOTHELIAL_MIGRATION

CDH5 JAM3 AFDN TXK ITGAL ITGAM ITGA4 SIPA1 ROCK1 MMP2 PTK2 MYL7 NCF4 MMP9 NCF2 CLDN20 PECAM1 VAV1 VASP VCAM1 ITGB1 MYL12A THY1 VAV2 ITGB2 CTNNA1 JAM2 PIK3R5 CLDN18 MAPK14 MAPK12 VCL CLDN17 GNAI1 MYL2 GNAI2 CTNND1 MYL5 PXN RAPGEF3 MYL10 ARHGAP5 BCAR1 GNAI3 CLDN16 CLDN23 EZR CXCL12 PLCG1 PLCG2 PTK2B RHOH OCLN CDC42 ACTN2 ACTN3 PTPN11 PRKCB PRKCA CLDN10 CLDN8 CLDN2 CLDN6 MYL9 CLDN5 CLDN1 ICAM1 RHOA CXCR4 ESAM ACTN4 PRKCG ACTN1 MYLPF F11R NOX1 CLDN19 RAC2 MAPK11 MAPK13 CLDN15 CD99 CLDN22 ARHGAP35 ACTG1 RAC1 ITK CTNNA2 RASSF5 CTNNB1 CLDN11 CLDN7 MYL12B CLDN4 CLDN3 ACTB ROCK2 RAP1A PIK3R3 RAP1B CLDN14 PIK3CA CLDN9 MSN PIK3CB PIK3CD NCF1 CTNNA3 NOX3 RAPGEF4 CYBA PIK3CG VAV3 CYBB PIK3R1 PIK3R2

KEGG_INTESTINAL_IMMUNE_NETWORK_FOR_IGA_PRODUCTION http://www.gsea-msigdb.org/gsea/msigdb/cards/KEGG_INTESTINAL_IMMUNE_NETWORK_FOR_IGA_PRODUCTION HLA-DRB4 TNFRSF13C HLA-DRB5 HLA-DOA HLA-DOB HLA-DRB3 CD80 CD86 CD28 TNFSF13 TNFSF13B CCL27 IL15RA IL15 ICOSLG ITGA4 TGFB1 ICOS CCR9 MAP3K14 CXCR4 CCL25 TNFRSF17 HLA-DMB HLA-DMA CCL28 LTBR PIGR IL2 CD40LG HLA-DPA1 CD40 AICDA HLA-DPB1 HLA-DQA1 HLA-DQA2 HLA-DQB1 TNFRSF13B ITGB7 CXCL12 CCR10 MADCAM1 IL4 IL5 IL10 IL6 HLA-DRB1 HLA-DRA

KEGG_LONG_TERM_POTENTIATION http://www.gsea-msigdb.org/gsea/msigdb/cards/KEGG_LONG_TERM_POTENTIATION GRM5 PLCB2 ADCY8 CALM2 PLCB1 RAF1 PRKCB PLCB3 PRKCA CALM1 PLCB4 RPS6KA6 PPP3R2 GRM1 PPP3CC PPP3R1 CREBBP PPP1R12A PRKCG GRIN1 GRIN2A GRIN2B MAP2K2 GRIN2C BRAF GRIN2D ITPR3 CACNA1C MAP2K1 GRIA2 GRIA1 RPS6KA2 RPS6KA3 NRAS CHP2 PPP1R1A PPP1CC PPP1CA PPP1CB MAPK3 RPS6KA1 MAPK1 HRAS ITPR1 EP300 ITPR2 KRAS PRKX ARAF CALML5 ADCY1 RAP1A RAPGEF3 CAMK4 CAMK2A RAP1B CAMK2B CAMK2D CAMK2G PRKACA GNAQ PRKACB PRKACG PPP3CB CALML3 PPP3CA ATF4 CALM3 CHP1 CALML6

KEGG_NEUROTROPHIN_SIGNALING_PATHWAY http://www.gsea-msigdb.org/gsea/msigdb/cards/KEGG_NEUROTROPHIN_SIGNALING_PATHWAY JUN FRS2 CALM2 CALM1 PDPK1 RPS6KA6 TP73 MAP3K5 RPS6KA5 BRAF

MAP3K1 MAP3K3 IKBKB ABL1 AKT1 SHC4 AKT2 NRAS AKT3 SH2B1 NFKB1 NFKBIB NFKBIA NFKBIE PIK3R5 MAPK14 BEX3 MAPK12 RELA KRAS SHC3 TRAF6 NGF CALML5 BDNF NGFR SH2B3 CSK CAMK4 YWHAB BCL2 CAMK2A CAMK2B CAMK2D FOXO3 CAMK2G IRS2 SORT1 CALML3 CRK SH2B2 CRKL PLCG1 PLCG2 YWHAZ ARHGDIA CALM3 YWHAH ARHGDIB YWHAG YWHAE CDC42 SOS2 PTPN11 RAF1 SHC1 RIPK2 RHOA BAD IRAK4 PRKCD MAPK9 MAPK10 MAGED1 MAPK11 MAP2K2 MAPK13 MAP2K1 BAX MAP2K7 MAP2K5 RPS6KA2 RAPGEF1 RPS6KA3 GRB2 MAPK3 RPS6KA4 MAPK7 KIDINS220 RAC1 SOS1 MAPK1 RPS6KA1 YWHAQ HRAS MAPK8 PRDM4 MAPKAPK2 GSK3B PSEN1 NTF4 IRAK2 NTF3 IRAK1 RAP1A PIK3R3 RAP1B IRAK3 TP53 PIK3CA PIK3CB PIK3CD GAB1 FASLG SHC2 ATF4 IRS1 ZNF274 NTRK2 PIK3CG IRS4 NTRK1 PIK3R1 PIK3R2 CALML6 NTRK3

KEGG_LONG_TERM_DEPRESSION http://www.gsea-msigdb.org/gsea/msigdb/cards/KEGG_LONG_TERM_DEPRESSION GRM5 PLCB2 RYR1 PLCB1 RAF1 PRKCB PRKCA PLCB3 PLCB4 IGF1 CRHR1 GRM1 CRH IGF1R PLA2G3 GRID2 PRKCG JMJD7-PLA2G4B CACNA1A PLA2G6 PLA2G2E PLA2G10 MAP2K2 PLA2G2A BRAF PLA2G4A ITPR3 PLA2G5 PLA2G12B MAP2K1 NOS1 GRIA3 GRIA2 GRIA1 PLA2G4B PLA2G2F NRAS MAPK3 GUCY1B1 GUCY1A1 PRKG1 PRKG2 MAPK1 PLA2G1B HRAS ITPR1 ITPR2 GNA12 GNA13 GNA11 KRAS GNAI1 GNAI2 GUCY1A2 PPP2CB ARAF PPP2CA PPP2R1A PPP2R1B PLA2G4E GNAQ GNAS PPP1R17 GNAI3 GNAO1 GNAZ PLA2G2C PLA2G2D PLA2G12A LYN

KEGG_OLFACTORY_TRANSDUCTION http://www.gsea-msigdb.org/gsea/msigdb/cards/KEGG_OLFACTORY_TRANSDUCTION CALM2 CALM1 OR11H4 OR52W1 OR5AU1 GRK3 OR2M2 OR2M7 OR2T33 OR4F5 CLCA4 OR52K1 OR52I1 OR51D1 OR2J2 OR8I2 OR2D2 OR2D3 OR4K13 OR4K2 OR2AK2 OR4N5 OR2L3 OR4K17 OR2L8 ARRB2 OR11G2 OR11L1 OR5P2 OR5P3 OR6C1 OR6C75 OR10AG1 OR6C76 OR6C70 OR5J2 OR52A1 OR4M2 OR51S1 OR4N2 GUCA1B OR52R1 GUCA1A OR51F2 OR13H1 OR9K2 CAMK2A CAMK2B CAMK2D CAMK2G OR51B5 OR10J5 CALML3 OR2H1 CALM3 OR7G2 OR7G3 OR10A2 OR10V1 PDC OR7A10 OR13C5 OR9G4 OR10H5 OR4P4 OR2C1 OR1F1 OR1D2 OR10A4 OR5B3 OR7D2 OR56B4 OR13F1 OR13C4 OR13C3 OR2T11 OR13C8 OR2T4 OR52B6 OR3A1 OR2AT4 OR3A2 OR4F15 OR4F6 OR1C1 OR4C15 OR1A2 OR4N4

OR56B1 OR2A1 OR5I1 OR2A12 OR7D4 OR9A4 OR5C1 OR4K14 OR1L6
OR4L1 OR2M3 OR2T12 OR14C36 OR11H6 OR2T34 OR2T10 OR2T8 OR2T3
OR2W3 OR6V1 OR6Q1 OR9I1 OR13C2 OR1G1 OR3A3 OR1Q1
OR13A1 GUCY2D OR5H6 OR6F1 OR2M5 OR8D2 OR4C16 OR4C11
GUCA1C OR1E1 OR1E2 OR1D4 OR1D5 OR1A1 OR5F1 OR5AP2
OR10H4 OR8D1 OR4C46 OR4C6 CLCA2 OR4S2 OR6B2 OR5D18 OR5L1
OR5D14 OR8U1 OR6C6 OR4B1 OR4X2 OR8D4 OR4X1 GNAL
OR6C65 OR6C68 OR8B4 OR52E2 OR52J3 OR5D16 OR4K1 OR4D6 OR5A1
OR5D13 OR5A2 OR2A4 OR52K2 OR4D11 OR52M1 OR2A5 OR8K5 OR6B3
OR51A7 OR51T1 OR51A4 OR51A2 OR8A1 OR51L1 OR5AS1 OR1B1 OR1J1
OR10G7 OR10G4 OR5M1 OR5M10 OR6M1 OR6X1 OR8G5 OR12D3 OR5M9
OR5B17 OR1L8 OR10Q1 OR1N2 OR1N1 OR8J1 OR51V1 OR1S2 OR1S1
OR9Q1 OR8U3 OR9Q2 OR8B12 OR8K3 OR8K1 OR6T1 OR10S1 OR1K1
OR4D5 OR2A2 OR8H2 OR8H3 OR10G9 OR5T1 OR5T3 OR5T2
OR56A4 OR5B21 OR56A1 OR8H1 OR51F1 OR10G8 CALML6 OR52N1 OR5M8
OR5M3 OR5M11 OR5K2 OR51B4 OR13D1 OR52A5 OR51M1 OR4D9
OR51B6 OR4D10 OR5AN1 OR4F17 OR51G2 OR5AR1 OR2AE1 OR51E2
OR4F16 OR51Q1 OR51G1 OR2Z1 OR51I1 OR51I2 OR52D1 OR52H1 OR5V1
OR2B2 CLCA1 OR1I1 OR52E4 PRKX OR52N2 OR52E6 OR52N5 OR2J3
OR2B3 OR52N4 OR10J3 OR52E8 OR9G1 OR13J1 OR8J3 OR5B12
OR56A3 OR56A5 OR51B2 OR5AK2 OR52L1 OR2AG2 OR52B4 OR2W1
OR10T2 OR2T1 OR6N2 OR10X1 OR6K2 OR10Z1 OR5AC2 OR2T5
OR14I1 OR2T2 OR10A6 OR1L4 OR10A5 OR2AG1 OR6K6 OR6N1
OR10C1 OR14J1 OR4A16 OR2G3 OR1M1 OR7G1 OR2A25 OR4K5 OR4A5
OR5H2 OR2T27 OR4C45 OR2G2 OR2C3 OR4A47 OR13C9 OR11H1
OR4A15 OR7A5 OR7C2 OR10R2 OR7C1 OR51E1 OR52I2 OR2H2 OR4S1
OR2T6 OR4C13 OR4C12 OR6C74 OR6C3 OR10A7 OR14A16 OR10G2
OR10H3 OR2T29 OR10G3 OR10H2 OR10H1 OR4C3 OR1L1 OR11A1 ADCY3
CALML5 OR1L3 PDE1C CNGA3 CNGA4 OR8S1 OR52B2 OR4E2 OR5L2
OR5K1 OR2L13 OR4D1 PRKACA PRKACB PRKACG OR1J2 OR10J1 OR4F3
OR2A42 OR2V2 OR4F4 CNGB1 OR7A17 OR13G1 OR2Y1 OR6S1 OR9A2
OR2B6 OR2F1 OR4M1 OR12D2 OR2B11 OR8G2P OR8B8 OR8G1 OR4D2
OR4Q3 OR10A3 PRKG1 OR1J4 PRKG2 OR6A2 OR2M4 OR2S2
OR10P1 OR10K2 OR10K1 OR2A14 OR2K2 OR2F2 OR2L2 OR6B1 OR2A7
OR6C4 OR6C2 OR7E24 OR2G6 OR6K3 OR6Y1 OR10AD1 OR8U8
OR4K15

KEGG_REGULATION_OF_ACTIN_CYTOSKELETON http://www.gsea-msigdb.org/gsea/msigdb/cards/KEGG_REGULATION_OF_ACTIN_CYTOSKELETON

BRK1 FN1 ENAH DIAPH1 SSH2 RRAS2 ITGA11 ROCK1 PTK2 MYL7 ARHGEF7 ARPC4 CD14 FGD3 ARPC5 MYL12A NRAS ARPC1B ARPC3 TIAM1 SCIN PAK6 PFN1 PFN2 PIK3R5 IQGAP3 KRAS CFL2 CFL1 MYL2 MYLK BDKRB1 MYL5 PIP5K1C BDKRB2 CSK MYL10 APC PIP4K2C MYH9 ARPC5L MYH10 ARHGEF4 FGF9 FGF8 FGF7 FGF6 FGF5 WAS PDGFD FGF3 FGF4 FGF1 FGF2 FGD1 PPP1R12A FGF21 ACTN4 ACTN1 MAP2K2 MAP2K1 ARHGEF12 FGFR2 FGFR4 FGFR3 FGFR1 FGF14 FGF17 FGF16 FGF10 FGF11 IQGAP1 FGF12 PPP1CC FGF13 PPP1CB ACTG1 ITGA10 HRAS ITGA8 PFN3 FGF23 LIMK2 LIMK1 FGF18 MYL12B PIP4K2A ACTB ROCK2 MOS PIK3R3 TMSB4Y GIT1 MSN ARPC1A BAIAP2 IQGAP2 NCKAP1 VAV3 ABI2 F2 F2R ARHGEF6 ITGA9 ITGAL ITGAM ITGAD ITGAE PAK4 ITGA4 ITGA3 ITGA2B APC2 ITGA7 ITGA5 MYH14 PDGFRB PIP4K2B WASF2 BRAF SLC9A1 DIAPH3 VAV1 WASF1 ITGB3 PIKFYVE ITGB4 ITGB5 DOCK1 ITGB6 PIP5K1A ITGB7 PIP5K1B ITGAV ITGB1 ITGAX VAV2 ITGB2 FGF20 PFN4 GNA12 PDGFC VCL GNA13 CHRM1 CHRM2 CYFIP1 CHRM4 CHRM3 ITGB8 RDX PXN CYFIP2 PAK5 PDGFA FGF22 PDGFB PDGFRA TMSB4X BCAR1 PAK1 CRK EZR CRKL CDC42 ACTN2 ACTN3 SOS2 PAK3 RAF1 PAK2 MYL9 RHOA TMSB4XP8 INS MYLPF RAC2 WASL CHRM5 RAC3 MYLK2 SSH3 PPP1CA MAPK3 ARHGAP35 INSRR ARPC2 FGF19 RAC1 SOS1 MAPK1 GSN EGFR EGF SSH1 NCKAP1L RRAS ARAF GNG12 MRAS ITGA6 DIAPH2 ITGA2 ITGA1 TIAM2 PIK3CA PIK3CB PIK3CD ARHGEF1 MYLK3 PIK3CG PIK3R1 PIK3R2

KEGG_INSULIN_SIGNALING_PATHWAY http://www.gsea-msigdb.org/gsea/msigdb/cards/KEGG_INSULIN_SIGNALING_PATHWAY GCK CALM2 PPARGC1A ELK1 CALM1 EIF4E EIF4EBP1 PRKAG2 FBP1 HK2 HK1 RHEB BRAF PDPK1 IKBKB EXOC7 AKT1 SHC4 AKT2 NRAS AKT3 PYGB PYGM ACACB INPP5K PYGL ACACA PIK3R5 PDE3B PHKB PHKA2 PHKA1 FLOT2 KRAS SHC3 SOCS1 PRKX PRKAB2 CALML5 PRKAB1 PRKAA2 PRKAA1 PDE3A SOCS3 FOXO1 PRKACA PRKACB G6PC2 PRKACG IRS2 CBLC CALML3 FLOT1 CRK SH2B2 CRKL RHOQ PRKAG1 PRKAR1A PRKAR2A PRKAR1B CALM3 PTPN1 SOS2 RAF1 SHC1 PRKAR2B TSC2 SORBS1 SLC2A4 MTOR MKNK1 SOCS2 FASN PHKG1 BAD INS PHKG2 PRKCI GYS1 GYS2 INPP5D MAPK9 HK3 MAPK10 MAP2K2 PTPRF MAP2K1 PRKAG3 RPTOR PPP1R3C RAPGEF1 PPP1R3A RPS6KB1 RPS6KB2 PRKCZ TSC1 GRB2

PPP1CC PPP1CA PPP1CB MAPK3 PKLR INSR SOS1 RPS6
PPP1R3D MAPK1 HRAS MAPK8 PCK2 PCK1 GSK3B CBLB CBL
EIF4E1B FBP2 LIPE MKNK2 EIF4E2 ARAF TRIP10 PPP1R3B
PIK3R3 SOCS4 PIK3CA PIK3CB PIK3CD SHC2 G6PC SREBF1 IRS1
PIK3CG IRS4 PIK3R1 PIK3R2 CALML6

KEGG_GNRH_SIGNALING_PATHWAY http://www.gsea-msigdb.org/gsea/msigdb/cards/KEGG_GNRH_SIGNALING_PATHWAY JUN CALM2
ELK1 CALM1 MAP2K4 JMJD7-PLA2G4B MMP2 PLA2G2E PLA2G6
PLA2G10 MAP3K1 MAP3K3 ITPR3 MAP3K4 PLA2G4B CGA PLA2G2F NRAS
MAPK14 MAPK12 KRAS GNA11 PRKX CALML5 ADCY3 ADCY2 ADCY1
PLA2G4E CAMK2A CAMK2B CAMK2D CAMK2G PRKACA GNAQ PRKACB
PRKACG GNRHR GNAS GNRH2 GNRH1 CALML3 PLA2G2D PLD1
PLA2G12A PLD2 HBEGF CALM3 PTK2B MAP3K2 CDC42 PLCB2
ADCY8 SOS2 ADCY9 PLCB1 ADCY6 ADCY7 PRKCB RAF1 PLCB3
PRKCA ADCY5 PLCB4 PLA2G3 PRKCD MAPK9 MAPK10 MAPK11
PLA2G2A MAP2K2 CACNA1D PLA2G4A MAP2K3 PLA2G5 MAPK13 PLA2G12B
MAP2K1 CACNA1C MAP2K7 CACNA1F MAP2K6 CACNA1S ADCY4 GRB2 MAPK3
MAPK7 SOS1 PLA2G1B MAPK1 HRAS MAPK8 ITPR1 EGFR ITPR2
MMP14 SRC FSHB LHB PLA2G2C ATF4 CALML6

KEGG_PROGESTERONE_MEDIATED_OOCYTE_MATURATION http://www.gsea-msigdb.org/gsea/msigdb/cards/KEGG_PROGESTERONE_MEDIATED_OOCYTE_MATURATION CDC16 ADCY8 ADCY9 ADCY6 RAF1 ADCY7 ADCY5 SPDYA IGF1
RPS6KA6 CDK1 ANAPC10 IGF1R ANAPC1 INS ANAPC11 SPDYC MAPK9
MAPK10 MAPK11 BRAF MAPK13 MAP2K1 ADCY4 AKT1 AKT2
RPS6KA2 RPS6KA3 AKT3 CPEB1 MAPK3 ANAPC7 ANAPC5 ANAPC2
ANAPC4 MAD2L1 RPS6KA1 MAPK1 PIK3R5 PDE3B MAPK8 CDC23
MAPK14 MAPK12 KRAS PRKX GNAI1 CCNA1 GNAI2 FZR1 ADCY3
HSP90AA1 ARAF ADCY2 PLK1 ADCY1 PDE3A ANAPC13 MOS
PIK3R3 PGR CCNA2 BUB1 PKMYT1 PRKACA CDK2 PRKACB PRKACG
CDC26 GNAI3 HSP90AB1 CDC27 PIK3CA PIK3CB CDC25B CDC25C
CCNB2 PIK3CD CDC25A MAD2L2 CCNB1 PIK3CG PIK3R1 CCNB3
PIK3R2

KEGG_MELANOGENESIS http://www.gsea-msigdb.org/gsea/msigdb/cards/KEGG_MELANOGENESIS CALM2 CALM1 TYR
CREB3L1 FZD1 FZD4 FZD6 FZD7 LEF1 CREBBP CREB3 FZD8
FZD9 POMC WNT9B WNT9A CREB1 WNT2B WNT11 EDN1
WNT10B NRAS EDNRB KRAS PRKX GNAI1 GNAI2 CALML5 ADCY3

ADCY2 ADCY1 CREB3L3 WNT5B CAMK2A CAMK2B CAMK2D CAMK2G PRKACA GNAQ PRKACB PRKACG GNAS GNAI3 WNT16 GNAO1 CALML3 DCT CALM3 PLCB2 ADCY8 ADCY9 PLCB1 ADCY6 ADCY7 PRKCB RAF1 PLCB3 PRKCA PLCB4 ADCY5 CREB3L4 ASIP KITLG PRKCG MC1R WNT3A DVL3 DVL2 MAP2K2 MAP2K1 KIT FZD3 DVL1 ADCY4 TYRP1 WNT4 WNT10A MAPK3 TCF7 MAPK1 TCF7L2 HRAS WNT1 EP300 GSK3B WNT7A WNT7B WNT8A WNT8B WNT2 WNT3 WNT5A CTNNB1 WNT6 FZD10 CREB3L2 FZD5 TCF7L1 MITF FZD2 CALML6

KEGG_ADIPOCYTOKINE_SIGNALING_PATHWAY http://www.gsea-msigdb.org/gsea/msigdb/cards/KEGG_ADIPOCYTOKINE_SIGNALING_PATHWAY

STAT3 CHUK PTPN11 PPARGC1A NPY TNF SLC2A4 CD36 SLC2A1 MTOR ADIPOR2 PRKAG2 ACSL6 POMC MAPK9 MAPK10 PRKCQ RXRB RXRG IKBKB PRKAG3 RXRA TNFRSF1B ADIPOR1 AKT1 AKT2 TNFRSF1A JAK2 LEPR AKT3 CPT1B LEP ACACB NFKB1 NFKBIB NFKBIA NFKBIE IKBKG MAPK8 CPT1A PCK2 CPT1C PCK1 RELA STK11 PPARA TRAF2 PRKAB2 PRKAB1 PRKAA2 PRKAA1 SOCS3 TRADD ACSL5 ADIPOQ G6PC2 IRS2 ACSL1 AGRP PRKAG1 G6PC IRS1 CAMKK1 CAMKK2 IRS4 ACSL3 ACSL4

KEGG_TYPE_II_DIABETES_MELLITUS http://www.gsea-msigdb.org/gsea/msigdb/cards/KEGG_TYPE_II_DIABETES_MELLITUS PIK3R5

GCK MAPK8 TNF MAFA SOCS1 SLC2A4 SLC2A2 MTOR SOCS2 SOCS3 INS PRKCD PRKCE MAPK9 HK2 CACNA1A MAPK10 HK3 HK1 PIK3R3 CACNA1D CACNA1E CACNA1B PDX1 CACNA1C ADIPOQ IKBKB IRS2 ABCC8 SOCS4 PIK3CA CACNA1G PIK3CB PIK3CD PRKCZ KCNJ11 MAPK3 IRS1 PKLR INSR PKM PIK3CG IRS4 PIK3R1 PIK3R2 MAPK1

KEGG_TYPE_I_DIABETES_MELLITUS http://www.gsea-msigdb.org/gsea/msigdb/cards/KEGG_TYPE_I_DIABETES_MELLITUS HLA-DRB4

HLA-DRB5 LTA HLA-DOA HLA-DOB HLA-DRB3 CD80 CD86 CD28 TNF ICA1 IFNG PTPRN2 HLA-C HLA-B INS IL1B HLA-DMB PTPRN HLA-DMA HLA-F HLA-E HLA-A IL2 HLA-DPA1 IL12B HLA-DPB1 GAD1 HLA-DQA1 HLA-G HLA-DQA2 IL12A CPE HLA-DQB1 GAD2 IL1A HSPD1 PRF1 FAS FASLG GZMB HLA-DRB1 HLA-DRA

KEGG_MATURITY_ONSET_DIABETES_OF_THE_YOUNG http://www.gsea-msigdb.org/gsea/msigdb/cards/KEGG_MATURITY_ONSET_DIABETES_OF_THE_YOUNG

MNX1 NEUROG3 GCK HHEX HES1 MAFA PAX6 SLC2A2 BHLHA15

HNF1B HNF4G NKX6-1 HNF1A FOXA3 HNF4A NKX2-2 ONECUT1 NEUROD1 INS FOXA2 PAX4 PDX1 IAPP NR5A2 PKLR

KEGG_ALDOSTERONE_REGULATED_SODIUM_REABSORPTION http://www.gsea-msigdb.org/gsea/msigdb/cards/KEGG_ALDOSTERONE_REGULATED_SODIUM_REABSORPTION PIK3R5 SFN NR3C2 PRKCB PRKCA FXYD4 KRAS FXYD2 IGF1 SCNN1G SCNN1B SCNN1A ATP1B2 KCNJ1 ATP1B3 ATP1A4 ATP1B1 NEDD4L INS PRKCG PIK3R3 HSD11B2 HSD11B1 SLC9A3R2 PDPK1 ATP1A1 IRS2 ATP1A3 ATP1A2 PIK3CA PIK3CB PIK3CD MAPK3 IRS1 SGK1 ATP1B4 INSR PIK3CG IRS4 PIK3R1 PIK3R2 MAPK1

KEGG_VASOPRESSIN_REGULATED_WATER_REABSORPTION http://www.gsea-msigdb.org/gsea/msigdb/cards/KEGG_VASOPRESSIN_REGULATED_WATER_REABSORPTION ADCY9 ADCY6 DYNLL2 DYNC1LI2 VAMP2 DCTN6 CREB3L4 AQP4 PRKX RAB11A DYNLL1 DCTN2 AQP3 CREB3L1 ADCY3 CREB3 RAB5B AVPR2 RAB5A CREB3L3 NSF DCTN5 AVP CREB3L2 RAB11B STX4 PRKACA PRKACB PRKACG CREB1 GNAS DYNC2H1 DYNC1H1 DCTN4 DYNC1I2 DCTN1 DYNC1I1 CREB5 AQP2 DYNC2LI1 ARHGDIA ARHGDIB DYNC1LI1 RAB5C

KEGG_ALZHEIMERS_DISEASE http://www.gsea-msigdb.org/gsea/msigdb/cards/KEGG_ALZHEIMERS_DISEASE UQCR10 NAE1 CALM2 NDUFA5 NDUFA4 COX6CP3 CALM1 ATP2A1 PPP3R2 EIF2AK3 PPP3CC ATP2A3 PPP3R1 ATP2A2 NDUFS7 MT-CYB ADAM17 BACE2 ATP5PO MT-CO2 MT-CO1 COX8C MME MT-CO3 COX5B ITPR3 PSENEN LRP1 COX4I1 APOE MT-ATP6 MT-ATP8 CHP2 ATP5PD NDUFA2 NDUFA3 NDUFA1 CAPN2 LPL CAPN1 APAF1 ATP5MC1P5 HSD17B10 APH1A UQCRQ ATP5F1D CALML5 GAPDH ATF6 COX7A2L IDE ATP5MC3 ATP5MC2 ADAM10 ATP5MC1 ATP5PB ATP5F1E COX5A CDK5R1 ERN1 GNAQ UQCRHL ATP5F1A BACE1 CALML3 APBB1 ATP5PF CYCS SDHA CALM3 NDUFA4L2 NCSTN CASP9 PLCB2 COX8A CASP8 COX7C CASP7 PLCB1 PLCB3 PLCB4 TNF RYR3 FADD SDHD SDHB SDHC COX7B BAD COX7A1 COX7A2 COX6C ATP5F1B GRIN1 GRIN2A GRIN2B COX6B1 CACNA1D BID ATP5F1C GRIN2C GRIN2D UQCR10P1 CACNA1C NOS1 CACNA1F CACNA1S COX7B2 CASP3 TNFRSF1A UQCRB COX6A2 MAPK3 COX6A1 UQCRH UQCRFS1 UQCRC2 UQCRC1 MAPK1 NDUFB3 NDUFB1 NDUFB2 ITPR1 NDUFA10 ITPR2 NDUFAB1 PSEN2 GSK3B PSEN1 NDUFA9 NDUFA7 NDUFA8 UQCR11 NDUFA6 NDUFV3 COX6B2 COX4I2 IL1B SNCA MAPT NDUFB10 NDUFS8 NDUFC1 NDUFC2 NDUFS1 NDUFV2 NDUFB6 NDUFS4 NDUFB7 NDUFV1 NDUFB8 NDUFS6 NDUFB9 NDUFS5

NDUFS2 PPP3CB NDUFB4 NDUFS3 PPP3CA APP NDUFB5 CDK5 FAS CYC1 CHP1 CALML6

KEGG_PARKINSONS_DISEASE http://www.gsea-msigdb.org/gsea/msigdb/cards/KEGG_PARKINSONS_DISEASE UQCR10 NDUFA5 NDUFA4 COX6CP3 UBE2L6 NDUFS7 MT-CYB MT-CO2 ATP5PO MT-CO1 COX8C MT-CO3 VDAC2P5 COX5B COX4I1 MT-ATP6 UCHL1 MT-ATP8 ATP5PD NDUFA2 NDUFA3 NDUFA1 SNCAIP APAF1 SEPTIN5 UBE2G2 UBE2G1 ATP5MC1P5 VDAC1 VDAC2 VDAC3 UBE2J2 UQCRQ ATP5F1D UBE2L3 COX7A2L ATP5MC3 ATP5MC2 ATP5MC1 ATP5PB ATP5F1E COX5A UBA7 UBA1 SLC25A6 UQCRHL SLC25A5 ATP5F1A SLC25A4 ATP5PF CYCS SDHA SLC18A1 SLC18A2 NDUFA4L2 TH SLC25A31 CASP9 COX8A COX7C PPID SDHD SDHB SDHC PINK1 COX7B COX7A1 COX7A2 COX6C UBE2J1 ATP5F1B COX6B1 ATP5F1C UQCR10P1 UBB COX7B2 PRKN CASP3 UQCRB COX6A2 COX6A1 UQCRH UQCRFS1 UQCRC2 UQCRC1 NDUFB3 NDUFB1 NDUFB2 NDUFA10 NDUFAB1 MT-ND6 NDUFA9 MT-ND5 NDUFA7 NDUFA8 UQCR11 HTRA2 NDUFA6 NDUFV3 MT-ND4 MT-ND4L MT-ND2 COX6B2 MT-ND3 COX4I2 MT-ND1 SNCA NDUFB10 NDUFS8 NDUFC1 NDUFC2 NDUFS1 NDUFV2 NDUFB6 NDUFS4 NDUFB7 NDUFV1 NDUFB8 NDUFS6 NDUFB9 NDUFS5 NDUFS2 NDUFB4 NDUFS3 NDUFB5 PARK7 CYC1 GPR37 SLC6A3 LRRK2

KEGG_AMYOTROPHIC_LATERAL_SCLEROSIS_ALS http://www.gsea-msigdb.org/gsea/msigdb/cards/KEGG_AMYOTROPHIC_LATERAL_SCLEROSIS_ALS CASP9 TNF CAT PPP3R2 PPP3CC PPP3R1 ALS2 BAD CCS MAP3K5 TOMM40L GRIN1 GRIN2A MAPK11 GRIN2B SOD1 GRIN2C BID GRIN2D MAP2K3 MAPK13 NOS1 BAX NEFM GRIA2 SLC1A2 NEFH GRIA1 MAP2K6 TNFRSF1B CASP1 CASP3 NEFL TNFRSF1A DAXX CHP2 RAC1 MAPK14 MAPK12 APAF1 GPX1 RAB5A BCL2 BCL2L1 PRPH2 PPP3CB TP53 PPP3CA PRPH DERL1 CYCS TOMM40 CHP1

KEGG_HUNTINGTONS_DISEASE http://www.gsea-msigdb.org/gsea/msigdb/cards/KEGG_HUNTINGTONS_DISEASE POLR2G POLR2H POLR2E UQCR10 POLR2F NDUFA5 PPARGC1A NDUFA4 POLR2I COX6CP3 POLR2J TAF4 NRF1 DCTN2 CREB3L1 NDUFS7 MT-CYB CREBBP CREB3 POLR2K ATP5PO MT-CO2 MT-CO1 UCP1 CLTCL1 COX8C DNALI1 POLR2L MT-CO3 VDAC2P5 COX5B COX4I1 CREB1 HIP1 AP2A2 MT-ATP6 AP2B1 DCTN4 MT-ATP8 TAF4B ATP5PD AP2A1 NDUFA2 NDUFA3 NDUFA1 DNAI1 APAF1 ATP5MC1P5 VDAC1 VDAC2 VDAC3 UQCRQ ATP5F1D DNAH1 BBC3 BDNF COX7A2L ATP5MC3 ATP5MC2 CREB3L3

ATP5MC1 ATP5PB ATP5F1E COX5A GNAQ DNAH2 SLC25A6 SP1 UQCRHL ATP5F1A SLC25A5 SLC25A4 ATP5PF RCOR1 CYCS TGM2 SDHA DCTN1 POLR2B DNAL1 POLR2A NDUFA4L2 POLR2D POLR2C DNAL4 SLC25A31 CASP9 PLCB2 COX8A GRM5 CASP8 COX7C PLCB1 PLCB3 PLCB4 DNAI2 IFT57 CREB3L4 PPID SDHD SDHB SDHC TFAM HAP1 COX7B COX7A1 COX7A2 COX6C POLR2J2 ATP5F1B GRIN1 SOD1 GRIN2B COX6B1 SOD2 ATP5F1C UQCR10P1 BAX COX7B2 CASP3 REST UQCRB POLR2J3 COX6A2 CREB5 COX6A1 UQCRH UQCRFS1 UQCRC2 UQCRC1 SIN3A NDUFB3 NDUFB1 DLG4 TBPL2 NDUFB2 EP300 ITPR1 NDUFA10 NDUFAB1 NDUFA9 NDUFA7 NDUFA8 DNAH3 UQCR11 NDUFA6 NDUFV3 COX6B2 COX4I2 GPX1 TBP AP2S1 AP2M1 CREB3L2 NDUFS8 NDUFB10 NDUFC1 NDUFC2 NDUFV2 NDUFS1 NDUFS4 NDUFB6 NDUFV1 NDUFB7 NDUFS6 NDUFB8 NDUFS5 NDUFB9 PPARG NDUFS2 TP53 NDUFB4 NDUFS3 NDUFB5 CYC1 CLTA CLTB CLTC HDAC1 HDAC2 TBPL1 HTT

KEGG_PRION_DISEASEShttp://www.gsea-msigdb.org/gsea/msigdb/cards/KEGG_PRION_DISEASES NCAM2 EGR1 NCAM1 ELK1 NOTCH1 PRKX C6 CCL5 C5 IL1B SOD1 STIP1 MAP2K2 HSPA1A MAP2K1 BAX PRKACA PRKACB PRKACG IL1A HSPA5 PRNP C9 C1QB C1QA LAMC1 C1QC MAPK3 FYN C8A C7 IL6 C8G C8B MAPK1

KEGG_VIBRIO_CHOLERAE_INFECTION http://www.gsea-msigdb.org/gsea/msigdb/cards/KEGG_VIBRIO_CHOLERAE_INFECTION ATP6V1G1 PDIA4 ADCY9 PRKCB SLC12A2 SEC61B PRKCA TJP2 KDELR3 ATP6V0A1 ATP6V1G2 KDELR2 ATP6V0B ATP6V1G3 ATP6AP1 PRKCG CFTR ATP6V1F ERO1A ATP6V0A4 ATP6V0A2 ATP6V0D1 KCNQ1 ARF1 ACTG1 ATP6V0E2 SEC61G ATP6V1H PRKX ATP6V1E2 MUC2 KDELR1 ADCY3 ATP6V0D2 ATP6V0E1 ATP6V1C2 ACTB TJP1 TCIRG1 PRKACA PRKACB PRKACG GNAS ATP6V1D SEC61A2 ATP6V1A PLCG1 PLCG2 ATP6V1E1 ATP6V1B2 ATP6V1B1 SEC61A1 ATP6V1C1 ATP6V0C

KEGG_EPITHELIAL_CELL_SIGNALING_IN_HELICOBACTER_PYLORI_INFECTION http://www.gsea-msigdb.org/gsea/msigdb/cards/KEGG_EPITHELIAL_CELL_SIGNALING_IN_HELICOBACTER_PYLORI_INFECTION JUN JAM3 ATP6V1G1 CHUK PTPN11 CXCL1 MAP2K4 CCL5 NOD1 ADAM17 ATP6V0A1 ATP6V1G2 ATP6V0B ATP6V1G3 CXCR2 ATP6AP1 CXCR1 CXCL8 F11R MAPK9 MAPK10

MAPK11 MAPK13 IKBKB ATP6V1F ATP6V0A4 CASP3 ATP6V0A2 ATP6V0D1 NFKB1 RAC1 NFKBIA JAM2 IKBKG MAPK8 MAPK14 MAPK12 EGFR RELA ATP6V0E2 ATP6V1H ATP6V1E2 PTPRZ1 ATP6V0D2 MAP3K14 ATP6V0E1 ATP6V1C2 ADAM10 TJP1 MET CSK SRC TCIRG1 IGSF5 PAK1 GIT1 ATP6V1D ATP6V1A PLCG1 PLCG2 HBEGF ATP6V1E1 ATP6V1B2 LYN ATP6V1B1 CDC42 ATP6V1C1 ATP6V0C

KEGG_LEISHMANIA_INFECTION http://www.gsea-msigdb.org/gsea/msigdb/cards/KEGG_LEISHMANIA_INFECTION JUN HLA-DOA HLA-DOB PRKCB STAT1 ELK1 TNF ITGAM ITGA4 MYD88 FCGR2C FCGR1A HLA-DMB HLA-DMA PTPN6 IRAK4 IL12B MAPK11 NCF4 NCF2 PTGS2 MAPK13 IL12A NOS2 MAP3K7 IL1A JAK1 JAK2 ITGB1 ITGB2 CR1 MAPK3 FCGR3B NFKB1 FCGR2A IL10 NFKBIB NFKBIA FCGR3A MARCKSL1 MAPK1 HLA-DRB4 HLA-DRB5 HLA-DRB3 MAPK14 MAPK12 RELA IFNGR2 TRAF6 TGFB2 TGFB1 IFNG IFNGR1 TAB2 IL1B IRAK1 TGFB3 HLA-DPA1 HLA-DPB1 HLA-DQA1 HLA-DQA2 HLA-DQB1 C3 NCF1 TLR4 TAB1 IL4 TLR2 FOS CYBA HLA-DRB1 HLA-DRA

KEGG_PATHWAYS_IN_CANCER http://www.gsea-msigdb.org/gsea/msigdb/cards/KEGG_PATHWAYS_IN_CANCER JUN STAT3 TFG STAT1 HGF BIRC5 FN1 BIRC3 IGF1 XIAP PML JUP HHIP IGF1R FZD1 FZD4 GSTP1 FZD6 FZD7 FZD8 LEF1 FZD9 MSH6 PGF CTBP2 CTBP1 WNT9B PTK2 WNT9A PTGS2 HIF1A FLT3 FLT3LG ABL1 WNT2B JAK1 WNT11 AKT1 WNT10B AKT2 LAMC2 SMO NRAS LAMB2 ARNT TPR LAMB3 AXIN1 LAMC1 AXIN2 STAT5B STAT5A LAMA4 LAMA5 NFKB2 LAMB1 NFKB1 CTNNA1 NFKBIA TPM3 MYC PIK3R5 CSF1R SHH LAMB4 CCDC6 NCOA4 KRAS CSF2RA TRAF6 TGFB2 TRAF5 TRAF3 TGFB1 TRAF2 RBX1 TGFBR2 TRAF1 TGFBR1 HSP90B1 MET TGFB3 RALB RALA FOXO1 CSF3R BIRC2 APC SUFU PIAS4 LAMC3 CBLC WNT16 PLCG1 PLCG2 PLD1 BCR PAX8 CASP9 MAX CASP8 RARB FGF9 RARA FGF8 FGF7 FGF6 FGF5 MTOR FGF3 FGF4 KITLG FGF1 PTCH2 FGF2 FGF21 BAD MAPK9 MAPK10 WNT3A MAP2K2 MECOM NOS2 BAX MAP2K1 FGFR2 FZD3 KIT RB1 CASP3 FGFR3 PIAS2 FGFR1 FGF14 FGF17 FGF16 FGF10 CUL2 GRB2 FGF11 FGF12 WNT10A FGF13 WNT4 SMAD3 ETS1 SMAD4 SMAD2 RUNX1 IKBKG HRAS WNT1 RUNX1T1 WNT7A CBLB WNT7B CBL WNT8A WNT8B

WNT2 CTNNA2 FGF23 WNT3 RASSF5 WNT5A WNT6 CTNNB1 CCNA1 FGF18 STK4 STK36 FZD10 FZD5 PTEN PIK3R3 TCF7L1 RALGDS PTCH1 MITF CTNNA3 FOS LAMA1 PIAS3 NKX3-1 ITGA3 APC2 ITGA2B CREBBP COL4A6 PDGFRB MMP2 BRCA2 COL4A1 MMP1 BRAF COL4A2 MMP9 MLH1 COL4A4 SPI1 IKBKB MDM2 ARNT2 ITGAV ITGB1 AKT3 EGLN2 DAPK3 EGLN3 DAPK1 PIAS1 FGF20 RELA APPL1 FH VEGFA HSP90AA1 VEGFC VEGFB WNT5B CCND1 TRAF4 BCL2 PDGFA FGF22 PDGFB BCL2L1 PDGFRA MSH2 VHL VEGFD RALBP1 HSP90AB1 MSH3 CRK CYCS CRKL CCNE1 DCC CDC42 CDH1 SOS2 E2F1 CHUK ZBTB16 RAF1 PRKCB PRKCA SLC2A1 CEBPA FADD RHOA RAD51 ELOC PRKCG ELOB CXCL8 RAC2 DVL3 ERBB2 DVL2 BID TGFA RAC3 RXRB RXRG RXRA RASSF1 DVL1 SKP2 RET MAPK3 TCF7 DAPK2 FGF19 RAC1 SOS1 TCF7L2 MAPK1 MAPK8 EGFR EP300 EPAS1 GSK3B CDKN2A CDKN1B CDKN1A EGF LAMA3 LAMA2 CDKN2B ARAF AR ITGA6 ITGA2 GLI1 EGLN1 CDK2 BMP4 CDK4 BMP2 GLI2 PPARG GLI3 PPARD TP53 E2F3 E2F2 PIK3CA PIK3CB CCNE2 CDK6 FAS KLK3 PIK3CD FASLG FZD2 HDAC1 HDAC2 PIK3CG CKS1B IL6 NTRK1 PIK3R1 PIK3R2

KEGG_COLORECTAL_CANCER http://www.gsea-msigdb.org/gsea/msigdb/cards/KEGG_COLORECTAL_CANCER JUN CASP9 RAF1 BIRC5 APC2 RHOA LEF1 MSH6 BAD MAPK9 MAPK10 RAC2 RAC3 BRAF MLH1 MAP2K1 BAX AKT1 CASP3 AKT2 AKT3 AXIN1 AXIN2 SMAD3 MAPK3 SMAD4 TCF7 RAC1 MYC TCF7L2 SMAD2 MAPK1 PIK3R5 MAPK8 APPL1 GSK3B KRAS CTNNB1 TGFB2 TGFB1 ARAF TGFBR2 TGFBR1 TGFB3 CCND1 PIK3R3 BCL2 TCF7L1 MSH2 APC RALGDS TP53 MSH3 PIK3CA PIK3CB CYCS PIK3CD FOS PIK3CG DCC PIK3R1 PIK3R2

KEGG_RENAL_CELL_CARCINOMA http://www.gsea-msigdb.org/gsea/msigdb/cards/KEGG_RENAL_CELL_CARCINOMA JUN SOS2 PAK3 PTPN11 RAF1 HGF PAK2 SLC2A1 PAK4 CREBBP ELOC ELOB PGF MAP2K2 TGFA BRAF FLCN MAP2K1 HIF1A AKT1 ARNT2 AKT2 RAPGEF1 AKT3 NRAS ARNT CUL2 GRB2 MAPK3 ETS1 EGLN2 EGLN3 PAK6 RAC1 SOS1 MAPK1 PIK3R5 HRAS EP300 EPAS1 KRAS TGFB2 TGFB1 FH VEGFA ARAF VEGFC VEGFB RBX1 MET TGFB3 PAK5 RAP1A PIK3R3 RAP1B PDGFB EGLN1 VHL PAK1 VEGFD CRK PIK3CA PIK3CB CRKL PIK3CD GAB1

PIK3CG PIK3R1 CDC42 PIK3R2

KEGG_PANCREATIC_CANCER http://www.gsea-msigdb.org/gsea/msigdb/cards/KEGG_PANCREATIC_CANCER CASP9 E2F1 STAT3 CHUK RAF1 ARHGEF6 STAT1 RAD51 BAD PGF MAPK9 MAPK10 ERBB2 RAC2 BRCA2 TGFA BRAF RAC3 MAP2K1 IKBKB RB1 JAK1 AKT1 AKT2 AKT3 SMAD3 MAPK3 SMAD4 NFKB1 RAC1 SMAD2 MAPK1 PIK3R5 IKBKG MAPK8 EGFR RELA KRAS CDKN2A EGF TGFB2 TGFB1 VEGFA ARAF VEGFC VEGFB TGFBR2 TGFBR1 TGFB3 CCND1 PIK3R3 RALB BCL2L1 RALA CDK4 VEGFD RALBP1 RALGDS TP53 E2F3 E2F2 PIK3CA PIK3CB CDK6 PIK3CD PLD1 PIK3CG PIK3R1 CDC42 PIK3R2

KEGG_ENDOMETRIAL_CANCER http://www.gsea-msigdb.org/gsea/msigdb/cards/KEGG_ENDOMETRIAL_CANCER CASP9 SOS2 RAF1 ELK1 APC2 LEF1 BAD ERBB2 MAP2K2 BRAF MLH1 MAP2K1 PDPK1 ILK AKT1 AKT2 NRAS AKT3 GRB2 AXIN1 AXIN2 MAPK3 TCF7 CTNNA1 MYC SOS1 MAPK1 TCF7L2 PIK3R5 HRAS EGFR GSK3B KRAS CTNNA2 EGF CTNNB1 ARAF CCND1 PTEN PIK3R3 TCF7L1 FOXO3 APC TP53 PIK3CA PIK3CB PIK3CD CTNNA3 PIK3CG PIK3R1 PIK3R2 CDH1

KEGG_GLIOMA http://www.gsea-msigdb.org/gsea/msigdb/cards/KEGG_GLIOMA SOS2 CALM2 E2F1 PRKCB RAF1 SHC1 PRKCA CALM1 IGF1 MTOR IGF1R PRKCG PDGFRB MAP2K2 TGFA BRAF MAP2K1 MDM2 RB1 AKT1 SHC4 AKT2 NRAS AKT3 GRB2 MAPK3 SOS1 MAPK1 PIK3R5 HRAS EGFR KRAS CDKN2A SHC3 CDKN1A EGF ARAF CALML5 CCND1 PTEN PIK3R3 PDGFA CAMK2A PDGFB CAMK2B PDGFRA CAMK2D CAMK2G CDK4 TP53 E2F3 CALML3 E2F2 PIK3CA PIK3CB CDK6 PIK3CD PLCG1 SHC2 PLCG2 CALM3 PIK3CG PIK3R1 PIK3R2 CALML6

KEGG_PROSTATE_CANCER http://www.gsea-msigdb.org/gsea/msigdb/cards/KEGG_PROSTATE_CANCER CASP9 SOS2 E2F1 CHUK RAF1 IGF1 CREB3L4 MTOR CREB3L1 PDGFD NKX3-1 IGF1R GSTP1 CREB3 CREBBP LEF1 BAD INS PDGFRB ERBB2 MAP2K2 TGFA BRAF MAP2K1 FGFR2 PDPK1 IKBKB MDM2 RB1 CREB1 AKT1 AKT2 FGFR1 AKT3 NRAS GRB2 CREB5 MAPK3 TCF7 NFKB1 INSRR NFKBIA SOS1 TCF7L2 MAPK1 PIK3R5 IKBKG HRAS EP300 EGFR RELA PDGFC GSK3B KRAS CDKN1B EGF CDKN1A CTNNB1

HSP90AA1 ARAF AR CREB3L3 HSP90B1 SRD5A2 CREB3L2 CCND1 PTEN PIK3R3 BCL2 PDGFA PDGFB TCF7L1 FOXO1 PDGFRA CDK2 HSP90AB1 TP53 E2F3 E2F2 PIK3CA PIK3CB CCNE2 PIK3CD KLK3 ATF4 PIK3CG CCNE1 PIK3R1 PIK3R2

KEGG_THYROID_CANCERhttp://www.gsea-msigdb.org/gsea/msigdb/cards/KEGG_THYROID_CANCER PAX8 HRAS CCDC6 TFG NCOA4 KRAS CTNNB1 LEF1 CCND1 MAP2K2 TCF7L1 BRAF MAP2K1 RXRB RXRG RXRA PPARG TP53 RET NRAS TPR MAPK3 TCF7 NTRK1 MYC TPM3 CDH1 MAPK1 TCF7L2

KEGG_BASAL_CELL_CARCINOMA http://www.gsea-msigdb.org/gsea/msigdb/cards/KEGG_BASAL_CELL_CARCINOMA HHIP FZD1 APC2 FZD4 FZD6 PTCH2 FZD7 FZD8 LEF1 FZD9 WNT3A DVL3 WNT9B DVL2 WNT9A FZD3 DVL1 WNT2B WNT11 WNT10B SMO AXIN1 WNT4 WNT10A AXIN2 TCF7 TCF7L2 SHH WNT1 GSK3B WNT7A WNT7B WNT8A WNT8B WNT2 WNT3 WNT5A CTNNB1 WNT6 STK36 FZD10 WNT5B FZD5 GLI1 TCF7L1 BMP4 SUFU APC BMP2 GLI2 PTCH1 GLI3 WNT16 TP53 FZD2

KEGG_MELANOMA http://www.gsea-msigdb.org/gsea/msigdb/cards/KEGG_MELANOMA E2F1 RAF1 FGF9 HGF FGF8 FGF7 IGF1 FGF6 FGF5 PDGFD IGF1R FGF3 FGF4 FGF1 FGF2 FGF21 BAD PDGFRB MAP2K2 BRAF MAP2K1 MDM2 RB1 AKT1 AKT2 FGFR1 FGF14 FGF17 FGF16 AKT3 NRAS FGF10 FGF11 FGF12 FGF13 MAPK3 FGF19 MAPK1 PIK3R5 FGF20 HRAS EGFR PDGFC CDKN2A KRAS CDKN1A EGF FGF23 FGF18 ARAF MET CCND1 PTEN PIK3R3 PDGFA FGF22 PDGFB PDGFRA CDK4 TP53 E2F3 E2F2 PIK3CA PIK3CB CDK6 PIK3CD MITF PIK3CG PIK3R1 PIK3R2 CDH1

KEGG_BLADDER_CANCERhttp://www.gsea-msigdb.org/gsea/msigdb/cards/KEGG_BLADDER_CANCER HRAS E2F1 RAF1 EGFR CDKN2A KRAS EGF CDKN1A TYMP VEGFA ARAF VEGFC VEGFB PGF CXCL8 CCND1 ERBB2 MMP2 MMP1 MAP2K2 RPS6KA5 BRAF MMP9 MAP2K1 CDK4 MDM2 RB1 VEGFD RASSF1 TP53 E2F3 FGFR3 E2F2 NRAS MAPK3 DAPK3 DAPK2 DAPK1 THBS1 MYC CDH1 MAPK1

KEGG_CHRONIC_MYELOID_LEUKEMIA http://www.gsea-msigdb.org/gsea/msigdb/cards/KEGG_CHRONIC_MYELOID_LEUKEMIA SOS2

E2F1 CHUK PTPN11 RAF1 SHC1 BAD CTBP2 CTBP1 MAP2K2 BRAF MECOM MAP2K1 IKBKB MDM2 RB1 ABL1 AKT1 AKT2 SHC4 AKT3 NRAS GRB2 STAT5B SMAD3 MAPK3 STAT5A SMAD4 NFKB1 NFKBIA SOS1 MYC MAPK1 RUNX1 PIK3R5 IKBKG HRAS RELA GAB2 CBLB CDKN2A KRAS CBL SHC3 CDKN1B CDKN1A TGFB2 TGFB1 ARAF TGFBR2 TGFBR1 TGFB3 CCND1 PIK3R3 BCL2L1 CDK4 CBLC TP53 E2F3 E2F2 CRK PIK3CA PIK3CB CDK6 CRKL PIK3CD SHC2 HDAC1 BCR HDAC2 PIK3CG PIK3R1 PIK3R2

KEGG_ACUTE_MYELOID_LEUKEMIA http://www.gsea-msigdb.org/gsea/msigdb/cards/KEGG_ACUTE_MYELOID_LEUKEMIA SOS2 CHUK STAT3 ZBTB16 RAF1 RARA PML JUP MTOR CEBPA LEF1 EIF4EBP1 BAD PIM2 MAP2K2 BRAF MAP2K1 KIT FLT3 SPI1 IKBKB AKT1 AKT2 RPS6KB1 NRAS AKT3 RPS6KB2 GRB2 STAT5B MAPK3 TCF7 STAT5A NFKB1 MYC SOS1 TCF7L2 MAPK1 RUNX1 PIK3R5 IKBKG HRAS RUNX1T1 RELA KRAS CCNA1 ARAF CCND1 PIK3R3 TCF7L1 PPARD PIK3CA PIK3CB PIM1 PIK3CD PIK3CG PIK3R1 PIK3R2

KEGG_SMALL_CELL_LUNG_CANCER http://www.gsea-msigdb.org/gsea/msigdb/cards/KEGG_SMALL_CELL_LUNG_CANCER CASP9 MAX E2F1 CHUK LAMA1 RARB PIAS3 FN1 BIRC3 XIAP ITGA3 ITGA2B COL4A6 PTK2 COL4A1 COL4A2 PTGS2 NOS2 COL4A4 RXRB RXRG IKBKB RXRA RB1 SKP2 AKT1 AKT2 PIAS2 ITGAV LAMC2 ITGB1 AKT3 LAMB2 LAMB3 LAMC1 LAMA4 LAMA5 LAMB1 NFKB1 NFKBIA MYC PIK3R5 PIAS1 IKBKG LAMB4 RELA APAF1 CDKN1B LAMA3 LAMA2 TRAF6 TRAF5 TRAF3 CDKN2B TRAF2 FHIT TRAF1 ITGA6 CCND1 PTEN ITGA2 TRAF4 PIK3R3 BCL2 BCL2L1 BIRC2 CDK2 PIAS4 CDK4 LAMC3 TP53 E2F3 E2F2 PIK3CA PIK3CB CCNE2 CYCS CDK6 PIK3CD PIK3CG CCNE1 PIK3R1 CKS1B PIK3R2

KEGG_NON_SMALL_CELL_LUNG_CANCER http://www.gsea-msigdb.org/gsea/msigdb/cards/KEGG_NON_SMALL_CELL_LUNG_CANCER CASP9 SOS2 E2F1 PRKCB RAF1 PRKCA RARB BAD PRKCG ERBB2 MAP2K2 TGFA BRAF MAP2K1 RXRB PDPK1 RXRG RB1 RXRA RASSF1 AKT1 AKT2 NRAS AKT3 GRB2 MAPK3 SOS1 MAPK1 PIK3R5 HRAS EGFR KRAS CDKN2A EGF RASSF5 FHIT STK4 ARAF CCND1 PIK3R3 FOXO3 CDK4 TP53 E2F3 E2F2

PIK3CA PIK3CB CDK6 PIK3CD PLCG1 PLCG2 PIK3CG PIK3R1 PIK3R2

KEGG_ASTHMA http://www.gsea-msigdb.org/gsea/msigdb/cards/KEGG_ASTHMA HLA-DRB4 HLA-DRB5 HLA-DOA HLA-DOB HLA-DRB3 IL3 TNF CCL11 EPX FCER1G MS4A2 HLA-DMB FCER1A HLA-DMA IL9 CD40LG HLA-DPA1 CD40 HLA-DPB1 IL13 HLA-DQA1 HLA-DQA2 HLA-DQB1 PRG2 RNASE3 IL4 IL5 IL10 HLA-DRB1 HLA-DRA

KEGG_AUTOIMMUNE_THYROID_DISEASE http://www.gsea-msigdb.org/gsea/msigdb/cards/KEGG_AUTOIMMUNE_THYROID_DISEASE HLA-DOA HLA-DOB CD80 CD86 CD28 IFNA5 IFNA4 IFNA2 TSHR TSHB IFNA1 HLA-C HLA-B HLA-DMB HLA-DMA HLA-A HLA-G TG CGA IFNA17 IFNA21 IFNA6 IFNA7 PRF1 IFNA8 IFNA10 IFNA13 IFNA14 GZMB IFNA16 TPO IL10 CTLA4 HLA-DRB4 HLA-DRB5 HLA-DRB3 HLA-F HLA-E IL2 CD40LG HLA-DPA1 CD40 HLA-DPB1 HLA-DQA1 HLA-DQA2 HLA-DQB1 FAS FASLG IL4 IL5 HLA-DRB1 HLA-DRA

KEGG_ALLOGRAFT_REJECTION http://www.gsea-msigdb.org/gsea/msigdb/cards/KEGG_ALLOGRAFT_REJECTION HLA-DRB4 HLA-DRB5 HLA-DOA HLA-DOB HLA-DRB3 CD80 CD86 CD28 TNF IFNG HLA-C HLA-B HLA-DMB HLA-DMA HLA-F HLA-E HLA-A IL2 CD40LG HLA-DPA1 CD40 IL12B HLA-DPB1 HLA-DQA1 HLA-G HLA-DQA2 IL12A HLA-DQB1 PRF1 FAS FASLG GZMB IL4 IL5 IL10 HLA-DRB1 HLA-DRA

KEGG_GRAFT_VERSUS_HOST_DISEASE http://www.gsea-msigdb.org/gsea/msigdb/cards/KEGG_GRAFT_VERSUS_HOST_DISEASE HLA-DRB4 KIR2DL1 HLA-DRB5 HLA-DOA HLA-DOB HLA-DRB3 CD80 CD86 KLRD1 KLRC1 CD28 TNF KIR2DL2 KIR2DL3 IFNG HLA-C HLA-B HLA-DMB IL1B HLA-DMA HLA-F HLA-E HLA-A IL2 HLA-DPA1 HLA-DPB1 HLA-DQA1 HLA-G KIR3DL1 HLA-DQA2 KIR3DL2 HLA-DQB1 IL1A PRF1 FAS FASLG GZMB KIR2DL5A IL6 HLA-DRB1 HLA-DRA

KEGG_VIRAL_MYOCARDITIS http://www.gsea-msigdb.org/gsea/msigdb/cards/KEGG_VIRAL_MYOCARDITIS CASP9 CASP8 HLA-DOA HLA-DOB CD80 CD86 CD28 EIF4G3 ITGAL ICAM1 CXADR MYH13 HLA-C HLA-B MYH14 HLA-DMB HLA-DMA MYH1 HLA-A DMD MYH15 RAC2 BID HLA-G RAC3 ABL1 ABL2 CASP3 EIF4G2 PRF1 EIF4G1 ITGB2 ACTG1 RAC1 SGCD HLA-DRB4 SGCG

HLA-DRB5 SGCA SGCB HLA-DRB3 DAG1 CD55 LAMA2 ACTB HLA-F HLA-E CD40LG CCND1 HLA-DPA1 CD40 HLA-DPB1 MYH7B HLA-DQA1 HLA-DQA2 HLA-DQB1 CAV1 MYH3 MYH2 CYCS MYH4 MYH7 MYH6 MYH9 MYH8 MYH11 FYN MYH10 HLA-DRB1 HLA-DRA

```
######Video source: https://ke.biowolf.cn
######ÉúÐÅ×ÔÑ§Íø: https://www.biowolf.cn/
######Î¢ÐÅ¹«ÖÚºÅ£ºbiowolf_cn
######ºÏ×÷ÓÊÏä£ºbiowolf@foxmail.com
######´ðÒÉÎ¢ÐÅ: 18520221056

#install.packages("colorspace")
#install.packages("stringi")
#install.packages("ggplot2")

#if (!requireNamespace("BiocManager", quietly = TRUE))
#    install.packages("BiocManager")
#BiocManager::install("org.Hs.eg.db")
#BiocManager::install("DOSE")
#BiocManager::install("clusterProfiler")
#BiocManager::install("enrichplot")
#BiocManager::install("GSEABase")


#ÒýÓÃ°ü
library("clusterProfiler")
library("org.Hs.eg.db")
library("enrichplot")
library("ggplot2")
library("GSEABase")
library("DOSE")

pvalueFilter=0.05       #pÖµ¹ýÂËÌõ¼þ
qvalueFilter=0.05       #½ÃÕý¹óµÄpÖµ¹ýÂËÌõ¼þ

#¶¨ÒåÑÕÉ«
colorSel="qvalue"
if(qvalueFilter>0.05){
   colorSel="pvalue"
}

setwd("C:\\biowolf\\Diagnostic\\10.DO")
#ÉèÖÃ¹¤×÷Ä¿Â¼
```

```
rt=read.table("diff.txt", header=T, sep="\t",
check.names=F)     #¶ÁÈ¡ÊäÈëÎÄ¼þ

#»ùÒòÃû×Ö×ª»»Îª»ùÒòid
genes=as.vector(rt[,1])
entrezIDs=mget(genes, org.Hs.egSYMBOL2EG, ifnotfound=NA)
entrezIDs=as.character(entrezIDs)
rt=cbind(rt,entrezID=entrezIDs)
gene=entrezIDs[entrezIDs!="NA"]       #È¥³ý»ùÒòidÎªNAµÄ»ùÒò

#DO¸»¼¯·ÖÎö
kk=enrichDO(gene=gene, ont="DO", pvalueCutoff=1,
qvalueCutoff=1, readable=TRUE)
DO=as.data.frame(kk)
DO=DO[(DO$pvalue<pvalueFilter & DO$qvalue<qvalueFilter),]
#±£´æ¸»¼¯½á¹û
write.table(DO, file="DO.txt", sep="\t", quote=F,
row.names = F)

#¶¨ÒåÏÔÊ¾¼²²¡ÊýÄ¿
showNum=30
if(nrow(DO)<showNum){
   showNum=nrow(DO)
}

#Öù×´Í¼
pdf(file="barplot.pdf", width=6, height=6)
barplot(kk, drop=TRUE, showCategory=showNum,
color=colorSel)
dev.off()

#ÆøÅÝÍ¼
pdf(file="bubble.pdf", width = 6, height = 6)
dotplot(kk, showCategory=showNum, orderBy="GeneRatio",
color=colorSel)
dev.off()
```

######Video source: https://ke.biowolf.cn

######ÉúÐÅ×ÔÑ§Íø: https://www.biowolf.cn/

######Î¢ÐÅ¹«ÖÚºÅ£ºbiowolf_cn

######ºÏ×÷ÓÊÏä£ºbiowolf@foxmail.com

######´ðÒÉÎ¢ÐÅ: 18520221056

```
######Video source: https://ke.biowolf.cn
######ÉúÐÅ×ÔÑ§Íø: https://www.biowolf.cn/
######Î¢ÐÅ¹«ÖÚºÅ£ºbiowolf_cn
######ºÏ×÷ÓÊÏä£ºbiowolf@foxmail.com
######´ðÒÉÎ¢ÐÅ: 18520221056

#install.packages("colorspace")
#install.packages("stringi")
#install.packages("ggplot2")

#if (!requireNamespace("BiocManager", quietly = TRUE))
#    install.packages("BiocManager")
#BiocManager::install("org.Hs.eg.db")
#BiocManager::install("DOSE")
#BiocManager::install("clusterProfiler")
#BiocManager::install("enrichplot")



#ÒýÓÃ°ü
library("clusterProfiler")
library("org.Hs.eg.db")
library("enrichplot")
library("ggplot2")

pvalueFilter=0.05       #pÖµ¹ýÂËÌõ¼þ
qvalueFilter=0.05       #½ÃÕý°óµÄpÖµ¹ýÂËÌõ¼þ

#¶¨ÒåÑÕÉ«
colorSel="qvalue"
if(qvalueFilter>0.05){
   colorSel="pvalue"
}

setwd("C:\\biowolf\\Diagnostic\\08.GO")
#ÉèÖÃ¹¤×÷Ä¿Â¼
rt=read.table("diff.txt", header=T, sep="\t",
check.names=F)     #¶ÁÈ¡ÊäÈëÎÄ¼þ
```

```
#»ùÒòÃû×Ö×ª»»Îª»ùÒòid
genes=as.vector(rt[,1])
entrezIDs=mget(genes, org.Hs.egSYMBOL2EG, ifnotfound=NA)
entrezIDs=as.character(entrezIDs)
gene=entrezIDs[entrezIDs!="NA"]        #È¥³ý»ùÒòidÎªNAµÄ»ùÒò

#GO¸»¼¯·ÖÎö
kk=enrichGO(gene=gene, OrgDb=org.Hs.eg.db, pvalueCutoff=1,
qvalueCutoff=1, ont="all", readable=T)
GO=as.data.frame(kk)
GO=GO[(GO$pvalue<pvalueFilter & GO$qvalue<qvalueFilter),]
#±£´æ¸»¼¯½á¹û
write.table(GO, file="GO.txt", sep="\t", quote=F,
row.names = F)

#¶¨ÒåÏÔÊ¾GOµÄÊýÄ¿
showNum=10
if(nrow(GO)<30){
   showNum=nrow(GO)
}

#Öù×´Í¼
pdf(file="barplot.pdf", width=10, height=7)
bar=barplot(kk, drop=TRUE, showCategory=showNum,
split="ONTOLOGY", color=colorSel) + facet_grid(ONTOLOGY~.,
scale='free')
print(bar)
dev.off()

#ÆøÅÝÍ¼
pdf(file="bubble.pdf", width=10, height=7)
bub=dotplot(kk, showCategory=showNum, orderBy="GeneRatio",
split="ONTOLOGY", color=colorSel) + facet_grid(ONTOLOGY~.,
scale='free')
print(bub)
dev.off()
```

######Video source: https://ke.biowolf.cn
######ÉúÐÅ×ÔÑ§Íø: https://www.biowolf.cn/
######Î¢ÐÅ¹«ÖÚºÅ£ºbiowolf_cn
######ºÏ×÷ÓÊÏä£ºbiowolf@foxmail.com
######´ðÒÉÎ¢ÐÅ: 18520221056

```
######Video source: https://ke.biowolf.cn
######ÉúÐÅ×ÔÑ§Íø: https://www.biowolf.cn/
######Î¢ÐÅ¹«ÖÚºÅ£ºbiowolf_cn
######ºÏ×÷ÓÊÏä£ºbiowolf@foxmail.com
######´ðÒÉÎ¢ÐÅ: 18520221056

#if (!requireNamespace("BiocManager", quietly = TRUE))
#    install.packages("BiocManager")
#BiocManager::install("limma")
#BiocManager::install("org.Hs.eg.db")
#BiocManager::install("DOSE")
#BiocManager::install("clusterProfiler")
#BiocManager::install("enrichplot")


#ÒýÓÃ°ü
library(limma)
library(org.Hs.eg.db)
library(clusterProfiler)
library(enrichplot)

inputFile="all.txt"        #ÊäÈëÎÄ¼þ
gmtFile="c2.cp.kegg.v7.4.symbols.gmt"     #»ùÒò¼¯ÎÄ¼þ
setwd("C:\\biowolf\\Diagnostic\\11.GSEA")
#ÉèÖÃ¹¤×÷Ä¿Â¼

#¶ÁÈ¡ÎÄ¼þ,²¢¶ÔÊäÈëÎÄ¼þ½øÐÐÕûÀí
rt=read.table(inputFile, header=T, sep="\t",
check.names=F)
logFC=as.vector(rt[,2])
names(logFC)=as.vector(rt[,1])
logFC=sort(logFC, decreasing=T)

#¶ÁÈë»ùÒò¼¯ÎÄ¼þ
gmt=read.gmt(gmtFile)

#¸»¼¯·ÖÎö
kk=GSEA(logFC, TERM2GENE=gmt, pvalueCutoff = 1)
```

```
kkTab=as.data.frame(kk)
kkTab=kkTab[kkTab$p.adjust<0.05,]
write.table(kkTab,file="GSEA.result.txt",sep="\t",quote=F,
row.names = F)

#Êä³öÊµÑé×é¸»¼¯µÄÍ¼ÐÎ
termNum=5     #Õ¹Ê¾Í¨Â·µÄÊýÄ¿
kkUp=kkTab[kkTab$NES>0,]
if(nrow(kkUp)>=termNum){
   showTerm=row.names(kkUp)[1:termNum]
   gseaplot=gseaplot2(kk, showTerm, base_size=8,
title="Enriched in Treat")
   pdf(file="GSEA.treat.pdf", width=7, height=5.5)
   print(gseaplot)
   dev.off()
}

#Êä³öÕý³£×é¸»¼¯µÄÍ¼ÐÎ
termNum=5     #Õ¹Ê¾Í¨Â·µÄÊýÄ¿
kkDown=kkTab[kkTab$NES<0,]
if(nrow(kkDown)>=termNum){
   showTerm=row.names(kkDown)[1:termNum]
   gseaplot=gseaplot2(kk, showTerm, base_size=8,
title="Enriched in Control")
   pdf(file="GSEA.con.pdf", width=7, height=5.5)
   print(gseaplot)
   dev.off()
}


######Video source: https://ke.biowolf.cn
######ÉúÐÅ×ÔÑ§Íø: https://www.biowolf.cn/
######Î¢ÐÅ¹«ÖÚ°Å£°biowolf_cn
######°Ï×÷ÓÊÏä£°biowolf@foxmail.com
######´ðÒÉÎ¢ÐÅ: 18520221056
```

```
#if (!requireNamespace("BiocManager", quietly = TRUE))
#    install.packages("BiocManager")
#BiocManager::install("limma")

#install.packages("reshape2")
#install.packages("ggpubr")
#install.packages("ggExtra")


#引用包
library(limma)
library(reshape2)
library(ggpubr)
library(ggExtra)

gene="TMEM27"                        #基因名称
expFile="normalize.txt"              #表达数据文件
immFile="CIBERSORT-Results.txt"      #免疫细胞浸润结果文件
setwd("C:\\biowolf\\Diagnostic\\21.immuneCor")      #设置工作目录

#读取基因表达文件,并对数据进行处理
rt=read.table(expFile, header=T, sep="\t", check.names=F)
rt=as.matrix(rt)
rownames(rt)=rt[,1]
exp=rt[,2:ncol(rt)]
dimnames=list(rownames(exp),colnames(exp))
data=matrix(as.numeric(as.matrix(exp)),nrow=nrow(exp),dimnames=dimname
s)
data=avereps(data)

#获取目标基因表达量
data=t(data[gene,,drop=F])
data=as.data.frame(data)

#读取免疫细胞结果文件，并对数据进行整理
immune=read.table(immFile, header=T, sep="\t", check.names=F,
row.names=1)
```

```
#数据合并
sameSample=intersect(row.names(immune), row.names(data))
rt=cbind(immune[sameSample,,drop=F], data[sameSample,,drop=F])

#对免疫细胞进行循环，绘制相关性散点图
outTab=data.frame()
for(i in colnames(rt)[1:(ncol(rt)-1)]){
    x=as.numeric(rt[,gene])
    y=as.numeric(rt[,i])
    if(sd(y)==0){y[1]=0.00001}
    cor=cor.test(x, y, method="spearma")

    outVector=cbind(Gene=gene, Cell=i, cor=cor$estimate,
pvalue=cor$p.value)
    outTab=rbind(outTab,outVector)

    if(cor$p.value<0.05){
        outFile=paste0("cor.", i, ".pdf")
        df1=as.data.frame(cbind(x,y))
        p1=ggplot(df1, aes(x, y)) +
                  xlab(paste0(gene, " expression")) + ylab(i)+
                  geom_point() + geom_smooth(method="lm",formula = y ~
x) + theme_bw()+
                  stat_cor(method = 'spearman', aes(x =x, y =y))
        p2=ggMarginal(p1, type="density", xparams=list(fill =
"orange"), yparams=list(fill = "blue"))
        #相关性图形
        pdf(file=outFile, width=5.2, height=5)
        print(p2)
        dev.off()
    }
}
#输出免疫功能和 p 值表格文件
write.table(outTab,file="cor.result.txt",sep="\t",row.names=F,quote=F)
```

```
#install.packages("glmnet")


set.seed(123)
library(glmnet)                    #引用包
inputFile="diffGeneExp.txt"        #输入文件
setwd("C:\\biowolf\\Diagnostic\\12.lasso")        #设置工作目录

#读取输入文件
rt=read.table(inputFile, header=T, sep="\t", check.names=F,
row.names=1)
rt=t(rt)

#构建模型
x=as.matrix(rt)
y=gsub("(.*)\\_(.*)", "\\2", row.names(rt))
fit=glmnet(x, y, family = "binomial", alpha=1)
cvfit=cv.glmnet(x, y, family="binomial",
alpha=1,type.measure='deviance',nfolds = 10)
pdf(file="cvfit.pdf",width=6,height=5.5)
plot(cvfit)
dev.off()

#输出筛选的特征基因
coef=coef(fit, s = cvfit$lambda.min)
index=which(coef != 0)
lassoGene=row.names(coef)[index]
lassoGene=lassoGene[-1]
write.table(lassoGene, file="LASSO.gene.txt", sep="\t", quote=F,
row.names=F, col.names=F)
```

```
#install.packages("pROC")


library(pROC)                #ÒýÓÃ°ü
expFile="diffGeneExp.txt"      #±í´ïÊý¾ÝÎÄ¼þ
geneFile="interGenes.txt"      #½»¼¯»ùÒòÁÐ±íÎÄ¼þ
setwd("C:\\biowolf\\Diagnostic\\16.ROC")    #ÉèÖÃ¹¤×÷Ä¿Â¼

#¶ÁÈ¡ÊäÈëÎÄ¼þ£¬²¢¶ÔÊäÈëÎÄ¼þÕûÀí
rt=read.table(expFile, header=T, sep="\t", check.names=F,
row.names=1)
y=gsub("(.*)\\_(.*)", "\\2", colnames(rt))
y=ifelse(y=="con", 0, 1)

#¶ÁÈ¡»ùÒòÁÐ±íÎÄ¼þ
geneRT=read.table(geneFile, header=F, sep="\t",
check.names=F)

#¶Ô½»¼¯»ùÒò½øÐÐÑ»·£¬»æÖÆROCÇúÏß
for(x in as.vector(geneRT[,1])){
   #»æÖÆROCÇúÏß
   roc1=roc(y, as.numeric(rt[x,]))
   ci1=ci.auc(roc1, method="bootstrap")
   ciVec=as.numeric(ci1)
   pdf(file=paste0("ROC.",x,".pdf"), width=5, height=5)
   plot(roc1, print.auc=TRUE, col="red", legacy.axes=T,
main=x)
   text(0.39, 0.43, paste0("95% CI:
",sprintf("%.03f",ciVec[1]),"-
",sprintf("%.03f",ciVec[3])), col="red")
   dev.off()
}
```

```
#if (!requireNamespace("BiocManager", quietly = TRUE))
#    install.packages("BiocManager")
#BiocManager::install("limma")

#if (!requireNamespace("BiocManager", quietly = TRUE))
#    install.packages("BiocManager")
#BiocManager::install("sva")


#引用包
library(limma)
library(sva)
outFile="merge.txt"       #输出文件
setwd("C:\\biowolf\\Diagnostic\\05.sva")      #设置工作目录

#获取目录下所有".txt"结尾的文件
files=dir()
files=grep("txt$", files, value=T)
geneList=list()

#读取所有 txt 文件中的基因信息，保存到 geneList
for(file in files){
    if(file==outFile){next}
    rt=read.table(file, header=T, sep="\t", check.names=F)      #读取
输入文件
    geneNames=as.vector(rt[,1])      #提取基因名称
    uniqGene=unique(geneNames)       #基因取 unique
    header=unlist(strsplit(file, "\\.|\\-"))
    geneList[[header[1]]]=uniqGene
}

#获取交集基因
interGenes=Reduce(intersect, geneList)

#数据合并
allTab=data.frame()
batchType=c()
```

```
for(i in 1:length(files)){
    inputFile=files[i]
    header=unlist(strsplit(inputFile, "\\.|\\-"))
    #读取输入文件，并对输入文件进行整理
    rt=read.table(inputFile, header=T, sep="\t", check.names=F)
    rt=as.matrix(rt)
    rownames(rt)=rt[,1]
    exp=rt[,2:ncol(rt)]
    dimnames=list(rownames(exp),colnames(exp))

data=matrix(as.numeric(as.matrix(exp)),nrow=nrow(exp),dimnames=dimname
s)
    rt=avereps(data)

    #对数值大的数据取 log2
    qx=as.numeric(quantile(rt, c(0, 0.25, 0.5, 0.75, 0.99, 1.0),
na.rm=T))
    LogC=( (qx[5]>100) || ( (qx[6]-qx[1])>50 && qx[2]>0) )
    if(LogC){
       rt[rt<0]=0
        rt=log2(rt+1)}
    rt=normalizeBetweenArrays(rt)

    #数据合并
    if(i==1){
       allTab=rt[interGenes,]
    }else{
       allTab=cbind(allTab, rt[interGenes,])
    }
    batchType=c(batchType, rep(i,ncol(rt)))
}

#对数据进行矫正，输出矫正后的结果
outTab=ComBat(allTab, batchType, par.prior=TRUE)
outTab=rbind(geneNames=colnames(outTab), outTab)
write.table(outTab, file="merge.txt", sep="\t", quote=F, col.names=F)
```

```
#if (!requireNamespace("BiocManager", quietly = TRUE))
#    install.packages("BiocManager")
#BiocManager::install("limma")

#install.packages("ggpubr")


#ÒýÓÃ°ü
library(limma)
library(ggpubr)

expFile="GSE118916.txt"         #±í´ïÊý¾ÝÎÄ¼þ
conFile="GSE118916_s1.txt"       #¶ÔÕÕ×éÑùÆ·ÐÅÏ¢ÎÄ¼þ
treatFile="GSE118916_s2.txt"     #ÊµÑé×éÑùÆ·ÐÅÏ¢ÎÄ¼þ
geneFile="interGenes.txt"       #»ùÒòÁÐ±íÎÄ¼þ
setwd("C:\\biowolf\\Diagnostic\\15.testDiff")
#ÉèÖÃ¹¤×÷Ä¿Â¼

#¶ÁÈ¡ÊäÈëÎÄ¼þ
rt=read.table(expFile, header=T, sep="\t", check.names=F)
rt=as.matrix(rt)
rownames(rt)=rt[,1]
exp=rt[,2:ncol(rt)]
dimnames=list(rownames(exp), colnames(exp))
data=matrix(as.numeric(as.matrix(exp)), nrow=nrow(exp),
dimnames=dimnames)
rt=avereps(data)

#¶ÔÊýÖµ´óµÄÊý¾ÝÈ¡log2
qx=as.numeric(quantile(rt, c(0, 0.25, 0.5, 0.75, 0.99,
1.0), na.rm=T))
LogC=( (qx[5]>100) || ( (qx[6]-qx[1])>50 && qx[2]>0) )
if(LogC){
   rt[rt<0]=0
   rt=log2(rt+1)}
data=normalizeBetweenArrays(rt)
```

```
#ÅÐ¶ÏÑùÆ·ÀàÐÍ
con=read.table(conFile, header=F, sep="\t", check.names=F)
treat=read.table(treatFile, header=F, sep="\t",
check.names=F)
conData=data[,as.vector(con[,1])]
treatData=data[,as.vector(treat[,1])]
data=cbind(conData, treatData)
conNum=ncol(conData)
treatNum=ncol(treatData)

#Êä³ö½ÃÕý°óµÄ±í´ïÁ¿
Type=c(rep("con",conNum), rep("treat",treatNum))
outData=rbind(id=paste0(colnames(data),"_",Type),data)
write.table(outData, file="test.normalize.txt", sep="\t",
quote=F, col.names=F)

#¶ÁÈ¡½»¼¯»ùÒòµÄÎÄ¼þ,ÌáÈ¡Ä¿±ê»ùÒò±í´ïÁ¿
geneRT=read.table(geneFile, header=F, sep="\t",
check.names=F)
data=data[as.vector(geneRT[,1]),,drop=F]

#ÉèÖÃ±È½Ï×é
Type=c(rep("Con",conNum), rep("Treat",treatNum))
my_comparisons=list()
my_comparisons[[1]]=levels(factor(Type))

#²îÒì·ÖÎö
newGeneLists=c()
outTab=data.frame()
for(i in row.names(data)){
   #data[i,][data[i,]>quantile(data[i,],
0.99)]=quantile(data[i,], 0.99)
   rt1=data.frame(expression=data[i,], Type=Type)

   #¶Ô²îÒì»ùÒò½øÐÐ¿ÉÊÓ»¯£¬»æÖÆÏäÏßÍ¼
   boxplot=ggboxplot(rt1, x="Type", y="expression",
color="Type",
                  xlab="",
```

```
                  ylab=paste(i, "expression"),
                  legend.title="",
                  palette = c("blue", "red"),
                  add = "jitter")+
       stat_compare_means(comparisons = my_comparisons)

    #Êä³öÍ¼Æ¬
    pdf(file=paste0("boxplot.",i,".pdf"), width=5,
height=4.5)
    print(boxplot)
    dev.off()
}
```

```
#install.packages("venn")


library(venn)                    #引用包
outFile="interGenes.txt"         #输出文件名称
setwd("C:\\biowolf\\Diagnostic\\14.venn")     #设置工作目录
geneList=list()

#读取lasso回归的结果文件
rt=read.table("LASSO.gene.txt", header=F, sep="\t", check.names=F)
geneNames=as.vector(rt[,1])       #提取基因名称
uniqGene=unique(geneNames)        #基因取unique
geneList[["LASSO"]]=uniqGene

#读取SVM的结果文件
rt=read.table("SVM-RFE.gene.txt", header=F, sep="\t", check.names=F)
geneNames=as.vector(rt[,1])       #提取基因名称
uniqGene=unique(geneNames)        #基因取unique
geneList[["SVM-RFE"]]=uniqGene

#绘制venn图
mycol=c("blue2","red2")
pdf(file="venn.pdf", width=5, height=5)
venn(geneList,col=mycol[1:length(geneList)],zcolor=mycol[1:length(geneList)],box=F,ilabels=F)
dev.off()

#保存交集基因
intersectGenes=Reduce(intersect,geneList)
write.table(file=outFile, intersectGenes, sep="\t", quote=F, col.names=F, row.names=F)
```

```
#install.packages("vioplot")


library(vioplot)                    #ÒýÓÃ°ü
inputFile="CIBERSORT-Results.txt"     #ÊäÈëÎÄ¼þ
setwd("C:\\biowolf\\Diagnostic\\20.vioplot")
#ÉèÖÃ¹¤×÷Ä¿Â¼

#¶ÁÈ¡ÃâÒßÏ¸°û½þÈóÎÄ¼þ
rt=read.table(inputFile, header=T, sep="\t",
check.names=F, row.names=1)

#¶ÔÑùÆ··Ö×é
con=grepl("_con", rownames(rt), ignore.case=T)
treat=grepl("_treat", rownames(rt), ignore.case=T)
conData=rt[con,]
treatData=rt[treat,]
conNum=nrow(conData)
treatNum=nrow(treatData)
rt=rbind(conData,treatData)

#Êä³öÐ¡ÌáÇÙÍ¼
outTab=data.frame()
pdf(file="vioplot.pdf", height=8, width=13)
par(las=1,mar=c(10,6,3,3))
x=c(1:ncol(rt))
y=c(1:ncol(rt))
plot(x,y,
    xlim=c(0,63),ylim=c(min(rt),max(rt)+0.05),
    main="",xlab="", ylab="Fraction",
    pch=21,
    col="white",
    xaxt="n")

#¶ÔÃ¿¸öÃâÒßÏ¸°ûÑ»·£¬»æÖÆvioplot£¬¶ÔÕÕ×éÓÃÀ¶É«±íÊ¾£¬ÊµÑé×é
ÓÃºìÉ«±íÊ¾
for(i in 1:ncol(rt)){
```

```
      if(sd(rt[1:conNum,i])==0){
        rt[1,i]=0.00001
      }
      if(sd(rt[(conNum+1):(conNum+treatNum),i])==0){
        rt[(conNum+1),i]=0.00001
      }
      conData=rt[1:conNum,i]
      treatData=rt[(conNum+1):(conNum+treatNum),i]
      vioplot(conData,at=3*(i-1),lty=1,add = T,col =
'blue')
      vioplot(treatData,at=3*(i-1)+1,lty=1,add = T,col =
'red')
      wilcoxTest=wilcox.test(conData,treatData)
      p=wilcoxTest$p.value
      if(p<0.05){
          cellPvalue=cbind(Cell=colnames(rt)[i],pvalue=p)
          outTab=rbind(outTab,cellPvalue)
      }
      mx=max(c(conData,treatData))
      lines(c(x=3*(i-1)+0.2,x=3*(i-1)+0.8),c(mx,mx))
      text(x=3*(i-1)+0.5, y=mx+0.02, labels=ifelse(p<0.001,
paste0("p<0.001"), paste0("p=",sprintf("%.03f",p))), cex =
0.8)
}
legend("topright",
       c("Con", "Treat"),
       lwd=3,bty="n",cex=1,
       col=c("blue","red"))
text(seq(1,64,3),-0.05,xpd = NA,labels=colnames(rt),cex =
1,srt = 45,pos=2)
dev.off()

#Êä³öÃâÒßÏ¸°û°ÍpÖµ±í¸ñÎÄ¼þ
write.table(outTab,file="immuneDiff.xls",sep="\t",row.name
s=F,quote=F)
```
