# Supplementary material for: Identification of senescence-related biomarker for aortic dissection based on bioinformatics and machine learning algorithms
Source: Medicine (Baltimore). 2026 May 29;105(22):e48873. doi: 10.1097/MD.0000000000048873 (PMC13249447; doi:10.1097/MD.0000000000048873)
Supplement: Supplementary file 5 [file medi-105-e48873-s005.docx]

**Supplementary file 5 Table S4.** DEGs between AD and control groups.

| SLC11A1 |
| --- |
| MT1X |
| TNC |
| MT2A |
| CXCL5 |
| SIGLEC9 |
| SERPINA1 |
| PLP1 |
| ANGPTL4 |
| HK3 |
| IL31RA |
| MT1A |
| SERPINA3 |
| PROM1 |
| GPM6A |
| THBD |
| FBXL22 |
| SIGLEC10 |
| PRIMA1 |
| CCL2 |
| NRXN1 |
| PTX3 |
| ACTC1 |
| MYRIP |
| C11orf91 |
| MTTP |
| PHYHIP |
| LIF |
| ST14 |
| SLC38A5 |
| HMOX1 |
| ADRA1D |
| GRIK3 |
| GCKR |
| TREM1 |
| MARCO |
| LGI1 |
| PPP1R1A |
| PLEKHA6 |
| CNTFR |
| IL1RL1 |
| LSAMP |
| HMGA1 |
| CLSTN2 |
| APOD |
| FOSL1 |
| FRMD7 |
| CCL20 |
| RERGL |
| RAMP3 |
| CXorf36 |
| C16orf89 |
| SLCO2A1 |
| PALMD |
| SORCS1 |
| SOX10 |
| SPP1 |
| NRN1 |
| SCN7A |
| FGF12 |
| MT1G |
| OLFML2A |
| CHRDL1 |
| GREM2 |
| P2RY14 |
| VEGFA |
| NOSTRIN |
| SLC39A14 |
| MPZL2 |
| PTPRB |
| KANK4 |
| CXCL14 |
| CDH8 |
| PLIN4 |
| PCSK1 |
| MEOX1 |
| NEBL |
| SGCG |
| ESM1 |
| MMRN1 |
| LHX6 |
| FPR2 |
| RSPO3 |
| VIT |
| C2CD4A |
| ADCY4 |
| PI16 |
| SELP |
| DIO3 |
| SCARA5 |
| SPOCD1 |
| PCDH17 |
| VWF |
| SCN4B |
| SEMA3G |
| CA9 |
| AQP7 |
| CA4 |
| IL6 |
| TRIL |
| CCL7 |
| OLR1 |
| ADRA2B |
| GPIHBP1 |
| EBF2 |
| SHE |
| PI15 |
| ADIPOQ |
| RELN |
| CFD |
| SELE |
