# Supplementary material for: Identification of senescence-related biomarker for aortic dissection based on bioinformatics and machine learning algorithms
Source: Medicine (Baltimore). 2026 May 29;105(22):e48873. doi: 10.1097/MD.0000000000048873 (PMC13249447; doi:10.1097/MD.0000000000048873)
Supplement: Supplementary file 6 [file medi-105-e48873-s006.docx]

**Supplementary file 6 Table S5.** The results of GO and DO analysis.

| pvalue | p.adjust | qvalue | geneID | Count |  | ID | Description | GeneRatio | BgRatio | pvalue | p.adjust | qvalue | geneID | Count |
| --- | --- | --- | --- | --- | --- | --- | --- | --- | --- | --- | --- | --- | --- | --- |
| 1.95E-08 | 4.25E-05 | 3.56E-05 | CCL2/HMOX1/APOD/CCL20/VEGFA/CXCL14/FPR2/SELP/IL6/CCL7/SELE | 11 |  | DOID:9408 | acute myocardial infarction | 9/75 | 92/8007 | 1.58E-07 | 4.86E-05 | 2.83E-05 | PROM1/CCL2/PTX3/HMOX1/VEGFA/SELP/IL6/OLR1/ADIPOQ | 9 |
| 3.11E-07 | 0.000338 | 0.000283 | SLC11A1/MT1X/MT2A/MT1A/HMOX1/MT1G/SLC39A14/SCARA5 | 8 |  | DOID:865 | vasculitis | 10/75 | 122/8007 | 1.67E-07 | 4.86E-05 | 2.83E-05 | SLC11A1/CXCL5/SERPINA1/THBD/CCL2/HMOX1/VEGFA/VWF/IL6/SELE | 10 |
| 1.09E-06 | 0.000514 | 0.00043 | SLC11A1/MT1X/MT2A/MT1A/HMOX1/MT1G/SLC39A14/SCARA5 | 8 |  | DOID:3393 | coronary artery disease | 15/75 | 348/8007 | 5.83E-07 | 9.18E-05 | 5.34E-05 | ANGPTL4/PROM1/THBD/CCL2/PTX3/HMOX1/SPP1/VEGFA/SELP/VWF/IL6/OLR1/ADRA2B/ADIPOQ/SELE | 15 |
| 1.18E-06 | 0.000514 | 0.00043 | MT1X/MT2A/MT1A/MT1G | 4 |  | DOID:552 | pneumonia | 9/75 | 108/8007 | 6.32E-07 | 9.18E-05 | 5.34E-05 | TNC/SERPINA1/THBD/CCL2/HMOX1/TREM1/SPP1/VEGFA/IL6 | 9 |
| 1.18E-06 | 0.000514 | 0.00043 | MT1X/MT2A/MT1A/MT1G | 4 |  | DOID:11335 | sarcoidosis | 8/75 | 84/8007 | 9.98E-07 | 9.94E-05 | 5.78E-05 | SLC11A1/TNC/CXCL5/CCL2/SPP1/VEGFA/IL6/SELE | 8 |
| 2.04E-06 | 0.000573 | 0.00048 | MT1X/MT2A/MT1A/MT1G | 4 |  | DOID:4989 | pancreatitis | 9/75 | 117/8007 | 1.25E-06 | 9.94E-05 | 5.78E-05 | SERPINA1/CCL2/TREM1/SPP1/VEGFA/SELP/IL6/ADIPOQ/SELE | 9 |
| 2.11E-06 | 0.000573 | 0.00048 | MT1X/MT2A/MT1A/MT1G/SLC39A14 | 5 |  | DOID:2916 | hypersensitivity reaction type IV disease | 8/75 | 88/8007 | 1.43E-06 | 9.94E-05 | 5.78E-05 | SLC11A1/TNC/CXCL5/CCL2/SPP1/VEGFA/IL6/SELE | 8 |
| 2.11E-06 | 0.000573 | 0.00048 | MT1X/MT2A/MT1A/HMOX1/MT1G | 5 |  | DOID:10591 | pre-eclampsia | 13/75 | 278/8007 | 1.48E-06 | 9.94E-05 | 5.78E-05 | THBD/CCL2/PTX3/LIF/HMOX1/VEGFA/NOSTRIN/SELP/VWF/IL6/OLR1/ADIPOQ/SELE | 13 |
| 2.61E-06 | 0.000596 | 0.000499 | MT1X/MT2A/MT1A/MT1G | 4 |  | DOID:5844 | myocardial infarction | 13/75 | 279/8007 | 1.54E-06 | 9.94E-05 | 5.78E-05 | PROM1/THBD/CCL2/PTX3/HMOX1/VEGFA/SELP/VWF/IL6/OLR1/ADRA2B/ADIPOQ/SELE | 13 |
| 2.74E-06 | 0.000596 | 0.000499 | MT1X/MT2A/MT1A/MT1G/SLC39A14 | 5 |  | DOID:1936 | atherosclerosis | 14/75 | 344/8007 | 2.90E-06 | 0.000158 | 9.21E-05 | TNC/THBD/CCL2/PTX3/MTTP/HMOX1/SPP1/VEGFA/SELP/VWF/IL6/OLR1/ADIPOQ/SELE | 14 |
| 7.37E-06 | 0.001459 | 0.001221 | SERPINA1/SERPINA3/VEGFA/MMRN1/SELP/VWF/CFD | 7 |  | DOID:2348 | arteriosclerotic cardiovascular disease | 14/75 | 345/8007 | 3.00E-06 | 0.000158 | 9.21E-05 | TNC/THBD/CCL2/PTX3/MTTP/HMOX1/SPP1/VEGFA/SELP/VWF/IL6/OLR1/ADIPOQ/SELE | 14 |
| 8.84E-06 | 0.001588 | 0.00133 | MT1X/MT2A/MT1A/MT1G | 4 |  | DOID:2349 | arteriosclerosis | 14/75 | 356/8007 | 4.33E-06 | 0.000207 | 0.00012 | TNC/THBD/CCL2/PTX3/MTTP/HMOX1/SPP1/VEGFA/SELP/VWF/IL6/OLR1/ADIPOQ/SELE | 14 |
| 9.48E-06 | 0.001588 | 0.00133 | CCL20/VEGFA/CXCL14/FPR2/SELP/IL6/CCL7 | 7 |  | DOID:2320 | obstructive lung disease | 13/75 | 308/8007 | 4.62E-06 | 0.000207 | 0.00012 | SLC11A1/TNC/SERPINA1/THBD/CCL2/HMOX1/TREM1/IL1RL1/SPP1/VEGFA/VWF/IL6/ADIPOQ | 13 |
| 1.67E-05 | 0.002606 | 0.002181 | MT1X/MT2A/MT1A/MT1G | 4 |  | DOID:3083 | chronic obstructive pulmonary disease | 11/75 | 220/8007 | 5.48E-06 | 0.000227 | 0.000132 | SLC11A1/SERPINA1/CCL2/HMOX1/TREM1/IL1RL1/SPP1/VEGFA/VWF/IL6/ADIPOQ | 11 |
| 2.26E-05 | 0.003275 | 0.002742 | MT1X/MT2A/MT1A/HMOX1/MT1G | 5 |  | DOID:0050700 | cardiomyopathy | 9/75 | 149/8007 | 9.27E-06 | 0.000359 | 0.000209 | TNC/CCL2/PTX3/ACTC1/SPP1/NEBL/VWF/IL6/ADIPOQ | 9 |
| 2.64E-05 | 0.00343 | 0.002872 | CCL2/APOD/CCL20/CXCL14/CCL7 | 5 |  | DOID:4138 | bile duct disease | 7/75 | 86/8007 | 1.42E-05 | 0.000468 | 0.000272 | SERPINA1/HMOX1/TREM1/SPP1/VEGFA/IL6/SELE | 7 |
| 2.68E-05 | 0.00343 | 0.002872 | CCL2/CXCL14/CCL7 | 3 |  | DOID:2462 | retinal vascular disease | 6/75 | 58/8007 | 1.52E-05 | 0.000468 | 0.000272 | TNC/CCL2/VEGFA/VWF/IL6/SELE | 6 |
| 2.95E-05 | 0.003575 | 0.002992 | SERPINA1/IL31RA/SERPINA3/TREM1/C2CD4A/IL6 | 6 |  | DOID:8947 | diabetic retinopathy | 6/75 | 58/8007 | 1.52E-05 | 0.000468 | 0.000272 | TNC/CCL2/VEGFA/VWF/IL6/SELE | 6 |
| 3.64E-05 | 0.004002 | 0.00335 | CCL2/APOD/CCL20/CXCL14/FPR2/CCL7 | 6 |  | DOID:9741 | biliary tract disease | 7/75 | 87/8007 | 1.53E-05 | 0.000468 | 0.000272 | SERPINA1/HMOX1/TREM1/SPP1/VEGFA/IL6/SELE | 7 |
| 3.83E-05 | 0.004002 | 0.00335 | CCL2/CCL20/FPR2/IL6/CCL7 | 5 |  | DOID:13207 | proliferative diabetic retinopathy | 5/75 | 35/8007 | 1.64E-05 | 0.000477 | 0.000278 | CCL2/VEGFA/VWF/IL6/SELE | 5 |
| 3.86E-05 | 0.004002 | 0.00335 | CXCL5/CCL2/CCL20/VEGFA/CXCL14/FPR2/IL6/CCL7 | 8 |  | DOID:13580 | cholestasis | 6/75 | 62/8007 | 2.24E-05 | 0.000619 | 0.00036 | SERPINA1/HMOX1/SPP1/VEGFA/IL6/SELE | 6 |
| 5.38E-05 | 0.005322 | 0.004455 | CCL2/VEGFA/CXCL14/FPR2/IL6/CCL7 | 6 |  | DOID:2986 | IgA glomerulonephritis | 4/75 | 19/8007 | 2.47E-05 | 0.000653 | 0.00038 | CCL2/PTX3/IL6/SELE | 4 |
| 8.62E-05 | 0.008167 | 0.006836 | MT1X/MT2A/MT1A/MT1G | 4 |  | DOID:9455 | lipid storage disease | 8/75 | 130/8007 | 2.64E-05 | 0.000658 | 0.000382 | SERPINA1/MTTP/HMOX1/GCKR/VEGFA/IL6/ADIPOQ/SELE | 8 |
| 9.33E-05 | 0.008469 | 0.007089 | CCL2/APOD/CCL20/CXCL14/FPR2/IL6/CCL7 | 7 |  | DOID:26 | pancreas disease | 9/75 | 171/8007 | 2.80E-05 | 0.000658 | 0.000382 | SERPINA1/CCL2/TREM1/SPP1/VEGFA/SELP/IL6/ADIPOQ/SELE | 9 |
| 0.000105 | 0.00919 | 0.007693 | SLC11A1/SIGLEC9/SERPINA1/HK3/SERPINA3/PTX3/PTPRB/FPR2/IL6/OLR1/CFD | 11 |  | DOID:9352 | type 2 diabetes mellitus | 10/75 | 215/8007 | 2.85E-05 | 0.000658 | 0.000382 | SLC11A1/CCL2/PTX3/MTTP/SELP/VWF/AQP7/IL6/ADIPOQ/SELE | 10 |
| 0.000135 | 0.011217 | 0.00939 | IL31RA/LIF/VEGFA/ADIPOQ | 4 |  | DOID:9970 | obesity | 12/75 | 313/8007 | 2.94E-05 | 0.000658 | 0.000382 | CXCL5/CCL2/MTTP/GCKR/SPP1/PCSK1/SELP/AQP7/IL6/OLR1/ADIPOQ/SELE | 12 |
| 0.000139 | 0.011217 | 0.00939 | HMOX1/IL1RL1/APOD/IL6/ADIPOQ | 5 |  | DOID:654 | overnutrition | 12/75 | 322/8007 | 3.89E-05 | 0.000808 | 0.00047 | CXCL5/CCL2/MTTP/GCKR/SPP1/PCSK1/SELP/AQP7/IL6/OLR1/ADIPOQ/SELE | 12 |
| 0.000147 | 0.011217 | 0.00939 | CCL2/NRXN1/GRIK3/CDH8/RELN | 5 |  | DOID:104 | bacterial infectious disease | 11/75 | 271/8007 | 3.89E-05 | 0.000808 | 0.00047 | SLC11A1/TNC/THBD/CCL2/PTX3/TREM1/CCL20/VEGFA/FPR2/VWF/IL6 | 11 |
| 0.000152 | 0.011217 | 0.00939 | SLC11A1/SLC39A14/SCARA5 | 3 |  | DOID:850 | lung disease | 15/75 | 499/8007 | 4.73E-05 | 0.000947 | 0.000551 | SLC11A1/TNC/CXCL5/SERPINA1/THBD/CCL2/PTX3/HMOX1/TREM1/IL1RL1/SPP1/VEGFA/VWF/IL6/ADIPOQ | 15 |
| 0.000155 | 0.011217 | 0.00939 | VEGFA/CXCL14/FPR2/IL6/CCL7 | 5 |  | DOID:6000 | congestive heart failure | 10/75 | 229/8007 | 4.89E-05 | 0.000947 | 0.000551 | PROM1/HMOX1/PPP1R1A/IL1RL1/RAMP3/SPP1/VEGFA/VWF/IL6/ADIPOQ | 10 |
| 0.00018 | 0.012645 | 0.010585 | SLC11A1/CCL2/PTX3/FPR2/ADIPOQ | 5 |  | DOID:10871 | age related macular degeneration | 6/75 | 72/8007 | 5.28E-05 | 0.000959 | 0.000558 | PROM1/CCL2/VEGFA/SELP/CFD/SELE | 6 |
| 0.000189 | 0.012874 | 0.010776 | HMOX1/IL1RL1/APOD/IL6/ADIPOQ | 5 |  | DOID:2007 | degeneration of macula and posterior pole | 6/75 | 72/8007 | 5.28E-05 | 0.000959 | 0.000558 | PROM1/CCL2/VEGFA/SELP/CFD/SELE | 6 |
| 0.000218 | 0.014411 | 0.012063 | CXCL5/CCL2/CCL20/VEGFA/FPR2/IL6/CCL7 | 7 |  | DOID:1575 | rheumatic disease | 9/75 | 188/8007 | 5.91E-05 | 0.000973 | 0.000566 | SLC11A1/CCL2/PTX3/TREM1/SPP1/VEGFA/SELP/IL6/CCL7 | 9 |
| 0.00023 | 0.014721 | 0.012322 | SERPINA1/ANGPTL4/SERPINA3/PTX3/PPP1R1A/VEGFA/SLC39A14/PI16/SPOCD1/PI15 | 10 |  | DOID:418 | systemic scleroderma | 9/75 | 188/8007 | 5.91E-05 | 0.000973 | 0.000566 | SLC11A1/CCL2/PTX3/TREM1/SPP1/VEGFA/SELP/IL6/CCL7 | 9 |
| 0.000239 | 0.014873 | 0.01245 | SELP/IL6/SELE | 3 |  | DOID:419 | scleroderma | 9/75 | 188/8007 | 5.91E-05 | 0.000973 | 0.000566 | SLC11A1/CCL2/PTX3/TREM1/SPP1/VEGFA/SELP/IL6/CCL7 | 9 |
| 0.000258 | 0.015521 | 0.012992 | CCL2/VEGFA/CXCL14/FPR2/SEMA3G/IL6/CCL7 | 7 |  | DOID:4448 | macular degeneration | 6/75 | 74/8007 | 6.17E-05 | 0.000973 | 0.000566 | PROM1/CCL2/VEGFA/SELP/CFD/SELE | 6 |
| 0.000264 | 0.015521 | 0.012992 | SLC11A1/IL31RA/IL1RL1/FPR2/IL6 | 5 |  | DOID:374 | nutrition disease | 12/75 | 338/8007 | 6.24E-05 | 0.000973 | 0.000566 | CXCL5/CCL2/MTTP/GCKR/SPP1/PCSK1/SELP/AQP7/IL6/OLR1/ADIPOQ/SELE | 12 |
| 0.00028 | 0.015943 | 0.013346 | CCL2/LIF/MARCO/CCL20/RAMP3/FPR2/CCL7/ADIPOQ | 8 |  | DOID:10952 | nephritis | 8/75 | 147/8007 | 6.36E-05 | 0.000973 | 0.000566 | MT2A/MT1A/CCL2/PTX3/SPP1/VEGFA/IL6/SELE | 8 |
| 0.000285 | 0.015943 | 0.013346 | MT1X/MT2A/MT1A/MT1G | 4 |  | DOID:0050338 | primary bacterial infectious disease | 10/75 | 238/8007 | 6.77E-05 | 0.001009 | 0.000587 | SLC11A1/TNC/THBD/CCL2/PTX3/TREM1/CCL20/VEGFA/VWF/IL6 | 10 |
| 0.000302 | 0.01647 | 0.013786 | ADRA1D/GRIK3/MARCO/RAMP3/FPR2/ADCY4/ADRA2B | 7 |  | DOID:3211 | lysosomal storage disease | 8/75 | 150/8007 | 7.34E-05 | 0.001067 | 0.00062 | SERPINA1/MTTP/HMOX1/GCKR/VEGFA/IL6/ADIPOQ/SELE | 8 |
| 0.000313 | 0.01661 | 0.013904 | CXCL5/CCL2/CCL20/VEGFA/CXCL14/FPR2/IL6/CCL7 | 8 |  | DOID:6432 | pulmonary hypertension | 6/75 | 77/8007 | 7.72E-05 | 0.001095 | 0.000637 | TNC/HMOX1/VEGFA/SELP/VWF/IL6 | 6 |
| 0.000345 | 0.017624 | 0.014753 | SLC11A1/MT1X/MT2A/MT1A/ADRA1D/RAMP3/MT1G/SLC39A14/FPR2/CCL7 | 10 |  | DOID:3978 | extrinsic cardiomyopathy | 4/75 | 26/8007 | 9.08E-05 | 0.001227 | 0.000714 | CCL2/PTX3/SPP1/IL6 | 4 |
| 0.000348 | 0.017624 | 0.014753 | HMOX1/IL1RL1/IL6/ADIPOQ | 4 |  | DOID:820 | myocarditis | 4/75 | 26/8007 | 9.08E-05 | 0.001227 | 0.000714 | CCL2/PTX3/SPP1/IL6 | 4 |
| 0.000375 | 0.018554 | 0.015531 | SLC11A1/SIGLEC9/SERPINA1/HK3/SERPINA3/PTX3/PTPRB/FPR2/OLR1/CFD | 10 |  | DOID:3963 | thyroid carcinoma | 9/75 | 200/8007 | 9.54E-05 | 0.00125 | 0.000727 | TNC/SERPINA1/CCL2/LIF/SPP1/MT1G/VEGFA/CXCL14/CA9 | 9 |
| 0.000393 | 0.018799 | 0.015736 | SLC11A1/SIGLEC9/SERPINA1/HK3/SERPINA3/PTX3/PTPRB/FPR2/OLR1/CFD | 10 |  | DOID:1074 | kidney failure | 8/75 | 156/8007 | 9.68E-05 | 0.00125 | 0.000727 | CCL2/PTX3/ADRA1D/SPP1/VEGFA/IL6/ADIPOQ/SELE | 8 |
| 0.000397 | 0.018799 | 0.015736 | CCL2/CXCL14/CCL7 | 3 |  | DOID:854 | collagen disease | 9/75 | 201/8007 | 9.91E-05 | 0.001252 | 0.000728 | SLC11A1/CCL2/PTX3/TREM1/SPP1/VEGFA/SELP/IL6/CCL7 | 9 |
| 0.00042 | 0.019018 | 0.01592 | CCL20/CXCL14/FPR2/CCL7 | 4 |  | DOID:326 | ischemia | 9/75 | 204/8007 | 0.000111 | 0.001373 | 0.000798 | ANGPTL4/SERPINA3/THBD/CCL2/LIF/VEGFA/SELP/ADRA2B/SELE | 9 |
| 0.000421 | 0.019018 | 0.01592 | CCL2/LIF/MARCO/CCL20/RAMP3/FPR2/CCL7/ADIPOQ | 8 |  | DOID:13241 | Behcet's disease | 6/75 | 86/8007 | 0.000143 | 0.001735 | 0.001009 | SLC11A1/THBD/CCL2/HMOX1/VEGFA/VWF | 6 |
| 0.000428 | 0.019018 | 0.01592 | SOX10/SPP1/VEGFA/DIO3/SEMA3G/IL6 | 6 |  | DOID:9588 | encephalitis | 5/75 | 55/8007 | 0.000152 | 0.001792 | 0.001042 | PTX3/LGI1/APOD/VEGFA/IL6 | 5 |
| 0.000442 | 0.019034 | 0.015933 | MT1X/MT2A/MT1A/SPP1/MT1G/PI16/SEMA3G | 7 |  | DOID:1781 | thyroid cancer | 9/75 | 213/8007 | 0.000154 | 0.001792 | 0.001042 | TNC/SERPINA1/CCL2/LIF/SPP1/MT1G/VEGFA/CXCL14/CA9 | 9 |
| 0.000446 | 0.019034 | 0.015933 | CCL2/CCL20/CXCL14/CCL7 | 4 |  | DOID:4451 | renal carcinoma | 12/75 | 380/8007 | 0.00019 | 0.002167 | 0.00126 | TNC/ANGPTL4/CCL2/ST14/SPP1/MT1G/VEGFA/CDH8/ESM1/CA9/IL6/ADIPOQ | 12 |
| 0.000468 | 0.01919 | 0.016063 | SLC11A1/MT1X/MT2A/MT1A/ADRA1D/RAMP3/MT1G/SLC39A14/FPR2/CCL7 | 10 |  | DOID:13375 | temporal arteritis | 4/75 | 32/8007 | 0.000209 | 0.002271 | 0.001321 | CXCL5/VEGFA/IL6/SELE | 4 |
| 0.000468 | 0.01919 | 0.016063 | THBD/LIF/FOSL1/SPP1/VEGFA/ADRA2B | 6 |  | DOID:525 | central nervous system vasculitis | 4/75 | 32/8007 | 0.000209 | 0.002271 | 0.001321 | CXCL5/VEGFA/IL6/SELE | 4 |
| 0.000476 | 0.01919 | 0.016063 | SLC11A1/SIGLEC9/SERPINA1/HK3/SERPINA3/PTX3/PTPRB/FPR2/OLR1/CFD | 10 |  | DOID:13608 | biliary atresia | 3/75 | 13/8007 | 0.000211 | 0.002271 | 0.001321 | SPP1/VEGFA/SELE | 3 |
| 0.000516 | 0.020446 | 0.017115 | CCL2/APOD/CCL20/CXCL14/CCL7 | 5 |  | DOID:11714 | gestational diabetes | 5/75 | 60/8007 | 0.00023 | 0.002434 | 0.001415 | SERPINA1/CCL2/APOD/IL6/ADIPOQ | 5 |
| 0.000531 | 0.020646 | 0.017283 | SLC11A1/CCL2/PTX3/FPR2 | 4 |  | DOID:9452 | fatty liver disease | 6/75 | 95/8007 | 0.000248 | 0.002572 | 0.001496 | SERPINA1/MTTP/HMOX1/GCKR/VEGFA/ADIPOQ | 6 |
| 0.000583 | 0.022282 | 0.018651 | NRXN1/LIF/CLSTN2/SOX10/SPP1/VEGFA/SEMA3G/IL6/RELN | 9 |  | DOID:0060036 | intrinsic cardiomyopathy | 7/75 | 135/8007 | 0.000253 | 0.002582 | 0.001502 | TNC/CCL2/ACTC1/NEBL/VWF/IL6/ADIPOQ | 7 |
| 0.000594 | 0.022293 | 0.018661 | CCL2/NRXN1/GRIK3/RELN | 4 |  | DOID:2913 | acute pancreatitis | 3/75 | 14/8007 | 0.000267 | 0.002673 | 0.001554 | CCL2/SELP/SELE | 3 |
| 0.00061 | 0.022521 | 0.018851 | CCL2/NRXN1/RELN | 3 |  | DOID:423 | myopathy | 12/75 | 398/8007 | 0.000292 | 0.002828 | 0.001645 | TNC/PROM1/CCL2/PTX3/ACTC1/SPP1/VEGFA/NEBL/SGCG/VWF/IL6/ADIPOQ | 12 |
| 0.000627 | 0.022759 | 0.019051 | SLC11A1/HMOX1/SLC39A14/SCARA5 | 4 |  | DOID:66 | muscle tissue disease | 12/75 | 398/8007 | 0.000292 | 0.002828 | 0.001645 | TNC/PROM1/CCL2/PTX3/ACTC1/SPP1/VEGFA/NEBL/SGCG/VWF/IL6/ADIPOQ | 12 |
| 0.000674 | 0.024059 | 0.02014 | NRXN1/LIF/CLSTN2/SOX10/VEGFA/IL6/RELN | 7 |  | DOID:3388 | periodontal disease | 7/75 | 139/8007 | 0.000303 | 0.002887 | 0.001679 | PROM1/CCL2/CCL20/VEGFA/IL6/CCL7/SELE | 7 |
| 0.000698 | 0.024504 | 0.020511 | PLP1/LIF/FPR2/IL6 | 4 |  | DOID:4450 | renal cell carcinoma | 11/75 | 342/8007 | 0.000309 | 0.002897 | 0.001685 | TNC/ANGPTL4/CCL2/ST14/SPP1/MT1G/VEGFA/CDH8/CA9/IL6/ADIPOQ | 11 |
| 0.000738 | 0.025527 | 0.021368 | IL31RA/LIF/VEGFA/IL6/ADIPOQ/RELN | 6 |  | DOID:8398 | osteoarthritis | 8/75 | 187/8007 | 0.000338 | 0.003113 | 0.00181 | HMOX1/CCL20/SPP1/VEGFA/FPR2/DIO3/IL6/ADIPOQ | 8 |
| 0.000839 | 0.028547 | 0.023896 | CLSTN2/MPZL2/CDH8/SELP/PCDH17/ADIPOQ/SELE | 7 |  | DOID:750 | peptic ulcer disease | 5/75 | 66/8007 | 0.000361 | 0.003227 | 0.001877 | CCL2/TREM1/VEGFA/IL6/SELE | 5 |
| 0.000898 | 0.030098 | 0.025194 | ACTC1/VEGFA/NEBL/PI16 | 4 |  | DOID:2723 | dermatitis | 8/75 | 189/8007 | 0.000363 | 0.003227 | 0.001877 | LIF/HMOX1/IL1RL1/VEGFA/FPR2/SELP/IL6/SELE | 8 |
| 0.00095 | 0.03134 | 0.026234 | LIF/ST14/FOSL1/SPP1/RSPO3 | 5 |  | DOID:0080000 | muscular disease | 12/75 | 408/8007 | 0.000367 | 0.003227 | 0.001877 | TNC/PROM1/CCL2/PTX3/ACTC1/SPP1/VEGFA/NEBL/SGCG/VWF/IL6/ADIPOQ | 12 |
| 0.000985 | 0.032007 | 0.026792 | THBD/LIF/FOSL1/SPP1/VEGFA/ADRA2B | 6 |  | DOID:783 | end stage renal failure | 4/75 | 38/8007 | 0.000412 | 0.003515 | 0.002044 | CCL2/ADRA1D/IL6/SELE | 4 |
| 0.001034 | 0.033055 | 0.02767 | PLP1/CCL2/LIF/SOX10/FPR2/IL6/RELN | 7 |  | DOID:576 | proteinuria | 5/75 | 68/8007 | 0.000415 | 0.003515 | 0.002044 | CCL2/PTX3/HMOX1/VEGFA/ADIPOQ | 5 |
| 0.001047 | 0.033055 | 0.02767 | IL31RA/MT1G/VEGFA | 3 |  | DOID:1115 | sarcoma | 8/75 | 193/8007 | 0.000417 | 0.003515 | 0.002044 | ANGPTL4/PROM1/PTX3/CCL20/SPP1/VEGFA/CA9/IL6 | 8 |
| 0.001082 | 0.033646 | 0.028164 | VEGFA/CXCL14/FPR2/IL6/CCL7 | 5 |  | DOID:5679 | retinal disease | 11/75 | 358/8007 | 0.000457 | 0.003791 | 0.002205 | TNC/PROM1/CCL2/VEGFA/SELP/VWF/CA4/IL6/ADIPOQ/CFD/SELE | 11 |
| 0.001135 | 0.033646 | 0.028164 | SELP/IL6/SELE | 3 |  | DOID:784 | chronic kidney failure | 5/75 | 70/8007 | 0.000475 | 0.003885 | 0.00226 | CCL2/PTX3/ADRA1D/IL6/SELE | 5 |
| 0.001135 | 0.033646 | 0.028164 | CCL20/CXCL14/CCL7 | 3 |  | DOID:5395 | functioning pituitary adenoma | 3/75 | 17/8007 | 0.000488 | 0.003941 | 0.002292 | LIF/PCSK1/IL6 | 3 |
| 0.001143 | 0.033646 | 0.028164 | PLP1/LIF/SOX10/FPR2/IL6/RELN | 6 |  | DOID:3969 | papillary thyroid carcinoma | 6/75 | 108/8007 | 0.000496 | 0.003947 | 0.002295 | SERPINA1/CCL2/SPP1/MT1G/VEGFA/CXCL14 | 6 |
| 0.001143 | 0.033646 | 0.028164 | CCL2/MARCO/CCL20/RAMP3/FPR2/CCL7 | 6 |  | DOID:3326 | purpura | 5/75 | 71/8007 | 0.000507 | 0.003981 | 0.002315 | HMOX1/VEGFA/SELP/VWF/IL6 | 5 |
| 0.001189 | 0.034529 | 0.028904 | ACTC1/VEGFA/NEBL/PI16 | 4 |  | DOID:4248 | coronary stenosis | 3/75 | 18/8007 | 0.000582 | 0.00451 | 0.002623 | CCL2/SPP1/IL6 | 3 |
| 0.001267 | 0.036302 | 0.030388 | CCL2/NRXN1/LGI1/CLSTN2/RELN | 5 |  | DOID:3069 | astrocytoma | 6/75 | 112/8007 | 0.000602 | 0.004581 | 0.002664 | TNC/PROM1/CCL2/SOX10/CA9/IL6 | 6 |
| 0.001299 | 0.036745 | 0.030758 | GRIK3/MARCO/FPR2/ADCY4 | 4 |  | DOID:11077 | brucellosis | 4/75 | 42/8007 | 0.000607 | 0.004581 | 0.002664 | SLC11A1/THBD/VEGFA/IL6 | 4 |
| 0.001357 | 0.037882 | 0.03171 | SLC11A1/HMOX1/SLC39A14/SCARA5 | 4 |  | DOID:263 | kidney cancer | 12/75 | 437/8007 | 0.00068 | 0.00505 | 0.002937 | TNC/ANGPTL4/CCL2/ST14/SPP1/MT1G/VEGFA/CDH8/ESM1/CA9/IL6/ADIPOQ | 12 |
| 0.001426 | 0.038819 | 0.032495 | ANGPTL4/GCKR/GPIHBP1 | 3 |  | DOID:2957 | pulmonary tuberculosis | 3/75 | 19/8007 | 0.000687 | 0.00505 | 0.002937 | SLC11A1/CCL2/PTX3 | 3 |
| 0.001426 | 0.038819 | 0.032495 | ANGPTL4/GCKR/GPIHBP1 | 3 |  | DOID:6713 | cerebrovascular disease | 6/75 | 116/8007 | 0.000725 | 0.005265 | 0.003062 | THBD/CCL2/SPP1/IL6/OLR1/ADIPOQ | 6 |
| 0.001477 | 0.039719 | 0.033248 | CXCL5/CCL2/CCL20/CCL7 | 4 |  | DOID:1091 | tooth disease | 7/75 | 162/8007 | 0.000763 | 0.00547 | 0.003181 | PROM1/CCL2/CCL20/VEGFA/IL6/CCL7/SELE | 7 |
| 0.001546 | 0.041072 | 0.034381 | LIF/SOX10/SPP1/VEGFA/DIO3/SEMA3G/IL6/ADIPOQ/RELN | 9 |  | DOID:799 | varicose veins | 3/75 | 20/8007 | 0.000802 | 0.005686 | 0.003307 | SERPINA1/HMOX1/VEGFA | 3 |
| 0.001634 | 0.042112 | 0.035251 | RAMP3/ADIPOQ | 2 |  | DOID:824 | periodontitis | 6/75 | 120/8007 | 0.000867 | 0.006066 | 0.003528 | PROM1/CCL2/VEGFA/IL6/CCL7/SELE | 6 |
| 0.001634 | 0.042112 | 0.035251 | SPP1/DIO3 | 2 |  | DOID:655 | inherited metabolic disorder | 10/75 | 331/8007 | 0.000964 | 0.006665 | 0.003876 | SERPINA1/CCL2/MTTP/HMOX1/GCKR/VEGFA/VWF/IL6/ADIPOQ/SELE | 10 |
| 0.001643 | 0.042112 | 0.035251 | PLP1/FPR2/IL6 | 3 |  | DOID:5082 | liver cirrhosis | 8/75 | 220/8007 | 0.00099 | 0.006769 | 0.003937 | SERPINA1/SERPINA3/THBD/VEGFA/SELP/IL6/ADIPOQ/RELN | 8 |
| 0.00176 | 0.044052 | 0.036875 | PROM1/VEGFA/DIO3 | 3 |  | DOID:3082 | interstitial lung disease | 7/75 | 170/8007 | 0.001013 | 0.006777 | 0.003941 | CXCL5/SERPINA1/CCL2/HMOX1/TREM1/VEGFA/IL6 | 7 |
| 0.00176 | 0.044052 | 0.036875 | CCL2/ADRA2B/RELN | 3 |  | DOID:16 | integumentary system disease | 11/75 | 394/8007 | 0.001015 | 0.006777 | 0.003941 | CCL2/LIF/ST14/HMOX1/IL1RL1/VEGFA/FPR2/SELP/IL6/ADIPOQ/SELE | 11 |
| 0.001855 | 0.045262 | 0.037888 | THBD/SELP/VWF/IL6/ADRA2B | 5 |  | DOID:11123 | Henoch-Schoenlein purpura | 3/75 | 22/8007 | 0.00107 | 0.006905 | 0.004016 | HMOX1/VEGFA/IL6 | 3 |
| 0.001883 | 0.045262 | 0.037888 | ANGPTL4/CCL2/HMOX1/IL6 | 4 |  | DOID:1557 | hypersensitivity reaction type III disease | 3/75 | 22/8007 | 0.00107 | 0.006905 | 0.004016 | HMOX1/VEGFA/IL6 | 3 |
| 0.001919 | 0.045262 | 0.037888 | TNC/PTX3/LIF/ST14/FOSL1/SPP1/VEGFA/RSPO3 | 8 |  | DOID:9809 | hypersensitivity vasculitis | 3/75 | 22/8007 | 0.00107 | 0.006905 | 0.004016 | HMOX1/VEGFA/IL6 | 3 |
| 0.001919 | 0.045262 | 0.037888 | CCL2/NRXN1/GRIK3/LGI1/CLSTN2/PCDH17/ADIPOQ/RELN | 8 |  | DOID:557 | kidney disease | 12/75 | 461/8007 | 0.001088 | 0.006945 | 0.004039 | MT2A/MT1A/CCL2/PTX3/HMOX1/ADRA1D/IL1RL1/SPP1/VEGFA/IL6/ADIPOQ/SELE | 12 |
| 0.001949 | 0.045262 | 0.037888 | CCL2/NRXN1/GRIK3/LGI1/CLSTN2/PCDH17/ADIPOQ/RELN | 8 |  | DOID:974 | upper respiratory tract disease | 6/75 | 126/8007 | 0.001118 | 0.007059 | 0.004106 | HMOX1/VEGFA/SELP/IL6/ADIPOQ/CFD | 6 |
| 0.001953 | 0.045262 | 0.037888 | APOD/ADIPOQ | 2 |  | DOID:11723 | Duchenne muscular dystrophy | 3/75 | 23/8007 | 0.001222 | 0.007633 | 0.004439 | PROM1/SPP1/SGCG | 3 |
| 0.001953 | 0.045262 | 0.037888 | ST14/RSPO3 | 2 |  | DOID:4079 | heart valve disease | 4/75 | 51/8007 | 0.001272 | 0.007861 | 0.004572 | SPP1/VEGFA/SELP/VWF | 4 |
| 0.002009 | 0.04607 | 0.038564 | TNC/PTX3/LIF/ST14/FOSL1/SPP1/VEGFA/RSPO3 | 8 |  | DOID:289 | endometriosis | 5/75 | 88/8007 | 0.001349 | 0.008248 | 0.004797 | TNC/CXCL5/CCL2/LIF/IL6 | 5 |
| 0.002113 | 0.047046 | 0.039382 | CXCL5/CCL2/CCL20/CCL7 | 4 |  | DOID:2452 | thrombophilia | 3/75 | 24/8007 | 0.001387 | 0.008394 | 0.004882 | THBD/VEGFA/VWF | 3 |
| 0.002113 | 0.047046 | 0.039382 | CXCL5/CCL2/CCL20/CCL7 | 4 |  | DOID:1883 | hepatitis C | 8/75 | 233/8007 | 0.001433 | 0.008582 | 0.004991 | SERPINA1/CCL2/MTTP/HMOX1/SPP1/VEGFA/IL6/ADIPOQ | 8 |
| 0.002138 | 0.047046 | 0.039382 | CCL2/HMOX1/APOD | 3 |  | DOID:37 | skin disease | 10/75 | 350/8007 | 0.001471 | 0.008721 | 0.005072 | CCL2/LIF/HMOX1/IL1RL1/VEGFA/FPR2/SELP/IL6/ADIPOQ/SELE | 10 |
| 0.002138 | 0.047046 | 0.039382 | SERPINA1/SERPINA3/IL6 | 3 |  | DOID:18 | urinary system disease | 12/75 | 478/8007 | 0.001486 | 0.008723 | 0.005073 | MT2A/MT1A/CCL2/PTX3/HMOX1/ADRA1D/IL1RL1/SPP1/VEGFA/IL6/ADIPOQ/SELE | 12 |
| 0.0023 | 0.04946 | 0.041402 | PROM1/LIF | 2 |  | DOID:2018 | hyperinsulinism | 4/75 | 54/8007 | 0.001576 | 0.009156 | 0.005325 | CCL2/IL6/ADIPOQ/SELE | 4 |
| 0.0023 | 0.04946 | 0.041402 | SELP/SELE | 2 |  | DOID:11162 | respiratory failure | 4/75 | 55/8007 | 0.001688 | 0.009708 | 0.005646 | TNC/PTX3/VEGFA/IL6 | 4 |
| 0.002316 | 0.04946 | 0.041402 | SLC11A1/CXCL5/THBD/CCL2/SELP/IL6/SELE | 7 |  | DOID:12930 | dilated cardiomyopathy | 5/75 | 93/8007 | 0.001726 | 0.009829 | 0.005716 | TNC/ACTC1/NEBL/IL6/ADIPOQ | 5 |
| 0.002417 | 0.05111 | 0.042783 | PROM1/LIF/ADIPOQ | 3 |  | DOID:1485 | cystic fibrosis | 6/75 | 138/8007 | 0.001785 | 0.01007 | 0.005857 | SERPINA1/PTX3/HMOX1/TREM1/MARCO/IL6 | 6 |
| 0.002448 | 0.051261 | 0.04291 | MTTP/HMOX1/GPIHBP1/ADIPOQ | 4 |  | DOID:403 | mouth disease | 7/75 | 188/8007 | 0.001815 | 0.01014 | 0.005897 | PROM1/CCL2/CCL20/VEGFA/IL6/CCL7/SELE | 7 |
| 0.002628 | 0.054517 | 0.045635 | CXCL5/CCL2/CCL20/CCL7 | 4 |  | DOID:2218 | blood platelet disease | 5/75 | 96/8007 | 0.001986 | 0.010989 | 0.006391 | THBD/VEGFA/SELP/VWF/IL6 | 5 |
| 0.002674 | 0.054941 | 0.04599 | NRXN1/RELN | 2 |  | DOID:3829 | pituitary adenoma | 4/75 | 58/8007 | 0.002055 | 0.011266 | 0.006552 | LIF/VEGFA/PCSK1/IL6 | 4 |
| 0.002717 | 0.055306 | 0.046296 | LIF/SPP1/VEGFA | 3 |  | DOID:229 | female reproductive system disease | 7/75 | 193/8007 | 0.002108 | 0.011445 | 0.006656 | TNC/CXCL5/CCL2/LIF/VEGFA/IL6/SELE | 7 |
| 0.002827 | 0.057004 | 0.047716 | TNC/ST14/SOX10/VEGFA/RSPO3 | 5 |  | DOID:870 | neuropathy | 7/75 | 194/8007 | 0.002171 | 0.01146 | 0.006665 | PLP1/THBD/FRMD7/VEGFA/VWF/IL6/SELE | 7 |
| 0.002916 | 0.058221 | 0.048735 | PROM1/LIF/VEGFA/ADIPOQ | 4 |  | DOID:3996 | urinary system cancer | 12/75 | 500/8007 | 0.002176 | 0.01146 | 0.006665 | TNC/ANGPTL4/CCL2/ST14/SPP1/MT1G/VEGFA/CDH8/ESM1/CA9/IL6/ADIPOQ | 12 |
| 0.003016 | 0.058221 | 0.048735 | LIF/RAMP3/VEGFA/IL6 | 4 |  | DOID:1428 | endocrine pancreas disease | 4/75 | 59/8007 | 0.002189 | 0.01146 | 0.006665 | CCL2/IL6/ADIPOQ/SELE | 4 |
| 0.003023 | 0.058221 | 0.048735 | SERPINA1/THBD/MMRN1/SELP/VWF/IL6/ADRA2B | 7 |  | DOID:4535 | hypotrichosis | 4/75 | 59/8007 | 0.002189 | 0.01146 | 0.006665 | CCL2/LIF/ST14/IL6 | 4 |
| 0.003039 | 0.058221 | 0.048735 | TNC/APOD/SPP1 | 3 |  | DOID:0060089 | endocrine organ benign neoplasm | 5/75 | 99/8007 | 0.002274 | 0.011691 | 0.006799 | LIF/VEGFA/PCSK1/IL6/ADIPOQ | 5 |
| 0.003074 | 0.058221 | 0.048735 | SLC11A1/SLC39A14 | 2 |  | DOID:4481 | allergic rhinitis | 5/75 | 99/8007 | 0.002274 | 0.011691 | 0.006799 | HMOX1/VEGFA/IL6/ADIPOQ/CFD | 5 |
| 0.003074 | 0.058221 | 0.048735 | PROM1/LIF | 2 |  | DOID:1712 | aortic valve stenosis | 3/75 | 29/8007 | 0.002421 | 0.012326 | 0.007168 | SPP1/VEGFA/VWF | 3 |
| 0.003074 | 0.058221 | 0.048735 | CCL2/APOD | 2 |  | DOID:2163 | nasal cavity disease | 5/75 | 101/8007 | 0.002482 | 0.012326 | 0.007168 | HMOX1/VEGFA/IL6/ADIPOQ/CFD | 5 |
| 0.003224 | 0.06001 | 0.050233 | SLC11A1/CXCL5/THBD/CCL2/SELP/IL6/SELE | 7 |  | DOID:2825 | nose disease | 5/75 | 101/8007 | 0.002482 | 0.012326 | 0.007168 | HMOX1/VEGFA/IL6/ADIPOQ/CFD | 5 |
| 0.003224 | 0.06001 | 0.050233 | SERPINA1/THBD/MMRN1/SELP/VWF/IL6/ADRA2B | 7 |  | DOID:4483 | rhinitis | 5/75 | 101/8007 | 0.002482 | 0.012326 | 0.007168 | HMOX1/VEGFA/IL6/ADIPOQ/CFD | 5 |
| 0.003275 | 0.060454 | 0.050605 | SERPINA1/THBD/MMRN1/SELP/VWF/IL6/ADRA2B | 7 |  | DOID:3454 | brain infarction | 4/75 | 62/8007 | 0.002628 | 0.012937 | 0.007524 | THBD/CCL2/IL6/ADIPOQ | 4 |
| 0.003328 | 0.060902 | 0.05098 | LIF/SOX10/SPP1/VEGFA/SEMA3G/IL6/RELN | 7 |  | DOID:3947 | adrenal gland hyperfunction | 3/75 | 30/8007 | 0.002672 | 0.013047 | 0.007588 | SELP/VWF/IL6 | 3 |
| 0.003384 | 0.061413 | 0.051408 | SELP/IL6/SELE | 3 |  | DOID:9884 | muscular dystrophy | 5/75 | 103/8007 | 0.002704 | 0.01306 | 0.007595 | PROM1/SPP1/VEGFA/SGCG/VWF | 5 |
| 0.003471 | 0.06247 | 0.052292 | IL31RA/LIF/VEGFA/IL6/ADIPOQ/RELN | 6 |  | DOID:1307 | dementia | 6/75 | 150/8007 | 0.00272 | 0.01306 | 0.007595 | SERPINA1/SERPINA3/SPP1/VEGFA/IL6/RELN | 6 |
| 0.003501 | 0.062493 | 0.052312 | SIGLEC10/HMOX1 | 2 |  | DOID:2213 | hemorrhagic disease | 6/75 | 152/8007 | 0.002906 | 0.013838 | 0.008048 | THBD/HMOX1/VEGFA/SELP/VWF/IL6 | 6 |
| 0.003554 | 0.062934 | 0.052681 | PLP1/SOX10/FPR2/IL6 | 4 |  | DOID:9744 | type 1 diabetes mellitus | 3/75 | 31/8007 | 0.002939 | 0.013882 | 0.008073 | CCL2/VWF/IL6 | 3 |
| 0.003786 | 0.066507 | 0.055671 | ACTC1/VEGFA/NEBL/PI16 | 4 |  | DOID:1176 | bronchial disease | 6/75 | 153/8007 | 0.003002 | 0.014067 | 0.008181 | SLC11A1/TNC/SERPINA1/THBD/IL1RL1/VEGFA | 6 |
| 0.003853 | 0.067133 | 0.056196 | TNC/ST14/SOX10/VEGFA/RSPO3 | 5 |  | DOID:421 | hair disease | 4/75 | 65/8007 | 0.003122 | 0.01441 | 0.008381 | CCL2/LIF/ST14/IL6 | 4 |
| 0.003944 | 0.067261 | 0.056303 | APOD/VEGFA/ADIPOQ | 3 |  | DOID:3070 | malignant glioma | 7/75 | 207/8007 | 0.003125 | 0.01441 | 0.008381 | TNC/PROM1/CCL2/CCL20/SOX10/CA9/IL6 | 7 |
| 0.003945 | 0.067261 | 0.056303 | ACTC1/HMOX1/APOD/FOSL1/SOX10/CA9/ADIPOQ | 7 |  | DOID:399 | tuberculosis | 6/75 | 156/8007 | 0.003306 | 0.015125 | 0.008796 | SLC11A1/TNC/CCL2/PTX3/CCL20/IL6 | 6 |
| 0.003953 | 0.067261 | 0.056303 | ACTC1/NEBL | 2 |  | DOID:2228 | thrombocytosis | 3/75 | 33/8007 | 0.00352 | 0.015976 | 0.009291 | VEGFA/SELP/IL6 | 3 |
| 0.004029 | 0.067546 | 0.056542 | ANGPTL4/CCL2/HMOX1/IL6 | 4 |  | DOID:2738 | pseudoxanthoma elasticum | 2/75 | 10/8007 | 0.003711 | 0.016632 | 0.009673 | SELP/SELE | 2 |
| 0.004032 | 0.067546 | 0.056542 | CCL2/RAMP3/GREM2/ADRA2B/RELN | 5 |  | DOID:10223 | dermatomyositis | 3/75 | 34/8007 | 0.003835 | 0.016632 | 0.009673 | CCL2/VEGFA/IL6 | 3 |
| 0.004123 | 0.067833 | 0.056781 | MT1X/MT2A/MT1A/HMOX1/MT1G | 5 |  | DOID:11981 | morbid obesity | 3/75 | 34/8007 | 0.003835 | 0.016632 | 0.009673 | AQP7/ADIPOQ/SELE | 3 |
| 0.004142 | 0.067833 | 0.056781 | TNC/APOD/SPP1 | 3 |  | DOID:62 | aortic valve disease | 3/75 | 34/8007 | 0.003835 | 0.016632 | 0.009673 | SPP1/VEGFA/VWF | 3 |
| 0.004142 | 0.067833 | 0.056781 | PROM1/LIF/ADIPOQ | 3 |  | DOID:811 | lipodystrophy | 3/75 | 34/8007 | 0.003835 | 0.016632 | 0.009673 | CCL2/IL6/ADIPOQ | 3 |
| 0.004347 | 0.069429 | 0.058117 | IL31RA/IL1RL1/IL6 | 3 |  | DOID:28 | endocrine system disease | 10/75 | 399/8007 | 0.003836 | 0.016632 | 0.009673 | SERPINA1/CCL2/TREM1/SPP1/VEGFA/SELP/VWF/IL6/ADIPOQ/SELE | 10 |
| 0.004381 | 0.069429 | 0.058117 | SIGLEC10/IL1RL1/FPR2/C2CD4A/IL6/ADIPOQ/SELE | 7 |  | DOID:1116 | pertussis | 3/75 | 35/8007 | 0.004166 | 0.01793 | 0.010427 | CCL2/FPR2/IL6 | 3 |
| 0.004407 | 0.069429 | 0.058117 | TNC/HMOX1/APOD/SPP1/IL6 | 5 |  | DOID:4724 | brain edema | 2/75 | 11/8007 | 0.004508 | 0.019119 | 0.011119 | VEGFA/IL6 | 2 |
| 0.004431 | 0.069429 | 0.058117 | APOD/ADIPOQ | 2 |  | DOID:656 | adrenal adenoma | 2/75 | 11/8007 | 0.004508 | 0.019119 | 0.011119 | IL6/ADIPOQ | 2 |
| 0.004431 | 0.069429 | 0.058117 | APOD/ADIPOQ | 2 |  | DOID:2377 | multiple sclerosis | 6/75 | 169/8007 | 0.004895 | 0.020609 | 0.011985 | SLC11A1/CCL2/HMOX1/IL6/CCL7/CFD | 6 |
| 0.004431 | 0.069429 | 0.058117 | PROM1/LIF | 2 |  | DOID:1596 | mental depression | 3/75 | 38/8007 | 0.005263 | 0.021687 | 0.012613 | CCL2/PTX3/SELP | 3 |
| 0.004504 | 0.070077 | 0.05866 | TNC/FOSL1/SOX10/SPP1/CA9 | 5 |  | DOID:3973 | thyroid medullary carcinoma | 3/75 | 38/8007 | 0.005263 | 0.021687 | 0.012613 | TNC/LIF/VEGFA | 3 |
| 0.004557 | 0.070393 | 0.058924 | PROM1/VEGFA/DIO3 | 3 |  | DOID:866 | vein disease | 3/75 | 38/8007 | 0.005263 | 0.021687 | 0.012613 | SERPINA1/HMOX1/VEGFA | 3 |
| 0.00491 | 0.075012 | 0.062791 | SLC11A1/MT2A/CCL2/CCL20/CCL7 | 5 |  | DOID:14499 | Fabry disease | 2/75 | 12/8007 | 0.005377 | 0.022001 | 0.012795 | IL6/SELE | 2 |
| 0.004934 | 0.075012 | 0.062791 | CCL2/CCL7 | 2 |  | DOID:10825 | essential hypertension | 5/75 | 122/8007 | 0.005582 | 0.022679 | 0.013189 | HMOX1/VEGFA/IL6/ADRA2B/ADIPOQ | 5 |
| 0.004959 | 0.075012 | 0.062791 | CXCL5/CCL2/CCL20/CCL7 | 4 |  | DOID:3770 | pulmonary fibrosis | 5/75 | 123/8007 | 0.005777 | 0.023212 | 0.0135 | CCL2/HMOX1/TREM1/VEGFA/IL6 | 5 |
| 0.005103 | 0.076269 | 0.063843 | CXCL5/CCL2/CCL20/CCL7 | 4 |  | DOID:3213 | demyelinating disease | 6/75 | 175/8007 | 0.005793 | 0.023212 | 0.0135 | SLC11A1/CCL2/HMOX1/IL6/CCL7/CFD | 6 |
| 0.005225 | 0.076269 | 0.063843 | LIF/SOX10/IL6 | 3 |  | DOID:3620 | central nervous system cancer | 5/75 | 124/8007 | 0.005976 | 0.023782 | 0.013831 | MT2A/MT1A/PROM1/VEGFA/CA9 | 5 |
| 0.005225 | 0.076269 | 0.063843 | HMOX1/APOD/ADIPOQ | 3 |  | DOID:0050339 | commensal bacterial infectious disease | 3/75 | 40/8007 | 0.006082 | 0.024039 | 0.013981 | CCL2/FPR2/IL6 | 3 |
| 0.005362 | 0.076269 | 0.063843 | SLC11A1/CXCL5/CCL2/TREM1/CXCL14/IL6/CFD | 7 |  | DOID:2921 | glomerulonephritis | 4/75 | 79/8007 | 0.006281 | 0.024303 | 0.014134 | CCL2/PTX3/IL6/SELE | 4 |
| 0.005399 | 0.076269 | 0.063843 | TNC/ACTC1/CCL7/ADIPOQ | 4 |  | DOID:1785 | pituitary cancer | 2/75 | 13/8007 | 0.006316 | 0.024303 | 0.014134 | LIF/IL6 | 2 |
| 0.005463 | 0.076269 | 0.063843 | VEGFA/RELN | 2 |  | DOID:288 | endometriosis of uterus | 2/75 | 13/8007 | 0.006316 | 0.024303 | 0.014134 | CCL2/IL6 | 2 |
| 0.005463 | 0.076269 | 0.063843 | LIF/SPP1 | 2 |  | DOID:6132 | bronchitis | 2/75 | 13/8007 | 0.006316 | 0.024303 | 0.014134 | THBD/VEGFA | 2 |
| 0.005463 | 0.076269 | 0.063843 | ST14/RSPO3 | 2 |  | DOID:2789 | parasitic protozoa infectious disease | 5/75 | 127/8007 | 0.006604 | 0.025174 | 0.01464 | SLC11A1/HMOX1/VEGFA/VWF/IL6 | 5 |
| 0.005463 | 0.076269 | 0.063843 | PROM1/ADIPOQ | 2 |  | DOID:1247 | blood coagulation disease | 6/75 | 180/8007 | 0.006629 | 0.025174 | 0.01464 | THBD/HMOX1/VEGFA/SELP/VWF/IL6 | 6 |
| 0.005463 | 0.076269 | 0.063843 | PROM1/ADIPOQ | 2 |  | DOID:4896 | bile duct adenocarcinoma | 5/75 | 128/8007 | 0.006823 | 0.025575 | 0.014874 | SERPINA1/PROM1/SPP1/VEGFA/IL6 | 5 |
| 0.005463 | 0.076269 | 0.063843 | CCL2/RAMP3 | 2 |  | DOID:4947 | cholangiocarcinoma | 5/75 | 128/8007 | 0.006823 | 0.025575 | 0.014874 | SERPINA1/PROM1/SPP1/VEGFA/IL6 | 5 |
| 0.005463 | 0.076269 | 0.063843 | CXCL14/CCL7 | 2 |  | DOID:1168 | familial hyperlipidemia | 4/75 | 82/8007 | 0.007159 | 0.026664 | 0.015507 | CCL2/MTTP/IL6/ADIPOQ | 4 |
| 0.005576 | 0.07713 | 0.064564 | ACTC1/ADRA1D/RAMP3/FGF12/SGCG/SCN4B | 6 |  | DOID:12206 | dengue hemorrhagic fever | 2/75 | 14/8007 | 0.007325 | 0.026934 | 0.015664 | CCL2/VEGFA | 2 |
| 0.005669 | 0.07713 | 0.064564 | LIF/SOX10/VEGFA/IL6/ADIPOQ/RELN | 6 |  | DOID:13001 | carotid stenosis | 2/75 | 14/8007 | 0.007325 | 0.026934 | 0.015664 | SPP1/IL6 | 2 |
| 0.005682 | 0.07713 | 0.064564 | IL31RA/LIF/MT1G/VEGFA/ADIPOQ | 5 |  | DOID:0060095 | uterine benign neoplasm | 3/75 | 43/8007 | 0.007447 | 0.027041 | 0.015726 | VEGFA/VWF/IL6 | 3 |
| 0.005682 | 0.07713 | 0.064564 | GPM6A/NRXN1/CLSTN2/CDH8/RELN | 5 |  | DOID:13223 | uterine fibroid | 3/75 | 43/8007 | 0.007447 | 0.027041 | 0.015726 | VEGFA/VWF/IL6 | 3 |
| 0.005701 | 0.07713 | 0.064564 | HMOX1/GPIHBP1/ADIPOQ | 3 |  | DOID:10603 | glucose intolerance | 3/75 | 44/8007 | 0.007938 | 0.028647 | 0.01666 | HMOX1/ADIPOQ/SELE | 3 |
| 0.005917 | 0.077346 | 0.064745 | CCL2/CCL20/IL6/CCL7/SELE | 5 |  | DOID:0060086 | female reproductive organ benign neoplasm | 3/75 | 45/8007 | 0.008449 | 0.030301 | 0.017622 | VEGFA/VWF/IL6 | 3 |
| 0.005949 | 0.077346 | 0.064745 | GRIK3/PCDH17/ADIPOQ | 3 |  | DOID:8857 | lupus erythematosus | 4/75 | 87/8007 | 0.008798 | 0.031359 | 0.018238 | CCL2/SELP/IL6/SELE | 4 |
| 0.005949 | 0.077346 | 0.064745 | CCL2/APOD/CCL20 | 3 |  | DOID:3952 | adrenal cortex disease | 3/75 | 46/8007 | 0.008978 | 0.031807 | 0.018498 | SELP/VWF/IL6 | 3 |
| 0.006016 | 0.077346 | 0.064745 | SOX10/VEGFA | 2 |  | DOID:0050622 | reproductive organ benign neoplasm | 3/75 | 47/8007 | 0.009527 | 0.032807 | 0.01908 | VEGFA/VWF/IL6 | 3 |
| 0.006016 | 0.077346 | 0.064745 | PLP1/SOX10 | 2 |  | DOID:9553 | adrenal gland disease | 3/75 | 47/8007 | 0.009527 | 0.032807 | 0.01908 | SELP/VWF/IL6 | 3 |
| 0.006016 | 0.077346 | 0.064745 | PLP1/SOX10 | 2 |  | DOID:11729 | Lyme disease | 2/75 | 16/8007 | 0.009543 | 0.032807 | 0.01908 | CCL2/IL6 | 2 |
| 0.006016 | 0.077346 | 0.064745 | PROM1/ADIPOQ | 2 |  | DOID:12205 | dengue disease | 2/75 | 16/8007 | 0.009543 | 0.032807 | 0.01908 | CCL2/VEGFA | 2 |
| 0.006037 | 0.077346 | 0.064745 | PLP1/CCL2/RAMP3/ADIPOQ/RELN | 5 |  | DOID:321 | tropical spastic paraparesis | 2/75 | 16/8007 | 0.009543 | 0.032807 | 0.01908 | VEGFA/IL6 | 2 |
| 0.006037 | 0.077346 | 0.064745 | PLP1/CCL2/RAMP3/ADIPOQ/RELN | 5 |  | DOID:4606 | bile duct cancer | 5/75 | 140/8007 | 0.009864 | 0.033514 | 0.019491 | SERPINA1/PROM1/SPP1/VEGFA/IL6 | 5 |
| 0.006159 | 0.078361 | 0.065594 | ACTC1/VEGFA/NEBL/SGCG/PI16 | 5 |  | DOID:4897 | bile duct carcinoma | 5/75 | 140/8007 | 0.009864 | 0.033514 | 0.019491 | SERPINA1/PROM1/SPP1/VEGFA/IL6 | 5 |
| 0.006188 | 0.078361 | 0.065594 | PROM1/LIF/VEGFA/ADIPOQ | 4 |  | DOID:345 | uterine disease | 3/75 | 48/8007 | 0.010095 | 0.034098 | 0.019831 | CCL2/IL6/SELE | 3 |
| 0.006408 | 0.079343 | 0.066416 | HK3/GCKR/APOD/SLC39A14/ADIPOQ | 5 |  | DOID:3146 | lipid metabolism disorder | 4/75 | 92/8007 | 0.010665 | 0.03569 | 0.020756 | CCL2/MTTP/IL6/ADIPOQ | 4 |
| 0.006413 | 0.079343 | 0.066416 | TNC/PTX3/SPP1/OLFML2A/VIT/VWF/IL6 | 7 |  | DOID:10762 | portal hypertension | 2/75 | 17/8007 | 0.01075 | 0.03569 | 0.020756 | SERPINA1/HMOX1 | 2 |
| 0.0065 | 0.079343 | 0.066416 | THBD/SIGLEC10/CCL2/SPP1/FPR2/SEMA3G/ADIPOQ | 7 |  | DOID:1969 | cerebral palsy | 2/75 | 17/8007 | 0.01075 | 0.03569 | 0.020756 | PLP1/IL6 | 2 |
| 0.0065 | 0.079343 | 0.066416 | TNC/PTX3/SPP1/OLFML2A/VIT/VWF/IL6 | 7 |  | DOID:8466 | retinal degeneration | 7/75 | 261/8007 | 0.010843 | 0.035794 | 0.020817 | PROM1/CCL2/VEGFA/SELP/CA4/CFD/SELE | 7 |
| 0.006524 | 0.079343 | 0.066416 | SOX10/SPP1/SEMA3G/IL6 | 4 |  | DOID:1192 | peripheral nervous system neoplasm | 9/75 | 396/8007 | 0.011249 | 0.036925 | 0.021474 | TNC/PROM1/CCL2/LIF/HMOX1/HMGA1/VEGFA/IL6/RELN | 9 |
| 0.006594 | 0.079343 | 0.066416 | CCL20/SELP | 2 |  | DOID:5041 | esophageal cancer | 5/75 | 145/8007 | 0.011369 | 0.03711 | 0.021583 | SLC11A1/MT2A/CCL2/VEGFA/SELE | 5 |
| 0.006594 | 0.079343 | 0.066416 | LIF/ADIPOQ | 2 |  | DOID:0050737 | autosomal recessive disease | 8/75 | 334/8007 | 0.012477 | 0.040497 | 0.023552 | SERPINA1/PTX3/HMOX1/TREM1/MARCO/SELP/IL6/SELE | 8 |
| 0.006594 | 0.079343 | 0.066416 | LIF/ADIPOQ | 2 |  | DOID:987 | alopecia | 3/75 | 52/8007 | 0.01256 | 0.04054 | 0.023577 | CCL2/LIF/IL6 | 3 |
| 0.006594 | 0.079343 | 0.066416 | HMOX1/VEGFA | 2 |  | DOID:1398 | parasitic infectious disease | 5/75 | 150/8007 | 0.013025 | 0.041811 | 0.024316 | SLC11A1/HMOX1/VEGFA/VWF/IL6 | 5 |
| 0.006676 | 0.079892 | 0.066876 | TNC/PTX3/SPP1/OLFML2A/VIT/VWF/IL6 | 7 |  | DOID:7166 | thyroiditis | 3/75 | 53/8007 | 0.013225 | 0.042218 | 0.024553 | CCL2/VEGFA/IL6 | 3 |
| 0.00673 | 0.080101 | 0.067051 | IL31RA/LIF/IL6 | 3 |  | DOID:3407 | carotid artery disease | 2/75 | 19/8007 | 0.013354 | 0.042399 | 0.024658 | SPP1/IL6 | 2 |
| 0.006927 | 0.081617 | 0.06832 | GPM6A/NRXN1/CLSTN2/CDH8/RELN | 5 |  | DOID:3310 | atopic dermatitis | 5/75 | 152/8007 | 0.013731 | 0.043358 | 0.025216 | HMOX1/IL1RL1/VEGFA/SELP/SELE | 5 |
| 0.007004 | 0.081617 | 0.06832 | PROM1/GPM6A/DIO3 | 3 |  | DOID:10534 | stomach cancer | 7/75 | 274/8007 | 0.013896 | 0.043451 | 0.02527 | CXCL5/PROM1/CCL2/HMOX1/SPP1/IL6/SELE | 7 |
| 0.007004 | 0.081617 | 0.06832 | CCL2/SELP/SELE | 3 |  | DOID:13378 | Kawasaki disease | 3/75 | 54/8007 | 0.01391 | 0.043451 | 0.02527 | SLC11A1/CCL2/VEGFA | 3 |
| 0.007196 | 0.081617 | 0.06832 | APOD/ADIPOQ | 2 |  | DOID:12252 | Cushing's syndrome | 2/75 | 20/8007 | 0.014749 | 0.04534 | 0.026369 | VWF/IL6 | 2 |
| 0.007196 | 0.081617 | 0.06832 | RAMP3/ADIPOQ | 2 |  | DOID:13406 | pulmonary sarcoidosis | 2/75 | 20/8007 | 0.014749 | 0.04534 | 0.026369 | CXCL5/VEGFA | 2 |
| 0.007196 | 0.081617 | 0.06832 | LIF/ST14 | 2 |  | DOID:1532 | pleural disease | 2/75 | 20/8007 | 0.014749 | 0.04534 | 0.026369 | SLC11A1/HMOX1 | 2 |
| 0.007196 | 0.081617 | 0.06832 | CCL2/APOD | 2 |  | DOID:3962 | follicular thyroid carcinoma | 3/75 | 56/8007 | 0.015341 | 0.04691 | 0.027282 | TNC/LIF/VEGFA | 3 |
| 0.007196 | 0.081617 | 0.06832 | CCL2/CCL7 | 2 |  | DOID:5295 | intestinal disease | 5/75 | 157/8007 | 0.015608 | 0.047293 | 0.027504 | SLC11A1/SOX10/VEGFA/IL6/SELE | 5 |
| 0.007197 | 0.081617 | 0.06832 | MT1X/MT2A/MT1A/HMOX1/MT1G | 5 |  | DOID:12365 | malaria | 4/75 | 103/8007 | 0.015629 | 0.047293 | 0.027504 | HMOX1/VEGFA/VWF/IL6 | 4 |
| 0.007232 | 0.081617 | 0.06832 | NRXN1/SOX10/FGF12/RELN | 4 |  | DOID:2645 | benign mesothelioma | 3/75 | 57/8007 | 0.016086 | 0.048033 | 0.027935 | SPP1/VEGFA/CA9 | 3 |
| 0.007335 | 0.082001 | 0.068641 | LIF/SOX10/VEGFA/IL6/RELN | 5 |  | DOID:3087 | gingivitis | 2/75 | 21/8007 | 0.016204 | 0.048033 | 0.027935 | VEGFA/IL6 | 2 |
| 0.007417 | 0.082001 | 0.068641 | SOX10/SPP1/SEMA3G/IL6 | 4 |  | DOID:8283 | peritonitis | 2/75 | 21/8007 | 0.016204 | 0.048033 | 0.027935 | CCL2/TREM1 | 2 |
| 0.007417 | 0.082001 | 0.068641 | PROM1/LIF/VEGFA/ADIPOQ | 4 |  | DOID:9743 | diabetic neuropathy | 2/75 | 21/8007 | 0.016204 | 0.048033 | 0.027935 | THBD/VEGFA | 2 |
| 0.007417 | 0.082001 | 0.068641 | MT1X/MT2A/MT1A/MT1G | 4 |  | DOID:4766 | embryoma | 8/75 | 352/8007 | 0.016679 | 0.049191 | 0.028608 | SLC11A1/TNC/PROM1/IL1RL1/CCL20/PCSK1/CA9/IL6 | 8 |
| 0.007605 | 0.083652 | 0.070023 | LIF/HMOX1/ADRA2B/ADIPOQ | 4 |  | DOID:1602 | lymphadenitis | 3/75 | 58/8007 | 0.016851 | 0.049198 | 0.028612 | SLC11A1/CCL2/VEGFA | 3 |
| 0.007821 | 0.083916 | 0.070244 | LIF/IL6 | 2 |  | DOID:9942 | lymph node disease | 3/75 | 58/8007 | 0.016851 | 0.049198 | 0.028612 | SLC11A1/CCL2/VEGFA | 3 |
| 0.007821 | 0.083916 | 0.070244 | ANGPTL4/GPIHBP1 | 2 |  | DOID:2234 | focal epilepsy | 3/75 | 59/8007 | 0.017637 | 0.051212 | 0.029784 | LGI1/IL6/RELN | 3 |
| 0.007821 | 0.083916 | 0.070244 | NRXN1/RELN | 2 |  | DOID:14221 | metabolic syndrome X | 2/75 | 22/8007 | 0.017717 | 0.051212 | 0.029784 | IL6/ADIPOQ | 2 |
| 0.007821 | 0.083916 | 0.070244 | ST14/RSPO3 | 2 |  | DOID:10652 | Alzheimer's disease | 9/75 | 430/8007 | 0.018439 | 0.053035 | 0.030844 | SERPINA1/SERPINA3/CCL2/HMOX1/APOD/VEGFA/IL6/OLR1/RELN | 9 |
| 0.007821 | 0.083916 | 0.070244 | PROM1/ADIPOQ | 2 |  | DOID:2237 | hepatitis | 9/75 | 431/8007 | 0.018692 | 0.053498 | 0.031113 | SERPINA1/CCL2/MTTP/HMOX1/CCL20/SPP1/VEGFA/IL6/ADIPOQ | 9 |
| 0.007989 | 0.085299 | 0.071402 | PLP1/CCL2/RAMP3/RELN | 4 |  | DOID:341 | peripheral vascular disease | 3/75 | 61/8007 | 0.019268 | 0.054876 | 0.031915 | VEGFA/IL6/SELE | 3 |
| 0.008216 | 0.086212 | 0.072167 | TNC/GPM6A/NRXN1/CLSTN2/CDH8/PCDH17/RELN | 7 |  | DOID:11612 | polycystic ovary syndrome | 5/75 | 166/8007 | 0.019406 | 0.054902 | 0.03193 | HMOX1/VEGFA/IL6/ADRA2B/ADIPOQ | 5 |
| 0.008387 | 0.086212 | 0.072167 | ADRA1D/RAMP3/ADCY4/ADRA2B | 4 |  | DOID:680 | tauopathy | 9/75 | 434/8007 | 0.019466 | 0.054902 | 0.03193 | SERPINA1/SERPINA3/CCL2/HMOX1/APOD/VEGFA/IL6/OLR1/RELN | 9 |
| 0.008387 | 0.086212 | 0.072167 | LIF/RAMP3/VEGFA/IL6 | 4 |  | DOID:11984 | hypertrophic cardiomyopathy | 3/75 | 62/8007 | 0.020114 | 0.056456 | 0.032833 | CCL2/ACTC1/VWF | 3 |
| 0.008471 | 0.086212 | 0.072167 | VEGFA/SELE | 2 |  | DOID:1520 | colon carcinoma | 5/75 | 168/8007 | 0.020326 | 0.056776 | 0.033019 | PROM1/HMOX1/VEGFA/SELP/SELE | 5 |
| 0.008471 | 0.086212 | 0.072167 | APOD/IL6 | 2 |  | DOID:1681 | heart septal defect | 2/75 | 24/8007 | 0.020916 | 0.057771 | 0.033598 | ACTC1/VEGFA | 2 |
| 0.008471 | 0.086212 | 0.072167 | FPR2/IL6 | 2 |  | DOID:272 | hepatic vascular disease | 2/75 | 24/8007 | 0.020916 | 0.057771 | 0.033598 | SERPINA1/HMOX1 | 2 |
| 0.008471 | 0.086212 | 0.072167 | PROM1/DIO3 | 2 |  | DOID:3371 | chondrosarcoma | 3/75 | 63/8007 | 0.020981 | 0.057771 | 0.033598 | TNC/VEGFA/IL6 | 3 |
| 0.008471 | 0.086212 | 0.072167 | LIF/ADIPOQ | 2 |  | DOID:13810 | familial hypercholesterolemia | 2/75 | 25/8007 | 0.022598 | 0.061353 | 0.035681 | MTTP/ADIPOQ | 2 |
| 0.008471 | 0.086212 | 0.072167 | LIF/ADIPOQ | 2 |  | DOID:14069 | cerebral malaria | 2/75 | 25/8007 | 0.022598 | 0.061353 | 0.035681 | HMOX1/VEGFA | 2 |
| 0.008471 | 0.086212 | 0.072167 | GREM2/ADIPOQ | 2 |  | DOID:687 | hepatoblastoma | 2/75 | 25/8007 | 0.022598 | 0.061353 | 0.035681 | HMOX1/MT1G | 2 |
| 0.00859 | 0.08702 | 0.072843 | ACTC1/VEGFA/NEBL/PI16 | 4 |  | DOID:688 | embryonal cancer | 8/75 | 373/8007 | 0.022786 | 0.061574 | 0.03581 | SLC11A1/TNC/PROM1/IL1RL1/CCL20/PCSK1/CA9/IL6 | 8 |
| 0.008797 | 0.088701 | 0.07425 | GPM6A/VEGFA/LHX6/RELN | 4 |  | DOID:2621 | autonomic nervous system neoplasm | 8/75 | 375/8007 | 0.02344 | 0.062759 | 0.036499 | PROM1/CCL2/LIF/HMOX1/HMGA1/VEGFA/IL6/RELN | 8 |
| 0.009101 | 0.090935 | 0.07612 | TNC/APOD/SPP1 | 3 |  | DOID:769 | neuroblastoma | 8/75 | 375/8007 | 0.02344 | 0.062759 | 0.036499 | PROM1/CCL2/LIF/HMOX1/HMGA1/VEGFA/IL6/RELN | 8 |
| 0.009136 | 0.090935 | 0.07612 | HK3/GCKR/SLC39A14/IL6/ADIPOQ | 5 |  | DOID:9471 | meningitis | 2/75 | 26/8007 | 0.024335 | 0.064856 | 0.037718 | CXCL5/VEGFA | 2 |
| 0.009144 | 0.090935 | 0.07612 | ADRA1D/ADRA2B | 2 |  | DOID:936 | brain disease | 9/75 | 454/8007 | 0.025237 | 0.066954 | 0.038939 | PLP1/CCL2/PTX3/LGI1/APOD/VEGFA/IL6/ADRA2B/RELN | 9 |
| 0.009299 | 0.092057 | 0.077059 | HK3/GCKR/SLC39A14/IL6/ADIPOQ | 5 |  | DOID:4607 | biliary tract cancer | 5/75 | 178/8007 | 0.025354 | 0.066959 | 0.038941 | SERPINA1/PROM1/SPP1/VEGFA/IL6 | 5 |
| 0.009436 | 0.092995 | 0.077844 | CXCL5/CCL2/CCL20/CCL7 | 4 |  | DOID:1588 | thrombocytopenia | 3/75 | 68/8007 | 0.025617 | 0.067346 | 0.039167 | THBD/VWF/IL6 | 3 |
| 0.00965 | 0.093176 | 0.077996 | GPM6A/NRXN1/CLSTN2/APOD/VEGFA/CDH8/PCDH17 | 7 |  | DOID:10159 | osteonecrosis | 2/75 | 27/8007 | 0.026125 | 0.067459 | 0.039233 | VEGFA/ADIPOQ | 2 |
| 0.00965 | 0.093176 | 0.077996 | CCL2/LIF/VEGFA/VIT/SELP/IL6/SELE | 7 |  | DOID:13129 | severe pre-eclampsia | 2/75 | 27/8007 | 0.026125 | 0.067459 | 0.039233 | THBD/IL6 | 2 |
| 0.00976 | 0.093176 | 0.077996 | LIF/ST14/RSPO3 | 3 |  | DOID:1724 | duodenal ulcer | 2/75 | 27/8007 | 0.026125 | 0.067459 | 0.039233 | IL6/SELE | 2 |
| 0.00976 | 0.093176 | 0.077996 | SLC11A1/SLC39A14/SCARA5 | 3 |  | DOID:2978 | carbohydrate metabolic disorder | 2/75 | 27/8007 | 0.026125 | 0.067459 | 0.039233 | VWF/IL6 | 2 |
| 0.009799 | 0.093176 | 0.077996 | TNC/ACTC1/FOSL1/CCL7/ADIPOQ | 5 |  | DOID:3068 | glioblastoma multiforme | 3/75 | 70/8007 | 0.027614 | 0.070953 | 0.041264 | TNC/PROM1/CCL2 | 3 |
| 0.00984 | 0.093176 | 0.077996 | C2CD4A/IL6 | 2 |  | DOID:13141 | uveitis | 2/75 | 28/8007 | 0.027966 | 0.070953 | 0.041264 | SERPINA1/CCL2 | 2 |
| 0.00984 | 0.093176 | 0.077996 | SLC11A1/IL1RL1 | 2 |  | DOID:8670 | eating disorder | 2/75 | 28/8007 | 0.027966 | 0.070953 | 0.041264 | IL6/ADIPOQ | 2 |
| 0.00984 | 0.093176 | 0.077996 | PLP1/GRIK3 | 2 |  | DOID:9446 | cholangitis | 2/75 | 28/8007 | 0.027966 | 0.070953 | 0.041264 | TREM1/IL6 | 2 |
| 0.00984 | 0.093176 | 0.077996 | NRXN1/RELN | 2 |  | DOID:11963 | esophagitis | 2/75 | 29/8007 | 0.029858 | 0.075098 | 0.043675 | CCL2/IL6 | 2 |
| 0.010092 | 0.094865 | 0.07941 | MARCO/RAMP3/VEGFA/FPR2/ADIPOQ/SELE | 6 |  | DOID:4798 | aggressive systemic mastocytosis | 2/75 | 29/8007 | 0.029858 | 0.075098 | 0.043675 | VEGFA/IL6 | 2 |
| 0.010105 | 0.094865 | 0.07941 | CCL2/PALMD/VEGFA/CCL7 | 4 |  | DOID:6364 | migraine | 3/75 | 73/8007 | 0.030761 | 0.077034 | 0.044801 | VEGFA/IL6/ADRA2B | 3 |
| 0.010558 | 0.098273 | 0.082262 | LIF/SPP1 | 2 |  | DOID:3324 | mood disorder | 5/75 | 189/8007 | 0.031743 | 0.07908 | 0.045991 | CCL2/PTX3/SELP/VWF/RELN | 5 |
| 0.010558 | 0.098273 | 0.082262 | IL6/ADIPOQ | 2 |  | DOID:574 | peripheral nervous system disease | 3/75 | 74/8007 | 0.03185 | 0.07908 | 0.045991 | TNC/VEGFA/IL6 | 3 |
| 0.011118 | 0.10304 | 0.086252 | LIF/ST14/FOSL1/SOX10/VEGFA/RSPO3 | 6 |  | DOID:0060041 | autism spectrum disorder | 5/75 | 190/8007 | 0.032369 | 0.079688 | 0.046345 | NRXN1/VEGFA/CDH8/ADIPOQ/RELN | 5 |
| 0.011528 | 0.106391 | 0.089057 | HMOX1/IL6/TRIL | 3 |  | DOID:12849 | autistic disorder | 5/75 | 190/8007 | 0.032369 | 0.079688 | 0.046345 | NRXN1/VEGFA/CDH8/ADIPOQ/RELN | 5 |
| 0.011894 | 0.108466 | 0.090794 | PLP1/CCL2/RAMP3/FGF12/SCN4B/RELN | 6 |  | DOID:11394 | adult respiratory distress syndrome | 2/75 | 31/8007 | 0.03379 | 0.081801 | 0.047573 | VEGFA/IL6 | 2 |
| 0.011902 | 0.108466 | 0.090794 | IL31RA/LIF/IL6 | 3 |  | DOID:319 | spinal cord disease | 2/75 | 31/8007 | 0.03379 | 0.081801 | 0.047573 | VEGFA/IL6 | 2 |
| 0.011902 | 0.108466 | 0.090794 | SLC11A1/SELP/IL6 | 3 |  | DOID:4610 | intestinal benign neoplasm | 2/75 | 31/8007 | 0.03379 | 0.081801 | 0.047573 | VEGFA/IL6 | 2 |
| 0.011984 | 0.108567 | 0.09088 | SLC38A5/ADRA1D/VEGFA/C2CD4A/ADRA2B | 5 |  | DOID:5100 | middle ear disease | 2/75 | 31/8007 | 0.03379 | 0.081801 | 0.047573 | VEGFA/IL6 | 2 |
| 0.012063 | 0.108567 | 0.09088 | SELP/SELE | 2 |  | DOID:0060085 | organ system benign neoplasm | 6/75 | 262/8007 | 0.035607 | 0.085841 | 0.049923 | LIF/VEGFA/PCSK1/VWF/IL6/ADIPOQ | 6 |
| 0.012063 | 0.108567 | 0.09088 | VEGFA/ADIPOQ | 2 |  |  |  |  |  |  |  |  |  |  |
| 0.012294 | 0.10974 | 0.091861 | HMOX1/APOD/IL6/ADIPOQ | 4 |  |  |  |  |  |  |  |  |  |  |
| 0.012294 | 0.10974 | 0.091861 | LIF/HMOX1/ADRA2B/ADIPOQ | 4 |  |  |  |  |  |  |  |  |  |  |
| 0.012672 | 0.111937 | 0.0937 | SCN7A/FGF12/SCN4B | 3 |  |  |  |  |  |  |  |  |  |  |
| 0.012819 | 0.111937 | 0.0937 | HMOX1/APOD/IL6/ADIPOQ | 4 |  |  |  |  |  |  |  |  |  |  |
| 0.012819 | 0.111937 | 0.0937 | ANGPTL4/MTTP/GCKR/GPIHBP1 | 4 |  |  |  |  |  |  |  |  |  |  |
| 0.012849 | 0.111937 | 0.0937 | ADRA1D/ADRA2B | 2 |  |  |  |  |  |  |  |  |  |  |
| 0.012849 | 0.111937 | 0.0937 | HMOX1/IL6 | 2 |  |  |  |  |  |  |  |  |  |  |
| 0.012849 | 0.111937 | 0.0937 | SOX10/VEGFA | 2 |  |  |  |  |  |  |  |  |  |  |
| 0.012985 | 0.112488 | 0.094161 | HK3/GCKR/APOD/SLC39A14/ADIPOQ | 5 |  |  |  |  |  |  |  |  |  |  |
| 0.013067 | 0.112488 | 0.094161 | IL31RA/LIF/IL6 | 3 |  |  |  |  |  |  |  |  |  |  |
| 0.013067 | 0.112488 | 0.094161 | PROM1/LIF/VEGFA | 3 |  |  |  |  |  |  |  |  |  |  |
| 0.013192 | 0.113115 | 0.094686 | GPM6A/LIF/SOX10/MEOX1/SEMA3G | 5 |  |  |  |  |  |  |  |  |  |  |
| 0.013349 | 0.114012 | 0.095437 | ACTC1/HMOX1/SCN7A/FGF12/PI16/SCN4B/ADRA2B | 7 |  |  |  |  |  |  |  |  |  |  |
| 0.013632 | 0.115281 | 0.096499 | TNC/HMOX1/SPP1/ADIPOQ | 4 |  |  |  |  |  |  |  |  |  |  |
| 0.013656 | 0.115281 | 0.096499 | VEGFA/DIO3 | 2 |  |  |  |  |  |  |  |  |  |  |
| 0.013656 | 0.115281 | 0.096499 | VEGFA/SEMA3G | 2 |  |  |  |  |  |  |  |  |  |  |
| 0.013878 | 0.115652 | 0.09681 | PROM1/LIF/VEGFA | 3 |  |  |  |  |  |  |  |  |  |  |
| 0.013878 | 0.115652 | 0.09681 | THBD/SIGLEC10/SPP1 | 3 |  |  |  |  |  |  |  |  |  |  |
| 0.01391 | 0.115652 | 0.09681 | IL31RA/CCL2/LIF/IL6 | 4 |  |  |  |  |  |  |  |  |  |  |
| 0.013912 | 0.115652 | 0.09681 | MT1X/MT2A/MT1A/MTTP/HMOX1/MT1G | 6 |  |  |  |  |  |  |  |  |  |  |
| 0.014192 | 0.116412 | 0.097446 | CLSTN2/MPZL2/CDH8/PCDH17 | 4 |  |  |  |  |  |  |  |  |  |  |
| 0.014477 | 0.116412 | 0.097446 | ACTC1/VEGFA/NEBL/PI16 | 4 |  |  |  |  |  |  |  |  |  |  |
| 0.014479 | 0.116412 | 0.097446 | SERPINA1/SERPINA3/VEGFA/PI16/PI15 | 5 |  |  |  |  |  |  |  |  |  |  |
| 0.014485 | 0.116412 | 0.097446 | SCN7A/FGF12 | 2 |  |  |  |  |  |  |  |  |  |  |
| 0.014485 | 0.116412 | 0.097446 | MTTP/GPIHBP1 | 2 |  |  |  |  |  |  |  |  |  |  |
| 0.014485 | 0.116412 | 0.097446 | MTTP/GPIHBP1 | 2 |  |  |  |  |  |  |  |  |  |  |
| 0.014485 | 0.116412 | 0.097446 | VEGFA/IL6 | 2 |  |  |  |  |  |  |  |  |  |  |
| 0.014485 | 0.116412 | 0.097446 | SOX10/IL6 | 2 |  |  |  |  |  |  |  |  |  |  |
| 0.014485 | 0.116412 | 0.097446 | LIF/VEGFA | 2 |  |  |  |  |  |  |  |  |  |  |
| 0.014717 | 0.117846 | 0.098646 | TNC/SOX10/SPP1 | 3 |  |  |  |  |  |  |  |  |  |  |
| 0.01482 | 0.118017 | 0.09879 | LIF/HMOX1/FRMD7/GREM2/EBF2/ADIPOQ | 6 |  |  |  |  |  |  |  |  |  |  |
| 0.015147 | 0.118017 | 0.09879 | HMOX1/IL6/TRIL | 3 |  |  |  |  |  |  |  |  |  |  |
| 0.015147 | 0.118017 | 0.09879 | LIF/SOX10/IL6 | 3 |  |  |  |  |  |  |  |  |  |  |
| 0.015147 | 0.118017 | 0.09879 | THBD/FOSL1/ADIPOQ | 3 |  |  |  |  |  |  |  |  |  |  |
| 0.015154 | 0.118017 | 0.09879 | SERPINA3/PROM1/SPP1/VEGFA/IL6 | 5 |  |  |  |  |  |  |  |  |  |  |
| 0.015154 | 0.118017 | 0.09879 | MYRIP/LIF/SPP1/IL6/ADIPOQ | 5 |  |  |  |  |  |  |  |  |  |  |
| 0.015335 | 0.118017 | 0.09879 | SELP/SELE | 2 |  |  |  |  |  |  |  |  |  |  |
| 0.015335 | 0.118017 | 0.09879 | THBD/SELP | 2 |  |  |  |  |  |  |  |  |  |  |
| 0.015335 | 0.118017 | 0.09879 | TNC/SPP1 | 2 |  |  |  |  |  |  |  |  |  |  |
| 0.015335 | 0.118017 | 0.09879 | ADRA1D/ADRA2B | 2 |  |  |  |  |  |  |  |  |  |  |
| 0.015335 | 0.118017 | 0.09879 | HMOX1/VEGFA | 2 |  |  |  |  |  |  |  |  |  |  |
| 0.015575 | 0.119447 | 0.099986 | TNC/SERPINA1/FBXL22/SPP1/CHRDL1/IL6 | 6 |  |  |  |  |  |  |  |  |  |  |
| 0.015959 | 0.121958 | 0.102089 | GPM6A/NRXN1/CLSTN2/PCDH17 | 4 |  |  |  |  |  |  |  |  |  |  |
| 0.016206 | 0.122982 | 0.102946 | FGF12/SCN4B | 2 |  |  |  |  |  |  |  |  |  |  |
| 0.016206 | 0.122982 | 0.102946 | MTTP/GPIHBP1 | 2 |  |  |  |  |  |  |  |  |  |  |
| 0.016481 | 0.124499 | 0.104216 | NRXN1/CLSTN2/VEGFA | 3 |  |  |  |  |  |  |  |  |  |  |
| 0.016577 | 0.124499 | 0.104216 | LIF/SPP1/RSPO3/IL6 | 4 |  |  |  |  |  |  |  |  |  |  |
| 0.016577 | 0.124499 | 0.104216 | IL31RA/CCL2/LIF/IL6 | 4 |  |  |  |  |  |  |  |  |  |  |
| 0.016758 | 0.124946 | 0.10459 | SLC11A1/HMOX1/IL1RL1/APOD/IL6/ADIPOQ | 6 |  |  |  |  |  |  |  |  |  |  |
| 0.0169 | 0.124946 | 0.10459 | NRXN1/MYRIP/LIF/SPP1/IL6/ADRA2B/ADIPOQ | 7 |  |  |  |  |  |  |  |  |  |  |
| 0.016939 | 0.124946 | 0.10459 | ANGPTL4/GPIHBP1/SELE | 3 |  |  |  |  |  |  |  |  |  |  |
| 0.016939 | 0.124946 | 0.10459 | VEGFA/EBF2/ADIPOQ | 3 |  |  |  |  |  |  |  |  |  |  |
| 0.017055 | 0.124946 | 0.10459 | NRXN1/SOX10/VEGFA/LHX6/RELN | 5 |  |  |  |  |  |  |  |  |  |  |
| 0.017097 | 0.124946 | 0.10459 | SCN7A/SCN4B | 2 |  |  |  |  |  |  |  |  |  |  |
| 0.017097 | 0.124946 | 0.10459 | CCL2/RELN | 2 |  |  |  |  |  |  |  |  |  |  |
| 0.017165 | 0.124946 | 0.10459 | IL31RA/LIF/VEGFA/IL6/ADIPOQ/RELN | 6 |  |  |  |  |  |  |  |  |  |  |
| 0.01721 | 0.124946 | 0.10459 | HMOX1/ADRA1D/ADRA2B/ADIPOQ | 4 |  |  |  |  |  |  |  |  |  |  |
| 0.01721 | 0.124946 | 0.10459 | MT2A/CCL2/CCL20/CCL7 | 4 |  |  |  |  |  |  |  |  |  |  |
| 0.01779 | 0.128142 | 0.107265 | IL31RA/LIF/VEGFA/IL6/ADIPOQ/RELN | 6 |  |  |  |  |  |  |  |  |  |  |
| 0.017807 | 0.128142 | 0.107265 | MTTP/SPP1/AQP7/ADRA2B/ADIPOQ | 5 |  |  |  |  |  |  |  |  |  |  |
| 0.018009 | 0.128142 | 0.107265 | RAMP3/ADIPOQ | 2 |  |  |  |  |  |  |  |  |  |  |
| 0.018009 | 0.128142 | 0.107265 | VEGFA/SEMA3G | 2 |  |  |  |  |  |  |  |  |  |  |
| 0.018009 | 0.128142 | 0.107265 | HMGA1/IL6 | 2 |  |  |  |  |  |  |  |  |  |  |
| 0.018009 | 0.128142 | 0.107265 | VEGFA/SEMA3G | 2 |  |  |  |  |  |  |  |  |  |  |
| 0.018062 | 0.128142 | 0.107265 | HK3/GCKR/APOD/SLC39A14/ADIPOQ | 5 |  |  |  |  |  |  |  |  |  |  |
| 0.018188 | 0.128616 | 0.107662 | CCL2/CCL20/IL6/CCL7 | 4 |  |  |  |  |  |  |  |  |  |  |
| 0.018646 | 0.131427 | 0.110015 | SLC11A1/MTTP/SLC38A5/SLCO2A1/CA9/CA4 | 6 |  |  |  |  |  |  |  |  |  |  |
| 0.018942 | 0.133081 | 0.111399 | HMOX1/ADIPOQ | 2 |  |  |  |  |  |  |  |  |  |  |
| 0.019376 | 0.135694 | 0.113587 | NRXN1/LGI1/VEGFA/SEMA3G/RELN | 5 |  |  |  |  |  |  |  |  |  |  |
| 0.019545 | 0.136424 | 0.114198 | HMOX1/VEGFA/ESM1/RSPO3 | 4 |  |  |  |  |  |  |  |  |  |  |
| 0.019646 | 0.136424 | 0.114198 | NRXN1/LGI1/VEGFA/SEMA3G/RELN | 5 |  |  |  |  |  |  |  |  |  |  |
| 0.019756 | 0.136424 | 0.114198 | SLC11A1/CCL2/PTX3/MARCO/FPR2/ADIPOQ | 6 |  |  |  |  |  |  |  |  |  |  |
| 0.01984 | 0.136424 | 0.114198 | ACTC1/VEGFA/NEBL | 3 |  |  |  |  |  |  |  |  |  |  |
| 0.019894 | 0.136424 | 0.114198 | LHX6/RELN | 2 |  |  |  |  |  |  |  |  |  |  |
| 0.019894 | 0.136424 | 0.114198 | LIF/ADIPOQ | 2 |  |  |  |  |  |  |  |  |  |  |
| 0.019919 | 0.136424 | 0.114198 | ACTC1/ADRA1D/FGF12/SGCG/SCN4B | 5 |  |  |  |  |  |  |  |  |  |  |
| 0.020348 | 0.13893 | 0.116296 | SPP1/VEGFA/SEMA3G | 3 |  |  |  |  |  |  |  |  |  |  |
| 0.020866 | 0.141577 | 0.118511 | CA9/CA4 | 2 |  |  |  |  |  |  |  |  |  |  |
| 0.020866 | 0.141577 | 0.118511 | CCL2/IL6 | 2 |  |  |  |  |  |  |  |  |  |  |
| 0.021034 | 0.142272 | 0.119093 | CCL2/FGF12/SCN4B/ADIPOQ/RELN | 5 |  |  |  |  |  |  |  |  |  |  |
| 0.021387 | 0.144211 | 0.120716 | VEGFA/ADIPOQ/SELE | 3 |  |  |  |  |  |  |  |  |  |  |
| 0.021858 | 0.14509 | 0.121451 | SPP1/CA9 | 2 |  |  |  |  |  |  |  |  |  |  |
| 0.021858 | 0.14509 | 0.121451 | HMOX1/ADIPOQ | 2 |  |  |  |  |  |  |  |  |  |  |
| 0.021858 | 0.14509 | 0.121451 | SLC11A1/IL1RL1 | 2 |  |  |  |  |  |  |  |  |  |  |
| 0.021858 | 0.14509 | 0.121451 | CCL2/RAMP3 | 2 |  |  |  |  |  |  |  |  |  |  |
| 0.021858 | 0.14509 | 0.121451 | APOD/ADIPOQ | 2 |  |  |  |  |  |  |  |  |  |  |
| 0.021917 | 0.14509 | 0.121451 | SPP1/PI16/SEMA3G | 3 |  |  |  |  |  |  |  |  |  |  |
| 0.022189 | 0.146447 | 0.122588 | ACTC1/SOX10/MEOX1/SEMA3G/IL6 | 5 |  |  |  |  |  |  |  |  |  |  |
| 0.022868 | 0.150021 | 0.125579 | TNC/HMOX1 | 2 |  |  |  |  |  |  |  |  |  |  |
| 0.022868 | 0.150021 | 0.125579 | VEGFA/C2CD4A | 2 |  |  |  |  |  |  |  |  |  |  |
| 0.022998 | 0.150419 | 0.125913 | HMOX1/VEGFA/CA9 | 3 |  |  |  |  |  |  |  |  |  |  |
| 0.023593 | 0.153849 | 0.128784 | NRXN1/CLSTN2/APOD/VEGFA | 4 |  |  |  |  |  |  |  |  |  |  |
| 0.023898 | 0.153993 | 0.128904 | PLP1/SOX10 | 2 |  |  |  |  |  |  |  |  |  |  |
| 0.023898 | 0.153993 | 0.128904 | CA9/CA4 | 2 |  |  |  |  |  |  |  |  |  |  |
| 0.023898 | 0.153993 | 0.128904 | CDH8/SELP | 2 |  |  |  |  |  |  |  |  |  |  |
| 0.023898 | 0.153993 | 0.128904 | APOD/CCL20 | 2 |  |  |  |  |  |  |  |  |  |  |
| 0.024108 | 0.154888 | 0.129653 | RAMP3/VEGFA/SELE | 3 |  |  |  |  |  |  |  |  |  |  |
| 0.024674 | 0.157487 | 0.131829 | CCL2/ADIPOQ/RELN | 3 |  |  |  |  |  |  |  |  |  |  |
| 0.024779 | 0.157487 | 0.131829 | CCL20/FPR2/SELP/SELE | 4 |  |  |  |  |  |  |  |  |  |  |
| 0.024946 | 0.157487 | 0.131829 | GCKR/ADIPOQ | 2 |  |  |  |  |  |  |  |  |  |  |
| 0.024946 | 0.157487 | 0.131829 | SPP1/IL6 | 2 |  |  |  |  |  |  |  |  |  |  |
| 0.024946 | 0.157487 | 0.131829 | ST14/RSPO3 | 2 |  |  |  |  |  |  |  |  |  |  |
| 0.024946 | 0.157487 | 0.131829 | NRXN1/RELN | 2 |  |  |  |  |  |  |  |  |  |  |
| 0.025175 | 0.158474 | 0.132655 | IL31RA/CCL2/HMOX1/IL1RL1/APOD/ADIPOQ | 6 |  |  |  |  |  |  |  |  |  |  |
| 0.026013 | 0.162807 | 0.136283 | LIF/RSPO3 | 2 |  |  |  |  |  |  |  |  |  |  |
| 0.026013 | 0.162807 | 0.136283 | FOSL1/SOX10 | 2 |  |  |  |  |  |  |  |  |  |  |
| 0.026414 | 0.164368 | 0.137589 | HMOX1/VEGFA/CA9 | 3 |  |  |  |  |  |  |  |  |  |  |
| 0.026414 | 0.164368 | 0.137589 | PROM1/VEGFA/DIO3 | 3 |  |  |  |  |  |  |  |  |  |  |
| 0.026886 | 0.166833 | 0.139653 | MYRIP/LIF/SPP1/IL6/ADIPOQ | 5 |  |  |  |  |  |  |  |  |  |  |
| 0.027099 | 0.167197 | 0.139957 | VEGFA/DIO3 | 2 |  |  |  |  |  |  |  |  |  |  |
| 0.027099 | 0.167197 | 0.139957 | EBF2/ADIPOQ | 2 |  |  |  |  |  |  |  |  |  |  |
| 0.027609 | 0.169867 | 0.142192 | NRXN1/FGF12/PCDH17 | 3 |  |  |  |  |  |  |  |  |  |  |
| 0.027896 | 0.170718 | 0.142904 | CCL2/PALMD/VEGFA/CCL7/RELN | 5 |  |  |  |  |  |  |  |  |  |  |
| 0.028202 | 0.170718 | 0.142904 | FPR2/IL6 | 2 |  |  |  |  |  |  |  |  |  |  |
| 0.028202 | 0.170718 | 0.142904 | SELP/SELE | 2 |  |  |  |  |  |  |  |  |  |  |
| 0.028202 | 0.170718 | 0.142904 | VEGFA/ADIPOQ | 2 |  |  |  |  |  |  |  |  |  |  |
| 0.028202 | 0.170718 | 0.142904 | HMOX1/VEGFA | 2 |  |  |  |  |  |  |  |  |  |  |
| 0.028218 | 0.170718 | 0.142904 | PLP1/CCL2/RAMP3 | 3 |  |  |  |  |  |  |  |  |  |  |
| 0.028583 | 0.172448 | 0.144353 | CCL20/VEGFA/FPR2/SELP/SELE | 5 |  |  |  |  |  |  |  |  |  |  |
| 0.029323 | 0.175454 | 0.146869 | C2CD4A/IL6 | 2 |  |  |  |  |  |  |  |  |  |  |
| 0.029323 | 0.175454 | 0.146869 | SOX10/IL6 | 2 |  |  |  |  |  |  |  |  |  |  |
| 0.029323 | 0.175454 | 0.146869 | TNC/LIF | 2 |  |  |  |  |  |  |  |  |  |  |
| 0.029456 | 0.175769 | 0.147133 | ACTC1/FGF12/SCN4B | 3 |  |  |  |  |  |  |  |  |  |  |
| 0.030346 | 0.180586 | 0.151165 | MYRIP/LIF/SPP1/IL6/ADIPOQ | 5 |  |  |  |  |  |  |  |  |  |  |
| 0.031618 | 0.185614 | 0.155374 | LHX6/RELN | 2 |  |  |  |  |  |  |  |  |  |  |
| 0.031618 | 0.185614 | 0.155374 | GCKR/ADIPOQ | 2 |  |  |  |  |  |  |  |  |  |  |
| 0.031618 | 0.185614 | 0.155374 | VEGFA/SELE | 2 |  |  |  |  |  |  |  |  |  |  |
| 0.031618 | 0.185614 | 0.155374 | ANGPTL4/HMOX1 | 2 |  |  |  |  |  |  |  |  |  |  |
| 0.031618 | 0.185614 | 0.155374 | SOX10/VEGFA | 2 |  |  |  |  |  |  |  |  |  |  |
| 0.032791 | 0.190448 | 0.15942 | CHRDL1/GREM2 | 2 |  |  |  |  |  |  |  |  |  |  |
| 0.032791 | 0.190448 | 0.15942 | CCL2/MARCO | 2 |  |  |  |  |  |  |  |  |  |  |
| 0.032791 | 0.190448 | 0.15942 | SPP1/IL6 | 2 |  |  |  |  |  |  |  |  |  |  |
| 0.032791 | 0.190448 | 0.15942 | RAMP3/FPR2 | 2 |  |  |  |  |  |  |  |  |  |  |
| 0.032928 | 0.190739 | 0.159664 | SPP1/VEGFA/PI16/DIO3/SEMA3G | 5 |  |  |  |  |  |  |  |  |  |  |
| 0.033308 | 0.191918 | 0.160651 | TNC/PROM1/LIF/VEGFA/ADIPOQ | 5 |  |  |  |  |  |  |  |  |  |  |
| 0.033308 | 0.191918 | 0.160651 | CCL2/CCL20/CCL7/ADIPOQ/SELE | 5 |  |  |  |  |  |  |  |  |  |  |
| 0.033406 | 0.191972 | 0.160696 | NRXN1/GRIK3/SCN7A/FGF12/SCN4B/RELN | 6 |  |  |  |  |  |  |  |  |  |  |
| 0.033981 | 0.194445 | 0.162765 | MTTP/GPIHBP1 | 2 |  |  |  |  |  |  |  |  |  |  |
| 0.034014 | 0.194445 | 0.162765 | SLC11A1/SLC39A14/SCARA5 | 3 |  |  |  |  |  |  |  |  |  |  |
| 0.035187 | 0.198032 | 0.165768 | PTX3/VEGFA | 2 |  |  |  |  |  |  |  |  |  |  |
| 0.035187 | 0.198032 | 0.165768 | VEGFA/SELE | 2 |  |  |  |  |  |  |  |  |  |  |
| 0.035187 | 0.198032 | 0.165768 | PLP1/GRIK3 | 2 |  |  |  |  |  |  |  |  |  |  |
| 0.035187 | 0.198032 | 0.165768 | LHX6/RELN | 2 |  |  |  |  |  |  |  |  |  |  |
| 0.035187 | 0.198032 | 0.165768 | IL6/ADIPOQ | 2 |  |  |  |  |  |  |  |  |  |  |
| 0.035187 | 0.198032 | 0.165768 | VEGFA/NEBL | 2 |  |  |  |  |  |  |  |  |  |  |
| 0.035372 | 0.198559 | 0.16621 | CCL2/VEGFA/SELP/IL6/ADIPOQ/SELE | 6 |  |  |  |  |  |  |  |  |  |  |
| 0.03556 | 0.19859 | 0.166236 | HMOX1/APOD/IL6/ADIPOQ | 4 |  |  |  |  |  |  |  |  |  |  |
| 0.03556 | 0.19859 | 0.166236 | SPP1/VEGFA/PI16/SEMA3G | 4 |  |  |  |  |  |  |  |  |  |  |
| 0.035707 | 0.198901 | 0.166496 | LIF/HMOX1/APOD/FGF12/ADRA2B/ADIPOQ | 6 |  |  |  |  |  |  |  |  |  |  |
| 0.036074 | 0.20043 | 0.167776 | THBD/FOSL1/ADIPOQ | 3 |  |  |  |  |  |  |  |  |  |  |
| 0.036411 | 0.200766 | 0.168057 | HMOX1/VEGFA | 2 |  |  |  |  |  |  |  |  |  |  |
| 0.036411 | 0.200766 | 0.168057 | FGF12/SCN4B | 2 |  |  |  |  |  |  |  |  |  |  |
| 0.036411 | 0.200766 | 0.168057 | FGF12/SCN4B | 2 |  |  |  |  |  |  |  |  |  |  |
| 0.036564 | 0.201101 | 0.168337 | HMOX1/APOD/FOSL1/IL6 | 4 |  |  |  |  |  |  |  |  |  |  |
| 0.037072 | 0.20338 | 0.170245 | TNC/SPP1/VEGFA/SEMA3G | 4 |  |  |  |  |  |  |  |  |  |  |
| 0.037256 | 0.203881 | 0.170664 | FOSL1/SOX10/SPP1/IL6/ADIPOQ | 5 |  |  |  |  |  |  |  |  |  |  |
| 0.03765 | 0.205005 | 0.171606 | SOX10/SEMA3G | 2 |  |  |  |  |  |  |  |  |  |  |
| 0.03765 | 0.205005 | 0.171606 | SOX10/VEGFA | 2 |  |  |  |  |  |  |  |  |  |  |
| 0.038196 | 0.206942 | 0.173227 | MTTP/APOD/OLR1 | 3 |  |  |  |  |  |  |  |  |  |  |
| 0.038196 | 0.206942 | 0.173227 | IL1RL1/C2CD4A/IL6 | 3 |  |  |  |  |  |  |  |  |  |  |
| 0.038906 | 0.209744 | 0.175572 | LIF/SPP1 | 2 |  |  |  |  |  |  |  |  |  |  |
| 0.038906 | 0.209744 | 0.175572 | MTTP/GPIHBP1 | 2 |  |  |  |  |  |  |  |  |  |  |
| 0.039646 | 0.210745 | 0.17641 | SCN7A/FGF12/SCN4B | 3 |  |  |  |  |  |  |  |  |  |  |
| 0.039646 | 0.210745 | 0.17641 | HMOX1/FOSL1/IL6 | 3 |  |  |  |  |  |  |  |  |  |  |
| 0.039646 | 0.210745 | 0.17641 | CCL2/HMOX1/CNTFR | 3 |  |  |  |  |  |  |  |  |  |  |
| 0.039646 | 0.210745 | 0.17641 | FOSL1/IL6/ADIPOQ | 3 |  |  |  |  |  |  |  |  |  |  |
| 0.039646 | 0.210745 | 0.17641 | ACTC1/FGF12/SCN4B | 3 |  |  |  |  |  |  |  |  |  |  |
| 0.039672 | 0.210745 | 0.17641 | CCL2/HMOX1/CNTFR/DIO3 | 4 |  |  |  |  |  |  |  |  |  |  |
| 0.040177 | 0.212392 | 0.177789 | APOD/ADIPOQ | 2 |  |  |  |  |  |  |  |  |  |  |
| 0.040177 | 0.212392 | 0.177789 | IL6/ADIPOQ | 2 |  |  |  |  |  |  |  |  |  |  |
| 0.040381 | 0.212953 | 0.178258 | NRXN1/GRIK3/RELN | 3 |  |  |  |  |  |  |  |  |  |  |
| 0.041464 | 0.217088 | 0.18172 | NRXN1/CLSTN2 | 2 |  |  |  |  |  |  |  |  |  |  |
| 0.041464 | 0.217088 | 0.18172 | PROM1/DIO3 | 2 |  |  |  |  |  |  |  |  |  |  |
| 0.041464 | 0.217088 | 0.18172 | ANGPTL4/CCL2 | 2 |  |  |  |  |  |  |  |  |  |  |
| 0.041826 | 0.218457 | 0.182865 | CCL2/SELP/IL6/SELE | 4 |  |  |  |  |  |  |  |  |  |  |
| 0.042766 | 0.222835 | 0.186531 | ACTC1/NEBL | 2 |  |  |  |  |  |  |  |  |  |  |
| 0.04325 | 0.22482 | 0.188192 | SLC11A1/CCL20/FPR2/SELP/IL6 | 5 |  |  |  |  |  |  |  |  |  |  |
| 0.043599 | 0.226091 | 0.189256 | NRXN1/LGI1/SPP1/VEGFA/SEMA3G/RELN | 6 |  |  |  |  |  |  |  |  |  |  |
| 0.044084 | 0.227873 | 0.190747 | LIF/ADIPOQ | 2 |  |  |  |  |  |  |  |  |  |  |
| 0.044152 | 0.227873 | 0.190747 | SERPINA1/SERPINA3/VEGFA/PI16/PI15 | 5 |  |  |  |  |  |  |  |  |  |  |
| 0.04461 | 0.229693 | 0.192271 | MT1X/MT2A/MT1A/MT1G | 4 |  |  |  |  |  |  |  |  |  |  |
| 0.044937 | 0.23083 | 0.193223 | SLC11A1/CXCL5/CXCL14 | 3 |  |  |  |  |  |  |  |  |  |  |
| 0.045064 | 0.23094 | 0.193315 | ANGPTL4/HMOX1/VEGFA/CA9/ADIPOQ | 5 |  |  |  |  |  |  |  |  |  |  |
| 0.045416 | 0.231115 | 0.193461 | ADRA1D/ADRA2B | 2 |  |  |  |  |  |  |  |  |  |  |
| 0.045416 | 0.231115 | 0.193461 | PROM1/LIF | 2 |  |  |  |  |  |  |  |  |  |  |
| 0.045416 | 0.231115 | 0.193461 | LIF/VEGFA | 2 |  |  |  |  |  |  |  |  |  |  |
| 0.04572 | 0.232116 | 0.194299 | PROM1/VEGFA/DIO3 | 3 |  |  |  |  |  |  |  |  |  |  |
| 0.046454 | 0.233945 | 0.195831 | ACTC1/VEGFA/NEBL/SGCG/PI16 | 5 |  |  |  |  |  |  |  |  |  |  |
| 0.04651 | 0.233945 | 0.195831 | THBD/FOSL1/ADIPOQ | 3 |  |  |  |  |  |  |  |  |  |  |
| 0.04651 | 0.233945 | 0.195831 | VEGFA/EBF2/ADIPOQ | 3 |  |  |  |  |  |  |  |  |  |  |
| 0.04651 | 0.233945 | 0.195831 | VEGFA/EBF2/ADIPOQ | 3 |  |  |  |  |  |  |  |  |  |  |
| 0.046764 | 0.234142 | 0.195995 | PROM1/ADIPOQ | 2 |  |  |  |  |  |  |  |  |  |  |
| 0.046764 | 0.234142 | 0.195995 | SPP1/IL6 | 2 |  |  |  |  |  |  |  |  |  |  |
| 0.046923 | 0.234398 | 0.19621 | ACTC1/SCN7A/FGF12/SCN4B/ADRA2B | 5 |  |  |  |  |  |  |  |  |  |  |
| 0.047307 | 0.235237 | 0.196912 | HMOX1/VEGFA/IL6 | 3 |  |  |  |  |  |  |  |  |  |  |
| 0.047307 | 0.235237 | 0.196912 | PROM1/GPM6A/DIO3 | 3 |  |  |  |  |  |  |  |  |  |  |
| 0.04811 | 0.238222 | 0.199411 | HMOX1/IL6/TRIL | 3 |  |  |  |  |  |  |  |  |  |  |
| 0.048126 | 0.238222 | 0.199411 | SOX10/ADIPOQ | 2 |  |  |  |  |  |  |  |  |  |  |
| 0.049502 | 0.242282 | 0.202809 | CCL2/SEMA3G | 2 |  |  |  |  |  |  |  |  |  |  |
| 0.049502 | 0.242282 | 0.202809 | APOD/VEGFA | 2 |  |  |  |  |  |  |  |  |  |  |
| 0.049502 | 0.242282 | 0.202809 | ANGPTL4/CCL2 | 2 |  |  |  |  |  |  |  |  |  |  |
| 0.049502 | 0.242282 | 0.202809 | APOD/VEGFA | 2 |  |  |  |  |  |  |  |  |  |  |
| 0.049502 | 0.242282 | 0.202809 | FGF12/SCN4B | 2 |  |  |  |  |  |  |  |  |  |  |
| 0.049737 | 0.242887 | 0.203316 | TNC/CCL2/FGF12 | 3 |  |  |  |  |  |  |  |  |  |  |
| 6.76E-07 | 0.000122 | 9.83E-05 | SERPINA1/SERPINA3/VEGFA/MMRN1/SELP/VWF/CFD | 7 |  |  |  |  |  |  |  |  |  |  |
| 1.79E-06 | 0.000162 | 0.00013 | SERPINA1/SERPINA3/VEGFA/MMRN1/VWF/CFD | 6 |  |  |  |  |  |  |  |  |  |  |
| 7.03E-05 | 0.002932 | 0.002353 | SERPINA1/HK3/SERPINA3/PTX3/VEGFA/PCSK1/MMRN1/VWF/CFD | 9 |  |  |  |  |  |  |  |  |  |  |
| 7.73E-05 | 0.002932 | 0.002353 | SERPINA1/HK3/SERPINA3/PTX3/VEGFA/PCSK1/MMRN1/VWF/CFD | 9 |  |  |  |  |  |  |  |  |  |  |
| 8.10E-05 | 0.002932 | 0.002353 | SERPINA1/HK3/SERPINA3/PTX3/VEGFA/PCSK1/MMRN1/VWF/CFD | 9 |  |  |  |  |  |  |  |  |  |  |
| 0.000108 | 0.003266 | 0.002621 | IL31RA/GPM6A/NRXN1/GRIK3/FOSL1/PCDH17 | 6 |  |  |  |  |  |  |  |  |  |  |
| 0.00038 | 0.008593 | 0.006897 | PROM1/MTTP/CA9 | 3 |  |  |  |  |  |  |  |  |  |  |
| 0.00038 | 0.008593 | 0.006897 | GRIK3/SCN7A/SCN4B | 3 |  |  |  |  |  |  |  |  |  |  |
| 0.000695 | 0.013969 | 0.011211 | SLC11A1/PTPRB/FPR2/OLR1 | 4 |  |  |  |  |  |  |  |  |  |  |
| 0.000953 | 0.017242 | 0.013838 | IL31RA/GPM6A/NRXN1/GRIK3/CLSTN2/FOSL1/CDH8/PCDH17 | 8 |  |  |  |  |  |  |  |  |  |  |
| 0.001461 | 0.024035 | 0.01929 | SLC11A1/SIGLEC9/PTPRB/FPR2/SELP/CA4/OLR1 | 7 |  |  |  |  |  |  |  |  |  |  |
| 0.001669 | 0.02518 | 0.020209 | IL31RA/THBD/CNTFR/IL1RL1/SELP/SCARA5/CA4/GPIHBP1 | 8 |  |  |  |  |  |  |  |  |  |  |
| 0.002102 | 0.029262 | 0.023484 | SLC11A1/PTX3/PTPRB/FPR2/OLR1 | 5 |  |  |  |  |  |  |  |  |  |  |
| 0.002292 | 0.029628 | 0.023778 | TNC/SERPINA1/ANGPTL4/SERPINA3/MMRN1/VIT/VWF/ADIPOQ | 8 |  |  |  |  |  |  |  |  |  |  |
| 0.002457 | 0.029643 | 0.023791 | CNTFR/LSAMP/NRN1/CA4/GPIHBP1 | 5 |  |  |  |  |  |  |  |  |  |  |
| 0.003533 | 0.03858 | 0.030963 | SLC11A1/SERPINA1/HK3/FPR2/CFD | 5 |  |  |  |  |  |  |  |  |  |  |
| 0.003837 | 0.03858 | 0.030963 | SCN7A/SCN4B | 2 |  |  |  |  |  |  |  |  |  |  |
| 0.003837 | 0.03858 | 0.030963 | LGI1/CDH8 | 2 |  |  |  |  |  |  |  |  |  |  |
| 0.00479 | 0.045628 | 0.036619 | CA4/GPIHBP1 | 2 |  |  |  |  |  |  |  |  |  |  |
| 0.00614 | 0.052958 | 0.042502 | MTTP/ST14/SLC39A14/CA9/GPIHBP1 | 5 |  |  |  |  |  |  |  |  |  |  |
| 0.006765 | 0.052958 | 0.042502 | PROM1/GPM6A/ACTC1/MTTP/CA9 | 5 |  |  |  |  |  |  |  |  |  |  |
| 0.00681 | 0.052958 | 0.042502 | TNC/SERPINA1/MTTP/SPP1/CHRDL1/IL6 | 6 |  |  |  |  |  |  |  |  |  |  |
| 0.006986 | 0.052958 | 0.042502 | CA4/GPIHBP1 | 2 |  |  |  |  |  |  |  |  |  |  |
| 0.007022 | 0.052958 | 0.042502 | GPM6A/GRIK3/LGI1/CLSTN2/CDH8/PCDH17 | 6 |  |  |  |  |  |  |  |  |  |  |
| 0.010388 | 0.075212 | 0.060362 | MTTP/ST14/SLC39A14/CA9/GPIHBP1 | 5 |  |  |  |  |  |  |  |  |  |  |
| 0.011683 | 0.08133 | 0.065272 | PTX3/PTPRB/FPR2/OLR1 | 4 |  |  |  |  |  |  |  |  |  |  |
| 0.013726 | 0.086466 | 0.069394 | PROM1/MTTP/CA9 | 3 |  |  |  |  |  |  |  |  |  |  |
| 0.013726 | 0.086466 | 0.069394 | PTPRB/FPR2/OLR1 | 3 |  |  |  |  |  |  |  |  |  |  |
| 0.013854 | 0.086466 | 0.069394 | MTTP/ST14/SLC39A14/CA9/GPIHBP1 | 5 |  |  |  |  |  |  |  |  |  |  |
| 0.017742 | 0.107041 | 0.085907 | IL31RA/GPM6A/NRXN1/GRIK3/FOSL1/CDH8/PCDH17 | 7 |  |  |  |  |  |  |  |  |  |  |
| 0.030785 | 0.17424 | 0.139838 | PLP1/GRIK3/CNTFR/RAMP3/IL6 | 5 |  |  |  |  |  |  |  |  |  |  |
| 0.030805 | 0.17424 | 0.139838 | SERPINA1/HK3/CFD | 3 |  |  |  |  |  |  |  |  |  |  |
| 0.033386 | 0.183119 | 0.146964 | GPM6A/CLSTN2/PCDH17 | 3 |  |  |  |  |  |  |  |  |  |  |
| 0.034718 | 0.184823 | 0.148331 | SERPINA1/PROM1/CA4 | 3 |  |  |  |  |  |  |  |  |  |  |
| 0.037463 | 0.19025 | 0.152687 | FBXL22/ACTC1/NEBL | 3 |  |  |  |  |  |  |  |  |  |  |
| 0.03784 | 0.19025 | 0.152687 | GPM6A/PCDH17 | 2 |  |  |  |  |  |  |  |  |  |  |
| 0.041601 | 0.195055 | 0.156543 | MTTP/CA4 | 2 |  |  |  |  |  |  |  |  |  |  |
| 0.041784 | 0.195055 | 0.156543 | GPM6A/CLSTN2/PCDH17 | 3 |  |  |  |  |  |  |  |  |  |  |
| 0.042885 | 0.195055 | 0.156543 | CA4/GPIHBP1 | 2 |  |  |  |  |  |  |  |  |  |  |
| 0.044021 | 0.195055 | 0.156543 | PROM1/CNTFR/SLC39A14/CA4/GPIHBP1 | 5 |  |  |  |  |  |  |  |  |  |  |
| 0.044184 | 0.195055 | 0.156543 | SLC11A1/FPR2 | 2 |  |  |  |  |  |  |  |  |  |  |
| 0.046344 | 0.19972 | 0.160287 | ANGPTL4/SERPINA3/ACTC1 | 3 |  |  |  |  |  |  |  |  |  |  |
| 0.048166 | 0.202745 | 0.162715 | GPM6A/PCDH17 | 2 |  |  |  |  |  |  |  |  |  |  |
| 8.58E-08 | 1.53E-05 | 1.32E-05 | CXCL5/CCL2/LIF/CCL20/SPP1/GREM2/VEGFA/CXCL14/IL6/CCL7/ADIPOQ | 11 |  |  |  |  |  |  |  |  |  |  |
| 1.24E-07 | 1.53E-05 | 1.32E-05 | SIGLEC9/SIGLEC10/SELP/ADIPOQ/SELE | 5 |  |  |  |  |  |  |  |  |  |  |
| 3.31E-06 | 0.000233 | 0.000201 | CXCL5/CCL2/LIF/CCL20/SPP1/FGF12/GREM2/VEGFA/CXCL14/SEMA3G/IL6/CCL7/ADIPOQ | 13 |  |  |  |  |  |  |  |  |  |  |
| 3.78E-06 | 0.000233 | 0.000201 | CXCL5/CCL2/LIF/CCL20/SPP1/FGF12/GREM2/VEGFA/CXCL14/SEMA3G/IL6/CCL7/ADIPOQ | 13 |  |  |  |  |  |  |  |  |  |  |
| 7.95E-06 | 0.000393 | 0.000338 | CXCL5/CCL2/CCL20/CXCL14/CCL7 | 5 |  |  |  |  |  |  |  |  |  |  |
| 4.28E-05 | 0.001764 | 0.001518 | CXCL5/CCL2/CCL20/CXCL14/CCL7 | 5 |  |  |  |  |  |  |  |  |  |  |
| 0.000131 | 0.004373 | 0.003764 | SIGLEC9/HK3/SIGLEC10/PTX3/GCKR/SELP/OLR1/SELE | 8 |  |  |  |  |  |  |  |  |  |  |
| 0.000142 | 0.004373 | 0.003764 | CXCL5/CCL2/LIF/CCL20/VEGFA/CXCL14/IL6/CCL7 | 8 |  |  |  |  |  |  |  |  |  |  |
| 0.00084 | 0.023064 | 0.019855 | MARCO/FPR2/SCARA5/OLR1 | 4 |  |  |  |  |  |  |  |  |  |  |
| 0.001226 | 0.030293 | 0.026078 | CXCL5/CCL2/MARCO/CCL20/CXCL14/RSPO3/CCL7 | 7 |  |  |  |  |  |  |  |  |  |  |
| 0.001365 | 0.030659 | 0.026393 | SLC11A1/SLC39A14 | 2 |  |  |  |  |  |  |  |  |  |  |
| 0.001804 | 0.034483 | 0.029685 | GRIK3/SCN7A/SCN4B | 3 |  |  |  |  |  |  |  |  |  |  |
| 0.001815 | 0.034483 | 0.029685 | GREM2/VEGFA/RSPO3/VIT/SELP/CCL7 | 6 |  |  |  |  |  |  |  |  |  |  |
| 0.002193 | 0.03835 | 0.033014 | CCL2/CCL20/CCL7 | 3 |  |  |  |  |  |  |  |  |  |  |
| 0.002329 | 0.03835 | 0.033014 | GREM2/VEGFA/RSPO3/SELP/CCL7 | 5 |  |  |  |  |  |  |  |  |  |  |
| 0.002721 | 0.042004 | 0.03616 | CA9/CA4 | 2 |  |  |  |  |  |  |  |  |  |  |
| 0.003562 | 0.049629 | 0.042723 | SELP/SELE | 2 |  |  |  |  |  |  |  |  |  |  |
| 0.003654 | 0.049629 | 0.042723 | SPP1/OLFML2A/VEGFA | 3 |  |  |  |  |  |  |  |  |  |  |
| 0.003818 | 0.049629 | 0.042723 | SIGLEC9/SIGLEC10/SELP/ADIPOQ/SELE | 5 |  |  |  |  |  |  |  |  |  |  |
| 0.006018 | 0.074324 | 0.063983 | SERPINA1/ANGPTL4/SERPINA3/GCKR/PPP1R1A/PI16/PI15 | 7 |  |  |  |  |  |  |  |  |  |  |
| 0.007267 | 0.078611 | 0.067673 | IL31RA/CNTFR/IL1RL1/GREM2 | 4 |  |  |  |  |  |  |  |  |  |  |
| 0.007267 | 0.078611 | 0.067673 | IL31RA/CNTFR/IL1RL1/FPR2 | 4 |  |  |  |  |  |  |  |  |  |  |
| 0.00732 | 0.078611 | 0.067673 | SCN7A/SCN4B | 2 |  |  |  |  |  |  |  |  |  |  |
| 0.011804 | 0.121487 | 0.104583 | MARCO/RAMP3/FPR2 | 3 |  |  |  |  |  |  |  |  |  |  |
| 0.0132 | 0.130417 | 0.11227 | LIF/FGF12/VEGFA/IL6 | 4 |  |  |  |  |  |  |  |  |  |  |
| 0.013888 | 0.131938 | 0.11358 | SELP/TRIL | 2 |  |  |  |  |  |  |  |  |  |  |
| 0.015503 | 0.138312 | 0.119067 | TNC/MMRN1/VWF/ADIPOQ | 4 |  |  |  |  |  |  |  |  |  |  |
| 0.015679 | 0.138312 | 0.119067 | GREM2/VEGFA/RSPO3/SELP/CCL7 | 5 |  |  |  |  |  |  |  |  |  |  |
| 0.017338 | 0.147668 | 0.127121 | IL31RA/CNTFR/IL1RL1 | 3 |  |  |  |  |  |  |  |  |  |  |
| 0.018313 | 0.150777 | 0.129797 | SLC11A1/SLC39A14 | 2 |  |  |  |  |  |  |  |  |  |  |
| 0.020832 | 0.156682 | 0.13488 | ST14/PCSK1/RELN/CFD | 4 |  |  |  |  |  |  |  |  |  |  |
| 0.021198 | 0.156682 | 0.13488 | SERPINA1/SERPINA3/PI16/PI15 | 4 |  |  |  |  |  |  |  |  |  |  |
| 0.021216 | 0.156682 | 0.13488 | FGF12/SCN4B | 2 |  |  |  |  |  |  |  |  |  |  |
| 0.021567 | 0.156682 | 0.13488 | ST14/PCSK1/RELN/CFD | 4 |  |  |  |  |  |  |  |  |  |  |
| 0.028668 | 0.202317 | 0.174166 | MARCO/SCARA5 | 2 |  |  |  |  |  |  |  |  |  |  |
| 0.032138 | 0.216639 | 0.186495 | PROM1/APOD | 2 |  |  |  |  |  |  |  |  |  |  |
| 0.032678 | 0.216639 | 0.186495 | SLC11A1/GPM6A/GRIK3/SCN7A/SLC39A14/SCN4B | 6 |  |  |  |  |  |  |  |  |  |  |
| 0.033329 | 0.216639 | 0.186495 | ADRA1D/ADRA2B | 2 |  |  |  |  |  |  |  |  |  |  |
| 0.038264 | 0.242337 | 0.208618 | SLC38A5/AQP7 | 2 |  |  |  |  |  |  |  |  |  |  |
| 0.040759 | 0.242632 | 0.208871 | SERPINA1/SERPINA3/PI16/PI15 | 4 |  |  |  |  |  |  |  |  |  |  |
| 0.044796 | 0.242632 | 0.208871 | PROM1/APOD | 2 |  |  |  |  |  |  |  |  |  |  |
| 0.045134 | 0.242632 | 0.208871 | VEGFA/ESM1/IL6 | 3 |  |  |  |  |  |  |  |  |  |  |
| 0.045926 | 0.242632 | 0.208871 | SPP1/ESM1/VWF | 3 |  |  |  |  |  |  |  |  |  |  |
| 0.048343 | 0.242632 | 0.208871 | NRXN1/FGF12/SCN4B | 3 |  |  |  |  |  |  |  |  |  |  |
| 0.049163 | 0.242632 | 0.208871 | GRIK3/SCN7A/SCN4B | 3 |  |  |  |  |  |  |  |  |  |  |
