# Supplementary material for: Identification of senescence-related biomarker for aortic dissection based on bioinformatics and machine learning algorithms
Source: Medicine (Baltimore). 2026 May 29;105(22):e48873. doi: 10.1097/MD.0000000000048873 (PMC13249447; doi:10.1097/MD.0000000000048873)
Supplement: Supplementary file 7 [file medi-105-e48873-s007.docx]

**Supplementary file 7 Table S6.** The results of GSEA analysis.

| core_enrichment |
| --- |
| CALD1/KCNMB1/ROCK2/MYL6/MYH11/ADCY5/MYLK3/CALM2/MYLK/ADCY2/ADCY9/NPR1/MYL9/PRKCH/CALM1/RAMP1/CACNA1D/ACTG2/KCNMA1/PLCB1/PLCB4/KCNMB4/ADRA1B/ADRA1A/PPP1R14A/AVPR1A/AGTR1/ADCY1/ACTA2/RAMP2/PLA2G2A/GUCY1A2/ADCY4/ADRA1D/RAMP3 |
| MYC/ESPL1/GADD45B/CDC45/ORC6/CDC7/CCNE1/CCNE2/ORC1/CDKN1A/CDK2/PTTG1/CHEK2/TTK/BUB1B/MAD2L2/MAD2L1/E2F3/PLK1/MAD1L1/CDC20/GADD45A/CDC25C/E2F2/CDK1/CCNB2/CCNB1/MCM7/CDC25A/E2F1/CCNA1/E2F4/BUB1/CDKN2D/ATR/CDC6/CDKN2A/ABL1/MCM2/MCM4 |
| CXCL5/CCL20/LIF/CCL7/VEGFA/IL6/CCL2/CCL18/CXCL3/PPBP/CLCF1/CXCL1/BMP2/IL1R2/IL1RAP/TNFRSF9/TNFRSF1B/IFNE/TNFRSF10D/HGF/CCR1/RELT/TNFSF8/PRLR/IL1R1/TNFRSF11A/OSMR/EPOR/GDF5/IL4R/IL15RA/CXCL6/IL18R1/CSF3R/TSLP/TNFSF18/FAS/CXCR2/PDGFRA/IL20RB/ACVR1B/IL6R/TNFSF13/CXCR1/IFNGR2/IFNAR1/CXCL16/TNFRSF14/LTBR/PDGFA/TNFRSF1A/CXCR4/IL21R/IL7R/IL22RA1/IL2RA/PLEKHO2/PF4/VEGFC/IL10/IL1B |
| IGFBP3/CD82/RRM2/SERPINE1/GADD45B/GTSE1/CCNE1/CCNE2/CDKN1A/CDK2/BID/CHEK2/STEAP3/FAS/PMAIP1/GADD45A/CDK1/TP53I3/CCNB2/SESN2/CCNB1/BAX/ATR/CDKN2A |
| CCL7/IL6/CCL2/CXCL1/NLRC4/NOD2/CARD6/MAPK13/BIRC3/TNFAIP3/CARD9/PYCARD/MEFV/NFKBIB/IL1B/RELA/NFKBIA/NFKB1/IL18 |
| ITGA1/CACNA2D2/CACNA2D1/CACNB4/TPM2/ITGA9/TNNC1/ADCY5/ADRB1/SGCB/ADCY2/ADCY9/SLC8A1/TGFB2/ITGA8/DMD/ITGB8/RYR2/MYBPC3/CACNA1D/ITGA7/CACNA2D3/SGCD/PLN/TPM1/TTN/ADCY1/IGF1/ITGA6/MYL2/DES/TNNT2/ITGB4/ADCY4/ACTC1/SGCG |
| PRKAA2/MYL3/ITGAV/LAMA2/ITGA1/CACNA2D2/PRKAG2/CACNA2D1/CACNB4/TPM2/ITGA9/TNNC1/SGCB/SLC8A1/TGFB2/ITGA8/DMD/ITGB8/RYR2/MYBPC3/CACNA1D/ITGA7/CACNA2D3/SGCD/TPM1/TTN/IGF1/ITGA6/MYL2/DES/ACE/TNNT2/ITGB4/ACTC1/SGCG |
| LIF/IL6/CLCF1/MYC/IFNE/SOCS3/PIK3R5/PRLR/PIM1/OSMR/EPOR/IL4R/IL15RA/CSF3R/TSLP/JAK3/IL20RB/IL6R/SPRY4/STAT3/IFNGR2/CISH/IFNAR1/PTPN6/IL21R/IL7R/IL22RA1/IL2RA/SPRY2/IL10/CNTF/IFNGR1/IL12RB1/BCL2L1/CBLB/IL11/GHR/IFNAR2/IL10RB/AKT1/IL12A |
| SELL/HLA-DOB/HLA-DMB/NCAM1/ITGA9/CD40LG/NEO1/CADM1/NLGN3/HLA-DQA1/NLGN1/HLA-DOA/HLA-DQA2/CLDN5/NRXN3/PTPRM/CNTN2/CDH2/HLA-DRB1/CD8A/ITGA8/ICOS/ITGB8/HLA-DQB1/NLGN4X/PECAM1/ICAM2/ESAM/CD34/NEGR1/CNTN1/JAM2/ITGA6/MPZ/CDH5/L1CAM/SELE/SELP/NRXN1 |
| VEGFA/IL6/BMP2/MMP1/MYC/ITGA2/MMP9/RARA/HGF/FGF5/CCNE1/RAD51/LAMC2/CKS1B/CCNE2/CDKN1A/PIK3R5/BIRC5/PGF/WNT2/CDK2/BID/DAPK1/EGLN3/PTGS2/HIF1A/BIRC3/TCF7/CSF3R/TRAF1/FAS/TRAF4/PDGFRA/NKX3-1/SPI1/FN1/SLC2A1/LAMB1/BCR/E2F3/RAC2/STAT3/PPARD/RASSF5/E2F2/LAMB3/NFKB2/RUNX1/TRAF3/PDGFA/FZD5/BAX/E2F1/CCNA1/NOS2/DVL1/SUFU/VEGFC |
| HSPA6/HSPA8/SRSF9/SNRPD1/PRPF4/SF3B5/HNRNPA3/THOC3/CHERP/EIF4A3/SF3B3/PQBP1/SNRPD2/HSPA1B/BUD31/SNRPB/LSM7/EFTUD2/U2AF2/CTNNBL1/SNRPF/U2AF1/DDX23/LSM4/DHX38/PRPF31/SNRPC/DDX39B/PCBP1/USP39/RBM8A/LSM2/SF3B4/SNRPA1/DHX16/PRPF19/NCBP2/TCERG1/SF3A1/SRSF4/HNRNPA1/SNRPA/ACIN1/HNRNPM/PRPF38B/SF3A3/HNRNPC/WBP11/SNRPG/PRPF40A/PRPF18/HNRNPU/PUF60/CCDC12/MAGOH/SNRPD3/PPIL1/HNRNPA1L2/MAGOHB/SF3A2/RBM25/SNRNP70/TRA2B/DHX8/XAB2/PRPF8/SRSF2/SRSF3/NCBP1/SRSF1 |
| CACNA1A/BST1/ADRB2/ATP2B2/PLCD1/RYR1/ERBB4/TNNC1/SLC25A4/PTGER3/OXTR/EDNRB/MYLK3/ADRB1/HTR4/TACR2/GRPR/CALM2/CHRM3/MYLK/ADCY2/TRPC1/CAMK2A/ADCY9/SLC8A1/TACR1/CALM1/CAMK2G/RYR2/CACNA1D/GRIN2C/PLCB1/PLCB4/ADRA1B/ADRA1A/AVPR1A/AGTR1/PLN/ADCY1/PTGFR/PDE1C/GRIN2A/NOS3/RYR3/ERBB3/ADCY4/ADRA1D |
| VEGFA/MMP1/MYC/MMP9/CDKN1A/PGF/DAPK1/TYMP/E2F3/E2F2/E2F1/VEGFC/CDKN2A/THBS1 |
| IL6/ANPEP/IL1R2/ITGA2/ITGA5/CD1D/CD33/IL1R1/CD38/EPOR/IL4R/CSF3R/CD14/ITGB3/IL6R/CR1/IL7R/IL2RA/TFRC/IL1B/CD37/CD8B/CSF1/ITGA4/CD59/FLT3LG/IL1A/CD7/IL11/ITGA2B |
| SGCB/DSG2/SLC8A1/CDH2/ITGA8/DMD/CTNNA3/ITGB8/RYR2/CACNA1D/ITGA7/ACTN4/ACTN2/CACNA2D3/SGCD/DSP/ITGA6/DES/ITGB4/SGCG |
| PNP/RRM2/UPP1/POLE2/TYMP/TK1/POLR2L/CANT1/NME1/POLR1C/UCK2/POLR2D/POLR2I/POLR2H/TYMS/POLD2/POLE3/POLE4/POLD1/POLR3A/TXNRD2/POLR3D/PRIM2/DPYD/UMPS/POLR3K/NME3/POLR2A/POLD3/POLR3C/POLR1E/ITPA/NME2/NME5/POLR1A/UCK1/DHODH/NT5E/POLR1B/PNPT1/NT5C/POLR3F/RRM1/POLD4/POLR3H/POLR3B |
| IL1RAP/TNFRSF10D/PIK3R5/BID/IL1R1/IRAK2/BIRC3/FAS/IRAK1/MYD88/TNFRSF1A/BAX/TRADD/IL1B/RELA/FADD/NFKBIA/NFKB1/BCL2L1/IL1A/CFLAR/AKT1/IRAK3/IRAK4/CASP8/CASP3/RIPK1/TNFRSF10A/BIRC2/NGF/CSF2RB/TNF/TNFRSF10C |
| IL6/IKBKE/POLR1C/PYCARD/RIPK3/IL33/NFKBIB/IL1B/RELA/NFKBIA/NFKB1/IL18/POLR3A/POLR3D/POLR3K/IRF7/POLR3C/RIPK1 |
| MYC/EIF4EBP1/RARA/PIK3R5/PIM1/TCF7/SPI1/STAT3/PPARD/RUNX1/CCNA1/RELA/NFKB1/PML/AKT1/ARAF/MAP2K1 |
| ATP1B1/MYL3/SLC9A6/CACNA2D2/CACNA2D1/CACNB4/TPM2/COX4I2/TNNC1/FXYD2/SLC8A1/ATP1A2/RYR2/CACNA1D/CACNA2D3/TPM1/ATP1B2/MYL2/TNNT2/ACTC1 |
| LHCGR/SSTR5/GRIK1/THRA/NR3C1/CALCRL/GLRB/HTR2A/UTS2R/GRIA3/CHRNE/ADRB2/SSTR2/ADRA2C/LEPR/PTGER3/OXTR/CTSG/EDNRB/P2RY8/VIPR1/ADRB1/HTR4/GZMA/TACR2/GRPR/CHRM3/VIPR2/GABRA2/TACR1/GABRA4/AVPR2/CNR1/NMUR1/LPAR4/P2RY13/HTR1F/GRIN2C/GRIA2/ADRA1B/THRB/ADRA1A/AVPR1A/GRIA1/AGTR1/OPRK1/APLNR/GABRB1/ADRA2A/NPY5R/PTGFR/NPY1R/ADCYAP1R1/GRIN2A/ADRA2B/GRIK3/P2RY14/ADRA1D |
| SPP1/IL6/TLR2/LBP/IKBKE/PIK3R5/MAPK13/IRAK1/CD14/MYD88/IFNAR1/TRAF3/TLR5/IL1B/CD80/RELA/CD86/FADD/NFKBIA/NFKB1/TLR1/IFNAR2/MAP2K3/CD40/AKT1/IL12A/IRAK4/CASP8/MAP2K1/IRF7 |
| PSMB10/PSME2/PSMB3/PSMB8/PSMD11/PSMC4/PSMC5/PSMD4/PSMD12/PSMD2/PSMC2/POMP/PSMA6/PSME3/PSMD14/PSMC3/PSMD3/PSME4/PSMB9/PSMD8/PSME1/PSMB7/PSMB5/PSMA7/PSMD13 |
| MYC/ITGA2/CCNE1/LAMC2/CKS1B/CCNE2/PIK3R5/CDK2/PTGS2/BIRC3/TRAF1/TRAF4/FN1/LAMB1/E2F3/E2F2/LAMB3/TRAF3/E2F1/NOS2/RELA/NFKBIA/NFKB1 |
| WNT5B/CCND1/DAAM1/DAAM2/LRP6/VANGL1/SMAD2/WNT9A/FZD8/SKP1/PPP2CB/NKD2/ROCK2/BTRC/FZD2/FZD1/JUN/WNT2B/TBL1X/FZD4/PPP2R5A/SFRP5/CAMK2A/FZD6/WNT3/PRICKLE1/FZD7/CER1/SFRP1/CAMK2G/LRP5/DKK1/CXXC4/PLCB1/PLCB4/WNT5A/SOX17/NKD1/WNT11/VANGL2/SFRP4/DKK2/SFRP2 |
| HK3/HK2/NANS/NPL/UGDH/UAP1/RENBP/GMPPB/GALE/GALK1/GNPNAT1 |
