# Supplementary material for: Identification of senescence-related biomarker for aortic dissection based on bioinformatics and machine learning algorithms
Source: Medicine (Baltimore). 2026 May 29;105(22):e48873. doi: 10.1097/MD.0000000000048873 (PMC13249447; doi:10.1097/MD.0000000000048873)
Supplement: Supplementary file 8 [file medi-105-e48873-s008.docx]

**Supplementary file 8 Table S7.** The results of CIBERSORT.

| T cells CD4 memory resting | T cells CD4 memory activated | T cells follicular helper | T cells regulatory (Tregs) | T cells gamma delta | NK cells resting | NK cells activated | Monocytes | Macrophages M0 | Macrophages M1 | Macrophages M2 | Dendritic cells resting | Dendritic cells activated | Mast cells resting | Mast cells activated | Eosinophils | Neutrophils | P-value | Correlation | RMSE |
| --- | --- | --- | --- | --- | --- | --- | --- | --- | --- | --- | --- | --- | --- | --- | --- | --- | --- | --- | --- |
| 0.3169899 | 0.0091354 | 0 | 0 | 0.0033343 | 0 | 0.011714 | 0.0111537 | 0 | 0.033075 | 0.3178777 | 0 | 0 | 0.142624 | 0 | 0 | 0.0300991 | 0.91 | -0.017523 | 1.0891221 |
| 0.2852588 | 0.0130544 | 0 | 0 | 0 | 0 | 0.0090842 | 0.1003777 | 0 | 0.0101267 | 0.206789 | 0 | 0 | 0.0600111 | 0 | 0.0291958 | 0 | 0.99 | -0.04571 | 1.0982992 |
| 0.2792547 | 0.0184098 | 0 | 0 | 0 | 0.0238438 | 0.0149951 | 0.0470784 | 0 | 0 | 0.2498847 | 0 | 0.0123086 | 0.1519123 | 0 | 0 | 0 | 0.89 | -0.011324 | 1.0835466 |
| 0.2805342 | 0.0117984 | 0 | 0 | 0 | 0 | 0.0421591 | 0.0595429 | 0 | 0 | 0.2078653 | 0 | 1.55E-05 | 0.0826439 | 0 | 0.0444148 | 0 | 0.95 | -0.034009 | 1.0943631 |
| 0.2405783 | 0.0164354 | 0 | 0.0088032 | 0 | 0 | 0 | 0.0963504 | 0 | 0.0769635 | 0.2598714 | 0.0386989 | 0 | 0.0500432 | 0 | 0.0299316 | 0 | 0.32 | 0.0733493 | 1.0511517 |
| 0.4272916 | 0 | 0 | 0 | 0 | 0.0213431 | 0 | 0.0215254 | 0.0194769 | 0.0870112 | 0.115779 | 0 | 0 | 0.0289996 | 0 | 0.0085479 | 0 | 0.77 | 0.0044619 | 1.1006273 |
| 0.2043783 | 0 | 0 | 0.0457101 | 0 | 0 | 0.1062707 | 0.0732042 | 0 | 0.0082668 | 0.3353179 | 0 | 0.0378053 | 0.0472591 | 0 | 0.0214588 | 0.0004011 | 0.94 | -0.023358 | 1.0958101 |
| 0.2648443 | 0.0526089 | 0 | 0 | 0 | 0 | 0.0053115 | 0.0891635 | 0 | 0.0172027 | 0.2198762 | 0 | 0 | 0.051442 | 0 | 0 | 0.0726912 | 0.49 | 0.0428088 | 1.0630688 |
| 0.4309719 | 0 | 0 | 0 | 0 | 0.0234488 | 0.0268699 | 0.0835649 | 0 | 0.0322522 | 0.2022773 | 0 | 0 | 0.0069736 | 0 | 0.0020029 | 0 | 0.94 | -0.026013 | 1.1062305 |
| 0.3334638 | 0.0308557 | 0 | 0 | 0.0686144 | 0 | 0.0155467 | 0.0543103 | 0 | 0.0132769 | 0.185086 | 0 | 0.0071446 | 0.0952646 | 0 | 0.0371378 | 0 | 0.35 | 0.0666617 | 1.0548688 |
| 0.4352994 | 0 | 0 | 0 | 0 | 0 | 0.0409342 | 0.0343251 | 0 | 0.016239 | 0.1860046 | 0 | 0 | 0.0349861 | 0 | 0.0178281 | 0 | 0.52 | 0.0381499 | 1.0819772 |
| 0.3045917 | 0.077482 | 0 | 0 | 0 | 0 | 0 | 0.0584999 | 0 | 0.038441 | 0.2849319 | 0 | 0 | 0.027689 | 0 | 0 | 0 | 0.92 | -0.018825 | 1.0899829 |
| 0.2805038 | 0 | 0 | 0 | 0.0367845 | 0 | 0 | 0 | 0 | 0.0779081 | 0.1794136 | 0 | 0 | 0.107229 | 0 | 0 | 0 | 0.49 | 0.0436977 | 1.0703361 |
| 0.1339166 | 0 | 0.008927 | 0.0134906 | 0 | 0 | 0.03484 | 0.0503488 | 0 | 0.0046919 | 0.3946566 | 0.0507184 | 0 | 0.1201385 | 0 | 0.0053315 | 0 | 0.86 | -0.00492 | 1.091734 |
| 0.291753 | 0 | 0 | 0 | 0 | 0.0375565 | 0 | 0.0424064 | 0 | 0.0330993 | 0.1363982 | 0 | 0.0001993 | 0.1050101 | 0 | 0 | 0 | 0.89 | -0.010469 | 1.1052745 |
| 0.2435349 | 0.0289326 | 0 | 0.0890944 | 0 | 0.0095247 | 0.0034249 | 0.0096025 | 0 | 0 | 0.2066415 | 0 | 0 | 0.0965802 | 0 | 0 | 0 | 0.75 | 0.0067789 | 1.0830914 |
| 0.2534549 | 0 | 0 | 0.0363244 | 0 | 0 | 0.0186995 | 0.3950058 | 0 | 0 | 0.0720136 | 0 | 0 | 0.1046986 | 0 | 0 | 0 | 0.46 | 0.0505687 | 1.0814192 |
| 0.2308755 | 0.0380591 | 0 | 0 | 0.0817516 | 0 | 0.0072252 | 0.021642 | 0 | 0.0109142 | 0.1521213 | 0.082778 | 0 | 0.0371992 | 0 | 0.0536596 | 0.1446276 | 0.28 | 0.0837927 | 1.0464378 |
| 0.2949976 | 0 | 0 | 0.0322553 | 0 | 0.0496308 | 0 | 0.0570788 | 0.0137341 | 0 | 0.1173117 | 0 | 0.02417 | 0.0685474 | 0 | 0 | 0.1857903 | 0.68 | 0.018365 | 1.0855053 |
| 0.1954221 | 0.0161793 | 0 | 0.0445855 | 0 | 0.0012038 | 0 | 0 | 0.1096113 | 0.0354418 | 0.3128497 | 0 | 0 | 0 | 0 | 0 | 0.140507 | 0.12 | 0.1259691 | 1.0499076 |
| 0.2074051 | 0.023619 | 0 | 0.0487731 | 0 | 0.0134536 | 0 | 0.0565326 | 0 | 0.0519902 | 0.2678602 | 0.0282338 | 0 | 0.0738525 | 0 | 0 | 0.0179742 | 0.29 | 0.0786157 | 1.0416163 |
| 0.2030133 | 0.0211696 | 0 | 0.0072145 | 0 | 0.0415546 | 0.0191728 | 0.2562094 | 0.0216768 | 0.0050415 | 0.2369573 | 0 | 0 | 0.0727674 | 0 | 0 | 0.036417 | 0.36 | 0.0632726 | 1.0653155 |
| 0.3219209 | 0.0382343 | 0 | 0 | 0 | 0.0052852 | 0 | 0.2700491 | 0 | 0.0579902 | 0.1896825 | 0.0414617 | 0 | 0.0232336 | 0 | 0.0011647 | 0.0203986 | 0.02 | 0.2140891 | 1.0020499 |
| 0.2098752 | 0 | 0 | 0.0335841 | 0 | 0 | 0.0570567 | 0.0996432 | 0.0332731 | 0.0164976 | 0.2069461 | 0 | 0 | 0.0592111 | 0 | 0.0550083 | 0 | 0.91 | -0.017006 | 1.0786259 |
| 0.324956 | 0.0744124 | 0 | 0.0438708 | 0 | 0 | 0 | 0.0702022 | 0 | 0 | 0.2138568 | 0 | 0.0133665 | 0.0127671 | 0 | 0 | 0 | 0.78 | 0.0021938 | 1.0877854 |
| 0.2616679 | 0.0251925 | 0 | 0 | 0 | 0.0592618 | 0 | 0.1514263 | 0 | 0 | 0.3649528 | 0 | 0.0104998 | 0.0776542 | 0 | 0 | 0.02303 | 0.13 | 0.113513 | 1.0448255 |
| 0.2520237 | 0.0365597 | 0 | 0 | 0 | 0 | 0.0248577 | 0.2398277 | 0.0575295 | 0.0303872 | 0.0381942 | 0.0451754 | 0 | 0.0942533 | 0 | 0 | 0.0246393 | 0.49 | 0.0444556 | 1.0604376 |
| 0.3311836 | 0.0166728 | 0 | 0 | 0 | 0 | 0.01442 | 0.0623401 | 0.0231675 | 0.0488453 | 0.1571029 | 0 | 0 | 0.0082636 | 0 | 0.046279 | 0.0271617 | 0.94 | -0.022827 | 1.0813553 |
| 0.2170904 | 0 | 0 | 0.0934711 | 0 | 0.0084471 | 0.0351187 | 0.1298254 | 0.0095434 | 0.004675 | 0.0933102 | 0 | 0 | 0.1754366 | 0 | 0 | 0.0653367 | 0.64 | 0.0252994 | 1.0760006 |
| 0.3140625 | 0.0156616 | 0 | 0 | 0 | 0 | 0.0357074 | 0.1357655 | 0.2128352 | 0 | 0.1074782 | 0 | 0.0056643 | 0 | 0 | 0 | 0.0679803 | 0.69 | 0.0155314 | 1.0952061 |
| 0.3065013 | 0.0122706 | 0 | 0.0130102 | 0 | 0.0315109 | 0 | 0.2025262 | 0.094036 | 0.0226975 | 0.1796346 | 0 | 0 | 0.0258014 | 0 | 0.0018393 | 0.0177557 | 0.53 | 0.0367294 | 1.0726505 |
| 0.3433022 | 0.0314426 | 0 | 0.029907 | 0 | 0.1085074 | 0 | 0.106853 | 0.0632841 | 0 | 0.2232753 | 0 | 0 | 0.0313473 | 0 | 0 | 0.0579731 | 0.2 | 0.1013357 | 1.052315 |
| 0.2239504 | 0.0088314 | 0 | 0 | 0 | 0.0487345 | 0 | 0 | 0 | 0.0093334 | 0.4233416 | 0 | 0 | 0 | 0.0080474 | 0.066361 | 0.2052598 | 0.37 | 0.0621117 | 1.0941525 |
| 0.2128984 | 0 | 0 | 0 | 0.0132702 | 0 | 0.0234002 | 0.1207935 | 0.0754809 | 0.0363877 | 0.2019105 | 0.0263578 | 0 | 0.1619498 | 0 | 0.0089451 | 0.015951 | 0.28 | 0.0828227 | 1.0427774 |
| 0.2977753 | 0.0014639 | 0 | 0.0214496 | 0 | 0.013894 | 0 | 0.1776717 | 0 | 0.0388845 | 0.1962615 | 0 | 0.0023658 | 0.0413352 | 0 | 0 | 0.0558926 | 0.75 | 0.0064223 | 1.082581 |
| 0.2498784 | 0.0722243 | 0 | 0.0111151 | 0 | 0.0453753 | 0 | 0.0968301 | 0.1274655 | 0.0855264 | 0.1062577 | 0.1080399 | 0 | 0.0030407 | 0 | 0 | 0.0304037 | 0.23 | 0.0921557 | 1.0457129 |
| 0.3709738 | 0 | 0 | 0 | 0 | 0.0112385 | 0.0152403 | 0.1653872 | 0 | 0.0312308 | 0.3209952 | 0 | 0 | 0 | 0 | 0 | 0 | 0.84 | -0.003069 | 1.0973432 |
| 0.4257463 | 0 | 0 | 0 | 0 | 0.0295677 | 0.0035934 | 0.0233836 | 0 | 0 | 0.3596188 | 0 | 0 | 0.0294348 | 0 | 0 | 0 | 0.73 | 0.0113043 | 1.0938419 |
| 0.3628174 | 0.0323714 | 0 | 0 | 0 | 0.0605026 | 0 | 0.0191067 | 0 | 0.0353292 | 0.2363231 | 0 | 0 | 0 | 0 | 0.0272917 | 0.0933866 | 0.29 | 0.0800582 | 1.0531335 |
