# Supplementary material for: Identification of senescence-related biomarker for aortic dissection based on bioinformatics and machine learning algorithms
Source: Medicine (Baltimore). 2026 May 29;105(22):e48873. doi: 10.1097/MD.0000000000048873 (PMC13249447; doi:10.1097/MD.0000000000048873)
Supplement: Supplementary file 9 [file medi-105-e48873-s009.docx]

**Supplementary file 9 Table S8.** The correlation of HK3 and immune cells in integrated dataset and validation dataset.

| integrated dataset | | | |  | validation dataset | | | |
| --- | --- | --- | --- | --- | --- | --- | --- | --- |
| Gene | Cell | cor | pvalue |  | Gene | Cell | cor | pvalue |
| HK3 | B cells naive | -0.592236 | 7.14E-05 |  | HK3 | B cells naive | -0.413315 | 0.181717 |
| HK3 | B cells memory | -0.172974 | 0.292323 |  | HK3 | B cells memory | 0.480384 | 0.113937 |
| HK3 | Plasma cells | 0.056432 | 0.732936 |  | HK3 | Plasma cells | -0.491274 | 0.104808 |
| HK3 | T cells CD8 | -0.055664 | 0.736438 |  | HK3 | T cells CD8 | -0.51049 | 0.093605 |
| HK3 | T cells CD4 naive | -0.057658 | 0.727353 |  | HK3 | T cells CD4 naive | -0.393042 | 0.206256 |
| HK3 | T cells CD4 memory resting | -0.036135 | 0.827122 |  | HK3 | T cells CD4 memory resting | -0.258741 | 0.416938 |
| HK3 | T cells CD4 memory activated | 0.270381 | 0.09596 |  | HK3 | T cells CD4 memory activated | 0.276192 | 0.384863 |
| HK3 | T cells follicular helper | -0.266668 | 0.100792 |  | HK3 | T cells follicular helper | -0.561662 | 0.05738 |
| HK3 | T cells regulatory (Tregs) | 0.00227 | 0.989056 |  | HK3 | T cells regulatory (Tregs) | -0.236525 | 0.459216 |
| HK3 | T cells gamma delta | -0.08136 | 0.62245 |  | HK3 | T cells gamma delta | -0.50758 | 0.092071 |
| HK3 | NK cells resting | 0.29961 | 0.06388 |  | HK3 | NK cells resting | 0.767076 | 0.003596 |
| HK3 | NK cells activated | -0.216322 | 0.185934 |  | HK3 | NK cells activated | -0.280728 | 0.376781 |
| HK3 | Monocytes | 0.401417 | 0.011315 |  | HK3 | Monocytes | 0.58042 | 0.052086 |
| HK3 | Macrophages M0 | 0.311857 | 0.053275 |  | HK3 | Macrophages M0 | -0.027537 | 0.932302 |
| HK3 | Macrophages M1 | 0.089929 | 0.586141 |  | HK3 | Macrophages M1 | -0.591945 | 0.04259 |
| HK3 | Macrophages M2 | -0.00081 | 0.996096 |  | HK3 | Macrophages M2 | -0.125874 | 0.699712 |
| HK3 | Dendritic cells resting | -0.050346 | 0.760841 |  | HK3 | Dendritic cells resting | -0.276192 | 0.384863 |
| HK3 | Dendritic cells activated | -0.103131 | 0.532124 |  | HK3 | Dendritic cells activated | 0.124768 | 0.699238 |
| HK3 | Mast cells resting | -0.267491 | 0.099705 |  | HK3 | Mast cells resting | -0.629371 | 0.032395 |
| HK3 | Mast cells activated | 0.172974 | 0.292323 |  | HK3 | Mast cells activated | -0.393042 | 0.206256 |
| HK3 | Eosinophils | -0.169602 | 0.301975 |  | HK3 | Eosinophils | -0.393042 | 0.206256 |
| HK3 | Neutrophils | 0.483878 | 0.001803 |  | HK3 | Neutrophils | 0.387962 | 0.212706 |
| HK3 | P-value | -0.482629 | 0.00186 |  | HK3 | P-value | -0.523215 | 0.08088 |
| HK3 | Correlation | 0.490916 | 0.001508 |  | HK3 | Correlation | 0.566434 | 0.059034 |
| HK3 | RMSE | -0.462372 | 0.003041 |  | HK3 | RMSE | -0.062937 | 0.851682 |
