# Supplementary material for: Identification of senescence-related biomarker for aortic dissection based on bioinformatics and machine learning algorithms
Source: Medicine (Baltimore). 2026 May 29;105(22):e48873. doi: 10.1097/MD.0000000000048873 (PMC13249447; doi:10.1097/MD.0000000000048873)
Supplement: Supplementary file 4 [file medi-105-e48873-s004.pdf]

Supplementary Table S3. Expression profile of integrated dataset.

geneNames	A00710	A00711	A00717	A00720	GA1	Normol3	X262708	X262982	Normal1	Normal10	Normal2	Normal3	Normal4
TSPAN6	3.56772573	4.03040564	3.22193766	4.38585946	4.56559299	4.55678049	4.00912262	4.60884663	4.55819747	4.25103628	4.65584385	5.12040318	4.13221731
DPM1	5.01420057	5.52600999	4.55218943	5.32549365	5.35191685	6.58752593	5.76769667	5.6661199	5.66716985	4.93144268	5.40243214	5.36382967	5.09170421
SCYL3	4.75689659	4.67822101	4.09729169	4.5435392	4.92831493	5.02905907	4.94476053	4.93109665	4.24198	3.8863917	4.04294091	4.65226598	4.91918097
C1orf112	3.11877258	3.47876335	2.8625806	4.54578032	3.47640173	3.5544154	3.68746304	3.67752086	3.93605282	3.00725231	2.45379167	3.71164739	3.93541059
FGR	4.50004098	3.96248275	4.71797529	3.99253609	2.15647166	2.54567412	2.2110849	2.59655866	2.54677536	1.6164697	3.0350896	1.9776778	3.52190866
CFH	8.14667575	9.01832977	6.08224989	8.29305355	7.24369465	6.46880903	7.78957291	8.43003225	7.84596957	6.71817101	7.93330971	6.80914743	8.0234006
FUCA2	5.12358743	5.48390151	4.73411404	6.06484185	5.05872492	5.24809511	4.9801345	5.62058506	5.21265577	5.03452728	5.56824897	5.57740917	5.33163556
GCLC	4.19856198	4.84717549	4.16335927	5.03401569	4.83124597	5.05349781	5.08725665	5.02999338	5.01021071	4.36773788	4.26716491	5.13084237	4.58248716
NFYA	6.16965144	5.58585162	5.38577818	6.22827218	5.77012922	6.24607866	5.81352531	5.4322062	5.29580484	5.29522534	5.60848376	5.50086423	5.54102193
NIPAL3	5.01706458	5.42437218	5.09862552	4.48090849	4.68507646	4.62876727	5.49187394	5.00149453	5.10102497	4.90855238	5.39938318	4.79538263	5.74996905
LAS1L	5.66009496	5.51262825	5.73094958	4.88375792	5.34749288	5.12604711	5.03580514	5.07359379	4.95500105	5.74006684	5.33580938	5.5602769	5.39451739
ENPP4	5.0621045	5.79843426	5.01525147	5.32790631	6.59987744	7.04330594	6.3816627	6.36020806	6.20662819	5.03521895	6.28020126	6.20839714	5.22033334
SEMA3F	1.14028398	1.42609576	1.34897673	0.87153483	0.97933365	1.6025772	2.48855449	0.31615598	0.29672701	0.29672701	3.86378375	0.29672701	2.04678423
CFTR	0.01995973	1.38617089	0.02215683	0.23372788	0.034078	0.12493735	0.01995973	0.09357936	-0.1075483	-0.1075483	-0.1075483	2.11720072	-0.1075483
ANKIB1	6.2531581	6.46383465	5.98054194	6.71255643	6.38029657	6.8909008	6.5500768	6.62954878	6.68165606	6.80471355	6.53456529	6.68104315	6.44365666
CYP51A1	5.34390315	5.47553815	6.37886824	5.53964029	5.56009667	5.63558861	5.90204784	5.79067653	5.8214212	4.515188	5.56481483	5.61815618	4.76470191
KRIT1	3.95750241	4.47487793	3.82689521	5.09411997	5.17275438	5.08359223	5.08945464	4.97572562	4.59889868	3.43213332	4.43474597	4.91125462	4.27240049
RAD52	4.47325518	4.42415726	4.1816492	3.91569521	4.64367326	4.01828876	5.59867389	4.71972712	4.78342137	4.91771968	3.85614772	4.36258793	4.58164999
BAD	4.43090655	4.60307157	5.05611667	3.86237984	3.66780114	4.25283949	3.4482597	3.72931577	4.56004622	4.25331041	4.17377918	4.58743569	2.54653366
LAP3	6.30651152	6.3806872	6.36385066	6.59210037	5.32350648	5.63002116	5.89726853	5.061867	6.09163053	5.9609868	5.74803698	5.28554637	6.79782899
CD99	5.47108576	5.64073503	6.11039269	6.01532952	5.65410586	5.65410586	5.60033385	6.1714002	6.11420583	5.72368926	5.84756624	5.62266232	6.42491618
HS3ST1	1.70132942	2.16241043	3.30467975	2.45929619	1.69433376	1.131639	1.75029855	1.73118839	2.90107905	2.23153413	1.41006384	2.75228562	0.56025693
HECW1	0.39798577	-0.22720274	0.5859803	-0.12989353	0.2983218	0.36925733	0.34070215	-0.0643809	3.71158005	-0.05109809	-0.05109809	-0.05109809	-0.05109809
MAD1L1	2.90908325	2.0454026	3.70383632	2.03223127	1.11022729	1.2207418	2.18259406	1.10974312	1.52000242	-0.2066987	4.01171827	3.51837244	-0.2066987
LASP1	5.57667848	5.87727523	5.96302915	6.24607519	5.8385515	4.96206997	5.29729465	5.08161971	5.88722472	5.05490931	5.45990292	5.39515561	6.49163161
SNX11	4.13392151	4.41665257	4.73287902	4.23698993	3.90011696	3.92149511	4.00274427	3.27215193	4.03126122	3.78807676	4.0063101	4.06574971	3.45823063
TMEM176A	4.31786724	5.36421027	3.15344505	4.63103521	2.81790249	1.9591441	3.48466858	1.6003948	4.31521508	3.1186634	4.13349579	3.9982814	4.21517222
M6PR	6.30705551	6.46737722	6.09070802	6.70065929	6.0219135	6.09830117	6.18141589	5.30969956	5.84459877	6.3212379	6.08253489	5.75960427	6.51100902
KLHL13	4.79236216	3.30783089	3.77023423	3.91668385	4.39191159	4.78051723	4.42212096	4.61694034	4.14563947	4.46669004	3.85474694	4.7663781	4.06763077
CYP26B1	5.10053242	3.40711364	4.76579941	2.03715452	4.55356093	4.93802275	3.32660935	4.26366223	3.80583702	5.85640963	4.60451243	4.7691731	4.38165937
ICA1	1.51095359	3.7214921	2.70912005	3.79420899	3.82399388	2.17383549	2.60056114	3.24900491	3.87754145	3.55014575	4.16821514	2.47325213	5.78389592
DBNDD1	2.9369334	1.75038648	3.03470356	1.29050972	3.3569596	3.09733855	2.88943448	4.66745692	3.41433694	4.15993345	3.2262446	3.80636251	3.58200997
ALS2	5.5091302	5.71527243	5.04720183	5.6715903	5.8418204	5.94485809	5.89895681	6.03766528	6.10336334	5.14078767	5.97978865	5.25638623	6.28189274
CASP10	3.37482265	4.40666649	3.04947339	4.59138734	3.70269124	2.35781291	3.358717	3.25675316	4.52380842	2.18179543	5.03615059	3.46471202	4.52186925
CFLAR	4.37634892	4.55756673	4.27310235	4.29483778	4.5608262	3.93528814	4.43777619	4.65766743	3.58242686	3.70574347	4.67565537	3.91533768	4.26707953
TFPI	6.48782079	6.34606434	6.7383742	7.85160254	6.87232689	4.83566658	6.50051579	5.92527938	5.86215444	4.43621571	6.57745325	5.91282358	5.61521996
RBM5	5.66128768	5.88011101	5.49337026	6.15285716	6.11259253	5.69435879	6.30672641	6.46664589	6.05072844	5.77128559	5.96109357	5.85521218	6.2996766
MTMR7	0.91832997	1.66683423	0.90298992	0.99785556	0.90040832	1.15030299	0.97979889	1.50312037	3.97761317	-0.54803737	3.76051945	2.88097175	-0.54803737
SLC7A2	4.61264898	7.14848658	4.32092892	5.53261035	5.64975069	5.80876652	5.19282595	5.71329199	5.81283977	4.29520786	5.62319717	5.1774189	6.30956817
ARF5	5.62099516	5.64777739	6.11679131	5.83799466	5.60690421	5.10397182	4.97009176	5.82370223	5.35070763	6.68257765	5.88955672	6.41191078	5.69917228
SARM1	5.53714629	5.05721375	5.49448989	4.36638122	4.97916466	4.5759212	4.78523024	4.71963027	4.54894822	4.6357202	5.37853468	5.27417272	4.53997927
POLDIP2	5.58316391	5.65059599	6.05756889	5.89573988	5.37984459	5.09473642	5.61231878	5.60435239	5.31543355	5.82816371	5.30073622	5.84390523	5.68582254
PLXND1	6.40859847	6.50687876	6.73243668	6.81864558	6.02158986	5.52129192	6.30355748	5.68827281	5.8847305	6.48392825	6.96399964	6.03987755	6.20341977
AK2	5.70768495	6.2429705	6.35589944	6.70935833	6.12506973	5.79783984	6.11098971	5.87626512	5.94595534	6.03764407	5.84604311	5.85592414	5.91193342
CD38	3.29797133	3.18690942	1.23600796	3.78215059	2.68892485	2.43801715	1.73642915	2.17476518	2.4133807	-1.18661537	2.92850513	2.22681953	3.9447831

FKBP4	6.24879027	5.35761674	6.21413856	5.59240425	6.0302785	6.07035662	6.24403173	4.95220474	5.25896873	5.90484693	5.47257443	5.8160268	5.26894466
KDM1A	4.49956325	4.82602925	4.20514794	5.1622648	4.07790399	4.72958704	4.36952467	4.49673101	5.06478897	5.30382255	3.81108112	3.97363734	4.82696301
RBM6	6.33854376	6.40301989	6.16794313	6.38996925	6.99181503	6.16677357	6.97623493	7.24423663	6.85435711	6.52645868	6.77422506	6.38589725	7.23730829
CAMKK1	4.85648848	4.2450216	4.64629324	3.2023985	3.28883406	2.88990763	3.59159929	3.74208695	3.06856493	4.65404475	4.29191062	5.10433307	4.47378994
RECQL	5.32330787	6.21827136	5.96631624	6.94251432	6.34072295	6.67937112	6.46413024	6.53997695	6.65598662	6.50145434	6.34121863	6.53686448	6.6865843
HSPB6	6.27997111	6.50356428	7.73537063	5.6580229	7.13498254	5.94019412	5.42391333	4.97316347	6.67872133	7.48180421	5.92183034	5.91603513	8.01243236
ARHGAP33	3.46690784	1.9219265	4.2737428	1.11348558	2.73561296	1.37625973	2.91169156	2.65942673	3.51170007	3.59515923	3.44845549	3.45888245	-0.79202494
NDUFAB1	4.63656996	4.09701118	4.83706807	5.10903393	4.68322439	5.33989548	5.02453557	4.34018878	4.56771451	4.31844896	5.07747006	4.7750231	5.07953927
PDK4	6.5615363	6.52734114	6.07119141	5.82278376	6.16511671	6.77380569	7.44419304	7.50777601	5.85927887	4.34922644	6.15053526	5.2187794	6.8086035
SLC22A16	0.29172816	-0.10459631	1.17954007	0.98860639	0.79023008	-0.1032174	-0.10459631	-0.10143227	2.5363559	-0.46980956	-0.46980956	-0.46980956	-0.46980956
ZMYND10	0.82049832	0.22817284	0.89947293	0.70253152	1.50496791	0.43457259	1.05495623	1.08840346	1.83294056	3.36077015	-0.28854187	2.21145101	-0.28854187
ABCB5	1.24523639	0.34541386	0.43138529	0.22742864	0.40478684	0.2288357	0.22742864	0.23065728	-0.18249819	-0.18249819	-0.18249819	-0.18249819	-0.18249819
SLC25A13	3.35588372	4.26394307	3.20251963	5.1665167	3.96798574	3.30321211	4.20485006	4.03269607	4.07552495	3.56698212	4.5477031	3.87028963	4.69275684
ST7	3.48407132	3.92783292	2.78508278	3.26020463	4.75825911	4.68072324	3.88554698	4.05776356	1.42417139	3.09635135	3.92618247	4.31837828	4.51932707
CDC27	5.56880036	5.98741162	5.40428521	6.92847665	6.54841607	6.49724765	6.39626501	6.48831132	6.53681032	5.18094798	6.61768488	6.36409532	5.13971408
SLC4A1	0.64626717	0.03833859	0.57189457	0.32874182	0.14528953	0.15490579	0.02134909	-0.14031077	-0.15794355	-0.15794355	-0.15794355	-0.15794355	3.30081864
CALCR	0.56896006	0.96467463	0.48110463	0.47851117	0.54749686	0.67011359	0.51885842	0.74610614	2.85636242	-0.30154246	-0.30154246	-0.30154246	1.89578728
HCCS	4.53487677	4.60065745	3.92950199	4.76362757	5.58954329	5.57173477	5.22732968	4.48081691	4.88423267	4.4157252	4.60100821	4.59004114	4.81445507
DVL2	5.14638212	4.71987324	5.0054965	3.61447861	4.47673115	4.00829419	4.67697592	4.48951723	4.159276	5.27364029	5.29362815	4.54467547	3.99535738
UPF1	6.46944573	6.09699635	6.38671131	5.49464154	5.85234747	5.63904152	5.90940328	5.77338475	5.7144652	5.90839188	6.18929492	6.33975828	5.56896883
SKAP2	3.74751488	4.08429348	4.12058782	5.39555424	3.7190289	5.55611814	3.62479761	2.84541155	4.92833341	4.40159044	3.39213095	3.10359131	3.85478472
SLC25A5	5.60506674	6.00963009	6.24014753	6.92397424	5.45247522	5.68630492	6.01151602	5.84415556	5.24527185	6.30934743	5.45297931	5.79348161	5.49178273
POLR2J	4.83219855	3.13782107	5.14759054	4.07646398	4.20762848	4.38343457	3.64209314	4.16099043	4.49235226	3.77599085	3.6297601	3.13695106	4.69728307
DHX33	5.10075243	4.77533576	5.39257294	5.09182674	4.57648119	4.77273613	4.92695893	4.64419769	4.25015078	4.09375206	4.82699827	4.98749717	5.22555705
MEOX1	0.97933782	3.55391175	0.74149555	1.70567861	2.68104565	1.67885868	2.59327998	2.61819618	4.33217581	3.35008704	4.35772949	3.17801245	5.59478177
THSD7A	1.96591291	3.68823684	2.21132183	4.16741704	3.94551802	2.77880338	3.89876601	3.76588996	4.90017382	3.67012159	5.34583237	3.76339117	4.99730295
LIG3	5.36656496	5.49202959	5.17657981	5.40406655	5.59379992	5.41160785	4.46094625	5.31122734	5.19736989	5.27389362	5.30262194	4.5757556	5.39086259
RPAP3	4.00984019	4.78476667	4.25602148	5.03560751	4.96095643	5.58761681	4.95782466	5.10103733	5.22264974	4.67681602	4.41981322	4.30063513	5.14976979
ACSM3	2.67056815	2.58396881	2.44308443	3.59533165	2.65603311	3.13350084	1.80555608	1.97056309	3.27069562	3.17058397	2.12429553	2.82561072	0.74431817
CIAPIN1	4.81351772	4.13043042	5.22381677	4.89876003	4.41715167	4.23647013	4.68417741	4.25312126	3.95486582	4.54103914	4.21236583	4.81197852	4.22666034
SPPL2B	3.66040671	3.52317668	5.14674251	2.57112717	3.53206575	2.04670434	3.54884576	3.90671795	3.76165398	4.27644839	2.90820493	4.06532136	3.17411702
COPZ2	4.68088096	5.47171911	5.46177985	5.51305328	4.91404192	5.41014415	4.57413687	5.25892405	6.0299414	6.03049842	5.44097073	5.39252907	4.48197664
PRKAR2B	3.42746014	3.84033493	3.18285989	3.38167243	3.97601857	4.94023416	3.70442021	3.3175812	3.57537404	3.57185269	3.3217074	4.63536447	4.46541117
MSL3	5.68053545	4.90546896	5.16800101	5.95468244	5.44413953	5.10902844	5.22906015	5.40723265	5.49430768	5.13759883	5.27783741	4.50666722	5.17454718
CREBBP	7.27499259	6.41596524	6.77739248	5.94890455	6.88587527	6.4526977	6.63734775	6.96829838	6.71490026	7.29317786	6.62828416	6.71546536	7.01799236
MPO	1.6384666	0.97330223	2.03798163	1.47314078	0.83250299	0.92704507	0.63234111	0.51771849	2.72931292	2.92222652	-0.60955276	3.0952667	1.09742807
WDR54	3.2656091	2.93740263	3.91236243	3.05770862	1.74154596	2.35515292	2.19333911	2.25294981	3.11834262	2.37095749	2.600004	1.57455751	2.93474776
CROT	4.29614431	4.84421005	5.05338271	4.98153948	4.97938434	5.46109511	5.68088098	5.30938059	5.23290192	5.40115438	5.02198386	5.3819605	5.29617069
ABCB4	4.54329501	3.95994317	3.79135476	3.19771051	3.23258035	5.15498505	4.60613066	5.03841464	3.76489358	4.41766129	4.14250529	3.34305903	5.38071345
RHBDD2	5.08960739	5.30057703	5.15066945	4.5854018	4.28227705	3.75665016	3.96596345	4.0160684	4.55216634	5.03911871	4.41944004	5.10911038	4.60374499
SOX8	4.40431251	2.87041393	4.77697212	2.44448931	3.34772245	3.00836889	4.07522593	4.44477204	4.22204612	4.80244616	4.03389889	4.96006047	3.7435243
IBTK	5.48917644	6.45224296	6.01581761	7.05984318	6.4442324	6.59055771	6.5159866	6.4676738	6.26831841	6.06407982	6.53688216	6.27859828	6.3619366
ZNF195	3.93516194	4.64682635	4.67485481	5.12117525	4.72173391	4.94020082	4.70284999	4.52333115	4.93326715	4.61525847	4.51076374	4.1542732	4.25103235
MYCBP2	5.97345157	6.49493507	5.61655168	6.44697503	6.34965626	6.49166838	6.46228071	6.15648591	6.09632843	6.13624716	6.13776897	5.78812962	6.76012818
FBXL3	5.92751378	6.9095563	5.81000625	6.59942869	7.43142824	6.99497513	7.2609319	7.7307042	7.33738118	6.12006179	7.33280103	7.57700645	6.49679009
ITGAL	4.31258621	3.07649126	3.85083518	3.61057727	2.07413722	0.49085418	2.36846983	1.16288852	2.68954189	3.06338337	3.61667134	2.96709483	3.11992702
PDK2	6.26420879	5.64555574	6.0790482	4.62578226	5.51230094	5.62192637	5.48832211	5.50228645	5.30925681	6.14861646	5.98262122	5.98093735	6.11524121

ITGA3	6.83703058	7.31885899	7.72789053	6.38636181	6.80832285	6.60015804	7.39333035	6.3723767	6.61627182	7.21077934	7.28806238	6.6507595	6.77747583
ZFX	6.14973261	6.59263442	5.99141293	6.31677997	6.59917719	6.64788109	6.99146424	6.71887816	6.78218179	6.86189447	6.85365811	6.99904535	6.55268752
LAMP2	6.32105464	6.34008839	6.00538122	7.31479315	6.92775389	6.69193641	6.8150131	6.93787624	6.84345159	6.00208917	6.72603099	6.71619477	7.05608398
ITGA2B	0.69191209	0.66305878	0.16640624	0.11430847	0.86556465	0.13245075	0.59572422	1.47999362	-0.14702965	-0.14702965	-0.14702965	-0.14702965	-0.14702965
GDE1	5.93928212	6.01199501	5.31488061	6.19777157	6.10744387	5.87846351	6.40680641	5.75613532	5.86962584	6.17016247	5.94268828	5.66448037	6.68351218
CRLF1	5.38521375	4.04402694	3.34975119	0.97522662	3.78973579	4.40234385	4.03974674	5.23890194	3.0291788	3.1139911	3.65627528	3.76345259	4.30302102
OSBPL7	4.21361464	3.05929681	4.47951833	3.01571674	3.83951186	3.4361192	4.00910144	3.78884745	4.13838013	4.57609214	4.24208773	3.8322175	3.95796046
TMEM98	6.05175445	5.50029815	5.65647755	4.965002	5.16256398	5.22345868	5.05027718	6.32081788	5.49453765	6.10723904	5.36279069	6.18198109	5.59613374
MAP3K14	4.21925623	4.42666432	4.56101341	4.12275862	3.26255689	3.18947402	3.45348566	3.40075514	3.56655031	4.01814849	3.065363	3.33038468	4.25205856
ABCC8	0.08071559	0.08071559	0.08296736	0.08071559	0.08071559	0.44111175	0.08071559	1.8461575	-0.00092804	-0.00092804	-0.00092804	-0.00092804	3.10938203
TMEM132A	4.29019629	1.95409089	4.10303383	3.64367826	1.74398738	1.72559895	2.0288614	3.13439954	2.73085265	4.39910318	2.65909873	3.70084306	-0.96716575
AP2B1	4.00958835	4.18175587	3.47131193	4.73883496	3.86929598	3.74140396	4.23821743	3.58413527	4.32208819	4.67437513	4.5293552	4.04436598	3.95532808
TAC1	-0.01966441	0.33682001	-0.01734727	-0.01966441	0.37216625	1.98264911	0.62561525	-0.01670829	0.03506416	0.03506416	0.03506416	0.03506416	0.03506416
ZNF263	5.49165182	5.17666627	5.36744059	5.2111616	5.33235539	4.86417669	4.80033474	5.05430721	5.22118997	5.62728719	5.02491811	5.03657224	4.40749575
CX3CL1	6.07508982	5.09713011	6.55142162	4.58487071	5.22342738	5.40176188	5.69557001	4.76811727	4.9634155	5.93300659	5.93041577	5.37393795	4.37759801
SPATA20	5.34867553	4.93617327	5.69081602	4.95777352	4.21160402	4.46775479	4.59649246	4.86306727	4.91696239	5.28471189	5.0208811	5.47899907	4.94684342
CACNA1G	1.30093339	0.84616337	1.55420069	1.53008862	0.849008	0.91433918	0.9896233	1.46713867	-0.59560271	2.58244983	2.49191482	-0.59560271	-0.59560271
TNFRSF12A	3.70042846	4.09613782	5.33300805	5.10061977	5.84524705	5.40349932	2.86854522	5.17617089	4.93413998	4.87197502	5.23524912	5.4982535	4.38741566
DLX6	0.28189794	0.11928707	0.12176854	0.11928707	0.16318514	0.4810229	1.19434781	0.12245285	2.74716821	-0.04572331	-0.04572331	-0.04572331	-0.04572331
MAP3K9	3.04860736	3.12854225	2.23509626	1.97507976	2.54196096	3.19367803	2.00531575	1.87588843	3.01042494	-1.16096951	1.88211231	3.12235971	3.67807189
RALA	4.7285216	5.13012095	4.57691448	6.14042889	4.87255687	5.07777529	5.09370617	4.66064842	5.01905825	5.08510747	4.31403548	4.87643837	5.22860774
BAIAP2L1	1.16775794	2.40061694	1.75422197	2.07856506	2.00897473	1.34581523	1.06836538	2.04523736	-0.69647564	-0.69647564	3.11985369	-0.69647564	2.93438091
ETV1	5.42043386	5.22462051	4.44966625	4.96933405	4.65140137	5.62544795	4.46697988	5.17465744	5.45956341	3.88044826	5.51175087	4.31897807	5.46441911
AGK	4.93962327	4.3515256	4.70800367	4.95394311	5.16407921	5.374704	4.8988936	4.86216183	4.84783062	5.61242029	4.28076563	5.34023886	5.01783293
ALDH3B1	3.98725409	4.44105096	4.53149335	3.78640853	3.20390537	2.94305001	2.85336066	2.80829396	3.21102212	4.41862251	3.54688863	3.10369017	3.61520659
TTC22	0.54470707	0.45882556	-0.04710131	0.10134633	-0.02343939	-0.04819761	-0.04957038	-0.04642043	-0.11960369	-0.11960369	-0.11960369	-0.11960369	1.80402188
PHTF2	5.17732489	6.2258325	5.51196995	6.69218284	6.88879709	6.30128264	6.6278508	6.69105466	6.94691721	5.70264443	7.34313341	6.43945481	5.40019849
CCL26	0.35415904	0.40241756	0.00124472	0.5534207	0	0.49138282	0	0.00158797	0	0	0	0	0
FARP2	5.33675973	5.662848	4.89082839	4.94448463	5.3998465	4.86909907	5.20407906	4.9565174	5.35163147	4.82528569	5.31692652	4.3485977	4.48200157
USH1C	-0.01545576	0.22321584	-0.02217824	-0.10548958	0.09862112	0.33221075	0.1256184	1.03753849	0.0289143	0.0289143	0.0289143	0.0289143	0.0289143
GGCT	3.25740773	3.93316891	3.66738088	4.51864699	4.20624008	3.39820489	3.72210078	4.30762556	4.50956187	3.71363399	2.88307301	2.87734044	3.31517123
DBF4	2.92099762	2.86324398	3.57725865	4.97311268	3.29721438	4.16288258	3.4961726	3.77563282	4.0031824	4.09648448	3.36237284	3.22954752	1.4555523
TBXA2R	4.50832904	4.78255792	5.21162544	2.81577433	4.68467376	4.86129208	4.9917223	4.19353642	4.53395126	5.52922375	4.30839124	5.04171463	3.21554809
IFRD1	5.10553356	4.87760307	5.04885712	5.80183747	5.97660519	6.12301612	6.25283169	6.33539084	5.76099412	5.05257589	5.61635047	6.1659913	5.40918613
COX10	4.48378719	4.22137106	4.11678454	4.06743084	4.22390893	4.13932207	3.96100607	3.16134436	3.5932655	4.38606858	3.35709342	3.64770257	4.318173
GTF2IRD1	2.16248186	0.91672825	2.71648708	1.07727483	0.89862625	-0.07518782	0.68571607	1.18527059	3.15471459	3.33946327	2.42769674	-0.08008835	-0.08008835
PAF1	6.16626148	5.54810711	6.11507799	5.16436517	5.59623688	5.8160277	5.4366364	5.19330887	5.98181411	5.98072607	5.3044638	5.26958838	6.14089935
VPS41	5.98369353	6.49699339	6.22165672	6.66659888	6.6224742	6.83664027	7.02650651	6.76694843	6.87295515	6.49635321	6.93450127	6.69357217	6.73764693
ARHGAP44	3.81677663	4.06996193	2.83088724	2.39639441	2.64807323	2.03809652	3.35383545	3.15324438	3.01668772	3.86353176	3.47072114	1.70953575	3.46544062
ELAC2	6.10099626	5.63094231	6.35324602	5.30860111	5.5161225	5.38258809	5.5161225	5.55626021	5.68658662	5.69471393	5.48251174	5.99653201	5.69947153
SCIN	2.8372693	4.25185107	2.64565115	2.96205428	2.35998293	4.02517041	2.25012364	2.33318003	1.39682047	4.41284767	2.09170595	2.62298897	2.69957353
ARSD	5.58868766	4.65469885	5.40337719	5.06216429	5.28237819	4.22628682	4.86702992	5.25983207	5.0788825	4.92129513	5.72212636	5.18417945	4.62805047
PNPLA4	4.52315621	3.74353068	3.79264516	3.41444738	3.7989133	4.63096163	3.51846513	3.73923261	3.84319413	4.73115019	4.11381231	4.73745534	3.96319528
ADIPOR2	1.97228076	2.24142784	1.13518498	2.23695675	2.51645831	2.48258194	2.83412492	2.48208274	0.49177515	3.37257693	2.41821153	3.44170969	4.04934875
CDKL3	0.63445592	0.5878483	0.43136113	0.58251851	0.7818154	0.88203891	0.91406733	1.02961236	0	0	0	0	0
PRSS21	0.68629043	-0.1670142	0.19623506	0.72928127	-0.03786392	-0.1658007	-0.1670142	-0.16422971	-0.01980845	-0.01980845	-0.01980845	-0.01980845	-0.01980845
MARK4	5.2494922	4.608312	5.47390774	4.4027593	4.91752349	4.52094402	4.78269137	4.80665396	4.75889724	4.77396777	4.84165846	4.76761006	5.3702387

PROM1	2.84955006	3.18083054	3.85293711	2.91645924	6.40041989	5.39990974	4.44860194	7.15113325	6.27109662	6.52146085	5.77563469	6.8718299	5.84347959
CCDC124	5.50311517	4.94176782	5.94460509	4.60164089	4.44604283	5.10235937	4.33449922	4.04924598	5.19415252	5.64089756	4.06470536	4.56232027	4.75811069
CEACAM21	0.09279084	0.44060367	0.26338083	0.86948057	0.73049738	-0.71227898	0.16430583	-0.51209369	-0.00587948	-0.00587948	-0.00587948	-0.00587948	-0.00587948
PAFAH1B1	3.38059277	4.24883048	3.26962949	3.78439474	4.59488662	4.21514212	4.54696766	4.6937023	4.76018919	4.45482146	3.87306707	4.1384372	5.00223472
NOS2	1.18688108	1.6752765	1.9238889	1.52678675	0.81606514	1.50874914	1.03130294	1.56864084	2.96838367	-0.47551616	-0.47551616	1.6264821	1.86582165
KIAA0100	7.3203559	7.33346304	7.16422575	7.26137275	6.96853521	6.64398866	7.30974865	6.89294204	6.98973653	7.18142598	7.49571394	7.1050997	7.44558141
GAS7	5.00155847	6.88874426	5.9485376	5.33398419	4.93705386	3.43560688	4.67187622	4.80990466	4.98036751	5.07015267	4.88156988	5.4529118	6.75181034
TRAPPC6A	1.78871967	2.32100392	2.809515	0.89483524	1.84713584	1.08801003	1.25144377	2.47079689	2.94333915	-0.34427743	3.85798122	3.58591356	-0.34427743
MATK	1.32611525	1.93166272	2.70333257	1.12290363	1.03035448	0.7695514	-0.10221449	0.53924543	2.23266281	-0.60084762	-0.60084762	2.95523766	-0.60084762
CD79B	1.34441997	2.40689983	0.91894173	0.85403318	0.61088188	1.36086076	0.97272499	1.37644624	3.40564726	1.86874736	3.48556381	0.81841832	4.55984618
SCN4A	1.04824533	1.28777454	0.84969154	0.90932217	1.73282355	0.92441952	0.91280946	1.49369267	-0.30841876	-0.30841876	3.3387602	-0.30841876	2.92383
ST7L	4.92070451	4.64816414	4.66197114	4.68562402	5.35772119	5.06385092	5.39435589	5.18583361	4.43253945	4.93202309	4.65535272	4.97538573	4.83795628
TKTL1	2.44109007	0.57969867	0.3290432	0.68884773	0.23614195	0.33148414	0.31397857	0.20321423	-0.37262644	-0.37262644	-0.37262644	-0.37262644	1.82432212
PAX6	0.86708934	0.7465909	0.84661044	0.66862737	0.68057468	0.82123474	0.80298601	0.77074527	3.63350667	2.7004902	-0.26925574	-0.26925574	-0.26925574
RPUSD1	3.77721631	3.48857527	4.85638968	3.2037151	2.75607326	2.36725562	2.58238342	3.35065665	1.97424076	4.17184073	3.79384346	3.02872873	1.93768971
RHBDF1	5.52498164	5.33755443	5.99291057	4.16776776	5.60700165	4.77235739	5.33755443	5.92545052	4.86838122	5.43233144	5.19674525	6.05330582	4.3695927
LUC7L	5.63247485	5.55642993	5.49160912	5.91302979	6.1201394	5.17124093	6.42503853	6.30829787	5.69918685	5.63860738	5.77843954	5.27714945	5.48743505
CACNA2D2	4.15101598	2.61555856	3.26838373	1.59512119	3.60628607	3.71691694	3.3190832	4.15526142	3.58731388	5.31217207	3.39447174	4.04068312	3.27534364
BAIAP3	1.55090377	1.12336882	2.09390558	1.60239925	3.06249518	2.73825577	1.74547224	1.50707528	2.04323204	2.45183577	-0.79873376	3.5536433	2.60734191
PIGQ	4.52003853	4.22283489	5.24324865	3.5789572	4.0848303	3.31484237	3.88554011	3.57379186	3.95026261	4.28702847	4.86248729	4.73610578	3.36117174
TEAD3	6.83987728	6.02008147	6.65534853	5.2058535	6.75521869	6.09072237	6.47339859	6.63239681	5.18435014	6.91353301	6.2721627	6.34869226	5.63809712
SELE	1.81823966	2.57292586	1.96314299	3.8446122	2.00687843	1.8692159	4.96967239	2.12407388	4.16718587	2.96094855	3.59947878	3.85843028	5.13019507
DNAJC11	5.47327919	4.77244611	5.53814759	5.24885354	5.11360086	4.66318356	5.00831444	4.7827919	5.12161977	5.1537177	4.87892846	4.98709498	5.6650547
FMO3	2.85056383	1.70632886	-0.34944445	1.33634219	2.92678098	1.78859292	1.24659575	0.9929638	1.09483508	-0.47665863	2.83535591	-0.47665863	-0.47665863
MYLIP	5.29155236	5.40901452	4.96502204	4.15063573	5.39956192	6.34054123	5.21541364	6.03696993	5.0924441	5.99822752	5.7983769	6.03174746	5.71018127
NOX1	0	0.22768433	0.00124472	0.16615742	0	0.00069205	0	0.00158797	0	0	0	0	0
E2F2	1.0278559	0.63339735	2.83212657	1.55426886	0.01206296	-0.02274259	1.47903464	0.12893108	-0.51563877	-0.51563877	2.38639027	-0.51563877	-0.51563877
PSMB1	5.88144702	5.72950655	6.0062295	5.92939353	5.46167219	6.39575189	5.63914311	5.79333793	5.99274943	6.27112675	5.83549477	6.05469568	5.81965874
SYN1	4.08941569	1.54074433	3.48788842	2.51486235	1.68404295	0.94847316	1.1138508	1.32205368	2.69318024	2.93928745	-0.95651157	1.48911022	3.7793255
JARID2	3.68028657	2.69616789	3.15442007	3.82745136	2.22842598	2.95398334	2.70001894	2.49938812	3.4510682	2.35569371	4.20388343	3.59776156	4.01904304
CDKL5	3.89694829	3.99747196	4.14482661	3.5650248	5.24854629	5.51195139	4.55673514	5.13254454	5.03982046	5.36126586	4.85925912	5.16931989	4.94753682
CAMK1G	0.92021429	0.01608731	-0.06069413	-0.02709905	-0.188109	-0.04772452	-0.04300893	0.351007	0.00806729	0.00806729	0.00806729	0.00806729	0.00806729
CDK11A	3.38469634	3.8041689	4.20762021	3.35776326	3.42866791	3.53265963	3.79837207	4.1981113	4.14682701	3.901896	3.07524103	3.31616181	4.25683774
NADK	5.72138151	5.32065005	5.78419817	4.53156185	5.24913215	4.83308089	5.65792414	5.7382024	4.84875849	6.05676889	5.09307825	5.83855603	5.36177347
TFAP2B	0.00485815	0.03747928	0.00124472	0	0	0.38035793	0.08315574	0.00158797	0	0	0	0	0
DLEC1	1.55321887	1.53889896	0.95542491	0.17165844	0.12055187	0.1032987	0.41058251	0.16869872	-0.26715403	-0.26715403	-0.26715403	-0.26715403	3.43261461
CYTH3	7.03781034	6.90829179	7.09722835	6.37663035	7.07556748	6.92460859	6.69853583	6.88937021	7.02119215	7.12764605	7.11546012	6.97180237	7.15502008
ADAM22	3.36152512	2.94483595	3.23655239	3.38979026	3.59636585	5.29117689	5.28827582	6.46516044	4.9135643	6.56961269	4.40309283	5.22515957	4.6953641
SYPL1	5.23128448	6.16731955	5.28346798	6.6425337	6.7251491	6.86576385	6.85047092	6.75254522	6.43313112	5.89790738	7.02170657	6.95703815	6.07107548
CYB561	3.91143087	4.57081596	4.2246464	4.944007	3.9334322	3.30396457	3.5759351	3.47324067	3.60127573	4.85206776	4.65788482	4.10853012	4.93516274
SPAG9	6.67691156	7.10552262	6.69433207	7.27146232	7.38483815	7.31860577	7.37200756	7.46420919	7.35139632	6.98340286	7.2212796	7.36088217	7.19872382
CELSR3	1.62361793	1.49120353	1.98613047	1.2823862	1.55252064	1.38343538	1.79397407	2.19196324	2.58925096	-0.64422413	-0.64422413	2.68380053	3.11259795
AASS	6.13073433	6.45861471	5.30794112	5.62454002	6.67068819	6.65948966	6.66122369	7.32288903	6.78879456	5.4547842	6.44870719	6.10737933	7.13775792
SS18L2	2.48227181	3.81566347	2.5611565	4.29035619	2.93190125	3.26459965	4.05625054	3.03919997	4.00429382	3.53199331	2.88735915	3.62755962	3.0516527
MPND	3.59520459	4.33207885	3.84612734	2.73739445	2.95784942	2.90624487	3.24112317	3.50256711	3.28746173	3.912458	3.29662539	3.22530351	3.36778824
MGST1	3.0610181	4.45062561	2.85017093	5.1543177	4.96846797	5.21002972	6.3076506	4.21575693	4.24855695	3.48626298	4.46461203	4.34414682	4.79714794
CRY1	4.18932786	4.96697131	4.40402475	4.45811215	5.68353293	5.18566384	5.05917859	5.75888905	5.05321771	5.00659354	5.21371559	5.96209517	5.327733

NFIX	7.79982694	7.62202084	8.36158318	6.55488496	7.30017742	7.5603233	7.87768853	7.75493639	7.51610301	8.31734241	7.53354296	7.83439125	7.30865586
ST3GAL1	7.65146	7.41034902	6.83800241	6.61219267	6.11213463	6.31433392	6.37244042	5.46507783	5.69118654	6.31971983	5.70948894	5.84695153	6.79703622
MMP25	1.71217846	0.12220006	0.16435032	0.50714718	0.59529106	0.13145413	0.55131185	-0.01910704	-0.17652235	-0.17652235	-0.17652235	-0.17652235	2.19051025
IL32	4.63589478	5.02151567	3.85595118	5.44566183	3.44756072	2.70110365	3.68323753	3.58812203	4.22770813	4.2696905	4.34201517	4.35630605	3.30154794
PKD1	5.7262665	5.64301633	6.25309873	3.57410795	6.17334754	5.19610445	5.82233036	6.46024778	4.74537105	6.52567878	6.44035077	6.9018363	4.89886804
MAPK8IP2	0.16698266	0.56453738	0.31145053	-0.01055226	0.34264822	0.33950027	-0.0269315	0.18794658	-0.13899857	-0.13899857	2.88954671	-0.13899857	-0.13899857
MED24	4.06586855	3.81143983	4.77818992	3.99927907	4.06536557	3.14856318	4.16295744	4.07787727	3.22518198	3.16460532	3.93836831	4.44362729	3.97059544
RHOBTB2	5.63605131	5.88894964	6.05502682	4.5234824	5.42972239	5.60865317	5.411293	5.39755912	5.74098939	6.4544828	5.49037555	5.89073726	5.62306084
HEATR5B	5.72401091	6.14831019	5.15330545	6.14529768	5.91933873	5.96007249	5.93855821	6.25408369	6.10731734	6.12149149	6.10209765	5.86131603	6.15907463
SEC62	6.30973626	6.76310665	6.34768834	7.0476181	6.98254714	7.31719212	7.18206324	6.96780133	7.26771062	6.7236313	6.7733949	6.82265387	6.46764991
RPS20	7.70645054	8.80150242	8.22813666	8.74409924	8.10356893	8.09456924	8.55203424	8.39129338	8.34405455	8.36531773	7.73696084	8.00862417	8.35000029
CSDE1	8.41875473	8.92352424	8.39140917	8.69931756	9.05758395	9.18811774	9.26705487	9.20910521	8.8982906	9.01328238	8.71361984	8.8440166	8.78645009
UBE3C	5.41102609	5.54942391	5.1007853	5.67396313	5.56434721	5.09509726	5.84090374	5.48758122	5.13447375	5.06126576	5.6441653	5.39262213	5.18448414
REV3L	5.79376302	6.44043796	5.87558761	6.98358275	6.57251806	6.75270084	6.5582446	6.59605485	6.31932881	5.88993181	6.21635236	6.28834487	6.31636841
FAM76A	3.48504396	3.11204943	2.42562996	1.94386588	3.56758731	3.65379928	3.46027684	3.61613051	3.88788174	3.52794068	2.78410675	4.32967702	3.28735607
TRAF3IP3	3.35732413	3.2580029	1.80898201	3.96754224	2.39262176	1.51788921	1.65636384	1.23365788	3.91452476	-0.99793814	3.40467164	-0.99793814	2.78894682
POMT2	4.34733417	3.51074538	4.83652971	3.99435462	3.92003731	3.60489661	4.35198017	3.99435462	4.20842054	3.20969017	4.7177159	4.025275	3.48024021
VTA1	4.6402486	5.23288869	4.88200939	5.48953184	5.25266238	5.86692048	5.13036837	5.39208765	5.58552443	5.00934335	5.15457245	4.90363529	4.67229223
MLXIPL	0.00457763	1.34740734	0.0354086	0.67161865	1.97446677	1.29103649	-0.00047193	0.5611096	-0.18886784	-0.18886784	-0.18886784	-0.18886784	-0.18886784
BAZ1B	7.1454625	6.99690445	7.29776466	6.816273	6.81002042	7.18361861	7.10921152	6.60286648	7.14666562	7.28761945	6.61278779	6.59433677	7.32453903
RANBP9	6.43049808	6.33442859	6.34096787	7.01759558	7.58968478	7.18413683	7.20482947	8.01578111	6.69296495	6.5564745	6.45570235	7.03794388	6.70446882
ETV7	4.13696606	3.71226296	3.31397492	1.71095426	2.75678313	2.8597913	2.25905165	2.82261201	2.88953548	3.27436775	2.3662978	2.70394806	2.50121154
METTL13	5.0161227	3.93544782	5.25872764	4.33688056	4.31365112	4.57744021	4.67753451	4.51775219	4.30930248	4.01068353	4.96373757	4.72146887	5.02375046
DYRK4	4.44080706	4.25075063	4.63350927	4.4570219	4.19297485	4.84024674	3.77146802	3.79415243	4.66390837	4.90842012	4.10363296	3.82612155	4.57079293
ZNF207	6.34201346	6.15550015	6.00032496	6.82764892	6.60300465	6.33661243	6.8146982	6.63083389	6.32936808	6.60923962	6.45312402	6.22965404	6.41626307
UQCRC1	6.00267169	5.81784819	6.38561282	5.8015783	5.6667209	6.07981216	5.69032857	5.66272574	5.59606455	6.17031996	5.7454316	5.9099035	6.61765147
STARD3NL	4.43703392	4.83746751	4.34858204	5.49694754	4.64587652	4.89598662	4.57271354	4.41908119	4.84156898	3.77225584	4.61776961	5.0412857	5.03135503
CD9	7.96483068	8.31376732	6.9805604	6.93859082	8.05276865	8.04672229	7.85659271	8.33487051	8.15610802	8.03966042	8.14520154	8.17646226	8.12819615
HHATL	1.04421064	0.5986756	1.34877985	0.5986756	0.89314247	0.78796515	1.16848217	0.60197318	2.55212213	-0.34873943	0.70994897	2.62043658	6.61128786
NCAPD2	4.17879123	3.68625866	4.86669184	4.5121584	3.25882833	3.17043303	3.92743074	3.63882004	3.92710851	3.76969444	3.51334178	4.62938042	4.40282544
IFFO1	5.55537505	5.45088234	5.89147522	5.01760769	5.14187651	4.55494745	5.45656228	5.11696728	5.4459499	5.73100902	5.57137008	5.7698706	5.74536547
GIPR	0.16868602	0.53297349	-0.23123192	0.24095433	0.03140701	-0.23232716	-0.03995216	0.04462293	-0.16003541	-0.16003541	-0.16003541	-0.16003541	2.08489745
PHF7	4.52801834	3.69925624	4.17327353	3.38196353	3.55337437	3.31453459	3.59530258	4.34557762	4.06868637	3.34834276	3.96878878	3.73697982	4.7351257
SEMA3G	1.00644105	2.88352978	1.18232898	3.167491	4.44897845	2.25906503	4.14015715	4.41938353	3.78940023	5.65230078	5.62867934	4.69554759	5.81417041
NISCH	6.61833146	6.33878434	6.79971149	6.13478891	6.13762645	5.48641122	6.50014577	6.39261207	5.86140691	6.43692578	6.85881281	6.6371365	6.08750632
STAB1	6.18625334	6.41835226	6.86415549	6.90529366	5.45700671	4.69166753	6.02452796	5.02481344	6.08207589	5.90160853	6.70574614	5.89833265	5.77208168
FUZ	4.03203434	2.34324374	4.15160868	1.59566935	2.19806999	2.47209658	3.06941685	2.42151638	2.91638983	-0.90480279	4.05893841	2.4247241	1.20644126
SLC6A13	0.27705659	0.51255777	0.24531377	0.04075601	-0.06285707	0.64129866	0.35492564	-0.23232372	-0.03666892	-0.03666892	-0.03666892	-0.03666892	-0.03666892
IDS	7.56677809	7.49618171	7.15049518	6.38318866	7.7872497	7.38573892	7.45285313	7.81887747	7.93144589	7.47771003	8.10678049	7.8125526	7.23320557
ZNF200	3.52083071	3.62864351	3.52165677	3.78933282	3.77798372	3.91017975	4.08154491	4.23835823	3.9074555	3.99670639	4.23401143	2.89907333	3.22278619
CD4	5.73833872	5.62348393	5.65608376	5.97123609	4.58746288	4.00452997	4.71654769	4.00286298	5.43413006	4.86942479	4.93321758	5.06632856	5.4753878
LRRC23	4.60775257	4.0950021	3.56454463	2.78622352	3.99134692	3.9513481	3.82559406	3.09569045	3.85651931	4.05352456	4.09228737	3.75952448	3.32652508
BTK	4.1715935	4.8266606	4.43821891	5.40115168	2.93228662	2.60307078	3.81013156	2.6448937	3.83965685	1.79357061	3.27374352	2.04841359	4.4683145
HFE	5.01413481	4.85274677	4.48782759	4.28831432	3.85616568	4.39683186	4.17951362	3.52150855	4.86640278	4.93693129	5.28351277	4.82369037	4.80397483
SCMH1	6.21661068	5.93166166	5.80569378	5.14987791	6.13841023	5.83021267	5.88473116	6.28772687	6.18522995	6.79249093	5.76867778	6.07434532	6.38168987
FYN	5.07709572	6.0342124	4.6721125	5.43606967	5.44889477	4.77968446	5.20743596	5.28125414	5.34898272	5.07081378	5.54240823	4.94686121	5.81324792
HIVEP2	7.44620596	6.73251843	6.57297597	6.31803688	7.07034957	7.64643727	6.71438828	7.50040499	6.56748066	6.74735829	6.54201942	6.58038	6.98600776

FMO1	0.35592729	0.87798599	0.35829663	2.83336678	0.67585984	0.77645834	0.88849309	0.52334844	-0.33433238	3.40697911	2.62029489	-0.33433238	3.1200376
LYPLA2	4.48313244	4.22010755	4.65502176	3.89790453	3.55181436	3.97363014	3.59008242	2.62789872	4.28324802	4.52550642	3.50625616	3.34558715	2.86860924
CLCN6	5.48410991	4.90641135	4.98541224	4.46127401	4.91794736	4.29631965	5.0848728	4.83431596	4.68777713	4.65159293	4.66585561	4.6232973	4.90419833
MRC2	7.37692819	6.90477415	7.51466597	7.38964952	6.45965331	5.99248883	6.64211861	7.00416827	6.69585463	7.19988997	7.49588899	7.29305842	6.70279471
SLC6A7	0.64809586	0.23465333	0.23724791	0.28413768	0.23465333	0.23609588	0.23465333	0.23796341	0.62808914	-0.15991321	-0.15991321	-0.15991321	-0.15991321
TSPAN9	4.58234674	4.16308114	4.55739775	3.27481342	3.55317143	3.44380305	3.44627296	3.17368444	3.88475814	4.54037415	4.56478936	4.44405179	4.19932422
BTBD7	5.66447379	5.96173807	5.35147756	5.49591038	6.00694607	5.79288221	6.15856635	5.9439649	5.34174462	6.13326747	5.53346483	6.24334016	6.12089579
APBA3	3.98745353	2.38910789	4.05395711	2.43431949	3.04324819	2.74694533	3.00382323	3.03215608	3.12238538	3.69485157	3.02659417	3.65871432	0.34404394
MKS1	4.36342049	3.84592602	4.14737683	3.27820728	3.51935443	3.40284178	3.45094814	3.17064468	3.29732559	3.60231058	3.57818535	3.54451587	4.14140467
ABHD5	5.14046601	4.80667155	4.57776676	5.48613778	4.72767791	5.31243542	5.0707416	6.10077132	4.78514222	3.96453979	4.88807563	5.17764269	4.54061972
AKAP8L	5.77571664	5.02705722	5.95124005	4.86375636	5.67322286	5.11018967	5.59068408	5.84258819	5.51843603	5.42352189	5.41170103	5.38032036	5.62922339
MBTD1	4.46193078	4.73056587	4.43592879	5.29536212	5.34938624	4.93472859	5.40697812	5.47963574	4.84308779	4.20008932	4.70138436	4.72451235	5.07469777
UTP18	3.55224246	4.2071711	4.37878165	5.24072389	4.25291411	4.28470944	4.77365047	4.61801508	4.49677413	3.76072894	5.03308834	4.58686277	3.68841657
RNF216	5.35178701	5.08047637	4.95048507	4.86517849	5.50030432	5.40171291	5.35178701	5.91165706	5.09870243	6.15787027	5.02379254	5.60259906	5.5984165
TTC19	5.2724885	5.42550552	5.18831899	5.63565647	5.68498605	5.18449316	5.71593952	5.51680106	5.26121835	5.5217578	5.26126002	5.31768294	5.86179613
PTBP1	6.07271425	5.89074039	6.04943898	5.98578634	5.32186512	4.80032311	5.60042974	5.29970615	5.43487288	5.75331131	6.03333745	5.56836528	5.56631845
DPF1	0.55818125	0.36978888	2.01854032	0.62676988	2.08337546	1.7217912	1.31825253	1.88765988	0.22891624	2.59505102	-0.43706191	-0.43706191	2.27915828
SYT7	2.29794389	2.03972526	3.09719771	4.20112725	2.32470859	1.62007828	1.51500892	1.7368685	2.37533073	2.77592892	1.54108448	1.67915199	4.70304502
LARS2	4.54275552	3.68987624	4.43993515	4.4818262	3.86242928	4.15851898	4.0376484	3.91903899	4.59051748	3.60604348	4.35375023	4.16346525	4.61539691
PIK3C2A	5.90473042	6.57323973	5.9914475	6.91703387	7.22138125	7.33416924	7.12497383	7.35190494	7.02648763	6.27361022	6.85358962	7.2592038	6.39527518
PLAUR	6.069696	4.997988	6.45610336	6.62743906	3.43450066	3.23291972	4.108377	3.64269913	3.22752512	3.48871752	4.17133093	4.78616383	4.58422604
ANLN	3.3988134	3.0219803	5.69920876	6.28700361	3.21035956	3.34220301	4.02046727	3.22464195	4.76386861	3.98143938	3.18526718	3.9399906	3.62103239
WIZ	5.40409613	4.47698518	5.7579782	4.26967497	4.7558565	4.80876983	4.91781859	5.02975504	5.26837696	5.50086635	5.49141019	5.26924559	4.77524432
RABGAP1	6.28893409	6.74589504	6.09147596	6.1496673	6.79511275	6.78628692	6.94901974	6.85536463	6.54945875	7.17003487	6.41694617	6.71375948	6.7278238
DCN	4.74389089	9.29235689	3.70515422	8.6259067	7.45841276	5.18923846	7.17644762	7.40048568	8.03516038	8.28276892	7.89912947	8.89248011	8.61769394
QPCTL	3.11476337	2.32591463	4.20490922	3.12271325	2.50604396	3.11814545	2.25241301	2.69613055	2.80950249	2.87285404	2.39160337	4.01452612	3.14316973
PPP5C	5.11849618	4.27748515	5.40752142	4.1594948	5.04466312	4.9034261	4.89667845	5.67399129	5.08674129	5.21937608	4.8598213	5.0942636	5.57597352
CEP68	2.61961877	2.85444516	3.17694906	1.45390613	3.1779451	3.07908674	2.43547162	3.21142557	3.92977086	3.16290028	-0.37357045	3.71720672	1.13881814
MAP4K3	5.45656694	5.80112788	5.50286653	6.29903909	6.40095177	6.4508242	6.51207646	6.78918328	6.39995131	5.36209229	6.53902653	6.19644515	5.91968976
ZBTB32	0.09173861	0	0.00124472	0.17494462	0	0.00069205	0.21886537	0.00158797	0	0	0	0	0
TYROBP	6.63828762	6.11154759	5.74289711	6.59182694	4.81642826	4.59307123	4.66642644	3.91899785	5.61896366	5.72724931	3.6823747	5.0394565	5.4756339
TMEM159	5.45584935	6.09816807	5.27546605	4.02259033	5.66450084	5.89010057	5.78015201	6.10999999	5.99307693	6.62994174	5.86001584	6.37730482	5.48411477
BRCA1	2.4672821	2.78899994	3.27711313	4.70202176	2.92436966	3.32331101	3.00325626	2.64328509	3.54112286	3.3287618	1.93069824	3.06871433	3.61284363
ERCC1	5.52901586	6.0043502	6.47059494	5.80846437	5.45126408	5.04157887	4.89225123	5.40312981	4.96602134	5.67064628	5.61461423	5.76398778	4.76239134
CD22	2.28838583	0.92600131	1.35536221	1.42931737	0.94848196	0.40970856	1.04606253	0.0842029	-0.48831807	-0.48831807	3.16661993	-0.48831807	2.15327432
SEMA3B	3.44812104	4.78727683	3.59141902	2.56879919	3.05813011	4.98135594	3.71570416	4.86314565	3.67950714	5.14894054	3.81188579	5.23761746	4.6061372
MBTPS2	3.28963024	4.03881662	3.33158316	4.0092409	4.22955251	4.38329796	3.8870685	4.35833864	3.76241493	4.26204019	3.56679357	4.21544551	4.85192452
PRICKLE3	4.68806864	2.90717786	4.49572222	3.28928763	3.67342874	3.76641388	4.02970443	3.5607299	3.08894479	4.5032091	2.86757276	4.27364957	2.58716794
LTF	0.77280028	2.77479585	0.39447994	1.00274213	1.15071889	0.11297659	0.38855727	0.95013134	-0.54063779	-0.54063779	3.97494132	-0.54063779	-0.54063779
EXTL3	5.95852425	5.9841151	6.00703848	5.11548369	5.55705473	5.44222585	5.59606549	5.58234326	5.37729762	5.88884078	6.11425704	5.97263488	5.80049007
ELOVL5	5.99510484	6.35886882	5.92659759	7.47480446	6.97174996	7.15344419	6.64212306	7.12445272	6.556786	5.93165603	6.49978454	6.26275023	6.33890268
ALOX5	4.79780276	4.95524473	4.64866116	5.19346471	3.6130336	3.04084433	3.48518003	3.51069966	4.79592	4.16883457	3.55884962	3.31554643	4.59177767
KDM5D	4.33195122	4.31027829	3.53131281	3.42017356	4.36551874	3.43511905	5.23514864	4.90180919	-1.06405962	1.41055622	-0.44035798	-1.54074073	7.37365517
CALCOCO1	7.90249445	7.10793813	7.38174262	6.2294917	7.66581188	7.80242298	7.58475236	7.83360155	7.30338439	8.36086986	7.24590938	8.06394997	7.26205297
UBR7	4.72823162	4.70081305	5.02637171	4.93593733	4.4329364	5.19658202	4.9706118	4.42254758	5.12810026	4.51404872	4.80345797	4.17465115	5.44104447
MAP4K5	5.34604353	5.85157213	5.51181577	6.34445497	6.31637144	6.25030974	5.99356491	6.3818382	6.58432532	5.8323105	5.66815386	6.06717143	6.21098387
EHD3	6.39645264	6.68092709	7.33233078	5.24621336	6.65021145	7.15799502	6.87830735	5.6276562	7.57272758	7.32051631	7.88677587	7.12848255	6.56599007

PSMC4	5.85547871	6.07342616	5.6608806	6.0445111	5.40676948	6.03829859	5.19642426	5.18765781	5.1541513	5.40188777	5.41919467	5.870951	5.10615484
MAN2B2	6.936992	6.48166321	6.62840638	5.3733541	6.41916701	6.42119647	6.03193742	6.68669576	6.43915778	7.19648105	6.74232045	6.77328769	6.46268797
CLDN11	0.62003301	3.39584919	-0.30242716	-0.04338473	0.54141826	0.04732779	1.04720608	1.43289701	0.01084481	0.01084481	3.03254793	3.51361468	0.01084481
SLC25A39	4.41505667	4.54706875	5.4312652	4.54757684	3.88247089	3.42875709	3.76530964	3.74371008	4.6500014	4.90644338	4.36242479	4.66363572	2.8552073
MVP	6.7547394	6.41858844	7.40871555	6.30958709	5.70193115	5.547937	6.13637177	5.71029739	5.64671656	6.30207445	5.64235868	5.58367031	6.00134299
NUB1	6.5120928	6.2959373	6.17580322	5.83774971	6.39664924	6.70235509	6.31154198	5.98988391	6.48154935	6.24843161	6.15212026	5.80806131	6.34529455
PGM3	5.21423342	5.57656283	5.35820532	7.14690462	5.68179838	5.80156733	5.52620874	6.09967415	6.08878829	5.38797563	6.04245216	5.70027456	5.79811063
RWDD2A	4.5080175	4.38259287	4.09246795	4.27433858	4.70300795	4.71706559	4.23234166	5.12019528	4.74132547	4.80532595	4.60140877	4.47122012	4.52159962
CLK1	6.0121692	6.57972697	6.15599942	7.0384089	7.19130507	7.17443	7.62502101	7.55859658	7.2899434	6.58144482	7.49525224	7.34352257	6.50871568
POLR3B	4.30740789	4.90936416	4.63828588	5.46486562	4.46455823	5.45470177	4.35682798	4.33251642	4.54080719	4.38357041	4.18389097	4.53651357	4.83900653
ANGEL1	5.5070772	5.00597596	4.80896308	4.98041886	4.95128446	4.37351874	4.7142013	4.40057589	4.55946909	4.76956057	4.43478578	4.56923914	5.09932455
RNF14	5.29630196	6.14301841	5.47587477	5.58853574	6.05264251	6.17354855	5.93547047	6.22898866	6.13363438	5.6900368	6.28312992	6.17877068	5.12208424
DNASE1L1	4.96453372	4.70992945	5.43899613	5.14962836	4.10691918	4.89776705	4.46871503	4.48726756	5.12115701	5.42758846	5.30595952	4.72325509	4.33797996
DDX11	5.02704902	1.66709814	4.09350893	3.16992467	1.38097583	2.1418055	3.561712	3.4446262	4.70582659	-1.16237252	4.57115725	3.08989816	2.77658005
HEBP1	5.81034711	6.1579906	5.5835058	5.2023894	5.93834839	6.3293187	5.73237267	5.76825062	5.44851089	6.29863565	5.56855261	5.77101103	6.47575764
GPRC5A	5.71043935	4.48870634	5.23662676	3.91228468	5.7406724	5.6946489	5.01188066	6.58570107	4.63057852	6.24486942	4.90924271	6.41674236	5.04999807
MAMLD1	4.85202492	3.9176549	4.66692384	3.46284865	4.52000758	3.69746684	4.1565435	4.19576851	4.40251814	4.60193434	4.57072208	4.40902431	4.87528596
CD6	1.5103864	0.86126908	-0.19934262	0.52221887	0.24670787	-0.10823066	0.00140533	-0.15674106	1.26964385	0.25306923	0.25306923	0.25306923	0.25306923
TACC3	3.68592841	2.75119514	5.14541775	4.57097069	2.3594018	2.36293833	3.2398621	2.58369478	3.21224774	1.5772083	2.46664851	2.17595433	4.20725392
UFL1	5.6225089	6.25647696	5.49543	6.6169359	6.38449127	6.80609531	6.37952483	6.3312544	6.50133124	6.57257781	6.11359349	6.57488287	6.11494006
POLA2	4.12371479	3.54751429	4.56342972	4.34908988	3.83995881	3.20658864	3.89637733	3.8477969	3.508968	3.96408006	3.94429366	4.15247755	3.41589691
ZC3H3	4.21872959	3.65807318	4.46594801	3.54477737	3.56478114	3.18274721	3.60255193	3.45044573	4.0540676	3.99234276	3.91446151	4.03440279	2.56978512
CAPN1	6.0023275	5.87095078	6.51857295	5.68752773	5.57933744	5.01551712	5.96372452	5.72758569	5.35121324	6.34082539	6.2344561	6.23441482	5.88442029
ACPP	1.30711052	2.16793187	2.75693064	1.77678672	1.19197894	0.98144741	1.59587975	0.62783983	0.40562994	-0.78059289	-0.78059289	-0.78059289	3.21940387
MDH1	5.14997436	6.28538883	5.36858679	6.37097627	5.46770967	5.89975134	6.03216534	5.65488514	5.92920962	6.36757193	5.96168802	6.18916067	7.18605649
SLC30A9	3.88198145	4.71359876	3.76842008	5.21869658	4.9212833	5.08964794	4.78491001	4.94940059	5.25126585	4.59140716	5.08079336	5.19999647	4.26020661
MTMR11	5.44134134	5.65478237	5.70786681	4.31905126	5.93595288	5.1992747	6.30363248	6.10683837	5.63338812	6.42392383	6.26508079	6.19030715	5.6470115
COX15	1.77126457	1.62984291	1.70050567	2.33557865	1.99051029	1.92093299	2.18140633	1.5148928	0.12611224	0.12611224	4.09744728	0.12611224	2.1191578
CCDC88C	3.59282007	3.22392553	3.33299687	3.15891651	3.73531247	2.04245378	2.87503528	3.17660773	3.72361504	2.88606245	3.67836375	2.91746067	4.35816442
YAF2	4.34444617	4.65542848	4.63400664	5.39220416	5.26826058	5.26041055	5.40723416	5.47927746	4.81992732	3.96475137	4.52976467	4.85622957	4.78325464
ZMYND11	2.66538459	2.66724163	2.03893687	1.729689	3.32256595	3.88531787	3.1865172	3.39408732	1.09177704	3.42141387	1.72764145	4.95665374	0.02888581
WAS	4.46380538	3.4714792	5.21239323	4.13655302	2.26323399	1.73130868	2.23135182	2.06540894	3.74732286	4.20155537	1.24375561	3.14288574	4.12252667
DPEP1	0.20561021	0.81901108	0.20817312	0.20561021	0.20561021	0.20703515	0.20561021	0.20887989	-0.11807874	-0.11807874	1.64193299	-0.11807874	-0.11807874
BID	2.72484974	3.17940603	4.05587075	3.66476448	2.12218538	1.81883881	2.23019473	1.9344915	4.04035672	3.4099433	3.07973187	2.60557037	1.17850348
MATR3	1.24349596	0.77661908	1.3592248	1.1514807	1.94786039	1.37746608	2.00451564	1.90579547	-0.1219854	-0.1219854	-0.1219854	-0.1219854	-0.1219854
NPC1L1	2.56615597	1.26847149	2.42689761	0.90137944	2.34567777	2.35876905	2.77160861	1.38198513	0.28982343	2.42573677	2.22970649	1.88391661	2.00615364
XYLT2	5.37476705	4.5926298	5.31268064	4.68062816	4.18516578	4.64847717	4.55938696	4.25996546	4.47592781	5.17393955	4.24968479	4.88474426	4.3773742
RGPD5	4.54847906	4.49787839	4.04305334	4.46936899	5.15518779	5.29354668	4.97416651	4.9379156	4.9540653	3.62913105	4.82984687	4.09501456	4.44666164
STMN4	1.54998294	1.0582983	1.63262155	0.71807703	1.41952293	1.74748668	1.30694966	1.71343769	-0.57447236	2.90040644	2.93079437	1.81402402	2.82083974
NUDCD3	6.48158993	5.97257281	6.2818882	5.48712706	5.99525268	6.0640946	5.91977421	5.9645389	5.96745922	6.54991099	6.11920222	6.2273052	5.98100921
ISL1	1.26796444	1.09206127	0.77219684	1.42674028	1.06721385	0.77104808	0.76960963	0.77291031	3.08320148	-0.35192141	-0.35192141	1.93840463	2.82883513
CHDH	3.31352239	2.10433046	1.95392665	1.48708318	2.62089389	2.29812305	2.82599265	3.04147735	2.41740427	3.29811886	-0.11315996	2.6498268	3.99071695
IL20RA	0.79113153	0.83469509	0.7183466	0.91670816	0.86184474	0.71743378	0.79523984	1.20711766	3.44068561	4.32877569	-0.42200275	-0.42200275	3.94282931
GLT8D1	5.02955153	5.56086481	4.98930329	6.05024181	5.28439985	5.38680714	5.27473123	5.52365195	5.62286139	5.3093243	5.86569664	5.38244679	5.49207399
ATP2C1	2.59498036	4.06729237	3.12560096	4.60831723	3.8952771	4.07623091	4.32961721	4.32383342	4.00347034	4.19968823	4.29391181	4.61235395	4.11464388
IGF1	2.36998664	5.42451283	1.84069712	5.97267372	3.54177282	3.5261258	4.65064418	3.70141472	4.16029842	3.92208305	4.52273963	4.60321637	5.87095354
SLC38A5	5.68898758	2.6060662	5.41110092	5.93293673	2.72689274	2.08076294	2.42566256	2.05165843	2.17112322	2.90668273	1.49402675	2.86084297	2.14801905

RALBP1	6.41562859	6.76182196	6.55967197	5.80570305	6.25556561	6.33350966	6.59285391	6.28733103	6.75910182	6.93060758	5.98703081	6.17576426	6.5085849
RUFY3	5.69603403	6.0134299	5.64621642	6.48635433	6.17878676	6.33477129	6.72643367	6.42243581	6.17665423	5.42501096	6.32297663	6.20510908	6.33361689
CNTN1	3.64469775	1.5284561	3.74902288	1.53780911	4.66667801	7.00117996	3.5951585	4.01124338	4.22357887	4.46536623	5.16755881	4.96890249	4.34466968
SLC11A1	5.28507763	2.6482261	6.13226781	5.34198143	2.00573607	1.71838152	1.83954637	2.41876473	2.54084769	2.15210848	0.6650728	-1.53122775	2.92767942
WWTR1	8.47478833	7.87675029	8.73998461	8.14279171	7.48103081	7.16713277	8.25009036	7.80496877	8.1327824	7.6121848	7.97798792	7.64220576	7.20280943
AGPS	4.83056458	5.67901111	5.19942489	6.25734064	5.88648025	6.01306922	5.9566617	5.77288741	6.07307946	5.09665009	5.90322285	6.09683862	5.28824416
CXorf56	4.71456784	4.92627125	5.08798799	4.41284866	4.67665078	5.79012281	4.80009104	4.97204077	5.14209383	5.72414065	4.49253881	5.24066147	5.34445593
ATP1A2	6.22419948	4.91958689	6.69605972	3.82331845	6.41182711	7.96001225	7.74003032	7.82903915	5.81529136	8.19041506	5.73864568	7.59889232	7.23905386
TTC27	2.9635025	3.77653595	3.41886942	4.07310512	3.89864216	4.29614776	3.79914388	4.35317032	4.0477294	4.71079629	4.15853564	4.78128881	3.63245409
ZNF582	3.08570529	3.2952471	3.07513276	3.29478464	3.9034631	4.14474514	3.83203912	3.97045269	3.71614199	2.6321904	3.74049854	3.05756357	3.47069507
VSIG2	0.20964745	2.92020285	0.18338337	-0.13819626	3.32448039	1.18137297	1.97850694	2.88007873	2.45064947	-0.28241947	-0.28241947	-0.28241947	4.16624491
PHLDB1	6.56605795	6.30618591	7.15502537	6.63899358	6.70819404	5.89442598	6.6076793	7.40950487	6.4133138	6.95675962	7.08673463	6.78754412	6.92710322
MARCO	4.63204029	2.71283702	6.04067342	4.59262151	0.75544279	-0.34909452	1.21310254	0.78553116	1.3421322	2.62161166	-1.29107446	-1.29107446	-1.29107446
PRDM11	4.44148468	4.09740518	4.17521518	2.42732876	3.99006394	4.36931253	4.07985993	3.93398766	4.63247972	3.21731512	3.64725436	4.70096468	4.72574936
SNAI2	6.77573908	4.73083494	6.39285704	7.71367653	4.36058431	5.65573269	5.0136634	3.6738127	4.67484133	4.22032666	4.23213037	3.43885298	5.6329018
CD74	9.19476971	9.24405368	7.92680592	9.50703811	8.08717976	6.78613208	7.38744889	6.69152468	8.81476725	8.79384615	8.24919808	8.18020472	8.68232962
HGF	6.59648279	5.39641739	6.32237261	7.90305786	4.58922761	4.26903825	5.1772886	4.67725751	5.40558617	3.75081879	4.42457888	4.59385564	4.57711205
ZRANB1	6.32060898	6.35201084	6.29976377	6.14447115	6.77469434	6.84045246	6.77880067	6.79299931	6.49643892	6.27564889	6.37920035	6.48109047	6.90623759
NCDN	5.09057024	4.47546182	5.25802286	4.57257441	4.11105191	4.08081232	4.34992551	4.09685541	4.45240179	4.40446055	5.03871444	4.33962646	4.44563728
ZFP64	4.05428084	3.21026898	3.82260149	2.76402659	2.88247416	2.67860303	2.99146364	2.76575934	3.43753108	2.59926271	4.11847907	3.22396912	1.95443323
MNAT1	3.06416656	3.22344709	2.79618821	4.17514831	4.08416612	4.22783311	4.03965389	4.06736801	4.07355413	3.54493431	3.09907763	4.17546242	3.80519822
SAMD4A	5.10543054	4.79376491	4.7533149	3.69929461	6.54228881	5.92449686	5.38864392	6.5627629	5.09354119	6.33317387	5.9288785	6.43305785	5.63237791
RUNX3	4.23399567	3.03008877	3.11934723	2.84111215	2.70115817	1.93155061	2.20219877	1.68183452	3.0173761	3.50640552	2.48131092	2.92380469	-0.310072
PLEKHB1	4.26306345	4.07205853	3.56196195	2.78844399	3.72845002	4.76087621	4.46089943	3.87059332	3.91354373	4.78570018	3.99377663	4.73729257	4.19933861
SERPINB1	6.11374107	6.73979364	5.65885897	7.07556521	5.62920614	5.98656075	5.68404027	6.05510459	5.74549717	5.60939316	5.09793922	5.56338252	6.09236548
CYP3A43	0.22423539	0.06792077	0.33084788	0.02806045	-0.0818289	0.17012823	0.02848453	0.60501056	-0.08460164	-0.08460164	-0.08460164	-0.08460164	-0.08460164
SLC7A9	0.51947948	0.38455666	-0.0538102	-0.2238277	0.11592705	0.90906015	0.25444467	0.50568585	-0.04765657	-0.04765657	-0.04765657	-0.04765657	-0.04765657
SPAST	4.38516914	4.56375034	4.54616411	5.44678658	4.92329436	5.18943743	5.10081663	5.30829579	4.93260238	5.07491602	4.95379612	4.73855922	5.49413866
NRXN3	1.36216399	1.59667429	0.87517129	0.85562145	4.40990411	2.7977161	2.04917117	2.05758807	0	0	0	0	0
OSBPL5	5.95271909	5.23256982	5.88752429	4.71510596	5.40193197	5.66458655	5.85896638	5.60856673	5.14350537	6.07023399	5.3934281	5.71009313	5.37592256
AQR	5.71166229	5.97288599	5.42923541	6.2052272	6.17771737	6.34271317	6.09530915	6.01247317	6.05468218	5.92307095	5.91651467	5.71815314	6.14358034
CPS1	2.94474999	3.62688213	3.73009382	3.73443318	3.38724345	3.91924714	3.52791157	2.37261917	2.37372847	3.73405958	3.64997359	3.05729856	4.45590196
FHL1	7.56371102	9.20195452	7.75562834	7.2521205	9.66225489	9.54518581	9.54518581	9.6009221	9.30557516	9.53642175	9.24913165	10.0951452	9.24913165
NLRP2	0.23425083	1.0970063	0.04728772	0.19903568	0.43578778	-0.06744868	-0.1658314	-0.1628613	2.39684165	-0.07246398	-0.07246398	-0.07246398	-0.07246398
SLC45A4	5.22305971	5.11478382	5.25965544	3.77482583	4.72517743	5.09845868	5.05034852	4.48049192	4.25753668	5.643797	5.55352304	5.0160463	4.81274154
RNF10	6.86551626	6.73620505	6.68435668	6.16072494	6.21853627	6.10242648	6.40860089	6.35414874	6.24070749	6.53800565	5.92599929	6.48725946	6.50090325
ZNF839	4.56974854	4.38908598	4.48985846	3.84000584	4.26384896	3.79975548	4.25105495	4.39494104	4.56947622	3.30784011	4.43463683	4.69839374	4.12510932
ZDHHC6	4.93350578	5.27976537	4.82608571	5.64204604	5.49786826	5.53256687	5.55074086	5.61149232	5.63142369	4.90699059	5.76895686	5.42158332	5.55846685
GRAMD1B	1.7489945	1.12090608	1.53345593	1.51923187	0.99521675	-0.24319491	0.64544121	0.57183816	2.72053334	-0.38382317	2.70645476	-0.38382317	1.81219004
RNH1	5.18583144	5.2944764	5.89886111	4.72651827	4.48087674	4.66278563	4.42880441	4.67143136	0	0	0	0	0
NDUFS1	5.80228417	6.11169955	5.73943958	5.94380282	6.00747926	6.44618105	6.12286203	6.05421723	5.92472041	5.80937665	6.15562544	6.24243061	6.80004218
RB1CC1	5.77448938	6.49307256	5.84194377	6.68524865	6.71635497	6.99212414	6.86648569	6.94581213	7.02898239	6.03420316	6.16524378	6.56884365	6.15950617
ERP44	4.92149077	4.88804056	5.26965605	6.09242257	5.77503057	6.1358868	5.87909492	5.57210098	5.83285641	5.31785369	6.09725069	5.42164337	4.8338195
ALAS1	5.48392895	5.3703791	5.66398581	5.2419685	4.767758	4.99364493	5.06033401	4.80200227	4.11424197	5.28469579	5.30969561	4.77041652	5.51700166
BIRC3	4.47895574	5.34034086	3.95720901	7.33268193	5.81436958	6.30289324	5.19906761	5.15781815	5.35652695	3.68008444	4.96140892	3.6293937	4.3217127
AKAP11	6.18925157	7.12145167	6.53579059	7.18338553	7.22964915	7.25749432	7.23623334	7.18008657	7.10316296	6.8164137	7.10120086	7.29425352	6.97105464
GLRX2	1.61414604	3.35981335	2.99199876	3.49681171	2.92939506	4.08025043	3.28577712	3.19080622	1.40851051	2.167493	2.34502391	3.27707604	3.09658228

SNAPC1	4.23527463	2.87752072	4.44471951	4.74995049	4.14442798	3.98637316	4.17212597	3.44540087	4.25982232	0.60994757	4.09241671	4.45931409	3.17728591
DERA	3.7911058	4.20113767	2.93898762	4.64509922	4.11037053	3.95383712	3.69328472	4.2226139	4.29935917	3.5480953	4.26159165	3.06723042	4.3701221
STRAP	5.46321219	6.77576192	6.47521573	7.584574	6.81550311	6.77532443	7.06417982	6.7581447	6.49617316	6.15056725	6.35544261	6.43269305	7.14219367
ABCC2	0.6777054	-0.00462189	0.73112936	0.57736552	0.95578614	1.01382532	1.1786743	0.41007332	-0.18258358	-0.18258358	3.63105126	-0.18258358	-0.18258358
DEF6	3.533267	3.44765437	5.10604286	3.76676253	2.3866551	1.28560409	1.75133184	0.93923175	4.30509069	3.65382862	-1.08282115	3.05797518	3.01748156
PLEKHO1	5.05641687	4.78182599	5.43143904	5.17179908	5.83898262	5.10065652	4.90360665	5.79738659	5.51122117	5.63955935	4.9923839	5.37222771	5.28579255
GCLM	4.6607905	4.36326353	4.66531559	4.4173058	4.29325803	5.10485456	5.12987488	4.82791815	4.60997791	3.80847709	4.84594348	4.32923494	4.50233314
UBR2	6.14384684	6.56037747	6.12046369	6.66268401	6.80416254	6.83736739	6.80664757	6.88538802	6.87413668	6.67332912	6.50091993	6.43529455	7.08183983
EHD2	7.22802522	6.9919405	7.94227069	6.66470214	7.00361184	7.01778275	7.14484363	7.05884126	6.86225591	7.85901488	7.2857635	7.33602492	6.62499923
DEPDC1	1.36671626	1.13214045	2.13828287	4.3677446	1.20365755	1.2136827	1.79640101	1.24784063	3.07443981	3.11325897	-0.86224103	3.16060448	-0.86224103
CCDC28A	3.2326263	4.30240285	3.70926221	3.86460893	3.42315592	4.15610644	4.70009223	4.29770657	4.26035069	3.81941884	3.66128587	3.52790771	2.97325498
RRAGD	5.65641316	5.33334475	5.36396994	5.0816663	6.14762434	6.45625316	6.01039735	5.38176739	5.81663939	5.15171055	5.13541458	5.39389001	6.43209465
HSF2	4.23619173	4.34944174	4.26073804	4.76038232	5.19794732	5.16000158	5.20063352	5.2019994	4.97764458	4.87016754	4.83222414	4.54850009	5.16454105
PHF20	4.37648419	4.24043205	4.13339332	4.08440847	4.24743787	4.67350264	4.41475194	4.60431824	4.63288195	4.36130295	4.49029182	4.09041767	4.15927045
HSD17B6	0.45337795	2.43192175	0.30703644	0.2797941	1.34086791	1.17376688	0.50662116	0.0341882	-0.52291725	-0.52291725	1.30876687	-0.52291725	3.17070938
NR1H3	5.32090173	3.70761865	4.54964236	3.88380928	4.12317431	3.84455121	3.71833663	3.78010646	3.91177956	4.39765752	4.2462594	3.55541303	4.60391957
TYMP	4.90784547	4.47770668	5.25861704	5.11422037	3.20848207	2.82942159	3.1002646	3.12862258	4.6272569	4.25001059	4.24235899	2.54014255	4.65054576
NCAPH2	4.55096566	4.77985593	5.62728499	3.76384804	4.15024152	3.84145136	4.09813783	4.50147552	4.13216152	4.8314928	4.60435093	5.23756196	3.79332241
TOMM34	5.08923511	3.87870879	5.58920876	5.12427383	4.03610331	4.77415233	3.64030509	4.09129142	4.9926932	3.45888468	4.78740089	4.35533437	3.51559676
SEC63	6.63673215	7.05470116	6.50116128	7.45222136	7.16864127	7.05948737	6.88039263	6.82082409	7.12859641	6.61218802	6.64715732	6.66389787	6.75932024
KPNA6	6.76526969	6.60472457	6.31390225	6.39518998	6.81768879	6.75497227	6.81503118	6.53024289	6.15262167	6.62339282	6.627728	6.49026144	6.71508041
VIM	9.89992815	10.4805718	10.9537353	10.2258614	10.4805718	10.6421203	10.6421203	10.4805718	10.4546959	11.0689905	10.303941	10.8410635	10.1373867
FAS	2.5583868	2.5583868	2.70515648	5.0357932	3.76455333	2.52766597	3.39437842	2.32631715	3.03588201	-0.79333833	2.3847245	0.52925976	3.70558774
RNASET2	5.04590794	4.48523415	4.60334182	4.56629081	3.92561845	3.11728345	3.96376778	3.28888008	4.52546661	4.14230109	3.27076608	3.61966137	5.09956747
CD44	8.10913079	7.74426316	8.26037632	9.11696309	8.75151409	7.6576673	8.48108053	8.44332694	7.40392007	8.1961572	7.93580917	8.47794419	8.69498479
KCNG1	0.47802413	0.25981566	0.42661272	0.54261733	0.27037841	0.13774139	0.8117985	0.13958828	1.34561173	-0.22048246	-0.22048246	-0.22048246	-0.22048246
AGPAT4	4.82764282	4.91692323	4.17801861	4.30559709	4.70059565	4.44502722	4.38592392	4.88260534	4.60798635	4.56874054	4.4081475	4.6434511	4.6749687
SLAMF7	3.87281171	2.64313921	2.19786005	2.57552228	1.0638323	0.65259588	1.35368426	1.09563886	3.38428851	-0.83571138	2.99676445	-0.83571138	4.13136746
BTN3A1	5.16907122	5.66505099	4.62615554	5.58498406	4.82042708	3.84878916	4.7057454	4.16247955	5.32882594	4.22043046	5.16768516	4.40709274	5.32524531
MIPEP	1.69439283	1.74572105	2.50476361	2.20309898	2.09410895	2.28787447	2.00278628	2.07450513	1.13159626	2.05675663	2.88287082	1.82264082	-0.36270175
PRKCH	4.21398005	5.30059401	3.33152514	4.89878294	5.04522952	3.78898804	4.77346658	4.28732822	4.75539852	5.02566814	5.59811831	4.075233	5.78532317
INSRR	0.1442425	0.22939946	0.14680617	0.60852511	0.1442425	0.14566786	0.1442425	0.23460998	0.05200523	0.05200523	0.05200523	0.05200523	0.05200523
IFNGR1	5.42746489	6.69016085	6.0836266	7.55299007	6.4585094	6.77135799	6.69659217	6.60802194	6.34177815	6.23173916	6.0679046	6.07446562	6.3886413
B4GALT7	4.47820999	3.96025879	4.67210974	4.40661893	3.93039508	3.22066761	3.33401567	3.69252299	4.25294885	4.11152265	3.11744875	4.15655029	3.32955836
SH2D2A	2.4606088	0.21914054	0.62911193	1.88938657	0.88897402	0.48135525	0.39217876	0.06034685	-0.29527678	-0.29527678	-0.29527678	-0.29527678	3.48209996
VRK2	4.61014954	5.12979474	4.2611956	5.56476624	5.2616565	5.44875536	5.29269289	5.29284336	5.29378059	4.13404502	5.17422113	5.05228609	5.28027278
TNFRSF1B	2.77954781	2.3831494	2.85192863	3.40830518	0.95830028	-0.44481871	0.75366401	0.06285934	0.22578631	0.22578631	3.98250777	0.22578631	0.22578631
VEZT	5.86741462	6.25796246	5.69742301	6.32401209	6.49112252	6.70206492	6.47376453	6.84065405	6.38662805	6.97256719	6.35826255	6.428033	6.71608347
POU2F2	3.46355094	3.53244758	4.02015712	3.23074513	2.11893396	1.41480756	1.79139404	1.78993423	3.87508593	3.3988059	2.42714451	3.75168355	3.05593066
BRD9	5.22076291	4.73284275	5.44318902	4.86320577	4.78813325	4.82453281	4.74030173	4.93334977	4.66554242	4.69329447	5.58936859	4.80375073	5.26530418
SNX1	7.05928617	7.14628861	6.8745683	6.2887177	6.8667838	6.79967017	6.86067319	6.87311316	6.80359838	6.9671287	7.14791144	6.95009214	6.98272206
TBPL1	3.44642878	3.81178527	3.38783118	4.08107819	4.08278855	4.9352493	4.27606084	4.47224154	4.51511876	4.34047177	4.20104223	4.06838951	3.01441464
ARNTL2	4.5283061	4.2270203	5.47644418	6.36734937	5.66431267	4.43768392	5.02858124	4.79979479	4.36515772	3.37618246	5.27532046	4.24185073	4.45366017
BCLAF1	6.24154322	6.90697155	6.46336308	7.58785168	7.30913818	7.58544834	7.35515106	7.29199357	7.35926348	6.79976051	6.6586774	6.97420294	6.98816569
SLC39A9	5.6448297	6.23008064	5.81969005	7.08329005	6.37308554	6.53895781	6.43040334	6.25323316	5.97316754	6.33820106	6.35645033	6.47543124	5.78618104
ANK1	3.42835746	2.78003337	3.8049076	1.41700392	2.3951168	3.54069019	4.17377127	2.14796342	-0.36486842	3.6738038	1.53254804	3.46399935	5.44939794
IBSP	3.09338506	0.57227824	4.18099353	3.12356028	0.24900233	0.26473625	-0.02997841	-0.02738571	2.2641115	-0.60445354	3.63583612	-0.60445354	-0.60445354

TFB1M	4.15115285	4.18766513	3.51756665	4.15797137	4.25473627	4.49450003	4.48039531	3.90754552	4.91202812	3.86866418	4.47096592	4.05807369	4.07175929
RABEP1	6.34180083	6.48645687	6.17980023	6.77419883	6.8914343	7.22301576	6.70829395	6.91919493	7.07068271	6.84318388	6.44371083	6.60246617	7.04797867
HMGB3	3.38379486	3.03490642	4.33345137	4.88421195	3.06572443	3.44712089	3.36503972	2.92364827	4.05698804	3.6699313	3.58637359	3.69765708	3.99657879
NUP160	4.5312416	5.23394985	4.62954776	5.48433313	4.95870689	4.82898906	5.24647367	5.1900755	5.30925549	4.95297922	4.99977687	5.02229447	5.53646517
BAK1	4.01524309	4.0159468	4.5966704	4.44570279	3.14290496	2.93036724	3.50718863	3.15537851	2.81556932	4.09011464	4.51439056	4.51082021	1.02310193
MUSK	1.27150333	0.186733	0.38576545	-0.12698869	-0.0722693	0.05814633	0.22553984	-0.38256137	0.04561795	0.04561795	0.04561795	2.38573417	0.04561795
IKZF2	4.53236907	4.72151102	4.06160527	4.71986696	4.89245391	5.48882203	5.39573336	5.49058108	5.24984983	3.67755776	5.42898199	4.46581175	4.71512986
GRN	6.95053041	6.50225465	7.3624697	7.10015253	5.72997845	5.69521385	6.10994541	6.07161198	5.64096341	7.04830178	6.7556023	7.08598548	6.02625779
FAM13B	6.35813453	7.29001982	6.72417453	7.00292494	7.49449922	7.32758655	7.5674301	7.86299754	7.33527795	7.19290179	7.32985422	7.64088373	7.36550779
ARHGAP31	3.88362202	3.51395387	4.00244042	4.02838998	3.06417783	2.96144131	3.49318072	3.19706738	2.56372409	3.66784401	4.13020532	1.54375131	4.49744076
CENPQ	1.68101255	2.90528805	2.59148257	2.79253579	3.35953575	3.95388627	3.23969594	4.26567205	3.84158549	2.75886741	3.07401736	3.05114613	3.48789101
SARS	7.14247103	6.55927273	7.01124085	6.66402808	6.34265128	6.88159352	6.40381289	6.88159352	6.71738803	7.2143787	6.57568246	6.36521886	6.73209234
RANBP3	5.72750779	5.30791617	5.79941086	4.8569395	5.36511821	5.34474656	5.16002606	5.35327948	5.48982348	5.62475379	5.69199314	5.39279222	5.53087871
ARID4A	5.23688106	5.73483362	5.38667229	5.95895482	5.97396956	6.70217412	5.73121151	6.05285209	6.65879342	5.92806588	5.50567982	5.76848714	5.81826069
PNPLA6	5.53271363	5.451445	5.96635997	5.10691065	5.12997828	4.43049638	5.84963881	4.95182168	4.82276391	6.01611689	5.51530863	5.36355152	4.37628671
IFT88	3.8273017	4.42577344	3.80883929	5.05280082	4.74424307	4.37676028	4.59837344	4.41027924	4.55232815	3.36070176	3.96497722	3.58447883	4.09233773
ALG1	4.53294	4.23282504	4.89714227	4.58713909	3.74070403	3.57652529	3.98937349	3.62042564	2.65755836	4.08586321	4.60777387	3.7952369	4.51160221
ZCCHC8	4.92014638	4.29634832	4.48861776	4.55098732	5.10506507	5.17849785	4.98159983	5.21155437	4.90648665	4.76301124	3.97450999	4.47611672	4.92919437
ABCF2	6.16492903	5.40740614	6.02461285	5.68065044	5.26178142	5.31666683	5.5945122	4.85839585	5.24078399	5.28438097	5.23470946	5.12449117	5.48660388
CHPF2	5.8509756	5.44849446	6.16880566	5.95702711	5.52561036	4.48516008	5.27103984	5.40784862	5.10772858	5.579817	5.99755241	6.31191013	4.52646781
LRRC7	0.04728646	0.04728646	0.04985548	0.04728646	0.20341227	0.12730568	0.10954	0.51253794	-0.05896979	-0.05896979	-0.05896979	-0.05896979	3.45371418
FUT8	2.37224138	2.97679464	2.54376472	4.04201361	2.28133738	2.98719932	3.1454466	3.00500111	3.66300949	2.73691608	1.35497319	3.92354097	1.7006589
UBA6	5.02296607	5.75815277	5.2240261	6.57543284	6.19384228	6.19741553	6.12584973	6.31771768	6.07540335	4.84220603	6.11565984	6.00223204	5.00048164
GAB2	7.55304033	7.00330033	7.5990666	6.31887167	7.07655874	6.66484888	6.95357643	7.50021355	7.07863011	7.64468529	7.00843863	6.83547479	6.86404384
ATP6V0A1	6.10006126	5.44209653	6.00584201	5.52636031	5.68649924	5.41439904	5.55079185	5.42262429	5.28267529	6.01633721	6.12047832	5.60568694	5.66436542
PIAS1	6.26794672	6.15612255	5.80235989	6.2956192	6.61007219	6.98694299	6.65047768	6.65887193	6.5064789	6.28568182	6.24348435	6.67390534	6.18465395
SLC4A7	5.11793321	5.86776356	5.53700704	7.69269879	6.28085088	6.20778728	6.708981	6.93489539	6.25250728	5.60722282	5.87629341	6.85640156	5.89565254
APBA2	2.21417574	2.62584122	2.13001142	3.37041283	2.25732968	1.73129643	2.337917	2.18262124	2.34004757	1.40192948	3.23959411	2.90213657	1.68903305
MAP2K3	4.5883226	4.9729973	5.54651169	4.79235317	3.91808585	4.47876167	4.64719208	4.42345493	4.40802421	4.55603674	4.99950579	5.01507967	4.89710937
EFCAB1	1.72324381	0.37194392	1.27871702	1.10603231	0.5168351	0.74417164	0.83843554	0.83883801	-0.49384617	-0.49384617	-0.49384617	1.62062009	-0.49384617
TMSB10	6.71954403	6.55025857	7.55628135	7.35241312	5.82688762	6.10192965	5.4628556	5.91282106	7.1416892	6.84752848	6.39254615	5.76207842	6.97217184
ASTE1	3.95389431	4.24839258	3.69588599	4.34537894	4.3410564	5.06201469	3.7515062	4.1413823	4.23404804	4.96624054	4.31416066	3.87177699	3.34825169
RNF19A	6.00005832	6.69279132	6.0109771	6.64132205	7.07254779	7.33991692	6.80315551	7.27214031	6.95214304	6.55686285	6.63025615	6.952863	7.02055627
PEX3	4.56493192	5.1604888	4.33142684	4.394119	5.02209647	5.25292125	4.69383109	5.2270166	4.81801233	4.87553536	5.22492664	4.63417598	5.32450945
GABARAPL	5.92928371	6.55090905	5.52863801	5.74696177	6.57325262	6.52596916	6.57482274	6.87944436	6.9514071	6.88170284	6.88930254	6.87287567	6.7764482
MYOC	4.43689956	4.7832419	3.79844573	1.46432139	4.49272487	6.70316589	3.23749371	4.96184622	5.14522405	6.02081836	3.37703191	6.37929625	4.98781728
SH3YL1	4.25289616	4.37788077	4.1395662	5.01150679	4.52134658	4.57336532	5.28998356	5.54974389	4.83607486	4.98685935	4.96075827	4.51750496	5.14357815
FAM136A	5.17975069	4.456869	5.4399374	5.44786266	4.63777923	5.4172203	4.9492708	5.17306049	5.04391013	5.43781795	4.72003779	5.56198315	4.9708972
VCL	7.93623046	8.09192327	8.11267021	7.7135759	8.64305049	8.75668543	8.68681259	8.71704754	8.12767721	8.64801979	8.35610846	8.77900046	8.77052505
DEPDC1B	0.10305971	0.50058985	1.0460335	2.38965127	0.57791544	0.2561357	1.34995731	0.28545256	-0.4466451	-0.4466451	-0.4466451	3.55840743	3.0104629
DAPK2	4.78189572	4.02328595	3.95664749	3.09797127	3.63567281	3.4064662	3.00371754	3.34636582	3.57333118	3.36194341	4.21054417	2.16933266	5.45455779
NSMAF	5.14205587	4.86743971	4.3485129	5.36299453	5.04153072	4.82050208	5.2519048	5.22519549	4.81296627	4.40377539	5.11508571	4.93427117	5.00154788
ADSS	4.61902054	5.40783434	5.2969999	5.95931722	5.75464394	6.09017006	5.7961669	5.75559062	5.98791463	4.94555225	5.58758048	5.85293063	5.24082855
STAP1	0.49486278	1.09558978	0.88902601	1.09025676	0.91873817	0.43781292	0.43638463	0.74939879	3.65440437	-0.33502716	-0.33502716	1.47490346	2.54397832
TIMP2	8.96282536	9.02029137	9.02029137	8.39052573	9.48727987	8.96282536	9.26563832	9.61459517	9.05258172	9.2277159	9.2277159	9.40529962	8.93955632
RFC1	6.40166816	6.64090621	6.16465227	6.54699554	6.52852681	7.08078367	6.22285853	6.02795193	6.6454393	6.5479915	6.10814915	6.19599628	6.24659512
TBC1D23	5.51237883	5.60110119	5.07800664	6.61945034	6.02224056	6.00427527	6.35602036	6.26071955	6.36843965	5.06082134	6.23563248	6.0014098	5.25596988

CUL3	4.63235096	5.33650049	4.52531556	5.50995426	5.40772023	5.68983498	5.46278761	5.51047335	5.42470324	4.59828406	5.39527505	5.75092488	5.10642251
MYOM2	0.59795916	0.51791524	0.7162741	0.52118888	0.68955668	0.90961528	0.52037516	0.66189474	0.1645069	2.28754674	2.98705936	2.97737265	9.12303992
OTC	0.6507688	-0.04172716	1.26302534	0.164802	0.05117476	-0.040372	0.24279061	0.24515918	3.44224711	-0.03088929	-0.03088929	-0.03088929	2.75123827
CYP46A1	1.83832491	0.99402913	1.33577396	1.19745048	1.16133952	0.97603991	0.74654032	1.26629333	-0.45413681	-0.45413681	1.4902577	3.46099977	3.19431851
ZZZ3	5.04758159	5.48365156	4.77562003	6.00193258	5.7423392	6.02117878	5.97039741	5.7337276	5.60474045	4.6220158	5.19461866	5.5887463	5.38278135
USP2	1.43470579	2.56259643	2.11357902	1.70786705	1.4006458	1.55627077	1.20520546	1.66690934	-0.76155606	1.67150578	2.07381533	0.53279171	5.94574453
TUBG2	5.7709962	4.21272255	5.60077758	4.10470717	4.94108931	4.58077372	4.98426207	5.15139192	5.76168004	5.62727495	5.20983319	5.22162272	4.82345736
RPL26L1	4.17308498	3.26687457	3.38703681	4.31962567	3.51391679	3.93686518	2.37898566	3.29534466	5.10944244	4.78724067	3.71697922	3.22632534	3.76752924
FLT4	0.56297849	3.9542789	2.65123501	3.73497071	3.67020033	0.93242695	1.88675325	2.05683184	3.27418382	2.61663777	5.04120416	2.64716575	4.37766884
NSUN2	6.42193316	5.89442737	6.28688143	6.32396557	6.0712826	5.52770682	5.91281294	6.64284516	5.84017065	5.81188819	6.18240324	6.03326974	6.33411889
FBXO42	6.41505526	5.63662785	5.77129302	5.13928029	6.31290967	6.1311392	6.4913114	6.67458941	5.73656183	6.72597608	5.93682494	6.35722793	5.93055577
MFAP3	4.97036316	5.69828302	5.08327475	6.28171331	5.8000839	5.62668968	5.65844295	5.47947047	5.77054792	4.99934144	5.9410199	5.74154269	5.86364704
MRI1	4.74083709	4.81996051	5.22212613	4.27819095	4.25041854	3.61021324	4.6221649	4.2571678	4.76640093	5.28028247	3.72196814	5.00646579	5.2792395
METTL1	4.24904117	2.9135634	5.21472465	4.50990362	3.09410528	3.13335895	3.29621634	2.91307103	2.53388309	4.34056243	2.24487886	3.61943227	3.27527403
HOXC8	0.20650238	0.16759134	0.17007944	0.16759134	0.22999457	1.1460401	0.16759134	0.17076557	1.73286025	-0.17178429	-0.17178429	-0.17178429	-0.17178429
AGA	4.2238158	4.86832019	4.14476427	4.63317157	4.05524748	5.31129191	4.12011954	4.12270233	5.14786087	4.47858454	5.24020702	4.68818975	5.27897157
PI4K2B	3.75075528	4.20654607	4.46792354	5.89255602	5.303266	5.22235327	4.81315205	5.61412464	5.15268446	3.78799552	4.35753572	5.193942	4.48678109
MAT2B	5.36571951	5.41096991	4.97168296	5.62602855	5.58050541	6.30158178	5.87083947	5.69239523	5.51190832	5.22105372	5.84134415	5.99580766	5.07060762
TLL1	2.39817644	4.55154965	2.35089596	3.20117892	3.5032418	1.93187584	3.16284567	3.34621941	4.17879429	3.90832213	4.61564634	2.30466915	4.82825719
EDC4	5.52749196	4.81305476	5.64373777	4.90888738	5.08827247	4.60345645	5.29682249	5.17009117	4.99622566	4.97788415	5.35863909	5.30295301	4.99597803
TRIO	6.42686711	6.70886504	6.45346104	6.44861301	7.20218844	6.58024337	6.67399982	7.36916525	6.56041067	6.38899844	6.86249218	6.73649374	7.3875125
VCAN	10.4034201	9.94569789	10.2975386	11.3233651	10.0038307	8.8472668	10.0704872	9.68830287	10.2041802	9.184985	10.5975696	9.32337687	9.32337687
CLEC16A	5.14639231	4.58064473	4.9354837	4.30648074	4.86433057	4.1294224	4.55692411	4.64226827	4.3677984	4.86542494	4.68711414	4.77852461	4.68539751
MSR1	6.09655333	6.28932346	6.62191168	7.59168547	5.5031347	4.68856381	5.32465641	5.07842929	6.21015644	5.26948788	4.78747891	5.45233063	5.22080715
CDH1	1.73758665	0.64088405	-0.08132843	0.93144187	-0.08342256	-0.08225825	-0.08342256	-0.08075094	-0.10230911	-0.10230911	3.30285744	-0.10230911	-0.10230911
SKIV2L2	4.94335078	5.7476818	5.25322101	6.06013216	6.08241491	6.26744209	5.98941607	6.11170926	5.64168167	5.01423131	5.47633617	6.18139641	5.45209218
DNAH5	1.64251952	1.82227563	1.70008847	1.7222561	1.99520574	1.66818643	1.87631326	1.80890946	3.51204957	-0.91905756	3.49340622	3.55556358	2.81579915
ZFYVE16	5.35878986	6.12819884	5.64248742	6.87164322	6.4855175	6.55386059	6.64080991	6.59463329	6.56184052	5.78177088	6.23277266	6.38480651	6.34074049
C6	2.4380423	4.08862045	1.41652298	2.26113022	2.87864147	2.89080209	2.28077649	2.91163282	4.07035517	2.21427417	4.69114688	4.35923913	6.61903798
RAI14	6.18679113	6.77925785	6.46453219	6.83402594	7.59845618	7.39849305	7.42880596	7.50439296	7.39793687	7.755216	7.18246981	7.34298547	7.3463068
SOX30	0.24607906	-0.04606027	0.20473246	-0.03592455	0.5501879	0.22890412	0.10381194	-0.04286292	-0.06682993	-0.06682993	-0.06682993	-0.06682993	-0.06682993
PNKP	4.36375373	3.91483249	4.97102398	3.92895333	3.92118809	2.73906177	3.98485108	4.35814088	4.05515115	4.30791616	4.19964565	5.03332975	3.26360881
PHLPP2	5.26909767	5.50050873	5.14235579	4.76303143	5.53269694	5.15233325	5.42903972	5.60687541	5.32523671	5.59033457	5.76658271	5.53746992	5.32432249
STAU2	4.96375744	5.40471015	4.86976716	5.70872015	5.6717586	6.2580738	5.84503452	5.81809959	6.00541568	5.95262101	5.83604742	5.92336143	6.13075553
PQLC2	3.69998196	3.22387155	4.78180132	2.5627968	2.10080723	2.80783445	3.4613484	2.05300891	-0.18793705	4.49457842	3.86260732	3.80232202	3.38202124
CTNS	4.5050802	3.97860461	4.41661522	4.25781176	4.34912679	3.44613571	4.35235423	3.66457357	3.88391722	4.46399903	4.12167901	3.9267558	4.15895564
RTN4R	0.76250018	0.68649646	0.79478818	0.02108407	1.51575029	1.71023548	1.09768655	0.02422527	-0.04824618	4.5157932	3.28450587	2.43643754	-0.04824618
PHF23	5.80968948	4.66109704	5.61756934	4.78763301	4.37373379	5.05992246	4.56853641	3.99696662	3.92130568	5.54193914	4.93079425	4.62094562	4.29497026
INPP4A	5.8674866	5.35566795	5.21773685	5.31259672	5.32507764	5.16582225	5.43901805	5.72878776	5.35989991	4.79423156	6.17913106	5.7853702	5.83009475
RAB27B	0.00916353	0.95866072	0.00124472	0.61000061	0.19206419	0.14201455	0.13039953	0.11698454	0	0	0	0	0
PSMA4	5.10825695	5.34151548	5.36363801	6.53079972	5.80593917	6.06012658	5.92750896	5.84170029	5.88935581	5.24257736	5.36009282	5.59470514	5.91021273
MYO16	1.26307694	1.37371625	1.50575966	1.11845876	1.17733839	2.28394106	1.63416832	1.07265903	1.09187853	2.98349496	1.40525154	2.74885253	3.52911802
LSG1	5.77633045	5.41628738	5.19254284	5.3093744	4.85661288	5.01557878	5.44028067	4.99726555	5.23648628	5.37729053	5.71779307	4.61852663	5.71537743
PARP3	5.48515186	4.45822412	5.17177161	3.99399951	4.11603765	4.51655914	3.58300659	3.85789366	4.22983026	4.58598489	4.78390501	4.37643689	3.88507498
TNC	9.57872987	5.74502663	10.0144224	9.15507353	5.72406205	4.9167112	7.12197748	5.55982133	3.62068827	4.8008899	6.15256022	5.27087143	7.28080322
THAP3	4.20437934	3.64803759	4.80269674	2.81835632	3.57894829	4.19984301	4.37860823	3.89150326	3.41932511	4.14411522	4.79959493	4.62890905	4.35927831
TDP1	4.59602811	3.52413888	4.15423164	4.85611907	4.14384156	3.41764094	4.53401394	3.7318153	4.4261713	2.28381569	3.556851	4.27136386	3.98575187

AIFM2	4.92488491	4.64742857	4.88134349	3.61167862	4.22167931	4.68419203	4.60244471	4.27599022	4.11802414	4.83103645	4.09690881	4.35323844	5.01081785
SPATA7	4.36583982	4.61383847	3.89801722	4.65521561	4.59123249	5.53301401	4.51748959	4.73493039	4.69170804	4.86449436	3.93950977	4.72878127	3.79796457
MED17	5.33966341	4.93983831	5.2897516	5.12609016	5.52762756	4.99124913	5.50187993	5.69198991	5.23184175	4.58777387	5.06725052	5.02280545	5.30614386
RETSAT	5.67611914	5.35453108	6.18467433	5.51580114	5.64388208	5.97979844	5.84831409	5.33006246	5.56206327	5.5222816	5.87033567	5.84295513	6.03807896
CAPG	5.85802973	5.79520928	6.39706243	6.10676166	3.80626164	3.73175978	4.24872009	5.02280921	4.6319359	5.10189433	5.15538376	4.95902622	5.54364025
AP2S1	4.70338175	4.15455573	5.23829057	4.85604355	3.85827521	4.01553767	3.48484603	4.01218676	3.3897068	5.08537556	4.12825793	3.90452492	3.20818178
TG	1.85855627	1.19275236	1.69444565	2.09250721	1.03956941	1.84591684	1.49744716	0.92239924	2.87443064	3.20714455	-0.83207785	3.05556271	2.24369148
ADAM28	1.68397676	2.08453619	-0.39340125	3.92837891	1.70496652	0.99432	1.21241332	1.0822332	2.56940237	-0.47759309	3.63663786	-0.47759309	-0.47759309
DCUN1D1	4.61098058	5.14847574	4.66998265	5.55876339	5.47726485	5.99948637	5.7965333	5.69316586	6.21685846	4.87153521	5.84344822	6.1294874	4.49714135
LCP2	5.47856743	5.6906644	5.21599697	6.3248963	4.59347155	3.73070303	4.39380446	3.73651195	4.94899622	3.34830841	4.91827924	3.77218131	5.59826434
TRIT1	4.047725	4.6713373	4.20284774	5.35338807	4.83614127	3.98780391	4.80114051	4.42737453	4.16648976	4.91987748	3.94081533	4.21890132	5.13109643
ADRB1	0.47498817	0.38556314	0.83205814	0.73483283	0.54505307	0.69844224	0.57660798	1.07291963	-0.33041329	-0.33041329	1.5489134	-0.33041329	4.54608104
CUL7	5.36496274	4.79703508	5.68744297	5.42692307	4.1800788	4.4185965	4.80938394	4.96001155	5.20108234	5.18177704	5.42330322	5.50650925	5.07370906
CTNNA1	7.28195173	7.38667093	7.16368011	7.11438293	7.59227965	7.65548587	7.6931538	7.81940275	7.07045011	7.72517388	7.30177349	7.46902488	7.86557161
PHKA2	5.22386553	4.57811207	4.69713117	4.54196229	5.0994772	4.99840283	5.27040703	5.75311513	4.98711456	5.14341345	5.322034	5.40156234	5.6027185
CNTLN	4.65833621	5.34566886	4.45557963	5.49534684	5.30419792	6.00603675	5.14128941	5.59162773	6.21521071	5.47102236	5.23511351	4.98470282	4.76624933
EPHA3	3.71105056	4.7863132	2.57757758	5.17853237	4.53464747	4.40473849	4.16040288	5.60859007	4.85855434	3.84739306	5.27495112	4.16873884	5.916442
HSPA5	7.52984894	7.83861098	8.45669321	9.35633988	8.14907863	8.45669321	8.2542169	6.82618435	7.66327648	7.57437928	7.71355101	7.26471761	8.08724936
DSG2	2.26466769	5.70428411	2.51743808	4.95266877	2.82050925	3.16757837	2.19908254	2.7721828	4.18863286	4.25587782	3.76378419	3.96702902	6.06077835
GEMIN8	5.5451489	5.46707736	4.45479826	4.41706256	5.04394488	5.29424183	4.92630961	5.11022786	5.04582975	4.81934854	4.85997904	5.41865284	5.0991528
OFD1	5.45230051	5.33549561	5.30582496	5.06100357	5.60917794	5.95938474	5.77682779	5.95929378	6.18572252	5.67355545	5.86133904	5.49323791	5.1943831
GPM6B	4.27935218	2.58892261	5.0351156	4.09401576	4.23745625	4.87902973	6.17004734	6.93426533	4.45562804	5.27357674	4.82187202	5.55318005	4.85153263
PREX2	5.02502817	4.88440045	3.70764746	4.85177746	5.85882211	6.44552213	6.09709979	4.29680533	4.72701517	5.19759206	5.50518709	4.1824739	6.15768816
WDR37	5.52624815	5.30391601	5.24979224	5.22081767	4.75084827	4.57417841	4.90732334	5.35236455	5.05058759	5.57066006	5.03491315	4.72012389	5.63781558
YTHDC2	5.17204596	5.91969188	5.23051876	6.1572455	6.12913213	5.8872337	6.07221392	6.30548065	5.78708376	5.31995772	5.97697998	5.72310899	5.63053788
CTPS2	4.48949665	4.72984314	4.04138662	4.12499583	4.67960814	4.92423213	4.65983484	4.92546706	4.70655865	4.20308784	4.85071291	4.27679176	4.3833374
ATP6V1H	5.0101636	5.34252699	5.019742	5.16487706	4.92407662	5.70291187	5.54922456	5.26594173	5.18081203	5.86453532	5.21775753	5.50151032	5.31439814
POLR2B	5.91522249	6.38147545	5.96185685	6.80895483	6.60868635	6.82425789	6.66243603	6.60304274	6.30210759	6.44259346	6.36935738	6.4506897	6.4968534
ARAP2	3.69048277	5.29757622	3.47511193	5.4542252	4.0042434	3.64499461	4.02125931	3.491785	4.75687321	2.74914641	5.00135935	2.92610384	3.55321272
TPR	6.88471986	7.09695896	6.94761603	7.59891374	7.32898266	7.9502892	6.95896252	7.42998733	7.71568501	7.49094769	6.42793147	7.04130085	7.5852242
CP	11.0322161	7.35698388	8.75082633	9.44920914	7.60055247	7.00800495	6.44990031	6.15670183	6.42749474	6.15594594	5.88978681	5.84471683	7.29934666
KIAA0556	4.74427969	4.13351575	5.03755621	4.16769498	3.90073131	3.66581339	3.8766202	3.93476271	4.06645946	3.82404727	4.60997571	4.06154165	4.35365383
DTNBP1	5.12687333	4.55470871	4.65638531	4.07646894	4.14016886	4.20038899	4.63397038	3.90901323	4.23240121	4.32391121	4.6583043	3.94171583	4.61387587
XK	2.66013475	3.47275419	2.62690543	2.07765804	3.48080241	4.30467081	3.64571282	3.2055816	3.39111085	3.89652136	4.93002512	4.28231553	2.92572391
ANO2	-0.04669222	2.1224778	0.1331486	1.41970097	1.96372913	0.45013091	1.82594831	2.03400348	1.23693858	0.01294246	2.4893084	0.01294246	0.01294246
C12orf4	4.27637854	5.32652631	4.50223245	5.76277789	5.16016642	5.33932572	5.04140083	4.9521089	5.28782191	4.75587955	5.22796866	4.77430807	5.285484
SCML1	4.66917589	4.11800695	4.0846672	5.26322883	5.03933654	5.28987505	5.18477096	5.42442973	4.631698	4.2475956	4.92587987	4.73057721	3.6466448
WWC3	6.58774787	6.7577572	6.43118571	5.58987762	7.07888037	6.34081452	6.69733234	6.4184053	6.60028754	6.83023728	7.07116534	6.87193049	6.70301359
ARHGAP6	5.1359564	5.04939748	4.35692974	3.58109126	4.57840254	5.50565385	5.02493169	5.18590036	5.01299908	5.18449763	4.66609804	4.98482335	4.74246221
FAM184B	0.11975428	0.03908649	0.26169092	0.04928149	0.03908649	0.20629256	0.26580904	0.04236533	0.03524522	0.03524522	0.03524522	0.03524522	0.03524522
MAP4	8.11244416	8.20237684	8.17926483	7.65883814	8.73798495	8.3817547	8.43367537	8.80054059	8.42512493	8.80133315	8.55771958	8.67363581	8.99014928
GOPC	5.16200405	5.69365269	5.42115776	6.53885422	6.05639273	6.43338121	6.2980746	6.31752522	6.15724111	6.31264995	6.52873685	5.95865372	6.13261046
ROS1	0.03173168	0.17944995	0.02624956	0.36800884	0.04673508	0.02513084	0.02373	0.02694437	-0.04005532	-0.04005532	-0.04005532	-0.04005532	-0.04005532
USP28	4.89467338	4.58159448	3.94527679	5.00404872	5.14758946	4.75817483	5.14547795	5.28812422	4.4179101	4.68641786	4.66208153	4.72554942	5.76887756
HDAC9	0.75176504	0.98579527	0.23413349	0.1023856	2.06690338	3.16094905	1.75719934	1.48179451	2.7241677	0.55941255	4.12330603	4.90836868	0.55941255
TSPAN17	4.4571254	4.44014706	4.76066777	4.34375016	4.07878091	3.41996464	4.46817275	3.67719325	4.25675787	4.40073607	4.56745536	3.60572548	4.00223234
NOP16	3.00533101	2.51023075	4.95210429	3.56771751	3.21118455	4.18587607	2.22306834	2.86958659	3.6082949	2.96137123	1.66617522	2.28741591	3.62128805

CC2D2A	5.36965602	5.54607291	5.01836069	4.91199683	5.22145501	6.43387153	5.83828778	5.45553338	5.89486482	6.05196498	5.58309858	5.42397365	6.22942951
RRM2B	4.66267474	5.69540695	4.98732928	6.48880758	5.97013937	6.01457635	6.16702604	5.55923918	6.12158107	4.41784529	6.11428861	5.32786599	4.76432295
ZNF800	4.78944466	5.44360174	4.93086887	5.91821751	6.01989393	6.11890391	6.01246217	6.15371037	5.82591437	4.42398859	5.62108039	6.01921138	4.96646094
TNFRSF17	0.06050994	0	0.00124472	0.35619814	0.26405128	0.00069205	0	0.00158797	0	0	0	0	0
SNX29	5.03548374	4.89457863	5.26175934	4.57281841	4.18785878	4.50223492	5.16345636	5.14763164	5.15443236	4.92299492	4.53300938	5.04213317	5.34189359
LMO3	4.09666463	6.13282708	4.1173053	3.42988169	6.1683543	7.0520661	6.16774915	5.15221733	6.77710205	5.57932646	6.19416718	6.37982818	5.34587744
MRPS10	4.46878809	5.37571631	4.82664275	6.05282164	5.61170717	5.5839976	5.42013068	5.09798108	5.27231829	4.67657365	5.22690623	5.22007968	4.51317841
GUCA1A	-0.04162271	-0.04162271	-0.03958204	0.44453497	-0.04162271	-0.04048812	-0.04162271	-0.03901929	-0.01889952	-0.01889952	-0.01889952	-0.01889952	-0.01889952
RSF1	6.23937513	6.69474389	6.3502935	6.38852409	6.79084915	7.24117198	6.61903895	6.86964729	7.16895858	6.8275347	6.5606724	6.6665941	6.63525279
VPS13D	6.89361021	7.24686734	6.61769226	6.68555762	7.48320117	7.36253139	7.30135063	7.28502398	6.86548765	7.1167701	7.50408273	7.09548669	7.46451096
CELF2	3.69879965	4.95449984	3.1807185	3.80035457	3.97493405	4.43161394	3.94197094	4.67420823	4.09458654	4.43331758	5.15667626	3.57439259	3.38747315
FAM120A	5.97456704	5.94099193	5.98995262	6.33498389	5.75406911	5.53809584	6.16353212	5.72961589	5.80543503	5.45818689	5.81272875	5.57642545	6.18509895
R3HDM1	5.18404412	4.60736865	4.6312005	5.57174907	5.23490542	5.3289858	5.32806663	4.88292199	5.63112479	4.90477303	5.16558744	4.96364472	4.99771997
COL9A2	4.81865976	2.13304206	4.85232261	2.86800072	4.71162073	4.53318932	4.48606845	5.28911454	4.56773075	5.78691144	4.20260004	5.81684035	3.11251938
KITLG	3.91080044	4.94852944	4.1553292	7.02907509	4.25767387	4.5199764	4.84587526	4.48051803	4.63131705	3.25588126	5.22774362	4.09632139	4.37405318
ERCC8	1.58749176	3.16523067	2.85355904	4.39664875	3.92680369	4.0774853	3.53923231	4.27219851	4.00565192	3.30761656	4.16835981	3.22665344	-1.51732596
ADAMTS6	2.95786179	2.9465098	3.57562283	4.24056297	4.93373203	4.27235179	3.7076327	5.75008192	4.66270309	4.34410529	4.51812635	5.3305948	4.08438811
H6PD	7.41032921	7.57492326	7.0677928	5.98025637	6.83877229	6.11507967	5.76224559	6.59843234	5.881327	6.53353918	6.84800265	6.4374989	6.45059053
VAMP3	6.31838304	7.09714947	6.40692132	7.04285542	7.0901189	6.98388098	7.12193882	6.97607736	7.16033336	7.30798482	6.96051818	6.992891	7.09732413
PER3	6.74314306	7.61902996	5.51467227	4.46294806	5.65811671	6.27575958	6.21915063	6.0586293	6.61712381	7.1509561	6.72020484	7.26723788	6.95225074
UTS2	-0.14541063	1.35723828	-0.14293528	1.29072306	0.60680662	0.729834	0.14146627	1.43297054	4.50143116	-0.41865101	-0.41865101	-0.41865101	2.30985664
TNFRSF9	2.47182383	2.16617266	1.95863857	2.69165586	1.81336069	2.10302177	1.79909474	1.87296596	1.51568271	-1.14486391	-0.08795899	0.66814683	2.25272851
LTBP1	6.76012182	5.41201416	6.2952226	5.57015922	6.09522278	5.62774104	6.98062155	6.7290977	7.15760998	7.76583135	7.30800987	7.64049274	6.9172696
RCN1	6.42938861	6.4639817	6.28564669	7.75434037	6.44560114	6.68155591	6.75823632	6.37507881	6.73418903	6.57839028	6.44985151	6.0934794	6.59984575
ELN	6.07962378	7.63217514	8.87148045	6.67906007	7.93944685	8.35944658	7.64691662	9.59482863	8.1514433	9.05018859	8.52318083	9.13778564	7.31729835
RFC2	5.45008593	4.18378701	5.1220832	5.33087952	3.64940179	3.59489707	3.64593143	3.58536858	4.44776703	3.8720752	4.23983664	4.3880607	1.78916879
ARID1B	3.34747007	3.02951865	2.39476621	3.06748315	3.31887118	2.88268085	3.56031036	3.63813551	2.82361247	4.08342785	4.19187878	4.04228442	-0.16653163
CLPTM1L	5.25999873	5.26234742	5.76947744	5.12498761	5.00264774	4.61115647	5.2055425	4.75933235	4.46634596	5.45951874	5.450272	5.19975509	5.20997338
NEDD4L	4.50333095	4.09599016	3.32714414	3.98716781	4.14444893	4.34313649	4.68866544	4.35201449	3.79300724	3.52819468	3.51100552	3.96643365	5.71765554
FOXP3	0.39441826	0.31529187	0.42775389	0.5690705	0.32866707	0.26033539	0.10851019	0.11178354	0.81517409	-0.11717661	-0.11717661	1.73815794	-0.11717661
PPP1R3F	3.1614545	2.80816948	4.09543918	1.35353093	3.48089067	3.46883357	3.49851163	2.69484352	2.72298958	2.67188941	4.41828521	4.06047366	4.30109008
HEXB	6.10464007	6.34423094	6.33001866	7.33768166	6.53484456	6.63100276	6.69812659	6.79224206	6.69479677	6.5104569	6.95611756	6.65463539	6.27609957
PTCD2	4.19413801	3.65790955	4.14548065	4.20072717	3.79883581	4.58673993	4.56653645	3.79883581	3.64644635	4.47667415	3.70518639	3.75207455	3.59393476
JKAMP	4.70340264	5.38751836	4.58119181	5.8257783	5.5856003	5.59317406	5.58376283	5.75776728	5.528409	5.43021277	5.41799313	5.93297666	5.47238625
DKK3	8.65375924	8.52434958	8.34520863	7.01133216	9.29433404	8.67512773	9.04973846	9.04973846	8.88610495	8.85385675	8.91021408	9.49216419	9.03295321
ARHGEF5	4.59396041	4.30529226	5.2124046	3.55923036	4.27390829	4.39781937	4.4754301	4.81861099	3.9112665	4.58133987	4.32858751	4.66194739	4.49341233
NFE2L3	-0.12573556	0.60247996	-0.07216514	1.10458204	0.56867881	0.29251234	0.22751693	0.14700767	-0.03116987	-0.03116987	-0.03116987	-0.03116987	-0.03116987
LIMA1	6.20881403	6.58971128	6.26814002	7.26144345	6.50080585	5.66975648	6.41947306	7.05971337	6.91191522	5.96263913	7.19423385	6.72571592	7.05095128
LETMD1	5.00951526	5.55306094	4.92865157	5.27054596	5.10559448	4.59648074	4.97263222	5.23159284	4.76616998	4.81481868	5.46733023	4.90618893	5.31526475
SLC4A8	3.57756392	2.31423399	4.2975898	3.55126131	2.43690286	2.75980281	2.62974408	2.50953078	1.98411027	2.43686011	3.33774696	1.96907893	1.75751594
LAMC3	3.87828844	3.19006225	4.03839641	1.61907489	2.13738142	2.25929584	2.61484727	5.3596254	2.58873837	4.22569238	3.21027195	5.25244309	2.24566114
PTGER3	5.2811771	5.38269079	6.10273439	5.37187314	6.95535578	7.11167003	6.81183345	6.62243664	6.8476654	5.89913513	6.66088211	7.00584534	4.88206643
TNIP3	0.46874206	0.37602136	0.8489139	0.73958599	0.51508597	0.37746294	0.51457298	0.3793292	-0.23733084	-0.23733084	-0.23733084	-0.23733084	1.6769091
MAPK9	5.50552005	5.7358177	5.27412361	5.42691915	6.00278314	5.70654962	5.89228003	6.05871415	5.82445141	6.00673259	5.90316262	5.81003904	5.37593544
COL23A1	0.9010325	0.96586994	0.95402479	0.77855303	0.4208902	-0.00535202	0.18937055	2.05614561	-0.26608875	-0.26608875	2.04634216	-0.26608875	-0.26608875
BCAR1	6.1074191	6.16293098	7.21868155	5.37364492	6.06646753	5.42529644	5.93491325	5.71453355	5.53875637	6.88257057	6.21283705	6.56040115	5.05137543
FAM160A2	4.72558988	4.92595734	4.79765442	4.35569158	4.56133591	3.82785988	4.50223983	4.82604089	4.49654496	4.65000199	4.67415516	4.47099607	3.84158688

HERPUD1	6.35953994	7.02710485	5.61771088	6.58867037	5.92073338	6.0085123	6.43958754	5.89408048	6.01602571	6.08286259	6.34827896	5.98122913	6.47644664
HOMER3	5.0234738	3.67272656	5.34553525	4.25732643	3.75083482	3.08244423	3.73094964	4.21894421	3.93937224	4.01784298	4.48386358	4.10391563	3.2095243
RAD51	0.49262298	0.25624018	3.20070697	2.70911874	0.15129822	0.97499783	0.77182567	0.64802449	3.05198103	-0.44925234	-0.44925234	2.60179682	-0.44925234
POLQ	1.53243998	1.40624392	2.56552516	3.98303706	1.23443152	1.28267072	1.8929976	1.34454589	-0.49116932	-0.95593146	2.08415424	2.46581983	1.7921823
PIK3CB	5.28081002	5.62432487	5.13137196	5.65611067	5.67849485	5.85754258	5.90624763	5.83067993	5.56026646	5.23723479	5.86849171	5.79337706	5.59200468
CYBA	5.13465492	4.85000475	5.31193659	4.91849186	3.81823161	3.65708507	3.55087176	3.2665043	4.11459877	4.77723797	4.1343406	5.13456795	3.03416921
THOC3	4.58752352	3.96448069	4.78693484	4.80228351	3.72797615	4.35974478	3.58475596	3.64997249	3.79196782	4.5883552	4.32734632	2.82740539	3.884871
HEBP2	5.17200209	4.76334983	4.54970967	4.27535657	5.20578413	5.1651292	4.96393761	5.33099409	4.72635767	5.03150905	4.73276526	4.92302301	4.37710058
MPHOSPH9	4.12651419	4.57962907	4.69748495	5.5377422	4.43669184	4.86922045	5.06881199	4.97702185	4.71853187	4.48514874	5.26251494	4.59008046	5.24256755
PLEKHA5	5.3966042	5.24783114	4.35755488	5.0606742	5.50018621	5.83810947	5.44760018	6.0702009	5.75464205	5.61169007	5.27157318	5.4279266	6.23427481
SIKE1	5.10473539	5.00944198	5.00814452	5.84787248	5.84846222	6.30631677	5.91121644	5.95914432	5.83040816	5.37619433	6.26921779	6.09427327	4.51952091
RRP12	4.59867097	4.6675014	5.62887344	4.96412549	4.02097936	3.03310651	3.92326492	3.93051793	3.73201969	3.04304351	4.04231673	3.25812709	4.93542548
FNIP2	6.68266569	6.09809138	6.864132	7.11269234	6.1390198	5.87239733	5.94546318	6.11907059	5.3479067	6.41617763	5.69180498	5.84889932	6.11942243
MSMO1	4.08197976	4.93622066	4.65330798	5.34303211	5.24800677	5.06701891	6.37699227	5.65828008	5.51158425	3.99868123	5.84230315	4.6921631	4.4117178
TTC17	6.06492204	6.32311971	5.60525327	6.49383076	6.46425887	6.19915513	6.4024467	6.44962469	5.91433365	5.67043137	6.28471976	6.1252108	6.56706486
FOXN3	7.54715471	7.38122095	7.03840151	6.77737967	8.01087016	8.02208092	7.88387899	8.07055764	7.4117198	8.16554213	7.39186217	8.06492552	7.90963491
AKR7A2	5.14917126	5.00826996	5.09961051	5.00226449	4.00562835	4.44084482	4.3164117	4.08483149	3.83171846	5.27230927	5.09094647	5.09490603	4.84082133
MRTO4	5.27763723	4.69339275	6.47469551	5.40875746	4.35881486	4.37630273	4.37630273	3.70802139	4.57871194	4.15540071	4.68893965	4.42332824	4.47784974
USE1	4.92288162	3.83104829	5.08827731	3.85433501	3.79880797	4.27278414	3.90826072	4.10808711	3.96931411	4.93723447	3.15893718	3.98174018	4.74536831
MCF2L2	2.13777445	0.78385582	1.13803882	0.91436208	1.33504921	0.74147212	1.51095292	2.91221333	2.72186877	2.91449815	-0.61207774	-0.61207774	3.57461032
NRIP2	2.24115349	2.12541649	0.48472072	1.57421646	2.82019482	3.56572909	3.17421979	2.5439918	3.51240435	-0.86509545	4.27065868	2.64160581	3.8804303
LAMA3	-0.53995405	1.30681557	-0.6500092	0.04819978	1.59177062	0.86078022	0.92971329	1.72335355	0.2000926	0.2000926	1.36631846	1.32703073	2.25999493
ANAPC4	4.71660498	5.38540252	5.07621847	6.02251159	5.89854076	5.73062267	6.20945467	6.09430131	5.51178183	4.83692445	5.32997808	5.70619202	5.27949797
KCNQ1	3.16754147	2.72532989	3.36979623	2.56690339	2.51273446	1.09073565	1.38483438	1.18394669	1.71212431	3.53448705	2.4799156	2.24860817	3.5445856
TRAPPC3	5.71753596	5.46519884	5.30930944	5.04793047	5.16032778	5.66202996	5.58492147	5.40963234	5.69417261	5.6681824	5.74673875	5.71049738	5.23023018
THRAP3	7.48814144	7.26085724	7.47735715	7.27310138	7.06904827	7.21536302	7.08430822	7.17610544	7.20435725	7.47559712	6.90524569	7.09864912	7.49693619
PHPT1	4.30747755	4.60744795	5.06041773	4.50590021	4.27476433	4.16869855	3.92534336	3.85060529	4.71359236	4.90704988	3.97839227	4.9557851	3.80669943
ENTPD2	0.19724937	0.19724937	0.19977621	0.34042962	0.61683327	1.01296143	0.76268131	1.10303958	0.46203474	-0.11577531	3.35407996	-0.11577531	2.91250399
LY75	4.4951242	4.63243642	4.535239	4.74457897	5.41347155	5.05864118	4.58249275	4.76907793	5.37655618	3.50909195	4.40687657	4.71306343	5.18708547
ARID4B	5.21830467	5.35700203	5.36496328	5.82250482	5.71610122	6.07072249	5.85505754	5.98158438	6.12460749	5.32403623	5.26461078	5.68309528	5.37839126
OPN3	3.15550637	3.0628736	3.1289205	4.54505985	3.66489618	3.97064349	3.70359725	2.82938251	4.20645959	4.3817446	1.65802442	2.86654633	3.60949761
SDCCAG8	3.35437284	2.87493028	2.95799119	3.56428875	3.09298201	2.94904487	2.51957404	3.1750746	3.1400343	4.81725924	5.03288644	4.40272218	-0.43847706
PTPRN	-0.01958289	-0.09658442	-0.02512646	1.37255373	-0.09658442	-0.022097	-0.07956086	-0.09492623	0.00736904	0.00736904	0.00736904	0.00736904	0.00736904
HHAT	1.80870426	1.53377692	0.99775738	1.52154187	0.59616747	1.37170371	1.7471847	0.39143791	1.52684485	-0.19033936	-0.19033936	1.6236001	-0.19033936
KIF1B	1.9287026	2.43872305	1.75785281	1.99268379	3.13576994	2.83685105	2.8684695	3.79057339	3.98661415	3.41179413	0.0196245	3.29638533	0.0196245
FOXC1	6.99811878	6.53473396	6.86887003	6.07144009	7.32630874	7.66452701	7.1497072	7.78719623	7.60288859	7.5724929	7.39857297	7.29854335	7.63695477
TBC1D22A	5.26410561	4.67795732	5.19911947	4.36185859	4.64296377	3.94212331	4.37313989	4.45379014	4.80726567	4.96007712	4.29339992	4.29582798	4.52494267
SYNE2	7.19099364	7.21033346	7.28659868	6.6028075	8.15183518	8.34583343	7.71423731	8.1589342	7.57116163	6.52973464	7.09043953	6.98068159	7.89957286
PLEKHH1	2.50619897	1.97246087	2.00895142	1.94546101	2.47132078	2.33916237	1.75465048	2.79634872	0.27634075	3.07568681	0.14974201	2.99305694	3.46194309
ATP9A	7.19060457	7.15076328	7.17738572	5.66446473	7.11715823	6.97798476	7.16916212	7.1062262	7.00704928	7.39981069	7.4404669	7.33467083	7.31587042
CBLN4	0.18095156	0.80225509	0.18351838	0.2207503	0.18095156	0.18237868	0.5934514	0.18422622	-0.14595209	-0.14595209	3.49655088	-0.14595209	-0.14595209
CHRDL2	3.19763303	1.24827096	4.17066545	0.41707708	0.10847113	-0.60774811	-0.01356454	-0.60646966	0.85472805	-0.47934171	2.83230938	-0.47934171	3.35745188
FAM168A	6.90706866	6.75543392	6.49868551	6.53904219	6.61396883	6.45631767	7.11575399	6.33280742	6.26526657	6.63484682	6.3600801	6.64858722	6.66684835
RELT	3.85894779	2.85596691	4.32561387	4.03608507	2.13555969	1.77507363	2.74538847	2.04289109	2.89014474	3.20021929	0.45443882	2.66279415	3.17229876
GALC	4.78857455	5.06028407	4.73296101	6.01497139	4.63663656	4.44608645	4.76039114	4.80939447	5.11147678	4.5187048	4.59889125	4.77658914	5.08876623
NOP58	3.1077331	3.70959467	3.67937686	5.11710643	4.20846644	4.04417299	4.20904448	3.53490795	4.16624494	4.17666305	3.75792333	3.16823924	4.13097383
KCNH2	0.78375212	0.92861232	0.64947775	0.63328417	0.74260468	0.7151827	0.68515277	1.58331557	2.13144365	3.26891116	2.18394665	3.57614084	5.6204589

CUL1	6.19687416	6.34505799	5.52277111	6.04610192	6.28591317	6.47679681	6.25246596	6.33184722	6.28612319	6.04980807	5.87167026	6.02905407	6.10496299
FAM114A2	5.11258474	5.48449194	4.96365447	5.97280907	5.63213015	5.95303124	5.57916601	5.62343516	5.60465645	5.73976337	5.83605827	5.7066863	5.55355491
CYFIP2	5.43756592	5.95065049	4.60290727	4.00572776	5.76828632	5.33530298	4.88326824	5.13243192	5.24637432	4.52990565	5.4591194	5.24103324	5.43959248
TAB2	6.68402006	7.08946953	6.16139954	6.86703195	7.28447475	7.11634353	7.16157097	7.05302888	7.09407657	7.17574346	6.68554509	6.78306895	7.5369971
EIF2AK2	5.90580448	6.60723218	6.23080268	7.07289748	6.84776008	7.09630184	6.73548158	6.63684332	7.10034967	5.7663483	6.67933965	6.1546192	6.8627006
USP36	6.27564039	5.95983583	6.21079097	5.60697034	5.82287699	4.87610209	5.76152499	5.31803914	4.96342618	5.48997204	5.60091411	5.77735445	5.93797277
MCOLN3	3.80274694	3.05196741	3.84872764	2.71507178	3.22025772	2.6385067	4.22408299	5.67428359	3.96848972	4.74817914	4.65965722	3.24582434	4.15071681
CCDC85A	1.06073022	0.92354276	1.1978129	1.01560923	1.8051452	2.00105659	1.17511907	2.01359919	1.97062025	-0.06258699	2.03475411	4.8286007	4.07604718
PUM2	6.46117407	7.04581818	6.09299155	7.01095512	7.13172993	7.0740091	7.27707641	7.05445206	6.92666595	6.72188725	7.004125	7.02853619	6.66224034
MRPL43	5.08931158	4.48005152	4.57552132	4.10898248	4.64543759	4.73099462	4.69750142	4.36299761	5.12305962	4.63389941	4.50375625	4.59426706	4.44002895
ITIH4	4.25854031	3.49301915	4.3875872	3.31588408	3.97571202	3.95151686	5.10114915	4.41619553	3.00335962	5.01298358	3.25622301	4.23911313	4.51792776
ITIH1	0.32581124	0.03531648	0.03790128	0.03531648	0.28504929	0.0367536	0.03531648	0.03861408	-0.17476236	-0.17476236	-0.17476236	-0.17476236	-0.17476236
ZFR	6.72783155	6.41020347	5.92254066	6.65807001	6.87348489	7.00846687	6.91130488	6.77776354	6.55900672	6.44999692	6.12189372	6.9232618	6.63545099
ZNF280C	4.18791002	4.16754499	3.93811843	4.92510973	4.65486037	4.85076472	4.17982454	4.43962946	5.05082748	3.99120264	4.63088585	4.54309066	4.09444922
PHF21B	0.02461187	0.00647955	0.00895857	0.00647955	0.00647955	0.38228991	0.18165041	0.16473633	0.0522348	0.0522348	0.0522348	0.0522348	0.0522348
TRAF1	6.22555561	5.52618589	7.507713	5.47841863	4.98078152	4.8579536	5.22360763	5.52519376	4.77571306	5.32374666	4.97239851	4.8677931	5.45899675
RC3H2	5.86097165	6.12328964	5.56188556	6.22279978	6.33142334	6.42105115	6.34411701	6.31458044	6.22099659	6.29417551	6.11099818	5.98135393	6.60318801
IL17RB	2.08518093	0.42610736	1.04355913	1.24070045	1.02762773	1.44433363	1.94559831	2.04578739	2.11534587	3.73825577	3.34136395	1.45303862	3.37327369
TRAF3IP2	6.32034761	4.44545899	5.5832528	5.2397679	4.34485937	4.71185883	4.22117363	4.28614857	4.05700843	3.81230154	3.9043826	3.9522144	4.1762877
GYG2	2.33584873	2.84816268	2.91385764	2.28220997	3.94479999	2.45646667	2.3758778	2.54755522	3.38428755	2.73870543	3.34823798	4.29610839	3.24558696
DCBLD2	5.27247708	5.86730073	5.3468	6.54613235	5.85146424	6.2838523	5.83035742	6.15971984	6.36988284	5.73522344	6.29031497	6.2815582	6.25998457
SOAT1	3.77820157	3.08752691	3.12334709	4.50089494	4.08489453	3.67360724	4.20419877	3.76207043	3.54043008	4.22174654	3.10784675	2.45958864	3.49948482
PKP2	1.88143968	4.35517339	1.93190502	1.6653299	2.47862004	3.33047637	1.88211612	1.84422652	3.15984056	-1.12496522	4.17863179	3.42143432	7.52951335
MSH4	1.66617341	1.30585234	1.77019876	1.70042804	1.88334061	2.5345556	2.71713964	1.48885244	2.51248066	-0.91123701	0.39567762	3.11924336	-0.91123701
F7	0.70398596	0.6059162	0.55860656	0.61426304	0.43557426	0.4370161	0.43557426	0.81009797	1.69529503	-0.24003805	2.75603511	-0.24003805	1.38138482
GDI2	6.58805967	7.21317159	6.75750079	7.99243321	7.14534247	7.35757725	7.47191991	7.19908786	7.59782829	7.41050637	7.3901896	7.38674875	7.22183808
PRDM1	4.11760974	4.21224054	3.00470646	5.24265183	3.25999615	3.30410247	2.8521702	2.59893048	4.83181368	2.81523995	3.40822893	3.00378501	4.38787986
ATG5	4.53828232	5.27230346	4.68654446	5.70230725	5.73707047	5.91618459	5.71712118	6.04000114	5.87776544	3.935396	5.57859639	5.68941055	4.94588065
TMCC3	3.25891505	3.673945	2.28694268	3.74723491	3.88551446	3.56547863	4.34191835	4.03146349	3.51171905	4.41349736	4.30722818	3.5555204	4.38932166
PITHD1	5.87051157	5.44326467	5.28372239	5.52606532	5.58879465	5.43196548	5.29705195	5.50111424	5.81337353	5.60933203	5.43396535	5.5879459	5.23268465
MTA3	5.73955772	5.10793943	5.30956994	4.71547299	5.57543699	5.41547274	5.60952772	5.89720488	5.47329278	5.27924518	4.8031275	5.21086685	6.03750678
USP13	4.92511118	4.80348056	5.31653701	4.94653181	4.89460238	5.12903231	4.46235161	5.1450292	5.01023346	4.97077996	4.56516948	4.92282572	6.53673377
ATP11B	4.2653191	4.89085936	4.11510666	5.43157428	5.45176805	5.64255453	5.32103892	5.61036403	5.95751118	4.39646442	5.15058904	4.92218804	5.01075794
LAMC2	3.19950373	4.15763666	3.85703103	4.15396228	3.97376202	4.02949169	3.07532623	3.92335347	3.48438891	3.47184702	4.10730887	2.63698173	3.1663699
CDK14	3.32791391	4.17464652	3.44315147	4.74373876	3.76751784	3.71242338	3.80329735	3.75594235	3.96180446	2.85602516	3.95716734	3.92828854	3.62933703
SEC61A1	7.01416075	7.02220853	6.92567521	7.90525445	6.62806682	6.45304278	6.91552815	6.62209764	6.37689457	6.65173278	7.00281126	6.94708142	6.67453674
PPP1R12A	6.67442442	7.17999851	6.51989228	7.23968259	8.02441391	8.20499524	7.73667716	7.97751014	7.85734185	7.54615666	6.83254033	7.90232042	7.53624559
RASGRF1	0.68984978	0.54543159	0.743566	0.58450666	0.59595488	0.54687994	0.57634849	0.54875498	-0.25709174	-0.25709174	2.14025414	-0.25709174	3.02095058
CAMK2B	4.68957336	1.59357148	4.62717611	2.45249763	2.86540642	3.01246597	2.01040088	3.66851004	3.75531937	3.24177108	3.10859696	2.58857244	5.2527831
CROCC	5.08787629	4.42089569	5.43561758	3.83261315	4.04343466	4.28217874	4.61654968	4.67780059	4.78472795	4.14764479	5.3273273	4.43872588	4.79688094
POLR3E	5.60922682	5.1973302	5.36605465	4.43708561	5.11764026	4.95237036	5.25475422	5.2148461	5.10914499	5.32744593	4.70019658	5.20748895	4.67544161
ATP2B4	5.67377068	5.58987316	5.53638987	5.45350762	6.17396876	5.92708018	5.74231529	5.62269943	5.27301366	6.17074305	5.45939119	5.77497884	6.51503887
ZC3H11A	4.04407079	3.92145172	4.2362851	4.02189172	3.93002336	4.03394939	4.09202429	4.11664999	3.91827532	4.27067154	3.95943999	3.4675975	3.73794354
RIOK2	4.16867033	5.00292131	4.92729612	5.6496964	5.15367242	5.73781561	5.50296866	5.19992164	5.48863361	5.34413947	5.32412376	5.43294643	4.71094291
YIPF1	5.48452905	4.89721935	4.73437313	5.36888625	4.83604739	4.86828094	5.03843296	4.53957813	4.78328047	4.36257508	4.76730112	4.71381101	5.12028802
DGKG	3.09621082	2.73705371	3.01886227	2.61505292	4.75882449	5.28014488	4.40611509	5.20966538	4.21664864	5.00059095	3.61182907	4.74725425	5.59816408
FLYWCH1	3.25658315	2.9137037	3.12808419	1.7613767	3.24010111	2.38447113	2.3310207	3.34590381	3.02306397	4.33276539	3.82998432	3.51762792	2.95413216

UNKL	5.12048498	4.30858748	4.99070243	2.79760784	3.81136938	3.61550341	4.3155537	4.2712059	3.49969066	4.8123831	4.71487709	4.62854151	2.08364033
TBXAS1	4.46062203	4.61726914	4.27826059	5.19363618	3.70334042	3.04506621	3.51928573	3.127418	3.87244109	3.0963334	3.02595329	3.2683768	3.85540767
PARP12	4.8714533	5.29223769	4.9522459	5.35179212	4.9115015	4.12572372	4.80852619	4.50710037	5.2737381	4.55995261	5.36079822	5.08379358	4.89186824
ALDH18A1	6.11821018	6.22519851	6.01798786	7.20230165	5.76542697	5.3822569	5.9045582	5.60419499	5.93578468	6.06783024	6.33428433	5.91163679	5.78196562
TARBP1	3.54481554	4.88456222	4.39253055	5.39904733	3.99533756	3.92279584	4.53098682	4.51006321	4.9020183	4.73697372	4.61400233	4.28408693	5.01463327
MXD1	6.08772388	4.59882458	5.26846546	6.4453614	5.52469094	5.24553682	5.33381909	5.58261277	4.82748271	5.15462453	4.73303437	4.51749531	5.59718775
CDK17	4.59740624	5.55967801	4.99979148	6.87353259	5.9834263	5.49607425	5.65477295	5.85297783	5.37786288	3.73504452	5.37696716	5.44528014	5.49777014
DNAJC25	3.70064264	3.52986678	3.51597545	4.61093706	4.38345396	4.64284323	4.56276622	4.1856015	4.5303716	3.76006811	3.20391973	4.19445616	3.91834136
SLC2A3	7.00815928	5.42675048	5.94656525	8.02013117	5.27294532	4.9100683	4.8219391	4.10729841	5.0832375	3.80070898	5.34558741	4.76128151	5.19098072
PSD	0.53974906	0.77184019	0.59181954	0.3983459	0.14714254	0.39984707	0.31519862	0.40511643	-0.37109699	3.36525154	-0.37109699	2.27255157	-0.37109699
CTDP1	4.99131927	4.02121863	4.90649063	3.98727823	4.3311676	4.22087436	4.20384953	4.70598938	4.40380872	4.58680435	4.44126141	4.64034373	3.86097228
STYK1	0.12523485	0.38655022	0.26409425	0.89746445	0.09923637	0.27755068	0.73787597	0.845109	0.38826169	-0.08352757	-0.08352757	-0.08352757	-0.08352757
WNK1	8.13561044	7.73689493	7.70239192	7.64717748	8.22649224	8.31255332	8.14526558	7.8758788	7.82827694	7.75875537	7.77100568	7.58302154	8.21326047
CCAR1	5.41089519	4.57288786	4.94699003	5.98229575	5.14261177	5.23295408	5.4514313	5.33832317	5.14922688	4.69121235	4.66089296	4.9337759	5.5475349
OGFR	4.41775182	4.18716175	4.8635632	3.64251813	4.14702524	3.49867018	3.86445626	3.69428399	3.88087189	5.06547632	4.53601828	4.05218346	4.00286999
GNA15	3.14080868	3.45232585	4.41498088	3.72816561	1.7007262	1.01924522	1.87099093	1.24780043	3.36238754	3.43196132	-1.02528472	2.25365376	2.25979429
PIGV	4.53199233	4.04895063	4.27348822	3.43019798	4.40030447	4.1167839	3.82643343	3.53886734	2.87851623	3.93629028	4.08886527	3.89884875	4.48901106
PTPRU	6.22894715	5.08905522	5.61363978	4.44561394	5.22750302	5.03231554	5.26352828	5.2432065	5.14589655	4.89239077	5.18599944	5.41049256	4.26067664
SNRNP40	4.73304701	4.72038731	4.75227277	5.3612984	4.98659232	5.27163273	4.884599	4.31905557	5.1149855	4.73268308	5.07847758	4.87002781	5.10734711
RIMBP2	0.4045093	0.77948404	0.45123001	0.96606616	0.38507177	0.6923291	0.57481482	1.96658447	2.92929826	3.09600262	-0.40570726	3.09991753	-0.40570726
COL11A1	1.17175724	1.69712829	1.19635388	5.39114587	1.35672754	1.28912416	1.21446567	1.19824704	3.25784878	2.0052332	3.27593044	3.19777599	0.7937899
QSER1	6.13913901	6.32791193	5.65930665	7.12678829	6.63126038	6.66542808	6.43116988	6.7176738	6.4576812	6.76251658	6.216753	6.53807656	6.44647958
ACAA1	5.31266367	4.88406942	5.53848293	4.69747239	4.52481541	4.6368234	4.84957421	4.92307505	5.24027204	5.26509581	5.378817	5.19130371	4.70382287
BCAT1	5.05166904	5.62910886	5.15944978	7.92685552	5.02329605	5.00500846	5.02329605	5.44800003	5.38156472	4.15466796	4.72715948	4.89077731	4.62813112
HDAC7	6.15769231	5.82298547	6.47566579	5.38233148	5.57430959	4.85356344	5.49586867	6.03671742	5.40284183	5.22248939	5.99086366	6.03484791	5.47589428
LZTS1	3.38762205	3.06598866	3.80729707	4.08613443	2.26155861	1.59501989	1.93420822	1.53688759	2.95422709	2.27085718	2.29124854	3.14032605	2.80079367
PRDM6	7.28718721	6.85139403	6.88105398	6.16213633	6.7220074	7.73780319	7.81951907	7.3520562	7.63782098	7.57638531	7.35292755	7.40559473	6.9085948
SPAG4	4.27487258	1.78810654	2.67864772	2.08604566	2.3668937	0.86040138	1.13133156	2.13194415	0.87595846	2.87530721	-0.84230663	1.25450278	0.19281318
NCKAP1	6.51799691	7.11160371	6.56518094	7.00396719	7.68935253	7.54134215	7.68144466	7.61427722	7.51544475	6.99306464	7.36511001	7.67535552	6.84536361
MRPS35	5.32160543	5.76777262	4.61160989	5.87921402	5.24095382	6.02790532	5.72328788	5.61301932	5.71548093	5.52650041	5.79539896	5.74071031	5.65310173
GUCY1B3	4.94436336	5.33838675	4.0734454	5.93818104	5.04894677	5.98883697	5.580615	5.17659397	5.7170217	5.09803341	5.73940463	4.72820098	5.58908502
SFSWAP	5.78890017	5.43983892	5.6196957	5.33260321	5.72298588	5.17872092	5.912102	5.89058104	5.33005806	5.72860564	5.55987305	5.10886786	5.54715001
TNK2	5.258305	4.82111347	5.98740518	4.35101477	4.40871957	3.95535322	4.57730085	4.55029472	4.38479264	5.71112775	5.08116104	4.86898691	3.67607434
MON2	3.42368821	4.20549712	3.19837364	4.73366508	4.53259327	4.39771644	4.50996592	4.76644137	3.67433884	4.95109723	4.3459844	4.01670158	3.98217029
CDH3	0.06159864	1.22019466	0.00124472	0.49742094	0.08576568	0.17160604	0.06441473	0.00158797	0	0	0	0	0
GPBP1	5.65581343	6.38635899	5.81153077	6.76175922	6.63594458	6.8547855	6.63914118	6.65034533	6.86782184	6.70208002	6.47053544	6.72665089	6.37848969
DGAT2	2.61800201	1.5367595	1.56089154	1.99339434	1.89163381	3.76369688	1.85868706	1.70041875	0.98172328	3.41711833	-1.1881991	-0.01730642	-1.1881991
CS	6.84901953	6.61958154	6.5857402	6.93905169	6.90605597	6.40969173	6.62316893	6.48666787	6.17748069	6.85215087	6.29459007	6.70018332	7.61738024
LTK	-0.21664573	-0.2619312	0.14205826	-0.07169854	0.45073897	-0.26053906	0.01269628	0.06813998	-0.18250931	-0.18250931	-0.18250931	-0.18250931	-0.18250931
MRPS24	0.34429594	0.29896301	1.07014889	0.4131078	1.10014268	1.25760175	0.74136137	1.36043466	3.39808603	-0.3815868	-0.3815868	-0.3815868	2.23930884
ELMO2	6.23077425	5.86643334	6.4113839	5.61935397	6.09432319	6.04155909	6.10408851	5.68219868	5.80546722	6.17447205	5.78673023	5.87441486	6.29148467
VMP1	6.97542972	6.87489149	7.38067676	9.00889437	6.976738	6.27163276	7.00129754	6.10982547	6.38610036	5.95971287	6.64654436	6.04178822	6.6389273
APPBP2	5.38404677	5.81945717	5.22878034	6.17630938	6.19034444	6.44682271	6.09752355	6.50919571	5.99915659	5.53521118	6.01362176	5.93662661	5.17957605
POLD1	4.29867326	3.17051556	5.11051681	4.33933339	3.19074925	2.95867017	3.59112248	2.99140643	3.61625792	4.82747701	3.56663191	4.48904603	4.10639347
EIF4B	8.15827182	8.74280058	7.79706571	8.52977826	8.28486789	8.23476698	8.62061907	8.43155274	8.4133247	8.54925138	8.12862593	8.45777769	8.42506953
SLC6A16	4.74389277	3.19311108	2.77066008	1.97996831	3.17796898	2.30865196	3.07121627	2.72150597	2.56279485	2.50468917	3.86604637	2.36925456	3.35070566
SPHK2	3.91339786	2.82228885	4.34743326	2.4844762	3.34004241	3.05170907	3.76550278	3.80419729	3.08254948	3.02538591	2.06397924	3.74986482	3.63193394

RPL18	7.07619028	7.48193999	7.36785848	7.23085027	6.53364398	6.41336642	6.67836246	6.69391276	6.94614949	7.69572375	6.90535614	7.24295524	6.53791572
CA11	5.44202591	4.23481142	3.26834571	3.09645717	3.65426791	4.24982063	4.24006875	4.18063034	4.62024414	4.91904804	3.55817592	4.61663594	2.6952714
ISOC2	3.24972879	3.3310638	4.76944921	3.74280371	2.42237253	2.97920076	3.1026352	2.90733699	3.93149657	3.8599205	3.32600687	3.92749023	2.87178153
U2AF2	5.78888214	5.01484555	6.16306937	5.40864372	5.17350272	4.93349736	5.55528366	5.15580833	5.21907441	5.81745948	5.1663787	5.09882542	4.97399068
EPN1	5.03826412	4.88795949	5.31386396	4.52764193	4.59658775	4.1698552	4.33929947	4.65347696	5.00503703	4.80073423	4.34916983	4.95147305	4.94626564
MED29	6.12582694	5.95108074	6.00893854	5.34280356	5.56000442	5.71996992	5.22316261	5.46041844	5.80782731	6.09333365	5.66354123	5.65120095	5.6993487
AHRR	2.91926061	1.82377293	2.66243732	2.12021015	2.04844457	2.55233872	3.15950653	2.59000921	2.58375612	2.88280174	2.3957335	1.09445334	3.50289442
ZNF275	5.23167803	5.16040316	5.03995547	5.23936545	5.50366855	5.56297731	4.97396092	5.57304064	5.35738512	5.50447197	5.48205652	5.69003728	5.36920961
MTMR1	5.23938077	5.16597101	5.08884559	4.61126356	5.479566	5.04709713	5.30701022	5.86872799	5.29965472	5.24982895	4.92958665	5.20301469	5.56082914
GPC1	3.2876858	3.06677998	4.30665942	2.94868153	3.15035366	3.19194168	2.61829059	3.39109074	2.63270463	4.26189133	4.37986032	3.95489249	2.06846229
ADCK1	2.64846237	3.39514325	3.43972239	2.27539716	2.26396524	2.15957971	2.41558497	1.9230032	2.721031	-0.93824498	3.37498688	1.93629143	1.67414535
HAGH	4.79677038	4.10547547	4.90932858	2.81121887	4.49405569	4.45183981	4.56062292	4.62046701	5.21081369	5.14483067	4.03107284	5.14184065	5.06244479
RNF4	6.23742558	5.66086255	6.23213643	6.47657193	5.71449136	5.68215966	5.49606541	5.57410709	5.13625768	5.69705794	5.26435386	5.71368836	6.0931415
CASP8	5.17570849	4.9819227	5.19013198	5.12823971	4.56325673	4.51188161	4.68388935	4.8639401	5.12194678	5.18357506	4.61797853	3.8887011	5.16225331
LIMCH1	5.24058761	4.89967146	5.30662578	4.14798171	5.70302591	5.91475739	5.55428445	5.5725056	5.5691786	5.80449315	5.75345898	5.72223368	5.38251036
TM7SF3	5.62286621	6.56849696	5.223758	6.05389332	6.25330864	6.36728596	6.51807171	6.06253375	6.59816652	6.49813408	6.68184249	6.21508877	6.10779097
DLX3	0.75244697	0.42469825	0.6523559	0.59159389	1.11050729	0.76686999	0.4743359	0.42799061	-0.24520478	-0.24520478	-0.24520478	-0.24520478	-0.24520478
SPA17	1.74976181	2.44424381	2.42543521	2.60891447	2.47152564	2.9544757	2.16578449	2.07451453	3.32655015	-1.08925324	1.63105151	3.16812503	0.42013969
TSPAN32	1.19918178	1.07645995	0.48744709	0.1049512	-0.02625326	0.62573079	0.18150592	-0.30125539	2.63353624	-0.13319699	4.10703984	-0.13319699	-0.13319699
WISP2	6.53708194	6.89392386	6.25583255	3.62490572	5.88572279	6.94733755	6.14506157	6.18864331	5.09693869	7.02292558	4.55749864	6.63129126	5.71484462
DMRT3	0.8003396	0.4183961	0.42095571	0.4183961	0.4183961	1.30580024	0.57043228	0.73997002	2.02870525	-0.33635727	-0.33635727	2.70383344	-0.33635727
ST3GAL6	3.67998134	4.36017372	3.81152532	4.52903553	4.05638481	4.33312666	4.65023574	5.10458934	3.71536097	3.58684677	4.26134483	4.37387751	4.19119806
ATP2C2	0.02946566	0.33113302	0.03202863	0.02946566	0.33357671	0.03089064	0.30001615	0.03273541	-0.09983155	-0.09983155	-0.09983155	-0.09983155	-0.09983155
NGFR	4.53372834	3.56985908	5.20944882	4.49685108	3.07209615	3.13278552	3.63256291	2.45178591	3.17566699	3.92649248	3.22394784	3.76646872	4.3724576
CDON	5.41452829	7.1615205	4.18676475	5.00684468	4.91558094	4.83157576	4.66324753	5.13680019	5.21007127	5.01693542	5.65532848	5.32354425	5.91384202
TAF2	5.20357944	5.45521911	5.11325151	6.52287123	6.12305586	6.27849398	6.00839379	6.15695526	5.9698916	5.75718911	5.47872487	5.91819381	5.13153143
HIPK2	7.11031602	6.65653238	7.35660211	6.18346056	7.68899606	6.98997614	7.10160382	7.38455184	6.19052684	6.40541911	6.84291655	6.32723221	7.20970672
TNPO3	5.96446486	6.00449481	5.75142172	6.07100078	5.82935976	5.87018715	6.1865248	5.63833479	5.53915562	6.16358195	6.09677622	6.03920814	6.1776514
RFXANK	4.13748357	3.26199034	4.78090317	3.71553974	3.53593243	4.16688833	3.14317775	3.60793558	3.23610126	3.87091044	3.96362832	3.90362708	4.48212498
TMEM161A	4.19952795	3.00091512	4.75630752	2.41964575	2.18552499	2.41223373	3.22369544	2.1972363	3.54726752	3.22563882	2.61565571	2.61565571	2.80616204
LPAR2	2.70599788	1.38187406	1.72657117	1.61976598	1.56086392	0.31707345	0.95684316	1.50964939	3.03098483	2.75877358	2.11982067	1.24523921	2.36741869
CTSA	6.81146778	5.88816555	6.32781827	6.36346978	5.61081045	5.63705155	5.8783819	5.87543071	6.18873817	5.72293509	5.71529193	5.55876384	6.46607381
SUGP2	5.73190259	5.78828429	6.10244552	6.27510666	6.11172067	5.17603207	6.34091571	6.31699392	5.67923273	5.9639519	6.50497488	6.08467015	5.95243444
SLC12A2	2.06349219	3.02125708	2.2108994	4.06393724	3.77813705	3.5939991	3.70034115	3.67620459	3.18465218	3.34937454	3.35484513	2.23596373	3.88923897
SNX24	4.42929333	5.26411259	4.55668411	5.15519866	4.12361182	4.24824487	4.68105575	5.27269427	5.13262947	3.50728497	4.37720231	4.3051767	5.0192422
EYA2	1.22427508	-0.6192279	-0.52974286	0.05352688	0.86171254	1.21126955	1.07667513	0.10656882	0.07928768	4.37133273	0.07928768	1.56342172	0.07928768
CNN2	6.69943165	6.92827878	6.4464839	6.55095098	6.72309785	6.54885121	5.59930586	6.01996719	6.9820154	7.2996207	6.95591309	7.07270479	6.57403611
ABCA7	3.80895225	2.22963014	2.54908196	2.31070787	1.72571888	1.0192219	3.04233546	1.83710518	3.79685185	-0.8974284	4.51900093	2.43754815	1.65380604
SNCAIP	2.70046226	2.72462188	2.08260364	1.69459047	0.86509265	1.8620853	1.16776703	2.51681594	3.37549788	3.77881603	-0.18368993	1.81407034	3.20661783
DDX20	4.00928624	4.82149625	4.45775654	4.80480369	4.84790444	5.42675742	4.44277839	4.53932246	4.69170669	4.23101838	3.76349298	4.10467421	5.25606523
BTBD1	5.29481862	5.65800381	5.02860808	6.37191865	6.01986757	6.37191865	5.5794853	5.83185136	6.02579123	5.27489308	6.14448504	5.73505831	5.60624834
FAR2	0.11924689	1.0234456	0.03211734	2.07031467	0.79462607	0.37866057	0.43199201	0.76660391	-0.0590598	-0.0590598	-0.0590598	-0.0590598	-0.0590598
BCAS1	0.33693743	0.60135736	0.33953927	0.33693743	0.33693743	0.33838402	0.40635437	0.34025677	-0.06222229	-0.06222229	-0.06222229	-0.06222229	-0.06222229
CHI3L2	7.16040748	3.14454039	2.1930438	5.06427612	1.38984539	1.3034817	3.40288669	1.87957624	2.17165446	-1.63971339	2.07588738	-1.63971339	2.63329593
SBNO2	6.82705578	5.92823923	6.81256732	5.78876212	5.02951565	3.67933141	5.09116089	4.34621501	4.65367619	4.69206887	4.96319804	4.48332391	4.17696219
PMS1	3.90895319	4.13252374	3.908492	4.99504945	4.49416483	5.25339352	4.72420342	4.59172522	4.40614495	3.75859427	4.96717095	4.53676764	3.99081187
HMG20B	5.33415706	5.24954189	5.4705931	3.77447788	5.00974548	5.44905801	4.62055057	4.94919087	5.02895124	6.03870419	5.33069711	5.74393155	4.12334159

CALCRL	3.43658723	6.02611205	4.0909561	6.3125286	5.20435509	4.26314212	4.79408284	4.92573464	5.43960696	4.72060211	6.22555168	4.59834404	4.7582101
TAF11	4.35605398	5.05348733	4.87239072	4.98455749	5.24247839	4.991415	5.33262532	5.82490432	4.5611902	4.13892847	4.53707019	4.44574779	4.82801567
ANKS1A	6.15706124	5.91696757	6.26337501	5.2854157	6.10808632	6.13220071	6.34912975	6.35111209	5.89625864	6.46924513	5.90681148	6.48282623	6.19395564
AP3D1	6.59738586	6.57495805	7.11268042	6.48174472	6.09877685	5.76139188	6.60026041	5.94999467	6.24902039	6.59783875	6.44853265	6.28410187	6.28659249
ZNF76	5.26863187	4.54330472	5.11862748	3.84296433	4.71705005	3.83371613	4.08064006	4.72242476	4.0859399	5.21825635	5.43245358	4.58497723	4.23879295
SLC9A3R2	5.41182237	5.62720114	6.23041034	4.41304597	5.43479933	4.807757	5.34489756	4.95380358	5.4377051	5.772743	5.81173063	5.65621951	5.53021022
NTHL1	3.98297251	3.77033612	3.84937641	2.2060643	2.0356185	2.85638144	2.95750476	1.87031488	3.2555064	3.76283241	3.04611922	4.10256265	0.41478146
UHRF1BP1	5.86474595	5.4276547	5.81571232	5.81424336	5.28527727	4.71816694	5.62163873	5.67518609	4.89134456	5.42498732	5.5472616	5.36875145	5.77987131
GNAI3	4.60360326	5.015082	4.92249285	5.86758176	5.1434405	5.1610524	5.46721876	5.28255435	5.3192352	5.10566903	4.89608877	5.3657842	4.99835407
IPO5	4.78493165	5.6264181	4.68091084	5.55252522	5.78826057	5.38240421	5.58799314	5.71469561	4.69959388	5.95238304	5.06658101	5.501914	5.75853558
OAT	5.97738482	6.4557335	5.3875662	6.65103347	6.50594858	6.61036285	6.54181894	6.42344528	6.71534919	6.85618784	6.80673966	6.81717364	6.21515406
WDR3	4.70886496	5.08896954	5.64158687	5.84485011	5.04230062	5.65828067	5.22126626	5.18005823	5.06304567	5.11327211	5.307328	4.63721593	5.67736653
PKN2	5.24753658	5.90032211	5.21811324	5.99378788	5.97979022	6.44529704	6.14956542	6.15689496	6.02235183	5.76431705	5.5202363	6.136681	5.72286642
WDR18	3.03439688	3.59274207	4.2859614	2.64612703	2.31099338	2.29862771	2.16822317	2.33980042	3.25454486	3.48799044	3.0329267	3.42192698	3.5450237
TRAM2	6.37460299	6.57762479	6.48487916	6.61005999	6.01841782	5.94668645	6.33391632	6.17268447	6.04766592	6.28116113	6.24882685	6.08741917	6.28737289
NTN1	5.43776401	3.06510801	5.10561594	1.89622256	3.40022756	4.87363401	5.00427056	5.39057035	3.00631725	6.45144767	1.73338696	5.6240094	4.21938237
GLP2R	0.01582442	0.0277976	0.04668459	0.01909503	0.02225395	0.00069205	0.12155091	0.18916308	0	0	0	0	0
MCM10	1.36211135	1.47735407	2.83585295	3.5859215	1.01218357	1.37773802	1.74202646	1.12290014	3.70478323	2.13816746	-0.84226786	2.56906317	-0.84226786
DGKA	5.68523796	4.92995263	5.79656238	5.24961979	5.39484406	4.60110832	5.36033533	6.01216789	5.12132281	4.83618584	5.46636432	4.7368308	4.70057715
ERBB3	1.27923366	2.86681966	1.30912492	1.41692682	2.04250244	2.27773755	3.29895979	2.61783767	3.19807683	3.25557892	4.60866413	3.60162341	4.97243733
ANKRD44	5.45755153	6.57626132	5.11053952	5.67734706	5.79502031	5.97986707	5.57437598	5.81637262	6.36091892	4.98169224	6.58297267	5.75546217	5.80340035
KARS	6.24222776	6.46751632	6.47483296	6.82915368	5.90651636	6.07160915	6.22993636	5.83145194	6.09835163	6.62854049	5.87646473	6.4134323	6.65164121
ADAT1	5.12789917	5.04514869	5.32924553	5.52704578	5.01519942	4.58769888	4.91079622	4.73288732	4.84627834	4.70334774	5.57913568	5.14954541	5.40409858
PDIA5	4.81858854	4.4922232	5.11984639	5.80310722	4.48301527	4.29442509	4.38519264	4.4396005	4.80270894	4.53924947	4.95504443	4.81869316	5.49039923
TBC1D22B	4.91420151	3.9625537	4.29835191	4.42962329	4.4796743	4.81698756	4.57232334	5.17642571	4.30548524	3.62762964	4.39893184	4.36127908	4.71677189
NDUFB4	4.71510528	4.49980374	3.26633045	4.03023077	3.94773063	5.45262927	4.10456217	3.68924412	3.87664484	4.85742861	4.56498114	4.20185659	4.44858295
SPEN	7.64371369	7.13020361	7.44628754	6.39774303	7.45297211	7.64371369	7.11341121	7.5524048	7.30365521	7.53773667	7.17360061	6.86614113	7.5326008
MYLK	8.42598743	8.80711304	8.63801818	7.50426949	9.16786047	9.65734861	9.15977556	9.30708874	8.87464756	9.33531854	8.95110707	9.2280234	8.87464756
ZC3H15	2.49504059	3.45896568	3.29229091	4.87478329	3.87087161	4.71276919	4.19202055	3.93126623	4.48123705	4.69483609	3.53494675	2.68270344	4.23306929
MAP2K4	3.49351176	4.44755816	3.19283702	4.48293538	4.48259189	4.69940197	4.34511072	4.5023781	4.94003741	4.74056977	4.41565014	4.16687098	4.58198912
TMEM206	4.0060936	3.40315599	3.63335713	3.83618148	3.04811211	2.93966315	3.55726732	2.92458778	3.051531	3.42455649	2.88860408	3.03448199	4.11438951
SNAP91	2.9224517	1.88435799	3.08559727	1.66639409	2.12420923	4.64457626	3.74506993	3.97048446	4.67473721	3.70765958	1.8998387	3.84523293	2.60546725
SLK	6.23012159	6.93528079	6.68578266	6.80771667	7.19522427	7.56013223	7.24497971	7.16982471	7.53203354	7.20111803	6.65211617	6.95741756	6.84131177
CYB5R4	3.63565782	3.88299513	3.95524705	4.80727308	4.82080545	4.4653768	5.06068772	4.96126858	4.0235953	2.46396442	4.29231195	4.9245713	4.26178345
COL17A1	0.42433994	2.08054185	0.72817994	0.97428088	0.1760373	0.17738786	0.41819157	0.1791363	-0.32986636	-0.32986636	2.84059911	-0.32986636	2.05170583
GSTO2	3.70023711	1.83080076	3.49507124	2.73113352	3.52315173	3.99548728	2.84539239	3.34146544	3.72532809	3.869535	2.6767088	2.93032188	3.4231479
SEC61A2	0.8983982	1.11793357	0.12744151	0.80589436	1.4044915	0.53926226	1.0846804	1.91805847	0.96944376	0.29280964	0.29280964	0.29280964	0.29280964
PRKCQ	1.09103728	2.39994721	0.1978946	1.28321553	0.65210425	0.4176946	0.67725616	0.62283481	-0.34525907	-0.34525907	2.66342123	1.90613666	0.71551661
TLE2	6.25606467	4.89277505	5.91128053	3.94644352	4.39056178	3.30357286	4.30753306	3.72244308	4.05972183	3.35167488	4.5734662	3.9416868	3.14485521
ASB1	5.93711419	5.93827863	6.05038727	4.96230703	5.80270959	5.70593104	5.90207556	6.12712908	5.4607462	6.3295773	6.00051657	6.25467158	5.67842749
FAM107B	5.36611478	6.59851051	6.03872808	6.44143811	6.23543881	6.25638754	5.65472394	6.90537258	6.08284686	6.58242371	5.96265813	6.49194202	5.94780775
ME1	5.14944932	5.40167707	4.65269524	5.72196768	5.70422104	5.92331934	6.14726129	5.84668675	5.70278034	5.34024974	5.66149196	5.51727555	5.16795429
TBC1D1	4.85087058	5.13907802	4.63949203	4.32529971	5.60417316	5.1986378	5.50145863	5.46182755	4.57672043	5.8786069	5.24539494	5.20960575	5.27486934
CDK13	6.12912178	6.09965328	5.63235556	5.86898823	6.24943072	6.22685907	5.63123569	6.14786548	6.06218434	5.73364527	6.22036767	5.81823293	5.81509782
MTHFD2	6.88151009	6.13941311	6.33946263	6.58978773	5.8674074	7.75515173	6.9883045	5.62595875	5.39576724	5.71013874	5.59439453	5.4368171	5.49611772
SLC9A7	5.05093023	4.87805517	5.25782721	5.24339003	4.61833741	4.52288443	3.92298304	4.34220516	4.98431827	4.45658473	5.28471907	4.97707865	4.52604741
FOXJ2	6.3392903	5.88683144	6.32503858	5.47917911	6.51778351	6.52148618	6.21901677	6.14728214	5.8899415	6.61799433	6.58590381	6.33886347	6.09863153

YBX1	7.44746827	6.60668322	7.31896633	6.65554967	6.39210399	7.3127732	6.5653777	6.1479018	7.79171724	7.73405034	6.58795337	6.44763988	7.38624314
PDE4A	5.26136869	4.90425485	5.12995831	3.90827065	5.14492311	4.45080014	5.32797607	5.22360443	4.44534385	5.3000335	4.88294271	4.75206162	4.85257615
PPP2R5A	2.67362688	3.9355253	3.35561217	3.463642	4.53205366	4.50999474	4.49996659	4.42651139	3.71575477	4.81240429	4.32992225	4.46670373	3.78985804
ELAVL1	6.00232222	5.69350843	5.52603633	5.62478807	5.74858603	5.41205633	5.50478655	5.25572718	5.18517483	5.92457844	5.12723573	5.6279432	5.84208964
TIE1	0.29869331	4.29095531	2.8627078	5.04254561	4.14160656	1.35646944	2.94589118	3.41848805	4.09053111	4.9739677	5.92493562	4.69592924	5.18551948
DIP2B	5.85544959	6.03194951	5.68612007	6.18351524	5.57111217	5.24922891	5.74180592	5.5832522	5.81739749	5.02172541	5.68128335	5.71660562	6.02171478
SMARCD1	5.76238416	4.98577106	5.61913754	5.62806795	5.15110406	4.80532536	5.36248635	5.19125817	4.44741254	5.62881225	5.66352503	4.81115144	5.66161606
KDM4A	5.61140128	5.50588779	6.03104612	5.71182608	5.06834696	5.02329521	5.48298815	5.46775567	5.5049505	5.43945864	6.02938388	5.90173482	5.56432992
NFYC	5.50450242	4.97107089	4.76704052	4.74626423	5.20111084	5.29292534	4.91375883	5.058229	4.97358286	5.18587964	4.83875066	5.05201583	5.64551288
ZMYND12	1.27826354	1.70602558	1.38400547	2.55966571	1.30909763	2.7084757	1.72266671	0.95293221	3.30921515	-0.80840101	-0.80840101	-0.80840101	3.82845592
NGEF	0.67550693	2.30712635	2.85511836	1.91085125	2.26394069	1.08656836	1.54774333	1.25603611	4.64505649	3.34395066	3.30426264	3.50915062	3.83364309
ASPM	2.40589257	2.61783125	4.09244541	5.59605842	2.28851414	2.37485228	3.07972245	2.75476033	3.33668107	2.78726528	2.85248758	2.70489803	3.25040275
CD84	5.66144151	5.11011045	5.37896187	5.96859662	4.23187119	3.6636838	4.30482008	3.79389716	4.48281495	3.96534536	4.31112104	4.97986142	5.06035807
ELOVL1	5.48314593	4.94573287	5.57589205	5.41218315	5.05227454	4.83048536	4.73702558	4.36876593	4.94206729	5.58373895	5.29469923	4.89057123	5.52668447
SPI1	4.59548313	3.80031273	5.18208718	4.11353374	3.07546536	2.32452183	2.38314153	2.4763116	4.64311948	4.11771178	4.08281422	3.11478946	4.09314548
ZNRD1	3.34847603	3.84161277	3.78723768	3.08274972	2.98089895	4.10512649	3.37551605	3.46860057	2.70333473	2.73054084	3.10948931	3.56656966	2.67401806
MPPED2	5.44151502	4.47196924	5.41077558	3.88961393	5.08150237	5.94526525	5.62024295	6.20741175	5.83325137	6.59207984	6.1032128	5.98013235	4.95910626
ZBTB11	4.9608632	5.76765915	4.9555957	5.80695301	5.65420131	6.17286145	5.8518454	5.71314075	5.93733751	5.62455895	5.0201434	5.28364456	5.43791194
ATXN3	5.47723587	5.80490488	5.39700172	5.15140573	6.12755224	5.89123239	6.06238446	6.29962932	5.84405074	5.61441971	6.08149439	5.93187471	5.48576131
GOLGA5	5.59469316	5.97087148	5.85490823	6.55809335	6.33455949	6.77462724	5.60055606	5.90024983	6.61524611	6.26384383	5.83379102	5.92540651	6.57860636
FGFR2	4.08433322	3.74892434	3.32478925	2.95388373	5.20980629	4.59723164	4.96866446	4.93679861	4.50049521	4.75144778	4.41769201	5.00435571	4.86158989
LRRC40	3.72843182	4.14479562	3.9580286	5.32371101	4.88371514	5.05265337	4.77688008	5.08299678	4.88260497	3.39878613	4.81016739	5.17377148	3.70558251
ISOC1	2.8476087	3.3816501	3.35651328	2.61098871	2.66761943	3.63191668	3.05562796	3.27798002	3.10729885	3.96430075	3.3699309	3.71344768	3.55065686
EML1	5.65025584	5.76617726	5.43135497	5.73881025	5.60372523	5.87682654	5.10800672	4.97062182	5.67878856	5.37393991	5.85922288	5.39364599	6.04380075
TRMT11	4.14753324	4.57380319	3.81070109	4.97731173	4.56705114	5.15205055	4.73494097	5.18017823	5.08419948	4.30884915	5.20387392	4.92808646	4.38564405
THUMPD1	5.94212423	6.55386145	5.85808831	6.43663148	6.44438185	7.08839496	6.55632508	6.52972311	6.62471192	6.65257431	6.25641174	6.68512816	6.27242219
KIF26A	1.09469261	1.35685426	1.97479652	2.63912194	2.48695875	1.65037664	1.90551287	2.48842458	0.84105534	3.70240377	3.90669747	3.97664004	4.96002959
ATG2B	5.76405444	6.15794205	5.42542294	6.18454045	6.36425701	6.33292509	6.38677956	6.67746293	6.21329036	5.73366532	6.3828178	6.18384355	6.64968361
ARFGEF1	2.21407463	3.56957683	2.5777021	4.26425834	3.736423	3.61491335	3.94510921	4.06952468	2.79464373	-0.60876369	3.35819675	2.63641454	3.01504225
ZFAT	4.08890752	4.161478	2.80874238	2.73327066	3.95228243	3.53663362	3.59661976	3.66495381	3.13393729	3.64909093	4.40797141	3.75072252	3.27315905
MTFR1	4.97310073	4.74315343	5.05409205	5.1458183	5.36079619	5.87752435	5.72838069	5.67167135	5.04168344	4.79121382	5.19748357	5.29401845	5.09499237
STAG3	0.8483963	0.75970142	0.99530785	0.09446584	0.12288289	0.25035502	-0.22714181	-0.22714181	0.96596361	-0.0901477	-0.0901477	-0.0901477	1.6745725
FECH	5.00348989	5.16298054	4.86742451	5.26368292	4.24745896	5.19902646	4.76897488	4.79043872	5.20286049	4.97376519	5.52967917	5.70260274	5.41163185
MYO9A	6.56017756	6.88963169	6.28161548	6.75730771	7.20467146	7.28662695	7.05985585	7.19104947	7.07846474	7.07739083	7.00034953	7.07516865	7.03366642
DDX3Y	2.82141834	4.34095533	3.01648171	5.19183212	5.26274505	5.72957802	5.45112568	4.66099295	-1.20673782	0.99447586	-0.28311481	-0.76336649	7.02773103
PFKP	6.36076789	6.07546148	6.44563195	5.48831342	6.98892929	6.24583103	6.88189064	5.70403498	5.43835398	6.64068362	6.36514088	5.85702939	6.05869447
IDI1	4.2558062	5.30505273	4.14311545	4.72692988	4.6807457	5.41238668	5.43641498	4.83472555	5.70903907	4.12274033	5.55359262	5.13438294	4.80772324
SP100	6.50831387	6.8842443	6.11816934	7.08876129	6.62687984	6.1073986	6.54831791	6.31158205	6.39134531	6.64165626	6.30497406	5.74719346	6.57287646
KLF6	6.80220763	7.62013798	7.12916227	7.34767526	8.16184913	7.51264436	7.51264436	7.98822018	7.64343498	7.39564819	7.508069	7.42383692	7.6496957
NEO1	6.04199749	6.09849244	5.49536184	4.54987605	5.96362822	6.35378871	6.06207016	6.10025895	6.02512781	6.0925564	6.36061389	6.1985643	6.02882288
TRAM1	6.37351285	7.08614002	6.45044026	8.13875197	7.72523427	7.64533224	7.8682064	7.81401672	7.9446128	6.51034043	7.69679333	7.40719934	6.90046064
PHKA1	5.11468554	4.46508513	4.6725911	4.08161467	5.05915369	5.32239686	5.64801065	6.03003713	4.37768479	4.53746215	4.417403	4.71498871	4.99520053
TNFRSF1A	7.07773089	6.61719488	6.86573898	6.73873943	6.08389917	5.40142464	6.36435352	5.83310596	5.63240303	6.3138727	5.9917983	5.93439686	6.18234376
CACNB1	4.90815914	3.53899518	5.15323714	3.83295315	4.7481477	3.99402044	4.38205108	4.90868408	3.632644	4.80313733	4.17736542	3.56551548	4.53732213
EVI5	5.46135078	6.03115487	5.38419619	5.9640739	5.97138459	6.05608539	6.13540084	6.27395746	6.20466408	6.13077488	6.19150352	6.23325954	6.10112881
STOML1	3.76513623	3.46332331	4.6250379	2.96916277	3.05794475	2.93317468	3.34996897	3.15314513	4.27620511	3.21184	4.53574533	4.1024077	2.95346079
DHX29	5.2615384	5.91900451	5.18303142	6.36526038	5.92131901	6.52009774	5.92850313	5.85035349	5.97501079	5.20055971	5.40592414	5.85634717	5.45065856

DNTTIP2	4.83382418	5.03866313	5.7516888	6.23894994	5.8968548	6.70106377	5.80078894	5.55751029	6.20125567	5.6844198	5.4463684	5.45961091	4.80884335
METTL22	4.91222662	3.66050242	4.62531775	4.00073251	4.24190213	3.66948992	4.48786205	4.26435497	4.27499578	4.01641325	4.7144627	4.44929293	3.28778626
TP53BP1	6.3416278	6.67057003	6.23958534	6.55720701	6.12963059	6.01359351	6.33439551	5.82507741	6.35493652	6.16946506	6.38577089	6.22171398	6.68496338
TRO	5.0103399	3.93212918	4.17481005	4.79920898	4.42889816	4.48830274	4.60188149	4.5296015	4.77346677	4.52559696	5.23490817	5.20213928	4.18904006
RRP15	4.65712947	4.70005638	4.74264462	4.76410188	5.51559598	5.85633451	4.96110778	5.23465984	5.36411078	4.22693828	4.79178284	4.6383789	4.86311064
RHOA	7.7975491	8.30988914	7.72702963	8.51373659	8.34357635	8.23890675	8.47712044	8.33267143	8.25280859	8.33641844	8.21543053	8.22955976	8.25633084
DHX8	5.95241935	5.9909542	6.13931175	5.91527401	5.6755879	5.9267361	5.72934872	5.68178392	5.62671432	5.69569328	5.81172729	5.9435958	6.18928831
PRKCZ	1.39976162	0.826055	0.61740309	0.96787088	0.50671654	0.19335068	0.79049593	-0.10685347	3.57306954	-0.1860952	3.31455843	-0.1860952	-0.1860952
ZFY	1.48975151	3.22804826	1.87441064	3.65530905	3.99249527	4.42100574	4.21536725	4.39713993	-1.8164876	-1.8164876	-1.8164876	-0.75944259	5.73042326
IARS2	4.88738009	5.44436369	5.15490663	5.67256498	5.52962173	5.82997528	5.82052362	5.70337693	5.66337123	5.92124397	5.24989163	5.54542877	5.56051126
SYT1	0.66461547	0.82188456	0.9733971	0.78071092	0.98880421	0.90006512	1.18759865	1.42646185	4.26446735	-0.38862856	-0.38862856	-0.38862856	2.05395062
NAV3	0.26546476	0.69705787	1.08921068	2.82953751	0.68683432	0.46876579	0.32597625	2.18333926	-0.27124108	-0.27124108	2.69402352	2.63531604	3.03333732
IDH3G	5.74148057	5.03469817	5.62318551	4.83263037	5.01582471	5.22258298	4.54911497	4.8772074	5.4934097	6.41824043	5.47054662	4.82096523	5.17546103
ROGDI	3.89423133	3.45912162	4.57886157	1.94300894	3.07071751	3.31233671	3.36057385	2.68810225	2.80111276	3.98802891	3.79934288	4.03909419	3.60215094
PDZD4	3.75610278	3.51666506	2.32434082	2.19933654	5.03726199	3.44306425	3.62168797	3.47048026	3.62410112	4.73024195	2.9861014	2.34022291	3.0864451
ATP2B3	1.7966056	1.3592561	1.94566727	1.65451313	1.8485421	1.57672554	1.7470949	1.48216672	0.39433012	-0.79684367	-0.79684367	2.90408241	2.66021947
ROCK1	6.35955622	7.18333523	6.86564984	7.04113224	7.60618452	7.66702826	7.51350476	7.61998598	8.00756629	7.55404268	6.77812962	7.36159845	7.21417694
CBFB	5.13106547	4.70973588	4.88597797	6.26374156	5.46505543	5.04077441	5.68806592	5.38719652	5.3767298	4.33703049	5.01370675	5.24208914	4.55012748
PDK3	5.36679572	5.40713605	4.84100333	4.89877431	6.03949343	6.20157552	6.38343151	6.08032138	5.63791332	5.56130468	5.83527904	6.47181405	5.08662707
HYAL2	3.44336644	4.54478527	4.43337388	4.53813649	4.6721249	3.10458948	3.8423983	3.27935505	4.26810202	4.98825937	4.54726428	4.12419236	3.52529505
HDAC4	5.69577181	5.19681057	5.51997089	4.34429129	5.13303893	4.82810285	5.15971048	5.02989735	4.61681474	5.00986356	4.9064669	4.86473804	5.00531629
RASSF1	4.1314385	4.53364897	5.23970615	5.10752896	4.20608388	3.61105156	3.71157386	4.2772537	4.53824587	4.89443186	4.42603918	4.57509109	4.15756968
FGFR3	0.33574225	0.33882822	0.29593403	0.3594016	0.93710463	0.25960864	0.80868226	1.60985849	-0.15390396	-0.15390396	2.19851936	-0.15390396	1.16152433
IFI35	4.64098933	4.63880767	5.1924588	4.05167271	3.51394476	3.82978993	3.49815315	3.19273405	4.44752451	3.54370321	4.15807445	3.45348705	4.68695245
HEATR6	5.05398806	4.59645029	4.32550901	5.08378048	4.46816052	5.33517443	4.82944066	4.54018511	5.09770319	3.71140065	4.90266746	5.14517152	5.27060086
COASY	5.16635348	4.90381643	5.25361712	4.60947112	4.61216346	4.22309502	4.60011409	4.349672	4.23292006	5.27945848	4.83284168	4.54979082	4.54810409
PLEKHH3	4.40708802	3.85568889	4.91844362	2.56391571	3.63237726	3.41770461	3.83906888	4.11628787	3.64823991	5.06904569	4.60695997	4.20317641	3.53840291
MEF2A	7.62250731	7.88146436	7.09019112	7.45018459	8.4940417	8.2878298	7.72489618	8.55618575	8.13320613	7.76440321	8.30957894	7.80679031	7.9737915
OTUD5	6.20666252	5.65286191	6.2012854	5.36506726	5.6970828	5.65398547	5.46361756	5.58906118	5.75099596	6.45528107	5.65540604	5.62256579	5.57132897
TFE3	6.69771951	5.82400143	6.35757335	5.68290259	5.9459018	5.55343849	5.87964568	5.55170846	5.4566153	5.99482784	5.79637144	5.54727588	5.43428226
TBC1D25	5.08173475	4.3578768	4.85300179	3.85306467	4.20257963	4.29971954	4.23875242	4.59986161	4.0760997	5.06155413	4.45502429	4.80128461	4.41254171
ACSL4	5.80920873	6.43860142	6.01441248	7.16200184	6.77665635	7.03011604	6.78628651	6.79815277	6.75749825	6.09928544	6.53379777	6.49795378	6.51447601
INPP5A	5.71461573	6.30287821	5.57254101	4.70670132	6.78599545	6.64799382	6.37481388	6.38084028	6.29452162	6.60628518	6.62046354	6.80762795	6.35088417
GPKOW	5.39002207	4.50638049	5.23872169	4.16639533	4.6236023	5.33865773	4.60086031	4.59183041	5.31527456	4.82654686	4.43019963	4.43158793	5.45319544
GRIPAP1	5.91384113	5.24781613	6.02396847	5.11846715	5.34873409	5.06229727	5.45231529	5.25862979	5.75640706	5.2539424	5.07516707	5.04956496	5.94636591
FTSJ1	5.4039882	5.02753295	5.65705942	5.58992857	4.76460183	4.64012646	4.84113796	4.96262904	5.24252233	4.95048446	4.73976976	4.9674758	5.18928281
PRR11	0.59641543	0.04925162	1.88775149	1.97609912	-0.29272253	-0.31184953	0.33958484	-0.37811268	0.17460728	0.17460728	0.17460728	0.17460728	0.17460728
REEP1	3.60442946	4.88729107	3.85443686	3.09054577	6.1346691	6.73478106	5.63129024	5.95169229	5.30580858	6.14807853	5.88051906	6.14845476	6.17567499
ATP11A	1.66728351	2.26674833	1.75927625	2.2083624	1.23992614	-0.12292377	0.7673568	0.70851889	2.66681255	0.11994611	0.11994611	1.76683872	0.11994611
POLR1A	6.01278666	5.11338316	5.9567819	5.82764173	5.15597398	5.42100468	5.46230307	5.02557723	5.18078078	5.64007664	5.50595758	5.31355867	5.81073679
LAPTM4A	7.34200258	7.35904164	6.47537193	7.70851747	7.73765422	7.99707639	7.58355624	7.84263938	7.4133649	8.04106306	7.63350674	7.79223653	7.20068315
TTC7A	5.07844991	5.62583149	5.40927201	5.16812422	5.00546911	4.29725799	5.04693932	4.99069313	4.55445966	5.38143554	4.87178851	4.51126535	4.95652427
IP6K2	5.60800631	4.93556125	5.85709882	5.30641285	5.58424239	5.29876968	5.86538913	5.78535551	5.27536251	5.71657233	5.48340425	5.03493402	5.51172786
STON1-GTF2	0.80030295	0.7489169	0.69855394	0.78563975	1.03751687	1.3581242	0.72224628	1.63154808	-0.5949944	-0.5949944	-0.5949944	-0.5949944	-0.5949944
SRBD1	4.4738803	4.97657263	4.92357695	5.6153817	5.00265066	5.75146003	4.63681068	4.97518021	5.32051084	5.20059287	4.40261887	4.84517448	5.37835876
KIF2A	2.92345728	4.16411717	3.19930705	4.4995364	4.12907666	4.24530951	4.14658507	3.67472475	3.48967174	3.99555226	2.41133444	3.80843483	4.26129088
RASGRP2	4.44737376	4.2542067	4.64186141	2.64953308	4.95551184	4.49158124	4.95686894	4.96633412	4.45783525	5.7506026	5.0437625	5.14806145	4.23342547

PSME4	5.54824633	6.226498	5.97996903	7.0915038	6.59645466	6.48642064	6.56073533	6.96501333	6.18345891	5.51480675	6.65902812	6.39045393	6.56956709
IFT80	4.36899345	5.0649415	4.3186745	5.3226647	5.15158899	5.39099143	5.67816526	5.73185982	5.58746012	3.7517311	4.79180948	5.1254117	3.95428142
SIRT2	5.17723678	4.41213834	5.33782808	4.05037168	4.49822488	4.47026214	4.22409612	4.6037242	4.61311691	5.82970024	4.44594618	5.06213147	5.39878629
ERLEC1	5.34166024	6.26352782	5.57386221	6.81453945	6.46542129	6.71108691	6.35605582	6.43191749	6.7902248	6.3737755	6.63254537	6.82970098	6.19112976
PPP2R5B	5.06162471	5.25099048	4.76513383	3.85514101	5.21535883	4.0269271	4.85338086	4.68653382	4.52317496	5.29826565	4.80846944	5.68621661	4.63294534
PYGM	5.55320031	4.67205905	5.06045672	3.11321425	4.90370506	5.71260291	5.10413686	4.57623006	4.58380981	6.2019741	4.83961629	5.96852984	5.79508458
PITX1	1.2656346	0.32264392	1.68481208	0.55694643	0.51051112	0.16186724	0.54932733	1.56246466	1.82038022	-0.40458106	-0.40458106	1.88707246	-0.40458106
MAST4	1.27838801	1.79111173	1.07638504	0.54449743	1.08198756	1.18451412	0.29507867	0.89518057	0.71433376	0.71433376	0.71433376	3.9702214	0.71433376
SDK2	5.06523219	2.73763932	5.36262768	2.93468922	4.46148917	4.07790578	4.97010306	4.92716014	4.02319344	5.70735358	2.99726398	5.48953828	4.471638
NUP133	5.41822675	5.87446333	5.4555316	6.04190501	5.64870566	6.09378023	6.07180573	5.93718168	5.54031779	5.73196126	5.74739917	5.91961369	6.05014286
NUCKS1	7.2124319	7.89826988	7.22464973	8.11923422	8.11923422	8.06012554	8.11367723	7.99437523	8.22211011	7.74074842	8.25077129	8.12470056	7.54177701
VPS35	5.96621066	6.50365602	6.2763676	6.98718653	6.50810663	6.89989981	6.67210764	6.30523454	6.77537915	6.93560116	6.53713497	6.71376257	6.94560668
DNAJA2	6.3109042	6.41175138	5.77944609	6.47106356	6.38685931	6.63982319	6.49605393	6.24971742	6.35498744	6.40068787	6.31718541	5.93987706	6.52346103
BCL3	5.55089471	4.83075165	5.34064606	5.18239521	2.58732422	2.67979694	3.50326459	2.43226531	3.34485006	1.62003007	4.173301	2.77968155	3.57921898
KCNAB2	4.89059688	4.5210885	5.07423758	4.65216198	3.59571087	3.35335257	3.92227599	3.32349445	3.63452806	4.52657369	3.00672878	3.94240897	3.29747286
ABCC9	6.58135851	6.46305833	4.33444428	5.58738335	6.49699515	6.22493131	6.62186658	6.68082895	6.32240989	7.05034957	5.79680301	6.61901726	7.04232584
GAL	0.325742	0.18181595	1.47830044	0.8662964	-0.30415573	-0.30300966	-0.30415573	-0.30152595	-0.05305203	-0.05305203	-0.05305203	-0.05305203	-0.05305203
CLEC2D	2.65660663	4.08628996	2.80232653	4.67201821	3.91142254	2.72139118	3.35322388	4.15322549	3.36483685	-1.34436651	4.15135337	3.50782118	4.34650749
FUNDC1	3.8858042	2.76525381	4.16254614	3.94177437	3.76869946	5.17325439	3.45674252	3.57770861	4.83725161	4.82438958	4.25910901	4.07788799	4.27395787
MAOB	6.97903403	7.16530624	6.66305487	5.90101554	7.57727316	7.66495479	6.75965475	6.94038371	7.08158144	7.29268707	7.13548985	7.43026202	7.31042436
RORA	6.2734668	6.1750498	5.50296053	6.13596959	6.83842469	6.54548726	6.55092147	7.2210955	6.49941161	5.78676003	6.200473	6.51759554	5.28894308
DRD4	1.51657369	0.81150287	1.82269923	0.86203446	1.23691221	1.00837775	1.19032844	0.52680241	0	0	0	0	0
TGFBR3	7.21162159	5.78532462	5.5989231	5.26970312	6.12936447	5.39065034	6.26408508	6.33023174	5.4705587	5.28862232	5.54962341	5.69248052	6.13603878
HES2	0.36366255	0.00039113	0.0029454	0.02413026	0.00039113	0.00181127	0.03642866	0.00364979	-0.08079889	-0.08079889	-0.08079889	-0.08079889	-0.08079889
ATP1B3	5.40683683	5.45965921	5.44682745	6.67806588	6.18097108	5.36042307	6.18444735	6.13124745	5.87132546	5.41940775	5.67556776	5.79502961	5.63601383
NEDD4	6.30230896	6.12096749	5.94689745	6.43425496	6.86985684	7.48353068	6.77658895	6.98992262	6.66038878	6.65786483	6.51814244	6.88131292	6.49986968
PIGB	3.75573162	4.68113793	3.75177277	5.10513941	4.86106953	4.79431591	4.84410477	5.04094081	3.96424033	4.94618544	4.48188739	5.03906226	4.34160686
MAPK6	5.07427595	5.44706981	5.55482146	6.79309846	5.67254541	6.22942991	5.82745764	6.01886603	5.47341553	5.30950089	5.77941531	5.38547289	5.24880188
GNB5	5.2678017	5.01448173	4.77700011	4.19528462	4.89900871	5.10993985	5.15340987	5.0751305	4.55805443	5.09567643	4.91676582	5.10425122	5.1126838
RAB27A	5.52833001	5.36169573	4.91663688	5.9494447	6.52823336	6.07174718	6.07612537	6.1511969	5.89131358	5.12117636	5.16588948	6.1052906	5.05879269
LRP6	6.72213598	6.45672849	6.25537177	6.22554397	6.75014326	7.18695758	7.31288673	7.46103332	7.25616365	6.5997871	6.75427504	7.05637725	6.41102219
GUCY2C	0.39910742	0.59773757	0.31545296	0.31285635	0.335907	0.56523113	0.44213277	0.52580229	-0.15181378	-0.15181378	2.04497084	0.87692182	3.1401153
PHRF1	4.14838775	3.86325978	4.56960307	3.52243567	3.82693623	3.31928424	3.78714868	3.8007691	0	0	0	0	0
NUCB2	5.69097963	6.48453114	5.79521696	7.35731254	6.31435039	6.6606462	6.55434334	6.51401112	7.49066186	6.02828353	6.42362502	6.25127977	5.7429356
PFN2	5.80781424	6.97316135	6.23268014	7.0605781	7.25719545	7.44573128	7.65067521	7.36840792	7.42547482	7.41986919	7.55808502	7.74429614	6.98170191
PTPN3	5.1580951	5.20477556	5.37227197	4.78892347	5.81718166	6.14517075	5.99009038	6.17117074	5.61353489	5.81917123	6.0787157	5.94194624	5.54163056
SPTB	2.89559359	5.05597763	4.42765712	2.50407547	5.10243179	3.54923507	4.57808272	3.89169613	4.18209349	4.78577644	4.98907108	5.13174834	5.36498065
DAPP1	2.24337641	3.30056827	2.52115899	3.84304395	1.49382874	1.35020588	1.71042607	1.71662158	3.72442322	3.1113673	1.99030752	1.25148185	1.99330727
FGF10	1.32011751	0.93235197	0.80314029	1.42681729	3.32721034	2.56716637	2.59402575	4.47789793	4.13902582	2.56113905	3.80333586	3.53849357	3.91199735
SLC44A1	6.2133139	6.46940556	6.19132337	6.73165812	6.76709107	6.87717872	7.08042098	6.95026025	6.75723843	6.15768899	6.96466466	6.4389928	6.0609416
SMG6	6.14172906	5.56912966	6.0712493	5.41563423	5.41747146	4.87769875	5.33061286	5.32177411	5.12110304	5.41214348	5.14498147	5.41379236	5.4290575
EXOC5	5.13956254	6.02869301	5.58914358	6.6297338	6.52356048	6.71238752	6.61362602	6.35561932	6.57616216	5.85533702	6.53850322	6.41904087	5.15437341
CLTCL1	3.58792335	3.3623622	3.59936488	3.61495378	4.04426473	3.43787472	4.14137238	4.28153563	2.2835857	3.27908416	2.92930635	3.65391293	4.91859886
FGF22	-0.16941169	-0.30628471	0.07082961	-0.05140609	0.26493282	0.13984918	0.57023852	0.91954837	-0.01534273	-0.01534273	-0.01534273	-0.01534273	-0.01534273
FSTL3	5.04607219	5.15814345	5.73638106	4.20906194	6.66335685	6.16208504	5.62849997	6.9356087	4.66519743	6.78361704	5.31573759	7.22900142	5.60144783
DGCR2	5.72971781	5.6952173	5.9848766	4.71186691	5.30957766	5.30143976	5.68510366	5.32267151	5.20180545	5.98291565	5.7018389	5.66887901	5.30354471
RNF126	4.27229609	3.46186073	5.00133966	3.3401264	3.47756536	3.16972602	2.68574397	3.46740509	3.18923201	5.16588232	3.31162453	4.20514543	2.76336302

MNT	5.64867263	4.72813726	5.85248669	4.60689198	4.73869313	4.50893499	5.12266477	4.92013521	4.49311095	5.51098318	5.12316167	4.88442274	4.59326028
ZXDC	6.29671288	5.97929537	5.96356086	5.43390844	5.90648457	5.41616903	5.98964833	6.08684884	5.44908298	5.52865023	6.16869718	5.83767499	5.72278234
JMJD6	5.61123165	4.97142278	5.2381233	4.46362658	5.58926084	5.30982808	5.49314593	5.12588293	5.03370683	5.17447857	5.15057469	5.24099024	5.1749691
POLB	1.31330241	0.84309281	1.93415524	2.82665375	2.3186048	2.17922169	2.4091979	2.28143393	4.14363873	4.13880265	-0.19474305	3.07315275	-0.19474305
ST6GALNAC	0.72141548	1.23893182	0.83496343	0.6252139	1.59839916	1.15088469	0.98405832	1.46887682	2.50520281	3.01945512	3.07734049	-0.28608535	4.14885862
WIPI1	4.05322012	4.07950114	3.69999082	5.27788274	3.84508337	3.76103194	3.42855099	4.37950275	4.33072412	3.20805178	4.57836604	4.50266593	4.19529401
FRMPD1	0.17443639	0.0646847	0.06725718	0.08201382	0.13497413	0.43494798	0.35749936	0.44163553	-0.08977885	-0.08977885	-0.08977885	1.72566766	3.37583339
GBA2	5.2369813	5.74086501	5.19309167	4.37211883	5.49177468	4.70858588	5.16221293	5.53217801	5.24382198	5.34668539	4.6788246	5.48513917	5.37695592
NDST1	5.87309628	6.40036503	6.39671505	6.77935875	6.31181115	5.60978662	6.11672364	6.32896744	5.80641193	6.55178499	6.62237906	6.70858243	6.85728699
ASNS	5.31883846	4.35211817	4.67681292	4.14749237	4.67298055	4.2951272	4.4162581	4.70359256	4.16186921	3.67695239	4.77430878	4.67790008	4.07755998
AP3M2	5.01970926	5.94283229	5.75778619	5.49116979	5.8330082	5.67303754	6.05095632	5.89017469	5.72239363	5.75644295	5.83365158	4.73837588	5.98038017
ST6GALNAC	1.8065863	1.91247604	1.49308999	1.35642883	1.39731693	1.31703873	1.82663249	2.46718112	3.24948343	2.60953259	4.03577459	1.77741501	3.44343386
PABPC1	3.59359083	3.19287661	2.82259373	4.26207625	2.69764908	1.51769494	3.03497485	2.72041087	3.93486157	0.69542698	2.64923385	4.36537705	3.02801735
TESK2	4.3480668	2.85428787	4.11888427	3.31748968	3.7464853	3.58739653	3.78652354	4.03270636	3.88694796	3.32781673	4.02091915	3.74774343	3.15681881
CSNK2A2	5.63189934	5.54243795	5.10785662	5.5848123	5.6924301	5.37760798	5.83377435	5.67065401	5.4827516	5.80235895	5.9551496	5.70025457	5.29057597
PTPN21	6.47629842	6.77485962	6.06179765	5.3201256	7.09516585	6.93890444	6.8995065	7.28902831	6.84565727	6.28901201	7.12033776	7.18857082	7.18915941
EIF2B3	4.74695234	4.35294421	4.66225652	5.42331123	5.24121551	4.97093383	5.08999303	4.54058957	4.78803049	4.52644183	3.88459105	5.13023774	5.39961226
CAMK2A	0.48698436	0.31792512	0.45424796	1.24593996	0.39587473	0.45738976	0.349799	0.3211908	-0.22844194	-0.22844194	2.69682245	2.15570969	4.43361171
TCOF1	5.56581266	4.7238321	5.95563736	4.78124394	4.78207973	5.07810704	4.89331752	4.81688108	4.84807792	5.56416	5.06931037	4.72990799	4.45477836
CDC42	4.90935457	5.51169172	5.05797156	6.1686193	5.81718359	5.92780576	5.696957	5.65170289	5.64860871	4.99343622	5.03553318	5.34586106	6.05783867
OSBPL3	3.8027797	4.82452284	3.34659931	5.1369253	3.49174906	3.025337	3.32754366	3.03211855	4.31648708	1.56018147	4.25607654	4.02263664	4.50542462
RAD18	3.65306663	3.62180187	4.07512553	4.77079835	4.25539077	4.21937104	4.16906939	4.43157096	3.79422045	4.08719093	4.58309159	4.05050659	3.37268092
ATP2B1	4.66500762	5.87621255	4.59891482	6.51281086	5.49678457	5.51742543	5.57026631	5.66100735	5.58537907	5.28300533	5.78416919	5.8031051	5.51259484
NCK2	6.08801562	6.03557827	5.70527393	5.45786398	5.83165142	5.45062608	5.30211417	5.4487427	5.61844288	5.60430744	5.84298275	5.36336917	5.52347638
MAP4K4	7.53879757	7.29878789	8.54744934	9.21706258	7.08041103	6.80370256	7.56593343	7.49109984	7.0476764	6.98443016	6.86095551	6.75453727	7.67930758
MGAT4A	5.23693594	5.75818584	5.31785107	6.66552945	5.66033628	5.2155492	5.76817588	5.15775439	5.18232996	4.58032399	5.44636389	5.33075344	5.02110454
RPL31	5.7769291	6.73483746	5.28106749	6.05109758	5.89308732	5.7330176	5.39019179	5.62836961	6.60136262	6.74627661	5.47237546	6.00340521	5.99073754
WDR1	6.96189464	6.89549075	6.57953365	6.24422461	7.21447206	7.12046484	6.59409555	7.30069543	6.4661542	7.73769119	6.97856631	7.30943004	7.05509844
SNX13	5.3270386	6.11553183	5.38735456	6.50035166	6.37295752	6.33066177	6.43915216	6.36219999	6.25907881	5.75137836	6.28771199	6.23743435	5.43948723
ARHGAP10	3.89562024	4.69964017	4.05280451	3.64507836	5.33566301	5.66911089	5.11582593	5.62832185	4.61131084	4.27792702	5.10632016	4.7956463	5.32654631
RPS6KA2	5.69007659	5.98338508	5.00569621	4.65196441	5.90233592	5.54970738	5.77275181	6.64465858	5.88431687	6.10888379	5.94461822	5.41220273	6.43786746
ING3	3.86875673	3.9223254	3.90064615	4.59181895	4.62528557	5.01662104	4.95341888	4.80586154	4.81615371	4.57847973	4.87894095	4.97733196	4.48807851
VASH1	5.01720458	5.70427241	5.64466521	5.73814628	4.72829013	4.45480009	5.04677692	4.57925852	5.49787177	5.90655566	5.58873022	5.12109642	6.0038445
LMCD1	6.29948662	6.58226238	6.33236026	5.63667786	7.39065165	7.7356896	6.74337562	7.58873781	7.08871136	7.51524555	7.06855032	6.974546	6.61866589
SEL1L	6.77951867	7.19153054	6.62600978	7.30359651	7.51789207	7.77049452	7.47909894	7.16456992	7.02479433	6.9813498	7.08755874	7.2601987	7.12022844
TRIP13	1.25917877	1.12088617	3.98017194	4.08532616	0.68164057	0.98775373	1.19210467	0.6377676	2.10156288	-0.76340771	2.13883362	3.40346224	-0.76340771
ATP6AP1	6.04753471	5.15857972	6.02165158	5.44861344	5.34354113	5.28707022	5.33922875	5.22713325	5.09651172	6.24909275	5.92094311	5.895965	5.57096457
TCF3	4.70060209	4.38119706	5.15939537	4.70654379	4.12962717	3.82262129	4.10272624	4.12271842	4.24775002	4.63882474	4.25743131	4.47731455	3.48954286
TRIB2	5.23647556	5.242205	4.86732708	5.23012057	4.75690811	3.83863153	4.8373476	4.88889944	4.72931152	4.20426846	5.03418661	4.91889123	4.58942757
DAZAP1	5.95697353	5.55359813	6.45478049	5.66412686	5.59618713	4.50989138	6.0573647	5.94853652	5.23283337	4.54710672	5.68521279	5.20803719	5.63932776
MBD3	4.72678372	4.79363062	5.62549408	4.80992326	4.59580516	3.77008246	4.74891777	4.64271744	4.69398144	5.61230224	5.01699303	5.47362123	4.87518155
HLTF	5.07029102	6.00046915	5.34573834	6.25248302	6.02420394	6.79566604	6.49981824	6.51819721	6.35656174	6.45885903	6.07304915	6.41375279	6.23253717
FAM50A	5.63097623	5.35614504	6.19867382	5.12904583	4.9571447	4.94830832	5.47202308	5.0260901	5.80919317	5.36207532	5.38473726	4.78253809	5.64678262
FAM3A	5.08951576	4.73875667	5.57597764	3.85080956	4.38275969	4.26568685	4.2373583	4.34404715	4.31295119	5.52006718	4.51307573	4.58487569	4.18865052
CPSF1	4.18679478	3.55062282	5.07030679	3.91882296	3.39323393	2.9680395	4.30312023	4.13091988	3.81132674	4.0056343	3.26384329	5.00614766	3.03102187
MYO3B	0.05945579	0.0965852	0.01953169	0.08133034	0.03481495	0.15069501	0.01694187	0.14039639	-0.0246478	-0.0246478	-0.0246478	1.59562284	-0.0246478
CYBRD1	8.16995375	8.79217676	7.59777895	8.0716528	8.96197121	8.85826867	8.96197121	8.98058237	8.83091941	8.4021083	8.9069559	8.86081328	8.36651479

CDH19	3.89380754	6.22965718	2.66301467	2.9499874	3.54999009	4.38846821	3.87606091	4.56260022	4.03439543	3.15460833	4.44321283	4.10334138	5.71870009
PDCD2	5.03623391	5.11405423	4.62680198	5.40550036	5.60473023	5.57986222	5.47374574	5.07810565	5.49172974	5.75114799	5.17446318	5.16962685	4.3299103
SLC6A15	1.04391658	1.13624193	0.99679013	1.43495907	0.99419251	1.25580207	1.04811817	0.99750646	0.99664209	-0.51527455	2.50232847	-0.51527455	-0.51527455
RDH11	5.35347583	4.9731413	5.00073383	5.38100332	5.49173304	5.87547191	5.70738502	5.11289654	5.38266046	4.96807847	5.46639708	5.56548584	4.9843492
PRKACA	6.46242325	5.67795922	6.58123338	5.48503026	5.97012232	5.78822517	5.6700052	5.88030818	5.86560562	6.20339021	5.667637	6.13154939	6.47507366
ACTN1	8.6627677	8.17464817	8.68459797	7.7241729	9.17169471	8.27540177	8.35477723	8.82468247	8.0498785	8.79891152	8.44240781	8.57270463	8.36782484
ZFYVE26	6.18713028	5.57263526	5.97578473	5.81744787	5.60419049	5.16350663	5.93431288	5.59785505	5.50124307	5.53706317	5.77244547	5.3235013	6.05605435
RPS6KA6	4.45229734	4.57994432	4.31888454	4.34409889	5.56815797	6.04292268	5.77926407	5.81450637	5.11662023	4.76676903	5.38459658	5.17887894	3.94487795
EPN2	3.61828427	2.89247143	3.64936037	2.35388313	2.98183881	2.31786062	3.84344632	3.37254424	3.91390214	3.81727497	2.89974613	3.16931118	2.53020168
PTPN18	5.96165856	5.92900054	6.15320156	5.90688429	5.34139414	4.64127282	5.49788702	5.37327815	5.68646126	5.48254098	5.05673366	5.17160594	6.1201305
LIMS2	5.68132944	6.69897337	6.28174892	4.23556795	7.24574536	6.4498936	6.9738277	7.34279032	6.23071758	7.89127606	7.22746844	7.72853163	6.01437207
SPEG	7.08830911	7.02764483	7.39151428	5.08293128	7.16054983	6.75205597	7.31143748	7.74038009	6.42009201	8.00103005	7.63096267	7.94594984	6.60977333
LNX1	6.07699868	3.93168995	5.68738377	5.26536785	4.16395181	4.25723653	3.99335846	3.72831637	4.43225377	3.77078589	4.9700787	3.94224906	4.51222428
ALDH3A2	6.78405152	6.65298711	6.28389374	5.69124988	6.08431407	6.74316147	7.25668269	6.18937594	6.61451829	6.32746346	6.16193423	6.58429861	5.7725625
TFRC	5.57041685	6.16020097	5.8013546	6.57855986	4.52792614	4.67200628	6.06087765	5.96840937	5.15162751	4.97963301	5.50563702	5.73824892	5.02803816
SREBF1	4.20455814	2.07692306	3.40921003	2.62484124	2.80401256	0.75338916	1.20852772	2.26235447	3.12230426	-0.27361403	4.55282033	3.84320987	-0.27361403
AFF4	7.24926584	7.47242366	6.82002975	7.83206282	7.85149946	7.87095313	7.64913697	7.88717888	7.41956272	7.16272983	7.54007082	7.89647434	7.37064037
UBE2D1	4.02606347	3.95866399	3.33193974	4.66696841	3.91211425	5.02170225	4.42860531	4.39056593	3.82686977	2.99596871	4.20154919	4.32392401	3.84990873
MPP5	5.32766367	5.70161421	5.03689866	5.67421106	5.90362769	6.24918266	6.21398807	5.82939135	6.29747536	5.38724426	5.84058321	5.72398648	5.41977199
RHOBTB1	4.83620573	6.19157802	4.64213969	5.94394507	4.94721732	4.88864207	4.75158929	5.9259402	6.02717911	5.01991004	5.97116536	6.19563396	5.87580713
SMC1A	6.60213508	6.10299247	6.60608995	6.12738452	6.22994294	6.31559296	5.90858747	5.95902232	6.27130237	6.53777288	6.0627655	6.10925496	6.26331695
HSD17B10	5.53700225	5.49203375	5.8927859	5.07084829	4.85881727	5.29772678	4.54077277	5.05574292	5.01798898	5.49952987	4.94763766	5.56960445	5.3375989
MARK2	2.88211515	2.06135126	3.20533184	2.19957941	2.12774497	1.72962369	2.32874437	2.03816863	-0.10947379	3.72492528	3.9296635	3.61204291	2.92417268
HMMR	1.43533265	2.04539932	3.74853293	5.17170263	1.16833932	1.37820625	2.41658124	1.76469854	2.71390285	3.4097777	1.57880209	2.77960874	2.70827799
CHFR	4.97421954	4.52014997	5.32924409	4.88202763	4.7043053	3.9371922	4.94686541	4.93819966	3.65293178	4.93924056	4.89464822	4.27434883	4.20121954
TRHDE	1.15002828	1.58656144	1.41869221	1.27866324	1.91372285	3.54541093	2.34446874	3.91807757	2.19179494	3.65128788	2.59733131	2.79387856	3.76119142
P4HA2	6.1578883	5.43595617	5.86678488	6.50818337	5.14897043	5.69702013	5.52506513	5.58063294	5.40616233	5.94281162	5.6381275	5.84342448	5.93993598
FCGR2B	3.9376712	3.95771328	1.27309967	4.26679331	2.54063108	1.44824321	2.95040348	1.98754508	2.77975146	3.66456626	2.49495469	2.92310052	3.77619345
NFATC3	6.11116553	6.18295133	5.5429504	5.75912521	6.18010675	6.45811081	6.28848936	6.09994368	6.39065637	6.61452077	6.25860267	6.0676861	6.21042818
TRNT1	4.67132562	5.16750162	4.77322819	5.81745101	5.19975209	5.36672465	5.43384598	5.06227282	5.32101989	4.9875411	5.2606178	5.20899203	5.07420027
ACADVL	7.04097856	6.47698645	7.19517657	6.8483744	6.07638054	5.498024	6.40923909	6.38040919	5.84474848	6.38232032	6.18523254	5.93906237	7.03784426
STK10	5.2596481	4.92093559	5.32906379	5.34007374	4.22922792	3.55955553	4.19512189	3.8468249	3.71179337	4.59619531	5.02939692	4.08108613	5.11325874
FBXW11	6.50427846	6.92929608	6.0777652	6.43676948	6.69701801	6.79376948	6.77134731	6.7226159	6.32133202	6.53816752	6.67282211	6.7187512	6.68457492
ACAP1	3.50682254	2.17899849	2.73873973	2.35356346	1.49174289	1.08406895	1.84361952	1.71904508	3.46296884	3.2811918	3.95306144	-0.92953893	1.62453318
CRMP1	1.25615737	2.14151892	1.56665836	1.98412612	3.13179135	1.20793696	0.69886645	1.12091891	2.63442002	-0.69193949	2.54809072	-0.69193949	4.13235727
EVC	6.80310356	6.24547401	6.93889424	5.80780477	6.33069424	6.00655966	6.67635921	6.68557282	6.53932146	6.85850713	7.07990597	6.83581014	6.45912178
DERL2	4.41290638	4.83583501	4.02348632	4.98743817	5.10684043	5.37819525	4.6639493	4.86488774	4.88210039	3.46483296	4.586403	4.55643381	4.7307368
SIDT1	0.71561969	0.93257368	0.00459697	1.06645297	0.80415054	0.56655785	0.27553866	0.58552958	2.92251663	-0.24365155	2.56908908	-0.24365155	-0.24365155
NDE1	3.21328475	2.46247539	3.64053999	3.13123148	2.61613996	2.10811174	2.63483157	2.43595563	0	0	0	0	0
MRVI1	7.96463827	8.18762731	7.8963752	6.79992582	8.81715105	8.96172488	8.4082985	8.77535339	8.24967317	8.85693059	8.70186973	8.58841945	8.58229698
TMEM38A	3.05045434	2.68423744	2.20818785	0.97125631	2.83715187	4.90771851	3.06702211	3.89606985	2.94962112	5.03337216	2.22987744	3.32674444	2.92417868
AP1M1	4.67706386	4.05499258	5.18377416	3.94458725	4.29200637	4.22812468	4.09584882	4.15339696	4.44507724	4.68460953	4.16703062	4.93022179	3.92723573
PVR	4.30452188	3.86120177	4.44420133	3.43543898	4.56247341	2.91221421	3.43865964	3.4469714	3.58831301	4.64064046	4.00840539	4.52401857	-0.97058407
XRCC1	5.20868699	4.51916582	5.66022065	4.72377303	4.98438948	4.74595787	4.68237906	4.67781234	4.63143955	5.11642998	4.91233791	4.83687756	4.72894017
SCARB1	4.86781574	4.44745045	3.67721656	3.85818399	3.62257731	3.00010768	3.20084363	3.13749394	3.10628796	3.31277965	3.84206913	4.22165654	4.94950899
MCM2	3.51265771	2.95345794	4.56144188	5.05598939	2.64994029	3.02140717	3.39405846	2.75725143	3.55309391	3.33330079	3.40477267	3.83919005	3.84705769
MOV10L1	4.39975271	3.02640404	3.60127341	3.15663436	2.96008993	2.79201807	3.58027619	5.28690856	4.92558359	4.12803499	3.09436486	5.04369759	1.96686501

PANX2	3.03990531	0.53433734	2.53338732	1.578207	0.64713488	1.07043296	0.78497964	3.2040059	2.75752747	-0.59056358	2.72474602	3.21255979	-0.59056358
TP63	1.03877495	0.51907867	0.78839813	0.97145408	1.0812859	0.51105291	0.66810965	0.38648096	2.23439745	-0.18797355	-0.18797355	3.48551652	-0.18797355
ALPK1	4.49428661	3.83148635	3.14274398	3.68344869	2.91284478	2.63206394	3.10104009	2.20123654	2.9997598	3.69350948	3.41057569	2.64615174	3.03205946
LLGL2	3.25775919	2.97296356	2.51256341	1.26790197	2.43415882	1.76441514	2.90226566	2.47131088	2.44046357	3.93802363	3.33583006	-0.8935518	4.03128722
PDE8A	5.91805557	6.08168984	5.0235766	5.91350926	5.82935204	5.67139548	5.87351232	6.02827802	5.30288184	5.70554615	5.37828044	5.305729	5.7767031
CLCN4	4.86326908	3.48545431	4.1673677	3.58035108	4.42488193	4.93507622	4.17424804	4.96349962	4.54265911	5.41528111	4.13224545	4.9128768	5.32261792
NLE1	3.99681431	3.3324941	4.78055649	3.85021721	3.37005356	3.35690903	2.86022661	3.30930371	3.74210536	3.34111813	3.30606193	3.68069679	3.3540783
SDHA	6.58068449	6.00523259	6.192881	5.56679773	5.97550595	6.03969955	5.77451018	5.8942251	5.75778265	6.39354665	5.94850112	5.68683164	7.34069003
SMARCE1	5.66060319	5.82339865	5.62952161	6.29489494	6.2997818	6.56630306	6.22767291	6.22813878	6.364361	6.42664051	6.13347712	6.18459703	6.36844908
GSDMB	4.32268531	3.56166207	4.19178885	4.25193943	4.25362862	3.46751505	4.72480313	4.62212252	4.01128558	3.9072584	4.4756331	3.76883169	4.80864442
KDM5A	6.38392097	7.14333294	6.60533244	7.17666871	6.68367517	7.14798743	6.87702098	6.93561992	6.59161538	6.69262035	7.13464519	6.64815578	7.20646408
ADAM11	3.24529126	1.68654062	2.584424	1.66998106	2.35982151	2.56213612	2.10485043	2.90871129	3.32226451	3.80973392	2.53915115	2.74119117	4.3934859
PPP2R3A	5.36775697	5.48738937	5.30558397	5.70452451	6.03344758	6.21172788	5.8290182	6.33912311	5.83820918	5.44793471	5.304413	5.9555085	5.74272288
FERMT2	6.67071537	7.17011574	6.79776014	7.33679	8.07837539	8.23728385	7.83452651	8.19907613	7.43724756	7.66157252	7.03801089	7.90772467	7.21020212
DHRS9	2.65984156	2.62284687	1.92606425	1.96512095	0.48702095	0.41035034	0.35875431	1.93192869	2.5423297	3.29257163	3.69243267	2.31586844	4.75407482
CD5L	1.16995623	-0.01464166	0.12807648	-0.12388158	-0.12388158	-0.12304285	-0.03474105	-0.12195703	-0.02140094	-0.02140094	-0.02140094	-0.02140094	-0.02140094
PTGS2	4.80440941	4.47548846	3.31784323	7.4487798	3.51062906	3.26632845	2.7482917	3.4305241	4.6147804	1.93690548	4.14134688	3.61348918	4.47789212
IGF2BP2	2.1083881	3.24440173	2.91438927	3.01261231	2.2091388	1.70432374	2.46350904	3.53402197	2.57932798	1.25762937	3.94914371	0.11256389	4.16186628
MAP3K13	5.16448433	4.16717572	4.57231933	4.72475049	5.14752546	5.76263608	4.96641074	4.95013862	4.34812633	4.43482968	4.04512308	4.19160666	4.67062384
ST6GAL1	5.1707066	5.1707066	4.87761566	4.19734686	4.61819962	3.5558485	5.21475273	2.97270482	4.2088849	4.95996261	4.51698113	3.96254425	3.17295082
TBX21	1.05099476	1.90552523	0.3271243	0.5701464	0.73426536	0.71308024	1.3433866	0.60664313	-0.25701353	-0.25701353	2.94238663	-0.25701353	-0.25701353
FRY	6.47535671	7.01226118	5.7870286	5.69702054	7.57805362	7.14450135	6.69574622	7.55784129	6.92402622	6.54368261	7.21831288	7.42219744	7.56458267
PICALM	7.471667	8.1022943	6.92906267	8.42447567	8.46634871	8.22490309	8.00096615	8.26217775	7.88755356	6.91374262	8.14915397	7.77284273	7.33492447
NSF	3.62034418	3.28364737	3.54235322	4.88039859	3.73139285	4.21471527	3.96825123	3.39340244	4.1976134	4.0947872	2.41858908	3.84140067	3.81337545
GLI2	4.59930691	3.71024456	4.44315521	3.94306246	3.708767	3.88314897	3.78568458	3.72298624	3.21210814	5.25869906	4.32478687	3.98458521	3.95479314
CLASP1	6.95973557	6.9201398	6.52340757	6.63508607	7.06597076	7.11155126	7.21495553	7.06793375	6.84349587	7.13153623	7.23736301	6.92648166	7.36060682
MRPS34	5.16227549	4.66871762	5.60515316	4.6811077	4.25545165	4.59843523	3.91328221	3.91963579	4.69000825	4.95971599	4.52210933	4.27715493	4.65908777
NOTCH3	7.45330143	7.51791889	8.17127693	6.70992516	7.78164016	6.90957823	7.7850587	6.77843059	6.33836291	7.82519661	7.24782822	7.63581553	6.71984829
CLNS1A	5.67666196	6.23802876	6.07032947	6.38578556	6.45884117	6.27751732	6.56803764	6.6309724	6.68271923	6.30207942	5.5638884	5.78044422	6.38475851
PPP2R2C	1.79744317	1.65828979	2.04233144	1.56535385	3.07270249	2.71569527	2.04233144	1.55591886	3.99095446	2.54870182	3.63662301	2.50461345	3.29436023
TEAD2	7.15032092	5.17733381	6.70189604	6.00457998	5.8906553	5.42413434	6.25807228	6.05947812	4.99477107	5.99772059	5.63018099	5.66990613	5.6765711
EED	3.55047141	3.95533502	4.10672446	4.66374773	4.62849986	4.65905838	5.2138684	4.61871982	4.3631792	4.7506708	4.59901715	3.88556149	4.30070725
CDHR2	0.12780101	-0.04410859	0.05240522	-0.04410859	0.23369941	-0.04269392	-0.00911921	0.09317125	-0.01721307	-0.01721307	-0.01721307	-0.01721307	-0.01721307
TSG101	5.03829395	4.84731925	4.23954345	4.8061494	5.30675215	5.71192712	4.93154228	4.88354087	5.59075359	5.54520958	4.63554556	5.02653435	5.33301932
ATP2A3	6.04565582	4.98926428	6.09892078	4.14031454	5.47032105	4.51302593	5.41340338	5.90072593	5.42440157	5.51366816	5.87851023	5.24192581	5.1987756
CA12	7.27213375	5.36528315	6.37886382	5.37663227	3.87380614	6.02356709	6.21801445	2.88629658	3.69497242	3.22778783	2.28360788	2.41003111	5.13222521
MGLL	5.77183151	7.25718891	5.94838403	5.17522623	6.2818854	6.17923149	6.39854066	5.49376414	6.10593383	6.37791407	6.58751871	6.67210397	6.38340107
NTN4	6.87860948	6.12329285	6.3563916	5.35504707	6.26466689	6.87041909	6.27113586	7.06182658	6.8085033	6.81202524	6.47858836	6.87534216	7.23627513
BCS1L	4.81185822	4.40925473	5.30060615	4.48451136	3.81053896	4.11295869	4.31756789	3.99800467	3.36480311	5.19678811	4.82705912	4.53038944	4.06325972
NUAK1	6.12836157	7.5184212	6.37490385	5.78570115	6.9220584	6.49820997	5.76541032	6.27701183	6.9270773	6.32368333	7.59115516	6.45979074	7.0591356
DPP8	5.72838091	6.32379287	5.77827934	6.5170962	6.57110204	6.52848412	6.54330005	6.67509825	6.31717203	6.34952708	6.47797176	6.64131884	6.34995946
SLC24A1	3.49579142	3.23699264	3.51995259	3.14656055	3.16621697	2.99476461	2.38032629	2.60780125	-0.38428892	3.37730042	2.17115563	3.43642996	3.66742862
ZNF532	8.18494768	7.51189653	7.39499673	7.19965617	8.01169335	7.53262682	8.06995076	7.91459539	7.59679974	7.96708849	7.35431094	7.54057274	7.89264511
SCARF1	2.28156148	3.59778346	3.72975332	2.45546701	2.9685752	1.25007883	2.67201286	2.75658741	2.68524675	3.75389592	3.44178442	3.60304146	2.7936885
LMAN1	6.1637619	6.46024127	5.90829314	8.16122378	6.62415197	7.15146508	6.72957817	6.83119383	6.68628721	6.43544761	6.79466354	6.17376853	6.80070424
IPCEF1	2.82513479	3.80552252	3.06614891	3.38326287	3.34811399	3.22289143	3.07638903	2.57254218	3.65101941	3.60015142	3.95316738	2.79772654	3.55193709
ZZEF1	5.89228629	5.87296871	5.70051053	5.42806356	5.86276111	5.14857723	5.89455806	6.31218667	5.47015557	6.01472922	6.89124734	5.9732011	5.97413121

ENO1	7.88600793	7.71379819	8.28779135	8.26102224	7.4384773	6.83439849	7.65808621	7.18723456	7.2848577	7.61172972	7.39573744	7.60593642	6.96007087
SLC12A1	0.88143983	0.81794451	0.95183444	0.76736411	1.01021385	3.20627737	1.26064921	0.83567238	3.22791055	2.78651061	-0.51011785	2.93686839	2.5342476
ANO8	4.13381757	3.1796854	4.21746632	2.99133426	2.70093594	2.75067836	3.66562979	2.82821447	2.94911772	3.94124271	4.23547928	4.01220213	3.12199961
TUBE1	4.97650864	4.89385823	3.87225215	4.89482232	5.3257301	5.33309179	5.70690931	5.39830114	4.65980655	3.57283775	4.77684608	4.77582943	5.58269037
ARHGEF10L	6.05176549	6.08428821	6.42958004	4.64417104	6.68555704	5.87671692	6.77254603	7.05544711	5.78015897	7.39067796	6.56901188	7.03104611	6.02198764
TXK	2.17743447	2.13578829	2.28890744	2.48193591	2.24491023	2.00780288	2.39507056	2.03988851	2.54886662	-1.10016054	3.9760308	2.55691077	2.01765463
WSCD2	3.9394289	3.9013862	4.52030832	1.87637903	6.18347309	4.44571671	2.50105378	2.60386848	5.34747877	4.94720994	6.05308404	4.76995548	4.4084449
TACR2	1.57125545	2.08305559	2.0488501	1.08022552	1.43164228	1.73220576	1.00492761	1.42515449	2.78504036	2.9325943	-0.718659	2.44398503	2.47786926
ACTR6	3.79499751	4.21106657	3.43496714	4.53777023	5.20190836	5.3870207	5.40514314	4.86075645	5.47888685	2.35720428	5.35064554	4.88112267	3.01228003
TIPIN	2.13095366	2.6175491	2.6890131	3.62561669	3.02930329	3.6331569	2.86588238	2.96626056	3.18680516	2.91682378	3.4127925	3.58782724	2.82054349
SRI	5.14619645	5.78392985	5.08938054	5.93750063	5.94597416	6.00824453	5.76530406	5.987434	6.25690301	5.63786044	5.99579948	6.06478906	5.33989875
EIF4G3	6.17590703	5.9566736	6.33181092	7.08152504	6.2743135	6.34383918	6.26979215	6.3081809	6.24396484	6.17821864	6.29128208	6.0105079	6.8803293
NUP37	3.45198147	4.10082306	3.68450368	4.89183645	3.95196523	4.34373719	4.25060381	4.40334363	4.18986964	4.15557753	4.36974566	4.59722288	4.52416644
SEMA3A	1.79934351	2.9136083	1.75269093	2.95374122	3.13239343	1.85492013	2.13708873	2.1692041	3.69301968	1.32653222	4.21500563	2.26724774	4.24387145
GTSE1	0.99073708	0.59770095	3.62909263	3.59720815	0.05442434	-0.06981401	1.18711461	0.83534496	0.31653036	-0.63143775	-0.63143775	1.25497472	-0.63143775
SEMA3C	5.35854323	7.30244298	5.38839461	6.05758925	6.23661392	6.06683977	5.39837416	6.84208896	6.5439536	6.59113862	6.21839496	6.91123425	6.74651413
TTC38	4.96744287	3.92166558	4.90992225	4.1970724	4.49100006	4.16623898	4.64483245	4.44695883	4.42025819	4.10140454	4.50367999	4.28489691	4.9283314
ACAT1	5.20521242	6.14397031	5.07549171	5.31104949	5.74895767	6.53217416	6.14651871	6.57665856	5.98267522	7.04867071	5.604985	6.04629663	7.1263745
GRAMD4	4.65584574	4.2681119	5.0516084	4.19399363	3.90238764	3.54070629	4.15384032	3.98150878	4.08645276	4.16536657	3.62191525	4.26718765	4.51665464
CELSR1	3.26323703	2.78890503	4.34006629	2.97754725	2.49973604	1.67077464	1.94501578	2.05235061	1.027215	3.48030229	3.68185846	-1.354416	3.80192108
ZNF638	6.03721497	6.67192642	6.06623106	6.87378998	6.70643716	6.99767572	6.87378998	6.90155821	6.76197254	6.53816903	6.83364574	6.89051169	6.40891841
SLC25A40	3.51132304	3.5001995	4.27668006	4.57504533	4.71508884	4.81579417	5.0725417	5.18639393	4.82819782	3.38366093	5.18570608	5.26599846	4.13554077
ADD2	0.64680163	2.80621049	0.49745716	2.00575075	1.15832755	0.70629701	0.89938225	0.91140356	2.15203023	-0.50079298	4.11409389	3.02154158	4.19449878
RASAL2	3.43506921	4.41850436	3.35444998	4.01783644	4.91529088	4.34885209	3.91747678	4.13065963	4.1636873	4.60736774	4.10930922	3.63621511	3.27661567
ZNF37A	5.41387754	5.49106092	5.2026463	5.62052934	5.96262676	6.17325016	5.77222524	5.97512345	5.80633131	5.21580278	5.57715862	5.96400159	4.99986239
MARK3	4.59792645	4.98831735	3.99252958	4.97580615	4.8303571	4.23874215	4.98712648	4.97459569	4.64989786	4.54129833	4.18495943	5.14927022	3.60623574
SLC25A3	6.77092829	7.08442318	6.19452617	7.17410109	6.65288661	6.73864889	6.81769694	6.92696951	6.61310881	7.35042303	6.97927055	7.34886872	7.12966232
FNDC3B	6.44869192	5.93545542	5.68467969	6.76890825	6.3266164	6.14793656	5.90537761	6.35446641	5.86255873	6.04193306	5.7837093	5.91289357	6.2584563
FOSL2	8.2528049	7.43513248	7.9742199	7.62482864	7.909994	6.91434244	7.57350703	7.28857845	6.59426145	6.99511396	6.95042384	7.58281415	7.0314311
CACNG4	-0.08345037	0.89936587	-0.08123988	-0.05274862	0.14487375	-0.08222137	-0.08345037	-0.0806303	-0.06831888	-0.06831888	-0.06831888	-0.06831888	-0.06831888
FRYL	6.01369408	6.91151848	5.97603641	6.71211231	7.21979978	7.32872706	7.11088057	7.31009877	6.83124756	6.7682418	7.25529589	7.13363127	7.01314438
TMEM131	6.1304397	5.75852894	5.60213945	6.32542459	6.41982597	6.53958314	6.13922908	6.50239888	6.02741886	6.1800587	6.09644184	6.16975427	6.14911699
FSCN1	3.98264431	4.91369697	5.04856289	5.92405072	3.98081072	2.84199196	3.95764139	4.07977264	4.59019295	5.38300513	5.10857313	5.20217528	3.81342162
ACTB	10.1368871	10.7767457	11.117601	9.72951956	10.7767457	10.7767457	10.4632203	10.7767457	10.0847367	11.1611973	10.5265041	10.9310987	10.212195
MOCOS	4.2387524	2.84427239	4.64041452	3.9340665	2.49125478	3.29734215	3.44593668	2.86776402	3.90590605	1.60810349	3.68239688	3.77806454	1.99002865
PLD1	5.28691996	6.18834257	4.93157019	5.82310458	5.92054337	6.28478736	5.88028038	5.95199261	6.29680859	5.89341335	6.55299232	6.05641989	5.82999977
ATP12A	0.75143788	1.57161478	0.68906978	0.69318882	0.98607762	1.40655781	0.68649975	0.88333423	-0.23361598	3.58330642	-0.23361598	4.4394107	2.26980818
WDR62	1.49366807	0.92014729	4.6477481	2.72873739	0.83794162	0.97666874	1.77778392	1.02651718	1.41280483	2.74667772	-0.75071318	2.75327714	2.27727288
DLG1	6.1612029	6.68541579	6.13084293	6.80047843	6.88115555	7.11640746	6.84776908	6.927443	6.86090743	6.59069361	6.56298182	6.53082346	6.24261723
RAB7A	5.51213901	4.6022968	4.61257527	4.72409365	5.24329155	5.27985947	4.99660631	4.69272828	5.03126898	5.6961004	4.8574085	4.46266727	5.10586729
BCAP29	5.96503754	6.35734867	5.56209592	6.66336307	7.48771215	7.36288185	6.78523075	7.57031615	7.77922321	6.11157463	7.79493614	6.9187571	6.20680797
SEC31B	4.6301751	4.31549734	4.36587546	3.37603895	4.24078539	3.26548329	4.65760786	4.44470384	3.47758652	3.15687976	4.28898853	4.57953343	4.9669528
SART3	5.80342617	5.49411121	5.80463913	5.17679283	5.62855807	5.42400827	5.52703904	5.39683229	5.37146151	5.62551856	5.59145322	5.84485296	5.43740559
ARHGAP15	3.11729528	4.32755468	2.49603128	5.41714643	3.6452977	3.26759067	3.54398791	3.23916187	4.67406534	3.80091894	3.58760676	2.8205091	3.63674285
EXOSC7	4.02516633	4.57948983	4.75731002	4.22517081	3.53334732	3.71657499	4.62700247	4.02846434	4.36147088	4.74247477	4.73326991	4.39414137	4.69779161
KIFAP3	4.28986666	5.32035319	4.60601393	5.68343941	5.45860403	5.38912583	5.49415047	5.55399244	5.25587942	4.97517475	5.84103937	5.72127665	5.62850253
MKRN2	5.55181994	5.42281804	5.14347289	4.78867184	5.20259123	5.70166039	5.14394746	5.1308364	5.37044414	5.91006391	5.27412493	5.46205244	5.33008548

MCM6	5.2148378	5.13901358	5.13443803	6.10333521	4.93452262	5.67345136	4.97627281	4.60480387	5.17118646	5.00352167	5.53387948	4.96495702	5.9108378
REXO2	5.2700182	5.82894537	5.13918268	6.61402766	6.50426682	6.21860897	6.33497258	6.30455051	5.9374288	5.94046529	5.40192986	5.6887727	5.96476058
RBM7	5.38495067	4.9966222	4.86944703	6.22353483	6.01382319	6.18851799	6.21077352	6.11556656	5.54907951	4.66758418	5.65263752	5.44454666	4.02321343
RBMS2	6.76484358	6.93861321	6.23372997	6.29523159	7.02046494	6.5585764	6.91381342	7.03852564	6.56510805	6.7680875	6.97297154	6.829308	7.0470864
BAZ2A	6.99098244	6.62845027	6.51704858	6.0917877	6.51052213	5.82002608	6.55624696	6.58119852	5.65224042	6.58380654	6.54556208	6.15447003	6.64391445
PTPN23	5.39110099	5.10206371	5.60768856	4.83541126	4.91085575	4.48420575	5.07756473	4.98301231	5.00234218	5.11857095	5.46398492	5.10264337	5.39866442
MLH1	4.59356171	4.8685803	5.07990296	5.56503473	5.38585995	5.19826834	5.09969482	4.95640326	5.30423386	4.8777871	5.30660753	5.36956145	5.12049223
UNG	4.64346255	3.7486171	4.23067067	4.03011114	3.88991186	3.85212341	4.35850705	3.10580329	4.3674812	4.38193702	4.6911184	4.27879489	3.97974207
FMO4	4.52233017	3.99693729	3.54957527	4.00834155	5.26254922	5.1953134	4.09658975	5.45582754	4.66200713	4.68952564	4.18688618	4.67770475	5.04560103
KLHL20	4.63785586	4.64423065	4.14119757	4.44062365	4.70648303	5.33759834	3.93906736	3.99292255	4.5239562	5.24823802	3.61664198	4.77514564	4.96781334
RGS11	4.13638156	4.00377369	4.45941361	1.46676137	3.95324561	2.04249733	3.69583769	3.64071196	4.63873142	4.2125215	4.34796247	3.84059807	4.06412941
SLC46A1	5.62609267	5.26021749	5.43661453	4.88652503	4.68875942	4.51608202	4.9625357	4.38827324	4.66169002	5.96789886	5.24777329	5.31753008	4.70274067
PLXNA2	6.02686341	6.22151032	6.15241406	5.11302872	5.59675013	5.4728024	6.26551128	5.89249044	5.6466435	6.24757243	6.09029866	5.89374694	6.19206628
SPAG5	2.70169612	2.4421079	4.90570441	4.8047808	2.28321131	2.47135909	2.79273318	1.9167014	3.29870893	3.34958443	3.05260091	2.06605346	3.26497394
ANKRD13A	6.02595674	5.93142908	5.67997868	6.21064705	6.43208818	6.59977228	6.41729656	6.5990181	6.27131404	6.79613289	6.05893379	6.55531868	6.7695727
TPD52	3.02353364	3.83302504	3.03483637	3.73589797	4.45651203	5.16271713	4.61034153	4.17104034	5.01713598	4.1365998	5.23950048	5.07831297	2.70053956
ACACB	6.13732501	6.4953194	5.35708205	3.97169431	6.04966254	5.62127254	6.14101684	6.4259725	6.00725862	5.83444149	6.70014412	6.20336411	6.9213683
TRAF4	4.47820778	3.61111152	4.35712017	4.76165517	2.59150178	2.66130228	2.80711195	3.06349871	2.4800727	1.58856112	3.31999364	3.36218676	3.03668016
PAG1	4.94584003	5.31227692	4.24307109	5.76522993	4.69354538	4.14507077	4.69003431	3.96621179	4.96027991	3.90625738	5.48880821	4.08943552	4.40332173
GPATCH1	5.13221372	4.44793328	4.6104829	4.08061089	4.65526742	4.92856178	4.95455409	4.50266233	4.7628192	4.72213323	4.04958873	4.56435135	4.90089663
ICAM3	4.75281215	3.61002239	3.78154779	3.93524711	3.47928127	3.65027399	3.06688601	3.06238108	3.3284755	4.30121864	4.3531763	3.53360498	4.4051625
NT5C2	6.75304594	6.53670087	6.0948326	6.81001354	7.46847745	7.03779245	7.42366664	7.21812888	6.97450267	7.22245955	6.96498567	7.03089742	7.31876302
MCAM	7.66371358	8.5027718	8.22062672	6.92015501	9.40535829	9.35631298	9.04129088	9.11725007	8.4446503	9.21441985	8.77678029	9.08202227	9.33438539
GPC4	6.77779595	6.45698259	6.49939656	5.64624977	6.61626196	6.89376623	6.35478873	6.44604956	6.40030208	6.43419046	6.74701795	6.38621825	6.58721671
MBNL3	4.08072512	5.04828624	3.89192417	5.23737922	4.10566115	3.7092207	3.90987879	3.80365826	4.32017526	3.27804788	4.52160461	4.04322095	5.84003462
CAMSAP3	0.22945902	0.40553	0.23206095	0.37810731	0.22945902	0.23090566	0.22945902	0.23277847	-0.05271402	-0.05271402	3.78540863	-0.05271402	-0.05271402
RAP1GAP	1.12824398	1.34165749	1.232046	0.56867647	0.6826658	0.48873589	0.58046709	1.13235339	2.36568821	-0.32412774	2.77865466	-0.32412774	2.69055603
XAB2	6.12538495	5.45308988	6.11156953	4.46228965	5.2809815	5.10287811	5.12066155	5.10946814	5.15618236	6.24638677	4.82654468	5.74086618	5.14274678
ARHGEF1	5.16799682	5.26448091	5.75588882	5.25380868	4.86340572	4.12654109	5.04542779	5.2079613	4.92653571	5.22323881	5.14278742	4.58978441	4.95153643
STXBP2	4.32564146	2.94991824	5.12532098	3.60044428	2.21284151	1.16266665	2.6168872	1.80907101	3.6088788	3.38949596	2.81624805	2.55120943	3.77248699
MAP2K7	5.15049183	4.90820256	5.02108638	3.4532827	4.59165933	4.38187906	4.54867139	4.55150689	4.04447226	4.93884399	4.84746428	5.07635291	4.15957974
DGKD	5.56805691	5.19331833	6.17896277	5.30075954	6.00391917	4.79029147	5.63683962	6.6929845	4.7489134	5.13166457	5.66485991	5.64538466	5.89085217
CTTNBP2	4.13650734	4.03374074	2.93892298	3.37452138	4.03931822	4.81596837	4.35481632	4.37837268	4.56644779	4.31084513	3.55627983	3.87071394	4.56053792
RARB	1.53572724	3.68315594	1.94509764	2.50507839	3.19031339	5.40568349	1.97367033	3.01915414	3.39457335	-1.06497617	3.10407216	3.18339665	5.12163766
TOP2B	6.03404399	6.7872618	6.64482023	7.25250905	7.08811985	7.61110452	7.09282212	7.28105476	7.39408245	7.01872779	6.5478697	7.2241548	6.86677235
TM9SF3	6.40667281	7.19813266	6.50563333	7.79169422	7.77112612	7.72556766	7.82724046	7.73625036	7.73751256	6.66156514	7.86952024	7.8871915	6.43497132
NFKB2	5.82309311	5.27572196	5.92938046	5.63805849	4.60366895	4.25848152	4.93681363	4.92468766	5.02995298	4.89396539	4.77669324	4.64132847	5.31566735
UBE2T	1.97147856	-0.03738841	3.31729145	3.33125049	0.08676788	1.43148345	1.54138474	0.26836816	2.95935643	1.85837394	2.56890503	-0.69474803	1.2670288
PPP1R12B	3.13814286	4.07508129	2.85770275	1.99197432	4.87515987	4.54629566	4.55545414	4.7707434	3.80080513	4.08349503	3.8619633	3.33969348	4.10742059
DNAJC10	5.87320868	6.487606	6.19350048	7.12838819	6.85111517	7.24992958	6.73276758	6.8491235	7.32435499	6.44735175	7.07731476	6.99508174	6.24082504
GTF3C1	6.47506524	6.09747379	6.60394456	6.24545108	6.06037557	5.78625191	6.51072976	5.98551155	5.93972199	6.44660873	6.3481127	6.24353342	7.02398707
IL4R	6.45206067	6.15059471	6.43599699	6.46520685	5.8961676	4.20676083	5.72602947	5.2625285	5.07977069	5.48964824	5.2232106	5.20002945	5.4608181
USP33	5.61063715	6.41844896	5.83407681	6.57208072	6.55022352	6.63707294	6.95557205	6.81659954	6.62068161	6.09130629	6.45165657	6.30672811	6.10385086
PAK3	4.54722029	4.78545079	5.1518924	4.34078529	5.94060017	5.53449675	4.89865285	5.92395374	5.78774325	5.91596568	5.6503011	5.70796879	5.56414513
CAPN6	1.6518429	2.35742187	2.10735449	1.53591013	3.41973605	4.9012843	2.63246516	1.97368704	3.74495305	5.17347037	2.29589543	5.44966662	3.58625478
SNRPA	5.49277754	4.87555911	5.73807193	5.08026585	4.92397435	5.1881361	4.80578217	4.16443791	4.47556787	5.98678127	5.07218892	4.86835762	4.0637788
EXOSC5	3.70340428	2.30946946	4.21289224	2.2770193	2.96532768	2.89984603	3.20100642	1.05872223	-0.02803249	4.35023056	-0.95377237	3.39537863	1.16081629

DYNC1I2	6.35502496	6.75720516	6.49084165	6.82978033	6.86942904	7.13940137	7.05701226	6.73250236	6.94352922	7.14151023	6.59601679	6.88550214	6.95015037
APBB1IP	4.22307486	4.11085867	3.76055264	4.93784835	3.2117184	2.42991518	2.9447748	2.36386606	3.80705034	4.55146134	2.61578992	1.49926764	3.15192643
LRCH4	4.33119365	4.00540042	4.56804353	3.79952292	3.60695836	3.67356592	3.53404997	3.84120186	3.9548094	4.01723936	3.28004708	4.28347957	3.62561087
FAM76B	4.08510077	4.45487303	4.031636	4.92777936	4.90066484	4.89408508	4.95042969	5.32359821	4.96375323	4.4446992	4.51877965	4.9967488	4.01265791
SIRT6	4.0415471	3.43406839	4.72833008	3.89320204	3.35680931	2.98064906	2.90784435	3.43604519	3.11597968	3.53474303	3.9113519	3.43461902	3.29067706
POLD3	5.00811101	4.77638665	4.47435681	5.67382156	4.8217871	5.08982244	4.63014538	4.84429428	4.70634947	4.52166591	4.92278391	4.1414542	4.53252364
ACTN2	4.30583855	5.66388388	4.91686961	3.29889041	4.56838549	6.46218651	5.54800591	6.53005638	5.60117495	5.8787458	5.36369912	6.24877524	8.54596527
CAPZB	7.34616412	7.21641753	7.69411668	7.83457419	7.36028094	6.75361117	7.225029	7.17845984	6.94979511	7.89064847	7.11547704	7.14544423	7.33399672
GPR137B	5.65008108	5.22148262	5.56643222	5.63275703	5.05787532	4.57183601	4.30145596	4.73651756	4.98192973	5.11378727	5.0729544	5.12623819	5.28659172
NAALAD2	0.6321539	0.86200566	0.61584693	0.95387473	1.60241458	0.87244667	1.22787556	2.01221673	0.71123275	-0.47857626	3.5208524	1.14897058	3.06342747
SLC25A43	5.0977516	4.69998619	4.58193015	5.30551759	5.0695532	4.31739426	5.35274727	5.04493195	5.00715142	4.32749774	4.84572855	4.6068626	5.40631756
UBE2A	4.89635511	5.28016891	5.48225827	6.24559769	6.01276034	5.87854966	6.0096851	5.75759866	6.01705858	5.30770455	5.61530005	5.84351132	6.23240968
FGFR1	6.78024346	7.17526198	7.18893689	6.32299916	7.35761715	6.91136284	7.0089292	7.63357838	6.76315004	7.89965845	7.35845181	7.53793499	6.94290695
FBLN1	6.76627978	7.05284305	5.95987987	5.95148559	7.50985192	6.52119258	8.45831711	8.20678597	7.30541326	8.26691478	7.45330618	7.82235356	8.26691478
ITGA8	8.98767174	9.6238452	8.76540858	8.31807206	10.2064819	10.4368326	10.0032008	9.97295829	9.77721202	9.59074591	9.51918696	9.77721202	9.44206137
CST7	3.76115093	2.90869093	0.80673478	2.35316515	2.34220869	1.61081696	1.74914922	0.16370775	3.14184913	3.81319316	2.74792008	0.31846686	3.90099486
MAP2	5.24532844	5.18789796	4.81123238	4.70499533	6.53919684	6.85163708	5.77864035	6.55919767	5.93711914	6.20332451	5.97208417	6.12173405	6.27614423
PIAS2	5.28765272	5.35924269	4.75081812	5.22015633	5.59192963	5.7631703	5.67529869	5.86090846	5.32519847	5.11737926	5.95768364	5.17691783	5.48661099
AMPH	4.46962392	2.1928783	3.72483735	3.50174643	4.76320951	5.03792249	5.44459922	5.25997678	4.26065917	5.11210951	4.4461917	5.92598261	4.55616364
ARAF	6.02186031	5.70198812	5.88269175	5.07253069	5.62963349	5.13607132	5.29402876	5.43370059	5.26732151	5.74366485	5.53391335	5.64353291	5.02560577
MCCC1	5.39095346	4.52747018	4.43765574	4.58618472	5.08373008	5.41550961	5.00375164	5.80053393	5.01618376	4.87813778	5.55080997	5.11303781	5.32300909
LAMP3	0.83616966	0.54602003	0.66198603	1.47721803	0.75690627	0.54744974	0.54602003	0.77286616	3.56988342	-0.52452591	-0.52452591	2.29554549	2.87637586
FAP	5.45411613	6.18150179	6.0100827	8.52533045	5.38604086	5.35323436	6.24546189	5.92302681	7.36488619	5.77952553	6.69032818	6.2699629	5.67514643
NEBL	1.28909981	3.92170925	1.27727019	2.18887641	2.03348315	1.73244186	1.80765974	2.21794685	2.57771769	3.48528282	4.42743692	2.88332363	8.70680309
ACER3	3.89620874	4.10821331	3.74959872	5.06438973	4.79228724	4.80991165	4.7778128	4.82101142	4.94544628	4.12264824	4.74919179	4.58539696	4.5538902
UBE2K	3.55853955	4.49265672	3.92675644	4.8627385	4.87670418	4.93579236	4.92363626	5.03579151	5.1906259	5.00044428	4.4929806	4.38928605	4.70509922
PIK3C3	5.50179774	5.81892494	5.11529041	6.04605563	6.03299143	6.20284137	5.79836173	6.16959969	5.86182844	5.84703012	6.05248694	5.85787439	5.85667154
N4BP2	4.7047018	4.97960512	4.91439392	5.58886606	4.79536913	5.72816946	5.03660084	5.47059153	5.61926963	4.97802794	4.95528107	5.37008082	4.67322358
TULP3	5.93595124	4.83514823	5.49463201	5.35691221	5.69770223	5.31545337	5.53769982	5.8636472	5.53019642	5.43160132	5.34081099	5.40763905	5.89679192
SYNJ2	2.55248168	2.39718399	2.80685956	3.07373522	2.52742741	1.72624293	2.5672254	2.16947486	-0.0464902	4.44190683	3.74514382	3.05818422	0.98808769
ADCY2	5.24998999	3.85167637	4.07066558	3.60030175	5.28777976	4.86805438	4.42131019	5.08092656	4.66611648	5.25508135	4.72564425	4.50819299	5.0846583
PPP2R5C	4.69547235	4.94191975	4.27164231	4.88632967	5.08921218	5.33738042	5.4467143	5.44961644	5.58926525	5.11793986	5.03818344	5.51747808	4.92859712
RBFOX1	0	0.78632938	0.00124472	0.24049229	0.00951906	0.00069205	0	0.08715891	0	0	0	0	0
GNB1	8.00938994	7.70958919	8.01840009	7.73086601	7.8913337	7.31889419	7.97544467	7.87572542	7.69152143	8.02839801	7.81580663	8.02003206	7.96310869
EDN1	1.87887793	4.30504562	1.61991218	3.16975909	2.91713824	3.87995356	2.35853911	3.89578906	3.60893242	3.5267093	4.21130396	2.39794633	4.84715385
MLLT10	3.70126351	3.80325843	3.51425246	4.14878149	4.36989402	4.26267733	4.67868401	4.39726963	3.75682404	4.26183077	4.60761181	4.07665785	5.39111387
ZCWPW1	3.9191515	2.87790685	4.14761096	2.9969656	2.81647093	3.48998824	3.56513056	3.12978902	3.73540594	4.31475787	3.24630313	3.02260279	2.91253463
ADCYAP1R1	1.31968923	1.85294322	1.43369547	2.48964786	2.34142617	1.91820687	1.47381031	1.79958801	1.97221094	3.5144691	3.7406339	1.83805498	3.60817445
P2RY10	2.09578516	2.55719396	1.25785421	1.77480166	1.55269586	0.68019752	0.95065309	1.16103401	3.47887269	2.42555623	2.11993414	2.11993414	4.13585557
ITM2A	3.13734297	5.25731928	3.3149213	3.74342162	4.80109371	5.10323831	4.83794772	5.95430257	5.40298735	5.73156566	5.83877327	5.2312613	5.89631564
VDAC3	5.35404146	6.20938922	5.68856255	6.46364945	6.27042422	6.63047571	6.33121827	6.3397066	6.50119555	6.87512737	6.14030951	6.55419339	6.69342486
PCM1	6.96266012	7.66685984	7.12107469	7.79146319	7.74631565	7.90762974	7.85084606	7.79402345	7.88929505	7.64849941	7.08784148	7.61437064	7.58105785
TNRC6C	6.66832266	6.22977653	6.16869184	5.18685608	6.31387984	6.09144943	6.02309737	6.22470823	6.22310558	6.41527982	6.57574009	6.02275687	5.92497238
CBFA2T2	5.92975259	5.41212356	5.61709782	5.01001652	5.3503959	5.07743797	5.48163969	5.20657202	5.24484468	5.79651997	5.08960035	5.66791331	5.24880123
ITCH	4.22073841	4.86742787	4.12002732	5.24117881	5.18309243	5.10766397	5.14405389	5.43725749	5.15740616	5.32918232	5.14910706	5.21715115	5.17407248
PKD2L2	0.02609775	0.02609775	0.02865148	0.02609775	0.16767313	0.39467291	0.02609775	0.48622155	-0.05575288	-0.05575288	3.47942552	-0.05575288	-0.05575288
TP53INP2	5.97891968	4.98434584	4.93628111	5.25488804	4.75721377	5.62056572	5.17601585	4.73696118	4.06012551	4.74546294	5.15349723	5.24229105	5.85167694

SDF4	6.56905339	6.3138181	7.24968917	6.20408106	5.65996898	6.18911948	5.82156964	6.13389827	6.85506113	7.0856347	6.61472953	6.35783331	6.64103603
MYH7B	1.66149995	1.29813345	1.75294467	1.10624165	1.22822603	1.85720794	1.83838904	1.53981834	-0.76848102	2.9354607	-0.76848102	2.27796713	3.34113052
TP73	-0.030986	-0.030986	0.53017404	0.16673222	0.004569	-0.02971945	-0.00202483	-0.02807976	-0.01292902	-0.01292902	-0.01292902	-0.01292902	-0.01292902
TOLLIP	5.4254532	5.63544809	5.90322562	4.69148007	5.3734485	5.14770585	5.63075544	5.16230313	5.07787305	5.37519661	5.61878479	5.96483113	5.22859503
UBE2D4	5.23009568	5.6116339	5.25093813	4.12990806	5.31930037	5.24079986	5.16273974	5.08943089	5.16178177	5.65040182	5.21912819	5.35205592	5.72143891
CLUL1	-0.01822682	0.60829109	0.12179197	0.00166335	-0.01822682	0.32620306	-0.01822682	-0.01506661	-0.03619217	-0.03619217	-0.03619217	-0.03619217	-0.03619217
RUNX1T1	5.42354753	5.69698468	4.64662899	5.41579739	5.82055588	5.76803033	5.94124832	5.76343085	5.89226869	6.04172735	5.75392845	5.7977685	6.22863752
CDH17	0.65074057	0.7342562	0.51491456	0.51232427	0.85952386	0.98142803	0.51232427	0.51562888	-0.34132529	-0.34132529	-0.34132529	2.60569986	2.68863172
THOC1	4.37665499	5.09284093	4.61050533	5.80331174	5.35993793	5.4143669	5.59282619	5.60127204	5.27545428	4.45513254	5.16581635	4.82273073	4.58372739
FKBP7	5.08117501	4.73629777	4.7424826	5.90009442	5.33650253	6.26849651	5.0393441	5.40633699	5.7111023	5.54706943	5.21379254	5.0030432	4.91246342
OSBPL6	3.83072311	3.15697043	3.79001841	4.0301922	3.47305538	4.86662859	3.82153137	4.05828624	2.66639741	3.83290064	2.30492231	3.75492278	4.72966774
SLC1A3	5.77771648	5.06509673	4.40320053	4.27039691	4.48046079	5.34856312	5.0784301	4.01309124	4.19723086	2.01361774	3.08519854	3.86734354	5.15793032
XRCC5	6.77801005	7.12393964	6.89885746	7.66901463	7.26163255	7.6397183	7.26477117	7.14917591	7.17634544	7.25460771	7.01138684	7.06980553	7.58231133
LXN	2.64141305	3.93762739	2.81000084	6.19671087	2.73842533	3.30539233	2.69620313	2.21868743	3.13955173	2.10683597	2.70282501	-1.55067585	4.64712478
SP140	3.31118513	3.57697953	2.45715192	3.36905167	2.64949402	1.92131884	1.88601178	1.75125096	2.83054803	2.86697621	3.18311096	1.97001198	3.87199563
MKNK1	5.52189633	5.33412036	5.6447975	5.57909502	5.15307621	5.08196929	5.84368068	5.71107252	5.33387469	5.66101365	5.33447032	5.43371608	5.6010686
TNS1	10.1566809	10.2660528	10.4305792	8.5303659	10.3298386	10.3000658	10.4777798	10.3880756	9.4118598	10.2013094	10.2013094	10.1014128	9.63657451
REXO1	3.66310419	2.58361802	4.40220019	1.92912888	2.71377893	2.30944438	2.60961487	2.90268066	3.50489087	3.94950162	3.47057846	2.82990072	-0.69396038
SAR1A	6.66978359	7.36015484	6.45617283	7.67635065	7.27579303	7.24561263	7.2249463	7.13982468	7.11810001	6.62482852	7.08767959	7.04901365	7.10802443
CDC14A	1.46938834	1.66201406	1.60231741	1.85302654	2.34201074	2.71889509	2.26363941	2.34757777	3.69315322	-0.56828274	-0.56828274	3.93622714	2.58347647
RAPGEF3	6.10224052	5.20603754	5.67769712	4.0798145	4.98456243	4.42610891	4.62546517	4.43218492	4.57090373	3.97794022	5.44036044	3.91880127	5.16900692
CEACAM1	1.85032806	0.6352569	1.19210726	1.30008665	1.55399023	2.03321844	1.21434411	0.67460994	1.8319453	-0.55379007	1.32280522	-0.55379007	4.10491576
SENP1	4.5059959	5.15790822	4.9837483	5.26302509	5.44234954	5.22509014	4.92028808	4.94581346	4.74725418	5.249213	5.32977203	4.82828	4.64296691
CIC	6.32497321	5.69236192	6.7014731	5.16079982	5.62520242	4.93561441	5.43335086	5.3445085	5.1902449	5.91885445	5.86074851	5.9071148	4.37956112
LIPE	1.54256387	1.24329977	1.62827493	1.6226365	1.74008779	1.9808251	1.68106448	1.61895063	0.39993131	2.48831903	-0.63817542	3.07939286	4.26682825
FDFT1	5.99985947	5.57706283	5.59549917	5.54885976	5.53792315	6.32795558	6.2497193	6.10402682	5.70476213	6.04412821	5.6936133	5.91927289	5.81876726
PAFAH1B3	2.57688908	2.93634541	2.08106602	2.95028478	0.93655217	0.43464473	1.50037828	0.14624337	-0.73097913	-0.73097913	-0.73097913	2.82410631	2.72007431
OPHN1	4.32087033	4.56114312	3.91365571	4.06142032	4.61730777	4.85985077	4.51046048	5.1344183	4.47097418	4.50116264	4.8198774	4.65183841	4.58782112
KIF22	4.39288647	4.3596155	5.20412065	4.72031769	3.52179743	3.76526816	3.63166473	3.60894979	4.09924485	5.01719026	4.79934297	3.83566106	3.85276743
SCGN	0.18401462	0.71522399	0.12588417	0.12341392	1.86634821	1.30279406	0.71869403	0.12656539	0.18823275	0.18823275	0.18823275	0.18823275	4.13400969
PGM1	6.64433509	6.14204469	6.47089002	5.80838013	6.39577048	6.95554234	5.89926508	6.51266722	6.16155318	6.70833454	6.19899403	6.50621316	6.47670988
DDX1	5.56509733	6.61740137	6.14987184	6.87811561	6.6360123	6.93070288	6.64400486	6.71205674	6.99658919	6.84683479	6.85698071	6.49131574	6.68781172
DNM2	4.71731658	4.79145656	4.86292808	4.57991796	4.26270595	3.33961805	4.55598917	4.28344134	3.29206644	4.70064065	4.03858752	4.95514512	4.5112887
EPB41L2	6.92903512	7.69339131	6.41802904	7.77819508	6.90734533	7.22830001	7.08939662	7.19938135	7.70948386	8.0429033	7.18247481	7.2867834	7.56373539
RIMS1	5.5260647	4.51552294	5.22977142	4.58538495	5.68178788	5.68317705	4.50428023	4.14729784	6.54491253	3.93349664	5.80549848	5.45946911	4.84659186
MOXD1	0.68005499	3.48161014	0.91208272	4.11733831	0.83184591	1.52579886	0.97306726	1.37194654	3.2109778	3.05513421	3.96734696	1.80080905	4.16075283
STX7	6.15071505	6.57918673	5.50869628	6.39662775	6.61843008	6.78716289	6.75409888	6.82205009	7.06246879	6.40115447	7.138263	6.94432208	6.23224557
RABL2B	5.18486457	4.14346059	4.7382677	4.29295929	4.87232538	3.97044417	4.76889773	4.46599182	4.42905083	5.3330739	4.75227741	4.82390082	4.69559489
KEAP1	5.63001459	5.43919949	5.62484136	4.73225676	4.53570023	4.90965212	5.2101932	4.88016674	4.82501557	5.011512	5.2527025	5.47046479	5.15686168
DDX43	2.65158812	3.66229674	2.75370506	2.74192421	3.96393424	5.0971368	2.89516667	3.07102253	3.0659502	3.17882364	4.02852378	3.40696938	2.68692951
PTPRH	0.25324267	0.82905915	0.25909847	0.36749898	0.21686419	0.08955276	0.08812136	0.24590346	-0.13285801	-0.13285801	3.81480173	3.6406527	-0.13285801
DCT	1.16404157	1.31747788	1.24753094	0.52875996	1.21577197	2.13294911	1.49534066	1.02020546	-0.65421757	2.78854021	-0.65421757	3.33011298	-0.65421757
SLC35C2	5.25901904	5.13007399	5.33040242	4.86577368	4.75756968	4.28065472	4.59566852	5.01434989	5.08673952	5.35763115	4.75141735	5.29092986	4.77116842
EPHA6	2.46431493	2.47366412	2.21289596	1.64427135	3.52837208	3.53350949	2.85325491	2.03622097	4.02677216	4.25270996	-1.20983252	3.17671503	2.51702769
RFX3	4.27967775	4.69548198	3.61729175	4.75962593	4.8384137	4.98743693	4.96190245	4.87888023	4.3508899	3.65791937	4.85733517	4.9904724	3.28094294
RIF1	5.9946212	6.58423814	5.99213729	7.07402598	6.96285206	6.95454419	6.84437665	7.00919124	7.15081567	5.92879983	6.46627223	6.70256789	6.10810547
RAB21	5.93229578	6.09381112	5.66354092	5.70321706	6.66897263	7.01361153	6.53540082	6.56851739	6.2715242	5.38278384	5.9423802	6.27724485	5.52378345

SLC4A4	0.73622807	3.61260662	0.49044557	1.33575348	1.35346666	1.57545229	1.14489923	0.74898405	0	0	0	0	0
SMARCA2	6.96023786	6.92521633	6.75153977	6.87093357	7.10137204	7.28111428	7.33156999	7.57761325	7.24858577	7.73100714	7.13296797	7.32759857	7.47027695
SESN1	5.18370655	6.06546981	4.73763729	5.57518749	5.72223407	6.03246417	5.92165023	5.74999905	5.41056755	5.61862876	5.38951184	5.3952472	5.51925236
MID2	5.8887413	5.73381029	5.25106045	5.04729772	6.09184265	5.98628006	5.66302906	5.35298925	5.84828034	5.39906394	5.60981318	5.63160441	5.30337404
COL5A3	3.0396153	2.4288617	4.36047668	6.483761	3.30630267	2.87695862	2.75983085	3.19773591	-0.07166602	4.31637818	4.33279332	3.11342848	4.05038406
SRCAP	4.54496321	3.64754866	4.956956	3.96122787	4.72408862	4.26689546	4.84820692	4.89644094	3.73798726	3.76191973	4.62108685	4.62060247	4.82226427
CHRNA3	1.08104728	0.81273472	0.97486673	0.90989473	0.80649261	0.64785858	1.0432748	1.1232601	2.49686146	-0.53658533	-0.53658533	2.84098154	-0.53658533
KCNN2	0.22792944	0.65705408	0.17528535	0.35291284	0.41053189	0.17414137	0.21633466	0.17599585	0.39267349	-0.10147408	-0.10147408	-0.10147408	-0.10147408
CNOT4	6.15401764	5.80144192	5.50668816	5.84674088	6.27524918	6.4878959	6.05965056	6.10164499	6.22955668	5.65938752	5.845361	5.76685733	6.18666961
PSEN1	5.96855778	6.25237866	5.7766589	6.48618065	6.25781893	6.07949158	6.28959704	6.48786815	5.85750446	6.24435321	6.32528216	6.14227998	6.37679885
CPOX	4.57870619	3.73091841	4.6863489	4.67424552	4.28075773	4.41326726	5.11289798	4.92553635	3.95443656	4.18952119	4.97991649	4.54045745	4.41615189
CLDND1	6.07769093	6.5927032	6.01034636	6.77023571	6.95529779	7.0316653	7.01337325	6.90743597	6.48330318	6.52346463	6.6969074	6.7402805	6.08142662
MOK	4.42082784	4.58121745	4.37303477	3.93774917	4.08887274	2.98569902	3.77764309	3.40363345	3.43608233	3.66865339	3.65082811	3.08891827	2.7234914
HSP90AA1	7.7356164	8.32675659	8.23703981	9.03245962	8.92181243	8.75708002	8.78402943	8.39564519	9.00403252	8.42500112	8.17445789	8.60191018	8.52332385
RBL1	2.85777482	2.713456	3.21135227	4.09668913	3.25446636	3.60973415	4.03299991	3.55406044	3.42255093	1.57696723	2.99782721	3.09051349	3.20563027
DLGAP4	7.07707048	6.20271011	7.16982365	6.24886454	7.17427507	6.32999063	6.64386523	7.04544042	6.54721268	7.24056288	6.82485746	6.79469041	6.7863708
IGSF9B	5.53504046	5.50497177	5.63625818	3.4279839	6.04667691	5.24615093	5.85537522	6.04206903	4.98682675	5.9972164	5.52126352	6.09017391	5.49400328
NDC80	0.39558787	0.80437605	3.60852313	4.5132287	0.66620466	1.15593466	1.54764176	1.10143019	3.65885028	-0.67570832	2.891497	-0.67570832	-0.67570832
AP4E1	2.98288755	3.565581	3.47905843	4.4567043	4.02394978	3.94672245	4.2092461	4.12480044	3.87333362	2.98865297	3.16001	4.06785577	3.73261777
RSBN1	5.23110857	5.41110915	5.35108202	5.50841292	6.02834677	6.33684616	6.04665714	6.08823958	5.39316085	5.01161578	6.26897002	6.12242413	4.9714507
MAGI3	2.68354049	2.44452303	2.8688247	2.33494493	2.98561583	3.53326303	3.91806284	3.77140338	2.31469125	3.52810864	2.94282502	4.15431391	-0.69990394
CXCL2	3.73166382	4.23509883	3.57054675	5.05020257	2.03348466	1.21589977	2.63666937	2.79738881	3.48649915	2.2169292	3.81369433	3.63437415	4.4340274
COL4A4	5.90611727	4.9118966	5.44796222	4.65093089	5.16152945	5.33244485	5.87935775	6.38975701	5.58277922	4.9919899	5.72804153	5.45021858	5.51165578
TCF7	3.13931678	1.82080291	2.6586755	3.08956488	1.57336302	1.20290405	1.36230666	1.23486457	3.24311059	2.77198384	2.62519052	1.13566383	0.85123874
OSTM1	4.31579928	5.16815369	5.08461963	5.29280239	5.80075732	6.13343712	5.87145546	5.83826297	6.1139647	4.5526984	5.85124893	5.41663705	4.59661253
IMPG2	1.8015374	2.01638421	2.00534337	2.93639936	2.36385945	3.07661398	2.47870812	2.62325708	2.90087261	3.56687972	-0.16744193	3.97386181	4.07213762
PCNP	2.99992377	3.50861526	2.74303298	4.28326778	4.78224637	4.85020352	4.59923489	4.78315065	5.07610631	5.08788282	3.32669326	5.16736702	3.54392034
EXD2	4.94720167	5.00587062	4.65531418	4.69636536	5.102528	5.50803425	5.30704576	5.04634991	5.01603899	5.18744133	5.20593064	4.94804037	5.13444119
ARG2	1.86226704	0.89216616	0.74098184	1.2269065	2.79844658	2.60571573	3.08813289	3.82144602	-0.77624201	-0.77624201	-0.77624201	0.88142406	-0.77624201
MEF2C	6.94840469	7.53775232	6.33004804	7.42719736	7.5401013	8.13959915	7.33743	7.56328969	7.80382252	7.81658645	7.68156055	7.59175745	7.87192521
PTPRC	3.74215999	4.22333319	3.45830825	5.34646066	2.89002476	2.28732603	2.85248932	2.39981116	3.98902248	3.76219996	1.67473883	2.87574005	3.90056329
PKP1	0.65359855	1.1587023	0.66810941	0.89304598	1.3899787	0.5808234	0.73303893	0.66866518	1.75707584	2.66816407	3.56575777	-0.36497625	3.22573696
UBA5	4.91672336	5.45885313	5.10632412	6.09222576	5.79211085	5.93986942	5.87340503	5.88626704	5.54274687	5.11416554	5.53421685	5.99531094	4.90267931
STK17B	5.52499338	6.34588241	4.97035227	6.93007059	5.82647437	5.68391663	6.52859991	6.46144662	6.31008804	4.53738038	6.41323705	6.05183691	5.00686782
CDC14B	5.57565854	5.60063301	5.05734401	5.25909819	6.39580302	5.90493857	6.50884272	6.19762418	5.83873894	5.90509098	6.20760603	6.12837065	6.49476979
ZNF510	5.18499678	5.7113161	4.85395583	5.44064163	5.65094289	6.33862131	5.73630803	5.56309491	5.92660627	5.12376207	5.8746816	5.85981216	5.27964161
LRP2	0.57279007	5.32585997	0.52795268	2.13630074	0.52606741	0.52711559	0.54601985	0.78263315	0.22267279	-0.71616665	4.28724293	-0.71616665	3.51353118
ZNF506	5.40783742	4.8647781	5.07435917	5.04416741	5.74506969	5.27043714	5.78580253	5.90522637	5.55369656	4.72487709	5.8251097	5.29720817	3.8582943
JMJD4	4.34131998	4.19747957	4.88559836	3.65332726	3.54213657	3.45025222	3.29552711	3.94485051	3.28735263	3.57903263	2.45873536	4.16586386	2.84302534
DUSP12	4.20980272	3.96429912	4.07928883	3.65365913	4.36258174	4.80965991	3.97039792	4.2754845	4.34698372	4.66492178	4.21157733	3.24548952	3.65019463
AACS	4.18065001	4.02111618	4.58385455	4.31140351	3.97975392	3.37694204	4.14672867	3.99505567	3.99667274	3.56920727	4.04730206	4.55539212	3.80161666
KIAA0141	6.29623222	6.13341927	5.9379481	5.88629573	5.97119583	5.42871258	6.0639794	6.10007262	5.86804923	5.71194214	5.88995844	5.98828915	5.85469329
CADPS2	4.46718013	5.91881547	4.08292472	5.47594969	5.42822698	5.0289975	5.22214148	5.09094893	5.36083321	4.59565388	5.68830547	5.2762949	5.49694369
PCDHB4	4.88940721	3.87751807	4.70638199	3.65312957	4.53759624	5.43718599	4.32989078	5.25582939	4.90513532	4.1093489	4.83547677	5.53445118	4.43863692
PCDHA6	0.23207819	0.05370573	0.17759014	0.14239485	0.11518967	0.49258808	0.224544	0.15021599	0	0	0	0	0
PCDHGA2	3.65437158	2.47522823	2.87934267	1.9958884	2.211008	2.97222821	2.36190736	2.75497587	0	0	0	0	0
HSPB11	3.26294906	4.08507182	4.18167582	4.8795063	4.79136092	5.08926539	5.20276404	5.07908079	4.92261685	4.54912243	4.19575718	4.1013592	3.77939589

PHLPP1	4.68379313	4.63478618	4.39190639	3.57259995	4.61540274	4.77544	4.65516621	5.07654884	4.07350777	4.7942561	4.5052534	4.60333894	4.25255246
ATP8B1	6.51862703	6.7714656	6.2565799	6.22092513	7.60888471	7.12472168	6.89087683	7.29882203	6.64727953	7.07003115	6.95232678	6.98057729	7.30419026
IL12RB2	1.95019444	1.91869655	2.10692288	1.32265235	1.88942806	1.34694483	2.23136341	1.61147703	3.33436041	2.99149268	1.96558932	3.7640535	3.27460375
SMARCD3	6.16401361	5.56046791	5.67767908	4.9598576	6.10300726	5.79420342	6.13860426	5.70586476	5.31920538	5.99880816	6.30006486	6.06621589	5.61617304
WDR70	4.56314667	4.76982367	4.31740157	5.04942831	4.41202218	5.02643433	4.95142396	4.99835736	5.01548454	5.03545557	4.78389054	4.29428919	4.82606927
MPP4	0.11446054	0.92453198	0.85917726	1.06761529	0.84852078	1.3101042	1.19523754	1.96206763	-0.43289224	2.66639821	-0.43289224	2.87805948	-0.43289224
STRADB	4.18799738	4.40466831	3.82986784	5.00854641	5.00265992	4.5760306	5.02258129	5.25358492	4.78650586	4.40309982	4.76310436	4.37536119	5.46216675
BZW1	5.61666077	6.46589568	6.40673599	7.91864947	6.08620778	6.03838578	6.00303417	5.95452404	6.39652803	5.16141517	6.22572642	5.95997059	5.02105584
PGR	5.4370571	6.13515341	4.99059028	5.04414215	6.81554082	7.1908931	6.7364058	6.93301383	6.66191439	6.64463409	6.68381579	7.65134544	6.28042149
C1QTNF3	3.39872247	2.60108869	2.59399386	4.15637898	3.17990083	3.99897486	3.45810181	3.93143266	3.53443515	3.45596087	4.27753131	4.48869716	3.64327945
ME2	5.54772298	5.50584357	5.92441648	6.77052026	5.61985701	5.62051976	5.75279922	5.45685058	5.45886177	5.31154851	5.26564924	5.53316805	6.13800248
C5orf22	4.20148834	4.85411092	4.24038034	5.29702405	5.41069933	5.40985786	5.14825745	5.47862931	5.38629174	3.91864457	5.11609295	4.88550875	4.60397154
CCNT2	5.764512	6.03344207	5.16553627	6.40227928	6.34873231	6.42382316	6.19278125	6.37292781	6.16442591	5.26944359	6.47944332	6.0300177	5.66301414
FAM135A	4.5632127	5.3741008	4.71964467	5.61617716	5.7691055	5.74868696	5.66847658	5.80701444	5.34467578	5.49073756	5.86871186	5.57896419	4.72855752
COL19A1	1.87395593	1.82473063	1.63127377	1.23107511	1.4764877	1.6043139	1.63481212	1.79238008	2.35356414	2.55339885	-0.82680361	0.18926904	3.13639814
EPB41L3	4.82464283	5.34610777	4.92148404	6.06450315	4.1970229	3.47401829	4.06330518	4.23624005	4.80306	3.67618103	4.25342915	4.09205718	5.64716075
COBLL1	5.14082227	5.7764137	4.70786064	4.92668057	6.26197934	6.23719868	5.73641892	6.20356841	6.33593033	5.29873383	5.82175508	6.30712102	5.69374242
DLG3	4.20063144	3.60049592	3.75819504	2.18058357	3.51033845	4.07192199	3.48821484	4.04186424	4.47675473	5.16815947	3.23493815	4.22971536	3.52846637
KCNK2	1.37202513	1.32688226	1.22097244	1.50483473	2.27524125	1.33505193	1.6809656	1.32943252	3.71868819	2.05197034	2.61511777	1.77912747	3.88017571
SERTAD4	4.38423929	5.85458938	4.16226102	4.23162626	4.89587119	6.5393889	5.95748241	4.75468348	6.20889454	6.11925434	6.30071371	6.4058365	5.6369295
TRAF5	5.4298975	5.96574737	5.62169709	5.40965807	6.67541866	6.35756851	6.33291531	7.04392409	6.23160375	6.5422012	6.56085143	6.48390751	6.47145959
MRPL22	4.80454983	4.16161301	3.42959861	4.47200607	4.55735289	4.87940075	4.54833773	5.03327205	4.29734846	4.6287904	4.24647399	4.85936903	5.00973221
GEMIN5	5.48832804	5.40874393	5.76008781	5.25508064	4.98966092	5.82170873	5.5239565	5.68605467	5.12959932	4.96883416	5.19589284	5.15898753	6.01406821
OPRK1	1.49966539	1.1707836	1.17316909	1.1707836	1.46453038	1.99677197	1.66143197	3.78536184	-0.0206578	4.0634596	3.03630892	4.51215908	1.98416645
NFE2L1	8.13634025	8.06969619	8.21802634	7.43789284	7.62946384	7.91742521	8.18409563	7.61432175	7.85048101	8.24532212	8.05661525	8.04006415	8.32868649
SEMA5B	0.92718929	1.40351835	1.46526762	2.21854197	1.97970305	0.66221446	1.93045594	1.93105338	3.17362272	3.27317902	4.23130076	3.68780687	2.92123272
GSK3B	5.77585024	6.1186237	5.64404868	6.08723584	6.16843007	6.53232771	6.04511373	6.151341	6.11101577	5.75746092	6.0941552	6.03402414	6.29540488
ITGB5	7.82256321	8.1453932	8.57245807	7.26372662	7.73892361	7.82975734	8.31977524	8.04609493	8.37800002	8.34778509	8.47075194	8.02091029	8.07771352
ERC1	5.69707087	5.66912416	5.29155645	4.93717048	5.52875317	5.73317616	5.4827227	5.23831056	5.67613758	6.08557399	4.74509175	5.41973251	5.59053763
XPO1	6.06994908	6.87421458	6.54639721	7.76788482	7.03107893	7.31787577	7.3851316	6.78661177	6.95749752	6.8074773	6.53956482	6.70379802	6.41627701
RNF13	5.60690096	6.16608572	5.2970853	6.37474562	6.26215937	6.63937965	6.22820191	6.25005273	6.21127432	6.3258298	5.32026431	6.03191695	6.01225445
TRPM3	2.66106911	3.56473765	2.3207354	2.35558609	2.21308158	3.91173197	3.44539433	4.01560371	3.74109782	4.00619526	3.53776086	4.44504853	3.28997285
PALB2	4.35384432	4.11718974	4.18949206	4.65174859	5.09507327	4.70045849	4.48305772	4.74173374	4.63502689	4.67736285	4.50516235	4.35191921	4.24981123
DOPEY1	5.56190673	6.16821432	5.42054042	6.31158232	6.26588412	6.42882232	6.19481621	6.44081935	6.17799533	6.27737351	6.45976969	6.79990878	6.30022452
LYRM2	4.55165938	5.03735047	4.77567397	5.56022117	5.19018241	5.39695849	5.10565654	4.83707564	5.18532563	4.2548485	5.15199433	4.54714101	4.73751351
BCKDHB	4.74971923	4.74849679	4.20752426	4.42979898	5.25723765	5.9533365	5.38305609	5.41497497	5.42173246	5.57090677	5.32361615	4.99928162	5.37682093
KAT6A	6.86597969	6.69493677	6.18158372	6.27586687	6.78200653	7.02213196	6.79245211	6.75292701	6.41309944	6.7183267	6.43898194	6.48423437	7.13244788
ZCCHC6	5.39206002	6.04290892	5.69242062	6.79701347	6.09293958	6.45668955	6.08998667	6.00078245	6.03845696	5.63377719	5.51523255	5.24442901	6.20355308
ULK2	5.59434776	5.66733333	4.7902404	4.78538209	5.57218633	5.39429723	5.52998371	5.37013756	5.41882974	5.16408681	5.4441366	5.68845104	5.54804129
GRHL2	0.46281778	0	0.30481363	0.05492931	0.01902843	0.00069205	0.27790818	0.00158797	0	0	0	0	0
TNPO1	5.04566953	5.61082377	4.83024958	6.09182098	5.72550543	5.76868641	5.92364485	5.78038924	5.36217791	5.61812898	5.66354103	5.90639472	5.02546281
PLOD1	6.42940326	5.89580695	6.65213456	7.30457676	6.07950963	5.66610828	5.77016205	5.32998107	6.02932879	6.50094306	6.58549962	5.57775573	5.57756838
P2RX5	0.8528686	1.32182161	0.33714641	1.06651525	0.33459683	0.49579473	0.8595721	0.3378495	1.9694011	-0.39643807	-0.39643807	2.00239596	2.66228647
ITGAE	3.25195943	3.15551865	3.35711972	4.35028188	3.23398258	2.78644458	3.61779911	3.74205659	3.20669373	3.05128913	2.32378106	2.29462948	3.11802441
DIS3	5.78631717	6.22431125	5.87805252	6.65287797	6.63822369	6.85323356	6.37005404	6.81985967	6.39873391	5.88957495	6.0556631	6.23200123	6.15375664
PIBF1	4.10956568	4.68531048	4.36534573	5.05929615	5.16716891	5.57566265	5.03777494	5.29564988	5.80581579	5.21661397	4.74962127	5.21526822	4.77518258
TDRD3	4.9158971	5.94423475	4.93354582	5.77200009	5.66544099	6.17275422	5.5716768	6.25974582	6.12410067	6.57157966	5.5974768	5.7261788	6.00014543

NUFIP1	3.61156641	3.93513021	3.73576073	4.54283975	4.95134469	5.12147676	4.32530008	4.62278825	4.15290086	2.72085902	4.42726748	4.32502033	3.71109529
PDS5B	5.51617088	6.28704739	5.92359719	6.76783897	6.76861726	7.03690386	6.90167909	6.9275513	6.93557467	6.29990885	6.48951188	6.79683561	5.88786744
OXCT1	5.0537798	5.25661041	5.59806211	6.03038246	5.91007344	7.01754888	6.5186689	6.82791348	6.82509	6.89102485	6.66384715	6.61053457	7.50820995
RRAGB	4.85927519	5.45253843	4.61626721	4.94923474	5.24257893	5.43777093	5.67193247	5.20820993	5.06342272	5.34421568	4.85771656	4.63826254	4.13170543
CYLD	5.95592026	6.64061501	5.95122037	6.70637964	6.76964954	6.74862501	6.78260879	6.97923802	6.72044608	6.36840558	6.82662616	6.77415125	6.33202796
SLC27A5	2.22989987	1.09644196	2.90142024	1.06414908	2.20328318	1.80955543	2.28649866	2.21646972	2.42197112	3.9403221	3.07369055	0.44724133	0.81325882
ZNF324	4.5306962	4.32439854	4.69459248	3.17977571	4.14401798	4.01996677	4.02635137	4.08473513	4.15487783	3.74705789	4.7550806	4.87338365	4.23778047
ZNF671	3.6666964	2.81975583	3.40461489	3.10122145	2.914592	2.18931114	2.76539154	2.01265408	2.1998304	-1.33114214	3.5866063	2.86756262	3.70001363
ZNF416	3.95914631	3.05500007	3.4998147	3.30875651	3.94276937	4.16560896	3.22190616	3.96686923	3.93070266	4.36575205	4.11537605	4.08587895	3.45534261
ZNF586	4.47056495	4.74351825	4.0230144	4.88047591	4.80151568	3.64772188	4.81664349	4.39497796	4.35855668	3.96122461	4.50263455	5.17242343	4.22235154
ZNF446	3.55100474	2.8780769	3.77639515	2.00066834	3.07199217	2.84779794	2.45594752	3.42726059	2.60555747	3.5550122	2.91011358	3.79248473	2.99977779
ZNF264	6.2672202	6.0716379	6.03972451	5.69881214	6.5364139	6.10169798	6.27789361	6.2922036	6.12981027	5.99331606	6.73106283	6.04561511	5.89304986
RPS5	7.24285724	7.6202098	6.89576218	7.39355426	6.64353826	6.59945259	6.40607545	6.55086704	6.91102386	7.78055228	6.97694233	7.05862802	6.5479915
FAT1	7.18877359	6.95116698	6.88806443	7.57069644	7.87807283	8.40396853	7.57069644	8.13838211	7.72575669	8.31814851	8.05569823	8.07722893	8.0904932
YTHDC1	6.4093153	6.6571851	6.16258223	6.45984477	6.88197549	7.34796723	6.56289203	6.71911944	6.86709325	7.04240447	6.66647313	6.67194026	6.66779168
CHMP2B	4.46439668	5.60741884	4.78292107	5.67129264	6.09081048	6.33170206	5.64741247	6.06109347	6.11487521	4.94262086	5.89334223	6.29998941	4.10360353
SMAP2	5.63353565	5.81958975	5.01448047	5.95169549	5.20097436	4.62817703	4.72358343	3.97679481	5.0669562	5.06483302	3.94360121	4.42577557	5.23978713
PPIE	5.50577695	5.17358788	5.28040408	4.68270751	4.67094031	5.5139837	5.05566443	5.31067516	5.55477949	5.94202412	4.80287848	5.06820943	5.25038524
ZMPSTE24	5.24929314	6.01273882	5.78280884	6.75034584	6.83322129	7.09454198	7.21387788	6.93013784	7.06562507	6.87825205	6.9561916	7.14230963	6.60441072
STARD7	5.9258262	6.70251939	5.90743424	6.68999051	6.03392887	5.94183024	6.4153553	5.8821346	5.72536386	6.31244542	6.34533834	6.07204789	6.83390153
NOA1	4.96317693	4.6812591	5.14286637	4.98348771	4.85074297	5.04683484	4.8520771	5.55653084	4.75553543	4.95293756	4.04461201	5.03681165	4.87779852
REST	6.12110689	6.04282391	5.81385948	6.54812073	6.47427403	6.98663529	6.36413788	6.58135247	6.51843862	6.16370244	6.36863833	6.38909154	6.25314481
HAL	2.57908997	2.12965912	1.69758822	1.19873454	1.5985583	1.0892868	1.25946508	1.52037364	2.43729324	2.56904097	-0.82152623	1.58256388	3.13746263
SSH1	6.42428461	6.4958806	6.28734949	6.88353478	5.93301529	5.85172289	6.08100393	5.86195188	5.80087994	5.85387682	6.54021379	6.122846	6.31640275
GSTP1	6.32727019	7.25128493	6.66465149	6.21385358	6.17834337	7.44831542	5.94307502	6.17834337	7.04663199	7.67705017	6.74942126	7.37190807	6.27909909
APLP2	5.99786008	5.18896361	5.63401902	5.53520094	5.86022122	6.19046855	6.06193987	6.34506535	5.67961821	6.10773835	5.28830014	6.07104641	5.52105119
WBP11	6.46011806	5.82964192	6.32717865	6.31544621	6.24068284	6.21278544	5.96089685	5.95861533	5.97456702	6.22984003	6.07664354	6.02454981	6.21453241
EIF3I	6.53885274	6.3078818	6.77835637	6.42439333	5.97043744	5.98711369	5.81299768	5.81916663	5.84083987	6.15185951	5.74381776	6.17266011	5.9080387
COL16A1	5.34677474	5.2201657	6.6281345	6.13110417	5.36182547	5.13235846	4.84937735	4.4974983	5.98670836	5.93694413	6.52951787	5.5588306	4.98271046
TXLNA	6.92413027	6.46058131	7.04114807	6.83141097	6.31140725	6.12253932	6.47448124	6.06938879	6.24189384	6.53396218	6.39113121	6.48228243	6.29518262
APOB	5.42274524	5.61999201	4.45472876	4.00714678	5.59859812	7.61036121	5.6802835	5.77415944	6.53091126	6.51049732	6.13575444	6.57747083	6.54720534
NCOA1	4.64614966	4.43425007	3.65302012	3.79591912	4.57270456	4.64614966	4.04650834	4.62703189	4.69294617	2.9894922	4.70314895	3.80210339	3.55651501
AGBL5	5.52615937	4.95149947	5.22440319	4.05320633	4.46083824	4.38541542	4.37112108	4.04091206	4.69426126	5.19392521	4.42336623	5.10785406	3.70861358
EFR3B	0.36775817	0.31757623	0.88972042	0.74397439	1.0588704	0.87687049	0.72884924	0.81043388	0	0	0	0	0
KIF3C	5.15300638	4.6802019	5.09861147	3.88220177	4.79099487	4.65416616	4.43108296	4.85661022	4.93228121	5.45596524	5.24336988	4.80823402	4.85497119
RAB10	4.9704243	5.32859351	5.27825358	6.09302337	5.80480488	6.04166112	6.1191758	5.77828974	6.12865811	5.58027969	5.14187704	5.1808422	6.01897181
GCKR	4.07248979	0.08525756	3.05080452	2.36438265	0.76333243	-0.04124709	0.75644357	1.04658782	-0.85979967	-0.85979967	-0.85979967	-0.85979967	-0.85979967
HADHA	7.35656866	7.41658392	7.25099629	7.48228752	6.93369892	7.30149506	7.0556362	6.92899137	7.19878763	7.62575486	7.26038339	7.35316457	7.62447695
MAPRE3	5.37734323	4.12165936	4.61624587	3.91271747	4.62052285	4.30339385	4.46658938	4.09150927	3.67383849	4.6360039	4.44022876	4.35159259	5.19997624
CAD	5.23246851	5.1181701	5.51779043	5.4888028	4.81350166	4.43043632	4.75842785	4.70641317	4.76192764	4.88251609	5.69815895	5.12551857	4.83629513
CD59	7.93589512	7.97863226	7.80930045	9.40983247	7.64759325	7.7843326	8.89975659	7.84323236	7.86959314	7.53518189	8.20503041	7.92721958	7.73126631
CD82	6.48815053	4.82500151	6.42975728	5.86280378	3.52985727	4.96280568	3.93342413	2.73475567	3.48068518	3.63973644	4.59670254	3.92142722	3.31559317
BCORL1	5.2786846	4.60871586	5.35992829	4.20811501	5.09626641	4.95280571	5.08009479	4.99404426	4.48628029	5.15493427	5.06475548	5.15398446	4.92143258
ATRX	6.49695675	6.99397958	6.7877145	7.50369324	7.6327087	7.91027306	7.61426465	7.6698453	7.98118563	7.50862982	6.87414719	7.62157056	6.90304688
FCN1	2.19137539	3.54333005	3.2479426	4.69805118	1.18745468	0.84178856	1.71666734	0.73193123	3.95815812	2.19287108	1.99036464	2.64940383	4.97981419
MYNN	4.72491675	5.00133056	4.88291219	5.71251121	5.61353565	5.8601833	5.59224927	5.72284688	5.42310155	5.15756156	5.52283797	5.18586672	4.82347718
MECOM	6.35460115	5.56165788	5.99931066	5.91942378	5.9474415	7.17096201	6.28874711	6.79434967	6.85109692	7.10424508	6.30809781	6.76936694	6.68986679

SCAMP1	5.43444635	5.73917015	5.03403292	6.23334004	6.19483105	6.41608578	6.27507995	6.30126272	6.24339743	5.56648303	6.18066806	6.3792855	5.55650413
PREP	4.05448287	3.90843483	3.96554075	4.79464709	4.00431681	4.05983359	3.81191191	3.67271227	4.43209545	3.38955815	3.45744774	4.31689324	4.24906561
HACE1	4.46509662	5.12446841	4.54137568	4.95296053	5.15440992	5.24544424	5.36414337	5.42984502	4.91449531	4.95321316	5.41842779	5.31028107	5.11323601
SEH1L	5.18789334	5.47073658	5.10388529	6.05060916	5.15268452	4.93947767	5.0967454	5.35986432	5.06688256	5.07544571	4.95862764	4.95862764	4.8727952
WDR47	4.57711378	5.05771092	4.49316842	5.08137072	5.61800999	5.50650963	5.3403459	5.19305084	5.19051654	4.51377209	5.17236623	5.36750816	5.44375072
WDFY1	5.99721217	6.12958871	5.9756862	6.62278941	6.66086948	6.49740243	6.60461382	6.90168984	6.47086647	6.32225908	6.44952494	6.49183873	6.24010638
OVGP1	2.44843356	1.25012584	2.70100302	1.48579065	2.48122166	2.4121641	2.20827085	2.42505259	-0.63272569	-0.63272569	1.02159151	2.70556777	3.62658434
SLC25A24	5.24922805	6.12894606	5.58601404	6.58407232	6.1913436	6.03606965	5.97942234	6.13918675	6.07218776	5.60485238	6.16165473	5.89235399	5.64432577
MAP3K4	6.42449945	6.24549178	5.72081431	6.27476613	6.17421923	6.24375588	6.46488783	6.40759869	6.15622334	6.46986101	5.84099937	6.13447135	6.24434155
PILRA	4.86254221	3.82189565	4.31075799	4.53745299	2.82733457	2.39942004	3.10091232	2.17669735	4.43914077	3.29281659	3.24566769	3.0454009	5.23076539
IGSF9	-0.2201972	2.25463009	-0.3755467	0.3680242	-0.51250828	-0.51158625	-0.51250828	-0.38383638	-0.3246338	-0.3246338	2.14739538	-0.3246338	-0.3246338
ABCB1	1.09112708	1.82620828	1.23006942	1.53059505	2.32821704	2.43577718	3.08298557	2.19437169	2.86321389	3.03495843	2.77529142	3.38577984	3.88401906
ZNF213	4.51321004	3.09119771	4.83231231	3.17258456	3.80388739	3.61647886	3.10605012	3.52478175	3.10181757	4.66991447	3.27924331	4.05826592	3.74018
AKR1B1	5.56329799	5.11756752	4.57859868	4.95362788	4.45813932	4.34801112	4.55718251	4.65408538	4.25440813	4.66199621	4.43134635	5.31692966	5.21633862
CPNE3	6.74260594	7.06740398	6.48295321	6.92196148	7.09327777	7.45877341	7.25784723	7.00006537	7.03325527	6.81196782	7.35582133	7.10009867	7.18101994
RRN3	5.37110047	5.90212419	5.33206354	5.59878454	5.32100884	5.83329015	5.88641758	5.30290084	5.49997667	5.41589739	6.00876976	5.51022943	5.42484132
CTTN	7.00159021	7.02159994	7.03155503	6.29078991	7.00637576	6.37836958	7.58925206	7.3222259	7.21455692	7.3311122	7.2047931	7.18810525	7.19635274
WNT11	1.11669855	2.22426208	0.58475879	0.36673824	2.29847958	1.42798093	1.58270315	1.39932041	3.58267692	-0.43219989	3.65055112	-0.43219989	3.25740161
MTIF2	4.7641013	4.80751187	4.96409892	5.11521408	4.85860143	5.53527259	4.8953229	4.99897427	5.36662604	5.21462997	4.87910623	4.34913922	4.89322733
DDHD2	5.72281857	6.31402457	5.6652557	6.15709194	6.702901	6.35748099	6.29830302	6.66945759	6.26966831	6.29066341	6.46448142	6.78686377	6.49965481
TTC39A	2.98240379	1.60537138	2.05864983	1.20461177	1.42753209	1.60463796	1.44559466	1.82430879	1.55399173	2.52853254	-0.93587805	-0.93587805	4.07911862
EPS15	5.88836085	6.64087903	6.07098923	6.6603615	6.88312184	6.85311471	6.86107537	6.97093639	6.75403123	6.60097527	6.5338073	6.99955443	6.96812386
ORC1	1.13454183	0.53927695	2.13347751	2.92985911	0.62617495	0.66383316	0.85940549	0.54797912	2.56228713	-0.53079218	-0.53079218	1.67081884	-0.53079218
MGST2	5.15850726	4.90391989	4.4203662	4.45067825	5.08137486	5.41166348	4.80403734	4.19010348	4.93357186	4.74826489	4.88951915	3.99474731	5.1021573
CHERP	5.80587773	4.9272663	5.83732581	4.54939982	5.1065407	4.88537217	4.74504987	4.81850095	4.93311866	4.91388546	5.13008049	5.31558974	4.48938314
ATG16L1	4.94713247	4.74974383	4.95370181	4.36207558	4.48729549	4.47106571	5.04406058	4.69163931	4.37926037	4.93480378	4.66059014	4.41789836	5.77949764
USP40	4.52973792	5.31521234	5.16234828	5.39614293	5.25472827	5.47947679	5.53095805	5.56100225	5.33075868	4.6564951	5.59623997	5.1450454	5.60325047
POMGNT1	5.84851038	5.28854631	6.07945129	5.3147274	5.6552411	4.94871365	5.70039978	5.46585362	5.60560966	6.04857165	6.08174857	5.49883025	5.42289937
RAD54L	0.59464643	0.16791	2.76737298	2.95176622	-0.16622407	-0.13108879	0.64132047	0.37032911	-0.42646937	-0.42646937	2.497981	-0.42646937	-0.42646937
MAST2	4.64146807	3.84833877	4.96659111	4.39077638	4.92692907	3.91798799	4.47184644	5.14688652	4.5844458	5.39614228	4.83980925	4.42767961	4.01994538
DNAJA1	5.71010869	6.16861895	5.79379143	6.89416747	6.63051769	7.05718578	6.46213744	5.70209944	6.59952653	6.37460727	6.13205224	6.20560331	5.83143962
B4GALT1	7.16949399	6.83661884	6.72544702	8.08182431	6.58872414	6.19066624	6.274972	6.00040913	5.92792991	6.26720143	6.54053735	6.22315069	6.74693493
CHMP5	5.66724704	5.59225279	5.2620803	6.14660407	6.26240069	6.74803493	5.66534295	5.8972095	6.72894566	6.49551306	5.9962061	5.91143462	5.95271833
NFX1	6.49789896	6.5055816	6.06167379	6.26398873	6.41275719	6.26023561	6.06789676	6.34200179	6.48532784	6.71532928	6.10407702	6.30120653	6.57104358
DIMT1	4.7167075	4.47100796	4.8143966	5.56810193	5.38195006	5.36658816	5.55939638	4.97239061	4.68347569	5.00603071	5.09162434	4.93445099	5.28328522
IPO11	3.79789653	4.56571246	3.67064901	5.25273028	4.57055755	4.99788142	5.02142071	4.56109527	4.41607794	3.28512008	4.97792364	4.96636336	2.65144624
FOLH1	1.23373087	1.15953867	1.16205161	2.34381331	1.5514677	2.48180863	2.31454782	1.62750783	3.90944891	-0.75845573	1.19831451	2.00802394	-0.75845573
EIF2AK1	6.02247158	5.67862557	5.31293204	6.16180136	5.76969575	5.58379218	5.42356391	5.85242169	5.85971015	5.63307261	5.79596168	5.35261082	6.23306628
EPDR1	6.15926303	6.60476858	5.34704523	5.73754962	6.28450786	6.92250199	5.34682107	5.73912928	6.7794325	6.40026137	6.62964674	6.3196354	6.35211484
SNX10	3.0493449	3.85965923	2.53952774	4.41136412	2.60906802	2.83826946	2.83525031	2.58633903	2.83481267	1.81440526	1.79623382	2.88473572	1.58676125
SEPHS1	5.31589115	5.7995435	4.85871355	5.08317507	5.06797962	5.32174388	5.1968914	5.47310851	5.67372691	5.73593022	5.34443024	5.61326829	5.87244946
MRPL28	4.35069315	3.51118774	4.95584581	3.57530368	3.72299104	4.35847097	3.18639608	3.88931098	4.26634848	4.34772938	3.39019798	3.99807872	3.56137527
ITPKC	6.17587275	5.39933523	6.94961851	5.56867861	4.76429193	4.91716333	5.30885296	4.60561542	4.19838585	4.47742661	4.47337628	4.92490366	4.46038168
FAT2	0.48361951	0.40803499	0.3437704	0.36628899	0.38564447	0.41781503	0.34116538	0.50893787	-0.16475233	-0.16475233	-0.16475233	-0.16475233	3.2945916
RBM22	6.11229164	5.6812203	5.36240162	5.99080418	5.95109615	6.14356409	6.10567572	5.62318225	5.78745294	6.21763654	5.53979564	5.55642573	5.98102602
TMED2	7.09599022	7.57147424	6.71102881	8.14270909	7.62410872	7.53145663	7.44815294	7.35309399	7.69219103	7.46483175	7.72760182	7.53566343	7.40541017
ZFAND6	5.61148875	6.05691817	5.15865537	6.10746779	6.17362801	6.17072561	6.0157541	6.3029906	6.18278102	5.87507135	6.07165205	6.24183134	5.87158732

TXLNG	5.23995912	5.27741682	5.03789125	5.3465984	5.99566983	6.19562289	5.68507484	5.43349962	5.32216975	5.59124787	5.02853298	5.44342473	5.45634571
PPEF1	0.66964987	0.42286248	1.1575087	0.99715814	0.55843075	1.61427202	0.35541459	0.81839787	-0.2816115	3.9006768	3.58752315	2.71339184	-0.2816115
LAT2	4.31182409	4.40458911	4.83671437	4.73283461	2.97250723	2.30813665	3.0883593	2.59573217	3.74962819	4.04157256	3.03254255	2.51414144	4.48249991
HUWE1	7.77255102	7.2640466	7.50359636	6.96032005	7.25368818	7.11648501	7.65324161	7.27539485	6.95403962	7.47583701	7.32570058	7.32834208	7.5893627
ZW10	3.87428238	4.12107248	4.08828036	5.27236328	4.71101377	4.8802059	4.61598809	4.51257605	4.73542972	4.09797393	4.86077093	4.46727779	3.50353349
ALG9	4.9137238	4.83957609	4.60983782	4.44864753	4.94504992	5.33926217	4.91008125	4.71769891	4.66312799	4.78459312	4.88135636	4.7232055	5.31524463
NOX4	6.44487721	7.33521644	6.63546887	6.25395215	7.81877074	7.48872119	7.23346892	7.59654306	7.57890849	7.09448169	7.08841243	7.46485833	6.72495579
ACOX3	4.59048491	5.12761895	5.16186462	4.28832449	5.81818804	4.7111244	5.77970525	5.78070785	3.83838434	5.24580701	5.54826373	6.00567198	5.45838501
MTMR2	5.20712342	5.77521157	5.4564147	6.25976385	6.31689785	6.29050807	6.15323507	6.73366231	5.81431226	5.72589147	6.16471182	6.02523189	5.64312898
PPP1R15A	6.6129306	6.88977379	7.56817089	6.4108342	6.74718579	6.76946922	6.48253453	6.40076619	6.71964311	6.62237122	6.13672575	6.23908207	7.07761003
HSD17B14	4.18682724	2.13271002	3.8543186	1.90977127	4.06669668	3.784519	3.94251358	3.64135058	4.00672602	3.07601012	2.47226563	3.76017077	1.4344679
TRIP6	4.3335254	4.70237837	4.75855647	3.55142606	3.94635728	2.52992969	4.37277488	4.20739548	4.10478381	4.88628312	5.07368493	5.85647379	3.47868098
ACHE	0.64763443	0.61796014	0.75246496	0.22048106	0.7407963	1.54772267	0.52765963	2.08022726	-0.33789063	-0.33789063	-0.33789063	3.20200904	3.24420504
FTL	9.75091685	9.20173468	9.75091685	8.96291348	8.1086067	8.44495893	8.69291911	8.25880287	9.20067844	9.31980585	8.58642787	8.68806474	8.26208325
SRRT	5.92040859	5.44248982	6.33197463	5.39833689	5.39166935	4.97849285	5.55859666	5.7823884	5.84099016	5.73038268	5.47896362	5.56007112	5.29748249
BAX	4.33295565	3.73920232	4.74958084	4.15527298	3.33094774	2.69837122	3.61532316	3.53542807	3.84851304	3.83644077	3.31952063	4.13725981	3.36443605
NLK	3.49055168	3.98190849	3.87418825	4.90508561	4.34488293	4.23306624	4.17317671	4.34245857	3.50451978	3.90869307	4.37200456	4.33733295	4.58384006
PIGS	5.77749924	5.38500477	5.54022422	5.34987115	5.10886366	5.01791741	4.81581849	5.11975447	4.85889951	5.69739597	4.97869952	5.22263883	5.07858503
ADAMTS2	4.29959318	4.70584497	4.86649891	6.54245847	4.38512368	3.50503494	4.08741596	4.79755208	4.19038281	5.14767733	5.44455413	4.99369345	5.21900289
ATXN7L3	5.19358777	5.36013494	5.6379157	5.27813227	5.14735784	4.39729189	4.936406	4.85930724	5.20977155	5.31251983	5.02977421	5.94196628	4.86663595
PGS1	5.22181459	4.65149185	5.08815856	5.08671322	4.67807939	3.33925493	5.03337062	4.51411997	3.74422592	4.38231539	3.76700943	3.63947477	5.28462798
PSMC5	6.15789138	5.57154622	6.29806434	5.54292486	5.59663142	6.20824115	5.22618655	5.55989257	5.51505696	5.54338618	5.5172862	5.7429106	6.20832398
UIMC1	5.54819991	5.45869583	5.52430482	5.72357115	6.0979114	5.80265594	6.21276734	6.11087801	5.6796855	6.13316229	5.44969209	5.62626131	6.00428211
CETP	0.52671326	0.1072325	0.37730464	0.90985954	0.27937606	0.69945934	0.15262106	1.30621747	-0.17923371	-0.17923371	-0.17923371	-0.17923371	1.95309333
MMP2	8.68049104	7.54717633	8.45828586	8.30577469	7.54717633	7.06255979	7.85609082	8.67484663	8.25453916	8.23682077	8.49193715	8.41013532	8.42182839
LPCAT2	5.30291035	5.32757248	4.51838891	4.75220059	5.29997417	4.2732631	5.14360879	5.02846485	4.75518151	4.11949723	5.29056488	4.52486979	5.03183433
GNAO1	3.56047548	1.84778431	3.09779251	0.58094216	2.67848168	0.2874746	3.16092506	2.43793816	3.08318762	2.6482186	2.94999102	1.80434772	1.10455318
OGFOD1	5.73350305	5.81061336	5.74986644	6.24480988	6.19113234	6.51168628	5.6970232	5.65061652	5.60616338	5.75819094	5.63598686	6.00867191	5.88441209
SH3BP2	4.93004404	4.9571302	5.17808446	5.35178395	4.50063822	3.25479051	4.52263093	4.04713693	4.54304223	4.43120483	4.80138118	4.14316217	5.55738598
NOP14	5.56928807	4.9097591	6.07434219	5.34125663	4.57288839	4.82799435	5.01031061	4.72089304	4.93157176	4.44119517	4.95850829	4.30683029	5.27450206
ADD1	7.77342085	7.80477634	7.71951016	6.93194763	7.60499251	7.43071215	7.98159204	7.57798767	7.54700292	8.16192781	7.68364545	7.83474317	7.95074757
L2HGDH	3.51784098	3.57381228	3.71816261	3.44314631	4.04761359	4.29242904	4.10788589	4.25553703	4.19667491	4.20452195	3.34509171	3.95253189	3.95428541
TXNDC16	4.50816408	4.85398631	4.23918958	4.83624056	5.43668788	5.89651952	5.55438878	5.77132485	5.7420712	4.53677424	5.38361959	5.62672062	4.46648576
NID2	5.24730358	4.49403272	5.82877784	7.32008535	3.40368225	3.71102445	4.29333214	4.11735406	3.53408849	3.50997588	5.16487052	4.55225724	5.60475723
GMCL1	4.74697845	4.7134752	4.60830549	5.10732955	5.55693507	5.50530712	5.63691788	5.74943522	5.25882541	4.24705152	5.41494719	5.25342698	4.85936535
SF3B2	6.78968572	6.53922296	7.31917499	6.28405068	6.51021323	6.6117821	6.30055343	6.25692341	6.91311218	7.34948809	6.73178071	6.77433212	6.75776788
GNAS	2.01562431	1.38631974	2.0339452	1.45827077	1.74941726	1.61654322	1.56324477	2.27305382	1.36646864	4.91718624	1.36646864	1.36646864	1.36646864
DNM1L	4.62792232	5.36092986	4.84881666	5.81456625	5.8990728	5.93388088	5.45536787	5.79589198	5.5606232	5.29863519	5.76508341	5.36304307	6.01575487
PTHLH	3.90323093	4.02145401	3.95463052	3.301785	3.79886106	3.70500038	3.11506111	5.50682615	4.74355729	3.99405607	3.18554179	4.16396953	4.40351026
ERGIC2	5.49021315	5.79990128	5.2340627	6.46051646	6.33384997	6.65550651	6.22694512	6.17687901	6.34136874	5.08694951	6.18425915	5.86559091	5.85168959
TFAP2C	0.49941982	0.88902804	0.60664817	0.49206053	0.79757379	0.60640415	0.70901263	0.80690759	-0.3708033	-0.3708033	-0.3708033	1.03191709	2.348933
AURKA	2.38825237	0.56893627	3.30822687	4.86579717	0.93242787	1.04557252	2.04500271	0.81908214	-0.89437438	-0.89437438	1.09132482	2.18480297	3.60601271
CASS4	3.84443084	2.58257462	3.01352743	3.47651379	1.95248101	1.80777562	1.72131905	1.56617384	2.42539061	2.98553098	3.25695125	2.16578161	2.51163194
PIR	4.05574	4.02804262	3.79257559	3.31668236	4.17979137	5.00049716	4.02558014	5.17951191	5.10419137	4.0864232	3.33559921	4.75494762	3.54472981
RFX2	4.74578213	3.41884392	4.13983161	3.40582512	5.05373039	3.75864506	4.20226943	4.43969141	3.37145021	5.09753216	3.85699275	4.80123169	3.75471421
METTL2A	4.67933608	4.75289059	4.74160084	4.43003415	4.50707981	4.55641358	5.02609707	4.81129787	4.57439253	4.76255324	4.7631255	4.85990671	4.50222088
SULT2B1	0.21075823	0.14743548	0.15001669	0.39339903	0.14743548	0.1488706	0.67640291	0.34014457	0.03701225	0.03701225	0.03701225	0.03701225	0.03701225

ALG6	1.96115779	2.94898358	1.95167501	3.79688469	3.60950375	3.76903605	3.78216616	4.35020019	3.40379436	-1.47135329	3.40444555	4.15278154	2.5785692
CNOT3	5.48646953	4.17717649	5.45337493	4.57774575	5.02710752	4.56778422	5.0569812	4.81920662	5.32006966	4.70844288	4.88866571	4.29701172	5.04386038
GP6	0.24726727	-0.11053643	0.33275598	0.16024727	0.32242215	-0.21256479	0.5318191	-0.21077976	1.73764507	-0.14040712	-0.14040712	-0.14040712	-0.14040712
PTPN4	4.95337928	5.62726836	4.94753594	5.70484072	5.8904502	6.11651638	5.994227	6.07681602	6.19156512	5.15295546	5.96666963	5.97647588	5.05456796
DDX18	5.5539807	6.1327788	5.52129174	6.60991818	6.16428226	6.40366617	6.16912129	6.06977053	6.19139808	6.04959867	5.94468983	6.35519981	5.5654248
KHSRP	6.06543352	5.94135274	6.40581779	5.70761653	5.55641215	5.15921317	5.45638156	5.24917428	5.52852185	5.69299478	5.15413943	5.21296105	5.32530962
GNA11	6.59563099	5.85454654	6.56594609	5.06518735	6.01736929	5.74924086	5.82335436	5.74646555	5.53072331	6.03006053	6.16165843	6.15844199	5.6715269
ASAP3	4.38013988	4.61087103	4.65269021	4.23941651	4.08414489	3.84330188	4.88150845	5.00869994	4.69355819	5.19806775	5.50076651	5.30231072	5.49777078
EDEM2	5.09747488	4.66117604	5.26497999	5.28178333	4.21541824	3.80817179	3.94973921	4.05059533	3.63573515	3.97629393	4.97749342	3.67663888	3.82434318
DNMT3B	2.57378379	1.63418563	2.63588266	3.26877535	2.31879952	2.47851718	2.69489534	2.60871575	1.25857733	3.25915594	3.19127892	3.07770603	1.88497064
REM1	3.02509238	2.12035531	3.0383815	2.01276522	3.26170851	3.91855985	2.85002884	4.41795594	4.20630366	4.75604083	2.8307635	3.73149686	3.9840592
TPX2	3.70709024	2.56517244	5.4049516	5.63360733	2.43753164	3.35338572	3.67065959	2.9121304	3.51678211	4.47354345	3.5622576	3.44261716	3.58807103
PDRG1	4.00563027	3.58446988	5.03333524	2.99032699	3.18960458	3.6286788	3.67198243	3.15130581	3.62715627	4.71032116	4.0391701	4.04164631	2.358537
EPB41L1	3.09889305	2.86154015	3.33099953	0.55274504	2.65317551	2.99987902	3.18140688	2.47597932	1.54733002	2.9604162	2.84000799	3.55562422	2.25506234
DOCK9	4.87564261	5.91928218	4.70781718	5.23980646	5.80132245	4.96393882	5.44664281	5.70689503	5.66296945	4.8822003	5.64649539	5.44566496	6.09928319
ANKRD10	6.17973977	6.62404869	5.93509486	6.83884249	6.57763779	5.97550102	6.90092093	6.78808634	5.91078352	6.15248121	6.8993341	6.43006494	6.69226309
TGDS	3.42636106	4.35373665	3.24142449	5.00954479	4.9710122	5.02214775	4.77015147	4.43388299	4.35891732	4.15744567	5.32740344	3.90207269	2.99998086
DOCK3	1.98864981	2.45764801	1.10243024	0.34195747	0.82414824	1.22901068	1.08860685	1.96467781	2.60277863	-0.494106	-0.494106	-0.494106	2.22530841
C3orf18	4.79834443	4.61153277	4.69696715	3.17273683	4.29944485	4.14760182	4.28926217	4.3309362	4.31513998	5.41234828	4.04452902	4.48783889	4.52274295
COQ9	5.64112827	4.92434427	4.99215033	4.99719872	4.99395665	4.62865269	5.00593298	4.74406141	4.54426183	5.29791537	4.93429423	4.58780434	5.58074175
KIF9	1.69215785	0.92525491	1.35398413	1.29921511	1.77972874	1.36725139	0.77878245	1.00844281	2.39489551	-0.41293906	1.90912501	2.5649597	-0.41293906
ARHGAP28	1.11115372	2.73796288	1.29041751	4.92842294	1.63112653	1.4706494	1.59653283	1.6395983	3.09013157	2.46057038	4.02304963	3.38576421	4.24315274
CRLS1	4.34056479	5.17550757	4.60367626	5.82271127	5.58350365	5.40869649	5.5873029	5.53550248	5.17038564	5.03433941	5.59117075	4.88441333	5.22957151
PPP1R13B	5.43297914	4.184142	5.1645281	4.71066988	5.22883806	4.52078705	5.32195278	5.09692702	4.60237148	4.67209474	4.61301538	4.73144644	6.26712462
ATRN	1.7280794	2.24037091	1.44264826	2.44015691	1.79399245	1.74079555	1.79399245	1.76178041	3.39851025	3.25589423	0.26196107	2.77446818	2.44443111
SMOX	5.23961126	2.72520167	5.20957401	4.11407238	3.35470322	4.20831445	3.26841728	3.98637349	3.72836631	1.97137358	4.82105197	3.02703698	3.34040811
SIGLEC1	5.36704231	4.94619415	5.72217405	5.41964176	3.87960447	2.85419583	4.13817194	3.02607533	4.88389367	4.79346717	5.64354856	4.38651848	4.79489731
FKBP1A	6.59241516	6.81097691	7.14032732	7.94353752	6.62510865	6.58105433	7.07967693	6.60347827	6.37195505	6.90144915	6.54775096	6.46631009	6.70289166
NSFL1C	6.39242695	5.89945954	6.27220775	5.61982099	5.96503114	5.99616728	5.67236184	5.71208459	6.08492128	6.32603409	5.75578371	5.82046384	5.91452792
SLC4A11	0.61293427	0.06731288	0.11173762	0.50156758	0.01344404	-0.20589612	0.17570831	0.148174	-0.21303883	-0.21303883	-0.21303883	-0.21303883	-0.21303883
C20orf194	5.82169847	6.00280062	5.5216038	5.68581644	5.90835648	5.14649757	6.09757011	5.93246097	5.65329836	6.15377472	5.88245363	5.82268271	6.35046659
ZNF343	5.59617604	5.13578727	4.91415806	4.55209813	5.06789969	5.35297747	4.79943652	5.02220721	4.84254487	4.61575525	4.5101698	5.03784084	5.19895939
EBF4	4.48260355	3.82281094	3.77900523	2.1288756	3.72977072	3.22905521	4.00926677	4.15585638	3.17317549	4.84315534	2.58304175	3.11371863	2.21120314
CPXM1	0.42717758	3.78633397	1.03160244	4.68467772	0.91356795	0.05890176	-0.03866197	0.43310308	1.638068	-0.38364468	3.8739424	3.46739047	-0.38364468
MAVS	6.99282888	6.60478898	6.86633061	6.16926887	6.4252727	6.14193289	6.78055102	6.51713986	6.16060513	6.41529459	6.29130441	6.24717077	6.765755
XRN2	6.37661435	6.89495119	6.42565476	7.34364569	6.50807362	6.95483152	6.45581708	6.24257686	6.39412555	6.64879437	6.10413181	6.16563379	6.71215612
DYNLL1	0.83981742	2.75938818	0.72394361	3.04183304	2.95682223	3.12848186	2.13783241	1.15902823	3.51177291	0.59018964	0.59018964	3.27426295	3.32135218
TESC	0.4757424	1.8191874	1.12200491	1.98294001	1.2897153	-0.52048095	0.78800456	1.02975362	0.31617918	3.13447843	2.32693586	-0.26094989	-0.26094989
SNX5	4.9971936	5.73202942	5.47278186	6.41825584	5.60234985	5.62515697	5.86837618	5.65329523	5.89445101	5.87570405	5.70726001	5.87803143	5.54415296
RPL6	7.77640983	7.6169859	7.00202304	7.66295662	7.04132107	7.8833961	6.8224747	7.08498267	7.86052316	8.35567139	7.3187181	7.50794876	7.45723368
SIRPG	0.57993225	1.10393671	-0.01704391	-0.30232255	0.45972122	0.28529492	0.96371737	-0.1442795	-0.14176537	-0.14176537	3.68567887	-0.14176537	-0.14176537
MAPKAPK5	4.57975054	4.30423649	4.50781311	4.42910625	4.72454278	4.34575999	4.43966962	4.80394284	4.46484587	4.97975824	4.83475733	4.57033003	4.48362406
P2RX7	4.25570971	3.9079365	3.97783801	4.24763444	3.1173278	2.31232401	2.68531249	2.81491872	3.1812189	3.37812206	3.99712602	4.00124408	3.7305052
ESF1	4.49603839	4.26397799	5.43018768	5.28617863	5.24705246	6.05505544	5.04378904	5.19857635	6.14873854	5.06765971	4.47284554	4.80934377	4.10325563
RBBP9	5.3828371	4.99381437	5.54533299	5.67635015	5.57962499	6.09459345	5.51226908	5.74183464	5.76444674	5.59191871	5.44398198	5.79889552	5.58532615
ANAPC5	5.609545	5.81720244	5.64705596	6.32320917	5.95211061	5.64421386	6.33414534	6.31673869	5.86481154	6.07343091	6.42191626	6.36953738	6.356963
SLC23A2	6.54489043	6.17541829	6.03612208	5.95990815	5.93572322	5.44166277	5.88632932	5.86641297	5.82028466	5.68442456	6.36446764	5.88549893	6.10360189

DZANK1	3.31976708	2.75963616	2.5280852	2.88110397	2.70482552	2.8092735	2.55021652	2.97897116	3.03834061	-1.12099494	2.7628832	2.5317241	3.76544705
KDM2B	4.94110812	4.07254876	5.21517403	4.45574043	4.05992173	3.91055775	4.29043787	4.16910658	3.90511091	4.55452875	4.28807201	4.91002089	4.10295424
TASP1	3.87876611	2.94957444	3.18091238	2.88314933	4.0199792	4.29112847	3.82923781	5.35971869	3.1546696	2.29049676	3.14757858	4.84704794	-0.92976446
OAS1	4.70470281	5.13431795	4.01917562	5.46843955	3.7849102	3.65867403	2.92543599	3.19672891	5.05097021	3.25222271	4.07763087	2.65189434	5.5009909
RPLP0	7.97042411	8.18537918	8.17762061	8.60246045	7.29816398	6.86515839	8.06984238	7.51925623	7.66379445	7.76899364	7.81488694	7.72386338	7.62643074
PXN	6.00528916	6.13813274	6.47221574	5.89799317	6.22044609	4.99518903	6.01775338	6.39294031	5.49695886	6.03208072	6.34314754	5.91057123	6.08525392
SIRT4	1.69312729	0.16511801	0.46524873	0.42794094	0.60722314	0.60202706	1.23393913	0.94588451	-0.18129903	-0.18129903	-0.18129903	-0.18129903	-0.18129903
RPH3A	0.25020274	0.297612	0.16876491	0.16616735	0.16616735	0.16761156	0.21642515	0.4107801	1.66483486	-0.12732738	-0.12732738	-0.12732738	-0.12732738
KIF16B	5.73597314	5.70093526	5.64090963	5.57804024	5.69824154	6.30665911	5.45948954	5.75932832	5.97381558	6.03788458	5.61269598	5.58533224	6.23198901
TRMT6	4.88244301	3.98687836	4.60853193	4.74459711	4.839386	4.81702844	4.3591188	3.70666143	4.74869796	4.23128067	4.30238382	3.99346551	4.7005932
PEBP1	7.9191345	7.73458902	7.11719135	6.92021094	7.41894415	7.7375638	7.45315615	7.3262752	7.5259466	7.98236399	7.80685518	7.863641	7.78184307
TBX5	1.95845832	2.42057054	0.9910767	1.4121989	1.63646871	1.50015445	1.16159049	0.991782	3.69401547	2.46431595	3.58781431	-0.75014367	7.03599223
BRAP	6.39845251	5.46794531	5.3850693	5.36821552	6.05680641	5.963224	5.94203987	5.7039638	5.59010157	5.39425577	5.2758378	5.22530525	6.17709461
ERP29	5.92206076	6.31412481	5.85689229	6.34445509	5.50279631	5.56474238	5.6982169	5.20991962	5.75136649	6.65673226	5.89215856	5.91844872	6.13363942
NOS1	0.2196066	0.60590905	1.09254886	0.38278902	0.13080238	0.19632163	0.11464357	0.11787333	-0.18539073	-0.18539073	-0.18539073	-0.18539073	-0.18539073
FUS	6.70865096	6.77414976	6.88832796	7.2010007	6.73176281	5.88714725	6.76486985	6.95685518	6.42493894	6.68900268	6.43482027	6.64604982	7.16498406
IGBP1	6.04039734	6.25530253	5.38174252	5.88687268	5.70389091	6.24243341	5.95512413	6.00775834	5.9466471	6.43531967	5.65576359	5.70943899	6.35904265
FXYD5	6.56576729	5.35319932	6.61393443	6.45461043	5.35264071	4.54524514	5.15611109	5.19260384	6.0619451	5.38575242	5.81827505	4.24488976	6.2330279
ZNF302	4.94138215	5.91810271	4.86454207	5.60120131	5.83707786	5.92783185	5.9685165	6.1175512	6.31558961	5.44480729	5.85449975	5.69868883	4.86645693
GRAMD1A	5.84316057	5.43974254	6.20346537	5.46204148	5.41909957	4.02971508	5.34645952	5.36433791	4.72794562	5.41743054	5.17589476	5.06014103	5.63229215
HEPH	6.85251591	6.418509	6.6800453	6.31311317	6.83550059	7.40757468	6.51267329	6.93060789	6.63726982	7.31008106	6.76627419	7.19542655	6.96439714
CMTM1	3.91556583	4.08481831	3.72065936	2.88662313	2.05939624	2.64325183	2.94513764	2.30055032	2.83629495	2.28038652	3.66667308	2.88228413	0.76734851
KCNH4	0.41800507	0.15268078	0.60403476	0.18711426	0.50453907	0.09566068	0.41601891	0.09750879	-0.16578812	-0.16578812	-0.16578812	2.32327687	-0.16578812
GANAB	7.37194034	6.77054246	7.46831041	7.56931288	6.54314672	6.62793945	7.06185701	6.6685928	6.39863392	7.48497788	6.80752875	7.03289444	6.90888065
GMIP	4.44312641	4.38754811	4.84954271	4.52841859	3.48737933	2.81692097	3.84312095	3.55936262	3.35318992	3.63974389	3.72177573	3.79437752	4.04038144
RBM41	5.27164342	5.27805427	4.63277456	5.61165586	5.64005128	5.79445597	5.75741461	5.72056487	5.93872407	4.77015833	5.16893141	5.51190114	4.95868315
BIRC5	0.97400047	0.31110573	4.34429826	4.24157223	0.05751687	0.03475719	1.21631073	0.42661923	2.32523309	-0.70957245	-0.70957245	2.78487764	-0.70957245
LAG3	2.39236201	2.12248332	1.25977579	0.77588461	0.88132751	0.77588461	0.79522703	0.97192341	0.17044284	-0.29199212	2.12206054	2.8418591	-0.29199212
MLF2	6.14225779	5.81640134	6.2748724	5.63521652	5.34649756	5.67956086	5.4927687	5.29934477	5.5195054	6.40661019	5.48488653	6.05519285	5.64911228
OTUB2	1.62483236	1.98787811	2.33768901	1.67170772	1.12287978	1.46709282	1.2228807	1.1969267	1.44151251	3.65349742	2.35378409	-0.94294186	-0.94294186
DDX24	7.14687515	7.22840547	7.07978666	5.98817542	6.79180684	7.67445151	6.51904509	6.69683564	6.91219275	7.29637828	6.61281393	6.66627845	7.62011161
ZBTB25	2.32338863	1.84490852	1.3418115	1.25143411	2.07080012	1.71104718	2.37036212	2.2799732	3.68297358	3.68779185	-0.34273413	1.85145497	2.70450945
NECAP1	5.00703112	4.72284556	4.74893787	5.58898745	5.65470251	5.41070395	5.35020859	5.38331731	4.48927966	5.54680274	5.46652674	5.30722867	4.72337841
ARHGAP4	4.03925082	4.26420867	4.73855472	3.84357565	2.97966172	2.56844076	3.42157262	3.10945015	3.0112961	3.24089041	4.23992159	4.37231866	3.26139687
ANKRD24	1.76480427	0.90816358	2.33673788	1.84363063	1.01357846	1.58553279	1.13110615	1.83732069	2.78924983	2.39505571	-0.79457027	3.28973517	-0.79457027
DHX32	5.01199996	5.20580769	4.32828823	5.74744805	4.88571201	4.66299825	5.26313623	4.81400433	4.44306097	4.54952055	4.94748992	4.82401309	5.6823795
RCOR1	1.75928668	1.01342133	1.01280222	2.24868467	1.43530843	1.21839518	1.55326798	1.46516684	0.02563603	0.02563603	0.02563603	3.26841553	1.38866231
LTBP4	8.50727338	8.21988681	8.50727338	5.40316758	7.81510987	7.60181919	7.94093096	8.1066282	7.3338142	8.67462421	8.23518925	8.64741658	6.92987266
BLVRB	5.56744756	5.26532807	5.89621682	4.70923665	4.49539459	5.02744602	4.52035001	4.68595755	4.87018301	5.1199325	4.77399618	5.27210684	5.04485502
SLC9A1	6.17319506	5.06272364	6.50208529	5.36696965	4.62649821	4.9866064	4.61235113	4.51897145	4.89907164	4.78871645	4.12660611	4.62329298	4.99867785
SPTLC1	5.86328801	6.30436567	5.74158993	6.72754423	6.13085406	6.52542611	6.29542022	6.03481551	6.45981617	5.86522417	6.36541886	6.31560016	6.10451776
PAPOLA	6.1981171	7.01802337	6.18030068	7.68316905	7.62299323	7.20129758	7.61589146	7.38563658	7.10742276	6.39506306	7.17655205	6.7765573	6.37466612
CCNK	5.76266273	4.89885056	5.69839165	6.03738583	5.63302451	5.16464533	5.28102975	5.43121133	5.16482668	4.57884842	4.66204024	4.98363123	5.97752215
PCBP4	5.28117218	4.70559967	5.47524163	3.82792219	5.20616617	4.97386116	4.84682916	5.42374813	5.17941755	5.8450051	5.10547409	5.71439212	4.93639536
RGS1	3.78103312	3.52902916	4.33436862	7.01727721	4.18944426	4.23576561	3.59642277	3.96506871	4.10152185	4.23321208	2.80638263	5.16609186	3.58028412
YPEL3	5.56931936	4.32390229	4.98911918	3.27453667	4.37231965	4.53871674	4.4395359	4.89757092	5.58324021	5.89780824	4.37933657	4.53513665	4.69784816
MRPS33	3.36458931	3.52760357	3.97583586	3.9500398	3.799471	4.7028131	3.64734857	3.55719721	3.96663373	4.28450497	3.20415959	3.81831957	4.44693129

NDUFB2	3.46824939	3.83747362	4.76575201	4.05353643	3.34860282	3.48169688	3.89522938	3.68966643	4.5132496	3.54112975	3.76070947	4.47795346	4.30125635
NUDC	6.18182233	5.56356131	6.64823373	5.2597728	5.55400809	5.66124724	5.31182529	4.91864579	5.69375265	6.04277849	5.52545063	5.22981284	5.47750086
MAEA	2.73522126	2.00553212	2.78063571	1.76819767	1.73814069	1.8941912	2.37162978	2.08676434	-0.04744886	-0.04744886	1.30364938	3.04180006	1.94588769
ICAM1	5.97809829	5.4282456	4.77830827	6.76351516	3.97795801	3.28697576	4.70191212	3.08666878	4.55456521	4.60860191	4.45062339	3.57186406	4.89862544
STRN4	5.4810728	4.9242721	6.14167423	5.08363173	5.35292595	4.72568417	5.32011091	5.02932435	5.36807854	4.43076353	5.25843041	5.76025066	5.1335899
IRAK3	4.67305956	5.62042322	4.13506367	5.70703775	5.18939619	5.12013132	5.50482964	5.10919705	5.02702339	4.06199011	4.61620779	4.93632448	4.76282226
MUL1	5.50047929	4.51214493	5.59263016	4.86070423	4.77201436	4.82980856	4.9451139	4.62588651	4.61763838	4.88001855	4.02296943	5.08342777	5.35682608
TFAP4	3.08490923	1.80026929	3.14564645	2.06879182	2.0220278	2.57037836	2.81871058	2.39114195	3.83951629	3.04434692	3.11520445	2.41206296	2.44426494
PDCD7	5.62563306	5.63551444	5.83180979	5.3730179	5.41556522	5.63257083	6.12079585	5.08321205	5.5806007	5.18771136	4.97805228	5.17796896	5.42272065
SPG21	5.55694698	6.26477059	5.47262176	6.36414198	5.96034805	5.36024971	6.14974775	5.48212025	5.65117644	5.47660852	5.60545474	5.30047285	5.72293349
FETUB	0.23523015	0.18742942	0.5014758	0.30917706	0.62155593	1.49116761	0.18742942	0.47482776	-0.36978064	3.7745568	-0.36978064	3.82145494	-0.36978064
DNAJB11	4.27289381	4.14318	4.64080279	5.66330773	5.3214083	5.33215329	5.38226787	5.21749601	5.1991728	5.04446125	4.91328004	3.92562195	5.08543214
THPO	1.46860494	1.50501092	0.60009801	0.84937234	0.49945639	0.6299885	0.80127212	0.57310025	-0.40708011	-0.40708011	1.54540059	-0.40708011	-0.40708011
CHRD	3.52954075	2.55492848	2.66157894	1.642093	2.31110213	2.22094066	3.43711773	3.8937936	2.71012353	3.77286468	4.09652149	3.64705891	3.33837715
FLT3LG	2.61001299	2.46676773	2.87170479	2.24691313	3.26129092	1.60038002	2.70625231	2.82423404	2.41686085	2.95348764	2.90760973	2.91885069	-1.19089159
RAB11FIP3	4.25691256	4.30738357	4.58872673	3.17677557	3.94077255	3.52539886	4.06713676	4.19294706	0	0	0	0	0
GNPTG	5.3944194	4.99806217	5.85852362	4.97667099	4.70452233	4.74490261	4.6132185	4.93150647	5.20379385	5.83477745	5.56670053	4.94479583	5.14480954
ZNF268	4.65882288	5.20154892	4.71221244	5.21773875	5.39813716	5.96780257	5.25303346	5.30871342	5.50400517	5.27219799	5.38853549	5.44927505	4.79525134
GOLGA3	7.02249415	7.41711066	7.43988924	6.6908609	7.03482834	6.68291229	7.03188526	7.03789472	7.38316925	7.47624652	7.51734827	7.18027947	7.30438697
PABPC4	5.56053339	5.7883437	5.93890005	6.11724737	5.15746652	4.78549581	5.41596926	5.38468677	4.75453659	5.3971321	5.43195759	5.53285166	5.32456704
CD209	5.4592302	4.46357953	4.42969775	5.13033205	2.87736589	2.31387983	4.34382652	1.31537386	3.7552596	3.88387941	4.14970732	3.00864423	4.78427073
CERS4	4.98831977	4.50288484	4.81732694	3.14970787	4.39416352	3.99946628	4.65248692	4.20463768	4.19340366	4.61834481	3.9328949	4.60499591	4.11344966
MCOLN1	4.81046303	3.91646303	4.83394673	3.54480723	3.45726793	3.16553434	3.62182374	3.40991038	4.24862284	2.89696324	3.47113432	4.14636971	3.53497925
USP48	5.8834283	6.21560438	5.93143766	6.65042917	6.58891501	6.34080002	6.65256154	6.90555344	6.74109929	6.53974867	6.29437155	6.0864797	6.12895825
EFNB1	4.20482037	3.81054438	4.1177524	4.68494734	3.37662215	3.77253107	3.17345891	3.00717136	3.78281585	4.33116315	3.46129874	3.28119915	3.70551267
PDPR	5.96837633	6.37827546	5.50434867	6.38780623	6.15248951	5.17916934	5.6519065	6.76690684	5.68587479	5.97406236	6.42581807	6.69559699	6.04746487
AARS	6.41499783	6.45983354	6.6953793	5.93121745	6.05679892	6.06759233	6.60394827	5.89492239	5.67166582	6.13423042	6.13081629	5.9993472	6.38286187
GLG1	8.02658321	7.46500793	7.7780085	7.37906426	7.51873249	7.76047855	7.82431274	7.86884758	7.35799867	8.05189599	7.68068006	7.64074651	8.01136915
KIF4A	1.19024839	0.39611829	3.65966882	3.48269875	0.58478905	0.48852263	0.79495882	0.75276446	1.68991089	1.76448084	-0.74126065	-0.74126065	-0.74126065
TNRC6A	6.99753064	7.07190568	6.24129752	6.40534481	7.27735856	7.21506585	7.32159502	7.39022167	6.99950717	7.29105625	7.24535985	7.04478566	7.23060495
PLEKHG2	6.02113012	5.54479307	6.47281535	5.85422056	6.18777785	5.25994566	5.78465046	5.70044565	5.34862915	6.10594839	6.03506089	5.70720451	5.97249406
NAT14	1.00298269	0.58532353	2.7046188	0.91432833	0.66680294	0.09379939	0.58444733	0.9127821	0.73469487	-0.00057685	-0.00057685	-0.00057685	-0.00057685
PITPNM2	5.3474628	4.47589897	5.17157919	3.94261954	4.28555954	3.90711053	4.58382112	4.15471992	3.4064848	5.20601374	4.71167538	4.31822675	4.41667438
EXOC1	5.05608906	5.71730507	5.20855624	6.2790489	6.02233893	6.2249723	5.89727303	6.07806496	6.028263	5.38281836	5.50932343	5.61916698	5.95560098
RBM27	5.50779113	5.71152236	5.67671979	6.08292282	6.36670107	6.74971828	6.53387584	6.55615954	6.41630702	6.62017073	6.02693625	6.46002787	6.21276446
POU4F3	0.47134726	0.42957744	0.25936827	0.32252737	0.47463172	0.01720222	0.01578097	0.01904217	2.91471749	-0.14309881	-0.14309881	-0.14309881	-0.14309881
OSBPL8	5.83959802	6.7324491	5.9083675	7.25871474	6.89417742	7.29441095	7.05905963	7.05322506	7.38636872	6.27108759	7.0582093	7.00634641	5.69917357
DTX2	2.74917029	1.29481463	2.21683643	0.91948073	1.48124156	0.49955439	1.42402771	1.18188675	2.19036641	0.06668511	0.06668511	2.71862393	0.06668511
NLRC4	2.64144595	2.77352553	2.87029423	3.83495028	1.01756137	0.29566154	1.72920812	1.22221221	2.49251716	-0.81920896	-0.81920896	-0.81920896	3.50307211
PUS7	4.32569795	4.64404394	5.09170703	5.33976893	4.43503962	4.8381786	4.86438402	4.36932234	5.07996172	4.38908028	3.71205042	3.86281832	4.32120043
LAMB4	0.65626759	1.09836622	0.74300444	0.660216	0.87236684	0.7413981	0.65626759	0.65958126	-0.39054879	-0.39054879	-0.39054879	3.11347629	3.18048493
NRCAM	2.77800133	1.77010156	2.58613937	2.50608565	1.90144962	2.07126235	1.72924211	3.64092462	3.36877177	1.31856184	2.05894271	2.12564696	0.02562947
LAMB1	7.96265725	7.13363623	7.90273331	8.65249939	7.02539576	6.48532728	7.0123543	7.38613082	6.74311853	6.45800763	6.79953374	6.84733717	7.31032058
SLC26A4	4.52183507	3.44812444	3.60212964	4.18273366	4.95824315	5.55597742	4.3257543	5.48908262	6.66221498	4.75347154	7.31369234	4.51777658	5.4907822
DLD	5.65763265	5.65631172	5.69795673	6.30499133	6.1953904	6.40228723	6.40857733	6.37836015	6.28094404	5.57234742	6.09125013	6.31448405	6.43803786
WDR7	5.88741699	5.58753506	5.30237862	5.61613581	5.90175672	6.22053139	5.82700489	5.90872915	5.6309862	6.04161366	5.74910308	5.80746322	5.94669442
TXNL1	5.57442998	5.44783224	5.6964362	5.8307932	6.04860067	6.63668298	6.17746907	5.97373284	6.08663339	5.70088942	5.6644679	5.47932108	5.59987328

IL5RA	0.66844836	0.96234941	0.53124764	0.52864952	0.64875095	0.53009404	0.57668724	0.53196412	-0.29212004	-0.29212004	-0.29212004	-0.29212004	3.63179948
ABCC6	0.61700857	0.34709473	0.97923274	1.25726865	0.35626047	0.45944297	0.66524909	1.00683942	-0.33649243	-0.33649243	-0.33649243	3.33478084	2.17479309
CMTM6	4.68130275	5.82164323	5.13620196	6.77178088	5.80412553	5.54236364	5.82423085	5.61730019	5.76158801	5.03665831	5.85009126	5.61969312	5.56075894
ITGA6	2.86388268	5.59177033	3.47748912	6.11936274	6.51382989	5.0243734	5.08766115	5.01185077	6.11213421	5.79031783	6.28047455	5.15175239	6.61127766
RAPGEF4	1.75591514	3.45693456	0.66258932	2.77511181	2.13240792	2.08488613	1.42664843	1.7570351	-0.75257314	2.43632421	3.02774174	0.29956927	2.03640588
SMPX	0.67358009	1.39735361	0.92565111	1.14092227	2.47355157	4.74451868	3.11691503	1.25098462	3.39038884	3.46917922	4.25083116	3.92728343	6.75695343
FH	4.8445753	5.02754125	4.87173192	5.58310695	4.60640265	5.77447699	4.89310611	4.45600138	4.66152499	4.96732292	4.59784971	5.11864272	6.20667357
SEL1L3	4.68375243	5.11870203	2.97952367	5.24389944	3.67954953	3.3416717	3.73763998	4.02655508	4.59177155	3.75042944	3.67029765	3.13414666	4.46369309
TF	1.55144934	1.11583209	1.00509757	0.92942729	1.02531691	2.55467596	1.1896633	2.01216347	1.74918374	-0.76874523	2.18594786	3.32687832	0.59850116
CDV3	6.50539712	7.30038328	6.39886143	7.4548347	7.37609793	7.0577038	7.17589921	7.33362716	6.91901986	6.6549527	7.00256202	7.24516753	6.98496834
MYO15A	2.13898024	1.31506934	1.77153521	1.24840317	1.70220923	1.86035836	1.82498663	1.94652525	2.96504748	-0.79617038	-0.79617038	3.70772501	0.90734906
ALKBH5	7.04541871	6.5904159	6.28119079	5.72125605	6.29832257	6.2484941	6.38713536	5.84154732	5.95912061	6.65250026	6.08304611	6.69545899	6.08762704
NLRP1	5.25429153	4.9050229	4.97044401	4.39125581	4.67307197	3.22481415	4.64790248	5.17222554	4.6126435	3.86214471	4.69508041	4.96882861	4.62857232
PITPNM3	2.12712766	2.91294561	3.15116404	2.24780994	3.37770326	1.4307003	1.88628797	2.18247068	1.11918631	1.41866385	3.87039948	2.49864802	4.4169026
SPAG7	4.79164415	4.53116744	5.56444147	4.84714694	4.26178846	5.08050598	4.43128038	4.46397227	5.18977283	5.05379704	4.09532087	4.52167225	5.7265905
ORC6	0.9897314	0.39537976	2.45595286	3.50315559	0.95363845	0.73050987	1.05895903	1.10416792	2.83940267	2.958648	-0.58705725	0.44648581	-0.58705725
ZFHX4	6.76069575	5.59581383	5.79132743	5.91503553	5.40035841	5.85851952	5.15970744	5.52078385	6.26317864	5.57548391	6.42896999	5.83214732	6.31093044
CPA1	0.10304974	-0.16840187	-0.16599224	-0.14117106	0.273992	0.36926849	0.3474263	0.07210431	-0.0396444	-0.0396444	2.1183616	-0.0396444	-0.0396444
ZC3HC1	4.10636012	3.73372294	4.53693618	4.48303162	3.89377934	3.38486772	4.40101458	3.81835244	2.91392233	3.98896524	3.80020494	4.42465223	4.02466666
ESR1	4.53637834	5.30642645	4.69483268	3.67956463	4.36203295	5.43162466	5.52987141	4.41544469	5.59299681	4.58413455	5.04548755	5.2124176	5.64671729
RGS17	3.50614199	3.29680518	3.73411725	3.92851858	3.59710538	4.78385444	5.04623327	4.63169712	4.46797434	2.67555492	3.7855998	4.51052476	2.51720121
ANGPT2	5.44959279	4.27934855	4.82044973	6.19005754	5.31232481	3.98554441	5.67504102	4.90647178	5.95492357	5.30080655	4.54883437	5.06490049	4.68692381
TMEM101	4.70944728	3.83245366	5.04950294	4.33031865	3.9446437	4.13858849	4.25478675	3.36372618	3.92257657	5.09423239	4.80081704	4.72729993	3.06206585
CD200	6.29990198	6.99183725	5.69366771	6.46710164	6.74360713	6.95137966	6.20975453	5.98852573	6.47103447	6.48687013	6.31858852	6.23532795	6.69723011
CCDC80	6.50482953	8.78250315	6.56567781	8.62979422	6.98975378	5.89374417	8.1088651	6.58140598	6.88748303	7.6170873	7.14147279	7.32488048	8.09966363
CMA1	0.71572319	0.2772053	0.27976382	0.67665399	0.46392694	0.2786278	1.05238163	0.28046937	2.38873427	-0.05595776	-0.05595776	-0.05595776	3.10471334
PSME1	5.87914911	6.69052321	5.9136429	6.30772316	5.98820234	5.63178013	6.13704523	5.48447511	5.86288601	6.1542715	5.87413438	5.46554538	5.8435558
PPP2R3C	4.14744484	4.68004647	4.46086695	5.65944697	5.17622545	4.91862554	5.52325547	5.11629517	4.84517753	4.85806142	4.76582823	4.64447372	4.25709911
HAUS4	4.66034418	5.46484841	5.15195644	4.43718849	4.28578878	4.58657859	4.92433472	4.34212287	4.88759294	4.71429425	4.34941267	5.12299886	5.10879036
JPH4	0.50698665	0.94572379	0.77791842	0.23523982	0.14821374	0.27319104	0.65996601	0.00118982	1.60658478	-0.14772507	-0.14772507	-0.14772507	-0.14772507
CEBPE	0.2147187	0	0.31837761	0.27354586	0.04941141	0.00069205	0	0.00158797	0	0	0	0	0
SLC7A8	6.03975408	4.85004742	6.42882556	5.76350236	5.1889766	5.24399957	4.77986671	5.11776537	5.43449206	5.21675525	5.40316864	4.37694702	5.262512
OSGEP	5.35449264	5.00701271	5.11653364	5.05039754	4.79622118	4.23995251	4.43484275	5.17531047	4.76155127	5.57190333	4.76641445	4.73576613	5.52486039
SLC22A17	5.39200652	4.69205857	5.03834824	3.18005345	4.3057361	4.69880541	4.40043435	4.47569227	4.41535902	5.42314846	4.80026201	5.75497644	4.39521463
RNF31	5.18376955	4.62864882	5.34835308	4.85307682	4.86899779	4.26979901	4.64290243	4.66485928	4.66969419	4.79167958	4.97617553	4.79005278	5.11321397
SCFD1	4.85152929	6.07691705	5.13401013	6.47654767	6.18647426	5.97906613	6.13220642	6.2091811	6.43696898	5.5095918	5.97865845	6.32739601	5.28821125
G2E3	3.9238416	4.76169099	4.222515	5.61321216	4.80323501	5.24892002	5.21065046	5.31863727	5.0263104	3.46491611	5.3259016	5.25079073	4.07967054
HECTD1	5.48016031	6.19109838	5.60491294	6.25630978	6.12028147	6.34097007	6.23577697	6.32921513	5.96663594	5.27805624	5.93655631	6.21474234	5.67016848
HNRNPC	7.0563714	7.46896158	7.01330949	8.12383312	7.39524922	7.62838919	7.68596248	7.34279581	7.43282887	7.58581343	7.2569305	7.06605059	7.5026892
RPGRIP1	0.21790465	0.29162027	0.00124472	0.80275884	0.08594027	0.00069205	0.10207854	0.17057149	0	0	0	0	0
SUPT16H	6.6946595	6.83102947	6.72813831	6.89401777	6.97430369	7.70014372	6.89666406	6.63780001	6.94576982	7.18032722	6.4075376	6.65747663	6.89978253
TOX4	6.96946021	6.32683398	6.74956127	6.19383191	6.58945834	6.67836934	6.55533504	6.25183484	6.18156111	6.53133113	6.38344009	6.3813503	6.18468732
GEMIN2	2.88962904	3.18007279	3.37505297	3.4351713	3.95617134	3.30815924	2.83151047	3.59985702	2.94275004	3.81973489	3.65872269	3.74136087	3.12116022
TGM1	0.33367891	4.46356052	0.53319222	1.78069943	0.50385592	-0.15321399	0.37174957	-0.10569989	1.01597529	-0.21880193	3.27653142	-0.21880193	-0.21880193
TINF2	5.99807324	5.85951017	5.88486704	5.40263419	5.4907045	5.51909812	5.51977692	5.31332605	5.37351562	6.30535972	5.48076199	5.41966749	5.52313686
DAZL	-0.01570246	-0.01570246	-0.01324263	0.41072795	-0.01570246	-0.01433483	-0.01570246	-0.01256429	0.00039766	0.00039766	0.00039766	0.00039766	0.00039766
TBL1Y	0.26602365	0.4221097	0.08396077	0.15414481	0.1385098	1.24665553	-0.05048226	0.14637897	0.03272922	0.03272922	0.03272922	0.03272922	0.03272922

SEMA6A	2.30023793	3.92482384	1.95331712	4.1899522	5.26992949	3.14421047	3.51208668	3.62891888	4.36325621	3.66995522	4.7066391	3.22035157	5.09762505
TRPM7	5.34996225	6.33339253	5.67595873	6.80477042	6.67729743	6.7026843	6.97105671	6.94551343	6.66606152	6.00255592	6.59066187	6.45346256	6.16628472
TYRO3	3.60055894	3.4141336	3.84291525	2.80204031	3.12266346	2.64224806	3.51803947	3.67543385	2.71869613	3.7332248	2.94396256	3.30312	3.68137119
WDR76	2.95487784	2.88717836	3.9861506	4.62390416	3.97392966	4.01559856	4.40648802	4.03282164	4.2142879	3.51388843	3.69601292	3.34076331	4.15520299
CAPN3	4.45272164	3.88613388	4.30528409	3.55056671	3.95914905	3.3061507	4.27254062	3.9915516	4.05272917	3.35564215	4.45766533	3.8403479	5.16429388
SNAP23	5.60053856	5.74230851	5.24603747	6.03251697	6.20191399	6.13711855	6.2545635	6.19717881	6.27252842	6.01396508	6.02965278	6.18271475	5.68115181
TBX15	2.28290661	3.0157445	1.22972223	3.02091548	2.44660616	1.65656959	2.77358038	2.37914088	1.44998076	2.30275452	0.79892906	2.87923128	4.00448812
PHGDH	4.28258384	5.14973015	4.93283115	4.03298137	4.73046894	5.88450377	5.41687371	5.93945188	4.76457616	6.81176319	5.07600616	6.20282711	4.42119697
COL9A3	0.02312162	0.1239587	0.76246536	-0.06594915	0.30972012	0.60231807	0.24252508	1.27977534	-0.16425416	-0.16425416	-0.16425416	-0.16425416	-0.16425416
EZR	5.04268956	6.97934911	5.68887412	5.71012267	5.4502897	5.62734442	4.96337492	5.30898101	5.63032848	5.71698444	5.94940666	5.78869509	6.82186601
MYL6	6.74773357	7.67190591	7.56890405	7.26979415	7.88421487	8.19961599	7.60280632	8.04706034	8.24357318	8.70283924	7.68707491	7.9669546	7.94722395
TEKT2	0.54704676	1.11354346	0.08843862	0.43881331	0.49262001	0.28318157	0.44066783	0.08913767	-0.14068565	3.0848631	-0.14068565	-0.14068565	-0.14068565
CLSPN	2.26386865	2.11740543	4.8461159	5.20255652	1.6956761	1.980309	2.63404026	1.66219687	3.36812555	1.99574361	-1.33118317	2.89428859	2.68476813
RFFL	5.0694679	4.20718213	5.11554732	4.26494996	4.32499768	3.75105538	4.07341872	4.4995796	3.7235473	4.71478701	4.4215984	3.34638137	3.63251804
UNC13D	4.05302378	3.36648485	4.61660676	3.25503556	3.08676868	2.51304268	2.79573204	2.94292041	3.47121805	2.63693258	4.20499402	2.30299094	3.74573698
MFSD11	4.61381651	4.76229134	4.5174405	4.36616252	4.7227417	4.33289114	5.4882617	5.26513925	4.98819624	5.47935509	4.72162157	4.76781338	4.94954014
DPYSL2	4.68254044	3.73526386	3.51683514	3.14907862	3.20907143	2.98932462	4.03403266	3.83111576	0.58933729	3.09893882	3.59227219	3.59965959	0.12880828
TGFB2	5.16072045	4.91035706	4.40404835	5.28494493	5.98893511	5.96540287	4.80369119	6.39997393	5.5573715	5.19192723	5.74880437	7.06152757	5.9577519
GPATCH2	4.57188412	4.79245366	4.54454355	5.65563826	5.20794224	5.57641417	5.1957577	5.28074889	5.68783042	5.10678911	5.24519712	4.94914616	5.62853172
NUP50	5.55322194	5.69858991	5.8061349	6.47705546	5.61829068	5.849952	5.42959897	5.4426576	5.66998688	5.10425668	5.60165291	5.66236431	6.12642598
CDC45	0.55827324	0.2937124	3.05752643	3.02509855	0.05280011	-0.41334054	0.98045671	0.17712951	-0.42198547	-0.42198547	-0.42198547	2.98851371	-0.42198547
COMT	5.2690302	4.65929455	5.62812029	4.62098109	4.7327602	5.0102005	4.59489395	5.27695869	4.55107587	5.04105618	4.67717098	5.51311288	4.52941747
VNN3	3.09700747	0.35202651	-0.04481577	1.35763168	0.66604208	0.1278545	0.34442736	0.65693201	3.17074169	-0.27701679	-0.27701679	-0.27701679	2.89621051
ECHDC1	4.56802068	5.18542583	5.20413388	5.95215041	5.26820043	5.46802202	5.47932298	5.41291051	5.64042135	5.11248432	5.42245157	5.65174421	5.58657153
LRRFIP2	5.8891508	6.12326614	5.70853844	6.12209969	6.47905931	6.6303893	6.54868787	6.9252949	6.70485514	6.71890158	6.21613561	6.34357869	6.88887686
SEC22C	5.73827952	5.85206727	5.56629566	5.09051561	5.70368357	5.60582163	5.94163213	5.51972896	5.7288414	5.9137538	5.58539896	5.76788072	5.73717529
XYLB	3.80060661	3.04843265	3.60330657	4.26442854	3.34221507	2.77190757	3.17741472	2.78031982	0.52473841	1.32468511	3.36820422	3.57775968	3.75420146
HDAC6	5.49774656	4.90980629	5.11674937	4.7536549	5.36645228	4.52797434	5.37078405	5.46513426	5.28524134	5.31474582	5.25930363	5.79639136	5.21742218
GABRP	-0.1742607	0.09440151	-0.17177832	0.19800048	-0.10226901	-0.0662908	-0.03882568	0.0879638	-0.06450108	-0.06450108	-0.06450108	-0.06450108	-0.06450108
KRT31	0.01503388	0.48236482	-0.02914876	0.25079696	-0.03152994	-0.03020603	-0.03152994	-0.02849211	-0.02560261	-0.02560261	-0.02560261	-0.02560261	-0.02560261
CDC6	1.85424078	2.05955962	3.58279127	4.00536812	1.45455224	1.54300635	1.94825112	1.30821214	2.79876112	2.82252076	0.5731901	-0.12777192	3.40273906
UPRT	4.66138998	4.21979002	4.54106959	4.83094537	5.13011028	5.06888048	5.33610762	5.39197633	5.14111987	4.5139311	5.37153842	5.08768707	4.7862902
CDC23	5.07453354	5.18440344	4.9125964	5.86203564	5.48748644	5.91145753	5.58979654	5.39614425	5.23537517	5.47635457	4.78534946	5.85405597	5.807644
AAAS	5.4365758	4.48279347	5.34860514	4.86973581	4.69430719	4.70522893	5.08536601	4.73892013	4.99041863	4.69948748	4.95065994	5.40271131	4.51523314
CBX5	3.37118736	4.39425768	3.86066814	5.03238252	4.45863813	4.05674861	4.69974143	4.3267559	3.19526794	4.00447916	4.70019671	5.005578	3.27457574
FMO2	8.03673733	8.43365931	7.10690959	6.38383644	9.05442865	8.97689069	8.02108122	7.72042041	8.53970761	6.63681158	8.73034943	7.42984962	6.75992511
MSH2	3.85978945	4.75534842	4.64962217	5.82695031	4.91536246	4.69723299	5.11262474	4.5201103	4.95590101	4.82248221	5.02217692	5.72085551	5.06985315
MAP3K1	5.02398735	5.7516486	4.68517702	6.21933346	5.054923	5.14030848	5.18554899	4.86886769	5.61482209	5.35911745	6.00335218	4.88650124	5.79421203
DHPS	5.66479834	4.83085096	5.39824957	4.4028609	5.09918983	4.89854562	5.42272059	5.09918983	4.38432052	5.35220909	5.00838646	5.09316908	4.42386711
HOOK2	4.89785041	3.54165849	5.44382131	4.11909138	4.46097088	4.1137347	4.53288893	4.76344385	4.4681774	5.46730721	3.59281429	4.96668632	4.04919105
ARCN1	6.98609824	7.0927712	6.75864584	7.58798334	7.5742284	7.640172	7.24678272	7.43116716	7.31219584	7.30808429	7.22487261	7.32612268	7.38919958
EPB41L4B	1.48651182	2.25743854	1.63880475	1.49703882	2.03108777	2.13764301	1.86135204	2.45654266	2.93728024	2.50605516	2.70952056	2.95996824	5.46287981
TMEM38B	2.98042496	2.06861402	2.08255644	2.7053328	2.84568674	3.57202734	3.5521382	2.81855043	-1.26153276	2.37619173	3.01961491	3.54925947	3.28945683
PSMD5	5.51982671	5.59770259	5.4596259	6.30447239	6.01216946	5.85569235	6.13329081	6.13586757	6.02826888	5.58082881	5.73882386	5.70675166	5.42005145
PTGS1	4.65172912	5.64802081	5.46239122	5.55590053	3.47618374	2.94743574	3.91422362	2.94400566	3.57163908	2.89123091	4.54122188	3.41643898	3.78477601
NUP188	6.29233992	5.44800522	6.31174911	6.11467832	5.55978517	5.0557009	5.39049114	5.30195752	5.09963566	4.87055134	5.59370338	5.43874164	5.74018564
CRAT	5.79130751	5.22393745	5.87457896	4.70842245	5.13371399	4.87907258	5.08096229	5.1123242	5.07026984	6.0483864	5.75886485	5.54076821	5.25999009

SH2D3C	3.07678414	3.85331541	3.07372121	4.1252584	3.21973393	1.01596919	2.23155658	2.64802029	4.27532608	2.97363857	5.14481325	3.1068467	4.57266258
NANS	5.41387487	5.50341204	5.94164526	5.94574556	4.56340962	4.87806966	4.51888545	4.20037001	4.23561695	4.81238412	4.63493417	3.94705359	4.47096679
TBC1D2	4.23441851	4.60737354	4.23310523	3.85863467	3.16336807	2.79164704	3.3497873	3.17958013	3.88224889	3.22858441	2.84859157	4.40416903	3.6985143
CWF19L1	4.12822903	4.29273829	4.50852707	4.77748305	3.97983998	3.3385196	4.11105957	4.22096304	4.06818879	4.51973122	3.99054531	3.85996173	3.66980359
SEMA4G	2.46684723	0.78046817	0.82535879	0.40269378	1.46539233	1.24260288	1.09020867	1.66186501	0.52769818	-0.38033506	3.08524186	-0.38033506	1.94475239
BTAF1	6.02902334	6.6938334	5.73526183	6.81047326	6.72777985	6.65006237	6.50455076	6.73871652	6.45576058	6.08908136	6.61082024	6.76535754	6.48309511
IKZF5	5.24288142	5.01976435	4.61967799	5.16202702	5.56008028	5.71903908	5.2684398	5.60113799	5.81579775	5.24008107	5.59399121	5.60947733	4.82311422
BLNK	4.04553622	4.239784	3.42476899	3.63345722	3.04957509	3.25560537	2.15865843	2.73962823	4.06757758	3.89519641	1.10350819	3.5747574	3.68865495
TLL2	0.41200053	0.40031181	0.57279883	0.24193297	0.14535853	0.64834854	0.24216951	0.25709593	-0.01412801	-0.01412801	-0.01412801	-0.01412801	4.41071667
TDRD1	1.88274155	1.96831007	2.2105236	2.02124486	2.37705089	3.71762063	2.50138186	2.64671824	0.43048783	2.55339411	3.28700563	2.57241211	2.63266468
SORBS1	9.18719385	8.98176145	9.01606361	7.7014179	9.84779506	10.0688952	9.48795838	9.84779506	8.77063663	9.8077008	8.80185872	9.26116006	9.49407118
CRTAC1	5.23339332	4.94443248	5.46701169	3.18361955	2.94264874	1.71909653	3.5446844	4.92043165	5.68119772	4.31735675	5.73583803	4.13304676	5.06913915
BAMBI	3.97885329	3.8271554	3.24903982	3.03358096	5.32135518	5.8741016	4.04523544	5.71642977	4.3998265	4.1254674	3.40044737	5.14670073	5.09755784
IL11	0.05245533	0.05245533	0.05503346	0.70360866	0.05245533	0.32520269	0.19932934	0.05574443	0.37088721	-0.20795974	-0.20795974	-0.20795974	-0.20795974
MYO3A	1.00967436	0.85289124	0.85538624	0.85289124	2.30982076	2.52301112	1.26065102	1.2745767	-0.69126693	2.65882654	3.42856908	3.54023935	-0.69126693
WAC	7.75561719	7.49145948	6.74424371	7.6561514	7.94468588	7.82495151	8.02358248	7.71760173	7.63850814	7.54787502	7.12681339	7.15335548	7.70390802
CREM	1.57829076	1.50476894	1.59035282	4.18444474	2.23068038	2.33312969	1.81199461	1.64600491	3.6674969	4.68148894	-0.36217739	0.79710727	3.12775783
NUBP2	4.08967618	4.40734109	5.01945045	2.96217084	3.28719028	2.96754738	3.44194959	3.43367625	3.44268177	4.92296311	4.264296	4.74063598	0.57090471
TPSD1	0.17936834	0.12119792	0.12369326	0.12119792	0.12119792	0.33074844	1.02074056	0.12438139	-0.04145362	-0.04145362	-0.04145362	-0.04145362	-0.04145362
HIVEP1	6.34166928	6.33145437	5.66678569	6.37680029	6.45319152	6.40953814	5.53381596	6.16138239	6.47465738	6.12982538	6.15131241	6.15562354	6.15891245
TREM2	3.00807408	2.74821936	4.74254259	4.30532745	1.49975527	1.03667577	1.20886409	0.834831	3.40238665	4.04360497	1.46195973	3.21817789	-0.86925622
FKBP5	5.70780811	6.61868895	6.52223629	5.20455082	7.41295004	8.79099449	6.73281858	7.64521931	5.30087898	4.93296433	6.16197189	5.4481572	6.46615004
SRPK1	4.35614527	4.9355507	4.55197738	6.09146567	5.22402057	5.19287382	5.36263164	5.10439424	5.09097612	4.19715934	5.67952308	5.31776437	3.85110332
BRPF3	6.82329891	6.01639792	6.4819351	5.4566415	5.92382721	5.77500999	6.13227551	5.86740561	5.4526683	5.81281705	5.73697974	5.6360219	6.00262089
MRPS18A	5.10165565	4.29732558	4.49665398	3.92660085	4.6898636	5.05259945	3.86461316	4.62958695	4.66443432	4.55018159	4.73201933	4.62246544	4.61419231
TMEM14A	2.41257557	4.00624297	2.68032056	2.76106808	3.72508509	5.06452767	4.06998725	3.82506243	4.40823952	5.16019051	3.98292275	4.95469866	3.55131869
EFHC1	3.38109171	4.39574861	3.61326252	4.7142595	4.66291636	4.42855065	4.69770217	4.9927597	4.18764709	4.43994243	4.61612845	3.92678404	4.78789183
HSP90AB1	9.60845861	9.12604085	9.38219418	9.32357071	9.28347987	9.49358429	9.24156643	8.34715297	8.80982902	9.2123127	8.77505158	8.87433593	8.96109226
CDC5L	5.34978278	5.34287392	5.19450953	5.69580367	5.6224465	6.50744286	5.66384376	5.86376988	5.99374313	6.21418398	5.05608665	5.68487523	5.87547698
ITPR3	1.30668827	2.39656961	1.43558071	1.35866974	1.50571005	-0.69650852	0.70239011	1.71973866	1.96226796	0.01520859	0.01520859	2.96975827	2.97942162
ZNF184	4.07165127	4.92742598	4.29008069	4.6937833	4.78645017	5.26732468	5.09680757	4.75703849	5.10976253	5.54555667	4.50298797	4.57539594	4.62824315
DSP	2.66120056	6.67984503	2.81224778	3.97774655	4.30933862	2.99925292	2.8102469	3.9645421	4.10853171	3.4650057	4.98567415	3.48822088	8.59215898
SIRT1	4.71296913	5.52543766	5.08271042	5.50330988	5.99473176	6.07082359	6.059323	6.29978883	5.67859864	4.90031201	5.92367034	6.07321355	5.14111714
HNRNPH3	6.32318332	6.74570166	6.18116462	6.85757175	7.07637957	7.46359849	7.19897437	6.74299131	7.11540541	6.65252813	6.61275448	6.27057016	6.76462712
IFT74	2.14416047	1.69294147	1.71807962	2.2974964	2.21256662	2.44032505	2.53949753	1.49376232	0	0	0	0	0
JAK2	3.89716067	5.61247524	4.4660015	5.08345344	6.19022443	6.33163697	6.07007927	6.2566195	5.42295664	6.60357929	5.53488188	6.72666583	4.40152853
IL12RB1	2.46357699	2.21057334	2.91732168	1.4715159	1.00808842	0.43215004	0.85537134	0.78478584	3.55591746	-0.52291667	-0.52291667	2.72235518	-0.52291667
ABL1	5.17198058	4.07387829	4.82316624	4.41463378	3.97324909	3.5451955	4.13608767	4.65662695	4.06958246	3.87538669	2.16696829	4.28069459	4.50073263
ACOT7	4.28124372	3.50214187	5.16394667	4.47711781	3.97228419	4.44435209	3.72818361	3.43629979	4.01014834	4.20866012	4.40859494	4.03281238	3.93363344
SH3GLB1	6.24047172	7.24820147	6.31088053	7.63033856	7.29566563	7.14238402	7.15973365	7.30588536	7.15841236	6.38324033	7.02735108	7.06248292	6.47010378
CDC7	3.03508246	2.76120583	2.96799571	5.04119524	2.36410973	1.79030651	3.36493005	1.81411666	2.53373725	-1.53799421	3.78214999	2.3581494	1.5002294
SYDE2	4.39558579	5.11067028	4.59565245	4.0151766	5.89968625	5.73546218	5.7080582	5.69594618	5.70963486	5.22196675	6.00959942	5.9482935	5.38750792
PCSK5	5.35738242	4.92972558	5.32875116	4.89416386	5.48687218	5.52426328	5.03556074	6.14616994	4.74632169	5.63285964	5.00579125	5.47028532	5.55427238
SCD	6.06754152	4.51766841	4.40092061	6.9573098	5.89948258	7.77923168	6.49743824	5.47643086	5.04402485	4.21428261	5.81640968	5.95155621	6.4030976
TMED1	4.28242343	3.43352807	4.88481854	3.21277388	3.22199642	3.91223569	2.60545175	3.62348845	3.91334539	4.35195936	4.13062079	4.23622027	4.29594533
ABLIM1	6.52417781	7.57313803	6.01045429	5.99027265	7.16605015	6.06167923	6.72623677	7.61681864	7.15154622	7.417925	7.179103	7.23937658	8.08648279
ERMP1	4.16581952	5.49923422	3.87833166	4.76257346	4.87615911	4.96649517	5.53782351	5.00510687	4.94391279	3.86261177	5.26024515	5.02508314	5.9079571

RAB18	5.74489632	6.05529293	5.6896636	6.76824836	6.83502375	6.9362754	6.59752683	6.64363397	6.72122795	5.93836612	6.90011353	6.55631421	5.81300328
NRP1	7.9114284	7.56693351	7.9435682	8.55432112	6.58245246	6.24415817	7.11340512	6.2866298	6.54225718	6.55682421	7.17825926	6.86972154	7.35771517
PRTFDC1	4.05233791	4.72341063	3.39374118	4.9896076	4.14722371	4.55299159	4.57028987	5.11369066	4.79365231	4.82868678	3.97576555	4.8768051	4.72200106
PALMD	1.06130364	4.57843805	1.90228071	4.5173095	4.39858824	2.88968884	3.73824705	3.74984653	5.31177838	4.96169969	4.27540685	4.80288749	6.73512059
TSPAN15	0.83505315	2.40026574	0.06614406	2.05683335	0.68888039	-0.33018032	0.08807468	-0.63428664	0.20843344	0.20843344	0.20843344	0.20843344	0.20843344
H2AFY2	3.69691207	2.86336992	4.11918303	3.48790601	2.88964048	3.51834079	2.53511644	2.75972489	3.03907253	4.08716147	3.13014099	2.63427442	2.90441133
MAST3	5.6516683	5.19691924	5.96073756	4.2206548	5.27588724	5.09972753	5.48315107	5.47320739	5.43623853	5.79874335	5.44276017	5.77258894	4.39087289
MZF1	4.56365623	3.87758981	4.67093542	3.61143988	4.1132362	2.6779577	4.43608553	4.16470883	3.11215714	4.31406171	4.51754638	3.5332483	2.08334681
OCEL1	3.27181633	3.3246097	3.32690628	1.98048863	1.98522512	2.3292069	2.65681176	2.34711629	3.6895239	-0.66660669	2.4150846	3.01897608	2.32758482
MYO9B	7.05454452	6.62039675	7.23317262	6.59895486	6.92153401	6.27249506	6.5489925	7.17710015	6.45752374	7.25931884	6.9004602	6.82156479	6.6907747
KCNK6	4.85708378	5.57943526	5.39903962	4.00639723	6.01811522	4.52035884	5.09684164	5.42955323	5.21237659	5.82757431	5.203721	5.79415637	5.44816392
CATSPERG	2.35457041	3.53277455	3.6251243	1.63761924	3.29254404	2.22383582	2.29447502	3.00881216	3.04052466	1.90679993	3.55921726	3.35131956	2.61689174
PSMD8	6.79675548	6.26732952	6.26857833	6.48415524	6.3875603	6.69248774	5.98708676	5.70734059	6.36618568	6.75248385	5.94970474	6.29273659	6.57458504
FBXL19	4.23142562	3.79898201	5.11638315	3.23066391	3.23772427	3.0688048	3.95927948	3.65763533	2.92792739	3.55303943	4.55181356	4.67432666	3.07778818
STX1B	2.09228085	1.30201752	1.07391241	1.47235914	1.73667886	1.37761476	1.73667886	2.55109153	1.68600113	2.29414416	0.37613378	1.42126343	4.1199714
HSD3B7	4.61041284	4.82463047	5.07134214	3.84782411	3.61509263	3.41798464	3.85209445	3.38059129	3.86794477	4.22754795	3.76292107	3.72246914	3.13705507
SETD1A	5.5871248	4.74895635	5.76068507	4.54447805	5.0223808	4.86384159	5.02956543	5.0893125	5.03375485	5.64470356	4.99712358	4.67898651	4.85140317
BCL7C	2.98996605	1.96721692	3.96065744	1.71335096	1.63429161	2.27908323	1.94463269	1.97292689	3.37637216	4.32637483	3.33661254	3.6844076	-0.15181847
CIRBP	7.25483252	7.13335889	7.29327941	6.5410908	6.63613456	6.02410155	6.05922611	7.16494275	6.71270744	7.34665739	7.01447178	7.07969761	6.82710883
ATP5D	4.60651177	4.42319122	5.65411518	4.19328454	4.14444705	3.93883998	4.31155977	4.58560783	3.41365659	4.98677112	4.35176536	4.71949897	4.11298931
PCDH11Y	4.24245165	2.21960577	2.35900082	1.47591702	4.08652358	6.00764076	3.83000592	4.67096251	-1.29177211	1.52308878	0.07104372	1.9505067	5.83587021
IGFALS	0.16491906	-0.13090064	0.67357274	-0.13090064	-0.13090064	0.0216411	-0.07379431	0.31933322	0.04736779	0.04736779	0.04736779	0.04736779	0.04736779
HNRNPM	6.85915335	6.58567863	7.17126584	6.86439933	6.30911884	6.1664504	6.41950698	6.11315627	6.60819927	6.96442548	6.5347301	6.18972265	6.7526531
NDUFB7	4.49407091	5.66632549	5.50054955	4.05655005	4.60991658	5.43999565	4.24823651	4.77800887	5.90727796	5.80462203	5.17381784	5.11732873	4.77052652
TECR	5.25621489	3.77245441	4.53952432	3.6714646	4.09856922	4.39566278	4.42872807	4.21107714	4.78421348	4.40918447	3.86430661	4.14979061	4.12217077
TIMM13	3.56594367	3.68645925	4.99244204	3.07344302	3.13984432	3.21819348	3.03025219	3.44732984	3.32276718	3.76646459	2.17557772	3.71041933	3.19064743
CDC34	4.84603282	4.4207275	5.43112354	3.73511296	4.08886185	4.24464384	4.16773354	4.356289	4.67058416	5.17023058	4.48589168	5.17935765	3.76479021
MTAP	1.28919253	1.70720183	0.94873294	2.46923587	1.84788488	2.51997826	2.33436503	1.9930693	3.52106473	3.31625404	-0.17287514	3.63510753	3.13024699
POLR2E	5.52848538	5.481764	5.77613991	4.84420985	5.03199919	5.52662449	4.61400324	4.79000955	4.97047602	6.07594657	5.1158799	5.35001699	4.99470598
POLRMT	5.22076329	4.33795481	5.45187408	3.88718956	4.10679675	4.26924612	4.23954125	3.87888391	3.38246217	5.22446417	4.39311706	5.00484557	3.74190475
HCN2	0.00951906	0.11461093	0.15011969	0.25031091	0.0430781	0.00069205	0.17840291	0.00158797	0	0	0	0	0
IZUMO4	1.45325682	0.437371	1.05975004	0.2172194	0.55702108	2.09507538	0.47416976	3.01583648	2.87947177	3.72785151	-0.33759568	2.28229116	3.24007246
RASSF7	0.75045315	1.89784065	1.22735985	0.50229505	1.00551052	0.93235466	0.81594161	0.66751638	0	0	0	0	0
GADD45B	8.10627418	6.6472623	7.8465604	6.18604595	5.70635588	6.91201692	4.5817485	5.7605623	4.76423625	5.58123264	5.14297411	5.67803227	5.43053552
PALM	2.89091506	4.65419195	2.48758686	2.07793548	4.03028068	1.98986811	2.63595842	5.29173312	5.02706301	5.24075694	4.73767552	4.55207201	3.54495769
MADCAM1	0.07212058	0	0.24944412	0.17503671	0.15393288	0.30571928	0.04485834	0.00158797	0	0	0	0	0
MKNK2	6.28297	6.04744985	6.25643259	4.89721768	5.11091464	5.49909201	5.64336115	5.08397867	4.95017745	5.53504805	5.42532208	5.95801837	4.94057197
ARVCF	4.75800214	5.86210466	5.37606137	3.07988769	5.20179046	4.50221919	5.1258549	5.8475316	5.36707092	5.96298287	6.2250801	6.00518899	4.96162473
TRMT2A	4.62291483	4.09093555	4.95408931	3.15986552	3.88741339	2.98918241	3.68183617	3.88770003	3.82104215	4.91192681	3.28472224	4.816247	3.47948811
RANBP1	5.36981228	5.05992412	6.46561971	5.76487409	5.21613846	5.70992159	4.93344693	5.33191945	5.81473523	5.70806603	4.75872203	5.34585648	5.91390172
ZDHHC8	4.9737815	4.54860822	5.38844885	3.80189124	4.21747862	3.64311206	4.1324591	4.43060078	4.31103178	5.53912553	4.5483234	5.30759784	4.05109017
KLHL22	3.89962687	3.95400274	3.79887793	3.27440677	3.51439068	2.32451709	3.21871535	3.80183655	4.15577146	2.46002418	3.39038357	3.72532444	3.64054759
MED15	5.86754607	5.41868148	6.19888206	5.50673247	5.49064239	4.84118239	5.39357477	5.49064239	5.28059516	5.528662	5.67981085	5.47169572	4.96624893
SNAP29	5.64343313	5.09722984	5.4851237	5.19367853	5.27900449	5.50596191	4.96593886	5.03342176	5.54566473	5.54045192	4.99127021	5.18918554	5.68661982
CRKL	6.77966278	6.63503367	6.18892902	6.18061929	6.19703648	6.17960048	6.6462187	6.03136281	5.94581235	6.5857773	6.20723948	6.07501219	6.4294675
LZTR1	5.44429549	5.51268582	5.86274421	4.99974001	5.35343091	4.96133931	5.33403464	5.48550964	5.42639303	5.68599397	5.69946493	5.9642844	4.81360892
MMP11	2.01518314	2.2209823	1.59426097	2.66956272	0.57282121	0.67929529	1.28885855	0.47186265	-0.0754493	2.71820568	-0.54089598	-0.54089598	1.67621221

CECR2	0.03136944	0.03226476	0.13748322	0.15028856	-0.12048925	-0.0011819	0.54437701	0.49420765	0.01852287	0.01852287	0.01852287	0.01852287	2.4276978
SMARCB1	5.32754568	5.0024021	5.63079033	5.57400673	4.78766291	4.71112931	4.9635201	4.36650465	4.85494375	5.08170991	4.73324279	5.04919289	5.03584338
P2RX6	1.17850473	1.40375457	2.26382635	1.29942074	0.73792425	0.76306597	1.04160499	0.46876189	2.80468428	-0.38889475	3.34718383	2.26902971	-0.38889475
DERL3	2.31747446	2.3237212	1.47214451	2.13811206	1.28259989	2.05226133	1.5290836	1.06991539	1.00755973	3.47130274	2.85443653	-0.73765367	-0.73765367
SLC7A4	-0.11678023	1.41369529	-0.11482142	-0.11678023	-0.11678023	-0.11569116	0.04781957	0.17949919	-0.02846546	-0.02846546	-0.02846546	-0.02846546	2.65642931
BCL2L13	6.44046587	6.48374783	6.33571744	5.95645215	6.00105593	5.70030608	6.05129401	5.6294539	5.79554771	5.62244945	6.19241234	5.84320327	6.23194854
DDTL	3.19549717	0.83099757	0.64288024	1.47455338	-0.25973385	1.75428718	2.35349897	1.74926956	-1.43024029	-1.43024029	3.48426275	3.43378654	-1.43024029
DDT	3.58361074	3.77549856	4.14241551	2.96310248	3.56282391	3.54644665	2.41226386	3.08610297	3.39861009	3.37074307	2.75965347	3.90262923	3.12792083
OSM	1.43429098	0.46195917	0.92980258	2.21504638	0.19727242	0.19673611	0.47671656	0.02769779	2.75808763	-0.3407344	-0.3407344	2.63865084	-0.3407344
CABIN1	6.42143283	6.13320886	6.69235212	6.0435545	5.67379968	5.50853942	6.07453563	5.79600208	5.73255457	5.83649617	6.25621096	5.70222476	5.77391954
TBC1D10A	4.59844809	3.23130524	4.90516884	3.86718608	4.19057048	3.974584	4.28656542	4.9572283	4.09150029	4.44036636	2.91963886	4.47230543	3.95317884
SUSD2	4.04999153	5.23542378	5.65067467	5.4759797	2.87849481	4.23337168	3.76208803	2.61038882	4.36564284	5.61941031	3.75275613	4.59682093	3.98764251
SF3A1	6.95686933	6.32106065	6.85749641	5.97097991	6.40574673	6.31564524	6.12630966	5.98723288	6.05504479	6.50041816	6.01297597	6.13821272	6.43044296
GGT5	5.75591942	5.66631708	6.44952693	5.54799385	3.63822392	3.97672267	4.3515486	3.96433144	3.64533991	2.97405789	4.18181878	2.7147974	4.56236934
RNF215	3.25879057	1.99392712	3.82530804	2.1073943	2.65503205	2.36778441	2.85791977	2.90908003	3.70217562	2.68927037	2.55027609	3.76282364	-1.27377746
SEC14L2	4.17020031	2.8806768	5.10571754	4.11879602	3.21046613	3.14747522	2.65309423	3.77853067	2.07959552	3.01154287	3.15290151	2.99485915	2.31588309
SPECC1L	5.72625045	5.24047334	5.29147983	4.80967944	5.53441101	5.25945599	5.36956007	5.06026116	5.18681306	4.85620467	5.04899851	5.66777687	5.45221021
PPIL2	5.49210166	4.83724721	5.47783892	4.67456743	4.95816563	4.29849659	5.03639569	5.27043501	5.21073858	4.62986694	4.80110531	5.17320658	5.348643
UPB1	1.47126966	1.27877536	0.2528837	0.58570417	2.35256919	0.71285963	1.66231325	1.87582506	3.10945601	-0.46690276	-0.46690276	3.34059891	-0.46690276
YPEL1	4.35602695	4.34413295	4.08402454	3.14837637	4.3392075	4.48242279	4.56457899	5.62810416	4.51250766	3.94100181	4.10770565	4.74928955	5.05670109
SNRPD3	7.59670405	6.71555276	6.91646334	6.70288512	6.69949144	7.84706894	6.44412874	6.54543768	6.60050745	7.06840098	6.5359653	6.66907064	7.26858659
PES1	6.09481574	5.54691101	6.51289783	5.7863151	5.03277617	5.13415298	5.19281453	4.70570019	4.64400465	5.86635308	5.71938357	5.55563781	5.43915028
MAPK1	6.34898414	6.49983262	6.02076654	6.31071285	6.86551184	6.74973077	6.7735425	6.97240172	6.65163692	6.84872271	7.10317988	6.97925902	6.94742734
GGT1	0.65881879	0.04512551	1.32375141	-0.2713556	0.32435001	0.28602132	0.52899658	1.20345702	-0.01217984	3.95375586	-0.01217984	2.8764651	-0.01217984
PRODH	1.27458289	1.34247331	1.27718418	1.27458289	1.27458289	1.43567512	1.27458289	1.69715515	-0.50459094	2.80023809	2.80023809	3.62475454	3.64583187
PPM1F	5.65183606	5.72560976	6.19306344	5.50717047	5.33813612	4.88622064	5.48870963	4.85504444	5.31718733	5.28405238	5.8600227	5.95762772	5.36141478
SLC35E4	2.88615313	2.64556728	3.18971614	1.85435474	2.20331559	1.97896739	1.71266524	1.69305792	0.11553188	3.30993691	2.41135307	3.14039105	1.91246925
TOP3B	4.21443264	3.80319696	4.86275142	3.66078147	3.97603823	3.74538905	4.18486029	4.11177469	4.0476581	4.42793779	4.35846705	3.54237696	3.82527709
CRYBB3	1.01114686	-0.65312411	0.7299477	0.01102517	1.39458404	1.41626678	3.25984369	1.94924222	-0.07048978	-0.07048978	2.84494161	2.77429862	2.89915919
CYTH4	4.96121662	5.00772914	5.35396743	5.24328909	3.878425	3.20697941	3.80959584	3.12704564	4.54545231	3.76532154	4.43749683	3.42133083	4.66216573
MFNG	2.8998905	3.51736806	3.96291493	2.99326389	2.5210835	1.5764536	2.58492803	2.46856648	1.96341508	4.40763577	3.37543564	3.80569606	2.26179455
CARD10	0.29764998	2.41119469	1.26255118	2.50203677	3.2009166	0.95558207	2.58304384	1.72760616	4.53758992	3.9697408	4.46177061	3.29856242	4.31908996
LRP5L	3.23865315	3.68602303	2.54275899	2.04164828	2.28101482	2.11342178	2.79333419	2.37990037	1.54826095	-0.97059289	0.09467876	3.25401068	0.62902314
SLC25A1	5.03771397	4.62166003	5.35224696	4.72232871	5.21092643	4.89462827	4.37237202	4.9355379	4.59986724	6.02936939	4.67681115	5.29393072	4.04558533
GGA1	5.36692549	4.88978012	5.44831394	4.90849492	4.84292514	4.12220025	4.68140459	4.62461435	4.90684752	4.96628361	4.77721257	4.49638157	4.64258342
HIRA	5.5400743	5.17043662	5.96118738	4.86824479	4.85693492	5.0074845	5.03279012	4.87805368	4.70038417	5.62225976	5.12736528	4.89993934	5.18045652
SH3BP1	3.70131028	3.75159631	4.45136445	3.58463592	2.23136785	1.85384945	2.67308493	2.15769114	4.08522649	3.04152559	1.92832388	1.92514778	3.81725924
SEZ6L	0.20831778	0.42207863	0.27942055	0.30093012	0.25436331	0.4055221	0.36399283	0.16243301	-0.12002067	-0.12002067	-0.12002067	1.69665642	-0.12002067
LGALS1	6.87083956	7.67333241	7.63758728	8.23668485	6.62335715	7.52645652	6.68185424	7.0417887	7.4586097	8.25769867	7.66768334	8.19174489	7.16374257
HPS4	5.68292949	5.30888842	5.43613854	4.69948978	5.0723159	4.80671844	5.00851067	5.19512426	4.91271851	5.03424283	5.4712108	4.72679923	5.11349728
PIK3IP1	6.71795846	5.55799735	6.11839265	4.36505484	4.57113176	4.83467048	4.68438439	5.38385856	5.59396626	5.78579121	5.78856407	5.84622732	5.28830301
SRRD	4.49357815	4.26056622	3.75634913	3.64857912	4.61215404	4.47090737	4.12317036	4.53243538	4.1100926	4.40260893	4.39597861	4.8473985	3.4537959
PATZ1	5.49047952	4.47598458	5.17215711	3.55415811	3.82944004	4.08683287	4.19013418	3.91434301	2.8368069	4.86549559	3.70172004	3.60521958	4.62730816
TRIOBP	3.28231297	2.29817132	3.87169833	1.70773151	2.41427604	1.56556878	2.29255642	2.94116988	1.29169266	2.73784001	-0.20245268	1.79109885	2.9516454
TFIP11	5.44118476	4.48792448	5.17263732	4.94303469	4.86247402	5.42231719	4.76863721	4.86528762	5.05124089	5.48604261	5.04525985	5.23435226	4.27800549
GCAT	3.76440836	2.65852108	3.35935248	3.11990108	1.3987174	2.57653791	3.25227441	2.387874	1.54853852	2.83809712	3.34326896	2.60262397	1.04790357
CRYBB1	1.40073094	0.93456355	2.12042955	0.42675386	0.73050645	-0.21756179	-0.20944108	-0.44660571	1.9708486	-0.26236343	-0.26236343	2.84460579	-0.26236343

ANKRD54	4.74772255	4.02747076	5.17521658	4.26105829	3.94693282	3.90673054	4.36932276	4.01564499	3.89438093	3.28043076	3.71036622	4.22713696	4.28272435
EIF3L	6.99568899	7.4243705	6.24568571	7.02401966	6.84662797	7.03021217	6.69469061	6.87619436	6.88723258	7.49502751	7.04851108	7.42202966	6.97452987
MICALL1	4.90004066	4.1340254	5.80925845	4.69445118	4.86891608	3.72183037	4.71201118	4.74418886	2.71569184	4.14102316	4.73039289	4.56709224	5.53475292
POLR2F	4.11974875	3.19554272	3.1790133	2.90715518	4.08368408	3.31548726	3.66240797	3.43164816	3.46856025	4.064778	2.98343451	3.67055754	4.17153303
SOX10	0.87367587	0.87367587	0.87622117	0.88981247	1.71482554	2.03414787	2.21434012	1.63459243	3.16982317	3.25328806	4.6847054	1.8695884	4.61480626
CCDC134	4.1987504	3.62412687	3.35346414	1.93432708	2.40756723	2.75675295	2.75017402	3.27524867	4.11203787	2.17721436	3.4204999	2.87726758	2.08371386
DEPDC5	5.54528045	4.88180585	5.24324637	4.83880616	5.06892247	4.67180099	5.09227195	4.85546059	4.45528762	5.62344081	4.84941554	4.40805953	5.31345702
PICK1	4.88705303	4.1845202	5.00426816	3.72468257	4.43170297	4.21212355	3.92692525	4.91127079	4.65830858	5.63711108	4.27081245	4.67026756	4.63076154
TTC28	6.97840325	6.34385173	6.31787359	5.80682087	6.43035584	6.40054698	6.40452495	6.76906239	6.26413721	7.00870436	6.71049905	6.73766763	6.89529455
SLC16A8	0.61080703	0.31847556	0.48968455	0.31847556	0.50170263	1.31358532	0.31847556	0.32169357	2.35040292	-0.0683585	-0.0683585	-0.0683585	-0.0683585
CENPM	-8.81E-05	0.41495348	3.20187045	2.46277202	-0.49566726	-0.23073492	1.07475179	0.27024906	-0.23009914	-0.23009914	-0.23009914	3.79950226	-0.23009914
SLC5A4	-0.53605084	1.50729856	-0.09503155	0.67994745	2.06807989	0.78771478	-0.18685532	1.45884382	1.70968988	-0.18587043	1.62833833	-0.18587043	4.3389252
KDELR3	4.11453748	4.88928609	4.6814031	7.4411339	5.2282984	4.64082738	4.51112652	5.16433263	6.69530097	5.29087817	6.25032315	5.28025754	4.75263042
CYP2D6	0.79289133	0.08053462	0.34515844	0.33158876	1.04430315	0.38748496	0.35474989	0.01611739	0.57672789	-0.1559589	-0.1559589	-0.1559589	2.2027912
DDX17	8.20869975	8.3788395	7.63116169	8.59043451	8.84537018	8.27639244	9.11487875	9.24802642	8.1975731	8.25524668	8.46354589	8.20355855	8.62572828
DMC1	2.41637296	1.60691535	2.87176426	2.1885159	2.41805035	3.82097467	2.38705808	2.88452196	3.46957696	1.59679583	2.34002094	3.53810379	2.57630038
TCF20	6.22784361	6.16886039	5.88694015	5.50621782	5.82080572	5.84760126	5.93019579	5.93941818	5.85829191	5.73379722	5.82018718	6.16304514	6.42305815
HSCB	4.85802395	3.73278242	4.45904442	4.01826323	4.00742533	3.36359913	4.36561499	3.78484166	3.89489975	3.18702932	3.84635133	3.61961339	3.66376361
CBY1	5.79829783	5.51909908	5.60264252	4.73446907	5.20330948	5.5377861	4.82367071	4.94175263	5.34583532	5.5864789	5.30247106	4.97537045	4.69194947
TOMM22	4.91068699	4.80949268	5.17589831	5.19948894	4.59942687	4.7493325	4.75386698	3.7682166	5.10004683	5.55559106	4.5661086	4.42957931	5.00845972
XBP1	6.02978695	6.88489766	5.8467151	7.25110361	6.08664861	6.59990237	6.00199247	6.29944179	5.7613098	6.27424266	6.44158717	6.37119479	6.03659746
JOSD1	6.0978186	6.17193038	6.0509933	5.79685243	5.75596244	5.50303198	5.72990229	5.34656504	5.47898137	5.46441774	5.71302714	5.88517525	5.77547963
FBXO7	5.76555124	5.53988136	5.44205247	5.57941647	5.98951857	5.91966894	5.87558866	6.13784004	5.81587369	6.24454007	5.89831531	5.78201158	5.82566803
GTPBP1	5.28733299	4.72451456	5.37966789	4.41897557	5.04059377	4.73704869	4.7956835	4.87498686	4.70587922	5.11143584	4.77386668	4.9492006	5.12253211
POLDIP3	6.30533331	5.75931577	6.16838152	5.76475495	5.7236014	5.76475495	5.90053118	5.69872336	5.68563531	6.25194242	5.86081656	5.93805457	5.65365655
RAB36	5.03826556	4.87353509	4.82544188	3.45870441	3.25881231	3.11374673	2.76477022	3.29884786	4.11796444	3.75351937	3.49175722	2.59516891	0.62440702
TIMP3	7.05531322	8.46721652	6.54149696	6.02059058	8.83516379	9.60163446	9.11890196	9.6364967	8.60301384	9.49685061	8.3916827	9.61546957	9.37486126
PPP6R2	5.65057359	5.73106285	6.03624907	5.35280916	5.60387127	4.76251914	5.57538228	5.74400566	5.07167226	5.90600118	5.58431759	5.45774566	6.15022312
SBF1	5.85010353	5.69414168	6.22639448	4.62569677	5.69723003	4.94998389	5.73424529	5.64631017	5.27517774	6.42645181	6.15088512	6.23033372	5.3528659
SUN2	7.60438076	7.34284452	7.62871472	5.42576855	7.42091392	7.88240158	7.4127396	8.02753159	6.92826262	8.2539037	7.05463628	7.81429198	6.71832025
CYB5R3	7.48569711	7.29349463	7.95223333	6.32805061	7.71304441	7.44914585	7.30726734	7.72052746	7.70540254	8.619342	7.7191536	7.54737743	7.41526825
DNAL4	4.3314409	3.50936328	4.29431255	2.87993446	3.40423744	3.56684696	3.51841465	3.43308766	3.34803976	4.20367334	3.54900355	2.44849643	3.49505781
LMF2	4.81894548	5.15103095	5.32686346	4.83561863	4.19967851	3.75859774	3.96498613	4.12622593	4.37153347	4.96405837	4.72754634	5.09879167	3.18980222
RHBDD3	2.94573391	3.41814995	4.32614669	3.07874386	2.00542678	2.15711427	2.50220257	2.65319413	3.68561028	4.32235067	0.24109811	3.93509499	3.39727367
PACSIN2	5.7959895	5.59325981	6.03145348	5.05226022	6.02393985	6.49993564	5.92342012	6.06091495	5.54568109	6.31203061	5.51263343	6.09959915	5.85010994
TTLL1	4.76541681	4.7500443	3.76946043	3.47766676	3.66148789	4.51778209	4.17624132	3.35040632	4.61003958	5.17345161	3.75988743	4.75669806	3.59750648
RASL10A	0.45208039	-0.02193025	1.70252087	0.71283707	0.34335834	-0.23723555	0.09913008	0.94195585	-0.03047978	-0.03047978	-0.03047978	-0.03047978	-0.03047978
AP1B1	5.0856322	4.86003771	5.64433087	5.25939871	4.40285133	4.14913479	4.25373273	4.17389263	4.71606246	5.19291465	3.71236438	4.85198873	4.4395427
HMGXB4	5.54128989	5.08856153	5.62157907	5.26876762	5.03117931	5.71845721	5.46922343	5.19882509	5.28070614	3.92684885	4.99854888	5.0669665	5.39621101
TOM1	5.76850743	4.86757268	5.87031821	5.24895186	5.18529772	4.63796777	5.11041013	5.40198784	4.94321223	4.79046706	4.98180408	4.91534059	5.77638176
NEFH	0.49447325	0.60462027	1.10579607	1.04988556	0.42824754	0.49631943	0.4195387	0.45852965	2.14478814	-0.36734022	-0.36734022	2.30710793	1.72464052
CHKB	3.80933063	3.78782947	4.4054927	3.49728187	3.68987657	3.4784089	3.67267332	4.60447989	4.49527016	4.23130059	4.12987922	4.69692774	3.87665304
HMOX1	6.94914168	4.60576401	7.03170246	7.1179225	2.52149781	2.41890179	2.54625469	3.25184191	3.20702706	3.16099742	0.98008332	3.2066781	2.98567784
MCAT	3.89462631	3.18328808	3.98685112	2.5118571	2.65172446	3.12113547	2.99182939	2.28417862	2.23005632	2.67584936	3.33319094	2.63048987	2.96125689
THOC5	6.00162616	5.23275419	5.17628557	4.61591704	4.78454328	4.80985225	5.60664061	5.44993112	5.21006935	5.02772598	4.27783595	4.80970677	5.51068021
MCM5	4.34442994	4.03334974	5.18698784	4.70944469	3.74651131	3.65150872	3.61785411	4.17658338	4.09248949	4.25599255	4.55992695	4.61976471	3.50631916
APOBEC3H	0.77921043	1.83990553	0.19913216	0.28970754	0.97744719	-0.06635102	0.06718401	-0.31756686	-0.1722802	-0.1722802	3.35978296	-0.1722802	-0.1722802

ARSA	4.85735095	4.74805479	5.25514233	4.43682502	4.19255345	3.72433579	3.74372291	4.14207745	4.54093239	4.71763164	4.47285271	5.65169752	2.95185904
TSPO	4.13534045	4.93813598	5.24806738	4.89936974	3.44354518	3.04408286	3.63260004	3.47729319	4.35047232	4.07673176	4.8778275	4.71125368	3.93873996
RASD2	2.93434354	3.75914839	3.96036174	0.7549449	6.01462292	5.67730803	4.47073703	4.06646916	2.90164003	5.49021047	3.29500251	5.06319789	5.15955668
TTLL12	5.02716395	4.32627222	6.21057436	4.38684583	4.78579958	5.05736425	4.94395416	4.62957096	4.6737615	6.00542569	4.69636974	4.76995321	3.76041099
CBX7	6.44694435	6.47865392	6.28861666	4.30387444	6.37704374	6.14462365	6.36833486	6.48987023	5.97057519	7.12525072	6.57453962	6.99775207	5.88020115
PDGFB	1.25313329	4.12768976	3.8450039	4.10846421	3.32317852	1.74634358	2.69180901	2.69362677	3.87366977	4.43134923	4.55644587	4.23966776	3.8586105
ACR	-0.05208409	1.40826971	-0.02871482	-0.1727736	0.87217411	-0.20817434	-0.11230281	0.89316407	-0.14355465	-0.14355465	-0.14355465	2.29506582	1.89914059
RPL3	7.53234991	7.95978181	7.12167737	7.33012765	6.7059693	6.71598651	6.7681652	6.85611047	7.47616573	8.15177395	7.30450064	7.70277724	6.88159774
ZMAT5	4.06742411	2.42572347	4.21719647	2.98760324	3.60702384	4.14126745	3.27134939	3.31304147	3.17562007	3.52760156	2.78130253	4.14853688	3.29268459
RBFOX2	7.16501273	7.36126651	7.00946909	7.29462472	7.80207866	7.68996821	7.83868606	8.07998146	7.51053501	7.86943858	7.46837821	7.89699486	7.6503242
SYNGR1	4.46467342	4.66887919	3.81079568	1.93647094	3.74282694	2.427361	3.78236238	3.75072364	3.62779133	3.21108176	3.66782061	4.33200492	4.47167628
TAB1	5.43746165	4.85597531	5.79960066	4.6741682	4.95067072	4.49969034	5.00020574	4.99668173	4.68587903	5.25373218	4.66248447	5.04821691	5.3658347
ASCC2	5.57857435	4.94556298	5.64750704	5.5916239	5.40545149	4.51181031	5.09404591	4.97716106	5.08148028	5.54607234	5.08585403	5.31852694	5.65804092
MTMR3	2.30248179	2.1085869	2.11674214	1.16560032	1.7479249	1.42039337	2.23111455	2.28670901	1.71747066	4.53837406	0.22583604	4.00072025	0.22583604
APOL4	0.89802623	1.8712898	1.18634524	4.86062713	2.39712918	1.99757051	2.00097467	1.7325716	4.74277909	-0.81181887	3.78582629	2.71663476	4.65633234
APOL1	6.087632	6.23964015	6.01782022	6.51537155	5.63886796	4.42757198	5.30543882	4.34083277	5.09764431	4.26577418	5.26774966	4.76762949	5.87632301
PNPLA3	0.6601407	-0.21764549	0.41231736	0.43190073	-0.09693556	-0.27647137	0.19722856	0.23266281	-0.08682428	-0.08682428	-0.08682428	-0.08682428	-0.08682428
MYH9	8.66849964	9.09817975	9.59887325	7.95647024	8.95764099	8.66849964	8.82543972	8.70677542	8.93508659	9.17893274	8.92632912	8.77229415	9.03968975
CACNA1I	0.49863847	0.35657889	0.4750091	0.35890168	0.37028224	0.35802656	0.35657889	0.53310473	-0.1796976	-0.1796976	2.67342535	-0.1796976	-0.1796976
SAMM50	2.12131835	1.51502449	3.10603567	2.6810982	1.91848154	1.83833403	2.24889641	2.08693014	-0.26298619	2.70462167	2.55166007	2.82157203	-0.26298619
TXN2	5.41935044	5.05691624	5.66427719	5.17913254	4.40220099	5.00453682	5.03942874	4.41938232	5.41666134	4.89853744	4.87308145	5.30470921	5.37624207
FOXRED2	4.76610911	3.9284649	4.8810197	4.44886522	4.31630789	3.74831778	4.21223213	4.53743359	4.75348964	4.62934472	4.00721205	4.42984129	4.53201645
GRAP2	2.1956565	1.97118259	0.97949982	1.7083388	1.49913824	1.32965797	2.019296	0.50236008	1.87644483	-0.62216262	-0.62216262	-0.62216262	3.11912984
EIF3D	7.62766392	7.21756644	7.53798932	7.11037756	6.90968916	6.66048228	6.7060213	6.96676847	6.75308458	7.30575458	6.50978281	7.06960942	6.94841654
TNRC6B	2.96809864	2.820348	2.32290282	1.94868297	3.37473387	3.31926777	3.19272412	3.75927856	3.73448469	-0.20062629	3.2105658	3.96396434	4.42024949
SGSM3	3.76744143	3.82000001	4.09026368	3.37866869	3.63679755	3.9550615	3.79263184	4.21791643	3.91255713	3.93498986	4.27864981	4.10629564	4.09471962
IFT27	4.12514182	4.33503814	4.14981257	4.10629665	3.19991621	3.02906481	3.20391958	3.49740333	4.33941498	4.96126084	4.27330826	4.63537697	3.39694071
KIAA0930	6.10139425	5.87124253	6.40318532	5.58515629	5.41523028	5.26717074	5.61414911	4.89517739	5.77900583	5.86479782	5.62753803	6.05349587	5.20918013
NCF4	4.50203926	4.12817689	4.6990731	4.28610264	3.52617719	2.12413817	2.85626116	2.08800462	3.82595045	4.45214931	2.51231344	3.25661629	2.94234283
CSF2RB	4.31211174	3.85139205	4.46619695	4.06264208	3.07271075	2.52158213	3.14503656	2.62423367	3.02531325	3.18518744	3.67641765	2.78661101	3.61881755
SLC25A17	3.51091366	3.13848339	3.69535316	4.32226623	4.15451907	3.84125731	4.13014191	4.06989072	3.71212807	3.86410923	4.28701582	4.06014393	3.76140784
UPK3A	-0.20013667	0.27281182	0.45055336	-0.14824056	-0.08453126	0.79908739	-0.20013667	0.66958639	0.07493178	0.07493178	0.07493178	0.07493178	0.07493178
FAM118A	4.90135042	4.6661184	4.96729649	4.56957993	5.02502157	4.59499318	6.65017092	7.04184688	5.09668838	4.90838872	6.92152947	5.39236457	5.99183625
KCTD17	4.41532153	3.85849332	5.12459761	3.78885836	3.59433071	3.41640355	4.03393979	3.95158756	3.41191951	3.27668245	3.32920698	3.58479119	3.81415041
ST13	7.10045475	7.37300853	6.98143871	7.32356606	7.84850559	7.80929715	7.39841009	7.32388233	7.65468346	7.54677119	7.59066633	7.58449933	6.8772715
IL2RB	3.7887257	4.22818551	1.74045272	2.59708532	2.22375654	1.07588754	1.97578352	1.49190463	3.97280799	3.94505563	3.79352085	2.37254368	3.08181118
RBX1	4.46668727	5.13578086	4.22617431	5.35045982	5.12843937	4.52767148	4.49534473	4.65538552	5.10637369	4.00382068	4.6769522	5.24048249	4.62375916
EP300	2.1743302	1.27335296	1.21936708	0.654826	1.50559762	0.85437945	1.49893377	1.38744379	1.23737541	3.22924655	0.77484032	2.89070488	0.77484032
L3MBTL2	5.10872832	4.65233796	5.66587425	4.55966034	4.69113982	4.60528125	4.67501444	4.79704825	4.30780714	4.48416814	5.0289567	5.41769394	4.6321298
CHADL	1.57130701	0.67302506	1.19826792	0.25148493	0.90060112	1.34715307	1.05632868	1.41599494	2.40035485	3.57390153	-0.27108613	3.1595939	-0.27108613
RANGAP1	4.70076031	4.04031389	5.73498127	4.45843809	4.33653139	3.69607224	5.00370637	4.10360385	4.58074442	5.48376664	3.58536461	4.94669568	3.67987722
ZC3H7B	6.70667595	6.22704408	6.61984625	5.74386084	6.38236591	5.86099185	5.9886491	5.89753195	6.01897571	6.40108214	6.63723923	6.22908384	6.34954474
PHF5A	5.12389236	4.06045112	4.57861735	4.11785299	4.28016795	4.8853893	4.23367194	4.22024358	4.66286777	5.33018491	4.03874231	4.03910377	4.20026854
ACO2	6.17088722	5.87177622	6.48963619	5.64772711	5.53048601	6.04554085	5.64377156	5.62632445	5.03567139	6.16171981	6.046238	5.9014777	6.94417743
POLR3H	5.56928876	5.1972106	5.77009951	5.21923585	4.66661298	4.62816953	5.31270332	4.87705711	5.30638799	5.39982345	4.9662493	5.43756481	4.61811182
TRMU	4.05701266	3.23236792	4.86532747	3.83861539	3.82878757	2.76216101	3.99129105	3.932762	4.2084313	4.36356817	3.45231833	3.26148019	3.34550161
PMM1	4.30546869	4.16653371	4.91467969	3.52579286	4.20627545	3.56660129	4.07494987	4.60024849	3.93894527	4.34229041	3.47167083	4.31898052	4.10688341

CERK	6.34160498	5.90501625	6.4074972	5.41746521	5.72288461	5.80067216	6.06940069	5.78972387	5.66040151	6.34583902	5.88679652	6.08475151	5.6513034
BRD1	5.53029208	5.21407162	5.43085311	4.50059379	4.86903019	4.53061403	5.18687946	5.16516331	4.78572741	5.1039658	5.2415873	4.99996916	4.88457182
ZBED4	5.2166974	4.3367648	5.40072063	5.3044926	4.82558079	4.41543414	4.84782427	4.49227312	4.40121637	4.48747412	4.92402959	4.57741293	4.82150946
MLC1	1.99509957	1.09141184	1.83023629	1.14062991	1.56528957	0.81034853	1.7460313	1.15191567	3.8838362	-0.63412946	3.55898698	2.23051475	1.62729421
HDAC10	2.65546867	2.32294266	2.32624986	2.39769657	2.9323245	2.64444041	2.643247	2.77987272	2.15484452	3.33870271	3.17224674	3.64734574	-1.52295058
KCNK10	0.02129843	0.18792797	0.02385352	0.06131852	0.02129843	0.02271903	0.06071165	0.02455813	-0.02060844	-0.02060844	-0.02060844	-0.02060844	-0.02060844
ABHD4	6.26547663	5.44374546	5.93324722	4.95552979	5.83976765	6.24182604	5.39040862	5.62300843	5.72928755	6.31397477	5.9045394	5.6726361	5.31170451
KHNYN	6.01983155	6.49423184	6.07864187	5.91134974	5.89024848	5.61552646	6.24871736	6.0158728	5.80422596	5.92732026	6.23019111	6.15390661	6.27784083
FKBP3	3.8877696	4.43701679	4.35081283	5.44067932	4.95393926	5.60780822	5.24970582	5.14504349	5.87738009	4.85631863	4.72947621	4.98472429	3.19014547
SDR39U1	5.02679945	4.79615572	4.70341762	3.55464433	4.78792453	4.25700366	4.45554046	4.70781623	4.24953535	4.50016918	4.52235299	4.8703449	3.95528094
CTSG	1.73493461	3.68821076	0.59577792	1.61653103	1.71467763	0.59472024	1.44173619	0.59643482	2.87289281	-0.58785371	-0.58785371	-0.58785371	4.27537633
GZMH	2.21099577	0.807711	-0.20945775	1.07689785	1.87464882	1.30414958	0.84082908	0.21561488	2.84961447	-0.38693778	-0.38693778	1.12201558	3.68109356
GZMB	1.2984571	1.39691435	0.14973954	2.3747892	0.83366342	1.06500401	0.147357	0.15039656	-0.24070775	-0.24070775	3.04123793	-0.24070775	4.09692306
RBM23	5.74866401	5.23568741	5.72375647	5.21000741	5.39606713	4.90169218	5.41764318	5.10714536	5.28044572	5.66060374	5.15815112	5.78977914	5.19010232
PRMT5	6.42997	5.15269672	6.20219626	6.34874651	5.33765408	5.66363855	5.34848701	4.70890778	5.05054592	5.46745691	5.14568039	4.85855338	5.72505369
COCH	2.55705169	2.22118092	2.224461	2.28714585	3.31491718	4.43231751	3.24730241	4.07096132	3.85612721	3.30869319	3.5399926	3.92337316	4.00828922
AP4S1	4.35338232	4.53740311	4.64513586	4.25936922	4.23672131	4.04322483	4.48792986	4.65501912	4.5209828	4.79466952	4.20117995	4.45855251	3.88847538
POLE2	1.31597016	2.74566268	2.26797684	4.4535279	1.83844869	1.40876536	2.1297929	2.04881488	1.33743555	3.00960798	2.90270567	1.81607637	1.90191868
SOS2	5.77622271	6.06325232	5.54868507	6.29021123	6.45384831	6.63127016	6.46080914	6.74465942	6.34350143	5.8295151	5.94449663	6.47478655	6.07028421
CDKL1	3.39595002	3.1770587	3.57232921	4.32317843	3.37230842	2.95304854	3.48432308	2.8658809	3.7898789	2.02566416	3.95971559	3.03723916	3.49331577
NIN	6.17094151	6.30327432	6.46028334	6.86971317	6.17626186	6.87054851	6.36304393	6.80571013	6.91272506	6.62751364	6.10156942	6.64202073	6.97384459
PYGL	7.32053124	5.83814798	6.05174951	6.69983913	5.35648375	4.99654519	5.14644858	4.38648931	5.05483454	5.00227265	3.83002937	4.69187465	5.10016676
TRIM9	4.90317667	1.62554656	2.95970335	3.2823907	2.37156185	2.02203558	1.52967575	1.40442235	-0.01604818	-1.21219894	2.13179362	1.48166347	1.873552
PSMC6	4.74092293	5.4290705	5.36791383	5.979679	5.57965037	5.92066458	5.90805318	5.93056498	5.57470366	6.32657803	5.8176364	5.59768231	4.97807454
GNPNAT1	4.74491049	5.32160163	5.96865878	6.65122402	6.24706007	5.65320027	6.07560389	6.76664145	5.36696596	4.30866845	5.62813976	5.90950457	5.02764202
DDHD1	5.11779538	5.19403149	4.6192545	5.3958541	5.44669055	5.50185457	5.40261385	5.69265069	5.13576921	4.85809144	5.34556269	4.98543679	5.14814589
CDKN3	2.4420602	2.04014649	3.56559951	4.94227622	2.4047164	1.65500065	2.20654184	2.23131033	2.78767349	1.75535241	2.6871751	2.94003465	1.17091924
CGRRF1	3.77968485	4.40053248	3.36394181	4.48690826	4.57112098	4.71281478	4.8733502	4.57138388	4.62166912	4.23912544	4.26479167	4.2373487	3.99335128
ATP6V1D	4.89397981	5.42759257	4.69217058	5.92451211	5.59857525	6.11229926	5.42660428	5.74611163	5.51952223	5.9308098	4.96646825	5.28012413	5.35731195
PLEK2	1.69181389	1.61609588	1.70856228	3.27590717	0.71715387	0.42158431	0.84704825	1.27389586	0.66235325	-0.89254463	1.75115646	1.18206549	3.30219045
PIGH	4.35546881	4.91236561	3.46213932	3.65858895	4.6718352	4.31810502	4.28499036	4.66428043	3.65157679	4.33661743	3.77410891	4.34233773	3.01315741
PSMA3	4.82558507	5.70114977	5.13322689	6.17263984	5.36477093	5.42537436	5.62291669	5.56223067	5.49597532	5.0473178	5.22566474	5.54887432	5.4263186
VTI1B	3.81349804	4.49687668	3.23908096	4.3234119	4.79327631	4.6631861	4.49210459	4.368969	4.1918842	4.86039023	3.68568246	4.33110176	3.33925253
TIMM9	4.37162388	4.16042631	3.62580326	4.662406	4.46971023	5.02335296	3.95843457	4.30588872	4.80350323	4.62959119	4.56376473	4.4270834	4.56904411
GSTZ1	3.11737162	2.79441567	3.63503139	3.16822899	2.60281172	2.27273863	2.73490929	3.06436568	2.57584266	2.87641779	1.67454398	3.47667812	3.55007832
KIAA0586	5.01826864	4.71853266	4.72018728	5.4470876	5.60941768	5.73353641	5.25224086	5.14761819	5.3254646	4.44394794	5.02881327	5.29775008	4.24610194
TMED8	5.13750089	5.22944244	4.85364427	4.8632629	5.31389348	5.86963947	5.34644465	4.9979915	4.69543874	4.76522723	4.74302442	4.6864591	5.27282237
SAMD15	1.69911279	1.48607272	2.07510677	2.51534485	1.67179116	1.62250244	1.60279194	1.3060909	2.97157865	-0.90902453	2.06282224	-0.90902453	2.96996165
AHSA1	5.88914561	5.49533097	5.85710014	5.67302075	5.82725873	6.57890504	5.42474	5.37047456	5.8696455	6.14012798	5.71330019	5.89367444	6.35054435
DAAM1	6.17389887	6.98432831	6.05085788	7.07753099	6.48825333	6.76687665	6.21938397	6.42587201	6.81921506	7.01817544	6.54534862	7.07223791	6.9582756
ISM2	0.01073072	0.65251403	0.00124472	0.00727845	0	0.00069205	0	0.00158797	0	0	0	0	0
SPTLC2	5.59558619	5.99555951	5.39906632	6.43467478	5.90679533	5.40410702	5.95185134	5.5509668	5.91990594	5.68040786	5.89771092	5.37272881	6.13728695
RIN3	6.02793321	5.65641177	6.15750109	5.45085201	5.78319424	4.45225144	5.49869097	5.42419779	4.69627015	6.16683225	5.31972288	5.65061584	5.27240386
LGMN	7.47383075	6.55428231	7.50601791	7.2285186	6.19739891	5.55185816	6.43206104	5.6829397	5.99622246	6.28057288	6.12682242	6.26422005	7.06565627
ALKBH1	3.82096613	4.57781051	3.34682529	3.52771959	4.32252869	4.22592842	3.78203133	4.03911735	3.40254614	3.09599148	2.71084178	3.30350734	3.91665427
SNW1	6.01508464	5.79831428	5.62293596	6.26280951	5.9697947	6.76907797	5.72097097	5.79862197	6.13863083	6.25351284	5.35324231	5.84543694	6.3683515
ITPK1	5.72495487	5.64659523	5.84847143	4.41133853	5.29375953	5.37994458	5.47925064	5.61324477	5.57252905	5.2330544	5.81714567	5.88026146	5.89645603

DHRS7	5.72425422	6.11419717	5.37617841	5.38532015	5.92502882	6.13653447	6.29380533	5.89695201	5.86735086	6.21153421	5.60742696	5.94621515	5.95764276
PPM1A	5.93912866	6.65055017	6.06502121	6.49293402	6.99167359	7.12827496	7.06393499	7.11982786	6.93344885	6.20726393	7.12374499	6.8345471	6.42580094
SIX4	4.65453143	3.12133646	3.619664	4.95624476	3.98104179	4.522271	4.35711923	4.31269265	3.4996678	2.89844233	4.39907373	3.14986383	3.44439068
ASB2	3.95216836	3.30029788	4.28397487	1.49372299	4.57949579	5.22463791	5.34118614	5.04222726	4.21110689	6.36580808	3.95606965	5.81172941	4.98446966
CEP128	3.48210072	3.66788044	4.64938162	4.42055196	3.77193739	4.37916114	3.54112687	3.91007972	4.63388646	4.33140975	3.75904559	3.18084397	4.56549139
ERH	5.09086067	5.53971219	5.30534213	5.27602509	5.23609829	6.12849133	4.88629106	5.26753207	5.46749806	4.90266182	4.79625507	5.37084458	4.75525283
HIF1A	6.78237286	6.79407587	6.8612831	9.21778798	6.77987133	7.00135853	6.72719774	6.35166088	6.21626137	6.44123658	5.96882115	5.9374645	6.74325759
SRSF5	7.10750579	7.47860995	6.46044796	7.01894521	7.29343972	7.55931359	7.65705321	7.60617891	7.48773933	7.46580239	7.32951125	7.18949285	7.58311074
EIF5	5.41150721	6.66415565	5.58653136	6.81343333	6.27198493	5.95069263	6.33942144	6.20062397	6.4075183	6.60167402	5.78598082	5.90442163	5.95896662
SLC8A3	1.16468277	1.04591787	0.96360804	1.07434131	1.12474981	1.16136695	0.99359615	1.08847397	-0.46074714	-0.46074714	-0.46074714	-0.46074714	4.63494572
DICER1	6.42691759	7.00601049	6.30440858	7.44785776	7.15248345	7.05054466	6.79870493	7.12906808	6.77131227	6.45414366	7.07579871	6.65502489	6.97237049
ZFYVE21	5.68645388	6.12088756	5.87710899	4.48852726	5.65284502	6.06458845	5.76042024	5.8988129	6.0297179	6.49024474	5.94869962	6.06039782	5.76831071
MTHFD1	6.02951301	5.66333884	5.87002067	5.76602461	5.6676006	6.03826048	5.26945445	5.31083692	5.77365469	5.88515145	5.86847534	5.57701819	5.80906226
TCL1A	0	0.40638476	0.00124472	0.42066044	0	0.00069205	0	0.00158797	0	0	0	0	0
ZC3H14	6.22398132	5.99119863	5.86425723	5.93982405	6.14549944	6.38446373	6.32993664	6.07292239	6.06765461	6.16843353	6.24463447	5.95727674	6.35961344
TELO2	3.87032722	3.57905135	5.035961	3.14060778	3.5847125	3.26256546	3.59255389	3.93811547	3.89104643	4.50983028	3.61462388	4.37820602	2.63448124
BDKRB1	0.3682471	0.54549462	0.55018189	1.75174532	0.3682471	0.36963838	0.3682471	0.37143954	0.07061227	3.08205977	0.07061227	0.07061227	1.35220012
VRK1	2.98900676	3.70019911	3.78146668	4.93453018	4.0498757	4.67307475	3.96279871	4.44685193	4.66548434	4.7088413	4.26542346	4.4014493	4.72158429
PSMC1	4.9455677	4.70234743	5.53409404	5.01277212	5.07695986	5.59319266	5.15505442	5.1512321	5.71516692	5.64944577	4.85272304	4.70696771	5.9220067
PAPLN	0.40043718	1.21462144	0.17697304	0.26344983	1.34866816	-0.76233982	0.7298708	1.14772506	0.27537017	0.27537017	0.27537017	0.27537017	0.27537017
RPS6KA5	3.32900358	3.32135523	2.96847731	3.26643376	3.616	3.56441654	3.73233458	4.1031841	2.95568593	2.77329567	1.71370112	4.07380439	3.95524443
C14orf93	4.42279017	3.4046107	4.26346713	2.52760231	2.9598452	3.69210739	3.21127437	3.55979335	3.341673	4.7106739	3.02105488	4.39873084	2.61530777
PSMB5	5.34520789	5.15881034	5.45566157	5.71608193	4.92260814	5.55691558	5.01181635	4.922159	5.33766436	5.4414162	5.03542006	5.56971761	4.93690938
YY1	5.17054681	5.46056879	5.00163847	4.82848221	5.46541581	5.24728501	5.77799073	5.52507328	5.85098501	5.45001472	4.88810337	5.4801765	5.9924007
ACIN1	7.07210088	6.35636268	6.82668532	6.82861591	6.44944825	6.05932597	6.79210646	6.36188225	6.39288017	6.80935622	6.39053716	6.33425613	6.90872925
CCNB1IP1	4.92099418	5.00585299	4.22762929	4.92169449	5.58048343	5.40802352	5.91377082	6.02320699	5.58231061	5.57307971	4.96412181	5.32738977	5.41152658
TRIP11	5.84557269	6.38795772	5.74798561	6.56130801	6.48129627	7.15098618	6.54182292	6.29389777	7.14152428	6.05744021	5.82457715	6.18931198	5.80275374
APEX1	6.70159791	6.19047832	6.45271141	6.53874026	5.52631421	5.80017668	5.93715577	5.59648719	6.07092914	6.15179485	5.35592177	5.99832787	5.97347397
PABPN1	5.80331046	6.0166751	6.52298285	6.17456425	5.90195009	5.27592603	6.02491465	6.07299688	5.73112397	5.93574408	5.69547019	5.88223515	6.12885996
EFS	5.41527085	4.37206648	5.51580637	4.25804444	5.05128933	4.63461441	4.95941605	4.7609051	4.84436536	5.54620201	4.83001455	5.21019016	4.26532007
ARHGAP5	6.47340758	7.50970831	6.60658258	7.42831574	7.70975793	7.97497356	7.76418011	7.83984366	8.08061044	7.22033643	7.29740358	7.78078388	6.83555897
CINP	5.15116311	4.56322017	5.03284958	4.07534237	4.5720416	4.91797094	4.37248862	4.45483951	4.77233857	4.43058311	4.8028705	4.2412957	4.7864163
DHRS2	0.05894057	0.1176737	0.1240001	0.05144055	0.11328934	0.1233062	0.19238829	0.03672203	0.00392893	0.00392893	0.00392893	0.00392893	0.00392893
SRP54	5.51410914	5.88589444	5.60321587	6.41614595	6.07931411	6.42619355	5.9499741	6.0619155	6.36290426	5.91511928	5.78960225	5.81675186	5.68865969
CPNE6	1.22147525	0.41383921	1.44850139	0.41383921	0.80683456	1.48222655	0.63147466	0.84575832	1.38397842	4.66388786	-0.17733625	3.23764242	-0.17733625
CHD8	5.83259522	5.58801997	5.20351688	5.42104193	5.37368808	5.60341833	5.14192924	5.2871745	4.8823598	5.19443782	4.94245562	5.35690044	5.3639497
PCK2	4.49920804	4.01052428	4.68999014	4.02735235	3.38064552	2.92210577	3.41205027	3.85701479	2.85379203	3.03427472	3.95438789	3.68196802	2.9635557
KIAA0391	4.48570796	4.29846174	4.61556115	5.47887404	4.86448647	4.81700466	5.00473213	4.73673448	4.55248063	2.61945575	5.10438866	4.98031809	4.0053572
DCAF11	5.95451764	6.07162948	5.86393392	5.02945334	5.37986421	5.59292543	5.8158455	5.44231134	6.0461956	6.24884437	5.89661759	5.86570656	5.98658161
PSMA6	5.03483157	4.97888572	4.9056372	5.01823932	5.61185306	4.64358666	5.02710066	5.25976598	5.13744861	5.06399832	4.68043454	4.84177854	4.64424402
NFKBIA	7.25406148	7.52349534	7.14281234	6.25673436	6.65429165	7.6993367	7.22131893	7.48554295	6.42429148	6.81851944	6.00231049	6.52263453	7.28965802
PSME2	3.61845942	4.14702449	5.15607991	4.64632042	3.30038444	3.39789525	3.36807112	3.07329639	4.82570687	5.20502303	3.85304574	3.42850397	3.737636
BRMS1L	4.41445632	4.39707266	4.74017508	4.79274068	5.55474782	5.41783414	5.26722524	5.30356382	5.00809704	4.98731667	4.05971818	4.93009798	5.17684911
REC8	2.56525657	4.73077233	2.47927184	2.81173805	2.12164821	1.40759515	1.35768823	1.82495091	1.42384853	3.36102135	3.71493169	2.34608871	3.97264049
TM9SF1	4.77009097	4.83349623	5.16492827	4.49457394	4.48256496	4.42671764	5.26994292	4.95861172	4.30021811	4.34601472	4.69639479	5.06227303	4.59449495
SEC23A	6.41115192	7.0921889	6.44154471	7.64187426	7.51837734	7.43696287	7.473446	7.47245649	7.50287736	6.84773633	7.74469223	7.44862174	6.9812201
GMPR2	5.94151412	5.85822359	5.83149768	5.70423608	5.31668716	5.8254232	5.64258784	5.12237918	5.70325643	6.16722597	5.05372977	5.80172865	5.83549134

PNN	5.66032583	5.92009356	6.31915802	6.23291713	6.25314968	6.83631238	5.72510603	5.59840635	6.95434564	6.50723107	5.6985248	5.79639157	6.00798935
RABGGTA	4.36757389	4.36148994	4.98119144	3.89751333	3.85663911	3.89789919	3.73388539	4.12093126	4.54721023	5.1429014	4.55293217	4.54403432	4.45537946
NFATC4	5.76054519	4.65022819	5.91440438	4.60135784	5.07862558	4.60836321	5.49840405	5.41715433	4.33468047	5.18102988	5.14702337	5.30851548	4.87135059
PLTP	7.35881591	6.10593015	6.65030133	6.41197692	4.79259143	3.84404438	6.08257954	4.68829062	4.69024697	5.57197855	5.67307003	5.37377704	5.88863969
PCIF1	5.9226326	5.45454086	5.94770189	4.61407609	5.35693445	4.92411418	5.07790855	5.50964041	5.27000645	6.09903118	5.06276745	5.55891863	5.55328536
GSS	4.41382482	4.42203894	5.00991467	4.78397367	4.13875424	3.96391856	4.10894126	4.21728249	4.35582022	3.8513184	4.58410522	4.63840485	3.98890644
MMP9	2.66496731	3.00940616	3.28499105	4.22275036	0.40572936	0.39471188	0.24907638	0.24455146	-0.25118362	-1.2596977	3.10886781	-1.2596977	2.04106719
TRPC4AP	5.92586016	6.10063143	6.2101695	6.44796033	5.93040623	5.23786245	6.14373329	6.3127509	5.46912597	6.06753177	6.37787724	5.92797087	5.77691278
PYGB	6.65883104	6.58956886	7.30798291	5.68922139	6.85490358	7.39802473	7.49264533	7.62586178	7.03602164	7.97876734	7.44164331	7.81878703	7.39255347
ABHD12	5.11579891	4.64354506	4.95069118	4.63642583	4.3615556	3.99671016	4.00798049	4.76221386	4.53757167	4.95231171	4.83487565	4.57012501	4.82951724
PROCR	5.01460677	5.97238656	5.18388802	4.98993292	3.81030051	4.08767665	3.84521801	3.33535499	4.13120949	3.62571927	4.33447466	3.49195698	4.10066452
GINS1	2.8003409	2.04240245	4.18147997	4.52587706	2.03415722	2.14869845	2.56295674	1.44024375	3.21086037	1.9388524	2.29073923	1.99584073	2.42773729
NINL	5.28796589	5.01736112	5.45715231	3.7431491	4.78036664	4.99013688	4.89573219	5.41669244	5.02113259	5.34575203	5.274946	4.96381288	4.85356588
CD40	5.49939994	4.64109182	4.24643571	4.74473955	4.64357298	5.39703916	4.98357609	5.1401012	4.56672388	3.93834363	4.74217185	4.27994683	5.11789062
ZMYND8	6.28565162	5.80319957	6.17010581	5.57083275	5.32094949	5.745062	5.35590084	5.43912038	5.77017825	5.8796044	5.03736759	5.14267758	5.67443398
SGK2	3.55215003	1.30734195	2.49933198	0.63220851	2.53957655	1.02646393	2.39726543	2.41457652	2.48906385	-0.67870532	4.11047787	2.86636887	1.85881031
IFT52	5.16404434	5.17731441	5.1645754	5.00147239	4.63112712	4.9140068	4.53741693	5.32820681	5.2680099	5.65782114	5.25652577	5.2668717	4.72191179
MYBL2	1.67130182	0.40455405	3.67805063	4.35196493	0.04553757	0.11146113	1.50354399	0.38318398	2.38600593	-0.59814667	-0.59814667	2.59908467	2.11707691
NDRG3	5.98291401	6.08564529	5.65347666	5.24677621	6.52406849	6.76697079	6.59738829	6.70160243	6.30801741	6.84818328	6.50014274	6.50950585	6.49188151
SLA2	1.73961297	1.99993972	0.47998911	1.0388253	0.68548059	0.78374491	1.36509781	1.08117126	0.46773864	2.74037601	2.2534984	-0.27137645	3.06197168
C20orf24	3.33592122	3.13996292	4.51437505	3.81444025	4.08853399	3.80381609	3.38739252	4.45200689	3.5703257	4.32465781	3.11401477	4.18650553	3.63280202
NFATC2	5.00163171	5.72366848	3.79302838	4.85655491	5.21892375	4.02319894	3.73495232	4.17675827	5.0208072	4.24411404	5.81004939	3.9505276	5.77667139
RIMS4	3.97608625	3.212555	4.36264631	1.55821663	3.69041544	3.20956592	4.28817015	4.19675534	4.8239588	5.25205903	4.1149525	4.65337642	3.95120953
PABPC1L	5.07348835	4.12568761	4.7121398	4.37488245	4.57888002	3.0932607	4.51075482	4.35210761	3.78658817	5.37886246	5.06859757	4.00611949	4.03647555
STK4	6.51130639	6.35867585	6.0923246	6.94516355	6.37622838	6.20704735	6.28295832	5.79188515	6.27483964	5.7428628	6.26232711	5.73881747	5.92686448
SALL4	0.3120987	0.30147433	0.71432166	0.94355285	0.38637447	0.30290265	0.33398043	0.54275169	-0.1292151	-0.1292151	-0.1292151	-0.1292151	4.09245042
ADNP	6.71155637	7.10560903	6.2545697	6.94251987	6.92065534	6.99839182	7.10937255	7.03781658	6.98648912	7.21197024	6.76049003	6.95519598	7.16535084
PFDN4	3.98896279	4.19989856	4.82650975	4.38043528	4.60756719	5.49154143	4.43388228	4.70594186	5.84375864	4.02096806	4.35488924	4.59986509	3.51615615
DOK5	5.65828116	3.70024827	5.20683801	5.65551954	3.89735869	4.30864117	5.05907302	4.3961384	5.08927805	3.49153244	5.28181823	4.34690748	3.43317383
CSTF1	5.07693934	4.90393902	4.55969433	4.87346943	4.87202195	5.33932268	4.42432253	4.18308847	4.00401507	4.5537827	4.21420647	5.21209545	4.77258328
RAE1	3.64844113	2.26228895	3.60274154	3.80615552	3.51613094	3.60470365	3.51334985	3.2310708	3.62880523	2.08869548	4.15082125	3.66095919	3.41307555
TPD52L2	5.43429077	5.49417028	6.00887883	5.42370051	5.07798106	5.01522276	5.40229241	5.3283382	4.89301327	5.84438256	4.70294362	5.68940792	5.35127805
DNAJC5	1.3311878	1.39556752	2.01097977	1.3930803	0.72886134	-0.0832071	1.16698577	0.4984378	0.74830528	0.74830528	0.74830528	0.74830528	0.74830528
CTSZ	8.48037695	7.77942436	7.96741688	7.86541305	7.32114807	7.0785937	7.02540212	6.95877674	8.12749897	7.60557071	7.78813216	7.36982726	8.01416605
PRPF6	6.6509281	6.4991433	6.95979547	5.79971714	6.17498879	6.55486907	6.17125927	6.19275978	6.50012579	7.22229204	6.49398126	6.68461719	6.48843405
TUBB1	1.19425122	1.09354313	1.09613244	1.56202728	1.76031109	1.09498275	1.28579454	1.09684649	3.13387024	-0.57390753	3.40671388	-0.57390753	3.2071289
PSMA7	5.71619732	5.82183411	6.18654654	5.50600346	5.22345653	5.71952606	5.06776322	5.34120309	5.45499935	5.91289147	4.88727141	6.02496482	5.50823922
SLCO4A1	0.16580811	-0.30565927	0.81961154	-0.07361699	0.79973077	-0.16254034	1.38454674	0.01231749	0.1555504	0.1555504	0.1555504	0.1555504	0.1555504
NTSR1	0.44131227	0.05647243	2.66749692	0.44628605	-0.09377468	-0.09255418	0.00522158	-0.09097412	-0.26549361	-0.26549361	-0.26549361	-0.26549361	-0.26549361
TCFL5	3.96324354	3.82414971	3.94292985	3.73677321	3.44353809	3.79917124	3.37951905	4.55753332	3.99443353	2.94488012	4.35948805	3.96662222	3.10655254
DIDO1	7.06792213	6.58138595	6.96096035	6.28689431	6.76863928	6.4759807	6.73123196	6.51246472	6.35978909	6.68748686	6.59473165	6.73798841	6.60942084
SLC17A9	2.46366075	1.46798957	3.9451582	4.30853158	2.36495804	0.42004641	2.79327419	2.90105299	2.79253311	2.79445755	3.35421654	2.19863438	-0.94112331
BIRC7	0.44619045	0.07598071	0.00124472	0	0	0.00069205	0	0.00158797	0	0	0	0	0
ARFGAP1	4.26792112	4.28873952	4.96475769	4.95745753	4.22065268	3.38137249	4.25673968	4.45653829	4.4972567	4.7603541	4.53010061	4.27703576	3.68811791
COL20A1	0.14227227	0.24978342	0.2206143	0.15045189	0.26793491	0.14371884	0.18046944	0.22572185	-0.01863429	-0.01863429	-0.01863429	-0.01863429	-0.01863429
EEF1A2	0.29783862	1.60109662	0.44409395	0.66829581	0.70851703	0.60068161	0.24773586	1.49953025	-0.12627543	-0.12627543	-0.12627543	2.68924275	7.51829985
PTK6	0.20087574	0.2319863	0.22951237	0.45313925	0.05778911	0.15006835	0.30076003	0.21320434	1.6806629	-0.07333236	-0.07333236	-0.07333236	-0.07333236

GMEB2	4.99055699	3.85157781	4.84318708	3.67355644	4.10907475	4.12958285	3.87757556	3.75031772	2.84043547	3.84013252	4.13888074	4.13790586	3.66630207
C20orf27	2.99631271	3.85464513	4.55538725	3.56402619	2.28186969	1.63574457	2.7812304	1.08016984	3.69295416	2.91739863	1.36388128	1.8325217	-1.03635738
CDC25B	4.79400006	4.28475045	5.66904417	5.05789443	4.45985064	3.77472282	4.75688027	4.96598075	4.40494272	4.47841232	5.45823185	4.90073573	5.11546908
ISM1	1.66949113	-0.89071828	-0.28890238	-1.41140003	1.84308759	0.90807331	0.53982523	3.50016632	0.20257612	0.20257612	0.20257612	0.20257612	0.20257612
RNF24	7.84007795	5.85055413	7.55154722	6.16924745	6.40416759	6.25618818	7.15078941	6.2486826	6.15225046	6.36959553	6.48573523	5.52570963	5.7604659
ARFRP1	4.85585189	4.29202466	5.37788121	4.15684223	4.73084214	4.07932771	4.72838297	4.85469492	3.85766174	4.83009557	4.3464723	4.84164706	3.99240199
SEL1L2	0.16079018	0.87705409	0.16334847	0.16079018	0.62173454	0.16221255	0.16079018	0.46457478	-0.17739837	-0.17739837	2.86892868	-0.17739837	2.98841596
TRIB3	3.20898041	3.95941188	4.60861074	2.72577827	1.68888892	1.93253571	1.68754051	2.44378101	2.56956548	1.80332689	2.97860897	2.75463324	3.08796635
RASSF2	5.92634255	5.8580409	5.87217227	6.26191179	5.66911829	4.9620926	4.90487846	5.23755107	5.85012376	5.34148914	6.23339695	5.24227621	5.87075639
CSNK2A1	6.01933449	6.24566482	5.84398239	6.21814051	6.36244985	6.57667411	6.36000684	6.17713045	5.88103586	6.3849402	5.98739999	6.19182195	6.21698144
ANGPT4	-0.13449056	-0.25399316	-0.12233596	-0.08385505	1.04170987	1.92621083	-0.0983758	0.84401356	-0.1647918	-0.1647918	-0.1647918	-0.1647918	-0.1647918
RSPO4	0.21574143	0.43985506	0.21830592	0.67685834	0.21574143	0.21716725	0.92361581	0.55993484	1.95886012	-0.19623116	3.60641086	-0.19623116	-0.19623116
CDS2	7.10610206	7.26123136	6.70392275	6.25587171	7.64612794	7.52561709	7.82083958	7.43242007	7.04797931	7.6135747	7.36576949	7.61586568	7.8991936
HM13	5.71916733	5.08360717	5.50364081	5.96557678	4.889928	4.78827596	5.04702087	5.01825612	5.16415818	5.78390731	5.34225619	5.44140631	4.9174769
SNPH	4.58012912	3.73296501	4.48992585	2.95894812	3.66651087	4.17751463	4.5336696	3.92480073	4.00998426	5.22893988	4.29126794	4.90825727	4.66151662
MYLK2	0.00830368	0.36245129	0.33172944	0.35170754	0.05401412	1.14971073	0.16427331	-0.04702068	1.48635095	-0.09288272	-0.09288272	-0.09288272	2.68034048
SIRPB1	3.95380528	1.70419989	3.27097607	4.09151089	1.67291691	1.84160516	1.87041443	1.41626248	1.29504954	2.71675524	1.73622279	2.5199464	4.01903091
SEC23B	5.70700017	5.74127407	5.69593079	6.21442133	5.17163642	6.00739818	5.22113917	5.34598024	5.20918171	5.55039544	5.31050171	5.51012339	5.28091336
FERMT1	2.2072537	3.19469557	2.26319512	3.67798108	1.81287626	1.87180948	1.92052056	2.24290464	3.73916438	2.37763274	4.39340359	3.3106839	4.28658638
PLCB4	5.32137008	5.19998272	4.19866119	4.38363839	6.39847605	6.22884472	6.09200095	6.97780422	5.87528827	5.89744866	6.15547962	6.06747542	6.52623184
MYL9	8.75843917	9.27249729	9.00311061	7.57088593	10.1024477	10.1647199	9.50733839	9.40988348	9.1410255	10.1565852	9.27199854	9.79915523	9.19410571
HCK	4.78592418	4.54058585	5.17830669	4.94659024	2.95692702	2.23431784	3.23394819	2.22073279	4.22893685	4.22719379	0.87404494	3.21858366	3.81422431
TM9SF4	6.19087833	5.64367739	6.19218308	6.05477754	5.58996593	5.56543543	5.5922262	5.2950251	5.39782596	6.36445526	5.71578097	5.03352546	6.12215678
CRNKL1	5.40766088	5.40716879	5.32325567	5.54449345	5.87513584	6.31897383	5.5847958	5.83504593	5.84378382	5.62032215	5.70471255	6.08516845	5.37361207
POFUT1	6.13018466	6.58157258	6.42816451	5.96856449	5.89358828	5.54157309	5.81334948	5.44735015	6.15476785	6.44318919	6.41755344	6.3283488	6.18504995
SAMHD1	6.8806691	6.99417392	6.53302554	7.81317144	7.08407371	6.87682412	6.53144308	6.28591723	6.25922354	5.49490124	6.41471668	5.7950631	6.93114116
KIF3B	6.23312981	6.42218487	6.19520084	6.53709788	6.09984198	5.54553016	6.16050957	6.11886686	6.24005048	6.41816362	6.43146741	5.87050715	6.1790821
NOP56	4.25197465	3.63990704	4.41159655	4.52783743	3.12627651	3.66249785	3.22192835	2.94949641	3.39247923	4.99667338	3.27022403	3.53766495	3.59458874
MANBAL	6.02477104	5.7048137	5.46719064	4.54058619	5.94727715	5.90185758	5.48540313	5.72096011	5.78758548	6.48802118	5.78445752	6.22195364	5.38882419
IDH3B	6.1487388	5.54929877	6.10854573	5.38712942	5.72238776	6.05021665	5.94237242	5.35704863	5.53442598	5.92029308	5.56706108	5.53910848	5.96560434
MAPRE1	6.30549747	7.00371292	6.32156418	6.87972399	7.05165568	7.23949357	7.03075873	6.9184364	6.637038	7.26222556	6.87411851	6.9356726	7.22930472
JAG1	8.11981015	7.42508172	7.62268009	7.43631216	7.47226328	6.9049426	7.97472657	6.71708939	7.51088473	7.50598333	7.92933053	7.59642979	7.33486651
CDK5RAP1	4.46221378	4.20294599	4.53587009	3.85344927	4.62811667	4.02732551	4.46291743	4.58674116	4.39115966	3.58126069	4.50121555	4.70283439	4.56101885
SNTA1	5.0699036	5.2192219	5.56986725	3.37178753	5.47322603	5.62988501	5.65399027	5.78712902	5.05699266	6.67698714	5.7761247	6.02565856	5.10785534
TTI1	4.93436174	4.16452632	4.98158679	4.76201942	4.80390399	4.7885745	4.9260167	4.48455652	4.95429923	5.25206557	4.42161333	4.43502447	5.0153425
E2F1	2.19124489	1.56933142	4.74099642	2.79239945	0.84132195	1.36332281	2.74293497	1.27165696	2.93775268	-1.12287573	3.14937197	2.47850941	2.1214976
RPRD1B	5.66818475	5.87817052	5.6182546	5.74820744	5.66220254	5.6395716	5.67344736	6.30164486	5.5367084	5.63740938	5.59669042	5.71231795	6.2153155
PXMP4	4.96478626	4.36178213	4.55382882	3.69819702	4.13172271	4.51406548	4.29005374	4.14762432	4.77839096	4.50006721	4.00289217	4.09748793	4.42035839
CHMP4B	7.58370512	6.91701007	7.09686924	6.32115899	7.17184086	6.9863551	6.4904224	6.44586931	6.93857169	7.01701872	6.48733904	6.57084103	6.79242907
BPI	1.68501222	0.56069889	0.78935585	0.91353634	0.24529437	2.02905559	1.64288659	0.3833305	1.28304615	-0.43964584	-0.43964584	-0.43964584	1.74937651
CST3	7.67395588	7.39472266	7.18700413	6.40612589	6.81708946	7.56835827	6.26839607	7.08995656	7.20670468	7.98454293	7.54111083	7.54938774	6.83880965
ACTR5	4.39998776	4.45971367	4.16396149	3.79179596	3.88068538	4.06273286	4.29438927	4.86261402	3.69774582	5.14920749	4.52309666	4.74759896	4.42816188
WFDC2	1.13699158	2.76828657	0.74190679	-0.01991286	0.16062856	0.71323649	-0.01991286	-0.01721679	-0.21086101	-0.21086101	2.18676341	2.54897991	-0.21086101
AHCY	5.31340851	5.48193196	5.94343784	5.36545381	4.5279937	4.49461491	4.5660663	4.32513065	4.7014202	5.46100933	5.17215878	5.72049537	3.70124561
PPP1R16B	2.72307747	5.04804895	2.7826488	3.72131011	5.12708935	3.01348337	3.66794449	4.15563437	4.37291604	4.68502979	5.64741676	4.74635036	5.48528772
FAM83D	0.4418505	-0.12827127	3.6264361	3.1791031	2.7863905	1.52092648	0.54640933	4.15403367	-0.98038424	1.7696854	0.21596442	4.68568763	5.7140974
DHX35	3.55017175	3.68779592	3.23804721	2.59544016	4.32798522	3.77360286	3.66799599	4.20763616	3.39317978	3.85889926	4.3263902	4.011268	3.29812476

DNTTIP1	5.00897059	4.6994664	5.30322869	4.46692014	4.03557875	4.08039606	4.30873082	4.32718467	4.62327147	4.48093258	3.50001939	4.72372262	4.37005948
MAP1LC3A	4.05506588	2.95887781	4.2069347	2.05003343	2.94179701	3.74218592	2.4787996	3.7587331	3.56018549	3.03576642	3.69495803	3.75015253	4.3607622
SYNDIG1	4.29247704	1.84617725	3.84078441	3.21103582	2.88726201	3.14856183	3.92556165	4.29178088	3.17990109	4.20069078	1.67777295	3.48727639	3.91334014
PIGU	4.44703772	3.76482946	4.5670876	4.90194772	4.42590203	4.94559375	4.09529916	4.27227048	3.67457178	4.88136297	4.899123	4.35911619	4.86820164
TNNC2	2.87273269	0.7316199	3.2805115	0.64884817	1.57883191	1.92719142	1.64918919	0.44628405	2.81451067	3.12741051	2.40021704	2.33065838	-0.4704644
ACOT8	3.85564173	2.9587267	3.79536112	2.97293502	3.41787566	3.48558691	3.4806232	3.87593491	2.93800415	4.19389114	3.86618965	3.06477833	2.44655069
ZNF516	5.85278771	4.6063625	5.4360058	5.0456773	5.14735231	5.27090524	5.41292447	5.31029328	4.60816773	5.57036992	5.6656141	5.25166351	4.93356075
CDH20	0.01908435	0	0.05218883	0	0.02998317	0.00069205	0	0.1734345	0	0	0	0	0
ADNP2	5.89590511	5.82592229	5.41574982	5.59936621	5.60270516	5.67879385	5.07677542	5.41543768	5.27104935	4.99819227	5.92848427	5.4783258	5.43804224
RBFA	4.40029626	4.11839799	4.44523702	4.46745307	4.90629955	4.38884782	4.83451122	5.30285653	4.25685354	4.08593709	4.61415347	4.97844949	5.04742493
USP14	5.28072905	6.25004986	5.762226	6.74412868	6.68254682	6.46847241	6.75233255	6.22435556	6.40454369	5.76696806	6.66297627	6.31215853	5.94766754
VAPA	3.91285779	5.12376169	4.32484703	4.95770557	5.61143895	5.56715438	5.35581551	5.63436425	5.43100283	5.36566436	5.35165203	5.22590752	5.02295185
METTL4	3.49113186	3.76403541	3.81523133	4.68040452	4.51975998	4.70588447	4.85009603	4.66382274	4.87913043	3.83298054	4.39277128	3.36677842	4.26441
LPIN2	5.93164868	5.87857414	5.66682704	5.92469111	5.5996555	5.69935403	5.25750587	5.53937309	5.64616	5.57890302	5.87597898	5.71552267	5.56106182
SMCHD1	5.93639979	6.61189838	6.10058779	7.00870824	6.99185192	7.10277607	6.96521388	7.03188733	6.9921784	6.44292291	6.86856287	6.69375457	6.80138276
MYOM1	5.03568598	5.25054224	4.68208809	3.30352508	5.91618744	6.43090283	5.77045444	6.09631139	5.57900766	6.0917128	5.43313208	5.55688724	7.92283706
MYL12A	6.43170875	8.12105119	7.18889859	8.28581795	8.35158656	8.59011717	7.95913366	8.67040488	8.34662917	8.1699436	8.14802996	8.48728482	9.08144564
CEP76	3.56669812	2.85216301	3.07041985	4.0103687	3.60543646	4.11879643	3.77336553	3.80207789	3.38077177	2.2231834	3.90025427	4.1533284	3.78162084
ST8SIA5	0.47539535	0.34255302	0.41698073	0.2279464	0.19962362	0.27818563	0.37014142	0.35536872	-0.21460321	-0.21460321	-0.21460321	-0.21460321	1.4178741
CEP192	5.63134929	5.9585015	5.74323647	6.83283606	5.82711198	6.01826207	6.26517232	6.32856854	6.08199445	6.5256704	6.45567313	6.0989047	6.2886856
RNMT	5.56675316	5.83283567	5.24979146	6.11797451	5.94289649	5.94982371	5.72604486	6.0102698	5.87734983	5.98814311	5.79324356	5.71071666	5.8200218
SMAD7	6.71780474	6.6229741	6.65625573	5.75752219	5.40844667	5.43549844	6.15127367	5.28437667	5.43265	5.93213572	5.58718544	5.89298841	4.80143563
LIPG	1.06477414	0.9083357	1.916773	2.9104283	0.94229407	0.76658124	1.66245517	0.98094717	-0.66384997	-0.66384997	-0.66384997	-0.66384997	2.8891071
LAMA1	-0.05508728	0.60284489	-0.08820707	0.39325993	0.0013279	-0.11864707	-0.13583243	-0.04464759	0.00706227	0.00706227	0.00706227	0.00706227	0.00706227
RNF125	1.33905901	1.77004761	0.74403873	2.02265095	1.74861109	0.93189005	1.27254864	1.41145933	2.33290239	-0.68151667	2.79937284	-0.68151667	1.48470668
ANKRD12	1.90838387	3.38288952	1.7353526	3.38119643	3.85335939	4.25477018	3.18018485	3.83640574	4.43395893	4.34820179	2.14261871	3.9961113	4.082643
NOL4	1.74982621	0.99318653	1.35414722	1.01965524	1.34931296	2.11177508	2.21884046	2.05042489	1.0086685	-0.73715234	-0.73715234	-0.73715234	2.12929733
POLI	4.85916676	4.96436252	4.54914847	5.29779206	5.67411452	5.34943818	5.74454143	5.77308694	5.38381443	4.88979712	5.57514412	5.75261485	5.3605492
MIB1	5.79996599	6.18162791	5.91863398	6.25155529	6.62407643	7.00106598	6.81647897	6.95392115	6.00006936	7.24517033	6.1068073	6.51583212	6.90877806
RBBP8	0.05549968	-0.05358189	0.28937803	2.12778536	0.69959854	1.0085257	0.74975093	1.17618314	0.1230507	3.25534646	0.1230507	0.1230507	0.1230507
RIOK3	5.24472804	5.56853384	5.24207389	5.96768284	5.9130707	6.06914892	5.74988541	6.04297034	5.76398883	5.31158943	5.22105214	5.5838349	5.57842942
CSTF2	4.32805547	3.84640166	4.49641595	3.59577793	3.77400801	4.74109913	3.67273038	3.5170463	3.93762025	4.42517107	3.50273907	3.89093546	4.21297587
MXRA5	5.20900633	6.82896423	5.2882567	8.1427818	5.63160849	4.43015595	4.1117251	4.16311036	5.81821227	5.4194642	6.46240563	6.789969	7.08446642
VSIG1	0.27719609	0.06995532	0.07254027	0.32947444	0.06995532	0.07139252	0.06995532	0.07325311	-0.07511694	-0.07511694	-0.07511694	-0.07511694	-0.07511694
PSMD10	4.78189531	4.8524146	4.90945211	5.80255033	5.46433335	5.54400473	5.06993053	4.79425454	4.9985647	5.48567067	5.23091174	5.75874299	5.12265477
ATG4A	4.83363989	4.45483905	4.31411963	5.1617579	4.78246085	5.26253044	4.76479754	4.33433146	4.95444383	3.7399149	4.3301523	4.22085165	4.93754925
STS	5.31517747	5.6013118	4.87442982	4.48834274	5.71230208	6.21342015	5.16750109	5.40013108	5.83305504	5.53872786	5.48219004	5.55283406	5.67701621
TBL1X	6.62056106	6.33763054	6.44324104	5.56361467	7.18215885	7.3729043	6.90543349	7.19250001	6.56636415	7.56907053	6.7274316	7.25314898	6.85760606
GPR143	0.93507893	0.66711777	0.05116398	0.24813617	-0.29839424	0.11487389	-0.13586529	-0.29557791	-0.08387667	-0.08387667	-0.08387667	-0.08387667	-0.08387667
PGRMC1	6.0014812	6.40816657	6.31673565	6.88583508	6.91494835	7.33090008	7.16449313	7.06585513	6.82868337	7.09820832	6.75169183	7.29948298	6.75954163
POLA1	4.11754517	4.76527029	4.50301278	5.08002981	4.69345115	5.11374474	4.78595001	4.58854207	5.08161638	3.92286953	4.76698103	4.44355124	4.39803481
MID1	6.49343074	6.41675567	5.59994045	5.71371546	5.33876798	6.21516555	5.58205106	6.33451541	6.24404737	6.22683402	6.23928607	6.01416619	5.96192832
NKAP	3.99087183	3.57243557	4.35842907	3.3011034	3.82501822	4.33901026	3.69491999	3.87939445	4.50122334	3.71984786	3.65222242	3.18671613	4.44020593
NXT2	2.9222896	3.29123498	2.91870725	4.60457048	4.22563138	4.12820436	3.53513284	4.0517207	4.17271259	2.20455607	4.3978359	3.69673375	2.36132886
GUCY2F	1.22497945	0.88231973	1.99836451	0.91411786	1.01663793	0.8837458	1.14561338	1.2534597	2.82042256	2.86796449	1.9053654	3.95935523	0.830751
ALG13	5.68096058	5.91821051	5.16307301	6.49908636	6.34720534	5.79275285	6.08173409	6.16290286	6.20130588	4.78638724	6.53342335	5.77790262	5.67145016
PRPS2	5.08115456	4.14839454	4.41681771	5.6483549	4.9587848	5.34759021	5.15538062	5.13071529	4.76479721	4.27943451	4.89576041	5.40221254	3.4336647

TLR8	3.33191978	3.82385771	2.20423127	5.57845332	2.66854306	2.42628982	2.96109273	2.36493355	3.61380272	2.52219509	2.89412353	2.58375069	4.48465553
MOSPD1	3.23024101	3.75171359	3.26602876	4.39140651	3.72094494	4.75802914	4.17935847	3.91773129	3.61940733	3.22814377	3.56668368	4.69850924	3.38404387
AMMECR1	3.07313276	4.17422113	3.51842821	4.79423834	3.86971846	4.54030332	3.78272853	4.15169598	2.82363429	3.07392249	4.19798382	4.42341745	3.92957192
CHRDL1	0.91901131	7.04393455	0.50250782	2.75201084	5.97003367	4.27413061	4.64926534	3.99266283	6.13945793	6.36311288	6.51020648	6.20859639	7.00764818
WDR13	6.17409874	5.88313293	6.51039515	5.25657903	5.71169052	5.18823286	5.87593255	5.91922791	5.70015135	5.90672031	5.7609521	5.75861248	6.68791353
SUV39H1	3.36906015	2.23940421	4.74644556	3.05907021	2.59432508	2.47423398	2.45757263	2.92346304	3.73254291	4.25952873	2.61064503	4.02487111	2.52615271
SRPX	5.19604182	5.15056632	5.85035474	5.22230654	3.62879809	3.80947651	4.86328892	4.86244608	4.79316022	5.09126784	4.90250894	4.99851202	4.92433565
XIAP	2.35959164	2.62491226	2.92878368	3.26687242	3.72638461	3.60897984	3.51408855	3.44995827	3.18677951	4.3791177	0.94708162	2.85580162	3.42299607
STAG2	6.27814865	6.69286206	6.25106363	6.97586037	7.01204797	7.22442541	7.18122885	7.31412332	7.06245195	6.94829732	6.84939234	7.23195328	6.87731945
ATP11C	5.20830298	5.71998819	5.24620746	6.70687448	6.19256799	6.18529818	5.8801027	5.9539291	5.8475381	5.01306859	5.94838816	5.36506192	5.34262814
MCF2	3.47126334	4.06405602	3.64461259	3.25084528	4.42059329	5.65104855	3.8064153	4.19266616	5.06291809	5.37226111	4.24164947	4.36844453	4.00782966
ABCD1	4.6558026	3.55236957	5.20876813	3.85824832	3.99168164	4.36570949	3.97194595	4.35544527	3.9113762	5.14225125	4.75230224	4.88251718	3.4766955
CCDC22	4.25558492	3.62183842	4.6995509	2.95277347	3.08973646	3.23638469	3.51106994	3.47468232	4.03317988	4.37199464	3.21694938	4.29685047	3.97692729
CACNA1F	0.32218577	-0.09002014	0.27213096	-0.03789187	0.09752903	-0.04694708	0.151623	0.42475911	0.04159256	0.04159256	0.04159256	1.63990653	0.04159256
SYP	3.35393643	2.89598809	3.18086198	1.91982582	2.69236219	4.61574264	2.6648319	2.71708473	3.4439466	3.87000715	2.43229264	3.75086585	3.88923215
PLP2	7.59250686	6.63709482	7.17202691	6.46881365	7.1171258	6.48357948	6.63355341	6.65406805	7.20838788	7.54035558	6.96635606	6.39440678	7.23412485
BMX	2.69029415	3.85415944	2.61310172	2.49595091	3.12514726	3.19053841	2.6993339	6.17913391	4.8244278	-1.26107421	4.14224367	2.71218646	4.56664713
PLS3	7.75970581	8.76862284	8.43783953	8.69426439	9.52399116	9.54979658	9.52399116	9.6700034	9.44510901	9.27958932	9.55001475	9.44510901	9.09526411
NAA10	4.49547475	4.81176207	5.42670905	4.00682262	4.49547475	4.01693505	4.40191594	4.42184921	4.91584466	4.96308459	4.41509882	4.67288607	3.34689127
RENBP	3.43365892	3.12261463	4.76485094	3.09518743	1.59724473	1.53010188	2.5336206	2.0721656	3.54448773	2.71337516	2.96051542	3.41408653	-1.08385283
ELF4	5.13618498	5.07770058	5.35738154	5.46532329	4.51651112	4.13486544	4.21853009	3.92152994	4.79891739	4.76546982	3.11629432	4.00243784	5.27866186
SMARCA1	5.9219982	6.15477066	6.27450117	6.78128505	6.63910167	6.96224842	6.70766956	6.81219166	6.99530314	6.74180177	6.43159884	6.49461165	6.29680603
MTMR8	1.63990295	1.19944722	1.51393674	1.21684169	3.09410878	3.52941192	3.01749134	3.26725675	4.17955829	-0.74028607	0.32735009	3.53705296	3.55750044
ASB9	0.91535604	1.83337321	0.74361652	1.39893443	1.00214607	0.37396881	0.99771615	0.91054223	2.67925371	-0.70224754	2.50094803	-0.70224754	-0.70224754
ZC3H12B	4.54984392	4.05923159	4.01743133	3.73048422	4.4063118	4.61119166	4.19494511	4.62930939	3.9720456	4.09325843	4.33069299	4.54924921	4.30170185
RBBP7	5.92461589	6.00304857	6.19632966	6.54315343	6.48949085	6.71804012	6.63761951	6.84977331	6.52474524	7.07168666	6.57686114	6.5618623	7.00376061
KCND1	4.428721	3.34277664	4.55648823	3.13725519	3.10533496	3.51028402	3.57029661	3.1146294	3.68428732	4.18807965	3.58474652	3.98826662	3.34592228
SLC25A14	3.97316364	3.29970064	3.65012383	4.16660593	4.47912728	4.49667025	4.85303544	4.59259454	5.0641409	4.7270862	4.78739875	4.56217607	4.21772985
FMR1	5.65193249	6.33951594	5.8216028	6.35047442	6.48156647	6.46409021	6.67920107	6.5359155	6.15596327	6.25798664	6.27675497	6.1844507	6.53853772
PIM2	5.23007132	4.43128336	4.40456828	3.85589186	3.72451089	3.33246398	4.15793355	3.82918156	4.21245825	3.11103259	3.53346769	3.7726926	3.32690558
SCML2	3.22072707	2.81575219	4.06004019	3.38689704	3.30392908	4.47212447	4.11333037	3.763956	3.61548522	3.03781717	3.27315786	4.72863768	4.28981025
SLC35A2	4.18277492	3.79676749	4.51963092	4.11192445	3.82938459	3.21796885	3.59579035	3.4505358	3.92499545	4.53633839	3.40036993	3.88773994	3.63372641
PQBP1	5.81441438	5.35342699	5.61785109	4.34441521	5.09400171	5.45724157	4.88171996	4.84686555	4.84080883	4.6853811	5.19326759	4.39809609	5.6842502
PCSK1N	0.67669072	0.04166983	1.79109078	-0.30834208	-0.1566193	-0.30700902	-0.30834208	0.54872204	3.70143916	-0.49520301	1.76566601	3.10181068	-0.49520301
EMD	5.42898882	4.8708635	5.37485052	4.3302661	4.75327238	4.86799017	4.24242275	4.90781305	5.15255856	5.39240914	4.8023335	5.23153393	4.43084869
TAZ	4.23829048	3.50808592	4.80868703	3.11687201	3.91396265	2.95498161	4.18836725	4.36223349	3.83831042	3.78509054	4.50934018	4.37106296	4.86828011
RAB40AL	1.68804178	2.25540755	3.04973357	1.58700204	0.70925536	0.17848331	0.90190819	2.22964984	2.85353963	-0.38119709	2.20037595	3.02971724	-0.38119709
PGK1	7.34644714	6.7839985	7.03450756	7.93697152	6.81742602	6.33506475	6.3621435	6.08260239	6.06414057	6.43862422	6.09108533	6.57296905	6.55019551
MAGT1	5.81949544	6.03100735	6.28819524	6.81377711	6.57293716	6.46973631	6.44738111	6.20608075	6.49283325	6.08557197	6.54103497	6.49706642	5.53204343
SMS	6.53095621	6.4481527	7.52945088	6.75512135	6.41492028	6.75329007	6.39700007	7.13890838	6.35042427	7.06642118	6.26066695	6.83597084	6.73672489
PHEX	1.40339464	0.58753627	1.55998427	1.4235573	1.25936014	1.9159676	0.65706211	2.28787186	2.64545313	3.01332999	-0.61745622	2.94026939	1.73203741
UBL4A	5.17418339	5.21028645	5.47844723	4.03258021	5.24075743	5.0688706	4.79195151	4.76540863	4.56035334	5.27621361	5.05450258	5.25257095	4.56098873
CD99L2	6.56828373	6.43483594	6.75325967	5.78899503	6.00628932	6.25841005	6.2959617	6.12024514	6.3483227	6.83268634	6.51309513	6.39964126	6.53249202
EEA1	5.67087333	6.30779477	6.1205062	6.83185072	6.71073453	7.38097319	6.66563889	6.69917163	7.37403504	6.35664156	5.95165162	6.52836918	6.50162488
RP2	4.4623067	4.47313619	4.1371722	5.19576376	4.55910311	4.5432973	5.09771388	4.9908615	5.07711585	4.47549026	5.02744238	4.44518091	4.47907278
CDK16	5.71996459	4.92824341	5.68998076	5.52391593	5.14051507	4.87583257	5.18096783	5.3322266	5.01390295	5.43009189	4.87979439	5.28376924	5.32956228
USP11	6.63944325	6.42801004	6.36003488	5.29383391	6.33950831	6.27589418	5.72995156	6.21364743	6.15416375	7.22756651	6.43176456	6.85731515	6.05638653

PCYT1B	2.80516013	2.74651304	2.91653022	2.96355305	2.30388524	2.65252691	2.55948359	2.02002905	3.23626067	-1.29175454	3.10459732	3.0935237	3.24804346
HTATSF1	5.57102782	5.9483179	6.79579567	5.85080837	6.03629573	6.60809352	5.6642887	5.8305338	6.74476263	6.20440754	5.74396688	5.93826798	6.174807
CD40LG	1.74541126	0.85215009	0.72856117	0.99054647	1.02790001	0.72741598	0.72598199	0.89183045	3.5237856	-0.56374719	3.79914756	-0.56374719	3.3169261
TIMP1	9.34741227	7.89988126	9.70811248	8.99183989	6.85456841	6.56682324	7.89988126	7.28941904	7.88438802	7.57924237	7.80835964	6.85218086	7.64282551
KLHL4	1.33579254	3.44285798	1.50097567	2.92941215	1.70458647	1.9305821	1.99769518	2.02554525	2.48259155	3.53059731	3.05105096	2.58039559	3.00594068
GABRE	4.16946399	4.10163242	2.42574716	3.52638035	3.51739732	3.10144152	3.24335002	2.63640403	3.99137874	3.37441696	3.50178302	2.25495415	2.85665961
PCDH11X	5.18067167	3.94067118	4.59865491	3.75505738	5.8992843	7.21552955	5.83331986	6.18747718	5.84866991	6.80968871	5.68223615	5.84153259	5.52360512
FGD1	4.88490808	4.17994815	4.90289318	4.62667912	3.9449392	3.95616233	4.0470059	4.23754229	4.19304773	4.99051067	4.12329044	4.68396041	4.33201656
PIN4	4.60320996	3.78254285	3.89603292	3.56947569	3.21274437	3.72515022	3.80419863	4.42128974	4.6467335	4.68600922	1.68706666	3.23828033	3.76667112
PORCN	4.60043186	3.17438575	4.0544279	2.48811169	3.65441525	3.24358866	3.35930957	3.67065889	3.40270101	3.90188861	3.18084725	3.67212681	3.12539688
MAGED2	6.33969484	6.33969484	6.86824928	6.56038835	6.21036312	6.97905464	7.11399117	6.42698114	6.48378817	7.67679821	6.92247548	7.50154332	6.24437658
RBM3	6.40923554	7.68581957	7.30912118	7.97489622	7.30064041	6.22117127	6.64125177	7.44169921	7.63120882	7.24320732	7.47371747	7.78432872	7.65069608
KLF8	2.93473246	3.16556843	2.50673689	2.46929749	4.21358729	3.77427102	3.76194687	3.73220329	3.66810274	3.20000685	3.66306799	3.37456088	3.63570815
SRPX2	4.7197555	4.46229876	4.19974024	5.38565896	5.5880983	5.18657725	4.41604063	5.92051176	4.6550993	4.51385385	5.60128183	4.98189083	5.62647356
SYTL4	4.63761277	3.74358246	3.94752705	3.99688305	4.60605669	4.47825109	4.67315487	5.44342405	4.011313	4.82110427	3.51196933	4.07090994	4.73505613
ZDHHC15	3.83221113	3.77713229	3.60490355	3.87195181	4.64783663	4.7986764	4.40749269	4.89919435	4.33935519	4.41104904	4.47283136	4.52130633	3.63357482
CENPI	2.13887858	1.52941347	3.76567134	4.32722474	2.37420108	2.8130373	2.93928818	2.32851328	3.26619549	2.73584413	3.63647945	1.76685572	-1.21899904
DRP2	2.06959726	1.86359177	2.1525084	2.2216118	1.95596253	3.58059086	2.38464508	2.18000183	3.313227	3.15874536	3.58838694	4.11872247	-1.11132729
TAF7L	0.63565339	0.47303287	1.08465558	1.0061732	1.41297103	1.14130228	1.46293236	0.6937267	2.17118685	2.98161539	-0.35714838	3.01649274	-0.35714838
GLA	4.31531701	4.23946077	4.22781808	5.28994215	3.60582457	3.95799417	3.87952737	3.51838734	4.50524502	4.43525468	4.06873668	3.69621193	3.65006451
ARMCX3	5.81076289	6.00481301	5.89331409	6.82450238	6.31530538	6.60722267	6.66922122	6.43927929	6.50390811	6.06641279	6.57209244	6.68168586	6.0450849
BEX4	5.21628155	5.37996691	4.78214875	5.62943531	6.15326066	6.28194613	6.15773362	6.37795583	6.25855187	5.79062624	5.67383766	6.27148849	5.18448496
NALCN	6.03857547	6.39765546	5.93587983	4.71119363	6.10579564	6.61525215	6.09774928	5.91318577	6.15652308	6.14208554	6.41943073	6.0582528	6.66776106
FGF14	2.94552488	2.51435385	2.73492868	2.25339482	1.8914759	2.67152912	2.55176375	2.68751516	3.41022181	3.52529936	2.44496621	4.25161668	2.94971796
HTR2A	3.31195021	3.36070467	3.23026304	2.88769163	3.39792673	5.69778359	3.63800792	4.74804915	3.75564807	4.49353014	2.46426027	5.39401461	2.96575368
NDFIP2	3.65312035	4.63346396	3.81524139	4.24523568	4.57226642	4.63515834	4.58384454	5.19787363	5.20472254	3.56324236	4.86643522	4.5557055	4.2392946
TNFSF13B	2.83519445	3.50787731	1.94696538	5.11305636	2.33527205	2.27971812	3.38142853	1.63526222	3.99743245	3.05493671	-1.14092568	3.58728717	3.51512596
FNDC3A	4.3853205	5.29134862	4.00779742	5.63957568	5.37304856	5.65169106	5.32353545	5.2925195	5.05289	3.4512986	5.18781331	5.258217	4.67262406
CDADC1	4.27096191	4.81470384	3.80561784	3.7621332	4.04511313	4.76343217	4.14718915	4.13793382	4.34979672	4.52258327	4.54768276	3.91290135	4.07482492
CAB39L	5.49913024	5.89366169	5.28034284	4.58837377	6.38716424	6.48741438	6.28259849	6.47583152	6.18224009	5.90619505	6.26817639	6.46208024	5.79928266
KLF5	5.19199579	3.96955549	4.52982006	3.41739081	4.11189453	4.65121347	3.88954252	4.2556029	3.91264421	3.71442948	4.22746734	3.01616356	4.16522705
STK24	6.37295268	6.18568365	6.02600118	5.63771582	6.09090943	6.0429776	5.610109	5.53953082	5.46755965	5.82300758	5.54772537	5.55579683	5.93404249
ACP5	5.19113642	2.44776335	4.08635242	3.53296387	1.15808895	0.31888169	0.76229871	0.81745555	-0.96321561	2.80161761	2.75132965	2.78727109	3.86816956
DNAJC3	3.49382923	4.26118359	3.36749519	4.16699047	4.18463571	4.49048274	4.50959478	4.0307745	3.64001666	3.35152592	3.05571977	4.72490851	3.96080651
UGGT2	0.80945413	2.08041555	1.4383373	4.01767773	3.55051859	3.33989693	3.3184915	4.23096653	4.2976872	-0.37738415	2.32479982	3.67092164	3.96835557
ARHGEF7	1.36354008	2.03781216	1.20833088	1.51731109	1.87393625	2.05257739	2.81300849	2.33940393	1.67057896	3.33781934	2.94916737	1.65792838	1.67057896
FGF9	0.49910914	2.74020239	0.46772958	0.73816866	1.10676265	0.74152352	1.1338062	0.73793317	3.00789011	-0.35084921	3.32422483	3.86599449	3.75780429
SGCG	4.27688774	0.6535123	0.8660013	0.26994693	3.34509582	5.8780178	2.07237482	4.55799208	4.8402518	3.24478169	4.83774664	4.53894182	6.41925186
PARP4	6.21641031	6.55133163	5.66528853	6.70794982	6.07033267	6.26009824	6.0094648	5.99399891	6.36505735	6.12985712	6.34880439	5.99315872	6.5538669
MRPS31	4.34145423	4.44277345	3.80913638	4.28277175	3.98019866	5.16254788	4.18258345	4.94821752	4.91396226	4.59096012	4.38866282	4.3208048	4.47050867
SLC25A15	2.20584929	2.6140836	4.27650184	3.65728656	2.7003473	3.02223807	3.07052426	2.57775063	2.1051007	2.50458457	2.76120585	3.48693822	2.91163671
KPNA3	5.11517174	5.84207557	5.34587352	6.42179769	6.28003028	6.46044429	6.50385559	6.14561651	6.56570899	5.64224674	5.93194169	6.40335124	5.96847536
FLT1	2.66693116	5.27249873	3.71243294	6.21789627	7.16121679	4.24582511	5.05486349	4.26453509	5.77637525	4.65808869	5.88897572	4.75192548	7.41869451
DGKH	1.87211838	2.42301706	1.76168683	2.7079395	3.19620196	3.25625369	2.85080372	3.21396018	3.33880963	4.27028948	3.82314274	3.36810113	-0.3758085
KATNAL1	4.90487605	5.63513737	5.24129659	5.29761633	5.99262423	6.03360972	5.99262423	5.97664507	5.99366105	5.37733709	5.9174172	5.90173936	5.52723288
INTS6	5.09991798	5.38137229	5.10134784	6.01258411	5.6838014	5.90534928	6.0472178	5.81484097	5.65634614	5.17904107	5.71655288	5.43422006	5.48072502
DHRS12	3.62376329	3.25785837	3.05918748	1.76171535	3.3454356	2.97598782	2.72799158	4.35653963	3.75158125	4.05987986	2.58473127	4.06614638	4.61076159

TSC22D1	7.73950109	7.5427571	7.00432219	7.31788988	7.29099742	7.52357466	7.12786756	6.41138808	7.13245662	6.72782228	7.81789198	6.98048588	6.86381258
CLN5	4.42954106	5.34843428	4.50249794	5.27303764	5.65656993	5.5492245	5.29644644	5.38349827	5.72924172	4.60479926	5.38306044	5.14615456	4.61226973
OLFM4	0.68504537	0.66344527	0.66580257	0.70161198	1.06019366	1.02766122	0.763828	4.3857424	4.73549084	5.21672277	4.51145433	4.03729324	-0.47978104
MSLN	-0.04477741	4.99011604	0.10239965	0.61119215	-0.06062438	-0.05990115	-0.06062438	0.3858467	-0.11453322	-0.11453322	4.98053752	-0.11453322	-0.11453322
MGRN1	5.06027043	4.3636948	5.44974225	4.86512375	4.57249702	4.08241319	4.54766794	4.44698881	4.02427202	5.19436016	4.32128467	4.73989947	4.2349505
ZNF629	5.77763078	5.1689277	5.80748534	4.84765551	5.03440271	4.9330058	4.64684562	4.977633	4.90234655	5.35228497	5.15802457	5.30751559	5.48846625
TRADD	4.29971916	3.95456939	4.90772261	3.0903578	3.20212122	2.98963194	3.0773012	3.16957274	3.86395156	3.49411588	3.58532824	3.52954882	0.31473303
HSF4	4.81232849	4.098435	4.44059374	2.5979846	3.5476026	3.27791251	3.91153444	3.21929253	3.78006897	4.75120513	4.03339911	3.86129364	2.22215968
CORO1A	4.56163595	4.97743899	5.34014517	4.87630896	3.52813027	2.76562879	3.8661148	2.9871524	4.15271707	4.73318851	3.19503489	3.28159057	4.1051801
MAPK3	6.6140245	5.68031252	6.26735366	5.15775951	5.65711049	5.41262817	5.42305168	5.45005524	5.9937814	5.84602721	6.04855542	5.32903889	5.42934353
GDPD3	4.07051085	3.07212571	3.88593424	1.7110731	2.80845357	1.35756451	3.52458316	3.37842089	2.56053286	-0.83804922	3.46525452	3.45530758	1.91921277
ELMO3	0.83054479	1.74530677	2.0456969	0.84760883	1.11514184	0.48755548	1.45387966	0.69162387	-0.26812795	2.83301726	3.75938028	1.35084797	-0.26812795
PHKB	4.41004842	5.40961415	4.33752315	5.52214325	5.20802428	5.08808921	5.00159057	5.190286	4.86805897	5.76209196	5.28630232	5.52507486	4.79528804
LYRM1	4.98942798	4.59244449	4.76461044	4.81903533	5.62774357	6.02023916	5.18910399	5.49460902	5.45480014	4.87176918	5.1834297	5.1035169	4.83854587
NUTF2	5.97248164	5.30407696	5.53798509	5.69842934	5.85526622	5.93908325	4.88219186	4.71775632	5.22406808	5.70596613	5.50619078	6.14240785	4.54128556
NUP93	4.85712951	4.53731719	4.83606599	5.03718131	4.8220424	4.51762631	4.1851024	4.42117311	4.53085337	4.29924998	4.6853721	4.77073126	5.00778029
CENPT	5.23465261	5.14247556	6.03451355	4.85592861	5.32816834	4.55368899	5.57780758	5.08641309	4.69081818	5.11845853	5.56158549	5.39972105	5.31799193
TSNAXIP1	1.64204191	0.8761453	1.83116187	1.86321256	1.62453604	2.10792941	1.6474819	1.16038326	2.55905847	3.2305375	1.55479026	1.43496332	-0.81394605
NFAT5	7.34104057	7.20256272	6.80946447	7.32267538	7.80502318	7.56616077	7.68819563	7.70997706	7.42166387	6.60911647	7.57696832	7.34095468	7.30777354
LONP2	5.79126478	6.0461703	5.55714815	5.66845539	5.98511889	6.08667711	5.96747602	5.9987994	6.15788951	5.9136446	5.87823295	5.99138643	6.02130772
N4BP1	5.69561916	5.78816323	5.65546581	5.82566094	6.04709135	5.67277938	5.93114175	6.04069679	5.87997117	5.80304856	5.9515624	5.74333271	5.60871123
CBLN1	0.4909249	0.70128107	0.73117723	0.61778934	1.09445128	1.15285367	0.62729721	0.4942264	-0.38947994	-0.38947994	3.03044829	1.31517983	4.40219186
ARL2BP	5.69314531	6.43297251	5.22630466	6.22406572	6.16156717	6.12312522	6.20819566	6.1325871	6.08987618	6.00866256	6.32945529	6.27944562	6.59378673
PLLP	-0.28172365	2.66748347	-0.07531648	-0.20591221	0.14196935	0.02347326	0.11281719	1.17873854	-0.23850606	-0.23850606	3.77306678	-0.23850606	-0.23850606
ZNF423	4.99012873	3.64213584	4.47546425	3.43255008	3.52139471	3.40043395	4.3822735	3.1252405	3.47763616	2.45409303	3.82396721	2.2238803	3.24765992
CCL22	0.84704208	1.55855935	1.05273255	1.63537522	0.88209087	0.84846852	1.27306243	0.96625181	2.80075656	-0.49326506	-0.49326506	2.38361059	1.58955924
DHODH	4.00165242	3.6726056	4.51496082	3.18176148	3.38846859	3.8688564	3.27761764	3.19340059	3.14607609	2.66764624	3.54545167	3.2552218	5.13124836
CTCF	6.39367181	5.77073763	6.07976042	5.90999904	5.94610549	6.5619302	5.80542344	5.64944712	6.05840726	6.3925566	5.8225311	5.66449809	6.51445988
ACD	4.77860841	4.02024265	4.98095888	3.36590753	3.71686913	3.45093453	3.87223264	3.44152979	3.5281003	3.76644502	4.4198031	2.27077116	3.11749819
POLR2C	6.31992781	6.1442863	5.94036203	6.03091593	6.28497094	6.04085551	5.90590443	5.55717622	5.87069322	6.14813946	6.04153456	6.06770509	5.93852718
PARD6A	2.01457089	3.04003612	1.95833073	0.29833048	1.64644436	2.64645276	2.51926364	1.56623171	3.23213464	2.6058116	1.45899764	3.28422305	3.22949197
ZNF821	5.00441119	3.74565985	4.77573669	3.62627181	4.01471789	3.89618682	4.3660081	4.37556492	3.13703241	5.53517431	4.19272847	4.1301647	4.49797487
MMP15	1.41910068	4.32298199	2.15706085	2.92694949	1.824933	2.71595117	2.66259624	2.52424433	2.07085444	-1.00330772	3.82388582	2.70060187	4.19447301
CYB5B	4.05828535	3.80968026	3.78835572	3.6087025	3.68885879	4.34833775	3.68662691	3.44360581	3.43869983	4.31771761	4.03646732	3.31378415	4.18485967
CCDC113	4.42828835	3.33711355	4.52821947	3.88276645	3.90768518	4.64380241	3.36088959	3.85134593	4.42720819	3.4678341	4.02782657	4.29052356	5.16449574
NME3	3.74775123	4.41810993	4.09308753	2.67606417	2.47943061	3.03373397	3.0020038	3.17330014	3.52769144	5.11970004	0.73225578	4.33066703	1.65237319
NDRG4	4.88545395	2.17306055	1.62385737	2.65577558	2.28166403	2.38779418	2.66285581	3.35020999	3.15606028	3.66218508	2.92521408	3.60767554	5.84076576
PSMD7	6.59523051	6.51472412	6.63289035	7.00147729	6.17052901	6.60335175	6.65802737	6.07384048	6.41566627	6.68368046	6.19014389	6.35733463	7.05855669
SETD6	4.43921502	4.35366602	4.09940205	3.33683635	4.60577403	3.99711641	4.72698388	4.66347072	4.44438358	4.19109375	4.24912873	3.84556632	4.79379906
SLC38A7	4.57843273	4.04480243	4.84610566	4.43222017	3.70291799	2.9973929	3.86033063	3.38808724	3.56776803	4.51544598	3.48711846	4.31974376	3.49781605
VAC14	5.33785086	4.83251577	5.52726633	4.71406747	4.66234551	4.3603527	4.91491208	4.47288262	4.75634985	4.41208064	4.97336487	5.24168933	4.42568511
HAS3	1.91277945	1.47239227	1.91859718	1.79404433	1.8492862	1.75560794	1.77293283	1.35788517	2.69796366	2.69983509	0.13214022	2.50995113	3.63614643
COG4	5.22780321	4.69389302	5.6229808	5.36465795	4.81580562	5.09815255	5.6599017	5.02443128	4.86425579	5.32850031	5.67755539	5.12352935	5.43583651
SMPD3	0.32764205	3.95430964	0.32153108	0.57076306	0.18896844	0.13720512	0.29387401	0.33044878	0.41347401	3.11119815	3.56117522	1.17487425	-0.27649206
SLC7A6OS	4.93037499	4.9940352	5.02248491	4.30577167	4.78103011	5.23948742	4.63027517	4.87740953	5.28580579	5.32142751	4.56895293	4.51201448	5.59785918
SLC7A6	4.81141095	5.07392235	4.99638906	5.76770165	5.67470143	5.15098965	5.44934266	5.46710686	4.94893929	3.95719748	5.68740364	4.9721085	5.21154476
PLA2G15	4.45560627	4.29566438	5.21286598	4.065215	3.19311263	3.35549233	3.84008695	3.24936704	3.11053756	5.42003671	2.45732208	4.69448388	3.9990814

ESRP2	0.52689031	1.86139018	0.40028977	0.39781391	0.42009899	0.57710171	0.47647738	0.60380138	-0.29536047	-0.29536047	-0.29536047	3.21769866	-0.29536047
FA2H	0.08836048	0.87344507	0.09086734	0.13786533	0.13110715	0.08975426	0.22201941	0.09155865	0.11388887	0.11388887	0.11388887	0.11388887	0.11388887
WDR59	5.5274612	5.24194766	5.28684207	4.64881155	5.25099172	4.78150538	5.68248038	5.40933532	5.18080435	5.95043166	5.23859873	5.13527245	5.23847928
MON1B	5.34081701	5.21056158	5.08919893	4.18482489	5.18904108	4.73220004	4.89192774	4.58486432	4.47699553	5.3480648	4.96925835	4.94031815	4.82229511
AXIN1	5.23927913	4.46892121	5.37435411	3.95282088	4.64465855	4.19372202	4.69142201	4.64179265	4.75516641	4.24032449	4.89832024	5.30817723	4.44994261
HCFC1R1	3.69660704	5.30233345	5.06686058	2.24599149	4.57112276	5.22562621	3.78296878	4.4199934	4.8341677	6.62628149	5.12395218	5.63801777	4.11730945
NPRL3	5.37340857	4.25972736	4.94339829	4.59824214	4.92761783	3.89676239	4.47589443	4.33135647	4.57137161	4.51678882	4.56419582	5.00924842	4.81030692
MLYCD	5.5052365	4.93913805	4.93614864	3.64870679	4.55188581	4.37947086	4.47512654	5.22655449	4.2443682	4.56025501	3.4873844	4.71224888	5.32271267
MPG	5.14434547	4.73899218	5.66755803	4.05943219	3.92071857	4.41393971	3.74675103	4.60290122	4.55761942	5.28550331	4.60529133	4.89317194	3.39176489
NECAB2	0.35569353	-0.20707805	-0.04803944	-0.12934383	0.30664908	0.42864672	0.1645212	0.21061774	1.39816869	-0.12397266	-0.12397266	-0.12397266	-0.12397266
HSDL1	4.9715787	4.64763762	4.1809684	4.37839208	4.77217421	4.41793519	5.13282437	4.98590791	4.93985781	4.88869515	5.05937413	5.2283868	4.74466253
TAF1C	4.49658717	4.08122716	5.00189862	4.01592867	4.47729286	3.41155018	4.28721575	4.50621572	3.74061324	4.61777097	3.57311495	5.15193231	4.65785221
NAGPA	3.53977188	2.0933074	4.80920051	3.38881834	2.69179029	2.42511624	3.30279564	3.25987999	2.24254296	3.01072591	3.05555589	3.41553266	0.68311062
WFDC1	1.53142636	2.99088401	1.86063105	1.60616562	4.94277036	4.27214235	2.24493243	1.79941633	1.71035058	-1.10843161	3.78277892	1.81852604	2.55837101
SEC14L5	0.91334567	0.43455012	1.14971666	0.03263469	0.17572101	0.1346737	0.80910549	1.16106607	-0.15051486	-0.15051486	-0.15051486	2.85077128	-0.15051486
COTL1	5.71427942	5.91113961	6.36217268	6.61185852	4.29468698	4.93167836	4.6413754	4.33325558	5.18568811	5.20504069	5.46542762	5.44561632	5.47689071
USP10	5.96380711	5.97388192	5.50195752	6.34346021	6.11362437	5.83748094	5.91821019	5.56767445	5.80141892	5.89361284	5.09071865	5.48000002	6.12111074
CRISPLD2	5.00422472	5.36161436	3.03423891	7.08396913	4.64895124	3.86250513	5.38940502	5.98701506	3.96119189	5.13839305	4.70628599	5.47792301	6.09553738
TSC2	6.21790731	5.70758219	6.42141936	5.35417042	5.63939292	5.10564697	5.8915554	5.73016077	5.46376712	5.89469653	5.73345937	5.9712109	5.95219827
ZNF500	4.61205862	3.92794726	4.4672998	3.09777968	3.91236371	3.45518431	4.15228776	4.2789464	3.39868037	3.60859253	3.99603292	4.72267181	3.5840539
NME4	6.17427856	5.99853325	6.34361827	5.31132709	5.72401392	5.55804313	4.79423628	4.87319231	5.80920538	6.42222321	6.07610062	5.38840436	5.99516771
ABCC1	6.04532989	5.30326456	6.47209443	5.17649524	5.43056438	5.57557081	6.35548925	6.31466244	5.54862631	5.70483532	5.93613121	5.42093837	5.66998283
LMF1	4.33725416	3.21911255	4.7367849	2.4251369	4.17536862	2.61786414	3.45704716	3.66695239	4.07398602	3.46644762	3.69600909	4.3484505	4.04551589
FOXF1	0.46867021	0.32699059	0.73770382	0.45917576	0.61244994	1.48389441	0.74447265	0.93765547	1.02288333	-0.1826354	4.03324903	-0.1826354	-0.1826354
NARFL	4.17826731	3.71386823	4.24762918	2.67569745	4.10713304	3.60186588	4.12032091	3.77738527	3.90489653	4.40433722	4.47870793	4.43311387	2.75788571
MTHFSD	4.75291065	4.90219782	4.8347094	4.18407956	4.08670697	4.08862724	4.16901039	4.22352896	3.18750893	4.28495984	4.90912298	3.96730925	4.90290399
CLCN7	5.28734092	4.90460871	5.5943693	4.66811088	4.64197844	4.08414188	4.64666114	4.61950911	4.47985731	4.78683363	5.45631738	5.71686004	4.30440358
HAGHL	0.4603676	0.47867108	0.93614058	0.35233623	-0.08581925	-0.30275626	0.52044639	0.84946786	-0.16343997	-0.16343997	-0.16343997	-0.16343997	-0.16343997
FAM173A	1.13123978	-0.01811919	3.21117888	0.2101031	0.16483201	1.29317979	1.49148799	0.97821014	-0.29350589	3.12509104	4.3887094	1.81385969	-0.29350589
SLC7A5	4.09370304	3.05482256	5.30400981	5.50499881	1.94848599	1.77225633	1.9043255	2.1757446	1.42041399	3.37527362	1.4597234	2.26349478	1.86760428
METRN	2.42190641	3.3262458	3.37132991	2.27082615	2.24457817	2.14616096	2.23053733	2.49510548	2.71795291	2.65642828	2.30300515	4.00650471	1.218383
FBXO31	5.74109137	5.1068904	6.04922162	4.67053495	5.63221134	5.05058255	5.55682722	5.42731812	4.89519761	5.81341298	5.49716844	5.71714606	4.82124571
STUB1	6.06471226	5.79791642	6.3638411	4.9430191	5.51980401	5.5037042	5.37136258	5.41120235	5.79271231	6.52406104	5.88354059	6.19266763	5.09035739
RHBDL1	0.78797726	0.08589385	1.1959532	-0.85614423	0.00798318	0.69319847	0.33112368	1.07661545	-0.27567185	3.86479729	-0.27567185	-0.27567185	-0.27567185
NUBP1	5.57142668	5.3640091	4.96225949	4.84849175	4.45918023	4.7879258	4.46875892	4.84772836	5.41154303	5.45422706	5.02817074	5.28117383	5.13824664
UBE2I	6.56780828	6.18496148	6.21399265	5.78807974	6.23954461	6.01117256	5.96100511	6.04220466	6.00900341	6.40093208	5.75793672	5.94993685	6.44073382
MEFV	3.02308914	1.64380359	3.01912446	3.33327204	1.87746967	1.59096444	1.53523377	1.37094012	2.18650771	-1.18802545	1.88255645	2.31506235	3.60384512
EEF2K	6.29516692	6.52676904	6.39743399	5.65416104	6.43482633	5.87151394	6.76577356	6.75285972	6.27111997	6.69305235	6.74306753	6.54399623	6.41596638
PIEZO1	3.55933717	3.78018471	4.79671243	3.67065175	3.45263639	1.24456147	3.08027367	3.14412822	2.07873075	-0.68067611	4.55512899	2.89817775	3.31850418
GSPT1	6.00371684	6.53800581	6.35555707	6.96878479	7.08930731	7.06872964	6.9153881	6.93583814	6.43560625	6.50327746	6.43609584	6.94313788	6.74512088
ZNF174	4.82984855	4.17611449	4.45874227	3.68165695	4.0046525	4.93798513	4.02250783	4.13227631	4.3569511	4.88480601	4.65371937	3.60463336	4.87068192
CLUAP1	5.08217646	5.20954958	4.85930276	4.27045734	4.86639566	5.16246598	4.95654541	4.87526066	5.22358633	5.3314266	4.66420397	5.04104382	5.1201728
UBFD1	5.4504313	5.40875935	5.3562248	6.2371628	5.67894927	5.2037632	5.78283783	5.14543431	5.50878641	4.93893783	5.72357148	5.73923851	5.63089054
EARS2	4.69795849	4.09163963	5.1432819	4.97549023	4.46029413	4.52463893	4.00296525	4.59901087	4.02469746	4.29695044	4.33083156	4.53652582	4.86469129
GGA2	6.05217542	5.70043379	5.85513839	5.83991708	5.59453857	4.98760727	5.79216208	5.63342712	5.67581896	6.0848453	6.1591043	5.6913104	5.62910658
CPPED1	5.49377983	5.15621661	5.46205656	5.33857844	5.1486636	5.70160504	5.67674945	6.04989009	5.78967655	5.23064207	5.65535623	5.62240321	5.54260762
USP31	5.2987829	5.53377197	5.22041602	5.07445072	5.94473229	6.0750584	5.7713171	6.65697757	5.42111106	5.8038573	5.64771362	6.51332738	6.06112085

HMOX2	4.89188331	4.65066833	5.3126267	5.44268313	4.46065975	4.61230767	4.26676698	4.48101337	4.49370361	4.77214948	4.67663531	5.31085874	5.0341007
DNAJA3	5.79348321	5.22513616	5.4145621	4.81004923	4.86692364	5.07659952	5.13697358	4.41258632	4.97715474	4.62388411	5.12968296	5.13622756	5.97069134
BFAR	5.70688578	5.60879374	5.71426839	5.91342619	5.48040053	5.45587776	5.27751041	4.9764321	5.33712494	5.75143828	5.41067132	5.12245594	5.40432829
SALL1	0.29444261	0.49836709	0.28636306	0.31815909	0.2837646	0.28520931	0.2837646	0.56268969	-0.21342077	-0.21342077	-0.21342077	-0.21342077	3.60380634
RBL2	6.88443665	7.03189481	6.34191319	6.6697269	7.15156187	7.37071344	7.268752	7.48742701	7.00667893	7.2871768	7.23330091	7.08730162	7.25381913
QPRT	0.9204006	2.01059818	0.87890452	1.95096703	0.8820664	1.45384461	1.99420586	2.59800493	2.22782109	3.19039918	2.36586369	0.44942393	3.89710716
XYLT1	5.11952377	4.19641907	5.96242575	5.48007049	4.69181956	4.28260731	4.20463501	4.0852587	3.93175664	5.16534143	4.34234775	4.3899687	4.95318518
PYCARD	3.98095568	3.73467164	4.4569888	2.2895395	2.55396418	1.49737387	1.16564361	1.36428471	2.89001351	2.154552	2.17871409	3.89993022	3.17691018
RPGRIP1L	3.60050436	3.941167	4.07661218	5.43108967	4.18288703	4.61832665	4.22120057	4.09495503	4.68259653	3.37033993	4.52919376	4.12980913	4.61602521
MAZ	5.27754509	5.37194332	6.60476571	6.24883705	5.49982812	4.68961548	5.64995765	5.45265599	5.75213301	6.3850101	5.34983805	6.00760879	5.54582644
STX4	5.11073536	4.71434773	5.40382772	4.44199513	4.35275556	4.026974	4.53931891	4.09636961	4.34310571	5.08850224	4.09016373	4.00376648	4.54500342
CDIPT	5.80653038	5.413618	6.23197966	4.47639515	4.70179131	4.97090104	4.51223025	4.96994826	4.76873974	5.43444569	4.39896596	5.58594005	4.50253976
BCKDK	5.42610355	5.06368202	5.38008032	4.98208431	4.58778306	4.48133052	4.40418445	4.23533141	4.27496843	5.02223424	5.23762256	4.77613769	5.01260225
KAT8	5.31733764	4.67742396	4.8526324	3.83933859	4.52809113	4.59194978	4.51435061	4.68432043	4.74667695	5.74052018	4.48898371	4.75531208	4.87802471
NOMO1	5.52559172	5.40412131	6.52914163	6.43038173	5.85670176	6.08979216	6.08722382	5.89802865	5.68782085	6.33885848	5.85783996	5.36119916	6.75260928
IL21R	1.27208681	3.48557449	1.49278782	2.63604523	1.13313169	0.09484685	0.79211639	0.3330154	3.5829645	-0.67457339	-0.67457339	-0.67457339	1.78684258
SYT17	1.66861823	2.76088129	2.03199452	1.70131987	1.67281135	1.98369891	2.17861027	4.05430934	3.59411201	4.28406126	3.05914008	3.96387699	2.72528061
CCP110	4.75741575	5.45646165	5.4131241	6.04586416	5.85610325	6.11226982	5.98852121	5.98063542	6.03425226	5.25953289	5.88848942	6.0571474	4.77657931
C16orf62	6.01738319	5.87017137	5.59236418	6.10834428	5.76617478	6.05834989	6.1288991	6.22221806	6.20591448	6.51266115	5.90526945	5.9814602	6.448063
RNF40	6.44400134	5.79529151	6.6003749	5.8152057	5.80912302	5.76152897	5.56613201	5.59457014	5.79303372	6.34098914	6.11147487	6.04791749	5.92607146
AQP9	3.87985029	3.71273342	3.51636896	2.99577602	0.61031824	-0.0932283	0.5870932	-0.31930963	0.96677076	3.46290626	-1.34739475	1.65331158	1.71341844
AAGAB	4.79093386	5.57511771	5.57096384	5.94255143	5.04860304	5.81786488	5.1050518	5.14880884	4.97631347	4.90542032	5.43458455	5.09510444	5.24364053
IQCH	2.09269008	2.08644758	2.23428393	2.9399789	1.7096555	1.78614169	1.95817798	1.29803121	-0.3262925	-0.98768241	-0.98768241	1.76505194	2.880945
LACTB	4.19156406	4.03174172	4.84884641	5.28822967	4.47591484	4.22333187	4.66411211	3.89915727	4.83759202	3.15643724	3.79028567	3.48533713	4.00841794
CORO2B	4.28129878	3.74008924	4.21906797	3.39342321	3.34671524	2.63361204	2.45768367	3.88010428	3.39164609	4.55795631	3.3242265	4.44389462	4.15379901
CSK	2.91522808	2.64268617	3.643247	3.09031845	1.71165869	0.54269932	1.98010699	1.46775206	3.1492597	4.23482333	3.76582301	2.09030919	3.51397856
HERC1	7.14888781	7.51542066	6.5951182	7.01753044	7.13472294	6.98944808	7.32776808	7.06831568	7.02564938	7.19795966	7.37083224	7.00422036	7.84516495
TRIP4	5.08315258	4.87625726	5.1320583	5.7424975	5.37378053	5.26876284	5.28050342	5.33420951	5.47420228	5.45316457	5.52180829	5.0616859	4.78488299
MTFMT	4.33169652	3.70411093	4.0916785	3.50841008	3.95242117	4.44762038	3.79776822	4.19450862	3.77841504	2.983538	3.79531832	3.44023601	3.93075192
RASL12	6.7363149	6.05940059	6.76428879	5.20067407	6.92430531	6.80962115	6.68790659	7.15488817	5.68633417	7.16935892	6.02989524	7.19391966	6.38448971
AP3B2	0.73897392	1.25491764	0.86684966	0.52418673	0.75254881	1.47666237	0.71819677	0.92638903	-0.13147724	-0.13147724	-0.13147724	-0.13147724	-0.13147724
IGDCC4	2.05122827	1.50544104	1.71131515	4.18579104	1.52769543	1.38017415	1.64858683	1.60731198	0.64286413	3.99442038	2.38758047	3.18071115	1.80016775
RAB11A	5.5783604	6.47857284	6.08231143	6.83334203	6.75658068	6.8762664	6.87011426	6.70128756	6.93585422	6.02933466	6.73355816	6.53009209	6.05031483
CTSH	5.68653729	5.66847193	4.74626856	5.04624906	4.5257639	4.23282258	3.72620195	4.17179705	4.88583647	5.08920813	4.94415222	5.7002559	5.69380407
TTC23	5.50388007	5.41062642	5.05314302	5.17451836	5.26199616	5.29426186	5.0062121	5.05160931	5.33653726	5.22663021	4.5769074	5.09761685	5.43725005
CD276	5.86060668	5.57488447	6.38926989	6.6804573	5.65687022	5.16588594	5.46249755	5.85458873	5.56998761	6.44769107	5.9699363	6.03752463	5.23130219
FAH	4.80755781	3.93918673	5.01084121	3.67194442	3.81751976	3.84869175	4.54375164	4.07040642	3.25035876	4.52389258	3.51507749	3.60896346	4.16322013
RPAP1	4.79432032	4.34672289	5.01687211	3.89035577	4.05989181	3.86106592	4.34872485	3.7774069	3.31295909	4.74119979	4.40917609	4.39514535	3.86956531
HOMER2	3.32748287	2.97720047	3.32624485	3.00772583	3.45933408	4.24152929	3.46669113	4.01830789	3.79725411	4.00537569	3.88417473	4.28344403	4.30576596
EHD4	5.27827058	5.51836952	6.16132975	5.70962922	5.27882875	5.24057802	5.56044223	5.69095863	5.3332772	5.99818843	5.38864588	5.82981192	5.87560237
TMEM87A	6.05932657	6.43800963	6.08185115	6.76743027	6.64631872	6.55712593	6.68171654	6.90925902	6.69689184	6.09645302	6.57696692	6.92066839	6.49691073
CEP152	3.02030685	3.51368153	3.96716466	4.22206622	3.17348225	3.81455626	3.97467568	3.19969914	3.62419811	3.93117109	2.88141591	2.62537863	3.55975549
ATP8B4	3.6993511	5.10296761	3.61304491	5.65348052	3.57569044	3.12932251	3.75238966	3.4041699	3.84619984	3.70870843	3.64051783	3.65445231	3.90051969
OCA2	2.70373255	2.83908971	2.5011836	0.53091655	3.88297557	4.70931158	4.50438434	3.87278311	4.20841178	4.22952418	2.74674301	4.73185186	3.75868809
DTWD1	5.49717893	4.41382672	4.5994758	5.49011075	4.93076586	5.49866842	5.07337865	5.13945182	5.34243225	4.08877849	5.29492788	4.8421103	3.8190617
TGM5	-0.08385173	0.17366176	-0.08198304	-0.08385173	0.0023089	0.15178748	-0.08385173	0.96433765	0.06196344	0.06196344	0.06196344	0.06196344	0.06196344
FAM189A1	1.86691933	2.69621656	2.03970897	1.46884893	2.31574496	2.04341326	1.85394882	2.49648039	2.70641758	-0.86078749	3.99519585	3.53610309	1.77447785

GABPB1	4.90681172	4.16869873	4.54762037	5.57167065	5.65304372	5.0767997	5.28011663	5.35135766	5.08883005	4.80039042	4.71653623	4.77537891	4.59209451
TJP1	7.00681941	7.58357404	6.47892776	6.93866012	7.49729812	7.14839101	7.05195884	7.08829451	7.31305071	6.62265261	7.19080384	6.97745434	7.83242761
BMF	5.25396839	3.76884367	4.52144021	4.65118055	3.57289479	4.34773287	4.36953519	4.88568264	4.09127133	5.17250786	4.51134638	4.62022772	4.58993915
DMXL2	5.21948591	5.82562064	5.38077148	6.94273635	5.43986004	5.56496889	5.60856284	5.55514327	5.44975647	4.96991641	5.92783473	5.96415974	5.78091202
DNAJC17	3.71948541	2.34727197	3.68486073	2.15721906	2.86431412	2.00525064	3.22305221	3.47855063	2.62947918	4.65195368	3.22376154	3.369979	4.3775452
EIF3J	4.99741162	5.86013147	5.28169829	6.79972774	6.64195407	6.63386958	6.44801132	6.43891815	6.09178371	5.50647338	5.61053393	5.80865054	4.98102525
SPG11	6.04048731	6.25616361	6.00337356	6.80542727	6.37834659	6.44837376	6.36571018	6.37595884	6.33689228	6.25908497	6.49264403	6.10010086	6.69827901
VPS18	5.83224629	5.49245618	6.19741315	4.6184618	5.0769556	5.29597764	4.62003328	4.87427735	4.7358784	5.16103783	5.43264525	6.0468258	4.75016643
OIP5	0.64851605	0.82408644	1.24155128	1.34468098	-0.12656917	0.11393323	0.1223344	-0.12348511	-0.35173962	-0.35173962	2.99935332	-0.35173962	-0.35173962
SLC30A4	4.26432947	4.54201318	4.21305215	4.93327935	5.12301845	4.99568882	5.24480175	5.40435641	4.97749268	4.39309312	5.00553744	5.19425441	4.90057813
MYEF2	5.29314536	6.1055079	5.56502718	5.3418007	6.80320941	6.72908859	6.67293229	7.04710649	6.48844675	6.55798216	6.84450207	6.5336922	6.147168
SGK3	1.30235258	-0.31900169	1.24515063	0.56408609	0.36232177	1.0899674	0.96243753	0.72989384	3.00106107	0.08289021	0.08289021	4.04764767	0.08289021
PDGFRL	3.89070525	4.34944217	3.27333894	4.99553863	3.73043203	3.70739513	2.78559987	4.38786267	4.70651863	4.07951152	4.69677183	4.24377772	4.57269111
CSPP1	4.28044638	4.0198089	4.1373869	4.7020882	4.39132746	5.04939566	5.04199458	4.83450925	5.09077878	3.97278221	4.67429497	4.03970851	4.26098793
ZDHHC2	4.75539351	5.62158568	4.9427482	5.5005249	6.22726434	6.14847282	6.09658675	6.33393213	6.4926955	5.45155235	6.18552286	6.19949951	5.32780259
BRF2	4.55522803	4.33423842	4.88581801	4.29999283	3.53439197	4.30808233	4.33519127	4.1463378	4.06810693	5.0817913	4.02908123	4.17100975	3.86059192
TRIM35	5.1375188	5.17418185	5.1795188	4.51407257	4.63930584	4.9673147	4.70131123	4.65391305	4.89046104	4.34271957	5.08218947	5.15491093	4.75322421
ZFAND1	5.07901275	5.90660051	5.13172564	5.92905623	6.20913114	6.26658474	6.19619395	6.02435474	6.34569539	6.26521382	5.80066862	5.98559326	5.4228704
RP1	0.23185433	0.40082487	0.4708636	0.3085574	0.23861227	0.38478704	0.22333445	0.22665173	1.96130681	-0.16952766	4.27057947	-0.16952766	-0.16952766
CA2	2.4090188	-0.04789752	1.38187435	1.32130004	0.16750004	0.56098893	0.27073217	0.68828177	2.11839205	-0.47886732	-0.47886732	3.02441054	-0.47886732
FZD3	1.48877391	0.69238783	1.26569745	1.26999714	1.98565774	2.9945486	2.24438491	3.03706802	1.74031987	-0.78207233	2.31558751	3.53815955	2.2939567
INTS9	4.92124562	4.25367831	4.33705456	4.10629197	4.25087501	4.36699923	3.82500262	3.87479082	4.11965615	4.35731573	4.06421356	3.62436977	3.62890969
RIPK2	3.75865138	4.11695515	4.02153293	5.25948986	4.1464906	4.72122812	4.60556028	4.72665133	4.46226159	4.62151089	4.42821063	4.15689567	4.10823547
EYA1	0.69672229	0.9558651	0.69932707	0.87786078	0.74564659	0.69817051	0.80831828	0.70004539	2.91581637	-0.44860021	-0.44860021	-0.44860021	4.29461
NBN	5.09115423	5.4813944	5.37714732	6.6339949	5.9134235	6.22937478	5.98574365	5.8362177	6.48905009	5.46561113	5.50052918	6.21239485	5.11731282
TRPA1	2.0838985	2.14148066	2.2846696	2.7029551	2.18580868	2.05781927	2.14721916	2.05968827	2.84193944	-1.19433452	3.68241	-1.19433452	-1.19433452
DECR1	4.58874277	4.99732781	4.49071694	6.08294253	5.34347373	5.36514125	5.40162243	5.48404176	4.81564478	4.45478985	4.50022824	5.20161653	6.39670941
IMPAD1	6.56631478	7.19934454	6.90920704	7.50911282	8.05599657	7.94965466	7.62249794	7.82756466	7.46533041	6.65851047	7.93617351	7.56032227	7.05143264
SFRP1	7.7904364	5.66587837	8.19858163	5.4617357	7.48358637	8.45751768	7.6181849	8.79491761	7.71372031	8.20039323	7.91552778	8.34902703	8.0842392
LAPTM4B	5.29739589	5.30911228	5.00970206	6.35076958	5.11735915	5.65276349	5.98367911	5.65644177	5.48717951	5.57002014	6.13659916	6.14001328	6.28980999
UBE2W	4.91194673	5.30747732	4.82703853	5.71055391	5.75542339	5.98226712	6.0158092	5.91193908	5.89330105	5.00370188	5.93311096	5.98821521	4.94217633
POP1	3.27576802	2.95110431	4.04831177	3.99139413	3.73588649	4.03736959	3.33430122	3.44000865	4.08778489	1.73413867	3.00570392	2.61366988	3.371084
NIPAL2	5.14611864	5.03356186	4.34028622	5.68655266	5.13118875	5.50070191	5.32527219	5.67256805	5.59478689	5.23181013	5.52190121	5.21897277	5.06230738
IKBKB	5.72546323	5.57235549	5.61738589	5.39677276	5.42203781	4.89053368	5.83173367	5.85205016	5.16401212	5.47876658	5.75782992	5.78010232	5.43058437
PLAT	6.28796636	5.21166215	6.02464203	6.314244	5.4027273	4.35796768	5.37920594	6.05836699	5.20957229	5.47471967	6.21117115	4.99671497	5.9082184
JPH1	1.33975715	1.46298148	1.55867783	1.40527037	2.49101033	2.89527761	1.83612811	2.20812729	-0.76857321	2.58697816	-0.76857321	3.45757128	3.98471982
STK3	4.96998382	4.87626488	4.81388535	5.45995505	5.55715632	5.84757679	5.78709908	5.64514131	5.62379958	4.84255711	5.30973006	4.90867685	4.80554891
GDAP1	3.40220689	3.44084622	4.13172008	4.47722849	3.0945699	4.21666909	3.99215849	3.29281087	4.7932993	3.02446199	4.05319445	4.18070501	3.97452924
RAB2A	5.93948265	6.37490352	5.58898333	6.78677756	6.77498543	6.91091442	6.78933353	6.75846601	7.03479179	6.39003758	7.0507575	7.00375524	6.18274055
EIF3E	6.83589482	8.00337111	7.36800121	8.36942138	8.04337199	7.95624102	8.0605923	8.16712497	8.09931599	8.38712846	7.5309616	7.97181538	7.86205921
WISP1	2.12595725	1.54538142	2.08768149	3.49803319	1.64562469	3.21917681	1.3150466	2.73832649	-0.07028637	3.5934817	4.48334267	2.00353511	2.16705137
NDRG1	9.17125469	7.62849084	7.14829653	6.91080586	7.50957036	6.60712692	6.97079661	6.61685755	6.39463168	6.76800957	6.48013885	6.46465066	6.78871037
IL7	0.82813302	2.26505973	0.43660714	1.87509509	0.44018652	0.79255066	0.96002989	0.68676047	3.53439079	-0.46684266	2.74306457	2.19704127	-0.46684266
STMN2	0.54939003	1.57333352	0.55193892	0.54939003	0.6986612	0.55080718	0.83559349	1.19768534	-0.24419768	-0.24419768	4.04102336	-0.24419768	2.61381624
ARMC1	4.78235614	4.87415865	4.69698301	5.57706413	5.6487494	6.04683495	5.87757327	5.80044247	5.52979155	4.85252906	5.96093747	5.86383048	4.11305566
TRPS1	7.35806285	6.90543423	6.63182785	7.75790269	6.50673715	7.03045636	6.54027351	6.7276195	6.64616539	6.42639783	6.82123065	6.66092923	6.83265138
SPAG1	1.24780193	0.88424116	1.11656404	0.75668332	1.99589812	2.70366322	1.85215717	2.12903821	3.51401735	-0.47204137	-0.47204137	2.6068114	2.55779802

CHRAC1	5.33875179	5.27922398	5.02271605	4.64613077	5.52343454	5.46904504	5.29359238	5.6228202	5.73132779	5.37350156	5.50687947	5.32995701	5.21110114
NCALD	5.87609625	6.2854573	5.34608344	4.81782479	6.45024281	6.95886168	6.76451832	5.2786755	6.53344358	6.58357416	6.70177752	6.53703344	6.14720475
SNX16	3.11168146	3.51697966	3.26375812	4.05473769	4.46663409	4.97283628	4.83668217	4.77990026	4.21802341	3.67244518	4.8526753	4.94470289	3.39346146
UBR5	6.79396445	7.06897014	6.57551683	7.29739583	7.2030694	7.30944302	7.56632125	7.50178485	7.01265206	6.95965432	6.95821529	7.04953623	7.7496486
GSDMD	4.75055031	5.02528342	5.18864209	4.24684504	4.3741935	3.79667469	4.50834606	4.49790152	4.60004614	5.1121877	4.66830288	5.06004163	3.65044104
TSTA3	4.7798771	4.67933056	5.07318191	4.48773244	3.99271387	4.3037005	4.0505285	3.73631493	4.6049226	4.65025418	5.09595716	4.05510984	4.74638668
EEF1D	7.28682321	7.30403361	7.62576249	7.33370154	6.77247842	6.29814256	6.90895945	6.76627757	6.85608973	7.87080398	6.89089602	7.34978087	6.89223711
SQLE	3.94571677	4.3263224	4.12643671	5.92059332	4.76849895	4.54408351	5.08346327	4.79525883	5.1298192	4.96576984	5.64013512	4.26379784	4.34297417
SH2D4A	2.9557321	4.19291688	2.65665758	3.69884402	2.79640364	2.90035763	2.31523555	3.00129994	4.2346719	3.24469041	3.96077768	3.24201524	2.81236293
INTS10	6.6404526	5.82648415	5.55288126	6.46656411	5.41089881	5.4923981	6.4098917	5.78577148	5.77045657	5.80755984	5.37697695	5.62799814	5.56863312
ERI1	4.45450404	3.86608298	4.49622997	5.34748388	4.74619362	4.79266682	4.61762521	4.18607931	4.49007212	3.56114907	4.55539908	4.33286988	3.80249207
SLC39A14	5.78017394	2.51425546	4.08943367	4.66578272	2.94082186	1.44105289	3.20887625	0.70885309	3.78829012	-0.33888316	-0.33888316	3.1635254	-0.33888316
MTMR9	5.06272023	5.21498274	4.8462629	5.86739126	5.72054785	5.78317833	5.80030068	5.84731133	5.28205637	5.07419776	5.87722203	5.76096185	4.97703308
LEPROTL1	5.32110443	5.93639832	5.10651874	5.41885965	5.96784844	6.27171346	6.01341046	5.63414736	5.95361782	5.39548751	6.01691315	6.16603099	5.75383465
DCTN6	5.7837426	5.09295511	5.14369977	4.90338062	5.58181503	6.30191908	5.02414503	5.47760109	6.43048636	5.90040804	5.20928517	5.67317758	5.97363047
R3HCC1	5.07878365	4.96779622	5.58168899	4.46216479	4.59328017	4.99030884	4.21546744	4.89223795	5.01971481	5.50269322	5.07129122	4.791037	4.60585225
GSR	5.64860381	5.27174654	5.1356471	5.78748651	5.05051632	5.46704832	5.32832414	4.95286172	5.23764855	5.13423331	5.33263351	5.15143453	5.6149829
TNFRSF10A	4.44600297	2.95038588	2.35056148	3.46944416	3.82711888	2.68210706	3.36520236	3.03559414	1.64651702	2.83105079	2.4245365	2.35544154	3.76152447
UBXN8	3.03784218	2.94143724	3.38895915	4.95875644	2.84709405	3.64079513	3.43879626	3.1820199	3.35903901	2.87552689	3.69106017	2.68252067	3.18197019
PPP2CB	5.77420336	6.18755017	5.70797031	6.02880067	6.7365574	6.7655385	6.27370257	6.6309827	6.57573517	6.46416344	6.64148101	6.79419426	6.40840401
ERICH1	2.5627844	1.65317396	3.05845862	2.52854136	2.81315135	1.4519873	2.83981477	2.90044246	3.4755149	3.93812832	2.68238979	2.89519414	3.15199359
TUSC3	0.7555665	1.80724145	0.12557838	3.80197459	1.45508708	0.66238918	0.97060241	0.79880169	2.88230706	-0.6554212	4.36527432	2.78241982	5.43300083
ARHGEF10	5.37368047	5.02416294	5.28048838	5.20483911	5.77088338	5.41341443	5.94104203	6.50951535	5.88486549	6.17209775	6.04399646	5.90335958	5.71521151
KLHDC4	1.23099603	1.06989331	1.63373341	0.2145134	0.62259878	-0.34385483	0.87004712	1.18222506	0.4608907	0.4608907	0.4608907	0.4608907	0.4608907
MCM4	4.60541538	4.08868185	5.30891738	5.59366978	3.63123777	4.00424488	4.05719237	3.46992613	4.54790748	4.51341156	4.29398634	4.84062715	4.56477421
KCTD9	5.8737059	6.5261668	6.28207874	6.58773084	7.22433721	7.37329858	7.41609702	7.16370798	7.22872032	6.81732507	7.11698317	7.33218666	6.34042113
ASAH1	6.63796072	6.56612324	5.73324704	7.13200384	6.43411624	7.1094779	6.65863123	6.14921069	6.97833714	7.22754644	6.90480572	7.10055091	7.46876814
BNIP3L	7.22505914	7.493761	6.64844558	7.59561242	7.54077433	7.90460194	7.09106082	7.65743554	7.56915174	6.76323012	7.33738927	7.10937936	6.85280505
MAN2B1	5.81430987	5.54857849	5.88159539	5.80945567	5.45359848	4.68501265	5.38009368	5.58563139	5.27839559	5.51239686	5.73317345	5.85060447	5.1486395
KCNN4	2.48834229	2.12919932	5.08448617	3.18200528	1.66805848	0.68077569	1.11321175	0.97444856	3.37524691	-1.26824473	2.87838591	2.61216877	3.35126631
TULP2	1.65190792	0.06167993	0.35602109	0.5469309	1.00633811	0.76996016	0.71952768	1.09933154	-0.5054735	-0.5054735	-0.5054735	-0.5054735	-0.5054735
NUCB1	7.85389331	7.21753745	8.12766783	7.15207694	6.85388712	7.19995078	7.05083313	7.12501031	6.90120399	7.79068168	7.19438404	7.05550226	7.43669869
DHDH	-0.11014862	0.06315027	-0.10779478	0.61112129	0.19776849	0.14962494	0.1600218	0.70878083	0.04644812	0.04644812	0.04644812	0.04644812	0.04644812
GYS1	6.32174846	4.94769895	5.78533277	5.44408056	5.73990138	5.18511012	5.32376144	5.92462184	4.95220894	6.30838108	5.27322725	5.77556959	5.67041244
MAP4K1	2.95350371	2.68901249	2.87056722	3.27646696	1.98733136	0.9414465	1.91914156	1.7963569	2.76694185	2.50610187	2.6948982	-0.90548158	1.65340592
ECH1	5.77435453	5.19287176	5.54219369	4.82221573	4.40635219	5.33435962	4.40582149	4.66144422	5.02569723	5.77526551	5.3134832	5.16472005	5.69450762
HNRNPL	6.88676492	6.28372444	6.70823509	6.82598886	6.21078754	5.82831063	6.37566588	5.94915351	5.8632542	7.02353754	6.60717393	6.80448928	6.31491567
NFKBIB	5.07108527	4.2038637	5.61165419	3.61331146	4.44832267	4.74998872	4.52036869	4.06728662	4.02118382	5.58619504	3.94269624	4.23025243	3.81615209
LHB	0.23690329	-0.18183001	1.05973334	-0.18183001	0.83379802	-0.18068114	-0.18183001	-0.17919382	-0.05925373	-0.05925373	-0.05925373	-0.05925373	-0.05925373
TUBB4A	1.33309546	-0.1793498	0.02385177	-0.1793498	0.0031602	0.51270943	0.7115475	1.11215045	0.42879246	-0.25243663	-0.25243663	-0.25243663	2.22741089
SARS2	2.58376577	3.06411255	3.47014033	2.45566202	2.1888872	2.35012378	2.50465696	2.76409118	3.14387998	3.5923462	0.95405111	3.51395868	3.00472269
SNRNP70	6.68343343	5.78084889	6.32263244	5.4075409	6.30464806	5.31155708	6.07281097	6.33742757	5.79696781	6.00880628	6.29021237	6.08366562	5.5417779
CLPTM1	6.70478995	5.33697049	6.37188446	5.51352263	5.74966889	5.67509663	5.43018835	5.49599547	5.66551854	5.93788263	5.6513233	5.66618021	6.18885856
RELB	4.75462844	4.59738093	5.04700846	4.94694446	3.08437823	1.81786663	3.10806581	2.63641089	2.63743593	3.70330805	1.61949948	2.8382565	3.46199562
CLASRP	5.57805968	4.52555273	5.61204393	4.44206099	5.17130461	4.35812497	4.77608904	5.17181328	4.9315992	4.95250474	3.77057994	4.53327035	5.01094157
LIN7B	3.26415098	0.54135799	3.93142772	1.38708905	2.36557152	2.33852692	3.05445022	2.72091725	2.98169138	3.92828455	3.05568581	2.85400132	3.13722453
PPP1R37	2.54536267	1.8330209	3.47994824	1.24632901	1.64940163	1.31634872	2.04596226	1.49436152	2.49397827	4.21476482	1.21887576	3.24635924	2.77042707

FCGRT	5.66482676	5.74052943	6.15690518	5.78162769	5.03268532	4.08129563	5.15156298	4.74345505	4.97578316	5.90682291	6.24206206	6.06646184	4.66153462
PIH1D1	5.75513502	4.39995662	5.70924036	4.64715613	5.07729708	5.44066237	5.23434495	5.31082392	4.39264779	5.18765133	5.01811056	5.04794234	5.45121312
CKM	0.70447641	1.24711997	1.02241184	0.70447641	0.70447641	0.7059223	0.99743984	0.70779415	0.07640507	2.06708013	0.98422658	2.60687708	10.0826723
ARHGEF18	4.13535552	3.84169256	4.53461374	4.07299972	4.32483439	4.24659913	4.19485357	4.20477394	4.05692101	4.25105291	3.37736226	4.34642373	4.4465605
PPP1R13L	5.30623687	4.56189751	5.7162889	4.13554791	5.7312289	4.46579294	4.92892496	5.3591888	4.64999245	5.47137089	5.00759194	4.98689888	4.95702409
PEX11G	1.05558073	0.82228656	0.8724959	0.13065819	-0.21103214	0.21364915	-0.59606687	0.12667071	0.03161539	0.03161539	0.03161539	0.03161539	0.03161539
ERCC2	4.30056389	3.60124139	4.43858184	4.15038873	3.12945612	3.62659691	3.70908229	3.43399978	3.58735443	3.71599163	4.2762531	3.65425053	3.55520529
DOT1L	4.61072842	4.22402473	5.38365345	4.61072842	3.82342042	3.65451073	3.74152794	4.25245594	2.60013433	4.06173944	4.56043945	4.01447596	4.4807654
PLEKHJ1	2.85553457	2.80211708	4.52074692	2.85384467	2.55527347	2.70737066	2.55881026	2.76352318	4.05577429	3.43682438	3.62233333	3.99071143	0.8093684
SLC17A7	0.20841105	1.36689068	0.0285933	-0.26404601	0.79245784	1.19090017	1.21985143	1.89103843	-0.32036019	2.97190193	-0.32036019	-0.32036019	2.87057245
RNASEH2A	3.11283396	3.40932721	4.11334172	3.73721956	2.31709122	2.48874458	2.84501057	2.43649182	2.87055395	3.54762292	3.24575983	2.84394335	3.03473343
KLC3	0.05947296	0.18961566	0.13115181	0	0	0.00069205	0	0.00158797	0	0	0	0	0
CD37	4.65760879	3.75690784	3.91175662	3.92516217	2.90692582	2.0959877	2.77804412	2.18394665	3.83115248	3.29382679	3.2068696	3.64500827	3.23016123
SF3A2	5.50655192	4.87043237	5.63928243	4.3730953	4.96573255	4.4397987	4.45117469	4.75758953	4.87729318	5.76430394	4.73155016	5.10544591	4.01351304
AMH	-0.59052766	0.2045463	1.5855153	-0.68766117	-0.21199819	-0.68666647	-0.28447364	0.21622494	-0.27866481	-0.27866481	-0.27866481	-0.27866481	-0.27866481
DKKL1	1.86213624	-0.00327286	0.34197162	0.04084451	-0.00327286	1.10126415	-0.00327286	0.82397965	0.04048375	0.04048375	0.04048375	0.04048375	0.04048375
LYL1	2.38322513	1.67295917	4.44283828	2.25072023	1.83724397	2.12604104	2.57218527	2.18040974	2.89582021	2.89769213	3.03338585	4.34248558	3.42850269
OAZ1	6.8778252	7.01922485	7.38447852	6.80005023	6.51937906	6.40784888	6.61432287	6.28794524	6.85042561	7.04880862	6.9550413	7.00289515	6.64430138
TRMT1	4.4800976	3.95558539	5.07464491	4.11914309	4.1201432	3.87975187	4.16510874	3.88177139	3.63718826	4.19907401	4.2390024	3.63411683	4.55702382
STX10	5.02241783	4.60442406	5.26956808	4.21334738	4.22903726	4.06126254	4.46403962	4.45101567	4.55342939	5.0172577	4.42548725	4.80904795	4.80588141
RETN	-0.27625171	-0.48268893	2.8682147	1.34874218	-0.48268893	-0.06457397	-0.48268893	-0.48012329	-0.06507123	-0.06507123	2.46469809	-0.06507123	-0.06507123
FCER2	0.58389356	-0.0073677	0.87072438	0.2321602	0.05353407	-0.00598457	0.31124183	-0.00419396	-0.13049563	-0.13049563	-0.13049563	-0.13049563	2.92601254
DMPK	5.45177773	6.28952126	6.38360819	4.59767923	6.6748804	6.39985451	6.87601731	6.94126609	6.12932504	7.62885007	6.79233479	7.16841212	6.65914381
TBC1D17	5.50649473	4.7135295	5.68911102	3.8143395	4.9124143	4.66411582	5.34595331	5.45605877	4.11070076	6.1310412	5.72083763	5.32702856	4.80383648
IL4I1	2.20347787	0.56424135	2.09276509	2.78171666	1.36164933	0.29747879	0.66789586	0.42475977	3.40231375	4.20098342	1.94006584	2.73143773	2.96233635
TLE6	2.6704374	1.15451408	2.12720073	0.5514143	0.45792779	-0.07001513	1.40073419	0.17578925	-0.43888955	-0.43888955	-0.43888955	-0.43888955	-0.43888955
CCDC130	4.94812921	4.2153557	5.13388654	3.9244511	4.75225338	4.52908788	4.2294883	5.01878673	5.02635291	5.28887676	5.28850041	4.92445434	4.54450636
PTOV1	3.85109705	2.80517263	3.70779843	2.37539244	3.10956735	2.12512125	2.72198275	2.70186119	1.32979246	4.40128211	2.65992001	4.75247924	-0.17003856
AES	6.77105839	6.49854867	6.44418387	5.37423099	6.20741755	6.49036915	5.74163182	6.11554529	6.41316709	6.60698795	6.31671114	6.16400421	6.26597625
NOVA2	0.94392981	2.2330449	1.68747661	3.10889869	3.45731105	1.70031177	2.2684257	2.70262809	3.5230803	3.85172283	4.35941881	3.05215939	5.62898666
SGTA	5.7792013	5.51974236	6.09443525	5.1291796	5.32180766	5.22043778	5.01054833	5.14010856	5.27751423	5.73546481	5.43551487	5.17118948	5.50273497
LILRB1	4.63566203	2.54731618	4.36997169	2.75769606	1.30201779	0.54868572	0.96796224	0.3574744	2.45593164	-0.98691985	3.90271345	3.57556881	2.1322583
MED25	4.36632275	3.5282273	5.09961574	3.10237631	3.40257241	2.68468724	3.59728384	3.49627384	3.51746384	4.69020362	3.6017507	4.0028665	0.81120932
LILRA1	3.19761869	2.49429502	3.06505676	3.0892177	2.03135332	0.90137819	1.17924658	1.08095524	2.49706317	2.94033738	2.85082707	2.90840435	3.07752416
SNAPC2	4.97857566	3.82693991	5.05739011	3.6525334	3.9498604	4.19016895	3.7545068	3.78581939	2.71165852	4.91226334	3.91060549	4.0883781	3.73691623
C19orf53	4.88449517	3.37533842	4.87975443	4.08542351	4.23145879	4.79845335	3.60337135	3.4910526	5.62777288	5.2400289	3.91835173	3.87444613	3.96670722
TIMM44	4.60410116	4.80640319	4.90746634	3.69892285	4.12210807	3.75260242	4.46464898	4.6456978	4.59434879	4.75729679	4.10155573	4.07191191	4.46986739
CCDC61	3.52766261	2.56836287	4.47280848	2.38794723	3.06510601	2.79310097	2.52824854	2.57907062	2.57781247	3.7025931	3.44243015	3.56821919	2.77723024
IL27RA	2.46010729	1.82883991	2.93574759	3.83440337	1.93419085	2.18014985	1.87596737	2.05989969	2.68545293	-1.03123613	2.31109464	3.46168827	2.6497899
ASF1B	1.56401643	0.35538826	2.96359611	3.11350405	0.18029911	0.06852584	0.74615122	-0.14458051	2.17111545	3.00913876	-0.36527473	1.37666057	-0.36527473
TNNT1	0.22669986	0.8025642	1.50043565	0.22669986	0.22669986	0.22808606	0.22669986	0.22988064	-0.2670806	-0.2670806	-0.2670806	-0.2670806	4.27744368
VRK3	5.2356337	4.49915721	5.05696501	4.38000553	4.83626595	4.16037243	5.56400373	4.98691609	5.27967668	5.30541592	4.86659988	4.99327083	5.38579491
FAM32A	5.6604771	5.48142669	5.73665132	5.56680776	5.23252246	5.54102281	5.45111439	5.00856087	5.77934384	5.69349256	5.71831085	5.42862642	5.64341005
PPP6R1	4.19993139	3.88466347	5.60086025	4.0949177	4.06492218	3.09973799	3.91820721	2.7487763	3.72190876	5.02127072	4.7678274	4.42786237	3.654161
C19orf44	4.93542894	3.78921146	4.44160382	3.20146253	4.04392828	3.79383897	3.95950957	4.02532005	4.44322055	3.88708165	4.28350039	2.45074201	4.32371576
MED26	4.12552252	3.64713547	3.47719071	3.43888809	3.4838495	3.60116894	3.26221355	3.45272357	3.07335456	3.59618461	3.40301271	3.77230735	3.61548509
OLFM2	3.66102418	2.44753265	4.64238458	2.02691986	3.23587085	3.1755151	3.57470246	3.92414539	2.76237392	4.5723242	3.51186228	4.89578982	2.29311203

RASAL3	3.46239404	2.66218395	2.98747874	3.66124333	2.19799037	1.49507583	2.46727249	1.83905529	2.2373982	2.20013672	1.83884615	1.87594349	3.88415077
AKAP8	5.89578798	4.99419322	5.73157693	4.7899699	5.1044017	4.6353178	4.91673862	5.14355408	4.48491172	5.15884548	4.82302624	4.86970265	5.24989057
EPHX3	0.28283717	0.01679422	0.72697892	0.43258843	-0.21646105	0.68772598	0.36419378	0.68097639	1.44321409	-0.0768608	-0.0768608	3.33791235	-0.0768608
ILVBL	3.90916293	4.52342148	4.84446757	3.68401129	3.0932884	3.06158751	3.95252709	3.29572352	3.99712713	3.90548918	4.17976545	4.62054839	1.32050839
ZNF419	3.37253195	3.71133415	3.59717042	3.79331509	4.2572251	4.05441995	4.10812289	4.33521086	4.24252244	3.2391935	4.34125292	4.41358022	4.13677365
SYDE1	5.92082718	6.08742208	6.07857554	4.92590447	5.97761092	5.24088706	5.22630902	5.41084634	5.09456558	5.79241779	5.01536033	6.04813867	5.27807627
AURKC	2.39308132	0.81104001	2.50172903	1.39669892	2.8567004	1.66092566	1.73169454	3.27413586	3.33051683	-0.70218598	4.16923324	-0.70218598	2.74617492
POP4	5.0445794	4.21035456	4.7964877	4.91305064	4.47151012	5.19450094	4.28509918	4.54151209	5.34810986	5.06839819	4.19358546	4.63691104	4.9590754
CCNE1	1.12545042	0.306785	1.81090306	2.9262683	0.96388596	1.58258167	1.14224088	1.33700137	2.9500356	-0.74130739	-0.74130739	1.93285456	1.45075983
PDCD5	4.07131807	4.09054562	3.79573126	4.2780878	4.3734461	4.30633027	4.48629579	5.03212986	5.16189778	3.91065313	4.04026112	4.10864721	4.03297347
ANKRD27	5.25910553	5.09351922	5.09802172	4.69024655	5.57704556	5.26367643	5.52380711	5.49619279	5.26677654	5.43797356	5.36128458	5.35039017	5.44224291
RPS16	7.39519718	7.64342599	6.81908007	7.00400183	7.14453888	7.31495662	6.23710094	6.80876937	7.08869244	7.54852126	7.13492404	7.46357235	6.97198816
TIMM50	5.04369922	4.6593151	5.27668264	4.1345382	4.4287101	4.4776562	4.55422348	4.04017993	3.49970791	4.86510214	3.66166137	4.81415251	4.68618614
FBL	6.02582528	5.76740548	6.29335505	6.07445646	5.25089351	5.34786586	5.32381763	5.06563319	5.83088204	5.9337548	5.44253064	5.55842971	5.39982296
DYRK1B	4.02880106	2.92623949	4.33120256	2.09508229	2.20101798	2.14463762	2.95655237	3.15833774	2.97480236	3.57284336	2.86971055	3.4208335	2.9391474
CLC	0.36470269	-0.07553583	-0.07369052	2.12376037	-0.07553583	-0.07450987	-0.07553583	-0.07318165	-0.09896018	-0.09896018	-0.09896018	1.7722825	-0.09896018
CNTD2	-0.0347898	0.46910165	0.47868562	-0.13404301	0.27154029	0.19855807	0.51251605	-0.44194606	-0.03848179	-0.03848179	-0.03848179	-0.03848179	-0.03848179
GPI	7.03877261	6.54922654	6.87099893	6.5160321	6.23288535	5.64496523	6.14197915	5.42458806	5.91923605	6.50901234	6.12018377	6.02635273	6.31104539
AKT2	6.29153612	6.01349556	6.41549815	5.88673847	6.08046995	5.43548574	6.28847247	6.25476208	5.61989397	6.22763542	5.93551718	6.31667921	6.02212656
PLD3	6.15431368	5.13578344	6.01347238	5.07419447	5.07294289	5.25820592	4.68044843	5.38577532	5.16139099	6.09375315	5.57424649	6.02529158	4.903073
PRX	4.1470086	3.07121755	4.63186279	2.94548745	3.40488016	3.37138593	3.7821431	3.7614693	3.19876563	4.03984609	4.35125297	4.26865553	2.46738804
PIAS4	4.46331726	2.83622011	4.92556044	3.3335791	3.5340422	3.72801071	3.67553344	3.9614277	3.88122877	4.35795405	3.71725173	4.44131317	3.69758157
NUMBL	5.76258745	5.45560862	6.54671008	5.10448578	5.11476919	4.50933657	5.37164754	5.34425961	5.14713254	5.44576027	5.65831047	5.00030229	4.63328354
EBI3	3.89368844	3.21097467	2.83073883	1.71772708	1.73279947	1.00247661	1.50802289	0.96198325	2.44777107	3.06293696	1.76568026	2.68020338	2.04755329
CCDC94	4.18624651	4.02443848	4.8457425	3.23429198	3.31533365	4.53898871	3.42931038	3.40964408	3.91438867	5.3937826	2.9828636	4.54094652	3.90697131
TBCB	5.53034891	4.95015418	5.83617466	4.79596617	4.71593633	5.53433026	4.97492501	4.90039568	5.54799536	5.39685086	5.14359437	4.72746885	4.69731737
FSD1	0.2886764	-0.01390099	-0.01136649	0.52995413	-0.01390099	-0.01249184	0.48312672	0.14805768	2.21897397	-0.12180078	-0.12180078	-0.12180078	-0.12180078
POLR2I	4.53740072	2.74501955	4.01422626	2.92895603	3.01637577	4.62460459	2.35030033	2.95469279	3.85137643	3.85487776	3.42543072	2.53845232	1.64649003
CLIP3	6.60800369	5.52287068	6.2961529	5.17050823	5.77018579	5.53162403	5.8581974	5.75533624	6.15817597	6.35360186	5.68323018	5.66416536	6.20058844
SLC1A5	4.30196309	5.13796194	4.17231268	5.18348599	4.07020802	3.53147728	4.16137724	2.80069658	3.24385678	4.16899634	4.11843596	4.21697263	1.92923169
PRKD2	5.32853902	5.1489205	5.17087534	4.75561454	4.94696203	3.94176805	5.08894644	4.63617675	4.56219364	4.51433878	5.51273504	5.27204622	5.20011497
APLP1	4.18678645	3.5058865	3.85091613	2.72703675	3.77241351	4.48693309	3.35979379	3.41264565	3.55446548	3.74862062	3.84891511	4.43524812	3.68871807
CCDC9	5.50789047	4.61634467	6.16477491	4.65244789	4.56636355	4.3463052	4.49400554	4.39544683	4.21219937	5.00667575	3.80703676	4.62023054	4.68495768
HNRNPUL1	7.55679034	6.84860701	7.42618546	6.74428859	6.77656142	6.62018688	6.63170792	6.6112892	7.09497525	7.60104641	6.97247634	6.94933678	6.84864614
FZR1	5.10365086	4.55268418	5.41816515	4.11565651	4.23136415	4.03246007	4.39302952	4.35690021	4.17182822	4.85859569	4.86307503	4.65378167	4.14435757
BBC3	1.75551118	1.94455871	2.08613915	0.70872625	0.50660678	-0.03464939	1.25979875	0.1886006	0.5542758	-0.18169934	2.16576876	2.04701772	-0.18169934
TGFB1	6.92349865	6.36216292	7.56005855	6.76702623	6.32961571	5.21341338	5.82686711	5.60695661	6.15319641	6.42567434	7.17428612	6.12339862	5.44994996
DENND3	5.89844931	5.67557486	4.95166261	5.28681343	5.44286715	4.20591323	5.27371211	5.66468087	5.1434816	4.08993875	5.19053562	5.11040535	5.42462914
CEACAM4	0.96154641	0.40898238	0.95985559	2.32503149	0.22239729	-0.17567637	-0.05468197	1.42581675	-0.32944954	-0.32944954	2.8586335	-0.32944954	-0.32944954
PLIN3	6.39561191	5.86385438	6.9666787	6.27215876	5.01828984	4.97822289	5.50864066	4.92622807	4.98382864	6.28597965	5.13884378	5.23234791	4.62669358
MYH14	5.41367931	4.74522372	5.72338575	3.84455967	4.76420307	5.01172545	4.97423494	4.3392672	4.73170789	5.42974968	4.55869864	4.93309977	5.42012863
MRPL4	4.42589077	3.0619838	5.19201226	3.75559509	3.58862847	2.97653801	3.22457669	3.41973906	3.26672533	4.65132069	3.5982079	4.0181026	2.77516111
SIGLEC8	2.51488995	0.01339482	0.01516358	-0.02577789	0.41490705	0.22808556	0.19052775	-0.07416457	0.06989037	0.06989037	0.06989037	1.80457539	0.06989037
CD79A	0.21444292	2.61583102	-0.25972623	-0.2328814	-0.01192344	-0.26039343	-0.26122889	0.49885622	-0.09978525	-0.09978525	-0.09978525	-0.09978525	-0.09978525
RPS19	6.53980799	7.22461875	6.7824615	6.60786157	5.71212202	5.82520559	5.96931676	5.80498128	6.39646451	6.98502733	6.31431881	6.98256477	5.61922708
NKG7	1.81968967	2.94560504	-1.43637697	1.30828795	1.40859164	0.64640239	0.61378184	0.612104	-0.17148006	-0.17148006	-0.17148006	2.70198278	-0.17148006
ICAM5	0.91371566	0.14957582	0.30904841	0.33709059	0.33080151	0.26402246	0.10812372	0.04192975	-0.11905787	-0.11905787	-0.11905787	-0.11905787	-0.11905787

ETFB	4.39269533	3.96276955	4.94137583	4.02478291	3.82672341	3.64797541	3.99017227	3.84944819	3.86578336	4.67658133	3.27080942	4.52946832	4.52521815
CD33	2.9458429	1.92419407	3.70547505	3.64338798	2.41942153	2.15720682	1.64321142	0.74010626	3.05529008	2.4260415	1.39200131	-0.96643355	-0.96643355
BABAM1	4.85369605	5.27145377	5.65188446	4.90077099	4.81684274	4.8415687	5.04154108	5.19842508	5.17715679	5.72959836	4.96692963	5.09233277	5.07129195
TYK2	5.40679976	4.95215362	5.7564829	5.33600824	4.61492435	3.83465403	5.07257929	5.01697868	5.05701321	4.81898987	5.2044776	5.43249263	4.21836158
CDC37	5.99484052	5.66726327	6.79414505	6.09151761	5.56729846	6.07521686	5.40381535	5.28103237	6.10796727	6.30661266	5.04798853	5.49201308	6.34915475
NAPA	5.94187635	5.64853546	6.22607204	4.79955722	5.52182666	5.53193313	5.38645346	5.36681345	5.61433894	6.44467714	5.03681326	5.62484618	5.83090588
RABAC1	4.76945746	5.27850857	5.39405936	4.52179836	4.35626831	4.59716004	4.06200463	4.5956379	4.62569012	5.34747751	3.15583474	5.1996242	4.67239112
ATP1A3	0.47966806	0.38112864	0.48059764	0.14974289	0.14104238	0.14248849	0.40064745	0.35651628	-0.32560429	-0.32560429	-0.32560429	-0.32560429	7.19255333
MEIS3	1.29342046	2.2443635	1.78251398	3.7856887	2.14816981	0.87423329	0.91283082	3.76117131	0.42191601	3.61541486	-0.67006271	1.54192399	3.17142523
PTPRS	6.98438575	6.80219555	6.34812079	5.58494598	6.36305226	5.93543689	6.47469182	6.6235748	5.86663461	6.74642123	6.40873333	6.63343876	6.42336875
MEGF8	4.56656035	3.91777327	4.97167672	3.49862384	3.81715167	3.38266667	4.0220357	3.63521668	3.72262578	3.90303484	4.56539947	4.58038155	3.6836882
KDELR1	7.14831236	6.62808525	6.63790881	7.25233945	6.50430988	5.86584242	6.54334521	6.33280808	6.22270327	7.06434705	6.40119055	6.48548655	5.87804912
CYTH2	5.58886498	4.63447184	6.03134309	4.92573931	5.0450527	4.53819172	5.20797575	5.17688879	5.07212861	5.8832153	5.24955174	5.31756791	5.15240485
GRWD1	4.82739816	4.15900262	6.01254081	4.65169384	4.44749918	3.84505844	3.98736293	3.52852557	3.36208937	4.65614106	4.35854557	4.987377	4.09846635
GRIN2D	0.71279344	0.07061792	0.70448022	1.43071053	0.16515374	0.0200637	0.43120191	0.26902983	-0.24994487	-0.24994487	0.84608061	-0.24994487	2.01168622
CLEC11A	2.91264756	2.4910165	3.36575872	4.03999831	1.06820936	1.50597414	0.93952582	1.84712098	3.50313404	2.84594531	3.46455447	3.93933377	2.40016159
CCDC114	1.32061374	0.7129807	0.83127662	0.62203344	0.6965291	0.973824	1.52585827	0.72127787	-0.40021853	2.69309489	0.685678	0.64909639	-0.40021853
CARD8	4.57715052	5.39281087	5.07057855	6.11823975	5.24972017	4.63795747	5.60313911	5.26623684	5.22800889	4.600424	5.45304896	4.28620413	5.89591115
LIG1	3.87003139	3.17290488	4.63912278	4.15505603	3.34559218	3.06660533	3.44568612	3.1119245	3.36741544	3.99613915	3.81629651	3.83231786	3.26088873
SIGLEC6	0.61700266	2.15375373	0.42176503	1.2024531	1.68558903	0.59477849	1.75843191	0.56990394	2.57563194	-0.51809667	3.11336578	-0.51809667	-0.51809667
ZNF175	4.69632908	4.58647303	4.63729837	4.9536671	5.28300555	5.35912088	4.981567	5.19941751	5.01093522	4.75801891	5.09448454	5.16775182	5.04457667
PLA2G4C	5.80841738	4.19913814	4.65907329	4.33994751	4.62504012	4.05619734	5.72510484	5.2852322	4.92713147	5.11368074	5.28153815	4.51812026	4.95363724
SIGLEC5	1.37429611	0.61156355	1.22363276	1.33308546	0.84110694	0.87779365	0.82397363	0.61484898	1.8257553	-0.73394764	1.34450139	-0.73394764	-0.73394764
HAS1	0.23624038	4.94397199	0.3494827	2.02208268	0.23624038	0.23725438	0.23624038	0.2385671	1.70908922	-0.03824004	5.04505145	2.40067745	4.45190076
RAB3D	5.76885112	5.24535019	6.04739267	5.03924959	5.24886291	5.12139865	5.65147294	4.78865985	4.57320899	5.10281368	5.18419898	5.03965231	4.95550212
DBP	2.68516619	3.67315157	3.27631696	1.1244348	1.52976666	2.78415425	2.19808418	1.96224243	3.55759668	4.92621691	4.20364184	4.68082319	0.38377337
TMEM205	4.64272173	3.02215797	5.00831494	2.72322326	3.0180777	4.04963513	3.75171103	4.18345188	2.91112951	4.84784714	3.11388492	3.68193901	1.36162918
CAPS	2.5234885	2.06437378	3.00601587	2.09537528	2.57754074	1.54646549	3.19709452	2.51701048	3.46272034	2.98231123	3.50819641	3.02571219	-0.85298354
FAM83E	0.10060248	0	0.22777033	0.15382012	0.39944551	0.15057578	0.15835818	0.12455679	0	0	0	0	0
RASIP1	0.79159699	3.51370414	2.0885722	3.28281913	3.91751984	1.41251234	3.46977465	2.72022658	3.29360835	2.29960933	4.40638513	3.49815945	4.95069871
BCAT2	4.08707625	3.39618009	4.53633476	3.10248895	4.09125115	4.09939675	4.51909292	4.90949678	3.44894165	3.9435072	4.06170756	3.77544835	2.7767311
MIER2	4.1207288	3.73903991	4.90041637	4.0856205	3.98897924	3.50870462	3.95581563	4.11439707	3.28889016	4.30432175	4.71275884	4.32317711	4.07100124
PLEKHA4	6.54012766	5.50177888	6.66481375	5.05621393	4.65401829	4.05580098	5.41034097	4.43801995	5.40779173	5.87004665	4.99208103	4.63858194	3.97841128
PPP2R1A	6.2047967	6.21897051	6.82632762	5.59402357	5.79778351	6.00626522	5.6036448	5.86414251	5.73340879	6.60651951	6.28687625	6.77873208	6.17454648
TNPO2	5.90663987	5.52051388	6.17093612	5.13651918	5.60029292	5.12289492	5.56400496	5.37553692	5.74947979	5.73115819	5.72809985	5.93882786	4.88374264
GCDH	3.33726452	3.21616273	3.6053214	1.13396646	2.99534879	3.24893059	3.00593909	3.4221475	3.5361189	2.18553693	3.2719332	3.90078692	3.97264157
LILRB5	3.90385046	2.87751977	2.68209465	2.57965131	2.82527381	1.97652841	2.74671399	1.2003134	3.85544354	3.26631537	3.80347832	2.50175082	0.87799279
DNASE2	6.17342361	5.58124414	5.62774595	4.95974386	4.85881516	5.29910379	5.17098009	5.08179049	5.06727258	6.35071966	5.0456583	5.85852756	4.96166537
MAST1	0.15464333	0.00025555	0.17434723	-0.0290474	-0.06636546	-0.06493485	-0.06636546	0.02564997	1.8977698	-0.10055943	-0.10055943	-0.10055943	-0.10055943
LENG1	3.96313778	3.83451843	3.75038379	2.06515152	2.65844416	3.11572187	3.31455637	2.72965288	4.27639074	3.25492588	4.28160151	2.83423956	2.06002915
PRPF31	5.63090971	5.23216151	5.79287851	4.9267961	4.88507824	5.06554553	4.97350934	4.81252787	4.94843527	5.28141996	4.21401294	5.01906833	5.43325474
TFPT	3.98637129	3.4659037	4.72387227	2.44844991	2.75418825	3.41920298	2.51602029	3.18949477	2.99235676	3.87698696	0.20096929	3.18034418	4.02155234
JAK3	5.10042316	4.74076547	4.31848599	5.00675025	3.3939	1.91979139	3.632658	2.88338048	3.71578254	1.55712099	3.21461464	4.05292037	1.96095953
RPL18A	6.87269962	7.13905329	6.57054876	7.57430188	6.68940282	5.96207778	6.4160539	6.30933493	6.41739049	7.3935552	6.56764214	6.96170642	5.74798873
KCNN1	0.08759627	0.24273147	0.04598132	0.07662726	0.06759389	0.15541388	0.04338878	0.14925992	1.89309258	-0.00291869	-0.00291869	-0.00291869	-0.00291869
ARRDC2	4.98469022	5.30207299	5.17857321	3.89802785	4.76967199	5.25969917	5.24339007	5.5295781	5.0336328	5.69557231	5.17768386	5.03203689	4.16592894
PIK3R2	4.1379785	3.72868172	4.26000766	3.73616068	3.78047953	3.72999713	3.88105447	3.86757616	3.71317751	4.03583863	4.01859887	4.18367383	3.10941779

RAB3A	3.13594681	2.60358913	1.90420547	0.70821396	1.38921906	2.51451789	2.3095024	0.8644909	0.75750777	4.01066768	1.11942037	3.51126042	3.52717414
PDE4C	0.67929781	0.74784631	0.24152477	0.65305444	2.26165793	0.41140041	0.20812811	0.87275712	2.26894286	-0.40607267	-0.40607267	0.74984005	2.24324948
ISYNA1	5.09543359	5.68458024	4.35547961	3.30005292	5.26772928	4.84069117	5.912718	4.1873447	4.99160358	5.62951829	5.34738533	5.83196108	4.43741951
ELL	5.70937246	5.01139912	5.66702063	4.71031184	4.6818784	4.32534406	4.92180672	4.84811737	4.62551267	5.39863852	4.99758408	4.60704641	4.31717164
CRTC1	4.64921512	3.90337468	4.83327727	3.53709259	3.56667831	3.21108879	3.84778852	3.86004318	3.77611982	4.25692933	4.06591479	3.62196098	3.28913453
COMP	8.94941814	4.66663528	6.55275455	4.69307663	4.73339354	4.66604049	5.99269772	6.91987994	4.40790215	5.70539738	5.52819861	7.24286558	7.38452021
UPK1A	0.50929992	-0.04562922	0.3018521	0.39678423	0.2078394	-0.04425538	-0.04562922	-0.04247679	-0.05365228	-0.05365228	1.65539872	-0.05365228	-0.05365228
COPE	5.12441668	4.88559036	5.64399913	5.23639211	4.65219502	4.44502954	4.05161463	4.40840864	4.72493021	5.45203437	5.22305538	5.45430753	3.85283209
DDX49	4.65048185	4.80324075	5.42227872	4.69526347	3.81928408	4.2453806	4.21464007	4.00007619	4.34349905	3.83971125	4.77699158	5.35779552	4.30457774
ETV2	0.84759006	0.26955504	0.46382995	0.26955504	0.84646579	0.27097032	0.26955504	0.27280255	-0.27651561	-0.27651561	2.73654505	-0.27651561	-0.27651561
ARMC6	3.64937739	2.9622954	4.93716188	3.85701791	2.44061358	2.34877088	3.10545994	2.57875954	3.44802867	2.23797672	3.49047468	4.36369189	2.23098148
TMEM147	4.93481919	4.55296046	4.67941148	3.82395745	3.69814221	3.99838071	3.7424529	3.36593506	4.4022646	4.96481538	4.11932661	5.05067025	2.92169745
TMEM59L	1.43886241	0.83393185	-0.17142996	1.06386893	-0.25980235	0.22937781	0.590113	1.8278885	-0.17651259	3.678633	-0.17651259	2.75600019	-0.17651259
HAMP	0.01863552	1.26609646	0.06047791	-0.16210474	-0.52384985	-0.40364706	-0.42024205	0.01437763	-0.2192868	-0.2192868	-0.2192868	-0.2192868	-0.2192868
USF2	6.06557537	5.90213901	6.20799698	5.24155777	5.62873684	5.45434432	5.41678754	5.41519009	5.94342578	6.22507078	5.6375057	5.7493185	5.82562288
LSR	3.61647218	3.27576929	1.95088362	1.29566305	3.35748696	2.47575168	2.1054049	3.32357057	1.94198436	3.05737111	3.26929629	3.09854718	-1.02157089
FKBP8	6.86201136	6.89022655	7.33932447	5.6298823	6.27706778	6.25243554	5.97642729	6.21711962	6.13076911	7.18945937	6.53381964	7.13927497	5.79546148
SUGP1	5.46780403	4.23451787	5.56814934	4.47338655	4.92645261	4.70270583	4.62516968	4.51700656	4.80356853	5.30655092	4.48670749	4.68478097	4.31885523
HPN	0.20193888	-0.1331903	0.43374825	0.3631142	0.29777999	0.11458684	-0.1331903	-0.13012996	0.70634397	-0.05467101	-0.05467101	-0.05467101	-0.05467101
ZNF14	4.01247598	4.3066534	4.21336885	4.01014795	4.47666593	5.25157145	4.53840132	4.71746057	4.89162691	5.05483658	4.27636455	5.0633023	3.7163653
SCN1B	4.28739915	3.71040763	3.7941357	2.58944162	3.5390483	2.51955696	3.96545616	3.07945629	3.03625821	3.26615732	3.03334226	1.99584772	4.75850045
PBX4	0.85261239	1.13909413	0.5090999	0.00544034	0.25693956	-0.14545081	0.09116003	0.2404752	1.16807377	-0.11941193	-0.11941193	-0.11941193	-0.11941193
ERF	5.38849955	4.63942555	6.07957866	5.19793573	4.25703359	4.2307798	4.69105893	4.14440337	4.91836878	4.59456736	4.89197287	5.10594302	4.19453324
GSK3A	6.11145779	4.87730951	6.02920125	5.24858959	5.45381215	5.4155937	5.58351817	5.09764196	5.17523238	5.84364664	4.92221211	5.72893736	5.0710661
ATP13A1	5.15159185	4.50280566	5.49797061	5.03118243	4.61608457	3.82873023	4.65892486	4.77119632	4.41973046	5.0054315	4.59000973	4.88338596	4.66983236
ZNF574	4.94449741	4.56367589	5.23963134	3.48287758	3.82607936	4.09307491	3.79426446	3.80437236	4.02627674	4.31510966	4.15900959	4.47825801	3.23007515
GRIK5	5.5457911	4.38348045	5.54972605	3.40539392	4.57085537	4.42400295	4.99114667	4.98678761	3.59258044	5.38665812	4.58550068	4.4591493	3.80441683
SIPA1L3	5.56307428	4.69797869	5.75971787	4.6038279	4.51468095	4.48781448	4.62570728	4.34746418	4.70916	4.96711061	4.53873981	4.80223276	4.1980967
ZNF85	2.8646619	2.52297937	4.02885161	4.37121573	3.35707115	3.52272151	3.15659966	3.7658917	3.8241274	1.60031036	2.72281324	3.74712068	3.61228696
ETHE1	4.09590311	3.25347544	4.88779925	3.46603528	3.10219689	3.12415138	3.74171854	3.02656659	3.02334111	4.12235857	3.45793962	3.52430712	2.22766866
CADM4	4.21761276	2.71695773	3.25884032	2.19513012	3.14156779	4.22056376	3.62138167	4.15258021	3.88173046	4.45157788	2.55413269	3.50555101	4.2195576
SMG9	4.91850537	3.79332059	5.69272459	5.06088982	4.42967463	4.10226231	4.673424	4.33986567	4.40196912	3.44311411	4.38138692	4.18926074	4.11433841
AVL9	5.52311686	5.71769836	4.93971988	5.91932878	5.84190297	5.55800588	5.60575751	6.1396869	5.45253645	5.58703415	5.31052248	4.97915738	5.62058843
RUNDC3B	1.94167986	2.40922171	1.98424612	2.467152	2.71687578	3.66749108	3.40650045	3.53943279	3.83099759	1.01069069	4.58727523	3.55952492	2.85278969
GTPBP10	4.94560189	4.94247042	4.34538622	5.03834144	5.30350957	5.25375579	5.17418748	5.22848058	5.44202919	3.96793736	5.18625084	4.99794617	4.78200064
RASA4	2.94365195	2.98038534	3.13015344	2.39347732	3.09021002	2.29990463	3.45017157	3.0775002	2.44383531	2.66053328	3.12979065	2.46214977	2.49373544
CDK6	5.56026501	6.44926963	5.96077892	7.01265465	6.46869416	6.58955024	6.35470548	6.63163265	6.80160188	5.94889192	7.12461001	6.97886117	6.13635699
PMPCB	5.31835615	5.84565146	5.37880674	5.99532151	6.14123782	6.14633608	6.07679263	6.1279146	5.98111139	6.18194708	5.95693082	6.05013188	6.27898546
DNAJC2	4.07540386	4.49770702	5.14284577	6.01696784	5.2573203	5.01322424	4.90236886	4.42654813	5.51906079	3.56997898	4.35598352	4.91157687	4.2676306
TFPI2	5.03312536	4.19520842	6.12161518	5.61380914	3.81591227	3.6790398	4.3715227	6.14108903	3.7021569	3.95777035	4.49883657	4.52605329	3.69089563
BET1	3.74843291	4.59605079	3.96848271	5.21380481	4.83169798	5.13205149	4.94728356	4.69794368	4.64975066	3.75464873	5.05779603	4.68171673	3.7438764
NAMPT	7.3733364	6.83023026	6.12055224	9.70571714	6.22490739	6.35106025	6.9908581	5.4833754	5.86127365	4.73331263	5.78791361	5.7479107	6.18378851
TWISTNB	4.13327206	4.8226005	4.34356874	5.36442834	5.41303622	5.78395921	5.20259226	5.48044197	5.31654611	4.17849856	5.14273564	5.56689262	5.22056031
PIK3CG	4.14402602	5.09609862	4.33290692	5.1038294	3.98496821	2.94492026	3.52798827	3.12175498	4.67192014	4.53551275	4.83882267	4.08440166	3.29217025
PON3	0.37746444	2.13741789	0.35022978	1.57414565	0.63502695	0.69234473	0.39917435	0.62928966	1.75422198	-0.21235285	3.45500189	-0.21235285	3.26509393
PON2	4.39933657	5.43128751	4.4744303	5.58231322	5.22599313	5.47074506	5.60941984	5.65516646	5.46492684	5.20440557	4.87189827	5.14322204	5.27966627
ITGB8	3.22521467	6.42987675	2.97844593	4.29154783	3.93496321	3.96447439	4.24791769	4.58888889	4.67721054	4.2121519	5.68016186	4.96797134	5.28335827

HBP1	6.59962017	6.69886157	5.9000071	6.77204009	6.67463964	6.7537074	6.93173002	6.9191356	6.42546103	6.5510548	6.87165483	6.79991491	6.12622389
DUS4L	3.12028134	3.3264055	2.70078338	3.26992803	4.3608719	3.58480996	4.11310544	3.81066547	4.03589187	2.23485148	3.13101466	2.53559881	2.86592111
SP4	4.16342481	3.9902257	3.27005009	3.57635231	4.35546708	4.86546435	3.87139076	4.61063741	4.57358901	5.20963274	3.92578683	4.21462773	4.17271337
WDR91	5.42848377	4.35320561	5.13749132	3.86959724	4.07772214	4.46996735	4.44727707	4.35700412	4.58489536	4.2931065	3.73261603	5.13314185	2.99189716
DNAH11	5.00780551	3.94029242	2.84992389	2.87912962	4.32804455	4.73494011	2.9727496	3.69375728	3.87248909	3.05770744	2.32366322	2.29495981	3.68382557
CBLL1	5.63745386	4.96590152	4.98250265	5.30510601	5.83480372	6.12373039	5.27955774	5.25779542	5.45519423	5.28555661	5.09423889	5.1077244	5.04084924
DLX5	0.703513	0.37369697	0.24828721	1.79535715	1.28193256	1.07158092	2.07390449	0.24896048	3.55593778	-0.23962356	-0.23962356	-0.23962356	3.96064132
MTPN	-0.05978563	1.57817779	0.67548569	2.65497001	2.36604042	2.55104549	2.09219377	2.00055006	0.59765488	0.59765488	0.59765488	0.59765488	0.59765488
PTN	5.1498399	4.63764565	4.68668799	5.28231901	4.72933243	4.33314841	3.72561262	6.5204377	6.51880465	5.49644193	4.8970891	5.83727727	4.47958427
MPP6	5.30918375	5.46961096	5.61978907	5.66107622	6.22485759	7.01001997	6.23815271	6.23245051	6.51537229	6.76614052	6.17719137	6.17871556	5.18524305
ZC3HAV1	6.2659742	6.53736793	5.84866902	6.90617687	6.47561955	6.89746303	6.5024495	5.95279081	6.29637043	5.89710251	6.40777341	5.88802947	5.86661551
TTC26	2.14955584	3.20432252	2.6578985	3.14550709	3.39520985	3.35288262	3.6498969	3.83839364	3.38075346	3.84139147	2.39789015	3.05832837	3.70715312
OGDH	6.60331658	6.0204051	6.94539219	5.87102733	5.9806122	6.58520652	6.67300422	6.66230647	6.24559307	6.98884163	6.38168521	6.62437491	7.165733
ADAP1	2.3208164	1.46352782	3.2275538	1.83849995	2.72352268	2.97219175	2.08736733	2.31268338	3.36406712	2.41508833	2.09673792	3.7375535	2.90996158
TFEC	3.06492271	4.24831771	3.10355009	5.26529996	3.68646164	3.15360656	3.66560549	3.02613653	3.80590023	3.4028911	3.18760836	3.94487823	2.82499192
H2AFV	6.48827053	7.18155032	6.44564657	6.60696295	7.18367498	7.1975605	7.25633935	7.33838636	6.78059357	6.89458329	6.79053844	7.21585321	7.05091132
CAV2	6.03635933	6.49348734	5.79296157	5.72422435	7.33214071	7.24515717	6.81795679	7.19921713	7.17461499	6.27902706	7.58106979	7.24824831	6.60776278
CAV1	6.6358057	7.55556843	6.27801559	7.17772357	7.45529792	7.98645277	7.43893812	7.68698823	7.53110792	7.54101266	7.82343373	7.92686269	7.38613982
MET	6.22761923	6.80263295	5.8938305	5.7481993	5.6565643	5.59601194	5.51638793	4.4030087	4.91284102	4.11371241	5.89179039	6.11075162	6.44569637
RNF32	0.36925029	0.30512727	0.20138219	0.33212778	0.34602605	1.22551584	0.25856468	0.33523926	1.28774033	-0.22894347	-0.22894347	-0.22894347	2.43443865
LMBR1	5.34011014	5.53414771	5.05958023	5.7212775	5.97708211	5.7051492	5.88562517	6.03022054	5.68395896	6.00227554	6.08404089	5.71754454	5.66251262
WNT2	0.93111408	1.96661969	1.77498132	2.39949446	0.65394556	0.60367438	0.58513359	0.48310376	-0.71432309	3.57805028	3.12744854	-0.71432309	1.05121116
HOXA1	0.90213215	0.56041101	0.53863331	0.77711487	1.63513113	2.03301364	0.39176194	0.59387986	1.77272906	-0.35546754	-0.35546754	-0.35546754	3.69889539
DNAJB6	6.61552507	6.30978364	6.59161675	5.96447668	6.61116682	6.6449182	6.995155	6.6720836	6.65485089	6.88742831	6.27630624	6.85343718	6.75737255
HOXA2	4.19293581	1.56505132	3.63419752	1.96370036	3.50712535	4.77615723	3.86044368	3.82392672	4.35204961	4.8197317	4.02417955	3.5898318	3.75351258
HOXA3	4.77114196	2.15927389	4.57371746	2.87408127	4.59013305	5.36743222	4.5460781	4.2895896	4.55335066	5.87581908	4.48667627	4.90041735	4.01499505
LFNG	1.47758447	2.14100249	3.14873943	2.7715663	0.90564851	-0.320873	0.9049896	0.8077043	-0.39051382	3.80458262	2.58162442	2.30593673	-0.39051382
HOXA5	-0.324417	-0.324417	-0.32275494	0.77310677	0.09422959	2.2294433	-0.1949112	-0.3222966	2.78807153	-0.20441964	-0.20441964	-0.20441964	-0.20441964
BRAT1	4.32299808	4.08352385	5.15786645	3.54335656	3.22346473	3.22085627	3.77635795	3.65806198	3.60548484	3.84177959	4.32528392	4.58304374	3.16674241
IQCE	4.97470484	4.87603896	4.88756942	4.39290426	4.36252082	4.09748446	4.53222569	4.20728445	4.46078688	4.66560927	4.87699055	4.59861902	4.31856444
ANKRD7	1.07039935	0.44224753	1.57021054	0.84558636	0.72405824	2.18450185	0.96251513	1.27004003	1.98872204	-0.52766519	4.0921428	2.59659185	-0.52766519
VIPR2	5.16629204	5.24002999	4.96483401	2.79410577	5.0990785	4.74953307	5.30418657	5.08843619	4.51149926	5.63406533	5.37936826	5.39094392	4.91975479
TSPAN12	2.82261989	4.29728317	2.93588107	4.41190309	4.74105337	4.91907031	5.1371602	4.59207529	5.01163866	5.14845034	5.39454005	5.42458886	5.01671454
SSBP1	5.55233557	5.30486227	5.89780231	6.24673109	5.89250216	5.90512975	5.49033704	5.35090773	6.22700935	6.23733085	5.55982732	5.4412109	6.05799855
HIBADH	5.17646739	5.39745167	4.84136655	5.70258859	5.63037755	5.75081902	5.83215114	5.6350658	5.786313	5.85111056	5.21830058	5.84530759	5.81011167
TAX1BP1	6.64774721	7.14908233	6.5311152	7.20981033	7.21438224	7.94249396	7.185149	7.27087479	7.77654844	7.38525004	6.72219761	6.95404662	6.90647401
CPVL	5.22609747	5.35438152	3.65245373	5.98002385	3.86701172	3.05780977	4.01123006	3.09396742	4.63080465	5.00563052	4.4474117	3.73153928	5.35844902
CHN2	3.4787405	2.70732959	1.8809849	2.51035327	2.4744271	2.16898728	2.60430219	3.10795079	0	0	0	0	0
GRB10	5.90466362	6.00222918	5.56804287	5.72720889	6.55413297	5.6201093	6.32783408	6.71623899	6.07900127	6.04012998	6.4313345	5.91754847	6.21008397
ABHD11	4.36762302	3.34279084	4.94719417	2.94161963	3.57246711	3.34370781	3.05239853	3.97554117	3.73282491	4.19784262	3.51740386	3.63244115	4.6362015
COBL	0.98631341	4.17961978	1.05039541	1.3300197	1.38911751	1.18095492	1.55732464	1.48267238	3.05291672	2.83405003	3.76060443	-0.72544251	4.57464665
FKBP14	4.83652342	5.06377388	5.36907902	6.74539229	5.47689525	5.60757609	5.24564489	5.26110996	5.84498647	4.8963648	5.95331948	5.81876745	5.09888799
PLEKHA8	4.76558264	4.9176077	4.28398524	5.22986922	4.89613733	5.43390993	5.13179083	5.16109974	4.67194923	4.34860498	4.74715343	4.58238898	5.09915034
STX1A	5.00067864	0.95063441	5.87796991	2.82629639	2.05527744	2.60871681	2.84029525	2.61607697	2.64023034	2.39529056	3.03913444	3.46130288	2.86933221
NOD1	4.60191638	5.348155	4.65305815	5.36480722	4.37677341	3.94795221	4.19676381	4.39609717	3.84734395	4.50156591	4.79162237	3.60478737	4.97457881
GARS	5.68232556	5.71699126	6.1638713	7.57045579	6.11063596	6.16269034	5.95442729	6.10137518	5.83723401	5.88361706	5.61643343	5.57858233	6.37494373
EPHB6	4.6907059	4.90074421	4.51428359	2.64900002	3.74929001	4.66123637	4.73454655	5.00653511	4.30849164	6.03062379	4.02643277	5.66250283	4.15804167

GHRHR	0.50161137	-0.2092031	0.03020358	-0.2092031	0.18707201	0.46533327	0.23232127	0.03313514	0.06892906	0.06892906	0.06892906	0.06892906	0.06892906
CASP2	4.98940794	4.98829143	5.19621742	5.57542483	4.78034465	4.29423097	4.69977209	4.82885913	4.48981934	4.44505431	4.98403455	4.44175641	5.01690042
CHCHD2	5.62475044	6.72989838	5.9417013	6.0416987	5.67795808	6.09204443	5.99112383	5.93453817	5.93312497	6.779568	5.90952063	6.50165195	5.790611
HSPB1	7.53876526	7.11289839	8.59158743	6.1372624	8.15674411	8.21438359	7.14755826	7.64817844	7.63015587	8.92789258	7.4665477	8.12728454	7.00264155
NPTX2	5.64556647	4.88645606	5.35083001	5.35690504	4.73938869	5.83392081	5.97851118	5.00553106	5.26055008	6.82185551	4.08714443	5.90738039	4.81092297
PDAP1	5.42001191	5.59984741	5.89258009	5.24240483	5.24609865	4.92605598	4.68124686	5.03546354	5.28888294	5.7816112	5.05151246	4.82297654	5.04904564
BUD31	6.11346858	5.79540879	6.01120706	6.37811449	5.78366994	6.58368041	5.97723542	5.08706202	5.77984346	5.41463235	5.05392125	5.33421682	5.76359119
PTCD1	0.56174677	0.05340933	0.53533327	-0.42706879	0.09163668	0.87378209	0.3840695	0.43592305	0.03294623	0.03294623	0.03294623	0.03294623	0.03294623
CYP3A5	0.45272534	0.96043633	0.45531666	0.61371807	0.60584783	0.65970055	0.45272534	0.45603126	-0.09498789	-0.09498789	2.9593723	-0.09498789	-0.09498789
ZKSCAN1	7.03358565	7.20491433	6.85651755	6.96200754	7.26879167	6.97186624	7.25165266	6.97858772	7.01711376	7.01873334	7.21941604	6.67545518	7.2060088
EIF3B	6.74997743	6.4729478	7.13409099	6.77502667	6.00528852	5.95675387	6.5170927	6.27996198	6.14848564	6.33082691	6.48993324	6.51743254	6.48057606
SNX8	3.87662833	4.14491514	4.59864648	3.99277168	3.28551368	3.07557579	3.36198474	3.42801946	4.07926557	3.85268327	3.80958673	3.24990569	3.58398958
NUDT1	3.52674228	3.02859129	4.68922708	3.08961983	1.40898893	2.11379276	2.62304077	0.45853625	3.71663394	3.09798567	-0.92098653	2.11858815	0.99330904
PTPRZ1	4.27388978	6.4227198	3.58284329	3.53531591	5.96146506	4.58895336	4.2529856	3.79241814	4.77384858	2.18325427	5.63532447	6.22041962	4.51529094
TAF6	5.76088209	5.27905601	5.85885844	4.90893818	5.29974142	5.09067567	5.26801579	5.13282476	5.34013658	5.82343718	4.92189059	5.35035443	5.01716186
WASL	6.14440618	6.56832461	6.06492734	6.32046363	7.01755141	6.99349047	6.77608081	6.9432075	6.93836406	6.38338739	6.88369564	6.56287577	5.79042557
AIMP2	4.54138902	4.17358575	5.61539016	5.07809272	4.17188978	3.96036613	3.77568093	4.18129579	3.85908415	4.96708563	4.00393959	3.77846328	5.12411271
TFR2	0.68069296	-0.18353787	0.0724062	0.36245955	0.0402076	0.17408487	0.02370464	0.22595604	-0.15011502	-0.15011502	-0.15011502	-0.15011502	-0.15011502
FSCN3	0.08062014	0.25646901	0.41087628	-0.26761033	0.15881678	0.97183967	1.46610622	0.23323709	-0.14886196	3.36470704	-0.14886196	-0.14886196	-0.14886196
MOSPD3	3.46710764	3.63372792	4.66940404	1.67397053	2.44843389	3.06312387	2.41647633	2.15888019	2.3049707	3.61024572	2.04798188	3.5837777	-0.84220796
PCOLCE	5.6317335	5.89220248	6.30692174	7.08176446	5.04662419	4.84275343	4.84523063	4.71929477	4.79037899	5.86321202	5.83530847	5.64114006	6.12493439
FBXO24	0.93732761	0.30633872	0.54882351	0.1811045	0.47099607	0.87396606	0.45445051	0.84449858	-0.20303375	-0.20303375	2.26330079	-0.20303375	-0.20303375
RBM28	5.00956245	4.92460016	4.86484131	4.92373268	5.26344187	4.77911568	5.0407287	5.05914317	5.08355988	3.88192317	4.97122113	4.53810158	5.15959709
USP42	5.26385865	4.91851014	5.00576273	4.71754585	5.33368784	4.84831227	5.10124478	5.41157544	5.04380873	4.62371941	5.09870596	5.10624308	5.12885322
IMPDH1	5.35351081	5.23267206	5.90587905	5.55668822	4.76255082	3.97915217	4.80283744	4.37435085	4.71242569	4.29874425	5.31881856	4.52723204	4.33106898
AGFG2	4.60600488	4.22200066	4.85977933	2.85759615	4.24397583	4.44491331	4.40248193	4.27554779	3.12025585	4.71502598	4.44207658	3.94680833	4.29461635
LSM5	2.57277608	3.46672266	3.88029761	4.03841925	4.34551998	4.68177177	4.05234348	3.78696418	4.67812053	2.79197211	3.58851318	3.46692215	2.74996871
SERPINE1	8.53065783	6.62261888	10.2050345	9.38926787	9.31139426	7.3233799	7.10102632	8.20221073	6.50708246	5.9858289	7.51569742	7.28660979	7.63022044
AP1S1	4.66878566	4.1793438	5.28809175	4.91124741	3.96930007	4.08547527	4.27880563	3.60457904	4.47770115	4.10678356	4.29345604	4.06816266	5.06893509
C1GALT1	4.73590618	4.7538079	4.09014555	6.46925475	5.34090416	5.39231702	5.52687707	5.58159729	5.05289076	3.77867114	5.31288431	5.00595901	3.56741378
PLOD3	5.46870659	5.15089916	5.95946097	5.95828304	5.05535457	4.50600725	5.20531579	4.56602314	5.09882452	5.48187687	5.47345693	5.27037362	5.42036283
RPA3	2.67393368	2.77168153	2.80709205	4.14926947	3.04715695	3.41149263	3.77174967	3.04817877	2.37316257	2.09412898	3.17511106	3.41169361	2.6277802
ZNHIT1	4.79601416	4.7304378	5.47387514	4.78146369	4.00720882	4.9442524	4.30971062	4.32506303	4.62799906	5.74744502	4.40739575	5.18850927	4.34208915
CLDN15	2.92250218	4.85794615	2.86460032	2.19948258	3.12359894	1.65486141	2.63747565	1.96857382	0.2538791	3.74505305	3.22552228	3.51583024	1.3732403
GLCCI1	1.57184168	0.80213074	0.9023059	0.59368966	1.31835254	1.69034194	2.39113198	1.51410548	-0.12161225	2.88976689	-0.12161225	3.00844224	1.70734887
PHF14	5.17124587	5.48882049	5.11660443	5.56461995	5.14221196	5.86692839	5.18858969	5.44076166	5.54938014	5.71859713	5.09300996	5.22474666	5.26803706
NRF1	5.16895398	4.71390427	4.51595893	4.04808746	4.76423376	4.78683875	4.60694597	4.96342942	4.81735422	3.98015499	5.12309016	4.51846493	5.09482472
TMEM106B	5.32262719	6.2285384	5.3343013	6.27329395	6.76454297	7.13833356	6.66935299	6.66913263	6.93278998	5.38011646	7.03938641	6.90648971	5.32699189
EZH2	2.15323199	1.10979565	4.25071007	4.9135	1.81698614	1.90901451	2.42005577	1.9073817	4.08627987	2.10683568	3.86042105	-1.06942399	2.82634507
CEP41	3.47787769	3.86901504	3.9765186	4.53043962	3.10506053	3.99877294	3.17253994	3.51194581	3.73985313	1.8414336	3.54324141	3.77051193	3.38073371
ZNF862	5.26654366	4.59566605	5.02132398	4.06321197	4.54717936	4.26960037	4.78625492	4.65510593	4.45951337	4.58261794	5.32027537	5.24644386	4.38843592
SFRP4	5.54059141	7.55038311	4.78582202	5.76213622	6.29738055	5.6971805	4.36140788	5.77800755	7.20870672	6.37099539	7.18492808	6.47661994	6.41693314
MEST	0.01337252	1.82908633	-1.00654386	2.36675703	0.25423776	0.28905546	1.195269	0.00437837	3.6407372	0.22171091	0.22171091	0.22171091	0.22171091
MEOX2	4.28091948	5.99137725	2.73439817	4.78959526	4.63006215	5.46067161	3.81583578	4.27810629	5.51784405	4.86194002	5.40458806	4.39790454	5.63784488
ANKMY2	4.84859907	5.25830126	4.36957087	5.03020165	4.98878987	5.49397924	5.16120013	5.32893605	5.22679093	5.13867011	5.11475832	5.42898621	5.18498201
ACTR3C	1.77629746	1.90914583	0.56210721	2.19383374	1.02922311	1.82357718	1.7548419	1.95842658	-0.64200079	-0.64200079	2.95109845	-0.64200079	0.38958693
POU6F2	0.89441915	0.49213225	-0.00587597	-0.2816108	0.88191274	0.06442107	0.09853833	0.0422195	0.18275425	0.18275425	0.18275425	0.18275425	0.18275425

TSPAN13	1.07389994	2.49277214	1.76288789	3.92865489	2.42189953	2.22082783	2.37877244	1.50915383	3.6711364	2.1028576	4.30716373	2.70106893	4.01976291
RARRES2	4.77893395	6.26016601	2.94381807	4.67016749	4.27414565	5.36298484	3.81277492	3.92024298	5.47347996	6.34854656	5.35687565	5.88740254	2.8315072
AHR	6.4516106	6.75233713	6.76803115	7.59670297	6.44783244	6.30614429	7.01537113	7.20695343	6.92754708	6.49008805	6.91914649	7.12768996	7.142518
CHCHD3	4.81998492	4.8055224	5.66384473	5.67493154	5.36044711	5.7960345	5.55908984	5.18259968	5.46514634	5.41987121	5.21566307	4.46239816	6.37978772
GIMAP2	2.9542636	2.94891733	3.19127133	4.65169404	2.78798409	1.68332819	2.65730575	2.50899766	3.80555691	2.36511024	2.93316828	3.46297915	3.73872162
TMEM176B	5.08371396	5.61804748	4.3709732	5.92663801	3.77764435	2.76765381	4.12352451	1.90864557	5.01730946	5.64053736	3.64110278	4.04420222	5.65742307
GLI3	5.903979	5.15313655	4.72370816	5.13054671	4.70180956	4.73039749	4.93357989	4.77128949	4.75529617	4.40763284	5.08686379	4.61771059	5.32249655
PSMA2	3.75180989	3.95460156	3.70231193	4.58128008	4.99647316	4.68557401	4.88162535	4.79305517	4.68331997	4.39771143	4.16042062	5.21082268	3.33149957
MRPL32	4.33151059	5.70640662	4.41876066	5.30068128	4.76075456	5.66380694	5.16341016	5.41613694	5.439792	4.99839154	4.592651	4.47701379	4.95466163
BLVRA	2.50425699	3.88332485	3.62427167	2.35266567	2.54104196	2.91891362	1.8715537	2.02038672	2.76391119	2.98183903	2.1794055	3.99989169	3.18477915
URGCP	5.92978143	5.55330405	5.6918182	4.68290307	5.57166958	5.3311166	5.44632209	5.38327143	5.22072447	5.54354162	5.63875683	5.38550017	5.7267981
RHEB	5.23443465	5.18799219	6.09117052	5.76178033	5.9523701	5.79600019	5.91704247	5.95109299	5.65552036	5.01884047	5.74879522	5.50798554	4.97764068
PRKAG2	5.80751577	5.67746673	5.06915071	5.64144497	6.42351418	6.55678156	6.5265793	6.51458563	6.20009441	6.22827738	6.13897423	6.20835189	7.31318069
AEBP1	9.30917025	8.53687963	10.0490428	8.13030967	8.41766551	7.77177416	8.55782307	8.4348718	8.62724996	8.61184413	8.75133107	8.51646115	8.14404741
POLD2	5.37202422	4.88775132	5.84616721	4.50855825	4.87405826	4.92698298	5.28216915	4.68815413	5.07079142	5.20562354	4.22599442	5.09066102	5.00284073
MYL7	0.85427004	0.50038071	2.37005071	0.50038071	1.37135732	1.64159968	1.06554303	1.25675404	2.7896325	2.16310759	2.13963212	2.4343854	9.70745338
GCK	2.48574425	1.36498362	2.07322735	1.48853341	1.90300863	2.54746747	1.72087065	1.6157572	3.00245054	2.27232124	1.88679905	3.07453818	1.76662274
BCL7B	6.27034812	5.50341373	5.9638177	5.16089764	6.09060849	5.40445044	5.79321047	5.66586222	5.27141067	6.07279818	5.26166827	5.46322476	5.26556508
YKT6	5.83209543	5.88225227	6.42943779	6.446452	5.62050195	5.20949268	5.45333787	5.16845096	5.51503303	5.840473	6.20701683	5.88886292	5.94447992
TBL2	4.92695311	5.06323349	5.60677406	5.61452903	4.67753342	4.49088778	4.97378209	4.81056439	4.89399968	3.75771871	5.09351112	5.1787599	4.80337298
CLIP2	6.35681961	6.10123776	6.51930671	5.1616008	5.97594157	5.598562	5.41748408	5.4397377	5.80599815	6.84179065	6.32394441	5.84350408	5.33828258
EIF4H	6.71240509	6.4257429	6.11318002	6.38765172	6.00567427	6.00850659	6.3809284	6.16776345	6.21441564	6.17004076	6.33671539	6.30511727	6.40767496
LIMK1	4.55914867	4.01932637	5.20753571	4.55256073	3.8045809	3.00267396	3.4341774	3.36962963	3.3181542	3.24804814	3.08087995	3.59390656	3.76870907
SLC1A1	2.98000972	2.94905416	3.90378668	4.70806461	2.80513488	3.93580746	3.71023566	3.01662402	2.64309418	2.46718198	1.58457972	3.24043453	3.39867646
FKTN	4.36815882	5.55506541	4.8072021	5.71360169	5.38298375	5.52715667	5.0529246	5.35877049	5.21114837	4.33638255	5.00555185	5.08957705	5.08909871
FSD1L	2.48051059	3.5269268	2.83895521	4.05517229	3.35573858	3.55409191	4.01029352	3.69316254	3.15734972	4.3921447	3.18280992	2.83416184	3.49186814
CNTNAP3	3.14340748	4.63760635	2.4759547	1.84304815	3.31257811	2.44795646	3.42751957	4.66218385	4.77424056	4.56398882	4.72724393	5.00994234	4.75435798
SPIN1	3.57331249	4.39032289	2.89158603	4.19296835	4.66853351	4.37282029	5.00659531	4.56780602	4.71215744	4.21379003	3.32808731	3.94825606	5.15551769
PRUNE2	8.61296036	8.33619039	8.56719252	7.22041991	9.37326286	9.63584192	9.09263377	9.61362905	8.66799367	9.11636633	8.44121263	9.17048289	8.93709484
MEGF9	6.09297957	6.52425212	5.61125225	5.60093717	5.79282248	6.03679404	6.3225477	5.74805829	6.09105262	5.57831261	6.0923545	5.64976137	6.25060854
TRIM14	1.81592105	1.56601597	1.00467303	1.37030514	0.52345407	-0.58858226	-0.18001799	-0.53042323	0.26416967	0.26416967	0.26416967	0.26416967	0.26416967
CORO2A	2.66160625	1.89520238	2.35084016	1.69737744	2.85290464	2.80556418	2.20255703	2.0058337	2.20761236	-1.11911509	1.25422225	0.64320978	2.89698957
TGFBR1	5.12273454	5.97171122	5.18479429	6.20949685	6.89269533	5.89640111	5.89640111	7.41192389	5.75451228	5.96715791	6.21304365	6.63919192	5.83905325
SEC61B	4.50362765	5.11684794	4.28373377	5.64336753	4.65514815	5.21431545	4.40596767	4.11520837	4.69550414	5.0155359	5.1722874	4.18493806	3.88882212
C5	4.1628203	4.92987556	4.22150098	4.36567014	4.97281521	5.50820564	5.62002822	5.21929855	4.70170996	3.39094173	4.38969646	3.96656697	4.51296143
OGN	6.70520175	7.91359687	6.78940858	8.34473316	8.95894448	9.75493042	8.55105003	8.72406362	9.81281402	8.90262453	9.19131463	9.27845374	8.25410172
ASPN	5.18658645	7.83925217	5.45476925	7.51575538	8.68707428	7.07524087	7.7773314	8.7691031	8.78600202	7.7267999	8.22831491	9.05019922	7.37689797
ECM2	6.21606231	7.29631407	5.68668608	6.64700229	7.71438648	7.56202491	7.29537791	7.21410738	7.80949992	7.12225258	7.79879216	7.54266406	6.73307442
TLE4	4.87787043	4.82251407	4.22488517	5.33814338	4.95897783	4.44031467	4.19002325	4.88338177	4.7725058	4.49961695	4.86123842	4.29642257	5.23309119
LHX6	1.04894653	2.49974387	1.47517902	3.00201029	4.07097728	1.42883097	3.96659554	2.94099171	4.12514267	4.69629857	4.77898285	4.0984727	5.32115513
PTGR1	5.72683065	4.71152637	5.1693593	5.20832452	4.23870274	5.27693424	5.53626216	4.86449848	4.99656582	5.10202762	5.17878087	3.69942553	4.62590529
SUSD1	5.53460913	4.7916785	4.71743033	5.85730264	4.65465566	4.27128136	4.58375799	4.55837315	4.44909043	4.7627919	5.78128666	4.66761728	5.27874587
AKNA	6.53553172	5.70928361	6.22470274	5.54547364	5.26987937	5.09582672	5.43124697	5.16393821	5.15290286	5.70319841	5.70319841	5.18057654	5.41749828
TNFSF8	4.77385869	4.26504671	4.81991421	5.72615965	3.28251016	3.0068249	3.44797225	2.63333865	3.24932164	1.85945263	3.5508461	3.00539158	3.59809446
DNM1	0.64652169	3.36389017	0.13668289	3.94173557	2.29847494	0.4563943	1.53811116	1.52477865	1.00745398	3.70619562	4.30643826	4.24570967	1.51253404
ENG	7.89859055	6.02877666	7.43664869	6.36421837	6.08665773	4.87138181	7.09626289	4.54639778	5.75746066	5.9556215	5.99125344	5.53526489	5.28612617
AK1	6.01881421	4.80254478	5.88398149	4.74647787	5.28755139	4.81671247	5.98391888	4.93875891	5.01679705	5.71050248	5.13575625	4.86731766	5.85106052

CDC37L1	4.57254232	4.81073564	4.79849278	5.72233629	5.73735721	6.47069482	5.96572892	5.64659983	5.55527544	3.60093319	5.25440592	4.81937149	4.40828888
TBC1D13	5.04399108	5.11521174	5.45993761	4.4482271	4.66132024	4.42719862	5.05730244	4.57710704	5.41054096	4.97381446	5.15447654	4.89286836	4.9609185
KDM4C	0.88443559	0.59397622	0.26494926	1.27820375	0.89699906	0.99642795	1.07450297	0.93982479	0.74908109	0.74908109	0.74908109	0.74908109	2.05868999
DOCK8	5.61670157	6.17696238	5.48686443	6.29028742	4.66772538	4.21167592	4.54148855	4.2995506	4.9457572	5.1852209	5.36454546	4.86491669	6.08887911
KANK1	1.91455681	2.39242544	1.26856463	-0.6389196	2.74336451	3.08420847	2.68088607	2.5248462	0.96587257	0.96587257	0.96587257	4.57299646	3.84331745
NCS1	5.15516737	5.6441828	5.6613084	4.71230616	6.45254234	6.29647288	6.67128504	5.78017083	5.67671436	7.46974527	6.29471706	6.67991064	5.83075819
TESK1	4.91566222	4.08671277	5.1122307	3.72517006	4.83822563	4.47181599	4.70696364	4.48434605	4.61109686	5.03320107	4.73720134	4.53177821	4.37774656
KCNT1	-0.09756979	-0.10522906	-0.00445157	-0.10522906	0.52742667	1.10888275	1.07801412	0.51235946	1.62580998	0.01997302	0.01997302	0.01997302	3.60474292
CA9	5.25585813	1.44961724	1.85291416	2.87969881	2.93503753	-0.68840518	-0.51764128	-1.11093706	-1.45362982	-1.45362982	-1.45362982	-1.45362982	-1.45362982
FUBP3	5.72871841	5.35934843	5.38899061	5.21088812	5.79572134	5.96333728	5.60967857	5.75552084	5.67129375	6.16687056	5.5630832	5.67575305	5.84088948
TYRP1	1.87424212	1.35960775	1.45522696	1.31421746	2.11196259	2.96867676	2.68474513	3.12989836	-0.44573824	1.48579188	2.24664272	2.4630015	4.75928075
CREB3	5.20203117	5.03035401	5.48918995	4.75981826	4.92023046	5.32541763	4.50732427	4.17883975	4.11814966	5.70892535	5.07444455	4.95576196	4.86054693
RGP1	6.07968888	5.42733861	5.6627167	5.20767757	5.69305929	5.43798542	5.58696195	5.66325245	5.1929396	5.84312098	5.66132232	5.49716728	5.75252212
MPDZ	7.17406703	7.29405056	6.69903783	6.65460507	7.31811081	7.57755812	7.32488847	7.51034604	7.23611993	7.17005765	7.23430694	7.28147084	7.47788048
DDX58	4.75897244	5.49301961	5.09675228	6.52832395	5.92639263	5.86540547	5.27946094	5.30663235	6.09333721	5.23695746	5.8423706	5.31695098	6.06197316
EDF1	5.8228121	5.12825014	6.07620816	5.07305609	4.95096339	5.98049132	4.7621353	4.87288343	6.32188197	6.26826894	5.35856183	5.03010308	5.27100174
PIP5K1B	0.98430668	0.93813773	0.77004615	-0.351417	2.5051201	3.7588959	3.25022955	2.68419573	3.11959313	3.83661507	2.94454013	2.26313477	3.46421362
GLIS3	6.51648704	4.45870075	5.56147011	6.05805669	5.0101025	3.94659289	5.12307418	5.67213777	4.38225329	4.2438094	4.01050311	4.66718581	4.2692687
BAG1	5.54330306	5.55431677	5.52799515	4.53429122	4.85333455	5.16707944	4.84469808	5.2672294	4.84823053	5.81063175	4.60795811	5.30553198	5.30593576
RAPGEF1	6.0775511	6.45766692	6.46150189	5.72840099	6.49759077	5.60817417	6.1237761	6.07451834	5.991706	6.42557119	6.26676944	6.49910646	6.47114241
NPDC1	3.73465041	4.25504226	5.01802826	3.40726554	4.03911281	2.88870561	4.25628436	3.82538217	4.59107735	4.75077722	4.22949883	4.83974362	3.39038878
APBA1	4.87199956	4.83422244	4.68556553	3.7175555	4.21674031	4.59034878	4.87056344	4.45905107	4.32629945	4.49420234	4.75032231	4.97386502	4.87046639
SETX	6.42918902	7.10613624	6.48496535	7.45138113	7.10785706	7.28970723	7.14981559	7.14292245	7.14733907	6.5085238	6.9031406	7.28920776	6.92371827
PTGDS	2.70581857	2.19649749	2.53992375	2.42490901	2.6531903	1.87580458	2.91669328	3.23240497	4.3322213	6.00945522	4.04424468	4.08500419	6.23698075
ABCA2	5.68729844	5.46392815	6.02631875	4.3065384	5.36657942	4.90712874	5.61794634	5.59590401	5.15414052	6.27050418	5.97030022	5.94640318	5.1425363
SHB	5.87593193	4.62904325	5.69293414	4.60464313	4.43460197	4.43460197	4.1722818	4.33110795	3.86350068	5.31539038	4.10375585	3.94279089	4.47168024
UBE2R2	6.33181839	5.978845	5.64812111	6.03370986	6.27362853	6.14066626	5.62258971	5.80390941	5.71638202	5.45917083	5.54374797	5.09427571	5.84738778
EXOSC3	3.80481139	4.77187192	3.96705978	4.93563711	5.32646892	5.41330226	5.19160722	5.32608498	5.46241078	4.9157987	5.48646432	5.04735737	4.5754167
ZFAND5	6.4958474	7.19727146	6.50201226	7.39038642	7.29774667	7.40277251	7.29896724	7.30080822	6.8955712	6.55475212	7.06275145	6.83929475	6.93069018
DVL1	4.75424153	4.31470329	5.1062221	2.75916442	3.64915381	3.88884426	4.23791682	4.04168141	3.9731531	4.64358927	3.90259527	4.06216789	0.82257892
PDLIM1	7.4716758	6.35211934	7.42030502	7.29929114	7.94917312	7.490127	7.23671367	7.67929453	6.86765905	7.86108509	7.37426321	7.83654388	7.7205278
CCNJ	4.25431546	3.86399786	3.87647726	5.0073906	4.87347009	4.57740973	4.52940138	4.66305129	3.64230574	3.86016313	4.30712981	4.66793225	4.66748214
GATA3	0.68791179	0.71604615	0.18869504	0.21771092	0.14239485	0.30046024	0.10507228	0.00158797	0	0	0	0	0
ATRNL1	7.08160281	7.42499905	7.02481622	6.13421746	8.18076856	7.78219048	7.40402796	5.82765942	7.30428651	6.81275601	7.95799918	7.24285987	6.08873601
HPS1	5.42359482	4.96582946	5.42409082	4.60968652	4.94155803	4.40248137	5.09884742	5.13686538	4.98461763	5.72529162	5.02153426	5.10003424	4.87497979
PHYH	5.01871584	4.43491612	4.68827256	4.50794952	4.76247307	5.81576671	4.62940029	4.99457639	5.0371873	4.97437087	5.06837986	5.03261944	5.04696899
RASSF4	4.91180564	4.57287121	3.94996101	4.81903229	3.16701529	3.08474418	3.37804439	3.21803087	4.00787941	4.41652313	3.69034507	3.05801703	4.83874721
DNMBP	5.15934206	5.02576455	5.56081876	5.31555723	5.15387216	4.19305739	4.74933452	4.797337	3.98669275	4.42957712	4.49648639	4.57120466	5.78353368
RAB11FIP2	5.56049686	6.07664944	5.3682884	5.78666459	6.25976076	6.47530893	6.17090134	6.14243406	5.95495607	5.82723676	6.26335299	6.11162843	6.08631451
CXCL12	8.45673777	7.18029294	7.10936977	8.21098414	6.55187847	5.15783258	7.17027128	5.836654	6.36412443	5.91766759	7.37837392	6.14245078	6.7567629
ERLIN1	4.75362469	4.96096486	5.03150204	5.62033784	4.72271672	4.56538828	4.78986088	4.55032183	4.89835843	5.22456274	4.79316482	4.74946862	5.20017836
EIF3A	6.60332486	6.53802649	7.08096319	7.26986926	6.87777453	7.56460939	6.95180273	6.88036098	7.28262587	7.31276785	6.40212411	6.56006328	7.26462528
PKD2L1	1.29769193	-0.06622547	0.24546391	0.05753014	0.03107566	-0.06490073	-0.06622547	-0.06318573	-0.05838657	-0.05838657	-0.05838657	-0.05838657	-0.05838657
CUBN	4.47911856	4.09711828	3.61105257	3.94573437	4.34252691	3.60810134	4.64461324	4.83501552	4.40131344	4.29986734	4.57602789	4.53674436	4.80121019
TRDMT1	4.32742775	4.7365385	4.53339173	5.02349505	5.49603723	5.51672134	5.45305368	5.65687275	4.99143272	4.39381689	5.57278728	5.43191764	5.07831698
DDX50	5.68842816	5.79240023	5.41155161	6.48910548	5.95377825	6.47620256	5.86505737	5.95896353	6.25778103	6.43582395	5.87539562	5.7996352	5.6475728
MAPK8	4.57120402	5.30473192	4.64052174	5.85046999	5.27802972	5.42487921	5.54804661	5.73850444	4.7450015	5.09103583	5.10431666	5.63336399	5.34453038

SEC23IP	5.60751764	5.87866991	5.44254774	6.72129144	5.9881914	6.21208758	5.65341668	5.73430649	5.36621012	5.43709637	5.35990879	5.99098932	5.62543698
ATE1	5.10935638	5.64414576	5.07598592	5.72435133	5.63500492	5.93993759	5.67377834	5.89399549	5.89521063	5.57200268	5.95684426	5.96493851	5.82295642
NSMCE4A	4.54836498	4.83945898	5.00680131	4.7398004	5.07990939	4.96009497	5.44069605	5.76051744	5.06128136	5.28797605	4.81634364	5.11788861	5.16577297
PLEKHA1	4.484202	4.95400006	4.27405381	4.87416733	5.18147764	5.22129295	5.42097518	5.42627383	5.01383385	4.66477077	5.48699794	5.45923372	4.70982519
UNC5B	3.40361251	2.74645418	3.54000008	3.90375929	2.39359981	0.54492063	1.10370092	0.35565301	2.98752338	3.98721756	4.53549152	3.08778308	0.75098024
CDH23	1.60475379	1.58359998	1.70859292	-0.13992071	1.07606477	0.05958732	-0.07967931	0.79632629	1.19526004	0.00026784	3.10470815	3.92562967	0.00026784
SPOCK2	3.83268916	5.91368842	1.91014976	3.46865774	2.91121614	2.32685003	2.57627284	1.75082544	2.56008578	-1.23151148	4.49996253	3.09669894	3.79530873
MICU1	5.56044691	5.09496016	5.35498041	5.51255999	5.48474634	6.1326771	5.33905239	5.280391	5.32370042	5.37595086	5.65752322	5.42554901	5.97801257
PPP3CB	5.73870166	6.28742438	5.70122052	6.43706291	6.65016645	6.43418685	6.66736653	6.7423171	6.35613247	6.22967079	6.53189627	6.51223286	5.92525936
BMPR1A	4.80194987	4.79918094	4.09518293	4.41738961	5.46425055	5.47437135	5.39337987	5.43212053	4.81906373	5.47442811	3.75956179	5.63203829	4.69079611
MINPP1	2.92478257	4.15418593	3.75618678	4.45608141	3.83098369	4.30843727	4.52272723	3.83345414	4.17608957	2.44756135	4.69180207	4.6406615	2.79690552
ACTA2	8.53863713	10.3570205	9.34530836	8.03478029	10.8477323	10.8477323	10.1085859	10.8477323	10.2379365	10.9569685	10.4534144	11.0595758	10.4158574
LIPA	6.23939384	5.89537263	5.70143248	7.39890325	5.4232	5.38061759	5.8011069	5.24145903	5.48768525	5.61659902	5.8466812	5.70022394	5.94986851
LZTS2	6.16511631	5.46596745	6.31438494	4.4375815	5.10942267	5.09015917	5.36969182	5.42293871	4.70645462	6.3294565	5.75815274	5.92097872	4.41495923
SFXN3	6.41281715	6.44298714	6.69728637	5.64601426	6.41082374	5.90775009	6.18440836	6.80722811	6.402805	6.64157031	6.78388897	6.77854807	6.31524076
KAZALD1	3.96764459	4.59924378	4.69222675	3.14712557	3.83400581	3.58936785	3.86255835	3.94254314	4.8149059	5.50716548	4.72681669	4.9463616	4.35777805
FBXW4	6.50315786	6.24088192	6.30484935	5.07892981	6.29199334	5.70200382	5.76938425	6.00581561	5.70282164	6.50733991	5.48362332	5.81835057	6.41847214
NPM3	2.23519149	2.51087647	4.15887717	3.81411811	1.78999041	1.6748787	0.79818383	2.23147829	3.0826787	4.34715626	2.81750236	3.92254862	0.2850815
TNKS2	1.63484771	2.47691316	1.14432494	3.23864487	3.15256201	2.77845378	3.30611233	3.17646428	0.58149779	3.31943644	4.59859681	1.2645615	2.77756694
PITX3	0.27587193	0	0.00124472	0.07133326	0	0.00069205	0	0.34990497	0	0	0	0	0
GBF1	6.38359997	6.06362427	6.38534661	6.47749853	6.03700778	5.95461293	6.00889878	6.07100382	6.14781488	6.19340846	6.36782667	6.4198359	6.58345245
ARHGAP21	6.4950955	7.29003604	6.21172855	6.96662174	7.12760825	7.11825923	6.99832909	7.4473507	7.38867689	7.2546545	7.12875246	7.15157574	7.3633433
CPEB3	4.63790061	4.72536303	4.24200343	3.97039553	4.69990282	4.5217299	4.8077122	5.12612061	4.94426517	4.44612817	3.59359038	4.93922894	5.12977871
FBXL15	3.55846522	3.58047457	4.78824372	2.1424787	2.43176631	3.64777086	2.47445983	2.37127423	2.31458274	4.67790817	3.06487101	4.18367889	-1.0929516
CUEDC2	5.40171169	5.03517483	5.59802144	4.46104399	4.82204871	5.06695207	4.54116365	4.5974845	4.94067461	5.94289946	4.97342928	5.29439644	4.78994196
SUFU	4.01691602	3.15742058	3.66113825	3.09991613	2.38180782	2.67847613	3.31961932	2.54446827	1.44964118	2.53822353	1.18429043	2.9288504	3.35206722
ANKRD26	4.25731717	4.53032023	4.53697942	5.43863483	5.15581964	5.35364684	5.17287321	5.0604923	5.9404143	4.43467386	4.91777612	5.03612884	5.18907984
ACBD5	4.75324265	5.20806378	5.04834725	5.00935634	5.03160982	5.40415184	5.75120205	5.36570181	5.62667505	4.98881484	5.28725054	5.26351307	5.13630766
LHPP	4.21928804	3.16416684	4.25841902	3.09863042	3.2488287	3.51403619	3.11483736	2.71824952	3.10246185	2.26052946	3.48973583	3.94693538	3.8918634
LARP4B	6.28981576	6.03847223	5.92619632	6.27280322	6.07453161	5.708167	6.06271662	6.09440977	5.73776818	5.46419984	5.65902571	5.82234608	6.40060829
GTPBP4	5.44886307	5.16591236	5.70628186	5.99469603	5.35659352	5.59125183	5.06628324	4.95265714	5.21478098	4.97837909	4.7256416	4.43605768	5.27557331
BCCIP	5.02098682	5.25494497	5.21412033	5.72762533	5.1481875	6.19510283	5.56949268	5.5629144	5.49631405	5.42891228	5.23717712	5.16757471	5.50988631
MTPAP	4.84234167	4.41054833	4.87447225	4.91224723	5.20924273	4.80676131	5.18344535	5.27453626	4.82150171	4.52109394	5.48892255	5.26204213	4.86587654
SH3PXD2A	7.11351431	6.75523569	7.39894316	7.39592308	7.00812154	7.07008526	7.53618765	7.23998127	6.85822154	7.31712167	7.31032108	6.90209549	7.04551981
PITRM1	6.52888968	5.03018274	5.77001777	5.92676975	5.34336354	5.48735271	5.68440387	5.73504529	5.22115905	5.06884251	5.29706902	5.40189658	5.74765214
MAP3K8	5.24067327	6.05395207	4.57859648	6.10210135	5.68722111	5.3261486	6.00632422	6.20164705	5.57512471	5.26806391	5.34506258	5.40839217	5.42062898
DKK1	0.46030301	1.08654087	0.81047496	1.7381362	1.15919745	0.84476499	1.91942715	2.31383368	2.46846489	2.60938877	3.06450375	1.36438133	3.26670194
EBF3	1.910035	3.54301447	0.89314886	2.70793763	3.29483682	1.69999505	3.06016215	3.17233243	3.24309932	3.58152596	5.11006667	2.37544146	5.38320715
GLRX3	5.08536953	4.87647358	5.55829486	5.86781694	5.58590687	4.92913387	5.82657878	5.62266389	4.99087786	5.02307913	5.36021051	4.84570966	5.0150374
SORCS1	1.36625939	1.33483423	1.33742672	1.35600476	1.78426252	1.91162325	2.01415228	1.84149843	1.47229362	2.2805588	3.75048232	3.48956474	5.26343363
FAM208B	5.57610314	6.60729877	5.8119217	7.35522619	6.64715796	6.66148598	6.53360211	6.54146886	6.20703438	5.75150152	6.17179819	6.16089055	6.64381512
XPNPEP1	5.07106	4.78269713	5.40051296	5.53262735	4.40396374	4.60944195	4.58394405	5.31708094	5.27444244	4.08275643	4.33068066	4.8363001	4.69337668
SMC3	5.28640348	5.97586174	5.91003734	6.30405069	6.36715194	6.88134453	6.2529405	6.35484493	6.89102332	6.29370779	5.16334712	5.81588644	5.93509801
SHOC2	1.14167185	3.05576232	1.84738451	3.56114446	3.55908138	4.02038044	3.7800606	3.86144873	4.16975711	3.84888349	2.56159834	2.80750359	2.25882792
TFAM	4.86218985	5.2960676	4.31971318	5.499599	5.68258142	5.8242027	5.68095648	5.84142937	5.46921316	4.42355894	5.66850891	5.2219037	3.71455264
CCDC6	6.7136523	7.04876279	6.723241	7.12911565	7.55586726	7.44601901	7.62775875	7.85325389	6.88515742	7.21442744	6.90092498	7.73275191	7.01790511
CUL2	4.70705047	5.16940766	4.61734245	5.5226672	5.21308035	5.76697097	5.43777868	5.51963679	5.4056594	4.99406206	4.57601532	5.33831346	4.82739777

CCNY	6.17053419	6.65360713	5.52692368	6.20340351	6.26007577	5.82141025	5.88633245	6.21489	6.72393654	6.24784514	6.49828423	6.33372498	6.48367618
UBE2S	3.24156883	2.44634201	5.01030104	3.8214719	2.68573325	2.91496807	2.75182713	2.76528217	2.87918134	2.94682843	3.10704891	3.58108093	0.44154882
RPL28	7.73411142	6.85301949	7.16420543	7.30940945	6.66776793	6.95704963	6.00627619	6.42833338	6.74832945	7.20134912	6.72471308	6.77879362	6.29240671
ZMIZ1	7.34990667	6.29739529	6.94324145	6.38074479	6.15606176	5.47565286	6.19570877	5.8329625	5.8331337	5.97101563	6.05485945	5.63489488	6.04650049
DNAJC12	2.9778898	3.5617267	2.60645382	2.96868109	2.03160003	4.14352455	3.34997691	3.59386142	3.24678253	1.63508469	3.57987236	3.1610243	3.38529596
PPIF	4.86013609	4.13391076	5.11730717	5.94174293	3.57354553	3.17556419	3.52675579	3.25223777	3.39686972	4.14712919	3.38146717	4.15995565	5.47576705
PBLD	4.47566142	3.98234651	5.06186428	4.41979538	4.05387656	4.61917233	3.96672443	4.52551471	4.892467	3.99316479	4.2506563	4.43855616	4.60177508
TSPAN14	5.53052956	4.97172379	5.12027759	4.85764321	5.11297061	4.11858352	4.80403844	4.65196682	4.61900395	4.42222844	5.39174273	4.25142406	5.56889649
LGI1	1.46310049	1.59672073	1.46769296	0.81227025	2.19645146	4.22189038	2.35589528	3.41366598	4.48607149	4.29335418	3.04261893	4.4931775	3.24997365
TBC1D12	4.54478085	4.92750809	4.37358431	5.40542012	5.18691612	4.98006907	5.4083949	5.51647905	5.18496486	5.16854415	5.61912587	5.61600042	4.29436235
KRT23	0.70370904	0.05058122	0.05314111	0.20997565	0.50493226	0.22940506	0.05058122	0.05384704	-0.10569779	-0.10569779	-0.10569779	-0.10569779	-0.10569779
NUFIP2	6.13595624	6.81082663	6.19284168	7.33422766	7.14098109	7.41182739	7.15236302	7.07236479	6.71041887	6.01767079	6.98042533	6.66059875	6.41876087
GIT1	5.02269791	4.85234007	5.27127488	4.52129873	4.60903118	4.17504547	3.93485418	4.82307402	4.41825064	5.05801681	5.30592597	4.69657132	4.18563471
RPL19	7.67467024	7.83238501	7.72100507	7.3337393	6.8404874	7.70759983	6.65737837	7.04274549	7.94731783	8.10535991	7.29453124	7.55948096	7.10513449
FBXL20	6.090329	5.75589153	5.90209936	5.63294613	6.47038963	6.30270389	6.42694895	6.69560271	5.86899295	6.41493321	6.43410014	6.49428396	6.31577406
RUNDC3A	0.40639132	0.09872562	0.21067299	0.20305987	0.23601324	0.10017139	0.09872562	0.20305987	-0.21830001	-0.21830001	-0.21830001	-0.21830001	-0.21830001
UBTF	5.46276128	4.99963865	6.22944225	4.95722638	4.51076828	5.45573199	4.88304667	4.47333315	5.4711724	5.6979709	5.34779877	5.10705797	5.42722552
PSMD3	5.91998345	5.18689083	5.71723352	5.56318669	5.5307135	4.97872442	5.24500862	4.99844175	5.42680552	5.41731768	5.53367784	5.58959005	5.16712417
CASC3	6.32512627	5.68694217	5.62489094	5.08170538	6.1819238	5.73847441	6.12944326	6.07590452	5.44292065	6.03647557	4.98087587	5.25073948	5.87802165
RAPGEFL1	3.51876052	2.65122412	3.09126937	2.97282407	3.8838907	1.69512355	2.44447399	4.1647008	3.07041489	3.89942861	3.89702693	0.85500568	2.82255199
RGS9	2.42844796	2.663751	2.97808194	0.89780467	2.81066264	3.44781356	3.42704884	2.81412475	3.29043975	3.42258933	3.1721081	4.3485044	3.02662274
RNF43	1.05339951	1.32166003	0.97077488	1.14531246	1.02794099	1.38678007	1.04777634	1.37614341	2.00932118	2.78117821	3.69749999	2.02099995	3.81240259
WNT3	2.22718295	2.50713389	1.6292388	2.2104134	3.19816254	2.30556678	2.59980934	3.55473132	3.05382394	3.96577012	1.75321579	1.69128679	2.16318442
ASPA	0.93404205	1.63803321	0.91193397	1.75505159	2.27483032	1.76526034	2.14433275	1.58674836	3.25429105	-0.23483978	3.66246663	1.4075783	2.60288887
RAD51C	4.06430451	4.14226033	4.03759474	4.4156455	4.58027025	4.66996466	4.53701803	3.88135902	4.50159559	3.39424531	3.26122098	3.83244792	4.94400573
MTMR4	6.08151525	5.92853024	5.75717902	5.18494549	5.99600496	6.03377167	6.36218614	6.22251338	5.8099451	6.19183661	6.17146539	5.68943689	5.91634664
TRIM37	5.48441064	6.07009989	5.41250073	6.03236309	6.11212358	6.04804532	6.04849926	6.07818567	5.90878221	5.99182746	6.17062723	5.75959109	5.77082185
P2RX1	3.22382015	1.95739109	2.37234037	1.44628109	1.5628957	1.34371017	0.99622976	1.58507424	3.04481422	3.67894084	3.1124257	-0.64936208	3.11466028
DHX40	5.52947227	5.78048612	5.65474907	6.24818343	6.14805634	6.44932681	6.38520222	6.48935588	6.02537823	6.09849507	5.87423614	6.02645657	5.54485772
TUBD1	4.1714095	4.11302666	3.55933747	3.55807156	4.29313943	4.30127152	4.80211683	4.37819088	3.87824926	3.82674323	4.17291188	4.4591141	3.69318889
KPNB1	7.07186786	6.91559194	7.27073107	7.63336805	7.129708	7.03991515	7.44906025	6.86055095	7.19455392	7.39211556	6.99421866	6.86845232	7.39905173
GOSR2	6.03097609	6.53782758	5.84878566	6.42109456	6.44981014	5.7585247	6.18830161	6.25874097	5.992307	6.42151388	6.59386031	6.12070379	6.58611918
PNPO	5.28230528	4.55618195	5.41375921	4.79575529	4.35226984	4.91599425	4.5426146	4.25585805	4.33268836	4.59691909	4.79319143	5.03088194	4.76000724
RPS6KB1	3.48903433	3.83201264	3.19312876	4.52354827	4.4400458	4.29962328	4.38678247	4.34057259	3.99331078	4.12000997	2.75245838	3.59146639	4.51171575
TRIM16L	3.62869544	3.63841214	3.97028632	3.13559048	3.8743028	4.70476659	4.30676052	2.64242887	3.58612249	2.54326717	3.54010215	3.07403888	3.21597084
CDK5RAP3	5.28319727	4.75996212	5.2804033	4.91603037	5.16610105	4.61117591	5.46976047	5.78776015	5.32457828	5.54245008	5.17657414	4.98120174	5.56040261
CBX1	6.06726627	6.04187538	6.6380755	6.60489674	6.81352263	6.53373711	6.45307914	6.76529493	6.59459155	7.10817443	5.97905049	6.55572151	6.83210578
RECQL5	3.90574995	3.31168291	4.73366694	3.25553271	3.53531556	3.00692751	3.6968601	3.17179295	3.11893315	4.76142498	3.73857061	3.43462386	3.50604797
PIGL	3.10317896	3.3845649	2.49419374	2.13196323	3.77730895	2.12240898	2.27098886	4.18735194	2.21359285	3.7718834	3.0612569	2.31576685	2.26030183
GALK1	3.52048547	3.54782157	3.91906543	2.70042618	2.851073	2.68535764	2.83807972	2.82857218	2.58963804	2.37265016	3.43720656	3.82020039	2.625541
INTS2	4.46961533	5.12953273	4.84557405	5.44215432	5.41792691	5.72874303	5.31447617	5.22999572	5.25834606	4.44752165	5.81852917	5.21358304	4.52610542
CAMTA2	5.10324271	4.34039243	5.24567993	4.18149243	4.69406921	3.83892671	4.60648258	4.28829723	4.05502692	5.42391343	4.77175885	4.07825352	3.59360228
MED13	6.64406427	7.20623033	6.71105659	7.45998221	7.58075107	7.41688782	7.38594025	7.72621668	7.20275248	6.89546849	7.66578049	7.56230752	7.28728283
HOXB6	0.9257225	0.26790528	1.02752239	0.94621796	1.18154775	2.16042357	0.27449548	0.80345192	3.27097179	3.35132701	-0.42722536	-0.42722536	-0.42722536
ENO3	3.49505517	2.35936098	4.49045327	3.15905317	3.13865334	3.2055979	3.60691153	3.99030725	3.04611072	4.30302924	3.30229319	3.77340027	5.9325932
PFN1	7.96555274	7.77013231	8.75909549	8.66223431	7.97556832	8.18661524	7.61354558	7.5762	8.0562991	8.60128554	7.99199094	8.02761906	7.85679372
RNF167	5.47130489	5.11061693	5.42855398	4.43244036	5.16451194	5.83797248	5.1241066	4.5141922	4.95614494	5.8488854	4.58958346	5.17822634	5.33410213

SLC25A11	5.14812168	5.32012746	5.24503992	4.69665006	4.67470775	4.42284694	4.47749957	4.08638035	4.47594752	5.05577194	4.60080456	5.59868832	5.49271328
RASD1	2.67842235	1.43515009	2.44213081	2.31370762	2.1480824	0.86363855	1.66530213	1.15085623	3.43092914	-0.60477226	3.729496	3.91553209	3.59802243
CHRNE	1.68207565	1.88952061	1.01014523	1.51278951	1.2467832	0.84534726	0.77885148	1.28176887	1.04481627	3.40776076	1.72851589	-0.46300077	5.83921629
RAI1	2.2934201	0.89474307	2.40474001	0.98773304	0.74730646	0.3222826	0.48148013	0.60206394	0.23067275	0.23067275	2.16596572	0.23067275	0.23067275
NUP88	4.99885213	4.92010169	5.25184926	4.95181342	5.45775793	5.89036667	5.20372284	5.16141351	5.26780412	5.61838139	5.17194125	5.32781587	5.06423605
C1QBP	5.60196224	5.6194221	6.15515688	6.16208504	5.28003507	5.35539445	5.19343339	4.78996418	5.70153968	5.93946734	5.27511897	5.12789216	6.29046657
SLC6A4	0.64591257	2.07055928	0.79028002	0.43050033	0.52076728	0.88987901	0.49023603	1.11448998	1.55092538	2.81174762	-0.35004585	-0.35004585	-0.35004585
BLMH	5.65697267	5.40004925	5.80766224	5.97280286	5.57323577	5.6881106	6.11996209	6.04050857	5.59254306	6.30447364	5.41580794	5.58061279	6.09525755
CPD	5.63652646	6.30182787	6.25176957	7.14601883	6.85044349	6.90494291	6.7863437	6.85145989	6.63042166	6.59042707	6.58518864	7.12330877	6.70236774
GOSR1	5.7233192	5.74568996	5.66537516	5.99764878	6.43192128	5.95795455	6.40047223	6.26404009	5.85483203	6.34284215	6.04140277	5.85932304	5.73210389
CCDC47	5.9714898	5.86576636	6.47992049	6.5716973	6.56937273	7.04399226	6.26963354	6.55348239	6.54005081	6.25295271	6.08102028	6.35350457	6.60299444
MED31	3.91483897	4.09309723	3.24411856	4.10114102	4.0294259	4.48092089	4.64137666	4.30587664	3.6410549	3.71961269	4.25403848	3.94000414	4.11052274
DRG2	4.94223426	4.45846336	4.88590285	4.29707411	4.36756152	4.08734664	4.11833887	4.40673817	4.73070773	5.55117829	4.3783683	4.176107	3.62491791
FTSJ3	5.77903832	5.50993804	6.10471329	5.2055536	5.09293976	5.14235402	5.23719491	5.01938536	5.3723768	5.81405621	4.96159851	5.21069118	5.80739933
AKAP10	5.28676903	5.81083386	4.81000847	5.87545994	5.54366817	5.67059949	5.79691658	5.92642519	5.37226885	5.01940893	5.02314375	5.17283938	5.16471968
ALDH3A1	1.69262117	1.72812333	1.04520112	0.44016253	0.23321616	0.95240619	0.34295473	0.12950558	0.16212445	2.78507113	-0.30199887	-0.30199887	3.17357775
SMARCD2	5.99332442	5.87340628	5.95428705	5.07949368	5.06887091	6.16010515	5.64081569	5.74874592	5.5882735	5.44025065	5.37775983	5.02326813	5.90866118
ICAM2	1.77243005	3.78597214	2.7187454	5.13979809	3.24456648	2.33936297	3.14813535	2.91784523	4.13586142	4.04627812	5.04240414	3.46595467	2.65388009
SYNGR2	5.75214318	5.73769484	5.48388279	4.87215652	5.62503727	4.9608962	4.95021327	5.05888236	5.00582107	5.34769904	5.48471316	5.40716659	5.12394975
B9D1	3.3469221	2.83961241	3.01803717	3.17355476	2.79520718	0.60899381	1.48018925	2.10897784	-0.67065133	3.71311898	-0.67065133	3.02636029	2.78275528
UTP6	4.95078443	5.15619806	5.73066335	5.94409793	5.42214097	5.19662033	5.80622817	5.69771232	6.01105633	6.18510402	5.48136037	5.31085285	5.46875635
DDX5	7.30520034	7.91158073	7.34791299	8.27802258	8.04249346	7.98160134	8.34688216	7.98415772	7.69488826	7.24448912	7.73940339	7.79779943	7.90950509
C17orf75	4.13684212	3.63730655	4.02823959	3.8190645	4.6509938	4.59020306	4.42925797	4.59463991	3.74305501	4.3798716	4.20648489	4.49709418	3.69062877
CYTH1	5.2998751	5.40251342	5.28519271	5.47405497	5.05221052	4.03526888	4.99375109	4.8185562	5.223085	4.91400961	5.14651593	4.01853512	5.58410332
PSMD11	6.15092833	5.66731025	6.07051111	6.30903522	5.78291216	5.71434356	6.36393291	5.5784096	5.31509206	5.68011462	5.65123543	5.84245618	5.82940056
LGALS3BP	6.91478843	7.26136638	7.35380776	7.08268378	6.55757572	6.51365238	6.29877661	5.68229929	6.56946668	6.97557924	6.96440191	6.57250164	7.65003644
CCL7	1.44760107	0.03828091	1.57971948	1.04570419	0.03828091	0.0396987	0.03828091	0.04153417	-1.3782074	-1.3782074	-1.3782074	-1.3782074	-1.3782074
CCL2	7.03455756	6.9499156	6.3792238	8.29190319	4.75536916	4.5608387	5.09162444	3.21782611	5.57515026	3.98596955	4.58229198	4.07444492	5.86436523
CCL8	2.64990584	2.47133831	1.72522896	4.34379685	0.9805757	1.93388276	0.57904323	0.15293085	3.21009748	-1.11359843	-1.11359843	2.10388221	3.23221932
PEX12	4.53900854	3.40930815	4.44308856	3.74702727	3.93370613	4.80226607	4.1722409	4.10063443	4.1619269	4.31597551	4.47974252	3.67215248	3.78411577
DHX58	4.72633567	4.26654672	5.12967557	4.04883096	3.93463917	3.88690803	3.86384226	4.05450574	4.5472358	4.62657843	4.57459263	3.91885559	4.35502991
KAT2A	5.22453531	4.90245817	5.47704285	4.50292819	5.10260145	4.13987063	4.68340396	4.38008815	5.01697195	4.64603742	4.95070511	4.56697158	4.50349089
RAB5C	6.3730236	5.15313695	6.01179131	5.70313037	5.68808485	5.4902906	5.45401008	5.39060524	5.61783283	6.21851005	5.2723292	5.55705044	5.36264639
NAGLU	4.24472294	3.87658047	5.17336971	3.7211244	4.0880452	3.78555971	4.43323484	5.00526561	4.47424939	4.71420303	4.42545541	5.16246062	3.52599477
HSD17B1	0.94372603	0.12510197	0.70101707	0.1647452	0.73646453	0.58744804	1.08535118	0.93172548	-0.1513763	-0.1513763	-0.1513763	3.43393698	-0.1513763
MLX	5.71423567	4.99840638	5.46471983	5.41709053	4.9173352	5.4249596	5.15882886	5.02831741	4.89154274	5.44750846	4.95208522	5.17967138	5.09569538
CNTNAP1	5.80147899	4.50567759	5.39857476	4.65681789	4.28507846	3.64333879	4.48362911	3.97437739	3.72354496	4.15000693	3.78928806	4.31566998	4.23185157
ABI3	3.12692076	3.6335412	3.25442817	3.77631076	2.55088039	0.37721718	1.87726465	1.59709596	3.39006356	4.07115831	3.93608242	-0.83755632	3.97138565
EZH1	6.07356365	5.82785343	5.99265879	4.6610087	6.03547632	6.3099717	5.82008598	6.21764449	5.53162399	6.36310081	5.83237607	6.14070602	6.6924934
DLX4	0.15659101	-0.03403036	-0.0315006	-0.03403036	0.02018927	-0.03262385	0.67923202	0.20703062	3.45453831	-0.08925442	-0.08925442	-0.08925442	-0.08925442
PPP1R9B	7.01728142	6.66484437	7.55973963	6.53018193	6.11139985	6.07886181	5.95354835	5.89775717	6.09980128	6.4037598	6.09849434	5.65726498	6.52720542
COL1A1	7.36225052	7.8116616	8.95997372	10.0842526	7.23040528	6.76298448	7.23710204	7.85699798	7.85766767	8.31654672	8.239962	8.22471527	8.11320602
SGCA	6.18488584	5.65402893	6.34421861	4.05971657	5.69309987	5.78467345	5.2039909	5.85074159	5.30139364	6.35207171	6.23783604	6.37680693	4.62642285
MRPL27	5.09825455	5.42063393	5.00399097	4.6918588	4.55232143	4.89433834	4.00821468	4.19425905	4.59858404	4.85183572	3.84144839	4.81014989	5.35580332
VAT1	7.03893743	7.01127405	7.12179177	6.34716876	7.10533726	5.75354733	6.98337389	6.71506974	6.91387449	7.78999117	7.25827352	7.39120753	6.70563612
LRRC59	5.94483085	6.06342243	6.4144966	7.54854633	6.03322113	5.48838438	5.92658977	5.65793774	6.21262344	6.02053702	6.39977251	6.36261067	5.86213195
RND2	3.75824984	2.11558971	2.46221021	2.05325848	3.94414999	3.56598479	3.36784981	3.38963754	3.09430134	4.5240964	3.61922461	3.8146434	3.77679416

HDAC5	6.13586976	5.82203465	6.3913737	4.28948728	6.02986124	5.961348	5.92671609	6.03905487	5.90651409	7.02972334	6.37122126	6.51894803	5.37256081
ABCC3	7.10618827	4.75796531	7.05759288	5.33557975	3.73166048	4.43028733	5.0519924	4.68801651	4.63884357	3.97469569	4.46595151	4.13246133	4.14654561
LUC7L3	6.18509452	6.69103388	6.0425431	7.08744815	6.99859784	6.93128246	7.07118036	7.08934248	6.89440637	6.5285499	6.10639977	6.43371476	6.86145405
MPP2	4.73298146	3.36968272	4.32336084	3.20722583	4.02926767	4.57958453	4.04504564	4.96539627	4.04611092	4.66900355	4.51826711	4.993362	3.86773808
SMURF2	5.79336748	6.04275689	5.46605246	6.00894437	6.12953529	6.17915337	6.08290136	6.59392015	6.0673102	5.64841967	5.93124575	6.12557348	5.79938323
DUSP3	6.718892	7.43297988	6.52188831	6.09286926	7.12304455	7.10794841	7.25946063	6.6244641	6.92605585	7.40081356	7.12179782	7.60290731	7.2514691
EFTUD2	5.84328664	5.2947507	6.15052837	6.06290776	5.47542207	4.69381702	5.51117404	5.47587223	5.12559024	4.96670895	5.62673716	5.25372849	5.50179326
HLF	5.98691906	6.01289591	5.43442909	3.29565276	5.28295984	6.30794649	6.22563068	6.14545532	6.05468481	6.75887202	6.39585118	6.97923461	6.17380982
SLC16A6	2.61996175	1.84591953	2.29376423	3.62452626	1.94703574	1.57713169	1.68083692	1.4042396	-1.13383055	-1.13383055	2.10376376	-0.1024292	2.78713265
PRKAR1A	7.10317556	7.85447443	7.37895495	7.83937447	7.86333162	8.10169394	8.10050891	8.07801144	7.93686536	7.71825417	7.81974559	7.98644113	8.31267206
EFNB3	0.03899816	2.84589383	-0.16802792	0.68087614	-0.18686541	-0.27161601	-0.2723919	-0.07708798	-0.06045594	-0.06045594	-0.06045594	-0.06045594	2.43736378
FAM20A	4.0644447	4.55642537	3.91077664	4.45316536	2.9085085	1.88950107	2.57904211	2.25349408	2.79331532	3.16283566	3.40372714	2.76910378	4.38962674
YWHAE	7.00543681	7.40679288	6.97917378	7.8204896	7.25903741	7.67973347	7.05119599	7.10648048	7.08995557	7.59293946	6.80789894	6.9926915	7.61136572
MMD	3.69437639	5.07669204	3.30715864	5.03518986	4.23118319	3.82314795	3.95892442	3.81348521	3.75354237	4.27232697	5.01758408	4.32462332	3.2978187
RANGRF	3.96877208	3.62185862	4.4657393	2.7641729	3.20403694	3.78239662	2.89338644	3.55330215	3.41754255	4.29898586	2.76834624	4.16988156	2.36591214
MAP2K6	1.09123323	3.06455376	1.09720547	2.88845586	2.50879373	3.04590814	3.75254152	2.55570208	2.91251419	3.4628257	2.1068788	2.84610128	4.30149913
DHRS7B	2.3530808	2.31476528	2.90864515	1.55655838	1.9588219	1.40096682	1.11212796	1.79536758	2.21280765	-0.1350337	1.59240417	3.26805343	-0.1350337
WSB1	6.95937325	7.41117602	6.77860646	7.53602795	7.67324354	6.74580021	7.52614956	8.06772441	7.35734622	6.71277407	7.72663426	7.23425087	7.4527282
RCVRN	0.17584786	0.21401797	0.43970014	0.45263894	0.15191058	0.15333431	0.69034199	0.15517748	-0.11909268	-0.11909268	-0.11909268	-0.11909268	2.08545112
SLC9A3R1	0.985289	1.32959376	1.08437261	0.67583558	0.14112682	0.5035483	0.50577766	0.24468127	0.58888576	0.58888576	0.58888576	0.58888576	0.58888576
MYH3	3.44580721	2.94095122	3.83262843	2.67643371	3.47577668	3.41158396	3.37827244	3.54364542	3.55383674	4.02595166	2.99310217	3.04582013	3.59052816
NAT9	4.66001083	3.84463689	5.14684266	4.3595718	4.28774465	3.54530003	4.37958752	4.61978061	4.43410041	4.63748711	3.92757279	4.42259964	5.29805025
TMEM104	4.47148568	3.33364857	4.66976428	4.41030866	3.73825122	3.79972192	3.81937943	3.78689232	4.02586584	4.05406681	4.5329541	4.67898849	3.48006059
VTN	1.02064322	3.96845255	1.36471903	1.04534948	0.90302647	0.99220645	0.59630067	1.74755697	-0.66034905	3.83182763	1.73324221	2.91957011	3.6004525
TNFAIP1	5.94772491	6.19946063	5.36320636	6.28230877	6.03567243	5.67413946	5.52864784	5.45582089	5.27714424	5.38202435	6.00374913	5.62053637	6.26672124
IFT20	3.70247151	3.98607756	3.52952993	4.66040267	4.41153876	4.13395464	4.43975627	4.68260275	4.49510318	4.13324116	3.87709642	4.11400602	4.18640694
TMEM97	3.49860835	2.7494109	4.01538407	4.15612662	3.80950409	4.08435111	4.14108058	3.54036193	3.9716127	3.17107938	1.81879474	3.5655202	4.08698259
CDR2L	4.70179909	3.87221164	5.30588614	5.07520872	4.07462168	4.00799742	3.32949242	3.2310673	4.32479985	3.93653398	4.70691206	4.50085863	3.748728
PMP22	6.12100486	6.21431038	5.98419598	5.83994582	6.72629962	6.44386383	6.38271903	6.95343768	6.48450357	6.41243943	6.54058123	6.41096188	6.20489211
UNC119	5.26676953	3.83486307	4.62562154	3.87500459	4.01339413	3.81668689	3.88356539	3.72736623	4.76091517	4.19854142	3.98713574	4.21544102	4.21852928
ALDOC	6.96185085	5.25979689	5.58580183	4.60846191	5.57732624	6.19884772	5.20347088	5.07334416	4.86142772	5.80326345	4.38234916	5.1106243	5.54373649
SUPT6H	7.42768284	6.90015286	7.33314877	6.66998322	6.81494552	7.01644738	6.61819597	6.57848949	6.81977618	7.2169152	6.77156776	6.70086904	7.19550999
RAB34	6.73354156	6.16307105	6.54101297	5.88568838	5.8577575	5.7064757	6.00903458	6.16867564	6.03471726	6.41840351	6.16071082	6.42569401	5.67366366
PHF12	6.42264181	5.2569478	6.19379363	5.29811824	5.96675005	5.62217849	5.76488837	5.99343644	5.15429825	6.25452497	5.74549046	5.22042606	5.84483721
TMEM33	5.28681749	5.28221187	6.10628214	6.50745334	6.11044767	6.00626632	5.92620331	5.88695379	5.80434387	5.31818804	6.23887931	5.97820015	4.96727249
GABRA4	2.82870117	2.71678924	2.71560819	2.64557192	3.44738036	3.00985465	2.69728261	2.82870117	3.9374879	1.16984456	2.98620809	2.34395578	3.59072553
SLAIN2	7.01266165	6.92337912	6.50174102	6.60588051	7.3879315	7.1822656	7.13861625	6.96928287	7.1691009	6.77220236	7.16282928	7.16712297	6.72267172
OCIAD1	5.7777224	6.51462805	6.26369228	7.04127408	6.91913833	6.96172076	7.01879261	6.91560674	6.88855172	6.31279989	6.53063935	6.89580222	6.10324533
DCUN1D4	4.15364698	4.87559169	4.04535995	5.07278891	5.06160071	5.46356411	5.31314292	5.17208372	5.39398422	6.01953069	4.8430929	4.51103276	5.5487609
USP46	4.83040461	5.02624856	4.31028808	4.58522484	4.95157191	5.42879978	5.39849287	5.5116143	5.19310734	5.55100745	5.70346214	5.44696949	5.65467047
CHIC2	4.5310405	5.42797758	4.43106443	5.50188071	4.40379486	4.41916588	5.16370957	5.60395607	4.80314029	3.49323608	5.22254855	4.941299	3.6615678
KIAA1211	2.28510679	1.59259581	2.51501155	0.59971739	0.19075925	1.07365255	0.934354	1.78269964	0.12282279	-0.34344876	2.1775814	2.74242544	-0.34344876
LAMTOR3	5.12748926	5.52502846	5.42568481	5.53558754	6.19135686	6.0814966	5.83114995	5.98477003	6.24123175	5.51260835	6.03548547	6.07827265	5.62390037
NFKB1	5.46069514	5.5513017	4.69395721	5.74701008	4.78796697	4.81236233	5.14522889	4.5398117	3.63673493	4.52572701	5.22728402	4.55960586	5.04176573
AREG	1.6026994	1.16032661	1.16288132	2.19598213	1.74865762	2.3971344	1.16032661	1.82335205	3.80771961	-0.99087761	2.82101491	2.38103102	3.07571009
MANBA	5.09019656	5.08672856	5.11835976	5.76620597	4.86434669	5.73375758	5.22421112	5.38390879	5.19339915	5.53510037	5.56530294	5.48357877	5.6117102
UBE2D3	6.65941391	7.14945855	6.51432689	7.68517704	7.4588939	7.45715928	7.50354507	7.2239388	7.36980624	6.73567565	7.25713455	6.81711191	7.02629975

MAPK10	4.96779237	5.34739763	4.17616512	5.42917043	4.53356724	5.86162898	5.73925606	5.41032307	5.44530779	4.99730239	4.65515873	5.38667484	5.68947286
ELF2	5.93138903	6.15023175	5.37155222	6.02628515	6.3760268	6.2214208	6.25416455	6.53599668	6.33927372	6.18247672	6.13237836	6.52420784	6.07079188
NDUFC1	4.42615016	4.22620802	4.6907642	3.45113661	4.11827266	4.95094312	3.62364054	4.07474879	4.11587804	4.43499804	3.99641119	4.11926251	5.04250474
TBC1D9	5.3842829	5.82418122	4.99477931	5.75680284	5.06024043	4.66480954	5.09440155	5.26525711	5.55580737	4.76432488	5.33343123	5.32527137	5.67775517
ZNF330	5.2249208	4.91271304	5.4135266	5.72785545	5.16911266	5.94123361	5.33284454	5.23485732	5.42963072	4.77222513	4.79253188	5.04070438	5.85724625
INPP4B	5.29347664	5.77576262	5.10498319	5.00059806	6.14743404	6.33213376	6.25699106	5.34933369	6.08499256	5.55167525	5.5722321	5.92272629	5.87095593
GAB1	4.44985131	4.8948453	4.3188033	4.45601252	5.62056823	5.43568696	5.46277109	5.76557625	5.13797555	5.25692122	5.4862396	5.54003974	5.04800855
KLHL2	4.06196183	4.15270369	4.04173059	4.60985597	4.40689939	4.66337771	4.9429116	4.05526693	3.96695435	3.43680453	4.46969742	3.8441566	4.5616499
CPE	6.97152145	7.39927149	7.05214873	6.11939128	7.52857649	7.58162946	7.688041	7.60468954	6.99260524	8.06063748	6.87557087	7.54550083	6.73147807
RPL34	7.37185869	8.0448889	6.81401297	7.59820613	7.20750505	6.79076801	7.04652834	6.91012962	7.5328935	8.11947257	7.12557181	7.62748067	7.2159587
WFS1	6.39854649	6.36762275	6.36467892	4.39016183	6.23716433	6.66607278	6.37416303	6.47822814	5.71552686	7.58669768	6.82785144	7.12663757	6.34912414
GRPEL1	5.19868999	4.91694413	5.11196275	4.72663155	4.5060008	5.09037029	4.2543163	4.62926899	4.19626425	4.17623872	4.0604153	4.51794996	4.08100132
GAR1	4.82673329	4.21861036	4.89245438	5.255056	4.27510095	4.45771498	3.93354836	3.9564782	4.88117439	3.22993989	4.10823381	4.25902409	3.92959376
FRG1	4.88430341	4.97216753	5.49935656	4.93329562	4.44410425	5.83013371	4.71423982	4.40922223	5.52646048	5.27275909	4.44522703	4.81391517	5.90419085
CLCN3	5.46295916	6.02570285	5.22263499	6.01469975	5.34716215	5.83909789	5.7774754	5.50826135	5.9147253	5.6074722	5.58812852	5.58884403	6.32219506
AADAT	2.85378644	3.03540325	2.51018012	3.36739178	3.58463021	4.21228999	3.74775087	4.17242466	1.88516497	2.10713529	4.34172068	4.21257665	3.78174923
GALNT7	3.90816408	4.05673054	3.97383571	4.75878744	4.63645468	4.81006043	4.67238923	4.83752394	4.9170595	4.66322697	4.05750073	4.19417597	4.50274791
DHX15	5.96737768	6.74548245	6.10638426	7.20693311	6.59310038	6.59049727	6.98125334	6.83942737	6.62850786	6.69330899	6.54896553	6.4973942	6.63233833
SOD3	7.19582485	6.34666749	7.06603638	4.87080907	6.48385832	6.82474153	6.37522683	6.7610052	6.82417074	8.12355196	6.414089	7.59703143	6.0042081
SEPSECS	3.93555337	4.40456312	3.87632305	5.04679677	4.76488544	4.99476358	4.52622411	5.12796088	4.46414819	4.6912282	5.04008433	4.45403266	3.77992424
CPZ	0.63915099	1.06991387	0.6417365	1.19069411	0.63915099	0.6405885	0.95352778	1.07558327	2.94688838	-0.21954467	3.3161644	-0.21954467	-0.21954467
TRIM2	5.77660385	5.22370935	4.94638159	4.7441334	5.15235272	5.14363071	5.23190885	5.59933102	5.17623826	5.40686823	5.00227444	4.80210672	4.92445594
SLC2A9	1.55051862	1.08886819	0.91758557	0.95889856	0.13489798	-0.07784675	0.80979006	0.51017797	0.26825715	0.26825715	1.86736285	0.26825715	0.26825715
FBXW7	4.69550595	4.91789346	4.36646464	5.59103998	5.32741085	5.28007473	4.92058165	4.86013846	5.23565603	5.0613907	5.30755432	4.42752865	5.21308382
NEIL3	1.31866557	1.15142942	2.86101578	3.38194191	1.22973376	1.42311952	1.71291127	0.93737324	2.82960263	-0.96807398	4.08315494	2.20854867	2.57528142
TBC1D19	3.64957612	4.7621917	3.88815922	5.04465083	4.61996748	5.04164671	5.11190218	4.56876529	4.87938113	4.16277562	5.56053362	4.50277004	4.85025656
CLNK	0.01863542	0.47762942	0.02110346	0.03465079	0.03559415	0.02000762	0.27114674	0.02178406	-0.02076429	-0.02076429	-0.02076429	1.76526544	-0.02076429
SH3D19	7.05856354	6.86197933	6.688753	6.61982994	7.26480577	7.31600859	7.35527426	7.93437038	7.59840478	7.39397598	7.50989688	7.46950395	7.69630037
STIM2	1.92770791	1.66855516	1.10922139	2.58839885	1.72290411	3.00109913	1.52292833	2.04455786	3.03585025	4.33040827	3.17317042	2.67047855	1.79798264
MFSD10	3.94648493	3.21331882	4.66194111	3.21763209	3.46499151	2.39625305	3.15910165	3.32464184	2.21127027	2.68059144	2.92852924	4.9732158	2.52487011
GLRB	5.02569121	5.054156	4.36691133	3.44130044	4.82212707	5.57280251	5.19658411	4.29232474	4.85837842	4.06502058	5.51520029	4.66106352	4.56834291
BST1	3.15685161	4.55506187	4.23658889	4.50495133	2.92315246	2.73110936	2.94340737	3.54071611	4.4224087	4.34953271	3.08871226	4.51794369	4.69659438
RAPGEF2	2.23184962	2.57000914	1.92283568	2.53530636	3.2694708	3.24200721	2.90756784	3.69717652	0.65060527	3.48751324	1.64685719	3.51387502	3.51387502
HGFAC	0.15182358	-0.14985627	-0.14749722	-0.14985627	0.159115	0.42824237	0.97390272	0.38460304	-0.06003785	-0.06003785	-0.06003785	-0.06003785	-0.06003785
SNX25	5.63643237	5.56524331	5.80791959	5.88730641	6.40131675	6.15238484	6.60341313	6.67570074	6.53664713	6.02008286	6.57575944	6.15155311	6.16195728
LRP2BP	3.58555994	3.25639785	3.4789682	3.26220241	4.68649657	4.03399551	3.76862476	3.97543264	4.31488217	4.09274948	3.62674329	4.34246748	3.9332397
UFSP2	5.47156355	5.47292766	4.82281817	5.74366447	6.0710793	6.29627015	6.06792297	5.94177823	5.71592389	6.65360112	5.95800953	6.20097064	5.9103187
KLF3	6.56975481	6.84254438	5.69348902	6.74605047	6.36924566	6.7903612	6.52279538	6.88915135	6.58435572	6.08922244	6.27173968	6.55990043	6.08105613
KLHL5	6.02766623	6.22728613	5.54641036	6.82433935	6.08128774	5.95963869	6.19840066	6.14540465	6.24828378	5.60178807	5.92568849	5.87165708	6.14023209
FAM149A	5.17879714	4.31517173	4.34181423	3.34363626	4.19147908	4.31268761	3.67999227	4.47797308	4.76880071	4.85647798	4.3308006	4.99763594	3.66419909
NCAPG	1.18306132	1.13097528	3.98993424	5.32126866	1.27521373	1.22320547	2.2025886	1.42123533	3.27066687	1.63671822	-1.09795131	0.47209295	-1.09795131
UGDH	5.84149573	6.5748113	6.35480932	7.76571621	6.57608225	5.84705722	6.42544777	5.68358661	6.58482801	5.82344316	6.22463343	5.51449956	5.95914982
PPARGC1A	2.58552677	3.93758501	1.95190001	2.29216444	2.50695359	3.61518181	2.58147618	2.60620641	3.17080276	-1.33321244	2.60573398	2.30052046	6.79469506
DDX25	0.01417616	0.0657153	0.06914301	0	0	0.00069205	0	0.00158797	0	0	0	0	0
CRYAB	6.94010491	6.41022201	6.64533325	5.71848547	7.72919212	8.472728	6.70224182	7.33659415	7.73046793	7.73352283	7.13725197	7.11410765	8.32896291
HTATIP2	5.51648085	5.28679134	4.65237083	4.5908333	5.19399069	5.38590478	4.78001818	3.94708421	5.59564205	4.57633537	4.02171803	4.09454896	5.19316148
CTSC	6.50316026	6.61190319	5.73030184	7.10054878	5.33936726	4.3709855	5.40674636	4.36398163	5.36769102	4.5855089	5.74633891	4.81950994	5.18738207

CCDC34	2.36363997	3.20216665	2.83884584	4.59221206	2.19147103	2.38142492	2.47452283	1.77243711	3.42582929	2.92655988	3.21136366	3.50983314	0.48797883
ZBTB16	6.34672392	6.48693234	6.62722756	4.08094824	6.97258514	6.76848898	6.85971151	7.07624805	5.55881149	6.68281435	6.09699094	6.07564492	5.9731668
ELP4	3.27847873	3.54611719	3.16379922	4.20612306	3.93554144	4.98585008	4.25266588	4.34567411	4.37849334	3.59283098	4.04025734	4.58668389	1.74624693
MTCH2	4.8644555	5.63239021	5.1057366	5.83038908	5.44180938	5.63839642	5.21917116	5.4062834	5.82096145	5.18527361	5.39310814	5.66925638	5.40778465
FNBP4	6.39311432	6.48525799	6.05076047	7.13275009	7.07287051	6.27887687	7.1885277	7.29811885	6.4034183	6.40992566	6.31231665	6.03980819	6.45060393
TECTA	3.04678059	2.42281073	2.86009063	2.38576919	2.09431507	2.60216894	2.25275551	2.99672175	3.16100008	3.72666836	3.2620741	1.31883509	3.96044245
CRTAM	1.19662625	2.08115851	1.27261919	1.38317849	0.93239596	0.57934796	0.5608011	0.6449107	3.68991309	3.10404294	2.03210147	-0.75425998	-0.75425998
B3GAT1	0.29296521	0.13216372	0.07944315	0.4652998	0.31420999	0.54296575	0.14479722	0.29534777	0.0181769	0.0181769	3.58371597	0.0181769	0.0181769
HSPA8	7.48851243	7.99364546	8.37143448	9.31941125	8.24503497	8.44813514	8.70606637	7.43132267	7.72925793	7.7499921	7.68331252	7.886811	7.53885734
VWA5A	7.00295753	6.30877546	6.04880782	5.5809702	5.66578066	6.07556737	5.99433035	5.92889935	6.02606367	5.73337623	6.98905612	6.45093281	6.21566757
DNAJC4	4.75462413	3.35107732	4.33196649	3.54037625	3.16609952	3.34264878	3.03492637	3.64493172	2.58252629	4.62478416	3.64812969	3.82396088	2.61169956
SIAE	4.28713005	3.43575064	2.96435268	4.71731595	3.69737876	4.12605728	2.8656821	2.98058255	4.13500149	2.35671913	2.40649408	2.44758319	3.4915474
SNX15	4.1309206	3.95856539	4.33991173	3.20948172	4.21872104	3.76102601	4.37342227	4.58833235	3.66892813	4.36732138	3.67927551	3.76588898	3.75578086
LPXN	4.86569793	4.58500629	4.90155881	5.04045255	4.5124628	3.9612219	4.03902807	4.70253081	4.85999846	4.54274775	4.0985514	4.88990438	4.84482884
DTX4	4.12239386	4.10581624	3.12144438	3.79399765	2.41716937	2.31430638	2.5306828	3.06565709	2.46038421	2.14136817	4.08137292	1.7640341	4.13660897
ATG2A	3.69157397	2.51256041	3.90251282	2.2251067	2.44573179	1.67114421	2.62385583	2.77865441	3.04925345	2.7614545	1.80172977	3.95713097	2.68203688
EHD1	4.39405389	6.07376259	5.15945215	4.71879179	5.32804809	4.77323041	4.52223247	5.14152273	5.14246764	5.77063792	5.49579892	5.85121195	5.27163289
OSBP	6.51723444	6.32301037	6.40838891	6.33169413	6.57142607	6.39365409	6.89708113	6.37871022	6.39887347	6.36252785	6.43630506	6.4485277	6.69603
UNC93B1	5.01455943	4.65701802	4.85916332	4.49464999	3.72902789	3.14946342	3.83716601	3.57656035	4.00432091	4.92594541	3.74748958	4.55001833	3.08793849
PUS3	4.28187319	4.01616945	3.14181179	3.57413319	3.748783	4.66841509	3.8727033	3.3292901	4.28456717	4.02378544	2.98800781	3.52600173	3.84312701
DCPS	3.95063746	3.04380845	4.09294665	3.28763784	3.17181045	4.20179774	3.13528547	3.27970536	2.9126845	3.50569112	3.46423214	4.01964763	3.00923083
FOXRED1	4.46334313	3.53386616	4.87027969	4.4327944	4.27216293	3.96416952	4.34961695	4.00641125	4.04646308	4.75905798	4.79259343	4.04646308	5.04187426
PPP6R3	6.37566708	6.73921533	6.21487368	7.03641316	7.112777	7.16282989	7.18413176	7.15832429	6.86750586	6.59752539	6.98460486	6.70017744	6.7804362
NRXN2	4.98334016	3.8649721	5.02354382	3.59558135	3.9760625	4.42488293	3.47497861	4.02091524	3.16731445	4.59778128	3.70924052	3.78347938	3.49091612
MS4A6A	5.81446014	6.86475261	5.23014287	7.40920207	5.4370447	4.76308812	5.67752119	4.60816538	6.37681797	5.58005166	5.48666134	5.30916068	6.26129963
MS4A4A	4.6028435	5.12561095	3.7200436	5.50464629	3.97212703	3.11389277	4.95537253	2.38137759	4.29248052	1.52552187	3.08970712	3.99864134	4.72435364
ST3GAL4	4.91344771	5.2141618	5.01607366	4.58459572	4.25029636	4.01815072	4.52208454	4.51348252	4.40641913	4.06732748	4.58049003	4.65928136	4.95517359
CPT1A	6.7153803	7.18101038	6.99384296	6.03717958	6.27469493	6.13948273	7.05099408	7.1159903	6.79198424	6.74050488	6.95093889	6.65099893	7.23377786
CCND1	7.19654624	8.14951993	7.76191038	7.08394504	7.72902387	7.46861899	7.99275723	6.39473694	8.08211084	7.09619097	8.47352878	7.6733875	7.50384022
CCDC86	5.07925987	4.06731333	6.2228198	4.57156364	3.86197523	4.63547644	4.09367244	3.41268951	3.51547153	4.70987494	4.40655584	4.9263889	3.68422777
PRPF19	5.87737375	5.96570357	6.02577478	5.39101419	4.97116706	5.6375591	5.40887436	5.24010801	4.89672869	5.33885783	5.43393631	5.73406226	5.4890993
TMEM109	6.56715773	6.52709157	6.87548465	5.54926549	6.38233349	6.05000218	6.33140873	5.91014595	6.21537289	7.35762079	6.35838861	6.97096964	6.34717343
HPX	1.63178368	1.49717098	1.24630245	0.58923506	0.96817372	0.65760924	0.2882708	0.90892986	-0.19490831	-0.19490831	2.12161638	-0.19490831	0.84134012
TRIM3	4.79624507	3.975148	4.58841322	3.53037751	4.62434758	4.20344419	4.41161499	4.6950892	4.10158074	4.51372364	4.47141759	4.28353108	3.88686256
CHORDC1	4.97970883	5.18491949	4.91251982	6.00067301	6.12740203	6.06366215	6.37127817	5.90280711	6.08032159	4.8613962	5.76643246	5.57440778	5.04786663
FOLR3	0.48258353	0.07726938	1.26782484	0.30704689	0.07726938	0.0786583	0.07726938	0.08045639	-0.02768074	-0.02768074	-0.02768074	-0.02768074	-0.02768074
PANX1	5.98302985	5.92283392	6.34235427	6.89501275	5.7840821	6.02537786	5.86510336	5.30783979	5.75509304	5.90827009	5.81635629	5.56429242	5.76547463
ARHGEF17	7.64826439	7.46780042	7.67731114	6.21453732	7.43993452	6.93320857	7.86165285	7.23258043	6.91944448	7.89900262	7.97996096	7.75060584	7.01335306
CEP164	3.96000085	2.69110926	4.16223474	2.93038462	3.15309305	2.2054866	3.19277125	3.32540652	3.85774483	3.81792017	4.24363989	3.72308014	-0.48415078
RNF141	5.76904355	6.15959072	5.23213474	5.90500043	6.97610451	7.06725577	6.09994018	6.7825386	6.94554345	5.87396389	7.0863125	6.86908525	6.33772758
EIF4G2	7.87678647	7.98354937	7.69352649	8.70886653	8.28802159	8.47676611	8.46168151	8.14369961	8.15302533	8.03975656	7.95578208	8.08128332	7.95006451
IL10RA	5.18064824	4.80325719	4.78464145	5.3189275	4.07678697	3.11922658	3.68844183	3.38739487	4.66932957	4.14815077	4.96846564	4.26477218	4.76429956
BIRC2	5.01365957	6.105087	5.38580456	7.01522147	6.40695912	6.34591223	6.4813069	6.30306265	6.22176101	5.21950694	5.97452032	6.00407435	5.18766189
UBE4A	6.26164965	6.23764622	5.95831149	6.80194561	6.3576912	6.70849939	6.62252998	6.32114753	6.29820014	6.33564767	6.61393256	6.37305367	6.69040083
DDX6	7.13139035	7.8706843	7.2944925	7.83265905	7.96844773	7.96375961	8.03936284	7.74735377	7.80798863	7.56791677	7.89636568	7.67702447	7.94278876
CBL	6.20530215	6.11929664	6.35305824	6.12241998	6.59235546	6.44153313	6.52754636	6.10010545	5.8108488	6.38390975	6.14272577	6.36207391	6.4703656
HIPK3	8.04370959	8.25584053	7.26352593	7.87273822	8.38221502	7.98630951	8.49542562	8.27129395	7.63182101	7.82382412	7.73066696	7.93591144	7.98987035

FBXO3	5.5885499	5.61715536	4.99589941	5.6634052	5.78777245	5.99303573	6.04608162	5.88504862	5.34654115	5.73902627	5.35856806	6.05615986	5.51993101
PDHX	4.64332534	3.9517871	3.28829313	3.91757413	3.95883438	4.66908022	3.99071745	3.66310058	4.47674446	4.44756423	3.7075357	4.51141433	4.65507505
SLC1A2	1.18315596	1.25004828	1.31349327	1.16569135	1.83370421	1.58729848	1.61193155	1.74069372	-0.12018974	2.77977071	3.87012076	3.8063091	3.45797014
COMMD9	4.54850834	3.96365938	4.60726223	4.74334434	3.52173788	4.14059468	3.76674892	3.83241605	4.68997038	4.11768695	4.02998159	4.05051844	3.63365568
SLC15A3	4.53556871	4.45466869	4.53556871	4.17351355	3.54595999	2.68053009	3.25098114	3.05914414	3.57672186	4.44937861	2.88277837	4.2182968	4.02619784
CD5	2.33949689	1.7230779	0.58220442	1.23497133	1.12156197	0.67906828	0.57559112	0.7830622	2.81437932	-0.45725433	3.43676195	1.16113563	2.9475789
ACCS	4.96213911	5.0860612	4.7636703	4.00899701	4.22126262	4.03492671	4.74603048	5.80103178	4.55066043	4.9781936	5.07013302	5.32984128	4.61821223
MDK	5.06672307	3.34982486	4.33832521	4.95474113	3.00951914	2.74746951	2.42976286	2.85209609	4.14485423	2.78618454	3.32904706	3.50842294	3.7257734
AMBRA1	4.71106825	4.33723466	4.36196999	4.06629507	4.09383758	3.53061462	4.27479972	4.3709583	3.9855952	4.63165481	4.9465219	4.0514998	3.86240898
MADD	6.18396747	5.68256027	5.63594959	5.58810428	5.56575938	5.14437441	6.04849527	5.70038229	5.36634589	5.78950287	5.71432758	5.72906161	5.81864691
PTPMT1	4.8913839	3.94852869	4.31503703	4.75428899	4.46052546	4.82844123	4.36945821	4.82844123	4.39700748	4.84888509	4.21466141	4.73576404	4.41170369
NAA40	4.73695884	4.13195644	5.04230364	4.42006078	4.42228586	3.74519316	4.48825343	4.43599142	4.15898498	4.88575521	4.50109107	4.27227273	4.11793274
CARS	5.73574838	5.33622614	5.91689455	5.44754021	4.83096036	4.55176141	5.15146927	4.9683248	4.64844256	5.04246469	4.59436739	4.88541934	5.44036057
SLC22A18	2.15769048	2.02318906	3.24776267	1.52153423	0.96096558	0.22585385	0.48359103	1.03919967	0.80424295	-0.2335197	3.28707277	4.16269079	-0.2335197
CD81	6.91059472	7.38019297	7.35576052	6.50116529	6.48937962	6.19647459	7.03338885	6.5899783	6.80651453	7.49646656	7.40057896	7.69152201	6.77187178
SLC35F2	4.45710688	4.51174201	5.78616227	4.1839235	4.04704237	5.14870395	4.64056924	5.43640214	4.60769246	5.35683492	3.08754348	4.55407844	4.69697207
C11orf21	0.27247317	1.97698616	0.93297108	0.48901644	-0.19224234	0.5159003	0.01516569	-0.21333966	-0.11046592	-0.11046592	1.86882182	-0.11046592	4.53976628
ELMOD1	0.01150476	0	0.00124472	0.14492325	0.12207218	0.00069205	0.08483364	0.62030857	0	0	0	0	0
SOX6	3.29552515	4.41260807	1.65550723	2.02028312	4.05770049	2.59490569	3.91867829	3.23604712	3.91930456	1.75554241	4.38548834	4.50994209	2.97478475
C11orf58	5.90706646	5.98999796	6.23534732	6.52252922	6.33607557	6.78322281	6.45818009	6.60657072	6.51154824	6.69843318	6.57440774	6.41058509	6.2410935
PITPNM1	5.06970836	4.36878075	5.5146451	4.41645716	4.06034716	3.67802169	4.39675154	3.5899257	2.77317528	4.41753167	4.27030633	4.2712649	3.80372537
RPS13	5.52338064	6.13991396	4.86504468	6.42039289	5.89889247	5.98049523	5.59063067	5.81195961	5.49040552	6.42093227	4.8708398	5.98820468	5.51123102
AIP	5.81288999	5.8698941	6.05421484	4.61258748	5.16763635	5.53882306	5.54833793	5.66972302	5.40319649	6.28552276	5.10317893	5.89732414	5.45452872
NUP98	6.9575101	6.5451788	6.35346995	7.16052642	6.74080274	6.85036634	6.54340622	6.26947634	6.23311067	6.45597409	6.26223006	6.45469081	6.79612705
NDUFS8	3.13512528	2.88097718	4.14516727	1.8848161	2.19506905	3.11934346	2.04636381	2.18955791	3.37617189	3.23172859	2.00096539	4.34187397	3.66090455
TCIRG1	4.55134918	4.35698347	5.15220323	4.23716198	3.98903062	3.10680336	4.15129157	4.14655961	3.89827525	4.10124663	4.06717844	3.89363789	3.42973067
CHKA	3.85216334	4.24367531	3.67576284	4.54075692	3.73219756	3.08718484	3.48269318	3.40589197	3.95029827	2.45392008	4.30309975	2.38713228	3.64385006
EXPH5	4.33121982	4.8544253	4.28089119	3.45264199	4.63331828	4.82333159	4.28622519	4.02369075	4.60711082	3.62623463	3.5404106	5.20591194	4.2174652
HPS5	5.36723701	6.40469648	5.44714064	5.85687069	6.35200302	6.38909655	6.51236115	6.68613669	6.71221613	6.30219403	6.84684365	6.50599658	6.30717748
GTF2H1	5.7182045	6.21527339	5.302818	6.32358133	6.20592742	6.24285329	6.13591536	6.16984674	6.27227843	5.99085311	5.6903893	6.04348123	6.37090308
POU2AF1	2.45026812	1.99064666	0.40536427	2.20003664	0.70586351	0.12191177	0.12062346	0.12357962	2.32966117	-0.40124932	-0.40124932	-0.40124932	-0.40124932
PTPN5	0.23554246	1.51172462	0.23797349	0.53274941	2.30565041	1.70682113	1.53256845	1.23935683	3.54416057	3.33371196	3.71434224	1.42750195	-0.41955336
VWF	2.00900593	7.57822956	4.06526304	6.32412138	5.95535204	3.6928384	6.39383846	6.75937967	7.37171322	7.51228387	8.61950338	7.36584433	9.328648
PSMD9	4.87023023	4.68562028	4.7310008	4.84513484	4.84451473	3.90198765	5.15624204	5.03903072	4.71896033	4.36234546	4.81031909	4.61357836	4.81916233
PPFIBP1	4.36150685	4.359389	5.08729992	4.81368481	4.94744383	5.57169055	5.2814481	4.8850783	4.93347049	5.90343916	5.13139698	5.49264416	5.12125354
PRPF40B	5.72343499	4.95741905	5.55719518	4.07914946	5.22603199	4.77928837	5.14218342	5.18554953	5.19291575	4.87301352	4.81344908	5.00919496	4.89875672
CD69	2.2328091	3.97477257	1.65430112	3.7348025	1.85271791	3.11684642	2.50800235	3.07673103	4.16394066	3.62446837	4.16619413	2.2497826	3.96636113
PRDM4	6.0196473	5.90428889	5.50173192	5.7419276	5.86501477	5.25202933	5.80153583	5.42913827	5.49243216	5.70394929	4.42472868	5.52779974	5.85226564
CLEC2B	3.63975736	4.11195745	2.80979045	6.37622401	4.17243147	3.5517728	3.72887285	3.33707392	4.91859083	3.53832972	2.57532471	1.38096283	3.32432086
COQ5	3.82978172	4.1943903	4.24293914	4.53519163	4.18873226	3.39039542	5.11551485	4.2324797	3.61422904	4.62294234	4.33539946	4.42937238	4.79832127
SELPLG	4.26696542	3.96058225	4.45469724	4.37642794	2.82095925	2.28996546	3.10547451	1.97476249	3.33910241	3.57090037	3.15440061	3.46686642	3.93626104
CORO1C	6.81718768	7.6596227	7.09785663	7.50072466	7.85197735	7.50072466	8.14923037	7.6596227	7.63914904	7.88175454	7.70503315	8.04740849	7.84350868
CAPRIN2	5.3729758	5.37048529	5.28695049	5.93139141	5.87786719	5.73638822	5.91020971	5.8419148	5.55551149	5.73139705	5.57949005	5.75076494	5.79702597
TSPAN11	0.93376469	2.34973814	0.8527466	2.75438375	1.98727764	0.45452123	2.19554117	1.52794458	3.60092891	2.81577406	3.54290299	4.7532441	4.14677343
KCTD10	7.26930965	7.45915871	7.20352605	6.30319267	7.45915871	7.5004347	7.24290017	7.50575044	7.56169759	7.7754885	7.74517603	7.69670474	7.3646263
SLC11A2	5.8551743	5.94229752	5.54033033	6.00957787	5.85486509	5.22468401	6.3281001	5.99749522	5.80691985	5.72067722	5.99799652	5.40139401	5.66427266
MLEC	6.58857292	6.96378886	6.99546882	7.46284473	6.49339461	6.40647106	6.85901685	5.94994604	6.60823838	6.27620875	7.02841671	6.1150789	7.2308105

MVK	3.70330093	3.20291653	3.80054766	2.54206827	3.24296919	2.95071366	3.29682218	3.43069498	3.56475639	2.80027171	3.96593609	4.08532045	3.64591361
CSRNP2	5.59143487	5.06430624	5.63674855	5.83894807	5.87051486	5.67845785	5.92125862	6.09767847	5.53705274	5.68424396	5.57013892	5.5853899	5.71680276
CAMKK2	6.11038878	6.16767058	6.03282779	5.93936864	5.65144834	5.40878539	5.8606805	6.27977676	5.88271847	6.4588563	6.37191905	6.04404579	6.32581997
BIN2	4.46149354	3.84938016	4.71084412	4.67890879	2.95057319	2.81090343	3.18867438	2.18959861	4.54723462	3.97962632	3.13725117	3.19310188	4.55290327
IL23A	0.10730681	-0.00686222	0.73445241	0.9878474	0.5022562	0.28006903	-0.00686222	-0.00363717	-0.25197725	-0.25197725	-0.25197725	-0.25197725	-0.25197725
ATP5B	6.94145946	6.84171166	6.83182385	7.35758341	6.33376173	7.07887084	6.83324989	6.46819022	6.61492919	7.12632903	6.43085642	6.96971583	7.93911014
PTGES3	6.04764152	6.84664714	6.5506514	7.52390376	7.54795877	7.44267139	7.72813544	7.21623652	7.31047936	6.2571982	7.19641904	7.17629102	5.7248652
BCL7A	5.02226759	3.67959327	4.63277045	2.78353913	4.10909355	5.09011507	4.23541678	4.25975686	3.8696025	5.39656324	4.42245426	5.04839219	4.34888978
RSRC2	5.38843015	5.70302873	5.70216201	6.26832243	6.23307921	6.18871435	6.41458974	6.38882471	6.27033941	6.23088976	5.69692001	5.33395746	6.19612121
CYP27B1	0.80724856	1.16636901	0.86704657	2.12030564	0.5996212	0.25566468	0.41563088	0.31923751	-0.63788676	-0.63788676	-0.63788676	-0.63788676	3.40432166
LIN7A	0.51456419	0.74261605	1.0389264	1.95523295	1.58553396	2.70455076	1.19583046	1.63441825	3.48246375	-0.65199406	2.8564321	-0.65199406	3.74255099
KRT18	0.96167164	6.44370946	0.6100241	4.19774963	2.21637334	2.2812895	0.85134469	0.70082028	-0.13495032	-0.83403741	4.69474344	-0.83403741	4.16527687
ACSS3	5.13334004	4.18951001	4.3745455	4.33011161	4.89267231	5.14550241	4.84188617	5.1293106	4.65935909	5.23533591	3.95123466	4.52462857	5.10062369
GLI1	2.89382247	1.43314553	3.2727797	1.41154222	2.42375777	2.09301716	2.75071249	2.76127921	0.74519385	4.20248957	3.61895447	0.30810654	-1.024766
PPM1H	4.88115837	4.7301235	4.42020909	3.78265652	4.49295494	5.34546907	5.77757361	4.12504242	5.72518375	5.63303177	5.24045368	5.56850415	5.30941609
METAP2	5.72800286	6.72749181	6.41657718	7.2436972	6.79493104	7.16580168	7.04258913	7.13042904	6.75160216	6.92548699	6.17305945	6.17660771	6.3735985
LTA4H	5.15646382	5.19944136	4.77007577	5.80846472	5.41172749	5.60044942	5.54051957	5.50556054	5.4626178	5.73940434	5.06180296	5.31469354	4.80953334
ELK3	6.70881419	5.90117251	6.93629519	7.48482425	6.46905858	5.29240957	6.03969055	6.38986748	5.98913356	5.05106002	6.18124006	5.41173452	5.54628661
SLC6A12	1.86968864	0.47257289	1.34342973	1.0027862	0.75920026	0.47643123	0.5927617	0.48303658	2.89277751	-0.42385274	-0.42385274	-0.42385274	0.87855975
WNT5B	1.51397566	1.42847266	3.83739461	2.67114174	1.01329263	0.92413354	1.55304598	3.69036554	2.97179674	3.56331381	3.56122638	3.18246383	3.88819957
MAGOHB	3.40114926	3.45242972	3.59779472	5.63370393	4.92554409	4.43730707	4.83052489	4.85346271	4.94260827	2.39238627	4.27846784	3.97369039	4.06075223
TRPV4	3.52214959	2.6081145	2.75050902	3.5148105	1.88497464	1.00047518	2.24849305	1.35720203	3.52885468	2.37023627	1.94471812	-0.85435665	4.11439117
ITFG2	5.12248612	5.18795371	4.88029035	4.96407002	4.83221346	4.34866122	4.5559882	4.44992641	4.49905758	4.6680792	4.49283276	4.98733279	4.54077125
FOXM1	2.52138798	1.45650798	4.84011246	5.09710275	0.95239827	0.96557555	2.37146477	0.99259337	2.54146141	-1.22338529	2.90443014	2.53158757	3.26251798
PRR4	-0.33434981	1.56918403	0.30752272	1.49351295	1.06625106	1.12861376	0.99935883	2.96958994	0.06411343	0.06411343	0.06411343	3.22102294	2.28308311
PARP11	4.19222078	4.80641193	4.00474837	4.87949363	4.79317773	4.79475691	4.38811836	5.23645998	4.34392346	4.25982099	4.55608905	4.6755249	3.77498913
ARPC3	5.20951643	5.10180705	5.84648607	6.6308818	5.90279699	6.32375247	6.03305339	6.09955504	6.39238063	5.96320007	5.47419528	5.58278368	5.46931741
GPN3	3.11611256	3.96712191	3.42114947	4.10334218	4.0039639	4.88359488	4.48124835	4.14597194	4.56142039	4.22357536	4.14656318	4.40030834	1.70799513
VPS29	5.47287136	6.079719	6.08799302	6.66299659	6.64713715	6.9129715	6.95103389	6.69787412	6.56826578	5.78488957	6.32559172	6.07983641	6.29436126
MYL2	0.35397561	0.35397561	0.94926975	0.35397561	0.53326054	4.19080449	0.51918817	2.3331242	2.01458297	-0.38629345	2.32352063	-0.38629345	6.02795828
RAD51AP1	1.11425432	1.71071618	2.52561192	3.46311637	1.47095377	1.15461905	2.15017266	1.22969701	3.51909414	-0.93557316	-0.93557316	2.67611639	2.01290684
CUX2	0.15465557	0.14586868	0.56373746	0.36516294	0.26788172	0.26160222	0.31613462	0.14915767	0.09454773	0.09454773	0.09454773	0.09454773	0.09454773
SH2B3	5.77633781	5.89950716	5.98454963	6.59083315	5.55206676	4.6967183	5.06799628	4.98652976	5.3200516	5.36385283	5.50606952	4.80662183	5.77862138
AKAP3	1.61191044	0.47600737	1.58674497	1.45502538	1.21112142	2.03896087	0.84390941	1.90133249	2.09547908	-0.56452964	-0.56452964	2.53760032	-0.56452964
MANSC1	6.11585065	5.22267526	6.1361376	4.83523155	5.86357023	6.04512164	5.64138213	6.16440302	6.30048209	6.04064721	5.82917398	5.74420849	6.16773718
KCNA1	0.62467976	0.47114593	0.54608233	0.64346047	0.56403435	1.10601598	1.17491034	2.43990383	0.84620043	2.6610685	-0.34243281	3.78749013	3.93835763
DUSP16	5.87088875	5.48846597	5.02957127	4.80753547	5.45016289	5.74220474	5.46694891	6.12323329	4.97644622	5.80777178	5.17653854	6.32692359	5.80925637
CREBL2	1.4562052	3.40743306	1.08429353	3.23004419	3.50081909	3.51351765	3.32117883	3.56582278	4.01059034	4.83622363	0.60482067	4.14468371	-0.45930469
ACAD10	4.69516977	4.14950974	4.90347276	4.50596604	4.27970616	3.44817937	4.42706346	3.95377153	4.27814633	5.10997331	4.30205661	4.44233268	4.35274141
ALDH2	5.27403673	5.33713511	5.06940117	4.20514288	5.27527438	6.13608585	5.36163058	5.74488753	5.25098363	6.53086015	5.31646362	6.16357727	5.44304726
CDKN1B	6.05947948	5.65987006	5.55569565	5.19759475	6.22429637	6.73049526	6.28988541	6.6367825	6.39201681	6.20778028	5.6282605	6.26992596	5.09809467
GPRC5D	0.04243131	-0.20221964	0.3498501	0.00300304	0.70930241	-0.20090275	0.45292608	0.65162087	-0.04492339	-0.04492339	-0.04492339	-0.04492339	-0.04492339
NAA25	4.08753946	4.69934682	4.20346202	5.80331479	4.7792807	4.65403777	5.33049376	5.07243243	4.12628723	3.48018028	5.16686035	4.49437979	4.56987939
SCNN1A	0.20252875	1.55223301	0.47166219	0.2434178	0.36437113	-0.13023655	0.03148256	0.56760451	-0.0492844	-0.0492844	-0.0492844	-0.0492844	-0.0492844
LTBR	6.02426467	5.64851697	6.25236164	6.03603497	5.59617631	4.79482698	5.16209743	5.41396589	5.44316411	5.58472968	5.68734698	4.97181206	5.56343973
OGFOD2	2.37767534	2.83987264	2.5400311	2.30028826	2.60108579	2.46368425	2.80632557	2.88684036	3.37991611	3.78098871	1.99103335	3.53346627	1.2344972
CDK2AP1	5.06480594	5.52599511	5.31869451	6.00024981	5.75739175	5.00193964	5.63649223	6.00426302	6.05920204	5.67421612	5.75646216	5.73800272	4.47817871

OAS3	5.83336737	6.21998945	6.35837968	7.01146675	5.21653345	4.85995902	4.46041211	3.95139973	5.98945635	5.14954167	6.33932999	4.08294785	7.02273053
OAS2	5.12206253	5.5541608	5.11175463	6.69100966	4.67371447	4.34402612	3.89895936	3.37910124	5.54497317	3.90161461	5.6567328	3.59126638	6.59684882
ART4	4.20880238	5.92680753	3.7071885	4.22486208	4.88862707	5.02817749	3.9733013	4.63399032	4.83104217	4.47740791	5.55056013	5.02437449	5.35641215
MGP	10.5237215	10.6814538	9.9470762	9.86783713	9.9470762	11.5519101	10.0049081	10.2982248	10.4554432	9.6018545	10.5465674	9.67595244	9.63958436
RASAL1	0.07911571	0.29343769	0.08170279	0.12694908	0.07911571	0.08055409	0.07911571	0.08241621	-0.05334895	-0.05334895	-0.05334895	-0.05334895	-0.05334895
ARHGDIB	7.3758168	7.17111211	7.07551483	7.2527231	6.34695994	7.17111211	5.94835321	5.82215112	7.42649385	6.56235651	7.04559958	6.14890853	7.41011999
GTF2H3	4.34043038	4.30684892	4.95204713	5.23412392	5.039675	4.88543474	5.12060131	4.65304793	4.65618092	4.17468801	4.83122434	4.77573297	5.06876649
EIF2B1	6.06761734	5.88280438	5.43178002	5.76099092	5.96503398	5.71213913	6.33552484	6.23337713	5.9439379	5.39961959	5.90281054	5.81736578	6.11342487
DDX55	5.08488487	4.85365285	5.2337023	4.66352556	4.42342013	4.22559873	5.09749454	4.65937143	5.01240087	4.78622823	4.2234034	4.77934294	5.39014287
SLC38A1	7.06135545	6.49112982	5.35312766	6.61131191	6.22258125	6.85860596	6.17442312	6.87747427	6.54156067	5.6341512	6.53508701	5.38781502	6.36427082
RERGL	2.23891803	1.79916101	1.67064458	2.2080363	5.09974483	6.35208064	3.33693854	4.73192629	5.05878441	3.41244969	4.93142692	2.15482591	4.8406257
ENDOU	3.89809002	1.09996166	1.92561059	0.75411785	1.22888183	0.5679944	0.6844378	1.310845	-0.55376363	-0.55376363	-0.55376363	-0.55376363	-0.55376363
C12orf49	4.81460542	4.72562851	5.02336455	5.14385013	4.71871505	4.71871505	5.16543327	4.50551647	5.08113499	4.45037352	5.04466746	4.65029178	5.06089845
VDR	5.0176205	3.37511657	5.49710193	5.17459237	3.36391661	3.9024753	4.04782619	3.94272271	3.64045315	3.39964685	3.74963166	3.5894081	3.95691061
FZD10	0.55756092	0.98114906	0.7409	0.56151886	0.52736888	0.30893482	0.34976317	0.71953313	-0.33645345	-0.33645345	3.78922237	4.10517532	-0.33645345
RFC5	3.92161569	3.27988043	3.83694806	4.14446761	3.52836809	4.08795583	4.09896991	3.78426145	3.61895493	3.8522647	3.42183099	4.04918524	3.83464669
STX2	5.32403874	5.80703261	5.21819476	5.43776164	6.16696047	5.29165463	6.08047008	5.86996275	5.7937283	5.69080517	5.71645227	6.07753713	5.9138257
COPZ1	4.04562552	4.39462757	3.92907412	5.35591704	3.94266838	3.90813529	3.91975135	3.6840148	4.17477996	4.26386753	3.58564139	4.24713522	2.42348198
TBC1D30	1.97744783	2.41500236	1.50317768	2.02072753	1.35105551	1.08653717	2.3293059	1.74510554	0.41201099	3.03703216	3.42255219	1.74086609	2.72224821
CAND1	5.60321396	6.29807614	5.8817285	6.80346221	6.43310908	6.58960569	6.55874381	6.50960858	6.4570917	6.19860963	6.70145457	6.8524285	6.05427797
IL26	0	0.09223956	0.00124472	0.43564788	0	0.00069205	0	0.00158797	0	0	0	0	0
RAB5B	2.24999449	2.01448239	1.09193471	0.92314658	2.7207488	2.73788251	2.34038805	2.57284908	0.57549137	0.57549137	4.29134467	2.11043385	0.57549137
MDM1	3.85593164	4.35995985	3.98462843	4.72510794	4.6010711	5.24614477	4.80356538	4.70834435	4.53401746	4.85065433	4.53295948	5.05400173	4.17045692
NUP107	3.85537089	4.18364809	4.18839793	5.11802814	4.85140676	4.57738774	4.71502996	4.78637828	4.662365	5.42537032	4.49173761	5.03775123	4.68538665
CNOT2	5.71181955	6.24061196	5.40898599	6.59224257	6.35976016	6.5995879	6.34820507	6.59367245	6.06668671	6.38127916	5.88227328	6.25209174	6.35682423
TIMELESS	3.87396954	2.52025597	4.7962753	4.50645936	2.95551095	2.70595669	3.67004353	2.62609638	3.04428197	2.05238064	0.5638618	2.47104675	3.32669202
CPSF6	5.65971566	5.55002986	5.66561088	6.24580903	6.24408569	6.21345765	6.32022081	6.28861409	5.84673834	5.84388427	6.51621737	6.09008865	5.7035872
KRR1	4.8532768	5.72658231	5.00053206	5.80511997	5.90648978	6.60882063	5.87968043	6.03050346	6.0646328	5.13168584	5.56860865	5.46047557	4.5316177
MRPL51	5.2488534	4.74684592	5.56455522	5.57710444	5.3659395	6.4117426	4.7761267	5.22362119	5.90665169	6.04756014	5.15363266	5.48158996	6.03562986
GAPDH	9.69332566	8.82217127	10.415629	9.54795304	8.82217127	9.20359781	8.95321105	8.08641004	8.64728049	8.96630939	8.65317403	8.96630939	8.72163911
NOP2	4.96228891	4.89359632	5.73525928	5.19500909	4.24626147	4.3135809	4.2631176	3.92207731	4.50762081	4.33300882	4.21647388	4.13530216	5.32012267
CHD4	6.91073699	6.5304441	6.96389797	6.47073968	5.9240732	6.05843116	6.54644545	6.02241493	6.14226682	6.4958525	6.29556698	5.88086822	6.94663366
ACRBP	2.14461439	1.05618771	2.38871215	2.10690503	0.70596264	0.62306388	0.85524036	0.52076478	-0.3485855	3.1238014	-0.3485855	-0.3485855	1.76644667
UHRF1BP1L	5.85514146	6.43640874	6.1185958	6.74517752	6.81881687	6.81133481	6.95232107	7.07822472	6.55843721	6.22805608	6.08783685	6.57940159	6.25014263
COPS7A	5.57551108	5.64000151	6.14886525	5.68184277	5.21355844	5.07120998	5.38912764	5.22066505	5.54406851	6.00472429	5.88532672	5.96807931	5.78235864
ING4	4.96231878	4.63175793	4.59829176	4.25894209	4.34655202	5.13688957	5.23722288	4.65158626	4.65794674	5.0104122	4.82153873	4.90512761	4.55273725
GNB3	4.11582706	2.77392451	3.91425856	2.7854704	3.95017951	2.73459616	2.18130085	3.4621058	0.33022847	3.72341012	2.90901226	3.34345341	4.17977134
CDCA3	0.87373407	0.00577063	4.01749354	1.83644966	-0.18102137	0.44525405	1.04297403	0.69097692	0.74566924	2.2553989	-0.46351106	3.06839202	-0.46351106
CHPT1	4.23036804	4.86777159	3.88017486	4.18412281	5.00569688	5.1273582	5.52364711	5.65966246	5.29324094	3.61218235	5.19550354	5.28772402	5.03370402
USP5	5.98549232	5.95645823	6.70656529	5.75501607	5.28309364	5.48983973	5.69311853	5.22397851	5.67511655	6.59197925	5.67761075	5.95562634	5.6415615
TPI1	7.84962028	6.79335085	7.70154988	8.06640507	6.98018772	6.4908102	6.31954151	6.10137506	6.20371037	7.11171422	6.29867671	6.71416025	6.73897185
GNPTAB	4.01994246	4.85305652	4.20816463	5.25469887	4.79427089	4.93487095	5.0019674	4.5604571	4.91529791	4.87669051	4.75711772	5.13532982	4.11485109
SPSB2	3.11687346	2.36323999	4.76337519	2.6391228	2.63082035	2.34875411	3.132625	3.83777176	3.39586552	3.87166384	3.42077333	3.90539055	3.55213984
ENO2	6.70808529	5.89659657	5.98717045	4.7770292	6.41772137	5.43086158	5.90596999	5.50084793	5.02871891	5.90471274	5.96105535	5.59906127	4.84206664
ATN1	7.69551627	6.72531547	7.83090834	6.40076367	7.00969614	6.59701054	7.05325356	6.87522445	6.59773901	7.2870456	6.85503974	6.9457049	6.79127334
C12orf57	6.34357313	6.09140256	6.27469705	5.12062674	5.59123689	6.0172794	5.07671409	5.62791091	5.75438098	6.54292516	6.09600685	6.1073413	5.44139972
PTPN6	4.76081218	4.97022118	5.15737834	4.73333861	3.28582885	3.00890356	3.67670528	2.99532513	5.12209787	4.74956768	3.37327775	3.4953562	3.83378574

LPCAT3	6.06807048	4.35644702	5.08559058	5.10160561	4.49538544	3.90462005	4.0132547	4.1898487	3.29449672	3.44157181	4.59023653	3.69435788	4.94857733
NT5DC3	5.28279852	5.97270781	5.40077518	4.79725758	7.5916643	6.62403019	6.85779522	7.50723394	6.50362053	6.78341421	6.52039072	7.32311156	6.92750406
NANOG	0.04200181	-0.003693	0.36991853	-0.003693	-0.003693	0.08639801	0.02907484	-0.00043236	-0.00387377	-0.00387377	-0.00387377	-0.00387377	-0.00387377
SUDS3	5.91973421	6.25058429	5.59932565	5.83587572	6.08653214	5.7614949	6.50056584	6.0071971	6.00283705	6.00221272	5.7183912	5.93617672	6.11213697
GOLT1B	3.97219667	4.98180141	4.33694571	6.68067973	5.7803785	5.84261211	5.81724547	5.68845	5.8796303	4.15404633	5.81750453	5.76375768	3.21708294
GYS2	0.50652358	0.56936828	0.45762961	0.63188287	0.61960106	1.07571774	0.66966537	0.57094002	-0.29057475	-0.29057475	-0.29057475	-0.29057475	-0.29057475
LDHB	6.09666161	6.68198427	6.64277423	7.64910993	6.62669469	7.15599997	6.91678435	6.8531419	6.84209427	7.24064676	6.36024606	6.85959858	7.7842271
PRKAB1	5.21137378	4.91211132	4.99229453	4.25876179	4.78245808	4.64090126	4.58130749	4.88622989	4.61527473	5.36329313	4.64999595	5.09144028	4.59880732
CMAS	4.49412935	5.06107603	5.13845717	4.53921114	5.23083437	5.68869633	5.21585181	5.21382445	5.6516734	4.56229121	5.42588775	5.34495214	5.10023466
HCFC2	5.50887124	5.81363393	5.32091061	5.85508523	6.18652009	6.3914555	6.20582814	6.34476532	6.15266952	5.85513187	6.50510476	6.28696282	5.87916505
ST8SIA1	4.46970351	4.4009436	4.07318813	3.50522625	3.47802113	3.37528013	3.92000343	4.36923492	4.40234087	2.39946623	5.0903592	3.67562709	3.21166926
CLEC4A	1.4977441	1.45374506	2.18103688	2.13458047	2.74047409	1.73258032	1.76619003	2.18435587	4.14967448	-1.03152532	2.47318263	-1.03152532	2.93172509
RAB35	5.69680419	5.06005038	5.80657662	5.04012884	4.88028131	4.82013467	5.12986557	4.86757436	5.30059291	5.42016764	4.87404535	5.38878273	5.4049962
PHC1	5.56668795	4.7874367	4.9424026	4.23897604	5.21693878	5.08855119	5.21827493	4.97667186	4.80984672	4.64623685	4.65363962	4.66312626	5.40909069
COX6A1	4.71103668	4.95450175	4.08996615	4.7084422	5.32856207	5.29088873	5.27083139	5.01259812	4.95235578	5.70434478	4.92205151	5.40093511	4.56574293
RIC8B	5.12064614	5.34729441	4.5838378	5.10412101	5.24228005	5.78323216	5.46776528	5.3696151	5.10297763	5.42358156	4.85618909	5.22630842	5.62584004
SRSF9	5.67424602	5.93031562	5.87983523	5.75486016	4.74498311	4.58396334	5.50057864	5.56956549	5.16862036	5.11228075	5.95998102	5.18226367	4.88485006
FGFR1OP2	5.12513587	5.74447372	4.79311166	5.75762744	5.77189005	5.86223133	6.24468979	6.05094069	5.94053845	5.45792347	5.93131196	5.61043561	4.65607465
KLRB1	1.57452038	3.11811743	0.9813417	0.97889499	1.21642861	1.1912531	1.2896275	0.98201642	3.56131162	-0.6466306	1.19962046	3.05736625	-0.6466306
COL12A1	5.59659888	7.22585085	5.72513441	8.90966901	8.17532268	7.66992517	6.37342558	7.46959163	7.25303403	7.37641058	8.18535225	8.05754102	8.35581784
TDP2	5.58035377	5.44574	5.90322476	6.21796131	6.14067843	6.21656157	6.17307951	5.38393701	5.69139516	6.02942343	5.60047086	5.55837942	5.34444682
FRK	4.91585644	5.21744383	5.18582425	4.72869727	6.00013139	6.32713716	6.37013207	5.90094477	5.92042674	5.83528776	5.90776023	6.12052439	5.80010269
DSE	3.32716026	3.51600029	2.76857431	4.80452585	2.30341394	2.50841316	2.55521718	2.16791912	3.01448553	2.6092664	2.74261303	2.68046713	3.01448553
RWDD1	4.81209538	5.31492653	4.70859036	5.14747054	5.47250947	5.91586668	5.34156275	5.52616927	6.37279061	5.68299055	5.05689993	5.41160009	5.20860788
RSPH4A	1.0447112	1.47495223	1.30871988	1.37338158	1.88261486	1.33673708	1.06724991	1.91082978	3.15963066	2.49524539	2.05270436	2.05270436	3.45209537
MAK	0.79780396	0.69646151	0.62072377	0.64325775	0.53585799	0.5152839	0.51383627	0.51715801	-0.54711991	-0.54711991	-0.54711991	-0.54711991	-0.54711991
TMEM14C	6.19778164	6.09557944	5.28804863	5.92102429	6.03147534	6.81261953	6.25501559	6.05552504	6.61505654	6.17096839	5.96635274	6.5430316	5.65865936
PAK1IP1	4.29238748	3.92001361	3.7905012	4.6179089	4.46745448	4.55281956	4.2089898	4.84016463	5.19550099	4.34396403	3.60497866	4.07476243	3.85442484
GCNT2	4.07229612	3.65045947	4.47548862	4.00145248	5.08421216	5.24299846	6.04293642	5.28270027	5.10603386	4.9978353	4.67685892	3.11868668	5.11479054
NEDD9	6.30928332	6.46062786	5.930919	5.83335546	8.562199	7.89990463	6.82157372	8.13119973	6.0892044	6.50418912	6.03761718	7.34917039	7.57054135
ASF1A	4.0575001	4.38670951	3.90219786	4.36065429	4.6092016	5.61461546	4.77989992	4.56604571	5.56002385	4.15765242	4.94249099	4.66909642	4.36366254
MCM9	4.25176196	4.58879839	4.0144123	4.47709646	4.00063711	4.15863246	4.03183182	4.40754698	4.15895987	4.65196736	4.80123345	3.37541092	4.13756502
FAM184A	3.15509085	3.38579282	3.60415314	2.95290172	3.66172774	4.39239655	3.74216411	3.93937868	3.60430852	3.74539405	3.3971148	3.00367947	3.67805709
RNGTT	4.04824016	4.41712428	4.43907056	5.12403067	4.81937946	5.40152166	5.11597534	4.92160037	5.13543546	4.45423582	4.79437702	4.58454363	4.0692318
MAN1A1	5.68199739	6.0308917	5.47557072	6.69748606	6.51193903	6.37007445	6.25743548	6.46286052	6.07299674	5.52715386	6.47544431	6.14851973	6.08326861
GABRR2	2.24995307	1.78760651	2.05312304	1.31440854	2.28085609	0.948295	2.90121466	2.68885346	-0.66124886	-0.66124886	-0.66124886	1.53158158	-0.66124886
SERINC1	7.94021069	8.5378088	7.85901271	8.18253921	8.63608261	8.61845422	8.91669862	8.75845632	8.62557908	8.2193547	8.56465423	8.55342151	8.25381961
HDDC2	5.26471864	5.09045018	4.98524391	4.83984922	5.27191539	5.62690182	4.65385862	5.21572402	5.6745271	5.42113752	4.79427568	5.0093093	4.96841539
TPD52L1	2.69174418	2.66207004	1.72386484	0.66400075	1.14086199	2.20513325	1.97531679	2.05980355	2.59902429	-0.09076212	-0.09076212	-0.09076212	3.05196935
HINT3	5.11418443	5.43138885	4.92447577	5.53765423	5.95204054	6.87842974	6.42229639	5.95036069	6.18327529	4.34692696	6.18836885	6.06453485	3.9118401
NCOA7	6.3610576	6.3887362	5.03985623	6.66473223	5.85955214	5.80264423	6.13575133	6.44140357	5.87786866	5.41742823	5.34976798	5.21210353	6.17862979
SASH1	5.34653845	4.94976402	5.15901553	4.71861463	4.66558972	4.52622998	5.47004158	5.54278268	5.00435608	6.21496556	4.88533998	5.24680573	5.75761658
UST	5.24850275	3.6152304	4.51600457	3.55313665	4.37182413	4.43784816	4.46085071	4.87968907	4.73505553	4.23512771	3.61285111	4.49659736	4.43179773
ULBP1	0.5716711	0.67530237	0.55565776	0.36829622	0.38896232	0.57102097	0.3572998	0.36061556	-0.3039894	-0.3039894	-0.3039894	-0.3039894	-0.3039894
FBXO5	3.74145075	3.01752845	3.96302302	5.13029758	3.25486508	4.08440292	3.76445275	4.44674353	3.83006664	3.39253482	2.9594104	3.95545896	3.81890563
MTRF1L	5.03803978	5.57014328	4.43031992	5.57391849	5.38439351	5.67239488	5.52733617	5.59164188	6.08393637	5.40528228	5.22307956	5.91349567	5.14417856
PPARD	5.75154395	4.93493429	5.97210869	5.72437663	5.02956919	4.75802758	4.73797399	4.6234421	4.60393165	5.13748239	5.12641972	4.43397854	4.7940196

FANCE	3.80195632	2.76309703	3.98591229	1.60599859	3.07774179	2.57394743	3.03898099	2.69246918	2.01251239	4.25601242	-0.9932939	3.50626864	2.22696419
TULP1	0.21168348	-0.04006155	0.22033808	0.10176628	0.21093487	0.24464102	0.32367169	-0.03677042	-0.03935117	-0.03935117	-0.03935117	-0.03935117	-0.03935117
SLC26A8	0.31109944	0	0.05413843	0.35427683	0.16785502	0.15867245	0.14670879	0.15581224	0	0	0	0	0
MAPK14	5.02787996	5.5708224	4.86790452	5.60057988	5.32512846	4.99047229	5.7507776	5.40752492	4.83188412	4.87709709	5.61028037	5.57791097	4.85688245
KCTD20	6.27050117	6.91209385	6.34196293	7.06264216	6.96942768	6.691605	6.691605	6.92935109	6.86571836	6.48891578	7.14368848	6.86020615	6.99352368
STK38	5.60324644	6.13265144	5.50345102	6.43346012	6.20904766	5.68091558	6.341613	6.09166766	5.79441393	5.68619408	5.92313452	5.61087439	6.03754187
SRSF3	6.33084816	6.56779434	6.53269146	7.44078488	6.95986132	7.11761686	7.08475474	6.53948459	6.84889712	6.72429012	6.83980784	6.83144785	6.40209019
SOD2	8.9564076	7.40761904	7.06513936	9.14404663	6.46648358	6.14618377	7.90595547	6.38820126	6.48244979	6.06540789	6.26816137	6.06782673	6.94243753
MRPL18	5.22763823	5.51871641	4.8215441	5.45387233	5.14444126	5.97526756	4.90282194	4.7209447	5.6131443	5.99274271	5.35161938	5.35694591	5.2117403
MCM3	6.01950636	5.54437067	6.37423513	6.29140328	5.54715601	5.71591286	5.87900477	5.31907119	5.75790124	6.20504123	5.42772497	6.11269492	6.13362812
RNF8	4.42378462	4.6386701	4.87917225	4.32958891	4.93541179	5.10838862	4.92823544	5.12805581	5.11234404	4.97246881	4.3314307	4.62665894	4.64063454
PHACTR1	5.0031551	5.24420715	4.90078901	3.62815629	5.22310096	5.89310348	5.52286779	6.25594851	5.25285336	6.29450458	4.35538371	5.81653092	4.92862422
MDGA1	5.4058342	3.91521728	4.55286287	3.62348358	4.30919019	3.61978932	3.77584312	4.22429513	4.65274036	3.84473739	5.03784584	3.85735482	4.51020736
ICK	5.77252371	6.2907995	5.14334048	6.21587523	5.95366987	6.34753647	5.88854575	5.77219995	6.44427966	5.88245092	6.20936765	6.17604211	6.05964643
FBXO9	5.50219024	5.62933827	5.08492386	5.81380273	5.83037905	5.44830073	5.97369839	6.15166138	6.08975582	5.31447344	5.68519155	6.26008282	6.01565762
CD83	4.54711821	3.86839766	4.88537912	4.68640376	2.77397879	2.5803784	2.68086788	2.89920232	3.8989233	3.21694435	3.46638733	3.90209066	3.07131095
MDN1	6.79241408	6.66802339	6.56382941	6.65778921	6.28532523	6.39603303	6.81443257	6.26813065	6.17425502	6.35690495	6.55018691	6.34625658	7.06146804
BMP5	0.23719661	0.83114138	0.40319508	0.24547628	0.50910922	0.94311243	1.06198813	0.24044396	-0.08196374	-0.08196374	-0.08196374	1.79119838	4.18690447
BACH2	4.37121502	3.69947425	3.86924689	3.58060739	5.50646643	6.04069785	4.047696	6.26756541	4.72894978	5.96958721	4.8896117	6.21349913	5.5802009
RBM24	3.46242289	2.79049233	2.21075607	2.32935329	3.91784104	3.8916756	2.88628272	3.63418703	3.0660331	3.84042243	3.20917281	2.68667158	6.51505654
CAP2	4.91029036	5.37003327	4.4899008	4.0066855	6.14578432	6.2962888	5.29993016	5.18481914	5.6535665	5.96628424	5.89124528	5.82990849	5.83220673
TREML2	1.15495282	0.786294	1.35508592	1.12029691	1.06211011	0.62634931	0.62491539	0.7378238	2.61476248	-0.42094041	-0.42094041	-0.42094041	2.11194386
ZNF451	5.57142933	6.08437688	5.52513651	6.60080568	6.36781221	6.25325555	6.45563829	6.44028689	6.20188752	6.07297073	6.49501037	6.49548058	5.76471143
BAG2	4.9453048	5.0720608	5.03179413	5.35556961	6.75970796	6.55001517	6.24384913	6.49992782	5.76201908	5.7994543	5.68033081	6.81589615	5.62965576
RAB23	5.30988181	5.84127226	5.81574702	5.86813296	7.4373981	7.13284635	6.78783438	7.53971164	7.17635245	6.8733547	6.53875768	7.5605428	6.25861987
FHL5	6.06904271	6.48303522	4.75061357	4.18082219	7.82811178	7.4064216	6.6762965	6.1044416	5.33473424	5.08043084	6.58857819	6.1913255	6.02198162
GPR63	3.76654662	3.58164855	2.99475553	2.49955406	4.2587225	4.35169282	4.06567793	3.02725535	3.8942876	3.37292766	2.25761749	3.74198574	2.95874661
KHDRBS2	0.84701991	0.94965129	0.63124923	1.12328397	0.79423476	0.48165842	0.62426219	0.48352133	3.30614544	4.19340857	-0.34340055	-0.34340055	-0.34340055
FBXL4	5.2996437	5.22933419	5.10959622	5.43345765	5.94472409	6.02120604	6.23795269	6.54427036	5.39800143	6.69729652	5.5340962	5.9695324	5.65983114
CCNC	4.28363299	5.16216947	4.46428497	5.78084151	5.34522786	5.31611854	5.45712132	5.57978491	5.37636477	4.36510482	5.30698217	5.44140883	4.63315214
E2F3	5.86927255	6.01770718	5.35718156	5.96234	5.09421121	5.04476859	5.19591836	4.3752538	5.00493157	4.3596181	5.12218922	4.93970176	4.23661475
PTP4A1	2.69846005	3.46197848	2.89231443	4.94329523	3.93855166	4.18999456	4.03947983	3.41012224	0	0	0	0	0
SIM1	0.00967355	0.36465814	0.0122145	0.01650768	0.00967355	0.26755636	0.00967355	0.09062982	0.40368791	-0.11077934	-0.11077934	1.03682181	-0.11077934
ASCC3	3.88545761	4.81379871	4.14681522	5.80846906	5.05386883	5.08851535	5.05170288	4.56884675	4.93878783	4.78176908	5.28105396	5.30812656	4.1872673
BVES	4.49095646	4.63734177	4.36555752	4.28749426	5.92001035	5.77046831	5.55798515	5.78547411	4.93022265	5.32613301	5.08428745	6.26766307	5.64177685
MED23	5.82004073	6.05560679	5.3318199	6.14921364	6.13129594	6.22769593	6.34387907	6.31437213	5.85441151	5.6348379	6.22594716	6.21078533	5.99855613
WASF1	4.78298271	3.18852961	3.46890441	4.69460787	4.71260177	3.3524602	3.95075297	4.02227524	4.68708461	2.596002	2.92677649	3.69298094	3.2754489
GPLD1	5.40188199	4.85614508	5.36754829	3.30020927	5.44078018	5.71221694	5.44305602	6.62874807	5.77581897	6.14560191	5.97562745	6.30620389	5.72033149
ALDH5A1	4.91108071	4.51608809	4.28826451	3.38796295	4.68898769	4.98783573	4.40084231	5.04294131	4.93862161	4.17710483	5.50968432	5.13550368	4.98863184
VNN1	3.21482319	3.38128879	1.96984692	3.47290096	2.81405995	1.89969979	3.189435	2.1337438	4.32164102	2.6680355	3.73169268	-1.69456429	3.48802936
VNN2	5.14314425	3.04883223	1.97430423	5.27004491	3.86926525	1.04712171	3.01043706	3.11555564	4.89176725	-1.37593357	3.60685105	2.23429955	4.58704989
ACOT13	4.08805294	4.04886405	4.25913612	4.16767479	4.16648567	5.22455866	4.56220218	4.09841917	4.87131231	3.96511607	4.06644289	3.87138808	4.71945265
SMAP1	2.84310961	2.29563218	1.92657595	2.81901936	2.60866942	3.12970206	3.08969016	2.80480985	0.83126439	3.24593646	3.39960547	2.40661666	-0.34565582
RPS12	6.79791822	7.61821748	6.71552467	7.07379672	6.29779056	6.3440674	6.64644564	6.34041625	6.81848728	7.14490588	6.12319376	6.61755034	6.72404449
C6orf62	5.87551112	6.36124831	6.25733206	7.16230632	6.38097811	5.9093479	6.32478694	6.27166879	6.24192279	5.76134278	6.63613079	6.34303952	6.04386393
GMNN	2.42661715	2.51034053	4.08581063	4.75066564	2.73104406	2.81910034	3.82113384	3.49935381	2.49069664	1.83257237	3.88722716	3.25894452	1.42308213
EYA4	2.21848759	1.77393582	1.9415193	3.10341286	2.87606203	2.70146653	2.65779147	1.70183035	3.37341663	2.71695453	2.67730854	2.97640948	3.42877136

SOBP	5.81309945	5.1557467	5.59104535	4.68501295	5.22200396	5.66029789	5.47424693	5.54927798	5.67596605	5.74096277	5.28161055	5.38248409	5.60921586
SNX3	6.39936156	6.61212259	6.39570757	7.31717929	7.47801386	7.42454725	7.15601094	7.08684213	6.98073054	6.76602411	6.35680247	6.84919437	7.07923766
HBS1L	4.68126885	4.79127268	4.72546683	5.35874418	5.23962269	5.66783795	5.13650549	5.28824811	5.09629638	4.63737166	5.2376445	5.21371176	5.01954876
TRIM38	5.86030039	6.4114042	5.91628513	6.55264916	6.61127915	6.55661949	6.41524982	6.57527429	6.30769094	6.59699802	6.18851073	6.06087643	6.46304456
PEX7	3.64018897	2.75796279	3.86645125	4.18824152	4.06557676	3.6894284	3.57685224	3.43608226	3.65959838	2.50157884	2.64867832	4.38231275	3.86889415
ZBTB24	5.2433948	4.947989	4.82917091	5.3559485	5.28133178	5.37374394	5.23012759	4.91070688	4.95065169	4.56910747	5.35575813	5.21580379	5.62185205
FIG4	4.88596955	5.04770308	4.74289542	5.04245601	5.40261689	5.62500513	5.46061285	5.47696232	5.32142291	5.34022037	5.47093064	5.31156042	5.17622249
PERP	4.8706329	5.74331672	4.64755733	4.70832243	5.73772892	6.12868735	5.49711774	5.51211654	5.41276324	4.08915804	5.90677464	5.62738738	4.6144945
SLC16A10	2.61215279	2.88585039	3.74233858	5.04363132	1.95645989	2.21465131	2.26195683	2.30996631	2.45635719	3.22519316	-1.40283481	3.06208038	0.49305221
HECA	6.03559584	5.85241045	5.8999953	6.08135671	6.62457009	6.13859965	6.93336992	6.8471342	6.11120465	6.02977651	6.42290297	6.50052752	5.69771783
PHACTR2	6.13780285	6.9494141	6.05971606	6.65534219	6.911268	6.84872879	6.45047603	7.17242314	7.3464747	6.47320724	7.05171045	6.61438321	7.12469685
EPM2A	4.49623854	4.99026988	4.45755571	4.0408777	5.2885097	5.43074932	5.17520505	5.40451517	5.15657961	5.23603858	5.050032	5.70396906	5.33861651
SLC39A7	6.30786969	6.01911299	6.65546913	6.84643715	5.84620286	5.71734781	5.85745388	6.16779386	6.41138699	6.60968535	6.52602761	6.61858971	6.06682713
SLC22A2	1.28342536	0.88930987	0.81297094	0.98783575	1.15062597	1.72269702	1.18952525	1.51701791	-0.01982639	3.48894372	-0.47709924	3.12672913	-0.47709924
PHF1	5.90025703	6.02788481	5.94320768	4.44588074	5.81141208	5.20328301	5.87765607	5.54764293	5.8036602	6.04244805	5.58605802	5.67104233	5.22525473
CUTA	5.55330496	5.29669248	5.75546501	5.14027575	5.26338808	5.78499807	4.8997459	4.87477364	5.20373568	6.02061078	5.19849398	5.8029255	5.27569736
PACRG	0.37724586	-0.22745156	0.64434919	0.88764506	-0.25151467	0.70590575	0.03945343	0.5231272	-0.07418932	3.71991639	-0.07418932	-0.07418932	-0.07418932
QKI	6.41299557	6.83992197	6.07307171	7.49744496	6.87976791	7.00226212	7.09046233	7.109988	6.86999716	6.23312455	7.11427744	6.76428205	7.06328059
PDE10A	3.647416	4.55471272	3.49324667	4.51189226	4.93754939	3.39552628	4.85730641	4.73275878	4.39947784	4.12815184	5.66687304	4.81637922	4.11126586
MDFI	0.1682218	0.35417899	1.05278677	4.84378848	0.55188204	0.51745905	1.64146492	1.05181107	0.43440598	2.83267451	3.9518001	2.78383668	2.43849911
TFEB	4.90333321	4.24215855	5.13613313	3.71344449	3.84622263	4.35465433	4.3122983	4.04864313	3.95848798	4.90489729	4.2456168	5.08747692	4.62412797
SMOC2	8.2420272	8.26450733	8.27920071	6.62028365	8.28653221	8.34852457	8.58334638	8.50324717	8.30337292	8.83514292	8.59978816	8.97170379	8.34681435
CCND3	5.5787429	5.56737588	5.50229366	4.55569183	5.1699738	4.86343615	4.90364066	4.84076343	4.84891965	4.79038312	5.50154859	4.8870029	5.6468977
BYSL	3.50050894	2.77884444	4.49537243	4.72082541	3.25036676	2.77702285	3.2630444	2.92657203	3.99512304	3.52033886	2.433671	3.60238699	2.94775172
FAM120B	5.82757132	6.15874439	5.54664045	5.49089921	5.6199204	6.25463512	5.64796009	5.70476662	5.71183131	6.09636834	6.11897126	5.78366395	6.01030632
TBP	4.4673874	4.21129111	3.8608475	3.78027823	4.7930414	4.41754595	4.16590335	4.68067254	5.00590163	4.54553127	4.22560165	4.37902227	4.67789322
GUCA1B	3.40617157	2.34153575	2.8062527	1.65883387	2.61134839	2.48986478	2.75665666	2.70638096	2.95563287	2.92647792	4.03923423	2.57868745	2.88305085
PRPH2	1.9538183	2.56028693	0.75086807	1.66784886	1.49828404	1.03144491	0.89444266	2.1907402	2.7194396	-0.63202799	-0.63202799	2.18112036	1.12722352
PPP2R5D	6.0249468	5.51176174	5.89584042	5.40171975	5.25652827	5.41173876	5.47905551	5.43169842	5.32890137	5.13256395	5.38867592	5.87528883	5.72890314
MRPL2	5.11747131	4.47943461	4.58979379	3.96765193	4.2676671	4.56583021	3.87548498	3.72997376	4.28290705	4.55055308	2.95673313	4.28379737	4.41332821
PTK7	4.08445913	4.18117471	3.63034034	5.35394342	2.57601142	2.18925036	2.80244215	3.81251967	1.82608264	3.20631202	3.51699077	3.3300905	3.46910946
SRF	6.29283498	6.08023111	6.21288823	5.07483077	6.66675896	6.47999461	5.7756529	6.0684868	5.76208634	6.70720425	5.93867281	6.45580753	6.17817411
CUL9	5.52071324	5.13618565	5.77068053	4.89946599	5.17446013	4.65732225	5.3947661	5.18668076	5.04163396	5.08590546	5.19924388	5.58451319	5.37042704
DUSP22	5.79844776	5.67304552	5.29927429	4.88407907	5.5710845	5.11305854	5.64661846	5.69311084	5.36080523	5.02647357	5.16095643	5.77811197	5.56577243
EXOC2	5.72357832	6.04602125	5.1529881	5.62643338	5.71261751	5.63281943	5.46081785	5.2136668	5.5010941	5.67577856	5.51925279	5.78037941	6.11486607
COX7A2	4.77497275	5.54928422	4.89410128	6.3287202	5.58103003	5.71672757	5.62212181	5.5342341	5.60268412	6.15679819	4.87681642	5.55928465	5.64958198
TMEM30A	6.31489892	7.38315956	6.61168774	7.27318815	7.97810406	7.86886853	8.21037473	7.70047668	7.80149111	6.60181179	8.05346259	8.11280589	6.24956488
GMDS	5.79711984	5.36519348	5.92236884	5.0369717	5.39684087	6.16927177	5.10747909	5.73428193	5.4744557	5.50302433	5.31007639	5.20088671	5.77056057
SENP6	2.62270997	3.81195189	3.01435485	3.86367197	4.30666889	4.64293377	4.2546001	4.4970045	3.88546896	5.16188496	3.27596196	4.37989653	3.9990887
VEGFA	8.93311104	4.77027518	6.96645982	8.14268912	6.34457636	4.17544605	3.76418214	3.26571334	3.67067073	3.46944782	3.31039812	3.18601417	4.47569809
PRPF4B	5.58930844	6.05751139	5.7068836	6.73457842	6.40569324	6.83660375	6.60665126	6.44573265	6.68081062	5.751403	5.96732182	6.08298869	6.18746467
TTK	1.25759919	1.28962669	2.64010291	5.1643899	1.16804269	1.16804269	2.05379839	1.72547618	1.23858965	-1.16579811	2.97308636	3.00633843	-1.16579811
SLC29A1	6.99533117	6.49431084	7.16297719	6.10138811	7.23098336	6.34871235	6.78012098	5.88227932	6.6735906	7.0349078	7.24028364	6.32815087	6.09104757
BTN2A1	6.26676468	5.78568665	5.95658829	5.55680698	6.2244558	6.03909403	5.93550769	6.00669536	5.74019447	6.09738885	5.6639735	5.65121033	6.23589899
LAMA4	5.91094157	7.26987961	6.24746207	7.7004134	5.87299929	5.10096114	6.15440262	6.08196786	6.45384714	6.0921772	6.61746841	6.61786933	6.79173051
FAM46A	7.48547671	6.65009756	7.33483078	7.94911556	5.99640283	6.8646033	7.12119527	6.70728401	6.58025323	6.12179274	6.56023371	6.42987095	6.40419743
CLIC5	2.17083228	6.70317155	1.61983646	2.87696715	3.8912029	2.6780632	2.92735709	2.43665325	4.59653575	4.24794052	6.13871998	4.02696805	7.11989799

FBRSL1	3.91403014	3.04545525	4.66372391	2.13506558	3.67094228	2.60495983	3.28600926	3.62441722	1.40856395	4.45631368	4.56602274	4.33609119	2.37850383
ENPP5	4.33949693	2.76435984	3.244009	3.67045615	4.07819995	5.7582574	3.82826813	3.87112454	4.03705073	2.05455718	3.64853225	3.20998305	4.20304462
LY86	4.13897845	3.31202112	3.1392927	4.40281885	2.57499144	1.9059854	2.24448304	1.10320234	1.8375863	2.15892414	1.18107639	1.803109	3.12493355
PRSS16	1.38188732	1.69317985	0.96378585	0.74192035	0.8714203	2.05570217	1.90629789	1.49004822	0.84091163	2.11773247	-0.6821331	-0.6821331	-0.6821331
TBX18	2.90513134	1.97389157	1.9530707	1.05821698	3.0658507	4.03523367	3.19185244	3.7488024	3.64805221	-0.17898321	-0.17898321	-0.17898321	3.01109934
PCDHB2	4.27382333	3.1310714	4.17726848	2.97877992	3.17160048	3.71977256	3.67953827	4.14711686	1.5780233	4.61189572	3.71411791	2.18216786	2.98814327
HARS2	5.01421292	4.86243669	4.9720481	4.57651361	4.6462877	4.80091437	4.41762376	4.39712123	5.00935364	4.8975606	4.9128963	4.43061231	5.04004358
NUDT12	3.49929802	4.67377803	4.01773931	4.65527989	4.48347308	5.10869111	4.82469765	4.94943642	4.81389927	4.71000025	4.8770163	4.9003795	4.84976751
CEP72	3.61954291	2.48762836	4.61158025	2.78287153	2.85133217	2.87834768	3.10651002	2.83837697	2.04780102	3.80949501	3.48308916	3.19420675	3.99560316
MAN2A1	5.58234011	6.21059871	5.7227043	7.18986541	7.17937197	6.99341218	7.09027062	7.23764396	6.43676277	6.66764096	6.87406449	7.02305704	5.76836039
SEMA5A	5.23966678	6.20555209	5.82348506	6.92221356	5.98942613	5.26088673	5.72134101	6.10259612	6.28875177	5.50522463	6.3201566	5.87991851	6.49014182
C7	7.48684408	9.85020779	3.7238324	5.9739627	6.06665594	5.22326296	5.4004906	6.1565538	7.34781045	7.05940526	8.59627729	7.04935329	9.06773808
PAPD7	6.04262353	5.51014045	5.48280271	5.47357856	5.45338042	5.42192618	5.73423491	6.15029713	5.47482219	5.38715889	5.71669619	5.7967	5.89135934
GHR	2.73043759	3.09599815	1.75472151	2.15257642	2.20729697	3.97178042	3.03384332	2.92517876	4.09106952	2.99596372	2.4352922	1.08168381	2.18409481
HMGCS1	4.50195251	5.48262861	5.06726924	6.25133007	5.82014666	5.81547016	6.43077401	6.17414019	6.12137368	5.26275644	6.47779141	5.63352244	5.56896927
DAP	6.33796423	6.89501004	6.26063689	6.70596292	6.64548228	5.8005293	6.31688235	6.77209407	6.97166881	7.02376188	6.88115608	6.55986824	6.55376904
NME5	1.58829694	0.45466636	0.68003788	2.47308338	1.01930202	1.38238406	1.97296897	2.80009466	3.05327979	-0.71713284	2.72960117	-0.71713284	-0.71713284
BRD8	6.0319822	5.6379521	5.96639486	5.68046089	5.24386656	5.46889905	5.95591046	5.4335065	5.49165534	6.10674772	5.37652459	5.48094495	6.00378219
KIF20A	2.16884863	1.27873728	4.61022716	5.15789525	0.26142262	0.42052956	1.68389094	0.9630588	2.58258165	3.5732585	2.11102962	-0.93956238	3.565353
NNT	5.24884705	5.58098235	5.28343621	6.25132158	5.36319043	5.958921	5.92150538	5.83912175	5.30833151	5.4289585	5.44563474	5.48074608	7.20089057
MRPS30	4.46536977	4.48642668	4.65587324	4.73752649	4.96154901	5.42733366	5.16836423	4.42400409	5.10592678	4.67686023	4.93268101	4.47391484	4.69606501
HSPA9	6.5176108	7.04727867	6.45138374	7.39831192	6.8964053	7.36255616	6.86949831	6.45806662	7.00032119	6.72245416	6.46471323	6.65290356	7.53916008
MRPS27	6.0539428	6.04798981	6.12418537	5.972413	5.6557349	5.70049547	5.72322368	5.45620468	5.82434066	6.04666685	6.09238227	5.84311808	5.83938078
PFDN1	5.45534549	5.77125044	5.45573911	5.43363202	5.21320149	5.45861588	4.8131887	4.857962	5.62844312	5.71514682	5.1231395	5.78186196	5.34705581
HBEGF	4.43374604	4.30443622	4.38264008	6.67041393	3.91987735	4.42780213	2.97205498	3.59104177	4.45640053	2.85891949	3.88411634	4.66569197	6.19863373
SLC4A9	0.25098857	0.41811625	0.23300234	0.23041601	0.67415494	0.23185397	0.48076836	0.5854554	-0.19971777	-0.19971777	3.36637228	3.39755785	-0.19971777
LOX	6.57136471	6.25113254	6.13541913	8.31717221	6.86495364	6.95413385	6.09606832	7.56519212	6.60641077	6.12983097	6.75908545	7.03432202	6.22795068
GZMK	3.55834498	2.74329121	0.04198886	1.54055984	2.79401913	0.90656481	1.20266071	1.29448074	3.68622901	-0.35846007	4.18496845	0.69453008	0.70756131
APBB3	4.71863993	3.37064923	4.35157846	4.15898738	3.60657581	3.25430717	3.67969063	3.88654379	2.86574913	3.69087315	3.69854576	4.45181458	3.85619992
TMCO6	4.17201483	4.0435042	3.89554645	2.91032825	2.4940223	2.46600577	3.27075708	3.21916906	3.31345308	3.84456472	3.7985027	3.47981145	-1.03385651
SPARC	9.20350844	9.572219	10.1554534	10.5527981	10.0993182	10.617413	10.0487691	11.1369274	10.2630213	10.8138573	10.4176252	10.9191844	9.97867982
IK	5.32620162	4.69228849	5.01543936	4.57190857	4.49959649	5.04345249	4.27463631	4.13261856	0	0	0	0	0
HMGCR	4.36993179	4.63414092	4.82527316	5.61491839	4.67426892	5.50404946	5.40240066	5.01380724	4.4464112	4.37814262	5.48601157	4.82493859	4.82101757
COL4A3BP	4.8130319	5.02646753	4.82569125	5.55355919	5.32941117	5.37480652	6.11911529	5.7416675	5.86456694	4.92748883	5.36367405	5.63056947	4.15469967
FAF2	5.87478659	5.86270555	5.48323708	6.07077085	5.63888875	5.68101459	5.35778158	5.44734727	5.77847805	6.15287988	5.43572596	5.46059642	5.71226843
HAND1	1.6232791	2.50074772	1.09111032	0.63066762	0.59760407	0.92492185	0.59760407	1.63397295	-0.63677721	-0.63677721	3.7407118	3.83410158	2.66283428
PCDHB5	4.3882493	3.4322482	4.2756925	3.00272508	3.67152989	4.95128529	3.77029764	3.9940944	4.18329374	4.90868398	4.025198	2.37466764	4.37500024
PCDHB6	4.42146794	3.30208914	3.61594638	3.07655266	3.61908432	4.21124093	2.87410347	3.57344136	3.01810304	4.21764017	2.98197023	4.0309411	3.8033228
PCDHB7	5.00924355	3.36620461	3.84402972	2.53982668	3.50496443	4.01829959	3.58662937	3.9654966	3.39636928	4.26216764	4.05870965	3.97598298	4.18701812
PDE8B	1.39455161	0.7679709	0.36996935	-1.10981197	3.12251117	3.88095342	3.05533592	3.91445104	3.77078258	4.76446254	0.03105775	3.1830868	0.03105775
CLK4	4.57580587	5.42026593	4.80595805	5.50494881	5.88387827	5.6764366	6.03540582	6.16825722	5.78247233	5.4920405	5.58717607	5.81613616	5.51443441
PCDHB15	5.58947524	3.93529118	4.91329973	3.75691498	4.46646545	4.93731072	4.58965792	5.5579411	4.42985979	5.3222104	4.29924964	5.55234684	4.38846645
HAVCR1	1.16263289	1.23214512	1.25009201	1.77638906	1.71402878	1.04870824	1.64602008	1.02085625	1.43361823	-0.68385909	0.66480526	3.25924825	-0.68385909
ITK	3.10201422	2.76109936	1.25146399	2.68630322	2.07867359	1.60986778	1.66544511	2.16995882	2.68050937	-1.02004111	4.47562967	1.43458243	4.98324898
RNF130	6.37596231	5.76254107	5.63068116	5.69371982	5.54094866	6.06301794	5.44367677	5.30131627	5.91975533	5.84561069	4.94908981	5.73424365	6.20378558
THG1L	4.8405139	4.12784732	4.73756549	4.46025042	4.01355371	3.62485321	3.77920656	3.8096384	4.68890938	3.77696581	3.78687173	3.83143025	4.04335456
ARSB	5.55325104	5.22226284	6.01545653	6.11296522	5.46154218	4.98877224	4.99758094	5.44791784	5.31608185	5.09942727	5.16454286	5.16363823	5.59099612

CLINT1	6.08113209	6.28978705	5.86547867	6.79838623	6.10669196	6.14657954	6.03957942	6.25823722	6.33111692	6.121575	5.78897341	5.9002364	6.11470562
THBS4	1.31114139	6.65911419	1.97640055	3.23274222	2.77842382	2.23910595	1.8504312	1.94475105	3.87472755	5.37842481	3.85920941	3.92181618	6.80955713
CNOT6	2.38038446	2.96604078	1.79888452	3.69460768	3.70657094	3.75015822	3.69906311	3.91278152	4.27578617	-0.83463886	3.55867262	2.90588708	2.48397174
BTNL8	0.21096316	1.06815627	0.18092078	0.20076465	0.17840162	0.17980224	0.17840162	0.18161547	-0.04779344	-0.04779344	-0.04779344	-0.04779344	-0.04779344
TTC1	5.48420565	5.6853631	6.16521332	5.72285626	5.60180117	6.05086441	5.62247163	5.21853201	5.77984282	5.6866917	5.21028267	5.28194366	5.69899282
MSH3	4.87742925	5.1293354	4.61446617	5.49134244	5.27804951	5.27339338	5.47388643	5.30942577	5.14631743	4.72212107	5.41746819	4.76344746	5.76839526
RASGRF2	4.43804678	4.08126254	3.66009475	4.2897011	5.2014959	5.28724995	5.49893004	5.39225015	4.62649281	4.22725638	4.15956152	4.78958227	4.64675768
CCNG1	4.97357061	6.73449847	5.86256412	7.02055445	7.0279681	6.90642404	7.39817866	7.10100558	7.40609071	5.43315138	7.03757372	7.14462175	5.24829295
POLR3G	1.85024242	1.69755831	2.00545515	3.13834884	2.38323614	2.19535875	2.50095763	2.03857909	2.74990724	2.53514219	3.02663234	2.52414746	2.39304299
DROSHA	6.18369283	5.76395459	5.8440024	5.98480833	6.14765339	6.32576718	6.11932496	5.71594427	6.06641457	6.07373072	5.73437715	5.89906421	6.77958206
CDH6	4.72588963	3.36630663	3.9687243	6.41097031	4.06443938	4.9386965	4.06319041	4.01381285	3.80853779	3.15798997	4.14013303	3.37194515	4.77302479
LMNB1	3.71161034	3.24287933	5.62635569	5.63340697	2.66740385	2.5738121	3.69327553	2.40791318	3.05114449	2.02640104	2.15480597	2.90996635	3.16863719
ARRDC3	7.0734527	6.76045014	6.67396373	8.57954967	5.86401508	6.71473466	5.4511472	5.52455729	6.08382177	6.07844073	5.96791477	5.50935608	5.45091639
GOLPH3	6.38561867	6.48170573	5.73783861	6.60611776	6.62830563	6.73088323	7.00387508	6.90762926	6.25130689	6.19985255	6.18361233	6.35328395	5.95649806
SUB1	5.50621295	6.42536599	6.05068948	7.18902783	6.97622156	7.11677938	7.05265878	6.8956358	7.0819999	6.32159076	6.96881819	7.38141794	6.05975144
NPR3	5.11717737	6.18474306	5.54852083	5.09514151	6.9548078	7.54026137	7.19595656	6.51255407	7.2684625	7.16324075	6.86466638	7.71035022	6.70981511
FAM172A	4.56017951	4.7227319	4.25400421	4.90621519	4.97156441	5.86731437	5.42496704	5.15466207	5.36325027	4.36674099	5.17087185	5.31860286	3.2641862
TARS	5.77368095	6.08294339	5.7728127	6.83876233	5.98399731	6.76886885	6.27916026	5.95952233	6.154003	6.21463493	6.04413397	5.65230246	5.85178046
LNPEP	2.02906149	2.68437147	1.88846562	2.87284076	3.95320659	4.59268768	4.25729026	4.44707973	3.50099153	4.75929245	4.00801878	4.56504653	4.26207301
PDE4D	4.94958421	5.24589595	4.47661344	5.11910698	6.1475012	6.98237379	5.64593327	5.79223743	5.37339635	4.85662533	4.9641299	5.47674259	4.37485801
RAD1	4.87316821	4.99271725	4.72125006	4.7959024	5.10992801	5.38012756	5.38739366	5.41996665	5.17417431	4.83257142	4.97616663	5.54294146	4.62434539
BRIX1	3.76610069	4.42116654	4.31333103	5.33739447	5.35496038	5.77358298	5.10813396	4.37743969	5.58104871	4.81124146	4.92649768	4.83479939	3.52595875
PRLR	2.16003508	2.15790153	1.98210514	2.54988838	1.96818793	1.806421	2.12742148	2.11450512	0.85459486	-1.028193	1.34737153	-1.028193	2.58699506
SLC12A7	4.79620855	4.62953259	4.85869294	4.31326744	4.10932972	3.30245237	4.32296688	3.77492949	3.52318494	3.65577124	3.66669551	3.69205342	4.28308017
RAD50	5.74122868	4.9265891	5.63405886	5.03118483	5.43717657	5.80873709	5.4431728	5.80712426	6.23707102	5.32192801	4.98757914	5.58248962	5.27429828
ST8SIA4	4.04047045	3.6816053	3.9323517	5.68239644	3.37818352	2.93198519	3.19842127	2.97399035	4.38246719	1.88286091	4.03912368	3.42058319	3.29573025
GNPDA1	5.37578706	5.29945176	5.42140284	5.1534818	4.4708611	4.59188754	5.26924444	4.35281774	5.12198924	5.46189421	4.72450668	4.86623264	5.09396798
PCDH12	3.0635429	4.87088789	3.65478422	5.17219846	3.47666283	2.47002267	2.90512145	1.8178776	4.33762332	3.82252546	5.28610764	3.61211492	4.03701335
SKP1	5.96077549	6.74586158	6.07918272	6.64855218	7.02127657	7.17254957	6.88732931	6.95444423	7.11834826	7.26061757	6.7215076	7.08040418	6.94592381
NUP155	4.50925583	5.0335226	4.96916354	5.634147	5.38186192	5.3117363	4.79702358	5.23687828	4.9800485	4.07134635	5.46778447	4.99814343	5.23288145
PPP2CA	5.57295233	6.18794986	5.50750837	6.8349932	6.40744929	6.28295716	6.40744929	6.29574183	5.9657406	6.32085629	5.75162405	6.69265722	6.12692483
FGF1	5.72963102	7.17388126	6.08495534	4.93623835	7.49876627	5.76290005	5.49116333	6.12820359	6.49940381	4.96160762	7.62974218	5.56601618	6.48926282
NR3C1	6.42410626	7.46563306	6.17839065	6.98037073	7.26313318	7.23868087	7.2806135	6.93993801	7.32434892	6.6600638	7.3737361	7.15204569	6.70923999
C5orf15	5.06041072	4.92662709	4.44578382	5.76677482	5.46077807	5.72888351	5.17845937	5.4572057	5.48639685	5.08865595	5.58080369	5.28369985	4.66230727
PPWD1	4.62903984	5.32710003	4.70842969	5.91566939	5.60761767	5.83613304	6.03541311	6.0583317	5.82213867	4.96826487	5.85486477	5.2068473	5.56813016
LIFR	5.8088365	6.9259351	5.50960973	6.17766662	6.93571711	7.11095437	6.95299017	6.82266486	6.62852845	5.96836569	6.81544112	6.18826255	6.67906261
TRIM23	4.83561164	5.57769769	4.64730692	5.02070956	5.20684144	5.68539157	5.66405834	5.39796618	5.70348772	4.736671	5.31578331	5.60683768	5.39931861
C9	2.5994107	1.89027805	1.27987314	1.5196738	0.90400272	0.70410398	0.96999819	0.40918191	2.6032785	-0.5270559	2.63136772	1.86001903	-0.5270559
SEC24A	5.85115782	5.74083713	5.5140169	7.13105045	6.47358204	6.27457727	6.30589629	6.46785854	6.42036889	5.34724343	6.72473293	6.12173046	5.55088441
TXNDC15	6.02710814	6.13542334	6.36067908	6.21372047	6.50062274	6.9533578	6.50580935	6.70361913	6.78271139	6.43501388	6.5303534	6.63626965	6.18486183
TTC33	5.26880541	5.25138514	5.15677136	5.07485101	5.27409931	5.95782909	5.6575644	5.65792501	5.58974219	5.24587357	5.18908207	5.58396683	5.4509585
RARS	5.21809877	5.76815811	5.50213552	6.8188949	6.20056892	6.15290178	6.17040877	6.21645887	6.44138626	5.58550028	6.03815335	5.69814654	5.88074038
WWC1	0.15632902	3.70648257	0.26942636	1.32626313	0.15085915	0.15179207	0.15085915	0.38446237	-0.34869578	-0.34869578	-0.34869578	-0.34869578	1.473699
H2AFY	5.99729035	6.11205302	6.04070159	6.58031293	5.77832528	5.34582453	5.6476855	5.57693823	5.31250583	5.70830614	5.70491172	5.4760612	6.29044895
TCERG1	5.55289533	5.70210118	5.5792633	6.66666993	6.19086525	6.14418932	5.59391506	5.85651476	6.17649964	5.66877538	5.80976741	5.35208127	5.65646406
DPYSL3	8.91465603	9.00224342	8.52866909	8.71769525	8.86280596	8.17922223	8.36968062	8.72482456	8.25093484	8.53508454	8.6616412	8.58951036	8.59971641
SMAD5	6.20475155	6.57144918	5.96538041	6.87167009	6.77968509	7.01226034	6.73179572	6.74552698	6.84854608	6.26785316	6.84085228	6.99509506	6.04552803

CSNK1A1	6.50148409	6.90746521	6.66755418	7.01133858	6.85683951	6.95751234	6.538959	6.98727015	6.57375645	6.50856794	6.72423697	6.871174	6.58347398
HMGXB3	4.66686626	4.38114929	4.76594259	4.96326529	4.44516765	3.72217924	4.2212495	4.57961342	4.03890795	3.68911959	5.21707492	4.42403664	4.23266303
ERGIC1	6.89864455	6.72781464	6.65159129	6.09573125	6.91727885	6.43363111	7.02508257	7.18516423	6.53997666	7.06585056	7.22101474	6.77103918	6.74983455
PDGFRB	8.02318557	7.50493775	8.08187338	7.70193409	7.21698302	6.92725997	7.98628931	7.55842626	7.23590369	7.51254262	7.92872085	7.43696479	7.16055967
CDX1	-0.14794804	-0.0246831	0.42153818	0.19290359	0.04696449	0.31525922	0.27685043	0.11692464	-0.01018473	-0.01018473	-0.01018473	-0.01018473	-0.01018473
ATP6V0E1	6.02694406	5.94469404	5.93200703	6.6077601	6.01223349	6.48728454	5.75655998	5.49371468	6.22061034	6.44474516	5.69544291	5.96233135	6.0539858
BNIP1	3.73742672	3.64377358	3.63329401	4.51064626	3.42873565	3.34673018	3.41351815	3.45435578	3.67166481	3.47181238	3.2673974	3.8326449	3.97421055
STC2	2.88262182	3.96803827	3.37774378	4.34854385	3.45254446	2.98056018	2.45770099	2.49726486	2.33285856	2.30223877	4.02137107	1.64879296	4.62783732
CPEB4	6.57003201	6.27310557	5.57816487	6.11172012	6.57124385	6.85639777	6.4119002	6.20996738	6.30007889	5.81914352	6.07568351	6.06890555	6.53341032
HRH2	5.93902005	4.13686847	5.53455128	4.81418218	2.7013431	2.90724275	2.54275087	1.84515585	2.38858022	2.85989701	3.03921898	-1.44040637	3.60400161
DBN1	6.43619442	5.62878557	7.23492652	6.06458221	5.9021865	6.01010292	6.09913248	6.25324123	6.22472249	7.16947722	6.09194394	6.20540863	5.84206122
ZNF346	5.09783219	3.82210311	4.281031	4.27136785	4.59456727	4.01640799	4.11647899	4.48509766	3.9125101	4.2217739	4.7732987	3.39975638	4.88670008
UNC5A	0.23661454	0.98189061	0.83141036	0.49569413	0.34239324	0.10694052	0.23342501	0.10876745	2.73453157	-0.0731504	2.91594839	-0.0731504	3.16688935
EHHADH	4.28813694	3.89844808	3.88115627	3.86633301	3.78040511	5.12392572	4.03308607	3.95479238	4.51812985	3.98572061	4.30793621	3.82657801	4.4117139
CNTN3	5.47762383	4.45679104	5.30270026	4.48917828	6.05514839	6.41648967	6.54672439	6.50241729	6.20876496	6.7724525	6.18039215	6.68173512	5.85226909
SMC4	3.28087346	4.01322125	4.42785162	5.74742157	4.17282484	4.8163837	4.49152435	4.36718993	4.91675609	4.29770903	3.9789411	4.13428724	2.90986636
ACTR8	5.3194699	4.92249818	5.34932045	5.70470702	4.99138239	5.5385014	5.30411286	4.99877634	5.52693316	5.14152116	4.54953399	5.15574673	5.47133166
TBCCD1	4.42625851	4.41778428	3.62142148	4.91228235	4.40609207	4.09442464	4.57671592	4.59698374	3.19909117	3.55069789	4.32176093	4.35188055	4.93443989
TIMMDC1	6.11896493	5.86715355	5.86011241	6.0896372	5.4855104	6.33010049	5.85879953	5.32624761	5.77570952	6.06931331	5.7885318	5.43355082	6.46416326
CRBN	5.18118291	5.69830083	4.78539744	5.68599688	5.8787474	6.0612892	6.16523982	5.98064318	6.22503239	6.00039367	5.7447802	5.92066881	5.18827797
BCL6	8.30056589	5.53371632	7.10358167	6.37581507	6.15387689	5.93681394	6.25362036	5.64421974	5.05221913	5.62647841	5.11659455	5.35705697	6.00726199
HGD	0.64733953	0.29015669	0.45151454	0.7882414	0.53679864	0.55067442	0.66204855	0.79437684	-0.22179147	-0.22179147	-0.22179147	-0.22179147	1.85546047
CLDN16	0.12056658	0.33872098	0.12316106	0.37599063	0.12056658	0.12200907	0.12056658	0.12387653	-0.19513875	-0.19513875	2.97155179	-0.19513875	-0.19513875
ARL6	3.18363016	3.8598212	3.07562207	4.25511512	4.55359324	5.02292244	4.60155232	4.59736487	4.96656061	3.16946725	4.95954438	4.62844958	2.71111862
NPHP3	5.71071774	5.60252727	5.69228354	5.16542972	5.53564426	5.39322708	6.32372711	6.10089539	5.44315714	4.86218221	5.64577026	5.84345893	5.76086507
CD86	4.05263645	5.26314898	4.38828944	6.96227335	3.87767179	3.56543714	3.69725561	3.48491315	4.94754021	3.83834779	3.06722103	2.79578783	4.67163939
AMOTL2	5.05909073	7.30857344	4.56265869	4.99405622	6.6001157	6.72279054	5.76370235	6.56779684	6.76627707	6.34525348	6.81445366	6.92598785	6.80784812
NIT2	3.78572717	4.62098391	3.98169597	4.02671884	4.54024909	4.20592514	3.8608601	4.72362497	4.44544601	3.74776236	4.73588193	4.30862852	3.84455201
FAM162A	4.72400204	5.06770877	4.47364321	4.89539366	4.72487393	5.57438449	3.91098024	5.1108261	4.63352526	5.36988622	3.66349465	4.96816708	5.02250167
OGG1	4.79604902	4.26447234	4.71670018	3.8739543	4.78111529	3.52547627	4.22881604	4.75706961	4.53263343	4.96208323	5.30537443	4.4850929	3.87295508
KPNA1	5.86323322	6.44266531	5.89324268	6.55545994	6.71469233	6.71951627	6.61149306	6.63492783	5.96482334	6.12022388	6.53873419	6.40426748	6.14678864
PCCB	4.48870083	4.38349195	5.34630866	4.58002376	4.13116459	5.18778664	4.6172505	4.59153674	4.87741536	5.11187848	4.89852438	5.37657604	5.31162497
UBE3A	6.21478618	6.65185804	6.37613685	7.02130831	7.10761977	7.08589836	7.22897497	7.27738132	6.81617703	6.73983222	6.58864554	6.90803016	6.59807594
ARMC8	5.63907127	6.1271526	5.26509238	6.09563503	6.30303	6.41178042	6.32716977	6.43422943	5.52092544	5.59283911	6.01066058	5.66361563	5.91049997
CEP70	4.76493701	5.50953562	4.65512808	5.69314817	5.90755469	6.22412151	5.87091947	5.93770929	6.07011377	5.908511	4.80127993	5.70614223	5.50164824
RBP1	4.77693919	4.03199625	4.71806996	3.69207191	2.90657769	4.17068579	3.6340989	3.7845572	3.94795391	3.50728499	4.26702831	4.46081948	3.72096429
SLC25A36	5.24925761	6.19877303	5.3337121	6.53296549	6.33900478	6.53728321	6.36712879	6.5636798	6.46570102	6.07528305	6.5641613	6.44140623	5.21231461
GRK7	0.18647266	-0.07871415	0.089118	0.10542121	-0.05362728	0.1029767	-0.07871415	0.02022761	0.00299245	0.00299245	0.00299245	0.00299245	0.00299245
RNF7	4.98391937	5.57914207	4.92722501	5.00013923	5.76317739	4.75180581	5.35358797	5.41549614	5.54478298	6.0258119	5.48599752	5.49466294	5.4679407
TFDP2	6.4771366	6.51651323	6.06319962	5.44524243	6.36055476	6.4254096	6.44069363	6.37821261	6.37197127	6.4855188	6.64934708	6.61317442	6.15549686
XRN1	6.18718927	6.77213027	6.00154671	7.09775948	7.1733955	7.05650187	7.03710876	7.20829422	7.203014	6.36326627	6.92418321	6.96198242	6.69574414
KAT2B	5.39108578	5.80625236	4.84405068	5.1741192	6.05667488	6.23906011	6.13444325	6.25225145	6.58824231	5.98537622	6.06670305	6.03845718	6.10928803
BCHE	0.18053875	5.51162539	0.18258272	2.89498455	1.16296592	0.4595281	1.03761829	1.80135894	3.6209727	3.97149844	4.96653453	4.09391126	3.67873435
SERPINI2	-0.08791476	0.4524271	-0.08555507	-0.08791476	-0.02479557	0.21076394	-0.08791476	0.06197078	0.00051552	0.00051552	0.00051552	0.00051552	0.00051552
PDCD10	3.36191407	4.56346382	4.0215025	5.11658626	5.18411201	5.39657578	4.646567	5.36126261	5.82105934	4.34452782	4.93531773	4.73063943	4.16655097
WNT5A	1.64323603	2.62232578	1.73079243	3.31161999	3.06546794	3.88300751	1.99358905	2.17889592	3.29484782	4.64253006	3.14895062	1.92141282	3.60948052
PFKFB4	5.20383454	2.87859409	4.2002246	4.54759504	3.09042609	3.03020763	3.74927941	3.03348322	2.24208282	3.17053462	2.9435274	3.82964064	3.30904046

COL7A1	3.04251483	0.86841936	3.46341868	3.37615575	2.27079848	2.81561482	3.39165182	3.53142356	1.90140829	2.62810199	3.31804935	0.8763544	3.00215135
FGF12	1.33931192	2.01852784	1.40348307	1.55513758	1.62020709	2.03039587	1.77839456	2.51140485	4.0455208	4.39360698	2.3998167	4.25149149	8.03256244
PRKAR2A	6.27754963	6.20239863	6.49576591	6.35889052	6.50111462	6.5211875	6.76919809	6.22848153	6.39942696	6.66632105	6.24532495	6.50918817	6.50969265
HES1	4.59560357	4.69717699	5.1419745	4.00520131	3.06399687	4.51855677	3.63444956	4.22294723	3.81610038	4.62003237	4.57983431	4.69217769	3.84910497
USP4	6.07144591	5.84148906	6.01543276	5.53169512	5.63677981	5.40576282	5.62948278	5.67586707	5.59219685	6.01923154	5.94973135	5.34073674	6.03060924
ACAP2	5.89546074	6.45316344	5.79078252	6.7706849	6.16157245	6.22294838	6.54347396	6.35381755	6.53100974	5.80918597	6.59736139	6.30103603	6.26097668
ECT2	2.87867507	3.54869299	4.15160946	5.75722609	3.59753593	3.67475265	4.04366001	3.4913189	4.63672663	2.98757917	4.33142764	3.09435206	3.26878342
GNAI2	2.41867391	2.04117764	3.25279044	2.2893746	1.42607212	1.08773561	1.38258402	1.55219457	1.00478511	1.00478511	1.00478511	1.00478511	1.00478511
TFG	6.17160113	5.82890084	5.77212154	7.0465294	6.19169019	6.09626887	5.89172977	6.21349749	6.03794745	5.79614286	6.14039792	5.97110367	5.87043349
USP9Y	2.41568621	3.56605094	2.00665962	4.25183416	4.24046116	4.49383018	4.11020257	4.61038489	-1.15437043	1.12350892	-0.28961548	-0.57057365	6.71607427
HYAL1	1.54381728	2.23871956	1.68265009	1.81330886	3.25562777	2.25429048	1.30380844	2.25122061	3.11598708	2.617503	3.64881138	2.80930465	2.55810493
TUSC2	5.03930229	4.63641372	4.76972958	3.33455674	4.34030864	4.6852633	4.74854196	4.14166781	4.61700935	5.55676195	4.9048508	5.47664648	4.36928007
NPRL2	4.89456372	4.26653224	4.07181489	3.69931002	3.67913842	4.49882334	4.20196469	3.93210571	4.26760301	4.82665752	4.43596407	4.55524803	3.78085924
RPL24	6.81681232	7.38958901	6.47364997	7.1186537	6.89622175	7.28913037	6.34715651	6.94824047	7.04294532	7.3406214	6.66790096	6.93589058	6.54581629
CYB561D2	2.69556202	0.79031741	2.65662217	1.29945424	2.94383191	2.38079076	1.78051433	2.56473669	0.04551492	3.8650683	3.83966617	-0.68218146	2.6815362
C3orf14	2.44307771	2.62984404	3.32456687	3.41921451	4.04880495	4.44684673	3.2636735	3.26698129	3.93972737	3.65663011	2.76816876	3.90172652	2.75484171
FXR1	6.01492514	6.0857477	6.16526659	6.59290334	6.51255106	6.92263539	6.58691335	6.55519707	6.74472482	6.64271925	6.26709358	6.27149555	6.65045443
CBLB	5.69562507	6.22277843	5.38994831	7.01036107	6.21008349	5.76367217	5.98129703	5.92671363	5.670101	4.9612399	5.79578257	5.19746685	5.8963036
BBX	4.19866034	4.62205279	3.94167236	4.2163988	4.73190427	5.51520892	4.66644086	4.63150758	5.01959767	4.83270224	4.25591789	4.53444995	4.82913755
IFT57	4.43076447	4.87164218	4.82207149	5.0543885	4.78875964	5.85916706	4.57475486	5.52372591	5.52262468	4.23327991	4.36972068	4.56859035	5.10424552
GNB4	6.00233503	6.86202337	6.31898391	7.59206775	7.3772999	7.3822976	7.12407341	7.44545946	7.45716104	6.8292868	7.84261755	7.87973674	6.61531691
IQCG	4.34494179	3.87882356	3.35871729	4.5551657	4.12451235	4.49227627	3.94809582	3.9170844	4.39592227	3.9109143	4.56896217	3.35981211	4.92304968
GBE1	5.41579283	5.69845534	5.29820243	6.36847517	6.60596441	6.59535495	6.59686065	6.48017546	5.31231009	5.5777995	5.26700226	5.57485827	6.15707558
UMPS	2.59802057	2.00294513	1.87500102	2.30550151	1.79124319	1.88363923	2.02589791	1.65946362	3.38924177	2.80564122	2.05853118	-0.63834474	3.29723292
NCBP2	6.11262138	6.22008732	5.87543248	6.80490326	5.81415674	6.30972861	6.29671808	6.0259394	6.12835451	5.93009296	5.85589084	6.15701207	5.8061666
SNX4	4.44479176	5.00913345	4.80314628	5.56755118	5.35094878	5.52030221	5.23693953	5.62156534	5.69568683	5.60698058	5.46571778	5.13039056	5.48236461
C3orf52	2.21201511	2.99334601	2.5296801	2.68961576	2.80863823	2.64668043	3.88401338	2.74802791	3.76630908	-0.80574749	-0.80574749	3.44940054	3.21273645
FRMD4B	6.51733593	6.98604849	5.69044282	6.57044966	6.11284721	6.09411113	5.57901646	5.83986114	6.78725047	5.54320627	6.84774501	5.97430424	6.7548388
SLC41A3	5.26614288	5.14348415	4.89159312	3.81519633	4.80352634	4.92160621	4.65347297	5.49872316	5.30346322	5.33509739	5.50337454	5.65303445	5.08723499
PLXNA1	5.97360241	6.01262288	6.8969496	5.66139177	5.83625143	6.02835469	6.18937464	6.05268477	6.12522182	6.9596149	6.97817408	6.91574525	5.90254252
ATP6V1A	5.20916248	5.66945399	5.43643068	6.39751726	5.57842693	5.6340795	6.12913715	5.28697758	5.4663469	5.08874952	6.04010949	5.53482193	5.70832634
ABTB1	5.14722914	4.88008104	5.13176382	3.40007514	4.46506403	4.04882748	4.8432877	4.9749658	4.87192638	5.4248603	4.53438847	5.32199317	4.36386256
PODXL2	3.79253819	2.43330118	3.04809884	2.46630222	2.00881116	3.92767413	2.57441527	1.83872579	2.6344612	4.5311711	3.49990457	3.89929963	2.52039758
UPK1B	-0.14826136	4.14858489	-0.14688784	1.10384146	-0.14826136	-0.1474977	0.01184455	-0.14650907	3.56813574	-0.10224547	3.69910498	-0.10224547	-0.10224547
CSPG5	0.95522787	1.23514526	1.25509523	0.9494122	2.30769567	1.56170281	1.13728735	1.53494388	1.96201013	-0.48606896	2.84337966	-0.48606896	2.3254972
KLHL18	5.03996301	5.05041845	5.46594539	4.99728529	4.91397018	4.51821643	4.74413655	4.19748664	4.68977881	5.65742717	4.30072021	4.94633803	4.82678727
SCAP	5.89961482	5.82732386	6.14096771	5.12898251	5.33274092	5.0664118	5.75288147	5.67194334	5.27924379	6.23844568	5.77669536	5.80140362	5.13033048
KIAA1257	0.57412676	0.61379738	0.56646039	0.49669393	0.9340103	0.48262307	0.55157509	0.86188829	-0.41538195	-0.41538195	-0.41538195	-0.41538195	2.77510076
NEK11	3.56395695	4.51389185	3.40981703	4.60006407	3.33142844	3.52721702	3.73580112	3.44141418	4.4276173	3.93136305	4.44315055	2.9671426	4.41059817
MRPL3	5.2498782	5.92298234	5.31195174	6.80377315	5.99188384	6.31070714	5.54217038	5.61260293	6.31213588	5.4169544	5.74715512	5.43440325	5.70918712
PLSCR4	6.48441107	7.45814604	5.84423869	6.64638867	7.72160065	7.60338489	7.61541032	7.92059249	7.91138409	6.76447317	7.95477929	7.92467614	7.17676524
HEMK1	5.18772705	4.43053684	5.13782739	4.03003447	4.32109569	3.74551006	4.54049543	4.00520559	4.35568662	4.76630809	4.81040957	5.12836498	4.66173001
CISH	4.61124096	3.79036335	3.9278734	3.40642377	2.86611036	2.72645073	3.57854862	2.53706016	2.9798602	2.11065426	3.25236944	2.0660875	3.32580253
MAPKAPK3	5.18191069	4.91485126	5.7795447	5.12033122	3.76380648	3.5637096	4.05834448	3.98554385	4.13456616	4.32676827	4.21117264	4.47874037	5.92329774
ACVR2B	5.5467952	4.95731938	4.97396594	4.0391782	4.92287284	4.74254525	4.82187291	5.00026929	4.57843016	4.43417323	4.88002635	4.24034264	4.94026099
WDR48	5.84435373	5.89819799	5.45208743	5.98792153	6.22602814	6.56983586	6.13578148	6.20292487	6.12680356	6.27855307	6.18189128	6.21929256	5.91552635
COMMD2	4.7008368	5.58594747	5.04038711	5.85910495	5.75372044	5.61796873	5.89018888	5.59240835	5.94256276	5.6395281	5.3056515	5.78697943	4.816811

GORASP1	5.61747103	5.57463115	5.4629308	4.84382792	4.95875053	4.95818861	5.08656438	4.61523854	5.02015496	5.35296564	5.59607259	5.31430169	5.26088699
PEX5L	0.47164731	1.47394962	0.47421101	0.63908543	0.47164731	0.5681173	0.47164731	0.474918	-0.29580994	-0.29580994	1.81021061	-0.29580994	3.22671693
RRP9	4.562451	3.73788877	5.41560246	4.57674808	2.91857163	3.42457844	3.559871	3.35793306	3.13419116	5.17137949	4.35711189	3.60616902	3.98135175
ABCC5	5.95770042	5.26583691	5.53628567	5.9052396	5.15263673	4.62589061	5.82092758	5.14649911	4.771839	5.13156295	5.71351093	5.35174011	6.27202599
AADAC	0	1.81954591	0.00124472	0.67740356	0	0.00069205	0	0.00158797	0	0	0	0	0
ABHD14B	5.70653254	5.0084371	5.07029691	4.67988122	4.29719705	4.89241532	3.87878189	4.26865967	4.54842206	5.45058608	4.5435735	4.85145833	3.28314268
EIF1B	4.53462758	5.72642411	5.32374614	5.80982347	6.30911094	6.45269586	5.87701221	5.86923663	5.13694822	5.35580746	5.05092384	5.7207206	6.07842343
ARHGEF26	5.969347	6.32776859	5.58542304	5.17497377	6.82670532	6.65443026	6.43359972	6.50142337	5.91601151	5.21041567	6.07273451	6.3254187	6.06552028
KLHL24	6.27616344	6.37224942	5.54399654	6.4192837	6.3597019	6.58939986	6.97287692	6.77362132	6.19744984	5.92205616	6.56650823	6.4617003	6.65475372
PLCH1	1.19875731	1.19589558	1.10310494	1.15980021	0.97223468	0.94383148	0.94238566	1.36101675	3.34017973	-0.58130397	-0.58130397	2.90854594	2.6109612
VIPR1	3.30712971	3.27244275	3.41063853	1.52928063	3.72395632	4.00386244	3.32477232	3.33006369	2.32429306	2.82791802	3.38201806	2.63373548	4.14822531
DNAH1	5.26374823	4.64546067	5.23744274	4.40655332	4.30155626	3.71131876	4.39104009	4.56624475	4.68486103	4.53958739	5.72013148	4.85439118	4.27894934
SSR3	6.29933514	6.97708354	6.56318466	7.9347127	7.08173185	7.06559384	7.05479639	6.96657639	7.03669739	6.85420589	7.51362851	7.10860556	7.09510729
ZBTB47	6.40771667	6.63646615	6.59875838	5.13644519	6.45335953	6.34909671	6.85379804	6.95730232	6.47813066	7.2971353	7.00828542	7.16369769	6.40744417
TNNC1	1.41815169	1.06122922	1.45706341	1.08835656	0.26050511	1.68637975	0.87321335	1.85299797	3.31800854	2.20851445	-0.44591264	1.74693707	8.46942991
NKTR	6.00956719	6.83439465	6.12053429	7.20839471	6.99563942	6.7323344	6.97439612	7.36741514	6.95630542	6.53008224	7.12249215	6.53036383	7.06811652
CLCN2	3.07147281	2.20627076	2.8190859	1.99679119	2.20332227	2.83732448	2.6194804	2.92390375	3.81738155	3.36824196	2.73221963	3.15254573	-0.85758734
FOXP1	7.44985457	6.9997732	7.03097585	7.33033049	7.58929986	7.46051421	7.62622815	7.72932144	7.34622863	7.55830052	6.92503233	7.25104988	7.47932115
EIF4G1	7.46798014	7.14987123	7.94398803	7.6531403	7.13128664	6.8701588	7.09612048	6.82545983	6.937989	7.32574192	7.11931531	6.98656539	7.4293577
SPCS1	5.03155958	4.55731712	4.24332369	4.8721468	4.36951082	5.09952483	4.38941846	4.52398816	4.72999005	5.34074344	4.92153331	4.42784587	4.22228207
NEK4	3.62285826	4.23087249	3.4797138	3.27524406	3.64363549	4.11181323	4.01918136	3.34554239	3.75535807	3.79356734	4.07885	4.32032946	3.06924259
SLC4A3	4.65622493	4.41081927	4.37525415	2.37478668	3.80156184	2.75886997	2.99653241	2.12093297	4.01385582	3.64831764	3.59098717	4.66725115	5.72957534
INO80D	6.0404602	5.91210249	5.51432924	6.02782566	6.29340551	6.29000584	6.14542934	6.44761483	6.00650468	5.37267594	6.53541366	6.33465646	5.80012573
EEF1B2	6.98161979	7.71505437	6.46881185	7.57596212	6.89170715	6.89921055	6.7363455	6.84651872	7.4266664	7.62748765	6.74574625	7.10382763	6.78476494
ADAM23	4.56252887	3.98188247	4.18142453	5.18543463	4.85367147	5.38125913	4.90333123	4.95188707	5.44744527	4.68794481	4.87200124	4.89620935	5.79290116
DGUOK	5.2463366	5.47719129	5.93990822	5.61522171	5.78910569	5.49026365	5.11627575	5.60479499	5.43484568	5.5497003	5.23681663	5.69394076	5.76240653
MOB1A	5.8861197	6.44943333	5.48586634	6.96614843	6.76437531	6.646514	6.6225545	6.51398317	6.60280006	4.99483942	6.75243945	6.67158971	5.68410716
KANSL3	6.38344596	6.67469387	5.9609096	5.88981472	6.25827242	6.14990771	6.13389375	6.45170661	6.26227137	6.48436088	5.96173683	6.48088788	6.44842904
LMAN2L	4.75289239	4.38618431	4.714868	4.1074619	4.14999204	4.93103006	4.54972593	3.96742335	3.94391552	3.75922969	4.45906085	4.98515367	4.5629983
RTKN	4.52712792	3.75514321	4.57566448	4.36662312	3.39013083	3.18233103	3.80677319	3.94841229	3.35332632	3.75653574	4.90018224	3.6810134	2.22688024
TTL	5.49630618	5.218554	5.7325314	5.89934687	5.70713049	5.0850144	5.6613409	5.50710305	5.08263295	5.19469162	5.45628632	5.15036186	5.71580927
IL1A	0.47131625	0.21624462	0.21880665	0.95696201	0.21624462	0.21766907	0.21624462	0.21951316	0.98792045	-0.18961839	-0.18961839	-0.18961839	0.8409784
CCL20	2.71676118	1.09539639	1.09794054	2.61884587	1.09539639	1.0968109	1.09539639	1.09864212	-1.08433789	-1.08433789	-1.08433789	-1.08433789	0.75112583
PIKFYVE	1.72206296	2.44300777	0.98795204	2.71463336	2.20966011	2.23013554	2.54880972	2.7320448	3.48607036	3.22758856	2.16272724	0.0344051	1.7722393
KCNIP3	1.6876416	-0.88089849	1.38803821	0.48489202	1.07816251	1.22417102	1.09147211	1.77425352	-0.02430295	-0.02430295	-0.02430295	-0.02430295	-0.02430295
FAHD2A	4.99241631	4.04414899	4.69654367	3.83050563	4.22144901	4.46040972	4.13511506	4.39209847	4.9106619	5.36595216	4.21412647	4.16605907	4.42060757
NCL	6.25159999	6.19288511	7.46169718	6.81946989	5.95394478	6.36639981	6.18369259	5.64286348	6.17934899	6.28031878	5.86302669	6.1858723	5.62099538
ACTR1B	5.45486468	5.71496745	5.3004657	4.57599607	5.55682402	4.73275265	5.70483752	5.8427047	5.27388455	5.80312193	5.73044407	6.23765588	5.69228013
SLC35F5	4.60810079	6.1798243	5.45319412	6.89015036	6.45327817	6.11282324	6.44932479	6.41372953	6.82784332	4.95690008	7.14403696	6.38351176	4.86268429
ZAP70	2.73204175	2.70245291	0.66426942	0.32182895	1.28015137	0.30040728	1.17165975	0.41098903	1.69515868	3.65316856	4.58136187	-0.46956012	2.08138327
ACTR3	6.25629757	6.87842886	6.53657252	7.73972252	7.5942464	7.58007746	7.24186703	7.3341941	7.29600398	6.84284664	7.28520116	7.11512277	6.53848803
STEAP3	6.15076666	5.41954384	5.50777499	3.82511462	4.08798505	4.07923145	4.02497122	2.74137441	4.3562539	4.52578024	4.61464806	4.29551351	2.13475439
EPB41L5	5.3195851	5.4860523	5.25437597	4.88076409	5.90556147	6.40710837	5.95984449	6.06102992	6.05573856	6.13549751	6.116486	6.23927888	5.22247027
TFCP2L1	3.32584158	2.31355761	2.30691965	2.14493487	2.14120196	2.56617378	2.13497421	2.50872228	3.2785551	-1.02763036	2.65685907	2.9199714	3.2239811
TP53I3	3.29030748	3.2076711	4.20577354	4.35537996	1.69076164	2.66341379	3.21206373	3.09232776	3.5636487	2.5271483	3.33809925	2.97971786	2.75171824
DNAJC27	4.41946454	3.86186771	3.76760635	3.91493273	4.51513653	5.11025074	4.16967859	4.38314117	3.81856397	3.55908064	3.3952484	4.51659709	4.18954458
POMC	0.14709542	-0.27134112	0.54690029	0.19374361	-0.17033608	1.3375939	-0.17197243	1.00939695	-0.10272155	-0.10272155	-0.10272155	-0.10272155	-0.10272155

STAM2	5.28010146	5.7989826	5.06156996	6.11128452	6.2556272	6.3143284	5.83855851	6.1769297	6.27697943	5.16563876	6.17807263	6.04302972	5.25175062
OTOF	0.12084738	0.11240326	0.26071091	0.11240326	0.12758306	0.30924633	0.11240326	0.30906098	-0.1090046	-0.1090046	-0.1090046	-0.1090046	-0.1090046
GPD2	4.773299	4.86277968	4.87110252	5.66085198	5.24348326	5.61996812	5.12765999	5.05459954	5.31350786	5.34464339	4.93286631	4.41655605	4.86253999
CENPA	0.15177717	0.60059283	1.41601537	1.96721199	-0.14248516	-0.47040917	1.31574614	0.53568365	-0.33734029	-0.33734029	-0.33734029	-0.33734029	-0.33734029
CYTIP	5.00175758	4.53143902	3.07549901	5.62535224	2.65683932	2.34760053	2.4123325	2.47223848	2.9265588	3.13834424	3.22519718	1.19953554	2.90055665
ACVR1	5.88100126	5.63583724	5.28648391	5.75071761	5.74737581	5.71759635	5.81970514	5.8801158	5.40919862	6.01834551	5.63333848	6.11652238	5.72977537
TANC1	4.71889508	4.32818624	3.97922967	4.18243625	4.61317023	4.41951654	4.4174218	4.77291346	4.69773929	4.40195834	4.95284444	3.54990173	4.99652474
SLC30A3	2.50879002	0.30886023	0.83920664	0.94188831	1.171233	1.44368123	1.49926056	0.46032937	0.79511055	-0.79711474	-0.79711474	-0.79711474	2.19310245
MPV17	5.96024626	5.40148258	5.56981624	5.77910254	5.34116193	5.14317195	5.54067869	4.88020164	5.45234905	5.67348383	4.50907783	4.85187097	5.84684697
GTF3C2	4.85164793	4.73749086	5.03636148	4.72522126	4.77416016	4.14289338	4.47468863	4.65666684	4.32802195	5.26412912	4.41097526	5.01596599	4.61607481
EIF2B4	5.90751071	4.673473	5.28939924	4.71439917	4.88740995	5.04898442	4.87551469	5.11662969	4.73097619	4.62273518	4.99128376	4.81039634	5.21103738
NRBP1	6.69981119	5.98041827	6.36807953	6.39326362	6.10168113	6.02371258	6.14220278	6.18931122	6.2793879	6.72744844	5.96938054	6.33807907	6.27475123
ITGB6	0.63194148	0.04437096	0.71360421	0.66387643	-0.0064627	0.07464963	0.45049299	0.20610481	0.01125288	0.01125288	0.01125288	0.01125288	0.01125288
FNDC4	5.1811854	4.38823505	4.68887749	4.74312285	4.21957466	3.01958317	3.43719926	3.86872569	3.59574266	3.29329085	3.96800604	3.99914111	4.36335053
ITGA4	4.38192175	5.84865393	4.80217516	6.82997672	4.37441318	3.75507866	4.95627113	3.987924	5.22808123	3.17379922	5.22401793	4.08395344	4.88437326
PSMD14	2.85057347	3.88869725	4.15540047	5.01395549	3.82322533	4.44484755	3.79257475	3.7278431	4.26233283	4.51939021	3.77029821	4.00868965	4.0816815
SNX17	5.9081349	6.1559886	6.44333887	5.87984936	5.76578295	5.2506682	6.18577817	5.94255134	5.80824506	6.43201957	5.72005407	6.48789337	5.41976797
ASB3	1.14705942	1.27218109	0.84408853	0.91363205	1.46404158	1.94862704	1.80517414	1.80390445	1.49318735	-0.38841776	-0.38841776	1.31637752	-0.38841776
PPM1G	5.88210509	5.65813648	6.46514616	5.632952	5.13089836	5.92733175	5.31727794	5.10860901	5.31013381	5.06182566	5.53672931	4.9475327	5.71968327
PDE1A	5.64513344	6.45737829	5.78169496	6.39908226	5.9064679	6.43180283	6.34892199	6.65586187	6.22551991	6.02309188	6.45566028	6.20557052	5.94693249
REEP6	1.53348331	0.66065027	1.06980639	0.71231193	0.50618233	0.9426671	0.25414785	0.58110669	0.15844874	-0.30027261	-0.30027261	-0.30027261	1.57010266
PCSK4	1.10093051	-0.08456946	0.87533614	-0.19078259	-0.05461594	0.4051178	0.80587841	0.72729492	-0.26065555	-0.26065555	-0.26065555	-0.26065555	-0.26065555
APC2	0.25395921	0.31291655	0.46739007	0.38886884	0.32987241	0.36523723	0.29401258	0.4130076	0.84528484	-0.14665498	1.56223506	-0.14665498	-0.14665498
IFIH1	4.84398125	5.39715171	4.60247805	5.41331328	5.42780137	5.23844367	4.4450232	4.85979424	5.58963791	4.22040796	5.38110482	4.55499236	5.1220399
RPS15	6.70792759	6.54889885	6.88808834	5.94994853	5.55805969	6.99959066	5.23761673	5.78366625	6.48485401	7.29878824	6.56472163	6.81852194	5.77420235
GCA	4.11426767	4.9221898	3.71811829	5.10100138	4.87356938	4.68304072	4.7481754	4.86397776	4.52170792	4.30402924	5.05127443	4.70938447	4.03164937
INO80B	1.6550414	1.15540039	2.18105006	0.75590577	0.88520681	0.91321366	1.75350754	0.48697073	1.06011542	2.27108034	1.91720064	2.24903941	-0.70172111
MOGS	4.91166137	4.67318979	4.81190114	4.69704005	4.13850495	3.57926561	4.53030068	4.62495845	4.36565173	4.28171267	4.96463383	4.82862556	3.90845333
TTC31	4.82034877	4.20476685	5.12827766	4.64271883	4.43270712	4.19001489	4.75048213	4.5953798	4.00638795	4.12140498	4.4556653	4.7022317	4.63806304
NDUFS7	4.36520918	3.74932634	4.6491987	2.86485715	3.46784033	3.50372616	3.41921899	3.59620404	2.87289038	5.04071585	3.61371088	4.08550692	3.64759317
PCGF1	4.57342295	4.09031635	3.92360324	3.13432549	3.96601197	3.9870539	3.02147327	3.6289909	3.31701661	4.69871474	4.04193047	4.03674048	3.75975729
GRB14	1.44979795	1.10485656	2.69193994	1.59355263	3.24880853	3.7024465	2.93809794	2.21710056	2.86706605	-1.09300033	1.95527966	3.84316801	2.48658593
CLIP4	5.43147558	6.0504316	4.894148	5.35708881	5.83401995	5.91672842	6.03611744	5.9237175	5.66643536	5.62788589	6.02888781	6.10523353	6.04918349
SPTBN1	5.73448882	6.30601437	5.77456981	4.71972719	5.59791027	5.92008553	5.83985132	6.65021372	5.92703234	6.77229873	6.6720628	6.84857198	6.15067163
AUP1	6.12830106	5.22611647	5.97895733	5.52976969	5.36322178	5.17963887	5.62657028	5.16786375	5.53945544	5.89762358	5.474069	5.77270302	4.85966406
RTN4	6.79418871	7.05172021	7.75825218	7.42050498	7.64397379	7.57300097	7.48544039	7.80792269	7.23881221	7.15842842	7.3278475	7.77344564	7.37419669
HTRA2	4.69354824	4.76558906	4.90530403	3.84473488	4.16393404	3.99614423	4.25876417	4.19969589	4.41534094	4.68327351	4.41557249	4.88928219	4.02521485
LOXL3	5.11922837	4.62285479	5.16421052	5.81161283	4.22080694	4.13270378	4.33116769	4.35871922	4.34464071	3.70913884	5.04782766	4.12583283	4.21564894
DOK1	4.81697954	4.50681986	5.29158528	3.99612923	3.86055423	3.74699758	3.90001116	3.61489796	3.98825724	4.2713444	5.4596467	4.68343994	3.5779551
GALNT3	2.6957911	3.2855634	2.27593474	3.36576091	3.07858914	3.0899798	2.19995014	2.30602327	4.43579424	-1.28525714	5.68605686	0.58448551	2.77385604
POLE4	4.23971891	3.56020699	4.83015405	3.60650069	3.5077123	4.25223376	3.94151763	3.27472575	3.82727764	3.50621065	2.05704731	3.73186279	3.33332444
TACR1	1.6028897	1.15634656	1.44399088	1.06883205	2.52993317	4.27073773	1.6883542	2.09858429	-0.05302347	-0.78410182	4.6275289	-0.78410182	3.94787653
CCDC88A	5.50456225	6.24893954	5.81126033	6.94331446	6.21128289	6.76649079	6.04800249	6.59929761	7.07144662	6.66510459	6.00571661	6.46474993	6.22924172
ACADL	5.35315204	5.35056081	5.2375425	4.09664187	6.55078626	6.8470714	6.32972427	6.96686406	5.73426905	6.29604134	5.33240919	6.25442576	5.81653287
MRPL19	1.95763425	2.90521867	2.66599979	3.84733871	3.80708317	4.41880652	3.79361231	3.4238603	3.22820568	3.70810468	2.63931693	3.14771423	4.25213998
LANCL1	5.64142128	6.28469856	5.578669	6.25795417	6.46428258	6.88270657	6.61523805	6.39376296	6.397121	5.65327811	6.75355597	6.77201148	5.28356036
WDR75	5.299911	5.98133529	4.97578484	6.28724119	5.87979964	6.08957167	5.81013702	6.00729895	6.112546	5.68512542	5.208715	5.55142023	5.14752829

EFEMP1	9.5218579	9.28646032	8.75337786	9.28646032	8.89019024	9.31268719	9.88270666	8.80088039	9.13420867	8.96011222	9.06670721	9.45095442	9.3531523
FANCL	3.42188807	4.40919746	3.61881522	4.8352142	4.43858462	4.87097967	4.47186921	5.09407787	4.40315374	4.40897728	4.62101264	5.0324498	4.24613956
FN1	11.2779266	10.4171854	12.3200801	11.7915926	10.8631884	9.77228625	11.7915926	11.2779266	10.6449362	9.70703813	10.9580822	10.0316342	10.2829385
STAT1	6.49593175	7.12307897	6.35184464	7.63781603	6.65987607	6.35545498	6.34502295	5.99379534	6.79021162	5.67092272	6.99698514	6.12258295	6.45185327
GLS	6.24822648	7.49863412	6.61968783	7.58296641	8.00286081	8.18148183	8.53386775	8.41827727	7.97156247	7.50885302	8.75109342	8.30718637	7.21080775
PAPOLG	5.03985315	5.30516727	4.77187901	5.51578096	5.57730585	6.03215317	5.65594071	5.69094446	5.50161657	5.01266473	5.31656767	5.0713753	4.85126031
DNAH6	2.11615475	2.9188205	2.13913232	2.34905856	2.29060571	2.33376425	2.32167148	2.57181026	3.32451957	-1.20011591	3.52217237	2.32214919	3.39645593
PECR	3.71211696	2.93580306	2.9763731	2.31620996	2.65224179	3.02540705	3.73551614	3.7645009	2.95466103	2.75592049	3.93498866	3.79587177	3.64250305
UNC50	4.46978034	5.18834162	4.44319859	4.93073626	5.2174911	5.30046026	5.08887778	5.32370543	4.91505702	4.62837781	5.6696336	5.63400627	3.91162769
IGFBP2	6.75315633	7.39567264	7.57114118	6.02384518	6.6475547	7.30258279	6.75113293	5.49083518	7.42607628	7.61925482	7.86521709	7.33371476	5.97397711
ELMOD3	4.90987325	4.32426274	5.08834946	4.43736579	4.54362648	4.41346026	4.71282367	4.07317919	4.14311292	4.2907164	4.65537438	4.99072853	4.55136854
IGFBP5	10.1332875	8.89091455	8.10476355	9.39481619	10.4554052	8.59785753	10.1332875	8.36128422	8.75526862	8.61788652	8.64913178	8.24480134	8.54146493
USP34	6.26464597	6.8295467	6.07093111	7.04175727	7.03693618	6.89406648	7.09245606	7.17446992	6.62982501	6.44535363	6.92996523	6.7424522	6.86450639
EFHD1	6.22402107	6.58348733	6.41974859	4.05495625	7.76562395	7.50685978	7.23904476	7.38165035	7.30356294	7.9297824	7.37660176	7.37317963	7.08346127
CCT4	5.83371685	6.46978089	6.63176916	7.08160533	6.57643166	6.21795849	7.19372587	6.27878831	5.94934329	6.47489671	6.25144374	6.47337859	6.31170561
GGCX	5.32922339	5.09272003	5.18708571	5.84328528	5.64554457	5.15816017	5.74171856	5.42081093	4.97048782	5.4332099	5.58645156	5.70156077	5.45916368
EHBP1	3.97220139	5.41928923	3.75320355	5.11673117	5.08274711	5.21679143	4.8562939	5.09637142	5.06147645	4.98446903	5.34532911	4.44593218	3.6806378
OTX1	0.11954132	0.32818449	0.11744857	0.28450409	0.36905756	0.1170381	-0.01301148	-0.0097285	3.31102425	-0.03493643	-0.03493643	-0.03493643	-0.03493643
TXNDC9	3.75844125	4.69646	4.99082329	5.8076692	5.47099615	5.28587487	5.54678678	5.37682879	5.77806789	4.87225809	5.79642446	5.40279948	4.51167427
COQ10B	4.84663455	4.86416941	5.30025408	5.62358407	5.78522069	5.82622287	5.93462952	5.77906833	5.49479337	4.59258844	4.98673207	5.80304132	5.1014226
GNLY	0.10152888	1.14245974	1.02865644	0.38733339	1.01717851	0.46675419	0.55963902	-0.05567225	2.39130941	-0.10779118	-0.10779118	-0.10779118	2.2043779
SF3B1	7.23092032	7.74992933	7.10789865	8.14098605	7.86209196	7.9994532	7.92182863	8.06526601	7.88191337	7.81961795	7.84800264	7.74374745	7.92697678
ST3GAL5	3.99236511	5.49173613	3.74387468	4.24478613	3.9650179	3.61943082	3.66104594	3.63646968	4.6904173	3.76692878	4.39547694	3.48788607	4.4400566
CHST10	4.24597556	3.8318799	4.00190507	3.13855042	3.49501526	4.18007289	3.77004307	3.85949447	3.99582188	4.15370854	4.76808623	3.97738707	4.86018547
PDCL3	4.16468368	4.74781927	4.83784548	4.5481988	4.47149721	4.91916453	4.10607101	3.66425614	4.90880137	5.37325238	4.59842407	4.30015955	4.22026797
MOB4	4.69768971	5.15204631	4.94403366	5.77192241	6.16221694	6.53563816	6.31946799	6.11780523	6.18016885	4.98702844	6.30447686	6.23216519	4.01612348
HSPE1	3.36542019	2.50137847	3.68615338	4.15969418	4.20080516	3.26876167	4.0957868	3.58928077	3.88476407	4.20064927	3.7815644	3.76170981	3.61408736
KDM3A	6.01784426	6.11577409	5.57554452	6.10473117	6.41958286	6.60893633	6.53179075	6.69553419	6.41580354	6.46240865	6.29297442	6.11936316	6.370959
PLCD4	2.74108361	1.787795	1.99001717	1.59818851	1.66607536	1.70964011	1.39646465	0.69835269	2.08382872	2.87229728	-0.59328203	-0.59328203	1.14514536
CHMP3	3.63551376	4.82325315	3.15301813	4.29302602	4.97627392	4.63491619	4.77965595	4.61169201	4.62434823	4.59921535	3.76329651	4.5380059	4.82587674
ZNF142	5.33619627	4.74901334	5.40862063	4.5448834	5.16121521	4.79129822	4.77152308	4.81107542	5.03633665	4.77752783	5.02936348	5.20382488	4.99106756
IL1R2	4.73897489	1.18970406	3.67532977	1.48806234	1.76333317	1.44778152	1.01659255	2.48377446	-0.53449892	2.37943718	-1.33247842	1.59727578	2.81264194
PRKAG3	0.85304404	0.1053643	1.27047625	0.96308084	1.42473358	2.00192655	1.52861993	1.18641463	1.46799011	2.84877132	2.23810696	3.34738327	-0.4359077
IL1R1	7.89882126	8.0288205	7.41008828	8.99264331	7.56064309	6.7893531	8.50911501	6.58754805	7.19797133	6.48146656	7.5686448	6.36592446	7.52142664
WNT6	-0.01349197	-0.01349197	0.50158924	-0.01349197	0.30384234	0.72379938	0.1797456	-0.01025072	-0.04558098	-0.04558098	-0.04558098	-0.04558098	-0.04558098
IL1RL2	1.30569427	1.05182004	1.46811483	3.2224867	0.86496088	0.79767674	1.07079143	1.38181012	1.29737864	-0.60855876	1.13643059	1.91756111	-0.60855876
IL1RL1	3.44086539	3.06206133	4.57436908	3.2238277	2.83646043	2.12405227	2.93340259	1.24005758	-0.93176118	-1.60836192	-1.60836192	1.20768059	3.55318361
IL18R1	2.99838413	3.23680272	2.37434087	4.14081014	3.48747685	3.15183773	3.46173694	3.29911896	3.82114836	3.30743003	2.65860638	2.17852187	1.6205034
IL18RAP	1.55155953	1.6099296	0.9258703	1.34073546	1.68254781	1.60016485	1.37856267	1.42857548	0.78976129	-0.47947552	1.7937964	1.87820247	-0.47947552
SLC9A2	0.7927391	0.61080904	0.81712407	0.46913061	0.46967443	0.92552412	1.03890399	1.16420046	-0.43486381	2.63762001	-0.43486381	2.51919837	-0.43486381
FHL2	4.84568722	6.16544474	4.63580713	5.47278542	5.17344659	4.27595226	5.03282141	4.3631423	5.60275052	5.43722248	5.26988239	5.21028156	6.21292564
MLPH	0.40425583	0.6967566	-0.73087573	-0.19009931	1.68297681	0.70676645	1.23951775	1.34047876	1.09449429	0.01525025	0.01525025	0.01525025	1.79598077
CNPPD1	5.76263107	5.65598049	5.99493195	4.52434855	4.81295788	4.65728055	5.46073018	4.99138141	4.98862307	5.75970442	5.11122217	5.87609977	4.24865773
UXS1	4.71115289	4.88294443	4.35610303	5.07755685	4.33687443	5.60832759	4.79921989	4.68485061	5.53132417	5.10490836	4.73564976	4.28503355	4.33828183
ABCB6	3.91409132	3.38426761	4.12141785	3.77645757	4.6491093	3.91674349	4.70216355	3.96641157	3.88982122	4.93280814	4.74656323	3.3948403	4.4801952
STK16	4.65406425	4.07099335	4.75844226	4.27725983	3.93742033	4.03450455	3.73891143	4.44175632	4.25460093	4.39667792	4.11465826	3.85654217	4.40627557
SLC5A7	0.48148754	0.4706105	0.47321472	0.4706105	0.6404447	0.67516197	0.56497726	0.64319175	-0.30620884	-0.30620884	2.97967102	-0.30620884	2.99388186

HDLBP	8.22410229	8.24532154	8.08397859	8.66849706	7.92407101	7.89430419	8.0513719	8.08397859	8.33896197	8.29569419	8.25610966	8.21022983	8.17904762
PPP1R7	5.79040878	5.1859923	5.63497298	5.12517189	5.15395025	6.01698882	5.12569802	5.39617681	5.33069267	5.56670629	5.37167122	5.32004423	6.0652158
PASK	3.28778798	3.22025471	3.64132066	2.6916687	2.10358731	1.63028914	3.30933237	2.67465994	3.69500966	4.93467532	3.44362155	-1.16023753	3.03638933
STK25	5.64712376	5.49222317	5.72442964	4.66059799	5.38512482	5.10147164	5.12275796	5.22023912	4.89651223	5.82089082	5.66609277	5.65125769	4.6897475
TPO	-0.3150715	1.48181041	-0.31285281	-0.08472633	2.48724574	0.87260697	1.96221508	0.83370478	-0.23276859	3.20818746	-0.23276859	-0.23276859	1.96362748
PROC	-0.11441156	-0.11441156	-0.11199421	-0.11441156	-0.11441156	0.46694904	-0.11441156	0.71066618	0.02727477	0.02727477	0.02727477	0.02727477	0.02727477
ID2	5.73907401	5.63063257	5.1906579	4.86086687	5.91272202	6.63634695	6.87811225	5.95829658	6.38495879	6.78598048	5.82716192	6.93470035	5.87826012
TAF1B	3.7415944	5.15357342	4.11868026	5.19926734	4.52861066	4.61121576	4.81757997	4.47421537	4.39675791	3.86279895	4.53753981	4.32542588	4.19863963
HPCAL1	4.74067761	3.66793102	5.08647833	3.58331869	3.25604589	3.84377988	3.45029869	3.27941501	4.15681205	4.49202306	4.36746814	4.58927136	4.05751343
ODC1	5.96126949	6.44183869	6.54284479	6.19765403	5.27602702	6.98981819	4.58350131	4.94893295	5.70910582	6.26592979	5.82350366	5.36549082	5.23848078
BIRC6	6.91312284	7.54902439	6.78708782	7.8229055	7.46750518	7.44336535	7.56721573	7.54182553	7.1539652	7.16688982	7.63178245	7.44012013	7.715561
NOL10	5.21998341	5.47276347	4.69069375	6.02113483	5.57773279	5.92258991	5.82118075	5.49372441	5.53437843	4.90675821	4.97685821	5.40608953	4.95785616
PLEKHB2	6.07476087	6.13167361	6.38244394	6.66854242	5.65636214	5.4305317	6.08617767	5.40212085	5.63921893	5.54209386	5.80849892	6.2263235	5.68731002
GORASP2	6.66078808	6.25323763	6.14711266	6.91694533	6.82331527	6.44083975	6.29476207	6.21021439	6.26555777	6.95344728	6.73870496	6.32657989	6.77773183
STRN	6.1227238	6.38570166	6.07682857	6.72716301	6.93765912	7.07669753	6.87362641	6.94950327	6.73225068	6.50124362	6.58617434	6.82702983	6.95932429
CEBPZ	4.93656402	5.71519099	5.49557287	6.61526543	6.13909222	6.48251384	6.1188694	5.99624434	6.28113401	5.8601418	5.38549576	5.66055456	5.42783797
PRKD3	5.45859401	6.25080283	5.49316374	6.63045667	6.44670561	6.54415079	6.48822485	6.48237781	6.3618138	6.23458687	6.79729879	6.59380654	5.61817033
DCAF17	4.72369666	4.74366339	4.33117754	5.33216597	5.35622119	5.64216865	5.61606619	5.46355354	5.28477129	4.10728456	5.31747342	5.45746224	4.52978357
QPCT	3.65360516	1.38979495	3.74749792	3.9273795	3.17109595	2.54399399	2.49893817	2.34435931	3.44938709	3.22500771	3.2925371	3.56865853	-1.42698543
RAB3GAP1	6.73194987	7.13559358	6.78866131	6.83740728	7.47611094	7.41858656	7.40200796	7.29002327	6.98208991	7.06548041	7.00180714	6.9707401	7.47068478
SLC25A12	5.57505033	5.89741871	5.06638415	5.16631563	5.62600113	5.67310645	6.04009606	5.78821596	5.4244393	5.64701253	5.2947996	5.52993415	6.49511709
DLX2	0.0693583	-0.01011939	-0.00785649	0.20157418	0.02790833	-0.00886125	1.30275618	0.45027057	0.13536525	0.13536525	0.13536525	0.13536525	0.13536525
LCT	0.26350556	0.17557597	0.07805379	0.1501578	0.09180844	0.53326645	0.33801534	0.16084681	-0.1104579	-0.1104579	-0.1104579	-0.1104579	-0.1104579
DARS	5.76848392	6.12670613	5.27119406	6.62239763	6.26116924	6.64992485	5.89023269	5.63560286	5.97859416	6.40202038	5.35473766	5.58581108	5.60957585
SRSF7	5.35165368	5.97149268	5.34984782	6.27080986	5.88074979	6.58017017	6.10256855	6.17227866	5.59149273	5.92085565	5.97858743	5.85848057	5.71555696
SDC1	2.32976558	2.05133283	3.87022126	4.12218262	1.22100105	1.4346732	1.21278319	1.54328832	-0.17269266	2.35456364	3.76197572	3.24216323	0.14349501
PLCL1	6.19695381	6.73770902	5.63760926	5.38061873	6.18728193	7.10571434	7.0020968	6.60554172	6.76640021	7.10512017	6.84377793	6.689641	6.84872295
SLC1A4	5.67195822	5.06065744	6.15916656	5.05918474	6.51415956	6.40071769	5.24299856	5.90244265	5.99650548	6.5771037	6.2832756	6.2750466	5.93029601
SOS1	7.16565697	6.24947919	6.50558613	7.36100283	6.95776695	7.44362755	7.54076648	7.18770925	6.95008665	6.22992008	6.79435806	6.82000978	6.6302945
KYNU	1.66031066	2.5944361	1.89934094	4.04396188	1.43556053	1.53172795	1.72461302	1.41077291	2.70911509	-0.9726326	3.44509738	1.90907081	2.70314515
WIPF1	6.77479573	6.01826014	6.54086544	6.20775862	6.09925251	6.0804317	6.51981927	6.30804963	6.27186954	6.34964256	6.38460835	6.58809576	6.29751829
ORC2	4.71452416	5.20427787	4.78733152	5.52326987	5.72257463	5.43660832	5.55246895	5.63951217	5.31079347	5.43818666	5.20930092	5.67454788	5.34725217
COX7A2L	5.07733045	5.55654141	4.77520783	5.08485292	5.23854381	5.39442409	4.42556399	5.02757846	5.45553517	5.7924476	4.97715594	4.97520846	5.25866426
PNO1	3.59118121	4.50404084	5.29676205	4.84830363	3.98690839	4.49013972	4.31915577	3.639064	4.35432424	3.96303524	3.65718293	4.14401252	3.68356825
ORC4	4.41092791	5.33592126	4.79149968	5.12023111	5.58237964	5.74159132	5.36009765	5.74017084	5.42478096	4.79077337	5.42502897	5.75645545	4.5698506
PLEK	5.41891679	5.33616533	5.16638233	6.02383186	4.00980442	3.38424608	4.242981	3.28374993	4.29118986	4.22602083	4.19114193	3.67578502	5.19861466
RND3	6.96025299	6.5799438	5.74887003	7.78024937	5.5694386	6.38585027	4.91273457	5.43596838	5.64583824	4.73523972	4.50104363	5.47091238	5.45712705
ATF2	6.09891051	6.36717635	5.57635129	6.54049446	6.63601486	7.02366275	6.55371487	6.69212403	6.44981119	6.58833387	6.51560668	6.38394897	6.37807374
THADA	5.54162484	6.05313129	5.60509105	6.59554096	6.03429582	5.6180708	6.06549944	6.00897775	5.44847625	5.09484914	6.28431013	5.95747237	5.72456907
AAK1	6.29152277	6.12275799	6.29311641	7.03049078	6.62705677	6.10375905	6.51502721	6.36566915	6.1281221	5.86496249	6.37632608	6.04154055	7.01838793
TRAK2	7.65330354	7.84356486	7.13261952	7.56290636	8.0075354	8.15332048	8.29621649	8.0737952	8.31603766	7.62431165	8.05075705	7.56985767	8.09644667
C2orf42	4.77141849	4.60390047	4.50699847	4.45058549	5.21487368	4.42341815	4.82440239	4.4538425	4.67840153	4.72877352	4.5978013	4.27100549	4.83070464
TIA1	5.46457277	6.08678734	5.4582097	6.71568701	6.3855705	6.28939711	6.68517939	6.64844708	6.39285355	5.54199221	6.76021123	5.99018397	6.179031
PCYOX1	7.13751404	7.24080432	6.72925627	6.97562844	7.52481759	7.87835103	7.84772924	7.35207293	7.42770253	7.88236456	7.6572144	7.61072242	7.62944904
EPAS1	8.99336874	8.54787328	8.4838743	7.26750545	7.86767748	8.53141616	8.51440719	8.16653486	7.56258712	8.43978622	7.87852063	7.93451929	8.37405169
ARID3A	3.46545794	3.02603382	3.91281225	3.04095435	2.23793938	2.34334257	2.33064178	2.51506606	2.62317548	3.01135228	2.68023164	2.81294116	2.35679165
SUMO1	4.85948301	6.16017391	5.99529085	6.71227117	6.6502666	6.79139115	6.740516	6.46766162	6.72046065	4.99648273	5.96044657	5.82240917	5.25976843

CD207	0.28808769	1.93531221	0.08700457	0.53175715	0.64557742	0.26339159	0.25367925	0.33444373	-0.17681172	-0.17681172	3.4417494	-0.17681172	-0.17681172
GRIN3B	0.45764519	0.18615306	0.05739744	0.05307496	-0.19076226	-0.18937991	0.00497733	-0.18759031	-0.17780711	-0.17780711	-0.17780711	-0.17780711	-0.17780711
VAX2	0.39197976	0.17041066	1.08904908	0.58349238	0.30860053	0.17176242	0.17041066	1.37308432	-0.26257897	-0.26257897	1.79592024	-0.26257897	-0.26257897
ATP6V1B1	0.91240596	-0.21882335	0.53868781	0.75731224	-0.19798705	0.24217535	-0.02506163	0.03544317	-0.01966221	-0.01966221	-0.01966221	-0.01966221	-0.01966221
NFE2L2	7.02251766	7.04499591	6.17740319	7.06516351	6.85836902	7.40469128	7.4338723	7.18432609	7.46046753	6.82505588	6.98130302	6.81493055	7.00430006
MSH6	3.64654404	3.64265085	4.21322796	4.66402205	4.12171728	4.82544226	4.42515087	4.45870198	4.59120039	4.20667698	3.25844083	3.57086859	5.14987013
PLEKHA3	4.98317613	5.46623703	4.91785604	5.27943113	5.98796508	5.89915561	5.7714016	5.66071567	5.54729889	4.83059929	6.09264877	5.92392747	5.088258
SPR	5.26843548	5.22164024	4.96956954	3.56755198	4.40424705	4.760871	4.88864206	4.16036413	4.34267515	4.82899135	3.90340864	4.61235408	4.17957347
EPHA4	6.63093619	4.7835913	5.94881983	5.3544106	6.05225415	7.02653901	6.26456603	6.21889886	6.48419843	5.56149237	6.75728411	5.25727889	6.72745903
PARD3B	6.04791099	6.96218678	5.62867116	4.88936712	6.59494087	6.61977245	6.36107348	6.48452282	6.72240772	6.68248149	6.63665072	6.35216002	6.58215802
FARSB	4.55353499	4.83387614	4.82360318	5.7580514	5.14109632	5.38468742	4.7763649	4.85161722	4.79299569	5.11551285	4.75238732	5.07131636	5.1079906
ALMS1	2.23628894	3.07463015	1.99707341	2.54418964	3.24955398	3.87996806	2.53198446	3.35503831	4.04813437	2.84413289	2.97224531	-0.33001562	2.30467702
BCL9	5.7276267	5.01312497	5.10913505	4.59947896	5.59373761	5.52331229	5.25562875	5.59005599	4.90966994	5.70289688	4.9846655	5.26278306	5.51641791
PRRX1	7.33733869	6.67532672	7.38223567	8.9278517	7.30078727	6.5706145	7.95439232	7.09339479	7.11076719	6.46894394	7.03837061	6.51158575	6.68206129
DHCR24	4.2246938	5.32330148	5.40580676	4.91509124	4.55509733	4.80023515	6.46071179	5.51782328	5.36031765	3.90869973	5.19922888	4.81130067	3.24359521
DNAJC16	5.24465542	5.34857503	4.65700211	5.34422444	5.24610524	5.39555346	5.19737319	5.18769411	5.1756747	4.96263068	5.35372704	5.01195151	5.04704439
MARK1	4.37324222	4.19802254	4.1283068	3.82962878	5.08844679	5.53332826	5.01062824	5.83167435	4.62124026	5.18132175	3.97983953	4.95901918	5.03033715
TNR	1.3370916	0.76738698	0.63075163	0.67827722	0.50232921	0.6436455	0.56502702	0.45711116	-0.34786364	-0.34786364	-0.34786364	-0.34786364	2.81027683
MORN1	1.16286286	0.71741233	1.03022836	0.73223507	0.76514099	-0.44404402	-0.11769326	0.19402395	0.03231265	0.03231265	0.03231265	0.03231265	0.03231265
GPX7	4.18064467	3.94072472	3.81762623	5.08983352	3.40234013	3.36908649	3.39107172	3.74998044	4.40321047	4.70573364	3.34325971	4.10993428	3.1470386
CACYBP	4.6892514	5.05637391	5.60294465	5.39254633	5.88268179	6.18705184	5.65049911	5.45859483	6.01380313	5.1620043	5.3621326	5.34867409	4.72369295
SCP2	1.19935815	2.54523495	1.51763706	2.38483709	1.42663941	2.57112324	2.33495331	2.29914363	3.31738712	3.86888653	0.17330428	0.17330428	0.17330428
TPSG1	-0.127831	0.84489806	-0.31553753	-0.31752094	-0.31752094	0.07535385	0.00984888	-0.00642948	-0.0925244	-0.0925244	-0.0925244	-0.0925244	-0.0925244
PAPPA2	1.26136638	0.74660484	0.50414636	0.10255281	0.41009659	0.23873231	0.21771092	0.50644945	0	0	0	0	0
RALGPS2	-0.27160832	0.83228321	-0.4068293	1.70987698	0.17247442	0.43867996	0.55912281	1.26393466	2.08738105	-0.08172455	-0.08172455	-0.08172455	2.78722627
ANGPTL1	4.98064335	4.86929038	3.87709145	4.33143393	5.67543804	7.1363362	5.43694637	6.45709221	5.34545211	6.76469107	5.13980244	6.63084492	5.74664202
CEP104	5.94660541	5.64320795	5.69715007	5.05033819	5.83706872	5.99113676	5.96475403	5.53865694	5.54946555	5.78571041	5.61608068	5.86053013	5.81606772
FAM20B	1.3797628	2.20688549	1.56969309	1.62992806	3.06325437	3.27903237	3.27306079	3.11593186	2.90383234	3.30732459	0.09741839	4.14017723	2.10772344
TCEANC2	2.05576648	0.77297785	1.40585003	0.88078322	1.16882046	2.10672297	1.84339569	1.09803008	2.87627595	-0.19955322	-0.19955322	-0.19955322	-0.19955322
TMEM59	6.26942242	6.33971545	5.9446021	6.94770781	6.72837566	6.82411077	6.85832632	6.82113149	6.6306295	6.60624336	6.57924099	6.80986867	6.02873992
LRRC42	4.63843825	4.91845989	4.54436896	4.73580685	4.30850832	4.90734443	4.39245557	3.72381486	3.72981698	4.14561516	3.56625306	4.39685146	4.12941116
WRAP73	3.83243027	2.52073131	3.57338871	2.66345474	3.61883975	3.15935337	3.62967214	3.38543102	3.20943657	2.85138182	4.01144524	3.31937059	2.17650128
MRPL37	5.69504511	5.59119417	5.99118781	6.08807162	5.53584344	5.42716353	5.40634418	5.16387516	5.58952563	5.69690279	5.30865866	5.8137982	5.99298419
ICMT	6.13152626	6.59888125	6.10453721	6.07260681	6.40208898	6.75379958	6.33182753	5.88775201	6.19177607	6.45535261	6.72091408	6.43436389	6.54101742
RPL22	6.21171028	7.13042641	6.65619953	6.75354519	6.53174017	6.64716899	6.19413851	6.20212943	7.34420763	7.50905296	6.64441605	6.60885349	6.41789806
CHD5	2.54415533	2.16230254	2.69273422	1.79153922	3.26995437	2.7231733	2.08888333	2.94306738	2.41563583	4.4161239	2.18991342	3.23918382	2.02155224
QSOX1	6.15773501	5.92544869	6.79242679	5.87776707	5.64071872	5.64225531	5.87729164	5.84832867	5.56476965	6.47195385	6.18573073	6.10489185	5.70477261
STXBP3	3.4238298	4.25727359	4.07653982	4.84780519	4.88353265	4.88718822	5.19918765	5.34197595	4.96786265	3.55448669	4.9764968	4.26658702	3.39186754
PHF13	5.61253971	5.03721336	5.80055366	4.92038077	4.79446943	5.01045288	5.53956504	5.58046483	4.5680689	5.78022659	4.86480576	5.23049031	5.24933639
ERRFI1	8.01772489	5.94100208	5.93109716	8.90264164	7.42316437	6.71065188	6.24818318	6.40760379	5.42834915	5.85717579	5.22305332	5.77864871	6.27512166
PARK7	6.69835191	7.01012119	6.57187791	6.53012826	6.30372322	7.10587244	6.57868272	6.18047131	6.58470937	7.09482605	6.19066827	6.62937697	7.01559541
KIAA1324	0.69197014	0.11375195	0.40992948	0.75166865	0.15547194	0.28228094	0.4446409	0.16650043	-0.19520338	-0.19520338	-0.19520338	-0.19520338	-0.19520338
AMPD2	5.84912508	5.43692628	5.65683664	5.64793686	4.77287063	4.62076866	5.14149755	4.56551568	4.94977337	5.49751323	5.18189494	5.02342664	4.3786158
SRSF4	6.21301932	6.17265275	6.10829515	6.51886391	6.29503931	5.56583561	6.23840356	6.54893968	6.0058341	6.25248251	5.45198471	5.6700178	6.56392958
MECR	4.57223729	4.16019219	4.60364456	4.13681483	3.97078098	3.87361006	4.15597496	3.62749885	4.06522445	4.02927387	3.61349243	4.23945777	4.66942243
KCNC4	4.76004717	4.91493351	4.31984278	3.03943617	4.70243113	3.94987	4.63627937	4.68396504	4.46411356	5.29076896	4.76749054	5.32655312	3.74687079
EDEM3	5.83171671	6.25498205	6.23840446	7.06803886	6.3668093	6.62892738	6.66756793	6.52652272	6.27888897	6.28932562	6.42746049	6.26454607	5.72073403

WDR77	4.41844643	4.95494853	5.35914831	5.06577268	4.64822251	4.21862193	4.62965454	4.4490404	4.72181733	5.11064575	4.6975353	4.45902768	5.30735775
ATP5F1	5.61926836	6.02796845	5.57125938	6.745923	5.85494183	6.34334849	5.87843017	5.85677139	6.03478383	6.22977051	5.79876081	6.2213241	7.06411975
RAP1A	2.82882722	3.90274896	2.25720907	3.46689341	4.89957091	5.13961582	4.79441453	4.90034941	0.22137518	3.7371586	3.63873782	3.70594471	3.88508153
HDAC1	6.10127411	5.61845003	5.65918327	5.95581693	5.5324805	5.46477921	5.97169392	5.48288245	5.42785881	5.80338344	5.76412515	5.59301253	5.7965896
CAPZA1	4.07747719	5.01831979	3.89630918	6.36381469	4.95568848	4.70690105	4.97946759	4.62941084	4.34481432	3.83856364	3.7123566	4.57057987	5.24582694
S100PBP	5.21736441	5.39404652	5.32542418	5.82569152	5.89404778	5.48456176	5.89751006	6.09770673	5.77229479	5.16817328	5.20310902	5.26735088	5.84511454
RNF19B	6.39105825	5.96199252	6.21431367	5.76711694	5.36914368	5.08392309	4.96087352	5.35629494	5.41487665	5.6476496	5.35427783	5.3166391	5.23820308
SCAMP3	4.32763099	4.90263434	5.17984709	4.51103368	4.015161	4.01630054	4.18524036	3.75352893	4.30644362	3.95860575	4.49983475	4.94031176	4.70245485
TRIM62	4.22699996	3.26012352	4.25006141	2.66066019	3.58763617	4.21463129	3.51269467	3.74550168	3.95211793	3.83390354	3.45889064	3.71372238	2.99936259
ASH1L	6.25008969	6.46190163	5.90801293	6.16618225	6.58258088	6.90052392	6.55719399	6.46840787	6.12691748	6.6494825	6.22868685	6.33063535	6.22479244
DLGAP3	0.1255605	0.02457919	1.22727581	0.26465389	0.23901612	0.10402056	0.05159145	-0.06439796	-0.14598729	-0.14598729	-0.14598729	2.71467707	-0.14598729
SFPQ	6.63231003	7.32458564	7.28066028	8.17632399	7.66385364	7.02087235	7.3408303	7.79747664	7.55621712	6.78062749	7.74677955	7.04363918	7.37707993
RHOU	4.89008749	4.06610148	3.31674552	3.23265262	3.87066973	4.90467436	3.97049522	4.06333342	0	0	0	0	0
GON4L	6.2657605	6.01798871	6.61416167	6.27463237	6.03411544	6.06304762	6.08332553	6.44646497	6.15166809	6.68102563	6.27748787	5.91834195	6.15444528
ARHGEF2	6.68424871	5.87617104	6.9212804	5.47026515	6.50912377	6.31949365	6.15910172	6.61287344	5.96872778	6.76163635	6.13988424	6.08730495	5.86031276
LAMTOR2	4.30423719	4.29502906	5.20570889	3.69395117	3.48159729	4.60638348	3.39717828	3.51159079	4.60845469	4.63973346	3.29588452	4.01992871	4.61758125
MEF2D	6.72991789	6.22977599	6.80995545	5.67498497	6.6045105	6.20705243	6.28061075	6.20336232	5.86787079	7.06860506	6.5966074	6.63201763	6.68862128
DOCK7	5.63138727	6.01277259	5.69045128	6.32684729	6.4001663	6.27730869	6.40473609	6.45103276	5.99537842	6.29297684	6.02813998	6.1808658	6.13335004
SRM	5.34677132	4.93002637	6.00655012	5.90464291	4.82617797	4.58356822	4.27729503	4.136617	5.62419403	5.01664109	5.30872883	5.02969294	4.47852399
FBXO2	3.63099545	3.53296741	2.11812493	1.6864314	3.80333669	3.62903299	2.73504369	4.08782088	4.03486871	4.2238821	1.77766175	3.88271546	0.90572521
FBXO6	4.6672603	2.85829453	3.31226418	2.7096784	2.87436226	2.68651583	2.8423136	3.24854322	3.11631926	3.50602015	2.79693758	3.19080389	3.88340699
C1orf21	4.09134357	4.68568636	3.01223723	3.48580583	4.54568482	4.48659794	3.96755235	4.87707879	4.32102719	4.14411922	3.61941567	3.09232009	5.35083586
SWT1	1.73430779	2.80575687	1.8413983	3.02984855	3.59595044	3.75848351	3.41629284	3.67866833	2.95196976	2.45717198	4.15940338	2.62780672	1.55951414
MAD2L2	4.40034376	3.0841893	5.3727359	4.00184944	3.279783	3.38794504	3.23023777	3.69098307	3.95350831	2.99538168	2.99978694	2.99762419	2.86663359
DNAJC6	0.83162787	-0.05072413	0.14159328	-0.08655381	0.66517395	1.5696878	0.25598788	0.3724689	0.11251938	0.11251938	3.18973993	0.11251938	0.11251938
LEPR	3.41923503	4.64040784	3.32231758	4.08152358	5.03183134	4.37460045	4.93656273	4.59871138	4.29641735	3.88098181	4.92334581	3.94433316	4.11748313
IVNS1ABP	5.42285257	5.95142021	5.32011109	6.98025054	5.94341367	5.37621709	6.14088775	5.79704648	5.56683944	4.7664442	6.0090419	5.47057772	6.09671145
KIAA2013	5.59440795	5.21620569	5.8085618	4.70551214	4.64692411	5.21224396	5.07370864	5.05826302	5.15708286	6.02956533	4.94023926	6.00536189	5.17397823
MFN2	6.77748041	6.47803047	6.95760667	6.34213057	6.21453251	6.33455121	6.40537275	5.78134593	5.94636265	6.84182916	6.59360178	6.46590166	7.47452094
PRG4	0.59714204	7.84484545	0.49877903	5.41716835	2.12584906	0.15335825	0.63729719	1.13342365	3.50618655	-0.58128517	7.24073467	-0.58128517	4.11154855
MIIP	3.46618388	2.70931758	4.47040778	2.35146664	2.42104274	1.68821954	2.48525791	3.03759613	3.8315548	2.98521103	3.43016183	3.41225121	-0.67138962
SMG7	6.43456467	6.29189857	5.93936201	6.35750572	6.0690767	5.93244841	6.27260953	5.4468675	5.92669737	5.4681724	5.91969233	5.51222398	6.44789026
NCF2	5.3730907	5.03902022	5.28300619	5.59296612	3.38605626	2.897436	3.17009744	2.85686603	5.02720487	3.28132209	3.32447282	3.20084504	5.12384082
SLC35D1	5.3630584	5.29273282	5.43083045	5.84071195	6.04379266	6.00238426	6.25874582	6.33147927	5.51946629	5.70920687	6.28748382	6.25803248	5.90170636
PLA2G4A	3.01989533	4.47884836	3.41290098	4.66615917	3.72244232	3.98775272	3.67484051	5.16262921	4.10343836	3.34829511	3.13633174	4.34172399	4.58411987
GADD45A	7.06911684	5.99366929	7.68516174	5.38583243	6.82474818	6.52710122	5.99297753	5.81022512	7.07230424	6.28777669	5.69782964	5.71009983	6.12857769
WLS	3.70697538	3.77588455	3.70307477	3.54711306	2.73178344	4.52037575	3.61405038	4.17837249	4.12747302	3.9588291	1.4571145	4.09380826	3.29452858
PRDM2	6.70563552	6.52386692	6.31590716	6.27593062	6.76393649	6.58865202	6.80741291	6.88340355	6.86081327	6.84524039	6.55503401	6.53867196	6.82194186
RGS2	7.27829374	5.76893004	7.02392009	8.89307136	6.03937892	7.2235215	5.91552525	5.97396128	6.72681436	5.55865715	6.56694525	5.56479729	6.6874338
TROVE2	5.63073422	6.13469878	5.47809642	6.6128531	6.59289761	7.05659965	6.69184515	6.70595917	6.59368015	5.63336316	6.57434691	6.5415901	5.27623053
AMPD1	0.87320255	1.0633743	0.32391341	0.37547427	0.7009159	0.58335349	0.37653571	0.32462067	3.28854047	-0.1918429	-0.1918429	0.84401215	2.35464554
UCHL5	4.39054248	4.70112659	4.25140298	5.38323015	5.3866734	5.74205182	5.31896044	5.36683716	5.75733023	3.78953244	4.99272278	5.00466576	4.54161323
BCAS2	4.47677948	5.13830496	4.26176277	5.58363817	5.55225031	6.11677586	5.26110515	5.21551692	5.87033461	4.67262984	5.03298034	5.70978453	3.8847785
SRSF11	6.00027538	6.29646275	5.80426593	6.83277016	6.67044612	6.42046815	6.874159	7.08749025	6.7765304	6.08040968	6.52005979	6.43722505	6.43698371
CTH	3.27243248	2.83410715	2.8646033	3.06832915	3.56969643	4.5797979	4.07273507	4.12376243	2.65543908	2.93462538	3.66720451	4.13630792	4.17153319
AGMAT	0.02449233	-0.16695816	0.18004159	1.19130921	-0.12897325	-0.16567165	0.37454955	-0.16400613	-0.16553826	-0.16553826	-0.16553826	-0.16553826	3.11251203
OLFML3	5.78881436	5.33724972	4.99309236	6.95687568	5.64650526	5.12923213	4.09660327	6.35534463	5.66019613	6.36707865	5.99660653	6.60199757	6.96161853

TNNI3K	2.8507863	3.05406002	2.8533713	2.8507863	2.95888728	3.3272159	3.07358923	3.1690997	3.68521755	1.86583386	3.803689	3.13309051	4.96679198
CFHR3	1.36161851	1.89532749	1.23560694	1.23302402	1.23302402	1.53894635	1.84751782	1.3889825	-0.21859406	1.64910304	-0.78972995	0.23548172	3.16129634
PLEKHM2	6.84176452	6.46744467	6.97471287	6.00811389	6.1566025	5.36645955	6.27630492	6.36177749	5.97403258	6.5024463	6.1846316	6.40603549	6.1601087
CRYZ	5.23453676	5.2754547	4.27382012	5.3454493	5.85253346	6.12643622	5.55067597	5.64958792	5.61162316	5.33955757	5.51281177	6.14511681	6.01375796
PHTF1	1.19706623	2.12225127	2.36042951	3.23472597	2.16541504	1.71123166	2.77929625	2.75361373	3.27261254	3.07820282	3.30184918	4.05779536	1.4788922
ZBTB17	5.12057535	4.71576193	5.38864904	4.13517118	4.67546804	4.51170633	4.51898676	4.76792089	4.39835023	5.20584727	4.85922375	4.81861096	4.62342605
CD58	3.52983907	4.1613055	3.28601086	4.88861323	5.0664475	4.62798968	4.87950335	4.71853525	4.97984388	3.86869349	3.94297753	3.97001523	4.11580945
TFAP2E	1.45442504	0.69762026	2.45816084	1.7886854	2.06925005	1.0782607	1.91852205	1.61200875	2.29383717	3.10691245	3.05810736	-0.8633726	2.96868391
CD2	4.34108675	2.94625677	1.29959157	2.78867828	2.74300306	1.07450172	2.62600347	1.02035019	2.23507183	-0.92610316	2.6726269	2.67924396	0.81638077
TTF2	4.79518167	4.26971594	4.84317702	4.5937341	5.08474163	4.39546879	4.25916915	3.90040653	4.40984386	4.69550041	4.23976564	4.14435013	4.82617408
NR5A2	0.68103114	1.74982302	0.89940841	2.36652067	2.07467524	1.36707315	1.33162727	1.94596406	3.32951008	2.68338695	3.73722068	-0.45283563	4.82750848
KIF21B	3.90908666	3.55058602	4.080524	3.49270508	3.14707992	2.48312056	2.96033989	2.87881402	3.3960697	2.9986176	3.13311121	2.07016727	3.40956341
TMEM9	5.59312539	4.83039497	5.02388088	4.83672242	4.55457778	4.97550269	4.52812937	4.40419377	4.68599779	5.30939521	5.0918276	5.21832813	4.44335843
ADPRHL2	4.30449603	4.21168262	5.16637616	4.10382565	3.4496514	3.98039808	3.61514244	3.95756238	4.74047188	3.95721496	4.29015627	3.73633633	3.42492339
MAP7D1	6.21285258	6.19536319	7.22290191	6.04493623	6.1121794	6.25856253	6.10073871	6.11121746	5.88692537	6.84859194	6.51717975	6.84103696	6.58893684
WARS2	4.43113317	3.99437656	3.92899348	4.90691421	4.86543032	5.01413713	4.56099484	5.11752375	4.431234	4.10480483	4.73846175	4.47455164	3.81927875
HAO2	0.19881511	0	0.00124472	0.17691999	0	0.00069205	0	0.13809328	0	0	0	0	0
OSCP1	4.55887873	4.10464398	3.21194276	3.31618238	3.12628953	3.68835803	3.72741083	3.18634615	3.73667751	4.28196021	2.86915971	3.31068472	3.79524275
MRPS15	4.73692573	4.38812197	5.3090038	4.49088222	4.40875381	4.96845781	4.72402607	4.4518625	4.29505658	4.9118675	4.36759427	4.80531068	4.89656487
EXOC8	5.260366	4.98229896	5.09917896	5.22223679	5.41240027	5.58262103	5.58190994	5.32525746	5.60507994	4.92806872	5.00051293	5.4732486	5.33655935
GNPAT	4.95620342	5.34904141	4.9968769	5.81739026	5.69346654	5.53705173	5.90651985	5.73539737	5.23050767	5.54568008	5.48646151	5.36544606	5.88528654
TSNAX	4.19824717	4.18705733	4.0734012	4.78284051	4.68998168	5.01597992	4.91762087	4.96121204	4.60944805	3.68271887	4.74593571	4.73981347	4.86585834
C1orf109	5.06852542	4.81701587	5.53068394	3.89482862	4.44358961	4.76995939	5.07350162	4.76649577	4.48173759	5.20228122	4.55047798	4.85115342	5.11459781
RRAGC	4.54377107	5.14876161	4.56662033	5.26049788	5.61009424	4.95627369	5.89419539	5.46222226	5.07107154	4.80290263	4.77021304	5.10041862	5.00939652
TBCE	3.11853787	3.53741267	3.19687896	4.67046911	4.17460759	3.72480197	4.12493215	3.65841043	3.50134663	4.67684046	1.71950159	3.82099142	3.75364456
NID1	6.10990129	6.08446317	5.90511992	7.34464859	6.32056548	5.93642297	5.67875016	6.79052473	5.67393738	6.03274096	6.15285302	5.43972678	7.16400796
LGALS8	4.16982267	4.32217013	2.19907191	4.19259214	3.61628279	4.08591597	3.52231222	3.30660195	3.87098351	2.55261379	4.42553752	3.66141793	1.90148105
NT5C1A	1.02291331	-0.08914839	0.73070742	-0.14195179	0.29895812	2.15277889	0.66533155	1.96462767	2.39477045	4.18395109	-0.14923475	2.77695379	-0.14923475
HPCAL4	0.41626111	0.57008559	0.52591843	0.43486646	0.28770547	0.7039794	0.38798491	1.32565626	-0.21511385	-0.21511385	-0.21511385	-0.21511385	-0.21511385
MTR	5.42146338	5.936598	5.18230729	5.2519707	5.44924282	5.2499506	5.43861188	5.61441612	5.17482015	5.28832573	5.46322085	5.81977554	5.9129198
BMP8B	0.63610538	1.0734585	0.63757773	1.00797148	0.61639239	1.10027959	0.88035305	0.76688653	1.34143528	-0.36083281	2.35174225	2.57909854	-0.36083281
SIPA1L2	5.28295271	5.6521737	5.1463395	6.18053201	5.02208251	4.75897895	5.00506881	4.60118631	5.35328996	5.01559298	5.74901675	5.15323175	6.34657367
RLF	5.62008635	6.13217852	5.71408498	6.65771256	6.66931546	6.87000741	6.61610054	6.52589486	6.10094344	6.23553962	5.8226496	6.27772792	6.00367305
KMO	1.2441567	0.92212893	0.98725093	2.07774387	0.74527261	0.51212425	0.74958958	0.57663082	3.71656933	-0.50913866	-0.50913866	-0.50913866	2.68135999
ZNF684	3.14159533	3.56467184	2.851113	3.75370414	3.71733379	4.10151553	3.44030794	4.03171697	4.20345631	3.45380705	3.80866616	4.10788375	4.08822996
KCNQ4	3.55507609	2.77923187	3.71687216	1.00015528	3.85457295	3.64413152	3.76877665	4.03335653	2.85734294	5.30210573	3.75410876	4.50669711	2.3624488
RIMS3	5.37447381	5.57348503	5.47991891	3.52859453	5.71492997	5.8221591	5.98081007	5.91435251	5.32537914	6.91877082	5.32681872	6.33340206	5.5770789
AKT3	6.52184159	7.1023244	6.27440479	6.86165457	7.56753112	7.39425216	7.37591828	7.60037553	7.40694184	7.17213269	7.32819119	7.39970218	6.43487047
ETV3	6.29741224	6.04657367	6.02591435	5.73193269	5.78832649	6.46775306	5.97360726	5.61266366	5.99006383	6.02011861	6.07749842	6.04628997	5.81772212
ACADM	4.14558751	5.04127608	4.69671958	5.30869769	5.5439173	5.84313649	5.45052935	5.81390302	6.03263393	5.71510364	4.03503479	5.00326775	6.4298889
ST6GALNAC	2.62412353	5.64797379	3.4265844	2.85692569	4.51496317	4.05487686	3.24320959	3.03288749	3.80301849	4.59117769	4.78919093	4.57825488	4.48412779
SLAMF1	1.5411462	0.86631473	0.60502112	0.93687429	0.81904329	0.67743335	0.62054431	0.50372473	-0.37025321	-0.37025321	-0.37025321	1.62250583	2.03483002
CD48	4.50148708	4.23608113	2.93160814	4.59933111	2.54619144	2.16895168	1.75815769	1.48094802	2.27242724	2.37849073	2.45936277	0.37655057	3.84572234
PADI2	3.76258972	0.96692673	1.06364298	3.16301884	1.36467974	0.73917183	0.74494517	0.96224641	2.77808366	2.74467701	-0.73390024	3.15664036	1.36801718
SDHB	5.54246492	5.63237347	5.02018605	5.34751296	5.42271471	6.24526973	5.39031901	5.3917103	5.75733777	5.85501235	4.80510551	5.6936703	6.15933636
MFAP2	5.3681565	3.57733779	5.54804687	5.75148513	3.14372629	3.76520205	3.52487856	4.10905908	3.99480748	4.87468984	4.14951054	3.95786356	2.66826844
RPF1	4.65655471	5.13014775	5.06965893	5.69789719	5.44908516	6.14795326	5.35667661	5.6568459	5.74987613	4.98490954	4.84655079	5.18596198	4.97487547

KDM5B	5.94203151	5.68621699	5.75279819	6.45484313	5.81441774	5.72396358	5.68797804	5.93582424	5.63544145	5.86320458	5.76170306	5.97441023	6.01493936
UAP1	5.6236175	6.34948133	5.78039994	7.46118693	6.10610453	5.59630981	6.0367046	6.04338243	5.73016593	5.30351802	5.86203368	5.40608598	5.57400566
CTBS	5.12086778	5.32663063	4.66200737	5.83603143	5.91567408	5.80924383	5.86093328	6.31485475	5.70186502	4.39661135	5.57032408	5.61248316	5.16365141
RGS4	5.64000592	5.98334477	5.6300425	7.21593601	5.54062891	6.38257011	5.58403177	5.13702886	5.96680984	5.10235941	6.14668263	6.30133564	5.54169511
KLHL12	5.40395047	5.87220308	5.0935743	5.4650078	5.43078431	5.61707095	5.40718609	5.03139182	5.46677158	5.50114794	5.20949402	5.9243021	5.77938308
IGSF21	4.31741517	3.81702067	3.91594375	2.93672936	2.49874532	2.38510603	1.79488806	2.6535158	3.44780414	3.11512846	-1.22097981	3.99518693	3.42955725
SSX2IP	4.02225873	4.94028991	4.50642895	5.68131544	4.64059811	5.51144769	5.23548723	4.92689549	5.23834942	4.48379403	5.017343	5.48966311	4.63228699
ZNHIT6	5.67325953	5.43038115	5.62578071	5.79096468	6.01698603	6.24190484	6.19977142	5.79935457	6.19109407	5.45296665	5.88490195	5.78864199	5.32946349
PLA2G2D	1.43950659	0.81934847	-0.12847065	-0.09483908	-0.1305144	-0.1293781	-0.1305144	-0.12790705	3.48854354	-0.01773567	-0.01773567	-0.01773567	-0.01773567
RBBP5	4.48063943	4.45869419	4.10177032	4.44628093	4.86545269	5.04911707	5.26096313	4.8224976	4.47855607	4.10155306	4.76660144	5.06939691	4.36131278
GBP3	5.82701206	6.337599	5.5793477	6.37429634	6.56584786	5.24392317	4.96848539	4.76207172	6.20050449	6.22805233	5.51827378	4.54339509	6.14402582
GBP1	5.10198672	5.60962381	5.05707574	6.55961204	5.86039204	6.31742244	5.22788051	5.52068884	5.83892332	5.51112416	5.69733356	5.37922398	6.50163564
KIF17	0.75890254	0.89209649	0.89084445	1.08882444	1.46698904	0.62669136	1.43055431	1.40108308	1.88033115	3.0302488	1.76704055	3.73217183	3.5495637
CDK18	6.76831126	5.28805769	5.5935998	4.18908764	5.42077246	4.236722	4.50240567	4.07919481	4.07266098	4.96006681	4.45131185	4.02720335	4.73417365
CD160	0.2868032	0.56101155	0.24145681	0.27451838	0.45036892	0.24030557	0.48141918	0.24217181	3.07313003	-0.09261753	-0.09261753	-0.09261753	-0.09261753
ECE1	7.17837447	6.2153039	7.34426245	6.68083737	6.44779282	5.85109914	6.05686284	5.96991501	5.72888115	6.26914243	6.13068618	5.60509387	6.03949195
HMGCL	5.6608083	5.29833571	5.44091201	5.04009735	4.89941903	5.00170886	5.3646722	4.68117554	4.61155867	5.87599312	5.02658778	5.21608105	4.79167687
GALE	3.61990152	3.05890313	4.60488596	3.16974166	2.54299345	2.31742778	1.97899529	2.85930654	2.60654926	4.47603065	2.14411587	2.97171723	3.37708434
ID3	6.97232746	6.41720582	7.05473922	4.8441275	5.62635308	6.57179054	7.17117062	5.96082181	6.21914914	7.77253233	6.86680006	7.24326723	5.72650092
CR2	0.42943782	0.35583473	0.24522823	0.3139151	0.24262538	0.24407253	0.27961346	0.245946	-0.13447687	-0.13447687	-0.13447687	-0.13447687	3.11508748
CD46	4.97127172	6.02661734	5.03828005	5.91951067	6.185842	6.27716494	6.25488582	6.13842399	5.96867186	6.63395612	6.04552754	6.19412739	5.85239062
PRPF3	5.82982103	5.84382139	6.26688983	5.76998427	6.07335392	5.65968817	6.00534186	5.85698084	5.84184735	5.99435133	6.09219747	5.69248649	6.19285988
APH1A	5.25212108	5.57284012	5.34908353	5.34696603	4.71877636	4.18166476	4.63221403	4.81745759	4.78162476	5.40086113	4.92570528	5.33854659	5.34493919
SLC2A1	7.50273911	4.57228939	6.1355231	6.06287255	5.58574937	5.71348948	4.82222649	4.52962578	4.18379458	4.77459475	4.78655034	4.93320952	4.08606929
EBNA1BP2	5.06690166	4.84842262	6.42961571	5.42844135	4.97877755	5.53082138	4.7375229	4.51993986	5.34967956	5.173878	4.59559895	4.69188596	5.00741604
CDC20	1.46078282	0.34975943	4.2689884	4.31425091	-0.30022262	-0.04935164	1.24521764	0.25890158	-0.42347034	3.27516356	-0.42347034	3.77308582	2.09509074
MPL	3.04912852	2.36886114	3.28143702	1.91118062	1.98412312	2.95760901	1.56027431	1.79032057	3.15139358	3.01379334	3.30835953	3.57454153	2.91636123
ARTN	0.0993507	-0.22392972	1.30813736	-0.19849493	0.1079131	0.04956585	0.7603165	1.04808716	-0.09392481	-0.09392481	-0.09392481	-0.09392481	-0.09392481
IPO13	5.12332858	4.60932005	5.69581374	4.49701007	4.53174537	4.8898085	4.63336429	4.37928641	4.69440085	5.26190084	5.14699832	5.29723357	4.27956962
ATP6V0B	4.60045706	4.99928586	4.56070068	4.74756853	3.71945984	3.40297142	4.34833143	3.47823052	4.23560167	4.33302692	3.57653336	4.41036499	4.00016033
B4GALT2	5.12558837	5.52308413	5.80549145	5.50405819	4.75521149	4.06626362	4.53061975	4.68837753	4.64248054	5.46750983	5.38813668	5.58426296	4.13473832
ERI3	4.95279101	4.63039162	5.26457	4.44728983	4.52795435	3.93582614	4.58153405	4.80157329	4.58702024	5.07926495	4.97475749	4.99226824	4.62069763
PTCH2	1.75638012	1.28040158	0.74885106	0.66950677	1.04743785	-0.37313022	0.92544218	0.38882117	0.40782357	4.27897785	-0.05183887	-0.05183887	-0.05183887
AKR1A1	5.45690302	5.92102943	5.92565564	6.05908279	5.08936611	5.09465969	5.34864354	5.10048978	5.67807638	6.02340319	5.79252226	5.84112255	5.2686568
PRDX1	6.59374991	6.73113403	6.67364192	7.1327123	6.70271165	7.05142233	7.37580192	6.6180348	6.83557816	7.35316165	6.9055782	7.2696559	6.98231554
PIK3R3	3.91162581	3.65856282	5.43343836	4.88184045	5.37724156	4.91833469	5.81686026	5.30148691	4.37026331	4.07945806	5.49224964	4.71423883	6.48752276
TSPAN1	1.17948864	0.40782304	0.9829892	-0.63545358	-0.42731173	0.79435429	0.63185422	-0.56947357	-0.05665653	-0.05665653	-0.05665653	-0.05665653	-0.05665653
BLZF1	4.94980135	5.87459505	4.96838827	6.07495419	5.97076846	6.12479586	5.88889531	5.86709066	6.17695024	5.67244807	5.46239578	5.79627921	5.4807522
SLC19A2	4.38456003	4.4393089	3.66739561	4.53633172	4.71792788	4.96541396	4.93743726	6.01346183	4.2242863	2.81135118	3.597332	4.92373749	3.8531078
FAAH	3.52130842	2.54462147	3.36142268	0.29769303	2.89977966	2.56569494	2.94416695	3.02709542	1.86170351	3.50672289	3.66932389	3.59934837	-0.66834334
NSUN4	5.22150327	4.61795023	5.24446172	4.54331589	5.02135433	4.41630245	5.11354023	5.16587291	4.14042092	4.95669331	4.86932575	5.34729876	4.50440224
TMED5	4.98588547	5.4463001	4.94362322	6.7105433	5.98989675	6.31342392	6.5460069	6.11102916	5.63504332	4.52774624	6.03956802	6.0383808	4.35603202
DR1	5.37820934	5.87653842	5.13952804	6.18758924	6.00354547	6.18856533	5.89398382	5.74199489	6.13909847	5.49973509	6.24560978	5.95215731	5.66503925
CNN3	6.45780409	7.47914821	6.15176422	7.51385786	7.80158407	7.72854293	7.02389785	7.64016541	7.65442457	7.27223835	7.86838924	7.53039547	7.62298478
PRRC2C	8.42808894	7.7286079	7.54937478	8.23063512	8.42808894	7.92706275	7.86220611	8.05447948	7.71732841	7.79230969	7.3024501	7.39472725	7.98566836
F3	4.1021304	5.80894136	4.29789633	5.07485883	5.90300364	6.37273	5.11370979	5.97824389	6.66411495	4.83920342	5.90363138	5.84111059	5.66373777
ABCD3	4.8215495	5.60664304	5.04730201	5.86711167	5.64095585	5.95102711	6.02905547	6.02772279	5.93199265	5.57461103	5.85278664	5.71218603	5.47540856

VAMP4	5.34153069	6.19999206	5.0914236	5.98142995	6.72645209	6.82955155	6.78409982	6.7414425	6.50626934	5.28679883	6.66517582	6.8832063	4.8016916
DPH5	4.05757779	4.51130315	4.30714194	4.85076235	3.65849853	4.72159957	4.10314434	3.66253265	4.86618465	4.43044558	3.73354838	3.386918	4.89407434
FASLG	1.37031248	1.31719209	0.66745166	1.02571051	1.2480675	0.8094886	0.57633278	0.49725868	0.21675625	3.43166126	1.809868	2.56210667	-0.27074136
PTBP2	5.01908385	5.30959866	4.64347164	5.94661156	5.88809532	5.72810867	5.88419298	6.30390453	5.59917515	5.49294175	5.66153588	6.23153995	4.94565044
TNFSF4	3.27067419	2.59767147	2.98227216	3.61483696	3.12102251	3.67671604	3.04385574	2.66616768	4.28537399	3.01594401	3.14108067	3.11114363	1.51594243
PRDX6	6.26166883	6.62073299	6.86500288	7.12746442	6.34306432	6.78486718	6.97446978	6.38440589	6.32498284	6.35842274	6.63978675	6.61409017	6.15011552
DARS2	3.96483777	4.05064757	4.62491751	5.25765753	4.45640057	4.67808909	4.28213449	4.64110077	3.5666794	3.83632057	4.46122069	4.19532696	4.49080603
HSD11B1	4.58708431	3.67164226	2.24832906	2.70422905	3.08206136	2.24607072	2.75984046	2.17473883	1.36802509	2.55353024	0.57982287	1.22972577	2.51720343
IRF6	5.53415824	2.07865784	3.80288584	1.68149615	4.53745569	4.20636882	4.0291096	4.54060777	4.59558059	-1.91538166	3.73158449	3.1815206	4.02668505
DIEXF	5.24853983	5.09472714	5.07389606	5.5370741	5.95403954	5.24389415	5.40183819	4.82865738	5.12741102	5.15388869	5.35395795	5.0793006	5.56948183
SERPINC1	0.84429525	0.55367676	0.41350466	1.03717013	1.2846633	0.41236138	0.97925747	0.71107083	-0.31333933	3.58198629	-0.31333933	-0.31333933	3.24668855
RCAN3	3.32269115	3.11113747	3.2736718	3.32804568	3.76414936	2.90117689	3.46273535	2.94146775	3.53021966	3.46303705	3.71280235	3.60028852	4.58435787
SYF2	5.47006432	5.2257527	5.58659973	5.04709461	5.28157896	6.39839825	5.17407172	5.6031003	5.99933888	5.9065013	4.93502691	4.7491377	5.33766709
SLC35A3	4.84027336	5.03705564	5.06937495	5.07512707	5.45657449	5.3748772	5.52258502	5.74036227	5.42767441	5.32582719	5.82017173	5.4400792	5.398594
RCOR3	5.62577245	5.75807071	5.16928196	5.94145782	6.21393803	6.14715467	6.186527	6.46418203	5.90850286	5.48953618	5.86951933	6.01828007	6.07903865
STMN1	0.04143439	0.17169255	2.93955156	3.00668388	0.35870558	1.04948305	1.68836858	1.22434709	0.35530663	0.35530663	0.35530663	0.35530663	4.7910885
MAN1C1	5.72773461	5.47416985	5.67886099	4.43572651	4.09906157	5.11915573	4.99628579	4.77461341	5.7595226	5.35677068	5.07204094	4.92837256	5.03021576
NEK2	0.08906575	0	1.20969723	1.70699029	0.0237395	0.19709668	0.33406778	0.40158361	0	0	0	0	0
RPS6KA1	2.96341107	3.22855539	3.4765548	3.0524345	1.17662041	-0.12819998	0.9603009	0.13175011	-0.23014765	-0.23014765	2.90537026	-0.23014765	3.63436076
DHDDS	5.13550649	4.66797004	5.24221986	5.33290084	4.74948171	4.44239713	4.70004753	4.69709222	4.47874482	5.43756216	5.26101584	4.9859798	5.13848601
NENF	5.50807605	5.56707056	5.69668817	4.62547591	4.98433588	5.52604944	4.04098679	5.31033746	5.28984909	6.54759922	5.15636901	5.78988539	5.34401438
NSL1	5.07659288	5.4494223	5.39971657	5.94699939	5.6688024	5.95716604	5.84604392	5.61017261	5.86653853	5.91714864	5.67495631	5.60623061	5.32849432
PROX1	3.08970237	3.39628875	2.492078	3.98268635	2.70886103	2.55328571	2.4977109	3.05867568	1.89665731	-1.43656155	5.40505651	1.6321427	6.09176245
ARID1A	7.14418508	6.30921748	6.86042765	6.37189319	6.71765701	6.39744576	6.70368088	6.61373877	6.28334669	6.86459555	6.64916521	6.41032048	6.79909966
CENPF	-0.00654162	-0.13407658	2.49956803	2.98991142	-0.29436144	-0.15957574	0.36666833	-0.08786845	-0.23743191	-0.23743191	-0.23743191	-0.23743191	3.57118308
RPA2	5.15245402	5.68109808	5.34786211	5.08394697	4.80058084	5.64554084	4.71780508	4.66389351	5.14164789	5.52175032	4.74107729	4.74454794	5.82545238
PPP1R8	3.17776452	3.27296577	2.70873286	3.61514697	3.27475537	4.06369168	3.15374912	3.22988023	3.93283112	4.67486706	2.87458467	2.76793644	1.26005003
STX12	5.6731238	6.03525689	5.60133078	6.61314538	6.66356434	6.62499792	6.31087438	6.60385456	5.84758809	6.02159066	6.08566083	6.7631673	6.07596642
OSBPL9	3.98699794	5.0814123	4.30176211	5.05059622	5.25606779	5.23648889	5.21157668	5.23812916	5.06833807	5.24816677	4.51532072	5.74168998	4.65917866
TXNDC12	5.9100386	5.68625417	5.78927421	6.72497332	5.71273703	5.89980171	5.62977943	5.63385929	5.96608065	5.59543285	5.39550863	5.8705195	5.78145557
ESYT2	7.59484477	7.74360616	7.3429684	7.22427793	8.03831437	7.99066996	8.01963689	8.27424285	7.72120345	8.1051762	7.87287148	8.1460052	7.78109844
CD3EAP	2.92655946	2.87852958	5.16564326	3.28345262	2.96600539	3.77111973	2.11138513	2.85218323	2.2424832	1.9285638	2.37349785	3.03679105	2.36168429
RCN2	5.10669485	5.61763366	5.86571417	6.09833614	6.19500526	6.66225112	6.34865057	6.64153128	6.65524008	6.28327527	6.74617744	6.49852761	5.78710296
CTSD	8.16171365	7.53924873	8.43524704	7.29199909	7.13862568	6.55864144	6.92672529	6.48893853	7.0786469	7.75365037	7.49583482	7.38849812	6.68309317
COLEC11	3.20628086	2.538221	1.49301548	-0.1507747	1.26820119	1.13600873	-0.14169973	0.65280989	-0.46622524	-0.46622524	1.15530903	-0.46622524	-0.46622524
STAG1	5.90823908	6.37766494	5.98491085	6.67637933	6.58872677	6.65297614	6.62012304	6.78888054	6.66720929	6.64578	6.30796494	6.50713316	6.58991878
STK11	5.19995946	4.6294014	5.43171545	4.56007295	4.4857998	4.51046442	4.78650656	4.7516396	4.5654037	5.18720672	5.16095212	5.05633714	5.05242309
IFT46	5.47883637	4.35978658	5.18945732	5.09241424	5.108356	5.69927454	4.9845518	5.35057623	4.77366757	4.30097866	5.30651883	5.28089475	5.28055043
MMP8	0.32329986	0.75415312	0.10989628	0.74932916	0.40297665	0.10876365	0.15084275	0.11059973	-0.16682865	-0.16682865	-0.16682865	-0.16682865	2.45986248
ZNF541	0.55746606	0.12494782	0.32757453	0.44505415	0.54265145	0.30495484	0.21387869	0.17038559	-0.15884187	3.372225	-0.15884187	-0.15884187	3.13393459
SLC8A2	0.91142142	0.33765648	0.74569399	0.33765648	0.54021407	1.7337521	0.95977629	0.5446595	0.0933229	4.07919197	-0.64305332	-0.64305332	-0.64305332
KPTN	1.87171436	1.11462883	2.30657041	1.49250415	1.35044765	0.3025132	1.19942255	1.77519046	3.30565821	-0.62680238	-0.62680238	2.21026684	-0.62680238
RPS25	6.92001422	7.53153008	6.06942192	6.83202438	6.70306989	7.27357642	6.2824817	6.60298836	7.38636411	7.2680303	6.33963888	6.41285471	6.77042577
KIF14	1.3374071	1.36710295	2.69921034	4.11836873	1.40352043	1.3694845	2.38435975	1.94881701	-0.24949322	-1.3175515	2.04907576	2.9225425	0.08423492
TNNT2	0.96165647	0.90172607	0.90432961	1.18470107	1.26765209	1.20520964	0.9463695	1.22067605	0.48359922	2.94455652	2.64259118	2.44798016	9.75658151
DDX59	4.47987065	4.65231766	4.11229014	4.98163838	5.21673761	5.27316085	5.08267279	5.26561488	5.19876449	5.34933942	4.41976773	4.51511119	4.96473909
CAMSAP2	6.43872899	6.97196628	6.44766462	6.97225671	7.33525948	7.48493232	7.28476874	7.36937868	7.25510573	6.71364711	6.66881389	7.22884589	6.69561473

ATF6	5.48772562	6.11943183	5.40086403	6.31365619	6.18700238	6.24594247	6.03397095	6.02103538	6.0442219	5.91133935	6.12189873	6.06548487	6.16555967
MREG	1.64886282	0.81930439	1.16620653	2.47428721	1.84126498	1.60038882	1.90023415	2.25293344	-0.69265639	-0.69265639	-0.69265639	2.67444234	-0.69265639
FASTKD2	3.81294656	4.77176679	4.55263839	5.64427188	5.23535063	5.28996746	5.31133913	5.35053634	5.06316361	4.73598292	4.78862869	5.40402438	4.31211252
NRP2	6.02364794	5.7177913	5.76868084	6.53575826	5.73496509	5.14072797	4.99719952	5.22897596	5.05215398	5.16714902	6.11887195	4.90667228	6.01139236
CREB1	6.21475393	6.47277265	5.95189811	6.76342685	6.84663717	7.11563833	6.92227301	6.95393292	6.8362905	6.12827633	6.83974907	6.87318441	6.25461277
KLF7	6.05072296	6.56543757	5.63251292	6.48357448	6.89704331	6.65098251	6.52235383	6.58466	6.22896262	6.3331539	5.92415251	6.28493491	7.10506982
B4GALT6	3.46039615	3.55682454	3.48130431	4.41736792	4.13164555	4.93352884	4.04471911	4.09007041	4.61602997	3.14731098	4.57798464	4.17037087	3.66125277
C1orf54	4.70299136	5.17421097	4.12144913	5.27647684	4.40596096	5.3072665	5.02137667	4.61684814	5.17463363	5.16942061	5.18962448	4.94539613	4.17438286
CASC1	0.92068038	1.73647506	1.09656674	0.68820502	1.22365594	2.16650452	1.07392437	1.49370618	2.77668817	-0.63967481	2.41582467	-0.63967481	-0.63967481
LRMP	2.65209129	3.63237037	1.68943737	3.85012942	2.38635426	1.65679791	1.96968492	1.93468556	3.37327882	3.09835074	3.30347036	2.10900243	4.16675959
ATP10B	0.02104238	2.10727592	0.02320036	0.13354639	0.03343718	0.08394837	0.05411053	0.02379546	-0.08057212	-0.08057212	-0.08057212	-0.08057212	-0.08057212
SPCS2	4.94930562	5.0313738	4.40825685	5.39763766	5.24004043	5.75136726	5.03263414	5.18052039	5.29970146	5.47495143	4.15204984	5.11024376	4.84010014
USP35	5.39058081	4.77746651	5.70556125	4.27527832	4.75128001	4.10188081	5.77721218	5.28580428	4.90164935	5.38859081	5.74885492	5.32517835	3.91578868
ELOVL4	3.24729005	2.78387724	3.1521082	3.25546872	3.37126738	4.11445134	4.00538697	4.01816709	4.60323716	3.60616853	3.57015618	4.05486869	4.29222732
FILIP1	5.79738169	6.06440638	5.46486804	4.79767305	6.51238655	7.58732867	6.63113763	7.15687655	6.77024848	7.38499455	5.12199001	7.02729204	7.5190071
CASP8AP2	5.05760966	5.87765018	5.53671794	6.32339215	6.18517949	6.49883073	6.23990732	6.15051312	6.66128375	6.223029	5.61641957	6.04671672	5.8325883
HMGN3	4.1678926	5.09240237	4.0793307	5.01622281	5.21758414	5.52418434	5.75229919	5.40374006	6.03345771	5.30639886	5.24192805	5.48537758	5.71164789
CNR1	2.34586176	2.14062931	2.02142279	1.68662666	3.06231276	5.63978798	3.57579583	3.8428796	3.32793876	2.43414044	1.74326176	3.22713374	1.17047218
ANKRD13C	4.91538564	5.53667219	4.76859838	5.41145154	5.5234244	5.48379148	5.48552299	5.65095095	5.56092677	4.96392388	4.85259677	4.88629149	5.1903991
SGIP1	4.67353602	3.8418577	4.49037998	3.69672506	4.61521597	4.91730251	5.30593682	5.38571762	4.75972176	4.87417951	3.33618268	4.9476789	4.530893
PHF3	6.47741801	7.09283977	6.41339094	7.22195255	7.18317439	7.67851632	7.26498166	7.29991352	7.45173134	6.79434839	6.43327799	6.90958227	6.99012717
PLAGL1	6.54694916	5.42263376	6.41440046	6.37392873	6.11173171	5.95597267	6.2347736	6.96644823	6.43410454	5.87419125	6.8291313	6.31255136	6.54850887
FBXO30	5.42299733	5.62996784	5.3478973	5.98362665	6.12752862	6.31434389	6.28780096	6.36922857	5.76775847	5.69528496	5.66827364	6.1957082	6.20160544
TNFAIP3	5.60116046	5.77515064	5.05684638	7.00179232	4.72190668	4.20449364	4.42845623	4.50613731	4.90628469	4.19414474	4.9270993	3.92493765	5.0844319
AKAP7	2.28018084	3.61497583	2.99186919	4.16301696	3.40809602	3.86444295	3.7457267	3.47809061	4.79665825	3.93412331	3.10630764	3.42836667	3.17493211
RAB32	4.7586852	4.87570541	3.94633174	5.01932773	4.86704925	4.3114031	4.79099073	4.68689685	4.02513685	4.63934534	4.31687906	5.23133995	5.01691958
MYB	0.34932867	0.6268083	0.86485115	1.15751413	0.62732153	0.33202411	0.59689168	0.3338703	2.75220601	-0.3577695	-0.3577695	-0.3577695	3.38036774
ALDH8A1	3.06115967	2.07436254	2.38818179	2.89574688	1.82380435	1.8585421	2.19664811	1.30676771	3.27383947	1.44239838	1.9688526	2.38959753	3.08478808
SGK1	5.74170144	6.41030373	5.1302683	6.53728363	4.21999836	4.55333899	4.61314187	4.77569377	5.4369505	4.99488544	5.26651181	5.31319054	6.37550769
RNF146	5.62339912	5.7641354	5.2291869	5.69973617	5.93428289	6.3698981	5.90416924	6.24263013	6.13168993	6.23883099	5.87739169	6.02866515	5.92766038
CTGF	8.90223436	9.12017216	8.3977562	8.93001932	10.4034952	10.0800617	9.5699853	10.4692607	9.76765318	8.3353805	9.76765318	9.67592183	9.48806534
TCF21	1.76517054	4.93901264	2.49137635	3.53470698	1.46882625	0.80495932	0.70375049	0.98279854	0.89932712	-0.99244262	3.15127629	4.11735815	4.34890224
PMFBP1	0.90082896	0.2845896	0.99695044	0.75592406	0.67138103	0.39080089	0.24810639	0.51321498	-0.23880959	-0.23880959	-0.23880959	-0.23880959	-0.23880959
FBXL5	6.28499862	6.87546245	6.14175925	7.19030949	6.96100753	7.16999774	7.2031921	7.17994086	6.91848871	6.7675534	7.2587821	7.09254702	6.77039072
MED28	5.9358823	5.86922608	5.40252389	5.20377484	5.84902141	5.37500386	6.13236155	5.52492438	5.30623767	5.14173434	5.73461426	5.38374387	5.37666498
SLC16A7	5.90057889	6.08595192	5.42103527	5.92915237	6.68980326	6.89982195	6.32344463	7.05286317	5.69900914	5.62804991	6.19256518	7.11514264	6.41245567
TMEM5	3.4124592	3.74264778	3.58676562	4.07114464	4.18091187	3.95003964	4.15976286	3.42472497	3.82197168	3.11302922	3.78121835	4.01815514	3.99420815
ZNF430	4.03965411	3.78644804	4.28721561	4.81560563	4.30258918	4.72348819	4.52367416	4.62741026	4.9547039	3.99037247	4.14121935	4.18086733	4.12698016
VAMP8	4.683891	5.40706498	4.75247915	5.49770914	3.34317945	2.0107914	3.10873781	2.24119799	4.64518825	2.6449603	1.89965516	3.86483494	4.35075217
DCLRE1B	3.98805513	4.0094041	4.34514212	3.8981749	3.44753707	3.69973419	3.47834621	3.11234776	3.87451497	3.54082294	3.7792696	3.12251857	4.40376272
MYL12B	6.27300414	7.73559202	7.05408867	8.10399594	7.71251511	7.50634308	7.74375203	7.85972429	8.06657786	7.84762866	7.60936186	7.97273208	8.14378479
FOXO3	4.2775521	3.99768296	3.42341346	3.25385858	4.30461992	4.45500609	3.68016245	4.59452958	3.49839375	3.94567331	4.33127379	4.36498997	3.47967718
ARMC2	2.86397537	3.58474486	1.80984937	2.54854199	3.61460332	3.86082555	3.10399703	3.51448727	3.81670776	2.65939609	2.66418114	3.30692182	0.59794321
RPN2	6.91918611	6.67256473	7.20213811	7.43980644	6.44074256	6.66229435	6.56197736	6.36248913	6.82275245	6.71401783	6.83318246	6.67464893	6.77055041
TGIF2	5.19985906	4.99975244	5.44230913	4.56086681	4.29060938	4.04202588	4.80884499	4.64807327	4.15576981	5.32047047	3.96351984	4.54134578	5.11677182
CASQ2	4.37467446	6.78605004	3.6601093	2.73817237	7.23862657	4.68768087	5.50756004	5.40639858	5.00738873	6.88536009	5.80013506	6.61475717	7.76679612
PKD2	7.2839289	7.67277117	7.2055029	7.03172084	8.91513339	8.51887223	8.46445244	9.19366121	8.14231914	8.30759856	8.47170199	8.88628507	8.16564172

ABCG2	2.94740259	3.61853072	2.63830007	3.09133605	4.86280201	3.2246199	2.94460159	4.90259222	4.84366775	4.00582444	5.20571815	4.05002392	4.78360794
SPP1	9.49368657	6.19665295	10.8222791	8.95242419	5.83852683	3.25084151	5.5924191	6.01541974	6.34802561	6.16099514	4.64032697	3.93413819	6.41924144
STBD1	4.86164838	4.42533479	4.86611226	4.28719551	4.61763769	4.87542705	4.72400065	4.95138744	4.54718724	5.06799222	4.65887008	4.50512992	5.38507242
CCNI	8.12967429	8.4475709	7.91084363	8.28581994	9.06016289	8.38983702	9.06016289	8.9067877	8.19738523	8.63463414	8.30420084	8.64378978	8.07914893
RARRES1	4.2437803	6.07417078	1.50415132	4.20804191	2.55849828	1.98092624	3.22492972	1.86741834	2.00662074	2.02369784	0.94677633	3.19921189	1.01445197
MFSD1	5.64812889	6.38339953	5.70121138	7.01343933	5.80556317	5.61551069	6.12276266	5.90327894	6.24049449	5.59315776	5.91824327	5.76916368	6.09174253
RAB3GAP2	5.72908985	6.16273972	5.40769637	6.2345732	6.13992585	6.31723723	6.23941294	6.2848413	5.91730723	5.73170652	6.16184307	6.09075197	5.49772079
PPL	6.28998092	4.95434245	5.78799837	3.5255313	3.90917527	4.95850458	5.60058137	6.10870674	5.07620954	5.90408977	6.07239836	5.34938971	5.71951104
UBN1	5.93752139	5.83318987	6.25921604	5.64331333	5.80879111	5.28615921	5.67691532	5.47361686	5.60158036	5.93523042	5.96132478	5.36848968	5.73756185
KLF12	5.69714488	6.02595568	5.42176691	5.88653693	6.5963699	6.92636334	6.45262891	6.87071614	6.56266867	6.75552717	6.47352877	6.64679226	6.1933117
UCHL3	3.58606149	4.35580654	3.57691296	4.94199643	4.16256649	4.40268949	4.52958839	4.77068378	4.63437819	3.51822843	4.43845093	4.57821195	4.42308585
PCDH17	1.39711648	3.08125656	1.97786559	5.33242073	3.39576338	2.51068959	3.09758971	3.23450191	5.18824681	4.21669796	5.79855474	4.75518856	5.28436515
HS1BP3	4.67250308	4.90012061	4.8354244	3.75913383	4.98660769	5.08890605	4.99445055	4.90144466	5.45754375	5.26096741	4.57362193	5.21542285	4.72319846
WDR35	4.62002994	5.34948365	4.54511568	5.47268094	5.26091815	5.63536212	5.30007997	5.29562505	5.15907846	5.07978727	5.40569093	5.32003682	4.71127665
CCND2	8.21849693	8.58833402	8.20774182	7.65392522	8.20379893	8.19820233	9.01556072	8.52203702	8.12007387	8.07784832	8.30921349	8.70535086	8.22640219
ELL2	8.92083607	6.11254347	7.73474788	8.81636689	7.86609947	7.27627957	7.51088333	7.19326105	6.50511916	6.25954607	6.19742501	6.91748323	6.21330218
DNAH7	2.82446713	3.56561685	2.95435657	3.04124839	2.78028226	2.4511965	3.16235328	3.13623838	3.29358564	-1.42326541	3.8241867	2.698743	2.29352315
CYP20A1	6.08745131	6.31065656	5.79455849	6.12385792	6.40371448	6.10062855	6.16465272	6.12663164	6.27172488	5.8317723	6.28172607	6.12859974	6.15152999
NDUFB3	4.15820132	5.23708324	3.81742105	4.6399112	4.54903011	4.95998453	4.46569898	4.0074297	4.40285248	5.06507714	4.15006792	4.40029647	4.66169442
GTF3C3	4.69268779	5.1652322	4.91654144	5.78526893	5.39458684	5.50648883	5.39862828	5.55964663	5.5659012	4.99494129	5.31832534	4.95929092	5.41260208
SATB2	4.75362542	3.30041109	4.06516227	4.11286547	5.13894991	3.50678656	5.23779904	4.89357071	4.29340169	4.99922564	4.83729657	4.15677644	5.60928862
UBE2B	5.3210948	5.70727058	4.99763797	6.01889248	6.20193392	6.04703899	6.39477111	6.22699546	5.67513914	5.40055562	5.51567073	5.67798184	5.45944769
TRPM6	1.4358134	1.91002324	1.19546024	1.28919287	1.67752589	1.20747922	1.81958633	1.25568921	3.05186631	3.48387682	2.28303092	2.05172051	4.8481533
KLF9	6.73784687	6.98811428	6.65241049	6.18309513	7.35498961	8.11799039	7.52116424	7.88518291	6.44163052	6.5225487	6.65074511	6.90135358	6.78570796
TJP2	7.11089101	7.142197	7.25546004	5.92416262	6.83147259	7.39154715	6.82144711	6.39770852	6.79819899	6.62485206	6.69285294	6.0301459	6.65946697
C2orf40	5.08794855	6.38007099	4.51827588	4.08393467	3.72530068	5.32324213	3.52539168	3.38243947	6.44447042	3.99055715	5.11933354	4.31157614	5.42759927
ITGB1BP1	5.17044659	5.93838312	4.7815816	4.9589004	5.31381022	5.29039799	5.22149537	5.14631594	5.63831189	5.4446152	4.55390361	5.4962852	5.03801247
CPSF3	4.45589945	4.67293307	4.76363167	5.45953148	5.00731293	5.21149826	4.79219303	5.09874602	4.88983952	4.39404834	5.04809034	4.90777569	5.05407025
PIGZ	3.21843258	2.68337969	3.81391502	1.48622388	3.00702691	1.97174822	1.74853462	3.42189636	3.69927294	2.93253132	3.6843758	4.27343592	4.26140744
SENP5	5.69051911	6.06947702	5.61489564	6.45043188	6.33206713	6.07967244	6.39784086	6.44579925	5.87378323	6.23599505	6.21049476	6.38339697	6.05470678
CCDC92	6.06101795	5.20605668	5.90411637	4.60944496	5.96326553	5.24693616	5.45492739	5.80241311	5.95687068	6.57361725	5.63682331	5.36035604	6.10334609
C1orf198	7.15694671	8.4370212	7.04763894	6.71725706	7.93915301	7.62911925	7.38995268	7.29424496	7.68921094	7.58228399	8.22154623	7.81423489	7.49309226
HEATR1	5.67159855	6.15425581	5.600994	7.05000925	6.0038803	6.28557866	6.28452273	5.66008452	5.78115308	5.46674763	6.11087142	5.48717704	5.91408981
PTBP3	6.28885008	6.79467202	5.92433982	7.31732691	6.718763	6.29389837	6.49480853	6.11129333	6.62080858	5.56506242	6.88911686	6.04889366	5.98460666
RAD23B	6.71893165	6.48134138	6.49407694	7.02476737	6.58033459	6.68636923	6.31223365	5.98695461	6.40116329	6.48400005	6.43815415	6.28081449	6.37495881
FKBP15	5.77648438	5.55823394	5.82556052	5.75329109	5.26659269	5.36995051	5.24373856	5.0914198	5.29576032	5.39921858	5.59880605	5.50053273	5.94776445
CTNNAL1	4.71135309	6.22476479	5.45405496	6.0068771	6.20773001	6.27696774	6.26524715	5.92558666	6.51257613	5.75623229	6.24184592	5.4989262	6.55715356
FAM206A	3.75289432	3.77657383	3.61798431	4.82845256	4.82617936	5.04954883	4.64440348	4.42531202	4.86832417	5.17114384	4.11098798	3.93129251	4.30985669
WDR34	3.40763692	3.23952733	4.61904921	3.49502038	2.93864476	2.69697063	3.25772075	2.45824992	3.60550139	4.24653577	2.78412036	3.29726536	2.46879102
SET	6.37156982	7.4840738	7.19882892	7.90092053	7.28397811	7.13929952	7.81576605	7.19576066	7.13250609	6.9852735	6.98898971	6.82297057	6.98993381
GLE1	5.316812	4.94927946	5.63349576	5.37680867	5.03613376	5.51174462	5.44047019	5.08517481	5.31311301	5.24108754	5.09469716	5.49186355	5.40038471
RAB14	6.23054306	6.79851917	6.18923082	7.00363749	6.85985394	6.90411689	7.13281204	6.88917297	6.9339637	6.49621706	7.05650803	6.87839126	6.6603872
CNTRL	5.34053367	5.18682893	5.44619265	5.80875516	5.49580925	6.00869179	5.12181854	5.57320595	5.79435223	4.74107132	5.30994214	5.13891937	5.49092113
TRIM32	5.6535301	5.45275398	4.97955535	5.12416929	5.51707909	5.85391335	5.38260229	5.66424991	5.14355149	5.51036079	5.84788208	5.55970925	5.13737226
FBXW2	6.36341087	6.74752528	6.43203011	6.61590416	6.87088248	6.67486581	7.06763125	6.9685702	6.61191683	6.61607317	6.80324251	6.76654021	6.8457576
PHF19	5.75803976	5.54827263	6.16614696	4.91634987	5.38023612	5.40714471	5.79723488	5.94963577	5.53927865	6.2658373	5.59178483	6.13155869	4.97088751
NEK6	6.76831491	6.01785594	6.76569225	6.91081495	5.53051292	4.50289739	5.34853013	4.75574297	5.16041819	5.60689058	5.75201118	4.98665204	5.40639118

BSPRY	0.70293092	0.62491802	0.36711205	0.23160642	0.34891025	0.04101635	0.18142578	0.04285781	-0.13296661	-0.13296661	-0.13296661	-0.13296661	-0.13296661
PPP6C	4.92667772	5.65699301	4.94542729	5.69487883	6.11626033	6.00827494	5.66265472	5.97804875	6.0955454	5.62522321	5.55462847	5.85423341	5.25801925
NDUFA8	4.57208532	4.71906753	3.97992787	4.68920857	3.82780996	4.55180184	3.77270561	3.41235953	4.76371439	4.92514776	4.50945151	5.07231087	4.69709445
HDHD3	4.71624096	3.50671554	4.39695625	1.60437712	3.33205639	3.08601448	3.7003213	2.72491662	3.53837003	5.22056012	4.73760213	3.99291125	-1.01115413
RBM18	4.75247037	4.45902155	4.09291283	4.98089848	4.96555509	5.43908687	5.23985112	4.85399682	5.18623534	3.96765611	5.15268558	5.25475159	4.59330015
SLC46A2	0.70088067	0.27028223	0.77377221	1.42668031	0.17859542	0.13910505	0.44479599	0.14090374	-0.24239603	-0.24239603	-0.24239603	-0.24239603	1.90136526
HSDL2	4.49881188	4.64799073	3.87424315	4.77295233	5.27433531	5.89626067	5.22302988	4.99413038	5.71761755	5.08931379	5.01540142	5.2744393	6.02645981
MAPKAP1	5.64746519	6.05914151	5.80792602	5.89137398	5.50981971	5.21435212	5.36770715	5.14592462	5.41473186	5.56531643	5.51988345	5.10664549	5.76714439
NR4A3	3.80451948	3.88875892	3.77609844	6.56296137	3.87867812	5.21563015	3.96521321	3.52966666	5.09381907	2.83898055	5.81582281	5.822424	5.65580469
INVS	3.82027206	3.93815709	2.72573362	3.48000314	4.18928185	3.84339988	3.8854189	3.87763291	3.67506266	3.02371253	3.96825069	3.49888582	3.38648912
GALNT12	4.02624204	3.59972158	2.54686635	3.86198873	3.07514474	3.64476933	3.91999091	3.89670816	3.69816811	4.18483759	4.44475973	2.00837366	4.05686555
DENND1A	4.75763348	4.28806783	4.50990777	3.61830934	4.03325414	3.29204873	4.21575525	3.67556195	2.56869532	4.30583768	4.24191656	3.93147502	3.30222528
ALG2	4.95498652	4.77408148	4.74079885	5.92830605	4.70131488	4.69274226	4.64620612	4.98734229	4.36592074	3.84385169	5.00703828	4.95591251	4.63530463
CSF3R	5.61148008	3.35355046	4.18568022	4.51252894	2.88290405	1.48154355	2.08371181	2.13688397	3.64818237	3.59181505	-1.65428297	3.37933117	4.30106375
KDSR	6.02788328	6.0603352	6.02389406	5.93276541	6.47139522	6.34081709	6.58078833	6.8961519	6.02874726	6.20192701	6.20242062	6.39507476	6.04348957
VPS4B	5.68928888	5.76867202	5.79258609	5.76180444	6.10395149	6.43606706	6.2623507	6.25679586	6.156262	6.06582868	5.8541093	6.29922604	5.59493551
ONECUT2	0.87824779	0.87032159	0.96043293	0.89276826	0.89205946	1.1594768	0.87032159	0.94960924	2.80543528	-0.5247048	-0.5247048	3.13828234	2.28757061
C19orf25	4.10270905	3.53296644	4.98609552	3.36175698	3.47654749	3.15921821	3.97341623	3.97041161	3.37560467	4.26936453	3.82984881	3.9344542	3.1684763
ZBTB45	4.43015671	3.60463407	4.73454645	2.35937342	3.52337329	3.47061155	3.32443904	2.92076099	3.32474499	5.04457909	3.78981268	4.89949283	2.84003434
YLPM1	6.93159341	6.49850547	6.67358629	6.86923281	7.04650535	6.43967267	6.62015395	6.69973859	6.66564564	6.69854494	6.65279281	6.34279332	7.10365976
DCAF4	2.59630422	1.63544379	2.2390716	1.37811569	1.7913593	1.81707649	1.69365954	2.15689274	4.73527634	3.44321247	-0.21134608	-0.21134608	2.23808023
FCF1	5.11710541	5.41553639	5.64128649	5.75434539	5.66625713	5.47157103	5.67972607	5.1262776	5.36662983	5.40716161	5.14228224	5.17237253	5.29636129
PGF	3.72240895	4.39898411	5.14759326	3.95433171	4.28181313	2.59171889	1.64799976	1.62361014	2.30110194	3.56295959	2.49281067	-1.65308342	2.84659578
IFI27L2	4.11050249	3.97868813	5.26266731	3.82040466	2.81718874	4.15334855	3.36835873	2.85431875	4.23176297	5.33069034	4.35650875	3.08095146	2.73364288
NEK9	6.50704572	6.66773547	6.32712348	6.26889235	6.48296585	6.40332811	6.78390464	6.42393032	6.28270293	6.46343111	6.57104641	6.45695263	7.02013627
ACYP1	1.81925097	3.84351683	1.2604123	3.16733037	3.01485597	1.70961434	2.98767061	2.46487296	3.35078595	3.73353211	2.87769805	-0.53266092	0.75741203
IFT43	3.81416405	3.19221487	4.06716799	3.25967873	3.66327374	3.72622319	2.81588388	3.88034473	3.15907174	3.18861968	2.92012677	4.48482641	3.59191742
NPC2	5.42660112	5.15664999	4.74263477	5.29538856	4.26092341	4.54875548	3.52938959	4.16282329	4.86502084	4.36902572	4.98770665	4.36902572	5.39876314
DNAL1	4.52869038	5.31152984	4.42293792	5.4123425	5.4655614	5.41768362	5.4295948	5.49444126	5.40186527	4.88962284	5.64902261	5.08075577	4.93471921
IRF2BPL	5.57735675	5.38646778	5.71362564	4.15572389	4.46626942	4.73534784	3.99319541	4.13972865	4.09992813	4.9736645	3.87284733	4.8927599	4.25795264
LTBP2	9.74098868	8.99704109	10.7496647	8.96791333	8.85603286	9.20500321	9.02341036	10.0288514	9.28358417	10.0067535	10.0067535	10.3317905	9.56224649
MLH3	5.42460034	6.10862312	5.39818773	5.95586384	6.00227177	6.19331186	6.09641444	5.92104169	5.67950246	5.79164051	6.1246869	6.1377361	5.36886443
TTLL5	5.4874356	5.44883815	4.69529217	5.69555249	5.33673199	5.00945763	5.19861703	5.12388319	5.1452277	4.74026518	5.04473853	4.59884647	5.61564363
FLVCR2	2.94098288	2.72792904	2.67667409	2.98294101	1.48169873	0.20933724	0.71880508	0.81169137	-0.79364551	1.72321956	1.8493077	1.47967831	-0.79364551
ABCD4	5.0761215	4.86284812	4.97099532	4.6593344	5.03084494	3.93584936	5.31573817	5.15114482	4.8054541	4.70531585	5.02291161	5.11845767	5.04264413
DLST	5.98722534	5.58262994	6.04341104	5.65836814	5.792157	5.69508601	5.84376001	6.21488874	5.80036393	6.07341025	6.00222143	5.23258112	6.34930408
PPP4R4	2.87200135	2.36420556	2.37479307	2.38812694	3.20659883	3.76303437	2.84908417	3.69990355	2.35006427	2.81081727	3.07274376	3.15078737	1.84135719
TGFB3	5.68732544	5.93735197	6.00283394	7.17162322	6.31663003	5.49547481	5.76441112	5.72124937	6.10671684	6.58978735	6.23735272	6.7970103	5.51238604
SLIRP	4.34466016	3.73115912	4.99066059	4.99297442	4.66144613	5.21648818	4.09947949	3.86626848	4.51249266	4.25756087	4.03358979	3.85491234	4.92934962
RBM25	1.31573025	2.72354584	2.1454926	4.09251134	3.611385	3.5198459	3.40734111	3.91497965	3.54030876	-0.36020892	-0.36020892	3.82166677	3.58571925
ALDH6A1	5.97215182	5.6363052	5.52017317	4.72814087	5.9394849	6.62315176	6.18568123	6.42540869	6.1308304	6.45980306	5.81908976	6.34535241	6.11850206
GPR68	0.76166098	1.27781715	0.78363734	3.44130486	0.74074716	-0.13233946	0.83936017	-0.14036817	2.21930265	-0.43745528	3.06284312	-0.43745528	2.5323648
ESRRB	0.8683845	0.5435675	0.86653018	0.73973876	0.96969478	0.51997626	0.92934875	0.39189965	0.87010225	-0.31034735	-0.31034735	-0.31034735	5.08077928
EIF2B2	5.63241855	4.48993331	5.34259921	4.8586392	4.87099029	5.81928673	4.44043616	4.65210558	4.26152653	5.37666484	4.6147931	4.50214025	5.32249715
COQ6	4.70849651	4.75002367	4.66874657	4.13734985	4.6521022	3.99013279	4.56438394	3.84537172	4.48693066	3.79123871	4.29643117	4.67484959	5.02566128
ZNF410	5.05541989	4.4746739	4.98940581	5.12122075	5.27020432	4.75427977	5.24421798	5.73715314	4.79522956	5.50886761	5.33488731	5.20780764	5.16964258
RHOQ	5.35902104	6.04530916	5.04871758	6.20677283	6.4813116	6.36984162	6.56468899	6.46635679	6.43099927	5.57568166	6.12299782	6.39513291	5.23498815

GPR75	3.79301336	4.17671641	3.9495178	3.71502162	3.89009572	3.01212564	2.81964519	3.14013475	3.47697792	3.38595048	3.11259247	3.70707202	4.71702021
SUPT7L	5.88987551	5.57903457	5.47154188	5.83921254	6.1933052	6.06496843	5.65778664	5.7579966	5.6837885	5.72740474	5.65947699	5.62984075	6.11286815
KLHL29	1.15832	0.22056693	1.2582989	-0.56353364	1.24064475	0.69705843	0.74749076	0.7062441	0.37846253	0.37846253	0.37846253	0.37846253	0.37846253
DNMT3A	5.79181305	5.37384716	5.78824301	5.24964233	5.24641713	5.28127341	5.53912659	5.55663899	5.5511591	5.760504	5.42408394	5.28496772	5.71983348
TMEM214	6.07537813	5.96922582	6.13864299	6.3018132	5.73860526	5.19640527	5.61318472	5.8688867	5.85119406	6.15312952	6.04872909	5.89660995	5.89470835
ATAD2B	4.91282076	5.37464436	5.18198642	5.99782758	5.7251386	5.78217674	5.73026272	5.83597809	5.715001	5.09607946	5.83051234	5.47855082	5.10052618
FKBP1B	2.3283438	1.86544106	2.06024135	2.65472632	2.17428431	1.49587881	3.15558604	2.27575185	2.56912909	3.73001528	-1.02136548	3.2650918	1.67625417
ATL2	5.07021019	5.3747323	5.13253738	5.7173434	5.8124453	6.33911045	6.39848166	5.94853089	5.60029616	5.37747207	5.56299603	5.9746724	5.11592611
YPEL5	6.07660972	6.44130192	5.73419599	6.2089203	6.72664938	7.04751832	6.73987151	6.44080353	6.81123437	6.2249903	6.40991569	6.76028534	6.327816
FAM98A	5.52840099	5.4302993	5.65715693	6.63294421	6.12206335	6.28640161	6.07029269	5.68768433	5.85084001	6.06015448	6.25762254	5.43835587	5.22774722
YIPF4	4.88824085	5.06114584	4.61680492	5.05168076	5.21169058	5.16410394	5.57816294	5.66395393	5.40563644	4.80508416	5.81225175	5.64912329	4.92291976
AFTPH	1.04167903	2.55872639	1.04560357	2.33001554	2.69743262	3.63903744	2.80937112	2.73702935	-0.2541094	-0.2541094	3.40787414	4.08922487	3.535476
LGALSL	5.146659	5.13929867	5.11493587	5.1632267	6.07140612	6.17969926	5.98463429	6.05735991	6.00389183	4.59122347	5.84665262	5.555928	5.24420034
CNRIP1	5.71762305	5.64048392	4.7975486	5.57983137	5.12974472	5.65909287	5.28069007	5.44217134	5.26518786	4.78418595	5.37656647	5.30418447	5.1615727
BCL11A	0.80472008	0.93413565	0.97657483	1.29950746	0.42068435	0.58370749	0.62834184	0.55658107	0.79346423	-0.38321361	-0.38321361	-0.38321361	3.48396814
CRIPT	4.48674675	4.82252516	4.69255856	5.86445436	5.76163496	5.77466449	5.94395801	6.06068327	5.17645199	4.13825251	5.32312336	5.40015403	4.43270906
EPCAM	0.35862918	0.31214329	0.31472386	0.82330293	0.31214329	0.51443554	0.40487907	1.12346692	-0.57971107	3.76398293	-0.57971107	3.57119036	0.641983
SLC17A5	4.76194394	5.34414604	4.38819789	5.04409517	5.08333568	4.89369803	5.16319322	5.55170471	5.60097088	5.16941623	5.90218996	5.06846973	5.21135275
OGFRL1	5.4253682	6.59130106	5.22864998	6.74907204	6.00194266	6.12804118	6.2752201	6.49583371	6.74643132	5.52463614	6.34188072	6.2921855	5.96455031
IDE	5.08071998	5.29678576	5.09085952	5.87296845	5.74687615	5.98163623	5.71142804	5.71255009	5.77395072	5.19980701	5.88886066	5.55514539	5.57919732
ELOVL3	0.00289371	0.00289371	0.481616	0.00289371	0.63609195	0.59209766	0.11644006	0.00608762	-0.11283492	-0.11283492	-0.11283492	-0.11283492	-0.11283492
IFIT3	5.44508611	5.83567019	5.39959372	6.61348915	4.7256604	5.2088322	3.96676465	3.72883348	6.10265553	3.8587053	5.34945977	4.0270578	6.74373559
IFIT2	5.10668813	6.0574522	4.91391228	6.5187558	5.08690725	5.23638652	4.39715615	4.14292724	5.97732788	4.79611557	5.17172869	4.35175062	6.29120947
GPAM	4.53835471	5.25314593	4.39335553	5.52813656	5.65790325	6.96120615	5.54362026	5.14191519	5.10368797	5.1226095	5.7140422	5.60133613	5.80673895
CUTC	4.27122043	4.33239871	4.27576456	4.00951173	5.1004975	4.94464314	4.69286201	4.89847306	4.03207085	3.77585925	3.5460519	4.52221416	4.37682193
PPP1R3C	7.57670032	7.91533207	7.30281996	6.79805357	7.89505383	8.39599475	8.50947392	7.15354709	8.12741336	8.61869265	8.36036272	7.82120258	7.55563539
PYROXD2	3.21856923	3.39615882	2.97203668	2.09631287	3.52038489	3.10760972	3.42751757	3.12021076	2.89877314	4.48103055	3.63065858	4.27943042	3.53775769
CNNM1	2.3050798	2.26155074	1.87584198	1.47788108	2.75981343	2.50284433	2.43881925	2.84316345	2.44614408	1.38679965	2.41184523	3.35899134	3.30904745
MXI1	6.47772777	6.30432535	5.91085184	5.35907311	6.96528976	7.08352324	6.76268305	7.05864781	6.60020944	6.49380116	6.03600741	6.28241094	6.06656208
SMNDC1	4.37235941	4.88502063	4.58938391	5.41225187	5.69573986	5.63759627	5.72167079	5.54509105	5.75668156	4.89816347	5.33397961	5.74363582	4.52333205
C10orf88	2.89243774	2.74802103	3.25372529	3.74888205	3.78134455	4.1708363	3.83935669	3.92077582	3.37985142	3.21386531	4.55453319	2.97031392	4.31006767
HELLS	3.28641139	3.5019594	4.50123403	5.85639598	3.78486947	3.67107159	3.83040828	3.78009517	3.49298567	3.042481	4.61222394	3.63834099	4.25512461
TCTN3	5.07679662	5.7962965	5.05322549	5.73578104	5.33232988	5.28242093	5.20203564	4.38650266	5.29999098	4.97533813	5.25332283	5.15275395	5.01371087
FAM45A	4.11231512	4.26290335	3.8491293	4.69051771	3.87565171	4.26777556	4.52297144	4.58991906	4.63056329	4.39510363	4.72319647	4.07066348	4.59992215
AVPI1	3.9958034	3.65315281	4.01811414	3.0577124	3.44122427	5.21445207	3.25898819	3.56745881	3.4671847	4.69726263	3.37484875	4.74648628	3.64158379
WDR11	5.8161655	6.32202196	5.46810665	6.39872679	6.33714473	5.83294046	6.45899943	6.35986337	5.81831596	5.61758202	6.56030972	6.23872197	6.0664815
C10orf76	5.49005778	4.81378001	4.62839828	5.00673584	5.23435246	4.40901039	4.84772913	4.77027786	3.89360006	4.529948	4.2791321	4.46716829	4.85913928
KCNIP2	3.46484715	3.08647485	3.30045417	2.37225092	3.16351676	1.80370653	3.43129627	2.59571172	2.62263309	3.61062911	2.21912905	2.75813731	5.83558979
GOT1	3.58423164	4.34563599	4.79526165	4.57637703	3.98306199	4.23466584	3.94748064	4.34449448	3.78236801	3.22180261	4.18318087	4.5759737	6.73743998
SFRP5	0.66263922	0.98687591	2.90762199	0.62292001	0.14126444	0.49430436	0.38590543	-0.13124427	1.83342051	3.86152315	2.47235437	-0.00943718	1.99502197
GNA13	5.71266756	6.38690189	5.81363011	6.92163829	6.60822584	6.71899683	6.74400137	6.4955735	6.44925707	5.90396328	6.66535976	6.52672596	5.94213347
HOXB5	0.2792254	0.44252166	0.34807251	0.66158632	0.77560277	3.00337005	0.0732414	0.33336888	1.95369508	-0.14557691	1.68570415	-0.14557691	1.7690276
HOXB3	2.65432051	5.00911423	3.90744367	3.55219179	2.24026934	4.89335426	2.62160147	1.53351235	4.28193496	4.2638418	3.68649329	2.7473781	4.44272237
DUSP1	6.71440422	7.40923503	6.75466816	6.92596635	7.9919609	9.2062652	8.52727036	8.64151653	7.57153982	8.08164098	7.69549445	8.9014076	7.92934699
PANK3	4.64904324	5.31166265	4.6554521	5.68723239	5.64217204	5.63918669	5.7397548	5.79180912	5.45493727	4.89420703	5.38783184	5.89378317	5.44991844
MSX2	2.43882039	1.78806499	3.32132021	2.57447967	1.36747335	1.26929569	1.9367243	1.3036912	2.58209161	3.84874433	1.05061156	2.93478984	1.19847308
TEK	2.06020238	4.87685357	1.89146259	4.18871506	5.19909258	4.68443282	3.89552385	4.54315227	5.34541705	4.77510158	5.60404724	4.61494555	6.05009114

RCL1	3.15620573	4.33636168	2.66586345	2.05919551	3.18336651	3.972604	3.62446714	4.13126763	3.14297643	4.18340355	2.69050852	3.5257491	3.76722971
MOB3B	5.34639745	5.79938658	4.92840236	4.69807129	5.5669143	6.09699483	4.45863252	5.00016251	5.83368722	5.17196538	6.07333825	4.81432001	5.1387377
CD274	4.14850127	3.32793588	3.22102456	4.58583442	4.46327852	5.15813942	4.11032427	3.52739721	4.08067989	3.55966456	3.49589682	3.44688099	3.47341054
GRIA2	5.94476968	5.82397167	5.7543304	4.2999803	6.93174885	7.51065993	7.75857283	6.46065051	6.74292306	6.43860686	7.28360685	6.45555977	6.9553234
NUP43	5.54875124	5.16248226	5.66502803	5.82279219	5.36934726	5.37318998	5.83736779	5.75615059	5.34543307	5.07417506	5.36926736	5.43916042	5.7029107
MTHFD1L	5.24141553	6.02882889	5.42042921	5.5750166	5.72410221	3.97051989	5.16826027	4.86268596	5.05997688	5.15338567	5.46179353	5.45516409	4.96371257
LRP11	5.5569641	6.34279186	5.60908873	5.65441539	5.7004104	5.8155951	5.11713551	4.83369569	5.62457401	5.32842619	6.04637439	5.85891265	5.61455501
PCMT1	4.56262591	4.44870297	4.53434678	5.08642226	5.03271656	5.55264356	5.32471993	5.13501806	5.19894668	5.3180988	5.28853125	5.23420249	4.72739358
PLEKHG1	3.68100327	4.98381373	3.79316428	4.78323523	4.18490067	3.69655461	3.32632698	3.96370978	4.05450333	4.39067531	4.16527628	3.89703582	5.14299197
MYCT1	0.01383595	2.50671288	0.34221336	3.64412743	3.17074672	1.93483347	2.45621676	2.06367842	3.45172158	3.99314605	4.34850205	-0.15554152	3.36571072
CXorf21	2.15325895	4.14523732	3.37073107	4.48062886	1.95633041	0.96004822	2.24550658	1.59772244	4.34747234	-1.17203525	3.00539525	-1.17203525	4.11760746
WDR55	5.35017612	4.71897556	5.56197675	5.14538085	4.80296819	4.29154724	4.96930867	4.94337725	4.96424903	5.29692775	4.69021848	4.74587059	5.26681016
ARAP3	4.95552914	4.23985643	5.02992339	4.65267531	4.81845222	3.33951769	4.443491	4.43674093	4.2641375	4.33840619	4.59392879	4.36971034	5.42028397
PCDHB8	4.5594252	3.60970766	3.90247023	2.43007107	3.93799852	3.98222846	3.57811733	3.5384619	4.00162405	2.93621699	3.70064207	3.62644883	4.06144551
PCDHB10	5.46777065	3.92911265	4.18380856	3.71587077	4.00188282	4.5145794	4.34799148	4.43816548	3.30570921	4.94012084	3.82442273	4.1930795	4.40791487
PCDHB14	5.48462116	4.36204271	5.25799019	4.15799886	5.02748785	5.58193955	4.88880451	5.69373844	4.66112396	5.07661268	3.8009259	4.48785555	5.34423373
PCDHB12	5.04839762	4.19660843	4.35389424	3.40907583	3.94684605	4.43539802	4.09176225	4.77344623	3.91650897	3.34218279	4.36173159	4.58804422	4.12986746
SLC25A2	1.07230466	1.5491468	0.4346595	0.77163894	1.28049998	-0.24052771	0.98166223	1.06335394	1.24524954	-0.29821945	-0.29821945	-0.29821945	-0.29821945
TNN	1.10447696	1.23102044	1.14519745	1.60765808	1.30044372	1.09724653	0.96048128	1.12438608	1.54747145	-0.64586603	-0.64586603	2.99078738	3.97868605
MRPS14	4.77813761	5.5507255	4.30588566	5.28274671	5.39353991	5.71632396	5.28547287	5.22661572	5.76852062	5.07289964	4.97708267	5.14827426	5.41746729
CENPL	2.55662593	2.63157721	3.78819943	4.86956514	3.32361074	3.17981316	3.64511346	3.66164273	3.62519975	2.96889911	2.6347217	3.94335646	3.21066576
TNFSF18	0.93048463	1.28088638	1.21371905	0.90136477	0.64808333	0.29355268	0.2921454	0.29537454	-0.34312795	-0.34312795	-0.34312795	-0.34312795	0.6879461
SEC16B	3.6235661	3.55119214	3.50472235	4.26667526	3.68790582	3.99421797	3.60075852	3.81769532	4.2141175	4.20549398	4.37344239	3.5081119	3.78901586
GORAB	4.54066502	3.623134	4.57712675	5.24199306	4.21075336	4.94273871	4.46875387	4.50747212	4.80744316	3.70392112	4.53767992	3.72239241	4.31768102
ACAT2	4.26064186	4.46784065	4.08511073	3.99381372	3.99252164	4.80755098	4.7060734	5.12687919	4.85033296	4.98266141	4.65517988	4.61489153	4.41708509
TCP1	5.77857756	5.8264735	5.66076695	6.63606112	6.47629981	6.62552064	6.34315437	6.19048183	6.0463218	6.22355675	5.38010701	6.03872821	6.19950651
SNX19	6.70054141	6.82677718	6.55531975	6.56221996	6.86896514	6.3576075	7.01273288	6.88570431	6.57420661	6.64196897	7.10763302	6.97609886	6.97222711
KCNJ5	3.78797626	1.44251574	1.69995751	1.00192855	0.96036511	-0.22540043	0.9007433	-0.38959994	0.01496437	3.99780241	2.22836151	4.26726838	0.01496437
TP53AIP1	0.04772095	0.04772095	0.05027543	0.48766753	0.07925006	0.0491412	0.04772095	0.22899266	-0.03303773	-0.03303773	-0.03303773	-0.03303773	-0.03303773
PDZD11	4.76801334	4.58363568	4.70705463	4.90498706	4.09043954	5.31708349	4.1463005	4.61893883	5.02801031	4.86273989	4.10007022	5.07958617	4.96211148
SLC10A7	3.76916157	4.44444904	3.5639637	5.27271752	4.35605351	4.73975272	4.64945384	4.7121164	3.84143846	3.63029004	4.64738398	3.88744834	3.71591533
NUDCD1	3.60815931	4.75352784	4.39329693	5.45908245	5.11423641	5.35475659	4.89491484	5.15502846	5.25289322	3.65599346	4.86430747	4.2046424	4.12047574
ENY2	4.27494502	4.85401944	4.40839801	5.26062258	5.46176509	5.59447755	4.98172914	5.51092141	5.80903309	4.61441386	5.2033612	5.40663912	4.84649846
MASTL	4.49108802	4.23978755	4.07676763	4.80386937	4.36973026	4.54923169	4.52643709	5.13348142	4.61284024	4.07077631	4.24569321	4.73180705	4.66970599
KIAA1217	1.02269235	1.53192009	0.99464672	1.92915073	0.85267302	1.59115439	0.04467131	-0.51762821	0.54824807	0.54824807	0.54824807	0.54824807	0.54824807
PLXDC2	8.19323459	8.32473233	7.8098994	8.16863683	8.51496047	8.55122224	8.07871319	8.89114805	8.37546722	7.97617629	8.51192873	8.51192873	8.19384942
EPC1	5.75008227	5.8800992	5.13979961	5.94808125	6.00601007	6.00041467	6.0630845	6.21604852	6.03136256	5.89174917	6.19933224	6.03254166	6.13173873
IQSEC3	1.23511291	1.32002632	0.65557306	0.45778422	1.24064131	0.11001298	0.43187071	1.1889879	2.89743984	-0.34533331	1.96810977	3.45544841	2.07314219
CCDC77	3.4747252	3.56225869	3.25702239	4.1876064	3.08555322	4.10294558	3.83584279	3.41817994	2.94583296	3.890113	3.5374273	3.74127822	3.61120364
TAF12	5.06415732	4.06168572	4.66710946	5.13733388	4.38710147	4.88617303	4.05446674	4.62253647	4.94200835	4.16345529	4.72266791	3.86684238	5.16785264
ENOX1	0.76106375	2.00717566	0.78179542	2.20535485	0.63861021	2.18659205	0.73220814	2.90860867	-0.04654179	4.62380528	1.47093588	2.56228389	1.21932733
MTRF1	3.76003847	4.61054568	3.50633321	3.92987294	4.13012622	4.22710078	4.9566469	4.1693201	3.8346825	4.3306572	3.66032894	4.39130659	4.12729106
DNAJC15	4.80321114	5.39089988	4.57899394	5.3674378	5.8857729	5.92115733	5.78522347	5.89032759	5.99716176	5.62487191	5.14171887	5.6151138	4.80407094
PROSER1	5.49315028	4.83124993	4.92719751	5.61552562	5.41146971	5.2415545	5.06159372	5.1102945	5.05512178	4.06736575	5.20056568	4.92396188	5.62147686
UFM1	5.4644369	6.340556	5.24588886	6.67068242	6.25803136	6.54104067	6.62716143	6.42900371	6.93546547	5.46322922	6.10607613	5.97212498	5.79723923
WBP4	4.29822773	4.50498257	4.56577052	4.44219153	5.02008537	5.40817632	4.87311169	5.19946897	5.3794515	4.69390401	4.61586665	4.22537466	4.82602639
ELF1	5.94891857	6.38196046	5.6896878	6.48617324	6.37257073	6.38658161	6.59706665	6.86151826	6.23540967	6.16855596	6.24542265	6.57756122	6.74582643

SMAD9	6.65729284	5.62821305	5.8256105	5.18104585	6.62669808	6.75737025	7.24591274	6.64389931	6.30082243	7.02327828	6.02899588	6.44593337	5.98647079
HSPH1	5.12212917	5.50251127	5.89100322	6.77365926	6.77445776	6.69556448	6.3838003	5.53868912	5.9673752	5.59951666	5.57533121	6.23088705	5.67501849
KBTBD7	4.29873391	3.83918458	4.31037764	3.73541004	4.61072228	5.4722472	4.48533101	4.57311334	5.07740358	5.00233945	5.05764946	5.2532421	4.13139969
ALG5	3.47970031	4.99192102	4.29784805	5.17302174	4.48854057	4.24373217	4.69511682	4.76014157	4.87152449	4.79826097	4.22445713	4.66360794	3.99722092
EXOSC8	3.76581356	3.78299709	4.48024192	4.82336681	4.34799763	4.88081732	4.52650041	4.82201734	4.40569569	4.11402926	2.95365507	4.38240039	4.65407513
ETF1	6.47882315	7.07959179	6.7064943	7.89003358	7.25910834	7.45919962	7.4849018	6.94265823	7.04731996	7.08676338	6.79546913	6.76442498	7.31170968
TGFBI	7.18400674	6.64158686	7.17087808	8.88348567	6.41864903	6.01478783	6.15205933	5.41052041	5.63363754	6.27948197	5.86778762	6.63035202	6.32806394
FAM53C	5.53592482	5.17765939	5.79778599	4.99419431	5.16368338	5.10821252	5.94288772	5.13338568	5.2252411	4.71972873	5.80045299	5.71251859	5.73846897
SIL1	4.9389187	4.73487375	5.69990287	4.90336455	4.13453434	4.60285792	4.82639637	4.3947831	4.96202736	5.70753219	4.44096468	4.86236731	4.47406333
PAIP2	5.79112251	6.26051172	5.9422141	6.79191078	6.94791158	7.14644973	6.99143416	6.76928632	6.57276446	6.7910007	6.2067003	6.41089293	6.40777503
MYOT	2.1001901	1.96142597	2.48212797	1.96142597	2.9759718	4.28804858	2.63635303	6.18683192	4.25066067	-1.27955488	2.88921637	4.03599168	6.45750898
KDM3B	7.51486051	7.30081816	7.09162088	6.14678903	6.98895625	6.54639947	6.992208	6.9675158	6.42649048	7.09757611	6.84440539	6.7490962	7.29297451
EGR1	8.63117145	7.74472442	7.86392855	8.3964574	6.84800617	6.79300937	5.84069988	6.04353919	7.05065738	6.56577029	6.20084363	6.45748676	7.83657613
SERP1	5.507055	6.50964143	5.90459926	7.36684376	6.69297061	6.82448021	6.64969737	6.49987769	6.65733991	6.1098353	6.69662019	6.75148499	5.19864384
PLS1	1.77744779	1.81583765	1.41928557	1.73793566	2.48915435	3.67226975	2.73767945	2.6148143	1.47725198	-1.02935213	2.69405548	3.7877854	0.00770375
ZFP30	3.91620503	4.93935517	4.71848042	4.29449141	4.72250406	5.54450664	5.37839569	5.03792081	5.25982249	4.63565869	4.9179786	5.28716145	2.94589368
NR2C1	4.69139394	4.90688231	4.36594936	5.38126375	5.36861715	5.22333329	5.53977352	5.69876303	4.98741993	4.62470238	5.59304524	5.22084933	5.01435956
UTP20	4.70140244	5.85460516	5.31131799	6.24143589	5.68893227	5.7634186	5.71936891	5.3025789	5.20278743	4.69465109	5.11829866	5.45981702	5.52060583
TMPO	5.05926586	5.47293807	6.10973267	6.57311534	5.68497076	5.47169457	5.65443438	5.96226728	6.15135782	4.90627912	5.88332751	5.66555549	5.60677981
ARL1	5.85311052	6.80418334	5.86828493	6.8729469	6.71737401	7.12397223	6.7693222	6.59574118	6.97755941	7.06901728	7.2112936	7.00644824	6.27000074
GLT8D2	6.05984676	6.3270926	5.533396	6.47649856	5.60368865	6.24545777	6.48828876	6.40585378	6.7026883	6.48459948	6.48335594	6.7026883	6.06910996
SOCS2	4.08487879	4.77014788	3.73439131	3.73188275	4.82810348	4.89721001	4.97567329	5.12849248	4.46041671	3.87933413	4.46787807	4.01019496	4.71033137
NFYB	5.83085748	6.06302071	5.39047788	5.28109916	6.28343065	6.93474331	6.2786731	6.43750292	6.30797017	5.7086949	5.87866847	5.91847419	4.58235011
APAF1	4.63043473	4.93044368	4.96100251	6.20433467	5.15185358	4.88818633	5.10440926	5.39843902	5.1930049	4.51421717	5.6551262	5.46380504	5.28855924
DUSP4	2.78985707	2.67678314	3.39610327	3.7943698	3.18660551	3.06910657	2.71909438	2.72048602	3.85295888	2.02986537	4.99296484	2.90257995	3.96496573
CLU	11.2216546	8.83680958	9.92900299	8.51055847	8.79610465	9.07770613	9.34574191	8.82786037	8.9982737	8.71557136	9.23295076	8.87749598	8.74189015
TNFRSF10B	5.58620169	5.84307115	6.2133892	6.01668168	5.61763426	4.65525669	5.96515104	5.62216327	5.38910083	5.02650688	5.51354892	5.62198704	5.98831419
SORBS3	6.21092122	6.51463168	6.59127391	5.23451479	5.9370569	5.56518987	6.44111581	5.9278434	5.98398929	7.04850551	6.62141985	6.47037148	5.72454946
PTK2B	4.55382039	4.66711225	4.42276682	4.26257591	3.86576871	3.06357962	3.78514778	3.30325699	4.04590162	4.32390559	3.78941853	3.79969244	3.50017861
ADRA1A	0.23004037	1.32584982	-0.01308798	0.75435062	1.12832622	1.98047417	2.9814128	1.99240254	1.66902142	-0.45874658	2.53215914	-0.45874658	-0.45874658
PPP3CC	3.5987361	4.84242433	3.7130741	4.48008267	4.38589492	4.74895807	4.52257341	4.51787641	4.44762896	4.3918271	4.0577582	4.11047996	3.72362015
PDLIM2	4.96013657	5.17520166	5.54777062	3.99345646	4.10850461	3.8721451	4.30942948	4.95457284	4.35434635	5.34043456	5.57018016	5.5638018	3.80422407
EPHX2	5.34054055	4.5079387	4.71071838	3.72222935	4.26552553	4.62014914	4.34180564	4.51328981	4.51675317	5.10483492	3.77743435	4.69087019	5.08616949
RNF170	4.18997048	4.74657587	4.01174474	4.53120826	4.72555304	4.50581721	4.49686543	5.22347117	4.62218966	4.24899649	5.14687958	4.82974989	4.13617213
UBIAD1	4.57836305	3.49623556	4.15140224	3.03495301	3.76459756	3.81975329	3.56733584	3.61138207	3.79190389	3.2219474	4.47141695	3.60573006	2.8458795
TARDBP	6.06148276	6.18776551	5.86664317	6.72848528	6.78572303	6.71043623	6.80551455	6.62973509	6.44399528	6.29323042	6.59451712	6.48527496	6.03255291
TNFRSF8	-0.09870613	3.1260368	0.38488799	0.65267901	0.10379246	0.00511262	-0.17675017	-0.00064928	-0.21536751	-0.21536751	1.3506738	-0.21536751	2.21939888
ZNF706	4.52248473	5.19833877	4.76144459	5.12129674	5.50964819	5.39533333	5.72363082	5.72300279	5.38334006	4.69091906	5.70047356	5.20913521	5.20628192
LYPLA1	4.36900682	5.14836599	4.40960833	6.1641942	5.40047395	5.11410189	5.53995844	5.32660115	4.85061126	4.51937315	5.7814382	5.17440567	5.36436873
CRISPLD1	6.0788558	6.66094189	6.84846589	6.7562423	6.66094189	8.19060462	6.83912574	7.41878302	8.33426121	7.83196426	8.04475235	8.24979419	7.48305737
COPS5	4.53048603	3.97104608	4.28564363	4.56561172	4.88680272	5.00885011	4.71454189	5.13069409	4.24717674	5.28839832	4.13925258	4.9658844	4.92720348
RDH10	4.15931788	4.03270908	3.68560396	5.00619548	3.86428708	2.92008593	4.01056635	3.50897856	4.3139472	3.72911317	4.66971497	3.359956	4.20700229
AKAP1	7.29623686	6.59615043	7.07079991	5.32536841	6.87826362	6.97395627	7.24177991	6.9786667	6.09347872	7.43997459	6.19035624	6.86341069	7.3975845
COIL	4.71214727	4.52696586	4.95573144	4.84184811	4.58261354	5.57780767	4.39229086	4.65888937	4.74431442	4.14935997	4.36466164	4.50960724	4.8510974
TRIM25	5.42243416	5.42269089	4.96930577	5.33734223	4.56966523	4.25067262	5.0714142	4.44603944	5.06140518	4.24591368	5.32581386	4.87771304	5.27822209
SCPEP1	6.70904243	6.12819672	6.28748262	5.73936711	5.83706725	6.56564735	6.06334581	6.10054885	6.32044568	6.7287825	6.02075898	6.3861498	6.29816797
SPOP	6.08532743	6.44013674	6.05345095	6.15196244	7.17926894	6.52614507	6.95397853	7.19271927	6.54702872	6.65551772	6.37232456	7.10760966	6.48414873

TBX2	5.48537941	5.0416999	5.60203241	5.05894637	5.01832574	3.80561544	4.87756136	4.9689678	4.29486855	4.88054075	5.34283728	4.97179738	4.64978512
SLC35B1	4.80512477	4.85705646	5.08909184	5.18367891	4.375656	4.16537378	4.24912834	4.34457169	4.54909799	3.78287651	4.40145807	4.24102319	4.771807
TBX4	1.31346634	1.0685156	1.15951553	0.94860149	1.27934444	1.32684118	1.63050444	2.41297571	1.82411132	2.28921929	2.63331758	1.98672082	2.53854134
TEX14	0.96617524	0.54735286	0.9058801	0.41437659	0.91715264	1.646592	1.4505319	1.20876294	2.6843007	-0.33556972	-0.33556972	2.71986162	2.50596041
FAM117A	4.92800097	4.39791682	4.80024419	2.75406652	4.51736812	4.87698453	4.45554879	4.61983122	5.09934705	5.47857634	4.83503242	4.50963221	4.48687406
NCAPH	1.69369192	0.85442509	3.8115167	4.26795045	1.12475252	1.20606742	1.89908394	1.40542086	3.13818901	2.92016943	1.54783939	1.07495719	-1.09308826
LRAT	0.63451506	1.1572953	0.62382591	0.64081719	0.6403236	0.87747669	0.62123271	0.62454102	3.70449562	-0.37880063	1.56479645	2.63508994	-0.37880063
MND1	1.53080587	0.54019983	1.31690224	2.66411828	1.41474544	1.09735783	1.73490704	1.33417945	2.46922852	1.62387549	3.59389787	-0.91066275	0.86905231
TRIM6	2.43931031	2.35964561	3.94224369	3.14948942	2.75894116	4.14171132	2.85268002	3.18060196	3.4192705	1.74488515	2.35761428	3.23893702	3.24376315
ABCC11	0.05781567	0.00808971	0.28798191	0.02915411	0.00808971	0.1019034	0.14523002	0.18608225	2.85921299	-0.06609856	-0.06609856	-0.06609856	-0.06609856
PAPD5	4.79386028	5.06794532	4.5130397	4.75207621	5.60284454	6.34733142	6.1399178	5.45768586	5.6273829	5.15638969	5.35127237	5.5411045	5.21969341
ADCY7	2.68003877	3.62477694	3.34655961	3.65451414	1.9440554	0.83061674	2.14994584	1.46768502	2.90090946	3.48401031	3.34236403	2.94421104	3.81735952
CEP89	4.38873283	4.38374364	4.88784996	4.29524242	4.49876396	4.15823584	4.34057269	4.35869055	5.00602587	4.24337959	4.41616113	3.67393207	4.81217815
TSHZ3	4.97900652	4.12854211	4.87233837	4.30655653	4.74662681	4.69291496	4.58479169	4.68860686	4.53256407	4.28595686	4.80707424	4.48962217	5.10269992
ECHDC2	5.76995472	5.59678473	5.47253889	4.58375259	5.81166103	5.08342985	5.95045262	6.0866577	5.24535493	5.73970637	5.68569772	5.16362209	5.87267658
PLBD1	4.32292653	4.82524953	3.78053646	5.50993368	3.42838805	3.6559661	3.90851199	3.04092972	4.70596992	3.72526085	4.52673479	4.42421216	4.89215116
TAS2R10	0.83385047	1.51106155	1.27897111	2.69998513	1.72259247	3.12103717	2.07059172	2.8613724	0.73845297	1.69336444	2.54604464	3.37438103	-0.80389835
PYROXD1	2.85123772	3.15115346	2.4171214	3.64951433	4.10116869	4.41328987	3.02710191	4.04634601	3.97831125	2.18272451	1.46283961	3.52168707	3.3960395
KCNJ8	4.83679234	3.78096066	2.89336561	3.73055307	3.63024674	5.22766594	4.74246253	4.28265921	5.08818637	4.23735503	4.03143807	4.87155921	5.24019104
TAS2R9	0.19236088	0.88348277	0.08556717	0.15066863	0.0830471	0.08444822	0.0830471	0.31250581	-0.09332039	-0.09332039	-0.09332039	-0.09332039	-0.09332039
PSPC1	5.25085431	5.37035881	5.72375427	5.75411463	5.85528435	5.34795086	5.3353196	6.3302879	5.72159871	5.05820382	6.03484406	5.52148485	5.78810259
ZNF549	4.42735233	3.89585374	4.16678797	4.17924608	4.95335846	4.75007236	4.86586048	4.60400385	4.700842	5.09417083	4.19782815	4.50274108	4.84550128
A1BG	0.61073687	0.17672448	1.64868605	0.73157611	0.38930433	1.09414707	0.69767936	0.84114458	1.16223397	-0.35534601	-0.35534601	3.84179777	-0.35534601
ZSCAN18	5.65114941	4.77578105	6.12186996	3.99706383	5.18501878	5.37332082	4.92712761	5.36528313	5.0254748	6.23013352	5.44314941	5.95015406	5.14092164
ZNF211	4.6187474	5.09382594	3.98206901	4.10418343	4.63291879	4.53866611	4.22585799	4.74388904	4.32367695	4.70729154	4.09467199	4.9632011	4.74471013
PDZRN3	5.6761872	5.78047601	5.49359406	4.97545312	6.46822607	7.06747771	6.75719134	7.04756419	6.15884695	7.00204165	6.44943373	7.09192507	6.49958923
LHX4	0	0.07365352	0.04170772	0	0	0.00069205	0	0.09223956	0	0	0	0	0
RNF2	1.23967678	1.57602574	0.7306645	2.80015419	2.34912584	3.17691465	2.76586664	3.02654806	2.59547007	3.92601358	2.28930456	2.83443752	0.79989823
TRMT1L	5.19610143	5.55521391	4.88860599	5.4078701	5.81748696	6.37511061	5.73405922	5.11260336	5.92124159	5.56487157	4.87881701	5.40461847	5.33553986
SEC22A	3.92376276	4.44223852	3.95806239	4.92583147	4.65698349	4.5612911	4.93679567	4.84547051	4.28810127	4.0270557	5.59659053	5.23941527	4.07687548
CSTA	1.99451075	0.81917615	2.25710325	3.64269592	0.89212809	1.51809217	1.04403648	1.17370315	2.88729979	-0.92752583	-0.92752583	-0.92752583	1.65455947
DPPA4	0.09376442	0.32671002	0.09635045	0.10895885	0.26550611	0.09520221	0.09376442	0.09706359	-0.07234791	-0.07234791	-0.07234791	-0.07234791	-0.07234791
POPDC2	3.07297237	3.2266419	3.4069369	2.8970779	4.99555351	3.30988343	2.91078172	4.80421428	3.60113466	3.49601676	2.52672265	2.12459712	6.82564432
B4GALT4	5.24845827	5.51411716	5.07424229	5.83670387	5.62301008	5.80870929	5.64315817	5.71737348	5.75998243	5.41046659	5.74768529	5.31946764	5.5109851
NAA50	5.65938369	6.40115621	6.16599604	7.25820554	6.84464221	6.90539987	6.9818925	6.70084801	6.42386617	5.63486439	6.59210118	6.39932584	5.82730794
CD80	0.7394944	2.15339446	1.00429463	2.96700107	0.67553874	0.37393102	0.45712747	0.94076253	4.05141855	-0.21074253	3.05854831	-0.21074253	1.4410605
KIF18A	0.0885399	0.07324678	1.26390048	2.87069638	-0.02477144	0.5163385	0.32668192	0.68160178	-0.33099983	-0.33099983	2.95698649	-0.33099983	-0.33099983
MAPK8IP1	4.35756076	2.83932937	4.23444214	2.7824187	3.10310934	2.93220723	2.98170485	2.76367251	3.37794184	3.69583198	4.03541787	3.55458046	0.79600221
CRY2	6.55952325	6.15175726	6.24693467	4.71492096	5.87175155	5.91535853	5.75530001	5.46285802	5.84945707	6.69311801	6.1466733	6.90641165	6.16769246
PEX16	4.10086766	3.92298209	4.46417569	2.88136876	3.16522739	2.73648264	3.75198236	3.75580924	2.56805293	4.67338114	4.09471035	4.42576236	2.85963868
DEPDC7	1.02910284	1.6996084	1.4924903	3.60815052	1.43217446	2.3608508	1.71523217	2.62073035	2.58279347	3.12432877	3.08395174	3.05604856	1.89380201
CAT	6.09423019	6.14244293	5.13760562	5.77167691	5.22862455	6.40841836	5.67484305	5.03514142	5.63930038	5.97297807	5.68795091	6.25743604	6.48075222
PILRB	4.03591405	4.57524856	4.96450521	3.6895136	4.20658815	3.26783854	4.64654568	4.25006038	4.04239673	4.59641454	4.5934006	4.34396637	4.23118088
ZMYM2	5.6620285	6.3917213	5.96311171	6.85176971	6.64489559	6.85248092	6.65431786	6.87130227	6.57359222	6.25147842	6.61547928	6.64378099	6.23893083
GJA3	1.82543032	3.65903011	1.93662888	1.61775154	2.06990624	1.54806416	1.63230965	1.63166861	1.57786287	-1.13253534	3.81409019	2.5233483	3.30070169
TBC1D15	1.95301931	3.30769821	2.44171938	4.14237224	4.11338117	4.20463778	3.69495325	4.03154835	3.95826549	4.36648273	4.20541685	1.23296194	1.47450001
HCRTR1	-0.12746732	0.08520458	0.20470748	-0.22545829	1.96180143	0.54294184	-0.03127126	0.19540244	0.74698857	-0.00894557	-0.00894557	3.97999744	-0.00894557

ZCCHC17	5.12255107	5.27240727	5.25428896	5.126088	4.6052009	5.0211283	4.69157107	4.54093965	5.38049458	5.53670542	3.82743217	4.67640382	5.21961593
FABP3	4.27240234	4.61807999	3.05801849	2.30389011	5.44353244	6.97903582	3.72875084	2.59873367	5.02031811	5.52523962	4.33435526	4.09060868	8.37256806
KHDRBS1	6.91944826	6.98869473	7.32681347	7.461394	6.70726799	6.91252863	6.99414398	6.85902006	6.63007855	6.83281742	6.76338746	6.90184391	7.07119455
TMEM39B	5.41531315	3.41256181	4.84498032	3.49522186	3.04346998	3.10327416	3.73136447	3.26636076	3.52903256	2.51564124	3.44371699	3.97230905	2.39916398
CCRL2	4.11627046	1.24152741	3.53735404	3.04011008	1.75035773	1.30843145	1.46708362	1.30602473	3.70784002	2.67418781	2.75277642	1.805827	2.04812018
CCR2	2.63964057	5.0497455	2.8696638	5.24656812	2.15676914	1.74517824	3.24404617	1.98546357	4.51398872	2.02749216	3.35788262	3.63150514	4.4700773
POLR3GL	0.46116752	-0.74635663	0.37403205	1.14733613	0.84671487	1.81385081	1.20938152	0.96541379	-0.02907449	2.41205224	-0.02907449	2.85915242	-0.02907449
TNFSF10	4.87827073	5.75315105	3.9509796	6.84517682	4.87117381	3.19970523	4.38285944	4.22525855	5.52630119	4.47103833	5.61038502	4.01742724	6.213912
ZNF639	4.60465963	4.93365393	4.88734356	5.24296748	5.53995844	5.43506036	5.71642606	5.53363721	5.41104271	4.27131014	5.54262055	5.42343671	5.14892453
SLITRK3	2.45553912	2.43839565	2.11791944	2.43574571	3.18758946	2.38669799	2.58820221	3.11318188	3.4913164	3.39737675	2.80467159	2.87217307	3.85419496
PIK3CA	5.30410529	5.92093881	5.42225508	5.8777592	6.29230489	6.57004337	6.37726429	6.60995371	6.26989512	5.70371216	6.00624203	6.59033682	5.9094964
PDS5A	6.04010904	6.7033818	6.26586069	7.07316903	6.86648629	6.92761892	6.91208209	6.80106276	6.72443708	6.54688601	6.83275694	6.74833581	6.87433496
TMEM156	0.95819237	2.0081848	0.38226473	1.16165469	0.64689813	0.38117629	0.37981338	0.99608773	3.20500845	-0.38756553	2.53628748	1.80035393	1.78686308
LIAS	3.5660518	4.38884775	3.81664631	3.21091543	2.91306431	3.66848416	3.85331441	3.52132274	3.29307318	3.78878492	4.48022605	3.22974976	3.33366805
CPXM2	8.28618202	7.9715353	8.17803768	6.80676517	8.11272932	7.8776126	7.71704125	7.64727741	8.20409218	8.36053948	8.16289363	8.02242127	7.26789226
TMEM54	4.89741163	3.70893946	4.5414411	2.68030785	3.38992061	3.09676479	2.57847718	3.40787491	3.37745618	3.08679648	2.25351883	3.15889453	2.06485005
ZSCAN20	4.87441619	4.37381463	4.95768956	4.13086441	4.81989952	5.21154758	5.04826362	4.67690291	4.5233089	4.80260314	4.74954518	4.97872813	5.12076288
CSMD2	3.35629032	1.70828735	4.0118603	4.12171104	2.65286525	2.87553132	2.93328269	4.0306813	2.18838996	2.72057565	2.52738046	3.44669534	3.27182027
HPCA	0.34437159	-0.0238702	0.42827849	0.01589178	0.08365501	0.18955621	0.47980383	0.40991194	0.06053393	0.06053393	0.06053393	0.06053393	0.06053393
LRIF1	3.88249235	3.71394835	3.7970473	4.47121775	4.7582542	5.79228038	4.34054747	4.01150801	4.5232724	3.38843615	4.81446574	3.76023558	2.99596615
CLCC1	5.9301479	5.69354093	5.92117573	5.52488689	6.17926258	6.10056223	6.05132902	6.13529019	6.3914949	5.57925737	6.09106722	6.04317703	5.98597584
GPSM2	3.39350282	3.39483089	4.22771896	4.56269172	3.8656703	4.01131435	4.07556535	4.29521167	3.87625019	3.71572996	4.1122365	4.48979467	3.86350637
GTDC1	4.910016	5.37792502	5.004211	5.41638628	5.4924698	5.67001729	5.28445074	5.61976961	5.61684004	5.62200244	5.40546921	5.37994122	5.61352588
CXCR4	5.85036099	4.78231361	4.40485372	6.18099428	4.41722443	3.59750546	2.95657113	3.08591777	3.31414932	3.69463679	2.81908341	4.22970452	4.95533972
ZRANB3	3.66122711	3.98521889	4.11344228	4.43968445	3.71733215	4.41964338	3.77979207	3.651208	4.32139611	3.61593732	3.57328802	4.48404896	4.31140956
ACVR2A	4.69087005	5.26659803	4.6983859	5.65015775	5.09488456	5.74015577	5.25562808	5.60823105	5.39633286	5.37529307	5.49169871	5.29380188	5.14972018
POLK	5.60033415	5.96773045	5.74233059	6.33644454	6.62172275	6.89098225	6.54888532	6.66322138	6.69511885	6.04823829	6.14943441	6.71270904	5.37321749
SV2C	0.00901096	0	0.00124472	0.01859599	0.01143644	0.00069205	0	0.05006034	0	0	0	0	0
FLT3	0.40242585	1.572629	0.40493778	1.08611973	0.68036028	1.32762717	1.07509679	2.05549336	0.69920986	-0.53318345	-0.53318345	3.62493845	2.77120582
RPL21	2.866995	3.64554389	3.59140433	4.51288058	3.81023917	3.15688572	4.11363043	3.83280377	3.60936809	4.30695912	2.73308421	4.22619956	2.85112691
MTIF3	5.20089814	4.94198393	4.97629717	5.43022079	5.40626232	5.73478401	4.94029944	4.749266	5.65220549	5.67233862	5.42232937	4.86511222	5.34609053
GTF3A	5.0717404	5.60469372	5.72923816	5.63327552	5.1319349	4.79282837	5.13216555	4.47391568	5.56969818	5.72854027	4.99088892	4.75327679	5.63178241
RASL11A	4.81389985	3.90049669	4.25289687	2.25851193	3.75085144	4.75294917	3.62349478	3.57947547	4.33105321	4.11961593	3.97836169	3.91251533	4.04733242
UBL3	5.17760878	5.79123554	5.2164474	5.35855059	5.71718265	6.05711399	5.63835246	5.62797703	5.69727719	5.76799194	5.75903357	5.73719271	5.35464236
FYTTD1	6.09323135	6.40986711	5.91958386	6.5730535	6.57223313	7.23572227	6.69975925	6.56542574	6.58259239	6.55830676	6.59481543	6.26945705	6.24797536
XPNPEP2	1.69483551	1.9036495	0.58227773	1.44054068	2.88133348	0.54680258	0.6864477	1.4332322	-0.5106103	3.21498982	2.94401867	-0.5106103	3.26602014
SASH3	4.3653386	3.98133188	5.36647234	4.36198642	2.6065113	1.90369057	2.3241415	1.91738325	4.21859423	3.31664312	1.19694671	2.98571375	2.40709889
OCRL	5.60654632	5.80752943	5.49158714	5.57536658	6.149456	6.22009867	6.12220958	6.12247651	5.8876676	6.23494639	6.27149544	6.03651151	6.21593682
PAEP	-0.00475628	0.24847008	-0.00259166	0.88924509	-0.00475628	-0.00355278	-0.00475628	-0.00199473	0.00616726	0.00616726	0.00616726	0.00616726	0.00616726
MRPS2	4.9891267	4.07153277	4.77192794	3.88049736	3.66293949	3.40629102	3.67670128	3.42258211	3.8848848	3.26859786	2.22164135	4.84976321	3.64075872
TBX22	0.18393367	0.26002214	0.091507	0.14347507	0.22503598	0.98216698	0.22661642	0.89611193	-0.18481102	-0.18481102	2.02008228	-0.18481102	1.18712029
FMOD	9.16852676	7.77437884	8.07983001	6.67571716	8.36098025	8.4666709	8.01708819	8.01708819	8.29042385	8.56893329	8.47052412	8.95662989	7.90410329
LAX1	2.75753753	2.79292918	1.42439172	2.45023025	1.85536471	1.0880722	1.36787533	1.05870791	1.43837884	-0.70783808	3.36567323	3.0582306	3.55041272
KIAA1191	6.66623716	6.3836504	6.05299661	5.9147829	6.83622497	6.81839564	6.30110964	6.47449115	6.39893009	6.9117681	6.6019682	6.71210061	6.71306554
COPA	7.44817411	7.36993041	7.20136747	8.05452347	7.28643799	7.10915493	7.27492809	7.27492809	7.1675563	7.3752806	7.60941354	7.37674569	7.57677708
CD244	0.96424808	3.1049446	0.68568344	1.83410606	1.02549988	0.1706959	0.51519861	0.06509655	-0.43387581	3.0133862	3.57267703	-0.43387581	3.58536045
LY9	2.05882696	1.00651513	0.60117908	1.08765455	0.70834895	0.19535091	0.49377986	0.31954684	-0.3027772	-0.3027772	-0.3027772	-0.3027772	-0.3027772

HS3ST2	4.7724551	0.71816343	3.27051467	0.54446082	1.75085896	3.56619752	1.81567752	4.27381806	-0.96347485	3.51755086	-0.96347485	3.02393884	3.70063264
RBBP6	5.69335524	5.59038621	5.58915329	6.12539378	5.94816816	6.5165885	5.90574448	5.95796071	6.23681974	5.76941503	5.3893431	5.7923023	5.56836712
ZC3H7A	6.06242471	6.51181378	5.92912295	6.44068951	6.63459114	6.50497322	6.76581693	6.7868683	6.87072993	6.7296141	6.39750516	6.49791172	6.51411043
SERAC1	4.23154171	4.72653153	3.78513662	4.31433517	5.04946705	5.17979532	5.09036796	5.0872442	4.56990523	4.96798883	5.21669759	5.58022828	5.19846514
ANXA11	6.77003463	7.21144772	6.84012352	5.86556512	6.92196185	7.10867988	6.98135178	7.15305707	7.10902484	7.33869124	6.91370814	7.23636288	7.57221231
LDB3	5.82396147	6.55401671	5.79948183	3.64403719	7.16450239	7.06975282	7.32433173	7.34085087	6.84467406	7.74305074	6.56235531	7.56727958	8.85713279
FAM35A	4.43464147	5.43378434	4.6144749	5.99347149	5.47683575	5.39996398	5.5329973	5.68956321	5.72991714	4.50449835	5.39346773	5.43301171	4.54463074
ZNF205	4.18247695	3.17618998	4.84451243	2.61034325	3.08491322	3.04593369	2.90998246	2.52331687	3.14326963	4.34209487	3.5203703	3.91535892	3.54821223
NAA60	5.49526364	4.90969636	5.1231841	4.25812903	5.10695455	4.2967574	4.95142287	4.80160473	5.45067636	5.74817852	4.36502226	5.0927163	4.66732716
RPL5	7.87953498	8.33375684	7.51455046	8.2202038	7.60520945	7.66918542	7.88634316	7.66611036	7.86667523	8.38219298	7.70391117	7.9679293	7.80552013
ODF2L	3.70956197	4.06351031	4.45159653	5.60273277	4.28206968	3.8691835	4.64227322	4.93241703	4.74303512	3.09934775	5.10371418	3.63721877	4.19829961
PTGFR	1.37726742	3.41593092	1.0589589	4.17575581	2.17247049	1.82029972	3.11932619	3.32463973	5.12984205	3.80242181	3.25218653	4.60290152	4.99428519
LRRC39	2.16801406	1.38045685	1.17828701	1.61893972	2.26980711	2.48053331	1.71070791	2.21153336	-0.21969585	1.54901021	2.32666573	-0.88457722	5.79576758
RWDD3	3.28638635	4.58146672	3.39553534	4.69438564	4.84399849	4.33521302	4.64693655	4.73663899	4.43553075	3.18425859	4.0040687	4.19531318	3.77505713
ZNF644	5.80766751	6.57745766	5.95302858	6.98614808	6.9959816	7.18494429	6.92994955	7.08834743	6.98992551	7.13016171	6.43214745	6.98597915	6.70537669
CCDC18	0.72491233	0.80137624	1.72372278	3.3129198	1.00430538	1.55543814	1.58773378	1.99809958	1.05840173	3.1872916	3.74018963	2.30228225	-0.6524175
RPAP2	4.54055399	4.44464606	5.45023685	5.3398845	4.96266905	5.12508265	5.0429425	5.09926881	5.45143806	4.48678121	5.09150921	5.15232834	4.78492554
PQLC1	4.86342586	4.7465667	5.27527776	4.66832286	4.33636671	3.80875524	4.61738625	4.37164258	3.9482513	4.49871626	4.6161583	4.87549585	3.76161892
BBS9	3.20195603	4.10080457	2.96109348	4.1462767	4.02288177	4.1029712	4.74030886	4.37054485	4.31742995	4.14517631	4.02949229	4.36004833	4.20235086
PMS2	4.7887182	4.53541962	4.42739812	5.19663839	4.55466106	5.02486614	4.56982497	4.69061287	4.86560831	4.47922618	3.89794467	4.46850579	4.89691985
ZMIZ2	5.74570574	5.41699879	6.08682824	5.15377487	5.42472486	4.58036255	5.32738174	5.51631622	5.25603897	5.52331221	5.70046992	5.59044651	5.19639231
EEPD1	5.44037389	3.91634426	4.99229199	4.02452825	3.52955979	4.48967682	3.92639489	3.50265561	3.69086428	4.43637956	3.5654825	3.44535495	4.07432539
KLHL7	2.86896796	3.48863839	3.31280821	3.51407778	3.46907367	4.60650312	4.23473133	4.24055715	3.43995223	4.44749728	3.95914952	3.09793032	3.96111185
HERPUD2	5.95679823	5.57935738	4.96331167	4.87050839	5.41405132	5.40480866	5.39627987	5.6379478	5.53553124	5.28446863	5.61063524	5.20597878	5.62946237
CBX3	4.67185677	5.6758492	5.69925221	6.75720818	5.81561914	5.9797439	6.29329082	6.12612031	6.04443944	5.70263771	5.95990775	6.13939282	4.8033184
HNRNPA2B1	6.72053677	7.59327481	7.47383273	8.21519587	7.82020577	7.80779666	8.07439844	7.64361026	7.71310041	7.35975248	7.45638106	7.21025939	7.62552116
WIPF3	3.30291943	3.85731234	2.59882876	2.55802526	4.0602629	3.03806152	2.56948857	3.10442853	4.2632349	3.21107481	4.06284895	3.17114249	2.9480654
FAM126A	5.86631179	6.61573774	6.00008627	7.28326168	6.84746314	6.82754244	6.76163891	6.97681088	6.8348835	6.32241785	6.95123215	6.66383232	6.16738921
HOXA7	-0.12650691	-0.12650691	-0.12427827	-0.05247864	0.2603667	0.5247693	0.20866364	-0.12366369	-0.02612492	-0.02612492	-0.02612492	-0.02612492	1.31156131
INHBA	5.21860843	5.84730365	4.96449998	6.88711092	7.57154973	6.29915438	6.38642024	5.7863532	5.97534745	6.30328563	5.82525761	5.5604831	5.80607375
FKBP9	6.48486328	7.12469947	6.75673272	7.05101262	6.83869161	6.9080101	6.78957639	7.43415438	7.40133088	7.55357175	7.46806506	7.33436383	7.48635426
ARL4A	2.71086273	3.72536635	3.75434901	4.37784173	4.96700429	3.98555852	4.01948615	4.84651518	4.8534103	4.11911843	4.63053153	4.01758003	4.21027696
CCZ1	4.38507847	4.38581631	3.61699466	4.68680881	4.24447378	4.71597768	4.60933467	5.60126401	4.80599998	4.24695048	3.86341817	4.309995	4.68619473
POLM	4.93552532	4.35408997	5.51519028	3.7605113	4.01814242	2.79930037	3.94049213	4.18151195	3.762198	3.80004874	3.41994025	4.70652345	3.91794719
RAMP3	0.03598269	4.4175499	1.25625291	1.10662981	3.68323973	1.05998319	3.81539141	3.53701608	5.12752587	5.58964312	5.29872207	4.33418229	5.2794367
TWIST1	4.67613286	3.51847316	4.79389705	4.93783131	3.80121932	4.85469165	3.3291564	4.09755765	4.81796756	2.88317462	4.08476697	4.01491949	4.31255444
SMU1	5.87166847	5.95378414	5.95079232	6.00281614	6.36044064	6.61635882	6.35634769	6.11193502	5.88566422	6.13338758	5.87868732	6.12312989	6.34083037
GLIPR2	5.41612362	5.45632294	6.17446493	6.00403925	5.88043074	5.20632191	5.17923775	6.5108798	4.63179244	5.46970943	4.90928341	5.59696475	5.98518911
CLTA	5.57811272	5.84210451	5.87374123	5.39942787	5.31887649	5.58691917	4.94318391	5.47570593	5.93963526	5.78870517	5.29509657	5.43985676	5.0044319
RECK	6.14295273	6.05078036	5.63483053	5.81330633	6.23776247	6.18339345	5.91256867	6.55319291	6.17627006	6.1352404	5.99514824	6.14122953	5.77814785
ACO1	5.71942069	6.08152997	5.15756961	5.70038969	5.41300751	5.8968329	5.64636958	5.7152811	5.53245969	5.36359013	5.4665496	5.68054775	5.89992893
DNAI1	0.35578737	0.08899163	0.09157734	0.20559879	0.2960333	0.09042925	0.08899163	0.0922904	-0.08181358	-0.08181358	-0.08181358	-0.08181358	-0.08181358
DCAF10	3.16017163	3.62506384	2.9557942	3.64769345	3.84003293	4.04028888	4.10539147	4.04983536	3.36684069	4.50498232	4.57692665	3.48515116	2.71094082
CNTFR	0.78136117	0.64499409	0.79653685	0.5355834	2.73776701	2.23412195	2.44485205	3.27097802	0.74134884	3.03036722	3.82091763	4.39212378	3.91550717
KIAA1549	3.83397938	3.56627524	3.73835999	3.11230483	3.71629221	3.11197841	4.02789248	4.06016257	3.25627365	3.2959943	4.3218513	3.66274834	3.81418596
TRIM24	1.86468821	0.87880063	1.57590807	2.2440672	2.27392664	2.49696895	1.62994665	2.08985315	-0.38650881	3.28647183	2.27986626	3.64847473	0.64969526
C7orf49	4.52045017	4.81633227	5.19732481	4.43506959	4.09163549	4.0555441	4.31622622	4.36558981	4.74949111	4.03552991	5.41732588	4.07220422	4.14276763

CALD1	9.23639075	10.0074953	9.34092371	9.53770062	10.5367906	10.6538082	10.3614149	10.2216508	10.2853159	9.71680052	9.99235821	10.398309	9.39767412
NUDT10	4.19280508	2.96523685	3.00059463	3.30578221	3.22310138	4.10864474	4.08485322	2.89104604	4.10183656	3.74701157	2.87416224	3.3145207	2.4837438
PLAU	4.83111985	4.73631062	5.27068319	5.74088885	3.68346113	3.70937559	4.16320334	3.49029884	4.45553997	4.59831545	4.39035804	4.70443946	4.88624864
SRGN	6.92523514	6.84516299	6.83057849	8.24542358	5.93725867	6.29422523	6.74592806	5.50659531	6.19391781	5.91801864	5.93193232	6.18629948	6.40375164
CHST3	6.58545274	6.54950178	7.45759904	5.93146646	6.53776031	6.40630734	6.14152233	5.4893443	5.51442265	5.77543212	6.20163422	5.50386354	6.18205451
BICC1	3.20075194	4.17211949	2.51148004	4.80113567	2.36365806	3.5393013	3.31816711	4.07541859	3.80187032	1.44644869	2.82679562	2.99550639	4.09195167
CISD1	5.07621266	3.68619509	4.66092346	6.00888205	4.43987855	4.50478043	5.57029024	4.44986121	4.50556293	3.23069421	4.41056635	4.22710744	5.40287767
EGR2	3.653398	2.5879387	3.09473781	4.70974325	3.07667659	2.90715648	2.35470827	2.29146173	5.03985133	4.24180024	4.91131165	4.504139	5.45131816
ECD	5.2739991	5.36587023	5.14150048	5.89072889	5.54830802	6.05014071	5.68509544	5.7213465	5.508974	5.9970409	5.29387778	5.61367305	5.93893705
P4HA1	5.73402072	6.07702416	5.90614379	8.13983006	6.42049081	6.37596031	6.40955707	5.84371731	6.14297036	5.88595427	5.89113645	5.83395362	5.83173736
SLC25A16	4.64634884	4.61697144	4.7603422	4.68775749	4.98557391	4.4517126	5.33817161	5.12765682	4.91192728	4.80881811	4.70400016	4.82052031	5.25993577
ZWINT	1.43285188	1.76770989	4.3575902	4.57103179	1.69407752	2.16360007	1.70576689	1.09546801	3.12053589	2.7149453	1.56481796	3.11246581	-1.0698424
VPS26A	3.71294638	5.12546079	4.34592641	5.3419096	5.77642695	6.06294245	6.01391331	5.71977817	5.27232458	3.81584079	5.60903407	4.87949083	2.32909431
RBM19	5.39787735	5.14576647	5.66955304	4.52051765	4.96759209	4.44906016	4.96320786	4.62555328	4.58976022	4.72106588	4.74275824	4.66366819	4.62915127
CIT	2.1909218	1.66572368	5.08928084	4.15816788	1.56060892	1.7835048	2.22555051	1.59155969	0.1073375	2.02896503	-1.09527696	1.9893626	3.81593711
IFT81	4.56080751	4.55227781	4.78933827	4.97150897	5.37655089	5.78315999	5.24214413	5.29764882	5.56821659	4.76258906	4.73196059	5.42819732	4.59227
ACADS	3.66209701	3.62429735	3.74830458	1.60919917	2.40395171	2.0787329	2.30473781	2.4963858	2.81380415	-0.94037791	2.61412604	4.14946501	2.59751471
HVCN1	4.96645317	3.43649704	4.31075504	3.60707856	3.24544018	3.89047928	3.40402374	2.96134721	3.92848261	4.24664592	4.38670761	2.72989569	4.06875717
DDX54	4.13062997	3.1590929	4.77844806	2.94703086	2.50386452	2.86895989	2.6261856	2.94337125	3.6201837	3.33152002	3.37432799	4.73574089	3.12075905
MED13L	7.93764771	7.57478014	7.04723653	7.24561566	7.71078621	7.75872006	7.69636429	7.66723727	7.20176825	7.15361331	7.42804542	7.12529881	7.61615905
CDKN2C	4.35086487	4.22266364	5.48200349	4.31505144	4.00208745	4.17321782	3.80691152	3.37169532	3.81041857	4.68004477	4.40666152	4.62006391	3.57580859
RNF11	4.61536934	5.30229662	4.54685957	4.96377361	6.2192337	5.85071118	5.74671605	5.66291861	5.52309416	6.05646126	6.12456169	5.67652637	3.96395747
RASSF8	6.34878711	6.94632063	5.66952694	6.91558699	6.99948182	7.25766281	6.84108244	7.41875095	6.65544634	6.10678464	6.18138006	6.45071578	6.40714553
BHLHE41	6.57910793	6.39934519	5.23822427	5.25001705	4.92851786	5.36511073	4.53466904	4.92371786	5.57862679	6.11095386	5.24014294	5.97468818	6.20301507
SSPN	2.37497826	3.80977454	1.7720416	1.59487576	4.53144678	5.06657982	3.4845096	3.99245373	3.43135001	5.33764576	4.14353348	4.37758375	2.1107222
ITPR2	5.23004808	5.85620901	4.98038063	6.4421847	4.87674768	4.60596306	4.97116469	5.08212943	5.5258009	3.98220478	4.93779186	4.4207069	5.72246396
CCDC91	5.06085357	5.86865159	5.04330223	5.90374491	5.74086039	6.14122373	5.94941222	5.95481326	6.06591819	6.3054523	5.61661359	5.59603153	5.6479691
NECAB1	0.76622157	1.10444006	0.76882443	0.98624574	0.80510804	0.85667757	0.76622157	0.76954221	4.29573494	-0.39361526	1.86263782	3.50066863	3.30458283
WWP1	5.15528384	6.18912903	4.92796736	5.90646673	6.17112447	6.01264323	6.08176617	6.29539306	6.08006803	5.8533012	5.7403748	6.18007569	5.81469086
ACOT9	5.13769051	5.77152623	5.54376717	5.64130692	6.16007112	5.55766464	5.92749706	6.53629295	5.60827984	5.90697978	6.00139029	6.25527562	6.33193522
PRDX4	5.01368372	5.56372247	5.82431551	6.88401905	5.23487052	5.51295908	4.97301071	5.3263027	5.9298042	4.88256567	5.47699212	5.33982141	5.74252869
DDX39A	4.05694521	4.12543714	5.09265524	4.76949882	3.77838725	3.22147526	4.65570183	4.07883653	3.54233917	4.48230298	4.00772644	4.86804065	2.58492792
PKN1	5.66089454	6.32808561	6.49680495	5.27738655	5.61884738	4.87651402	5.60034825	5.53323511	5.37804679	6.39380958	5.81844368	6.03248707	5.6629158
WDR83	2.41452983	0.73143308	2.44852725	0.1586415	1.90236055	1.94464701	2.00711033	0.95955968	1.45009554	-0.26392084	-0.26392084	2.6963006	-0.26392084
GIPC1	5.2109301	5.15621669	5.89393322	4.90955961	4.68316673	4.71889586	5.05910682	4.52489764	4.98213034	5.63587865	5.52170825	5.41919983	4.78858342
SPRYD7	4.43581848	3.98000593	4.8976897	3.85012733	4.52779088	5.24006992	4.54430218	4.23186343	4.331959	3.73244165	4.51431882	4.70237384	4.47482182
EBPL	4.00219837	4.59915295	4.17254674	3.46251055	3.3962408	4.29605364	4.67495769	3.47189415	4.3480418	3.31683481	4.27059979	4.54291916	2.69479193
ATP7B	2.19676069	2.7291738	1.89360344	1.91332469	2.211883	1.82858807	2.37102051	2.62135359	0	0	0	0	0
ZC3H13	6.88123646	6.72963077	6.82054401	6.48963327	7.13352609	7.76405921	6.61179827	6.72698717	7.46940638	7.39193745	6.38193354	6.72328685	7.19761453
NLN	0.81251637	1.43402383	1.69783411	2.96623915	1.13861471	1.25252817	0.91293329	0.73775538	3.15054792	-0.38301878	-0.38301878	-0.38301878	-0.38301878
CENPK	0.70238703	0.51036407	1.56312626	3.23799416	0.4536803	0.36452378	1.77930164	0.85978874	1.99046983	1.60789212	-0.9360856	-0.9360856	1.50670617
OPTN	3.75615768	4.47579905	4.30713164	3.34682632	4.51769845	4.43521348	4.304771	4.4799912	5.03088713	4.36753638	4.1632682	4.36208996	3.7850671
ITIH5	5.90661051	7.10820899	5.48877602	4.03271848	6.75774174	6.5507838	6.61262075	5.15988605	5.65398648	6.41697936	6.32440914	6.98911115	7.03815899
ATF1	5.8394722	5.00126421	4.58304398	5.39921835	5.21204552	5.52599088	5.54992207	4.99586181	5.67046671	4.06151518	5.2345873	4.9688149	5.03182861
TSFM	4.22498949	4.32054414	4.86430545	4.80028244	4.70883762	4.41524845	4.61816481	4.6111834	4.40886749	4.99893743	4.11638531	4.11156997	4.8473601
ARHGAP9	3.75695744	3.19058214	4.55293177	3.56399078	2.94876797	1.98900983	3.05520379	2.52326052	3.30150442	3.36826677	2.66892028	3.41341072	3.67918927
NCKAP1L	5.50245135	5.62571646	5.7482858	6.00702399	4.18629421	3.79477054	4.52974032	3.91600497	4.91118632	4.43545042	4.3848965	4.09031888	4.95628714

MMP19	6.19226374	3.98799019	6.47640152	5.68640237	4.85536858	4.17191177	4.97238084	6.20595601	4.71198202	4.11131049	3.58451771	3.39965297	4.74264376
PFDN5	7.08209011	7.22335493	6.8463668	6.68828036	6.49896422	6.69589386	5.9893628	6.66276047	6.99606151	7.01856202	6.13571356	6.54755748	6.68680906
SPATS2	3.11295462	1.90615314	2.31890979	4.48322513	2.52046221	1.87867275	2.76590758	2.87050376	2.82701571	-0.514611	4.20772118	3.22929982	2.88778955
ORMDL2	4.0737112	4.04886359	4.39739546	4.15553701	3.99354271	4.11551613	2.8305741	4.06008127	3.98827829	3.43662665	3.64618522	3.83012946	4.08638961
NR4A1	4.49416952	5.53915157	4.80709204	6.05073413	5.3865966	5.42307471	3.8180748	5.90250833	6.25231123	5.74014221	6.96377133	6.8940086	5.62501773
PDE1B	6.03430312	4.27474745	6.23568029	4.7307809	4.62854052	5.42283206	5.38095411	5.26853964	5.17580166	5.22851532	4.92995667	5.01888124	5.53084541
CDK2	5.73498543	3.9770901	5.49543483	5.58367098	4.73397177	4.41134003	4.48997928	4.60980803	4.13511056	4.1991738	3.82258684	4.46445206	4.09974194
LRP1	9.51962029	8.55091084	9.51962029	8.59378665	8.41292058	7.8483077	8.79390289	8.63154984	8.07678236	8.31854053	8.97507495	8.52490022	8.27647432
NFE2	0.74271504	1.90707613	0.16406	0.96047372	0.25601109	0.28110633	-0.20692496	0.06405291	-0.30883586	-0.30883586	-0.30883586	-0.30883586	3.79393443
IKZF4	5.69626	4.14400178	4.9577398	4.26011413	4.65005204	4.43107019	4.39350673	4.62151175	4.0435675	4.18840569	4.25169524	4.18374642	3.99853334
SMUG1	3.97092837	3.65907035	3.90538473	4.0667783	4.1220458	3.72194808	3.60262759	4.38632794	3.82120469	3.63045936	2.90505659	4.04216958	3.47875759
TUBA1B	8.18306993	8.20523483	9.00362582	9.16418197	7.84448473	7.82683201	8.41192158	6.98534634	8.39838301	8.21425017	8.2394442	7.99158406	8.30075819
KBTBD4	4.75751368	4.30867396	4.32973301	3.65322955	3.77531662	4.2961105	3.81734494	3.87400192	4.43806688	4.5777963	3.80080141	4.61590537	3.64123304
SARDH	1.39585221	1.76469002	1.40840156	1.92369183	1.2077425	0.88854087	1.56432044	1.61551421	1.88664428	-0.62251016	2.04877191	1.58078201	1.19781618
ATPAF1	5.34854987	5.31971412	5.2928546	5.25968786	5.88784191	6.21180553	5.93327186	5.63859983	5.59753813	6.19919662	5.53576735	5.17541384	6.26132699
STIL	2.03727683	1.87141611	3.46997125	5.05674254	2.1145804	2.41243561	2.50160875	1.76129116	3.4730962	1.1766765	2.94208431	2.7252483	3.85593651
HJURP	1.33742881	0.72071638	3.85938529	3.59876773	0.29748293	0.38413967	1.42144659	0.37955972	0.0426641	1.63283379	1.59154432	1.98169074	3.18452619
IL13RA2	3.76408457	4.01719988	3.92024161	5.45593626	5.21860114	6.5372005	4.72221318	5.61847332	5.18881593	1.56139374	5.1715833	4.83335825	3.46575405
COL10A1	2.8227131	3.04767857	2.19301341	4.11555388	2.68958924	2.2227571	1.79650802	1.71574138	5.27347146	1.26350841	5.88544682	2.8251471	4.05573188
AMD1	-0.43586583	1.37864329	0.6192263	2.0601995	1.85755828	1.71166714	1.83021265	1.19501546	1.26689166	0.76153576	0.76153576	0.76153576	0.76153576
NDUFAF4	3.38419094	4.10922394	3.6173735	4.42605721	4.96080259	5.66780686	4.95842331	4.40722493	5.19152998	4.34287191	3.90085329	4.96870623	4.25032225
USP45	4.43824595	4.85354821	4.55995689	5.31232461	5.32843827	5.52392611	5.50104285	5.57622886	5.28815359	4.74781362	5.48638638	5.20289276	4.58976388
PLP1	1.73259463	2.21244899	2.4833304	2.05394089	3.55819322	5.16772022	4.66420941	4.73525288	4.09046224	5.14076739	4.51689444	4.63446059	4.67196708
MORF4L2	6.80756522	7.29775435	6.93608267	7.77031873	7.88927674	8.12423889	7.60656284	8.16151842	7.76970136	7.93624168	7.44648081	7.66484106	7.45218327
RAB9B	3.88581455	4.28178363	3.65576944	3.64872276	5.1893417	5.47510376	4.76591992	5.43959744	4.82370141	4.90084036	4.24961078	5.70406807	5.69837494
NRK	5.44683483	5.63224847	4.69234327	5.88607698	5.43945721	4.56971546	5.11342227	6.51045925	6.36465775	5.71493527	6.79156907	6.04128511	5.84941921
FAM199X	5.19350045	5.78150826	5.15156444	5.65901154	6.24464373	6.39766393	6.32828226	6.44357361	5.95383723	4.939939	6.34953561	6.23089077	4.73794015
RAB9A	4.63264548	4.64881953	4.42178545	5.00591256	4.96017832	5.01958643	5.08972774	5.35636535	4.72610824	5.04394673	4.81711986	5.0038042	4.71458575
METTL8	4.55833452	4.88630177	4.50589765	4.59575099	5.30025418	5.04727687	5.40606009	5.28029837	5.106381	5.01085193	5.03140419	5.2721829	5.2727846
TTC21B	4.9620964	5.76435201	4.84092392	5.62972975	5.6020438	5.85992296	5.7281762	5.85377462	5.61861353	5.04863141	5.72019054	5.64482717	5.59287922
NMI	4.53563166	4.98080883	4.60523649	5.75927617	4.35456579	4.09532378	4.50245271	3.79172861	5.14651971	2.93590104	4.41132336	4.3023058	3.86560392
TNFAIP6	0.91541606	2.52482974	1.01654355	3.78384362	2.30042856	0.85035427	0.69226052	1.44243506	4.08742589	-0.92130947	3.89341552	2.29860951	2.07149321
ACVR1C	1.37016218	1.8349573	1.48245277	1.44408968	1.37540295	1.88755709	1.64423202	1.77623471	2.82071088	-0.82106424	-0.82106424	-0.82106424	3.79844825
BAZ2B	6.1504446	6.89128136	6.09140113	6.78707915	6.71872315	7.03894422	6.69176532	7.06497506	7.05835501	7.03564678	6.44605199	6.73235957	6.75127342
SLC36A1	4.12713194	3.78547105	4.58393775	4.60872732	4.02255563	3.09315808	3.47362432	3.68739012	3.98944295	4.27483643	4.07405695	3.82356048	4.16470255
LPGAT1	6.18691728	6.48330181	6.10171274	7.11261497	6.78671487	6.69211046	7.03921477	7.05549	6.64087241	6.28998672	6.58301621	6.27363778	6.81731717
BATF3	2.57394813	2.37256169	2.15258959	1.65755544	1.84103646	0.88750188	1.49309707	1.36023553	2.61263139	-0.83096485	0.8743535	1.33041806	2.31406508
G0S2	4.91574458	3.63494478	3.37054007	3.40367875	1.53911685	4.15656282	1.4946739	2.10525607	2.20259953	3.63929464	2.90560267	3.17458516	3.96351459
KCNJ2	2.45715965	3.51316135	2.57263639	4.75617395	3.77524219	2.70216958	2.82300262	2.32695362	3.53519251	3.63374237	3.91349092	3.54637364	4.7071868
RAP2C	4.04862152	4.70173023	4.86160396	5.38175121	4.81545001	5.01576528	4.79850778	5.0021679	5.45799661	4.33486472	4.73596711	4.96374318	4.5327421
EXOSC9	3.96771067	4.46988569	4.32391134	5.04187758	4.87328845	5.03844308	4.76695571	4.36940537	5.09662693	4.51379908	3.82047216	4.4782507	4.53565208
PLA2G12A	4.89905751	5.88521444	5.12867446	5.09695067	5.61486797	6.03873674	5.7356335	5.72302476	6.26994372	5.66716384	5.52291206	5.75442483	4.98081847
B9D2	2.9624397	1.1966825	1.67601358	2.38789148	0.94677144	0.92214078	1.03572438	0.82772931	2.6791548	-0.61632224	-0.61632224	-0.61632224	2.13737767
PFKFB2	4.50600752	4.81509927	3.99726839	4.58684559	4.27053042	4.56563486	4.37922256	3.77610341	4.78475557	3.80090274	4.89108333	4.47231684	5.1058649
C4BPA	-0.05491729	4.14151684	-0.05326037	0.88012848	-0.05491729	0.26752893	-0.05491729	-0.05280344	-0.20371964	-0.20371964	3.38116116	-0.20371964	3.91973298
C4BPB	0.84095416	0.79524578	0.26538205	0.56064759	1.71513758	1.10447252	0.64832108	0.2660741	1.83040465	-0.09682881	-0.09682881	1.54724469	-0.09682881
RAB38	3.86042676	4.67140856	2.64519507	3.96403803	2.36900688	2.94872766	2.12150122	0.90974996	3.80227105	3.32813256	3.51990289	2.2345205	2.10833086

GPR83	0.97853122	0.8546956	0.89341673	0.8010658	1.19305057	0.908774	0.84045217	0.87909665	-0.43273446	-0.43273446	-0.43273446	3.30887685	2.97523271
MXD4	5.16799645	5.49324593	5.84595865	5.01892919	4.74503996	4.51477453	5.12138939	5.04409818	4.69561701	5.28693732	5.5864726	5.3289257	4.73729195
CKS2	-0.49826276	-0.65745421	0.76499008	2.77586711	0.1463806	0.80028784	1.76065133	-0.16298644	-0.37364496	-0.37364496	-0.37364496	-0.37364496	-0.37364496
ACSL3	4.694701	4.6035318	4.55079652	5.26376404	5.20587963	5.79913772	5.44151504	5.49707845	4.71082628	5.3343134	5.18885401	5.61564101	4.79503183
CHPF	6.1652696	5.97908724	6.7339544	6.30087248	5.20239639	5.53759301	4.61834717	5.7244875	6.07646791	6.77793017	6.91675921	6.64990855	5.13918774
DNPEP	5.4770838	5.26434388	5.37314445	4.50636259	4.62535747	4.14889775	4.73670346	4.40428962	4.92746079	4.95755006	5.10438271	4.76315441	4.65736139
INHA	0.58622799	-0.49813915	1.3959591	0.52620541	0.45543714	-0.1718466	0.09175362	-0.01935598	-0.06846789	-0.06846789	-0.06846789	-0.06846789	-0.06846789
MOGAT1	0.51293178	0	0.94830084	0.03580804	0.47194498	0.80275884	0.27773961	0.44556327	0	0	0	0	0
OBSL1	5.83708268	5.41874062	6.02209808	4.39251861	5.37869738	5.07578539	5.07885201	5.76335523	5.33181413	6.1602173	5.74886167	5.97240428	5.87066537
FAM124B	0.11924801	2.21030994	0.7392504	3.0399625	2.42201876	1.51592478	1.53918707	2.00492221	3.32683172	-0.57074292	0.49567721	2.55994815	3.79886372
SLC12A4	5.96534693	6.05516301	6.24249192	5.45761427	6.52938333	5.58080274	6.50617886	6.67163536	6.14184989	6.86426274	6.46600327	6.81318185	6.38106649
PI3	2.34235905	1.18223321	-0.28987685	1.20950504	0.68890022	-0.29084463	-0.29205647	0.03800178	-0.11942527	-0.11942527	-0.11942527	-0.11942527	3.51572992
SNX21	5.43320147	4.99377947	5.64159762	4.32092635	4.9389267	4.94836816	5.12274073	5.37740654	5.27989692	5.55532503	5.59162609	5.78568582	4.48649923
SLPI	5.23156485	7.2316506	3.46791271	6.45319823	3.27163397	1.9054727	3.55588644	2.54406321	5.58633311	3.59624798	6.25813291	2.23032047	4.0566671
WFDC3	1.22133096	0.36798313	0.53731638	-0.56786306	2.17539731	0.21695312	0.12493589	0.99226288	0.03967164	0.03967164	0.03967164	0.03967164	0.03967164
TTPAL	4.85418719	5.15566434	5.16650523	5.26135617	5.25174213	5.07041498	4.72113369	5.24484984	4.33166413	4.22949674	5.21282841	5.04898794	5.1260071
PREX1	7.31227884	6.80210506	7.30267087	6.49938973	6.20022148	6.51226143	6.44373812	6.32057859	6.30012571	6.90348659	7.00488209	6.56697207	6.10836097
KCNS1	0.29984665	0.44386316	0.24336587	0.37672273	0.24076368	0.24221046	0.24076368	0.33719833	-0.15656388	-0.15656388	2.90549187	-0.15656388	-0.15656388
SLC12A5	0.1398465	0.46713874	0.2885387	0.25232627	0.10161418	0.17298864	0.11820961	0.05376467	-0.08192923	-0.08192923	2.9436145	-0.08192923	1.98360094
SDC4	6.23112505	7.2300368	7.17975811	5.849226	6.34698847	6.74531462	5.21890456	5.73333824	5.61408289	5.83841475	6.14149637	6.21801353	5.7705839
NCOA3	5.39760768	5.70187591	5.12378334	5.51327987	5.99487741	6.11330899	6.06304632	6.32902905	5.69462456	5.16998566	5.5803399	5.17431346	5.46288064
PIGT	6.88433058	5.86550932	6.86274965	5.98128168	6.14605948	6.550194	5.86196946	6.17820219	6.35283622	6.86599817	6.21857308	6.7580732	6.41721105
NCOA5	6.0968693	5.49834043	5.93727389	5.56713838	5.49253738	5.5522164	5.47520061	5.10920988	5.16162739	5.22492221	5.34307836	5.76228085	5.70824518
VAPB	6.69269332	6.9653582	6.3870087	6.52816254	7.01774157	6.88408731	7.14207219	6.72173849	6.88734528	6.97149474	7.06472527	6.88165067	7.06736798
PARD6B	2.55169698	3.64437508	2.81155515	2.98376296	3.265999	4.41950984	3.45636321	3.34454659	2.09107834	1.86430802	2.97685766	3.77410973	2.43060388
ATP5E	4.81704124	5.36003143	5.14375785	5.26367185	4.97273631	5.20035899	4.34806108	4.77872703	5.45931128	5.62917159	4.69234652	5.36984935	4.85557491
CHD6	6.58906132	6.50409258	6.23846686	6.13581911	6.81280807	7.11996069	6.69121496	7.10649225	6.70586044	6.98115894	6.8028906	6.71798625	7.27218155
PLCG1	6.18723779	5.72767931	6.13850808	5.24727535	5.93190223	5.65877214	6.0598467	5.88331047	5.86116026	6.12057842	6.28512686	5.80152871	6.21196813
TOX2	4.2728643	1.88027609	4.40489844	2.65424807	3.30374222	3.98581425	4.52306325	5.94878615	3.50708396	5.8074107	3.73417706	4.53818058	2.42490657
SRSF6	6.40620596	6.77191812	6.22458209	7.33031832	6.89348644	6.49983095	7.12348213	7.08490369	6.91161047	6.40651646	7.00042895	6.92840414	6.24333451
ARFGEF2	6.32987533	6.93606194	6.37011424	6.47991071	6.54405198	6.4325535	6.65543253	6.62508328	6.62638323	6.57625543	6.75827264	6.62228632	6.8612635
ZNFX1	7.28807971	7.15921323	7.06263289	6.7078206	7.13925434	6.98328279	6.80381389	6.83510476	7.05519521	6.90278609	7.37600313	6.87357994	7.46665425
ZNF831	2.32469353	2.06851454	1.46811256	1.44378953	1.67844298	1.38699282	1.46389617	1.42698062	2.15470981	2.82329111	3.1374762	2.3821976	-1.03124057
CSE1L	5.21018063	5.83594653	5.83489413	7.06406161	6.22760222	6.38698	6.36131526	5.97191195	6.33770589	5.86621135	5.66206433	5.86072389	5.68711753
RAB22A	6.30591513	6.53824209	5.87218725	5.95634503	6.88826979	6.74749829	6.64407851	6.67714396	6.79755355	6.09845858	6.55228844	6.30496623	5.94093894
PTGIS	8.9366673	9.36549929	8.4925611	7.36376013	8.4925611	8.04157572	9.14309313	8.52657898	8.37897602	8.57846559	8.60312349	8.55536044	8.47163239
STAU1	6.98781686	6.41550535	6.68891998	6.34511866	6.31609292	6.60307375	6.43635171	6.34850996	6.70701391	6.96061982	6.42025104	6.22956935	6.46842028
CDH26	2.61058708	2.83155725	2.03275958	2.1584578	2.71300479	2.94212863	3.22298604	3.13178331	3.14705523	2.12484772	3.49353331	2.17366211	3.8407813
SNAI1	5.16781946	2.67374595	5.07696798	4.53936691	1.58054722	1.4683526	3.88185458	1.92588153	0.43835247	4.32453341	2.15441563	3.511412	4.34603206
MOCS3	4.23372468	3.44612749	3.8841124	3.27537388	3.53698603	4.2669256	3.71706316	3.38998851	3.91520805	3.77776947	3.88820551	3.78103967	2.52842664
STX16	5.61922382	5.60896343	5.57554967	5.97336452	6.03513524	5.02930635	6.09202567	5.76975336	5.67291685	5.59517865	5.86222808	5.40332959	4.90140469
PMEPA1	7.18537767	7.31607108	7.59671175	6.52392889	7.80957187	8.20776252	7.85238728	7.81866641	7.7429919	8.17889804	7.93716871	8.22661832	7.14350182
RNF114	6.17210426	5.8408918	5.61549807	5.88405573	5.66989835	5.17231287	6.03034254	5.55079351	5.39180746	5.64453346	5.48141325	5.6563505	5.26521064
DDX27	5.62866822	5.00792635	5.57345444	4.45374939	4.84354454	4.9685866	4.85901876	4.76173781	5.33864684	4.60756487	4.78775455	5.2030821	5.22521512
BCAS4	2.45312657	1.70412501	2.86349316	0.00206011	1.69658065	2.13513274	2.45161545	2.29976926	3.08960638	3.35335258	-0.4945429	3.74136024	2.78945638
KCNK15	5.36127428	5.85282878	5.3638948	2.86274134	4.86616993	4.43381147	4.56988383	4.30514103	4.10134771	4.60230164	4.2798208	4.70734823	3.15770607
TP53TG5	1.55337552	2.40982509	1.62163057	0.66457254	1.34463389	1.17691983	0.68700648	1.36816668	2.37757353	-0.54231769	1.86752154	2.35592489	-0.54231769

PCK1	0.79627095	0.93034701	0.7988371	0.79627095	1.08248907	2.07879332	0.79627095	1.22973577	-0.49927768	-0.49927768	2.66438433	-0.49927768	3.10726421
ZBP1	1.65633926	1.78218618	1.54122327	2.11097252	1.25106983	1.36031341	1.10695556	1.23388827	2.68021711	3.27741493	3.005009	-0.68952986	3.29028549
NEURL2	3.68589925	1.57527651	4.29737598	1.85581842	2.0888429	1.11612504	2.69125015	2.20030299	-0.72853018	-0.72853018	1.61857967	3.42503115	3.03449731
MTRR	5.84876627	5.90563547	5.28030029	6.10408161	5.62359829	6.22784384	5.71103652	5.52125688	5.38058204	5.79268005	5.45089915	6.07089342	5.67302662
FASTKD3	3.44362371	3.94508572	2.73353616	4.09009225	3.91443033	4.45380685	3.61496652	4.18018617	4.07561993	3.54289806	4.33000074	4.06263888	2.90013636
PEPD	5.25246257	5.47696456	5.44321609	4.52592192	4.56636056	4.68050062	4.36916744	4.49874517	4.93039951	5.64892066	4.98034852	5.80806838	4.87481769
IQSEC2	5.29785962	4.35095045	4.85011944	4.06211221	4.17656348	4.29648353	4.22064994	4.21717908	3.79534517	4.86754935	5.10419769	4.2981819	3.9248057
VAMP7	1.90914115	3.30479116	1.97469868	4.22186425	4.14780748	4.08257168	4.15384241	4.04362965	2.84335564	3.04824557	2.62003441	2.77433011	2.66905868
IL9R	0.22589629	0	0.00124472	0.17724199	0	0.08448531	0.25398939	0.00158797	0	0	0	0	0
XG	4.98827069	3.71182499	5.18970339	3.70478606	5.26000612	4.92197153	5.17677636	5.97353554	6.15386147	5.09962457	4.98801611	5.1110455	4.60387239
STAMBP	5.60770684	5.48761457	5.43776657	5.35395934	5.85498081	6.0840929	5.56332238	5.812422	5.64379759	5.31286028	5.9199124	5.4674939	5.46455028
NAGK	5.19343671	4.82627644	5.02370334	5.33356972	4.5515476	3.81248504	4.71630591	4.72568672	4.93731304	5.24594544	4.86549458	4.74514234	4.70237962
MCEE	3.35142939	3.72834084	3.10907024	4.47377654	4.51626156	3.72777126	3.79718948	4.36384889	4.05824332	2.16897324	3.83415364	4.02676902	4.23242931
PAIP2B	4.80508821	4.79245708	3.68854963	3.62194744	4.82708787	5.63206143	5.07154413	5.11796789	4.97772373	4.91755619	3.97016119	4.67060852	5.23312512
SNRNP27	5.30310954	4.81858842	5.47398822	5.12977401	5.94841299	6.02071096	5.54471662	5.48245245	5.46497922	4.95910752	5.63863229	5.26266262	5.38909622
MPHOSPH10	4.97593514	4.69570857	5.49643666	5.93370182	5.26487804	6.31304929	5.67501797	5.32758371	6.4447092	5.37132274	4.6560325	5.26696636	5.32381462
ATP8A1	2.96256655	4.97387672	3.02679981	4.28667078	4.49368258	4.53584438	4.69310518	4.87440334	4.54601098	4.30485348	5.11942834	5.13352166	4.83388985
USP22	7.9130468	7.29736691	7.54054293	7.47824623	7.11835735	6.63354134	7.26241588	7.00714423	6.7138936	7.17717949	7.25222557	7.26947911	7.26194033
POF1B	0.52114945	1.29400182	0.52373226	0.61264702	0.54332958	0.52258546	0.77069024	0.7303198	1.70632664	-0.20159345	-0.20159345	2.47807048	5.45746394
HIF3A	5.38604151	4.50774434	5.48168807	3.46101182	6.22281523	5.56708944	6.20148988	6.05331061	4.70135038	4.31744379	5.05827785	3.96690278	6.4546293
ZNF576	4.48197365	3.97530563	4.57705977	3.00948679	4.01011566	4.32326394	4.11943635	3.711234	4.08827335	4.59181491	3.92929497	4.20592256	3.53121113
ZNF45	5.51995658	4.96342776	4.98748955	4.60909148	5.133865	5.70477881	5.01331118	5.18522261	5.68275738	5.2402726	4.97899024	5.01556885	5.61176578
LYPD3	3.75700213	2.39153211	2.51368043	1.57240587	2.20492389	2.31885333	1.91373569	2.15419848	0.61508092	3.78142857	3.56695788	3.51604151	1.47277225
NDP	1.61550627	1.08666244	1.77243567	4.54433567	0.83151648	0.85607852	0.87066419	2.13672014	2.05573342	3.30930325	-0.99083216	-0.99083216	2.3845319
USP9X	7.02482062	7.60665112	6.98196212	7.72682241	7.60806763	7.68715817	7.71120253	7.58766157	7.33999132	7.49834837	7.5444426	7.74573609	7.55797616
F13A1	6.68204139	7.56414952	5.31756478	7.06028966	5.87771134	4.80372999	7.26121794	4.40047903	6.73632805	6.08277322	5.92254599	6.34066254	6.83398021
TRERF1	3.89791809	4.37400053	3.80260901	3.58100361	3.03318033	3.03839505	3.8058084	2.67207299	3.43972959	4.12899401	4.41347272	3.8411924	3.65076956
PACSIN1	0.46390267	0.4843601	0.56189496	0.49409012	0.50993185	0.82330267	0.35085332	0.54726087	2.93198298	-0.06102506	3.22553987	-0.06102506	4.57656671
BTN2A2	5.18944192	5.02307803	3.80948389	4.81733966	4.35107728	4.68379374	4.48066897	4.74729261	4.83146749	4.05973249	4.70835587	4.31627736	4.41136752
SIRT5	4.17115302	4.48617849	4.0924532	3.88659923	4.48703096	4.38880259	4.35565516	3.74777573	4.24838012	4.4212784	4.22538948	4.2412548	5.01448318
MRS2	4.62843096	5.08483469	4.61897581	5.38816087	5.07423532	5.28568279	5.42785535	4.81714712	4.65165681	5.1487498	5.35017238	4.84666338	5.29597551
WRNIP1	5.59003559	5.78110743	5.77371845	5.1883534	5.40336291	5.26216414	5.32373045	5.8400258	5.46964052	5.62029934	5.5473016	5.64016363	5.74343223
RRP36	5.10683238	4.78698536	5.07828426	5.83666695	5.4081236	5.32007832	4.89372987	4.8068475	5.31603844	5.7438447	3.5175428	5.0603153	5.73774952
SNRPC	4.97723023	4.79654501	4.91684066	4.76130028	4.21501645	5.57186155	3.75071243	3.80475067	4.34835179	4.83237551	3.28290244	4.5119629	5.06779672
SERPINB6	6.4856155	6.35647401	6.64306629	6.17132177	5.80825452	5.8829337	6.10021148	5.80933271	5.88126975	6.03225789	6.11573771	5.76455669	6.20575987
XPO5	4.73280459	4.63055262	5.0115025	5.30876531	4.20485753	4.39547814	4.90128567	4.29458416	3.55483693	5.12675075	4.3238338	4.75417875	4.23115173
ABCC10	3.56230113	3.22584275	4.11463729	3.37575294	3.13441272	2.30932749	3.54614237	3.04840891	2.64175636	4.31124	2.10013077	3.5050656	2.79273379
HIST1H1D	5.08035822	4.74441258	5.13475201	6.12935296	4.55300635	5.08551598	3.83831466	4.16373815	4.27143904	4.39302222	4.74077503	4.38064786	4.22969574
PEX6	5.02623959	4.38159819	4.99813366	3.27512115	4.11553003	3.86631741	4.78526132	3.74984683	3.2779753	4.40021189	4.22933464	5.1717561	3.0213395
NQO2	5.34498315	4.75257111	5.28574065	4.36904565	4.46761076	4.21742712	4.06163926	4.69543728	4.76101862	4.64086886	4.12794594	3.96525972	5.12648135
UNC5CL	1.33941347	1.58475609	1.46311981	0.85099617	2.12181501	1.30088242	1.43261082	2.09829923	2.76121073	2.92314132	1.76946254	1.09838875	-0.61098351
AARS2	4.39582989	4.34638417	4.94397772	4.18886619	4.09551795	3.52069302	4.05027888	3.76779265	3.58973106	4.44010772	4.32152299	4.23438519	4.2270196
HIST1H1A	2.68692815	2.55727448	2.24961626	3.78345287	1.1961734	3.43003125	0.2500109	1.92495946	4.053585	2.8445142	4.56847264	2.8256103	-0.59846833
ZNF391	3.84936519	4.55580474	3.57043694	3.6036525	4.4981889	4.79027323	3.79359197	4.50670052	4.09168556	4.7184548	4.28892709	4.02292499	4.35052756
RPS10	6.10142763	6.89823053	6.24644482	6.04073395	6.33087517	5.40195397	6.48408259	6.45135542	5.99813998	6.79103777	5.85439318	6.31159035	5.98722229
MOCS1	6.0474733	5.94528759	6.0607834	4.77806741	5.80679972	5.60677903	5.08108741	5.42037093	5.07049171	5.699347	5.98718285	5.50395456	4.78093005
HIST1H2BJ	2.55182601	3.01979261	4.02899405	4.94472948	2.70079831	3.21464112	2.86522458	1.81125187	3.81395471	4.24651757	2.80055124	3.19296774	3.70861329

MED20	4.36744818	4.18912713	4.89399969	4.80780075	4.517284	4.81921314	4.3863674	3.85482796	4.58273967	4.71521348	4.74110015	3.74846389	4.20230806
TBCC	4.72004301	4.54597332	5.06435615	4.33264044	3.98151993	4.49352992	4.0042263	3.62281585	4.14475103	3.73228346	3.73028373	4.80323886	4.3388094
SPDEF	0.67124013	-0.04407343	0.23142435	-0.04407343	0.13283672	-0.04269883	0.03469803	0.30867158	-0.13412934	-0.13412934	-0.13412934	-0.13412934	-0.13412934
TCP11	0.33506787	0.25095227	0.68798555	0.67183122	0.22587419	0.17462756	0.25178953	0.62942767	1.13755451	-0.07924949	-0.07924949	1.58522395	1.8435727
MAD2L1BP	4.39842035	2.92116781	3.89781307	4.43174382	3.49483471	3.62567499	3.55235146	3.39970952	3.89081871	4.89203747	3.13940296	4.12623301	3.0703206
KLHDC3	6.12302726	6.14242271	5.89658851	5.77963055	5.71188004	5.49408436	5.42609964	5.70977415	5.71756008	6.36875882	5.82794622	6.0125378	5.69190441
GNMT	0.50759319	0.90768633	1.21083629	1.21262509	0.98280066	-0.07278745	0.7835055	1.05004075	-0.1762123	-0.1762123	2.94413224	2.59949762	-0.1762123
DNAH8	0.21770679	0.80099623	0.3054337	0.2127155	0.22373803	0.27829243	0.23860716	0.21439816	-0.15490727	-0.15490727	-0.15490727	-0.15490727	-0.15490727
TREM1	5.8667202	3.70613571	4.4123531	5.79123897	2.07237096	1.00926083	0.99533507	1.23347817	2.52666497	2.88729983	-1.68670728	-1.68670728	4.29636428
MEA1	5.29121566	4.90428935	5.12096148	4.47846508	4.65249316	5.44693583	4.00553166	4.31054752	4.8870139	5.4322736	4.22873517	4.55101776	4.73067263
KLHL31	0.68911583	1.96237964	0.77585692	0.88427592	0.70646999	1.04541491	0.80086451	0.8832362	-0.4927252	-0.4927252	-0.4927252	-0.4927252	7.26467802
COL21A1	6.34080362	7.64070219	7.24032322	7.16891803	6.95751055	7.57961639	7.44280672	7.79358358	8.20529266	7.66306982	8.4495552	7.94034235	7.40958427
CDKN1A	7.83996791	5.67960605	7.60418902	7.00158085	6.16630934	6.37275239	6.60106047	5.09411363	5.89316982	5.42679107	6.33164632	5.98835134	4.77982402
SOX4	7.47793662	4.85097154	6.88307031	6.84788131	4.91298235	4.28346756	4.73452963	4.768338	4.48492682	4.07709441	5.15218557	4.68036383	3.77053929
GLO1	5.3148394	6.30232863	5.49622929	6.38125449	6.24650971	6.56927593	6.35145134	6.26535298	6.27531265	6.08783728	6.47617786	6.41133982	5.60181282
CPNE5	1.97335648	4.62422357	1.48374283	2.58993989	1.83723667	0.67855823	1.05785439	1.26254448	-0.76526589	3.12280188	2.96125702	2.29320631	5.20899717
KCNK17	5.60262478	4.86346181	4.53273113	1.78312699	3.29563802	4.83980208	3.41291975	4.02387304	4.60560359	5.4634953	5.20261969	5.45633471	1.51472656
RREB1	6.15364231	5.79542353	6.25182087	6.03790686	5.51059457	5.24504502	5.26964413	5.41919911	5.75209581	6.21887685	5.85314463	6.0224511	5.921626
SSR1	6.49432048	7.02155104	6.66898024	7.91987026	7.15530931	7.35149441	6.99902689	6.93561442	7.15991506	6.40424977	7.31115124	7.18731836	6.54657836
RIOK1	4.92174752	5.36714006	5.2445391	5.19816461	5.10249994	5.56850166	4.50832589	4.22877466	5.33952304	5.26142861	4.82620421	5.0494032	4.94486508
NRN1	1.20877899	2.26355034	1.21670163	2.31777805	3.96542189	2.59220361	3.51051657	3.64234364	4.8806436	4.55782034	4.39671307	1.8714223	4.72293257
SLC35B3	4.10626785	4.79226352	4.264907	5.72298241	5.04316618	4.9824793	5.00209696	5.13948957	5.03920231	4.51987444	5.05660399	5.047784	4.81857018
RPP40	2.55076483	2.01053646	3.22936987	3.35329622	2.70534087	3.2752868	2.50366878	2.62792067	3.89709462	2.38424194	-1.45247812	2.27032241	2.59094106
ATXN1	6.20670908	6.82360663	5.87757137	6.40037178	6.30576718	6.5376808	6.6680597	6.74586423	6.71507274	6.81282397	6.84101505	6.88550376	6.26833852
NUP153	6.15437496	6.51592605	5.68160589	6.57661837	6.6576397	6.34366099	6.37494651	6.60949622	6.00012211	6.13589031	5.8848691	6.60102604	6.27221507
DEK	5.51972304	5.80513867	6.02152204	6.95275358	6.49471498	6.46347731	6.78576283	6.5907094	6.82637333	6.10231031	5.87448157	6.14054296	5.8655442
EEF1E1	2.72075474	4.15473004	4.01256997	3.86036532	4.24698278	4.66735588	4.34770206	4.35174525	3.81512326	4.63431463	3.72638423	5.09226692	3.40336092
RUNX2	3.8972532	4.14316623	3.07597015	6.01356032	3.35299204	3.25983765	2.93597956	2.29277546	2.94819465	0.67609858	4.36880093	1.40691815	3.54992984
LRRFIP1	7.36882666	7.43328371	7.45722028	7.33789631	8.17826772	7.48635585	7.69305222	8.02983004	7.64583013	7.65746235	7.37418121	7.38014073	7.98952471
RAB17	2.34340416	2.87537239	1.97180135	1.06828423	2.70172942	1.24001457	2.75402492	2.27797918	2.87621266	-0.71258588	3.56819993	0.69227842	0.9171558
CXCL6	0.39544465	1.59347882	0.18498259	2.68429677	0.26082579	0.18395182	0.43762744	0.43664414	0.16854951	-0.29389582	-0.29389582	-0.29389582	-0.29389582
EREG	2.21359234	1.6413156	1.64378582	3.57715979	1.66041811	2.86535221	1.84913585	1.64446703	1.97034325	1.52128181	3.79733766	3.64702986	2.3078405
AHNAK	10.1428363	11.3224909	10.6437243	9.75208561	10.6437243	10.6437243	11.0976759	10.9137191	10.7212321	10.8876845	10.7212321	10.8876845	10.0920193
SH3TC1	3.27162164	4.4855548	4.67011887	4.32000858	2.38579563	1.70040266	2.73666462	2.49805445	3.45964569	4.23610626	3.46586618	2.88521782	2.90187334
CNOT1	6.59973948	6.7152943	5.96832023	7.07240168	6.69198438	6.72227244	6.66653529	6.61906252	6.39375084	6.36574835	6.37542117	6.31911585	7.03487243
LRRC29	2.70625948	2.59663344	3.45123783	0.957471	2.03145477	0.47215818	0.81948308	2.43331811	-0.480026	3.41606939	3.56025366	2.44461234	1.73891818
BBS2	5.43059983	5.71079156	5.26264341	4.42464385	5.32789715	5.53660005	5.61564818	5.91356021	5.46204318	6.01923403	5.73977354	5.9211669	5.92964602
MT1G	5.53208021	2.41744544	5.50466346	4.4395505	0.50510002	0.38243288	-0.36148432	-0.35927957	-0.94088972	-0.94088972	-0.94088972	-0.94088972	2.74722359
MT2A	9.01014071	5.95973123	9.09406625	6.88224152	4.88429106	5.84919326	5.83670047	4.27979722	4.6071639	5.03215305	3.98853421	4.566719	5.48374612
C16orf70	4.69063734	4.40897891	4.13240517	4.21862464	3.99884082	3.99217865	4.42975438	4.15077399	3.32539181	4.46749909	4.10456991	4.92478948	3.52696322
GOT2	5.76990302	5.59126482	5.62160496	6.08930366	5.56184479	5.51948839	5.64104241	5.25116673	5.65893604	5.57296484	5.94032822	5.77035208	6.94585615
DOK4	5.19748522	5.59543276	5.52151675	4.66203101	4.84881079	5.11642355	5.12916454	4.92355892	5.37055008	5.54866784	5.41512461	5.58172625	5.09739256
GPR18	0.68444218	0.31882417	0.56025413	0.7279725	0.67189856	0.48713724	0.59686475	0.13981568	-0.10262421	-0.10262421	1.00271457	-0.10262421	-0.10262421
CLYBL	0.43196786	0.22084098	0.01612761	-0.37635455	0.39094954	1.24980738	0.27118661	0.51957208	0.17500726	0.17500726	0.17500726	0.17500726	0.17500726
TMTC4	3.77816802	3.61431219	4.11034101	3.56689835	2.72891025	3.2675602	3.77208813	2.20761492	3.58084106	2.83376028	1.96930091	3.06126668	3.51166297
RAP2A	5.84291183	7.05583559	4.31088373	6.66760144	6.61906556	5.99000803	6.57711187	6.27989443	7.28530167	4.54292362	6.53231613	5.9466288	5.52438524
ABCC4	4.31062585	4.48350191	4.52690763	5.81181779	4.2831265	3.66590588	4.76406956	3.907088	3.70027286	3.72157445	4.55324369	3.69909318	4.9033626

EFNB2	5.21112338	6.0800838	4.96668863	5.40850089	6.32420572	6.39483556	6.12430281	6.1513133	6.34083846	6.12787899	6.26735398	5.8800385	6.44524902
TM9SF2	5.21947066	5.88994377	5.47294197	6.48715838	6.31278777	6.31927526	6.23047108	6.1934395	5.67535426	5.62120521	6.21630188	6.02640242	5.46331452
C17orf53	1.35093008	1.08606827	3.69433274	2.26470322	0.5530272	0.32974899	1.15251243	0.56331148	3.02674984	2.90229859	-0.65647743	2.22638406	-0.65647743
IRF1	5.40300036	5.6252658	4.6292373	5.60986222	4.17620471	3.73797207	3.82933335	4.0479663	4.18303215	4.14238549	4.37475269	2.92257625	4.71955996
UPF3B	1.50402481	-0.27683061	0.94168379	1.2837034	1.18639718	1.500641	1.25831504	1.45418716	0.04471239	0.04471239	0.04471239	3.25319521	0.04471239
RNF113A	4.9399951	3.38138925	4.37710133	3.34052393	3.31564509	4.46161486	3.24498806	3.07341596	3.75189	3.64238036	3.38821778	3.4076013	3.89503146
NDUFA1	3.93988277	2.86364633	4.80723853	4.24350217	3.43645496	4.11955695	3.57415873	3.44264503	3.8342719	4.8327434	2.49898366	3.35536279	4.56802385
ATP5S	3.4136136	3.48204846	3.15431057	3.52213776	3.7398936	3.72854724	3.99841348	3.98898596	3.28376245	3.48649171	4.02154811	2.7905247	3.55844571
BMP4	6.03274243	6.32004318	4.73212661	4.58481527	4.91273215	5.13850533	4.91528122	4.89937783	6.21827311	5.39319182	5.99110594	5.25910058	5.56061696
PTGER2	1.51038566	2.31584122	2.09716618	1.85039519	1.09543705	0.87578266	2.1039565	0.93705272	-0.0379133	-1.1258023	3.23584395	-1.1258023	2.57454766
FAM193A	6.13312215	5.42019988	5.97083099	5.56848613	5.99153681	5.54925144	5.85518478	5.90435481	5.36404457	6.01710424	5.81815681	5.91301916	6.21523691
GRK4	2.9909593	2.55444392	3.83819469	3.25411291	4.2991544	3.11156366	3.95053316	4.08636438	3.76614434	3.79455891	4.04485979	4.17559105	4.13330736
SOX9	3.89455462	4.02193141	2.912715	2.58351455	2.10441669	2.22625414	2.14510575	2.22158564	2.87761599	2.42951459	3.08356751	3.32266519	4.30526524
TEKT3	0.62202763	0.86353893	0.87971002	0.74663422	0.67580248	0.81240352	0.99306887	0.96183996	-0.31945353	-0.31945353	3.27602183	1.91404856	2.13986056
HS3ST3B1	0.52276514	0.12768188	0.36593429	1.11413158	0.12841799	0.65583154	0.68298837	-0.14106353	1.74856627	-0.18781759	2.70632137	-0.18781759	-0.18781759
SLC25A35	1.00831562	0.25609963	1.77678058	0.55134774	0.32214567	0.442185	0.70744987	1.1318205	-0.02385918	-0.02385918	4.04599602	-0.02385918	2.30741085
MRPS7	4.87293793	4.3881128	5.40312767	4.76234049	4.52260728	5.07559947	4.47720409	4.1226666	5.2059139	5.35568045	4.50461343	4.54643687	4.61947286
GGA3	5.67576894	5.16581114	5.47195394	4.95424741	5.12540621	4.68565523	5.04545287	4.99147992	4.60406962	5.42599866	5.2819233	5.4564451	5.33402162
ARMC7	3.71351644	3.27538789	4.28700916	2.3325244	3.03583356	2.61482844	3.25551349	2.93400519	3.28947325	3.46368843	3.44084476	3.28607945	3.74457204
NUP85	4.88532132	5.0414276	4.92802479	5.07473364	4.87158602	4.20834681	5.01857908	4.99007594	4.68307555	4.82933876	4.80126552	4.90769619	4.85554341
SLC25A19	3.92618144	3.13300648	4.15580545	3.75228785	2.30792307	2.98469019	3.21571538	2.66273366	3.41316325	2.6757433	3.25810339	3.85932657	2.44047712
MIF4GD	4.92022035	4.83594582	4.83945918	3.65793093	4.50824089	4.35384078	4.53744633	4.29090176	4.33525143	4.88035848	3.66845718	5.15727127	2.91009387
NT5C	4.28864131	4.43894515	5.02376639	3.81141354	3.86929699	3.98688308	2.94111772	4.19173978	4.47244365	4.86672446	3.76957923	4.17464902	4.37538996
MSTO1	3.68950352	4.38782726	3.71970496	3.77111462	3.669218	2.93808975	3.02101965	3.7294152	3.62967122	3.38249836	4.25048451	2.87164607	4.15180366
C1orf61	0.04606918	0	0.1173386	0.0621447	0	0.0456106	0.01659492	0.00158797	0	0	0	0	0
TTF1	5.21316276	4.79732876	5.1161584	4.56468992	4.90738682	5.62700377	4.78014366	4.90948006	4.97291867	4.99886825	4.16658551	4.71820878	5.35753035
GTF3C4	4.80602748	4.98950036	4.79893523	5.31965793	5.057561	4.85021566	5.10714669	5.20759725	4.86986642	5.34630703	5.03325671	5.47856888	4.38480878
DDX31	4.51496502	3.86108865	4.54105297	4.44306486	5.04223116	4.14204982	4.00818667	4.18199928	4.47295486	3.46684693	4.99508068	4.64780512	3.92065964
PPP1R12C	5.30778595	5.44934634	6.51885945	4.71712875	5.95299111	5.49115174	5.88246278	5.897359	5.40162573	6.41536372	6.18962397	6.47384182	5.31384679
MBOAT7	5.31082711	4.1537218	5.1783553	4.60627923	4.05098255	3.24983371	4.20982044	3.1962381	3.70528573	2.81898599	3.89528807	4.06069083	2.53603269
OPRL1	1.35806397	-0.00141461	1.31244754	0.48606638	-0.25470325	0.67043746	1.3383582	-0.68526104	-0.09007431	-0.09007431	-0.09007431	-0.09007431	-0.09007431
SLC2A4RG	5.4872538	5.20454469	6.31262084	4.2931672	4.68877257	4.98232367	4.18939835	5.09193985	4.9354677	5.77000198	5.68337987	5.54154596	4.32986795
PPDPF	5.47914038	5.13379421	6.72551936	4.72095525	3.90565853	5.01261872	2.91414282	2.6269414	4.41015841	6.14395012	4.81478999	5.03312711	-0.06172195
IL1B	3.98868769	3.95723357	2.92123727	5.4726716	1.60513765	2.29651926	2.06897317	1.28801597	4.30570092	3.32341853	2.5746819	3.2954287	4.52331133
CHCHD5	4.40938667	2.83423805	5.22159667	3.08046626	3.36552619	3.4922409	3.52442332	3.83734238	3.41899436	4.32277279	3.16528156	1.87183177	1.77033732
PAX8	1.47835862	1.83132195	2.87659994	1.36857896	2.06088133	2.02807519	1.07267289	1.32040505	1.12960993	2.95980798	3.03270061	-1.10320805	1.42904706
INSIG2	5.12347557	6.07552614	5.11153977	5.59810959	6.0307028	6.0615457	6.3953786	5.73855671	5.97684762	5.26280041	6.15687311	6.00967397	4.78353138
POLR1B	4.75675825	4.71644734	4.5875196	5.4034128	5.32277367	5.29899375	4.51993943	4.66631884	4.59501251	4.74950943	5.21162239	4.53571773	4.6420311
CCDC93	6.32763273	6.32587744	5.71980835	6.13357391	5.91113695	5.64463509	6.17779404	5.94965644	5.89989229	5.61696736	5.90146598	5.79469923	6.19927479
PSD4	3.94702271	3.6879127	4.03065732	3.78009096	2.91389578	2.02146131	2.75343427	2.33885781	3.73667283	4.06692613	3.76279556	2.44621407	2.62881493
SLC25A23	5.96475755	5.75837258	5.43856627	4.25422778	5.74761544	5.65912343	5.99359438	6.31176127	5.94373135	6.66740671	5.21930033	5.76211309	6.2820029
PSPN	0.63148694	-0.60239264	0.46911691	0.06362572	0.58788134	0.97400107	0.5700906	0.39317898	0.017905	0.017905	0.017905	0.017905	0.017905
GTF2F1	5.96557184	5.36030838	6.02557141	5.64202155	5.5157418	5.03489326	5.22130467	5.24383051	5.55384727	5.94978267	5.53362069	5.3005926	5.07153436
ALKBH7	3.83524254	4.7596368	4.04412306	3.27950369	2.47940347	3.00242175	3.27280214	3.68814895	3.66684894	4.85091868	3.78843566	4.81435062	2.85679312
CLPP	4.87014823	3.89352915	4.75273769	3.53259563	3.80620566	4.54643682	3.62861369	3.87611487	4.14340148	4.44800137	4.35020279	4.47591882	2.95546847
TNFSF9	1.25118268	0.3946963	0.68090293	0.44469044	0.58347778	0.15733464	0.35395894	0.44117447	2.97814826	-0.32597024	1.61707231	2.23555182	-0.32597024
GRIA3	4.21381156	2.68195484	3.97693318	4.00767466	3.41857185	4.70419057	4.93439635	4.33056652	3.35389289	4.57435608	2.68166029	4.84908758	3.81544969

THOC2	4.02151384	4.72185669	4.17782084	5.23711125	5.22776487	5.17930106	5.24332512	5.25829792	4.59638048	3.99750716	4.46850277	5.08437618	3.93856113
MED1	6.48876497	6.42922595	6.05345161	6.46164332	6.46128781	6.87099618	6.22825542	5.96334569	6.17901748	6.37497183	6.41159324	5.92814221	6.86614618
RPL23	8.09877666	8.3379093	7.19493461	7.75983255	7.54887164	8.18477827	7.1735622	7.6281075	7.91315012	8.09093052	7.26717116	7.48882589	7.42264224
ATG4C	3.95141211	4.60275828	3.80818166	5.0214191	4.64817579	5.37696104	5.18840757	4.76772247	5.26697425	4.92143075	5.199601	4.48873503	3.73498864
CD70	0.05923181	-0.16716423	-0.16527517	0.95923647	-0.16716423	-0.16611394	-0.16716423	-0.16475423	-0.09962601	-0.09962601	-0.09962601	-0.09962601	-0.09962601
C3	4.98209878	10.9446933	2.60766708	7.25934827	7.39267198	4.22427398	6.95976487	5.45429495	7.19029565	7.86192189	8.82037354	7.208282	8.96269143
SH2D3A	0.66854791	0.14597646	0.16214564	-0.02097213	0.22511066	0.03334709	-0.0367731	0.69371276	-0.03651766	-0.03651766	-0.03651766	-0.03651766	-0.03651766
TRIP10	7.19827724	6.62310191	6.78518322	5.70626553	6.73351014	6.40518424	6.95447183	7.24395212	6.31950535	6.5970383	6.51694782	6.5484086	6.71209076
GPR108	6.47138385	5.29038388	5.73865032	5.13968358	5.24779021	5.2191074	5.55469228	5.2863591	4.99437903	5.45919067	5.33238847	6.0592752	5.27950384
TNFSF14	2.38570346	3.67903913	1.77893344	2.27446272	1.58931201	0.93602237	2.37824185	1.52548838	2.71198849	2.63907295	2.65393024	-1.26021842	3.86180007
FOSB	4.35535834	7.14696419	4.00814017	7.34591968	5.5678856	5.44835496	3.02437343	4.24682607	6.66237481	5.56701712	4.08828501	5.83284861	7.6074892
OPA3	5.35506051	4.97066465	5.71409747	4.64554969	4.68158442	4.45993913	4.53899769	4.55296165	4.62838977	5.00452582	4.35722277	4.85944017	5.02708073
SNRPD2	5.82200171	6.01106291	5.35114666	5.96053663	5.17173521	5.3054987	4.59685533	4.66138757	5.39270542	5.25330327	4.26724114	5.75717464	4.96395289
RTN2	3.83150032	3.13683172	3.36225058	2.00866925	3.06662376	3.35391501	3.12730305	3.93620601	2.85015042	2.54949507	2.9637046	3.76546213	3.45286936
EML2	5.2339944	4.66229145	5.33306573	3.8028825	4.7066974	4.86129124	4.89783647	5.33077466	4.56298502	5.59294184	4.6481664	5.53085475	4.68929597
VASP	5.16587724	5.56106211	6.51908956	5.3846592	5.97144714	6.05248542	5.23637303	5.58981711	5.77928408	6.56302734	5.98738753	6.06784015	5.48430694
SYMPK	6.00476764	5.27872676	6.06726735	5.58578671	5.67832661	4.98989493	5.75090807	5.62408603	5.2974446	5.81508812	5.84395525	5.30621051	5.60111563
GPCPD1	4.70862351	5.38008235	4.52541503	5.93562876	4.79023688	5.32393791	4.88492691	5.03112422	4.41626842	4.65192304	4.88659363	4.78667089	5.02620937
SDCBP2	0.98996731	0.16311752	1.14403893	0.17366133	0.09489905	-0.44113329	0.31135343	0.39701356	0.94169346	0.15367148	0.15367148	0.15367148	2.07663936
PANK2	5.30116863	5.08553493	5.25336554	4.93086234	5.00393352	5.06817153	5.68336479	5.56060471	5.07647877	5.74056981	5.25573676	4.90212858	5.66718985
TGM3	0.87928561	0.06270544	0.19987457	0.06270544	0.06270544	0.30278543	0.06270544	0.06596293	-0.16585087	-0.16585087	-0.16585087	-0.16585087	-0.16585087
GNRH2	0.25861997	0.05337822	1.56698865	0.30645197	0.15109249	0.14212107	0.00972975	0.92685045	0.92348522	0.00660882	0.00660882	0.00660882	0.00660882
CD93	6.36980513	7.72171316	7.15530851	8.0022799	6.88358665	5.73002589	6.80326041	5.96233454	6.23412602	6.50435326	7.32021224	6.52504752	7.24992617
GZF1	5.06720779	4.81496009	4.92944209	5.70835687	5.18899617	5.64438683	5.18597305	5.06301783	5.36118222	5.21480221	4.78644486	5.11593825	5.41560832
NAPB	4.21063258	4.51901349	3.79210434	4.8289893	4.78660587	4.62070346	5.07090162	4.7030012	4.14654261	4.89994849	4.40692643	4.78023346	3.60990209
CENPB	6.12896981	6.23295061	6.85555337	5.66849783	5.7334158	5.41775515	5.62929805	5.96486509	5.8290783	5.90311522	6.02360833	6.19999118	5.62056333
PSMF1	5.74418848	5.37853408	5.57887058	5.18793627	5.54760459	5.78622713	4.7948061	5.2648161	5.46169583	6.06414036	5.75902423	5.51150662	5.37339372
DTD1	4.75957755	4.48218526	4.65157288	4.10031701	4.25329869	4.76325698	3.82640829	4.05219173	4.60881818	4.43561304	4.37020109	3.18731022	4.48630038
RBCK1	5.58142155	5.00173927	5.64644451	5.00227558	5.34256319	4.53271103	5.14985684	4.8289839	5.27523532	5.35604055	5.62726203	5.50261989	4.52518195
TMX4	6.39189854	7.10009505	5.95862703	6.4191532	6.52542541	6.59672708	6.34832486	6.97090104	6.64216155	7.10171228	6.80752656	6.96486615	7.1772209
STK35	5.75557064	5.21016566	5.30338757	4.86304547	4.65520905	4.43507501	4.65240917	4.00873374	4.89532913	5.05947161	5.10220226	3.87027382	4.99334514
SNRPB	2.9081619	4.15259379	4.91744017	4.8331247	2.99952975	2.60595156	3.01186086	2.7056623	4.35999584	4.84911116	3.32078754	4.6421435	2.66146204
NRSN2	5.37849672	5.18381465	5.39860677	4.61989981	4.6708703	4.0571691	4.45087817	3.88676209	5.31387478	5.46114677	4.44077352	5.1291567	3.8826016
RRBP1	6.52604201	6.29347027	7.37132733	7.29365648	5.99361183	6.21342756	5.64763509	5.96793789	7.07522407	7.02051245	6.99412414	6.15849909	6.69743536
BMP2	5.36328134	3.55173526	5.86700536	4.61083226	3.09517615	3.94296465	3.15703714	3.47556126	2.24241763	3.60057873	2.68867246	3.85957075	3.17184176
ZNF133	5.01087429	5.0164745	4.20176004	4.58145686	4.44618848	4.56500109	4.51889585	4.89198826	5.09938411	5.13300374	5.06066473	4.66655383	4.73599904
FLRT3	1.03460453	4.66859456	0.89683176	1.71461932	0.88612218	2.0822113	1.19349605	1.60973843	-0.65343572	2.9860399	3.86598743	3.53920698	2.46759215
PCSK2	0.44800635	0.44800635	0.59008469	0.44800635	0.84848453	2.21211915	1.1617022	0.53747425	-0.21043349	-0.21043349	4.52483374	1.97327329	3.74881851
GFRA4	0	0	0.00124472	0	0	0.1952302	0	0.66237683	0	0	0	0	0
MKKS	4.67153549	4.88604113	4.22855642	4.89223814	5.07629441	5.29336862	5.07897839	4.83800686	4.68298717	4.92253445	4.90494114	4.88079523	4.71547231
BFSP1	1.7820056	1.31529033	1.92945535	0.97634395	3.25192506	3.17960825	3.0398938	2.16859007	4.32605106	3.50625128	2.83229701	3.57895155	1.88251961
DSTN	5.63740951	6.88346746	5.6812603	5.88255058	7.83102842	8.09967674	7.80753792	7.6531734	8.33120752	8.28623746	7.31351331	7.45001804	7.57632971
SNRPB2	5.1506061	4.034574	4.33810792	4.71617207	4.9507019	5.74020455	4.77203056	4.70007034	5.41191461	4.78830586	4.61914626	4.66503321	4.4695946
LRRN4	-0.13007938	3.1493261	-0.12901	0.88552628	-0.11437969	-0.07253826	-0.13007938	0.06930803	-0.04723982	2.68676521	-0.04723982	-0.04723982	2.91797936
TBC1D20	6.30254325	5.70081699	5.95879133	5.61893287	6.10628682	5.86142749	6.33946376	6.03296135	5.55263931	5.60435595	6.00830847	6.25033092	5.97266507
ITPA	4.21861556	4.45899596	4.90185884	4.4596315	3.54389079	3.28651487	3.51469286	3.82810336	3.38237331	4.07335476	4.44063752	4.45725005	3.69383981
TCF15	0.31523867	0.62849595	0.75684605	1.08759424	0.89930888	0.31665307	1.28020255	0.31848415	0.67545928	4.21631842	3.23066356	-0.07080029	-0.07080029

MCM8	3.4338747	3.57189429	4.22898125	4.24481337	4.59801654	4.21619191	4.44426121	4.19669023	4.34706024	3.91671595	4.53833422	3.61527132	4.5306453
FAM110A	2.06533881	0.48523664	2.84874267	1.13248777	0.15345074	-0.22734003	0.37053873	0.06426875	0.76829437	2.56518259	-0.16973536	0.8817724	-0.16973536
SIRPD	0.52241601	0.23874425	0.94974155	1.34538376	0.23874425	0.54145316	0.31383345	0.24198894	-0.45376914	2.83173195	-0.45376914	-0.45376914	-0.45376914
MRPS26	4.59037469	3.47237891	5.10529905	3.4267653	3.51787228	4.54427295	3.61909453	3.07938785	3.99505368	5.35382837	3.09398816	4.56449121	2.10874556
S1PR4	0.81231613	-0.10722708	0.64888922	0.46163645	0.17272745	-0.1455497	-0.2377664	-0.32292075	0.03590228	0.03590228	0.03590228	0.03590228	0.03590228
NCLN	4.77372338	4.28318353	5.52254309	4.73389862	3.95191143	3.84232332	4.20896707	3.48013473	3.9574447	4.16846122	4.45498766	4.39278216	4.10836084
CITED1	1.21524564	1.06789278	1.9387297	0.58962823	1.43392931	1.23611902	0.58962823	1.64185344	-0.39190514	3.54832367	-0.39190514	-0.39190514	-0.39190514
HNRNPR	6.27749035	6.59087079	6.43354478	7.58846783	7.18495623	7.29860045	7.08967282	6.95089328	6.74613822	6.8195977	6.53952771	6.48190538	7.11924728
ZNF436	5.55232077	5.75495739	5.06295352	5.91610281	5.37328085	6.16371732	5.56611131	6.05668096	5.58538	6.07505832	6.14158243	5.695412	6.28848942
MAX	1.92666257	1.55803992	0.92920698	1.74590996	1.04653317	0.92175246	1.87628596	0.37216833	0.5726112	0.5726112	0.5726112	0.5726112	0.5726112
ARMCX5	4.59629643	4.35497627	4.71369786	4.38680993	4.7750457	4.94675058	4.81093953	5.20328935	5.20818638	5.07987877	4.67513824	4.96269157	5.19067286
GDF5	0.92555202	2.04871417	1.77154856	1.95831042	-0.09220167	0.68289984	0.11440046	0.1693141	-0.39760616	-0.39760616	-0.39760616	2.14203125	0.64824672
MMP24	0.91654488	4.35958534	1.24186022	1.46036245	1.18542276	2.00387159	1.07474911	1.75693162	2.10085151	2.99773101	3.18606435	1.82177967	-0.64455188
NECAB3	3.50301743	3.3765903	3.95732691	2.51510302	2.48491814	2.53230267	3.76801636	3.13688213	3.1566521	4.16382086	1.56536033	3.99979571	2.142253
ID1	6.34978002	5.64127471	5.88623654	3.69776245	4.54342527	5.17254186	5.6923559	4.76765097	4.38758771	6.01025572	5.31532172	4.43862593	5.12546934
RALY	4.28369642	3.72365581	3.76078035	2.94644508	4.20874708	3.69873502	4.29228966	4.2123186	4.72104893	4.6594725	3.84001757	3.92744657	4.54255303
DYNLRB1	6.54283527	5.38848883	6.21801689	5.48179583	6.52298899	6.4659749	5.85981039	6.13914241	6.58456624	6.67886614	6.00373132	5.9104122	6.57495872
EIF2S2	5.7221935	5.3254154	5.90956371	6.57722494	5.92645364	6.44309765	5.78529227	5.65402909	6.12253875	5.99181909	4.98070726	5.24845596	5.68054615
ERGIC3	5.54803874	5.24914787	5.39428027	5.64474957	4.60709925	4.58617297	5.01019068	4.94848393	4.65598307	5.61448713	5.42730425	5.23051665	4.58903448
ROMO1	4.11704697	3.71169374	5.02919288	3.28469021	3.84501327	3.7054831	2.65628181	2.61847888	3.82286055	4.20625127	1.2835455	3.28784581	3.11162185
CEP250	6.17914905	4.76033815	6.22032576	4.79099454	4.87873966	5.23339567	5.20279904	4.96413604	5.29167966	5.30167391	5.2894178	4.72467842	5.62755256
PLAGL2	5.73679246	4.49716376	5.22708458	4.19615836	4.91589153	4.50415212	4.66900928	4.63400695	3.68942224	4.26836342	4.35600446	4.82742121	4.18147166
GRPR	0.58523702	0.00569001	0.99967198	0.56235502	1.00965431	1.49174499	0.727568	0.0088144	1.69504848	-0.23947486	2.990088	3.11219869	1.98773062
KDM5C	5.87142106	5.2991445	5.95439202	5.18637372	5.23785837	5.02050433	5.15424736	5.38551518	5.28075263	5.61625584	5.81196998	5.55581519	4.82931685
AMOT	5.51196927	4.98242984	4.82177132	4.2144252	5.2003214	5.45849374	4.75765789	4.94853595	5.21591417	4.13912331	5.570916	4.54710226	4.73308088
TMEM115	4.98335807	4.88306572	5.30611183	3.93680711	4.41971704	4.67369557	4.21614055	4.19358063	4.67175779	4.86234991	4.95027322	5.15291436	4.19585216
PSMB2	5.70406526	5.59364204	5.56463437	5.98407473	5.52709093	6.06381966	5.16970674	5.21078622	5.32476239	5.7365795	5.52185174	5.45542871	5.57327327
UROD	5.26240658	5.28429502	5.30364138	5.37528744	4.62580582	5.16452901	4.66923548	4.53087118	4.99938515	6.03941381	4.4673491	4.98578944	5.04099977
ST3GAL3	2.0207795	2.02522419	2.04821943	-0.06795484	1.69850657	0.99375351	1.25105059	0.91892134	0.83110989	0.37322013	4.39366876	0.37322013	0.37322013
TMEM53	3.80904578	2.70245829	3.73664842	2.75279056	2.84047987	3.7406019	2.44107991	3.26889975	3.3478587	2.61874745	3.62333803	2.96448678	2.80811752
HECTD3	5.83643744	5.25597683	5.61822648	5.07344048	5.37060262	4.56712053	5.56120176	5.51436012	5.03296422	5.85603258	5.46296499	5.49404263	5.67289705
KLC1	6.56650146	6.49159181	6.52727103	6.17677213	6.84810557	5.8578232	6.61044688	6.7414253	6.62186817	6.62363278	6.81195274	6.34680759	7.03029659
XRCC3	2.48133927	1.44191517	3.88461446	3.02794345	1.23154989	1.23517505	1.5471876	1.27352559	1.75471938	2.45659866	1.7309849	-0.75946828	3.20029182
TUBGCP3	5.59790658	5.48593486	5.11151007	5.3693358	5.30816471	5.14890918	5.62240735	5.81365435	5.18104877	5.81948614	5.60449961	5.53603414	5.78713949
MCF2L	-0.10438765	0.29013087	0.69137101	0.4664362	1.88396231	0.04084317	1.03767167	1.90361421	0.42679054	0.42679054	0.42679054	0.42679054	0.42679054
F10	4.26000178	1.334552	3.10989719	1.79805159	2.92403447	2.60123351	2.75946036	3.12343085	4.20549276	4.18383818	2.48790846	3.63327393	3.50381909
PCID2	5.65955182	5.37160639	5.00608571	4.84948521	5.37422974	5.00416959	6.00890866	5.46598122	4.87698462	5.24478573	5.60113371	5.30436539	5.75634958
LRFN3	4.79756062	3.57765136	4.90084604	3.25537595	3.97395168	3.79372052	3.94117576	3.95875157	3.36833065	4.71722152	4.28157537	3.72857579	3.69595564
IGFLR1	2.36994571	0.99062562	2.83216358	2.04395147	1.30084675	1.70700781	1.51011018	1.58958135	2.84173837	-0.93975909	2.51300712	2.60489873	-0.93975909
CAPNS1	7.83502679	7.39706415	7.90254372	7.76115936	7.33925173	6.96048813	7.60399358	7.32554152	7.43055237	7.88775654	7.51179491	7.62001358	7.73689986
PDCD2L	1.87509684	1.82346761	2.62722072	1.27609971	1.78920875	2.87467869	1.82841866	2.33291089	3.59844104	3.38326339	-0.86718681	3.76192064	3.40535936
RBM42	5.32587048	4.84631054	5.85973796	4.44425169	4.55190159	5.23132084	4.20738206	4.59730995	4.7889239	5.40985186	5.13625066	5.37912516	3.64592817
UBA2	6.40634268	6.88567793	6.34872315	6.88419669	7.17184738	7.55541701	7.27515642	7.09523805	7.04534156	7.45278916	6.91937273	6.9210993	7.15586836
FFAR2	1.80612985	0.90574428	1.54293671	1.19943365	-0.05100808	-0.04968248	0.28983069	0.08213638	0.40831647	3.51202066	-0.27813112	1.49680532	-0.27813112
HCST	0.97252853	1.11141039	1.87342793	2.17462799	0.09224077	0.40178797	-0.43745667	-0.95602771	0.22145369	0.22145369	0.22145369	0.22145369	0.22145369
COX6B1	5.00708687	4.71218513	5.37258127	4.35926916	4.36584511	5.42401215	3.69167131	4.64321843	5.04554266	5.05104599	4.9000559	5.11610539	3.90205618
THRA	5.84746459	5.79036492	6.27195404	4.50713988	5.78467324	6.06789144	5.37667291	5.9461133	5.98456185	6.51472194	5.81933038	5.86690892	5.78979001

CCR7	2.08618497	0.64769489	0.03420477	0.95277994	0.0319082	0.18233431	0.0319082	0.03483808	0.60319452	-0.15260882	-0.15260882	3.52346963	-0.15260882
NR1D1	6.39838405	5.26475639	7.02878067	3.77623135	4.05513275	4.66599454	4.0493488	5.18405899	5.12826758	5.91696921	4.77631101	5.164805	5.67589045
FRMD8	4.37500477	4.77247004	5.07396034	4.42825025	4.00356679	3.03394221	3.94451506	3.75321542	3.78518524	3.87002338	4.46307091	4.18931664	3.15808471
PRDX5	5.34471254	5.56789498	5.6590902	5.52220664	5.20832932	5.98427138	4.45024184	4.76031693	5.17195479	5.40584458	4.69446659	5.11719834	5.44796985
BCL2L12	1.25437763	-0.19617175	3.5733707	1.29438011	0.90841101	1.4043219	0.84521708	1.18818674	2.6168795	-0.28445353	-0.28445353	3.05630861	-0.28445353
IRF3	5.64619098	5.31709527	5.84824611	4.8303796	5.13454397	4.60786308	5.16758767	5.07676256	4.59851987	5.48051558	5.67895667	4.82839909	4.8976207
PRMT1	5.68013593	4.96082338	4.95702372	5.29298611	4.64586934	4.57722137	4.49389767	4.28963801	4.85465394	5.4054366	4.46199157	4.67468521	4.1907601
RRAS	6.25970948	6.43394519	6.29698322	4.68302204	6.17055013	7.14475547	5.89736491	6.55868489	6.55211093	7.19858163	6.55924366	6.76261196	6.13917519
PRRG2	0.40383965	0.86800101	0.44272347	0.20194376	0.35379801	0.42395628	0.6370329	0.43203114	-0.10593348	-0.10593348	-0.10593348	-0.10593348	-0.10593348
SCAF1	5.27898809	4.83719273	6.33519312	4.27889924	4.94551465	4.67641622	4.68765423	4.72727566	4.26778143	5.6957094	5.24553978	5.79088169	4.69071023
PRR12	5.41159456	4.45806765	5.92611995	4.06064054	4.78573607	4.65700478	4.68017444	4.70548648	4.49419108	5.4746404	5.45613543	4.86660543	4.77573715
TSKS	0.04191872	0.82199351	0.19486868	0.30527779	0.29184789	-0.09033621	0.60904362	0.16731405	-0.07855082	-0.07855082	3.61569847	-0.07855082	-0.07855082
FLRT1	0.16694743	0.14987241	0.77537162	0.14987241	0.6972939	0.39176031	0.27200179	0.45210362	-0.19333447	-0.19333447	3.6808519	-0.19333447	-0.19333447
ASL	4.40874188	4.03031504	5.20197069	3.77913448	4.06587635	4.07856027	4.23929759	4.60234213	4.88461576	4.06239287	4.67049598	4.0017999	3.93724637
SBDS	7.19054643	7.85590625	7.44046197	7.43947105	8.23250459	8.69000418	7.70351804	8.08924608	8.16080271	7.90066097	7.67522614	7.91449512	8.12499368
STAT5A	6.09662743	5.83624881	6.12587682	5.22747756	5.34208238	5.17660635	5.704347	5.35180289	5.59173186	5.63380197	5.59974089	5.34068958	5.42097819
WNK4	-0.01006727	0.24512113	-0.04483506	0.47767772	-0.04736336	0.05588452	-0.04736336	0.38457025	-0.08408291	-0.08408291	-0.08408291	-0.08408291	-0.08408291
BECN1	6.13896312	6.06815637	6.20608113	5.52967255	6.02947083	5.82759534	5.86473885	5.82579954	6.0449176	6.25916441	5.63755007	5.88073191	6.03409631
PRKCG	0.06406208	0.29045078	0.44979981	0.122382	0.04711051	0.31532029	0.1598967	0.12846767	1.70474088	-0.08702692	-0.08702692	-0.08702692	-0.08702692
TRAP1	5.42858998	5.23403331	5.93969922	4.88031066	4.82901568	5.22683124	5.113781	5.24757778	4.92162493	5.85057822	5.30032224	4.56044556	5.63394545
GLIS2	1.98345262	1.76572125	2.63107103	0.56973727	0.63104594	0.88135164	0.36150556	0.70975504	3.12690434	0.59066777	0.59066777	1.6895266	0.59066777
NSRP1	3.84095399	3.43280097	4.53925316	3.80568309	3.77372663	4.27058912	3.94749078	3.96503791	4.9172189	3.89988308	4.18681714	3.50992066	4.8132061
DNAJC8	6.93752399	6.52454388	6.73359247	7.00641815	6.77738698	7.53822869	6.75003868	6.41438431	7.05704438	7.3254519	6.08966606	6.53296753	6.87851688
AHDC1	6.75381652	5.41495879	6.82900074	4.89846151	5.45598159	5.33367592	5.64154564	5.31186473	5.34749986	6.48979108	5.8491761	5.42217353	4.70545882
IFI6	5.1519015	5.23868293	6.47351006	6.44580121	4.84279144	5.09808679	3.97261073	3.88176363	6.24024468	5.39387509	6.56828754	4.493509	5.53874413
DACH2	0	0	0.04264243	0.00198519	0.05688187	0.15547032	0.01536318	0.12723359	0	0	0	0	0
ZNF384	6.19589389	5.11226902	5.79715918	5.15641616	5.52500188	5.32518618	5.54040332	5.66497433	5.14198404	5.77118033	5.42151014	5.13548805	5.90184227
EMG1	3.76827445	3.78639608	4.2537751	3.67349084	4.10306158	4.11944991	3.75873366	4.25141697	3.62309854	4.64771304	3.21993821	4.0439519	3.91076053
UXT	6.28411303	6.33013167	5.42761978	5.86056999	5.86829456	6.15414718	5.69142053	5.36761935	5.65767522	5.77397561	5.15550761	5.97559976	5.30577814
CFP	1.12805634	0.77554432	1.42073873	0.97787052	0.37505955	0.48442257	0.17274262	0.88374486	2.35430068	-0.55344867	2.21452876	2.05863882	-0.55344867
ELK1	5.0354726	4.04858065	5.04650282	4.09210189	4.55475505	4.40299652	3.9939008	4.60096721	4.57049948	4.92758645	4.71520711	5.25221973	3.51697612
TIMM17B	4.69845234	4.06996867	4.42739331	3.45348732	3.66057678	4.33537893	2.93407711	3.4272346	4.32961429	4.14374605	3.25695883	4.38334327	3.32645531
ATG14	6.10201463	5.46423296	5.36129349	5.53226601	5.97786031	6.50360465	6.19005862	5.91666121	5.50682113	5.86040093	5.6052229	5.80304459	5.41134274
KTN1	6.50470175	7.22855605	7.00929467	7.67514287	7.66988562	8.06530593	7.66136241	7.71832522	8.39536045	7.37514391	6.71551902	7.24936579	7.1175179
SIX1	1.50598104	1.2523407	1.16264039	1.84595383	2.55296526	2.55258967	2.70365239	2.21231041	3.90531093	3.26294817	3.76602795	2.14780126	2.16560545
RHOJ	5.69007903	5.29655089	5.41425559	6.45209329	5.78660695	5.05812569	5.39228876	5.73981627	5.93659538	5.59830664	6.39168262	5.6936982	6.03549591
DLGAP5	1.42346581	1.52220431	3.20945707	5.23707192	1.25940978	1.26044934	2.21884534	1.629065	2.7026607	3.11953523	-1.22416543	2.16376547	2.29095546
HSPA2	4.57877921	4.89621046	4.76632417	3.76048966	5.53208663	5.39943728	5.02105307	5.09070483	4.53979927	4.8115626	4.87351153	5.24957042	4.63978616
ZBTB1	5.41402098	6.03304505	5.54055614	6.39015808	6.22724866	6.45339681	6.56770686	6.26224552	6.19818728	5.9342772	5.89093581	6.11051104	5.98657269
TRMT5	4.93263379	4.7337259	4.58119439	5.19159487	5.0591586	5.49461811	4.68431701	4.934564	5.22187558	4.78730933	5.10117273	4.7191709	4.56231396
SGPP1	4.40998576	4.60180065	4.04550341	5.13715223	5.12038457	5.03731212	4.64784253	5.00335794	5.03619149	3.42483771	5.23098503	5.30584769	4.45986395
PLEKHG3	5.85587629	5.84076515	6.32944455	4.61014874	5.28118674	5.55514641	5.73660005	5.64113296	5.70930517	6.14678722	6.29919284	6.09010396	5.38572339
PZP	-0.0212946	0.1749836	0.07268125	0.38393547	0.70851288	0.18851655	0.55887799	0.32461735	-0.01038934	-0.01038934	-0.01038934	-0.01038934	2.95448536
RHOT1	2.79321377	2.96315408	2.54797077	3.26658368	3.86497129	4.33071236	4.00210126	3.69109489	3.87396678	3.25116928	3.13233125	4.12770078	2.82386591
EVI2A	2.78405466	4.03915016	2.59739173	5.04532985	3.77032504	3.45099815	3.09222137	3.71061827	4.30831394	3.92777244	2.59293225	2.70651536	2.91022351
WDR60	5.69577988	5.5411352	5.40506883	4.98640412	5.55382669	4.99149499	5.58769526	5.79642047	5.66704996	5.60569638	5.17806029	4.9235049	5.94034733
AIF1L	4.83589789	5.473315	4.33015871	4.00019404	4.75263467	5.98102234	3.69869125	5.42917252	4.10945622	4.7561209	5.20997743	6.20665342	5.28088127

FAM78A	3.02527776	3.4178094	4.18012923	3.16034339	2.04549533	1.73078675	2.68381958	1.79943772	2.60815135	3.18505243	2.81001162	2.67882638	3.49658474
NUP214	6.74653764	5.85857484	6.41366649	6.25475464	6.38448271	5.965164	6.07807926	6.12286226	5.90952945	6.45807372	5.91196673	5.95541874	6.36934291
AVPR2	0.1583624	0.65768455	0.16091733	0.1583624	0.77151228	0.35377229	0.75174371	0.64390541	-0.23208326	-0.23208326	1.94353546	2.42201272	-0.23208326
SLC10A3	4.73628353	4.15445741	5.02345362	3.48093076	3.53819903	4.25539966	3.14506061	3.87987449	3.67078102	4.54369712	4.64449949	4.29131893	2.93310279
MAP2K2	5.10972045	5.13672384	5.68456332	4.88472487	4.82419296	3.80392866	4.63716099	4.53065384	5.0697679	4.73305254	5.88106069	5.32766701	4.02818399
HNRNPH2	6.45071236	5.91953281	6.28836354	7.04257308	6.53178494	6.66228499	6.47698962	5.99241989	6.66705702	6.39614277	6.40692543	6.4228029	6.53368515
ARMCX1	5.95319936	5.23421175	5.7869918	4.96869611	5.59875361	6.23211421	5.93305354	5.76213825	6.05281334	6.25096523	5.53460799	5.82946866	6.08414079
NXF5	-0.05267686	0.31868286	0.34649656	0.11421357	-0.2372025	0.5810077	-0.0796416	0.17738196	-0.05386476	-0.05386476	-0.05386476	-0.05386476	-0.05386476
TIMM8A	-0.45090561	0.7323142	0.07490354	1.08777599	0.66114748	0.73669743	0.06813973	0.45084205	0.23515507	0.23515507	0.23515507	0.23515507	0.23515507
ZC4H2	4.58142198	3.74162301	4.18295541	3.59218988	4.02155205	4.58482362	4.15796914	4.02667692	4.52445592	4.1092645	4.77247626	4.03173213	4.51240771
CANX	7.91856129	8.41531442	8.3327068	9.06729003	8.56308693	8.86487311	8.77973488	8.31545584	8.47643883	8.18529443	8.35883156	8.2553739	8.19336498
RGS13	0.4294658	0.58202099	0.52106454	0.44827797	0.56120683	0.57792407	0.83229483	0.43278101	1.80199009	-0.18182564	-0.18182564	-0.18182564	-0.18182564
IPPK	3.94696549	4.06065312	3.88321678	4.2227253	4.06973907	2.90403144	4.05210881	3.69253411	3.9727045	3.71110266	3.34379656	3.58208671	3.38104743
ZNF484	4.52759515	5.29482843	4.61095057	5.34324532	5.54448687	5.83657433	5.32452899	5.46626873	5.58001671	5.24334501	5.55749015	5.10213096	4.97526443
OMD	4.99537168	6.83578454	5.26190843	6.82918723	7.68621567	7.90145674	7.40330661	8.73010628	9.232522	6.43544946	7.77079743	7.71128607	6.38842201
FGD3	3.61024442	2.66925043	2.82755202	2.7162418	1.77084055	1.12502805	1.88869911	1.19685952	0.72152117	3.08095825	2.38378103	1.92282699	3.35967833
HIVEP3	4.07073084	2.99973854	4.63166035	3.44235196	2.60540094	1.76108761	2.16237149	1.71103503	1.68591507	-1.22647456	2.87212773	3.53851032	3.5434207
PPCS	4.93256002	5.8616581	5.32885822	5.38639938	5.26000062	5.81046984	5.14411991	5.22958478	6.14616972	5.51632237	5.46059004	5.61949579	5.23456132
EDN2	0.31124032	0.69488397	1.24146156	0.62374135	1.86729387	1.18027407	0.35413765	0.22866292	-0.31306951	-0.31306951	-0.31306951	-0.31306951	-0.31306951
BCL11B	2.56958216	2.37964931	1.64377197	1.89821117	2.15708592	1.49264946	2.00688856	1.91434389	3.73934084	-0.78981038	2.96658603	2.48958205	-0.78981038
COX7C	5.69312053	6.104076	5.08755597	5.84794631	5.5678039	6.53562348	5.04319995	5.60020661	6.07100661	6.53793851	4.90313547	6.25138872	5.97201492
TRAF2	1.02189326	0.22594931	1.5554619	0.73946519	0.21989788	-0.03707093	0.79400253	-0.23088374	0.31754886	0.31754886	0.31754886	2.79543994	0.31754886
ABHD8	4.052565	3.97104852	4.18534163	2.69346578	3.03999683	3.63530059	3.01192862	2.84566388	2.88059021	4.07060319	2.6324268	4.41118208	2.43948838
MASP1	4.34773085	5.243674	4.30616342	3.1122664	6.25365377	4.17968539	3.35681574	3.19029539	5.53059736	4.69342499	6.80828904	4.88866793	6.08739989
ATP13A4	1.07489338	1.22564533	1.16892386	1.12902609	1.05687575	1.4844231	1.16804947	1.24753239	2.86677986	2.76106991	3.03431862	-0.58868187	2.10160686
HRASLS	-0.15061823	-0.06407052	-0.34345696	0.01382381	-0.08547376	0.90774837	-0.01077238	1.08803604	1.10638051	0.09268855	0.09268855	0.09268855	0.09268855
HELB	4.0607666	3.77127533	3.3393247	4.62246765	3.85726764	2.99564065	4.05437434	3.50396056	3.68927846	2.89110626	3.74378401	2.61077896	2.74186119
RAP1B	4.19630588	4.43158435	4.01551557	5.36915266	4.91269129	4.72360409	4.8991617	4.93954374	4.38425204	4.22044397	4.44378235	4.73792132	3.61861747
TSPAN8	3.29105424	4.85136842	1.99248938	1.78006729	3.64716426	4.44868363	2.20453515	3.74789475	4.73996563	4.6362275	3.23839314	3.52471675	4.45072501
RAB3IP	5.08606573	4.95154471	4.42723666	4.57494907	5.55381574	5.574186	5.55493605	5.51534084	4.99911015	3.94065731	4.70004957	4.70669547	5.53769011
PTPRB	1.94413822	5.53186293	2.69271727	5.30241595	5.89596327	4.34062904	5.8353127	5.49425363	5.76519416	5.80987176	6.6909226	5.53001645	7.66042724
DYRK2	5.00034938	5.5135213	4.82915142	6.05896072	5.88512999	6.07124656	5.82413442	5.78602877	5.88599836	5.4396315	6.06592999	6.15735864	5.30863663
YEATS4	2.73458403	3.15960879	2.10666631	3.55221598	3.75288614	4.11549449	3.72169862	5.05163532	4.41972739	3.54346518	4.49762824	4.32614603	-1.45836945
TAS2R3	2.69613949	2.05313359	1.6288496	1.02897295	2.99937936	2.29529619	2.9998284	1.90212997	2.67439672	-0.54849061	-0.54849061	2.85030385	4.58404295
TAS2R4	1.60751684	2.66507478	1.27539187	2.33268123	3.52376655	2.6027425	3.23355689	2.72178106	3.59833436	-0.80897246	3.62331827	2.53422191	3.35168583
TAS2R5	3.00038015	2.97836897	1.41260479	1.08816928	2.87268229	1.69761903	2.48026066	1.35626215	1.60933221	-0.54519494	3.48893687	-0.54519494	2.19246375
CRYGN	0.92953915	-0.33135473	0.72971787	0.06619075	0.20003191	0.51241744	0.63052694	0.34511036	0.01236239	0.01236239	2.11700143	0.01236239	0.01236239
LRRC61	2.09893603	1.1307549	0.50984405	0.84454478	-0.28510233	0.77154349	0.20044949	0.56971628	1.83417696	-0.15945267	-0.15945267	-0.15945267	-0.15945267
IDUA	3.91646278	2.96229172	4.40933722	2.4943735	2.74085335	1.83518706	2.73734721	3.26975203	3.33344864	4.59564226	3.43782862	3.82793059	2.75221999
FGFRL1	6.27362191	4.90347365	6.21908206	4.37905565	5.88352803	5.72369485	5.84816552	5.95460769	6.02184016	6.44900967	6.1174259	6.28188073	5.27916
TMEM175	4.29018904	3.31463563	4.8260778	3.27902547	3.86334584	3.5852262	3.14037427	3.84701436	3.63267542	4.50046237	4.58026526	4.51137222	3.81821339
PIN1	5.12806125	4.96079566	5.70884559	4.21219958	4.47866712	5.25767322	4.11501575	4.46634026	5.03015506	5.40259893	4.84290208	5.15455482	4.49257395
FBXL12	4.83007773	4.69146605	5.25504945	4.24501746	4.57888648	4.08388512	4.35951491	4.57596787	4.61249499	5.14077861	4.34205742	4.58513601	4.30171617
PLA2G5	3.08884367	1.86863288	2.85830282	1.20283403	4.1551042	4.37863033	3.63439987	3.81369049	3.20829776	-1.12695312	3.60160201	3.67533432	5.61938631
UBR4	7.85043984	7.46000796	7.53599818	7.33572145	7.85043984	7.33066076	8.10376296	8.44231776	7.19824528	7.77016821	7.80662748	7.79359728	7.97848785
HP1BP3	7.21331486	7.13895616	7.33559228	7.22598782	7.49822793	7.2291757	8.16280591	7.52326124	7.14990781	7.37848987	7.33013642	7.56443626	7.68060333
SIN3B	5.97447627	5.54698041	5.86083186	5.37365055	5.66790924	5.0153428	5.50299857	5.68445325	5.33535198	5.93146485	6.02743136	5.79149633	5.51112405

SLC35E1	6.13658366	5.32058694	6.07510106	5.8682928	5.3798515	5.13864004	5.61273369	5.35015318	5.49066556	5.64252006	5.76849242	5.44525293	5.37282819
EPS15L1	5.32699577	4.80469212	5.30089351	4.42806063	5.05022869	4.50849835	5.02331689	4.92248149	5.11652335	5.36891401	5.24180493	4.73878766	5.61952226
KLF2	4.21582786	5.79600428	4.71413153	3.72597947	5.01957274	4.90668193	4.46490622	3.31196148	5.68601418	5.84290268	5.58083607	5.48612702	4.98527464
F2RL3	0.02406504	0.33839216	1.00922616	1.056312	1.02820704	0.75862298	0.5728874	0.41199098	-0.14374081	-0.14374081	-0.14374081	-0.14374081	3.38410699
UQCR11	4.72375367	4.65962696	4.77063253	3.5164866	4.27224048	4.62840653	4.31434548	4.73484679	4.73602812	5.46170374	4.57606467	4.5730153	4.19710211
GFER	4.5981346	3.48398934	4.65478571	3.04563898	3.53099662	3.98501213	3.917561	3.38595417	4.08899198	4.26201131	3.76825935	4.10427128	4.26988312
SYNGR3	0.22207761	0.12137307	0.04228864	-0.12004146	0.04767121	0.29724	-0.12004146	-0.11679364	-0.0944152	-0.0944152	-0.0944152	-0.0944152	-0.0944152
PKMYT1	0.00519072	0.13399541	1.53621444	0.76315652	-0.4089852	-0.40113696	0.14302293	-0.01963845	0.0080562	0.0080562	0.0080562	0.0080562	0.0080562
WFIKKN1	0.27976779	0.20542025	0.54709435	-0.14778518	0.23840768	0.29768894	0.17453829	0.58576692	-0.04281301	-0.04281301	-0.04281301	2.2204557	-0.04281301
WDR24	4.56672794	3.62450434	5.08458208	3.3766113	3.42356905	3.01845955	3.27747845	3.61504331	2.82938119	3.40110335	4.03000574	4.76854874	4.31489827
FBXL16	0.02176516	0.12362282	0.00124472	0.05278108	0.03805995	0.12843121	0	0.00158797	0	0	0	0	0
CHTF18	2.21929663	1.77904113	4.51552935	2.17254997	1.50273174	1.60457014	2.12811872	1.30012894	2.83008963	3.79424524	3.16808224	3.05770483	-0.91854907
MACF1	3.3570028	3.90931627	3.34393318	3.14745128	4.55948535	4.2823709	4.19275214	4.85520758	4.15998214	3.80560837	3.15601799	2.54560281	3.96062856
SMARCA4	6.05952012	5.43389464	6.52555906	6.04175219	5.39596825	5.26084879	5.62625733	5.39709358	5.41017508	5.88862223	5.65340012	5.29459102	5.96950614
KDM4B	5.74258049	4.93766011	5.73086959	4.76866486	5.34240152	4.62595817	5.3737613	5.17430381	5.11558275	5.24801115	5.42728707	5.13430969	4.85793628
TICAM1	4.39354932	3.40984731	4.96795233	3.54059564	3.49468478	3.56922443	3.17852157	3.82073363	3.74079988	4.26404054	5.02853948	4.22950832	3.4036616
IL17B	1.45910983	-0.08752037	1.72754646	0.01223654	1.10318842	3.12246037	0.37820689	1.0469145	0.87103096	3.99249787	-0.32806475	1.79780049	-0.32806475
METTL16	6.01662463	6.27935794	5.80479343	5.35564179	5.9133555	5.82922927	5.87465166	5.59229598	5.46631808	6.03748844	5.46406376	5.86339435	5.53738688
TUBA4A	6.85720429	5.64928849	6.33811267	4.82182535	6.10679662	6.31358195	5.92288791	5.15927083	5.60950612	5.32873898	5.41770699	5.57255635	5.52973632
AAMP	5.3025666	5.28902779	5.53017093	4.43424591	4.67053923	4.99072412	4.39533151	4.80087775	4.46778924	5.33969986	5.0748652	5.22656796	4.85275129
PNKD	4.6389509	3.76595902	4.99942431	4.39491464	2.71256512	3.77377319	3.45258738	3.09300478	4.28804361	4.99596502	2.26576666	4.29779759	3.53567472
TNFRSF19	1.31813501	2.91516321	0.79848063	2.89403078	2.91448802	1.67747189	1.80557889	2.06129012	2.90008001	3.33371778	1.45798206	2.62766912	5.66960246
RNF6	5.46106111	5.79446761	5.53302344	6.16218977	6.22467778	6.49462527	5.75852887	5.87082049	6.04539346	5.69488671	5.64503234	5.5693756	5.98546547
ECHS1	5.2697911	5.67382435	5.94574853	5.39238437	4.96667863	5.18191739	5.50844931	5.05848306	5.17583657	5.88402079	5.60219272	5.55212927	5.83765434
ZNF835	3.3900336	3.20924381	3.0591761	1.81417621	2.36304728	3.54841728	2.80028446	3.79840313	2.90683519	2.01958983	3.86088211	4.1972628	4.7853881
AKAP9	6.08644364	6.63536541	5.98721779	7.20963845	6.90543639	7.54126296	6.82255708	7.29155089	7.56938199	6.57668324	6.29619099	6.5585394	7.25844359
GNG11	2.80216188	3.01552705	3.41995824	3.96328252	3.37619494	4.21165985	2.91678247	3.91147941	5.00405616	4.32049035	4.61400703	3.57154114	2.94600245
HIP1	7.15669589	6.95080415	7.12819103	6.30118736	7.14307543	6.45623617	6.68288065	6.66097482	6.51165851	6.53040105	6.88077261	6.67388115	6.85588242
PTPN12	5.99636015	6.61494068	6.01867475	7.19557846	7.12731626	6.83471866	6.85998778	7.00983801	6.59769416	6.26165343	6.24641814	5.96642877	6.57238839
POR	3.36600069	1.8762317	3.91561863	2.31947979	1.81034287	2.2598621	1.85738259	2.1928001	-0.10588958	-0.10588958	-0.10588958	3.18253544	3.56788304
FGL2	8.10085551	8.3262349	7.70133752	7.81893304	8.22556144	9.64431418	9.92302583	9.01814029	9.26921355	8.83897684	9.32292215	9.47023205	8.65327416
STYXL1	3.99550268	2.5001533	4.02960372	4.26149265	3.31632327	3.96036222	3.17604931	1.94081919	3.57510952	4.04324675	3.12989799	3.00832976	3.31282059
STEAP4	7.30590963	6.78362367	4.62384315	6.87195699	5.60075543	6.34675986	7.26984692	4.65424243	5.46765773	4.88814095	6.57588083	5.11468251	6.59918733
GNAI1	4.29351175	5.03914432	4.19963473	4.77807496	5.34662199	5.64554826	5.42664573	5.4684482	5.13124164	5.12193382	4.54746997	5.68379139	4.95214775
PEX1	5.22844579	5.30948542	5.07262196	5.44250762	5.73538989	5.73730536	5.81110408	5.95811635	5.31104164	5.4496137	5.6173848	5.34973759	5.38785011
SGCE	6.21971725	6.01027617	5.28204058	5.70267766	6.11930554	6.58135907	6.02973564	6.33977669	6.20247875	6.71338122	6.2284056	6.56626123	5.79748488
CASD1	3.95910083	5.13607341	3.92860885	5.43955016	5.25030437	5.40493084	5.41393467	5.52137996	5.23875994	3.60746355	5.808265	4.87395356	3.88968648
ZNF780B	4.08763463	5.23988988	4.52947336	5.08758749	5.40366003	5.35217831	5.45463068	5.48009666	4.97531134	4.53704624	5.49096116	5.12702565	4.75051679
LRFN1	1.3202435	1.04467882	0.83642645	0.46927537	0.2981716	1.13304904	0.84782799	0.83067904	2.72468696	2.00769058	-0.46396911	2.0902778	1.27159356
ZFP36	7.78357351	6.74097728	7.65200424	6.61311463	6.52506625	6.31018248	5.57425893	5.93665539	6.55801359	6.44593711	5.56625618	5.97861317	7.16262715
SRD5A3	0.28090472	0.59164665	1.17142362	1.96675906	0.37546516	1.20781777	0.3227942	0.33460783	0.49051183	0.0258592	0.0258592	0.0258592	0.0258592
SPINK2	2.11858559	0.06032347	0.06280842	0.06032347	0.29161244	0.06170507	0.06032347	0.06349368	-0.31533967	-0.31533967	6.11331328	-0.31533967	-0.31533967
RASL11B	3.96378834	5.21447841	3.51094391	2.2376943	4.85549731	3.69894335	4.92965648	3.45031614	4.7659614	5.2944928	5.3366597	5.99336069	4.9375968
PAICS	5.37371111	5.11198384	6.24495481	7.10986971	5.19538069	5.29783856	5.42890334	4.78817727	5.38008791	5.54519887	5.17177313	4.89810456	4.89767883
KDR	1.21235032	7.91218915	2.97682437	6.63163913	4.2302636	5.21498189	3.88078632	4.03640874	5.55924179	4.69811279	6.63726285	4.96293775	7.4224634
PPAT	3.60623664	3.16941219	3.1834957	5.25381917	3.45218904	4.25694358	3.76189458	3.68875342	3.6956439	0.97535493	4.36539041	3.96805749	2.04310277
TUBGCP6	5.19186783	5.02514274	5.39987684	4.18935038	4.86344891	3.94537844	5.15915303	5.24692349	4.8971446	4.36754101	5.40929862	5.56596261	4.92784489

ADM2	0.46757567	0.21734104	0.79693916	1.18235714	0.33109391	0.21875476	0.28020405	0.41073839	-0.35704096	-0.35704096	-0.35704096	-0.35704096	-0.35704096
DGCR6L	4.86800768	5.44400095	5.66184683	4.17494502	3.9639874	4.58947257	3.87104335	3.69014437	4.79847668	6.10238088	5.06644913	5.40234907	2.99914276
DGCR8	2.49051352	1.74082273	2.56691364	1.59989828	1.92364083	0.88466371	2.19842182	2.51284935	1.45890812	-0.03938987	-0.03938987	2.15180249	-0.03938987
ASPHD2	2.26343061	1.44797525	2.14660872	2.36502921	1.70891134	1.67015878	2.09955516	0.93030556	-0.43846246	2.60067763	1.25508034	-1.17534323	-1.17534323
SDF2L1	3.71390732	3.32555163	5.62405712	2.88224858	2.15338494	2.47641228	2.65623891	1.12785753	2.27617679	-1.21241602	2.91282035	2.26114045	3.88389256
GAL3ST1	2.74624728	1.01917176	2.04404056	0.44160731	2.66000243	1.45710313	2.38668484	1.73279885	-0.54054012	-0.54054012	1.86137245	3.93634249	2.29137448
YWHAH	6.2949678	7.02652525	6.91665753	6.93182213	7.77958026	7.17367216	7.14991695	7.0294292	7.09086983	7.43446728	7.05729938	7.23185035	7.26196009
GNAZ	5.05834366	4.60637754	4.15309295	3.14770668	4.53291972	4.57022541	4.47807094	4.23096559	4.88401047	5.19000686	4.66131044	4.92797935	4.84409832
MGAT3	1.40853398	2.73269339	1.28526794	1.44231596	2.15689335	1.31623002	2.06121836	2.71661474	3.02663823	3.00097458	0.5734579	1.29369402	4.52768435
ADORA2A	1.60285791	1.20500462	2.23784218	1.08379476	1.54989083	1.00784534	2.19918595	2.07567547	1.38859463	3.02625531	2.24728655	-0.72821377	0.29904478
ATF4	7.80258703	7.80258703	7.96695993	7.33472382	8.02156485	7.42155678	8.05873858	8.12312525	7.51682752	8.00258448	7.15563299	7.73256685	7.55576027
A4GALT	6.72555283	5.29702006	5.92799759	4.16031808	5.32531388	4.55561542	5.00597023	5.44521117	4.71186091	4.63863951	4.73268116	4.82833063	4.9852773
CDC42EP1	6.29978248	5.92485775	7.02515435	5.18770118	5.95558563	5.32284964	5.53393102	5.47599118	5.81888231	6.82985981	6.19808549	5.78822089	5.36002897
APOL3	5.86482712	5.37895317	4.72269201	5.27807011	5.44655908	3.98684814	5.24903615	5.50603627	5.59602162	4.72033532	5.19885758	4.44628098	5.59867847
MCHR1	2.41631499	0.88299463	0.98438088	1.51569901	0.58125137	0.58263321	0.58125137	0.58442214	-0.39747212	-0.39747212	-0.39747212	-0.39747212	2.12056581
TPST2	4.55574798	3.97530615	4.92642005	4.9473082	4.40645388	3.51451427	4.19858396	4.04671523	4.18574078	4.00460701	4.63554852	4.43594453	4.49147452
BAIAP2L2	0.22091985	0.30834111	1.05920934	0.29628924	-0.10093491	1.03736473	0.55133671	0.05873927	-0.11887696	-0.11887696	1.75781718	0.91482963	-0.11887696
MPST	3.98206857	3.457672	3.73138326	2.96954149	3.87648384	3.71920267	4.04431515	4.8780999	3.97802652	4.53814893	4.15948786	4.15685946	4.15482655
TST	2.20661647	2.4380996	3.46063513	2.6511703	1.84937434	2.44312139	2.43473557	2.22720179	1.6624488	4.45042862	3.49168272	2.81619785	3.30886763
APOL2	6.21095715	5.78747945	6.30871873	6.06245526	5.47454515	4.69843417	5.14285706	4.91366335	5.42677984	5.25577421	5.20717991	4.95387463	5.58286752
RAC2	4.74109024	4.27453554	4.90133746	4.34019783	3.2024019	2.42640425	3.32615607	2.4508161	3.80079623	3.34400174	2.37488014	2.24741659	2.87224788
LIF	2.91104366	3.93908839	3.21306536	7.21305768	1.86151443	1.50067265	1.55781462	1.54631518	2.4224338	1.73672873	-0.3457505	-0.38212335	1.34016112
C22orf23	1.06674093	0.10685804	1.0353295	0.2935437	0.40833315	0.812183	0.10685804	0.11007197	-0.37341801	-0.37341801	2.07828158	-0.37341801	-0.37341801
APOBEC3A	1.25801576	0.17028727	1.52059286	0.81330558	0.35457913	-0.32898896	-0.22981078	-0.15342846	-0.30056073	-0.30056073	-0.30056073	-0.30056073	2.80527245
APOBEC3F	4.83317404	3.79632078	4.06224812	3.60043973	3.93515227	3.9768031	3.52306822	4.05812984	4.05453321	4.21986351	4.19557643	3.10966401	3.93088141
RIBC2	0.08785311	-0.00606665	2.26388357	1.28897428	-0.02977044	0.49265497	0.64816005	-0.24092198	-0.38371217	-0.38371217	-0.38371217	-0.38371217	-0.38371217
KRT17	3.39980585	1.91895732	4.32626948	2.40089628	6.15911865	2.7131337	3.76868171	4.80083797	4.67373462	3.12380453	4.4365572	4.75897095	2.81108477
RNF112	3.9482067	3.03396695	2.44637265	1.53820333	1.17539666	1.70995536	1.86280505	2.01708676	1.78924423	2.95674744	2.51879842	2.6770233	3.38418355
SPECC1	4.5833116	4.77170952	4.28941953	3.24753666	5.08559382	4.83671138	4.10906431	4.70545686	4.32486607	5.59804143	4.83840455	4.71468421	4.38019692
CPA4	0.01380362	3.60893638	-0.04689109	1.68148997	-0.09993651	-0.1191121	0.2322806	-0.11808899	-0.18125395	-0.18125395	2.89966609	-0.18125395	-0.18125395
DOCK4	4.9135633	5.2057857	4.89037898	6.26857873	4.74345733	4.13338048	4.60291044	5.56891065	5.08469345	5.20694054	5.00830406	5.8526918	5.67208402
POT1	3.92050057	5.41519732	4.16193956	5.92962582	5.62412183	5.69839633	5.20850913	5.72605032	4.94988791	5.21441313	5.14236089	5.80723641	4.60848813
ATP6V1F	5.91739089	4.86780577	5.33566836	5.8978838	5.27270994	5.51032422	4.98428302	4.4100193	5.13387402	5.81810486	5.13653051	5.63351648	4.51002003
CDHR3	2.14423656	2.32876303	2.07408125	2.18565584	3.01190778	2.51310111	3.02240885	3.68524955	-0.64965487	3.91963121	2.46002173	2.52841665	2.29895833
PRKRIP1	4.41881641	4.13504264	4.47347352	2.95468016	3.50952999	3.31438109	3.89511337	3.59042278	4.24088643	3.61748937	3.86263928	3.51060274	3.12909335
PODXL	2.95105164	6.90860899	4.67153551	6.11314377	6.35020608	4.14111231	4.92407472	5.74186434	6.20361324	5.79996325	6.71578366	6.31208528	7.24708964
FOXP2	2.47792136	1.94799912	1.75767658	2.13228863	3.55461823	4.13859438	3.56506775	3.90115235	3.68162987	-0.97315682	3.80970665	3.71692095	-0.97315682
MKLN1	-0.27940021	1.52862975	0.04543535	2.06781777	2.42136111	2.78051366	2.40371672	2.60729911	0.91994018	3.65750704	0.34289163	0.34289163	0.34289163
DNAJB9	4.68789982	5.94657326	4.47978828	5.9961793	5.80013724	6.50745742	6.5050255	5.98664434	5.74217817	4.94503936	5.55066626	5.09151232	4.24754825
FLNC	6.73694263	6.97883574	7.57998704	6.01362304	7.77152277	8.10696927	7.96949924	7.92778791	6.16046714	7.98154354	6.98394209	7.98818655	7.8917302
LRRC4	2.79632461	2.16673198	2.59201637	1.62818804	2.44280836	1.3471747	1.90864031	1.32805247	2.35969	1.95479174	3.00236003	2.61037737	3.73550369
CALU	7.23311754	7.84732793	7.55211705	8.84440865	8.42174015	8.43356246	8.3751608	8.30322634	8.51866869	7.91833082	8.46904304	8.15838299	8.15194238
CCDC136	5.20401239	3.66333839	4.79628593	3.7054457	4.52902381	4.85349976	4.91994283	5.34161344	4.22886898	5.00086409	4.20418931	5.08811286	5.30205896
SMO	5.16569419	3.82802649	4.86301913	4.38648694	3.59587181	4.03825133	4.51189846	4.14000332	3.77741102	4.44474764	5.11068126	5.10566385	3.64648235
IRF5	3.36064927	4.12458978	4.52874948	3.89901101	2.55297372	1.51316234	2.66641936	2.05107087	4.00755588	2.93097579	1.62603607	3.2496092	3.45473805
LRRC17	4.73835897	5.41704128	5.42360424	7.67280849	5.2445612	4.70850078	5.86332625	5.24108122	6.33793945	6.72882703	6.88663675	6.53352357	6.07833269
KLHDC10	5.81947187	6.03886758	5.55586761	5.97525851	6.06843543	6.1373083	6.364775	5.8900316	5.9003722	5.73604876	6.273374	6.07300027	5.93893198

NDUFA5	4.87676239	5.1762794	4.7785387	5.61493827	5.66011998	6.20299329	5.85652497	5.7981727	6.433821	5.45404071	5.37916613	5.54496121	5.53817221
MRPS12	2.62060093	3.49196786	4.02083591	2.4171449	1.82482827	2.47944441	1.98153419	1.56800534	0.09559617	3.89327892	3.63641725	3.64456745	1.71591022
MYO1B	6.48716173	6.64768956	6.51237527	7.31680483	7.45189503	7.09898445	6.93473331	7.33012585	7.14658265	6.69759835	7.16308011	6.88206321	6.59401696
HOXD1	0.09206524	0.09206524	0.09460984	0.77463473	0.54712704	0.09348	0.33750508	0.62510684	-0.10264773	-0.10264773	-0.10264773	-0.10264773	-0.10264773
HOXD3	-0.12843398	0.23007924	0.01406723	0.87382713	1.01950865	1.04340675	0.58718892	0.66881776	-0.01177887	-0.01177887	-0.01177887	-0.01177887	-0.01177887
MTX2	3.73801123	3.84111482	3.48685937	4.49704884	4.30741928	4.92671243	4.48333458	4.14496575	4.76635944	4.6371756	4.15553499	4.32817901	4.94473606
PDE11A	2.19145805	1.96171249	2.13078112	1.88235104	2.00547113	2.65388636	1.86567587	2.10309704	3.27491909	-1.07934209	3.9916636	-1.07934209	-1.07934209
CHN1	4.05843462	4.20550376	3.67143033	4.28728022	4.49583006	5.33029486	4.93018276	4.92013853	4.46840847	4.04430797	4.54088359	4.54088359	4.15070426
GAD1	3.99084836	1.97553257	3.37424219	1.53200513	4.65163723	3.52205717	2.87475198	5.18273436	3.39398096	4.32803325	4.47933958	3.62016604	2.36448908
OSGEPL1	2.9480496	3.8085176	3.03240531	2.84661646	3.70421303	4.04241973	3.9294087	3.47108964	3.45327416	3.00834682	3.93720875	2.9373998	3.76885027
ORMDL1	4.72390429	5.45586721	4.68192532	5.96653284	5.87160894	5.80074726	5.95369254	6.09547875	5.65780372	4.31421967	5.76879714	5.9524433	5.52100817
HAT1	4.1492228	4.1969965	4.61328474	5.64394452	5.20970833	5.28703129	4.90488272	4.996576	5.10121451	5.2289452	4.89035674	5.09813499	4.68093955
HOXD9	-0.09568192	0.42585374	0.04777876	0.04860932	-0.09568192	0.64776977	0.04935492	-0.09299653	-0.00128373	-0.00128373	1.47887201	-0.00128373	-0.00128373
HERC2	6.75318165	6.28580291	6.88586947	6.05440955	6.64046708	6.47049299	7.05714057	6.97224165	6.39868591	6.76681421	6.884378	6.57671203	7.42950287
SNRPN	2.36835817	2.58100479	2.49512508	0.85622267	3.33124077	1.99912325	3.11256097	4.25343642	3.08978106	4.03913801	-0.25311604	4.69766526	3.92861269
PSMG2	0.92600073	2.28107741	1.24813926	3.11927007	2.54971992	1.9976007	2.44688163	2.39045678	2.58870751	3.80708816	2.56503652	1.10597398	1.81471747
TWSG1	6.31636603	6.9485406	6.62372985	6.77522827	7.708394	7.85594739	7.89300955	7.70394557	7.60528487	7.90717834	7.67851533	7.97302776	7.32369927
ARHGAP22	4.84501196	4.29485488	5.42697922	5.26633317	3.01446636	1.83189586	2.65409427	2.54864889	2.69092929	3.89365245	3.9409753	2.3972457	4.00856882
WDFY4	3.71360797	3.60725266	3.99155723	3.78351011	2.1679599	1.22710191	2.50683755	1.53308245	2.64028204	3.38914315	3.7797463	3.86495866	3.84161313
EIF2AK4	3.36932056	3.84751501	3.45591328	4.72911061	3.63685202	3.85034391	3.55726865	3.65842837	4.48974247	4.40239563	4.56996612	3.27703993	4.20406895
MYO5C	1.09511026	3.57126512	1.57468313	3.34552262	4.8643685	2.70821944	2.50869347	4.40909832	4.61892442	3.99548995	4.46675771	4.22074816	5.89258478
CGNL1	5.96070901	7.40078036	6.87242549	5.93950986	5.82211068	6.57401151	6.39274968	7.09384862	7.51895842	7.51377948	6.2545969	7.03300576	6.82474078
TMOD2	4.34320848	5.90435218	3.66573799	5.35465419	5.65763906	5.8114514	5.60747687	5.33776587	5.9222062	5.79155493	6.01227833	5.55427196	5.63196726
TTBK2	5.9866939	6.01577101	5.58108247	5.23481196	6.61973047	6.37066684	6.54047585	6.71727716	6.39959414	5.87719474	6.37528435	6.35531688	6.32300596
INO80	5.74355762	6.29385608	5.69514324	5.97257315	5.93963535	5.69590504	5.66540564	5.83876656	5.62753271	5.65476475	5.7625719	5.91458522	6.15936722
DLL4	1.23187641	4.33482826	4.13488783	4.26210781	4.67776703	2.16910035	3.35539518	2.63816816	3.98865985	4.15826057	4.46370778	3.89444875	4.80520436
ALDH1A2	4.04967841	6.03997823	2.65382168	3.70153672	4.11684544	2.71645046	3.17060757	2.59829451	4.45317551	4.24169421	4.76362187	0.28021788	5.05552681
IVD	5.90828914	5.78233307	5.88475052	5.34726376	5.33012538	5.02418698	5.54264336	5.10783205	5.52931042	5.84096753	5.85316208	5.94303918	5.9289304
DUT	4.84856098	5.16568703	4.9048327	4.59980399	4.17722018	4.62244875	4.78930334	4.3142786	5.34231992	5.12688586	4.46374892	4.90786275	4.69730254
CHAC1	2.15098107	3.17839235	2.30052795	0.44949753	1.05093124	3.30147822	1.01432028	2.28833121	2.68213923	4.06615228	-0.53161428	4.75920845	-0.53161428
CLN6	3.75101131	2.47730381	4.28606332	3.73067518	2.27086418	2.02909275	3.36480738	2.66344033	3.12221539	2.78712656	4.00163411	3.33219546	-1.14052016
ARPP19	6.62752437	7.55967268	6.70640537	7.63409719	8.06975605	8.00657164	8.06824392	8.03080717	7.54626886	7.35509654	7.77508121	7.98815439	6.71827475
VPS13C	6.63450156	7.45341476	6.83812788	7.71761513	7.62342511	7.71527059	7.77750161	7.68565835	7.60779844	7.24102015	7.58197912	7.72509964	7.49948509
CALML4	2.77801202	2.46560993	3.11712461	2.76079357	2.55450824	2.5044506	2.69100002	2.67522508	3.99148282	0.99420348	1.94766777	1.90482926	3.16892032
ISLR	5.74310932	6.86453979	6.48393046	6.98597279	5.71595455	4.67631675	5.79756763	5.32992627	6.78985976	6.38654624	7.15485189	6.3006369	5.84352803
THAP10	2.05469499	1.41065492	2.59551744	2.44867942	3.40977091	2.87776352	2.93727796	2.22976994	3.49130057	-1.15930475	3.7977408	1.96420305	1.9700525
LOXL1	5.36072281	5.37894208	6.2224136	5.96791064	5.14861063	4.52138165	5.06601901	6.17465916	5.66436084	6.13507946	6.26152778	6.65000855	5.18121796
ANAPC13	4.64622469	5.65720161	5.73111054	6.80650626	6.47325793	6.68689409	6.20134547	6.18358601	6.66054777	4.82131962	5.86415209	5.6431047	5.10194661
MBD4	4.85615667	5.24170473	5.24755059	6.03611719	5.63708933	5.93723187	5.54616225	5.61352875	5.63450672	5.94738546	5.48711951	5.2455654	5.48078737
COPB1	5.33919894	6.33314993	6.06029821	7.19030342	6.35484058	6.388141	6.52610868	6.53358471	6.42143111	6.52214687	6.35324093	6.43340007	6.41599431
PSMA1	4.83311516	4.98128839	5.10746524	5.62683963	5.46051479	5.47894954	5.65818677	5.87546508	5.09479354	5.31933307	5.05945728	5.62387338	5.17017001
SUMF2	6.06419102	5.75806026	6.65879475	5.96026488	5.1514073	6.05728942	6.32365357	4.93252544	6.04292889	6.580594	6.12455767	5.98479058	5.33435461
PALLD	1.00619858	1.5477974	0.95795771	0.74723374	2.91012646	3.25514584	2.20797097	3.38282726	1.16473385	3.60374371	4.28251752	1.16473385	8.0489398
SPCS3	5.61671078	6.59020791	6.17367179	7.33928864	7.03588417	6.87511554	7.03111928	6.71704165	6.88024405	5.53008065	7.23807087	6.93443851	6.04907136
BBOX1	2.20948479	1.47927997	1.53831642	1.09547955	1.44726954	2.24689504	1.23969184	1.5220768	1.25365422	3.79200524	0.68249522	3.30925678	-1.22027722
SERGEF	5.46912361	5.33000341	5.48024285	4.45815184	5.3570742	5.19465551	5.48552993	5.61269311	5.11814927	5.8516457	5.36061149	5.56918481	5.26143853
KCNC1	0.01232898	0	0.05121383	0.00865146	0	0.00069205	0.04927192	0.04783158	0	0	0	0	0

TPH1	5.20589362	3.9662949	5.79759251	4.05776602	7.54926125	7.15003993	6.77870973	7.90939233	7.4030979	7.24296007	7.57203134	7.47820245	6.63809207
E2F8	0.95893056	1.12043407	1.61892954	3.0072809	0.31248731	0.53999612	0.90825066	0.24266418	-0.49243868	-0.49243868	2.33758237	2.53635996	2.9984962
DCTD	6.11471817	5.74854917	5.61582313	6.11840537	5.50694043	5.1499561	5.53910772	5.47477593	5.90481635	5.68406124	5.77555604	6.05981868	5.93622466
SOX15	1.62970063	0.77579092	1.16601805	0.16448949	0.63617425	1.73223527	0.28241333	0.63060704	3.22594719	2.85165255	-0.24977342	3.16545002	-0.24977342
RPAIN	4.86703055	4.2759745	4.85677546	4.21257378	4.95627648	4.68901289	5.17461044	5.24888783	4.58838383	5.23590391	4.83025172	5.06445888	5.15349395
USP6	0.67263472	0.6244323	0.7010263	0.6244323	0.63875456	0.69767521	0.6244323	0.84585952	0.14847923	2.12062673	2.42611438	0.71206585	-0.30947581
SHBG	0.03380766	0	0.35165545	0.17428848	0	0.00069205	0.16516182	0.09729163	0	0	0	0	0
PLD2	4.75714539	4.89445717	4.54360154	4.04423355	3.88535214	3.49414335	4.20200136	4.55408546	4.41632777	4.03089049	4.87783579	4.25209535	4.15216967
CD68	5.37851561	4.55413177	6.43422134	4.96412544	4.71846119	4.55538956	4.55413177	4.55701789	4.53873766	3.97780886	3.65383143	3.4332197	4.45084213
TXNDC17	3.33168059	2.8162616	3.06377378	3.84192056	3.60573685	4.65615197	3.6839266	3.47628975	3.2021479	4.13735841	3.2334246	4.01185433	2.17943047
ATP1B2	3.06965284	2.87931385	2.02648142	1.89410847	4.28542648	2.80518762	3.55524719	4.46716951	2.94061873	4.646575	3.20534077	3.52437314	4.33772524
FXR2	5.79151959	4.57509394	5.32790812	4.3582145	5.2848099	5.26464974	5.46001449	5.31798182	5.03166983	5.36427744	4.7400429	5.68158907	4.88134133
KIF1C	6.96353522	7.00582305	7.2827851	5.79577971	6.95416493	7.01345865	6.83098968	6.94076425	7.27458712	7.49134132	6.91366352	7.02853666	7.17768421
MPDU1	4.09849044	4.10500663	4.81690983	5.24011781	3.43867961	2.94419041	3.47028892	3.30566972	3.27398465	1.66911171	3.39263805	3.57925878	3.60506855
PHF20L1	5.47072634	5.92026955	5.57029871	6.56228781	6.20440204	6.42804154	6.33141134	6.30659163	6.70855028	5.95015922	6.18792936	6.08799047	6.19110713
LRRC6	2.42633977	3.70985994	2.5363816	3.83543529	3.41248049	3.08622663	3.1274783	3.31785116	3.75189669	0.7176804	3.21506536	2.31080177	3.72824338
CCNT1	5.91493894	5.95105408	5.63940271	6.17977006	6.16375862	6.57177846	6.15277978	5.95295475	6.15244373	5.9387224	6.25696932	6.13432968	5.68712592
PUS7L	3.89401313	4.65324268	4.29442548	5.79673905	5.13862009	5.32769216	5.01485585	5.12452795	5.73618776	3.94375193	5.21604552	5.26537811	3.77131394
KRI1	5.10223681	3.98716896	5.80394977	4.1970698	4.1603926	4.3580858	4.23480596	4.40012837	4.81391458	4.6358315	4.01100859	3.83698592	4.68836064
ILF3	7.07784676	6.90105816	7.39925903	6.97481561	6.75339115	6.35542491	6.96018417	6.68080523	6.71714299	6.92695902	6.96950519	6.92467116	6.9092905
SLC44A2	6.84847218	6.36493166	6.71656354	5.73839176	6.46344705	6.76827985	6.46184116	6.54485354	6.1384183	6.55226724	6.64863461	6.25794595	6.15672463
CDKN2D	3.75012368	2.791106	4.09455945	1.96884363	3.13770091	2.28351997	2.14717992	2.24504559	1.9120497	2.35666329	0.45245133	3.75646624	2.03190644
MTUS1	5.46702289	6.42312307	6.09816653	5.02443725	6.35939312	6.13675211	6.15566377	6.18855464	6.11167392	5.80246097	5.89881856	5.69963362	6.91571995
KLK14	0.61656777	0.09881029	0.76869466	-0.16471077	0.61869633	-0.21082028	-0.08190315	0.58964985	-0.08487446	-0.08487446	-0.08487446	-0.08487446	-0.08487446
SIGLEC9	3.75141947	2.52223313	4.9733736	4.19369591	1.48114199	1.33279976	1.85396649	0.87906962	2.88508332	-0.93431348	2.23559629	-0.93431348	-0.93431348
KLK10	0.07498542	1.30501582	0.07737829	0.28315895	0.07498542	0.07631582	0.07498542	0.07803816	-0.06768758	-0.06768758	-0.06768758	-0.06768758	-0.06768758
NGDN	4.82089474	5.02709265	4.97226737	4.47216592	4.93980733	5.4914826	4.74822202	4.9743512	5.49180536	5.0595156	4.01180785	5.04558452	5.38564175
RIPK3	2.70068916	3.13509228	2.66886322	2.89960861	1.89577579	0.26384459	2.10956607	1.75388215	2.77244407	3.19570746	2.59601299	-0.74171508	4.16477498
ADCY4	0.95772372	3.52997195	1.57569456	2.59605272	4.49626437	0.75228148	4.14621064	3.25133799	4.28704984	4.55310352	4.0736848	3.76192777	5.43205699
RAB2B	5.47313996	5.76526239	4.95147625	5.51240259	6.02439573	5.98828995	5.9131183	6.23459636	5.87106558	5.10922497	6.04128375	5.90819268	4.92209105
BCL2L2	5.95832436	6.53918137	6.40445992	5.76890899	6.16130135	5.93486036	6.7020413	6.18745025	6.28413962	6.52887758	6.06409173	6.22409804	6.22481446
AJUBA	4.2744601	4.85706984	4.03689403	3.14343622	4.60233365	4.90117653	4.53589309	4.33456122	4.41165212	4.69346811	5.38862978	5.2456092	3.88504304
PARP2	4.35360354	4.03599527	4.06856144	4.30855907	4.15514448	5.02467083	3.77079444	4.42457991	4.8255647	4.53090339	4.55564956	3.9304366	3.81011425
HEATR5A	4.68514098	5.59172882	4.72477726	5.5324114	5.00034953	4.84092863	5.2660524	5.23792614	5.52941236	4.91532021	5.4077211	5.46807313	5.07223247
SNX6	5.03626924	5.66916463	5.12805365	6.5714715	5.70043371	5.88514906	6.04340343	5.92830438	6.00118838	4.87175703	5.73846876	5.4008592	5.10316517
EAPP	5.41305377	5.72743855	4.98615817	5.76811878	5.56871848	6.18571328	5.51917787	5.72743855	5.65197355	5.61620642	5.14828307	5.50258685	5.75513805
EGLN3	7.07836183	4.17982159	5.60044725	6.50795362	6.17528508	4.62339587	4.43917177	2.9993168	3.76324784	3.87567434	4.24771493	3.5697399	4.01370333
MIS18BP1	4.42921138	4.9066034	4.77938517	5.84138565	5.22602893	5.20859117	5.16047734	4.85520323	5.51840967	4.46631477	4.53261649	4.89493287	4.83879667
NRL	2.69227601	1.99667155	1.98635496	1.41324999	1.55771866	1.86083727	1.38820662	2.02769715	-0.71029173	-0.71029173	2.96028113	2.93885959	-0.71029173
RNASE1	5.71693632	6.52300422	6.54880884	5.93182294	6.05622834	6.77532065	4.7831483	4.32600766	6.26952551	5.92843991	5.14968431	5.00198391	7.04418649
NEDD8	5.06370472	5.13993369	4.49255028	4.82815286	4.87389038	5.06254346	4.66338993	4.9229858	5.14860676	5.36146896	4.83201109	4.84072615	4.84571665
DAD1	5.52764555	5.84022292	5.70055168	6.49998629	6.04078435	6.70032281	5.46841535	5.58909574	6.10288398	5.83111051	5.94967751	6.00821444	5.61257688
TEP1	6.04475962	5.59506755	6.16102762	5.6531553	5.66437141	5.06864868	6.05518667	6.20048688	5.40430873	5.57270371	6.00494664	5.58983812	5.71225394
EPB41L4A	1.05745032	2.28629703	0.43123003	1.72032636	1.98477466	0.75319107	1.83511584	2.19620101	3.31437251	0.03301689	3.09275845	0.03301689	0.03301689
CDO1	5.48322113	4.77815814	5.09213448	4.51337667	6.57333481	5.5743905	6.23936319	5.76199309	6.24332767	4.8015463	5.37494361	5.60703403	5.3608447
REEP5	6.63092841	7.25453389	6.26698675	6.89496534	7.38689237	7.6431201	7.38323034	7.35432793	7.41141511	6.59728649	7.56509297	7.55298095	6.57424061
ITFG1	4.89667622	5.3607903	4.91292826	5.82478069	6.12747843	6.47037152	5.68209091	6.03769325	6.07152245	6.04134047	6.24433233	6.34512221	5.73024022

QRICH2	3.0413493	2.280741	4.14691729	3.48851296	3.3692982	3.50505485	4.55463025	3.0989361	2.72594193	4.66591148	3.94987104	4.24448963	4.00228434
SEC14L1	3.04378403	3.10898448	2.37370586	2.95639361	2.23293524	0.55380864	1.75524638	1.92632259	1.85806409	4.29834897	2.53668508	1.85806409	0.12946745
RHBDF2	4.45139343	4.79414898	4.86318618	3.95092226	3.65731427	3.05680864	3.48968795	2.73285541	2.58940448	3.39509628	2.85678172	3.67228543	4.06168313
ARHGEF6	6.85241563	7.57088906	6.46559115	6.98216156	7.12530242	6.71382441	6.60493021	6.24997631	6.96970292	6.73254767	6.70436653	6.73646125	6.94950215
MAP7D3	5.74985232	5.3273793	6.28683685	5.26911108	6.00845798	6.14044913	5.90449044	5.98883024	6.33593601	6.34121975	5.26924573	5.79020546	5.88610466
FGF13	4.47190148	4.9315147	4.37856096	4.12976313	5.28607737	5.46814891	4.98227595	4.69980348	5.49278969	4.55248799	5.3271721	5.25327056	4.02075617
ASH2L	5.72529586	6.11569732	5.31355549	5.60391165	5.56509694	5.72439732	5.73018685	5.65190273	5.72573973	5.74371326	5.31541886	5.81460779	5.59114453
TTI2	4.62879209	4.53966472	4.01539639	4.05689691	3.99633589	4.10374148	4.2057716	3.66337689	4.24415437	4.829632	4.24232956	4.08116871	3.63182202
ART1	0.03965764	0.25382711	0.00124472	0.23086951	0.06187051	0.00069205	0	0.74776598	0	0	0	0	0
CHRNA10	2.24391898	1.34937687	2.01568344	1.81229764	1.15243972	0.64451048	1.12238291	1.73172054	1.32944216	4.12752562	2.45221149	-0.39890303	1.34088805
CDKN1C	4.23707267	4.01241108	3.27876738	2.06566111	3.36589636	3.93005515	3.54729531	4.79664496	3.8140122	4.68763632	4.76234101	4.72203987	4.36846569
RPS4Y1	4.27118404	5.19764117	3.70998068	4.42253441	3.48985254	4.20536055	3.02352051	3.61504777	-1.60420568	0.97818978	-0.72511243	-1.09960295	7.20626416
CDH15	-0.04088441	0.02223465	-0.0941927	-0.02818459	-0.22618246	0.09122832	-0.22618246	-0.22287493	-0.23011857	-0.23011857	-0.23011857	-0.23011857	-0.23011857
KLF16	3.42100722	3.76353543	4.98114316	3.37899003	2.30143209	1.73235308	2.65154245	1.45576304	2.35683145	4.15515892	2.26911269	3.45599539	0.03870395
TMEM8A	5.29000309	4.36654088	5.78600031	4.79961108	4.54629033	4.06646977	4.63822267	4.24714169	4.60270741	4.39701021	4.81298464	4.41895768	4.60270741
DOHH	3.82527457	2.48203775	4.68992224	2.60666346	3.49600482	3.35996672	3.44607524	3.52271734	3.95726616	3.64344737	3.60531653	2.79138109	1.75924649
MAU2	5.76136169	5.26243122	5.54777675	4.82756	5.45003153	4.85782802	5.37316254	5.49429962	5.22817048	5.51539536	5.39162134	5.42522989	4.95796387
SHC2	2.56299627	3.07611855	1.62229024	0.80419845	2.89957409	3.02142769	3.30978238	5.40612364	-0.33553821	3.66828222	4.20760273	3.88788221	3.98516316
LBP	4.48220925	0.33771201	0.10115204	2.65364101	0.60116969	0.87105862	2.40108265	0.10170865	-0.55426102	-0.55426102	-0.55426102	-0.55426102	2.93964999
SYT5	-0.02265151	-0.02265151	0.53342282	-0.02265151	0.03637783	-0.02132724	-0.02265151	-0.01961284	-0.04658728	-0.04658728	-0.04658728	-0.04658728	-0.04658728
CBFA2T3	1.88813603	1.92703901	1.44486825	1.600625	1.6372864	1.11252023	1.92852232	2.07390443	2.03503423	3.15571254	1.75807536	3.97720419	4.43328367
GAMT	3.89891317	3.51160765	4.32777366	2.84241394	3.68629175	4.06309618	3.87553503	2.93265496	3.75982375	4.54257132	2.42563693	4.38168657	2.30974385
PHF10	5.62942987	6.2828223	5.50577667	5.87233219	6.78468664	6.62079796	6.75779965	6.77655203	6.22013619	6.01196479	6.47205579	6.67367864	6.08357703
PRRG3	0.05646638	1.70823569	0.3125499	0.75082242	1.10305519	0.30573241	0.23099775	2.02072295	-0.33071139	2.12216473	-0.33071139	-0.33071139	3.41061608
GALNT8	1.22309772	0.27751735	0.22424993	0.42182354	1.58477166	1.53235158	1.50524978	1.41252074	-0.2994807	-0.2994807	1.6274923	2.46553095	-0.2994807
KCNA5	5.27626698	5.74416138	5.45921091	3.68156365	6.03792318	6.91654328	5.78606687	4.44850502	5.52504581	5.97609177	5.72212003	6.25921087	5.96117903
NXNL2	1.15122975	0.20590085	2.54239473	0.72775519	1.50647153	1.19299481	2.46694274	2.35203557	-0.48891656	3.17271778	-0.48891656	3.44212265	2.93655422
STARD8	2.30618058	2.94737084	2.8747022	2.50475384	1.82413191	1.02050271	1.87507668	1.49647799	0	0	0	0	0
FAM155B	0.77617058	0.73801839	1.01127258	0.82580495	0.79555612	1.2966041	0.94710331	1.03732966	0.16825869	3.10352985	3.85593873	2.40779964	6.07060896
SAT1	7.04538657	6.84113399	5.9680685	8.15574618	5.56372005	5.35305568	5.92293829	6.1710472	5.81762828	5.74747089	5.00264104	5.07091523	6.14221116
GNL3L	6.34802584	6.21359638	6.86227972	6.55483484	5.83362346	5.9270899	6.62279909	5.98923304	5.72038898	6.36544089	6.40650862	6.08037823	6.77826711
SH3BP4	5.59940766	5.8766844	6.02274428	5.08322735	6.02415879	5.30318628	5.12789822	5.1976789	5.44280753	5.53380972	6.13816021	5.37651637	5.71294569
MOSPD2	4.74253083	5.45199805	4.68583418	5.9959799	5.70112986	6.06372687	5.63520445	5.93620321	6.05921637	4.75058172	5.80164682	5.47962026	4.75248441
DOCK6	5.46828123	5.45162997	5.80827515	5.24908851	5.60571719	4.14715185	5.03378609	5.20634812	4.93651779	5.52809335	5.09876498	5.01476503	5.4035328
ECSIT	4.8570507	4.28209091	4.9335937	3.19106872	3.85705743	4.07968914	4.19595914	4.38312141	4.28207571	4.9622562	3.55587557	4.35707026	4.88889705
LDLR	5.20649278	3.63999233	5.19766452	4.79108169	3.4861246	3.03276393	5.32646966	4.30623813	3.49337843	1.40244717	3.88369155	3.86734204	4.22711563
ELOF1	4.6982437	4.33713774	5.06620375	4.14207322	3.85694824	4.11087295	3.55185971	3.74783719	4.59476427	5.19350402	4.24831129	3.99437356	4.1999938
PRKCSH	6.55547792	6.31751757	6.98650487	6.5662413	5.75942016	5.66753577	6.1186929	5.74881109	6.28722897	6.70837041	6.31857653	6.35087363	6.0899667
CNN1	7.14888006	7.52797584	7.27165638	4.99862008	8.84365457	9.18615551	7.95157726	8.73788147	7.73990232	9.32779831	7.99563118	9.10888719	8.40291346
CDC16	4.99034894	5.53033108	4.9228965	5.70714609	5.67441214	5.20983602	5.87049726	5.76289503	5.28527791	5.08594513	5.56233597	5.40245217	5.48833101
APOE	6.93311641	4.63373984	5.38399879	5.19569557	3.9110056	4.35900126	2.44776264	2.83768801	4.1533506	4.45350429	4.73353756	2.87577105	4.48360556
TOMM40	4.53474769	4.26533787	5.86442193	4.4167605	4.52947803	4.07047406	3.33125985	3.5135368	4.20979075	3.92347559	3.48698643	4.26680558	3.80275422
APOC1	5.1318706	1.14456706	2.80575206	2.99947366	0.16086458	-0.46213046	0.42279594	0.32174589	-0.36585852	-0.36585852	-0.36585852	-0.36585852	2.01560578
GADD45G	3.15757046	2.6503324	4.36085219	1.53576053	1.67593849	3.97045319	1.14809465	1.67228092	2.87361342	3.27117371	-0.91725559	2.729145	2.45617078
LRCH2	4.20687578	4.6627737	4.24109482	4.74986103	5.19585237	5.65603217	5.21342361	5.41238541	5.28335061	4.70774727	5.07600065	4.58375747	4.75293641
DPP6	0.04502677	0.48468137	0.00124472	0.01032169	0	0.00069205	0.01655384	0.04482247	0	0	0	0	0
XPO7	6.72066427	6.67098365	6.13912513	6.4600575	6.65339549	6.49955598	6.75536829	6.58260482	6.56673869	6.8568496	6.7001332	6.63281478	6.66065814

ACE2	1.14033708	1.31518259	1.14286946	1.14952834	1.16455482	2.69952418	1.14033708	1.30548592	2.37895098	3.14723511	-0.33745347	3.75414257	4.00916932
FAM98C	3.73658215	3.1317818	4.69970633	2.35168416	2.99705136	2.82433225	3.69118442	3.24869747	3.6358758	4.79515333	3.55878328	4.13817497	-1.23827011
SAFB2	6.0712963	4.8392659	6.06941123	4.84792355	5.18115716	5.16858745	5.49243042	5.05145842	5.57689889	5.57053046	4.70274154	4.58849063	5.6829605
RPL36	5.5098374	5.7897503	5.58654842	4.70166473	4.79517652	5.62107606	4.33137484	4.72181782	5.93688665	6.03338887	5.13573927	5.30044326	4.74300897
ATP8B3	1.7452047	1.16720499	2.92061217	2.09375479	1.8783103	1.11590798	2.13655996	1.94021186	-0.81763721	2.85378653	0.26786581	2.9851935	3.27374321
KIF1A	1.06765159	0.94616203	1.1261703	0.98568708	1.45977494	1.21933008	1.25631075	1.04135844	0.76982467	2.03274903	2.91617862	-0.4254975	5.24467437
GTPBP3	4.25092883	3.78831189	4.6237261	2.93750745	3.15861471	2.93750745	3.62948669	3.24111695	2.97474404	3.63241524	3.39152863	3.41912444	3.59593175
PLVAP	1.73012031	5.87282587	4.52712989	5.68193983	6.28475003	3.96083596	4.55120059	5.08275557	6.11830474	5.90160534	6.53369392	4.88918778	6.86814701
BST2	6.62801806	6.3897187	5.26705493	6.38668347	5.15407655	5.2451566	5.723274	4.24495502	6.12111178	5.98142369	5.88918071	5.35527875	4.72855274
SLC27A1	3.2108344	2.01116239	3.04734637	1.5487997	1.15002665	0.2575021	2.18890546	1.51142668	3.77315302	-0.26712073	3.76703876	3.79695857	-0.26712073
NSUN5	4.35494493	3.84323564	4.81007746	3.14299326	3.6288745	3.24376051	3.58279044	3.68224615	4.03309396	4.37091899	3.99611494	4.18481252	4.1381327
USHBP1	0.18589084	1.02567986	1.11617299	1.54780309	2.47947091	1.31298806	1.3433975	1.59440292	2.43476236	3.05681735	4.19516793	2.53929628	3.46476285
DDA1	5.17082431	4.44718309	5.28389127	3.98646944	4.46834814	4.5305298	4.73587161	4.24382693	4.96916237	5.03182182	3.73194269	4.63305011	4.00926867
MRPL34	4.72866975	3.78860934	4.5711	3.57341481	3.4560281	4.79379775	4.01210551	3.54417403	4.22393189	5.07630503	4.51134637	3.97008704	3.83069171
PGLS	4.02212582	4.12909438	4.91594175	3.33981263	3.14857813	2.41857706	2.74309414	2.89107863	4.24713383	4.29178972	3.88515567	4.37343447	-0.79762993
LSM7	3.41029851	1.45432387	3.62846466	4.03103969	3.24613725	3.44081154	1.75850401	3.14845446	3.58496267	4.08672378	3.59009339	1.42836773	2.97409836
TULP4	1.5454703	1.51138418	1.18828155	0.90871402	1.58220293	1.27048386	1.81526595	1.74237939	0.39348354	0.39348354	0.39348354	0.39348354	0.39348354
SNX9	7.75528959	7.48742303	7.22955365	6.56458366	7.39847825	7.41132656	7.43901971	7.37039256	7.2119577	7.41234037	7.01903404	7.03257302	7.38148776
RTN4IP1	1.89675787	2.13347339	3.31366248	3.65441939	2.295244	3.38816314	1.57574033	2.60589867	2.93338562	-1.02368204	1.75794552	2.8385004	3.38085866
QRSL1	3.6942199	3.76187887	3.2073568	3.61407817	3.67314004	3.52566564	3.51334466	2.92386212	4.02435609	3.29099197	4.4944213	3.39084359	3.01671262
C6orf203	3.89483799	4.47275391	5.02548084	4.58676932	4.14059541	5.25915246	4.8144103	4.85922663	4.99352669	2.90722295	4.19862931	4.06595493	4.87923619
RSPH3	5.06559235	4.38843557	4.21640814	4.11439526	4.07073889	4.47377837	4.11317329	4.00974033	4.45891394	4.31267181	4.29817021	3.52123282	4.99223755
MAS1	0.01459206	-0.04894964	0.02165799	-0.04894964	0.07934951	0.70292404	-0.04894964	0.13949674	0.02862393	0.02862393	0.02862393	0.02862393	0.02862393
ACSBG2	0.67153648	0.64705187	0.64963898	0.64705187	1.24527809	0.87984505	0.82908156	1.05499357	2.90487015	3.33136442	-0.34564507	-0.34564507	-0.34564507
MLLT1	6.25548629	5.20676564	6.36617864	4.8660161	4.92378174	4.83961181	5.2204709	4.82212792	4.96307695	5.19089874	5.89068516	5.18131777	4.84751642
ACTN4	7.60530444	8.7154858	7.93903301	6.96089558	9.36665594	9.03194478	7.98766799	9.14458656	8.59224801	9.11277385	8.89418522	9.12650694	8.73105121
STK33	1.41797158	2.87707475	2.10207593	1.73962525	1.94422205	2.76396502	1.85092725	2.02989773	3.7788382	-1.06663656	1.158655	3.25388525	5.27402031
NDUFA10	5.73852275	5.64965294	5.41788211	5.50085333	5.84109045	5.29723737	5.79204017	5.95945115	5.22017993	5.71554954	5.87485279	5.51727876	6.08157243
ARPC1B	5.30479545	4.87249981	5.94163218	5.42079886	4.88973153	4.06146012	5.41677422	4.79804578	5.41242447	5.47234131	5.40637351	4.79589612	4.5592054
ZSWIM6	1.64567405	1.31353098	0.79653963	2.23280128	0.91915557	0.91991158	0.97501218	1.23755044	0.46380076	0.46380076	0.46380076	0.46380076	3.19675803
FCHO1	2.36589061	2.52359633	2.5724021	2.40044645	1.56420529	0.59394939	1.28550459	0.62604722	3.2698336	-0.67926317	-0.67926317	1.83631322	2.14418998
UNC13A	4.23072169	3.5682033	3.96130039	4.35000363	4.75051209	6.16042504	4.44594306	3.81257061	4.50814668	3.34062856	5.65395646	0.83745198	4.80765581
MAP1S	4.57167743	4.04124802	5.49293908	3.85168118	3.67813769	4.04176543	3.52755656	4.01399415	3.33018704	4.11545711	3.33743145	3.87347064	3.10696787
KLHDC7B	0.3562005	-0.05050348	0.21594336	0.89131914	0.24856547	-0.04910319	-0.00717036	-0.04729037	1.65560018	-0.3106214	-0.3106214	-0.3106214	-0.3106214
SCO2	1.66526807	2.25272022	2.8737512	1.66526807	1.66526807	1.6666779	1.66526807	1.66850307	2.50857745	2.82574458	3.84398957	3.6020678	0.60427008
PXDN	6.4549833	6.32704687	6.82266376	7.55458864	6.95533728	6.55507974	6.85881182	7.40978605	6.97661894	7.49049574	7.39722073	7.6456434	7.05624784
SSBP4	4.3754172	4.26120896	4.62471587	4.17044818	2.87932483	2.54072308	4.17343942	2.95601343	3.66840471	3.78274528	3.89296265	4.08688088	0.79289387
GDF15	4.46711903	-0.08475607	4.0735597	4.0945312	0.91300493	1.64591247	1.04387244	1.06958324	-1.20697681	1.99380081	2.36279726	2.95600018	-1.20697681
PGPEP1	6.5990178	6.14476333	6.2941982	5.22925972	5.73792033	5.27168247	6.27843115	5.70626404	5.41304548	5.929628	5.95165487	5.50123225	5.57908405
KIAA1683	3.43428653	3.73986409	3.1872409	1.85623408	4.30499318	2.85574506	3.90239381	4.84955261	3.59145633	4.72424137	3.91025343	3.89238907	4.54955714
LSM4	4.21970264	4.06857658	4.96180784	4.30951666	3.71775384	4.37198562	3.50546531	3.18223165	3.8751932	2.84953704	3.95190045	4.05919795	4.17315258
JUND	5.11788791	5.63303504	5.73809645	4.00014724	6.30924145	5.94438871	5.10779899	6.17998142	5.4228635	6.14896426	5.60661943	6.18279947	5.03141264
HRC	0.70994676	0.79041379	1.94313935	0.42082836	1.75156423	4.93981797	3.41718841	1.93439493	4.0069449	3.968851	2.25060575	0.99434468	7.70082873
TRPM4	4.21324341	3.06423875	4.65691093	3.97461038	4.17730219	3.89392593	4.71091321	4.94417576	4.16068912	5.20551253	4.93968933	5.08628062	3.73349916
ZNF557	4.54183067	5.13542367	5.16083536	5.24578035	5.22113734	5.2964102	5.28483732	5.40834085	4.84241167	5.39217765	5.38972162	5.15928689	5.56136046
OLFM1	3.28578771	5.00739064	2.72713652	3.00247347	4.73720863	4.30856348	4.36947959	3.38132972	4.3180021	3.75469834	4.0137455	3.59081752	4.89852555
CAMSAP1	5.59964391	5.67165813	5.70641963	5.00361551	5.15059961	5.46612722	5.35961901	5.21060597	5.51114669	5.54792372	5.33577775	5.1763286	5.34088805

UBAC1	4.98248454	5.0943537	5.00564703	4.19558093	4.36735937	4.2963173	4.8527959	4.81895452	4.70733328	5.15563298	4.27126836	4.84699038	5.20301328
ZBTB46	3.92418938	3.95188152	4.49679029	3.73289872	3.98545366	3.04744893	3.19118486	3.67703656	3.57580067	4.39038975	4.75155439	4.33339789	4.0363823
SAMD10	1.86128219	1.37515087	2.96427411	0.184745	1.91077723	1.05678821	1.21583223	0.95139396	3.20593113	-0.5782252	1.92515108	-0.5782252	2.72674045
LSP1	3.87874061	2.73036425	4.15404506	3.18634802	1.53415473	0.02432266	1.23358414	1.179697	3.46414304	4.41728446	-0.12301459	1.91906585	4.43853868
TNNT3	2.83142725	1.95640021	1.04440649	0.60954612	2.07045582	1.28753659	0.92182567	1.99239451	3.00629095	2.98157521	-0.54946179	3.56902353	1.52700758
TNNI2	1.09259478	-0.15055642	1.8492696	0.92872992	-0.15055642	-0.14928057	-0.15055642	0.75876976	-0.248286	-0.248286	-0.248286	-0.248286	-0.248286
COL5A1	5.72476373	5.73069183	6.71100955	8.10276346	6.08720526	5.42381649	5.94086758	6.54018924	6.46043568	7.16987581	7.26122449	7.36638206	6.34381871
ATXN10	5.29797625	5.9478242	5.67936842	6.29513943	5.91293833	5.82518514	6.3147081	6.2554165	6.01191652	6.36719599	6.2128219	6.04817818	6.25484647
TUBGCP2	5.97454566	5.20136395	6.07522441	5.19455861	5.45430122	5.16484865	5.18593368	5.06099411	4.10495942	5.34219891	5.39126828	5.73793292	5.51691944
CALY	0.08286461	0	0.00124472	0.02960834	0.02245113	0.00069205	0	0.27040318	0	0	0	0	0
CYP2E1	2.33567916	2.43523386	2.1931818	1.44929654	1.9763953	1.38387613	1.85827797	2.42626504	2.32900104	-0.87215664	2.17279785	2.20561997	2.12227449
PNPLA7	4.83561443	3.63328198	4.85704848	2.15637334	3.94827336	3.57396722	4.46295515	4.93860798	4.38885988	4.75812393	5.18715385	5.08911144	3.48088752
PAK4	4.3341604	3.85740353	5.00291662	3.76244095	3.52528501	3.57932782	3.49929559	3.79746154	4.15265321	3.59460935	3.68575905	4.12190744	3.29803397
ZNF337	5.79662307	5.74759055	5.56220938	5.49127537	5.5448323	5.72649918	5.34099024	5.51041305	5.62905488	5.52470347	5.72833798	5.78097394	6.22819743
CEP85	3.85262532	3.0470684	4.13397527	4.39032313	2.28560867	2.93454336	2.99505864	2.46346744	3.10248631	3.32976971	3.45595245	3.42104415	4.54577857
TAF4	5.28195097	3.90075176	4.89378226	4.31172302	4.83267588	4.62858326	5.1488983	4.66259358	4.73049381	3.61661655	4.09251814	4.29486777	4.15943724
GATA5	3.01305195	3.93945865	2.88700532	2.39159023	1.63163222	1.46627641	1.85000054	2.43585841	2.96786491	2.81413354	1.74880803	2.96163672	2.42096133
LAMA5	5.31452154	6.13438611	5.97822493	4.60570272	6.64921035	5.89699637	6.35220881	6.60003229	5.55959749	6.80263145	6.40416612	6.78286084	5.88323147
OSBPL2	6.00746551	5.63401873	5.87703886	5.17533881	5.95643168	5.99726634	5.84879453	6.14214808	6.0381404	6.42644027	5.85512211	5.99169258	5.9964973
ADRM1	5.44240505	5.03210957	6.270941	4.72605254	4.57447772	5.04735696	4.38041107	4.81826301	4.92951137	5.29978299	5.00618966	5.72051706	3.96675902
ASS1	6.88055721	5.94730729	7.0293439	6.08113574	5.32261535	4.8630727	5.49580644	5.54017924	5.64375269	5.76228762	5.38433352	5.693679	4.92572113
EXOSC2	5.5152261	4.62811618	5.28001172	5.38001164	4.61759142	4.51175085	5.29706754	4.72292679	4.44958135	5.08516134	3.5720961	4.96589694	5.35558212
POMT1	4.94815733	4.58474257	5.04853635	3.86104071	4.39732183	4.1506748	4.63762639	4.91576398	4.35951366	5.41171789	5.23246466	5.17276676	4.72132762
UCK1	5.54129053	5.10656505	5.47993855	4.23414421	4.60196333	4.70326036	5.01032848	4.43590417	4.867823	4.84729135	4.7696851	4.94541614	4.47323627
FIBCD1	0.2094267	-0.03574363	0.21899323	0.12757201	0.05787287	0.39684104	-0.03574363	0.1376436	-0.04426039	-0.04426039	-0.04426039	-0.04426039	-0.04426039
PRRC2B	7.69804581	7.21113206	7.55825254	6.70153673	7.19419198	6.97732294	7.27621645	7.09174216	7.08977254	7.45936298	7.46650314	7.22037407	7.36653124
CHMP2A	6.7606155	6.36916892	7.19900516	6.37335897	5.97503934	6.66623852	6.05593554	6.05110734	6.73211355	6.48838277	6.07867448	6.3213719	7.07904669
UBE2M	5.60724972	5.18992222	5.60680406	4.39429249	4.84666225	4.79014341	4.53619896	4.00473174	4.79673336	5.35480739	4.3589577	5.21485527	5.04288838
TRIM28	6.81240867	6.18651113	7.12077034	6.19320697	6.12524286	5.60085484	6.46135515	5.94560536	5.88931905	7.08494021	6.38086502	7.02512841	6.11625944
YIPF2	5.05641027	5.34512131	5.32421648	5.17890697	4.53440944	4.20576634	4.15840602	4.3043272	4.83585629	5.31993572	5.31993572	4.96953575	3.96119335
ATG4D	3.68242956	2.80667158	4.12652107	2.09489939	2.89922699	2.28145199	3.04626271	2.75424562	1.84616076	3.28909727	3.53311349	3.43101644	2.54011552
TMEM160	0.7536401	1.76286583	3.28460025	-0.35143286	0.8474386	1.01775918	1.1031851	0.40499805	3.14109071	-0.09955038	3.92601712	3.69042478	-0.09955038
ZC3H4	6.06267816	5.52614743	6.47614124	5.05462648	5.32634857	5.61987024	5.34104565	5.33835776	5.02900597	5.99204546	5.25827702	5.51384464	5.34003275
NPAS1	1.17371786	0.80942539	1.01495925	0.57715383	2.1406044	0.62238808	0.74282085	0.00794472	-0.28751917	-0.28751917	-0.28751917	2.49122942	-0.28751917
GMFG	4.27135795	4.43051215	3.9173272	3.98675518	2.34548212	1.54915299	2.44784742	2.26375257	4.26303041	4.05667519	3.26701977	1.4659258	3.87162723
MAP3K10	3.87910117	3.31899789	4.37658642	2.90973539	4.23415243	3.16940771	3.86842617	4.03896501	3.35732319	3.73236323	4.10330791	4.12111171	3.17275129
ARHGEF16	0.08453504	0.29033827	0.03101139	0.06790444	0.43945368	0.40563157	0.24732891	0.03172348	-0.14730004	-0.14730004	-0.14730004	-0.14730004	-0.14730004
LRRC47	5.83525841	5.51885846	6.01084247	5.12392141	5.41316174	5.47491489	5.22591122	5.12472897	5.20337606	6.12512396	5.37663605	5.39132174	5.48262735
SESN2	4.78937451	3.29214099	4.92224573	4.04494508	3.12357678	4.00639297	4.20239639	3.70203122	3.06170763	4.06968574	3.8985117	4.2800589	3.20261256
SMPDL3B	0.30046536	2.49609976	0.05191491	0.55777427	-0.08069287	-0.0795906	0.34717075	-0.0781636	0.81270159	-0.15931537	2.86129735	2.67917399	-0.15931537
ATPIF1	4.65944233	3.13639363	4.8546877	3.90860113	4.03204828	4.34980489	3.73592569	4.16851382	5.13225946	4.59906769	3.82357829	3.77004483	5.27124585
MED18	4.77375046	3.7853023	5.33587755	4.93220426	4.01266057	4.51606889	4.71989516	4.15901052	4.75549839	4.43618108	4.39037572	4.25475079	4.7859478
CLIP1	6.29444942	6.72533559	6.27212465	6.10138713	6.86895735	7.05755599	6.65081817	7.22770735	6.93921463	6.58311265	6.27586647	6.64359877	6.48325631
CCDC62	1.92615422	1.69921376	2.08626618	1.92172796	1.85833297	2.83360869	2.49907503	2.23485421	2.73871289	3.8248181	2.60120421	3.04197346	2.17682445
HIP1R	5.44160904	4.36093826	5.5996538	3.47054129	4.99859859	4.95524817	5.80893976	5.96801419	4.92089542	4.16237013	5.1204135	4.40884184	4.51745351
ZNF317	5.98917009	5.6292781	5.77877627	5.02385636	5.34762452	5.60268118	5.08286829	5.41809432	5.14865464	5.30847138	5.40539728	5.22925163	5.7736688
PPAN	0.96155674	1.00916285	1.71077558	1.16770249	0.53825666	0.4884439	1.04528018	0.70652306	0	0	0	0	0

EIF3G	6.4061243	6.46881369	6.75337944	5.84292514	6.10156456	5.9757359	5.664704	5.94780144	6.32554433	6.82424504	6.04225765	5.96980006	6.2449678
ANGPTL6	0.01532111	0.17778498	0.00720899	0.23309705	-0.08730564	-0.25628413	-0.25761059	0.16733912	-0.11662186	-0.11662186	-0.11662186	-0.11662186	-0.11662186
C19orf66	5.73241934	5.43205537	5.39601015	4.85762278	5.21498003	4.50841381	4.86773293	4.7945289	4.89985485	5.5056194	5.11770266	5.23177227	4.7134923
DNMT1	5.39446029	5.12659765	6.01004145	5.79858941	4.88139563	4.58539529	5.19873568	4.99147031	4.96541041	4.766721	5.17061388	4.95108251	5.37791213
ZNF426	4.72399119	5.6913585	5.11035088	5.05149742	5.41079627	5.49072237	5.42217575	5.26602268	5.29750274	5.49927255	4.75488508	5.68864926	5.18509594
SLC6A8	6.1988893	3.73937196	4.89557111	3.8634843	4.86246032	4.27169917	4.3165302	4.25651933	2.05258411	4.49446952	3.81769279	3.91548435	4.19963962
PNCK	0.69913322	0.14177423	0.3855521	0.03349566	1.43222408	0.47297427	1.54311823	3.02494309	-0.21538119	-0.21538119	-0.21538119	1.69177637	-0.21538119
DKC1	4.74549349	4.59623173	5.38778012	5.81121117	4.69616693	4.61053449	4.61557116	4.42468319	4.1482407	4.47120376	5.0365562	4.21843604	4.82804518
PLXNA3	5.79268485	4.88790429	5.5385374	4.91293158	5.3275717	4.21236355	5.07072048	5.39163819	5.24719278	5.37301701	6.23941374	5.79979642	5.07490342
MPP1	5.40744385	4.64331828	5.46903209	5.59147105	4.77777211	4.77644265	4.68899517	4.30150611	4.31320001	5.30749611	4.34409258	4.84100368	5.3076559
ZNF331	6.15581323	5.824499	5.70769485	5.89291131	5.61088974	5.48251553	5.37606766	4.95375056	5.45493681	5.80130731	5.32645458	6.16706126	5.71932868
ZNF236	3.20971913	2.19522969	1.80608441	1.81844578	2.1959227	3.11485208	2.57537676	2.85794459	3.50525235	2.51628889	1.16318815	1.95781712	1.15285835
LRP3	4.72775211	4.79940745	5.2291964	3.78531613	4.06176055	3.64942896	3.72210258	3.61174436	3.85413753	4.73200523	4.496649	5.02097538	3.98396803
C12orf65	4.38270729	3.81080189	4.05618905	2.99926552	3.74838523	3.92617193	3.87232469	4.11982315	4.06906695	2.57797676	2.91914463	3.59978033	4.09057713
NOL11	4.70339241	5.23063682	4.61327626	6.05148021	5.59883633	5.9103288	5.5535365	5.8311674	5.28298348	4.97161952	5.06552649	5.58842864	4.9787506
UBE4B	4.55763845	4.07886467	4.23301723	3.8611581	5.04541053	4.73079287	5.34994594	5.38830944	4.74922901	6.21320734	4.18125713	5.17398762	4.19128389
CASZ1	4.70295259	3.84380337	4.48505316	2.60611615	4.92107313	4.78864909	4.58339182	5.23671784	4.41168305	5.5732601	4.72466046	5.76830937	5.1542692
PKDREJ	0.68382017	0.64362424	0.84464341	0.66240559	0.68467891	0.89609293	0.88168434	0.93047658	2.49604767	3.32525241	-0.44175615	-0.44175615	1.58921405
HSD17B3	1.25372284	0.77524804	1.21505282	1.00617757	1.76314525	1.2451948	1.83285025	1.2860185	2.3732423	-0.28774898	3.87595261	-0.28774898	-0.28774898
HABP4	5.47966753	5.26336228	5.86034486	4.72264589	5.9302119	5.79052573	5.51458545	5.95209467	6.12649095	6.11491355	5.59139572	5.77298693	5.89336831
FBP2	0.68562202	0.36422715	0.98783142	0.36422715	0.77105973	1.67534395	0.48729663	1.24451192	3.37377533	3.84024819	2.93268484	3.48104909	-0.36725921
SLC35D2	4.63028253	4.76253873	4.57016401	4.95980945	5.21388648	4.51954777	5.18791857	5.58731246	5.10621813	4.76541354	4.20056623	4.90400521	5.10519524
PRRG1	3.45225755	4.24072187	3.09578262	4.49073206	4.53419484	4.77558573	4.34574004	3.86559656	3.65467759	2.89059544	3.46006784	3.13969904	3.54181418
UBA1	6.48967366	5.8343809	6.76728161	6.49761731	5.90128193	5.61563681	6.41822488	6.04152401	5.71546034	6.88605404	6.38047132	6.89842987	5.91906125
RGN	5.19555229	5.20000966	4.99619118	3.90233651	5.06253808	4.99313303	5.42049762	5.08374697	5.3628949	4.93293901	5.26391141	5.3553057	5.11252001
POLN	1.10925924	0.32423105	1.12950925	1.11060351	0.73774019	0.08888133	1.24810095	-0.12874844	0.02176417	0.02176417	0.02176417	0.02176417	2.75728084
PPIL4	4.47068832	4.99777368	4.53247339	5.24554496	5.16402043	6.28953226	5.10833429	5.30019946	5.74352782	4.1783084	4.83297073	5.67354699	4.43088946
ULBP2	2.04483689	0.29509808	2.33112289	2.38341591	0.66661558	0.9895162	1.4653348	0.29813923	3.71947417	-0.69673089	-0.69673089	-0.69673089	2.9893294
AKAP12	4.01320488	4.72279093	3.36614259	4.53044943	4.56595356	5.06105995	2.8530024	3.28241815	3.43778381	4.43441683	3.28673744	4.1906566	-1.10462345
SYNE1	8.00299939	8.35780156	7.9265977	7.18432486	8.78579015	8.77567744	8.86795542	8.84180659	8.63057873	8.65515589	8.715335	8.78073848	8.98806134
ULBP3	3.44880605	2.16946458	4.26865228	2.75774137	1.64075504	2.21377463	1.96089706	1.48338108	1.69145942	2.6334497	1.41277744	1.69145942	3.45438827
LATS1	6.37323381	6.17079295	5.72879857	6.20680401	6.58219253	6.65036648	6.54742055	6.74936125	6.2203901	5.94910483	6.60184836	6.57239223	6.26305002
EPS8L1	-0.05483271	1.84886926	0.11356799	0.20086396	-0.14563483	-0.03259085	-0.16804594	-0.09757649	0.8405992	-0.03001331	-0.03001331	-0.03001331	-0.03001331
LILRB2	4.0248797	3.78455155	5.03904839	4.84844732	2.64041036	1.56358179	1.85274708	1.68617822	4.25716412	-1.56094481	3.44050038	3.13080466	4.38055804
TTLL9	0.66271563	0.31106216	0.57745615	0.39303252	0.70725653	0.68967107	0.69431656	1.03711317	2.19872709	-0.28768527	-0.28768527	2.21030467	-0.28768527
RBM39	6.40878811	6.77949118	6.31688675	7.2304864	6.99065895	6.97249385	7.29663088	7.17888885	7.28200743	7.07030924	6.92213599	7.19178317	6.78828968
COX4I2	0.08316389	0.08316389	0.41449875	0.08316389	0.32241357	1.62334757	0.49780917	0.6419435	0.17705541	0.17705541	4.07740331	0.17705541	0.17705541
ZNF341	2.42314988	0.63755993	2.34507884	0.96150431	1.08601027	1.317152	1.57857038	1.54355621	-0.36491535	-0.36491535	2.13460026	-0.36491535	3.20845859
GGT7	5.44100466	4.83856944	4.81229139	3.84964929	4.90002162	4.18776416	5.06796128	5.09899186	4.54484082	5.66060398	4.69047648	5.37886428	4.63362668
ACSS2	6.42541563	6.15821681	6.24528572	5.36037284	6.02283634	6.31679915	6.50041396	6.52705277	5.69875231	6.39424612	6.16942873	6.34291777	6.28025917
EDA2R	5.56268961	4.74808235	4.73307458	4.94360443	4.5836395	4.91277084	5.27749049	3.95553209	5.50895939	5.21159454	4.97309336	4.4592581	5.34071667
ARHGEF9	6.09156298	6.33137333	6.1161925	5.44039273	7.23988885	6.87736804	6.63348511	7.2332574	6.42252772	7.10076637	6.89191156	6.9559963	6.91358292
C1QL1	0.9331375	-0.22656085	1.7877923	0.38505475	0.25860016	0.21198212	0.17331988	2.10517659	-0.12860732	3.82613296	-0.12860732	-0.12860732	1.98001292
GFAP	5.58449453	1.97629409	5.17159576	2.16443314	2.37480143	3.11802306	2.91010012	4.1742872	3.42064544	5.6053848	3.39476506	4.11479406	1.86273675
PYY	1.06105046	0.33644275	0.59672098	0.80599627	0.33644275	0.33785715	0.33644275	0.33968824	0.95436346	-0.23001908	-0.23001908	-0.23001908	-0.23001908
HIGD1B	-0.04752126	-0.04752126	-0.04539418	1.90300979	0.42710242	1.07068282	-0.04752126	0.25418539	0.27439038	0.27439038	0.27439038	0.27439038	0.27439038
ATP6V1E1	6.47118755	6.25493879	6.10417007	5.94316622	6.66447093	7.06849694	6.31875497	6.72100797	6.72392919	7.14793711	6.42576165	6.74970606	6.87737463

ZNF227	4.87849556	5.0610427	4.26098439	5.05792212	5.08112828	5.48409546	5.16800583	4.8500155	5.19143866	4.80756521	4.64488844	5.14640666	4.50879259
ZNF428	3.72452405	3.6034947	5.38184305	3.95596255	3.80882753	3.8683736	3.59534562	3.50776799	4.63755538	4.85764798	3.91809869	3.4382071	3.56425931
ZNF141	3.78990879	4.20839654	3.13053112	4.19733228	4.2565908	4.98514041	4.37903563	4.59040197	4.64233682	3.77918772	4.60123717	3.92371242	4.25416028
COX4I1	5.72480519	6.23536295	6.0844957	5.88514564	5.24068684	5.38213209	5.18400286	5.25798747	5.995172	6.45399588	5.73819193	5.53361974	6.53571819
GINS2	1.73579822	1.20332526	3.17847274	4.18386469	1.10805086	1.47865271	1.56489829	0.86666573	3.68264995	2.16089958	3.27561337	2.60865043	2.42289661
CHMP1A	5.41316852	4.93873213	5.69818248	4.65361537	4.65230512	4.49798051	4.58581491	4.91349282	4.83691481	5.00252254	5.17158256	5.5977569	4.92180489
SH3BGRL	5.77424608	6.50671181	5.69341875	7.23533023	7.43419066	7.76818826	7.3674495	7.67384836	7.59025975	7.22599171	7.53107687	7.60026772	6.13201353
COX7B	4.79970212	5.21249671	4.72968086	5.35198952	5.12475312	5.21956438	4.48591318	4.3817565	4.90402675	4.89795606	4.49880776	4.67552708	5.7162059
F12	0.16767532	0	0.25824183	0	0	0.2617209	0.14868655	0.00158797	0	0	0	0	0
PRR7	1.41520275	0.44981977	3.30613374	0.79343886	-0.33220908	0.22540883	0.15625597	0.6559624	-0.34963508	-0.34963508	0.72098023	3.83614991	-0.34963508
NFATC1	5.27643002	4.57271153	4.91951595	4.10602916	4.84048496	3.69347823	3.79863205	4.07304215	4.21127352	3.76669409	4.77396056	4.18915717	4.449034
IDO1	0.67828545	1.35101793	0.6447021	1.74454186	0.89811558	0.64356458	1.01460546	0.88040881	3.93265305	3.45903613	3.56664535	-0.53129909	-0.53129909
CAP1	7.58097738	7.94065113	7.27311965	7.85643768	7.94065113	8.0033597	7.80745947	7.66787217	7.38479911	7.81218618	7.48260465	7.67116102	7.98498774
PPT1	6.39104379	6.41014945	6.43136331	6.99243694	6.03997559	7.03358446	6.33616364	5.91397624	6.55415796	6.84956241	6.34983646	6.88736316	6.70454055
RAB11FIP4	2.66843091	3.2230623	2.04883433	2.32280015	1.89969117	1.8027592	1.90931069	1.67052712	2.91176106	3.49882536	2.89775784	1.2291688	3.7590308
RLIM	6.20370752	6.66646536	6.28483716	7.45073355	6.97617753	7.14754489	6.95157971	6.80548445	6.83179884	6.29578168	6.92056181	6.64141154	6.58449602
ABCB7	4.76443491	4.86057728	4.50179209	5.14360959	5.18331312	5.27311701	5.29153489	5.45063588	4.7347727	5.46484666	5.48805085	4.95014232	5.20548201
TRAF3	5.65760705	5.25795252	5.69618019	5.29416872	4.50430094	4.10973898	4.89920592	4.1578325	4.38517364	4.73975018	4.75321647	4.58690054	4.85039334
HAUS8	3.38703867	2.3085126	4.04521917	2.52437872	2.95793189	3.39343055	2.92355174	3.56605394	3.45991687	1.38586787	3.12376737	2.89207216	3.54385495
MRPS25	5.2644674	5.41474743	5.31729697	5.24139695	4.92943651	4.31326098	4.97755864	4.73449378	5.15020027	5.04184156	4.1806635	4.75223822	5.33425785
SH3BP5	5.67373469	6.86965971	5.91711544	5.94641805	6.09941078	5.93565384	6.98708001	5.85756936	6.38327591	6.43389343	6.26933371	6.20268146	5.83781968
HACL1	4.37569697	4.83247278	4.22128336	4.76164188	4.82074779	4.76317607	5.51933827	5.23064604	4.53800116	4.10648298	5.11939688	4.42559993	4.79822548
TBC1D5	6.15542019	6.68545618	5.78461375	6.23095005	6.22307176	6.55495398	6.85021722	6.48103957	6.49531658	6.31311871	6.51941999	6.51818444	6.7288861
CAPN7	5.63047843	6.20188336	5.53900451	6.57331612	6.56357632	6.4876922	6.72294005	6.66814317	6.47146415	5.42984876	6.7635065	6.38548019	5.93487382
RFTN1	6.37091131	6.32942478	6.10477019	6.07774729	7.16988004	7.84099501	5.97721372	5.60291205	6.93049341	7.11524685	6.71687557	6.63699736	7.05723292
C3orf20	1.11502607	1.72033892	1.08500927	1.00443212	0.81857168	0.48521022	0.37997196	0.84516458	-0.47262144	2.00587766	-0.47262144	2.24155324	3.51744794
SLC6A6	7.35376347	5.5829552	6.02170005	5.66476394	5.37078279	4.1935169	5.8328933	5.19945058	4.27788011	4.42207343	5.29212384	4.85866887	6.09696237
KCNC3	2.70409275	1.55859796	2.02074191	2.21942144	1.09381656	1.04104664	1.21739559	0.80721488	1.33882044	2.71178138	3.53510997	2.68097476	-0.56685527
NAPSA	0.39541842	-0.01109225	0.23709977	0.14673636	0.0793068	0.72825532	1.19318723	0.69917082	2.71034201	-0.22993143	2.88603364	-0.22993143	-0.22993143
NR1H2	5.9694823	5.93916831	6.11479545	4.429215	5.68220451	5.57215244	5.13734573	5.38862668	5.11881062	6.24665649	5.80730295	5.98175571	4.84155454
LRRC4B	4.28836653	4.00925931	3.59432893	1.00812982	2.25266095	2.75269862	3.20089384	2.84086794	3.1395127	4.34084861	3.41381965	4.36370846	2.71068821
PDLIM4	5.31423744	4.9385103	6.045882	3.94021396	4.84175714	3.90929495	3.94481741	4.33857055	3.95788964	4.7655097	5.26345139	4.47212519	3.66335773
KIF3A	4.69944443	4.95350301	4.87824275	5.12042979	5.45653786	5.4194372	5.22417844	5.37579715	5.35401364	4.95144005	4.99042086	4.97748328	4.85621753
MGAT1	5.95894817	5.9686976	6.24648913	5.77959326	5.26365953	4.79792868	5.28719481	5.58522584	5.28234707	6.15033261	5.91685713	6.07566009	5.14371384
GFPT2	4.11202722	6.75819396	3.80377538	5.45369646	4.16238982	3.51496034	3.94175869	4.20608756	4.2141988	4.23002585	2.8641936	4.8581979	5.07756899
TUBG1	4.53840382	3.65496244	5.50881785	4.67963505	4.35691253	4.15938006	4.3185376	3.89647311	4.22434705	4.82743868	4.4031527	4.2057401	4.52287135
PSME3	6.43444844	5.98813898	6.28584096	6.76204244	6.49074705	6.23156764	6.29215151	5.56971025	6.01104348	6.32519129	5.89146728	6.28783752	6.41337013
RPL27	5.65401966	4.97675128	5.57762148	5.6226946	5.57285381	6.47269891	4.65472943	5.72212996	6.48686731	6.83852928	5.26573647	5.80533923	5.57955736
PSMC3IP	3.48061917	1.81361401	4.30953186	3.46453857	2.43793742	3.18449546	2.86025013	3.00753417	3.67266368	3.84361355	-1.21911655	3.26402662	3.24053538
AOC3	8.4125791	8.03125333	8.33942045	6.94661026	8.36307712	8.10318364	8.42031913	8.43593337	7.4519485	8.42136513	8.11814053	8.56926782	7.46113477
ACLY	6.66222513	6.37973597	6.70704763	7.22050001	6.42923744	6.34181857	6.48002647	6.46448135	6.14331133	6.50139238	6.40159591	6.44034029	6.43201857
VPS25	5.05881306	4.7298407	4.71857019	4.47193874	4.12465638	5.52229435	3.87399056	4.51175533	5.31219113	5.11955066	3.89649279	5.26440143	4.07543527
RAMP2	0.94156399	4.81180005	1.22891604	4.16223822	4.63390581	2.61694006	1.4205448	2.82656203	4.94697491	4.8155381	4.72301481	4.09092805	5.08535757
AOC2	2.84842528	1.58019854	3.13607045	2.26968931	2.57244179	2.59037169	3.13091404	3.53427191	3.23494643	2.87551974	0.68475058	3.31253156	2.31683789
NDUFA2	3.6229501	4.39144515	3.44937401	3.59913248	3.01935295	3.74969761	3.04903947	2.56958728	4.09968487	3.96716456	2.62712968	3.30145952	1.88331104
ANKHD1	3.60063483	2.89265294	3.05503503	3.58896441	3.82605599	3.45334655	3.73179855	4.1642057	3.47447769	2.45078428	2.74248336	2.75945299	2.79352414
DIAPH1	5.97499197	6.50725569	6.80010771	6.45558431	6.67680508	5.72258984	6.12480607	6.22175395	6.42326945	6.16707082	6.20440751	6.10753033	6.68673996

NDFIP1	5.89636482	6.69388998	6.33852908	6.8751495	7.37965679	7.18918165	7.31455722	7.55502326	7.0246287	6.27278148	7.13269846	6.84608769	6.10865087
UBE2D2	4.74861138	5.69835228	5.04115035	5.49334781	5.80748504	5.63837649	5.61814882	5.68876702	5.77853076	5.3333331	5.83885557	5.35052227	5.14537592
EXOC4	5.08184312	4.99791092	4.43633788	5.30039133	4.9255846	5.62061788	4.95270246	5.20545013	5.2276478	5.13151932	5.14975539	4.95349069	5.35433817
ACAP3	2.59693871	3.13710741	4.3494213	2.54596891	2.19149228	1.44450381	3.51905257	2.86669197	3.09254146	2.55485698	4.08686202	3.4620183	1.48970101
C1orf159	3.19241609	2.28339261	3.90674434	2.68487662	2.89849847	2.31471439	2.76387357	3.39697644	2.63232289	3.54668076	3.00944465	4.09514484	2.69725472
ANO1	3.54235376	3.22751812	3.35355477	0.67526524	4.17966014	4.00561145	3.854524	3.93487056	5.14109095	4.7038021	4.54902285	5.29064118	0.28695404
PPFIA1	4.37184131	3.6129269	3.86103627	3.87031566	4.12424487	4.26481544	3.87305042	4.36898595	4.03377529	4.46888156	3.94447381	4.37558798	3.96610043
TMEM204	5.15998063	4.38747687	4.21955613	2.49582736	3.52477965	3.84064444	2.15268905	4.25949554	3.29486486	4.25672731	5.1153727	4.81481758	0.26600932
THOC6	3.60315331	4.17512267	4.71301097	3.42645604	3.89647656	3.38287048	3.36461926	3.85434449	3.48493468	4.10291241	3.93243516	4.81261601	3.38777011
TRAF7	5.22593595	4.6702533	5.55249574	4.89898057	4.41939725	4.0109323	4.88877371	4.57738184	4.30155752	5.25200493	4.78272926	5.16893426	4.20070575
NINJ1	5.84084199	5.70786595	5.3757851	5.16848928	5.10412836	5.20792958	4.29008707	4.53232304	4.56002049	6.13371313	5.13884388	5.45479106	4.76024472
NPHP4	4.34458959	3.88119354	4.54200949	4.18866248	3.4838968	3.21836747	4.00145161	3.95679788	3.92865526	3.96536868	4.46258441	4.29417126	3.43219859
MAP1B	7.0040072	6.59521103	7.13274852	7.063601	8.0070543	8.32313515	7.82864085	7.88707299	7.17778267	8.41622974	7.71017377	7.99654221	7.24672064
IL13RA1	5.74433579	6.47006663	5.47954733	6.45482824	6.74211201	6.07757285	6.31651938	5.82424079	6.55572664	5.35851905	6.04521078	6.01036609	5.40391233
WDR44	1.68740258	2.76032429	1.99492501	2.63709968	3.16679937	3.76546509	3.1455814	3.12312782	4.61014962	-0.63752898	3.07596606	2.937415	3.98336213
CKMT2	5.62626176	5.21952158	5.25403037	4.40226728	4.99079599	6.38297771	5.03563062	3.80415184	4.38832141	5.58380769	4.01069517	4.22768251	6.93561105
ZCCHC9	4.61795893	4.56029641	4.31312168	4.57743315	4.90264411	5.10128	4.81036364	4.91063478	5.33614681	4.76740005	4.52440206	4.69007033	5.07660723
TNS4	0.01604644	0.53840865	0.04284454	-0.04676462	-0.05416593	0.04284454	-0.05416593	-0.05110398	-0.00176525	-0.00176525	-0.00176525	-0.00176525	-0.00176525
TOP2A	2.83671855	2.8240252	5.23034888	6.12747864	2.5180899	2.6517163	3.94128552	2.95681498	4.07462028	1.8433738	3.61217925	3.74830839	4.63279136
STARD3	4.76894606	4.03744478	5.04933757	3.76785436	3.86708217	3.2510353	4.34101562	3.99297015	4.41917324	5.03704991	3.67477906	4.64738207	4.24603313
RARA	5.20301055	4.08622493	5.62580432	4.31335501	2.89375946	2.35900244	2.46195496	2.32078125	2.99343367	4.53058579	3.84635937	4.21656463	-0.77873194
PPP1R1B	0.47561638	0.53787811	0.47820619	0.49203517	0.47561638	0.87708208	0.64971649	0.99845783	-0.3064055	3.71311178	2.27599264	3.6762477	-0.3064055
KHDRBS3	4.32015212	3.93285694	4.3969894	3.63640194	4.76842538	5.25605067	4.1044215	3.89098748	4.05211743	3.80644158	3.79671772	3.85288288	4.40873038
CHD1L	3.62127397	3.34451503	2.42712081	4.0860648	4.15092949	3.93929762	3.23765917	3.55093285	4.23884819	3.92711109	3.65770792	2.98717952	3.96633889
PEX11B	4.01808762	4.72890286	4.19004853	4.55851396	4.63100413	4.62226301	4.86590024	4.22758363	4.29350761	4.32146284	4.37168694	4.44867481	4.76705742
FMO5	2.86774293	2.47568334	2.52838884	2.274757	2.3582442	3.70583452	3.4660047	4.03218269	2.80614741	-1.46770692	3.66990438	3.35453951	3.13469562
PIAS3	5.79823506	4.9965348	5.60767957	5.36957763	5.5383497	4.91779266	5.33898496	5.76383956	5.14367727	5.9528135	5.4898766	5.90242311	5.02570232
PRKAB2	5.53127726	6.1709135	5.50832817	6.19536503	6.64280802	6.422794	6.69558729	6.19415054	5.99359669	6.15197114	5.95146562	6.23638578	6.07277104
PDHA1	6.0869276	5.44404417	5.7225254	5.16245239	5.75151167	5.90620401	5.93372206	5.74325986	5.43074355	5.77701501	5.5331372	5.64561845	6.68444834
RAI2	6.00999144	5.79332186	5.73876844	4.23206969	6.41196702	5.88503611	5.9596943	5.82487202	6.21776891	6.11381527	5.26809564	5.92419253	5.7706769
MCCC2	6.03324562	5.29767038	5.9053935	5.58959949	5.93353652	6.74155531	6.22473148	6.3912591	5.53714773	5.86683594	6.14475944	5.43295283	5.94700811
ZNF304	5.41132566	4.70196742	5.03603685	4.65485886	5.40884154	5.11266567	5.02333656	5.67809084	5.19463988	5.19423192	5.47447762	5.31072461	5.6866569
ZSCAN5A	2.22102639	1.74999402	2.49009707	2.83606012	2.26618893	1.65741148	2.03466592	1.59217793	2.07733175	2.5172309	0.33897001	-0.7181617	3.28155488
ZNF132	4.26366872	3.33002853	3.86475944	2.83702182	4.09357648	3.70226654	4.45410553	4.31797806	4.39780355	3.73042507	3.95779322	3.44590661	4.40454817
CHSY1	7.20156336	6.19481623	7.26577122	7.77812421	6.20428042	5.78686233	6.02404998	5.84832603	5.83565286	5.89516757	5.58521435	5.84717229	6.03651635
SNRPA1	4.3612849	4.59251042	3.66093491	5.09783382	4.49300998	4.3558302	4.63980393	5.18437855	4.90540789	4.71548692	4.53066101	2.73785945	3.55435355
LLGL1	5.06103174	4.67281522	5.72228278	4.15069126	4.47770037	3.50823281	4.29990529	4.27310693	2.50793713	4.9582974	4.53163812	4.98825915	4.08216898
THAP1	4.15905092	3.13711805	4.15091545	4.25760187	4.11663686	5.26222876	4.28960301	4.11118241	5.04234228	4.80891143	4.25945577	3.78760427	4.41028958
RHPN2	1.81756436	2.04766366	1.13036412	1.01436153	2.35499569	2.06955287	2.17303606	2.45857646	0.836031	2.94672702	3.31159121	3.92895911	1.86400489
C19orf12	5.0811716	5.34620713	5.03864612	4.16844862	4.95340479	4.58910052	4.68066852	4.9817499	4.27419295	5.09761712	5.43473692	5.39242661	4.47637332
ACTR10	4.64523577	5.38418601	5.07243279	5.67754867	6.06979869	6.03358699	5.91144034	6.2033366	5.67105818	6.35018747	6.01454206	5.56513583	5.84437913
ABHD12B	0.24607911	1.36077426	0.37187807	0.34160963	0.35631661	0.24746128	0.24607911	0.24925065	0.39305264	-0.07556592	-0.07556592	-0.07556592	2.97646882
GCH1	3.54981888	4.97373681	3.22610506	4.02440086	3.13586264	2.75428115	3.56861382	4.08014831	4.0843521	3.433234	0.9742867	3.4813347	2.88274575
LGALS3	6.09064051	6.13124091	6.11457563	6.13271323	5.97124392	6.00513267	5.42703239	5.3457432	6.32428431	6.57806264	5.5721559	5.96618161	5.96918921
PODNL1	5.01275689	3.66724323	4.87826916	3.61794944	3.22333558	3.07718434	2.32214766	2.26716286	4.876393	1.71722591	4.28217473	4.27514798	3.8453626
DNAJB1	6.54635395	6.23661468	6.65647508	5.88898559	5.91825041	5.99746972	6.2909379	5.17990527	5.6328096	6.48310777	6.05407082	6.43773946	5.86450259
ZSWIM4	4.02317333	4.58817527	3.96688709	3.94194662	3.48519748	3.00404402	2.75259452	4.04169215	2.0459296	2.28848296	3.21163609	3.58734215	2.74527705

FBXW9	1.81739649	1.3924549	3.18413709	2.4435155	1.48964993	1.45513822	1.32083047	2.30584688	2.67559281	3.81625269	2.28515955	1.00853355	2.10856235
RFX1	4.50126878	3.47010136	4.77225833	3.41885706	3.70183316	3.29679557	3.69213291	3.2597219	2.98606625	4.78332848	4.22077399	3.62926978	4.04620973
ZNF20	0.26075573	0.70336273	-0.09201944	0.26330323	0.40960453	-0.09201944	0.18342668	0.15211604	-0.05246847	-0.05246847	-0.05246847	-0.05246847	-0.05246847
C19orf57	1.10147291	-0.21289755	1.98320687	0.31749511	0.99137299	1.22557094	1.01615753	0.68019066	2.43110797	-0.36421748	2.93892911	-0.36421748	0.66988127
DCAF15	4.38403914	3.73685084	4.82214754	3.5864454	3.26662335	3.09777495	3.3566332	3.6042262	2.90887893	5.33528916	3.96117056	3.37028552	3.22793453
CC2D1A	5.70331409	4.81792232	5.87127409	4.34248415	4.96187836	4.9114384	4.72146759	4.97719056	5.05397227	5.6810631	5.08249266	4.90425843	5.09811347
MATN3	1.78015578	0.7938694	1.4497899	2.03932539	2.76452269	3.11472396	1.18181691	2.84625944	2.07727008	2.73357343	2.42031956	1.49633093	-0.65287701
TRIM21	5.87495	5.59974874	5.37944733	5.10978656	5.33826425	5.23183539	4.85105349	4.81607277	5.09972901	4.80374398	5.54224405	4.72772444	5.52856359
SPATA6	1.31195058	2.77514597	0.78457166	2.19189775	2.39811397	2.48118562	2.92944672	2.6603296	2.6846367	3.10602687	2.53395321	2.56561551	-0.40340262
LRRC41	5.6355361	5.25961674	5.72059175	5.69748558	5.13838195	4.65192685	5.45174842	5.2072988	4.60010639	5.48169715	5.92205585	5.69979516	5.09162852
CCT6B	2.83814113	2.13016056	2.79777101	2.09933303	2.62476341	3.11363661	2.58609416	2.29059202	3.3071248	3.1217029	1.44132621	2.22529735	3.2629004
DHX30	5.96995972	5.40785588	5.94897238	5.05956046	5.53262716	5.08962303	5.79103284	5.39650791	5.1578332	5.73868252	5.7659122	5.81552988	5.7291371
RAF1	6.57004388	6.79516813	6.32466861	6.3590249	6.84132749	6.46191719	7.09953243	6.93000514	6.63795208	6.65362763	6.68010916	6.97421548	6.98669675
PPARG	3.91709464	2.29646645	4.09420265	4.34174324	2.92281982	3.32720376	2.78909223	4.27886728	2.92856354	3.97631018	4.26522626	2.90398606	4.13314488
NUP210	4.26942753	4.02481608	4.34320392	4.27721437	3.18286383	2.97533548	3.35163031	2.973271	3.22743134	3.34222579	4.42360148	3.94923951	3.73452538
FCRLA	0.23725096	0.82243999	0.11081492	0.44791913	0.10826259	0.10968165	0.21667599	0.11151877	-0.15291856	-0.15291856	-0.15291856	-0.15291856	-0.15291856
HSD17B7	3.24190964	2.86171795	3.26213816	3.1174873	3.67960984	3.22260036	4.72124062	3.92118785	4.36747204	2.24113855	3.39079357	2.9683444	3.73977758
ENOSF1	5.66171357	5.30078157	5.46976979	5.12939694	5.27981889	4.39462903	5.43900796	5.57424312	5.23521974	5.00839003	4.57252486	4.82242139	5.30596949
EMILIN2	5.09231527	5.09331752	5.77614975	5.48791498	3.72109508	3.43118494	4.00160267	3.79661745	4.15904752	5.26052064	3.16988302	3.10277595	5.54557455
ARFIP2	5.40173008	5.06644766	5.50171241	5.7549822	4.90220547	4.74103652	4.863889	4.85092662	4.89837257	5.27821923	5.3923993	5.02030032	5.03762588
TRIM5	5.59012553	5.95807874	5.21839018	5.81431947	6.36784671	6.15078081	6.5227858	5.66455103	5.83129333	5.60989097	5.91716098	5.54267355	6.07451175
CNGA4	0.95842956	0.45319132	0.83985143	0.55458168	0.84331408	1.04881626	1.16620548	0.45647132	0.70687232	3.40140865	-0.32071485	-0.32071485	1.78481358
TRIM22	6.74458928	7.0056755	6.58382868	7.36695622	6.45693504	6.68962587	7.26405472	6.07702436	6.73533956	5.92168684	6.82622493	6.36313325	6.88618274
RRP8	4.34887788	4.34653114	4.10488744	4.24819769	4.89451114	4.37993197	4.4159696	4.37374651	4.00409053	4.94494103	4.80588947	4.62037909	4.24688713
EFR3A	6.35409997	7.32694078	6.59805258	7.28443249	7.48202584	7.46270922	7.70137947	7.33466596	7.24691761	7.03187426	7.49955903	7.35146142	7.51526302
PTCD3	5.2560794	5.85577455	4.96944751	5.66068782	5.86594688	5.86757292	5.95675471	5.83638471	5.76534883	5.39143985	6.15242161	5.95786846	5.94746696
IMMT	5.85832774	5.69360674	6.0352223	6.53763621	5.93223994	6.36178667	5.98410515	6.02288096	5.79690977	6.01484981	5.84557583	6.18903469	6.79868636
MRPL35	5.0209824	5.43315695	4.52459957	5.18936565	5.40101004	5.77212655	5.46350271	5.05207968	5.16271559	4.57981696	4.948458	4.78327537	5.72226959
IQCA1	1.69658617	2.9730992	2.52793638	2.34487748	1.07336149	2.07226307	1.13275669	2.59035714	3.592532	2.53279141	1.27472741	4.11254501	2.21609111
ILKAP	4.72108528	4.81372261	4.68609338	4.92003579	5.06511241	4.48516911	5.4968094	5.67890538	4.60148931	5.4693382	5.39337004	5.33966193	4.35668808
PER2	6.98849952	6.45871844	5.79541235	4.96436812	6.2608774	5.66592457	5.22337039	6.19820552	5.34708327	6.32513304	5.66385462	6.64061712	6.66152407
RAMP1	5.28020714	5.37435145	5.79994761	3.01177111	5.7916046	6.393888	5.31456723	6.02865941	6.12004561	6.92650965	6.17973099	6.54830398	4.92971508
SCLY	4.65319634	3.75264903	4.16670452	4.06074618	4.00592099	3.67626402	4.40935611	4.2224369	3.71880913	3.77896937	3.80662231	3.99536061	2.56553972
PTPRE	5.24134454	5.28904963	5.56471092	6.16361658	5.25089719	4.3483387	4.7220093	4.5182996	5.0279731	4.4185805	5.26886826	4.31394206	5.14257007
RAN	6.45470299	6.54234987	7.04259754	7.92541113	6.57499124	6.91823938	6.60651379	6.09296359	6.3433688	6.66951071	6.5067244	6.78356539	6.35165859
PRKAA1	6.34961218	6.58968612	6.48882345	7.03045488	7.1901367	7.16767826	7.02226303	7.23352596	6.91002117	6.61457389	6.92766381	6.61033961	6.17878813
CARD6	6.07186531	5.79391369	5.93462394	6.05466888	5.24441193	4.22276826	4.69689948	4.51705475	5.2371649	4.12525388	5.0526379	3.93320929	5.47747551
RAP1GAP2	2.8892532	2.04677291	3.25862333	1.70660485	2.24507325	1.19791498	1.82863324	3.33891232	2.5821169	2.98565483	-0.57382346	3.18344109	3.21956274
INPP5K	5.06188145	4.97134394	4.90153429	4.13041856	5.14700374	4.7109229	4.91353816	5.49955216	4.60040809	5.12536102	5.34340983	5.68330769	4.97287735
MYBBP1A	5.22387155	5.02101701	5.96544162	5.51928543	4.59864252	4.19747868	4.5967481	4.55026669	4.8966858	5.05972086	5.01132238	5.17196122	4.56295239
RPA1	4.98827214	4.86262405	4.81867659	4.23044617	4.35822609	4.56197691	4.63820162	4.20726808	4.1241241	4.2389286	3.89931558	4.54906409	3.97104235
SERPINF1	4.01766499	7.63574642	1.11585319	7.41288447	5.39896758	3.84610762	5.70833608	5.46674705	6.43641994	6.96403416	6.9724951	6.6115286	7.73940566
UBE2G1	5.04694097	5.37993962	5.02469993	5.86602304	5.99512173	6.12716359	5.91209403	5.88058905	5.76132239	5.62133795	5.85026682	6.10029265	5.55161023
EEFSEC	4.99306905	4.44909879	5.01566477	4.01239125	3.61665003	4.0015501	3.81116244	4.42220096	4.2921122	4.75210859	4.62010804	4.25068002	3.71327714
TBC1D14	6.18127331	6.59677386	6.09035987	6.02760529	5.64990585	5.64163611	5.86060124	5.49133402	5.79255625	6.39853201	5.89856602	6.2571926	6.22241725
TMEM128	4.54621474	5.01492768	3.62051911	4.78079185	4.9627108	5.03915693	4.74320272	5.01302059	5.08645114	5.18895045	5.1358353	4.89827858	4.42044903
COQ3	1.61688379	3.94574888	3.29761596	2.36987007	3.32867344	3.35237914	3.21527498	3.09509215	2.74747993	1.92978531	2.309422	3.70831469	3.74447489

PNISR	6.56890481	6.65490357	6.05991963	6.99146597	7.06424587	7.27652888	7.3576484	7.17612886	7.34857145	6.87179459	6.83724021	6.67702704	7.06660918
POPDC3	0.47080057	0.47080057	0.69143706	1.37218463	0.59567346	0.47223454	0.71451572	0.67571945	1.17382157	-0.58752899	-0.58752899	-0.58752899	4.2592548
SEC61G	4.07591288	4.32135222	5.01111726	6.17581081	5.24119112	5.71172973	5.51158831	5.044623	5.59564466	5.06631216	4.41602997	4.54020392	5.05495023
LANCL2	4.71793593	4.56215063	4.36979253	4.34367684	3.91396033	3.83626132	4.11546739	3.80879999	4.00460175	2.70309576	4.36804934	4.11826768	3.90656419
FIGNL1	3.11406866	3.03576549	3.76543651	5.07137312	3.57247936	4.47578219	3.83212396	3.76163226	3.55397425	3.82938031	4.24415858	2.99709076	4.1350907
ENAM	1.2416689	1.10966331	1.11218983	1.12589845	1.22393563	2.63724521	1.15511688	1.30290084	0.53192159	3.68874653	-0.65409075	-0.65409075	3.44959589
ANKRD17	7.12503236	7.05371478	6.54617993	7.16479884	7.13838479	7.15971053	6.88121533	6.87684792	7.00861617	6.97120224	6.8736893	6.79390943	7.11723289
UTP3	5.23461696	5.47502455	6.12119664	5.68356967	5.62943773	6.26540115	4.81412172	5.76688364	5.70063095	5.865755	4.96665893	5.36402985	5.52402448
ITGB4	2.03468309	5.72107769	2.61696252	2.66710585	4.6032822	2.57851307	3.83414967	4.33404054	4.39499419	5.4764848	5.97047421	4.4969907	5.70522251
WBP2	6.53350266	5.35136616	6.18499297	4.75449432	5.7143389	5.49132821	5.39277267	5.59032122	5.47391624	6.37048044	5.69442848	5.72401766	5.07888018
H3F3B	8.09364009	7.73057338	8.29408855	8.05291617	7.4144477	8.16551295	7.06942587	7.39716035	7.61592815	7.12289295	7.62534636	7.32962878	6.60201943
UNK	5.30865517	4.80079264	5.30658316	4.63292329	5.34294961	4.69160815	4.72052024	4.93605102	4.14689088	4.94392617	5.24537601	4.75516384	4.84678249
TRIM47	5.19002716	5.40142127	5.98686861	4.04110712	4.10652902	4.54390157	4.45848745	4.22610619	4.99313528	5.80305549	5.3296543	4.76060543	4.88919463
ZRANB2	5.04789113	5.54921678	4.94759076	6.43999429	6.32303935	6.52315461	6.35486333	6.47636332	6.47750654	4.8647698	6.22658566	6.46562667	4.58601548
EIF5A	6.30134558	6.76400693	7.09632929	7.69449371	5.61587199	5.45732108	6.17537355	5.76718591	5.79257761	6.731754	6.22942173	6.39085989	5.88393229
KDM6B	6.48596937	5.27252282	6.05938196	5.03031958	5.11410841	4.00819854	4.73583329	4.67583545	4.68030693	4.28760265	4.98084495	4.24885967	4.68760383
CLEC10A	3.59187011	4.98490228	2.17534545	4.30700943	1.45713413	1.57868635	2.70163886	1.75394122	4.05150085	4.69007113	3.86479918	4.1122786	4.04867089
GPS2	4.26224762	4.94255613	4.62100996	4.23525397	4.3508296	4.56766471	4.94806332	4.68856179	4.81438519	5.29536687	4.40597443	4.61654783	4.33937627
XAF1	4.94174172	5.03797949	6.03816604	6.5878611	5.47770494	4.05178801	5.28990855	4.67225498	6.22577841	4.62403719	5.46122135	4.15350047	6.47110938
DLG4	5.74574497	4.19462083	5.57385931	4.5917714	4.36903337	3.67739971	5.05934345	4.23872101	3.95348072	4.14710261	5.11926393	3.73394931	4.42981223
VPS13B	5.40018347	6.08948317	5.13298855	6.25619951	6.29836129	6.1525945	6.24716837	6.3539368	6.32826326	5.54993926	6.62270894	6.40816378	5.51685107
RGS22	1.65254539	2.40530047	1.71513875	2.50729247	2.99842711	3.98992559	2.63507423	3.96551095	3.3062288	3.14886482	2.78546163	0.49652061	3.6224829
MATN2	3.55834521	3.50463784	3.82174003	2.15073042	3.5896357	3.78037201	4.52680548	6.1235855	5.7812371	4.59661276	4.62597195	5.93094178	5.09387173
REEP2	4.47671599	3.78315452	3.75806648	3.07243346	3.82411274	3.48425349	3.42141338	4.53611147	3.78530278	3.23949883	4.37010219	4.47987946	3.29403007
PCBD2	2.05415411	2.12205059	2.42443986	2.11052689	2.64466933	1.8727044	2.65299148	2.9697644	2.50189987	3.45336758	3.39104047	2.8834616	1.10502704
SDF2	4.92847797	5.29848321	4.87364148	5.31631386	4.91981568	5.476845	4.64228304	4.60399669	5.14960787	5.26099792	5.0309271	5.65749433	4.98464606
FLOT2	7.76359326	6.771758	7.26151615	6.15371775	6.49463441	5.87593198	6.55398707	6.29182273	6.0131223	6.48878382	6.27734858	6.43740008	6.24462535
ERAL1	4.79216516	5.15939319	5.59854571	5.04239256	4.51380189	4.38592672	4.60146426	4.13129956	4.53718582	5.3972292	4.0597938	5.19346588	5.24602556
PRMT7	4.88415602	4.0158323	4.64917815	3.82417749	4.31716531	3.69533878	4.54639436	4.47322682	4.15410399	5.31761154	4.65614651	4.5082359	4.44168393
NIP7	4.32830459	3.72337988	4.91196359	5.12612167	4.41017253	4.2556189	4.44915083	3.90523053	3.97346633	4.29685301	4.50197606	4.63428417	3.64130431
TERF2	6.14529966	5.88755351	5.89473051	5.46527607	5.8951821	5.20341208	5.75066283	5.42332591	5.32512309	5.43868885	5.3069946	5.98446392	5.6855182
VPS4A	5.59466754	5.5353203	5.46380464	4.81787531	5.10600231	5.1907849	4.73694883	5.27485398	5.31372736	5.48996698	5.24143546	5.36174959	4.72484362
MTSS1L	6.67680951	5.91439217	6.74978069	4.73917251	5.97925896	5.86875797	6.35205669	5.90205002	5.47856457	6.83964657	5.99028878	6.36795698	5.96959352
HSPA12B	0.40244534	2.32933877	1.31695275	2.89096641	3.24957796	1.80208281	1.50013828	2.28880899	3.65417589	4.86745772	5.00475975	4.49916023	4.48409913
SNAP25	2.92883437	3.52531977	2.64001576	2.53008552	6.50794511	4.69934232	4.48461803	4.03277085	3.32104175	-1.62157204	5.84655308	3.51747301	3.21069672
BTBD3	4.89686352	5.48837051	4.67428143	5.28714438	5.23259833	5.43558147	5.25528799	5.51800274	5.00208112	5.39244563	5.27112476	4.96857112	5.37651698
PCNA	5.03229084	4.83409081	6.30783684	6.80789238	4.74548027	5.48558642	5.74452434	5.010827	5.50769053	5.5707	5.32193521	5.38801834	5.89811735
NXT1	4.86789677	3.24830024	4.80965313	4.62988757	3.8094817	4.2288117	3.46607036	3.60594261	4.44912002	4.2786154	4.15000935	3.12208596	2.88554267
POLR3F	2.13163847	2.49970643	1.1891492	2.37656781	2.95178848	3.25400178	2.89799837	2.81339091	3.04993476	3.82859467	-0.67446222	2.82488869	3.04389566
RIN2	4.50354133	4.35100681	4.44235577	4.31609038	3.17131537	3.88479607	3.14886117	3.83025352	4.5535482	4.21135834	4.29530436	1.36405762	3.43465273
PTPRA	6.84395367	6.5351932	6.24614048	6.41720551	6.78359002	6.61074847	6.6375032	6.95865968	6.81941733	6.9820723	6.48131389	6.5581719	7.08547104
DAP3	5.29968343	5.88572469	5.63610904	5.76507152	4.91068293	5.37632883	5.39198919	5.20187881	5.69082985	5.4039573	5.35064029	5.03773312	5.96329798
ATP1A4	0.32565543	0.21642119	0.04984096	0.03689467	0.26040394	0.64044267	1.17244997	1.00520799	-0.30193139	-0.30193139	-0.30193139	-0.30193139	-0.30193139
NES	5.01960777	5.77024537	6.14009938	7.26483801	6.47890216	5.64701826	5.34007748	5.80550589	5.49276825	6.62518185	5.53047527	5.66145263	8.05121386
BCAN	0.29744778	0.01926405	0.2893987	-0.05491635	0.13875431	0.60610546	0.212685	0.0438314	2.92807222	-0.11400233	-0.11400233	1.43512414	-0.11400233
ARHGEF11	5.03705686	4.13243069	4.84607567	3.73025833	3.87195031	3.51024953	4.35231423	4.24264008	4.73386519	5.36289921	3.69873028	4.75367699	3.38819538
FCRL2	0.1505707	0.42327245	0.13801942	0.13544511	0.13544511	0.30228543	0.13544511	0.13872933	0.042153	0.042153	0.042153	0.042153	0.042153

DCAF8	6.54654888	6.81216966	6.26129883	6.4747539	6.84185924	6.5107589	7.0397997	7.21690868	6.49262645	7.18440425	6.70558501	6.99575217	7.10184021
SYT11	7.05000343	6.37116582	6.68799895	6.46226824	7.24331952	7.37514261	7.06087189	7.32430734	6.67141459	7.35214485	6.94958287	7.18430896	7.09513873
IGHMBP2	4.85530651	4.2247083	4.85212663	3.46577621	5.06386646	4.2613663	5.0761934	5.45052493	4.99222867	4.64690752	4.16910944	4.51701353	4.78939962
MMACHC	3.71219263	2.42313836	4.06080456	3.3404177	3.10819877	3.78932359	3.26763271	2.54965837	2.48101335	2.93539365	2.4083137	3.3687919	4.10703886
DPH2	4.18619074	3.94158753	5.01158552	4.27190145	3.37885922	3.37885922	3.58920341	2.97037535	3.94999865	4.00178482	4.29248654	3.77945416	0.55755658
TOE1	4.68754325	4.04578695	4.31882124	4.32324051	4.30680427	3.63158863	3.38849475	3.44965357	3.63614442	3.30852781	2.70186793	4.25364836	3.92726491
NASP	5.68362408	5.02271144	6.2597629	6.46725694	5.70095181	5.39811425	6.19544133	5.99944912	5.81309273	6.0579993	5.40606215	4.95391182	6.29023038
MUTYH	4.63599821	4.43911058	4.75447461	3.65049321	4.43141498	3.72161806	4.0775325	4.30712953	4.68697761	4.75814004	4.358167	4.01315974	4.76386892
CTNNBL1	5.48417545	5.42443996	5.92643422	5.43764621	4.93598334	5.40415902	5.14623229	4.86111522	4.76675841	5.71114759	5.17405436	5.29644905	5.10853837
LPIN3	5.01166214	4.92443756	5.22877991	3.58331411	5.26193914	4.43402777	5.18065732	5.02867091	4.86135806	4.98052374	5.64837566	5.21186613	5.25406861
ZSWIM3	3.97403961	3.00165731	3.03468402	3.03209325	2.99465134	3.44859864	2.66506385	3.11954902	3.40417706	3.65877859	3.02089092	4.09185055	0.89734694
RBM38	4.94007515	3.916676	4.72281716	3.384382	3.99099401	4.75259113	3.71094791	3.86153454	4.52833954	5.44917807	5.27532756	4.29157775	4.33491066
VSTM2L	-0.04483689	0.29416175	0.40501559	0.74098807	0.01343372	-0.04345517	0.34315925	-0.0416664	-0.02945243	-0.02945243	-0.02945243	-0.02945243	3.43235701
SERINC3	7.08841196	7.12904206	6.54835676	6.83444017	7.37096879	7.48885211	7.36890788	7.3199828	7.44768166	7.31484794	7.35424239	7.20079448	6.90016927
PPP1R3D	4.10094497	3.80010773	3.90281368	3.27004805	4.02032823	4.64720639	4.41307145	4.44239339	4.59043846	4.09006172	4.29918828	4.43737115	3.09611465
DMGDH	3.43783779	2.9203377	2.75360386	2.85929425	3.81231498	4.17200952	3.24724596	3.25972195	3.65495727	4.29418228	3.93131476	3.01618106	2.5158005
BHMT2	5.22181266	5.05823101	4.94614908	4.03829504	5.62773934	5.02431472	5.53609678	5.16159185	5.20019528	5.10649111	4.93722515	5.37884541	4.88848825
AP3B1	3.40054386	4.11875103	3.94425066	5.28037214	4.25019733	4.67836917	4.27603573	4.64892692	4.67822372	4.11941819	4.05515789	4.99537377	4.67896906
ZBED3	4.40081848	2.6455257	3.06747582	2.47394644	3.67740438	4.07136392	2.92548037	3.73627129	3.66064251	4.96679693	2.93589586	4.37089436	3.09718419
KANK4	0.81084017	1.15244656	0.81330839	3.22387072	1.03382249	2.56953853	1.28579138	1.37131706	1.8140021	4.04981568	2.715484	3.96356711	3.72900774
SYT4	-0.01639862	4.05976682	-0.02446658	0.60996446	-0.02595352	-0.0251268	0.06849557	0.11510032	3.29290307	-0.15213686	3.03238109	-0.15213686	-0.15213686
SLC14A2	0.2209232	0.09379162	0.24913317	0.16377466	0.1279908	0.23116091	0.0272512	0.06544745	0	0	0	0	0
FBXO44	5.07418415	3.7851358	4.67038862	3.09474176	4.33980663	3.60861644	4.16583011	4.12869835	3.99150476	4.97811543	3.30627163	4.00561981	3.10073588
RSG1	3.88161331	3.05955303	4.47235429	1.42275172	2.55905832	3.02496599	3.00927944	2.28464913	2.11221893	2.24974529	3.36188585	3.45350102	3.15589669
CASP9	4.6640753	4.14307874	4.3344385	3.21377232	3.78800371	3.5005776	4.34687844	3.77389106	3.93631273	2.73098064	3.16953706	4.20355308	4.76469515
DCTN4	6.34140748	6.31693377	5.8524584	6.73751145	6.45614755	6.71660035	6.50219279	6.3795312	6.19476472	6.27453516	6.66966068	6.68368721	6.32479435
PDE6A	0.59358936	0.59358936	0.68218068	0.59765479	0.70958707	0.5950379	0.74167257	0.59691318	2.9751117	-0.09210631	3.52584441	3.08562578	-0.09210631
ATP8A2	0.26497	-0.01366384	1.25243446	0.31173292	0.35633509	1.00173175	0.15714717	1.46828284	1.05373051	2.77955444	0.01821961	0.01821961	0.01821961
MTUS2	6.38636145	6.41279922	5.98733615	4.1797218	6.25663363	6.64561767	6.50906759	7.25158451	6.80688413	7.12519915	7.22611598	7.2329862	6.7890874
ZMYM5	4.54885049	4.50619138	4.46610725	5.45023956	5.19985483	5.25940624	5.34722116	5.28650987	5.62448875	4.07036176	5.07425168	5.20468387	4.859879
USPL1	4.65807137	5.5619343	4.4882279	5.81629495	5.70262545	5.79974069	5.24296938	5.48437124	5.42922162	3.83864232	5.54399479	5.24270869	4.904992
XPO4	5.03414779	5.64994448	5.0758771	6.04687366	5.63413765	5.86617837	5.69536781	5.74323659	5.56416225	5.2138809	6.23022832	5.80969633	5.42612027
TPTE2	0.22965586	0.0469269	0.20822793	0.43771083	1.53945553	0.62568262	0.43066306	1.22368574	2.62246159	-0.18272195	-0.18272195	-0.18272195	-0.18272195
POMP	4.31473504	5.89513103	5.78240817	6.77660037	5.84118669	6.007789	5.99462456	5.77180721	5.90630356	5.58859136	5.48442006	5.00705786	5.66438069
CDK8	4.23307267	4.75299661	4.55312113	4.74323962	5.19127628	5.21436394	4.8343053	4.8283363	4.32318609	4.68185007	5.0634214	5.08487738	4.79842597
ALOX5AP	5.41935248	4.66259833	3.63903319	4.92999499	3.99737786	4.12759553	4.11313648	2.50073258	4.47900409	4.2719944	2.8482952	3.30555578	3.89393682
WASF3	1.59609434	1.13711691	0.72899018	-0.6865848	1.57505011	1.80062997	1.49168925	2.37013532	0.4462886	0.4462886	0.4462886	2.22460295	0.4462886
GPR12	0.00778729	0.00778729	0.01036825	0.00778729	0.23093069	0.00922227	0.14523798	0.01107999	-0.07637104	-0.07637104	-0.07637104	-0.07637104	-0.07637104
CHRM3	0.05054766	0.96755334	0.06109402	0.24505694	0.48331058	0.35393519	0.54729787	2.75826712	3.34235842	0.02195851	1.48345757	3.5566798	1.13551343
MYH10	8.45931485	10.6855645	9.01610102	7.57014887	10.5087737	10.1691199	9.78513975	9.82873353	10.3090003	10.5726225	10.4818286	10.4235436	10.3090003
PEMT	4.4696346	3.39298748	5.00298768	3.96219301	3.49569987	3.42912831	3.56665799	3.59845526	4.12229468	4.30977383	3.975649	4.09455989	3.47528282
SCO1	4.63043521	4.72190064	4.59093848	3.96301573	4.69992694	5.0313216	4.84237955	4.81975228	4.4927058	5.07073688	4.65854389	4.81594633	4.33466915
MPRIP	7.88001088	7.66099624	7.7537722	6.95226031	7.73601863	7.39845735	7.73749309	7.88592445	7.16519104	7.52772832	7.7794178	7.53715375	7.5632308
CHI3L1	4.20021936	4.03120449	0.69373192	3.66363004	-0.64838123	-0.18633074	0.9815881	1.13730747	-0.7753451	1.83510741	4.01952397	-0.7753451	3.45371304
PIK3C2B	5.87865987	5.60784919	5.75589457	4.65310317	5.7074083	5.43594801	6.33363059	6.29714544	5.7720078	6.76651577	6.25251235	6.05996689	5.86785116
DSTYK	6.10918781	5.99852179	6.13868417	5.49308707	5.98548328	5.68768723	6.15178419	6.13400785	5.68641274	5.93929409	6.03685071	6.06516438	6.28296869
CHIT1	2.65398207	0.21787475	0.08400802	0.1805824	-0.03946064	-0.12204506	0.00379784	-0.03607096	0.01723042	0.01723042	0.01723042	0.01723042	0.01723042

SLC41A1	6.98332168	5.87671344	6.71932573	5.94031621	6.1685307	6.75013757	6.59701691	6.1685307	6.31862504	6.96588425	6.08680492	6.16947652	7.15702355
LGR6	4.60161681	4.39779182	5.09937401	2.44414889	4.72030117	5.36796159	5.35969324	5.58560612	4.54326579	5.80489463	5.45555453	5.76535381	4.52340959
TMCC2	1.19887681	2.02609548	2.06852672	-0.10336551	1.60751712	1.31220182	1.94411437	2.04855086	-0.30300856	3.54700492	-0.30300856	-0.30300856	1.81727002
DCLK1	1.91152911	4.47724253	1.83994551	5.37316523	3.62218871	3.78501537	3.6309851	4.40230335	4.24377905	4.36866508	4.86850345	5.20068778	5.48459849
CCNA1	1.48418521	0.87266799	1.04447065	1.76178722	1.6208439	2.36133612	1.35724411	1.42719873	2.58280197	2.96133935	2.31892285	-0.6487577	-0.6487577
COG6	3.24394068	4.21702669	3.11829475	4.85136016	4.22855977	4.98290308	4.56179115	4.50171294	4.40889977	5.04225122	4.96923894	4.84876111	3.8040673
RXFP2	0	0	0.00124472	0	0	0.36625951	0	0.00158797	0	0	0	0	0
EPSTI1	3.00385775	4.26989384	2.80265184	5.38283693	3.33593338	3.07599965	2.7723878	2.92208524	4.07492475	1.45959325	3.84078328	2.88765492	4.98374553
TRPC4	4.56497978	4.16708087	2.71717204	2.46513497	3.77169333	3.38782452	3.46426784	4.00001608	2.35930621	0.79225059	2.55127922	3.31710358	3.80269508
POSTN	5.98202291	7.04861994	6.21901342	10.1811205	7.89842171	5.10478457	6.97885227	6.99467871	8.4870002	6.08963044	7.84870145	6.65889843	7.51760215
RFXAP	2.60405477	3.39794664	2.83901269	2.91549303	3.75960122	4.1543756	3.99540755	4.7772029	3.98677492	-1.42628941	4.10313734	3.32828467	2.91420015
TPT1	8.75641661	9.36780082	8.29952737	9.23369295	9.09887445	9.05291625	8.68516771	8.82307863	9.27474231	9.13899352	8.8460912	8.99937917	8.99937917
KL	2.31937259	2.57393276	2.25621343	2.46097683	3.39131422	3.04875928	3.64671998	3.11546682	1.86118675	2.71422859	3.60620379	4.29341258	3.45648685
RFC3	1.82583379	2.48007232	2.59879581	4.37491158	2.12345441	2.39852819	1.257059	2.13607668	3.36145006	-0.75189239	1.7391878	3.09609636	2.15018802
STARD13	6.06179709	6.06889414	5.84698216	6.03910565	6.4876373	5.94923862	5.81153592	6.66574179	5.96634072	6.59266982	6.86345015	6.50567635	6.23393973
MORC4	4.75786686	5.66302817	5.04123541	5.86692584	5.73496744	5.26550419	5.36857719	5.72638203	5.6620378	5.59495962	5.80218387	5.91197534	5.37653387
BEX2	3.57866286	2.94467923	1.27905668	1.49408244	1.33680367	3.9635492	1.85882607	1.54305404	3.96409113	3.23720624	1.01564758	3.29026064	2.62698536
RNF128	0.08200193	0.26560981	0.08459788	0.28266601	0.08200193	0.08344524	0.08200193	0.08531375	-0.14587469	-0.14587469	3.23519103	-0.14587469	-0.14587469
TBC1D8B	3.99214169	4.66383959	4.8362086	6.17612583	5.23803287	5.18939018	5.04239698	5.02970445	5.44194915	4.42737085	5.9482155	5.03287515	3.62682387
TCEAL4	5.87368506	5.99040867	5.85766165	4.7473086	6.34955293	7.38136786	6.10715262	6.73727707	6.5988633	6.77228051	4.75824936	5.4065301	6.98798213
BEX1	0.0307867	-0.52791519	0.85114924	0.56238058	0.38553633	0.05286632	0.23696649	-0.27014435	0.2879178	-0.21019749	-0.21019749	1.76227139	0.91467867
FAM104A	5.24406112	4.98150784	4.80284984	5.5474184	5.45917253	6.11405715	5.36023485	5.7138773	5.37707851	5.20586687	5.52545592	5.23135481	5.14884659
SLC39A11	4.9147579	4.56051929	4.82230928	4.81652062	3.70381087	4.06732539	4.86571422	4.10005902	3.3602096	4.53608444	4.1631357	4.6387877	4.10308433
EPHB2	0.98943802	0.61146963	1.36890592	1.63565332	0.40756026	0.69965987	0.83896058	0.95746668	0	0	0	0	0
SRRM1	6.8177185	6.00875631	6.98879182	6.79499882	6.91482461	6.67123033	6.6491908	6.61736077	6.68462564	6.64102109	6.44357981	6.35387183	7.00956189
BTBD2	6.09128863	6.21177327	5.88826273	4.83772629	5.24605385	4.64545968	5.37182381	5.07235727	4.96401194	6.14687826	5.41886557	6.05888956	4.52917265
PRAM1	1.52340202	1.58414322	2.96740989	2.14478013	-0.01810912	-0.13594336	0.20521431	1.00236427	2.77148781	3.1714752	1.89877261	-0.55590614	1.36134224
ZNF414	3.72353871	2.7611659	4.62865914	1.69695254	2.48166721	2.26289381	1.99158105	2.57743989	0.98949247	3.10226812	2.79267926	4.03204926	-0.96072013
PDE6B	0.65222893	0.52896794	0.96786058	0.58328308	0.36865747	0.66125342	0.36662341	0.69377151	0	0	0	0	0
HSPBP1	4.17700902	3.71547832	5.29068259	3.35635451	2.79154969	3.33094495	3.05466604	2.76898634	3.99627693	4.65422568	4.37417547	3.63516205	3.09478216
CSNK1G2	1.8400564	1.87719703	2.34120363	1.93900241	1.0877363	0.34604061	0.81702924	1.01815932	0.82779838	0.36643702	1.66882631	1.90322277	0.36643702
CNDP2	5.88125958	5.69017337	6.06920968	5.96256543	5.17435047	4.97947575	5.30133441	4.90234096	5.68838789	5.34896847	4.92347682	5.03192428	5.85096617
MACROD1	4.14879795	3.5043421	3.80570893	1.97159878	2.62702742	2.93521023	3.62612319	2.0679524	3.42598422	4.54269965	0.83697883	3.84930306	1.05519688
WDR74	5.31128776	4.39257202	5.17952856	3.99566112	4.40790894	4.45774862	4.23149748	4.13227745	4.1821278	4.65386467	4.07878733	4.53356135	4.15562472
LGALS12	-0.01812317	0.83449552	0.66453049	1.17368711	-0.13049181	0.68519835	0.0989788	-0.31551862	-0.28812418	-0.28812418	-0.28812418	2.92985308	-0.28812418
RTN3	5.45668081	6.1436538	5.51688277	6.04589784	6.0484341	6.39522175	6.25043372	6.27027115	5.79285515	6.19123919	5.94093326	6.24301794	5.84885781
RARRES3	4.28651907	5.44218711	3.31190322	4.13835882	3.13900071	2.96077463	2.86151325	1.71186302	4.01216194	3.6971863	3.84163257	4.22185063	3.62707892
MYH11	10.4426511	10.8620282	10.8620282	8.53284383	10.828714	11.2474463	10.5151198	10.4426511	10.7128931	11.9204136	10.5767324	10.7128931	10.2101988
FOPNL	5.34867143	4.74769327	5.61023082	5.73430607	5.768575	5.99404285	5.95747168	5.48145235	5.79910872	5.23764505	5.31966932	5.45442776	4.92862924
MED10	5.27281845	5.48051678	4.63501096	4.38500702	4.97807042	5.50571625	4.76734408	5.44822405	4.93957006	5.20459196	4.94239703	4.90619511	4.32874097
PDZD2	1.52507313	3.4059933	1.08136168	0.48277911	2.55623792	3.26852733	2.98043001	4.15812382	-0.46198906	4.82906429	-0.46198906	2.60481832	4.52680217
MORC2	5.90439042	5.19874725	5.86207165	5.50448972	5.95886295	5.77037089	6.14026139	6.11906638	5.58109862	6.28198293	5.48581333	5.83373688	6.18920801
GSTT2B	4.81036384	2.94585385	3.97659924	3.52994312	1.46855741	2.96960744	3.20569006	3.3792797	3.84101854	4.30975541	3.54231917	2.69630708	2.163134
MYO18B	5.61264252	4.63955972	5.86964046	4.7595023	6.69535644	5.78639029	5.00484815	6.85653265	4.90243777	6.51324015	5.47202624	5.81020052	6.7807839
SLC2A11	3.76412445	3.31474749	4.10344662	2.789125	2.98514415	3.3524657	3.01317761	3.38373098	3.42379316	3.6926161	3.83029842	2.45441027	3.0634112
C1QTNF6	3.47230358	2.37491321	3.42636992	4.77816831	2.56976257	2.2729648	2.72699716	2.6320828	3.54710232	2.37608801	3.53371198	2.82517724	2.79925729
FAM83F	0.00247789	0.08963592	0.05791009	-0.01378918	0.00744706	-0.01383639	0.06332721	0.10237384	-0.11099788	-0.11099788	-0.11099788	-0.11099788	-0.11099788

GIMAP6	3.72350001	5.47933547	4.60367775	5.14255941	4.58136941	2.68504987	3.80499618	3.22255563	4.9587833	3.68883589	4.89194038	4.06149032	5.62248428
GIMAP4	4.55813824	6.06864899	3.63912539	6.70608014	5.64329604	4.48378408	5.09164918	4.33069741	6.21363377	5.50172277	5.85178365	4.26074659	6.22579334
ADCK2	4.17836415	3.79792921	4.8628814	3.18824687	3.22351201	3.27218181	3.61834108	4.09340032	3.43795844	4.98023581	3.17163995	4.45891972	2.34741515
MKRN1	6.53596295	6.34129016	6.40359414	6.12265017	5.9465261	6.24455519	6.20231941	6.23682825	6.04301128	6.2218845	6.2218845	6.28969956	6.30980514
AGAP3	5.34384832	5.21268529	5.6518779	4.84250488	4.75323872	4.04285632	5.28631704	4.86659159	4.36653036	5.51213274	5.26244304	5.56577719	4.59790289
KRBA1	4.4463369	3.75918724	4.83165559	3.10448205	3.86613658	3.48123137	4.06591739	4.16343921	3.6528408	4.34407859	4.03747951	4.2822551	4.41862272
ACTR3B	4.23562613	3.62368894	3.56091652	3.69993966	3.34860917	3.65710973	3.64207878	3.66373716	3.53137848	3.60098525	3.61424697	3.22787396	3.0857349
BTG1	5.88996083	5.78063024	5.49431335	7.14537839	5.81842001	5.64127599	5.72654895	5.86622045	5.5324577	4.58424475	6.06696352	5.34845204	5.44003215
C12orf29	4.13280784	5.17065287	4.49376615	4.98958077	5.52526042	5.86184991	5.66791348	5.44974645	5.35067107	4.14681995	5.16481017	5.13952771	3.52147265
ATP13A3	6.19258713	7.44713321	6.80510582	8.20693911	7.33290322	6.97880627	7.31824216	7.13429107	6.75568219	5.91021063	7.36817573	6.90299181	6.73809692
SFTPD	1.38202222	0.41948889	1.05386107	0.46596341	0.97837854	1.49687134	1.37960803	0.91973851	2.57530921	-0.36328003	1.98209389	2.44002211	-0.36328003
TMTC1	6.50284851	6.95705552	5.8459784	5.52794731	6.79926892	6.99900897	6.87838204	6.2990573	7.05427282	5.845849	7.1404484	6.09414179	6.93092744
KRAS	5.04428783	5.80099338	5.08044947	6.19227496	6.08686471	6.15898768	6.26218296	6.32472507	6.20836701	4.87533352	5.86480314	6.07612658	5.10499375
IPO8	5.87321744	6.44716872	6.00153327	6.66710618	6.7439191	6.81431005	6.84783357	6.48006722	6.49465878	5.81980707	6.62795153	6.57405712	6.39708301
LARS	5.38148218	5.93775947	5.68337746	6.43465106	6.10286568	6.29258747	6.36682678	6.11209216	6.08991879	5.7410041	5.89368913	6.03828063	6.00288237
IMPA1	4.14987969	3.98639015	4.39039171	5.02566956	4.81298327	5.16805661	4.79224346	5.1322527	5.27837348	3.95893442	5.02567508	5.12913765	3.83458782
LRRCC1	3.67309255	4.2774521	3.92889973	4.49133273	4.85023517	5.85833821	4.77806245	4.76922786	5.83996514	4.45761983	4.58724393	4.49133817	2.91896739
E2F5	2.30247466	2.20605492	2.33364208	3.10240514	2.78064521	3.34417629	2.85544967	3.20730062	3.50888406	-1.52927321	3.70587826	2.36245216	1.57472114
CA1	0.1724524	0	0.1724524	0.00821385	0.37150391	0.00069205	0.02283083	0.00158797	0	0	0	0	0
CCDC59	4.37629042	4.86483419	4.20405216	4.44822269	4.90321739	5.81604647	4.67800306	4.92344517	5.63477072	5.33127491	3.78000373	4.19637498	5.53060969
SWAP70	4.12443033	4.4282003	4.1314747	4.37196001	5.12675414	4.87487634	5.19361074	4.94408209	4.30446093	3.9394911	4.71319532	4.77119883	4.37913071
ARNTL	3.7304222	2.42220792	4.30781154	4.94040054	5.02105947	4.87728965	4.20421174	5.56598308	3.75972689	3.53340114	3.0855939	2.69412515	3.12436436
LYVE1	4.54465572	6.73567503	5.33490408	6.70168683	5.80517093	6.31463838	5.87258002	4.9281029	5.41903491	4.9494323	5.27461375	5.15474843	6.32530629
AMPD3	4.9921404	4.75346316	5.00113137	5.46343988	4.18421018	3.43074416	3.69199554	3.65496873	4.77718174	3.78690292	4.50096454	4.43934121	4.17264466
MICALCL	5.53979941	3.96230838	6.07200738	4.64705039	5.19153446	4.26833237	3.92985901	5.41348077	3.36604515	3.36502977	4.86226773	4.12993803	4.25608527
SBF2	5.59824629	6.00065564	5.19339278	5.75714599	5.85387967	5.88502295	5.7035222	6.0529986	5.67102662	5.76694647	5.57300547	5.45576828	5.86665563
MICAL2	6.45809674	6.48870195	6.72373389	6.40498527	7.01164175	6.51605379	6.93952587	7.03988085	6.32102939	6.28711407	7.19676212	6.53782385	6.2198587
RRAS2	4.50103551	5.1144921	4.4505779	5.0861243	5.77240271	5.83990138	5.89083733	6.24140928	4.88222318	4.64854561	5.08533742	6.03678634	5.54180436
HSD17B4	5.80976708	6.21749531	6.0419554	6.55141279	6.17128634	6.77574756	6.31935567	6.25536388	6.15746569	6.66661574	5.62449317	6.22588517	6.32522774
ZFC3H1	5.63449914	6.40788528	6.01596615	6.91014215	6.75781115	6.78036732	6.75445962	6.75853053	6.59760512	6.11441127	6.50140363	6.55508683	5.98310172
RNF122	5.76609782	4.23141329	5.07935649	5.09514708	3.57156356	2.95739136	3.69660766	3.89021571	3.98249583	4.56408107	3.55939288	3.65439419	5.09045854
DUSP26	5.79208064	5.06630332	5.66612779	3.70481101	5.54126096	5.75564142	5.7329189	5.55671917	5.59437691	6.59460271	5.30684375	6.08412287	5.43143851
DPF2	6.39701646	5.38188422	6.11897297	4.87683117	5.5135576	5.57701227	5.82848464	5.32269589	5.14668206	6.24254882	5.69850182	5.9842924	5.35714382
MEN1	4.72382018	4.01190342	4.83833527	4.0706346	3.47521176	3.43563908	3.53817036	3.79714214	3.2831254	3.20677235	2.88571904	4.1149301	3.77688676
GSC	0.6582167	1.66428959	0.46244059	0.28192492	0.79538681	0.23644923	0.3611545	0.23824511	1.73290901	-0.39407262	-0.39407262	-0.39407262	-0.39407262
UNC79	0.59244906	0.46754367	0.47625111	0.54411717	0.53534862	1.12350073	1.00688918	0.85956603	-0.26564083	3.46490203	-0.26564083	-0.26564083	-0.26564083
NUMB	6.57220844	6.06771553	6.32540645	6.4361839	6.24906428	6.01508076	5.94685921	5.9248912	6.08629593	6.3625618	6.11240064	6.00858906	5.9782073
CATSPERB	1.6374521	1.29820984	1.53134299	1.39780477	3.60481538	3.68853257	4.56573643	3.88028741	2.76331649	3.86451492	2.82769545	3.06350416	3.45178823
COX16	3.94790983	2.33871307	3.08410502	2.60513556	3.06271077	2.58125566	2.50097934	2.31241438	3.27279591	3.81464046	1.41606093	2.81584265	3.08637634
TTC9	4.58123165	3.71409087	4.65917439	3.06767258	4.53220738	5.01374704	4.16536144	4.13306585	5.23098107	3.43578796	4.56083304	4.49144404	5.16734616
MED6	4.43563493	4.68920082	4.412628	4.9354113	5.20720595	5.36596892	5.19126748	4.75485521	5.01743726	5.31542538	4.52624211	5.16482237	4.68977534
EIF2S1	5.89198072	6.3062822	5.8421972	6.86874431	6.67407197	7.08122949	6.55040989	6.44956689	6.23698862	6.43314888	6.47714138	6.42960504	6.33713627
ADAM20	3.76732979	3.87726558	2.13471755	2.56563483	2.68684283	3.18137426	3.9274874	2.94097046	2.89814558	-1.43295998	3.54210028	3.16862728	2.53834102
LOXL2	5.61024689	4.75572628	5.83692542	6.85720122	4.78485218	4.08312959	4.17947326	4.19606545	4.76947665	4.38206091	5.01977685	4.68555764	4.59980249
ELP3	5.80036602	5.52753436	5.11130246	5.45885994	5.42616962	6.15806569	5.16574363	5.23214309	5.7190395	5.53957292	5.62889049	5.20421923	6.02340747
PEBP4	3.9871529	1.83030547	3.38314764	1.60229244	1.74262256	5.82757554	2.80393044	2.16266568	-1.06086474	4.52977149	3.27306074	4.18420147	5.48191007
ADAMDEC1	3.16737543	0.76197128	0.38631687	2.01316423	0.50947444	0.48294893	0.31752485	0.64277006	-0.36221575	-0.36221575	2.63635847	-0.36221575	2.51920386

CTIF	4.77580068	5.16929799	4.96421532	3.61649557	4.7817443	4.35845186	4.89551283	4.99257531	4.83715287	4.31759465	5.20074497	5.27393751	4.52444597
MRO	3.40368154	2.13146934	4.03783678	5.15170223	2.12197095	1.70300298	2.03714581	1.65218919	1.48907013	-1.37405428	2.65521713	-0.32782168	3.52794008
MBD2	6.29513527	5.98484635	5.71252368	5.99598982	6.66210108	6.57052337	6.03798777	6.3431667	6.21606473	6.00246826	5.95256148	5.63684749	6.44199297
IER3IP1	3.36495978	4.28547241	3.48168969	5.37122659	4.99517207	5.09240078	4.52274984	4.89138171	5.36754036	3.28113114	5.37392122	4.95483682	3.4963839
MRPS36	3.05490512	2.73460733	3.60694987	3.0023717	3.38537939	4.48710924	4.10055984	3.85615735	4.45710652	3.90278398	2.48476712	3.86100079	2.71641773
CCNB1	2.25511835	1.86731746	4.76159145	5.43840922	2.20441422	2.12292229	2.67882878	2.07298746	1.74137325	3.24166706	3.84352686	2.76023742	3.98437072
CDK7	4.80746035	4.91556404	3.98703636	5.24502383	5.07325171	5.04556566	4.59561659	4.7141059	4.76141759	4.82611308	4.7074213	4.61874959	3.9819497
CD180	3.85711564	2.61394015	4.53335842	4.7932438	2.03291232	1.84915206	2.81895154	2.07068942	3.56483714	1.61044752	2.59324558	2.87991072	2.82572921
IRAK2	5.04590725	3.90355487	5.44671221	4.62711015	3.28840728	3.85426508	3.84933927	3.50379055	3.54443429	2.94179217	3.70082632	2.8683274	3.2384577
CAMK1	4.98549139	4.59995411	5.66525592	4.12555473	4.73665335	4.76797019	4.86481638	5.04882811	4.84389134	5.51089589	5.33466164	4.68510058	4.79703236
THUMPD3	5.04935692	5.21417864	5.63129432	5.83189648	5.66815016	5.77517134	5.66984992	4.98201829	5.24918339	4.94953931	5.16653186	5.12769082	5.65506528
VHL	5.5366592	5.12394218	5.50302346	5.74562538	4.77348124	5.04513539	5.23303652	5.23838516	4.92774984	4.78518974	5.35760973	4.91698525	5.12748522
BHLHE40	7.30630232	7.28453087	7.73280391	8.07396792	5.75172716	5.57508951	5.27141324	4.42527947	5.70571387	5.88611604	5.51451078	5.62377817	6.38921384
ARL8B	5.40430053	6.21022413	5.90832461	6.7719641	6.90140606	7.0949975	7.07792237	6.79638082	6.60256249	5.50802374	6.72872448	6.66674717	5.20364094
EDEM1	5.7558064	6.03775584	5.54831451	6.34915702	5.69017543	5.27461474	5.3068502	5.74415497	5.55009623	5.27952909	5.76835011	5.4205785	5.91166771
CNTN6	1.30163698	1.30411373	1.02666594	0.93257105	1.06679996	1.59507181	1.47417437	2.37228805	1.10537937	3.64237264	-0.60866187	3.76557366	-0.60866187
CHL1	-0.59526747	0.9099824	-0.52633344	0.6134435	0.54990884	0.38031144	1.14478248	0.97683635	-0.05391512	-0.05391512	-0.05391512	-0.05391512	-0.05391512
MEIS2	5.49970073	5.805157	5.11820354	5.41694499	4.68534779	5.77639036	4.77831645	5.0209951	5.54501752	5.25732194	5.66860719	5.63915188	5.29370129
GSTM1	0.62596111	0.62596111	0.62787564	0.62596111	5.12732822	5.7022869	0.62596111	0.6284036	-0.27338068	5.25386066	4.5837503	-0.73656601	-0.73656601
PRPF38B	5.03244688	5.16587936	5.26031286	5.81001634	5.47465332	4.86773947	5.44327394	5.59505761	5.6398242	5.32169326	5.05556852	4.56835983	5.4263873
TSPAN2	5.24953627	5.91223129	5.15508449	5.03466128	7.18563482	6.79105394	7.06279847	7.24747225	6.84054821	6.50622643	7.17650999	7.45294144	5.18020523
GSTM5	3.59137023	2.65757576	2.80773689	1.82517189	3.34646447	4.61875927	4.92441585	3.34198817	4.58048803	5.71639746	4.2718698	3.73004912	4.81430821
GSTM3	6.34478035	6.60817922	5.40052313	5.558289	6.32556166	5.80678763	6.48593946	5.83165779	5.80708581	6.72718485	6.02674047	6.33922393	5.79616126
SYT6	0.6013367	0.49167848	0.74939934	0.92083837	0.49167848	0.5905844	0.49167848	0.49498898	-0.32355565	-0.32355565	-0.32355565	-0.32355565	-0.32355565
VAV3	3.02990091	3.69714629	3.71715449	5.20483053	3.39558209	2.76239123	2.79152242	2.99595892	3.62756729	1.71419702	3.71564979	1.92181459	3.74446814
PSRC1	3.92889064	1.58545356	5.11567615	4.29248591	2.10827886	2.66632315	2.66344659	1.88020145	3.23414096	3.55091347	2.23491268	3.89167041	2.15015914
PTPN22	2.67053252	3.88943823	3.3678823	4.82825412	3.1263442	3.17035413	3.60338502	3.4492879	4.27943108	2.61020224	3.06793633	3.76321345	3.83868162
SORT1	7.46573536	8.35316664	7.32108648	6.59467649	8.52444282	8.76182655	8.58478896	8.77606295	8.27517152	8.36173848	8.65253984	8.47145024	8.53672846
WNT2B	3.53333129	3.47240231	3.04974709	2.17836959	3.69637939	3.42942274	4.24016485	4.77098811	4.02288252	3.38499361	2.86939178	4.47167264	3.52368208
PTGFRN	6.13393978	5.59930904	5.88755874	5.8332975	4.71769718	4.94850104	5.36150688	5.16827612	4.765772	5.16114228	5.42502708	6.05794866	5.7512402
NOTCH2	8.1141679	7.79627555	8.16883527	7.63546763	8.19633842	7.8326013	8.18891458	8.45181533	7.60876668	8.11263371	7.83353003	8.2429361	8.10149355
TRIM45	3.83048026	2.92288234	3.67820621	2.36768687	2.80056527	3.93565532	3.62601106	3.05881984	4.0185359	4.08960729	4.31686854	3.26604709	3.71501903
CEPT1	4.32440503	4.9361929	4.25310401	5.71121755	5.13001704	5.25227001	5.34021761	4.7843962	4.95734517	3.67806778	4.7536925	4.70199803	4.58869268
CD101	2.38664687	3.09704175	2.50412579	3.1493572	1.59290357	2.33864	1.65372711	1.79509429	3.49370193	-0.88642577	2.50858815	3.14045732	1.20567749
NGF	4.71252899	3.19491601	1.90255166	3.18060005	4.53911606	3.8851247	3.28040857	4.53437152	3.83273499	3.14063378	4.00423043	4.34035276	4.49104126
AP4B1	4.69267387	4.60745507	4.70652739	5.26651249	4.67852084	4.39866435	4.76859989	5.19713684	4.57170634	4.77735437	4.60035298	4.93480907	5.57986101
NAPG	5.65714926	5.60379496	5.5295502	6.20012859	6.12698909	6.23620936	6.28435408	6.24684118	6.30789952	5.99543016	6.15745882	6.19875691	5.87127311
SPIRE1	6.20764852	6.20743681	5.83578215	5.9361575	6.32437412	6.68635609	6.28655419	6.30684118	6.04843498	6.57802091	6.14914773	6.10658173	6.07657455
PPHLN1	4.60799858	4.60736758	4.40137501	4.93769196	5.12799694	5.31282571	5.21620484	5.33339912	5.01549464	4.4536998	4.56128016	4.91405036	5.17497811
FKBP11	3.93586736	3.49636665	3.70885541	4.98883941	3.39915384	2.09879097	4.29697165	3.74751747	3.04624015	-1.58559553	4.16206471	2.7529996	3.69495099
ARF3	7.21462753	7.35124048	7.21770375	7.07635904	6.78242893	6.25364266	7.08964085	6.70556185	6.52459836	7.02348191	6.85340173	6.86755504	6.52080204
TMEM106C	5.62319773	5.82871446	4.98945918	5.25467959	6.1292686	6.30945086	6.01055513	6.20402271	6.28796571	6.41637503	5.57142577	6.31691245	5.90102291
SLC38A2	7.05827897	7.79928682	6.73341288	8.41791432	7.7758465	7.87450814	8.42038502	7.96359443	7.67068897	6.7501543	8.03116403	7.41045498	7.19348478
YWHAQ	6.67468371	7.22462914	6.69566886	7.53277323	7.48963286	7.61599109	7.44100093	7.3390737	7.16977457	7.42513776	7.11789145	7.63558094	7.28279349
KIDINS220	6.7149976	6.81345359	6.15300309	6.48055112	6.75984951	7.21601904	6.89256229	6.85020891	7.01587563	6.85138668	6.74222901	6.98125928	7.08852286
GRHL1	0.52897286	0.73171149	0.78943776	0.560666	0.49039127	0.64952389	0.57331151	0.68851702	-0.10553516	-0.10553516	-0.10553516	-0.10553516	-0.10553516
ROCK2	6.69099832	7.36568422	6.5901876	7.06675065	7.68256007	7.89784657	7.69043407	7.75952471	7.93548469	7.6627707	6.95196714	7.68582427	7.60925195

RSAD2	4.45502134	4.94124987	4.65347441	6.18714206	4.25993152	4.53781832	3.53543094	3.71649053	5.05853442	4.8259645	5.38259653	3.53795097	6.27892896
MYCN	0.0242671	0.7604393	0.359738	0.26517552	0.33507205	-0.00152066	0.04506977	0.00027474	-0.00776355	-0.00776355	-0.00776355	-0.00776355	-0.00776355
LPIN1	4.18087361	3.6275583	3.88436455	3.50791624	3.47649624	3.75535651	4.02572269	3.49637367	0	0	0	0	0
CMPK2	4.27249566	4.49116294	3.87054843	4.53632007	3.57322115	3.48831883	3.50565717	3.17822108	4.79035317	3.01061823	4.78901557	3.70385173	4.67162244
IAH1	5.21517247	5.30689232	4.82702689	5.00751247	5.78869022	5.29527657	5.46633654	5.60239946	5.16288896	5.57869599	5.47076848	5.53767594	5.38109287
LDHA	8.76532159	8.31612425	7.65990601	9.50531312	8.74299021	8.06116669	8.71002356	8.14991266	8.24921488	7.87236681	7.71400378	7.84538093	7.9451982
SAA2	2.40457819	2.9344922	2.09842458	2.25851174	2.34871813	1.94290938	2.37343862	1.64823739	-1.09497039	-1.09497039	2.4475907	-1.09497039	-1.09497039
ANO3	1.03084798	1.38794623	1.31967004	1.36790895	1.73776585	1.62185783	1.50086608	1.26323171	2.80372769	3.44564097	2.52164998	2.55717532	-0.70996651
IL6ST	7.53870035	8.51793024	7.53951796	8.88935534	8.58739598	8.83085426	8.76193261	8.64841035	8.48405382	7.47808691	8.40438559	8.24626209	7.52334258
FST	4.12482943	2.70536476	3.18213196	3.67755365	3.61904777	3.76511076	2.35441447	3.86741376	4.99166404	2.60215011	4.73924906	3.84444645	3.94739815
NAV1	5.70730782	4.69474203	6.25078626	5.53958124	5.50091143	4.28083302	4.60717212	5.04260047	4.32873455	4.37707241	4.89644851	4.04983814	4.927366
CDC73	4.23492948	4.33303397	3.5881879	4.5929824	4.86701758	5.56959045	4.97620371	4.76930176	5.34385117	4.35474678	4.78332734	4.5667913	4.18201961
TIMM17A	4.18942557	5.17845823	5.05704682	6.02238297	4.62453638	4.60333628	4.89444125	4.5521126	5.01873969	4.44110676	5.50106665	4.33935915	4.55354623
CRB1	0.14473148	0.26556994	0.14707874	0.05474896	0.05474896	0.63128454	0.30618987	0.10210756	-0.11221671	-0.11221671	-0.11221671	-0.11221671	-0.11221671
RPS15A	5.70806779	6.27002484	6.1426117	6.01411714	6.27694225	5.57201788	6.55442145	5.95837524	7.02994738	7.13737044	5.80666167	6.0235932	6.37191388
NARS	6.32421863	6.47830752	6.32878006	6.75178266	6.34786832	7.05594219	6.51869727	6.62413089	6.89872314	6.49352453	6.32722726	6.36303573	6.54837751
KIAA1468	5.10953141	5.72476792	5.0858549	5.9123343	5.81997802	6.14319475	5.9447256	5.98626159	5.9767221	5.04680861	6.00339667	6.0415073	5.88617515
FBXO18	6.84104549	6.24431112	6.55348526	6.3838991	6.2316098	5.80498266	6.77863972	6.69114495	6.31756284	6.39491849	6.21590309	6.47379717	6.84086463
RBM17	5.52830147	6.17333008	5.67503379	5.90530632	5.70363448	5.70435273	6.07120135	6.02314927	5.85774277	5.85697743	5.86180377	5.76178233	6.35555315
IL2RA	5.46704505	4.62317543	2.2634629	4.32115645	2.12313947	3.01314813	2.88535473	2.0531135	4.01394355	4.21249947	1.97254354	2.9731831	4.17615296
ANKRD16	3.57981061	2.50054021	3.37491565	1.24717104	2.22792753	1.9723664	2.78991611	2.62789228	3.57296224	2.17134938	1.7853572	0.52614256	-0.76677878
ECHDC3	4.03712177	2.8128406	4.1064239	2.84298281	3.02469452	4.09693999	2.76426006	2.02117448	4.09875572	4.23879544	0.65973966	3.48331993	3.28835762
IL15RA	5.30294929	4.28287541	5.00358029	3.96648594	4.01558054	2.68739433	4.69753248	3.65296274	3.62728136	2.97225973	3.38200361	2.68708501	4.28028889
CCNH	4.98411848	5.59455038	4.76383146	6.25634686	6.04181462	6.19250065	5.97970013	5.86863974	5.74899622	5.4758692	6.16710586	5.72793162	5.35357108
HRH4	0.11724644	0.53394505	0.36993631	0.65729075	0.57073647	0.40156635	0.45930292	0.49595491	0.6975528	-0.21668619	-0.21668619	-0.21668619	-0.21668619
KCTD1	4.4354492	3.87949191	4.40993589	4.03828403	4.52675566	4.83274027	4.42646727	4.1660929	4.42277623	4.89233771	4.6354332	4.49796551	5.02983556
CABLES1	5.21058158	4.41366375	4.70285885	3.37676378	5.00047532	5.2800538	5.54490409	6.17486569	5.70600339	5.93082676	5.77821171	5.72516431	5.47326112
DOCK2	5.29635742	5.48107253	5.47492919	5.87774036	4.18352741	3.67618758	4.28675874	3.67481329	5.21337967	3.42575609	4.57839939	3.82704644	5.27873173
EMP1	5.35417872	6.90860011	4.91767265	6.7046114	6.58686881	5.28604921	5.95614791	6.7588854	6.37810315	6.06270179	6.71240348	6.3143074	7.14969596
SOX5	5.40445825	5.4057409	4.80831696	4.91696659	5.41467308	6.09555219	5.50864129	4.60099423	5.42586717	5.46146516	5.15253628	5.31640152	5.33448853
RERG	4.51861395	5.15621553	4.43593893	3.93165893	5.82439184	7.08843364	6.38252524	5.50151945	5.62343248	4.10554157	5.19362893	5.97475445	5.66383481
KLRD1	2.19498498	2.98193431	2.05220995	2.19259593	2.55056994	2.28251087	2.83959196	2.19375124	-1.07388556	-1.07388556	3.08464142	1.81949948	3.42763085
KLRC1	0.45831281	1.43776777	0.46078454	0.74964255	2.27575399	1.09535199	0.92061031	0.63035627	0.22945762	-0.35430014	3.41065847	-0.35430014	2.53699584
LRP4	4.99958466	2.61135316	3.52083151	3.55560408	3.10891854	3.52191351	4.27798144	3.70860741	2.99114984	3.98873829	4.25313939	4.51636285	5.05599949
MYBPC3	1.4313244	1.21976535	1.35571606	1.11112093	0.89170235	0.63513689	0.83175279	0.73158641	-0.47609415	2.2269153	0.81797716	2.94483464	9.41280289
DDB2	5.95440962	5.34209829	5.63187103	5.23364364	5.32134766	4.8735623	6.18624555	5.10556908	5.68985458	5.55183672	5.91901169	5.80010874	5.71926648
ACP2	5.21981674	3.96698252	4.93168793	5.07751002	4.04659072	3.81168342	3.32864645	4.02669848	3.43028172	4.4584239	4.1859096	4.68185186	3.61369153
RAB33A	0.83678748	1.24350072	-0.12631092	0.7190067	0.1535987	0.81539731	0.12622012	0.51554627	-0.29642531	-0.29642531	-0.29642531	1.63424133	-0.29642531
RBMX2	4.86271966	4.00290932	4.48840514	3.85277748	4.5472231	4.68926192	3.82452766	4.52336187	4.89607234	5.17685868	4.03107089	4.34719358	4.89943046
PIWIL4	4.5508355	4.10911028	4.6169937	4.48297281	4.71647303	4.96120914	5.27534499	5.90484983	4.89339527	6.01392289	4.84505635	4.72554487	5.17264251
PUM1	6.72814711	6.29171185	5.89042081	6.02020482	6.09181572	6.18021004	6.10564563	5.98004842	6.22570709	5.67923865	6.00995614	6.00030568	5.81007265
SPOCD1	4.67333611	0.96139994	4.92246852	3.99932032	1.87463658	0.22227218	1.43232865	0.57385508	1.62865256	1.62415167	-0.09088103	-1.22416629	0.92257075
YARS	5.97718947	5.62087308	6.53934778	6.65680111	5.39716075	4.80715984	5.81891133	5.38065267	5.32053388	5.56768141	5.1946011	5.01984631	5.32408556
PHC2	7.7043515	6.21964419	7.18777617	6.30117488	6.15556042	5.3770655	5.90389688	6.21231009	5.2025896	5.68972705	5.67089957	6.19819134	5.70070704
CDCA8	1.22255458	0.92760118	4.02828483	3.49446549	0.39593881	0.78275037	1.72718406	0.45748315	2.30761703	-0.64281329	2.57907951	2.06776195	3.13258301
GNL2	5.03527454	5.40486471	5.61981396	6.67213709	5.52437139	5.53318774	5.11117757	5.52599313	5.99424447	6.00512547	5.19886414	5.12853489	5.99211937
HOOK1	1.70522269	2.85484864	1.79367943	1.85667513	2.15776062	2.42786446	2.2942544	2.07709479	2.33222483	2.34397542	1.87779052	1.81958614	3.87929659

CYP2J2	0.70518883	0.82091272	0.81564502	0.71722263	0.64126231	0.88570861	0.48061483	0.43329403	-0.61624072	-0.61624072	-0.61624072	-0.61624072	6.8263858
BTF3L4	4.79674195	5.75641465	5.21072002	6.11425162	6.24046388	6.15480649	6.15275664	6.00152887	6.04360764	5.21080077	5.92087622	5.93048763	4.70619276
ZCCHC11	6.13182914	6.50657019	5.84367285	6.79541487	6.88202046	6.83318601	6.91660704	6.93457477	6.48477045	6.33256852	5.90460446	6.38213707	6.71377024
PRPF38A	5.56733715	5.67281795	5.4751193	5.63559966	5.83382835	6.47733546	5.90881635	6.10041293	6.06673398	6.05261801	5.43944973	5.74819792	5.6039881
DSC2	4.1160991	4.94470995	3.59662334	5.15462151	3.7934852	3.60808411	3.94922097	3.41287306	4.36400014	3.49614685	3.10705087	4.19689265	5.47843345
DSG3	0	0.0752624	0.00124472	0.01566971	0	0.00069205	0	0.00158797	0	0	0	0	0
RNF138	4.11780103	4.52625034	4.00095459	5.6410218	5.01829341	4.91891438	5.32500305	5.44831423	5.19222131	3.10006801	5.08931155	4.89531744	4.21903006
ELP2	5.47311511	5.80483918	4.82375584	5.84256398	5.75062146	5.54222215	5.97977278	5.85997235	5.49622632	5.50561189	5.75175241	5.86176806	4.89804555
DSC3	4.22957234	7.1406483	5.07719875	5.362401	4.42691699	4.52859886	3.86096718	4.50711554	6.36174492	5.60945977	4.59022087	5.51728166	6.69550368
DTNA	2.02043866	2.9332278	1.22588045	0.38214895	2.97133688	3.58189301	2.11441358	2.87500282	0.06957052	4.81006722	2.75536039	0.06957052	4.4059565
FHOD3	5.57818892	5.91647462	6.27073674	4.62432706	6.06615225	6.44333833	5.74130791	6.14207058	5.99541097	6.08866178	6.03154841	6.45584548	7.27821277
DAGLA	2.23935623	2.26410483	2.80403555	2.16750641	1.32484126	0.98746716	0.93692993	1.46342546	2.52640257	2.76557648	3.27129373	1.84611934	3.624517
SLC43A3	6.39671241	5.60472864	6.85247889	6.19533843	5.11638842	4.45314307	5.99840068	5.62052398	5.07306729	5.72234127	4.99440255	5.05898649	5.98030951
TIMM10	4.83314682	4.54476333	5.10723534	5.06676015	3.69135376	5.23634862	3.78922294	3.89766857	4.70875889	3.90886531	3.9373278	5.47955066	3.50813637
DHX34	4.92331896	3.59168231	5.29020329	4.22593801	4.13183804	3.56665171	4.37355562	4.02045452	3.81685904	3.37922027	4.43295239	4.4001501	4.2045137
APLNR	3.36602933	4.72731735	3.50374768	7.68505663	2.9917996	2.1811377	2.21144334	1.02588789	5.07099733	5.46097542	5.60282019	3.76962815	4.62645825
FADS2	5.36873608	6.24192346	5.47375792	5.70122926	6.31286236	6.8996835	6.35326873	6.5156755	6.55631776	6.6571558	6.99937034	6.76043949	6.0805422
TMEM165	6.35645056	6.41667541	5.58967986	7.17232628	6.51465608	5.87965999	6.30478019	6.76215063	6.45949342	6.14175586	6.08387487	5.84033344	6.10966695
CLOCK	5.81531521	6.19842641	5.68642142	6.33716727	6.5870688	6.96587322	6.5870688	6.49311208	6.33096272	5.52869789	6.45053037	6.35015501	5.83822697
PDGFRA	8.90227888	8.33427129	7.95360253	9.31095772	7.42521528	7.53069641	8.5296726	7.61248724	7.64875636	7.090337	7.73811247	7.11472288	7.69801973
COL4A2	5.44740556	5.73924744	6.96235353	6.09974739	6.48452962	5.94821559	5.82530576	6.59692318	6.09543076	7.18629538	6.46102963	6.34576229	6.13836681
CLDN10	0.01226925	0	0.00124472	0.07733163	0.16528733	0.00069205	0	0.00158797	0	0	0	0	0
DZIP1	5.33689773	5.45037941	4.43783561	5.52184533	5.29097208	4.75283255	5.02883692	4.83271255	5.41882839	4.16945563	5.00922503	5.31860969	4.93680094
UBAC2	1.74905968	2.2731465	1.99438633	2.49559197	1.60752473	1.20898144	1.65918694	2.05091874	2.11697725	0.21234195	3.16089213	2.73673602	0.21234195
ARGLU1	5.77963562	6.08319831	5.6848812	6.08858772	6.64622399	6.46364438	6.70284883	6.95638092	6.65704015	5.93122233	6.45480463	6.20779927	6.21488785
BIVM	1.52470385	2.035756	1.97004217	3.0715781	2.4730669	2.70786115	3.35099927	3.32936819	3.34134348	2.70553506	3.77137795	3.19407807	3.19608856
ERCC5	5.21491982	5.72067613	5.25136366	5.6650129	5.64161608	5.524001	5.5514954	5.65639686	5.65668328	5.80404024	5.49455717	5.11802613	6.03904118
TPP2	5.83453621	6.26850876	5.68904097	6.63303629	6.35397634	6.41676584	6.23404505	6.28296753	6.21410314	6.11144227	6.44418623	6.21282298	6.3700146
KDELC1	4.03283859	3.88382549	3.73869677	5.18028251	3.47155868	4.63432404	4.16762314	4.38055807	4.5497973	4.70892242	3.59389018	3.69497842	3.0650835
CARS2	5.21153245	5.24863024	4.99816076	4.6540882	4.78340973	4.02396664	5.08941855	4.86039422	4.93702779	5.5169519	5.21876493	4.86902728	5.23205117
ARHGAP32	5.65624576	5.60492824	4.84614495	5.76654124	5.05635534	5.14859416	4.84112975	5.74514046	4.60231389	5.63141217	5.25238118	5.04093121	5.89365708
STT3A	6.03124536	6.37969901	5.52072722	7.62003983	6.55215747	6.81956675	6.28856992	6.0694131	6.1288871	6.5708617	6.34300233	6.5608173	6.51084191
ADAMTS8	5.65363426	6.36249813	7.29326304	4.93468769	4.54906804	4.15241952	3.60146503	3.85851965	4.36234349	5.32107956	4.65331345	6.35986317	4.42864074
ETS1	6.00055343	7.13299611	6.09558929	7.72041752	7.2060975	6.9265008	6.40910425	6.79074	6.22719207	6.24685036	6.94759834	6.42052943	6.96913654
SLC37A2	4.38988734	3.49265218	4.03917623	3.8416663	2.71796685	2.14323535	2.44559764	1.86623201	3.20361509	3.74437477	2.51283369	3.42784379	3.73464316
KLB	1.65160939	1.69604764	1.67212094	1.61631689	2.20130097	1.85976064	1.59687328	1.43393313	-0.04568648	-0.75498928	3.63256739	-0.75498928	-0.75498928
APC	4.12389796	4.73025536	4.08134801	4.81634443	4.95691153	5.57931944	4.66361167	4.87996217	5.29142118	4.34486845	4.58657558	5.1730976	5.43259644
WDR36	5.01418094	5.55625051	4.87411168	6.73042475	5.72536765	6.06138647	5.88092214	5.6404168	5.45594159	4.74880953	6.12567869	5.61350351	5.65645376
OSTF1	5.0368955	5.67294303	4.88916315	6.01157474	5.17623097	5.03853066	5.20548073	4.87240821	5.52074044	4.74086712	5.35914876	4.94330418	5.60198513
RFK	4.40804253	4.97111122	3.99737634	4.90980244	5.53843753	5.66642251	5.62667265	5.52532964	5.07969812	5.12120554	5.44748093	5.7157059	4.94891341
UBQLN1	6.47557191	6.72597385	6.02177396	6.92521754	7.27321254	7.03181587	6.94985545	7.11712988	6.84780952	6.67830863	6.95390342	7.09621009	6.70417415
NAA35	5.11664798	5.4562596	4.87351756	5.65599411	5.51716406	5.72729705	5.69373149	5.77155879	5.19152655	5.20969138	5.69852665	5.39722308	5.0591747
C9orf40	2.10004881	1.7570336	2.11344802	2.51921581	2.7054213	3.3315706	3.07249823	3.15344581	1.44942131	2.77761477	3.03479454	3.35900191	2.82749137
ANXA1	7.37501782	8.18004296	7.54678057	8.43030958	7.742014	7.85660634	8.08900144	7.64486719	8.22554924	7.64672813	7.79465605	7.74597747	8.05522986
TMEM2	5.94855654	6.48325688	5.50042924	7.30205456	5.73556093	4.63927961	5.41779694	5.01358923	5.2196497	3.93941967	5.80127053	5.02372407	5.47209321
AGTPBP1	4.73612902	6.15437766	5.21993972	6.29496317	6.07070018	5.9673277	6.08500462	6.18038548	6.3528352	5.86780905	6.20712332	6.27346789	6.25581401
GOLM1	4.86463589	5.69293832	5.07399248	6.29812395	4.42962306	4.44245221	4.52309107	4.68991118	5.28567701	5.10986908	5.60703797	4.34835673	5.04148138

FAM189A2	3.17865661	4.28671235	3.63273873	3.10709564	3.20342306	2.31694735	2.65042386	2.79929691	2.67275131	3.7728119	4.20216989	3.44840414	4.94837393
PSAT1	1.62704187	1.45750444	3.01447034	3.46695721	2.55502008	2.73403599	2.75328082	2.93863122	2.19847229	1.97460796	2.37898946	-0.14078938	0.32519407
ISCA1	5.48630177	5.4425628	4.91833377	5.11758714	5.69610979	6.11846393	5.73593861	5.74877445	5.17687228	5.05665166	5.29105238	5.52999806	5.02787078
ADAM19	5.49746699	5.01854499	5.6118549	6.61738136	5.41408614	4.4576971	4.95747855	5.42119952	4.96700385	5.42197115	4.99635343	5.20409054	5.98492721
HAVCR2	4.87581111	3.95246398	5.00612025	5.52111568	3.25717964	2.53834752	3.74038748	2.84804678	4.36453502	3.35619404	3.38767852	3.69648835	4.54534454
CCNJL	4.00289239	1.77034518	3.11762576	2.37266871	2.50724531	2.87371018	2.96418785	3.76902632	3.64667285	4.39722464	3.1146283	3.32605712	3.39898719
TAOK3	5.66486429	5.76028556	5.2709849	5.78703519	5.47646702	6.06821229	5.68398302	5.38861807	5.90943939	5.67077164	5.49955438	5.53804675	5.82952333
USP30	3.00709256	1.26048948	1.09649015	1.28761398	1.76018168	2.27320526	1.61415417	1.95804567	3.21521542	-0.1595158	-0.1595158	1.49743126	3.191458
SDS	3.15167137	0.75831633	0.07273349	3.86100252	0.00276042	-0.23891121	-0.09204454	-0.1122572	1.8956508	-0.35698223	2.74358499	-0.35698223	-0.35698223
MSI1	0.15427107	0.03151511	0.03403058	0.57178484	0.13731981	0.24447059	0.19556068	0.7603877	-0.17390933	-0.17390933	1.34138333	-0.17390933	3.02483015
FBXO21	6.1724147	5.85847471	5.41187115	5.18301032	5.54612548	5.99469956	5.73631173	6.0651469	5.53371145	5.99018492	5.59499676	5.85318892	5.81377496
TBX3	3.78822336	3.74797828	5.17037189	4.80910757	3.79365656	3.79365656	4.12068572	3.6785579	4.71187081	3.68829102	4.21832221	3.0535863	4.06913966
OASL	1.76175849	1.31594024	3.13320091	3.1387772	1.32979019	1.37920959	1.03402178	1.0510356	-0.14492076	1.45913945	2.6243952	-1.10737225	4.42971231
HRK	0.66756038	0.4865262	0.48911773	0.4865262	0.66928263	1.06582228	0.51793973	0.64549892	-0.36060967	-0.36060967	-0.36060967	-0.36060967	-0.36060967
RNFT2	1.88114973	0.56681029	1.27750262	-0.03743411	-0.340451	1.10431272	-0.09666112	0.01335382	0.06541573	0.06541573	0.06541573	0.06541573	0.06541573
P2RX4	4.93255369	4.28176301	5.00540497	4.19773353	4.201913	3.33294496	3.87317837	4.27508094	4.07856001	3.7975278	4.50650375	4.90878689	3.90529749
DTX1	2.19958412	0.74970474	0.15449982	0.79589491	0.39851307	0.47692432	0.23876003	0.41733905	0.77151766	-0.26765112	3.42040855	-0.26765112	1.55452999
TRAFD1	5.77504089	5.29741497	5.61744155	5.48637354	5.16041515	5.19674777	5.00725112	5.13990621	5.4062219	5.63053836	5.21672536	4.88120168	5.62077748
DMTF1	5.95466533	6.5238129	5.77111732	6.8490256	6.76683558	6.73071244	6.89764251	6.80841607	6.7891083	6.17546028	6.70156021	6.26330308	6.82987756
CCDC146	1.28845595	2.00604731	0.35598316	-0.67359358	1.85777444	1.93264224	2.41589404	1.7719209	0.2978658	0.2978658	0.2978658	0.2978658	0.2978658
TMEM60	2.88699987	3.5217411	2.89658968	4.09282475	3.45898714	3.96669168	3.56219636	4.11823172	3.53217482	2.30337238	4.15891685	3.95928566	2.85489165
CD36	4.31424643	4.85828558	6.51313787	7.61831979	3.20378664	3.28193173	4.11008053	3.04760964	4.56383326	3.91329416	5.96122801	3.94214495	6.8081547
PNPLA8	4.39061684	4.99708815	4.16648447	5.46073179	5.26551561	5.62533704	5.27803423	5.5056432	4.75327067	4.66750662	4.95180663	4.87161215	4.99085216
HILPDA	7.15513669	3.63344756	5.47928358	7.0565477	3.68291406	3.31153702	3.75175473	3.47393959	3.56920955	2.15441656	3.84524995	2.50544908	3.39111254
FAM71F1	0.63650341	-0.22820679	1.33005976	1.00144601	0.24999396	0.44816069	0.53468955	0.22874392	3.07877612	-0.12463537	2.93624987	1.92156147	-0.12463537
RINT1	4.80957173	5.25811982	4.40117353	5.71326711	5.14058902	5.17026056	5.0115991	5.33048631	5.22488399	5.31353045	4.77942461	5.07200118	5.24861901
SRPK2	5.59697568	5.97876188	5.36439003	6.22836606	5.90775565	6.20158285	6.16397732	6.40310886	6.17163787	5.68003446	6.142197	6.09274924	5.8755886
KCP	0.49838184	0.30160719	0.04813068	0.0926994	0.81121326	0.44094241	0.38370527	0.1754401	-0.15036982	-0.15036982	3.35832359	-0.15036982	-0.15036982
TES	6.77986088	5.97332794	6.28249898	5.81966253	7.5587574	8.06469778	7.40130523	7.72038479	6.95208341	7.66346669	7.11913076	7.95031655	7.40490847
MDFIC	4.9090636	5.12685425	5.09708536	6.12871566	4.74278328	4.69824031	5.86474273	5.22938624	5.61461412	4.50581085	6.34522192	5.69818199	4.72726441
MTO1	5.20630471	4.69792415	4.55350247	4.48267314	4.77840265	5.1714536	5.06626408	4.73143868	4.80591123	4.71810332	5.16297488	4.45272828	5.05108285
ANKRD6	5.68241405	6.94390136	6.04454821	4.65509763	6.29014061	6.42970927	6.64707304	6.72563516	7.285159	6.71240933	7.3093271	7.19160278	6.64796171
HTR1B	3.29269542	3.81259948	3.19264105	0.9764405	4.2156646	4.11922324	2.95120295	3.48420999	3.43765774	4.33489392	3.61287311	4.08237121	-1.17708949
KHDC1	1.49506353	1.18166441	1.20156438	1.63750214	0.52100396	1.26046864	0.72427719	2.13609703	2.33764431	3.62176965	-0.31907843	2.6408354	2.88483091
SYNCRIP	6.17486254	7.06485359	6.70163313	7.70346705	7.54772874	7.67500334	7.50683441	7.54289668	7.51288785	6.90120291	7.33545912	7.34770153	6.46869707
SNX14	5.14089551	5.80089252	5.54733047	6.6085626	6.4257721	6.36007455	6.53858114	6.9812124	6.58359703	4.70033655	6.57603099	6.15992854	5.72352312
NT5E	6.16198182	6.45726929	6.34337613	7.33400904	6.32727755	7.26149353	6.7364012	6.77930832	6.57074334	6.01591763	6.63513513	6.32758618	6.2579233
MRAP2	6.3429506	6.51608467	6.4018716	5.78645699	7.39232261	7.63190922	7.87176411	7.72586137	7.70762581	8.06737442	7.43361337	7.81649819	7.1471191
EPHA7	1.97129136	1.81784547	1.69580625	1.75857747	1.94150134	2.09028859	2.03160278	2.01835565	3.7740843	2.01409174	4.5260321	3.74628807	4.94523647
AKIRIN2	5.40893841	4.72524652	5.91243627	5.48524041	5.3024274	4.90220919	4.82957366	4.98017787	5.12328689	5.20953287	4.96625937	5.30491932	4.02535877
ORC3	5.00552331	5.34153347	5.45179608	6.23491005	5.7593262	5.86453352	5.46095125	5.69654819	5.79552627	5.8923714	5.72788687	5.76490589	5.39198302
LCA5	4.37904122	5.06865824	4.38836468	4.92292369	5.25332791	5.90631681	5.09528426	5.15774199	5.64503689	4.79677849	5.61243037	5.01908137	4.27572008
MAP3K7	5.44133856	5.72895401	5.29224436	6.12309065	6.04740978	6.24367685	5.93319784	5.88175441	5.83472763	5.45816905	5.81015854	5.60664159	5.97980588
PRR5L	3.58039108	4.1153083	3.30746845	4.7178196	2.76407292	1.96843768	2.81099891	3.21164745	3.04887965	2.36005375	4.30139628	3.61909788	3.95843507
LMO2	5.25393258	3.86545235	5.23055917	4.04781049	3.39407691	3.61020118	4.21699717	4.27684476	3.66366569	4.44102545	3.66726161	2.79496479	4.39427551
PHF21A	6.9056761	5.82624237	6.06304076	5.78439241	5.81053376	5.89987465	6.0425989	6.05909645	6.01282281	5.89277921	5.64213605	5.33981469	6.1451459
NAT10	6.05744329	5.92283635	5.70040687	5.72496499	5.451928	5.41353135	5.75772518	4.91248528	5.16710671	4.68330266	5.77608117	5.3481944	5.83454851

PRRG4	3.39846987	3.11650811	3.52713439	3.35992188	5.30091962	4.51795679	4.07432154	4.68844007	3.16090219	2.33861531	4.9261216	3.99829966	4.23000374
CAPRIN1	7.1780611	7.35629811	6.89982557	8.0719458	7.45108213	7.64699995	7.67217069	7.46929727	7.27316772	7.50759115	7.40887525	7.20643928	7.19445132
ATP5G2	5.60516481	5.75147399	5.78313993	5.47532806	5.12893874	5.15489035	4.5994144	4.78755377	4.90435246	5.82339421	5.89852661	5.49092038	4.60600033
DNAJC14	5.37062282	5.43302462	5.76218761	4.913036	5.30650343	4.91162168	4.88370616	4.61494048	4.91022512	4.9727736	5.16783251	5.02279633	4.77508676
CD63	7.92125125	7.93392055	8.33880381	8.42650322	7.48224063	7.4601724	7.92125125	7.32091246	7.72435209	8.01050171	7.43553686	7.32008215	7.87061652
PRPH	1.43822895	0.25459434	1.50203823	1.00595371	1.13545457	0.81419269	0.85497217	0.77417181	2.68576631	3.52631872	3.80494838	2.07659861	1.30063874
AVIL	4.36749714	3.59986405	4.57135821	4.76766028	3.28355373	3.14202177	4.89070399	3.55165411	3.40016385	3.32791791	4.18065952	3.7582657	3.74469591
AMHR2	0.04841217	0.40547967	0.05095135	0.11575077	0.04841217	0.04982392	0.04841217	0.05165157	0.05000231	0.05000231	0.05000231	0.05000231	0.05000231
GDF11	4.83926376	3.20449902	4.53257761	4.5116129	4.26019661	4.90751649	4.76017643	4.49496195	4.48790882	5.1263857	3.51028712	4.32307544	4.4357781
GLS2	2.46912257	2.70736674	1.84812254	1.33957272	2.83931293	2.29482094	2.28592377	1.9929899	1.60699898	2.99597219	3.57998344	3.45764108	1.61894533
ITGA7	6.12891671	6.21354849	6.9887403	5.00426668	7.02267394	7.09511557	7.61747025	7.71701195	6.20809315	7.94363454	7.06340899	7.84133656	7.01031986
FAM186B	0.54835707	0.04085969	0.5761589	1.78866414	-0.35948551	0.12308227	0.02780928	-0.14000285	-0.04211228	-0.04211228	3.45025721	-0.04211228	-0.04211228
RDH5	2.76898105	1.5927498	2.24494377	1.95337064	3.09829547	3.48715127	2.67063723	3.33282622	3.72006576	3.21048018	3.44030685	3.258632	0.77313225
AGAP2	2.68980005	2.02217378	2.22072051	2.21064634	2.00035651	1.44759404	1.61317221	1.00240077	0.87829519	-0.87194194	3.40397469	0.97691281	4.14309022
BLOC1S1	1.93890694	2.26838892	2.2427688	1.85715005	3.35525468	1.93620654	0.88304088	2.60536812	0.68612608	4.39935932	-0.49136522	3.87431271	-0.49136522
CDK4	4.91256893	5.06521951	5.46831125	5.09325554	3.72513031	4.12333267	4.34735037	4.19974058	4.98757178	5.46909429	4.59329643	5.12017458	4.56778305
PPP1R1A	1.7902592	1.3606081	2.11591806	1.3551375	2.59562902	4.76426134	3.45694442	4.54659294	4.18786916	5.83412403	3.56302968	4.40006369	5.27122326
TROAP	0.51332607	0.22482581	3.14499412	2.53140136	-0.39879739	-0.19945543	0.74395903	-0.18196349	-0.2440878	-0.2440878	0.85164669	0.81473367	-0.2440878
TSPAN31	5.07160598	5.2077361	5.19031028	4.58321729	5.3357048	5.53072062	5.33525006	5.09335094	5.20783313	5.28956228	4.9217249	5.09725605	4.82742499
B4GALNT1	4.46826507	3.42405033	5.04062369	4.62893103	3.33625994	3.51752024	3.13029836	3.06626436	4.07097517	4.45634401	3.85626441	3.11693049	3.83293753
TFCP2	5.03665677	4.65011581	4.98190399	5.42496809	5.01310129	5.46390305	4.82609047	4.62405018	5.11899078	4.65074722	5.13231025	4.9808509	5.64252536
COQ10A	3.51248685	2.0854508	2.78134484	1.9060634	2.45179517	3.55635729	4.36394382	4.01627488	2.54870113	4.57295552	2.8341417	4.17206798	4.73990704
FAIM2	1.10122514	2.70103231	1.29078764	1.09636974	1.57647679	1.49132026	2.59799441	1.99183189	-0.98825663	4.29602317	3.22946871	3.96617832	3.47282926
PAN2	5.59121987	5.36776979	5.41890461	4.72022654	5.02335846	4.88064055	5.55091473	5.1349058	5.04034821	4.77585013	5.51719683	4.90376136	5.67984587
ESPL1	2.20791296	1.25408556	4.79423954	3.44101429	0.89507179	0.91918577	1.74783469	1.05022556	-0.94974286	1.52528415	2.27190888	-0.94974286	-0.94974286
KRT7	2.60691943	5.7879041	2.98157536	3.87221243	3.97136136	3.48573764	2.83971611	1.97634949	4.02070096	2.47045704	4.79809945	2.41776972	2.147762
ZC3H10	4.95664616	3.93029373	4.51100474	3.72388196	4.24150629	4.52743298	4.25800537	4.61061413	3.79399189	4.44647257	4.79396729	3.74070077	3.8166214
HNRNPA1	7.56297632	7.26554064	7.33553488	7.60088152	6.98000114	7.16415273	6.80569712	6.57123495	7.44297777	7.7235252	6.94021944	6.92963038	7.53408987
SLC26A10	3.39807525	3.6575232	3.33231555	2.53641544	4.23934952	2.10109517	2.96548617	4.74238715	-0.13947162	3.84844849	3.31424763	4.01435	4.04321771
ACVR1B	6.07740839	5.09328216	5.44602576	4.98942646	5.00548722	4.55752112	5.04277903	5.2170796	4.10873151	4.75585983	4.68894129	4.38719469	4.82520879
OS9	7.66643298	7.3220957	7.79771395	7.41205747	6.95918728	7.10302118	7.21702008	7.17945876	7.39539743	7.47149859	7.36346323	7.20949585	7.4825727
KCNH3	0.48734312	1.12180248	0.13548898	0.52188314	0.66858455	0.16323592	0.25642052	0.78930453	-0.26700452	2.90867045	-0.26700452	-0.26700452	-0.26700452
LTV1	4.41267622	4.69402992	4.15890996	4.90936456	5.46946429	5.45246769	5.20854055	5.16416531	5.23433116	4.10709558	4.62548462	4.484652	4.77749862
MAP7	4.04147977	2.92112929	4.10317354	3.07441936	4.0371347	3.90480637	2.8046103	4.45966062	2.99999047	3.89460544	3.76022087	3.35584237	1.94526529
CD164	6.28477442	6.7008179	6.15829744	7.33144152	7.34480046	7.65202166	7.67699814	7.2799793	7.55872563	6.11022522	7.70753424	7.45074549	6.16649006
NHSL1	3.53460616	5.4780312	4.00954677	4.25153716	3.39730543	2.89383219	3.06383168	3.46546839	3.82711225	2.40569682	4.63515532	3.67228044	5.81615195
AHI1	3.66598938	4.72950545	3.72149587	5.04891097	4.72461785	4.80240448	4.80975225	4.91227214	5.10316947	4.43195642	4.86355145	4.5429994	4.02779623
HEY2	6.08696734	5.40902762	5.53552596	4.17700228	5.75657095	6.59770078	6.36279646	5.91082627	6.01026147	6.82834237	6.23452411	5.99897706	5.53299547
PKIB	0.19603208	1.49302363	1.48806521	0.87472	0.3434517	1.29875275	0.6086552	0.32016301	1.11396535	-0.51620434	-0.51620434	-0.51620434	-0.51620434
NMBR	0.05851901	0.61988347	0.41071739	0.05851901	0.17820813	1.06733007	0.29178975	0.06169148	1.32868759	-0.1755724	-0.1755724	-0.1755724	-0.1755724
SMPD2	4.24132019	2.93854567	4.19348073	2.82361615	2.9829461	2.71269616	2.99494445	3.39672802	2.14508198	1.97742204	3.68111474	2.45522599	3.75619072
MICAL1	5.96269476	5.04624865	6.25480599	5.35533138	5.80620592	4.47964697	5.71711392	5.71316138	4.85782511	5.28770704	6.63517476	5.46613918	5.73208466
REPS1	5.58939789	5.25285277	5.00387585	5.57507269	5.53516148	5.40554499	5.37122333	5.91508798	5.14995153	5.96843427	5.38016266	5.07991306	5.2815499
STX11	4.37460041	4.17190985	4.84482654	4.91242062	4.59393991	2.91930695	2.91815123	4.647295	3.2919571	3.84611347	4.244735	3.75345428	4.65639958
TEC	4.18113771	3.59725974	3.00335524	4.01701221	3.72401937	3.99974	3.72841751	4.1491364	4.08813401	3.11537611	3.77433498	3.20799805	2.92379527
PRADC1	4.02729446	3.66331604	4.89089906	4.44861363	3.50129853	3.03956747	3.44191874	3.56740132	4.42589335	4.53291861	3.99714723	4.34183047	4.48053272
SEMA4F	4.70198013	4.2944435	4.74605907	3.75901914	3.69475489	3.99073491	4.14744013	3.74312894	4.53349255	4.37763213	4.69715413	4.15883478	3.89676978

CCT7	6.86914334	6.60791163	7.4290153	6.94753097	6.61807133	7.06138876	6.7259942	6.20629158	6.60411879	7.23197713	6.87190577	7.07453019	6.96327328
RAB11FIP5	6.20026957	5.73963364	6.58984163	4.96471074	6.11786694	5.44196721	5.92918707	6.24374595	5.77655852	6.61244982	6.25853886	6.84315262	5.60542449
SMYD5	5.2213314	4.47476021	5.31618186	4.69511304	4.65947357	4.19369159	4.46879583	3.97970583	5.07123929	5.4031471	4.970058	4.8047453	4.5787162
DYSF	3.41306689	4.24707701	5.59817684	5.915303	4.66952883	3.88589895	4.43446838	4.49190233	4.53318168	5.89852996	5.04113502	6.25071529	5.97237282
CCDC142	4.66441501	4.28858669	4.76263761	3.58899944	4.27643427	3.33688914	4.28200349	4.46277048	4.32207428	3.85473881	4.83864213	4.45300582	4.85644445
KCNMB4	2.66536935	2.1023922	3.22003407	2.89800906	3.34936093	2.9630393	3.36077225	4.7734155	3.79603236	4.868784	3.83908535	3.77070331	3.07477479
USP15	0.75875579	2.27503029	0.95587809	3.15767334	2.89667895	3.1539476	2.66705917	3.41741021	2.97930099	-0.48354944	2.70778857	3.34852907	-0.48354944
GNS	7.53260384	7.09829203	7.39330299	7.74154857	6.98026542	6.82602658	7.75900952	7.2026632	7.03872178	7.44110068	7.07836641	7.41957506	7.79079166
MDM2	6.45923356	6.43571235	6.70493087	7.45800997	6.36004513	6.62428912	6.95131388	6.59547971	6.90853832	5.95693549	6.78676142	6.52972085	6.44271156
KLHL36	6.11831395	5.74588477	5.88789801	5.01838351	5.77105288	5.5521859	6.0040932	5.80298166	5.45428801	5.5944863	5.93522445	5.88286218	5.3184559
MPHOSPH6	2.93603576	3.78993839	4.35498918	5.50794082	4.48918093	4.26092265	4.5724818	4.31354839	4.62029406	3.3177573	4.48991247	4.36107478	3.18751465
KIAA0513	4.89591729	4.31358834	5.05716247	4.07486402	4.41572124	4.36049705	5.08557274	5.17983174	5.25086186	5.15346244	4.4389421	4.90564977	4.16754655
DYNC1LI2	7.41793867	7.61309427	7.31899201	7.71995205	7.61309427	7.31420566	8.17157718	7.74069486	7.4982241	7.52294222	7.58828625	7.65548335	7.44096947
FBXL8	1.81002428	0.37002197	2.5726547	2.07322767	0.38140975	0.99785529	1.99183812	1.62635568	1.80529664	3.07704028	2.50858835	1.48654849	-0.33322434
FHOD1	4.87171537	4.432512	5.77343395	4.85926504	3.91397082	3.48273694	4.44030525	4.18654247	4.07299364	3.44104072	4.38273624	4.30894214	4.13507021
CCDC102A	5.3943769	4.57922259	5.58638144	3.96692053	4.36413573	4.80519993	4.81811296	4.47395137	4.68854314	5.50484096	4.98869393	5.29662772	4.08970618
SLC9A5	2.94317749	1.20098564	1.6714715	1.13041028	1.75334405	1.26335825	1.19851339	2.2447735	3.32912852	-0.57682959	2.85329826	3.04571362	-0.57682959
AGT	6.19684008	4.05871368	4.54391142	3.94018398	4.0154369	4.21209298	4.07295448	3.63572558	3.77857279	2.99137745	4.65966822	3.96300226	5.70308915
KCNK1	2.76710877	1.03687391	1.58670548	1.5424592	0.45706898	1.66289778	1.41701188	2.12450894	-0.3220033	-0.3220033	-0.3220033	-0.3220033	3.653009
URB2	4.18131036	4.29577318	5.09440699	4.48173231	4.42911907	3.69440252	4.52324307	4.28765272	3.92850906	4.19958812	4.21869933	4.72402505	5.02644106
EGLN1	5.92354832	5.81835377	5.53362039	5.89715202	6.15205344	6.46628513	6.35074007	6.12121256	5.92813773	5.94122183	6.1183288	6.21322713	5.8803759
CAPN9	0	0.04841187	0.44638527	0.00973881	0	0.00069205	0	0.00158797	0	0	0	0	0
COG2	5.18263139	5.03020888	4.60448301	5.06269223	5.08590493	5.54726201	5.49207994	5.03810346	4.97973272	5.23079641	4.81564973	5.0765373	5.75052351
ABCB10	4.58292648	4.23751472	4.73172907	4.57670234	4.97849606	4.75388865	5.44051794	5.62418665	4.99823985	4.97974527	4.95347946	5.86453535	5.22903259
NTPCR	5.13090604	4.82567816	4.57323906	4.22117706	4.99297933	4.81670135	4.94502158	4.54626372	4.25829761	5.13270651	4.71326446	4.41435408	4.88435379
TAF5L	6.0570579	5.40518132	5.45909014	5.30996366	6.20234822	6.34680358	5.84394681	6.51983335	5.69272481	6.19531195	5.63926373	6.5110531	6.02865446
GLUL	8.21506623	7.56085522	7.35926835	8.45239002	6.81869723	8.19322783	7.02346335	7.19799771	6.74442216	7.06749797	6.967169	6.95615144	7.34634078
STX6	6.07207342	6.2673644	5.61965479	7.09013839	5.75871293	5.91150986	6.04926829	5.84192278	5.62925853	5.23977392	5.83458216	5.34074265	5.66541834
RGS8	0.78170494	0.62290147	0.48151653	0.54242012	0.47891371	0.48036084	0.51089748	0.4822343	-0.26670664	-0.26670664	-0.26670664	-0.26670664	-0.26670664
RNASEL	5.3840154	4.87397522	4.94503176	4.74714782	4.74039923	5.65893952	4.76703215	4.95915588	5.0989057	4.65423645	5.16410005	4.46354275	5.33044606
DHX9	6.37508924	6.79429976	6.49629299	7.26729596	6.89731815	7.2392602	6.91754566	6.59243293	6.64936615	6.75079577	6.75888461	6.9751549	6.89113279
KIAA1614	4.55482546	3.53186886	4.64489207	2.87064432	3.54363108	3.22408475	3.33649311	4.19897434	4.04750335	3.63594648	4.05952706	4.6489492	4.07985872
CEP350	1.2111738	2.41655442	1.30903828	2.93321824	3.1235983	3.60658875	2.69002759	3.04747263	3.52153561	-0.14013377	3.35709633	4.22690382	-0.14013377
NPL	5.01038904	4.42375746	4.81591951	5.92365806	3.67860028	3.03249838	3.76819954	3.15179947	3.85675626	2.3550573	4.36888364	3.91443047	3.94570069
FAM129A	8.75163237	8.86298779	8.56117647	8.03319496	9.31711312	9.00403215	9.08701881	9.31711312	8.6723251	9.40597724	8.86064606	9.3440426	8.97366756
PIGC	3.99260401	4.68354308	3.49358729	4.48513579	4.37142512	4.74670628	3.87704584	4.47360448	3.86365936	3.88146761	4.2198837	4.62800299	4.15145739
LAMC1	7.66256444	7.71342335	7.86623419	8.57439769	8.27674014	8.15652768	8.40013329	8.92457118	8.13209523	8.50837417	8.00098308	8.47598363	8.79614287
RC3H1	6.29369725	6.25486821	5.7319072	6.50995107	6.60457903	6.531862	6.78691332	6.79340507	6.16033492	5.77627154	6.56791154	6.22669139	6.0628957
GPR55	0.62473944	0.45395298	0.45655684	0.50188533	0.45395298	0.45540069	0.62975759	0.45727489	-0.25032722	3.81795144	-0.25032722	-0.25032722	-0.25032722
SP110	6.18634561	5.24672279	5.49428151	5.59411888	5.82506173	4.80450407	4.82317271	4.54928653	5.74309889	5.58896993	4.76789295	4.23767677	5.88491142
MRPL44	4.06435405	4.05708933	5.03541398	4.48447297	3.87073512	4.33066404	3.9812151	3.83300238	4.38912483	5.15366826	4.40616831	4.0203755	4.6517997
DOCK10	5.51207246	5.73293645	5.99090827	6.85237768	6.7661166	6.01304132	6.00734405	5.87826998	6.28652928	6.06045685	6.63636241	6.04807888	5.95340778
TTLL4	4.99520463	4.7160215	4.43772745	4.94837617	4.61502904	4.0987779	5.09014888	4.78966682	4.77001446	4.26490116	3.7994318	5.07942061	4.52514092
USP37	4.67051458	4.93136077	4.60842357	5.5272077	5.36693841	5.60470601	5.34649075	5.39211757	5.18063721	5.23601749	4.93808958	5.06388239	4.65696633
HTR2B	4.01565343	3.07176482	1.18038652	0.98920605	2.44823836	1.87404799	4.1202873	3.25055308	3.75969673	3.51340819	1.92558395	2.98237146	3.22714794
ITM2C	5.81557471	4.42955056	5.81439303	4.80369164	6.3033844	6.91429504	6.79878622	7.04960948	4.92718819	7.6016859	5.21742995	7.20952762	5.8404726
SLC19A3	3.11554546	4.00368556	2.49648916	2.62206395	2.64493607	2.23763628	1.95851345	3.018573	2.70025002	-1.19933234	3.41255068	3.58414275	3.58321107

SERPINE2	5.76004881	6.14427464	6.39819526	7.58635116	4.47256383	4.56361553	5.54455535	3.83510801	5.5262588	4.13385852	6.46532499	5.09339646	6.12057114
DNAJB2	5.79375258	5.45082063	5.7999059	4.42861545	5.12398839	5.25625053	5.12746159	4.92206237	5.2947797	5.71713966	5.56208354	5.75325458	4.8807082
WNT10A	0.30888531	0.34815636	-0.04194004	0.11429714	-0.04431131	-0.04299291	-0.04431131	-0.04128612	-0.00596826	-0.00596826	-0.00596826	-0.00596826	-0.00596826
TMBIM1	7.51547241	7.63606624	7.52860301	6.47169804	7.71670428	7.30561691	7.66490448	7.81314513	7.41877028	8.07480939	7.79622064	7.48014405	7.40309483
CYP27A1	6.18156845	4.87820097	5.76155813	4.17920862	4.69945241	5.18119778	5.15737974	5.36762259	5.03266628	6.39272632	5.26594141	6.15588531	4.59693304
EIF4E2	4.11114461	4.32499193	4.30086944	4.17884059	4.12637155	3.91440897	4.61334092	4.20141719	0	0	0	0	0
ARMC9	5.7172059	5.73242863	5.64623156	5.01557894	5.57220482	3.97218358	5.8519059	3.53863669	4.96956473	3.99159765	4.80991322	3.88577928	4.97412947
CAB39	3.22003538	4.1082094	3.53205611	4.17392091	4.57775983	4.32994262	4.43876867	4.21719037	3.73214259	4.41583314	3.9854698	4.82791731	4.34453648
COX5B	5.43411575	5.6917741	5.30970169	5.34357443	4.93875304	6.22378738	4.91578138	4.98473897	5.48676277	5.84616215	4.59961623	5.6202923	5.9757152
REV1	5.46656704	5.90534529	4.97616123	5.65440122	5.72343462	5.77236191	6.18348458	6.11933309	5.92352784	5.69593287	5.94023758	6.2041379	6.26013686
TSGA10	0.64271239	1.23442602	0.25362603	0.66895614	1.35668428	2.01152809	1.54180085	0.80431481	1.414002	-0.31095986	2.0120604	-0.31095986	-0.31095986
MFSD9	4.25955408	3.35597118	4.37522042	3.74248178	3.43740982	3.93776735	4.20170559	4.18736232	3.8113765	3.6791198	4.94201351	3.54307992	4.20890454
TMEM127	6.78709731	6.3034199	6.55721361	6.09216783	6.11015683	6.00680813	6.13535326	5.9953535	6.06017925	6.582151	6.44252465	6.1193368	6.39438567
TGFBRAP1	5.46731828	5.52677782	5.07186407	4.61154193	5.08369197	5.15151401	5.07091308	5.3067183	5.03757974	5.30927891	5.2923193	5.34806254	5.11080684
GCC2	6.02689783	6.24797687	5.64208298	6.56437253	6.31712818	6.71108606	6.23491796	6.3939742	6.89690211	5.94428166	5.8184986	5.98721687	6.34183824
MRPS9	5.10473888	4.46567783	4.88517235	5.22280857	5.02958813	5.86061356	4.82548307	4.72727118	5.05153228	5.73495866	4.41428012	4.94380435	5.42703399
C2orf49	5.07580315	4.89726445	4.7984414	5.3422931	5.500044	5.77477527	5.52476743	5.46227469	5.60292506	5.04523941	5.7303662	5.06785549	5.42012954
ANKRD36	4.91817449	6.01703609	5.22536626	6.65210969	5.58193592	6.45798138	6.09301562	6.37719382	6.18782227	6.08156319	6.45510999	5.34211131	6.14300155
EPC2	5.65606522	6.06554906	4.98376203	5.83853745	6.12472182	6.54261915	6.14489979	6.30317894	6.40594262	6.11851857	5.99507448	6.21812981	6.05278866
ARHGEF4	3.58441279	2.90759861	3.13900429	1.81460202	2.63180522	3.02212553	2.65989442	3.48054856	4.19188852	5.02669629	3.19354991	4.47336425	3.08228247
ISCU	6.13703517	6.59837962	6.3361432	5.7798529	6.17854964	6.89722994	6.1922567	6.26215052	6.88319174	6.43408442	6.24626539	6.60146248	6.55122033
ALDH1L2	5.46896042	6.09925707	5.80673478	7.50633899	5.95583645	6.23132595	6.28936809	6.28159983	6.51324181	6.01146211	6.56293113	6.17931279	6.30636537
STAB2	1.19338828	1.70022839	1.19598599	1.83307215	1.33511512	1.26753918	1.19338828	1.19670235	0.09754957	-0.57948765	5.41597605	1.98139418	4.48195962
USP44	2.95190673	2.51498994	2.76772765	2.21174242	3.3168352	3.65352891	3.61584721	3.95457605	3.50098238	3.08065207	3.42413658	3.83855329	1.56305995
SCYL2	5.23341373	5.75085607	5.23869111	6.44530935	6.26945346	6.46373428	6.21045914	6.11563487	6.28353806	6.06487521	5.95698762	6.16288375	5.26358848
CKAP4	6.59105722	6.4984475	7.48857922	8.18790176	6.44422755	6.64184152	6.31465283	6.10467978	6.30444249	6.49424802	6.67033516	6.405022	6.4791811
PLXNC1	5.16204479	4.9491269	4.58793745	6.27675991	4.70578242	3.6969154	4.47009498	4.28197367	4.84478829	4.48825904	4.78350096	4.54486457	5.39097284
APPL2	5.38477652	5.91340545	5.02493318	5.5224415	5.49068414	5.34868532	5.92055854	6.40698058	5.9156617	5.36172149	5.70196882	5.76199145	6.11005011
PWP1	5.16090501	5.85112656	5.1717069	6.34365754	5.68220179	6.05558724	5.4326378	5.67720044	5.34341931	6.26757782	5.82436499	6.06877017	5.63274968
DRAM1	5.14935039	5.78128948	4.59266531	5.23360761	4.78553239	4.41340196	5.37546094	4.77473062	4.95833086	4.63700323	5.1206311	4.80476684	5.17489255
SLC41A2	6.04407491	6.79145685	5.76483635	6.44136732	6.91284341	7.270266	7.24907153	7.11925001	7.23943405	7.00714016	6.86362125	7.05579925	6.40732193
VILL	4.06961421	3.34902047	3.58839544	2.43690642	2.6613138	2.37504013	3.05701097	2.9020726	3.06275186	3.88096746	4.37648041	1.11533756	2.05889787
FLNB	6.50994035	7.00451014	6.32627644	6.91347162	7.12642958	6.23066304	6.83470161	7.88690268	6.85546013	6.76910902	7.64044258	6.8551896	7.55910794
NEK3	4.62329856	4.66683035	4.24349861	4.70481588	5.1722335	5.17581135	4.71994642	5.30911109	4.66158513	4.90118832	4.46408653	4.4374473	4.47213468
VPS36	5.52104195	6.17424824	5.54943604	5.99372885	6.16826184	6.32648166	6.21564564	6.39708285	6.15584036	5.81273409	6.28523202	6.23562683	6.28760238
RNASEH2B	4.27828298	4.54668652	4.37649902	4.95963541	4.83253559	5.72275394	5.19070822	4.92525403	5.04587119	5.54715496	4.21903531	5.14635075	5.3063333
CKAP2	3.82708494	3.85213988	4.75901244	5.97495276	4.02313085	4.37209062	4.46821483	4.48514747	4.98805902	3.5205146	4.17816772	4.69781185	4.74278013
TBC1D4	4.20281841	5.27072344	3.9387813	6.07026362	4.17506725	4.5139096	4.19523492	4.44063918	5.10387762	4.48607928	5.18915751	4.08753215	5.69353799
THSD1	1.58188374	3.76827907	1.79459228	4.07753773	3.03767667	2.10660008	3.48851495	3.51960007	4.31778139	4.47451115	2.54343041	3.77397526	5.07301284
BORA	2.52016128	2.59510394	2.7981367	4.55598169	2.57453548	3.25301383	3.78288803	3.25663389	3.96631544	2.93067195	2.27130487	3.68452828	0.18783804
LRCH1	6.58928896	6.55514022	6.05408938	6.36545985	6.82441092	7.01037103	6.68478001	6.76300743	6.53214205	6.55603002	6.61265634	6.37400228	6.44020649
SUCLA2	4.94880173	5.53332522	4.85936967	5.87108652	5.84131232	6.22023335	5.97018395	5.76023496	5.71843033	5.94838228	5.28057579	5.81125398	5.93834067
RCBTB1	5.34296967	5.53641315	5.13332483	5.04063755	5.45409227	6.03776359	5.33145307	5.26346932	5.53086832	5.45920606	5.78803093	5.45154936	5.66203937
MED4	5.36605383	5.33631509	4.70839967	5.63578476	5.71473901	5.75553405	5.62115798	5.60382654	5.83552425	4.92423363	5.62785047	5.86036686	5.21681076
PHF11	4.96860333	5.41012746	5.01729671	6.03517217	6.11445899	5.83540339	6.10282333	6.2216608	5.87104352	5.59196174	4.87694888	5.30650124	5.45132725
COG3	5.46770723	5.8403655	5.40285927	6.45647905	6.14756046	6.07495874	6.26374171	6.35998402	6.07428986	5.67346199	6.41284695	5.66956953	6.10810715
LMO7	3.30127861	3.71629123	3.28961085	3.40177751	3.73752748	4.77706489	2.99767169	3.71629123	5.24205851	3.26788654	4.72959836	4.42833133	2.1662443

SCEL	0	0.65667083	0.00124472	0.00661165	0	0.00069205	0	0.00158797	0	0	0	0	0
ITM2B	6.97113738	7.48188975	7.03865876	7.6255721	8.03241656	8.23057366	7.56227518	7.73651527	7.89557617	7.52437887	7.76561518	7.79704802	7.29784321
SPRY2	6.64351732	6.09705667	6.50685793	4.89344956	6.08176881	5.73471492	5.84983228	6.25313321	5.16295586	4.86773074	5.79393676	5.15178237	6.37775507
NUDT15	4.3347132	3.11019555	4.83864951	4.28526887	3.80283926	4.58089967	3.74508572	2.72670919	3.9969375	3.21575673	3.53578243	2.8599635	3.59075472
EDNRB	3.44841817	4.06923592	4.18267649	4.60338385	5.42326409	4.17158885	3.99768255	3.29567387	4.27757932	2.82360248	5.2477108	4.42144308	4.63876794
RCBTB2	5.00446641	5.2649476	4.8170867	6.16251881	4.60649121	4.96582553	5.34225642	5.01333411	5.3820431	4.30436694	5.46118745	5.37230297	4.928846
LCP1	6.48043184	6.62752652	6.79910455	7.29385213	5.33105056	4.6615992	5.67824355	4.84485207	6.25868584	5.19596207	5.57659817	5.15107813	6.73095602
SETDB2	4.26391038	4.88007469	4.84904259	4.97739936	5.31326757	5.48354542	5.50323406	5.46795937	5.32457757	4.07097116	4.85551664	5.09381909	5.17344017
SCRN1	6.63294701	7.13000694	6.30834225	6.46149116	7.25036199	7.41446112	7.10712142	7.03909243	7.02511027	7.29819016	7.04266573	6.8468736	7.11974488
C7orf25	3.76675138	3.64779928	3.96810173	3.97397201	4.61853646	4.37179827	4.24513895	4.59806544	4.9101766	4.1109078	4.37059799	4.15845308	3.95396535
TNS3	7.87598182	8.22574512	8.32938501	7.67609469	7.4243228	6.78657653	7.67137159	7.62536054	7.99160654	7.74520859	8.51257729	7.57642975	7.63781796
CHST12	3.74777591	2.76735864	3.86398991	2.39265878	3.2521671	2.76735864	3.76491309	3.60987648	2.9175045	3.69892286	4.22817967	3.85066773	1.99640382
IGF2BP3	1.08651555	0.56282995	0.49210132	1.59682304	0.59909464	0.61654346	0.50559824	0.53547626	-0.47520193	2.00111603	0.60205794	0.56576729	-0.47520193
GPNMB	8.79890214	7.7933819	7.63116078	7.82273563	7.98279232	8.20000265	7.64853146	7.2530105	7.95284651	7.83092779	7.76760121	7.84082939	7.95675198
RAPGEF5	5.28537283	7.08098932	4.70983468	6.61556574	6.03270187	5.12503025	5.22671892	5.12147988	6.57134851	6.27342416	6.2028688	5.99953213	6.02828854
RAC1	7.30445763	7.66368246	6.95664543	7.24063238	7.79500201	7.46106775	7.86336919	7.8665531	7.50614157	7.95090928	7.70962173	7.67424232	7.33307696
KDELR2	6.44878519	6.79205191	6.22034628	7.87161596	6.83315055	6.56202176	6.86386267	6.83705523	6.86498557	6.94424145	7.51410295	7.01989196	6.57229618
NUPL2	4.00997671	3.8367438	3.8834732	4.45001953	4.30207761	4.47135977	4.85795853	4.96266178	5.01488772	3.84344968	4.30050636	5.11222058	3.67753717
IL6	6.26021989	3.37274812	5.73873783	9.3579771	2.82042795	3.21244064	2.98804594	3.0009252	4.00714547	1.49712617	3.65986718	3.32370505	5.24822865
ZDHHC4	4.97886753	5.09692597	4.59314711	4.68222706	4.36464593	4.49959303	4.11148597	4.21066364	3.60839477	5.39017121	4.31597882	4.74470911	4.36175787
AOAH	3.84107053	3.63123464	2.91633073	5.57006452	2.26219984	1.14098608	1.8068671	1.06588297	3.46704859	-0.77601671	3.9955679	3.14879661	2.76763055
BZW2	4.33278242	5.07195732	4.82880716	6.3597522	4.40894132	4.47289772	4.6336977	3.35859584	3.88106824	4.35936993	2.97246176	3.7549716	5.78922904
DGKB	0.28600676	0.26837164	0.14037544	0.14729107	0.39806304	0.21535089	0.19531784	0.69012368	-0.14614978	-0.14614978	2.95877882	-0.14614978	-0.14614978
TBRG4	4.17467059	3.95196396	4.81640972	4.61449636	3.92436661	3.64472607	4.0073013	3.8465507	3.68231923	3.62668692	4.39000766	4.68469815	3.80769817
DDX56	5.48492607	5.42160518	5.76174932	5.17381256	5.17604239	4.69884327	5.44730088	5.15461722	4.84348367	5.20186246	5.15439166	5.86766072	5.26437823
HUS1	5.07055608	4.00855607	4.97458384	4.90613798	5.04564313	4.73390792	5.1262432	4.7040662	4.67104553	5.27763117	5.29937782	4.50058395	3.84982605
NACAD	4.55558997	3.87025857	4.66182684	2.42705396	4.53250776	3.84899529	4.24577997	4.35236497	4.03669963	4.33050756	3.65866051	5.46098214	3.43853287
DBNL	4.46969221	4.0856539	4.74415803	3.75605171	3.70750559	2.86349289	3.52198938	3.720578	3.89019979	3.5686071	3.47919805	3.21895128	4.04237561
CCM2	4.65527902	4.11963558	4.80718205	4.52090025	3.88735033	3.45596184	3.5167373	4.25057407	3.14102334	4.91400163	4.31856751	3.75310147	4.59190633
MYO1G	4.59166886	3.47618111	4.67909972	4.84255085	1.83334493	1.16407958	2.288464	1.34295584	3.28118756	-1.64634266	2.81325872	-0.56011075	3.70720611
TTYH3	5.25016915	4.79045733	5.87034179	5.43911946	4.03481215	3.34348641	4.44515885	3.8654602	4.51894275	3.36674934	4.59929055	4.32703655	4.28852177
CIDEB	4.91681433	4.20221214	3.78058608	3.69784655	3.23289419	2.75231224	3.55748749	2.77000699	4.46690104	2.66083606	3.55408206	3.78085384	4.45280413
TTC5	3.98849701	4.22065067	3.99576547	4.42164148	4.24976099	4.19343623	4.38727359	3.82857338	3.83861804	3.61537288	3.30914916	3.9875695	3.54354762
ZFHX2	3.76732269	2.69473284	3.89565529	2.51082948	3.34337017	3.54420214	3.57621613	2.94319262	3.19361155	2.56121777	3.75589268	3.14557115	3.81032961
MTHFS	2.36761902	1.51349827	2.5363599	2.55821886	3.26984264	3.39088482	3.34267066	3.05711553	-1.25332039	3.39955051	2.37595298	2.90856219	3.2645325
ADAMTS7	4.00068278	4.12492189	5.14304146	4.13417263	4.90634838	3.45612944	4.24487927	5.14254044	4.52490082	4.43942023	5.47297316	5.76309948	5.12928711
IREB2	5.41212825	5.99842012	5.58095409	6.67213759	6.21696956	6.09174768	6.34578031	6.33066322	6.17286432	5.42762171	6.28177235	5.93786393	5.59085853
ALPK3	6.88973171	6.98611401	6.70188874	5.30241172	7.67324157	6.46398571	7.20180203	6.83331591	5.67071105	6.88913007	6.94335449	7.13767615	6.55683757
TM6SF1	2.87064883	3.59956023	2.86299915	4.2187029	4.32717214	2.82665659	3.53003872	3.91955651	4.01616643	2.0260269	4.55401687	2.20999858	4.4671672
CIB2	3.19551935	1.99684564	3.60344923	2.64714509	2.50484756	1.55455226	2.45152135	2.13988074	-0.905638	2.3007397	2.89148396	3.12030227	4.29801477
CALCOCO2	6.54043175	6.90193862	6.45687256	6.44625669	7.25351876	7.44013228	7.19497876	7.44159152	7.06720865	7.0747221	6.84809572	6.7450669	7.26953402
RSAD1	5.22737918	5.01270314	4.95219382	4.35987919	4.5518425	4.33685469	5.13400863	5.04019856	4.38510294	5.36679139	4.47880941	4.92250242	5.00941505
NMT1	6.64199825	6.12806538	6.6352495	5.9623233	6.02060684	6.14966166	6.64199825	6.10295479	6.06407217	6.6936701	6.38159396	6.28476702	6.2435776
MYCBPAP	3.1368338	1.09541855	2.67928877	0.71138785	1.7963028	1.95253925	1.66530092	1.3502224	1.92966967	-0.79821474	2.00594902	2.3085937	0.82900569
SRSF1	6.68293521	7.5284458	6.62147376	7.74055113	7.17046574	7.24610528	7.28937408	7.19461138	7.04982909	6.76203102	7.2876375	7.26566755	6.70824775
VEZF1	6.70102689	6.55566449	5.57750505	6.21190317	6.7331533	6.83913305	6.6683137	6.52181561	6.13943083	6.22323729	5.77414921	6.2583901	6.53915616
TACO1	4.44795948	3.35377169	4.49948902	3.51413177	3.37805206	4.24383528	3.96190397	3.31692698	4.13224991	4.41855817	4.16314754	3.71980315	4.29232159

TEX2	6.51610652	7.31249585	6.66335684	5.85506473	6.43712951	6.57980161	6.40756183	6.06774496	6.58108278	6.13803038	6.61581336	6.20992165	6.79009997
DCAF7	6.29422856	6.41137556	6.19272434	6.25934113	6.24674649	5.89641911	6.86815539	6.39036592	5.90138562	6.51760157	6.24622697	6.37024988	6.29174411
LIMD2	3.24770238	3.15937789	4.11665581	3.876931	1.97879445	1.39317211	1.9118582	2.11038002	2.57284368	2.12321511	2.62600316	2.5891617	-1.0719607
BRIP1	1.95272215	1.89239119	2.76739675	4.78457243	2.32718658	2.62753515	2.42268556	2.22926824	3.53302343	1.43798385	3.0060026	2.84128254	1.85827596
KAT7	6.28974756	5.96411779	5.94306941	5.59861142	6.37547092	6.34313922	6.36413373	6.30027108	5.8058309	6.25090492	6.1860079	5.74541841	6.26370895
RTP4	3.19501454	3.24498422	1.70065493	3.33378313	2.71382511	2.52337474	1.38908456	1.82571042	2.87547931	2.3354381	3.74430019	2.92939425	2.78284947
ACTL6A	3.86568452	4.2690173	4.25280841	5.40677276	4.72284159	5.05861665	5.08613851	4.65210011	4.77333267	3.15195254	4.81356769	4.65998167	4.42469936
NDUFB5	4.57427858	5.32088796	5.01123175	5.38452528	4.94951456	5.53241476	5.582297	5.06440757	4.97903596	5.12975691	5.11029201	5.56844721	5.28828076
MRPL47	4.12730378	4.60966776	3.83647622	5.36779074	4.91732168	5.24391345	4.18294273	4.02281018	5.41080354	5.46914624	4.63118883	4.67435155	4.63285124
TRA2B	6.21002342	6.79432476	6.1442233	7.33068783	7.00799781	7.08964601	7.01983013	6.95531603	6.6200917	6.25699887	6.89106451	6.63526312	6.43365147
SCN2A	-0.41445205	1.25302396	-0.1540005	0.20295329	0.36007183	-0.12235991	0.56937708	0.4013984	-0.01020539	-0.01020539	3.35223202	-0.01020539	-0.01020539
ERMN	0.44380665	0.23187903	0.46717695	0.85842275	0.29561095	0.68720675	0.2713706	0.39278644	-0.22639053	-0.22639053	-0.22639053	-0.22639053	-0.22639053
GALNT5	4.71476791	4.94788517	4.54913673	6.34975148	4.58785322	5.07537725	4.44894906	3.45195663	5.24618944	4.80898114	5.35828421	3.99751823	4.96666082
SCN7A	0.76994882	4.36726989	0.77239842	1.05930922	2.26279902	3.28614128	3.45067525	3.0852467	4.96066144	5.44904284	5.99402848	3.0560896	6.14190517
TANK	5.81234361	5.68280994	4.69627726	6.67222032	5.90710597	6.05175427	5.44459162	5.73499758	5.80830923	4.5625476	5.45151659	5.31958329	5.20839803
BLK	0.2408726	0.75075522	0.04210751	0.4646551	0.03958191	0.37389778	0.03958191	0.04280398	2.83842929	-0.0641722	-0.0641722	-0.0641722	-0.0641722
GATA4	1.67931705	3.30223795	1.67413513	1.21142547	0.15528848	0.00069205	0.04606918	0.08286461	0	0	0	0	0
SKIL	5.9985133	7.16919925	6.0873646	7.47335008	7.10811167	7.19277938	7.31870792	7.48401663	7.69666753	6.80313001	7.6151078	7.37958142	6.44150567
EPRS	5.8468576	6.59287379	6.44579725	7.39590037	6.54999035	7.05298886	6.69592839	6.70813793	6.83881052	7.0156765	6.55946855	6.42629654	7.11021099
HLX	2.77777945	2.44990981	2.53546525	2.85688249	1.8618293	1.78247767	1.31599809	1.68204313	2.32370015	2.18132194	2.82800855	2.97516113	0.4278644
VPS45	5.16021815	5.58454558	4.83959199	5.74721141	6.14881074	6.19903908	5.7752947	6.15631873	5.72488736	6.03684684	5.78084963	6.19535776	5.95002283
IL10	1.64143181	2.08320218	2.47869259	2.26564338	1.24455564	1.50437497	1.15144628	1.15467771	3.49541455	2.74469348	-1.01905538	2.48973859	2.65200696
KCTD3	5.70246427	5.9912453	5.4842339	6.40349054	6.07567843	6.12575303	6.16425247	6.31697178	5.97174989	6.19848711	6.01401382	6.10494635	5.88290728
RPS6KC1	5.14788719	5.90980939	5.254287	6.01848576	5.73886517	5.81580858	5.75359104	5.94439168	5.89983504	5.46779758	5.97062861	6.153037	5.59106227
IL1RN	4.11829704	1.70046757	4.85529948	4.66728317	0.54229022	0.33937959	0.64597253	0.59128634	2.41694437	-1.06518571	3.71487116	3.30657312	1.72257919
SMPD4	5.80467849	5.42264862	5.95651637	5.41396955	5.25475689	4.69892863	5.33505899	5.38374975	5.23732685	5.5133091	5.42648552	4.97441737	5.4241864
WDR33	5.52437394	6.0174316	5.75243333	6.17726123	6.03896223	6.05328839	6.09578368	6.27823232	5.94686592	5.44441139	5.98622351	6.08617925	5.97294588
CCDC115	4.95793916	5.39117007	4.82749615	3.94639316	4.53640811	5.40029446	4.45626625	4.64932053	5.06621877	5.3696873	5.39200359	5.69137781	4.42058956
SAP130	6.30245725	5.48059414	5.46675653	5.25542779	5.47518353	5.58681148	5.40973937	5.4797365	5.4337275	6.0940292	5.5574696	5.16291636	6.04527797
BIN1	4.83653037	5.21586857	5.10579122	4.4689093	4.27661903	4.24944352	4.30326741	3.91988804	4.46486163	3.49487502	4.5723264	4.70958637	4.76341844
IMP4	5.29630465	4.52685188	5.43940644	4.67519028	4.70533926	4.78487005	4.63485543	4.3733523	4.50378279	4.75826207	4.62332324	4.36449402	4.15980052
HS6ST1	4.29875322	4.45258861	4.00879304	4.24792684	4.29844195	4.10761616	4.47945058	3.16391509	4.03317531	3.35822572	3.92384026	3.93526382	3.7062104
UGGT1	5.11621494	5.1491043	5.18028411	5.91896406	5.08990813	5.63486113	5.45828415	5.00793156	5.73750067	5.54196524	5.50019137	4.90678259	5.06776305
GYPC	6.55642102	5.81628018	6.19997184	5.25445319	5.97752842	6.89044548	5.7817161	6.01413104	6.44782902	6.95427714	5.99116864	6.30005392	5.98868977
STAM	5.57050853	5.02011459	4.79963357	5.80140803	5.79572248	6.04779518	5.68720666	5.70796321	5.58221221	4.90918789	5.00160225	5.64300981	5.47503598
ABI1	6.33936815	5.83089206	5.33992127	6.29228507	6.22082967	6.29726377	6.16887713	6.4598447	6.1758257	6.08699423	5.81766233	5.61892415	5.60413015
YME1L1	6.32889438	6.99196261	6.31505063	7.07817066	6.89586037	6.94142094	6.89825091	6.76385868	6.64375724	6.64922787	6.77468081	6.7443021	6.64507314
DNAJC1	4.98633432	4.55776539	4.97826341	5.38933291	4.6509768	4.77027191	4.83852582	4.62834295	5.29902415	5.70140534	4.78220001	4.37546331	4.50575114
NIPSNAP3A	3.95197005	5.3541958	4.73601046	5.59617783	5.43689117	5.33008692	5.53986855	5.53722131	5.90301067	5.4737888	5.92488742	6.32371631	4.32460557
LRRC8A	7.15269105	5.75739132	7.29295225	5.47166309	6.80261982	6.00413138	6.42414667	7.02536393	5.95182612	6.24733984	5.81020379	6.12033406	5.97483422
CDK9	6.16991426	5.71672114	6.11470707	4.93867107	5.94211086	5.48694373	6.031869	5.82849496	5.39458704	6.31993074	5.60494749	5.87399806	5.76769983
TXN	6.07703676	6.70970161	7.36206133	7.46012352	6.41669538	7.01919924	6.72498804	6.21330577	6.36657145	6.51119073	5.81449032	5.9088052	6.71405942
ODF2	5.65785547	4.98987765	5.93201991	5.91030767	5.26688738	5.29348705	5.38444113	4.92987033	5.43406022	5.05667701	5.03492896	4.82268718	5.37902842
KIAA0368	6.32777248	6.79271411	6.14786544	6.71507833	6.7005936	6.63986856	6.85664338	6.71507833	6.36188362	6.53825128	6.28849181	6.34257537	7.0749976
TOR1B	5.64611422	5.12032863	5.08633398	4.54138689	5.13555414	5.12682926	5.22346413	5.18230422	4.87010775	4.81217911	5.19258094	5.36318003	5.07673901
C9orf78	5.82434424	5.09911502	5.58438092	4.69735182	5.31885898	5.88365377	4.84119361	5.05332173	5.92667681	5.83169511	5.06632339	5.04227032	5.66022374
SMC2	4.37876373	4.78961419	5.20314189	6.20770457	5.18781431	5.53154172	5.41996181	4.91454964	5.62070337	5.38556092	4.66779697	5.02852627	5.25894447

KLF4	5.05052324	6.51143884	4.94042344	4.55887423	5.73323005	3.93907549	4.89334515	5.2405835	5.70583477	4.38972121	5.14411731	4.91465279	6.29108944
TOR1A	5.45523528	5.32996429	5.16078847	5.9090771	5.38982569	5.87210224	5.12581448	5.23886615	5.47981147	5.59195159	5.63250513	5.42501848	5.49057754
RALGPS1	2.85738683	3.08068351	2.40009119	1.58234328	3.68602558	2.56158471	3.44765006	2.99909576	3.1908149	3.6110384	3.16084882	4.15881621	4.13947002
FAM129B	7.31544308	7.19212246	7.99528683	7.38924212	6.37419346	5.43582432	6.82744922	5.80565615	5.91210709	6.03086228	6.73234087	6.27975399	6.1071385
OR13C9	0.33875159	-0.04380937	-0.04152949	-0.04380937	-0.04380937	-0.04254179	0.84408386	-0.04090077	-0.02724195	-0.02724195	-0.02724195	-0.02724195	-0.02724195
ST6GALNAC	5.11672923	3.65500276	5.06626762	3.76707352	3.3334385	3.87164622	3.93398064	2.88418091	4.03755926	4.42652119	3.98146161	3.88741471	2.58756615
TMOD1	6.44439701	5.59443109	5.73137361	4.61362976	6.26008178	6.65900388	6.11008914	6.48512813	5.30033689	6.3141851	5.34493867	5.45456329	6.80951601
DAB2IP	6.62055753	6.55590491	6.97006942	5.43509847	6.54258868	6.22099852	6.50643805	6.3233054	5.89843113	6.65991364	6.7653012	6.49271672	6.16550537
STXBP1	5.53933839	5.13991055	4.97863358	4.1150472	5.27786093	5.07721598	5.3818311	5.02422227	5.26809337	4.87242547	4.98199227	4.98589034	5.99809612
SLC2A8	3.74156021	3.73890487	4.33764774	2.20335128	3.20688366	2.4924776	3.08424096	2.80300896	3.64483297	4.31048501	1.44863229	4.0200072	3.38623671
ANGPTL2	8.33300653	6.50597044	6.16863788	6.885669	6.27371608	5.64220209	5.46745567	5.757825	4.85914199	5.71257712	5.46051012	5.71803899	6.33458805
CDK5RAP2	7.48768768	7.12487785	7.13404526	6.16902369	6.62906428	7.019479	6.78848028	6.55741471	6.95337445	6.7366815	6.49434098	6.36298221	6.96950063
ZFP37	3.51176735	4.0353893	3.75284223	3.97228918	4.20334602	4.98427906	4.61153406	4.05086655	4.41323864	3.33208788	3.88139573	4.14115802	2.98963155
SLC31A2	4.36100699	4.61498773	2.66161523	4.14143932	3.08039211	2.64798483	2.73040155	3.35319943	3.22925454	3.8458973	2.94258904	3.705766	3.84284546
SLC31A1	5.34557267	5.10070811	5.32209721	6.26045158	5.05458741	4.76416021	5.08812659	4.79009487	4.88786679	4.59867102	5.52090632	4.86178449	4.9581
TLR4	5.80922572	6.61288288	5.58257317	7.19017049	6.50276558	6.2804209	6.61616619	6.42520726	6.78934385	6.02071971	7.01759902	6.62267221	6.39855303
ZNF189	4.7956667	5.65556339	5.31803891	5.9634525	5.83297126	5.93563099	5.82642821	5.87404904	5.93294568	5.64222638	5.63782534	5.84176998	6.14211573
STX17	5.91162641	6.54336835	5.62121682	6.35264542	6.45179313	6.54722497	6.40131175	6.31749649	6.17490656	6.14653632	5.9454309	6.09723013	6.29430336
PRPF4	5.24116064	4.76712329	5.09170693	5.46739198	4.4465248	4.55782377	4.69210852	4.46517438	4.73174512	4.66905856	5.04542178	4.50996144	4.67289854
FPGS	5.50753195	5.12364163	5.77806987	4.63639517	4.91057029	4.40592996	4.97936031	5.16066881	4.30881254	5.0758932	4.86147195	5.03802018	4.49938046
USP20	5.09496959	4.57385913	4.91673453	3.71032338	5.06315034	4.72065507	4.92434708	4.96936784	4.54773855	5.34840788	4.64733665	5.67784598	4.53094249
KIF12	-0.04746495	-0.04746495	0.40108395	-0.04746495	-0.04746495	-0.04662891	-0.04746495	0.11857303	0.01254311	0.01254311	0.01254311	0.01254311	0.01254311
ATP6V1G1	4.98905241	5.00840681	5.09146736	5.58684749	5.39693942	6.11418495	5.45123545	5.54967463	5.92447024	5.73207033	5.18612665	5.49203653	4.79012913
TEX10	4.10786668	4.94375143	4.12832984	5.75260363	4.98056971	4.98843394	4.8933005	5.11374384	4.48246711	3.939079	4.0533031	4.69281854	4.34710622
GARNL3	3.63339872	3.19192836	3.71398545	2.84775411	4.11866557	3.90990848	4.31812854	5.10747082	3.21124235	4.7520428	4.18486299	4.53009076	4.05834111
MRPL50	3.96856615	4.37299897	4.1344571	4.95774357	5.04867776	5.46920712	4.64366782	4.74214715	4.6927674	4.85824921	4.54515908	4.01996182	4.86051989
DPM2	4.4894774	3.37572233	5.13501315	4.67869758	3.18751611	3.15954387	2.84104264	3.20945175	3.67694661	3.82482979	3.93883584	4.21199854	3.86390747
TSTD2	5.73227042	5.62374439	4.89529511	5.48641181	5.9116197	5.34777102	5.30692247	5.01840534	5.18771592	5.84850982	5.39547627	6.07023514	5.86116691
HEMGN	0.31382218	0.08082841	0.08340053	0.34020264	0.08082841	0.08225848	0.08082841	0.39103926	0.67391374	-0.02904227	-0.02904227	-0.02904227	-0.02904227
PSMB7	5.54017032	5.63503393	5.3943789	5.72731067	5.08443438	5.38649858	4.73725454	5.16244147	5.42183533	5.77888314	4.77351342	5.45189013	5.67152991
RABEPK	4.9539451	3.97781881	4.98945181	4.96755471	4.35905007	4.42181261	4.24255283	4.35953686	4.40573836	4.81514526	4.25229795	4.62219573	4.10742437
GOLGA1	4.86312718	4.21485031	5.0331682	4.6016065	4.46532903	4.56956072	4.60242485	4.58227184	4.41426349	4.7809709	4.87935556	4.72414461	4.48340413
XPA	4.4126796	4.93002729	4.87165912	5.21617601	5.13137439	5.43022992	5.72639546	5.54935398	5.69639509	4.90757828	5.77866563	5.14766677	4.89397937
NCBP1	5.18449671	5.83018012	5.33578479	6.38567491	5.7953698	5.95151941	5.80624221	5.87474416	5.74933085	5.12654012	5.65019607	5.706826	5.96331173
ANP32B	4.85448239	5.26533369	5.92862009	5.39746754	4.70879408	4.92279155	4.91296169	5.03435546	5.3235018	6.08924162	4.9535596	5.23103652	4.82881795
PDCL	4.53152796	5.00769006	4.80445495	4.8012449	4.88512666	5.29154165	4.58959904	5.22144414	5.47413102	4.63373131	5.33427068	4.75397169	4.60033202
RPL35	7.26346654	7.24071724	7.10658086	6.76838462	6.65262654	7.69281935	6.01533855	6.58588217	7.25434211	7.62993252	6.71866784	6.81269056	6.75283261
LMX1B	0.07192166	0.25818354	0.16076593	0.12726634	0.17466222	0.03405729	0.25987252	0.47936072	2.18029822	-0.04196309	-0.04196309	-0.04196309	-0.04196309
ARPC5L	5.81261231	4.25645368	5.50071034	5.8349595	5.26292604	4.93977619	4.56104844	4.20691926	4.5372222	4.37966074	4.7063145	4.48326499	4.73598837
ENPP2	6.74295531	5.62453624	6.89098736	6.24258779	5.49492019	6.92228252	6.41810183	5.1349527	6.34025514	5.64634438	6.08391634	5.88694563	5.70585324
DSCC1	2.32362283	2.55288703	3.2668759	3.33687219	2.86868798	3.91669802	3.22365976	3.71423283	3.98505153	1.80382914	3.37709038	2.99417538	2.39181408
DERL1	5.95925671	5.72754098	5.53181371	6.23076997	6.05229855	6.31767642	5.70549925	6.00721073	5.71990023	5.82612733	5.75978226	5.85307145	6.23545321
MYC	5.74364343	5.55209049	6.79196878	6.71651725	4.45130165	3.75025682	4.55470014	3.7755495	4.19687767	3.77134021	4.7098261	3.66422012	5.34215988
NOV	7.24233817	8.1695617	7.83470838	6.12587283	9.52829093	9.35240767	8.85115004	8.97522351	9.14397762	8.93483866	8.88753786	9.09502435	8.42808616
IL33	6.17762234	6.06640106	6.11756713	8.02205112	5.63519308	6.30910551	6.83314423	6.76435866	6.51238775	5.95689773	6.00425633	5.55052294	5.93317828
RANBP6	4.45486555	5.04372572	4.79337866	5.57956713	5.23693616	5.7938522	5.45862267	5.16647071	5.64743009	4.87360648	5.4894477	5.45601921	4.23777421
POLR1E	4.30916206	4.40035857	3.70251605	3.93214524	3.46241444	4.62484166	3.56858143	4.14101429	4.17140513	4.4784297	0.48751039	4.31769875	4.36160965

PLAA	5.4877906	5.7383457	5.86846618	6.26365417	6.21392815	6.48535797	5.99518455	5.9752022	6.06466137	5.65701269	5.9260692	5.8250161	5.80865988
IL11RA	5.34715616	4.87456014	5.07965054	3.70540528	4.73893933	4.55672656	5.19056275	5.03618514	4.76356898	5.55254663	5.65086661	5.81699067	4.68032012
UBAP2	4.92361306	4.49748819	5.03100567	5.19430642	4.91014595	4.56760574	4.59679441	4.51964741	4.81751534	5.1684239	4.29327173	5.0167699	4.89643861
APTX	3.92839907	4.30481831	3.85322887	4.23809674	3.74364746	3.41010383	3.32563502	3.89242693	4.30397104	4.44034855	3.56112596	4.49523442	3.06342054
RNF38	6.42415544	6.44226413	5.8035572	6.07938054	6.73324521	6.65717499	6.79807487	6.57866471	6.25628669	6.57493058	6.49473288	6.63452659	6.7153515
TLN1	8.28498338	8.79138506	8.49128016	7.54222237	8.97878837	8.55612931	8.78666575	8.78666575	8.24569485	8.92333174	8.80417047	8.9422522	8.56158338
CCL21	4.74919406	3.39736476	-0.23246033	4.56684181	2.60480884	0.89086257	1.53841553	1.02612337	5.45748793	3.71204304	6.46002876	3.80394406	5.27304251
SIT1	2.49251919	1.04851985	1.14274351	0.91220194	0.47295803	0.62440728	1.354879	0.21507213	-0.44824746	2.90083979	3.20827309	-0.44824746	-0.44824746
DNAJB5	4.84476988	4.34533122	5.09103554	4.25081201	5.73238806	6.19883919	4.94075262	5.88353142	4.71182072	5.49009877	4.03984171	5.91814196	6.09507667
SPAG8	2.60999397	1.31868098	1.94211621	1.15210618	1.58706986	2.5280652	2.16842491	2.30279123	2.43639145	4.02213105	0.9584892	3.0360012	3.72967364
DCTN3	5.30122861	5.14635293	5.49350765	4.90201286	4.69180169	4.99098397	5.09046529	4.49535943	4.63513715	5.47177462	4.81047882	5.18410715	4.97531291
CD72	4.04102505	2.03955586	2.87027038	3.17351449	1.97033404	0.64844651	0.69311024	2.36209127	2.29181376	4.13409373	3.13091631	2.87488965	2.40616409
TMEM8B	5.16707043	4.97237387	5.05241647	3.39011018	4.59766521	4.6391392	4.55353502	4.64070575	4.66486288	5.34781812	5.05002828	4.64863304	5.04977189
GRHPR	5.33724333	5.09468294	5.66747412	4.49617192	4.85829306	5.21013073	4.73421092	4.93560433	5.17900186	5.42480062	5.58663065	5.10158677	5.35942164
ALDH1B1	6.15040465	7.00799117	6.89573244	5.37884907	8.04706373	8.07339435	6.99683277	8.39032393	7.20619883	8.19890395	7.91983434	8.32208347	7.69763375
HINT2	3.74103611	3.28301001	4.06967866	2.66687753	2.66026741	3.5044903	2.57308137	2.92306494	3.18922061	4.19880201	2.33147039	3.0557591	2.89619926
IGFBPL1	4.00478987	4.51540037	5.07628186	2.97576699	6.4197068	5.69439439	4.42492471	5.62744744	4.47573583	5.56390586	4.52076264	6.49986695	5.52568783
DENND4C	6.35297114	7.02850101	6.39704378	6.98410092	7.12899565	7.21180696	7.1943585	7.22280607	6.83893616	6.7009464	7.02267169	6.69841194	6.92617242
RPS6	8.41921589	9.14854541	8.23280416	8.41255895	7.85140464	8.08364144	7.88897997	8.0914413	8.45701587	8.56080126	7.78067142	8.24485849	8.03167102
CNPY3	5.61284342	5.59840335	6.13910359	5.42969238	4.95452718	5.32739275	4.90826355	4.75241691	5.63401998	5.53939609	5.34312052	5.17328975	5.70406735
FOXP4	5.4059052	4.50468249	5.75137937	3.68535448	5.06196803	4.71442561	4.88471785	4.74734371	4.7281741	6.07004265	5.10579007	5.1877285	3.71371973
PPIL1	4.41346666	4.1201211	4.78866422	5.46559822	4.44947896	4.95297427	4.38131756	4.02901443	4.96112567	4.38358031	4.3679854	4.68671402	4.5913356
KLC4	5.11842103	4.17550291	4.93944939	3.54107356	4.35603019	4.6120825	4.69153046	4.42676577	4.25766025	5.28709953	4.41429381	5.3046441	4.60285963
KIF13A	6.93877679	7.3699052	6.90369029	6.3844671	7.43413986	7.48191355	7.71539298	7.61712142	7.48292698	7.59374065	7.47773759	7.45802477	8.05697272
PIM1	5.16423442	5.84449723	4.9719996	5.81859794	4.10566767	3.42362242	3.98963942	5.04545537	4.01244492	3.05176511	4.1404206	4.64130739	4.31582456
GMPR	4.2596844	5.00782133	5.41467669	3.48637164	4.90819545	5.50265076	4.98470088	5.07699893	5.31718694	5.4626091	5.52372641	5.30502505	5.46003441
TFAP2A	3.51196253	1.52400037	1.65708676	2.03079846	2.58211746	1.71721527	4.75219796	3.69777647	-1.21113386	3.71662001	2.02144354	2.47725933	-0.15425948
YIPF3	6.87106075	6.0247142	6.06393903	6.43447124	5.7600664	5.70162111	5.75808041	5.84831204	5.6108125	6.72723216	5.98465036	6.50555958	5.47296356
TMEM14B	3.78130258	3.93113981	3.78409894	3.98900863	4.07974702	4.26047714	4.85724562	4.55168373	4.31712298	4.51954709	4.29522578	4.81200329	4.0665403
TMEM63B	5.20127246	4.74170116	5.41307576	4.58798972	4.39319458	4.36571675	5.17254248	4.67982572	4.33147188	3.61587766	5.23471112	4.85025464	5.74467318
FRS3	3.77633338	2.51128951	3.64155049	2.47147975	3.02187742	3.15957279	2.84480462	2.62537088	3.42376781	3.93242318	3.59601111	3.16525059	2.66429488
TJAP1	5.24091842	4.65158132	5.3816462	4.11447693	4.67080415	4.10421177	4.75635576	4.73184432	4.44896874	5.25250756	4.40816144	5.19899251	4.5929081
CAPN11	-0.11662508	0.23940721	0.15945578	0.60846248	0.80833994	-0.23338326	0.5033427	-0.20296608	0.00490143	0.00490143	0.00490143	0.00490143	0.00490143
KIAA0319	0.42603506	0.53695031	0.71768599	0.53015892	0.58837882	0.38064642	0.47263143	0.38251778	-0.28179387	-0.28179387	-0.28179387	-0.28179387	-0.28179387
IRF4	2.90174202	4.13203552	1.64911673	3.5524039	2.05069967	1.5893484	1.74503618	1.79113363	3.7378022	3.08031871	2.60449845	3.40243387	2.2030261
SLC22A23	3.70290608	3.50892023	2.85541989	3.09309517	3.4569687	4.54072256	2.70613965	2.57467878	3.31217834	4.16079522	3.78595231	2.95443941	4.68740826
TUBB2A	3.46998387	4.49207837	4.38202686	4.67545274	3.11711241	3.40344208	3.6123639	3.50330094	4.14110045	4.99356116	3.79766641	4.60833019	4.02883982
LRRC1	4.58750334	5.0587012	4.54639186	4.24433441	5.14821088	5.00624658	5.28893713	5.88027081	5.22525584	5.33314823	5.17386736	5.79472642	5.17386736
FOXF2	0.01659492	0.03380766	0.00124472	0.03222744	0.19788784	0.00069205	0.20762336	0.11235265	0	0	0	0	0
BPHL	4.43425257	3.57166804	4.11011111	3.97452571	3.79934473	2.92465118	3.27236548	3.33141067	4.06914851	4.16369827	3.92595261	4.0736044	3.7995681
RIPK1	6.10746162	5.67849004	5.8992011	5.80974317	5.38558805	5.58602273	5.75178137	6.10502212	5.51576147	6.00490328	5.02117809	5.63593305	5.84695674
TUBB2B	1.41869992	1.35991483	1.76844243	1.56239297	2.40755347	1.91636788	2.14507644	1.76641113	-0.12479524	2.74056769	1.79750655	3.23989986	0.84884343
HMGA1	3.16434475	1.71406506	5.72752723	3.74682161	1.31984113	0.37811836	2.04419909	1.06666226	1.4225067	2.68311827	1.86462788	0.65348983	3.66390448
TCF19	3.58199141	3.66707038	4.98140145	4.69195503	3.03310949	2.8866982	3.71792159	2.93643516	3.20037997	4.24055355	4.24659567	3.80860567	2.74444007
FLOT1	7.98348235	7.59558099	7.68144789	7.14743717	7.18395395	6.52355683	7.07060546	6.46381612	6.78076202	7.09721391	6.79255124	6.81925112	7.21079377
IER3	6.92004981	6.14360287	6.72357493	7.74852374	4.03933398	3.59613754	3.90189205	3.37737772	3.99822698	3.64625302	4.99446327	4.07437512	4.89194165
MDC1	5.86203127	5.15204324	5.89515302	5.30541325	5.6216901	5.24120753	4.96592728	5.36806519	5.3410903	5.67342397	5.5594863	4.87635293	5.94226434

PGBD1	4.38355461	3.98245892	4.36008115	4.28268792	5.04018207	4.75619411	4.73655473	5.09117411	4.22981557	3.84116276	4.41644708	4.69006058	5.02659916
ATAT1	4.32333472	2.65938568	3.74506901	3.85474755	4.03507526	2.50175334	3.38618838	2.97005438	4.07973361	4.14411146	3.83700376	2.88593709	3.47307604
TPMT	4.37244536	4.24986108	5.22557113	5.3452324	3.99544483	4.61140074	5.05233132	3.94628635	4.91621406	3.87647888	3.99913923	4.24861153	4.51774004
RNF144B	2.38826481	2.64308573	1.93418227	2.97813507	2.44604857	2.20160253	2.77531433	2.21256662	0	0	0	0	0
NRM	4.535308	3.76946233	4.96090614	4.15221764	3.48142371	3.3830591	3.97596963	3.45537446	3.58410688	4.03819581	3.45589708	1.90913752	4.29213403
MTCH1	6.5387112	6.59331924	6.82414133	6.24432201	6.43773975	5.73347832	6.24193423	6.3529393	6.37532204	6.70710306	6.70310705	6.63796447	6.327183
VARS2	4.19415028	3.80422962	5.05260679	3.71726475	3.4752529	3.16745726	4.01586963	3.87891483	3.87915954	5.10696673	4.4453181	3.29626625	3.45751209
TAF8	5.63713959	5.20569899	5.24857153	4.48991463	4.89927178	4.80340963	5.44889804	5.2992645	5.01248215	5.30174141	4.96823231	4.59840998	5.60915727
FAM8A1	5.37817269	5.78335096	5.24576013	6.1197482	6.4023237	6.72562389	6.53416372	6.72263615	6.61383846	5.1738358	6.64442918	6.67468405	4.92255979
C6orf52	0.83405883	0.00199284	-0.13844258	-0.00324938	-0.14076986	-0.13947593	0.27277575	1.21616662	-0.1968366	-0.1968366	-0.1968366	1.5732676	-0.1968366
FGFBP1	0	0.75831609	0.00124472	0.02141593	0	0.00069205	0	0.00158797	0	0	0	0	0
FGFBP2	0.0405526	1.21960124	0.05570566	0.59499663	0.40167808	-0.17035013	-0.1716679	-0.16864414	-0.11003884	-0.11003884	-0.11003884	-0.11003884	-0.11003884
CPEB2	3.43893484	3.82578673	3.6794386	4.07809408	5.04708587	4.94827796	4.88086833	5.09847273	4.62434286	4.2266487	4.08783507	4.8413376	4.93318642
FHDC1	1.72143111	2.03233133	1.53956218	1.87176118	1.6944356	2.06771028	2.38541534	1.65751601	2.25314276	2.65926413	2.98554662	1.93271768	3.01865458
TLR2	6.26413638	6.21824151	6.65346401	7.97077497	4.62050427	4.02198525	4.92115996	5.05625358	4.93192247	3.09119312	4.27562844	4.99336059	4.76223251
MYO7A	4.54290703	3.84264826	4.74980778	3.6188056	2.98882615	2.73357098	3.38181098	2.34296974	2.85895907	3.7088286	4.2803129	3.53297922	3.54163513
FCHSD2	6.65476737	7.12723466	6.53800517	6.63763023	7.16615898	6.75037299	7.25185494	7.00078422	7.1387872	7.06680012	7.10448237	6.68038676	6.89563513
ARRB1	5.05302085	5.61215352	5.43275256	5.44614185	4.76165292	3.42419112	4.60853737	4.33184114	4.98385814	4.91699148	5.29310789	4.33666305	6.09914108
SLCO2B1	6.01458968	6.00001521	6.12404576	6.14872355	5.10759886	4.14743828	5.16232708	4.3916458	5.10162108	5.71506145	4.89314103	5.3186787	4.10942012
ANKRD42	5.0149043	3.81875221	4.4579687	3.69672427	4.1779847	4.6396064	4.15915622	3.66880627	3.87776681	3.36508509	4.52342821	4.04999352	4.63414046
IL18BP	5.0188009	3.79328716	4.83799565	4.22537523	4.02589346	3.44612115	3.98283648	4.233413	3.44188777	3.86985733	4.19720808	3.57747404	3.97541638
NUMA1	7.98414544	7.1449239	7.89149556	6.49620961	7.15423488	7.21462232	7.42780717	7.51382661	7.18596409	7.86067562	7.30759158	7.3092645	7.320461
CCDC90B	4.52457097	5.63078261	5.01660017	5.78519733	5.55038892	5.781196	5.60325824	5.43416583	6.1706678	4.35189892	5.03841344	5.43114137	4.7737283
SYTL2	3.91495168	5.27629024	3.13736383	4.86869003	5.33979381	4.66582847	4.21691947	5.10041281	5.12041558	4.0206812	5.25396067	4.90198292	4.83117891
RAB30	5.20432429	5.90922365	4.87789457	5.61633949	5.75775025	6.41441024	5.94318973	6.24163331	6.5261535	5.88627602	6.05152249	6.41840945	5.60604231
CREBZF	5.61838545	5.73037611	5.38010676	5.66348114	6.05478464	5.52811909	6.42458025	6.66274123	6.19737945	5.15735198	6.54662494	6.36970948	5.31399316
LRRC32	6.60405227	7.33833288	6.86396183	6.82671052	6.27000594	5.18980587	6.37796754	4.8850233	5.81375045	5.91061694	6.78558992	5.28224094	5.83257405
PRCP	4.91204648	5.69655196	4.61363797	6.39855923	5.53365493	5.41957981	5.49604294	5.44840904	5.53671822	5.34312389	5.69149545	5.24158591	6.32845435
NARS2	3.6131911	4.04313395	3.53552451	3.35899917	3.52341184	3.53477114	4.9395306	4.20921711	3.41099117	1.7922997	3.64280366	4.10523609	3.73127438
RNF121	5.29301558	4.74281432	5.22692025	4.74250649	4.50590373	4.38290631	4.68793331	4.81071927	4.66172206	5.06608772	4.82178614	4.46349846	4.66053418
MRPL15	4.04957087	4.97587892	4.6231195	6.03488928	4.35157913	4.22924145	4.72019797	4.66418155	4.89854353	4.80123109	3.40844524	5.10825928	5.86940594
PI15	2.35900746	2.08951943	4.76537213	8.92384966	2.95497895	2.23268486	2.20949848	4.14660612	1.65028134	2.13441823	2.61647586	3.25966051	2.16230756
TTPA	0.87923569	0.78260477	0.86450386	0.71513031	0.94646069	1.56041256	1.11729583	1.55432496	-0.49954755	2.77749101	3.13926397	2.17565956	3.45809203
GGH	2.3420937	3.42802386	3.28982894	4.78618325	2.66921231	3.13454958	3.47994948	3.52506247	3.63311256	4.00975714	4.26292937	2.18112433	3.23886823
SLCO5A1	3.23266559	4.27314073	2.59932298	2.68024227	3.46997075	3.45946262	3.80926544	2.80798031	4.14595584	4.03889867	2.14668849	4.51592222	3.55854658
SULF1	9.35117085	8.62095964	8.49859699	9.58740313	8.54229922	7.2290222	8.91448032	7.68969121	8.33318422	7.9990127	8.72738151	8.10517243	8.81547235
TGS1	4.89574067	5.77623278	5.0857097	5.14961284	5.28912663	5.96761686	5.08480327	5.46285773	5.56070917	5.32744838	4.28189504	5.16226065	5.31174016
SDCBP	5.83251819	6.22724492	5.64345095	7.02587147	6.54479332	6.59696574	6.62405949	6.5024976	6.55886206	6.00094042	6.55722026	6.61016191	6.2574608
NEK1	4.91765237	5.4398302	5.3920643	5.87912809	5.77588718	6.04399702	5.83664036	5.6830642	5.99227005	5.54399049	5.75350259	5.87432461	5.10693522
DDX60	4.51103763	5.99638696	4.86932319	6.06009056	5.68357251	5.89079123	5.2823626	5.42457071	5.94269952	5.16484088	5.75819652	5.22701136	5.99137822
SORL1	6.19918353	6.07090826	6.01643245	6.75108733	5.89465016	6.36647931	6.03989325	4.52283661	6.59546852	6.50288754	6.34225934	6.05033001	6.61224457
BUD13	4.94599593	4.45138306	4.88824342	5.08089026	5.20332757	5.46462062	4.39254928	5.10956403	5.08780283	5.24757857	4.34356142	4.87628369	5.30187584
TRPC6	1.73895164	2.39168684	1.78859062	3.48242468	3.0127061	3.84595687	3.17718999	3.23454218	3.28767375	3.07521034	3.20285103	4.07834499	3.12521352
MMP7	0.05301793	0.89859559	-0.08514927	3.12817692	-0.08695326	-0.08595027	-0.08695326	-0.08465178	3.72261436	-0.06955467	-0.06955467	-0.06955467	-0.06955467
C11orf70	2.97592833	2.93237854	2.60388033	3.49368779	2.60585492	2.29545792	1.7717358	2.89647849	3.11105574	2.24019382	-1.32363122	2.10785431	-1.32363122
DCUN1D5	4.95166746	4.88394572	6.15438642	6.71819759	5.99719127	6.02649988	5.82454196	5.91345582	5.64042531	5.15547425	5.97841864	5.61442963	4.64174384
YAP1	7.36279751	7.63167727	7.18958733	7.22274906	8.41146468	8.19281138	7.96690032	8.19476482	7.46552784	7.45336359	7.84903271	8.18039052	7.09464863

TRIM29	1.01156524	3.50586812	1.46720515	1.02128039	2.33347567	1.6824067	1.24800215	1.95127664	3.9940187	4.25826333	2.64956948	4.63141183	3.99563547
SLC37A4	3.58118467	3.27001327	3.65211955	2.79837387	3.00864823	2.37294111	3.3288641	3.33123308	2.99113726	4.57922775	2.53521299	3.53643256	2.86608986
POU2F3	-0.11746598	0.05384977	0.05926413	-0.11746598	-0.03392964	-0.11610572	-0.11746598	0.1514317	-0.06224322	-0.06224322	-0.06224322	-0.06224322	-0.06224322
RDX	5.85878385	6.4233588	6.30773419	6.92371522	6.89564466	7.10996595	6.99432399	6.9303026	6.86807518	7.03603825	6.07345747	6.56642697	6.91983519
PPP2R1B	5.47938697	5.42140569	4.75462581	5.56115072	5.22438173	5.04567218	5.34707179	6.22111307	5.18866738	4.84428843	5.32676992	4.32715743	5.76009904
FDX1	4.09423606	3.78235032	3.90259938	3.46544469	3.94500739	4.80369826	3.81555381	4.26910818	3.85783924	3.36801458	3.6130913	4.83356431	3.53598095
C11orf1	3.45252052	1.96049281	3.11736201	2.76261938	2.81209869	3.30825968	2.42248904	3.07009183	3.5319541	4.20782055	2.57755109	2.66623181	3.09853285
FXYD6	2.54176981	4.54198398	2.75877064	2.49045395	3.29647114	1.8099044	2.15398294	3.95706732	4.00439226	3.72318057	4.27297288	3.70628684	4.7581291
ARHGAP20	3.17257448	3.78445137	3.34228017	3.80978836	4.06387832	4.54284678	4.18544191	5.13550258	4.63541403	4.18590673	4.14523132	2.33990039	4.51279002
FXYD2	-0.36073614	-0.06475723	-0.07848299	-0.50579998	1.10253221	-0.04037463	0.03382411	0.962524	-0.06347602	-0.06347602	-0.06347602	-0.06347602	-0.06347602
MMP13	0.36866799	0.68095056	0.78285842	1.88446436	0.36866799	0.37004118	0.36866799	0.37181892	2.80360563	-0.25921017	1.38734601	-0.25921017	-0.25921017
TMPRSS13	0.14503983	0.18633014	0.06010234	0.10609616	0.55144553	0.22254757	0.19199225	0.23394585	-0.09903871	-0.09903871	-0.09903871	-0.09903871	-0.09903871
CASP1	4.35267901	4.80392876	3.53169777	5.48226808	3.76855024	3.21150868	3.89970343	2.7465974	5.02913698	2.84757309	4.99275389	3.66035044	4.48517426
CASP5	1.04510509	0.28256891	0.69354714	2.20924134	1.06264275	0.04953253	0.41441636	0.02122724	-0.40660035	-0.40660035	1.7258584	-0.40660035	3.71301714
ALKBH8	4.1642943	4.67659172	4.21676896	4.30311596	4.83830038	5.55565164	4.38554959	4.73266353	5.02353238	4.9537997	4.37234129	4.82798588	4.81506624
MAP2K5	4.44189122	3.96050692	4.69788483	4.43391228	4.76998191	4.41967656	4.9153739	4.8962619	4.5036014	5.00218576	4.68119765	3.9741939	5.18550722
UNC13C	4.83374803	5.20557927	5.36161019	4.08718385	5.75474445	6.05227707	5.50480545	6.77401633	6.05131349	6.26299896	5.53264259	6.33650139	5.07885391
CTDSPL2	2.69195754	3.56907451	2.83618807	4.18101123	3.93123038	4.2678692	3.67490958	4.03528812	3.96756343	4.25207684	2.65824264	4.0437425	3.18505964
SLTM	5.41835767	5.61108986	5.81700393	6.29789193	5.94010128	6.12470454	6.33501255	5.70990233	6.53729846	5.86918448	5.43511614	5.25038904	6.01702673
THBS1	6.84085249	8.5439428	7.14600183	9.79590548	8.93645356	6.29351333	5.52751321	6.01749324	7.68853896	4.91085338	8.72947347	7.23506463	7.97791464
MAPKBP1	5.76788268	5.23956191	5.86636393	4.83785057	5.61768816	5.1089423	5.74375277	5.70507935	5.23277424	6.00907267	5.9001422	5.93980426	5.31271648
NUSAP1	2.6805373	1.8417538	4.65738803	5.68671568	1.95632254	2.3517392	3.40502909	1.97746689	2.459858	3.16593678	2.67404693	2.04503933	3.32376419
NDUFAF1	4.25878682	3.42636734	4.40599397	4.17267825	4.07352123	4.63308816	4.65999605	4.2424759	4.40668533	3.67787797	3.72197897	4.07075932	4.59186517
KIF23	1.86768114	1.76906332	4.35204718	4.78203652	2.73739809	3.4676388	2.65802106	2.9641111	3.68649181	1.29980393	3.39932229	3.39284028	3.57863716
ITGA11	8.07900023	7.47618203	8.03054583	6.79563646	8.09249991	8.16990542	7.94537994	7.96808213	7.52169221	7.73658067	8.24968503	7.39239866	8.13817016
HAUS2	4.68298386	4.50292814	5.09350028	5.49162725	5.10929337	5.24081947	5.26055593	5.08817027	5.15836353	4.5697054	4.68177604	4.77762338	4.92540396
RTF1	6.48791763	5.92811993	6.09235539	5.75816102	6.09720361	6.17467151	5.98412936	5.76830673	6.00774069	6.27749851	5.68639095	5.9046544	6.28106602
PARP6	5.4038319	5.47692065	4.99968975	5.46094248	5.59300871	4.78728748	5.57962731	5.79575006	5.16075116	5.27665434	5.41822279	5.27053887	5.52644062
RPLP1	7.1113272	7.55323681	7.83749875	6.69757378	6.9340903	7.47875121	6.96434136	7.14128873	7.5295468	7.97919304	7.48254025	7.87316465	7.24603181
PAQR5	0.7653452	1.49181924	0.02371208	0.13541004	0.58579156	-0.08482834	0.00458126	0.06923359	-0.03802848	-0.03802848	-0.03802848	-0.03802848	-0.03802848
LRRC49	0.2041358	0.27566811	-0.30821448	0.51196065	0.39656312	0.39937253	0.69007439	1.15532535	0.25023677	0.25023677	0.25023677	0.25023677	0.25023677
TUBGCP4	5.03203026	4.59389438	4.57420107	4.42339255	4.96724635	5.12717283	5.10336484	5.13966041	4.58747098	4.99754084	4.75976311	4.85122242	4.7505268
ITPKA	0.03056469	-0.38038076	0.47199514	-0.21204569	-0.38038076	0.17373389	-0.06609292	0.48820824	-0.18291824	-0.18291824	-0.18291824	-0.18291824	-0.18291824
UACA	6.4718139	7.69136271	6.17205768	7.69622181	7.18943193	7.47891253	6.71861428	7.3362519	8.50237225	7.04495048	7.47821565	7.11277706	7.36411732
SMAD6	4.96486714	5.09583796	4.95803106	3.70038069	4.80939253	4.29433545	5.79109699	4.88410633	4.59208653	4.13517347	4.7785282	4.06506193	4.63737151
PLCB2	4.56438413	5.09548214	5.02851974	4.90139204	3.55611837	3.00059831	3.59685831	3.22004941	3.59168845	4.3494172	4.7665799	4.16009761	4.10293064
TMEM62	2.55476077	2.78336755	1.89191737	3.59783869	4.11076742	3.01413487	3.64989152	4.08201323	3.31667656	3.35829879	2.92375012	3.54654913	3.38708864
PAK6	0.01731255	0.15775708	0.27203524	0.0583767	0.14650638	0.31049408	0.07692923	0.28933834	0	0	0	0	0
ADAM10	5.47296417	6.01314748	5.57084481	6.77731093	6.16434804	6.26080619	6.22121203	6.09214857	6.31073752	6.27044854	5.9149943	5.83927175	6.34097693
DUOX1	0.16188535	1.73444286	0.17608014	0.15078508	0.67365118	0.23626743	0.22689584	1.28562603	0.4590385	-0.12781229	-0.12781229	2.13564171	4.13392049
STRA6	0.50843938	0.49885764	0.74266579	1.14442705	-0.10632891	-0.10492981	0.26563447	0.14574527	-0.3669912	3.75940746	-0.3669912	-0.3669912	0.85064893
CYP19A1	2.51327244	2.00552697	1.5853808	2.21455206	1.65596692	1.38414796	2.46220664	1.28931706	-1.08927471	-1.08927471	2.0902138	-1.08927471	1.10339796
ZNF280D	5.73822548	6.40267766	5.70295512	6.82724276	6.90991409	6.96159389	6.8623969	6.92757189	7.14410977	6.65165096	6.64741818	6.81063276	6.36803261
SEMA6D	2.53225176	1.83340669	1.13604238	1.32859883	2.1388142	4.12955576	3.44274789	3.10189691	1.20320669	4.63365843	2.20309487	2.23922383	2.62196305
RSL24D1	4.62256841	5.87597631	5.00954846	5.80028838	6.1747327	6.48726643	5.9404046	6.3108576	6.48821086	5.94208353	5.51872182	6.12568813	5.07775371
SPTBN5	0.53237332	0.41654119	2.04220819	1.39905503	0.4721177	0.44346392	1.0999223	1.29209489	-0.20195618	-0.20195618	2.79381966	3.02749828	3.68180524
GCOM1	3.0005087	2.94973616	3.07021972	3.21669971	2.98100169	3.48250023	3.52431052	3.92501299	3.81530441	3.72864747	3.33811082	3.40346147	7.76803261

GCHFR	3.0606417	1.1303276	1.5150163	1.34484106	1.88870746	1.87888207	1.09886628	0.15274244	1.28855016	3.31247562	1.60566953	1.5781408	-0.45083647
BCAR3	5.41992101	5.90484954	5.50591682	4.12064583	5.08604419	5.48050027	5.14491235	4.50163468	5.0306458	5.40196597	5.44017025	5.09296781	4.9259274
TTLL7	6.11384048	6.49946583	5.98510754	5.9763756	7.31434151	7.34978288	7.36105581	7.65640271	7.14594015	7.52563298	6.7585753	7.48250417	7.13982251
FNBP1L	1.94491511	2.10547486	0.84981296	2.63106023	3.55279166	3.39755372	3.14447634	3.35283292	3.72989658	3.72566857	1.71907766	4.1700502	3.39485069
GTF2B	5.42486177	5.09813759	4.81411378	5.68112882	5.64906827	5.89900097	4.78704771	5.16265257	5.16886035	5.71926478	4.56561217	5.38049621	5.16492549
RABGGTB	-0.3121144	1.1238238	0.53147214	2.2323209	1.47781135	1.03540577	0.79354092	1.17466225	0.32382337	0.32382337	0.32382337	0.32382337	0.32382337
IFI44L	4.47953092	6.19386055	4.57117717	7.84184513	5.43174314	4.94069432	4.55236605	4.64424375	6.5170439	4.64203635	6.41518201	4.0936544	7.07657465
GIPC2	1.08771554	2.26892935	1.27530225	1.97194922	2.04115804	2.46433778	2.85766733	2.82839855	4.09510686	-0.77803655	3.24470745	1.66644612	2.50348799
ARHGAP29	4.71716915	5.9611953	5.30967156	6.99165569	6.15257876	6.12412944	6.1129764	6.73583999	6.32267476	5.67431362	6.52399855	6.16385879	6.4544492
IFI44	3.58420468	5.97829804	4.85337605	7.22532136	5.31845101	4.27151598	4.31530248	4.64672058	5.95092025	3.0593581	5.50674072	4.01929522	6.5781163
SLC44A5	2.39841858	2.18616082	2.43674477	3.17441804	2.55643971	2.23945384	2.19708911	3.93656822	3.16034246	3.65123976	2.62222974	2.60088402	2.80376131
CLCA2	1.03135392	0.47139656	0.47397415	1.19863143	0.47139656	0.77125332	0.86091028	0.47468497	2.95456558	-0.3622919	-0.3622919	2.89999008	-0.3622919
DNASE2B	1.39636119	0.03427641	0.03669257	0.03427641	0.03427641	0.45843214	0.69095593	0.03735887	-0.13249097	-0.13249097	-0.13249097	-0.13249097	-0.13249097
DBT	5.20423869	5.09032809	4.96688563	4.79771958	5.6354901	6.1168528	5.68920287	5.86980291	5.43805655	4.89808712	5.33743493	5.61322999	5.39955807
IFT172	5.65173653	5.43492898	5.05647222	4.63541841	5.3305531	5.0275489	5.63502326	5.67478275	5.33641177	5.25223125	5.71437335	5.32587802	5.66227999
CGREF1	1.96404488	2.81212085	1.73247108	3.82813996	2.16895626	1.30209146	1.72963147	2.45534619	2.99292957	2.68939856	4.53478662	3.47153312	0.75441907
HADHB	6.4623855	6.74440213	6.24642732	6.8886727	5.85782449	6.26464997	6.87581251	6.02124256	6.02757788	6.46159531	5.8693286	6.5006501	7.63733027
KHK	2.8290446	2.6269893	3.28295865	1.67710336	1.98947859	3.34492526	3.05428462	2.70421019	1.43576038	4.35710521	0.17083899	2.4333172	3.44090153
ADCY3	5.85663744	5.19832503	5.59923906	4.98744447	5.05512879	4.53605687	5.32626146	5.59044723	4.70647694	5.57247961	5.77984363	5.33319274	5.6037734
PPM1B	5.69689991	6.06823308	5.62905909	5.79666989	6.89224278	6.91146185	6.44410895	7.13266471	6.52253535	6.53524286	6.58950047	6.75253172	6.91048532
PNPT1	4.18163972	5.29133767	4.72873308	5.69164927	5.40356693	5.52728479	5.51746391	5.12322944	5.18162665	4.02447179	5.26690228	5.09925586	4.95900473
DYNC2LI1	3.99565499	4.64885585	3.82464527	4.62300083	4.96058914	4.8047809	4.58087362	5.30589966	4.99331349	4.18226287	4.84054677	4.75164354	3.97393926
LHCGR	0.01189427	0.01189427	0.0142866	0.18266648	0.10924233	0.21093216	0.01189427	1.41328671	-0.09083286	-0.09083286	-0.09083286	-0.09083286	-0.09083286
THUMPD2	4.64307672	5.51229526	4.65569	5.03127689	5.35360926	5.5559139	5.39153012	5.74773115	5.73140112	4.95508053	5.55650959	5.88272133	4.95620856
CYP1B1	5.58439729	6.46657687	4.17305112	7.05708517	5.27811293	2.73650254	5.91346215	3.68084284	4.40648108	3.50382954	4.46637576	3.27698161	4.85244317
RAB1A	5.90850471	6.57121601	5.91875917	7.21049666	6.95446159	6.81044005	6.99151746	6.9812192	6.51176339	6.12183453	6.33875222	6.77655598	6.53130889
ACTR2	6.74418897	7.70888458	6.9274164	8.17236137	7.98273585	7.88092054	7.98014586	7.76461914	7.64063851	7.08757358	8.15910383	7.77117043	7.01908331
PREB	5.2705538	4.93562102	5.630096	5.37615817	4.8100962	4.72460514	5.13123798	4.79736801	4.96748086	5.53609851	5.23895742	5.26574799	4.74914665
SLC5A6	4.50813263	3.15100405	3.738863	3.94208018	3.86417463	2.49185985	3.68033944	3.47654524	3.40304655	-1.05958276	4.45835569	4.04151964	4.39319732
PREPL	6.1537803	6.78320064	6.21503832	6.81881591	7.07454656	7.77190594	7.28364277	7.24451589	6.9353203	6.89543735	6.89688793	7.42767732	6.81827141
SLC3A1	0.28552181	0.59223152	0.36440562	0.44453935	0.46762063	0.91997967	0.58178849	0.90515223	-0.17460089	3.53843588	-0.17460089	3.36058421	-0.17460089
EMILIN1	7.60704786	6.72015912	8.4304151	7.42465259	6.14077096	6.1157114	6.785675	6.22602663	5.78324223	7.42683796	6.83379768	6.93167636	5.85988973
FBXO11	5.83408256	6.54066691	5.90419016	7.08413236	7.03103508	6.99707865	7.04740679	7.07298241	6.75767387	6.09033626	6.4143775	6.57798853	6.60934631
SIX3	-0.09755584	0.21524601	-0.09499072	-0.09755584	-0.09755584	-0.09612967	0.09890271	-0.09428334	-0.05957447	-0.05957447	-0.05957447	-0.05957447	-0.05957447
CENPO	3.416824	2.95319209	4.35460357	4.65453607	2.52792505	2.37137581	3.15323156	2.90948235	1.88791121	3.19472349	3.92375364	2.6132898	2.58737512
LRPPRC	6.01780218	6.84681137	6.28050035	7.10015997	6.86448398	7.05501212	6.97447016	6.62598251	6.5879021	6.80244023	6.32570233	6.63170387	7.21137754
TRIM54	0.93888396	0.06806244	0.42900347	0.09314553	0.33113217	1.10276868	0.06806244	0.21727156	-0.28073479	-0.28073479	-0.28073479	-0.28073479	5.96812876
DTNB	4.26394045	2.71731483	3.35688816	2.73524555	2.82401793	2.05186572	3.03853881	3.1485686	2.99921422	2.34041931	1.53954366	2.07549834	4.17654494
ACTR1A	6.41090503	6.13656556	6.56015343	6.36150977	5.8780723	6.10435505	6.13923276	6.00961479	6.0642044	6.60810338	5.98297248	6.33007279	6.30496127
CYP2C8	0.8541178	0.30093433	1.10809355	1.14946715	1.69439074	0.92849646	1.57708728	1.27907186	1.0529356	-0.4525623	2.29145932	-0.4525623	-0.4525623
MYOF	7.15492023	7.85992853	7.24415276	7.8910379	7.97152466	7.35951874	7.56518418	7.57548336	7.53226331	7.838496	7.9789204	7.22610194	7.81636755
LOXL4	4.82483569	4.89829357	4.67549882	3.44984398	5.00803343	5.16037184	4.77059852	5.30097211	5.00419642	4.52242327	5.57222347	4.9396765	4.22823403
STAMBPL1	2.48214362	4.02325253	2.53496286	4.05272851	3.4138367	2.84882201	3.06105315	3.69643693	4.31960297	3.80975101	3.72067868	2.90984945	4.88647255
CH25H	2.89510135	2.00440866	0.9440147	3.48202259	1.2506425	0.65158045	2.26128123	1.42200897	3.96606953	2.87527483	2.83889361	3.47716723	3.2382948
ATAD1	5.67153601	6.4424509	5.51120588	6.191828	6.217264	6.5551447	6.28482604	6.25339664	6.51332673	6.20688387	6.56735474	6.34961696	6.09312634
BTBD16	3.01412373	1.5548604	2.25715674	1.87041114	3.49431887	2.77306276	3.78834122	3.5123065	1.47261273	3.92729591	3.71317299	3.82433491	1.78573995
KIF11	-0.28140271	0.04840064	2.14869338	3.49183556	0.01456923	0.27634387	1.04893273	0.35314859	-0.27878728	-0.27878728	-0.27878728	-0.27878728	-0.27878728

CUZD1	0.5302074	0.24647866	0.44599562	0.27260743	0.78425709	0.53907577	0.29959087	0.31272775	0	0	0	0	0
TACC2	3.3207652	2.85449236	4.18085746	0.39878726	3.10234392	3.56775913	3.86598342	3.74108703	4.28584918	4.51607543	3.15312767	4.24750857	4.34029044
DUSP5	3.43571955	4.09395459	3.99638878	4.79966238	3.19231876	2.78277849	2.08686919	2.68862132	4.30395972	3.62960836	4.33657583	2.51211833	4.4751338
CALHM2	6.01871091	5.42241871	6.18827279	5.33507628	5.27503132	4.96868437	5.80352862	5.40939599	5.42379279	5.40886567	5.96539068	5.59164841	5.31596317
ARL3	5.02339797	4.9489396	4.23001521	4.30508252	4.43913743	5.25548106	4.73414682	4.86255248	5.10079649	5.07086041	4.69653625	4.03921992	4.2483981
CEP55	1.71178389	1.3903179	4.31148767	4.93045285	0.99768383	1.1642235	2.35547093	2.01775217	2.73529097	3.1906246	-0.03494529	0.95216779	3.42669021
KIF20B	3.56309016	3.7505819	4.27703134	5.40332447	4.02152572	4.52980023	4.16519063	4.22579118	5.43165845	3.68706266	3.82348639	4.24101272	3.96557896
ENTPD1	4.87525797	5.94258552	4.64758725	5.38274497	6.635134	6.3553289	6.41747806	6.50207376	5.98777465	6.50850005	6.48679497	6.75260488	6.50502494
EXOC6	3.81551789	5.17379148	4.10969898	5.1690059	5.23269206	5.41445897	5.58811997	5.62688986	5.68718754	4.55986563	4.90624979	5.68933244	5.10479553
PLCE1	4.81288772	4.81082442	4.44773919	3.32789694	4.59560241	5.69259864	5.16511971	4.17971212	3.66182525	4.68401411	4.33375392	4.07918651	6.1517627
RBP4	5.03424302	5.10750438	4.81501152	2.98179286	3.3010527	5.02396036	3.09707534	3.80892787	4.27868658	5.32399833	3.83318759	4.69815872	5.09852982
DBR1	4.57979295	4.68003156	4.94708517	4.82998747	4.54818037	4.97227058	4.79249208	4.21382749	5.01953446	5.42689364	4.43312565	4.63929822	4.61168718
DNAJC13	6.0178817	7.09948337	6.11515888	7.20981451	7.21041051	7.28737473	7.27496515	6.99478666	6.92982206	6.81513854	6.92213137	7.16512815	6.7671475
ANXA7	6.74050489	7.07711656	6.64653938	6.46379114	6.65601655	7.13537625	6.6325761	6.49532369	7.00706766	7.00917218	6.76032543	6.88547839	7.0776382
FAM149B1	4.09052529	3.839339	3.79414552	3.9448049	4.28000383	4.23923058	3.84607751	4.14633008	4.0501247	3.47293174	3.64832028	4.3732487	4.1099819
ASCC1	5.19202948	4.60764184	4.46917071	5.34071445	4.77150889	6.0805305	5.42044974	5.03383376	4.96673367	4.76563273	4.42046682	4.79108515	4.50290832
ZNF365	4.77244612	3.87980665	4.59460443	3.94981875	4.92993784	5.17211504	4.22218573	5.34982348	5.05748369	5.16813946	5.13381344	5.74202669	4.34010248
OIT3	-0.18431295	-0.17166609	0.31038305	0.94484211	0.09830303	0.29530401	0.03870661	0.51123965	1.65643471	-0.0691337	-0.0691337	-0.0691337	-0.0691337
ADAMTS14	0.54088179	0.49435825	1.35376314	3.80204329	-0.06550546	-0.15055179	0.1298292	0.08531214	-0.37071571	-0.37071571	3.56228142	0.8186739	-0.37071571
RPS24	6.48617299	6.24301596	6.60673591	7.58010311	6.60831284	7.43701276	6.35577378	6.3965622	7.85729135	7.89337557	6.38797831	6.34791458	7.04583016
TET1	2.88764712	1.5912524	1.65710509	2.104078	2.64598033	4.20963292	3.06961453	3.65062793	2.2924651	3.53494335	2.09574028	3.29180041	3.39382019
DNA2	0.59685838	0.59069553	1.36380082	2.73442338	1.04350515	1.47819269	2.60003024	1.26589937	-0.48417212	-0.48417212	2.91312697	2.29494442	-0.48417212
AOX1	5.63881498	6.65970091	4.26392142	4.25276275	5.17868411	5.22454113	5.52461122	5.94891893	4.81396924	5.59069578	5.04226231	4.99520986	5.40054546
ATIC	6.03333102	5.74437052	6.01774386	6.16255066	5.14259695	5.57919741	5.1327034	5.17244456	5.33003437	5.64237449	5.81339922	5.34500107	4.79964729
SMARCAL1	5.00021466	4.88295893	4.83561196	5.03048454	4.37421313	4.41114683	4.50442608	4.45355619	4.75682443	4.99556638	5.09398716	4.92809634	4.76983441
BARD1	4.79397339	5.24980321	4.64735271	5.58724556	5.40824326	6.00711197	5.76815144	6.42441483	5.45109404	6.61002569	5.48065939	6.38647133	4.93234322
STAT4	2.89998795	4.26882451	2.03337473	3.80181656	3.04417606	2.02440259	2.80563672	2.37022572	4.34059537	1.71302756	3.88002595	2.67350165	6.28287309
MSTN	0.26100967	0.60662978	0.80307723	0.70573968	1.53433649	1.91714661	1.51886737	1.91397275	1.82832243	-0.34517475	-0.34517475	-0.34517475	2.34290223
ASNSD1	4.99482258	5.79675069	5.63523645	6.23498546	5.955854	6.32226766	6.36944473	6.61122464	6.46119891	5.49189188	5.81458964	5.56707006	5.077361
METTL5	4.76840248	5.30043202	4.93485978	5.03576702	5.93086914	6.00788025	5.48746	5.52045421	5.5939134	4.67669335	5.23209046	5.29595717	5.64799885
SSB	5.07684607	5.31503795	5.28672024	6.05857249	6.00907281	6.79130022	5.83376923	5.75632566	6.54104633	4.60903815	5.4391368	5.62791249	4.68782001
NAB1	6.1676245	6.50170635	5.77759382	6.48051915	6.50392228	6.96331218	6.76113538	6.79286161	6.96728096	7.18104971	6.88467544	6.97632679	6.20676127
CDK15	0.65693092	0.82766388	0.62653186	0.73676346	2.28917324	4.20582328	2.12196079	1.71418584	2.05107086	1.81694603	2.05107086	-0.64524458	3.20317054
PPIG	5.73736244	6.09276372	5.68614188	6.29804649	6.58086955	6.78825164	6.53736067	6.51213796	6.99723024	5.99299322	6.09791122	6.35288289	6.09302141
FASTKD1	3.12966737	3.96807183	3.65008483	5.03585554	4.01437597	4.53509469	4.92177916	4.39451461	3.58832039	3.69879652	4.198016	4.64242053	3.43768926
MDH1B	1.0552859	1.80344591	1.30935003	1.01226533	1.23617181	2.26616936	1.50220314	0.80762262	3.05011339	3.00694159	4.03969804	0.90544367	2.01390996
HECW2	4.44948077	4.62829795	3.71818297	5.52878938	4.76691006	4.21785541	4.42380273	4.30435554	4.33809197	4.52858869	5.24672543	4.73791127	5.23480457
IDH1	5.2088659	5.67249278	5.17265731	6.46662663	5.14427404	6.00340751	6.27715461	5.2477142	6.20071241	6.06578225	5.87203816	5.46799217	5.94349492
OLA1	5.20727319	5.77032216	5.83986786	5.90058415	5.76367673	6.4768452	6.09464466	5.84974	6.12040226	6.23337252	5.36514477	5.81630229	5.75991044
CIR1	5.26252185	4.86076872	5.0152226	5.49497031	5.56820243	6.06162851	5.18980551	5.6314658	6.29789995	5.90742871	4.99377058	5.65134008	5.25186088
SSFA2	6.92634405	7.09453345	6.98238065	7.52940119	7.42968885	7.1886922	7.51421082	7.66972196	7.28962348	6.99637315	7.54285822	6.9111598	7.295961
CHRNA1	1.1371886	-0.01681723	-0.01503689	0.09505763	-0.01681723	-0.01582739	-0.01681723	-0.01454593	0.03978162	0.03978162	0.03978162	0.03978162	0.03978162
FAM117B	4.97299109	4.62944394	4.26621206	4.08653102	4.67477851	4.98204523	5.31944557	5.37001043	4.85110258	5.35831658	4.60506158	5.05834672	4.78625015
WDR12	4.55812136	4.63373715	4.86637439	5.13375394	4.31698354	4.70201311	5.0836395	3.43589021	4.7550864	3.96767395	4.33206335	3.63164109	4.20171345
ABI2	3.36569374	3.6147446	2.97538027	3.47170054	4.06875475	3.89266407	4.17875489	4.18654468	4.05279493	3.69480654	3.40134428	3.06089633	3.81788889
ITGAV	7.08574159	7.60867153	7.58097128	8.46962474	8.17984394	8.09885091	8.07502301	8.38849484	8.19128331	7.8702124	8.28916725	8.1525947	7.74287345
SLC40A1	7.04274361	7.51946154	6.18403225	6.85088046	6.33151948	7.60619334	7.59541276	7.01414107	7.55510136	7.77052273	7.37081918	7.22753643	6.73923903

SLC35A5	4.6297564	5.3573143	4.36293677	5.6448835	5.68387357	5.65939031	5.73127007	5.69882494	5.48223766	4.87928494	5.73392008	5.9823946	4.32397909
DIRC2	5.08226049	5.57434396	4.21470493	4.99918834	5.21581338	5.10292917	4.95990685	5.23502354	4.86153993	4.56797482	4.71582178	4.97406511	5.34628421
SENP7	3.77311134	5.2873537	4.02439544	5.42213955	5.41763628	5.95056954	5.55022711	5.68067903	5.4605648	4.56511765	5.70268409	5.74432142	4.0119053
COX17	3.98831518	3.07548939	4.09491474	3.9158416	4.72646308	4.76648273	4.11602923	4.62763484	5.17031918	4.69621174	4.33373507	4.89755065	4.16674899
PARP9	5.7788268	6.36945743	5.43477199	6.63833324	5.98960296	5.60518108	5.6271835	4.81600461	5.83779939	5.30693878	5.72167627	4.92686374	6.50253022
MNS1	2.15447211	1.87311306	2.66015727	4.10831039	3.07888378	3.23817553	2.27837724	1.99512279	3.51881515	2.03545556	2.95803883	2.3379513	1.36540599
USP8	4.67805781	4.7974164	4.6458368	5.48385116	5.34803557	5.95058887	5.2800989	5.42463826	5.48383592	5.23077833	4.41232575	4.97727678	5.67497772
SECISBP2L	6.71295233	7.13455093	6.4777455	6.98961493	7.59520833	7.53298212	7.40803262	7.49741714	7.28372267	6.59652358	6.96762054	7.27997894	6.93735696
TMOD3	4.31270191	4.14564139	3.78232637	4.76470769	4.30076704	3.85607681	4.22452564	3.979492	3.95840139	4.61670388	4.1677104	3.03223286	4.60653032
SPPL2A	4.90399393	5.46622363	5.25841256	5.87908597	5.95081868	6.02316761	6.11122358	6.0506914	5.5419524	5.24674555	5.69434566	5.88012907	5.30251616
GLCE	0.83028365	1.18494847	0.22858654	2.3711245	1.90203451	3.16197446	1.25169612	1.50477286	-0.03166731	3.85555786	-0.03166731	1.9886824	-0.03166731
SHF	4.49079244	3.6274295	4.24194463	2.56719024	3.51840486	4.12252188	3.98185646	4.13608035	3.78373087	3.76460023	2.85964784	4.3337578	3.20109961
APH1B	4.5483124	5.16380814	3.8669251	4.52014898	4.87784936	4.75325812	4.73271224	4.89952802	4.21159946	3.94365339	4.4502119	4.87490742	4.74242381
CILP	0.94802162	5.81415093	0.82527483	3.2356364	1.70813477	2.34820448	1.01229242	1.83309744	-0.48953961	4.13928491	3.36457166	2.92337733	6.04209365
PARP16	4.68367142	3.67496456	4.01608254	3.54811637	3.92240941	4.0391408	4.04822803	4.25458777	4.37724044	4.39652491	4.68749879	4.15320868	3.63095896
PPCDC	4.40662748	3.57329731	4.75211997	3.31315979	3.50232493	3.22536583	3.71326436	3.88595087	3.90360608	3.98799234	3.3500906	3.90253464	3.583295
SEMA7A	1.06065914	0.26730981	2.33744925	2.2607889	0.12674892	0.38325317	0.74905206	0.34010201	-0.23503341	2.2877736	-0.23503341	3.17297491	0.83860271
UBL7	5.41343565	4.55005448	5.39547116	4.3850393	4.44458731	5.0597649	4.52067	4.55885881	4.7927315	5.18617181	4.80357606	5.03786214	4.08493699
ARHGAP24	0.47363734	1.28369195	0.54158034	1.00660835	1.00899589	0.87461291	0.15629465	0.58929732	0.63038259	0.63038259	0.63038259	0.63038259	0.63038259
FAM13A	5.7107603	5.81146979	4.75543767	5.7158706	5.29002611	6.01083071	5.54167001	5.45738071	5.60583373	5.34134192	5.25264469	5.00847027	5.3248636
HERC3	5.23822374	5.82787474	5.1845108	5.42071689	5.68482414	5.87116678	6.28615404	6.08245649	5.73345057	5.4990348	6.00156848	5.99095791	6.2598939
HERC6	4.01349065	5.16725351	3.90564896	5.45227382	4.28256527	4.33700581	3.40489603	3.52763877	4.90041416	3.6383264	4.71819296	3.30189532	5.42548979
HERC5	4.91856791	4.81629562	5.20727276	4.86615423	5.21882648	5.76054887	5.47466532	5.02565108	5.6579269	4.94135471	5.26317302	4.75529876	5.25745336
PCDH10	7.17075858	6.12645831	6.66884327	5.4715268	7.74642182	6.75564257	6.38069458	6.00792152	7.50665739	5.97210858	7.85045172	6.01700552	7.3037998
AP1AR	3.61976688	4.36730486	3.95605622	4.81450954	4.91287262	5.39416592	5.39928437	4.63377557	4.78326785	3.4434323	5.28315917	5.39694047	2.99128695
COPS4	4.59887012	5.44020435	4.68485193	5.37374307	5.36835526	5.84262707	5.37089917	5.27057571	5.59343499	6.02565263	5.10354292	5.07863116	5.4899777
HNRNPD	6.00091897	6.25788605	5.95271944	6.79386619	6.72204964	6.55701566	6.63222377	6.50747174	6.63609457	6.60031888	6.44905203	6.27814527	6.36697843
PRKG2	1.34568628	1.50401202	1.43992808	1.49748485	1.6104497	1.34713475	1.3801702	1.34900993	4.08132682	1.88570419	2.53133087	3.0801541	4.50038316
RASGEF1B	4.79844674	5.19622713	4.55296167	6.47790319	3.94525315	3.43361701	4.17966007	4.00605132	4.34292643	2.62362641	4.22819734	3.18925523	4.42673998
SEC31A	7.39639856	7.57548301	7.1886373	8.17298254	7.75382765	7.61980143	7.63614669	7.79509817	7.71233291	7.81519996	7.87732497	7.75879933	7.91843892
FGF5	0.70205558	0.98028211	0.81214062	1.11289759	0.55233753	0.47022901	0.72048179	0.67905331	-0.36439674	-0.36439674	-0.36439674	1.02985039	0.66804684
FGF2	3.05330416	3.89333515	2.81338185	3.0769543	4.37691469	4.37194237	4.81046354	4.53343556	4.45734439	4.07556166	4.37458557	3.74130817	4.38223666
BBS7	4.81696674	4.74058009	4.71854209	5.35299526	5.690528	5.95692907	5.49947688	5.65159193	5.67927651	5.48774212	5.39563392	5.79086079	4.58557936
KIAA1109	6.74005859	7.67941012	6.87779497	7.64221874	7.92025197	8.11615354	7.96412743	8.05689746	7.7868971	7.49861834	7.81931237	7.78116026	7.98371192
BMPR1B	5.56192359	5.57132948	5.38154759	5.17723572	4.94274011	6.48389633	6.5485973	6.68113935	6.60344493	6.31711498	5.98199661	6.47265248	4.83379482
RAP1GDS1	4.20437549	4.91460985	4.42801822	5.59039108	4.92257959	4.98698449	5.20277226	4.81497257	4.741485	5.13489038	4.98336387	4.64342387	4.34378314
LARP1B	0.79487397	2.00680212	1.12291741	3.07824163	3.07740769	3.34479033	2.77958038	3.03478435	4.52445932	-0.35928881	4.56937121	1.26112851	1.82759791
MMRN1	1.00998773	3.74843537	1.09121984	4.35844605	5.28947556	1.93327772	3.9214419	4.94216899	6.95011689	5.06019548	7.37773037	5.39928854	6.87326792
PDE5A	7.54881843	7.78988673	7.0883266	7.87921284	8.12882185	8.14301153	9.10089169	8.42969703	8.27987119	7.96985333	8.50859215	8.97024201	7.35190278
PRDM5	4.1663771	5.0886984	4.95737339	5.14485418	5.35684018	5.90151263	4.69774317	5.26181099	5.82847241	5.21304393	5.42907228	5.48422349	5.31136261
TRPC3	0.53448695	0.33268294	0.52308107	0.48953787	0.36739955	0.75930265	0.5714937	1.33493534	-0.30217561	3.015513	-0.30217561	2.61591265	-0.30217561
NAAA	5.15290466	5.31471327	4.3006449	4.6950693	4.30146262	5.07854392	5.0529308	5.38551627	5.57025073	5.83451672	5.39726442	5.12629771	5.24312441
NUP54	4.73809645	5.23280989	4.95494978	5.95419847	5.99288998	5.96967022	5.97255849	5.95856995	5.9669548	5.40827659	5.53513489	5.62005173	4.69552076
CXCL9	4.3939243	4.90524844	2.7359888	5.09675067	4.20376141	1.74880474	1.98997489	2.55931494	5.26436162	2.81535853	4.92342892	3.45248942	4.14691801
BMP2K	2.1292536	3.59478589	2.01763428	4.80843938	2.89948239	2.54504007	2.87760139	2.52692759	1.23492882	3.22762141	0.82939597	2.96542085	3.59948289
G3BP2	6.18956486	6.85279641	6.73851911	7.26976787	7.16906506	7.62258747	7.05305014	7.07693124	7.0475156	6.97999459	6.87758605	6.75675862	6.4470701
FRAS1	0.94201475	3.82227764	2.33725696	1.23150286	0.77228411	1.79485935	1.6007174	3.68676117	3.65315062	4.62214491	3.92820074	4.27147414	2.92096265

SCARB2	7.03726559	7.1342368	6.93136946	7.76917647	7.66959098	7.29781707	7.68708291	7.39850951	7.40974746	7.35234976	7.54114943	7.4482539	7.41602767
CCNG2	6.08195855	5.49651984	5.96353607	6.15749621	6.33070242	6.57361401	6.98959731	7.07923708	6.22232276	5.4120457	5.95612326	5.8068806	5.22325883
CNOT6L	5.32218937	6.01978984	5.36803473	6.21967383	6.17044166	6.39650322	6.16908007	6.24562498	6.14914352	5.87054033	6.35729052	6.17168807	5.90344923
USO1	6.83466306	7.49130763	6.74895296	7.71793453	7.69119411	8.02240512	7.66342171	7.57266058	8.09689172	7.64531862	7.54643136	7.7078794	7.41341406
CDKL2	3.05640337	3.39575816	2.59770219	2.72601655	3.54755126	4.91933455	4.91933455	3.7271282	4.80047649	2.51664203	4.93195132	3.45449643	3.58513546
SHROOM3	3.72691594	4.15423157	3.67001912	1.42807718	4.2747932	4.10222673	4.27731748	4.45121342	4.75444375	4.32118133	4.35379382	4.75131808	4.03448313
ANXA3	2.67743449	5.09149246	4.32252755	2.97536484	4.88784421	5.23971548	4.55552005	5.72091233	6.45669207	5.30189516	5.78537018	5.7617891	6.24593913
PPA2	3.91311859	4.48264865	4.23777744	5.62581726	5.27878557	5.17798492	5.58515096	5.50583122	5.42345907	3.82088846	5.31122671	5.4286573	4.02615482
CENPE	2.59864094	2.67744446	3.3765331	5.17846529	2.70286423	3.26537229	2.95889012	2.70124026	4.25727359	2.8426811	3.34952273	2.55671512	1.69514748
GSTCD	2.95070232	3.72492381	3.89867713	4.44757769	4.2185025	4.34868677	3.93714806	3.58774923	3.61884532	2.00435666	4.33378528	3.45451633	4.08094527
INTS12	4.91419255	4.36484236	4.77932931	5.01128745	5.18724234	5.63239428	5.09257652	4.85714549	4.85315025	4.84855101	4.96918139	4.52311426	5.37307546
ENPEP	2.45517325	3.59926755	2.15969365	4.53217189	2.88792648	2.80150653	2.49856282	3.12103423	2.96948953	2.93837276	4.50178271	2.79641346	4.85192083
CASP6	4.68905188	4.30546701	4.13547919	4.1233324	3.51714226	3.7627147	4.43278187	4.22501775	4.5959212	4.03431728	4.00778041	4.51371982	4.79175685
LEF1	1.12110785	0.97580559	0.98998144	3.00600893	1.94071307	0.95819104	1.4423417	0.55780565	3.03805342	-0.46770621	3.72947687	-0.46770621	3.1363395
HADH	5.03445589	5.07482493	4.83243364	4.16764316	5.666305	6.16746854	5.62517375	5.761771	5.1326706	6.24732431	5.21043308	5.94255429	5.5391749
EGF	0.31439233	0.57219158	0.39368753	0.32133886	0.48135569	0.30178153	0.30033424	0.30365519	-0.15156943	-0.15156943	-0.15156943	-0.15156943	5.13315894
PAPSS1	5.93560764	5.49353263	5.78632267	6.53189007	5.68837189	6.1974618	6.05613873	5.61982242	6.01616684	5.89553711	5.88579306	6.15195814	6.22890225
SEC24B	5.86642718	5.76766534	5.07103282	5.74844586	5.90790561	6.01731854	5.67784056	5.85855744	5.70020754	5.87289184	5.79155808	5.40637771	6.05070567
PPP3CA	5.64257468	5.46158005	5.28611731	6.58643043	5.73353207	5.88160777	5.80349947	5.93740931	5.948901	5.42705798	6.07002433	5.36900449	5.92976719
SLC39A8	4.11520937	6.54195189	5.62468188	6.07046907	3.85059345	3.87813924	4.52415247	4.01570183	4.68540506	3.27855783	5.56269035	3.88659829	4.60520734
MTTP	2.08810265	2.25645801	2.41620025	2.29845185	3.23417381	3.16446621	2.52149244	3.01811059	4.19473651	4.67851998	4.58536464	4.50243701	3.37132162
FBN2	3.22647224	2.15348194	3.93394394	3.5865183	2.31938049	3.19384966	3.35837335	5.19711663	3.38152843	4.2179617	2.57620957	5.34315603	4.06783676
MAPK8IP3	4.40790806	3.53160457	4.46143001	3.6484406	3.50773028	1.86114368	3.65449381	4.29832713	3.3834539	-0.7382381	4.11407419	3.34544422	3.68376989
RGS3	6.37838112	5.90066212	7.41258311	6.31445487	6.28045063	6.40229805	6.61267091	6.23947825	6.10576637	6.70964028	6.46149192	6.45337644	6.28520396
RNF185	5.7588233	6.00448326	5.3008779	5.17281033	6.31291126	6.36158514	6.18522286	6.1560591	5.92353955	6.19434106	5.98106746	6.08020756	5.69984154
KIAA1644	4.23991405	2.49935361	4.8761954	4.50800578	2.91116378	1.75725865	2.56978511	1.98737777	2.7928737	-1.86713603	2.24539132	3.23405984	3.80907197
PARVG	4.48377348	4.02566196	4.23172319	4.42140689	3.04149869	2.48441947	3.05109028	3.08073878	3.32358573	4.01488913	3.48203361	2.48593183	2.63626263
B4GALNT3	4.93998614	3.96419609	5.24763862	2.99739257	4.64239975	4.19948358	5.05174409	5.35183973	4.56706969	5.23146829	4.99100648	5.48652266	3.85113497
ERP27	3.39299849	3.63739521	1.63554206	3.04866121	2.70295491	2.76034085	2.59157229	2.18265676	3.98756349	3.27381474	0.59251872	2.27708178	2.07140227
ETV6	4.30648891	3.95048783	3.80352472	4.53272006	4.5867428	3.69137404	3.61327106	4.44784109	4.20950494	4.06086376	4.42202952	4.47858998	4.11461109
GABARAPL1	6.77809225	6.55736139	5.96578071	5.78859394	6.35141194	6.43745472	6.08902397	6.29507385	6.27814615	5.81585136	6.0488761	6.25578818	6.77385805
KIF21A	2.56742191	4.21554401	2.70241488	3.9841291	3.73028847	4.73177191	3.83619855	4.20564619	4.43558768	4.07221855	4.13263618	3.09900778	4.84244444
CPNE8	4.91394238	4.92029133	4.82719013	5.90815506	5.66235445	5.67640356	6.02950583	5.42448629	5.41320513	5.41884799	4.98738835	5.40379669	4.68206267
YARS2	0.39094069	0.50738511	0.69573812	1.49561155	0.44672514	0.20805794	1.35631046	1.08319812	0.24887244	0.24887244	0.24887244	0.24887244	0.24887244
FGD4	5.49581606	6.54093656	4.93666849	6.19671536	6.59525802	6.98563043	6.28235122	6.45526629	6.45357386	5.21097074	6.0409251	5.76243094	6.55699518
ALG10	2.65516679	3.89283333	2.99503779	4.36689234	4.2117252	4.10449122	4.41345751	4.40814843	4.13667001	2.94089669	4.24323997	4.16229189	2.91620181
AEBP2	4.52006198	4.01671356	3.87833796	4.06121978	5.18769975	5.07693461	5.19563114	5.20823166	4.48947509	5.09485347	4.6506196	4.61925083	4.48303588
SLCO1C1	2.17107337	2.30574444	2.55178958	5.39729502	1.88232024	1.99864489	2.85607023	1.73376355	1.27845287	-1.10217261	3.12047486	2.8169234	4.04619706
ETNK1	4.41428585	5.49953112	4.3843302	5.92526018	5.61223971	5.54647713	6.04289174	5.93097128	5.95471095	3.96456117	5.75318785	5.74704718	4.0449348
ZCRB1	5.41789219	5.27586988	4.9953184	4.61201439	5.53051708	6.33362044	5.1447892	5.442099	5.95459701	5.53496203	4.34667445	5.32501467	4.84765628
TMEM117	1.49487029	2.7190819	2.21598173	3.24991202	3.92055341	4.44011813	3.3994118	4.07757229	3.73020878	-1.0327115	4.40809611	4.14441426	4.11963382
PRICKLE1	3.3367112	3.77831261	3.03036142	5.9164217	2.94887847	4.58527986	4.31304139	4.64091116	4.12735591	5.41439087	4.55884636	4.6638298	4.53637803
C1RL	8.24673596	6.81169617	6.6814491	5.99300246	6.16402604	5.64991135	5.7936948	5.53938323	5.72436336	5.22411941	5.39989469	5.23182964	5.98870959
NDUFA9	3.8162716	3.88411519	3.99779173	4.0388026	4.5314097	4.69400328	4.33039577	4.16450664	4.47838104	4.26674474	4.58318114	4.06304173	4.13809775
CLSTN3	5.72726946	5.22465076	5.27370781	4.8874538	4.78554149	3.92031806	5.11105248	4.44780495	4.16595401	4.47651402	5.37821853	4.3199173	5.18168265
KLRG1	1.94191783	1.61856577	1.92574397	1.70401161	1.64376684	1.75810547	2.3295465	2.20153481	1.55904881	-0.57227379	2.99711665	2.96155612	-0.57227379
VAMP1	5.03043177	4.30342805	4.1012237	3.23291768	4.67613194	4.3347102	4.95925431	4.46819058	4.65822715	4.87284957	4.26250771	4.87332293	3.89747372

TAPBPL	5.13127336	4.11553413	5.02973945	4.09449677	4.27795361	4.22814866	4.33813396	3.9706294	4.40735935	4.94563644	4.62519269	4.25537032	4.30251281
CD27	2.57260045	2.18235517	0.63226497	0.84109377	0.87897706	0.35091888	0.5598522	0.39903391	2.39436293	2.85581488	-0.31345725	2.61212138	-0.31345725
RBP5	1.35539416	2.08191359	0.72689255	0.80773989	2.33015272	1.25905272	1.17796975	0.72758822	1.62449717	3.47436504	3.77460388	-0.51698484	3.33032313
PEX5	5.85242713	5.8296945	6.30893491	4.46326809	5.15465551	4.7890528	6.12551576	5.24668836	4.85743521	5.58105978	4.87934129	5.33628407	4.66595422
AMIGO2	5.06110072	4.97524031	4.62332627	3.81454691	6.56552971	6.75006185	5.55415053	5.10838071	6.16333354	6.0630076	6.75678532	6.57208106	6.0043123
SCAF11	6.62461849	7.19270367	6.80359345	7.59892228	7.60048246	7.72220924	7.62918763	7.72612311	7.5906756	7.08006873	7.25037376	7.79886726	7.02718488
PPFIA2	4.15514235	3.91690222	3.84691036	3.58413799	4.67538549	5.12621618	4.61266683	5.11919271	4.64562428	4.70260081	3.59194201	4.21779527	5.14114746
ANP32D	0.49522697	-0.07267173	0.38994001	0.00418003	0.76231494	1.02919337	-0.36168981	0.06152789	-0.19363204	-0.19363204	-0.19363204	-0.19363204	-0.19363204
LLPH	3.17299262	3.96637693	3.12575639	3.66673371	3.68770902	4.3674907	3.81532308	3.58114182	4.67094152	3.8450977	2.7284397	2.66235757	2.83361347
LRIG3	5.30131356	5.76912878	4.88897471	5.73234505	5.55396482	5.8978487	5.98152305	6.09422066	5.87868434	5.93164262	6.09462784	5.88003596	6.0758326
GLIPR1	6.85478348	6.86363518	6.33251287	7.27336913	7.63809227	7.95063962	7.2410463	7.5663774	7.57553812	6.77131021	7.2937838	7.58767855	6.72445748
TPH2	0.22340241	0.43820726	-0.05283893	0.13283452	-0.05540412	0.16073712	0.1667755	0.0618212	-0.05894936	-0.05894936	-0.05894936	3.29672826	-0.05894936
PHLDA1	6.22298327	5.49931872	6.65705953	6.9463904	4.49121155	4.63657557	5.2590818	4.71867765	5.19364365	4.68705092	4.87182822	5.14585916	4.59953257
TMEM19	4.106108	4.96313664	4.70202489	4.80239466	4.90445111	5.43559596	5.52630094	4.69668607	5.47710559	5.23318379	5.18311895	5.48260911	4.76610496
LGR5	4.18326265	3.77664392	4.09018862	1.68187575	4.35143163	4.48645149	3.64424448	4.45621016	4.63747662	3.21392592	4.8422094	4.77705168	4.65974915
PTPRQ	1.6166924	5.03190422	1.80757795	2.76644397	4.63145243	3.62775564	3.66237895	3.02645828	4.71449274	3.99779869	4.51877288	4.1280166	5.26192733
DUSP6	5.89446384	6.04321849	5.71656478	6.89034083	5.78579181	5.83694821	4.77927415	5.28438561	6.463277	5.60084719	5.70517743	5.2937374	5.64501694
POC1B	3.95367532	5.08855623	4.23174881	5.30279877	5.30911773	5.21873049	5.14132726	5.56328618	5.28214514	4.17708281	5.44275725	4.8728446	3.77756601
TMTC3	4.92584787	5.56856938	5.1580914	6.6888552	6.07593612	6.33822506	6.00279294	6.30862737	6.25887726	4.5523285	6.09840793	5.95228521	4.8648637
LUM	5.89056512	8.23679541	5.50289179	9.78226992	6.86673271	5.1570419	7.33413466	6.9177082	7.3251118	6.96238451	7.01880583	7.41172847	8.66445068
SNRPF	3.71085794	4.37789566	4.1519361	5.25253088	4.46607318	4.90987235	4.18042047	4.64792566	4.89236695	4.18422298	3.9933463	3.73013896	4.06615313
AMDHD1	1.64555641	0.85934748	1.70421751	1.18066083	1.83126307	1.46337743	1.14103421	1.99800246	2.77190656	-0.92255308	-0.92255308	-0.92255308	1.84551724
NEDD1	4.44255204	5.2100654	4.76526652	5.54716474	5.4994342	5.78762128	5.40709084	5.49611388	5.51530721	5.36835411	5.584686	5.4383443	5.2982327
SYCP3	0.20000741	0.10978752	0.11236631	0.3695015	0.5045943	0.71120072	0.45141348	0.43019549	-0.13371276	-0.13371276	-0.13371276	-0.13371276	-0.13371276
GAS2L3	0.3679884	0.85363118	1.20455635	2.5735507	-0.30104485	-0.05190018	0.32226145	-0.3028812	2.76284885	0.068079	0.068079	3.49118056	0.068079
TMEM132B	4.49247724	3.6929427	4.18822153	3.65613597	5.29797397	5.60303884	5.15102759	5.71768637	4.66912582	6.48670648	4.01846038	5.29632583	4.83680172
SLC15A4	5.79696851	4.9431827	4.96655862	5.35212383	5.78067496	5.55070432	5.45534651	6.33691947	5.35605219	5.83673481	4.81003041	5.26982539	5.36555863
TDG	4.4037678	4.94515482	4.53342602	5.73727253	5.05709016	5.37662066	4.99110391	5.41646663	5.08060413	4.57917293	4.44584287	5.08474507	4.89140232
SDSL	3.62540795	1.80715886	4.57072305	3.15522233	1.765843	2.69830347	1.81076621	1.71698885	1.79682133	3.35775681	-1.1317876	0.46625472	3.67882002
MMAB	4.26355578	4.63675095	3.93406262	3.61603078	3.97123384	3.88380053	3.61151629	4.23649608	4.07376197	4.30862798	4.58715384	4.328965	4.10598741
GLTP	6.06839424	5.39480156	6.17372392	5.6898247	5.38591478	5.43451445	5.20083676	4.69553704	5.72642789	4.97383773	5.24458963	5.33830064	6.14506522
GIT2	6.40365753	6.07308636	5.86854866	6.45673739	6.34091491	5.97252673	6.19313983	6.01655283	6.16727257	6.35855295	6.24515186	5.99023392	6.56606551
TCHP	4.79384708	4.00620821	5.08337195	3.99342474	4.36266169	4.19279488	4.23983584	4.68505351	4.82259858	4.16062706	4.73850017	4.26182239	4.86714843
MTMR6	5.11624001	5.34095811	5.14686539	6.04156884	5.81149702	6.29045802	6.00214357	5.87008219	6.05541182	5.23313503	5.82008395	6.27227764	4.70722765
SLC46A3	4.71778346	4.84468431	4.10882091	5.33634054	4.77460207	4.88420347	4.5512492	3.74816543	4.7116806	3.73432524	4.92250713	4.89643467	4.27734636
SLC7A1	4.87614207	5.11237591	5.64400269	6.09355847	6.11083591	4.61010043	5.2110534	5.69957065	4.78475824	4.69244348	5.00487879	5.26825627	5.44507195
LNX2	5.42348694	4.89705591	4.39532784	4.98233237	5.11504517	5.79371238	5.35786246	5.14546875	4.99064042	5.10016492	4.86396347	4.9081872	5.15413045
SUOX	5.42454177	4.75215935	5.36285522	4.45049035	4.64703458	5.10398524	4.88508421	4.81631592	4.60806402	5.06945317	4.55251617	5.15212022	5.2879031
CCDC65	0.60680657	0.57016397	0.83423423	0.71794398	0.62033135	0.98806079	0.57016397	1.34197654	1.29068127	-0.42967658	2.05164773	-0.42967658	-0.42967658
SLC39A5	0.37218033	-0.07431805	0.50626729	0.00578327	0.59906159	0.0927861	0.29326341	0.1601372	-0.01473629	-0.01473629	-0.01473629	-0.01473629	-0.01473629
TARBP2	3.31091751	3.20711522	4.06862882	3.61712158	1.84634507	1.27353488	3.6368731	3.09318227	3.04081104	2.82889167	3.99828431	3.14515923	2.82114792
RDH16	1.13786587	0.96974019	0.31483679	0.83465624	1.34621955	0.38215127	1.16061668	1.30424485	3.75987575	-0.36734418	1.83471142	1.26431283	2.36613198
DHH	0.36024846	0.42472344	0.35134446	1.14277381	0.54020132	0.37984683	1.23245157	0.79258748	-0.00223968	-0.00223968	-0.00223968	2.93102625	3.3577419
ACVRL1	3.86154029	5.1744963	3.83168251	5.01586178	3.83251167	2.55827773	4.03266155	4.24352934	4.30266738	4.24076518	5.41668907	4.35384101	5.7720548
GPR84	2.09543278	1.85374987	2.39912385	2.22097657	0.36716613	0.58788687	0.36356283	-0.11415613	0.85818271	2.99023289	1.86034559	2.27050283	2.9307648
N4BP2L1	4.8707826	4.35227872	4.40036385	4.70958283	5.44739614	5.24524114	5.47744325	5.61628922	5.16038117	4.18609084	4.90003145	5.05428452	4.68991196
SMARCC2	7.44090771	6.86342432	7.08942931	6.27280411	6.98809203	7.03995265	6.9229117	6.90299864	7.04956732	7.18515794	6.90204555	6.64418109	7.01828586

BRCA2	3.47138236	3.45732688	4.04555818	5.71902845	3.90277583	3.67852243	3.87860775	3.86538901	4.20532783	2.72986865	3.72567954	4.56403793	2.62372178
KANSL2	4.21886039	4.05700525	3.24177043	4.2637045	3.85063525	3.52193987	4.38373026	4.41970975	3.46994285	4.55003636	3.76787023	4.68229876	3.91796375
CERS5	5.76523956	5.14074119	5.26611177	5.65632062	5.43230976	5.09340137	5.80029516	5.78834112	5.52194103	5.51500232	4.84388647	5.71354019	5.39086528
MAP3K12	5.77308747	4.70778161	5.49453714	4.59776401	4.75778561	4.57457278	4.93577215	4.97316554	4.63272063	4.66354665	5.01258675	4.5342745	4.72789678
ITGB7	3.68009801	2.43445387	3.22401592	2.23672525	1.38785139	1.71033675	2.12123411	0.91766323	4.11391382	-0.78209911	3.32498198	1.8873288	2.76872489
GALNT6	3.28278102	2.7398122	3.98366355	3.33112834	1.94092235	1.16074352	1.70775619	1.54711704	2.57945502	3.08458547	3.6360645	0.48397078	-0.10219989
CSAD	4.98915435	4.22538321	5.33366987	3.90895377	5.1990619	4.65174517	4.98915435	5.43460477	4.70923382	5.13311093	5.52960177	5.2947123	4.67597111
LMBR1L	5.48449513	5.08753397	4.76316235	4.57186078	5.06754552	3.86418023	5.42283494	5.29556236	4.17238252	5.07237619	5.01149352	5.3538278	5.08149572
C12orf10	5.08078476	5.04197849	4.96330271	4.1535554	4.21120456	4.67169132	4.21552677	3.76603135	4.43815916	5.25022809	3.94176692	4.78169942	5.03732293
ESYT1	6.61164226	6.99391989	6.90676622	6.19430759	6.51581941	6.42790891	6.44531364	6.51369942	6.45266251	7.26193208	6.69245993	7.16408772	6.58185457
TMBIM6	7.58130743	7.91110354	7.43158562	8.03733782	7.58789122	7.58789122	7.80590781	7.18716124	7.2018277	7.69314476	7.67038087	7.74042792	7.51989508
ANKRD52	5.89853694	5.24949071	6.00511872	5.58304594	5.46186503	4.7767777	5.67922167	5.35511339	5.07596854	5.2777044	5.50786502	5.1258176	5.51683399
ZNF740	5.47665031	5.44229908	5.20546391	4.70091101	5.09187247	5.1815825	5.61410643	5.56165611	5.23082352	5.62748179	5.16811793	5.52300216	5.42156157
WDFY2	2.52859763	2.66708977	1.94514364	3.17325358	3.0067168	2.42846669	2.93273342	3.41391699	3.63756965	-0.40254597	4.6497812	3.03313566	3.37836021
HNRNPA1L2	1.17184184	0.62168296	-0.42470242	0.71114687	0.86037387	1.16963631	0.43583282	1.11617708	1.6614299	0.12581253	2.20249663	0.12581253	0.12581253
LPAR6	4.68516856	6.12338252	4.63466042	6.88220878	5.55420119	5.23271108	5.56455982	5.33120289	6.33888182	4.64561871	5.47392884	5.27701615	4.69367244
ESD	5.71532076	6.69912883	5.98429737	6.67084867	6.81862554	7.15686081	6.93657338	7.17146606	6.7983138	7.27864592	6.74186705	7.36044276	6.25242244
RB1	2.28905339	3.60544378	2.80070752	4.800082	3.7947598	3.70563384	4.05213367	3.84859671	3.92172112	2.97998681	3.84241043	2.46808218	3.43927721
SBNO1	6.4440046	6.5154713	6.22857734	6.54311548	6.63438166	6.82485916	6.93670634	6.56395267	6.63279396	6.48608311	6.55544612	6.33341553	6.67264771
MORN3	0.97339586	0.96538989	1.27755488	0.88179115	0.39412974	0.63881784	0.34336981	0.71845718	-0.39608973	-0.39608973	3.617747	0.76064689	2.94124553
SETD1B	6.27789876	5.83258694	6.63130493	5.07447569	5.97753385	5.43385688	5.65363075	6.12502687	5.72445706	6.54677481	6.27046521	5.76391536	5.33105061
VPS33A	4.59256693	4.77997637	4.77389624	4.06654336	4.66375087	4.80533114	4.64401865	4.66642375	4.23963877	4.18975911	4.43178676	4.79643737	4.5137931
VPS37B	5.62486482	5.11843335	5.46143511	4.70797279	5.00303152	4.36438048	4.64279462	4.87939629	4.73124984	4.43588699	4.56076397	4.84181796	4.22253454
RHOF	2.97343301	1.68583597	3.06122668	2.15132495	1.15021327	1.38183441	2.3557632	2.45161564	2.72860638	-0.90899929	3.64208999	1.5285237	1.31517897
DENR	5.3680892	5.971751	6.03156026	6.66289276	5.86630174	5.61401036	6.45117687	5.64837943	6.15515135	5.0790238	5.82489124	5.96926839	4.92098567
DIAPH3	0.83177791	2.56299545	3.35358782	4.32696329	3.40398723	3.95296333	2.55102191	3.10491179	4.37713041	3.11661307	3.64851578	4.81769187	3.98546568
SLAIN1	1.90498834	2.11204998	0.98502491	1.19181618	2.3432141	2.67414609	2.33904265	2.31584176	0	0	0	0	0
RBM26	6.1780304	6.26824343	5.98534966	6.92135219	6.81180644	6.82325274	6.7978809	6.7450934	6.62642064	6.08212155	6.68648087	6.35956751	6.62155795
SRRM4	1.58723523	1.48797795	1.11339224	0.98373663	2.62219657	1.91388707	1.73321494	1.80739868	1.39881209	3.37556891	-0.79334172	4.03503352	3.96875191
MBNL2	6.17570818	6.60079305	5.77158432	6.77298408	6.98166645	7.23628572	6.71834127	6.67734803	6.88045837	6.82281079	6.83019503	7.04705033	6.72374657
ABHD13	4.49815107	4.80644787	4.43543082	5.51117704	5.50359281	5.99451969	5.58313259	5.30042586	5.47030966	4.17051289	5.73532569	5.68796727	4.19483108
RAB20	5.04512701	5.18558245	4.6055485	4.23748073	3.50966812	3.58092706	3.80610149	2.58418185	3.66506757	3.49944092	3.94754981	3.39274914	3.89146601
GRTP1	2.93037735	0.29855578	1.36659791	0.00414575	0.15384016	1.51527948	1.93590471	3.15074774	1.4703277	4.10603994	1.53415753	2.51740706	-0.55324082
CUL4A	6.0909299	6.00565012	6.12255603	5.91919817	5.72101616	5.67212276	6.33156012	6.37925111	6.09606031	6.0393014	5.76415545	5.52403358	6.54184754
SSTR1	2.01033773	4.24175072	1.57277498	1.66920529	2.54311182	1.96911878	2.15975765	2.1230481	2.89523911	4.0169103	2.69744156	2.75870695	3.14271076
CDH24	2.37390646	0.71991713	3.32622256	2.73804628	2.56668562	1.39833774	2.71792626	2.41942216	1.07056802	3.77009825	3.38680537	3.45198751	2.50402625
REM2	0.75122805	0.48882971	0.58248659	0.20467944	0.98432272	0.80247637	0.20648943	1.56442104	-0.19508378	-0.19508378	-0.19508378	2.70162394	1.6319317
CBLN3	3.47590406	3.64552474	3.22501846	2.42857965	2.60117552	2.46860477	1.93219797	2.35468874	3.00862362	3.66621477	3.15637329	2.58492111	2.85461329
TSSK4	1.23116322	-0.03557646	0.41193596	-0.22958375	0.98567371	0.14071468	1.30393218	1.73391237	1.75751511	-0.15068568	3.2179553	3.04710454	-0.15068568
NOVA1	3.80916172	3.25837546	3.61922293	3.83347003	4.24562835	4.6101255	4.74271134	4.6101255	4.03231488	4.07836924	3.84274454	4.02292109	4.56564691
FITM1	0.49248388	-0.14524057	-0.14273653	-0.14524057	0.83291108	0.31878978	0.85827927	0.06930102	-0.14714438	-0.14714438	-0.14714438	-0.14714438	5.33931456
MDGA2	0.3425597	0.3425597	0.43494562	0.34711106	0.51459174	0.344003	0.60259916	0.81602068	3.93641015	3.36325295	-0.31195049	2.95181248	-0.31195049
TMX1	4.27093947	4.68492637	4.55275062	5.69364256	5.07570976	5.55828755	5.42402583	5.02943092	5.40196649	4.88939508	4.87777584	5.03710991	4.63653222
FRMD6	0.99501132	0.93685837	0.3263241	2.25581726	0.48843702	1.33423749	0.87393299	1.07301319	0.82868195	0.82868195	0.82868195	0.82868195	0.82868195
PELI2	6.13902344	5.97091521	5.43427017	5.49668056	6.06694194	6.0622981	6.05733057	6.46662861	5.50444738	5.84904714	6.04183852	5.64746118	5.7430002
RTN1	5.62453181	4.81611964	4.66260743	3.7030383	5.20828176	6.51262032	5.61861228	6.61269115	5.75214485	5.50484604	5.91677537	6.76874078	5.82570401
C14orf37	3.96223534	3.82188083	3.80168944	3.89479219	3.96260513	4.97130781	4.65115193	5.50467426	4.9585758	4.82454772	4.31524484	4.90382677	5.0551057

SYT16	0.01967685	0.01317347	0.01567216	0.02952368	0.55242884	0.07860634	0.08395072	0.01636122	-0.03792322	-0.03792322	-0.03792322	-0.03792322	-0.03792322
SLC38A6	3.26893351	3.41373251	3.91141238	5.32149434	4.41039383	3.97087786	4.27498173	3.73560235	3.79324072	2.86159374	3.70001147	2.12785316	3.17575679
NAA30	4.322708	4.78101864	4.26492827	4.75498859	5.2688977	5.34666386	5.43589035	5.4824128	5.43525888	4.13130768	5.28838983	5.18334555	3.86371879
ADAM21	0.93771622	0.61612262	0.52080156	0.94606781	1.00244939	0.7598332	0.76675991	0.52151689	1.32076358	-0.43232905	2.9410967	-0.43232905	3.03808802
DCAF5	7.22080865	6.5194073	6.77931648	6.19667702	6.49534865	6.33020439	6.67828241	6.52826051	6.44813999	6.73548946	6.43628976	6.46886633	6.66608389
RAB15	3.48958223	4.24193068	5.02943919	4.33952806	5.06596848	3.15419752	4.97904253	3.31357716	4.13440205	4.29246992	5.25250043	3.80675797	5.13025832
WDR89	4.00937568	4.34076819	3.95216579	4.67423273	5.03848911	5.76244526	5.15075369	4.83571757	5.05653583	3.63530207	5.19723806	4.69715933	3.96187193
ESR2	2.02525331	1.97498027	1.81093644	1.65917861	1.85806611	1.72894691	1.71099337	1.71592332	0.61635404	2.23207251	3.94526364	-0.8862954	1.11860266
KCNH5	0	0.3257852	0.00124472	0.01516369	0	0.00069205	0.01912563	0.00158797	0	0	0	0	0
STON2	4.50944375	5.17029691	2.40067251	3.64020053	4.53446383	3.39822603	4.05278028	5.21503786	5.57735925	3.88340198	5.75650538	5.23490518	5.74440191
EFCAB11	3.13954145	2.6874416	3.7656475	3.58539157	3.28202016	2.94765304	4.3373186	2.93898102	3.92361274	2.1038364	3.57787434	2.74003409	2.82175386
GPR65	3.04071239	3.7267474	3.84194145	5.60397999	2.63799174	3.05475884	3.19329586	2.50097886	3.12732997	3.30664531	1.96763833	2.41897886	3.88287369
PTGR2	3.86965919	3.70845362	3.82485068	3.937504	3.65010011	3.57911797	4.40525495	4.01668239	4.00758773	3.09084916	2.65756514	3.04460892	4.37224463
JDP2	4.46406305	4.66717765	5.67116933	4.3969625	4.2056329	4.65628635	4.50328865	4.29642138	5.07762023	4.08905639	4.30351616	5.20496076	4.53901953
AK7	0.26831232	0.10798305	0.49950579	0.39938114	0.77854491	0.22724194	0.65375233	0.31084762	-0.19367121	-0.19367121	-0.19367121	-0.19367121	-0.19367121
SLC24A4	4.95573375	3.6192878	4.24560458	2.69818416	6.06467106	4.50026419	4.80105695	6.83432532	4.7618452	6.28022858	5.36076907	6.38385656	5.79152938
FBLN5	8.47995528	8.10125714	8.25109229	7.47022897	9.04146984	9.1107398	9.05865789	9.29927437	8.54880087	9.29651104	8.43046749	8.88832	8.93612212
WARS	6.19335363	6.50922722	5.97473405	6.75430438	5.87290435	5.48216884	5.78169751	5.1787879	5.22217567	5.57424111	6.11986631	5.38800655	6.08821744
WDR20	5.50407719	5.01122469	4.60563408	4.97567502	5.38357364	5.17317118	5.29763458	5.58537282	5.34904205	5.31783212	4.94456772	5.46155905	5.54505554
NIPA2	4.66724876	4.53562517	4.75894831	6.12285684	5.16166627	5.40072818	5.41129406	5.0683519	5.25843995	4.75981285	5.24400919	5.13749872	4.47904423
SLC12A6	6.75576176	6.27069498	6.40266955	6.60820943	7.1408488	6.77058087	7.10401227	7.49639592	6.72141485	6.73027033	6.70206093	6.35812574	7.07362235
DUOXA1	0.17640093	0.53480357	-0.1994187	-0.14567823	0.33460074	-0.12886194	-0.26007043	0.56003821	-0.0186256	-0.0186256	-0.0186256	-0.0186256	-0.0186256
MFAP1	5.21337868	4.68015693	5.38935261	5.27761033	5.02992573	6.08749473	4.9573349	4.73690146	5.44337801	5.8436518	4.65328944	5.01648625	5.355348
TCF12	5.85602056	6.20543654	5.60945811	6.88661054	6.30247424	6.46349314	6.20904852	6.37797807	5.80220861	5.9866362	6.04925162	6.19485964	6.50714413
SORD	3.72195017	2.80127	3.77088867	2.56299514	1.96964069	3.36868149	2.76663269	2.92170546	3.1638186	3.58329294	2.66754944	3.23798975	1.2907605
SERF2	4.17492819	4.93785851	4.77693855	5.20732169	4.23571657	5.25270642	4.28397257	4.1110112	4.29455071	4.48025589	4.87981653	4.70167701	4.24628159
ZSCAN29	5.51047981	5.0457892	5.41420577	4.82855727	5.59769198	5.24694261	5.25533919	5.36195075	5.34308078	5.51801305	5.33950414	4.81323801	5.38681485
DUOX2	0.56421517	0.96394904	0.58762901	0.52451407	0.83379245	0.60577257	0.65555617	0.96998973	3.09497855	3.33125516	1.76357194	3.17203442	2.6399779
LYSMD2	2.51716074	2.82317566	3.56051919	2.96126023	3.37607148	3.56105756	3.65721637	3.6108179	3.79169577	3.78634138	3.58708558	3.41955259	3.229831
SLC27A2	0.40336231	0.41579635	0.2443425	0.26331818	0.24176312	0.82365246	0.6366582	0.62350232	0.5124913	-0.18030262	-0.18030262	-0.18030262	-0.18030262
FGF7	5.83438139	5.38064455	4.1700242	7.0630056	4.6997636	4.15239307	5.38892959	5.43874646	6.72204058	4.48848828	6.30200431	4.45941138	5.97310855
HDC	1.31839947	3.00898251	0.38917822	2.36339841	1.69126005	0.39144984	2.63524697	0.27075328	-0.67113254	-0.67113254	2.2362031	-0.67113254	4.6039361
BNIP2	5.61747701	6.51229649	5.87405262	6.51656787	6.94364404	7.05017005	6.84848508	6.80017878	6.76722168	6.08839796	7.11408247	6.99033663	5.75941966
GTF2A2	4.39154276	3.99686182	4.04036136	5.01543814	4.85804363	5.7652887	4.96142809	4.84557806	5.42746792	4.4146025	4.15469744	4.91940182	3.35960839
SRP14	6.83391431	5.88752803	6.52403041	6.31113626	6.51378708	7.15016547	6.10817393	6.05322102	7.55774241	6.97528097	6.27593148	6.06091169	6.62632866
BAHD1	5.46361991	4.87399555	5.437644	4.08671836	4.9636654	4.61360763	4.79709928	4.96858267	4.30374388	5.58023949	4.3562329	5.23833035	4.41014442
DISP2	0.31879606	0.32858233	0.33788792	0.18703533	0.18980643	0.31129413	0.44410716	0.17165627	1.34297523	-0.20130477	-0.20130477	-0.20130477	-0.20130477
CDAN1	4.5081416	3.52458408	4.70347136	3.26826451	4.40657957	3.66621538	4.76037749	5.20507175	4.50585501	3.01256729	5.32371176	4.54552783	3.92042733
TLE3	6.34288157	5.58093858	6.25137169	5.69434693	5.33937739	5.43723465	4.81730121	4.11773862	5.09226237	5.59751393	4.91199074	4.99047106	5.43726745
ANP32A	6.57209459	6.27753441	6.23061944	6.45814943	6.12510877	6.86887756	5.96653443	5.97503861	6.43379164	7.03958222	6.05249199	6.07052004	6.42772526
COMMD4	3.87393342	3.70886472	4.95105723	3.53393098	3.42929388	3.47739239	3.47449394	3.7205418	3.77052412	3.69348403	3.48095956	3.9653277	2.79958119
UBE2Q2	5.21439143	6.08536323	5.75655646	6.59565181	6.56049151	6.3234573	6.31003393	6.54644485	6.67802543	5.803916	6.79922451	6.24004654	5.6128888
PSTPIP1	4.63545265	3.75227248	5.29314483	3.82373605	3.50059718	3.87086622	4.43055001	4.34274878	4.56999915	5.02759437	4.04382145	4.90613988	4.08718073
ETFA	4.57156688	4.58378738	3.90167342	5.0629849	4.31976858	4.29959361	4.32456034	5.05111584	5.21013801	4.32262207	4.7443144	4.68086794	4.90596225
BCL2A1	5.06133764	2.60776919	4.00803122	4.81545266	1.5199593	0.92228683	1.51058389	2.34565126	3.22709441	2.83216701	2.11909664	2.15727155	3.89155256
HMG20A	5.4655769	5.65146809	4.939871	5.52663754	5.54264394	5.62181067	5.15756298	4.91233288	5.64498403	5.44776759	5.38975951	5.57810254	5.59330044
SCAPER	3.63741102	3.78439467	3.25438141	3.29203884	4.22177573	4.86570133	3.68794445	4.37625937	3.71761193	3.86680513	3.74706008	4.19522121	4.31893344

TSPAN3	6.69073114	6.86142646	6.25799437	6.78371175	6.71427856	6.9724496	6.42660412	6.32233952	6.73465514	6.84656549	6.85901807	6.92422624	6.74239182
WDR61	4.78425744	5.04754044	4.57650087	4.77177529	4.87063358	4.92334656	5.46568817	4.70817664	4.94103632	4.75297493	4.78059971	5.03227547	4.96406235
NCOA2	6.49503167	6.04609071	5.64905454	5.75802137	6.51504482	6.96997104	6.33225074	6.64918322	6.40556711	6.35352041	6.36088618	6.31229611	6.89188697
NEIL1	3.25023078	2.62755599	3.61662587	1.58859936	2.5271889	1.62945082	3.23464613	3.8320271	2.38042969	3.23871535	1.22177657	3.90938685	3.53398473
MAN2C1	5.88418802	5.66246884	6.46167979	5.07579077	5.65459197	4.51632815	5.72493925	5.70299922	5.47578715	6.15526529	6.22933274	6.18974764	5.48108423
DNAJA4	4.71853182	4.57190319	4.41512895	4.85420088	6.32531383	4.94552727	5.59810921	5.57674181	4.81425547	4.69585259	5.07418133	5.17712113	6.63533099
TPM1	6.78655969	8.45902307	7.06552162	8.15947819	9.52435499	8.97649257	8.8405272	9.14895097	8.98655217	9.1077783	9.01829128	9.11929362	9.58891744
IGF1R	5.28088105	5.21523901	4.98353913	4.16812426	5.7745708	5.02662671	5.58575101	6.08834268	4.20658597	5.77362776	4.94527947	5.86174279	5.54789438
ARRDC4	5.25692821	6.5279038	5.40661954	6.6114814	5.51003757	5.47671143	5.36367117	4.57434884	6.04690669	5.19366389	5.9942477	4.84548559	5.2082728
PIF1	1.32677002	1.02417801	2.82847362	2.27011817	0.40069606	-0.20848433	1.29573206	0.12315905	2.45243937	-0.39235536	3.38614085	1.84126239	3.50369782
USP3	5.47940543	5.4762389	5.26649416	5.90268199	5.76618651	5.59068456	5.89426457	6.00791767	5.43335555	5.51033973	5.92572091	5.44313848	6.02439423
CYP11A1	0.64895734	1.07187498	0.12691939	0.55634166	0.18067502	0.12579675	0.7805875	0.36913537	1.71957822	-0.19535592	-0.19535592	-0.19535592	3.58737338
BBS4	5.27825787	5.61751807	4.74484544	4.92500581	5.00212782	5.02130599	4.77051448	5.2765272	4.76724084	5.03715776	4.81943747	4.71992702	4.93512107
PML	6.6920916	5.98672786	6.77577568	6.2427967	5.98823788	4.99562097	5.89181006	5.56949781	5.62897729	5.86227265	6.41192193	5.59784728	5.70489758
CYP1A1	0.53931394	0.53931394	0.54180226	0.5824725	0.7980656	2.25262961	0.72022783	0.80198505	-0.160203	-0.160203	2.47445351	-0.160203	2.33510102
ADAMTS17	3.87105487	3.62070755	3.55445375	2.85743695	2.95529797	2.26656374	3.71250592	2.677604	2.94571058	2.94571058	4.00269595	4.06763855	4.15566451
ULK3	4.02453083	3.52605831	4.53031412	3.05370079	2.90714796	2.84822973	3.47181706	3.5187171	3.7842462	4.10212181	3.3796869	3.31477714	4.07421105
PCSK6	1.65628196	3.75385819	2.18952356	2.2333999	1.83596107	0.55745761	1.50348779	1.16062595	2.67836525	3.70080546	2.55116363	2.66328281	4.69427495
CELF6	2.77085477	2.21727046	2.33364128	2.3743172	2.54303044	2.71991642	2.21727046	2.22054835	2.77187316	1.68288508	2.18904279	2.77187316	3.17436338
SCAMP2	5.47424049	5.78660424	5.801096	5.53053521	5.06619676	4.8277135	4.98153061	4.80794524	5.00352376	5.42349713	5.26632424	5.34337913	5.34125268
HAPLN3	5.09696748	5.41118379	5.44195648	4.06854936	5.77294074	3.91485992	5.33301297	4.951618	4.55156711	3.88315951	4.78450895	4.52238427	4.72869647
RHCG	0.37588718	0	0.00124472	0	0	0.00069205	0	0.00158797	0	0	0	0	0
POLG	5.30051642	5.31963638	5.56124927	5.02740077	5.14625053	4.26337786	5.29789526	5.31373404	4.49248451	5.53045056	5.27308265	5.12327417	5.31771203
FANCI	2.98334459	3.05665149	4.62403603	5.17800454	3.45146797	2.94568527	3.59571207	3.28153373	3.08595828	2.72289363	4.23331266	3.08947832	4.00527978
ABHD2	5.23270427	5.61006305	5.4913693	4.9133906	6.29975636	5.17368604	6.01066698	5.76455249	5.07260364	4.89623742	5.17869684	4.83215292	6.13775711
WDR93	0.71923687	0.53845708	0.24865669	0.05437464	0.44640381	-0.06891353	0.13304654	0.3031069	-0.09520576	-0.09520576	-0.09520576	-0.09520576	-0.09520576
NTRK3	7.04374013	5.9485099	5.93248211	4.57359758	6.19218593	6.477965	6.70694805	6.59239126	5.57922886	6.94091582	5.84236707	6.18242288	5.83713187
DET1	4.45554124	4.1751577	3.91368744	4.10272791	4.30938634	3.80050499	3.39173474	4.28659135	4.11134844	3.57908087	4.34552628	4.06403508	4.52995369
MFGE8	8.5069244	8.03729095	8.15419137	6.63407763	8.71159287	8.17073314	8.5069244	8.68383617	7.99186952	8.80658355	7.77772905	8.21900824	8.36780975
ZNF710	1.79685662	1.17531767	2.44878454	1.52151874	-0.1091869	-0.40387554	0.13233589	-0.23891031	3.39982857	0.06550338	0.06550338	1.7846176	0.06550338
UNC45A	4.67379087	4.26578907	4.93242447	3.71575409	4.32306297	4.63531414	4.52519638	4.62472184	0	0	0	0	0
MCTP2	0.3506343	2.26349353	0.24894862	1.44336249	0.10214411	-0.41967271	0.03628052	-0.15463775	0.7696565	0.05589261	3.75851373	0.05589261	0.05589261
FURIN	5.86617942	4.88420373	6.24397015	5.59967837	5.09304698	4.37562714	4.8570508	4.88629523	4.60758066	5.60132401	4.81783083	5.34334883	5.21394062
IQGAP1	8.08848004	8.2100904	8.05376824	8.49773566	8.419639	8.74735988	8.42824867	8.66001025	8.59297121	8.33807057	8.39726176	8.3573726	8.39726176
CRTC3	6.59519075	5.55962533	5.76161106	5.0177506	5.82511999	5.37409668	5.89984539	5.97270337	5.56067968	6.29068322	4.70085707	5.27665087	6.45248273
SH3GL3	0.87343634	1.23923235	0.77634377	0.89528192	0.78220291	1.89825336	0.97113264	4.45123941	-0.56315795	5.05931432	2.47184784	3.84744538	-0.56315795
SEC11A	5.6185793	6.01348876	5.52179049	6.59710469	5.9051462	5.93135594	5.55186229	5.87580498	6.01867297	6.15732414	6.08112471	5.64307885	5.55471514
GLYR1	6.41004609	5.95447122	6.57643551	5.89421584	6.09720639	6.04730447	6.14765259	5.56663774	5.7580096	6.54074728	5.93604612	6.25699552	6.107801
PMM2	4.48739214	4.50812595	4.92906227	5.5964431	5.02444261	4.25498162	4.55173316	4.78000673	4.83735233	4.75885881	4.82405492	4.24460419	4.37476343
SLC5A2	-0.0004572	-0.15026559	0.22136656	-0.15026559	-0.02146937	0.09424399	0.62801128	0.74215477	-0.04752058	-0.04752058	-0.04752058	-0.04752058	-0.04752058
ITGAX	4.58123459	3.38159209	4.58353451	4.66849999	2.34632854	1.42644463	2.2339961	1.96227849	2.28546225	2.6692273	3.05717539	3.77038258	3.37912349
TGFB1I1	6.31587669	6.13148843	6.42367687	4.91147303	6.98997503	5.8367633	6.33167129	6.49517777	6.05140639	7.17836749	6.67297091	7.09771955	6.30016241
C16orf58	6.20939545	6.03945393	6.5343106	6.13555017	5.88156946	5.40698133	5.7228655	5.58580116	5.70690059	6.31886717	5.95523976	5.93387772	5.81031837
ARMC5	3.43587606	2.79614832	4.01947318	2.46373148	2.42228078	2.25132896	2.28235492	2.58385972	2.98233093	4.01140238	2.49582494	3.6758677	2.92231968
PARN	5.5698321	5.97214018	5.37382093	5.83677078	5.7164482	6.12831202	5.47556418	5.79119393	5.90839382	5.74274464	5.8259671	5.77938958	5.56667447
FTO	5.92980473	6.11147927	5.59263643	5.48427194	5.91746008	6.41042238	5.6702249	5.92027977	5.91837736	6.45340146	5.68899634	5.44727204	6.45743397
UQCRC2	6.17990485	6.44512904	5.64119491	6.61339169	6.18319056	6.31166157	6.00510673	6.10507851	5.99503043	6.45034172	6.02146977	6.31685657	6.98888838

CDR2	5.68080048	5.6892742	5.31530224	5.16381288	5.94853429	5.97037905	4.95940528	5.45061447	5.90107725	5.45668687	5.89114059	5.97905961	5.74047846
IGSF6	4.75624534	3.41713688	2.79448982	3.58444921	2.33168628	1.59838861	2.98864214	1.99638197	5.43539414	-1.19244308	3.46236446	2.70852746	4.10606984
ARHGAP17	5.61549084	5.62336223	5.50260412	5.97082927	5.96003615	5.12726069	6.0009974	5.93795676	5.35036519	5.28059456	5.74339983	5.48176099	6.19073979
MYLK3	2.09896611	2.3938389	2.04894113	1.79871676	2.33717641	2.75892393	2.01169712	2.66744178	2.58094919	1.9834506	-1.05242307	3.29948127	8.10511546
NKD1	4.61760859	6.33739483	4.45302987	3.27762654	6.11374991	7.43707924	6.35660994	5.65693166	6.49632782	6.99029919	5.93049173	6.37032509	6.46556785
DHX38	6.59920646	6.28571772	6.82629621	5.61988796	6.09177682	5.93406058	6.10714449	6.05072293	5.94812337	6.37377138	6.08846057	6.13499422	6.64628397
TXNL4B	1.61689659	1.15726813	1.27585264	1.42353117	1.24297644	0.86507857	2.19716119	0.0199028	0.02907546	0.02907546	0.02907546	0.02907546	0.02907546
CHST4	-0.02316956	1.22230171	0.19237555	0.02254769	-0.02316956	-0.0218905	-0.02316956	-0.02023462	-0.0344733	-0.0344733	-0.0344733	-0.0344733	-0.0344733
ZFHX3	7.43317382	7.17621857	7.14054208	6.58325129	7.62093488	7.32339458	7.46497997	7.36881523	7.0137684	7.14142095	7.41115013	6.94459921	7.32498378
CLEC18B	2.74256986	0.16515998	3.1136833	1.23570619	2.13601794	1.58812342	1.73924977	2.89406702	1.28616556	3.99229061	1.0377124	2.88514642	0.40221611
CPNE2	5.17831998	4.81536385	4.77243137	4.72666665	5.1966013	4.21313214	4.29189328	4.63989457	5.19499133	5.20818656	5.26186469	4.74696362	5.61987971
NLRC5	1.74673711	2.32872639	1.92168817	2.33533112	1.96751649	-0.96283274	1.29914229	0.45727738	2.40435243	0.25168	4.48818812	0.25168	0.25168
KATNB1	4.05978785	3.10670189	4.67013805	3.46889211	3.26417715	3.12070338	3.36827537	3.37007455	3.31919781	4.02454802	2.98030178	3.90321549	2.95660853
KIFC3	5.05470533	5.16019884	5.89524323	5.18663979	5.03916978	3.41332322	4.62643228	5.11650681	3.73989392	4.93152072	5.07601021	4.81294449	5.48377686
ADAMTS18	0.52540056	1.21979456	0.52792825	2.06153819	0.54208066	0.64875303	0.63056367	0.71050917	-0.33248751	-0.33248751	4.50393372	-0.33248751	3.73563548
NUDT7	3.47361867	3.4892598	2.50189345	2.54222897	3.94268999	4.63723159	3.52007714	4.08425073	4.41850841	4.41232079	3.33352793	3.75596915	4.33273107
GCSH	2.6548389	2.7851715	2.55927639	2.59231562	3.09396872	3.38555571	3.36795878	3.91561914	3.25873928	3.0318335	2.90623739	3.30659699	1.52638358
CMTM3	4.63177341	5.02954122	4.92670473	5.01394353	3.86206371	3.54708938	3.91082072	4.31932122	4.58480333	4.51196217	4.90192437	4.74287575	4.71232395
CMTM2	0.58538289	0.53117896	-0.02847348	0.56238821	0.13023225	-0.02961795	-0.03105103	-0.02776268	2.75589927	-0.46416292	-0.46416292	-0.46416292	-0.46416292
CDH11	8.14028904	8.09910354	7.45438825	8.83358797	7.35071445	6.60245628	6.67865664	6.11559367	8.24201035	6.50817335	8.7299445	6.78357429	7.43075241
NOL3	5.60449325	4.88631209	5.71740224	4.33089805	4.50744815	5.06663346	4.78680804	4.24277166	4.56608718	4.83212516	5.01808728	4.83629614	4.30576702
MAP1LC3B	5.84776628	6.61780453	5.55446035	6.61990314	6.46243998	6.50369795	6.49554922	6.33871111	6.28573464	6.16384035	6.37931342	6.26118339	5.97009126
MBTPS1	7.16217707	7.00416458	6.73021333	6.84273066	7.09128777	6.84378647	7.16767021	7.26868892	6.84828519	7.26466167	7.2661572	7.25851886	7.17672361
CDH13	5.15175791	5.790207	5.32072538	4.44740862	6.22154439	6.55101803	5.65084486	5.82794479	5.55528357	5.65197507	6.15777467	5.76441719	5.82833077
ZCCHC14	6.71340475	5.94978712	6.55883983	5.79265756	6.40943584	6.11242986	6.73566045	6.54539072	6.25289654	6.51089582	6.45563506	6.46337306	6.21126287
OSGIN1	3.45910071	2.59739496	2.74084584	1.15595507	2.30646207	3.7278821	2.08452111	2.47268578	2.94324485	3.13306477	3.66092117	3.46990815	2.94055039
IRF8	4.05762475	4.32316952	3.84742519	4.83160804	2.37006869	1.89548483	1.99815029	1.84746576	4.5360746	3.62701025	3.05370439	2.44574285	2.57819935
RHOT2	5.05652525	4.79153554	5.39377531	4.52983882	4.43017008	3.45210977	4.44181062	4.91048114	4.03487494	5.34139829	5.52829007	5.32085486	4.12605976
RPS2	7.28956937	6.92235242	7.27556744	7.26316432	6.08550995	6.1987437	6.08189213	6.57158334	7.05639313	7.48019732	6.23925184	6.6858724	6.1264874
NDUFB10	5.18138211	4.41235621	5.18338695	4.66832382	4.76259485	5.87669644	4.43530758	4.4590587	5.45079116	5.6195021	4.43712771	4.9939006	5.09649547
PDPK1	5.48075256	5.29025372	5.50082818	4.92120168	5.36869755	5.07687707	5.61888524	5.53719933	5.09211757	5.2774947	5.61046687	5.54018591	5.62993355
TIGD7	4.49652921	4.37959783	4.20306015	4.57737412	5.25925578	5.24710784	4.80799761	4.97812276	5.29596826	5.1379465	4.04618505	4.7025589	4.05223639
DEF8	5.26003643	4.67376184	5.63188906	4.32731907	4.49494108	4.0767545	4.5663047	4.36903613	4.69245329	5.72804726	4.71071299	5.27403947	4.53922994
TCF25	6.21502497	6.06970952	6.52042076	5.91149266	6.00620202	5.28528956	6.08120434	6.16328825	5.76949332	6.53882589	5.78668162	6.32674246	5.80182601
GALNS	4.58643965	3.53640586	4.4576695	4.14054863	3.95066322	2.94470351	4.39863159	3.90406895	3.66199258	3.07137141	2.86691919	4.00419973	3.68073865
GAS8	3.28145175	1.60205347	3.30103633	0.80243301	1.43179646	1.27285661	2.23123472	2.22090209	-0.30476428	4.6374344	-0.30476428	3.35841822	3.03828384
MED9	4.40282791	2.96669766	4.25432125	3.1759418	3.75239001	3.59859654	3.62735656	3.41854359	3.50366534	4.27199231	3.60059004	4.21090016	4.05097846
NCOR1	6.25597242	5.93121886	5.75942665	5.77472261	6.31915178	6.46502692	6.18690137	6.25345191	5.9762437	6.25533653	6.1934204	6.16832981	5.8305208
COPS3	5.4743687	4.9319088	4.84717429	6.03402115	5.41722512	5.74349484	6.01031868	5.22515728	5.82455771	5.35746537	5.53351166	5.44803077	6.04627605
ZNF287	3.90116208	4.10916321	4.04747956	3.83273957	4.43547724	5.36411796	3.99446356	4.70522402	4.96523868	4.79611486	4.56685939	4.63829951	3.60009713
MYOCD	4.45620441	4.31241527	4.31842427	2.81471228	6.25087403	6.06031646	5.85851029	6.40551395	5.09552052	6.30776157	4.58353368	5.5805308	5.94631237
KSR1	2.27374058	1.51430834	2.40759873	1.88659312	1.23613233	-0.24093808	1.50968289	1.16040172	2.02836173	0.13321367	0.13321367	0.13321367	0.13321367
RANBP10	5.34087021	4.51467567	4.99178448	4.20955906	4.48610033	4.44065315	4.57714321	4.77364715	4.32820665	4.71579297	4.99088164	4.33991796	4.73151427
DPEP3	0.55069232	-0.0301822	0.66789997	0.33542456	-0.0301822	0.14488447	0.28229486	-0.02692405	-0.26890856	-0.26890856	-0.26890856	-0.26890856	3.73615022
GFOD2	4.6424589	4.45069686	4.98658762	4.51346754	4.33920257	3.62360137	4.75287461	4.4490987	4.05395737	4.42588316	4.6989673	4.34311079	4.22119994
NOB1	5.34267856	5.07892377	5.64533534	4.56265779	3.90013199	4.8342782	4.49013719	4.2166607	5.02011466	4.79471319	4.934181	5.13440109	3.80347171
PRPSAP2	4.56200372	4.58383805	4.21898802	4.20234636	4.56826068	4.37109407	4.4301292	4.45326671	4.80100299	4.15465724	4.39390817	5.07303769	4.68463216

UNC45B	0.87311691	1.56449004	0.77146475	0.79964602	1.66320356	0.90045513	0.76888031	0.77217745	-0.50939245	4.48856815	-0.50939245	4.52231377	7.26877819
PCTP	4.45954225	4.64863139	4.51747812	4.44661891	3.79453357	4.58516077	3.88011722	4.04654664	4.52598301	3.78378041	4.25612316	3.72223797	4.05928193
TOM1L1	5.14275031	5.05295759	4.85120687	4.80810184	5.33023419	5.53453037	5.63982893	5.98528191	5.37835155	5.99621433	5.31438397	5.60284977	4.96904719
C17orf80	5.13327107	5.27340218	4.30317177	4.97294964	5.34600477	5.64632075	5.43076251	5.51217191	5.69260702	5.04956322	5.25742493	5.33289846	5.33757917
TOB1	5.84799215	6.43393218	5.78792383	5.3199964	5.75233953	6.87548279	5.65460333	6.35093254	6.26148825	6.70107645	6.17121682	6.36423085	6.40350272
VPS53	6.1309709	5.60126581	6.07290582	5.39400584	5.64579581	5.24768751	5.56525254	5.38418931	5.59357035	5.73374411	5.82006555	5.54011807	5.82389417
SGSM2	5.25807188	4.99847286	5.41888783	4.43135638	4.81241302	3.98142599	5.14834768	4.94066291	4.24871787	5.47280456	5.07270754	4.96770028	4.43154134
NPEPPS	6.30450405	6.60740815	6.18043429	6.66797923	6.78437047	6.51333232	7.35657459	7.1390045	6.24767309	6.92644819	6.49016716	6.57817253	6.78369083
SKAP1	1.58913764	1.68821555	-0.24710614	1.3284593	1.32405464	0.07967011	1.31029132	0.3352467	-0.07915448	-0.07915448	3.44571457	-0.07915448	4.67553322
LRRC46	0.37782193	-0.08747016	1.04737517	0.39509822	-0.04229536	0.3938142	-0.08747016	0.37356523	-0.16457254	-0.16457254	-0.16457254	-0.16457254	-0.16457254
SCRN2	4.69601502	3.95933278	4.65295168	2.99538327	3.80867728	3.26623391	4.1522774	4.18453245	3.37003101	5.36173077	3.34274124	4.68165582	3.54976968
SSH2	5.59296783	5.90755034	5.25632573	6.42767724	5.35806705	4.91480168	5.2027296	5.5579443	5.06967825	5.15622239	5.09083384	4.90638749	6.28370766
RHBDL3	0.74703046	0.37237537	0.9399244	0.59551779	0.55311786	0.44369932	0.61910665	1.32889525	-0.40480771	2.72577761	-0.40480771	-0.40480771	2.1960533
ARSG	1.97023623	0.56812281	1.06510019	0.35391017	0.32435201	0.29092214	0.69123629	1.34084326	3.24800422	0.04177995	0.04177995	0.04177995	0.04177995
ABCA8	5.54018794	6.6686071	3.70751788	5.00776697	6.07166859	4.45045967	6.82321178	6.80561117	5.94070837	6.22939972	6.24232948	6.05412987	6.96408955
G6PC3	4.67560753	3.71351823	5.15401426	4.4156634	3.64880756	3.3880018	3.36716221	3.23337157	3.78646907	4.93932353	4.64833506	4.9199267	3.06640595
CLTC	7.27908599	7.90699789	7.55031139	8.58294888	7.91183056	7.93002098	8.17991765	7.97658114	7.84105168	7.89377281	7.96848361	8.08142891	8.09674391
BCAS3	4.06117307	3.62435683	3.65789218	3.24441757	4.38010902	4.3817386	3.92841386	4.06758371	3.82584532	3.9245779	3.97652606	4.48775489	3.59336425
PTRH2	3.59849984	3.14569857	3.22171839	4.21807532	4.47823915	3.80933173	4.28421877	3.72467119	3.91462549	3.01107763	3.94854911	2.73311216	3.8055949
SS18	6.46488162	6.25094492	5.72502487	6.78649843	6.38674706	6.6412209	6.36249754	6.25262113	6.27041998	6.10419815	6.40072548	6.1937408	5.83500722
TAF4B	1.26869738	0.69627654	0.54834002	1.92504319	0.44407572	1.47085393	1.25304918	1.22294998	2.2697875	-0.14806496	3.85501815	2.00021995	-0.14806496
AFG3L2	5.31972237	5.47250615	5.69674315	5.67972785	5.21149842	5.25394557	5.0900303	5.54167576	5.31884958	5.12140718	5.05551739	5.47245437	6.32309661
IMPA2	4.83532865	3.60014631	4.51563215	3.8392003	3.94562521	2.64609415	3.31609843	3.33549528	2.76490641	2.71453651	3.39367902	4.33881824	3.92115343
GNAL	2.90373573	1.50292633	1.18175168	-0.35011275	1.43298765	1.42987334	1.94328433	0.86311633	0.17116371	3.55263545	2.65445229	3.85317104	0.17116371
SLC39A6	6.73349879	6.36390614	6.41279959	7.38565494	6.94780333	7.11668274	6.92983184	6.44778055	7.00232951	6.41998803	6.80506439	6.75965134	6.1228237
RPRD1A	5.28956658	5.82838404	5.26642462	6.09802143	6.34348691	6.54464197	6.29509232	6.24192561	6.14925761	5.07027708	6.22562129	6.39865062	5.30691976
C18orf21	4.21699673	3.89824053	3.93137486	4.15187983	4.35148747	5.17026489	3.83997127	3.8842823	3.96494888	4.18643219	3.55089247	4.24419177	3.77767285
GALNT1	5.56197404	6.3396837	5.87996362	7.31711457	6.29354224	6.3198917	6.14596408	6.11181639	6.94408247	5.84090371	6.76904454	6.6363768	6.4343234
ASXL3	3.82014742	4.30944863	3.7487228	3.27624621	3.91679245	4.9163422	4.76212868	4.79468664	4.98294709	4.06751242	3.64138653	3.61853061	4.55873316
ADCYAP1	1.28670798	3.21602923	1.40782287	1.41199247	1.13929816	1.89878771	1.28869341	1.15943456	-0.71456786	2.62400015	2.22461532	-0.71456786	4.79066609
ESCO1	4.87908165	5.67921635	5.06826467	5.72479374	5.97300679	6.24185547	5.57262719	6.09139601	6.30860893	5.37109554	5.31706106	6.05644617	4.86056166
OSBPL1A	4.84265243	5.44215996	4.60249807	4.61862021	5.85069093	5.63648817	5.475817	5.44572034	5.57596529	4.90064945	6.07910911	5.60628849	5.79789563
GATA6	6.47095912	6.20493311	6.16423141	5.36146014	5.25704835	5.85631119	5.94310312	5.9255634	5.65832786	5.88188052	6.39022896	6.05066781	5.9626793
GREB1L	3.70129945	3.99020459	3.57793614	2.94373536	4.49826742	5.35803253	4.92221213	4.60864963	4.26407592	5.1567032	3.44794849	4.78099902	5.03917643
C18orf8	5.23454311	4.92741941	4.50993436	4.4954089	5.44151745	4.85901315	5.07670535	5.4528404	4.67200398	4.83791597	5.20509493	5.31280303	4.7923795
PELP1	5.05502336	4.69443823	5.56389397	4.19205256	4.62657963	4.35850001	4.67900891	4.39552573	4.57473665	5.17599175	4.86645091	4.99804168	3.85661125
NPC1	6.52214372	5.46139665	5.91858735	5.42723645	6.07936902	5.43153718	5.57268737	6.14583991	5.03698891	5.36088097	6.02018381	5.52066163	5.27906168
SLC14A1	7.61280014	7.70589588	6.54749352	7.37274364	6.15104905	8.02882199	5.79398536	4.882562	6.5369325	4.75948476	7.87742162	4.59433632	6.28410717
ARRB2	5.76823131	5.22118786	5.70491404	5.56388259	4.6889408	3.84150484	4.72121637	4.57959079	5.04664689	5.49973088	4.41801051	4.19762117	5.13762394
SLC13A5	0	0	0.00124472	0.10207854	0	0.04264243	0	0.00158797	0	0	0	0	0
ZMYND15	2.42236606	0.84605367	2.03813182	1.98631239	0.89129638	-0.16066482	1.0367685	0.77030161	1.09136763	2.7349296	1.91735813	-0.43717681	-0.43717681
WRAP53	3.18173131	3.10213724	3.75947049	2.90892035	2.67680226	1.79265909	2.9925372	2.57939184	2.2603873	3.55468361	2.85060387	2.65388065	3.03802327
MINK1	3.81667284	3.57344636	4.21462894	2.94112861	3.42795899	2.18538913	3.44422769	3.27825105	3.25129408	3.1339689	3.76266543	4.27750868	-0.64546113
SAT2	5.04265121	4.61638876	4.89687839	4.010136	4.13281374	4.79819478	4.12531701	4.91090078	4.47636742	5.1266679	4.73651056	4.32327504	5.09695715
ASGR1	0.59861718	0.9458231	1.18411759	0.87584096	-0.38831377	-0.0621766	-0.06821263	-0.32221507	-0.11641434	-0.11641434	-0.11641434	-0.11641434	-0.11641434
PIK3R5	4.95448908	3.79568487	5.27561832	4.53354366	3.20873505	2.54080974	3.22164096	2.8375979	3.63874815	3.69721288	3.17544398	2.75257985	2.93920683
TP53	5.74974265	5.80796773	5.85500575	6.3531489	5.11887359	4.61376354	5.35499485	4.54242716	5.25930256	5.95336578	5.77139326	5.64164603	5.02417454

CCDC40	3.68643726	1.79276316	3.67546158	3.36903337	2.94352807	2.71817528	2.32611007	2.38010918	1.79777943	4.05585899	2.9305585	2.75919373	3.23050427
ARHGDIA	6.74190508	6.73155104	7.63543124	6.80276653	6.54663786	5.93481807	5.98871114	6.16772383	6.28611176	7.04657193	6.84509793	6.68557183	6.11416111
TMC6	3.27446714	3.24980039	3.96389318	3.38487338	2.41189514	1.09946656	2.55243602	1.45662566	3.27150021	4.28787071	2.18305204	2.15068356	4.06848018
SLC16A3	3.63379703	2.54403664	4.03997214	3.57463649	2.08398317	0.75983574	1.5092075	0.50168795	0.29853131	-0.9464731	2.432133	2.51234695	2.19605795
CARD14	0.30132973	0.19681714	0.69602393	0.46269653	0.30402216	0.19825475	0.25709406	0.33549946	-0.14268373	-0.14268373	2.1693505	-0.14268373	-0.14268373
TTYH2	4.84259912	4.37780775	4.07372758	4.50501161	3.48923363	3.43023548	3.26363966	3.34030988	3.63992577	3.32276236	3.16644798	4.16875822	4.77484565
RAB40B	4.0583807	4.08289349	4.41652916	2.8358135	3.49501361	3.50671857	3.93515314	3.89568004	4.15064595	4.10295787	4.18211862	3.60263966	4.02160269
EIF4A3	5.07098588	4.81724502	5.45931489	5.31192413	4.12256	4.50652036	4.49164184	4.44704291	4.39709224	4.49820105	4.99109571	4.60933087	4.39104036
CSNK1D	6.41692918	5.82624996	6.61743571	5.69117874	5.85088708	5.29517882	5.87853107	5.68513034	5.24031434	6.06365228	6.02879395	6.36104742	5.77961288
ANAPC11	3.50133163	3.19634089	4.30980535	3.38609288	3.10235486	3.23164383	2.96977937	3.49199193	3.76688508	3.99990405	3.57747584	4.04622242	3.24121811
TBCD	4.32946238	4.46683566	5.12270597	4.28380963	4.65930657	3.27166283	4.45415226	4.54046694	4.9620949	5.36212265	4.74760569	4.55533607	3.50312678
FN3KRP	5.46544902	5.27579556	5.34256665	4.52107913	4.84699077	4.89136581	4.82090281	4.57546695	5.15004233	5.68329562	4.98929799	5.34138995	5.40815308
NARF	5.12086062	4.03214497	4.94587493	4.41409416	4.40397581	4.0773676	4.75183257	4.14076034	4.38442596	4.79925669	3.80394948	3.90133507	4.23762777
RPTOR	4.57358131	3.89786138	4.81170536	3.48416384	4.06408341	3.67092291	3.94706531	3.91678997	4.02473961	4.31835941	4.53063713	4.15052688	2.69886766
FOXK2	5.06208216	4.04121964	4.54454619	3.99877769	4.86433553	4.30710427	4.7950496	4.36535943	4.48548208	4.41413833	4.5183612	4.66614817	4.10730663
TRIM65	5.33728915	4.51482739	5.34058801	4.50405033	4.09706839	4.11076989	4.69761639	4.40711398	4.79693559	5.75734448	4.43609093	4.96348417	4.30836441
CBX8	0.9546852	-0.25263626	1.2868491	0.5903093	-0.1460308	0.46037842	0.61884143	-0.20107714	-0.61279618	-0.61279618	1.25926397	-0.61279618	-0.61279618
SECTM1	3.95350924	3.5819745	4.00823048	3.7548214	1.45929565	1.03750127	2.38131919	1.79296343	2.3233027	3.0927643	4.12808584	1.92212424	2.70067805
RNF157	3.03078617	2.30702314	1.57796098	1.88485191	1.70989648	2.18396103	3.04343207	2.87095539	4.20434059	3.40626574	3.19955614	3.44803619	4.08053333
ZNF750	0.0832983	0.50798054	0.08587303	0.0832983	0.0832983	0.08472981	0.26234685	0.30094464	0.97208074	3.56322129	-0.08236049	-0.08236049	-0.08236049
CBX4	4.77039193	3.86492174	4.52170199	3.45754295	3.56833854	4.51089774	3.22932038	3.5129294	3.94077529	4.68567547	3.48178118	3.56259804	3.99049177
RNF165	1.0100475	1.48974571	0.98621222	1.66061248	1.91083275	1.63700845	1.45191148	1.4351444	2.74048266	-0.71676782	0.92622862	2.59868114	3.7915482
DYM	5.02646991	5.19633014	4.41760868	5.35995479	5.35192068	5.1972408	5.7126715	5.37210703	4.91547392	5.1999564	5.08054469	4.70259688	5.53738237
MAPK4	3.38618797	2.72254225	2.19492529	1.52733468	2.81585631	5.56866779	3.90668791	4.57106817	2.32880195	4.79676079	2.15784176	4.88295757	5.0681908
ELAC1	3.45338201	3.0990464	3.33007343	4.20959998	3.7618911	4.02363274	3.78499394	3.65816538	2.77824402	3.49009589	3.9443321	3.56765784	3.01826308
MBD1	6.31875779	5.95615631	6.322963	5.34900015	5.9052007	5.84853488	5.98473362	5.68814575	5.82823753	6.4232351	5.89430038	5.51401245	5.86419705
SMAD4	5.96910311	6.35396371	6.00010623	6.63996881	6.69020386	6.42704808	6.69834771	6.85314174	6.42585672	6.32337371	6.57994526	6.48570138	6.55257081
TNFRSF11A	3.20813255	3.91952653	5.70831637	3.79150936	2.73087051	2.65429905	3.10883638	2.85116543	3.34771794	1.30120298	3.05174226	1.7403209	4.25004313
ZCCHC2	6.12359782	5.54091578	5.70833268	5.13770266	6.04244876	6.31100278	5.85067116	5.94558577	5.45817266	5.77688891	5.90474149	5.57753164	5.97726178
FBXO15	0.16516898	0.22657337	0.11832199	0.11895385	0.1991674	0.30830812	0.20166804	0.03505419	-0.23821952	-0.23821952	-0.23821952	-0.23821952	-0.23821952
CBLN2	0.07676035	1.46682331	0.07880528	0.43713881	2.66483665	3.52941039	0.15394957	0.65223361	1.12967683	3.96885115	-0.12622082	3.30594198	-0.12622082
PMAIP1	0.7097308	1.24719818	0.69133095	1.38306943	0.22902824	0.53245218	0.9031829	0.23226769	1.87231701	-0.53364906	1.99301512	-0.53364906	-0.53364906
ERBB2	6.30266597	5.99946931	6.53546587	5.01209315	6.11916204	6.30266597	6.45297631	6.51727187	5.96432349	7.09174942	6.45762222	6.85993458	6.00052629
GRB7	0.83342404	2.76047485	0.66836104	0.37992044	-0.17423335	0.58250261	0.57425173	1.11219612	-0.21296033	3.74013998	2.53578509	-0.21296033	-0.21296033
MIEN1	4.10412032	2.82458468	4.86764713	3.82305541	3.70967115	4.39265974	3.4679178	3.64407575	4.53949775	4.51859126	4.1145128	3.99781495	3.16940728
PNMT	1.48160538	-0.88879752	0.97154686	-0.66318923	-0.43347538	1.52147623	0.67153375	-0.07236349	-0.01843951	-0.01843951	-0.01843951	-0.01843951	-0.01843951
ARL5C	-0.01837826	0.06696536	0.20465213	-0.14837153	0.53114195	-0.16689838	-0.11695231	0.18768195	-0.05256786	-0.05256786	-0.05256786	-0.05256786	-0.05256786
STAC2	6.52214363	4.21660723	5.64392464	3.53768189	6.0942744	4.93584562	4.56028408	2.56536211	3.90821534	4.75107499	3.85720667	3.13249843	1.35141333
IGFBP4	7.63399949	8.10172047	7.00707678	7.75729912	6.9388511	5.7783043	7.58649195	5.58377484	6.03421663	6.46989434	7.23487596	5.85697588	7.75050603
FKBP10	7.13097665	6.57450437	7.52391404	8.18084128	6.44163259	6.22431732	6.58500258	6.83868657	6.86317006	7.56252324	7.39937932	7.26228634	6.45953797
TXNL4A	4.95587332	4.25239855	4.99910429	4.84166371	4.29796568	4.11027823	4.55863936	4.75642106	4.68324695	4.82191565	4.94081905	4.7582678	4.40928479
CACNA1A	1.38970417	0.87127648	1.25639937	1.19268421	1.35989542	2.19999182	1.24517365	2.34004513	0.44933072	2.26311218	2.34748085	1.66478974	2.6296809
SAMD1	4.91509659	3.46503229	5.24595758	3.56848203	4.97433241	4.73665436	4.92660706	4.18237696	4.43457344	5.66330478	4.3892881	5.17990297	3.96682958
BRD4	7.4008722	6.55330686	7.29965127	6.49969607	6.96751096	6.17417622	6.85498273	6.74634079	6.15320685	6.57499877	6.53413177	6.66460913	6.6379126
SLC39A3	3.25210267	3.24549333	4.50051971	2.60679697	2.27138301	2.44117879	3.05722116	2.98095965	1.48421224	4.12828631	2.15923269	3.27256883	2.40772937
NFIC	7.73179906	6.86160065	7.66044864	6.55054674	6.69839877	6.56401261	6.54315016	6.75369745	6.61037594	6.79918199	6.98385129	6.38342671	6.77596639
PRDM15	4.86372251	4.21196232	4.82508644	3.94883119	4.22583371	4.01705649	3.88137392	3.95439613	4.45852692	4.0441929	3.76368529	3.7297709	3.75374679

PFKL	6.24193913	5.74729185	6.78394784	5.45546711	5.59077789	4.83668811	5.1152008	5.3123224	5.34906586	6.6833361	5.45364008	5.81470352	4.72268596
FEM1A	4.93892432	4.57782298	5.09360997	3.83771125	4.21754822	4.73428925	4.86539937	4.36394489	4.4656945	4.57075421	4.85554773	5.39361856	4.91297292
VAV1	4.73365463	4.19029261	4.71040413	4.40607834	3.49467755	2.37966745	3.47293069	2.77634918	3.54626851	3.28694419	2.21853825	3.68736409	4.18823393
SH3GL1	5.64550374	5.09168865	6.32395323	5.75146602	5.35407973	4.87174948	5.16910358	5.05419003	5.19025163	6.02534874	5.48171124	5.42756265	4.94268445
DUS3L	3.65679229	3.50598959	5.35970363	3.52244856	2.86125886	2.69687989	3.44469186	3.95217486	3.47277881	3.98929512	3.16761193	4.07766769	0.99601095
DPP9	5.25760714	4.50288192	5.826087	4.31089717	4.60950829	4.48093473	4.57973109	4.30027469	3.68234801	4.45504583	4.75838753	4.32491444	5.06508793
CCDC97	5.28342458	4.85815037	5.3007922	4.42215082	4.55308407	4.37445491	4.55308407	4.44720813	4.76393597	5.00723351	4.33794131	4.74449036	4.56382035
TMEM91	4.50305925	1.95854534	4.45724202	2.44207526	2.67003727	1.74231765	2.89104682	3.36392495	3.14098912	1.96070041	3.89299103	2.64425904	2.7042262
ZFP14	4.60261939	4.88983226	4.3287748	4.36291656	4.8295014	5.57697752	5.42667082	5.09615962	5.26300787	4.5628567	5.4058401	4.45533297	4.76885864
SIRT3	4.92513517	4.38908381	4.90996076	4.12222261	4.12422842	4.21125268	4.2550272	4.54098362	4.69806548	4.52244481	4.80651317	4.20553929	4.76077843
IFITM3	9.28297155	7.96395338	9.10756454	8.21441244	7.25424327	8.03201598	7.65255705	7.11641699	7.478287	7.64966027	7.70629851	7.48262483	7.04198776
HUNK	0.65261844	1.10536876	0.69250348	0.88634685	1.0557657	0.84054984	0.83815078	0.80716885	2.6228603	3.61292166	2.28889845	-0.36892513	-0.36892513
COL6A1	8.26702792	7.85862001	9.10511974	8.97978009	7.88148152	7.86640349	8.44343086	8.513129	8.13573681	8.92768385	8.44696855	8.67030285	8.15080367
IFNAR1	2.38951184	3.15186326	2.17142979	3.59458793	2.52212429	2.4356004	3.45680108	3.22818877	3.86424301	-0.35737934	3.23313602	4.28261909	-0.35737934
SOD1	7.02178127	6.80094711	6.94749351	6.49391661	6.50382694	7.18246485	6.6585948	6.17692107	7.24899367	7.46544018	6.36238957	6.62181922	7.27010249
COL6A2	8.40825654	8.24724759	9.16021907	8.66278792	7.84609	8.04387209	8.66278792	9.03486346	8.26144136	9.02949484	8.83816531	9.11329845	8.15400985
SIK1	1.45173451	1.57500057	1.18747709	1.98550212	1.19256029	1.96486044	1.15013738	1.09684444	1.44517284	2.01893943	2.26659684	3.42231526	2.81011385
TRPM2	3.93957638	4.03223008	4.40045028	3.69777256	3.03412424	2.4178514	3.0940641	2.14116651	3.94705834	3.45670475	2.81916526	4.28178548	3.47178067
SCYL1	6.25276612	5.40314584	6.50826977	5.8017553	5.83540477	5.49049289	5.3451999	5.59429638	5.74340148	6.79766278	5.77284193	6.27556627	5.23962856
TMEM50B	5.2740505	5.58484664	5.71330468	5.37497984	5.86739017	5.40333367	6.24796931	6.01191079	5.58231785	5.55586447	6.25789659	5.67110571	5.68707241
APP	8.41435981	7.78054875	8.43776513	8.03720942	8.50589769	8.81550002	8.39092493	8.67410845	7.877222	8.34376948	8.00443147	8.34376948	8.37418257
DOPEY2	1.83865283	1.3450331	2.31380615	1.81504309	1.5771421	0.17759952	0.92617015	1.41109157	0.59466729	3.15370579	0.01837426	3.02076933	3.32864989
URB1	5.79536427	5.13966246	5.85647274	5.15916845	5.96785273	6.4539307	5.33983583	6.46529136	5.16645561	6.12408047	5.94809099	5.8062506	5.12903908
AKT1	6.65561896	6.55185982	6.85671804	5.91919814	6.22761791	5.90907864	6.04040496	5.75126901	5.81881625	6.62485485	6.01481263	6.36785668	5.78868945
EMP3	5.15910731	5.62812842	5.94134369	5.43256095	4.61092964	4.66998686	4.26645242	4.48510549	5.52801683	5.61749292	5.32923883	4.93592418	4.32621211
SAE1	5.70320093	5.76592674	6.40617587	6.35481544	5.708511	5.80829096	5.84195109	5.39368533	5.74663831	5.8160825	5.75799795	6.02939155	5.95240578
NTN5	0.99987254	0.22550316	0.86655312	0.19612746	0.8079755	0.54935859	0.69051011	0.76680399	-0.12334524	-0.12334524	3.28818798	-0.12334524	-0.12334524
LMTK3	2.63490545	1.60722748	1.84136154	0.16593565	0.40274216	1.68965206	2.99881583	3.30180182	2.35448968	3.21785001	-0.66198704	1.54829668	-0.66198704
GEMIN7	4.15437758	1.78954469	3.30139246	3.02160551	2.87711965	2.73664941	2.25636629	2.5669782	1.93167788	4.25424724	2.75524309	3.39022037	4.11787748
CBLC	0.03477169	0.03477169	0.03708715	0.03477169	0.03477169	0.03605905	0.89937639	0.03772568	-0.00361675	-0.00361675	-0.00361675	-0.00361675	-0.00361675
WTIP	4.10656553	4.63798943	4.67782623	2.65380934	5.02836111	4.24338673	4.56175772	5.13575633	4.59550655	5.33913062	5.31833375	5.46798309	4.59388701
ADAMTS10	5.73784254	5.06611481	6.00054095	4.7207077	5.46323911	4.48878857	5.65551609	5.55670751	5.41398479	5.75367334	5.66803617	6.29695492	4.89020446
SLC6A3	-0.07190834	0.17976954	0.35121156	-0.1407274	0.01285081	-0.1783483	0.06162603	-0.17678702	-0.0654163	-0.0654163	-0.0654163	-0.0654163	-0.0654163
RNPEPL1	3.91732282	4.75370277	4.95251411	3.80766275	3.44229728	2.77841156	3.94797773	3.87649605	4.31496442	4.72736553	4.77982296	4.56223284	2.10822921
CAPN10	3.65524955	3.01168272	4.44933437	2.35192391	3.00590196	2.44657212	3.6274238	3.83297275	3.60136389	4.53379631	3.78721418	4.12977343	2.63899794
MYO1F	5.24336906	4.65519677	5.20011262	5.20751057	3.96910328	3.00107629	3.93447412	3.56414296	4.51027703	4.46785556	4.06397405	4.08108556	3.77229463
NLRP12	1.99093272	1.47240823	2.2187554	2.83769912	0.90703488	0.60779134	0.49477168	0.75368481	2.17151808	-1.13851729	1.20299257	1.42759183	2.30628113
CACNG8	0.12999348	0.02979724	0.134713	0.16408231	0.0982827	0.00069205	0.01731255	0.03926846	0	0	0	0	0
ZNF787	4.39759373	3.64759991	5.09274949	3.60586801	3.01656619	3.44698213	3.0757062	2.92262443	2.08945761	4.42349824	3.23421938	4.49204551	2.74276698
CARM1	5.47459232	4.65464199	5.64094957	5.14399118	4.73261352	4.39231429	5.00303356	4.78302954	4.91133481	5.21533123	4.77807741	5.14490123	4.92085594
EVI5L	4.83023665	4.42361444	5.16826999	3.74121967	4.26790853	4.58857769	4.14014807	4.39823985	4.14022471	5.1279276	4.77238796	4.95720579	4.66748923
SLC47A1	4.12981785	1.27186016	2.08409253	1.75449629	2.78948571	3.06597793	3.83052562	3.29576606	1.9109015	2.50600969	3.29253278	2.75091341	3.67083225
PSMB6	6.03243482	5.61871627	6.13880012	5.43380287	5.28418433	5.54951508	4.94323562	4.69995531	5.42477609	6.29719033	5.69225491	6.35657514	5.67759568
SIGLEC10	2.63561129	2.24282587	4.63146418	3.50879634	1.92033778	1.29768328	1.28365397	1.96384369	0.47874631	2.66530788	-1.06248214	-1.06248214	-1.06248214
ZNF473	4.97314664	4.2443248	4.17677312	3.94965718	4.26971311	4.97867123	4.22099846	3.91102231	3.63450138	4.2601153	4.64623781	4.27292169	4.08838792
FAM71E1	0.38903019	0	0.11183175	0.02272442	0.13729844	0.00069205	0.23267682	0.10235447	0	0	0	0	0
RPS11	8.45177706	8.53162809	7.85362879	8.02058435	8.04311529	9.0221341	7.24948045	7.81760966	8.86441205	8.86441205	7.80338633	7.73353928	8.26221467

RPL13A	7.92769063	8.46160776	8.11886243	8.18802551	7.50682652	7.18719047	7.19427364	7.42628545	7.2759161	8.01461156	7.41563425	7.8782236	6.98084874
CTU1	2.41576455	1.61729339	3.66566694	1.99490489	2.54995593	2.06525155	2.371206	2.92878451	2.38264778	2.22736591	0.60214049	1.63760665	1.48369396
NOSIP	4.92557311	3.90736999	4.92775356	3.29705893	3.71729814	4.13000249	4.09270765	3.40259861	3.59267192	4.8247916	3.79551761	4.17782065	2.37451142
IGLON5	1.02352581	-0.03221868	0.73616143	0.42198403	0.00534955	-0.03087155	-0.03221868	-0.02912755	-0.08417596	2.64088089	-0.08417596	-0.08417596	-0.08417596
RCN3	6.35785466	5.53343165	7.12269057	7.20055979	5.28591	5.58151387	5.31267542	5.64570145	6.3845822	6.41640034	6.18266767	5.58523923	4.57715774
ZNF614	4.3192254	5.0442422	4.38504524	4.82711425	5.35171945	5.55423122	4.63696146	4.56673607	5.31813662	4.99303697	4.22725535	4.79911698	4.62227662
SLC2A5	3.76743474	4.30714451	1.91026382	1.68786439	0.98702385	1.18504813	0.91881263	0.63806395	2.77520688	-0.84895561	-0.84895561	2.13239989	-0.84895561
RERE	7.39996999	6.85617083	7.20487504	6.12607194	7.01522021	6.84052814	7.19889708	7.14435783	6.77404227	7.29908885	7.05495699	6.8788482	7.31925702
MMEL1	0.27976264	0.20102606	0.39940452	-0.00652139	0.02818488	-0.00513208	0.31476917	0.79764948	-0.11293128	-0.11293128	-0.11293128	-0.11293128	-0.11293128
PRDM16	3.78942497	3.13395372	4.0214169	0.81883008	3.68912498	3.42402248	3.52491175	3.45298557	3.54249614	4.37702808	2.12477233	2.89425281	1.33840743
FHAD1	1.75443624	0.70789597	1.49392223	1.52976198	0.31775475	0.07561782	-0.00853963	-0.00554581	-0.29216085	-0.29216085	-0.29216085	-0.29216085	-0.29216085
EPHA2	5.14833173	4.93009347	4.78225657	3.98461319	4.36984029	4.30831848	4.01962181	4.02821836	4.57354913	4.14863533	5.92644764	2.819095	4.81595509
ARHGEF19	3.8509541	2.29255028	3.10767327	2.02984542	1.97897123	1.26209712	2.51951178	2.83828217	2.80637211	2.7945885	1.59003987	3.0456249	3.33575774
EFHD2	5.21072326	5.23094042	5.79493899	5.37398425	4.33946468	3.78907654	4.25541866	4.00860578	4.34374809	4.05917327	4.76903361	4.26526688	4.5336713
PEX14	4.79660162	4.53627243	5.16648434	3.91763947	4.14445994	4.09501256	4.12560782	4.12492388	3.69054663	5.03793316	4.72355324	4.45946302	4.55973719
PGD	5.70711	5.38371058	6.05335113	6.45444105	4.79083488	4.6924164	5.48600891	4.73196855	5.18674121	5.40700079	4.72013037	4.37984215	5.59032818
MYOM3	5.42274411	2.29974295	4.70302607	3.32584237	2.68020567	2.66107001	2.979098	2.86961329	3.4832814	2.27108676	1.58960995	-0.47862584	7.03066615
SH3BGRL3	7.42204059	6.5906228	8.44076269	7.67080729	6.58579739	5.85625721	6.66164603	6.6464388	6.80662491	6.86745048	6.92210715	5.97298541	7.01338494
CNKSR1	1.6871027	1.48313387	1.09098412	1.18891998	2.53837467	2.31437384	1.43324319	1.34610432	2.74827881	3.96742688	1.14532762	-0.70236848	1.98303473
RPL11	8.33672359	7.53213906	8.0248395	8.36346302	7.37743487	7.7772161	7.06510527	6.93622617	8.28235666	8.27179459	6.99498603	6.89879675	7.95144486
IL22RA1	1.50555718	0.67209502	0.56971997	0.70501713	0.47371864	0.35187668	0.6563922	0.22515599	2.55074867	-0.45899405	-0.45899405	-0.45899405	-0.45899405
ZNF593	3.81775002	3.33821917	4.78853917	3.23113291	2.4920367	3.47086295	3.12417742	1.76592923	3.66716699	2.73436195	2.50336642	2.45487907	1.80414546
C1orf216	5.02183097	4.79510568	5.75490883	4.52016728	4.7594035	4.71496318	4.93807689	5.07247659	5.14967814	5.31598227	4.99381223	5.53560653	4.24463536
KIAA0319L	5.64319431	5.63647838	5.2461296	5.46987999	5.75365783	4.80561648	5.4334634	5.77657494	5.75503602	5.13371783	5.52065778	5.53349676	6.09610339
PLK4	1.38568173	2.0471277	2.59547655	5.2346491	1.66166563	2.02799916	2.36622333	2.0796819	2.91744774	-1.16428728	3.28278494	3.24166917	2.71445519
MAP3K6	6.09275563	5.73981376	6.26481461	4.78863925	5.70199504	4.89657095	5.54870808	5.93106929	4.76902658	5.52274637	5.45712276	5.07150106	5.62281649
FCN3	-0.1947676	-0.1947676	-0.19307071	0.72087606	0.15228423	0.42615648	0.83707096	0.21057396	0.06549414	0.06549414	0.06549414	1.72427453	0.06549414
GPN2	4.8548254	4.0138846	4.70908039	3.68607854	4.05245919	3.79464376	4.11558329	4.24470527	4.59267977	3.37950976	4.12971074	4.49186337	4.55148598
SYTL1	2.20181071	0.41422376	0.37065657	0.20269095	0.93351123	0.039376	0.99457593	0.53684203	-0.16959483	2.74916096	-0.16959483	-0.16959483	3.57810066
WDTC1	6.01339105	5.72955804	5.84415203	4.67977623	5.47381396	5.1175805	5.50721895	5.69162429	5.40405989	6.02539478	6.00792814	5.93078604	5.19423437
NBPF3	2.58600001	1.72499404	1.70672907	2.4039262	2.28096033	0.58681047	2.24758909	0.79404916	2.9912797	3.41642467	-0.42399745	3.8280543	3.03435391
HSPG2	8.43136893	8.5956318	9.15771734	7.87218216	8.77918464	7.7833058	8.78710237	9.3757132	8.10020672	9.35772516	9.10389424	9.4153428	8.56787537
ITGB3BP	3.59109347	3.31975338	3.97564999	5.60887836	3.87785019	4.16866854	3.9208317	3.78123024	4.35384485	3.14226324	4.16724127	3.43370007	3.2608777
SERBP1	6.53466881	6.86357623	7.00942936	7.99364162	7.40487701	7.71232493	7.50387306	7.28507106	7.21399963	7.29070986	7.20736747	7.16155925	7.09647412
BCL10	4.31101491	4.66344303	5.21986281	5.62504935	4.70563771	4.51422076	4.72211401	4.73180038	4.15735502	4.12091772	5.01575387	4.83053456	4.20441519
CYR61	7.94272579	7.76516611	7.11948736	8.30417529	9.86791822	10.6720118	8.25017372	8.59569242	8.90566565	7.79672373	8.59818989	8.45250459	8.99854909
PRKACB	0.91836589	2.3584643	0.92683011	2.58074354	3.12643492	3.63089343	3.49704316	3.27374301	3.33975398	4.25751957	-0.03825776	3.22997061	1.33546535
PIGK	4.19295975	5.12732749	4.24779962	5.41000725	5.45273288	6.09556956	5.70274069	5.46680618	5.81311826	5.14680699	6.0224024	6.29070152	4.66325991
TINAGL1	5.81936157	7.4064169	6.39783464	4.20379158	7.16570801	6.85834722	6.63749382	7.30945548	7.11118134	7.54775678	7.71991123	7.45024229	6.92094399
RPS8	6.14856559	6.81044067	4.98309819	6.32239863	4.69523678	4.82187264	5.24922848	4.90352477	6.5744314	6.84507428	3.54048724	6.05718024	2.97758499
KIF2C	2.16756838	1.53259664	4.70778054	4.62519959	2.76368109	1.2550852	2.18002353	1.7439148	3.56317574	3.21716481	-1.27900474	2.48141362	2.44387143
PTPRF	6.36197469	6.46788689	5.9046916	5.22076776	5.28993245	4.98817113	5.4776024	5.3858471	5.75299914	5.45171141	5.76080501	5.46589747	4.43034251
BEST4	1.16446443	-0.21594842	0.79133427	0.12871364	0.12040067	0.4026031	0.21569386	0.67091627	2.0244104	-0.11579073	-0.11579073	-0.11579073	-0.11579073
MOB3C	6.08605896	6.08972923	5.92999771	5.36559604	5.30754054	5.31654448	5.50843863	5.28390113	5.6228269	6.06944725	5.87772855	5.70674883	5.32249946
CYP4B1	-0.28465517	0.37288771	-0.35985468	-0.3616497	4.40875653	3.72308748	2.19437161	4.09789616	1.26166628	0.34405673	0.34405673	3.98395571	5.11059778
TMEM61	0.43137118	0.12968679	1.69907111	0.0810554	-0.10991825	3.18558552	-0.25100499	1.49381345	-0.22796584	2.92243038	-0.22796584	2.87371768	-0.22796584
LMO4	5.0245437	5.38594194	4.66127237	5.06071842	5.37353124	5.63676184	5.41104363	6.11867587	5.58701185	5.10904632	5.5095968	6.03204711	5.08634545

SYPL2	3.84111151	2.68719392	3.39963701	2.39404878	3.52042404	4.01381229	4.66415628	4.03034992	3.47689432	4.1137239	3.94045039	4.92072303	4.25239485
MTF2	4.51689043	4.89659832	4.45946941	5.70186006	5.13508343	5.1391839	5.24226433	4.96132735	5.23785014	4.69900605	5.16114978	5.44783283	5.11745223
SLC44A3	3.17653185	3.74519126	2.63696363	3.48349907	2.9246616	3.57465432	3.57293917	2.73660887	4.01095747	3.82349838	4.15220519	4.18535935	4.19228469
IGSF3	4.25210806	3.8287212	4.04913484	3.28662952	3.90772145	3.91830678	4.18175797	6.23355302	4.35089787	5.27063109	4.09295339	5.84744116	5.14864312
ZNF697	3.21402989	3.94301271	3.52963412	5.11047852	3.33239148	4.08865154	3.96998086	4.02212072	3.60167649	4.06770074	3.28524158	3.56520636	3.05598946
CTTNBP2NL	6.1873052	5.95091149	6.1395652	6.43766408	6.33477102	6.16819236	5.88263535	6.64684639	5.65738148	5.9466762	6.089578	5.86339926	6.25924684
PSMA5	4.6383026	4.32390905	5.08450417	5.12658842	4.58794314	5.2462532	4.49449587	4.62361464	4.99054714	4.76055028	4.93082487	4.62169501	4.63750712
FNDC7	0.64638069	0.48862473	0.9301453	0.72857894	0.44418794	0.78593648	0.82026989	0.46397729	3.76987288	-0.17909024	-0.17909024	-0.17909024	3.27091987
C1orf162	4.52129893	5.20142354	5.12199432	6.04265569	4.28077834	4.08749417	3.96751653	4.06334692	4.70552079	3.78882332	2.5193465	2.69741904	5.10624649
CD53	5.96912889	5.66348428	4.88058519	6.32567474	4.36820383	3.9518174	4.82604698	3.56181486	4.13284787	4.26523755	3.60598741	4.53070155	4.86897326
CELSR2	4.17298769	3.88891224	4.23354239	2.63364517	3.22375805	3.99621223	4.30002855	4.20144843	3.48089046	4.2756631	3.56266554	4.87166171	3.9964549
ITGA10	7.843299	6.58285383	7.63036394	6.0355492	6.86966703	6.60765363	7.58420627	8.05210927	6.97782991	6.78732771	7.47210638	6.37644764	6.19666994
GPR161	5.51847576	5.0702377	5.19200419	4.42959527	5.36665371	4.82633685	5.4485542	5.48464708	5.08935196	5.69340417	4.78469093	5.35919661	5.6270451
ALDH9A1	5.52177511	5.79165517	5.29313514	6.0306033	4.9794977	5.49394302	5.45113784	4.93381646	5.09991549	5.9372805	5.1941925	5.77011128	5.75324575
ATP1B1	5.64745291	6.51507971	4.26331264	5.48759603	5.36697563	5.33025835	5.46493308	5.70255109	5.73346554	5.5885361	5.72369831	5.87291779	6.33518705
TIPRL	5.28688049	5.57369447	5.70493816	6.42055787	6.51698309	6.65183029	6.34064128	6.41375168	6.34019431	5.42815137	6.13315922	6.0410623	6.12572648
NME7	3.54988568	4.74435165	4.12243022	4.33248845	4.27289503	5.00194799	4.38659165	4.68655828	4.82520439	3.62780808	4.65331217	4.30346086	3.15886969
POGK	5.82825534	6.17226802	5.81711355	5.83993906	6.46851342	6.33040306	6.33444339	6.37032441	6.21480818	6.47347051	6.37020233	6.43791673	6.313672
CREG1	6.69701003	6.51762371	6.18418424	7.34661409	6.76469936	6.4632171	6.70428655	6.508193	7.03304142	6.87555519	6.46995628	6.66295566	6.39050448
DCAF6	4.98234946	5.47420452	4.96430682	5.47652653	5.76241479	5.61942833	5.71876169	5.95030098	5.33981879	5.80988878	5.39569323	5.46130619	5.43887859
GPA33	0	0.10487937	0.00124472	0.21725774	0	0.06393533	0	0.00158797	0	0	0	0	0
RXRG	0.60554582	0.87437443	0.72247262	0.76771425	0.889283	2.12197774	1.33347004	1.83943709	2.24218278	2.92140661	-0.54003318	1.06862276	3.93180902
TBX19	4.65532729	3.96747347	3.97587126	3.12972979	4.41136858	3.77203567	5.02888609	5.04018415	3.84285046	4.5124533	4.44242346	3.85401009	4.5154035
UCK2	4.76861321	3.52923332	5.01245334	5.12897009	4.26173329	3.76898073	4.19608488	4.27994274	4.37250321	3.00961287	3.83106425	4.04502332	4.35881986
TMCO1	6.00666437	6.01979632	5.51183681	6.56098674	6.14868322	6.68066202	5.85412498	5.75411973	6.32297673	6.50011196	6.38343587	6.34229123	6.04862942
XCL1	0.83095103	2.56183871	-0.02969374	0.00605114	0.83647565	0.73938065	0.91663598	0.37934738	2.47900309	-0.26356512	0.82592296	-0.26356512	-0.26356512
POU2F1	6.03379634	5.60433491	5.45645339	5.40466318	6.13921528	6.18529327	5.88618685	6.22899719	5.58697957	5.23035161	5.79911767	5.42293586	5.57259372
MAEL	0.56019281	0.92744699	0.87085623	0.46115182	0.46115182	0.46259485	0.67550486	0.68520565	-0.26563011	-0.26563011	3.98465067	-0.26563011	4.30079392
ILDR2	2.0510758	3.2268875	2.17409991	1.93016718	1.83353754	2.30003311	2.0742348	2.36123684	3.82209676	3.49223847	3.23125894	3.3353389	4.82098506
DPT	7.48486385	6.72816915	5.89384873	4.79178437	5.91581558	5.16789179	6.34631117	7.92020286	7.27521975	7.33592507	7.43932863	8.21061066	7.4927143
MGST3	6.44706489	6.78504498	5.86087792	6.05217453	6.97973798	6.39121814	6.80771598	7.0874632	6.57605239	6.93335084	6.25055401	6.65053963	7.53996028
ADCY10	1.93059091	1.88594042	1.5568923	1.73453148	1.79033958	1.88787643	2.1500309	1.92996126	2.33367034	-0.85845193	2.54735519	-0.85845193	0.19456075
RFWD2	3.11576754	3.96936774	3.82733876	4.76422868	4.344737	4.36289454	4.5795526	4.80271213	4.12015502	4.90798726	4.14664316	3.82715447	3.18725666
UFC1	6.02042275	5.89772294	5.59805831	5.74588389	5.55526353	6.48718455	5.15816214	5.50396409	6.257141	6.42635389	5.85583172	6.0595304	5.54902147
PPOX	4.15384916	4.1175074	4.16740769	2.46350584	3.67416468	2.54143342	3.62421544	3.27583903	3.70930019	4.80523487	4.51651819	4.01207746	2.45469445
FCGR2A	6.92124552	6.48625402	6.52237062	7.14319409	5.5512798	4.89417033	5.90384679	4.78199403	6.44403319	5.99886658	5.32749164	5.37811305	6.29724592
NUF2	0.95690418	0.78607196	3.03952556	4.4366086	1.09679886	1.26361073	1.66314938	1.15928997	3.06193323	-0.95016053	-0.95016053	3.23307295	2.76978983
RGS5	9.29432738	9.9750208	9.31671804	9.11070614	11.2911135	11.3737766	11.2331041	10.817636	10.1512391	10.8540668	10.1512391	11.5003892	9.61923037
SDHC	5.8125653	5.89695112	5.26261118	5.50807239	5.92560877	5.81442242	5.65841789	5.70024482	5.48633747	5.97200792	5.68278554	5.38441163	6.02374289
PFDN2	5.026414	4.73704843	5.48366106	3.95027742	4.15861152	4.71373392	3.36113829	3.19382294	5.20959135	4.64621898	3.73286135	3.88045552	4.85390586
USP21	5.16058467	4.37758599	4.97251017	3.92280736	4.16724411	4.15576201	4.97400842	4.53644903	4.77691753	4.20031425	4.41171451	4.05349848	4.52391424
PRCC	5.6219158	4.96316268	5.60665909	5.31608717	5.0652382	5.0504582	4.59042695	4.98863642	5.00029862	5.32422156	4.57967906	5.47735173	4.53190949
FCRL5	1.56630803	1.54254411	0.03273864	0.76696769	0.25047754	0.15849681	0.17441416	0.03339752	2.5721445	-0.11867124	-0.11867124	-0.11867124	-0.11867124
RRNAD1	4.4294876	3.97094038	4.66669546	3.26189836	4.07198558	3.50194081	4.19347486	4.32160868	4.0136077	4.25698813	4.10989632	4.196084	2.96630414
MRPL24	4.6841334	3.72175816	5.198049	4.24846126	3.89115276	5.00998972	3.61042249	3.56327812	3.93885828	5.25451729	4.20088322	4.19344278	4.40538118
PIGM	4.54506188	4.20906088	4.05792077	4.22236352	4.87129424	4.61512181	5.01662146	5.09633938	4.77358974	4.24800287	4.31619445	4.77063078	3.74433926
CASQ1	2.62806193	2.05662134	2.82247744	2.10700526	4.24538829	4.46872287	4.1601897	4.58095647	3.98584446	5.22197714	2.60421019	4.15242654	4.654579

ISG20L2	5.42899041	4.48530282	5.64026377	5.37942533	5.29513864	5.18467914	5.33075855	5.2084274	5.17700178	4.68320007	5.06180884	5.14134562	5.24848896
CRABP2	2.027103	3.88905354	2.64503416	6.06325221	3.42660495	2.90622323	2.35844941	3.45228032	3.46790793	3.18890332	4.72712423	3.67396241	4.65428637
HDGF	6.90447254	7.05042489	7.78335716	6.6317556	6.6370751	6.71248802	7.18662435	6.66472536	6.4904422	6.49760026	6.51744682	6.51119702	6.92748452
ABL2	6.54107339	6.23316282	6.36497412	7.05919697	6.06441077	5.53522086	6.29559092	5.98929481	5.58779064	5.5800166	5.95157878	5.7796164	6.26267182
XPR1	5.68164051	5.69730535	5.48247632	5.95295584	6.59048035	6.72590775	6.50639188	6.4190347	5.62438802	5.97593074	5.85015804	5.90507067	5.98237368
RGS16	4.36185686	4.28075187	4.12248687	6.14586638	2.73179301	2.75083562	2.79585047	2.52424841	3.1357826	1.60089384	4.18718689	2.41055808	3.47531593
TOR1AIP1	6.60603451	6.53734184	6.28676424	6.41930672	7.12873239	7.28696993	7.02220978	7.08961677	7.00497926	7.05684133	7.0179958	7.39498125	6.78107024
FAM163A	0.02115807	1.07797675	0.02355232	0.0597084	0.37067472	0.02248924	0.02115807	0.02421258	-0.07498833	-0.07498833	-0.07498833	1.92096722	-0.07498833
HMCN1	7.97964185	8.79473959	7.66197984	8.53679183	8.80534372	8.94581753	9.44397877	9.54114122	8.94239995	9.11481975	9.15604118	9.65676925	9.31854679
RGL1	3.36003386	3.19459001	2.56805416	2.78258928	1.64320722	1.57847502	2.44314333	2.75580062	3.02908209	-0.20413018	2.82489914	3.06117162	3.84036523
LYPLAL1	3.83791953	4.70687138	3.9526329	4.78721972	5.14023744	5.45542324	4.9328635	5.47541241	5.17580002	3.67095008	4.94374979	5.37277393	3.87260933
LHX9	0.00844593	0.20003251	0.00124472	0.00141639	0	0.00069205	0.0722722	0.00158797	0	0	0	0	0
RORC	0.14651369	1.7084309	0.04071345	0.24232729	0.05764383	0.03967605	0.03837704	0.04135775	-0.03113443	-0.03113443	-0.03113443	-0.03113443	4.73226436
TUFT1	1.23763318	1.91489402	1.90479041	0.81514423	1.36721464	1.6635324	1.78159727	2.42342111	2.04796571	-0.08119517	-0.08119517	1.31414508	2.25015093
SF3B4	6.50871316	4.99181774	6.64532613	5.21444273	5.61767866	5.33489682	5.37723931	5.02400115	5.39553712	6.06988789	5.68941779	5.14480674	5.03561859
ECM1	4.36156211	3.9543676	4.81487492	4.47323778	3.28896495	2.2937409	3.34839372	2.89697224	3.78546252	2.39170248	4.12139554	3.29049492	5.01242592
ZNF687	5.35637228	4.92978667	5.41731113	4.06931331	4.42698417	4.06978357	4.35162205	4.32054737	4.48694505	5.42608078	4.64753184	5.35612287	3.97949177
TARS2	4.31398178	3.58983005	4.61253358	3.78724108	3.6295818	3.23120557	3.5139204	3.32012971	3.59402411	4.31691193	4.07262196	3.73670714	3.99930161
CGN	0.28615888	4.04047017	0.25095059	0.74761961	0.35180856	0.36475817	0.35028742	0.64806187	0.81125442	-0.39747594	4.83292505	-0.39747594	-0.39747594
SNX27	6.25513967	6.01364133	5.86016173	5.86881738	5.90959359	5.77289383	6.30348744	5.74038959	5.86597455	6.20287927	6.00290399	5.85559631	6.2426953
SETDB1	5.91610296	5.23931429	5.61516606	5.52574973	5.48747687	5.16968913	5.50253549	5.51468333	5.24372952	5.72082917	5.45984098	5.43909234	5.77284566
ADAMTSL4	1.90878721	0.88985912	1.80652126	0.29613423	0.84441051	-0.18384956	0.1948719	1.10362943	0.41810145	0.41810145	0.41810145	0.41810145	0.41810145
MCL1	7.04222779	7.97437462	7.5112991	8.66126836	8.3997466	8.50932505	8.68386736	8.43861768	7.53138879	7.27394947	7.71328656	8.01603715	7.45883622
CTSK	5.98204519	4.8237482	5.44971273	5.8012357	5.71149478	5.83525197	5.70106484	6.53119269	6.37885548	6.76618983	6.22774001	6.64180593	6.13768019
RFX5	5.76280721	5.78012783	6.04224284	5.47855001	5.47571749	5.79999226	5.42615827	5.14764219	5.72383288	6.17838259	5.93164893	5.77160408	6.02845872
PI4KB	6.50309028	6.23765666	6.27936262	5.93278565	6.00785692	5.75722089	5.99334258	5.88188058	5.8322807	6.6055847	6.22848856	6.31199553	6.18552701
PIP5K1A	6.15257068	6.04327626	5.74859375	6.49970858	6.57131923	5.91333798	6.15791905	6.34285224	5.68338219	6.23274494	6.27223206	6.56640597	6.34712283
ANP32E	5.71795948	6.34295597	6.33390082	7.12661103	6.71163524	6.94066671	6.64645511	6.72460671	6.79964209	6.19691715	6.27234051	6.61632039	6.11446361
ANXA9	-0.08223321	1.72303715	0.20451293	-0.11618666	0.00646666	0.08987058	0.06796091	0.06636797	-0.08721865	-0.08721865	-0.08721865	-0.08721865	-0.08721865
SELENBP1	6.18841566	5.45528821	5.7252506	4.53604975	4.3070196	5.8348363	5.38180398	5.09797507	4.47389301	6.34347985	4.90687112	5.89258537	4.83859445
CERS2	7.31970948	7.16478334	6.60858568	6.48809532	6.91130276	6.59498875	6.83234908	6.75177449	6.43117424	7.06847536	6.79063918	6.83832165	6.48197991
ENSA	6.10436427	6.30938227	6.19390538	5.93441715	6.64582272	6.73034959	6.80482478	6.52434421	6.91011267	6.62051651	6.24158562	6.52125774	6.565164
SEMA6C	4.49521195	2.96155305	4.14276424	2.91553944	3.27254284	2.75530147	3.76022228	4.10791543	3.22711818	3.11609983	4.12082377	3.02034753	3.92331671
MRPL9	5.05875799	4.48377844	4.56137341	4.88110092	4.77662704	5.26464264	4.27255318	4.47138286	4.76159004	5.38815665	4.90361125	4.34494704	4.35853671
ARNT	6.86436454	6.85196845	6.28199607	6.69395185	6.80386588	6.55566265	6.58137411	6.97371852	6.56611481	6.61221518	6.62354345	6.68479074	6.6529934
POGZ	6.99185839	6.8268319	6.34672782	6.48048971	6.7829674	6.5344032	7.04968268	7.0100321	6.603499	6.79191453	7.09193679	6.77895595	7.16360306
C1orf56	3.88461098	2.46561739	3.25412562	2.15005876	3.04481208	3.64792414	3.06664295	3.41434564	2.77712416	3.47957442	2.82586301	3.96214317	2.4672027
OAZ3	0.28266827	0.17895694	0.60090634	0.49705634	0.36925448	0.2846814	0.29186233	0.19213866	-0.23571768	-0.23571768	-0.23571768	-0.23571768	-0.23571768
HORMAD1	0.59391632	0.54956302	0.55212401	1.03679066	0.54956302	1.59721713	0.63700614	0.55283025	3.09675727	-0.45067643	3.81057747	-0.45067643	-0.45067643
GOLPH3L	5.40887354	5.55460343	5.61340708	5.77528316	6.05513405	6.06619641	6.24064437	5.66465173	5.71402668	5.83271942	5.5267236	5.78933404	4.9400493
GABPB2	5.13352571	4.80708884	5.17805065	4.43298798	5.01127873	5.24739546	4.94799364	4.95667105	5.55369435	4.33725862	4.63140069	4.5889827	5.17781279
DTL	0.02289875	-0.37184817	1.36952319	2.61903404	-0.41833039	-0.36565272	0.83674171	-0.25894395	0.50416516	-0.13723113	-0.13723113	-0.13723113	-0.13723113
DYRK3	5.11464069	4.87836913	4.26703739	5.91226932	4.7309878	4.18474622	4.90816878	4.3136987	4.86387255	4.07063493	4.82199431	4.65378554	4.46646558
EIF2D	5.67962182	5.60629328	5.17808411	4.80379065	5.29141513	5.26818931	5.09333259	4.97732188	5.31548715	6.1480183	5.2979274	5.86031177	5.07820301
INTS7	4.44351689	4.26523016	4.7387609	5.61713061	4.52046931	4.92332045	5.51763538	4.76042588	3.88507964	4.08697671	4.53199798	5.02419437	5.11670917
VASH2	1.63999622	1.41066898	1.42059631	4.02598144	1.55853517	1.25860499	1.03943341	1.56583114	-0.78069048	3.01102786	2.99145871	0.24407785	-0.78069048
TAF1A	3.65435647	4.68343631	3.42331937	3.89196417	4.11873668	4.61903981	4.19393573	4.07867319	4.3707612	3.36637737	4.25060386	4.0440582	3.24967831

SMYD2	3.87467975	4.43662785	3.64713335	4.7736567	3.9376068	3.69147582	3.75999474	4.12962721	4.46951416	3.51024556	4.49415991	4.22854194	4.5909474
SUSD4	1.37267902	2.39457835	2.09103021	2.31002301	1.17689566	1.8822979	1.91133258	1.42705291	2.89895015	3.29302532	-0.84599	-0.84599	3.33673985
DUSP10	4.13003302	2.98889773	2.48655618	4.28905214	2.62405106	2.6715762	2.31925559	2.8030309	3.48006307	-1.14571777	3.25112588	3.40018645	3.99813424
HHIPL2	0.37913251	0.65206031	0.23132582	0.51135961	0.35854258	0.23017571	0.42391397	0.35785028	1.03175195	-0.15504761	3.39381478	-0.15504761	-0.15504761
TP53BP2	6.63183554	6.57066441	6.22075652	5.86926337	6.76543293	6.91314559	6.51550961	7.26258819	6.36836836	7.23379605	6.13529767	6.94684437	7.0085166
ATP8B2	5.77392864	6.59177292	6.30992627	5.57358889	6.61119393	5.82594985	6.55692626	6.69366646	6.22847562	6.70248765	6.73379897	6.96750329	6.67483278
ADAM15	4.74241884	5.13222512	5.42980336	5.21015505	4.49508945	3.99640825	4.44017704	4.45384975	4.52710434	5.60741743	5.28894255	4.59590065	4.08563424
JTB	4.82252288	5.73304811	5.36428955	5.39737383	4.57553019	4.29317777	4.84855716	4.84403234	4.7120741	5.47932258	4.50673746	5.26799711	4.92267488
RAB13	7.24738953	6.4364816	6.94952	7.15224251	5.99638583	5.72875255	6.41008072	5.95242203	6.66187642	6.33922919	6.40335136	5.80644762	6.21861046
S100A8	3.25353933	2.82427334	2.34365593	5.44177198	1.19415417	0.10661071	2.6215986	2.07361419	3.38316704	3.68839586	-1.19575178	-1.19575178	3.68839586
TPM3	6.41766991	6.79358171	7.00642979	7.4346225	6.18364766	5.8428851	6.35543738	6.10503444	6.6263548	5.8920242	6.43821029	5.58572855	7.16908598
NUP210L	0.3806741	0.50903614	0.33276138	0.25824183	0.35532353	0.21186149	0.16807445	0.24714084	0	0	0	0	0
SNAPIN	5.24592193	5.19065682	4.52644436	4.94823965	5.12720029	5.52223109	4.64100483	4.63679178	4.75391447	5.18748365	4.92648952	5.341151	4.65221566
SLC27A3	5.08212334	5.20815202	5.37665974	3.71942929	4.39635877	5.04098656	4.6796448	4.747244	4.53434443	4.97875107	4.58846582	4.92444793	5.02155244
UBAP2L	7.39727053	6.75505966	6.96674105	7.18958345	7.04398707	6.42149833	6.69544375	6.74664006	6.65703365	7.0034645	6.67288302	6.31608576	7.22605898
SLC39A1	6.95935266	6.16168602	6.55791495	6.07949737	5.67534781	5.93067063	5.36723594	5.58836823	5.69580085	6.19327703	5.53966741	6.12689887	5.35825048
HAX1	6.2875591	6.16760906	6.06150515	5.58157285	5.97430279	6.33187979	5.37954439	5.61482176	5.8512895	6.38188785	5.78388415	6.12257157	6.10875129
CREB3L4	4.45519698	2.07243528	3.97190053	2.69780276	2.3299131	2.1867037	2.22583108	2.22882233	1.47771983	-1.07810137	2.69771265	3.16609153	2.5777737
EFNA3	1.47912538	0.58263298	1.18510065	0.99258332	1.11264569	0.69645345	1.3706271	1.19123059	-0.49381545	2.49735694	-0.49381545	-0.49381545	3.3088592
KCNN3	2.53854543	4.43893739	2.79410358	3.14529119	3.95413402	2.65994281	3.16000226	3.74857885	4.42642777	3.9291258	4.86346747	4.24241805	5.01841783
C1orf43	6.67583657	6.60671416	6.11151855	6.58496825	6.33048739	6.71148674	6.59259771	6.58768511	6.17940059	7.04827134	6.33577928	6.79819935	6.56814136
GATAD2B	6.82862635	6.55915222	6.44114308	6.03518567	7.09499875	6.63375471	6.62362274	7.01006082	6.53423545	6.97582532	6.75664487	6.57550768	7.06270012
ILF2	6.81168525	6.51081671	6.58357982	7.66552882	6.38390479	6.79471393	6.46422115	6.33174154	6.34463225	7.12703546	6.32129611	6.73276021	6.79564454
RIT1	5.4053179	5.45712089	5.45734942	6.63977946	6.34376544	6.28478129	6.1434593	6.26299892	5.67103516	4.98159633	5.83358938	5.92184873	5.89189799
INTS3	6.30769746	5.81165901	6.28441649	5.40447943	6.49980271	6.01449882	6.8455491	6.26518853	6.07120856	6.51041259	6.67538634	6.41476189	6.37142463
HCN3	3.20700256	0.94800435	2.9501045	1.52343796	2.0024437	1.74043173	2.65734546	2.59914291	3.0742024	2.55526857	0.88754471	-1.02059375	2.58787605
ACTA1	0.52172179	0.15807585	0.16056689	0.15807585	1.24605977	1.21449267	0.91570539	0.16125384	0.04399016	0.04399016	0.04399016	2.53796559	4.84821466
C1orf131	3.83050327	2.68306438	3.65174253	4.15883022	4.00174061	4.25648977	4.04587387	4.05722285	4.45627535	4.12381504	3.55086398	3.7474941	4.18095937
GALNT2	8.21860198	7.16533302	8.2967141	7.35619273	7.41035712	6.97501753	7.39693009	7.38981653	6.98362636	7.60999965	7.47602933	7.49070872	7.30264151
TTC13	4.60529212	5.20605155	4.17744219	5.4212959	5.66079725	5.57582643	5.32209479	5.74235744	5.12205308	4.26756568	5.60508481	5.79704339	5.57476568
SCCPDH	3.5761123	3.77774226	3.74243003	4.52054586	3.95729511	4.60257201	4.46390586	4.49400984	4.75969443	4.63903458	4.15858286	4.40443402	3.83327859
LYST	4.84793833	5.8207199	4.58565112	5.69248631	5.96782444	5.73502139	5.76984661	5.92575237	5.97919789	5.66550111	5.60734807	6.01551638	5.78144541
CEP170	6.10987277	6.22468463	6.15463364	7.49953723	6.20685636	6.43768781	6.30771655	6.38258577	6.48738265	5.89131921	6.12106582	6.0638865	6.3405672
ACP1	5.51653594	5.97108034	5.08141064	5.63926368	5.39348403	6.03732954	5.10257924	5.82676474	6.06697918	5.84969874	6.06204303	5.43351836	5.30175029
SNAP47	5.04811984	4.69246677	4.66380797	4.05740107	4.35425047	4.31227606	4.62903183	4.2725561	3.89436907	4.98779375	4.50677027	4.65970133	4.34626398
SRP9	5.42044517	5.9041732	5.57759688	7.26708018	6.69576245	7.20876341	6.96577187	6.75443334	6.88109625	5.25364902	6.9854596	6.80665878	4.9779634
NVL	3.9754483	3.00752756	3.49351333	4.09406811	3.81974357	4.0779737	3.59584318	3.97240477	3.34315669	4.14975646	4.13692511	3.7184143	3.48567182
DEGS1	5.56736331	6.12949306	5.5528105	6.58457256	6.54869932	6.29745146	6.10395698	6.34289155	6.09792137	5.73755398	6.08860839	6.38971941	6.49670388
FBXO28	4.96531871	5.32166819	4.63375181	5.65761176	5.80098419	6.22004593	5.82196608	5.59475637	5.68280339	4.33512913	5.83818406	6.12612343	4.5619357
ARF1	6.35088816	5.76975684	6.18632243	5.96436043	5.57609744	5.97438399	5.20470438	5.62730355	5.93212166	6.68284737	6.28921839	6.16502061	5.48057289
LEFTY2	4.83986501	2.56667202	5.99191645	2.43181255	4.22894593	6.37562457	3.42863228	4.18234469	5.39375642	6.09035441	5.13769414	7.16782548	3.57440168
CNIH4	5.4069985	5.22322718	5.19489222	5.20436016	5.99580382	5.81300493	5.90645352	6.05117278	5.45031515	5.23380908	5.13040562	5.32528023	5.11049496
ITPKB	5.78100359	5.18340018	5.77024979	4.86523832	4.6321073	4.98827323	5.25471762	5.42363115	5.20709527	6.02230628	5.31864353	5.50930489	5.42255239
GUK1	6.24205346	6.88627782	6.66238158	6.2115586	6.00881232	6.04510156	5.63761137	6.14361559	6.30471562	6.85637972	6.12803906	6.4311554	6.12068004
CDC42BPA	6.81972477	7.26384595	6.8946459	6.92796953	7.44927757	7.54180969	7.40934764	7.64957533	7.37115664	7.13052512	7.10206921	7.15345278	7.15576324
CNIH3	1.67783477	-0.06905508	0.37382701	0.07256303	0.4261944	0.28295296	0.30774351	0.28425947	0.1660419	0.1660419	0.1660419	0.1660419	3.24197926
C1orf35	2.71918958	1.7796645	4.31285572	1.90864661	2.52851226	3.18826555	2.27311285	2.27180651	2.6137809	2.00200608	2.20358615	4.13281073	3.46579358

MBOAT2	4.24708675	4.63664169	4.46850436	5.02380984	5.22711832	5.53049852	5.19805876	5.61756061	5.07767079	5.33225322	4.79895579	4.87889822	5.37164653
PARP1	6.27199879	6.19672277	6.69869784	6.46981057	5.6059302	6.2105567	6.43391191	5.26537262	6.02876828	6.36063454	5.86226233	6.51915128	6.49752423
PSEN2	4.90729638	4.42190458	4.81001664	3.14734508	4.02506793	3.86464904	3.69280249	4.3284545	4.54030494	5.2872995	4.92161471	4.63400533	4.94363406
PYCR2	4.50548623	3.46874378	5.13215117	3.51124452	4.06666852	4.15750608	3.93536051	4.22701061	4.46884031	5.26681495	4.08884546	4.66374558	3.50356776
LBR	5.14784987	5.00799669	4.66658355	6.08554462	5.23744878	5.00203878	4.98345456	4.99868847	5.28469157	4.75579634	5.4819207	5.19168444	4.78161991
WNT9A	-0.13094205	0.84486273	0.23199533	0.09047925	0.86914503	0.46008174	0.5631761	1.50225771	0.40515647	-0.16475233	1.79257868	3.17542	-0.16475233
EPHX1	7.07824152	5.31424136	6.25008064	5.7196591	5.44447681	5.5712638	5.95179553	4.83887154	5.70146959	6.37357499	5.42882219	5.34329059	6.40814769
SOX13	4.62663858	4.63311586	4.38593901	3.58955459	4.76615527	5.03964119	4.9890153	4.67742964	4.56277368	4.83800415	5.12366274	3.78145291	5.26964865
ETNK2	5.13903375	4.67329823	4.81791839	3.03829643	4.57573049	5.19719105	4.16764129	4.39411767	4.43982197	4.83810339	4.15504171	4.38942265	3.34777119
PPFIA4	5.70158313	2.93334048	4.28582934	3.61503483	4.52154369	3.75536026	3.95994828	4.51308691	2.71354802	4.64955997	2.65842024	4.08756859	4.28088073
PLEKHA6	3.13526994	4.52849991	2.07269936	1.50189132	3.41839127	2.72811953	4.03295739	3.96187143	4.55066012	4.9493617	5.33625735	4.89944596	6.00900665
PTPN7	3.19417163	3.06335716	3.74788246	2.84312476	1.83447833	0.61729709	2.29254784	1.51468965	3.21376058	1.72306872	2.60906962	2.15817357	3.45017157
ARL8A	5.58998376	5.27147396	5.57485935	4.72496909	5.26311163	5.40398675	4.6582795	5.16748507	5.22260267	5.2715742	5.13567343	4.69677864	5.49058844
OSR1	5.86242539	5.18733593	6.88182104	3.74964015	4.83774749	5.8023767	6.109541	6.97115984	5.92976003	7.4644247	5.63914175	7.73438896	5.68748443
GDF7	4.24750224	4.62921283	3.71087943	2.22344584	4.14335824	4.47173574	4.71493942	4.17216401	4.70529962	4.70877957	4.48939081	5.40741956	4.19314047
PDIA6	7.14612615	7.03838299	7.55429158	8.22236379	7.05184723	7.07845826	7.2963773	6.48728596	7.10382417	6.92486187	7.04850172	7.0918054	7.12852393
RHOB	7.02073545	7.6481582	7.19903333	6.28304448	7.56803137	8.85010743	7.54820213	8.15556645	6.93877361	7.59516414	7.08399555	7.98882953	7.12331962
ATP6V1C2	1.49914732	0.9686988	1.50041046	1.04422431	1.86288461	1.67183924	2.76659418	3.17150719	3.98053871	3.07879375	2.42003085	4.06249512	2.69670962
GALM	5.47886137	4.39337151	5.5839491	4.2048055	3.63333594	4.28119205	4.63716401	4.01584106	5.34601327	5.24462218	4.46663338	5.73418361	5.20607661
CAMKMT	3.25012202	3.67159618	3.52541642	3.89896289	4.32792026	5.00386105	5.00333044	5.54962809	4.58963247	5.1816014	4.2731776	5.15580067	4.02816956
EML4	6.18324555	6.55336574	5.91065938	7.25009466	6.30547617	6.26180528	6.29367682	6.37734184	6.61089526	6.31314356	6.54372046	6.10720133	6.30829672
CALM2	5.67874593	7.3721223	6.52237262	7.95211055	7.78075855	7.65875647	7.77650513	7.68970941	8.01122025	7.62303257	7.73409097	7.83778501	7.00690836
CHAC2	0.36912551	0.17564415	0.1781303	1.20108862	0.56912753	1.65218782	1.41818554	0.1788159	-0.3066942	-0.3066942	-0.3066942	4.5350911	-0.3066942
RPS27A	6.37087012	7.53183265	6.72087576	6.87575013	6.78524568	7.5342666	6.21224038	7.02838175	7.40007736	7.6285261	7.07915118	7.21411897	6.75840432
WDPCP	2.57372368	3.61191935	3.27884972	3.44348005	3.23904096	3.11890718	3.59957729	4.30252565	1.65200176	3.87401597	4.21196281	4.08503387	3.27143321
VPS54	4.70396213	5.63062872	5.03031092	5.95532135	5.94604806	5.80929753	5.97577861	6.12641976	5.92337713	5.02798089	5.99381942	5.91932009	5.08060179
ASXL2	6.27340194	5.81739061	5.87191637	6.13006538	6.035856	6.38927767	6.14129391	5.49743632	5.56790529	5.55966689	6.15725588	5.86285805	5.97324338
ETAA1	4.16746638	4.96622793	4.46212358	5.36040337	5.63426847	5.9805818	5.42576208	5.84278993	6.46412348	5.45758178	5.40511934	5.74178231	4.29668977
SNRPG	4.24319005	5.13216809	3.84865538	5.15846662	4.96300908	5.23595202	4.59256737	4.41113932	4.27989459	5.09509243	3.767268	4.90956063	4.60509748
ABHD1	0.61005223	-0.00364403	0.32108893	0.6901716	0.15968767	0.78901297	0.91876342	1.0102357	2.61730521	-0.21704284	-0.21704284	-0.21704284	-0.21704284
MEIS1	4.29963411	4.60146657	3.45412621	4.19130677	5.11777249	5.22698074	5.25145958	5.35399898	5.11919703	4.74922261	4.98193312	4.85075613	5.02329283
CIAO1	6.37226101	6.20400596	6.23459022	5.89780687	5.89850363	5.70334126	5.89174109	5.81103445	6.05214565	5.95649623	6.05237935	6.35149165	6.09551324
ZNF514	5.31684788	5.17211995	4.61990072	4.60945514	5.53549325	4.6949378	4.9851661	5.44947345	4.72308599	5.51071747	5.53749739	5.04258504	4.90264739
SNRNP200	7.46644004	7.23433243	7.27471194	6.83356024	6.90799349	6.77827021	7.00077339	7.03973261	6.62070168	7.46161683	7.08808067	7.34309316	7.37935732
MRPS5	5.13706316	4.64927219	4.78900227	4.93806371	4.92770691	5.31359221	4.76593807	4.86677551	4.68261662	4.64675915	4.60647932	3.94459248	5.2054135
ANKRD53	3.73292605	1.78947285	2.5393666	1.33245455	2.12749266	2.37085941	2.24073627	3.01476199	2.88106032	3.09468255	2.52031991	3.30508876	3.72162858
TPRKB	3.54463166	2.94643426	3.39775281	4.67966441	3.64137792	4.27814485	4.3542043	4.2660605	3.75458181	2.93590097	2.12527938	3.93157589	2.39666888
EXOC6B	5.44211602	6.01882509	4.85821626	5.14570234	5.94217295	5.89832627	5.65992811	5.72192007	5.44108883	5.61732812	5.89896623	6.00614232	6.51430342
SFXN5	4.00270327	3.48558595	4.51918134	3.86504831	2.92416612	2.4955108	3.30348657	2.96163147	2.85474837	4.14196962	3.3855543	2.72198567	4.21525608
TEX261	6.55699974	6.18645805	6.44037599	6.24241647	6.26598693	5.64588593	6.22498705	5.52375286	5.9104477	5.77207783	6.44407863	6.09334305	5.92294135
DQX1	0.04147293	-0.07705628	0.41931158	-0.15723889	0.10249114	0.05544896	0.38857212	0.17991142	2.57667066	-0.26863102	-0.26863102	1.46773786	-0.26863102
DUSP11	5.23392774	5.36370986	4.72965369	5.84775965	5.86161277	5.75465092	5.66679798	5.74710726	5.88208653	5.50108308	5.33915429	4.74430707	5.85224173
ST6GAL2	5.05581616	6.88467438	6.17338923	5.42607001	8.27351051	8.20836322	7.71147203	8.23207499	7.94122495	8.4938635	7.91524448	8.46564779	7.93124152
NPHP1	3.88093897	4.15798191	3.825418	3.825418	4.15288098	4.18763212	4.0208107	3.58153868	4.32890625	3.94622257	3.95743797	3.43436571	3.40304966
MALL	2.6819769	1.68784923	3.63975617	4.04129602	2.17450223	1.24472573	1.8108327	1.69504005	0.53414794	1.95886625	2.50287039	2.7491017	2.81596198
THNSL2	5.58034725	4.62285466	3.7421963	3.81665492	2.26370429	4.32582778	2.88876377	2.0728928	4.64556425	4.19220484	4.2269328	4.76155408	5.00610051
RALB	5.69766093	6.60334395	6.11361542	6.49451824	6.04249194	5.58917732	5.91095737	5.31051519	6.07717689	5.59002088	5.78332323	5.35482286	6.1858557

TMEM177	3.29209895	2.42808304	3.26863658	1.97200892	3.07923189	3.41303184	2.91431244	1.91086657	0.54549494	3.63821704	3.63371326	2.70169327	1.23206363
RABL2A	4.88203407	4.42784354	4.31055354	3.83523056	4.64654129	3.79992131	4.98859815	4.48417441	4.49207755	4.43437333	4.15778199	5.03999986	4.52666907
SLC20A1	5.94586653	5.89002625	6.59832694	7.54088413	5.08648287	4.23690709	4.83214614	5.02519349	5.22361753	3.72439329	5.27120567	4.33050102	4.72604755
FBLN7	3.87248289	3.16800718	3.92328681	2.55978677	3.62527652	3.09169221	3.41920141	4.87314426	3.66916783	4.60179545	3.1934794	4.36809912	3.33472652
ZC3H8	3.23172282	3.1958856	3.03549762	3.5766454	4.02214739	3.91674457	3.96217211	3.74694445	4.08929912	3.45995897	3.94554438	3.33924341	3.84722241
LIPT1	3.18868297	4.11253532	4.0219026	4.04737078	4.3698424	4.7332067	4.73056132	4.79441019	4.03528177	4.81225005	3.59203535	4.25261158	3.38411712
LYG1	1.59889937	1.09448197	1.44468962	1.51708926	1.40216214	1.29271168	2.93915531	2.94188933	2.21190247	-0.59735247	2.8636317	-0.59735247	1.59476031
AFF3	3.74480941	4.90641202	3.32924247	2.79825412	4.47465558	5.92811057	4.56890584	4.90481619	4.58322927	5.97677106	5.17326806	5.67000273	5.81798661
UBXN4	6.47657365	6.71563394	6.28975301	7.34971749	6.93229107	6.97644763	7.02300388	6.9825824	6.90283406	6.74823627	6.40599363	6.78062564	6.93257996
SPOPL	5.51663616	6.14294561	5.08409431	5.95443094	6.60243063	6.65030829	6.54205825	6.54205825	6.58852725	5.0457131	6.81810241	6.65681134	5.38859747
THSD7B	5.63220455	3.99963028	5.77996253	4.00972827	4.21708417	6.35439695	4.01670598	3.57994628	5.13213986	5.60114956	5.2342178	5.67645514	3.66363981
GPR17	0.10458069	0.35707226	0.89094768	0.07880339	0.07880339	0.08010827	0.25402341	0.08179756	0.87362455	0.03481315	0.03481315	0.03481315	0.03481315
POLR2D	2.86388913	3.32576684	4.09443054	3.34404356	2.89370964	2.96973442	3.92327615	3.079153	3.73213831	3.59410478	3.39159063	3.5264696	3.03512541
AMMECR1L	5.41065432	4.88962876	5.26316686	5.27732194	5.32663213	4.97960184	5.40742434	5.66423061	4.37053908	5.18247091	4.86311537	5.82291379	5.37848058
GALNT13	0.34023444	0.59049849	0.18916308	0.57539358	0.66361013	0.82370509	0.68643304	2.00943998	0	0	0	0	0
PKP4	3.3926148	3.78128786	2.55318055	3.11043274	3.19301792	3.34976953	3.77918301	3.36314933	3.89589147	3.56576448	3.41003652	3.29792201	3.24057605
SCN1A	0.05658585	0.05658585	0.13035031	0.05658585	0.0725682	0.12525501	0.12012469	0.05990814	1.08655849	-0.11076957	-0.11076957	-0.11076957	-0.11076957
SCRN3	4.69516455	5.23514662	4.44359776	5.44800729	5.27538951	5.83883895	5.10939845	5.30678383	5.59402432	5.21283473	5.34809191	5.38598148	5.34048921
ZNF385B	2.5055433	1.29629399	1.70389462	0.80354973	2.98214625	2.08847329	2.85697595	2.95780434	4.97056957	-0.87112277	3.60915478	4.12038603	5.17050398
CDCA7	1.316637	1.37715412	2.21123407	3.5313514	0.69188392	0.92314461	1.13710071	0.66723036	1.39492427	-0.75355187	2.61866269	-0.75355187	1.90358029
DLX1	-0.0472729	0.15820297	0.39515729	-0.02275189	0.53909362	0.00146921	2.33994991	0.34211333	1.09813917	-0.15512053	-0.15512053	-0.15512053	-0.15512053
UBR3	5.89379403	6.59102789	5.85873999	6.64684528	6.73345275	6.79681905	6.87271134	6.86523073	6.43764918	6.5711016	6.66986677	6.88982328	7.09831096
PHOSPHO2	2.43760074	3.3452965	2.34414651	3.03364237	3.66813191	3.55744813	4.06495131	4.16681359	3.70064282	2.42483917	2.73115207	4.01301928	1.70901992
GULP1	3.79381094	4.00130102	3.50685844	3.29635059	4.56041091	5.08424696	4.79159283	4.97246058	4.60019791	5.17270826	4.47219297	4.4443173	4.08007039
FAM171B	3.8241533	4.2101955	3.3625937	4.74614076	4.17174523	4.98181613	4.21872173	5.07902679	5.0668826	4.72299599	5.19975788	4.29845281	4.41715731
HSPD1	6.01301164	6.5950762	7.1014726	7.67181855	6.54132924	6.76172097	6.82086536	5.72585969	6.55187388	6.78343083	6.37585393	6.07563305	6.82994498
CCDC150	1.18830065	0.9762513	2.10600088	3.75050072	1.49190188	1.10684056	1.40876464	1.13318358	1.15002339	-0.78611369	2.73320936	0.25773778	3.90622366
METTL21A	2.97548561	3.33190649	3.02140355	3.40107169	3.52706597	3.89027553	3.31383398	3.40107169	3.48781574	3.14925714	3.66046369	2.59363109	3.27659499
UNC80	0.00430272	0	0.03960254	0.00081974	0.00752182	0.00069205	0.01700412	0.16684074	0	0	0	0	0
PTH2R	0.04259058	0.07227015	0.50730304	0.00455706	0.04481743	0.50993448	0.49042993	0.05222049	-0.08751529	-0.08751529	-0.08751529	2.12843729	-0.08751529
CPO	2.61073428	1.45065974	1.52125894	1.04866407	1.56683828	2.57302935	1.75248457	1.85303956	3.1164126	2.92052272	1.9709824	3.45033399	3.08045904
NBEAL1	3.10139923	4.39928003	3.22856957	3.86193773	5.1524018	4.93233374	4.64645566	4.98668152	4.7889178	3.33301993	4.49791541	5.28112774	4.12911896
SPAG16	4.61446441	4.76452788	4.65755915	5.15703002	5.60444821	6.13146407	5.54601621	5.41928945	5.70989359	5.00079544	5.61710188	5.63721333	3.64206574
ABCA12	0	0.12331045	0.00124472	0.05052003	0.00672213	0.03380766	0	0.00158797	0	0	0	0	0
SUMF1	4.58215502	4.24097211	4.08301085	3.92327411	3.1715044	3.85926371	3.39511933	3.66638451	4.39311267	4.77119317	3.49268153	3.49818101	4.23292629
RHBDD1	5.45173584	5.43867546	4.93867575	5.2344204	5.74007366	6.12138413	5.22241592	5.41616268	5.40381358	5.98672022	5.2027059	5.83969145	5.48451958
TRPM8	0.00422037	0.00422037	0.32595158	0.06620796	0.00422037	0.00564135	0.00422037	0.00748096	0.02149288	0.02149288	0.02149288	0.02149288	0.02149288
HES6	0.71598683	0.22152007	1.23476422	0.76349084	0.87669404	1.34073592	1.57126775	0.39702417	-0.31551624	-0.31551624	-0.31551624	1.09074588	-0.31551624
ESPNL	1.46707235	0.84136598	0.16963081	0.52555664	0.37480209	1.18413662	0.24342983	0.87025195	-0.55675632	-0.55675632	3.27950857	-0.55675632	-0.55675632
ANKMY1	4.39727322	3.89362614	4.36111751	3.66123138	3.36238499	2.81027992	3.50494634	3.44620978	3.92725347	3.84935607	3.32889128	3.12984834	4.24158579
COPS7B	3.9507121	3.39605171	3.98980552	3.15595737	3.45960559	2.72224456	3.62334262	3.77258837	2.93957604	2.91178769	4.27463221	3.51654702	3.24095679
DIS3L2	4.79760959	4.97982337	5.23896753	4.48960654	4.44388105	4.06408256	4.49544487	4.64577403	4.32058156	4.85090951	5.02101932	3.81276319	5.29608038
CPNE9	0.43385866	-0.15967215	0.75290974	0.35959833	-0.11633065	-0.01042025	-0.15967215	-0.03213163	-0.05018731	-0.05018731	-0.05018731	-0.05018731	-0.05018731
FANCD2	1.47007164	1.691445	3.4302452	3.69402882	1.16747001	0.87134068	1.75131539	1.61258376	3.32501261	3.31462947	2.31500636	1.02543412	3.46174645
TAMM41	4.18976075	3.88864957	3.53705812	4.23157164	4.6752352	3.86410227	4.20837799	4.87348622	3.80120728	3.84387607	4.17476724	4.08431747	4.90494301
VGLL4	6.99827016	6.35872815	6.85405464	6.51164005	6.61872543	6.43805775	7.01571354	6.79972386	6.62154425	6.97930383	7.07172192	6.99446497	6.88588252
RAB5A	5.95513521	6.50289511	5.79900453	6.87453653	6.97977913	6.9183211	6.62352274	6.81432257	6.98808639	6.63033199	6.38811657	6.60466518	6.25618789

CTDSP1	5.66659537	5.82885617	5.49669913	4.49744996	5.31293089	4.53396071	5.04667293	5.27798864	5.11548256	5.94546345	5.32740395	5.59534916	4.71180216
STK11IP	3.85285488	3.81297841	4.67238913	3.59687958	3.2948928	2.79264806	3.70051919	3.64675701	3.50863156	5.16863878	3.70891239	4.27132867	3.55725169
GMPPA	4.77004007	4.16442993	5.04223343	4.91263977	4.31892188	3.84131863	4.17889726	4.24895398	4.47415352	4.81942251	4.78497979	4.23559855	4.66012633
GRIP2	1.88556413	1.35312845	1.71195482	1.51639532	2.47957598	3.27663186	2.94004366	2.21691385	1.73799674	3.30199593	3.38931383	2.6488034	4.83807104
EAF1	5.74921371	5.19722711	5.97029011	6.04226032	5.61185676	5.38397061	6.18198763	4.95677372	5.29611621	5.51301953	5.08484208	5.50760058	4.91295811
CNTN4	2.75678422	2.77579632	2.49087122	0.62878303	4.74337408	5.33820573	4.20495093	5.53167689	3.38125019	4.18635667	3.73547329	5.04363606	5.10811957
DYNC1LI1	5.2352599	5.28839381	5.56078564	5.24515766	5.4108282	5.90033947	5.79852048	6.32984224	5.51781906	5.84993266	5.82870492	6.24780445	5.39828482
RBMS3	5.26763832	5.54035965	4.89161962	5.42888786	6.2749239	6.40880795	5.86579604	6.63265773	5.7307315	5.28577648	5.36944679	5.33544704	5.8754246
GADL1	0.54163589	3.41591133	0.6162933	0.49039679	1.88612813	1.0336548	2.16045392	1.43041893	0.52018708	3.5607338	3.48841041	4.02587783	-0.72861158
OSBPL10	5.00462532	4.68657069	4.66068587	4.1195696	4.98814167	4.19438078	4.82678892	5.30969989	5.13098314	4.35157031	5.25315796	4.72691531	4.76791162
FAM198A	2.79841693	2.84443453	2.3916496	2.1862014	3.10069905	3.04897397	3.96629707	3.25947026	2.1821448	3.3940858	3.04984986	3.99894095	1.19344112
CSRNP1	6.16496763	5.82219625	5.82219625	5.68058504	4.5708533	4.8876982	5.0913296	5.09058943	4.98753136	4.83536834	5.21027138	5.92709018	5.00111523
SLC25A38	5.65905903	5.21046504	5.0177483	4.63176374	5.04794395	5.15541004	5.35419256	5.18969941	5.28268191	5.37237299	5.35304398	5.65271116	5.01177778
ITGA9	5.40316869	5.71593567	5.76362359	4.85675573	5.91272391	6.65784288	6.60355519	6.67125453	6.05527522	6.87044052	5.61293522	6.66799795	6.02877804
SLC22A14	1.63253362	0.71268517	0.24129137	0.70845819	0.62411943	0.84223682	0.18337345	0.90649061	-0.24908136	-0.24908136	-0.24908136	-0.24908136	1.64622977
GOLGA4	3.9498274	4.64642064	4.51045772	5.43518576	5.13867828	5.46101548	5.10880846	5.02561008	4.59482989	5.0295461	4.59393884	5.20917797	5.10583864
CTDSPL	4.21460437	4.78196747	3.83053251	3.45814451	4.71167367	4.32219471	4.65331451	4.02218976	4.8559689	4.23331929	4.30193141	4.1164568	4.31586256
STAC	5.25195551	4.05057532	4.86684936	4.3029975	5.83682118	5.84418527	5.05489315	5.22989102	5.5375012	4.39868291	5.01428148	4.75716973	4.83546584
IQSEC1	7.2671186	6.88912208	7.16704403	6.19968753	6.95059388	6.26041989	6.67209156	6.60502913	6.78531852	7.02228599	7.21251532	6.67710176	7.1430321
CAND2	4.61110543	4.37047186	4.81233971	2.75223627	4.40805156	4.66991666	4.62050764	4.88111236	4.08395419	5.77414939	4.98130609	5.3296866	4.61377991
RPL32	7.74938031	8.38877618	7.28814923	8.11383149	7.57541568	7.6281074	7.48485102	7.41635662	7.71837841	8.2532541	7.38730368	7.87273462	7.60289494
PTPRG	3.96996406	4.02386434	4.18812515	4.24428957	4.84413901	5.56497645	4.48507318	4.66340959	3.91761957	4.7493196	4.72929831	4.53108259	3.39476387
IL17RD	4.73993928	5.09083123	4.52648873	3.74015268	4.38927882	4.34306812	4.85962267	6.32659271	5.33700173	5.26270369	5.13005732	5.2499694	5.43001726
SHQ1	4.52057305	4.67197885	4.42163361	5.6412192	4.55953972	4.30990536	4.63457399	4.1162212	4.49981737	3.88471954	4.6416746	3.78767345	4.78610958
SLC25A26	4.22317291	4.05212142	3.56837647	3.25876432	3.97164573	4.0306489	3.44832371	4.11833545	4.1764344	4.28209404	3.5399334	4.21630991	3.09991355
UBA3	5.33750081	5.67303922	5.21409873	6.18158953	6.01585291	6.27677357	6.02253086	6.10976022	6.35778363	5.95273667	5.42710581	6.20312546	5.88999857
ARL6IP5	7.056859	7.25719074	6.81391285	7.50698506	7.85186018	8.48853622	7.84290674	7.91586724	8.24847431	7.82948509	8.01663594	8.07047812	7.33420266
TMF1	5.53019661	6.09605111	5.84329154	6.70270778	6.33503977	6.53602091	6.2084062	6.31499229	6.65061701	6.00493393	5.4807468	6.03113677	5.78614535
LRIG1	7.50096386	7.29930551	7.26970701	6.58377573	6.81610646	6.51641136	7.06546809	6.86308387	6.51298801	7.32072957	6.58563556	7.04979277	7.31196254
LIMD1	5.59440499	5.85699144	6.24109405	5.91386563	5.54705783	5.33919481	5.86981519	5.50551918	5.2741808	6.15939207	5.85528399	6.2722032	5.64838708
ZNF660	4.5645983	4.85111229	4.26145369	4.92747508	5.21117501	5.32122432	4.71897081	4.9251193	5.08932293	4.93414811	4.93716039	4.92661857	4.50714869
NFKBIZ	7.11606732	7.02521657	5.7155315	6.59932129	5.76504588	5.74691069	5.95728123	5.06236355	5.64726871	3.94593356	5.31796747	4.97149114	6.39862417
COL8A1	7.85205596	8.13052167	8.31873183	7.46712055	9.39060331	9.09670535	8.56567815	7.65430411	8.62044058	7.43741985	9.03593255	8.0898334	8.16992623
MYH15	0.63548301	0.69058673	0.63808287	0.92989472	0.65080378	0.86483151	0.63548301	0.63879982	1.79036649	-0.33374157	-0.33374157	-0.33374157	2.63908767
PHLDB2	6.81646533	7.26146839	6.16829381	6.81799477	7.24061383	6.85908506	6.77030525	6.93659623	6.96208388	6.49341698	7.32994099	7.22476639	6.50278177
ABHD10	4.38018355	4.34770857	4.37051704	5.15395615	4.68641763	5.35134997	4.54796668	4.64462654	5.10342525	4.91359191	4.65719654	5.07258842	3.93592748
PLA1A	-0.04729601	1.54519345	1.36586799	0.45493349	1.59529886	0.83717261	1.42012355	0.98918658	4.30718058	-0.31247583	3.78056462	-0.31247583	2.83353152
RABL3	4.31772451	5.53233953	4.69302029	5.31543371	5.43498341	5.71811409	5.24505722	4.93950185	5.05818188	5.06567351	5.24306531	5.21543899	4.85136294
ADPRH	4.87926787	4.85857753	3.97101409	4.52172697	4.15956563	4.51410172	4.53902876	4.4949461	4.30896053	3.62475941	4.55298907	4.17072753	4.45734257
IGSF11	1.64702762	1.38709482	1.36962407	1.61398514	2.07690438	2.08546361	1.97175867	1.293132	2.40498774	-0.7953516	2.09555348	-0.7953516	3.48333949
ATG3	4.97753195	5.09641369	5.30469735	6.19423829	5.28322399	5.79553847	5.20897322	5.33917937	6.07483909	5.99615173	5.31696611	5.62828351	4.92423807
NR1I2	0.34251787	-0.09010302	0.00740798	-0.08497826	-0.04323042	0.18717456	0.09712963	0.27408416	-0.08131727	-0.08131727	-0.08131727	-0.08131727	-0.08131727
BOC	5.54892905	5.07878573	4.53884187	3.49996113	4.17238931	4.39983595	4.55447649	5.32240984	4.4782158	5.10793663	5.57399817	5.36420289	4.78673424
SRPRB	4.99395218	5.28510359	5.82006112	6.73272861	4.83450084	5.26300157	5.27890764	4.90011255	5.20082561	4.73964204	5.10069412	4.99840776	5.61251702
TMEM108	1.00234949	0.78170517	0.78647658	0.82411742	0.49413188	0.99912521	0.98802081	1.60857914	2.14720134	-0.28872078	3.47880091	1.97897833	2.79270908
AGTR1	2.86987628	2.52788697	2.76616723	2.65477546	4.57703073	4.52087301	4.32909927	5.9429364	3.42366276	5.31680255	3.87136244	4.80668675	4.69677098
MED12L	1.76567631	1.565426	1.69495704	1.96642062	1.83098698	2.30643856	2.28346092	2.52053589	0.13719363	2.79895663	1.85807824	3.0429797	1.83003435

EIF2A	5.63985861	6.72487578	5.79086308	7.08954032	7.02463304	6.99964327	6.84625688	6.9964536	6.89231464	6.69551959	6.18179117	6.94735228	6.33437264
ALDH1L1	3.44027596	1.77308593	4.12518453	0.54584668	2.9022504	4.35940865	3.29002605	5.32248251	2.81449233	3.42659054	4.14749598	4.67022502	3.83755973
OSBPL11	4.75250179	5.29396133	4.6987511	5.76175845	5.03247013	5.39873283	5.12558373	4.41299055	4.91468384	4.20841884	4.87665836	5.09122986	5.72684523
TRPC1	5.00126836	6.02582097	5.02444792	5.40556043	6.04996485	6.15908815	5.97863468	6.23473346	6.11977983	6.08847079	6.06812177	6.33320789	5.87136675
NCEH1	4.57579378	4.10156977	4.91644062	4.6431755	3.78770711	4.73298352	4.25243555	3.92152911	4.42712528	4.64867247	4.52148307	4.35751797	4.35196093
FAM86B2	1.68626525	0.29483396	1.66493525	0.6747648	1.09694349	0.72875436	1.32345927	0.64486433	1.63164214	-0.51343637	1.45392718	1.89293495	3.1991864
LPP	4.46608178	4.96973036	4.05366647	4.21449987	5.98392405	6.10458084	5.7289206	6.11596978	5.4734775	6.91186248	5.57469962	5.91067058	5.30837949
TMEM44	4.47640766	3.67746728	4.47399752	4.73701506	4.11434076	2.06362141	3.35497744	3.29134427	4.1299546	3.19694788	5.30470305	1.90127086	4.44998276
AMT	3.31741096	3.40958881	3.39457648	3.01415166	3.40888415	3.52214078	3.59849745	3.49166299	3.36837872	4.68227422	3.96704357	4.29795028	3.82582633
TCTA	5.35278455	5.18203965	5.37259472	4.89161378	4.93560805	5.09594085	4.80800279	4.33530239	4.70703125	5.52186898	5.50776837	5.32482276	5.29703808
UCN2	0.02866362	0.27118709	0.20262785	0.62673547	0.11134932	0.56896249	0.25667965	-0.24339838	-0.06535209	-0.06535209	1.5127547	-0.06535209	-0.06535209
MANF	5.65616126	5.58517617	6.03590282	6.27088638	5.36174494	5.60648075	5.36420305	4.93479241	5.97668182	4.63476851	5.69472157	5.03692181	5.84082926
CCDC39	2.07821989	0.70641336	1.62620164	0.82039705	1.23325161	1.84508345	2.0530982	3.68197736	-0.4232476	3.67427948	4.11248816	-0.4232476	-0.4232476
STXBP5L	-0.00940033	0.16668239	-0.00681675	-0.00940033	0.00443131	-0.0079639	-0.00940033	-0.00610429	-0.08348824	-0.08348824	-0.08348824	-0.08348824	-0.08348824
EAF2	1.56768671	3.38049247	1.65772238	3.34718201	2.71690609	3.82552728	3.76229304	3.34718201	3.79952133	3.40752492	-1.10046846	2.89405437	3.27817484
TM4SF19	0.39369652	-0.11304152	0.52858025	0.44082929	-0.11304152	-0.11162254	-0.11304152	-0.10978554	-0.30834274	-0.30834274	-0.30834274	-0.30834274	-0.30834274
SLIT2	4.3525538	4.50408027	3.77459106	4.86269192	5.2827915	5.80249489	6.1551727	6.09604414	5.55841538	6.13208084	5.64250404	5.66872841	6.01964533
EIF2B5	5.61707416	5.35770582	5.59439133	5.04128634	5.42553141	4.92943543	5.87670035	5.3959555	5.34417842	5.09740835	5.40219248	5.24494113	5.59874667
ECE2	1.70405585	1.42500652	2.87874524	1.85395475	1.71130474	1.98766109	1.5686969	2.3198592	-0.80668656	3.11409473	2.8986307	0.95061725	1.89124988
DGKQ	3.88042295	3.13303696	4.96283244	2.14743914	3.40128129	2.70811061	3.31129084	3.92031159	3.45574907	3.03873861	4.49357599	3.64728855	3.51850163
FIP1L1	5.40189687	5.89130726	5.12288977	6.38577506	6.19868636	6.22863964	6.15962102	5.81242373	5.91144961	5.79318968	5.6124486	5.06416159	6.51269148
SLC26A1	0.42982369	0.25050071	0.43040601	0.25955657	0.69797082	0.51117434	0.59673766	0.51957383	1.3587418	-0.16935457	3.19398413	-0.16935457	-0.16935457
LYAR	3.95462155	3.86346896	4.6157577	4.36995819	3.95793475	5.41015002	4.03504009	3.49839044	4.52165988	2.61837449	3.79992514	3.30506716	4.72397896
EPHA5	1.56321288	1.53100722	1.50340769	1.45147773	1.42721127	2.53320767	1.4742873	1.50968514	4.04735979	2.55784352	3.06534739	3.00692802	3.25707565
CORIN	1.00056065	0.81085571	0.57923858	0.30323205	1.27304988	0.9219499	1.01844299	2.64614551	0.34372392	-0.23107693	-0.23107693	1.79154026	7.25987327
ATP10D	4.21444339	4.80240602	3.9826076	4.44990277	5.30677309	5.11587651	4.93287913	5.91233167	4.72365711	4.43772333	4.96020195	5.00023017	5.03464494
OCIAD2	3.87247988	3.324032	4.52403308	4.17916531	3.89339938	3.74936497	4.02647147	4.79353856	4.22292933	2.94676478	4.21041644	4.08139996	4.35845963
SLC10A4	3.976809	2.12317223	4.57554497	2.61517454	3.94508601	3.55276389	2.12317223	2.54414002	5.00083806	2.75369567	5.99941615	3.78259354	4.74330212
SLC10A6	0.77265936	0.90055039	1.34468871	1.08180497	0.72003238	1.49124882	0.77913315	0.32254986	-0.32967508	-0.32967508	2.86426469	2.94009434	1.18218534
SCD5	4.76631743	4.44266485	4.97698643	4.2838113	3.94800285	4.70703442	5.12347173	4.23922123	3.96076042	4.94953326	4.19269486	4.80336975	3.80268933
PLAC8	0.97150038	1.90260595	0.71087503	1.76001987	0.77025926	0.56460373	1.15384483	0.68428758	2.2383299	-0.56863563	-0.56863563	2.54752319	3.4321615
ENOPH1	4.44948979	4.05148278	4.55020926	5.16528163	4.5163679	5.354332	5.05229511	4.72303016	5.51172306	4.5889472	4.75989862	5.23835438	4.65171031
KLHL8	4.44408742	4.64579066	4.23301094	4.78982	4.28838006	5.07312922	5.19812792	4.77269926	4.77949495	4.33628162	4.33047358	4.42199603	4.19593761
SNCA	1.98301038	3.37413475	2.10913733	2.82460431	2.6404939	2.94963817	2.65500126	3.32704245	2.78908213	4.76329392	3.50350782	3.92591733	3.52498647
TBCK	5.17861909	5.52654531	4.97866298	5.65045176	5.82027203	6.41183587	6.31295854	5.91858021	5.77069239	6.0115717	6.02929208	6.33040086	5.00818689
CAMK2D	6.72417394	6.38406036	6.51063123	7.73784586	6.55901527	6.536775	6.96185629	6.81965544	6.43515088	6.21447579	6.59273118	6.49466945	6.66280356
CISD2	4.71121664	4.75716605	4.29525123	5.48727923	4.4778748	5.08955679	5.60984011	5.17420844	5.1598808	5.15188264	5.23772827	4.12339696	4.53948492
DDIT4L	3.24597194	3.06109101	2.7930752	5.84921578	3.54162172	3.86132445	3.61880869	3.49368055	4.33145116	3.84992031	4.80461229	3.15932504	2.80172615
ANK2	1.95114088	2.69087007	1.12780071	1.02423966	1.35362865	2.975683	1.94520534	1.89634167	2.01250615	3.11130409	-0.16460347	1.88117738	2.30447238
TIFA	4.57975831	4.84374946	3.88605065	4.25903551	4.28769848	5.41477364	4.37380288	4.73212245	4.29208528	3.58660329	4.25403359	4.05109901	3.89431532
SPATA5	4.45669039	4.95349285	4.38176259	4.78388551	4.97986922	5.22166037	4.83428566	4.97659411	5.13201254	5.09415396	4.49291614	5.00018201	4.86670005
CCNA2	2.4789524	2.64851772	4.52210555	5.25521273	2.87384106	3.32305529	3.65423498	3.21624225	2.7837588	1.53717964	4.10283609	3.41156897	3.6341998
METTL14	5.45300147	5.42139731	5.13477824	5.51559862	5.79447466	6.21040716	5.71888387	5.82667187	6.00031595	5.54692489	5.57178832	5.4925733	5.40860133
USP53	6.60907161	6.71137799	6.44183455	6.99448466	7.73691414	8.03608479	7.54337118	8.3144906	7.11805971	6.57745308	6.56813077	7.12411721	7.01300342
SETD7	8.01638299	7.5516679	7.57797359	7.40255217	7.59223856	8.09810509	8.14015091	7.87728537	7.67781009	7.60528296	7.70349585	7.71156743	7.4062813
NAF1	1.17604835	0.4382085	0.01366741	0.89919433	2.26476163	1.83191403	1.70667259	1.61792304	3.39017385	0.44574319	0.44574319	0.44574319	0.44574319
SFRP2	3.16748059	5.01172684	2.38332078	8.86139843	6.51084965	3.39009507	6.54122548	3.85131311	5.1639333	7.25194697	5.55096667	6.20100208	6.69324807

RPS3A	7.29378157	8.28581286	7.31826944	8.59731267	7.59836714	7.33016891	8.09082675	7.80031819	7.99316176	8.37258591	7.43433933	7.99316176	8.02835089
RNF175	1.82705776	0.75933701	0.73228495	0.96662359	0.56969264	0.54130579	0.5819684	1.33897441	-0.66180793	-0.66180793	-0.66180793	1.77589092	2.80344681
PDGFC	6.06312466	6.82971641	5.86399705	5.98857764	6.82628043	7.01552438	6.5922613	6.79212838	6.91781724	6.56386482	7.23036453	6.52571848	6.89341642
CBR4	-0.13396714	0.46702841	-0.08410459	0.62694135	1.06391716	1.62270361	1.1526866	0.61674891	0.34815649	0.34815649	0.34815649	0.34815649	0.34815649
CYP4V2	5.84218655	5.39405032	5.16410615	5.18647048	5.68518541	5.56931909	6.24268597	5.0586819	6.30104163	5.20582265	6.0400335	5.15180359	5.84834909
ROPN1L	0.54054952	0.05973416	-0.01753802	-0.00628616	1.00613044	0.36064273	-0.25522196	-0.03504988	-0.13184671	-0.13184671	-0.13184671	-0.13184671	3.16791617
NDUFS6	3.49868558	2.79491642	3.94196722	3.50088896	3.25753618	3.94565899	2.52486029	3.26292654	3.68376219	2.39721565	3.53822886	3.01283508	3.34779165
NKD2	0.22363501	1.26803222	0.22616024	0.49693509	0.22363501	0.73484353	1.16576727	0.45314535	-0.2033673	3.1186608	3.3225815	2.31669216	3.80047265
ADAMTS16	0.06191635	0.1832493	0.06418695	1.60018303	0.07872995	0.06317877	0.06191635	0.0648131	0.08219622	0.08219622	0.08219622	0.08219622	3.26944996
SRD5A1	2.96333972	3.54139575	3.52307668	3.93013256	3.84385384	4.10683272	4.02921977	4.18366249	4.45411066	2.93775364	4.32147464	4.20720659	3.71092013
MYO10	5.6320472	4.7739881	5.69138289	6.27542276	6.35671985	5.35866283	4.39285127	3.97082014	5.18038505	2.78190744	5.59581984	2.99137875	5.94051669
FAM105A	3.21587148	4.41263225	3.42624233	5.00339018	3.70660525	3.05921173	3.61663447	3.40528754	4.1368439	3.8609838	4.04599708	3.97012794	4.45906259
RPL37	5.6228718	5.05296209	4.96612477	5.17377994	5.10527145	5.6224482	4.48349119	4.71643273	5.84169974	5.83102483	5.04156619	4.50134992	5.428663
SKP2	3.94000488	4.15055908	4.19027585	4.99962481	3.81201966	4.8606679	4.54239964	4.19411202	4.41168438	3.95058437	5.04776796	4.44214558	4.34890234
OSMR	7.78770988	6.89195246	6.75789968	7.62488402	7.0319201	5.88597157	7.24272899	6.03906083	6.17973884	6.06871633	6.29359806	5.99669939	6.41915021
PLK2	5.11284533	6.07865122	4.90326022	6.66799756	5.69607466	6.70408461	6.42729049	5.92243211	6.99491074	5.96604959	6.76432971	5.91407202	6.29675426
GZMA	3.70318446	1.25064732	0.98432772	2.3545743	3.00447801	1.47721425	1.277864	2.27456732	2.78966186	-0.79998552	2.65487988	2.20701286	1.95646184
PIK3R1	7.4088831	8.04596867	6.61734207	7.21468651	7.8542804	8.81109418	8.03781913	8.014557	7.81221289	6.97279507	7.5068749	7.55423922	7.80742345
HAPLN1	5.60763447	5.21005658	5.98167106	6.01832156	5.67432778	4.98332616	4.56972827	7.19594375	6.56401742	6.3559818	5.90419459	6.70197681	5.82575598
LHFPL2	5.85998706	5.78610127	6.46526223	6.98215889	5.00728012	4.98689684	5.14911284	5.85722152	5.42327694	4.6996096	5.49451991	5.03190632	5.22299916
SSBP2	5.83557597	5.91517637	5.28209062	5.74234793	6.34347574	6.7006644	6.30665781	6.19866498	5.9615863	6.04307649	6.00448521	6.30361773	5.43776617
BHMT	0.48232985	0.29590973	0.47731664	0.493091	0.8747945	0.73616569	0.98197285	1.48911747	-0.27257899	-0.27257899	-0.27257899	-0.27257899	-0.27257899
ANKRD31	1.5826079	1.61800337	1.64989139	1.63669373	1.75895952	2.97662634	1.71463575	1.83215118	3.21267175	-0.88321154	3.50569746	3.54049358	-0.88321154
IQGAP2	2.10468846	2.98320863	1.70470086	3.50914502	1.72409544	0.70477468	1.23728423	1.09437981	3.1389914	-0.37893716	3.61157556	2.49645245	3.32791977
CRHBP	2.22239673	3.30963127	0.55580935	1.02429717	1.76686773	1.49605278	1.05423062	0.94685985	1.16403103	-0.63444078	2.43851689	1.99646446	-0.63444078
RASA1	5.36117508	6.03339744	5.89027456	6.60636223	6.05510945	6.23075199	6.10641949	6.15328835	6.12114807	6.036171	6.57670148	6.04817036	5.91070689
GIN1	3.59837799	4.30469296	3.86597063	4.27503934	4.6533586	5.30525188	4.64083793	4.48751629	5.03467427	4.2610456	4.45332175	4.37368714	3.81542347
PPIP5K2	5.18481618	6.05865934	5.31266368	6.4042226	6.42911479	6.74023762	6.43263722	6.67904875	6.57807035	5.2938397	6.20567445	6.43917931	5.48315385
PAM	8.19540924	8.35123076	8.14586242	7.94639916	9.00556838	9.22055478	9.08546335	9.24829896	8.68314915	9.02843332	8.71035307	9.09830504	9.54976398
BDP1	4.18980563	4.74474345	4.86891373	5.14687622	5.27151335	5.57174161	4.95986117	5.15949116	5.57130948	4.25737057	4.49606997	4.88674636	4.99290134
SLC30A5	4.79239477	5.30481467	4.97255256	5.97614412	5.47449072	5.6839734	5.91350107	5.59701839	5.53023944	5.02233171	5.83882582	5.24197312	4.65555917
BTF3	7.31158513	7.35506527	6.86896515	7.32568796	7.20585109	7.98083396	6.78067981	7.49513515	7.55716994	7.63626595	6.91485641	7.21388183	7.09793674
FBXL17	5.20216438	5.59207211	4.87962558	5.19016297	5.73421046	6.12550158	6.13201023	5.73403182	5.86407758	5.58940617	6.01777096	6.29168102	5.27412535
SPATA9	0.753795	0.40973432	1.0562333	1.24057533	0.590329	1.06445462	0.55088056	0.26016199	-0.3386873	-0.3386873	-0.3386873	-0.3386873	-0.3386873
TSLP	2.24825787	2.451386	2.29102422	4.70282622	2.67551937	4.10796809	2.87198792	2.81137399	-0.06915007	2.7240025	2.28347851	2.39921842	1.70723534
TNFAIP8	2.52997078	2.88993203	2.91221802	4.06353625	2.72034835	3.19654662	3.01833359	2.13005999	2.57316699	2.4083918	1.98050506	2.57621245	3.13903978
FEM1C	5.75862275	5.62692286	5.59398966	6.4069124	6.49918489	6.46253136	6.16591051	6.45011948	5.96305519	5.45371915	6.14104508	6.15423263	6.07180967
COMMD10	3.71030617	4.85375325	3.75735997	4.97550209	4.81316083	4.61610828	4.85094903	4.53678836	4.46325026	4.67250255	3.44065274	4.53856963	4.69271182
ATG12	5.34877142	5.51730411	5.17909687	6.08008106	6.18739896	5.97805358	6.2917528	5.95255472	6.07185835	5.44924839	5.98650135	5.72462418	5.75704426
MEGF10	4.26804508	3.37400356	3.84921811	3.15713848	4.0209342	3.895663	3.12412734	3.20694898	5.44198711	2.03324869	6.48435799	3.98388762	4.62267444
ADAMTS19	2.78704795	2.82798181	2.91901015	2.66747715	3.87655802	3.30950653	3.54310404	4.59057469	3.33744687	4.36260262	1.88933421	3.68712654	1.44603246
YIPF5	5.23746091	5.58347053	5.10212662	6.43642499	6.00180587	5.84249933	5.81439031	5.47068174	6.42905918	5.85335926	5.97651536	5.85172127	5.44554707
ARHGAP26	3.17976673	3.01656709	3.00656772	3.33699205	3.67187949	3.8443206	3.03399851	4.16995537	2.72829313	2.54856241	4.1749365	3.2646667	3.24221116
CXCL14	0.48562063	7.88744918	0.48772364	1.47005831	6.50145655	3.96505279	5.08522121	1.96712776	6.31435892	7.07441331	7.12344533	5.4448926	7.22254694
DDX46	5.2996186	5.73913694	5.74003257	6.42050402	6.01620719	6.29209134	5.8477306	6.18944774	5.91225682	5.9034085	5.71219825	5.51762086	6.57747064
TIMD4	0.72434894	0.11307741	0.21956082	2.11520668	1.05803559	-0.15217526	-0.31005758	0.01688356	0.09098104	0.09098104	0.09098104	0.09098104	0.09098104
RNF145	6.08014697	6.42444525	5.98084943	6.85849137	6.37963936	5.89728765	6.85849137	5.94312432	6.00922728	5.82888791	5.85350083	6.1593522	6.18928361

C1QTNF2	4.51792142	3.76370546	4.70839972	2.53591701	4.25528612	5.48923966	3.98343438	5.12037437	5.08513123	6.07426585	5.45303716	5.34121993	3.92560551
GABRB2	0.92956807	3.75386332	0.99231884	1.43127743	1.53137724	1.60447614	1.346778	1.48367473	-0.73185812	-0.73185812	2.50373306	2.09470666	4.01749214
FBXO38	5.13315758	5.29187444	5.08163771	5.93228677	5.57824431	5.77140667	5.53259042	5.96341178	5.59149752	5.44493631	5.42934087	5.60377695	6.00550337
PCYOX1L	3.78918813	3.05516007	4.02643993	3.2437584	3.11914191	3.10694819	2.99190893	3.45471645	2.86604044	4.12002605	3.94626008	3.78483934	3.93352138
TNIP1	7.02269048	6.90533725	7.07099178	6.16594516	6.28724186	6.32637495	6.09012596	6.11244595	6.70765575	6.50223897	6.16572953	6.00552903	6.76311991
G3BP1	6.14190152	7.13886938	6.57685839	7.64351148	7.13995512	7.10278167	7.26282292	7.04242511	6.97194325	6.17447896	7.27992344	6.96796701	6.44489343
ZNF300	3.71207337	4.05222873	3.90328739	5.11825318	4.05382911	4.6615003	4.72292854	4.68067996	4.22934122	3.62896454	4.57055732	3.91821198	3.60802864
N4BP3	0.44650028	1.96774028	2.12619575	2.32531057	2.14822825	0.53782055	0.61186662	1.13607308	2.36752913	-0.55667567	3.17128791	1.27400836	2.99445098
NHP2	4.4181811	4.04101096	4.42961224	4.07108414	3.93440628	4.68872717	3.98664047	3.00824146	4.59397402	4.67679895	4.21877246	4.1557643	3.06588243
RMND5B	5.07725176	5.08581861	4.96272197	4.05419775	4.92261647	4.62341023	4.76306772	4.78353128	4.74866548	5.23232301	4.47318014	4.71788594	4.84829122
BOD1	5.59875387	5.81750207	5.09765026	5.26733862	4.97065537	5.6576975	5.17377321	4.56249119	5.61351736	5.23515392	5.06036454	5.26880781	5.67383871
KCNMB1	5.78792093	5.76703831	6.08548744	4.4663543	6.34784465	6.49456659	6.11024135	6.19384178	6.34443324	6.80823177	5.81807262	6.02535571	5.9750879
FAM50B	4.85160064	4.0649564	4.7642563	3.342952	4.54018719	5.27590187	4.08411123	3.9702551	4.20894913	5.62947334	4.25350092	4.76587133	4.81116625
MYLK4	3.38151155	3.54288756	3.05616045	2.58451997	2.73546749	3.47352574	2.80786439	3.8530923	3.24331741	4.24890078	3.72307891	2.83184575	3.9046849
TBC1D7	3.38952472	3.02441608	2.9741203	4.05816624	2.92893467	3.10630097	3.17052479	3.29655692	3.24409661	-1.07518258	4.30970876	3.56788433	4.23012494
FARS2	4.71089712	4.15071886	4.38055487	4.57947376	3.58916078	3.95986153	4.15738953	3.6659197	4.20381501	4.81070014	4.48924867	4.7302173	3.56892745
GFOD1	3.85426792	4.72652409	4.08529688	3.5891825	5.41916722	4.70825516	4.33933396	4.51464785	3.6444451	3.80923325	4.94972845	4.11102303	5.72670553
CDKAL1	4.35255073	4.57421186	3.82857464	4.63939562	5.04166952	4.78588498	5.01649153	4.50199801	4.63845798	4.36153579	3.68460395	4.53039803	5.49529837
PSD2	3.21142747	1.72595745	2.4304802	1.18983245	1.75867229	1.68625848	1.59558485	1.76770334	-0.05191687	-0.66229503	3.71205417	3.61381644	2.16453103
LRRTM2	0.92361161	0.99711417	1.03168952	0.90385811	1.19746914	1.21051068	1.15871033	1.33575518	2.9500208	-0.47959744	3.32835806	3.07334381	3.0826864
ZMAT2	5.92251447	6.16127711	6.48930689	6.45037544	6.20263246	6.53534986	6.36572832	5.91677683	6.41857274	6.51217105	5.91485846	5.91846754	6.59532143
GFRA3	0.20446334	0.20446334	0.20703745	0.50324578	0.90807676	0.74753341	0.92929803	0.35467353	-0.32089599	3.57991463	-0.32089599	-0.32089599	-0.32089599
KLHL3	5.0706355	4.26119917	4.39053753	4.35641011	4.8573398	4.7982859	4.56222188	5.21423417	5.04554532	4.2813171	5.61926233	5.15299893	5.97773205
DCDC2	0.37603325	0.46778973	0.59228715	0.34349483	0.34349483	0.34494117	0.37850176	0.3468136	1.94711086	-0.24970443	-0.24970443	-0.24970443	-0.24970443
TRIM7	3.11920659	2.11775054	3.94294381	2.48367198	2.5204604	3.13661896	3.36355498	3.73148823	3.70999925	3.4305395	3.55150543	2.62680816	3.35003956
TRIM41	5.01074563	4.18699759	5.50625023	4.20047294	4.02362691	4.23445952	4.39387813	4.23178542	3.77899788	5.1284949	4.70576207	4.06570919	4.14979043
HIGD2A	4.51194486	4.25926304	4.90993173	4.55873759	3.25575064	4.17999687	3.70635936	3.65471401	4.11375354	4.42596271	3.17715311	4.48240846	3.50817737
FAM193B	5.58604619	5.21199746	5.67615088	4.62006904	5.35221167	4.12081642	5.49890373	5.77958993	5.14766661	5.53066199	5.54088269	4.74506713	4.33909901
PLA2G7	4.36043775	2.27395288	3.46423735	4.65933789	2.09010162	1.62786464	1.42945417	0.96244209	2.96280206	-1.51412758	2.0102048	-0.0241338	3.38501348
TNFRSF21	4.06374753	5.31804399	3.45320413	5.40409444	3.25623239	2.8179672	2.7878217	2.49528634	2.26128036	3.47423357	3.94027657	4.10312784	4.70200222
RNF44	4.9709349	4.28276002	5.11698016	4.0032251	4.04168997	3.61928155	4.15424226	4.01781787	3.87040841	4.38147622	3.8302367	3.63233336	3.77538035
MUT	5.26659207	5.69694513	4.95932421	5.34877618	5.92637654	6.34556676	6.01354471	6.28433497	5.92908693	5.92923636	6.17080717	6.1622159	5.56153374
RASGEF1C	0.23063752	0.23063752	0.23318896	0.23063752	0.2745361	0.23205609	0.55588587	0.98511461	1.39500007	3.32084588	-0.15006382	-0.15006382	-0.15006382
DOK3	4.40301987	3.45968466	5.02068003	4.0785917	2.81977038	1.34372774	3.13831343	1.82813803	2.48415444	3.70361372	3.26190027	0.8063347	3.99417033
ABT1	5.2725545	4.43917243	4.70035253	4.08432032	4.43461856	4.69396289	3.93305071	4.32511209	4.16221815	3.78059241	4.43470357	5.15231983	3.2859539
PPP1R18	8.11148763	6.97533624	7.68282344	6.89509524	6.79439739	5.9274583	6.59829084	6.01421481	6.25668694	6.59039781	6.36766302	6.20048212	6.36766302
DAAM2	6.10445113	6.28951239	6.29826097	5.05462361	6.08102646	6.56526171	6.2626511	6.38521886	6.21718909	7.08067013	6.29476603	6.66095167	6.0576555
PRIM2	2.37159281	2.99188139	3.71382759	4.59675365	3.97621574	4.0478761	3.78105958	4.5181711	3.844204	3.28289544	3.86397025	4.33266522	-0.35539745
MLIP	2.18715505	2.34487049	2.74295411	2.26390913	2.78092712	2.34655222	2.38070467	2.41850228	3.75496526	2.04987085	2.82650579	3.02981066	7.51400483
HMGCLL1	0.6092105	1.25227881	0.58277185	0.59927794	1.28816145	1.37706284	0.95273507	1.15945728	3.09715657	3.45601691	4.26437049	-0.40737533	-0.40737533
FGD2	3.71775486	4.35937327	4.98866538	4.22380844	3.14308097	2.0752775	2.90306857	2.11382451	3.87835663	-1.318107	3.67011883	3.78796564	2.76491403
SCUBE3	6.92330115	6.1400866	7.29684619	5.62508929	7.42126775	7.79412328	7.78062226	7.52478436	7.53116388	8.00567597	6.97138334	7.91384582	6.88883365
ANO7	1.46325585	1.12824504	1.89059689	1.14958022	0.4826511	0.85123839	0.99809585	0.87508174	-0.49863623	-0.49863623	-0.49863623	3.25227735	-0.49863623
CRIP3	0.16441105	-0.33945017	1.04525856	0.14076351	0.34504238	0.38241498	0.99435576	0.25753369	0.03188299	0.03188299	1.83044407	0.03188299	0.03188299
TTBK1	0.65286437	0.64405055	0.72626818	0.67890381	0.66912178	0.76008223	0.7902865	0.7285935	-0.40159686	-0.40159686	3.01203101	2.55992115	-0.40159686
TCTE1	0.77052557	0.28152161	0.40515665	0.29625746	0.58068422	0.28296027	0.32549358	0.65771863	1.84672927	-0.3079803	-0.3079803	1.91131575	2.42991018
RPL7L1	7.0219141	6.73149091	7.1389988	7.45501602	6.68659471	6.35829212	7.02470507	6.60387775	6.72592529	7.13655612	6.63545479	6.7558651	7.03913148

NFKBIE	3.61904842	3.6298078	4.55147097	4.09584747	2.57607042	2.59783604	2.79762572	2.0628988	1.51979292	1.66321397	3.05751881	2.97050203	2.66631104
CYP39A1	2.06765801	3.9627749	2.02415044	3.1033488	2.93849985	3.05701644	2.74122803	2.75075783	3.3722836	-1.50957479	2.48567254	2.75797521	2.71387177
TPBG	5.17250411	4.3713053	5.47385604	5.22126822	4.17980784	4.6990769	4.56821935	4.60059169	4.77931228	4.69687675	4.72693562	4.06206713	4.48459194
IRAK1BP1	3.41029905	4.19617923	3.2759251	3.44702526	3.95291837	4.37484028	4.15917692	3.39688234	4.72982386	2.86077446	4.91298906	3.52940632	2.23852852
PHIP	5.89606028	6.27356114	5.51227426	6.44405046	6.35864548	7.03213562	6.60244317	6.5482471	6.55043386	5.46032452	6.35015411	6.30374979	5.36207657
PRSS35	3.6832537	4.09074719	3.98963789	6.21674206	4.12846351	4.44915361	4.57535558	5.11731705	5.74016069	5.36654693	4.14694532	5.81543629	4.82669358
MMS22L	3.17114766	4.03564245	3.70404777	5.48057808	4.1377066	4.11143218	4.52970662	4.07847694	3.99358003	3.71644183	4.31284038	4.40598275	3.73351281
GABRR1	1.06786766	2.35545456	1.05142139	1.08285797	1.35212332	4.93555525	1.83763905	1.37288467	0.97494572	4.45186788	-1.55883704	-1.55883704	-1.55883704
PNRC1	7.11539763	7.76931358	7.53265523	7.44028479	8.32823723	8.30657121	8.11800821	8.09417739	7.6581367	7.12679043	7.57144356	7.59643725	6.56591115
PM20D2	6.31385979	6.12141632	5.88389317	5.10374781	6.38338752	6.7499871	6.82039364	6.39145691	5.958088	5.57616926	5.91557211	5.70594994	5.70131984
RARS2	5.19991463	5.74603578	5.1943344	6.25401942	5.8736705	5.66967993	6.00284545	6.06868143	6.26497091	5.78763918	5.81013619	5.21720102	5.50843421
SCML4	1.51824939	0.58100146	0.99181708	0.71453074	0.91554712	0.83581637	1.30469389	0.56280277	3.6251412	-0.35521237	-0.35521237	-0.35521237	-0.35521237
CLVS2	0.61539623	0.56652617	0.65458627	0.57056821	0.57981049	0.65230242	0.61324395	0.56985189	-0.32631068	-0.32631068	2.39473576	-0.32631068	2.17163919
RNF217	6.36443272	6.34739247	6.09364212	6.39501109	7.01319804	6.6253384	7.03040774	7.06692036	6.71194047	6.00059982	6.9677549	6.74135527	6.13323355
RSPO3	1.40421512	1.09355028	1.03909897	1.98261262	2.12534821	1.73333869	2.08552379	2.19083095	3.99326831	-0.58821277	3.56869552	3.85352328	4.07732745
ARHGAP18	5.27141591	6.7313692	5.75886445	7.19572904	5.29202009	4.82121761	5.34603875	4.76304072	5.9800688	4.43801549	6.33375377	4.73855416	5.48183481
SLC2A12	5.07286617	4.92479978	4.06670587	3.8440556	4.3755735	6.14292579	5.01252141	5.27753739	5.21997821	4.88672302	5.0446562	5.68718734	4.85872543
SHPRH	5.28672323	5.52280894	5.0206757	5.65314539	5.68906547	6.06969688	5.86099845	5.74542848	5.25327147	5.51090345	5.73689382	5.67247797	5.97607005
AIG1	5.45885521	5.59381922	5.07392818	4.87596065	5.54684909	5.96961982	5.3823091	5.73835351	5.51357502	6.07051493	5.72650729	5.49019714	5.76052689
DYNLT1	4.66904651	4.44636501	4.65892282	5.46757821	4.58918481	4.7569716	4.27743664	4.21795251	4.89290101	4.4475754	4.33854713	4.91683898	4.42985454
TIAM2	4.02958109	3.31178661	3.92269728	3.55819754	4.56049691	3.61879498	3.95809136	4.18913001	3.77995803	4.15410842	4.28474694	4.47792984	4.41612824
TMEM181	4.73739168	5.15773278	4.55934132	5.09877038	5.046727	5.21834474	5.42571375	5.04905581	4.82490042	5.25893151	4.57227807	4.74113943	5.10705504
PNLDC1	0.98379242	0.18832397	1.26110191	0.95326099	1.27064847	0.66587289	2.73322038	1.42793528	-0.28259865	-0.28259865	-0.28259865	3.1273477	2.36924904
WTAP	6.3039745	5.94656255	5.79310003	6.84390869	6.17999422	6.27237795	5.9646621	6.47528806	5.82388128	6.15672647	5.56727486	6.12049065	6.10208202
ZMYM4	2.45144541	3.73296897	2.22785242	4.16622645	3.57533684	3.89575427	3.69557419	3.44095409	3.65152622	-0.58040366	3.35535459	4.89806071	3.66258199
VIP	0.12028385	0.42732832	0.12281622	0.73970542	0.68635279	0.32771164	0.99197301	0.29819112	2.47415358	-0.07755293	-0.07755293	-0.07755293	-0.07755293
SLC22A3	6.87804304	6.52245979	6.83948204	5.77817445	7.35166366	7.45370912	7.38098367	7.85560917	6.51260979	7.81215556	6.8933474	7.8701932	7.42543191
VWDE	0.39811681	0.56138776	0.40072287	0.39811681	0.39811681	0.48265806	0.47435455	0.40144153	-0.23285564	-0.23285564	-0.23285564	-0.23285564	5.78014362
GNA12	6.56072312	6.54852041	6.65829429	6.19753817	6.08622738	5.63644969	5.86477147	5.91636288	6.12741553	6.27612801	6.51690426	6.05747566	5.89189102
C7orf50	4.95220384	4.97917076	5.69188166	4.02894051	3.87169476	4.40754841	4.06460415	3.84292311	4.93938568	5.33037077	4.37727662	5.2485746	3.36339752
SDK1	1.89753067	0.87045063	1.07849416	-1.5985143	2.63152761	2.57431748	2.47821581	2.81118221	0.27820601	4.86215459	0.27820601	0.27820601	0.27820601
C7orf26	4.98730297	4.20081806	5.18828238	4.05922351	4.47114962	4.2757704	4.47499529	4.70933706	4.3841619	4.52720008	3.64081686	4.51456748	4.65235785
RBAK	4.29972692	4.74367484	4.51638616	5.82769198	5.40881987	6.13027602	5.5888693	5.7202276	5.78243618	4.34328097	4.8388902	4.9103821	4.11480465
CREB5	0.96065597	2.88811423	1.41141722	3.18111218	4.52024521	3.02536866	1.54174903	5.49307089	3.14839606	-0.86799133	3.87429767	5.28257528	5.17039723
EGFR	4.03112544	3.69041639	3.44286985	2.84883925	3.93782588	4.58091228	3.75824721	3.7954016	2.30981318	4.33095589	4.5272054	4.38812847	4.35214894
CDCA5	1.82236526	1.48288486	4.77830763	3.94287617	0.67245054	0.94793775	1.97960585	0.43568561	3.23018299	3.20354749	3.31437732	2.87355661	0.41175781
IGFBP3	10.7862176	6.29525643	10.2038482	9.26985647	7.99649038	7.56186812	8.37376687	7.3504641	7.60741552	6.541723	6.48268412	6.78183505	7.23001976
PURB	4.87136297	5.64988635	5.07381596	6.00483422	6.15753122	5.95960364	6.06868825	6.04431981	5.70855967	4.8405974	5.86023096	6.16836894	4.83187702
IGFBP1	0	0.7591352	0.09152627	0.0997919	0	0.00069205	0	0.00158797	0	0	0	0	0
MDH2	5.9795622	5.50607861	6.19783407	5.74359317	5.49546537	6.15988935	5.30716576	5.22963635	5.54871791	6.02756795	5.90359232	5.88211252	6.6162151
POMZP3	4.10948013	3.55570213	4.73231315	2.94535058	4.170394	3.72394719	3.1468521	4.61747925	4.16831436	4.15039331	4.28996777	3.92714169	4.25153091
CCT6A	5.54271266	6.08499544	5.99504781	6.90875478	6.2737149	6.26431536	6.49385606	5.8772898	6.32577188	6.71149007	6.18600635	5.82368344	6.35967198
PSPH	3.48669102	3.01213137	4.03734871	4.05148458	3.50139939	3.02273372	4.12675017	2.37681928	2.70168219	2.19315927	4.02339167	4.24121769	3.81600959
TRIM50	0.25415996	0.13976397	0.29230232	0.03965764	0.21382149	0.00069205	0.28497086	0.0725262	0	0	0	0	0
ZNF92	3.00355295	3.18873855	3.5599942	4.17633384	4.26663778	4.95221544	4.09078697	4.07324754	5.08235157	2.61044395	4.39264167	4.41273372	2.95699606
ATXN7L1	5.51440564	4.56952799	4.78429414	4.60787274	4.41685292	4.38558196	4.6036086	4.3025061	5.14720084	4.12322439	5.04433303	4.6081391	4.42583149
TMEM168	4.53151216	5.36338583	4.39000357	5.55921531	5.39406493	5.30079495	5.40296072	5.39595873	5.38174639	4.87876636	5.89361689	5.25559812	5.2724852

C7orf43	3.23227704	2.22960777	4.1457033	3.23916973	2.29649809	1.93417012	2.48004439	3.04402788	2.41286765	3.34342746	3.40612813	2.13497272	2.21905674
SLC12A9	4.43485036	4.3250877	4.77661109	3.72583679	3.80600696	2.85065965	4.26998597	3.79431016	3.27089851	3.20339368	4.46753104	4.61727797	3.63650909
GIGYF1	5.82872833	4.95008879	5.76676079	3.90374955	4.94684427	4.00356025	4.90140621	5.50482816	4.77588171	5.44793268	5.33103396	5.22701128	4.62579703
TRIM4	5.4793088	5.61503496	5.38050698	4.98863617	5.62179917	5.64068138	5.78417294	5.5553402	5.51249848	6.12677495	5.71379949	5.40170329	5.30529411
MEPCE	6.13717458	4.42959761	5.75852182	4.47602584	4.9057801	4.7060584	5.0948912	4.81539823	4.79424871	5.14460714	4.803459	5.25293735	5.06648101
TMEM209	4.77034133	4.22543377	4.22111607	4.89817526	4.89998677	5.21399837	4.1436921	4.71331742	4.68816437	3.74184651	5.19907966	4.72239603	3.86641506
AGBL3	2.75102966	2.44601694	2.83706991	2.77677169	3.47252344	3.85313778	3.60581694	2.7095076	3.89960447	2.52289456	3.30819784	3.81508026	2.04626549
ZC3HAV1L	4.08626117	4.62800477	3.50883325	4.69998834	3.91447787	3.12246862	3.99765325	4.07539297	3.78482385	4.32506252	4.67868629	5.02620904	4.21845261
TMEM140	5.90512859	4.29450192	5.05059049	4.361525	4.56067733	4.80368408	4.63428152	3.61313212	4.68717814	3.82855321	3.83384482	3.98415005	4.47487327
TLK2	3.30908794	2.54463057	2.65375054	3.77091419	4.01401654	3.69694358	4.03671032	3.83316492	4.02563531	3.72171872	1.98323527	3.9945693	0.9535087
EPHA1	0.66033295	0.614989	0.60066375	0.50362216	0.26195733	0.45012479	0.46018329	0.57318317	-0.18886633	-0.18886633	-0.18886633	-0.18886633	-0.18886633
NOM1	5.33972279	4.5191316	5.67628067	4.87948483	5.0638708	4.46266147	5.03493289	4.95294676	4.82925785	4.89610541	4.71177785	5.04164084	5.31019165
NCAPG2	3.95054378	3.5290576	5.05944141	5.24270863	3.42338869	3.56960186	4.2037437	4.08066274	4.66961908	2.84092681	4.31734323	3.59229919	4.28662383
NLGN4X	2.27169109	3.88720113	1.45425995	2.06379835	4.19985949	2.06844575	3.31659652	3.29010449	3.21359093	4.57305235	4.09119811	2.12377263	4.50448358
SHROOM2	0.14570665	1.05455828	0.15229648	0.50378756	0.40212397	0.18705308	0.33505441	0.37857166	0	0	0	0	0
LUC7L2	3.37585864	3.97783722	3.27333224	3.99430374	4.14747224	3.67884357	3.72717262	4.45153996	0	0	0	0	0
DENND2A	1.16363077	4.15266352	0.65785486	2.05253421	2.17371837	0.84420739	2.08191147	3.08875015	3.30715884	3.47034409	4.43486464	4.02466848	4.22598136
TMEM27	0.35160111	0.09431222	0.09686113	0.22274916	0.31997043	1.00634862	0.66211085	0.27365405	-0.24397226	-0.24397226	-0.24397226	-0.24397226	-0.24397226
SH3KBP1	5.59834304	5.60496182	5.50696828	6.15034084	5.23311796	4.91345903	4.99387381	4.7202521	5.57344372	5.50644667	5.40839075	4.56696012	5.80259556
TMEM47	6.06318393	7.62551276	6.28292808	6.57809896	9.05489485	8.75664848	8.41448354	8.51882058	8.39635459	7.44515338	8.54940003	8.72553535	7.64759453
LANCL3	1.85618159	1.99393801	1.96109211	2.17120097	2.93868638	3.51297701	2.60823662	3.03935255	2.24209852	-1.18491824	2.91565833	3.14517341	2.69259421
SYTL5	0	0.09416945	0.00124472	0	0.02176516	0.00069205	0	0.00158797	0	0	0	0	0
CASK	5.86693561	6.33336487	5.32619912	5.7333208	6.28988165	6.53746846	5.75703941	6.40936779	6.81752679	6.45460513	6.63827926	6.38855143	6.33719121
KDM6A	5.41456465	5.53266936	4.57019152	5.87267363	5.5919121	5.81946766	5.4766	5.75472713	5.75770979	5.36470221	5.81543804	5.82268166	5.88267406
MSN	9.26652924	8.82775705	9.39990553	9.31977506	9.41757275	8.73469996	8.83961307	8.44111104	8.4863212	8.44086458	8.59743185	8.44086458	8.67916967
CCNB3	0.29676851	0.2110662	0.44302322	0.49141604	0.57289791	0.67135269	0.2483356	0.66250311	-0.21581857	-0.21581857	-0.21581857	-0.21581857	-0.21581857
HDAC8	5.39620681	5.02523592	4.61614941	5.39274001	5.14308781	5.20784631	5.380507	5.22749898	4.89808471	4.89511019	5.30888784	5.27591687	5.0027343
SLC16A2	2.679457	2.24534051	2.00325231	2.48345005	1.34360115	0.90738065	2.12400198	1.5327058	0.95202431	0.04602162	1.09703432	3.66305293	0.04602162
CXorf36	-0.11052597	3.72503481	2.21242085	4.46166366	4.33414106	2.48059474	3.52644142	3.33529306	3.81326041	3.51890365	5.98791244	4.1187764	6.37326802
ZNF157	-0.14569409	-0.02059426	0.30799802	-0.23896785	-0.31205336	0.41187626	0.11556663	0.73381634	-0.06914494	-0.06914494	-0.06914494	-0.06914494	-0.06914494
ZNF182	4.12910255	4.72632136	4.2318352	4.21177325	4.90515003	5.48427961	4.37473215	5.15582739	4.67186341	4.3565086	5.36842835	4.54194296	3.9821886
CHST7	1.65959623	2.66685946	2.59682362	1.75199535	2.01353124	3.07198548	2.97505979	1.83587817	2.91577163	3.13804157	0.81123173	3.6501184	2.78102897
NDUFB11	5.49502544	5.20980439	5.82828546	5.32157144	4.91035193	5.75427313	4.54064618	4.84473556	4.58207896	5.74163214	4.72283849	5.24355516	5.57879363
ZNF41	4.80844335	5.33325529	4.57511884	5.31906007	5.26444574	6.01654594	4.92091248	5.05549211	5.19001137	4.8099958	4.83772475	4.73841915	5.4436719
ZMYM3	5.86835393	4.91957095	5.41698085	4.49816471	5.06720223	5.19788636	5.62046046	5.38371424	5.29625297	5.99461952	6.06700391	5.17891934	4.94270433
TAF1	6.32112701	6.16755698	6.13420202	5.73492524	6.04032395	6.51899564	6.09938291	6.04127193	6.1146242	6.5244907	6.12196964	6.22287445	6.56570439
GPR174	1.2357839	3.08551096	0.25241935	1.16378142	1.25474259	0.14226759	1.19351519	-0.06838093	2.56830234	-0.33986077	0.72149238	-0.33986077	2.88216383
NONO	7.35889429	6.64966148	7.46834754	7.235184	6.85053668	6.68987689	6.8402975	6.98453895	7.02636102	7.2331017	6.56291872	6.80430999	7.15510164
CCDC120	3.95547526	2.82982016	3.6394072	2.1285673	2.92363266	2.30637996	3.08798503	3.04125836	1.61489128	2.74059784	2.81716798	4.02327798	3.27952685
LPAR4	2.42468437	2.35857582	3.41556984	3.22319592	2.84587183	3.20403962	3.46227773	4.35104243	4.45936988	3.35094117	3.46686914	2.77309787	3.9754974
EBP	4.31998171	3.41547008	4.92814529	4.46555863	3.40417254	3.80813005	3.72107072	4.39473608	3.60168221	3.89313435	3.94235924	3.63241374	4.42117612
OGT	7.04081704	7.5041752	6.50397226	7.51128159	7.65405549	7.31971115	7.82485133	8.16195561	7.26551035	6.96337201	7.83005128	7.65331787	7.51342478
SNX12	2.31732489	2.95949959	2.76478861	1.88177866	2.19425686	1.89476325	2.49488751	1.88707365	3.64391261	3.36265677	3.23402786	3.86498883	2.31218875
ITGB1BP2	4.95961338	3.91312099	4.98235872	4.26305835	4.81206341	4.30365752	4.83870491	4.85593931	4.16909818	4.75272134	3.94947689	4.33297636	5.52739842
IL2RG	4.40323868	3.53622146	2.32011448	3.6831798	2.42877917	1.87320691	2.01631706	1.81049375	3.57549926	3.10531605	2.87407983	1.88499679	3.1964105
ZNF711	3.7279077	4.11644292	3.8978567	4.1509484	4.59964625	5.38530179	4.90929763	5.37214386	4.82576759	4.45146497	3.57830847	4.5661591	4.04187617
DIAPH2	4.31493181	5.05667172	4.7096093	5.11131705	5.43507341	5.80258506	5.40841932	5.95703537	5.56702269	5.58629928	4.73083152	4.48376122	5.20454238

NXF3	0.21190845	0.43691451	0.22981432	0.48523713	0.71046742	0.42130824	0.14744939	0.21084845	-0.18669862	-0.18669862	-0.18669862	1.55833181	-0.18669862
RIPPLY1	-0.01220243	0.21687081	-0.10502026	-0.10750899	0.49323854	-0.10612529	0.61696609	0.41951112	-0.04260023	-0.04260023	-0.04260023	-0.04260023	-0.04260023
PRPS1	5.26551363	5.78469494	5.54725441	5.5633634	4.96219218	5.73129153	5.2300737	4.7900751	4.69078514	5.3319835	5.22995908	5.49406338	4.94767009
CXorf57	2.11698821	1.87978161	2.15674066	2.62918812	2.46601127	3.61197922	3.17707665	2.9723145	2.59163553	3.08210279	2.16904004	2.95520134	2.60485104
FRMPD3	1.75020155	0.39073024	2.08853361	1.19729519	0.91870216	0.49837257	1.65503065	0.6753698	-0.58061581	3.53838088	-0.58061581	2.06692068	-0.58061581
DOCK11	5.63456594	6.39053804	5.40590218	6.47135042	6.45966611	6.86291057	6.81745983	6.84278908	6.62131942	6.8506457	6.31733574	6.65779373	6.4291271
IGSF1	2.8909672	1.53038503	2.58479422	3.78792222	2.10091117	3.5006073	2.36316392	2.6431424	2.55776737	2.83793024	4.09841011	-0.32200427	3.74426668
GPC3	4.1560836	5.69625349	4.77960134	2.86432233	3.4220416	2.26359314	2.20146594	3.24499701	5.15502035	3.3829039	5.47190774	5.0901269	4.45189766
RBMX	6.49035221	6.86405479	6.33204088	7.31290538	7.09969577	7.27379277	7.01498509	6.83728222	6.74675265	6.81779328	6.83414569	6.56068873	6.80535261
MCPH1	4.50202855	5.00031358	4.74512192	4.8408678	5.19708449	5.68459699	4.92550735	5.15039326	5.5343791	4.34645562	4.57832491	4.98540535	5.11383336
MFHAS1	6.35007144	5.5254247	6.12432651	5.1585017	5.64071678	5.30651306	5.87788037	4.93691251	5.32466732	5.38127221	5.59017581	4.85378452	5.46799486
FBXO25	4.7436054	5.02206203	4.48180458	4.5376928	5.39856475	5.32611577	4.92586434	5.43096091	4.8877107	4.78442014	5.85280448	4.80650183	5.42824377
NSDHL	4.19581853	2.99828596	4.20836632	3.65056371	3.24383542	4.7629092	3.41802832	4.07257909	3.76891186	3.66362402	3.26734477	2.60115312	4.12841741
ZNF185	5.424239	4.21317174	5.26772022	4.34398388	5.3993255	6.27263855	4.95866646	5.32331223	4.98235203	5.41292191	5.11498849	5.82311816	5.99794202
CETN2	4.75495338	5.42483793	5.54457737	5.94919452	5.2054841	6.25557688	5.12568957	4.94042655	6.095016	6.11102332	5.24932961	5.40783851	5.63140759
RPL10	7.92313995	8.06901454	7.32195631	8.11743574	7.39200141	6.95834638	7.37745732	7.1385661	7.45497066	8.31185974	7.72026806	7.93743784	7.34834252
CSGALNACT	5.49172495	5.47525153	3.75117197	5.87148115	5.0128841	5.5811847	5.57259869	5.10112019	5.07304429	4.91680003	5.30138626	5.86984859	6.10081942
ATP6V1B2	6.21190863	5.99927735	5.94480875	6.98778473	5.78687962	5.71956053	6.26857826	5.9926945	6.065546	5.8752766	6.12069923	6.13588371	6.31282174
CCDC25	5.435085	5.82516928	5.17548512	5.37913866	5.64780751	6.12317676	5.74006841	5.88819844	6.0569105	5.47988056	5.71526247	5.91749503	5.75902116
HMBOX1	5.60344528	5.41928794	4.66655744	5.52089474	5.49978871	5.14449324	5.63178167	5.33666738	4.88642811	4.99927349	4.55038201	4.84353034	5.64436738
CHRNA6	0.08796223	0.60792036	-0.00089265	0.12171636	-0.00342958	-0.00201908	-0.00342958	-0.00019304	-0.0867594	-0.0867594	-0.0867594	-0.0867594	-0.0867594
GNRH1	3.72869906	3.8791486	3.10957383	3.14956563	4.46145757	4.31365842	4.84208062	4.81002473	4.16471538	3.7257393	5.11665956	4.47499565	4.00651573
BIN3	4.97862026	4.65920528	5.15604442	4.09943988	4.835222	4.03972473	4.99501632	4.91078269	3.95065161	4.19118695	4.09702433	4.70407893	4.7479183
DOK2	3.09668563	2.91128651	5.05225674	3.59032955	2.01801108	1.50318668	1.02242751	1.30908571	3.37863063	3.30183408	3.03877388	2.45236108	3.23182305
SLC25A37	6.43850403	5.67238618	6.37511051	6.10876729	5.4226745	5.09870462	5.70516273	5.6584834	5.30583167	5.16441452	5.67582451	5.62646576	5.19581218
CHMP7	5.62871185	5.37128672	5.37279656	5.36854049	5.73206554	5.63613422	5.80551064	5.67310644	5.42759163	5.71032598	5.40425686	5.62778183	5.76934298
DOCK5	6.07923194	6.22055245	6.11491591	5.77585662	6.65383424	6.75870494	6.78796581	6.84319647	6.56311288	6.39414918	7.14626701	6.36193468	6.4965176
STAR	0.14640224	0.43432454	0.14899746	0.20614115	0.14640224	0.26223445	0.14640224	0.14971313	-0.08172768	-0.08172768	-0.08172768	-0.08172768	-0.08172768
ERLIN2	5.10646548	5.08682245	4.96719108	4.65270445	4.68747964	5.19147392	4.68969512	4.29549236	4.95468454	4.72550745	4.59021357	4.33114784	4.96666973
PXDNL	2.2049244	2.49971953	1.28132911	1.06804461	1.06966395	1.97200713	1.43156936	1.97071335	0.72113002	3.02164392	3.0666758	3.44692534	6.4833109
ST18	0.36877494	-0.03726304	0.39077969	0.39250508	0.05578618	-0.10858809	0.05079377	0.31314845	-0.08384885	-0.08384885	-0.08384885	-0.08384885	-0.08384885
RGS20	0.19306297	0	0.00124472	0.13904789	0.12408308	0.20003251	0	0.00158797	0	0	0	0	0
TACC1	7.35322545	7.88372686	7.3617041	7.55762356	8.08454602	8.19889398	8.08454602	8.03394294	7.61281929	7.93429872	7.87347545	7.92357049	8.15341418
GOLGA7	5.99396643	5.83161157	5.16047311	5.63245627	6.08572013	6.60791661	5.67741506	5.88520833	6.26755626	5.6933832	6.31459629	6.07009845	5.40794891
GINS4	1.47096491	1.38803477	3.73879957	3.1540422	0.91682256	1.24976782	0.87837662	1.29125411	2.35966455	-0.86938513	-0.86938513	-0.86938513	2.68301571
DNAJC5B	1.20158058	0.18567282	0.18817703	0.20543296	0.22705934	0.18706512	0.18567282	0.18886761	-0.12476506	-0.12476506	-0.12476506	-0.12476506	3.20490449
TRIM55	0.33027946	0.47948209	0.26975063	0.36283154	0.26714766	0.4911461	0.26714766	0.27046844	-0.1928463	2.22056392	-0.1928463	-0.1928463	5.51211958
ADHFE1	4.7935299	4.63787114	4.24075554	2.77996592	5.16733805	4.93050851	5.32698356	5.96494525	4.92861556	5.56400744	5.32525157	5.36205471	6.23441419
MRPS28	3.15916683	3.83215388	4.11078086	4.62880637	3.99817523	4.53053286	4.68873808	4.01535287	4.24764606	3.7275261	2.90316535	4.2362643	4.51820965
PMP2	1.28169573	1.28169573	1.28429927	1.472244	1.48052974	1.28314326	1.55620516	1.7150018	3.74190942	3.44674661	3.40719076	2.90802961	2.64850921
LACTB2	3.09801037	3.22096026	2.83933627	4.07765357	3.59949801	4.49204141	3.46565768	3.25219975	3.46169074	2.88121269	3.59663732	3.75477717	2.98549765
TERF1	5.32030884	4.85958977	5.07497348	5.34325164	5.80211466	5.91738387	5.73057732	5.79955244	5.9476765	5.07366537	5.22163185	4.77630543	5.19518011
RPL7	6.57128842	7.40157363	6.54860705	7.55269348	6.99818937	7.15036652	6.93954275	6.93607062	7.14984921	7.66917859	6.51627487	7.1387531	6.83326184
SLC26A7	2.11662405	2.30841144	1.85967981	2.31382452	1.9393093	2.82957965	2.13605366	1.9835929	3.65162979	2.25659845	3.79742717	2.10676418	2.24877663
ATP6V0D2	1.84453883	-0.04660891	-0.09412845	-0.00151089	-0.09608661	-0.0949979	-0.09608661	-0.09358845	-0.0545044	-0.0545044	-0.0545044	-0.0545044	-0.0545044
SYBU	3.24976659	4.34926348	3.39989447	2.07053274	3.39124137	3.88855093	3.29978985	2.90614405	3.38086986	3.18644048	3.46061762	2.74891874	4.84357778
DPYS	0.81623158	2.41716058	0.02870329	0.29003874	-0.18147316	0.39696858	-0.12960821	-0.06597057	-0.10874869	-0.10874869	2.83935794	-0.10874869	3.57632656

MTDH	6.11060024	6.64576844	6.61993286	7.01459948	6.85779444	7.09487631	6.81090663	6.82627567	7.20116464	6.83604543	6.66635245	6.82912924	7.00135655
LRP12	4.88587695	5.05169432	4.74348977	5.97272617	5.41570727	5.54938122	5.55133597	5.63434666	5.10764472	4.72604376	5.40463788	5.57349335	4.78465941
EBAG9	4.38058682	5.01451185	4.32372037	4.82820029	4.94242691	5.65975862	4.57719932	4.56861188	5.2013668	4.06663136	4.26113252	4.85343068	4.92756818
RSPO2	2.08128849	1.67818944	4.33476361	1.33571381	0.82199524	0.7388948	1.93282999	3.06829925	4.29708586	4.35531986	0.75512689	4.74112618	1.96377087
POLR2K	5.03699551	6.00354778	5.52984491	6.71270598	7.14145384	7.18577167	6.53201921	6.46424156	6.01655265	5.21864491	5.73133609	5.48817897	5.13146632
MAL2	0.60289124	4.00355333	0.60507329	1.2262978	0.60289124	1.10265214	0.64238989	0.7059555	3.21045655	3.27093342	-0.46582838	2.70166274	3.46906395
EIF3H	6.85897413	6.9529114	6.12098995	7.14882531	6.9116057	7.02736011	6.69294857	6.80422317	7.19751889	7.09778513	6.96222913	7.0199025	7.21960033
UTP23	4.87087929	4.60193242	4.60981712	4.95229229	5.59725778	5.80349518	5.50500472	5.31459755	5.39745629	4.2440426	5.19370429	4.91263896	5.04659656
NDUFB9	4.97194273	4.62731161	5.20591421	4.87544239	4.29420754	5.61983078	3.99439496	4.09082219	4.47483632	5.65969277	4.50250746	4.18879995	5.58642456
TATDN1	3.79883258	4.72059304	4.4342776	5.02393865	5.26201642	4.83316968	4.95524288	4.91823891	4.45667901	4.59673367	4.16719909	4.53470052	4.63602075
FAM83A	0	0.42698973	0.065593	0.00849828	0	0.00069205	0	0.00158797	0	0	0	0	0
GSDMC	-0.05956636	0.41811146	0.21991196	0.23624559	-0.02459432	-0.05821953	-0.01406743	-0.05647592	-0.00375882	-0.00375882	-0.00375882	-0.00375882	-0.00375882
FAM135B	0.27027665	0.36596153	0.27287964	0.30489451	0.28569279	0.27172387	0.41603827	0.47309923	-0.16357483	-0.16357483	-0.16357483	2.86465951	-0.16357483
ZNF7	4.9872954	5.08507165	5.07921996	4.68291077	5.00714591	4.55601116	4.39901235	4.73642196	4.74626626	5.42172131	5.00027915	4.89806282	4.78960356
ARHGAP39	2.13130663	1.54036912	2.94910316	1.4897538	1.36570605	1.25421794	1.56651947	1.43515999	-0.72006409	-0.72006409	1.94132477	-0.72006409	1.46153276
SLC39A4	3.37788129	1.87637809	3.46563583	1.63503414	2.62069525	2.35473084	3.13467843	3.28327318	3.2997524	3.30163849	0.16181078	1.56832213	-1.19603336
VLDLR	4.88443418	5.69077355	5.42130276	4.74837323	6.02923582	5.44941234	5.4460691	4.94917068	5.2269585	5.8773825	4.8019089	5.83755557	6.21346291
AK3	5.31240467	6.04571957	5.44975796	6.13031195	6.47316241	6.77555121	6.26066206	6.54993142	6.34807413	5.21785488	6.29128497	6.08623776	5.27855189
UHRF2	5.31634547	5.73445642	5.71753361	6.57988579	6.01357147	5.80848912	6.22284737	6.3484961	5.64538332	5.76108845	5.84185875	5.84985845	5.1662039
NFIB	7.79016633	7.68164323	6.88421377	7.50730274	7.65727394	7.80763473	8.16961348	8.19850192	7.46035624	7.50957427	7.51764402	7.89180832	7.7058703
CER1	0.06151448	0.73308715	-0.02208668	-0.02451515	0.6730616	1.2889889	0.45360724	-0.021417	0.63877122	-0.11313211	2.53403873	4.06442106	-0.11313211
PLIN2	7.6991628	5.50171109	6.33207572	7.02501098	4.76627007	5.02816615	4.53486805	4.888426	4.44095389	4.78284606	4.27343713	4.58379013	5.22871076
HAUS6	4.38391387	5.05078343	4.25445424	5.55825922	5.33772613	5.61157165	5.03708784	5.20464934	5.10352798	4.40473558	4.86955204	5.00091155	3.85024567
CDKN2B	3.66224494	3.7756153	4.28975878	3.84313433	4.29839901	4.17560765	2.95792872	3.56341655	4.18689222	3.16167216	4.3287949	4.46003552	3.88968708
CDKN2A	2.63825148	2.00828287	2.5379542	2.40612994	0.98401477	1.82777461	0.62442922	0.99960656	-0.75799204	1.7077972	2.59393745	2.39107503	0.29137891
C9orf72	3.98439072	4.78304251	3.71584427	5.94903435	4.85439517	4.95572405	4.84189565	5.33638595	4.6698845	3.02881292	5.1861168	5.34151715	4.19590292
ZCCHC7	5.34928014	5.36007636	5.17901989	6.049759	6.00650846	6.14982318	5.89178105	5.92411458	6.08389479	6.29313286	5.68337409	5.71899087	5.95557698
FBXO10	4.35947355	3.43284559	4.35947355	3.87017039	3.90468663	4.04363525	3.91763217	3.57197049	3.82867063	4.16919161	3.7660327	3.80423217	4.66465654
SIGMAR1	5.98368535	5.46664162	5.79197031	5.53432796	5.20637634	5.38055382	4.94728552	4.78061215	5.58552414	5.8623868	5.29023825	5.21635764	5.22327538
CBWD5	3.16417528	3.66124278	2.92042162	4.22685792	4.73850723	3.65458532	4.2382826	3.90827143	4.15071052	4.09876387	4.36034091	3.1878873	4.29513751
CEP78	3.56729512	4.1150595	3.97008831	4.29348143	4.44626776	4.57826595	4.50855741	4.71786805	4.4279911	3.70567354	4.68550288	5.03098696	4.29066395
NTRK2	3.47195737	6.21587308	3.47763948	4.83429767	6.55774353	5.31746423	4.61462008	4.57331176	5.55970453	4.7782717	6.43892863	5.35117696	6.50471054
SHC3	2.6852526	3.32216817	2.62991634	3.19081921	3.09698179	2.44144981	3.62086332	2.87821612	-0.11505796	3.22819643	2.94516106	3.43091189	3.5711418
AUH	4.59998673	4.60535821	4.27781894	4.72319288	5.22793258	4.99499009	5.14825986	5.39134706	4.79036125	4.36456951	4.7253094	5.20873241	5.61160856
C9orf3	5.08925508	4.81218822	4.99414154	4.3948532	5.39890542	4.77173437	5.31386536	5.7573071	4.61534937	4.11055406	5.35668259	5.28714368	4.84970532
ZNF462	6.70646006	5.96421019	6.1885921	5.72595413	5.39409913	5.68736395	5.56360988	5.4497558	5.89163805	5.64662582	6.11680096	5.07429727	6.42169827
UGCG	4.44334659	4.84208448	3.57203253	6.21003644	4.88404482	4.85040081	5.051415	4.65823718	4.83497871	4.46558875	4.26778114	3.89489307	4.61557643
ACTL7B	0.04683773	0.19954844	0.11799595	0.02678381	0.22189494	0.12777193	0.13277981	0.00158797	0	0	0	0	0
SNX30	5.48368049	5.93577612	5.98632235	6.13865551	5.47781328	4.87235192	5.42950953	5.62619841	6.2276662	5.51201741	6.66948227	5.79203044	6.22649793
STOM	7.66812287	7.34882021	6.6924796	6.94813521	7.80873804	7.56765808	7.86506595	7.35013442	6.7311692	7.2766399	7.12893126	7.30546428	7.18113858
GSN	5.64571851	6.20082917	6.17460853	4.60796735	5.68631728	5.63924785	6.26258385	6.23028748	5.66499335	5.95569729	6.11914767	5.74427502	5.79835426
MRRF	4.54719535	4.39756744	3.95349617	4.51717337	4.39178599	4.16579667	4.13426978	4.33536942	4.33142827	4.34518634	4.02530239	3.8868763	4.49537075
NR6A1	2.63735442	1.846185	2.54055994	2.27260341	1.78351468	1.8870271	2.23660706	1.91317886	0.78455902	1.3194375	-1.10854406	1.76413009	3.80613139
CRB2	-0.04093929	2.53112113	-0.06195342	0.50835786	0.0172834	-0.0625631	-0.03797569	-0.06157477	0.06271361	0.06271361	2.52425538	0.06271361	0.06271361
ALAD	6.01191065	6.01243982	5.93472102	5.08475786	5.13356188	5.30283505	5.59492529	5.00449686	5.93694802	6.1340942	5.45408123	5.36324862	5.87658788
ASTN2	3.47009664	2.58507526	3.29829739	2.27091463	3.99080301	2.80553662	4.26381239	4.66787492	3.80385507	4.07231488	3.7072502	3.52614045	3.45912036
WDR31	4.29230865	2.94654628	4.1913572	3.59035419	3.12267228	3.34377526	3.31722427	3.26468834	3.39245818	4.18615084	2.68556015	2.96559688	4.14830899

POLE3	5.08423975	5.97764563	6.04118696	6.67494001	5.79980088	5.559412	5.92159128	5.52178496	4.81174144	5.28470688	5.41542634	5.20585681	5.91569378
SURF4	6.79084624	6.79084624	6.73412682	7.54308391	6.54055093	6.21666155	6.41704983	6.37858028	6.40640972	6.45945212	6.68043431	6.47181317	6.63855331
GBGT1	4.31127759	3.92774817	4.09168399	3.99383715	3.09240539	2.30890396	3.45319569	3.35298001	3.12828155	3.91402716	4.19852313	2.52783908	2.94612335
SURF1	5.11925182	5.09850192	5.05766947	4.31935245	4.28284754	4.29056495	5.0611418	4.32451365	4.10387575	4.44619856	4.35158359	4.50507729	4.43496732
SURF2	4.13862204	3.35485129	4.47692847	2.30051609	3.29385328	3.38156788	2.54958539	3.73828092	3.65876782	4.45514959	3.88695813	2.77895852	2.18993734
SURF6	5.31054509	4.48574357	5.59891808	3.96027145	4.51719759	5.01513279	4.61186256	4.43994104	4.849006	5.70675599	4.28130951	4.17603893	5.21344345
MED22	5.34402074	5.55707208	5.56717912	4.52956922	5.15576627	4.50451144	4.72588445	4.77309355	4.99445451	5.37052683	5.57075906	5.62331875	4.60869381
REXO4	4.97334361	4.43998488	5.28146159	4.04317759	4.11389942	4.2634291	4.25319707	3.73885387	4.50398561	4.9362946	3.81193828	4.43240979	4.62188155
RPL7A	6.00685218	6.69156969	5.67678159	5.95662031	5.34218084	6.17110595	5.42152769	5.74470937	6.26511148	6.95570361	6.1073318	6.66876119	5.65732955
GTF3C5	4.51898979	3.75398231	4.29074205	3.33175014	3.54201008	3.66304471	3.78165689	3.51408314	4.70565622	4.85655802	3.18959151	4.39392906	4.39601681
ASB6	5.24386855	4.52032421	5.38077827	4.03536127	4.32267537	4.15353274	4.44241939	4.40164343	4.54517891	4.29719502	4.46703088	4.92782993	4.3648436
PTGES2	4.83920835	4.43239302	5.1007448	4.02249767	4.04852441	3.78241081	3.69514248	3.78138242	3.92237762	4.66425363	4.48521922	3.86069527	4.35719734
CIZ1	6.64296067	6.16483972	6.6644877	5.64924608	5.96797	5.78066621	5.84682934	5.59047298	6.02699768	6.82673045	6.00661804	5.70258448	5.9357461
SLC25A25	4.54291878	4.50225994	5.42061486	4.11681084	3.71331991	3.73816252	4.03441077	4.48767483	3.86689358	5.0216684	3.40862871	4.18785795	4.39678528
SH3GLB2	5.90981032	5.44520948	6.26150089	4.28361958	5.66356636	5.10281608	5.60798675	5.53904266	5.50419408	6.30554062	5.45766598	6.25413386	5.16790493
PTGES	2.62790943	1.98833658	1.48987992	3.41739148	1.09264783	1.24108135	1.84340215	1.14128096	3.01562252	-0.79901211	2.83379346	1.02098001	-0.79901211
LCN2	0.42748997	-0.11257912	-0.11025577	0.75423297	-0.11257912	-0.11128737	-0.11257912	-0.10961506	-0.12429643	-0.12429643	-0.12429643	-0.12429643	-0.12429643
LRSAM1	4.85911602	4.37404484	5.25795298	4.09972787	4.83208873	4.04549337	5.04077705	4.53578798	5.10203005	4.84651707	4.90918047	5.31429515	4.36144996
GPR107	6.75216659	6.64561822	6.79236432	6.81730487	6.81806421	6.33188034	6.80079448	6.66670892	6.74098504	6.85603773	7.0699775	6.71329454	6.77344203
INPP5E	4.21146802	3.99838628	4.52039867	2.73874448	3.65209793	2.82410569	3.86920526	3.80012893	2.86504828	3.63914607	4.20530377	4.63086262	4.16986108
SEC16A	7.03638951	6.73448031	6.87018259	6.66844233	6.58016997	5.92907107	6.40501982	6.37791937	6.04558477	6.22177241	6.85781654	6.71373635	6.46597501
NOTCH1	5.53592574	5.11420243	5.51211163	5.20479066	5.41100136	4.65792234	5.37148631	3.76542886	5.14710011	5.84039462	6.00457303	5.11551935	5.09468496
CACNA1B	0.02585642	-0.02291852	0.25347169	0.02706405	-0.00927959	0.04598096	0.13090513	0.11044652	-0.04296717	-0.04296717	-0.04296717	-0.04296717	-0.04296717
NACC2	5.88211964	6.05554632	6.10966894	4.60495754	5.89456089	5.81043765	6.14263003	5.62437229	5.19757592	6.26287701	5.90982469	6.53795369	5.53120842
USP6NL	5.0900207	5.36374527	4.23837695	4.86744245	5.16199355	5.09882619	4.91674578	5.15934927	4.85656958	3.63914852	5.11191959	5.07926899	5.20836551
COMMD3	2.50946701	2.90587727	2.92178086	3.43142778	3.08437731	3.88363126	3.74837023	3.49817813	3.86280175	1.52785326	3.32282823	3.35389848	3.4609796
MSRB2	5.23265133	5.27773698	4.84430475	4.45585232	4.96528191	5.63755	4.99094998	5.14142141	4.82377737	5.5513721	4.78744002	5.62947505	5.08901814
PDSS1	1.78765551	1.71966608	2.05656994	3.4489852	2.27505336	2.43437901	1.57632653	1.94812621	2.35523377	4.48240353	2.785019	3.25048887	1.90177494
FAM171A1	4.74447898	4.64251396	4.36124942	4.92670286	4.24714205	3.69593547	4.44128551	4.33900409	4.04883238	4.8978648	4.91096738	4.52940935	4.22343973
SLC39A12	-0.0293404	0.50236014	-0.02690536	-0.0293404	0.39838917	-0.02798655	0.78183387	-0.02623386	-0.0981043	-0.0981043	-0.0981043	3.09426454	-0.0981043
TMEM236	2.16985116	3.03645765	2.78823906	2.06887538	2.72356008	2.79496292	2.7771781	3.08468457	3.76843817	2.79744042	2.37901465	3.67113886	2.57384185
RSU1	7.03604667	7.07066039	6.52480792	7.08100633	7.18855003	7.68056642	6.9975132	6.83878049	7.41105744	7.40131668	7.39378196	7.07476893	7.1588724
ST8SIA6	1.09127442	1.04030427	1.34008211	1.00235823	1.19574799	1.17008693	1.7873698	1.63398436	3.38318474	-0.56236809	4.67290015	3.14412697	3.90194223
PARD3	5.9951827	5.84803921	5.52370359	5.03878973	5.63739322	5.94141477	5.7781388	5.97160794	5.67972683	5.8002625	5.72476766	5.38094003	6.18708832
ZEB1	4.90003018	5.00263986	4.63600701	5.2077867	6.055863	6.32693192	5.91195408	6.0411273	6.1733614	6.48659433	4.99059104	5.73450179	5.66983834
FAM13C	4.64207359	4.61303375	3.99389616	3.88489431	3.85596605	5.41661239	4.76302024	4.61035434	4.91831163	4.59107945	5.22015882	5.18481737	5.08185084
NRBF2	4.51278297	4.49142573	3.92104478	4.79339448	3.99149742	4.53487881	3.28212919	3.59537157	4.14283554	3.98651193	4.59027591	4.23207683	3.93826149
CDHR1	0.77475947	0.92561242	0.72641863	0.65338444	0.85565145	0.72286641	0.95086666	0.81728736	2.77689332	-0.41188074	-0.41188074	1.67732449	2.46484714
POLR3A	5.44353856	5.14684411	5.4245519	5.84719847	5.14960975	4.79571802	5.436016	5.18296031	4.70023564	4.73544422	5.00095275	5.05889021	5.37210043
HERC4	4.99989424	5.52538541	5.15312594	6.061315	5.95071349	5.53759537	6.26038467	6.04663417	5.77525757	4.83387585	5.6528842	5.21769046	5.52271697
CAMK2G	6.41695424	6.88045325	6.57004717	5.31078438	7.08055854	6.91892605	7.56489159	7.21789067	7.07651824	7.83001248	7.12740972	7.33682346	7.18953109
GLUD1	6.38401571	6.77352001	6.24740872	6.62076055	6.65619882	7.04021834	6.78063701	6.8814667	6.57942798	6.43816486	6.77072647	6.58060444	6.57758619
ANKRD1	2.37880087	2.94281386	2.01388389	2.21950377	6.76683414	4.18431245	2.36044246	3.14623173	3.02061969	1.83155976	2.60640169	3.20161888	11.1025564
HTR7	3.73136474	2.9278626	3.62839789	3.61724092	1.96457983	2.70491808	1.83702374	2.28408266	3.54897386	3.16023225	3.43923568	2.62027872	3.1507833
RPP30	4.9951164	4.91441996	4.73729466	5.21709574	5.05721472	5.48640168	5.46979644	5.0388277	5.19750731	4.68317651	4.80153935	4.75811715	4.48405441
FRA10AC1	3.98122157	4.85327903	3.90427466	4.89707985	5.09168797	5.1243427	4.95972241	5.4407936	5.38239896	3.85996912	5.10604467	4.63155395	3.84907609
ADD3	6.19182582	6.87100401	6.0409715	7.1685604	6.76985754	7.07547352	7.29634009	7.69793105	7.55594039	7.01642613	7.1723486	7.35655952	6.7301409

HABP2	0.33299294	0.1594952	0.28522717	0.19970783	0.40212028	0.46109376	0.71460816	0.47544106	-0.17498875	-0.17498875	-0.17498875	2.57981605	-0.17498875
DNAJB12	5.68944707	5.28886614	5.68477895	4.66492154	4.99058151	4.90516173	5.07068076	5.27236735	5.2007823	5.57255637	4.13945792	5.40896357	5.62439946
EIF4EBP2	6.84864148	7.38675659	6.86739956	7.4879476	7.62849747	7.29418112	7.4547699	7.48430577	7.3273377	6.97627404	7.63486375	6.92334083	7.51636582
NPFFR1	-0.05032559	0.16281069	-0.04777632	-0.04777632	0.0242511	-0.04890823	-0.00610988	0.15994147	-0.043786	-0.043786	-0.043786	-0.043786	-0.043786
TCF7L2	5.82287767	4.8603052	5.26679803	4.45553541	5.63734739	5.39810019	5.38813778	5.47984492	5.39517508	5.15749614	5.0075149	4.45846702	5.6835014
MKI67	3.86158937	3.39391958	5.90321261	6.03377438	2.83549752	2.97529058	3.88227192	2.91835906	4.27841127	2.92916014	4.09365835	2.61233031	4.49942697
INA	0.51357887	0.51357887	0.89496528	0.58290843	0.51357887	0.51502364	0.72068519	0.69262937	2.44051406	3.65516215	-0.37134257	2.82243377	1.6428612
LRRC27	3.63813397	2.97453999	3.89247792	3.27530292	2.72856486	2.67148333	2.79841789	2.89462135	2.38308775	3.62094798	3.49623218	2.98782929	3.07327516
MTG1	4.17716683	3.88532451	4.33552997	3.94387645	3.80002482	2.86687208	3.99459458	4.1670256	2.99029644	4.00075894	4.43844437	3.96259026	4.20958127
PAOX	3.1249136	2.05667756	3.51799264	2.11736443	3.06988801	2.04162121	2.07577669	3.07899427	2.91849067	3.99218965	2.96557244	3.72967402	1.88864954
GSTO1	5.4724791	6.31802397	6.172952	6.75292662	5.36790119	5.2476261	5.49792544	5.45724816	5.50557792	5.93083771	5.10100391	5.79361818	5.82762013
TAF5	3.58381771	2.19751138	2.94878709	4.32605244	3.89745777	4.17202905	4.0056114	3.90051124	3.11177617	-1.63424589	4.14252715	3.26956032	2.62758753
PPRC1	6.19425036	5.49603497	6.68426098	6.30843341	5.59472709	4.6579503	5.43144762	5.05276701	5.19439964	5.23359479	5.65661996	5.29494936	5.478324
ITPRIP	6.00518737	5.29348912	6.70820871	5.8221658	5.13209622	4.89805833	5.06364216	5.67713676	4.45179983	5.16681565	4.2614245	5.53855863	5.81567981
CNNM2	4.84778872	4.65280353	4.53333117	3.63089804	5.86191027	5.20411542	5.41468258	5.20411542	4.8325762	5.02444538	4.89564482	5.24060493	5.4804039
PDCD11	5.73133107	5.54046155	6.06030968	5.89120485	5.04687778	5.10289853	4.90541636	4.73085525	4.68688356	5.56845768	5.20492597	5.30121676	5.56362838
ADAM12	4.20036808	2.66517119	4.67133629	6.61587689	2.81618773	2.73539331	2.56762315	2.54419034	1.96426443	1.57850945	3.58000494	2.30194931	2.76463807
RGS10	4.59264322	3.15912655	4.55686332	5.19088457	3.39985785	3.90494348	3.69217935	4.01192169	5.04351281	4.89837158	2.63922108	3.46233798	4.06554018
BTBD10	5.48759691	6.12231975	5.63129806	6.18410778	6.02939675	6.45633886	6.18451715	6.19754069	6.23284507	6.57433216	6.20145352	6.29552686	6.1672709
ADM	5.76614016	2.66106882	6.07501817	6.24538302	3.83933119	3.55125247	2.48476918	3.17435299	2.75609376	3.40970604	1.58326272	2.05204847	2.08493613
GAS2	0.29256168	-0.16949496	0.23568976	0.20436342	1.02399512	0.91393197	0.62871288	0.66312477	0.86415062	-0.19762335	-0.19762335	-0.19762335	-0.19762335
SLC5A12	0.17574169	0.35297259	0.15660335	0.20341877	0.15400187	0.2366566	0.15400187	0.24087506	-0.09822012	-0.09822012	-0.09822012	-0.09822012	-0.09822012
LRRC4C	4.95550959	4.8136227	5.40606804	5.16685452	3.12073918	3.50641454	3.57772012	4.11799674	5.01781826	4.36170292	4.7444293	3.68920734	2.46107475
IMMP1L	2.91998685	4.41325849	3.17420667	4.4447906	3.51403469	3.647196	3.67293488	3.92241645	3.29260693	3.69940173	3.27509921	3.580111	3.79447889
PGAP2	4.38346755	4.18639875	4.46699684	3.43653784	3.14645095	3.21881574	3.52450214	3.14197788	3.34400789	3.40500518	4.34418422	4.42851184	2.73482981
TUT1	3.81233285	2.65263083	4.03360552	2.98930387	3.74644771	3.45284333	3.23675795	3.83814485	3.40460399	4.40666155	1.96175094	3.58004257	2.7168696
SYT8	0.89532217	-0.04221447	1.01669795	0.54480278	0.45693175	0.4950505	0.22803019	0.77431624	-0.19143721	-0.19143721	-0.19143721	-0.19143721	-0.19143721
ZNF214	3.07004883	3.49305991	2.93396222	3.57975246	3.67155502	4.6579289	3.46973293	3.76435999	3.61109376	3.33873677	3.64079368	3.45756203	3.87406853
ZNF215	2.2530331	3.15590235	1.9916768	3.71309138	2.50960811	2.59749734	1.67760364	1.56050023	3.57498398	1.9373327	-1.23035837	-1.23035837	1.38123579
HSD17B12	3.57477877	4.00671075	3.23002973	4.57935943	4.65918329	4.93660438	4.64849558	4.68251788	4.53091531	4.0039626	5.30351622	4.51417787	3.88123818
APIP	4.35589092	4.68437107	4.18818633	4.63075176	5.02462162	5.14607667	4.9127171	5.56230238	5.2767898	4.9414641	5.30992747	5.57218329	4.54636652
PAMR1	1.37736043	5.67679347	1.57516865	1.9121009	2.24901774	1.62676163	2.04365316	3.61688569	2.76035374	2.37573696	3.93652434	3.9671484	4.97113947
DGKZ	4.71207048	4.34374646	5.23252173	4.48588241	4.19426599	3.38372878	4.15211527	4.08477643	4.21619523	3.49591175	5.44087978	4.51667446	4.68133982
EIF3M	4.97613766	5.53281492	5.46531613	5.89750616	5.76430346	6.15930178	5.40430769	5.99430551	5.68653375	5.98433494	4.94556509	5.10266295	5.88659687
TNKS1BP1	7.19389288	7.00504285	7.84604261	6.35964521	6.76012905	6.19054256	6.47368118	6.62267935	5.92983925	6.76768775	6.55598097	6.39524201	6.43859706
SERPING1	8.98573086	8.34375374	8.11991569	7.13757113	8.13043925	8.17168732	7.95055965	8.24883282	7.85960807	8.19185938	7.6602911	7.82187604	8.11024774
SSRP1	6.35930703	5.83562834	6.40076289	6.16842195	5.47910708	5.72697438	5.52576391	5.29628887	5.77701597	6.18342257	5.34166283	5.79588232	6.12682004
SLC43A1	4.97862079	3.73913284	4.1231111	3.95421916	3.74720168	3.30043849	4.07101644	4.76229859	4.52661815	3.0298193	3.69677654	5.00430659	4.42289118
PTPRJ	3.15903081	2.95059668	3.04735321	3.27514515	1.79065168	0.75601132	1.23373	1.05607721	-0.31415137	2.85376071	3.77559343	3.25426222	2.05917533
C11orf49	4.70573793	4.32135442	4.52216259	4.24602442	3.92032887	3.2034763	3.97310273	4.13634287	3.7280824	4.15314993	4.08156987	4.11409426	3.52145317
ARFGAP2	5.94303352	6.05430492	6.00064578	5.43223559	5.6250552	5.24920319	5.72140026	5.90634402	5.39459613	5.90203098	5.74334721	5.80210479	6.01242087
CELF1	6.39263392	6.64695201	6.20794762	6.58229206	6.7556977	5.99940322	6.92758268	6.78531816	6.10102021	6.32311334	6.27748853	6.26934959	6.74193451
CCDC81	1.65560763	1.1845068	2.02117518	2.0320805	3.77766301	3.41565394	2.98757943	2.51923501	2.41723036	3.55551023	3.3794873	3.6277469	2.75732169
SESN3	6.14569779	7.4105285	5.88477321	8.19009625	6.53442973	6.85360948	7.42860998	6.70952785	7.5215751	5.65505856	7.41301061	6.75852979	6.06735008
ENDOD1	6.92477281	7.92016952	6.62688418	6.72278808	7.26744827	7.90202042	7.01376448	6.80747451	7.23272569	6.41605508	7.15710551	6.54756084	7.04419985
CCDC82	4.96873265	5.84603956	5.45972778	6.33603485	6.27658908	6.34516733	6.08586554	6.5013391	6.48559751	5.48356844	5.91950812	6.03300742	6.01402469
KLHL35	1.48590196	1.76941608	2.57305561	1.10590983	2.59737373	1.88764499	1.17561763	2.25493419	3.61772645	2.99613126	1.44820283	3.81916856	3.59661434

SERPINH1	6.7409926	6.39561433	7.02954615	8.10023783	6.14362927	6.59317305	6.36835201	6.10101387	6.72754346	6.83607029	6.66508199	6.66673527	6.2932396
CAPN5	3.46212712	2.06783657	3.40992538	1.99750857	0.89859006	1.23814502	1.49138909	0.98178586	3.824799	3.42016113	1.97178372	3.3293415	-0.02586291
INTS4	5.44565803	5.02055487	4.96742253	5.52083748	5.0480371	4.90027848	4.97564009	4.96193498	4.76516715	4.1483715	5.05039751	4.653304	5.51537374
PAK1	5.06840903	5.16541505	5.54660116	5.71313366	4.70170211	5.31806798	5.01638478	5.00152796	5.34989392	5.84923747	5.19525704	4.97230444	5.78151638
RPS3	6.96207855	7.25463954	7.44813808	7.88194982	6.76675995	6.26746284	7.03031724	6.3919602	6.67375765	6.83366829	6.52660448	6.66001103	6.25139148
ZC3H12C	5.74411143	5.36107204	5.00372891	5.10402258	5.91156804	6.3974158	5.91580001	6.05055962	5.5868226	5.53378306	5.43765504	5.84061137	5.92903389
TTC12	5.07965103	5.62110965	5.21188096	4.68898639	4.72147168	4.6358524	4.20610332	4.81144895	4.65098882	4.82784316	5.14753466	3.5808366	4.85993167
NCAM1	5.7861879	3.33692546	5.749703	4.27608021	4.27226646	6.65130635	4.05622491	4.19849631	4.49083159	5.711055	4.91086873	4.67897077	6.90306697
DRD2	1.03106458	0.63433771	0.98639156	0.77925036	1.49836999	1.64634669	1.02480507	2.09043077	2.25495957	3.51555691	-0.33690231	1.43993759	3.01404041
C11orf52	1.29123593	1.63612056	1.74619612	1.53646517	2.66145404	1.79755348	2.67440037	3.55692693	2.36716131	3.75310082	3.12199406	2.34479168	-0.85106555
NPAT	5.10546324	5.42483798	4.55960752	6.03338028	5.75447641	5.99536352	5.37915097	5.5734313	5.80786472	5.361716	5.45309313	5.54897816	4.95204759
ATM	6.04654584	6.95985756	6.06124164	7.44535248	6.76810259	6.66329453	7.16968648	6.77962226	6.94664078	6.46675889	6.98932008	6.76408763	6.29560752
AASDHPPT	3.57741036	4.49768328	4.21759309	5.18692612	5.00553559	5.27079672	5.02896465	5.03095851	5.09126221	3.80404562	4.91537915	5.17360051	3.8072733
GLB1L2	2.49559152	1.91193331	2.17338032	1.689498	1.82900294	1.85690429	1.71950057	1.49995863	2.17389949	-1.08572537	1.51571035	-1.08572537	-1.08572537
LAMTOR1	5.42434667	5.20740972	5.80914332	5.25143681	4.61567818	4.4739248	4.67213974	4.87090649	4.8206724	5.28747347	4.16634754	5.01740305	4.64795671
P4HA3	3.36122018	3.00227905	4.52253353	5.63015548	2.58738235	1.83443825	2.24325829	1.33065259	4.29833128	-1.56683789	5.47405432	-1.56683789	4.00194479
GRIK4	0.08594027	0	0.00124472	0.01382303	0.01695594	0.00069205	0.02201949	0.00158797	0	0	0	0	0
ST14	4.40510477	2.52146731	5.59013325	4.42064625	1.51680523	0.88035245	1.50656463	1.06299707	2.1143174	2.45698773	-1.20128206	2.62474177	-1.20128206
HYOU1	6.94959161	6.63135498	7.9226868	7.18223637	6.63001806	6.50336158	6.81887943	5.55192059	6.37017027	6.51287718	6.52504638	6.23414796	6.50362589
ADAM33	4.9743656	4.86951156	4.6489474	3.19024243	6.02565617	5.8597637	6.70190414	6.6769562	5.56579257	6.29477342	5.6099994	6.26446667	4.93863558
MTA2	6.05261987	6.03710041	6.23416029	6.02333001	5.7940823	5.66029733	5.50982026	5.2166228	5.64435233	5.96009398	5.86393343	5.46727	5.93379922
TMEM138	4.7059097	5.0831824	4.85760777	4.77962928	4.57212001	3.79080411	4.73105342	4.87940982	4.55679593	4.53871397	3.9024717	5.11647952	4.80792226
FADS1	5.80617833	5.65585226	5.28810817	5.91137224	5.49286663	5.58013496	6.21721683	5.9614111	5.46941014	4.72372855	5.90768905	5.52063603	5.30156443
TMC2	0.28560665	0.33728711	0.17339255	0.17081428	0.17081428	0.17224776	0.17081428	0.70849759	-0.10197848	-0.10197848	-0.10197848	-0.10197848	-0.10197848
ROM1	5.19385101	3.97839773	5.45519213	3.74550907	3.22835468	3.69982325	3.7239491	3.33161161	4.24952368	5.11771284	3.07898818	3.5436001	2.9248603
EML3	5.94058561	5.30329286	6.43020732	5.20377888	5.1634249	4.54677444	5.47251353	5.58696587	5.07382222	5.72530362	5.47293031	5.35740079	5.44443868
INCENP	4.80007236	4.49524437	5.4543736	4.50273277	4.05468696	4.32251312	3.99878222	3.82464293	4.27981196	4.25780572	4.70513746	4.28235	4.01452358
PLCH2	0.94539732	0.59295975	0.725159	0.47894918	0.50048783	0.74570257	0.51703591	0.84325444	2.9258474	2.48184581	-0.36595591	-0.36595591	2.56418897
CPSF7	6.48812969	6.10180566	6.40514277	6.03650843	6.32283622	5.75970818	6.60734696	6.38234026	6.06355523	6.60762511	6.41824639	6.27375089	6.11160568
MS4A2	1.81413231	2.67650505	1.51048992	2.11569408	2.77249783	2.10048696	4.02570597	1.61078967	2.97661848	3.27864136	-0.94861615	-0.94861615	3.34675932
B3GAT3	4.69309709	4.71668152	5.43085192	4.42775774	3.9951305	3.71366417	4.06917944	4.15421302	3.37735499	4.29773749	4.56740862	4.64102631	3.70097583
EI24	6.17705345	6.29835858	5.99920048	6.46174563	6.26424611	6.32426333	6.52786526	5.4449025	5.62585011	6.08820242	5.73086331	6.10878957	6.13329691
CCDC15	2.16497499	2.70155924	2.48870007	3.8249496	2.86443255	3.47074666	2.12761681	1.81234608	3.54568865	2.29903948	2.81411944	2.46341006	3.7455843
CHEK1	3.11937478	2.93986133	4.2694213	5.02235945	3.82270516	3.44493338	4.11368357	3.70407808	3.35564406	3.54537258	4.83086459	4.1867169	4.10827831
FEZ1	5.83995966	5.84902783	5.85623813	4.81968831	5.93116397	6.27772174	6.1265738	6.29878208	6.52010265	6.64508472	5.9218323	6.55441508	6.25268118
ESAM	2.40775041	4.69367832	3.11917543	4.14433044	6.66308282	4.62324304	4.92995612	4.84394855	3.85805791	3.92077254	4.92024455	4.5560283	5.12619923
KIRREL3	0	0.23873231	0.00124472	0	0	0.00069205	0.01900166	0.07716326	0	0	0	0	0
MPZL2	1.36456365	1.71644508	1.48703267	4.0212254	3.89722224	2.61201516	3.36423576	2.68129468	4.05370443	3.94490235	4.93682657	3.55429228	4.92402729
SCN2B	0.9357375	1.12424186	1.24877323	0.95701939	1.08467848	1.05805682	1.59405636	0.92213043	0.2538601	3.45974166	2.07558627	-0.41358306	4.58464808
SIDT2	6.34787399	5.53888913	6.30143101	6.10868659	5.28900955	5.07039217	5.62594708	5.26885508	4.8074786	5.63713655	5.28023911	5.62128082	5.2327493
TMEM25	5.4082872	4.85081075	5.27560437	3.32757347	5.24183012	5.14892614	4.78952865	4.89112968	4.58309409	5.11809016	5.41492282	6.08330689	4.45130418
TAGLN	9.24450491	10.2657759	9.22435776	8.51871036	10.3578802	10.9219085	10.040521	10.2155595	9.55278275	10.2451897	9.93363242	10.3762164	9.61944993
JPH2	5.26532697	6.40190189	6.14308204	3.87136688	6.58598368	6.6030257	6.10645616	6.3226276	5.33633945	7.10373831	6.19713591	7.05836587	6.45765979
DUSP15	3.00471021	-0.18902999	4.2001271	1.15647823	2.16361501	2.44325884	2.56162668	3.07484928	2.97221609	4.32452162	2.39764845	3.6849337	2.53941568
COMMD7	4.74407316	5.11375737	5.01720821	5.15462206	5.35600935	4.94137844	5.40802697	5.40802697	5.35060303	5.52324277	5.25004554	5.31475656	5.2285126
KIAA1755	4.9781959	3.87921137	4.87289243	4.33883362	4.3494979	4.14030353	5.47178932	5.32132221	4.88391605	5.88036738	5.08605332	4.84874025	4.58594854
DSN1	3.88355606	4.00709756	4.87322439	4.64563891	3.52211912	3.73434608	4.06379936	3.84823695	4.35811824	4.91635864	4.10536433	3.61825596	4.34254622

CDH22	0.00657983	0.15265395	0.11491135	0.00657983	0.06808281	0.1898031	0.00657983	0.00988473	-0.0659873	-0.0659873	-0.0659873	-0.0659873	-0.0659873
LSM14B	5.34118187	5.16709071	5.57685891	4.55776711	4.95500766	4.89058353	5.08364652	4.7154749	5.02580214	5.28736472	5.05290518	4.94843694	5.29157004
YTHDF1	6.14646726	5.56641579	5.39339621	5.38928704	5.41583147	5.23181126	5.48140184	5.24383754	5.46475791	5.42869268	5.18414704	5.18458519	5.52082788
CABLES2	4.10343743	2.74386901	3.40006998	2.20890174	2.9452229	2.35734165	2.59090499	3.86016481	2.88408978	3.98976844	3.69308298	0.98227516	3.64590872
ORAOV1	4.72324694	4.20033975	4.6971333	3.91084997	4.60626664	3.62053704	4.69856493	5.01508312	3.24260649	4.46290184	4.33928829	4.3133226	3.53366007
GPHA2	3.03266514	1.41871318	0.94012775	0.06161088	1.04155244	-0.2214415	0.29858941	-0.21984821	2.28261607	4.21308126	-0.28411466	2.82804006	-0.28411466
TRPT1	3.76578031	3.13708713	3.94131441	2.18614961	3.14677848	2.86965684	2.61749558	4.13202496	3.20424089	3.33144997	3.38391085	1.22381507	1.54705099
NUDT22	3.47352575	2.54319566	4.72631955	2.35012829	3.1713574	2.92240236	3.19308756	3.2586012	2.979499	3.44072666	4.21040005	3.11250594	-1.04510949
FERMT3	4.91129968	4.25734195	5.60176524	4.91220514	3.67906827	2.91946065	3.80197329	3.22137788	3.74943093	3.83123786	4.08451308	3.10342831	4.02651547
PLCB3	5.10827112	5.26535592	5.79377532	4.57124792	5.107649	4.39136779	5.38780246	4.67805725	4.64302721	5.48204573	4.7029304	5.15327283	5.12964434
MRPL49	6.55742163	5.94357971	6.21600624	5.58106653	5.99607916	5.89345026	5.96441653	5.37093808	6.02880405	5.84573281	5.7779786	6.04326945	6.00380826
CDC42EP2	2.58474902	4.45707693	3.95109178	4.07186451	1.97713512	1.69483448	2.04487038	2.09483935	3.36127748	-0.99884876	4.97766781	3.95826734	3.82546937
FAU	6.79397212	6.51051761	6.80194245	6.35727269	6.20547961	7.21889168	5.89326224	6.2045903	6.47625443	7.33778207	6.21071932	6.76528342	6.53651013
TM7SF2	2.25355674	2.62024773	2.0139241	0.709506	2.33331618	2.77777879	1.95022533	2.46144445	-0.69926018	2.40288661	3.33308353	3.96159214	3.16976854
TBX6	1.16473954	-0.21229304	1.28354575	0.00662516	0.46384978	0.16334655	1.28287826	0.82652257	-0.06943109	4.46535872	-0.06943109	-0.06943109	-0.06943109
PPP4C	5.01728109	4.818805	5.70703815	5.29081826	4.36733706	4.27663294	4.38111038	4.52435211	4.09395735	4.47620782	4.7856846	4.87082196	4.12626573
ALDOA	2.42906938	1.46747867	2.71470568	1.98154543	1.85952017	1.40993934	1.04351048	0.93962355	1.42890933	4.69970856	1.42890933	1.42890933	1.42890933
FAM57B	0.11244989	0.07091071	0.30245679	0.40563049	0	0.00069205	0	0.00158797	0	0	0	0	0
DOC2A	0.27619969	0.35301098	-0.20339839	-0.05306644	0.3750808	0.00274842	0.11078931	0.40982432	-0.04936499	-0.04936499	-0.04936499	-0.04936499	-0.04936499
HIRIP3	4.40204013	4.07006156	4.85855199	3.04541925	3.61820001	4.17002417	3.7457085	3.14638979	4.55295086	4.9600516	2.66917406	3.8313614	4.14740993
TAOK2	5.59106827	5.82718507	6.12151754	5.26539639	5.44389619	4.81745448	5.56941363	5.33612066	5.28808967	6.06313679	5.65431391	5.55070336	5.92113153
TMEM219	4.90239073	5.53330661	5.7312514	4.98497266	4.60967359	4.62518969	4.65959669	4.83906441	5.36300741	5.76825633	4.75933979	5.63219425	5.31489014
HMGA2	1.4819985	0.38317872	2.29791286	2.5729838	0.48639158	0.73553223	1.04626801	0.39283523	-0.94389546	-0.94389546	0.12812996	0.09201565	-0.94389546
MMP3	1.72722796	0.69914164	0.70133556	0.89666127	1.49566331	0.70036143	3.15871451	3.46725071	0.84601388	2.16179991	0.6823702	0.73411967	2.57690226
CNKSR2	3.60402369	3.55738217	2.91324644	2.56545114	3.37040593	4.10004923	3.31841435	4.6165908	3.65718645	3.67316706	3.52981875	4.08078916	3.47805083
CNTN5	0.69811597	0.73123033	0.68219527	0.68002162	1.39088484	4.03071114	1.23975286	1.67557305	0.51003087	-0.67571841	4.24513956	2.75367245	3.5946856
KLRF1	0.1478342	1.35882258	0.06650999	0.90400442	1.39992521	0.99654358	0.42641728	1.19687186	2.39338586	-0.31111061	3.18906069	-0.31111061	2.62043386
CLEC1A	0.86624494	1.6412135	1.44894501	2.11582033	3.52816382	2.14693491	2.03674607	2.50788959	3.38188659	-1.01623456	3.89240959	2.96414485	3.84871943
MKX	5.5302939	5.69448783	4.59418718	4.04131319	5.98477963	5.34692741	4.56027428	4.27241873	6.00231463	4.3516019	6.12749472	5.51893603	5.19490493
MPP7	5.72941199	6.50134834	5.21338432	4.93957986	7.12365995	7.22443075	7.07287246	7.06026307	6.7718315	7.07141659	6.74858314	7.39297798	6.82294123
ITGB1	8.63729498	9.08915968	8.99749918	9.64289889	9.75682647	10.1601091	9.98483906	10.0686529	9.67136412	9.14289323	9.3060331	9.67136412	8.96596128
PCDH15	1.93413192	1.6984108	1.70568125	1.54420395	1.78080992	2.80394841	2.93390829	1.85703864	1.37964798	5.09826344	3.06203999	3.02403015	2.36960845
CTF1	4.44449546	3.77732391	4.52161899	2.08407539	4.19818636	3.57943553	3.67168162	3.48355689	3.4933665	4.62418695	4.03069893	4.15657985	3.60856224
CWC15	4.49349084	4.87300674	4.36242101	5.17238165	4.96980117	5.6639035	5.01419188	4.95624501	5.68487044	5.477242	4.69130018	4.77169065	4.95320952
ARID5B	7.72674821	7.23044474	6.89274879	7.39744501	8.17952227	7.36475052	7.53958193	8.18462229	7.17932081	6.91261453	6.98534601	7.60747498	7.41391141
CDH8	2.10440753	1.87177262	1.85678242	1.86827898	3.32720442	6.05964583	4.46142337	5.08249928	3.29524213	6.03869251	3.76895202	4.93939263	5.17196263
DCUN1D2	4.95352471	4.83258958	4.47526477	4.20363972	4.81211838	4.25526739	5.26063768	4.77824002	4.11904611	4.44165161	4.03528168	5.00544164	5.48338996
TMCO3	6.09947065	6.33049411	6.43164498	5.96330627	6.45923716	6.01761662	6.59276833	7.06594059	6.44609539	6.76034961	6.83657167	6.6798756	6.52153654
TMEM218	3.61747612	4.66278216	4.14298531	4.35432883	4.1671186	4.49516081	4.52885335	4.05488825	4.75512959	3.81569241	4.47953259	4.1335964	4.29888664
TIRAP	5.3712442	4.70442479	4.96260779	4.27142846	4.77856819	4.60128305	4.52395031	4.85154532	4.70650794	4.94358072	4.6096819	4.44514844	4.58848073
LATS2	6.16418325	5.98490192	6.19952435	5.22704209	6.16838389	6.4949867	5.82947191	6.58259104	5.97533241	5.62130836	6.57751069	6.44082755	6.20031762
SAP18	6.25441396	6.31144359	5.80165703	5.77102905	6.04343544	6.97813595	5.72777497	5.73245713	6.72263768	6.81980172	5.67499133	6.0877402	6.03624077
KIAA1328	5.14530371	5.07111018	4.97551627	4.67646255	5.54031821	5.46955961	5.21100346	5.68243635	5.65170859	4.94953235	5.07002252	5.35119887	5.34656005
FAM124A	3.43955957	3.28526871	3.34528695	3.3143018	3.86288558	3.57846317	3.43808488	4.48265032	3.74973744	3.9954268	4.08940133	3.64736035	3.62735993
CTAGE5	2.32691887	2.79106922	2.75260226	3.95098313	3.34652286	4.28158285	3.71816975	3.72390783	3.65715537	1.920488	3.13809217	3.74030875	1.72924905
HNMT	5.7173343	6.29977299	5.49888103	6.50334412	5.56876768	6.21074763	5.31011048	5.45454962	6.57524908	6.15763106	5.78607268	5.87108275	5.87979956
LYPD1	0.47523505	0.29169364	0.70492569	2.76103668	0.34737505	0.29293461	0.44446133	0.51724419	-0.35885842	-0.35885842	-0.35885842	-0.35885842	-0.35885842

PDCD4	5.86257295	5.72354078	6.00684009	6.62765351	5.85360667	6.30104187	6.68004622	6.46570813	6.63061999	6.60284347	5.95272146	5.94203308	6.04119464
ADRA2A	1.5913388	2.81573559	1.38230346	2.59036386	2.63203288	2.38714366	3.56571189	2.02231342	0.92151701	3.11162521	3.75368312	2.54105206	3.93337075
GPM6A	1.03848151	3.45976786	0.90776726	1.62163903	3.51351957	2.63250142	1.65776464	3.00016493	4.6935635	1.80246502	5.37128563	1.76244494	4.32037564
WDR17	3.7130416	3.48966044	3.55767693	3.11962493	3.85899401	4.58525634	4.19535439	3.64532818	3.78670248	3.82521417	4.15207903	4.12680233	4.60582742
VEGFC	4.24173906	4.45248542	4.44667523	5.97273956	4.18432265	3.19463083	3.4647309	3.40882862	3.99066738	2.81983894	4.77726858	3.30714916	4.72569194
CCDC102B	2.75918028	3.87621286	2.90480099	6.65497413	3.13348106	4.08918649	2.9350121	3.88622348	4.87261201	4.32111024	4.16005777	3.57499546	4.4329157
CD226	2.19479757	2.34565247	1.72506194	2.77925651	2.74228996	2.39270515	2.31879143	2.40309516	3.46667207	1.76931226	3.95955454	2.8545177	2.53872954
CNDP1	0.15826957	-0.06599657	0.19464337	-0.04143315	0.28173254	-0.06459362	8.21E-05	0.3554563	-0.02998033	-0.02998033	-0.02998033	-0.02998033	-0.02998033
FSIP1	2.21167505	2.1475596	2.15000393	2.77597847	3.13147285	2.77699195	3.12209127	2.71696683	3.33673074	3.51737951	2.2254253	3.3419139	2.334601
DLG2	4.30275047	4.09042625	3.33428737	2.94089771	3.94467241	4.45642081	4.33809514	4.60486227	4.18431375	4.65906993	3.94617547	4.14557492	4.23476067
RGS18	1.30198921	1.90867109	1.29996674	4.38172492	1.90267973	1.74137121	1.03401395	1.3076097	3.33277332	-1.15921422	2.89071395	2.26695972	-1.15921422
PRSS23	6.61470739	7.47730149	6.92069144	8.20293039	7.64250434	6.55997932	6.72508539	6.79223572	7.60707856	7.07626112	8.1542152	7.70556002	7.40895162
MTMR12	5.82841616	6.50633966	6.11073258	5.63876608	6.40961967	6.37228894	6.48351084	6.07058031	6.06228553	6.16644664	6.47523146	6.15974797	6.25126619
PPP1R1C	0.85685769	-0.14734897	0.21501449	0.23440887	-0.11694236	0.12826961	-0.14734897	0.02180017	0.03479791	0.03479791	0.03479791	0.03479791	0.03479791
CCT5	6.40821858	6.37150535	7.37201349	7.43835259	6.0657499	6.59698154	6.40447014	5.52134863	6.12979423	6.62445704	6.48582619	6.28752288	6.77525502
FAM173B	3.87558289	3.56758734	3.84181017	4.12947956	4.02968158	4.59284764	4.46105232	3.78047746	3.6907026	3.92237765	4.44388303	4.4788303	4.17709341
DOCK1	6.52629499	6.95522671	6.39447856	6.22921487	6.59840704	6.51480689	6.90504779	6.97868005	6.81927666	7.04983351	6.7829399	6.85187714	6.87350196
DIXDC1	6.87122153	7.2759798	6.68869257	6.61327052	7.55336767	7.41324789	7.30886389	7.67137808	7.44471114	7.61639941	7.24808065	7.34379286	7.40878184
DLAT	3.21677319	3.33573631	2.93687038	3.92905668	4.06244306	4.33336613	3.75405328	3.67586931	3.78126121	4.09145062	3.80421336	4.29784288	3.98597888
PIH1D2	0.89550568	1.89878871	1.77559609	2.06972062	2.65663945	2.87035784	2.36145695	1.70430918	2.74991316	2.75776707	2.80188825	1.37909509	2.23126407
TIMM8B	4.16385491	4.06390168	4.72078619	4.67497408	3.6213721	4.62759138	3.52415463	3.53054962	3.77132808	4.51839876	3.69275186	3.31609063	4.57784488
IL18	2.88388267	5.08401025	3.55976832	4.74800648	3.0655804	2.94851341	2.83833319	2.97662309	3.84007714	2.37582016	2.95025673	2.08130894	3.92912327
TEX12	1.11079827	1.11079827	1.11322866	1.90881222	2.09339138	2.48232963	2.9293249	2.42467997	1.97198987	-0.6289671	1.6408543	3.43644162	-0.6289671
PTS	3.6878291	4.702167	3.75686062	4.8351553	4.95069384	4.15457937	4.54301653	4.72977452	4.34161509	4.3271777	4.25952305	4.56632787	4.26708267
PIP4K2A	6.61959265	6.4227422	5.96352423	6.28994241	7.23503077	6.89023154	7.06219489	6.91129262	6.60476296	6.59344344	6.51705128	6.31346263	6.37138165
C2orf50	0.26232068	0.30958357	0.95228224	0.00055304	-0.00597741	0.09151946	0.13884243	0.35668783	-0.12845261	-0.12845261	-0.12845261	-0.12845261	-0.12845261
FREM2	0.10331608	0.16431606	0.10591765	0.10476251	0.11788623	0.26856525	0.15256005	0.20294396	-0.07777389	-0.07777389	-0.07777389	-0.07777389	-0.07777389
FOXO1	6.18564108	6.49574352	5.68428237	6.09360478	7.07983678	7.37014991	6.21866449	7.19697237	6.07205476	6.03432728	6.22545235	6.32925668	6.03665345
CRIM1	5.29653663	7.15420046	5.03454291	4.05575784	7.37193461	6.43790926	6.14064517	6.47558564	6.62502351	7.52822966	6.99442718	7.3473066	6.58547046
SEC24D	6.84890114	6.58460513	6.54990891	7.92081455	6.81883729	6.56262908	6.65566663	6.8562613	6.71692185	6.41361364	7.14370704	6.77020461	6.68952827
ABCB9	3.39497181	2.89442571	2.88741595	2.70753566	2.03368067	1.96258287	2.43884687	2.04267241	2.44355947	2.66005788	3.39818731	2.85466426	1.97997211
RILPL2	5.87599984	5.55354835	5.96333981	4.36089701	5.12746089	5.19007458	5.37325602	5.38705403	6.24149543	6.09320781	5.34480343	4.86808612	5.65249804
DHX37	4.83797954	4.91065201	5.37728968	4.3333327	4.24818927	3.66752188	4.16678756	3.97917846	4.08239303	3.77756829	4.3693178	3.76047732	3.58502373
UBC	8.12870348	8.02949039	8.31715084	7.3699042	7.61999802	8.59058791	7.33491105	8.11921121	7.75470514	8.29996484	7.73459931	7.76529031	7.87343499
ITPR1	8.20319153	7.96429967	7.68583631	6.72034585	7.80028623	7.75295844	7.57453078	7.25312392	7.1485821	7.26169098	7.62570086	7.25151252	7.41206771
PRSS53	2.29273642	2.00736762	2.4014402	1.44957008	2.43975141	1.66091649	2.84167584	2.72422145	-0.89739257	3.36968455	2.09252867	3.47541083	-0.89739257
SLC7A11	3.94967271	3.33348194	3.80133918	5.66067179	3.49462746	4.3620132	4.42175613	3.67845397	4.67083738	3.18219526	4.2818728	-1.41489416	4.13057347
ENKUR	2.19098712	2.51406956	1.78849224	1.95853046	2.05240853	1.67341116	1.91537981	2.12634014	2.89213958	3.71438318	1.91423538	-1.32260951	-1.32260951
CACNA2D4	2.11609038	2.47390185	3.23197473	2.60821765	1.83765666	1.75937884	1.52486861	1.30114768	2.77507865	-0.98901724	1.76208554	2.56444497	3.50954741
DCP1B	5.52960197	4.43700555	5.20060036	4.48562999	5.39388898	5.02140882	4.80578191	4.6099204	5.39073803	5.33754527	5.28963068	5.26343858	5.19153293
CACNA1C	4.65007348	5.36197726	5.19306499	3.58338314	4.95609071	4.41722364	4.95609071	5.12757389	4.55253221	4.99883165	5.47595479	5.32727423	4.7072729
THRB	7.31935271	7.42185341	6.87303179	5.43314693	7.67753066	8.18497122	8.36270605	8.10557229	8.46272723	8.36983966	8.12417292	8.41789159	7.85635671
NGLY1	4.38467886	4.21413077	4.39430766	5.81194869	5.08972867	4.97533652	5.18727523	4.93893969	4.9555251	3.90483825	5.29512955	4.87644588	4.67133842
OXSM	3.73079479	3.35535051	2.55912265	3.08148939	2.41789483	3.12067346	3.00502968	2.21150815	3.40954596	-1.09100493	2.96663292	3.64297627	3.98763698
UEVLD	5.16548691	5.16823772	4.68385368	5.64882813	5.8835226	5.78806256	5.78065057	5.66174781	5.47465623	4.90369204	5.38828731	4.95735364	4.59298332
TMEM86A	4.82232723	3.87682718	3.79614729	4.44169018	3.49175417	3.09359974	3.02275687	3.31119618	3.69568021	4.83477909	3.67414074	4.41647281	3.32737111
C12orf45	2.62809219	2.71612527	3.10133661	3.02833021	3.03017961	3.6216853	3.88802834	3.12797713	2.39744731	2.69816122	2.35365333	2.2235622	2.81078192

BTBD11	1.64991554	0.12373962	1.07087127	0.68859346	-0.43647832	-0.44814986	-0.54137518	-0.25936554	-0.10644941	-0.10644941	-0.10644941	2.28880166	-0.10644941
UBE3B	6.2319333	5.81933514	6.0863424	5.60378566	5.96240133	5.82879874	6.35562995	5.75536316	5.98059781	5.68528424	6.23172922	5.95272647	6.34973987
ANK3	6.85410331	6.91524598	5.60402231	7.1736414	5.41267233	5.07060283	5.02362398	5.05384737	6.37198108	4.61760096	6.0615052	5.21657654	6.68504063
IPMK	3.84063254	3.71761774	3.86237413	5.44805694	4.67997049	4.58353065	4.35245788	4.29153151	4.26972733	2.94318382	4.10268548	4.01166797	3.66447129
RAD9B	0.86325454	1.18278739	1.60116925	1.48965335	1.52877735	2.01878955	1.27630423	1.16969808	2.88337581	3.31624105	2.2527659	-0.66286632	2.95344283
PLBD2	6.65250763	5.51115493	6.7158377	6.04930744	5.78964538	5.47893015	5.76489982	5.47049181	5.43567678	6.45704712	6.06678257	6.46810309	6.01324268
DLG5	6.62643678	5.91742225	6.67029125	5.61896909	6.47560065	5.88337183	6.74112534	6.95537624	5.82977924	6.88311307	6.17069322	6.44501772	6.26321063
MAT1A	0.43272284	0.50414776	-0.02814218	0.02867085	-0.03064037	0.16615134	0.1120818	-0.02745326	-0.01013464	-0.01013464	-0.01013464	-0.01013464	-0.01013464
SLC2A13	3.98865903	3.83409838	3.66290727	4.48091992	4.76879273	4.8119668	4.69826556	5.35762256	4.6755024	3.56635445	5.38852236	5.21534597	3.66045136
GXYLT1	4.39242507	5.16913503	4.53061541	5.81271388	5.30527683	5.53390791	5.44543759	5.51259841	5.41155406	4.85944931	5.10532027	5.23765731	4.12947947
TWF1	4.60171306	5.63404145	4.88811503	6.81108391	5.97367969	6.28136318	6.27030443	6.06857702	6.28163573	4.83886529	6.0306787	5.98475244	5.27163274
DIP2C	6.56743213	6.3454616	6.48023601	5.40481555	6.64818636	6.21529439	6.92223027	7.00576515	6.53662665	6.95475724	6.99282361	7.1583622	6.79670026
EIF4E	4.57479403	4.94041474	4.85932575	5.59155268	5.53151888	5.81952001	5.71229778	5.54917312	5.40818076	5.25818566	5.32323347	5.84973327	4.86400416
MAGI1	5.15800566	5.9149891	5.12414438	5.23641541	6.0951972	6.11914018	5.4602885	5.58318649	5.80278424	5.71022746	6.14950586	5.10527754	6.06362824
CSNK1G3	5.16858117	5.73687827	5.2301149	6.03134013	6.0232576	6.33786168	5.94197441	6.14114669	6.30630505	5.71673586	6.09253868	6.24215641	5.262549
SRFBP1	3.66716844	4.43214856	4.50071342	5.2411293	5.35019976	5.35030574	5.06455989	4.85077767	5.04906364	3.41127293	4.34874311	4.75162676	4.03203807
AKAP6	4.22216523	5.03098748	4.1366087	3.7203376	5.64348387	5.70322758	5.26551535	5.71424837	4.72017725	4.69381007	5.31744265	5.23170273	4.48692185
NPAS3	3.77723423	3.20484057	3.14310113	2.8530219	3.72368938	4.11932327	3.63542292	3.88056635	4.23470641	3.88046877	3.33288649	2.96092044	3.81392634
FAM177A1	5.89854833	6.25329621	6.3575776	6.66642534	6.94519245	6.72516778	6.95521461	6.89623374	6.85829176	6.31079617	6.9378514	6.5752002	6.70445609
MBIP	4.8214086	5.20596849	4.62512831	5.39592171	5.61785862	6.02542613	5.53627768	5.39613156	5.62356554	4.97563788	5.44337686	5.53221909	4.7566389
MIPOL1	4.41060104	4.54914343	4.72556578	4.44273644	5.15126326	5.54390365	5.11820201	5.35371768	5.45688733	5.12170982	4.44475044	5.17989221	4.8290583
EXT2	6.68297091	6.35186979	6.19263613	6.81016976	5.97476487	6.16078835	6.25987137	6.144736	6.11011825	6.57475805	6.9212576	6.74354938	6.50863224
TMEM18	4.39272512	3.83995083	4.66437309	4.18825364	4.53263378	4.08630841	4.23124554	4.6492474	4.38762206	4.25232404	4.06427039	4.67030765	3.26819077
KCTD14	1.23315243	0.50979226	-0.49142181	0.17608441	-0.47164942	0.84244545	0.47382073	0.39720503	0.06630603	0.06630603	0.06630603	0.06630603	0.06630603
THRSP	0.26090268	0.84392124	0.10063389	0.13907733	0.98206576	4.59598754	0.09881543	0.10113536	-0.22229587	2.87557157	-0.22229587	-0.22229587	1.85074331
NDUFC2	4.15126706	4.115047	4.7394166	4.6240169	5.04179472	5.08549479	5.19680327	4.32751406	4.88632564	5.52311596	4.51573858	5.02473273	4.35127446
ME3	4.8265046	5.45982024	4.6360066	4.47259808	5.44983697	4.37104251	4.68343864	5.23411086	5.38196079	5.02644155	5.25988805	5.42389252	5.5278884
ADAMTS12	2.53067637	3.20124055	3.62333853	5.1442306	2.82617874	3.09292216	3.10023906	3.08148574	3.82754765	4.22691038	4.59522793	3.9313243	3.40658513
NUBPL	3.81922517	4.82522093	3.79696007	4.36197148	4.32749872	4.46661809	4.51802577	4.39219339	4.82247335	3.59411419	3.8211243	4.57883729	4.21492799
NEK7	2.58452093	4.40809156	3.02178091	4.57471754	4.67815892	4.73039604	5.03291475	4.79233792	4.4994663	5.27109264	3.7741713	5.04518426	3.64764347
FER	2.06621945	3.29466445	1.87750786	3.62244376	3.62836793	4.05148451	3.38667289	3.46735366	4.01355798	3.74602392	2.88893583	3.8463437	2.28884944
ANKRD50	5.7886868	6.13950483	5.63567413	6.75340619	6.43408706	6.80566365	6.40309848	6.52584332	6.46861374	6.08425596	6.79399908	6.78226795	6.18869889
UPF2	5.79698883	6.28829512	5.90807051	6.32377813	6.49009133	7.08256099	6.22664099	6.4746491	6.41744534	6.1198077	5.45356574	5.91983712	6.00541953
CDC123	5.55726328	5.76571733	6.03793513	6.5147034	5.96526685	6.17115427	5.76459742	5.60085619	5.79964058	5.77028596	5.44433447	6.01746842	6.09004907
SCLT1	4.41786461	5.16960624	4.67180361	5.87202684	4.90526951	4.90470933	4.85905502	5.16554951	5.81489891	5.60977824	5.06460848	4.8075562	4.76200298
CCDC3	8.43981675	8.79622693	8.1543964	7.00646939	8.34401296	8.34401296	8.78677389	7.7189556	8.03518964	8.58386389	8.33013275	8.12623252	7.83320887
C4orf33	3.16023239	3.55095	3.22982273	4.34484664	4.16477752	4.45915731	4.45762692	3.87671656	4.3487803	3.65201986	4.70144843	4.74485512	3.52940965
FRMD4A	5.85699025	6.28588028	6.28090293	6.21405245	5.81085579	5.09567313	5.80096122	5.90174531	6.27767336	6.33152854	5.73620648	5.53023212	6.3484223
PTPRO	-0.12611424	1.16042828	0.20348131	0.61708192	1.14199512	0.7042564	0.04835219	1.0483719	0.45388582	0.45388582	0.45388582	0.45388582	0.45388582
EPS8	6.67426665	7.24499563	6.38221108	7.13142529	7.61719601	7.83353112	7.38479433	7.32200918	7.56127582	7.0070408	7.24250903	6.77710181	7.64375862
ACAD8	4.32194829	4.10723698	4.78521178	4.17926572	4.54219318	4.18406368	4.8016173	4.53794761	4.5791254	4.67005407	4.78305069	4.93906664	3.88305083
THYN1	5.70785343	5.41644595	5.31086398	4.70238357	5.43258859	6.06837796	5.36073239	4.89166073	5.12749371	6.12425781	5.19517629	5.73130375	5.65457617
VPS26B	6.0596416	5.71538919	5.90846709	5.60826926	5.59436799	5.29123544	5.85934497	5.61111706	5.489704	5.47745998	6.02384919	5.78067905	5.80769998
NCAPD3	3.95168342	3.29094146	4.48382439	4.28757871	3.2740525	3.11435208	3.65398283	3.00300487	3.27198586	2.44133867	4.91169536	3.47322178	3.30538124
VTI1A	3.9766929	4.04323775	3.53981281	4.34569431	4.52005981	4.34904392	4.45217606	4.30225632	4.62066697	4.54733697	4.55128764	4.0792522	4.63863068
QDPR	5.61962675	4.99246086	5.69464094	4.52500763	4.60809588	4.91574186	4.80558843	4.33080547	4.80213986	5.40806307	4.8606662	5.41744583	6.1011505
FAM160B1	5.73672102	6.19288384	5.65641626	6.2026242	6.46953843	6.38243731	6.54102696	6.52657955	6.10183615	5.67261517	6.47732951	6.3746864	6.13943867

ANO4	0.82062125	0.96314943	0.53331423	0.98457694	0.53072519	0.77719005	0.53072519	0.63176759	-0.36939549	3.28730339	2.61871115	-0.36939549	-0.36939549
TEX9	0.53851767	0.86067709	-0.01678958	1.73134593	1.11239929	1.75866963	0.50654374	0.36534988	-0.17468293	3.72074671	1.63805033	2.21505179	-0.17468293
MMAA	-0.06793047	0.00255148	-0.06984771	0.19027497	0.77039856	1.29907216	0.25733235	0.33473623	0.22089501	0.22089501	0.22089501	0.22089501	0.22089501
ZNF827	5.87249817	5.2344291	5.77416588	4.50465927	5.32912724	5.91349792	5.2663994	6.03886109	5.68477134	6.16728285	5.64480967	5.52058214	5.70233614
EDNRA	3.21840504	3.28885941	2.76080942	4.30421569	3.5721637	4.63390114	3.8560226	3.09958316	3.21254579	3.79470547	2.91969836	3.70900366	4.40035857
NR3C2	5.45315074	5.09647719	4.52270499	3.47839694	4.66720045	5.26589853	5.20212839	4.78695534	5.02331816	5.14273884	4.73065479	5.43656365	5.81762375
DPYSL4	1.95958502	0.19093372	1.59371653	0.91053858	0.63201321	0.31965557	0.19093372	0.64092134	-0.49762739	2.00337787	2.52121318	-0.49762739	3.66949574
VENTX	1.58365907	2.78015278	3.0462205	2.50221339	1.05345334	0.27364688	0.64725238	0.53249389	0.38415824	2.96344517	1.82561471	2.64028157	-0.55371483
ADAM8	3.91911258	2.6410142	4.32850318	4.09171802	1.64515774	1.27295118	1.76485194	1.70803378	0.75394867	2.30194225	1.45381016	2.71878358	2.04755824
ITIH2	0.24070501	0.9694549	0.08602596	0.33969128	0.36221941	0.20043572	0.08351381	0.37600395	0.00333403	0.00333403	0.00333403	0.00333403	0.00333403
KIN	4.293518	3.99541179	4.16449102	4.83220976	5.2537998	4.9048659	4.97955343	4.83190459	4.50293511	3.95982248	4.57894343	4.90896084	4.14867888
PIGF	3.29360134	4.0006874	2.66433461	4.71426266	4.11261282	3.85538674	3.53797771	4.09654258	4.2295782	2.9242537	3.33442055	4.3431955	2.64515327
ANKAR	4.2242343	4.78430241	4.05518248	4.65897063	5.22483659	5.25442593	5.25634852	5.5186176	5.18818198	4.20038864	5.16012208	5.69780004	4.54535665
INPP1	5.13342552	4.4923765	4.54375133	4.49267984	5.5209053	5.56121464	5.48304012	5.45083775	4.72852752	5.40214793	4.37574828	4.55536064	6.03962335
MFSD6	5.10900824	5.13195586	4.89831863	4.74875691	5.91027347	6.0775684	5.12000149	5.55298681	5.62052103	4.70547827	5.359645	5.30064548	6.32772721
RNF144A	4.50680311	3.05397873	4.33379114	5.72350022	4.03585622	4.04268901	4.48738386	5.16462687	5.03607261	5.37963154	3.73724583	5.12480978	5.20296416
ASAP2	6.61610383	6.86898872	6.60453392	6.50544715	7.03471828	7.07933267	6.73774556	7.90664415	7.31217686	7.14343991	7.41525005	7.31072682	7.33804623
ADAM17	5.28565656	5.3719299	5.05603505	6.51654426	5.68756975	5.61706684	5.72811151	5.70375343	5.47903619	5.02215705	5.28011464	4.64249388	5.36309422
FLI1	2.4040827	2.97903252	1.15685951	2.8149245	2.3268076	0.36918895	1.6316961	1.5783667	2.63211509	2.80582836	2.47046115	2.49827472	3.62251444
KCNJ1	0.14308274	0.12640267	0.34527372	0.12640267	0.18041675	0.60962272	0.19516009	0.51398333	-0.1138777	-0.1138777	-0.1138777	-0.1138777	-0.1138777
WWC2	4.11501278	4.831085	3.77030104	4.19245197	4.65837736	4.87040125	4.48260537	4.65434505	4.58124683	4.19538389	4.93559047	4.75038087	4.14610164
ACSL1	6.04965572	6.10485853	5.83752452	6.71699748	5.97212279	6.45487246	5.86306058	6.10234955	5.44603168	6.22574486	5.72161982	6.13398859	6.06952822
SLC25A4	6.07368058	6.06498414	5.90296705	4.78534903	6.8213915	7.06280369	6.42629751	6.61721681	6.39265738	6.8123564	6.47088893	6.67047122	7.55071062
AMN1	4.33531978	4.84097236	4.05379967	4.35905061	5.27360235	5.03654207	4.84648041	5.09516707	5.01960243	3.95481795	4.87946203	5.13649144	4.35234819
BICD1	4.23323702	4.03466172	3.88207884	4.09583783	4.51028459	4.92101805	3.81940427	4.18914967	3.81761579	3.12808412	4.45030776	4.05463352	3.71294517
SAV1	6.55326492	6.37509737	5.2757792	5.50831236	6.5583976	6.52655577	6.41001389	6.53821502	6.23779467	5.10687282	6.21905916	5.83222481	5.75627033
CCDC122	2.72542798	3.33047474	2.71998803	3.52327002	3.50531686	4.57308629	2.59059625	3.46104167	4.31257663	2.78926812	3.44970565	3.10027909	3.98214444
SERP2	1.42047209	2.6479429	1.82582159	0.48857872	1.24913043	1.39469422	1.11263685	0.12790882	3.84744094	-0.14018616	-0.14018616	-0.14018616	-0.14018616
NBAS	5.53241616	5.94051247	5.53462372	6.363429	5.80394512	5.95668597	5.90286037	5.74453172	5.76712058	6.09938984	6.21760735	6.1070188	6.17191692
ZNF385D	6.79388755	6.08020208	5.89473696	5.90537621	6.81825543	7.02744387	6.89211393	6.96558462	6.43267128	6.64641373	6.18435093	6.53195309	6.10923613
TDO2	0.22672474	0.41413529	0.3268965	3.32514229	0.2527694	0.22790989	0.22672474	0.22944418	2.93780873	-0.23423679	-0.23423679	-0.23423679	-0.23423679
GUF1	4.66917499	4.90251107	5.21371072	5.41229527	5.5698618	5.61241868	5.49227589	5.33217212	5.28882219	4.79504225	5.12225745	4.9327195	4.88751243
SLC35F4	0.09462718	0.09462718	0.09719656	0.09462718	0.09462718	0.33054704	0.6053665	0.29814761	-0.13978644	3.06460494	-0.13978644	-0.13978644	2.68495545
GABRA2	1.17321135	1.40843922	1.1524077	1.271816	2.15212898	1.37179089	1.6741478	2.01728541	-0.72337639	3.96043689	2.87913526	2.9983437	3.40781708
SACS	6.25380056	6.96831831	6.51607344	7.52246285	7.99772328	7.47081599	7.27279258	7.68082239	7.12266051	6.88914935	7.10715562	7.98419553	7.51443975
PABPC3	0.90680417	0.86187796	0.91721508	1.36122107	0.9520413	1.00565807	0.898053	0.97402159	0.10677776	2.33243934	0.79798039	1.25264873	1.1459298
CENPJ	4.13224358	4.23874033	4.3833292	4.82977213	4.5969212	5.50618038	4.63453127	4.98751232	5.11114053	3.83913687	4.39831213	4.65615168	4.74049893
FBXO4	3.15940296	3.70074991	3.84924739	4.04723431	4.3460507	4.71028442	4.18933586	4.93115039	3.56100218	4.92537501	4.35464071	4.65242223	2.77180661
CCL28	4.26552382	3.56798376	3.91205831	3.03439783	3.96385525	4.92986889	3.55556465	3.98681778	4.68163061	3.96342999	4.05361163	4.18631674	4.14463462
PARP8	5.92166105	6.31466255	5.41709512	6.56388729	5.8323102	6.01293425	5.58865888	5.54218627	6.25963769	5.48393135	6.08067998	5.50055875	5.86732747
GFRA1	4.13656388	4.7091285	4.789026	3.05766335	4.42019301	4.95347214	4.56641321	6.95410748	6.304579	6.78706771	6.19258203	5.85680921	5.40255973
DST	8.27957958	8.89429144	8.38873168	8.74943048	9.09092895	9.43716741	9.15398811	9.49992206	8.87858436	8.72253281	8.57180298	9.14068564	9.04985936
BEND6	-0.10435384	0.35091656	0.5098274	1.66077016	-0.49533075	0.54023077	0.00607942	0.10674348	-0.12318566	-0.12318566	-0.12318566	-0.12318566	-0.12318566
TIAL1	5.68738286	6.03107583	5.67665703	6.39661378	6.35280466	6.27799177	6.45930809	6.51681658	6.32162901	5.84595379	6.07801155	6.21232102	5.91174638
BAG3	7.03339875	5.48699443	7.00944584	5.91101056	6.27411012	6.28822708	5.43858614	5.79921617	5.60434257	6.21508243	5.3758221	5.66790181	6.43157711
GLT1D1	3.09132427	-0.00104558	2.24529711	1.44692819	0.65731345	2.97434178	0.66080494	2.71387729	2.38721375	4.34801278	-0.71100481	3.52047305	2.80385627
SCHIP1	0.16219045	0.22335036	-0.34910575	0.0771014	1.4158104	0.48027593	0.48736725	1.66464188	0.2166462	0.2166462	0.2166462	0.2166462	0.2166462

KCNE4	4.6373976	3.86573221	4.76184212	5.22873265	4.12824002	4.90617222	6.06323695	4.03378239	4.57724296	5.19544627	5.36289231	5.10864148	4.08475438
AP1S3	2.34725129	3.08006089	2.93916467	2.9377228	1.94156694	2.43359519	2.75215543	2.26820705	2.76095931	2.19041935	3.2196887	2.35237334	3.7832715
RABGAP1L	4.94398615	5.70706813	4.80791342	5.866365	5.77167398	6.03859279	5.51693826	5.67730774	5.55088368	5.52284557	5.55637611	5.78213729	6.81317112
CCDC74B	2.54959897	1.22392812	2.33604509	1.73965229	1.44942836	1.0873676	0.91511571	1.16102406	1.4185767	-0.72340004	2.36204392	2.99905886	-0.72340004
TMEM56	-0.34270964	0.65325105	0.05580261	0.2653191	2.02856236	2.8315666	2.14209172	2.78375332	1.53975326	3.07023138	1.78080468	2.05678794	-0.00713376
MZT2B	4.57661153	4.57565789	5.6404254	3.95080466	3.30990546	4.75211828	2.81758948	3.4507725	4.46902436	5.21468262	4.2868021	4.6853544	3.55709654
ASTN1	0.14942008	2.57861992	0.48270479	0.7682152	0.31579164	0.32440163	0.21089116	3.90054155	3.43734492	4.3247351	-0.39188395	3.08157333	1.97977069
FAM168B	7.3452223	7.2751072	6.68349001	6.36362534	7.14602273	6.8990201	7.12250667	6.78380925	6.49288384	7.25232119	6.97405384	7.30618867	6.66403437
PTPN14	6.58077131	7.18303136	6.46997537	6.70562042	7.38622731	6.92985588	7.08001541	7.30660638	7.08649687	6.70544335	7.71893351	7.04029809	7.04986781
MGAT5	2.65495789	1.84932617	2.0871694	2.55150323	1.24267505	1.10707702	1.47996332	0.58514489	2.80303741	4.2071545	0.29115496	3.06796766	2.16753445
TMEM163	0.90759147	1.12157541	0.43072328	0.78320037	0.3590294	0.34183039	0.39012787	0.34366532	1.45970871	-0.16744504	-0.16744504	-0.16744504	1.85691413
HSPB8	6.34543488	6.66117925	6.55789311	5.07138936	7.73064857	7.13043804	7.25878852	7.02506972	6.53380884	7.72048497	6.84454492	7.48401204	7.19923644
GEMIN6	3.40005089	3.64748088	4.27278264	3.08538743	3.59967994	4.45380138	4.05150628	3.14482363	4.30124357	3.66263559	3.12902979	3.50318777	4.36305124
RNF219	4.1843443	4.76016342	4.84841095	5.59281796	5.4584059	5.97977257	5.35218792	5.6788974	5.56265191	4.89182228	4.28602657	4.9257853	4.81970614
CYSLTR2	1.60042539	2.99373475	1.46522016	2.35751626	2.22492783	1.85190691	1.0392657	1.2782336	-0.78435942	2.90539754	2.93357198	3.90227682	4.59813936
GRID2	0.37985538	0.35765439	0.36023963	0.36141875	0.37443028	0.86568965	0.52269215	0.63593398	-0.22907148	-0.22907148	1.83004442	2.77877849	-0.22907148
ARL11	3.04510847	2.35729642	2.97437529	3.62188222	2.01545312	1.5347988	1.79919453	2.26817641	-1.06585876	2.6423432	2.54438635	0.67070039	2.80702395
SETBP1	6.09522116	6.5983675	5.83701017	5.32745488	6.19984436	6.49439576	5.80973265	5.98613899	6.46806063	6.43212379	5.94099457	5.64912321	6.58206779
EPG5	1.51714948	1.65120018	0.71297318	1.54495382	1.44266779	1.33626044	1.4906401	1.19891652	0.42424252	0.42424252	0.42424252	0.42424252	0.42424252
PSTPIP2	4.49371369	3.95963227	4.22705153	4.35112657	4.55948779	4.48077157	4.09142331	3.77098903	3.83626902	4.3111074	2.62514068	3.47806359	4.25852451
ATP5A1	6.16149742	6.76469439	6.53905171	6.91828615	5.96379567	6.39654672	6.56168379	6.21708053	6.17149593	6.82425091	6.40129561	6.91737615	7.42043523
HAUS1	2.9095926	3.54023696	3.35615183	4.36066413	3.6651642	3.94981366	4.02593508	3.78521472	3.37950644	3.94607047	3.71865199	4.18424916	4.19337881
C18orf25	5.13370968	5.25967973	5.11677117	5.73035129	5.80596788	6.04991788	5.84164839	5.78636169	5.35492832	4.50184642	5.58230324	5.50097173	5.68306418
SPC25	-0.33847758	0.0122974	1.46346131	3.30703438	-0.48398597	-0.48291537	1.17920775	0.64354889	1.18164145	-0.43693864	-0.43693864	-0.43693864	-0.43693864
PDK1	5.645402	4.79266821	4.39953791	5.8158673	5.50297718	5.24059897	4.72460952	4.73376591	4.37899068	5.12149877	3.94882708	4.55848283	5.01908757
PDE3B	2.40004211	2.18348212	2.5158209	2.83304739	2.90749877	3.79310769	3.82770979	4.59524818	3.63060134	1.92812273	3.07048841	3.94772434	4.01571871
TCF7L1	4.70384919	3.63770339	4.67686377	2.3243338	4.59497971	3.61918694	4.0471331	4.22451547	4.10702335	4.95941796	4.54106997	4.04798257	3.73649736
TGOLN2	7.71038282	7.92483707	7.91421304	7.8104849	8.00575624	7.80731629	8.27544784	7.69024062	7.70887816	7.818063	7.82178303	7.75044329	7.89958046
SH2D6	0.06077234	0.29901615	0.46605812	-0.12367689	0.00414621	0.11418896	-0.18583955	-0.18358157	0.00847365	0.00847365	0.00847365	0.00847365	0.00847365
KCNK13	0.22537089	0.21724963	1.11126848	0.11115373	-0.23380253	-0.01461118	0.37743553	-0.09267295	-0.09560628	-0.09560628	-0.09560628	-0.09560628	-0.09560628
UHMK1	5.51014704	6.36913046	5.6010257	7.07007914	6.54890874	6.51407296	6.74425527	6.20999069	6.17528241	4.99747685	6.82829763	6.43940051	5.3467942
ATG10	2.21804282	2.27490662	2.35626423	2.19115737	3.44298587	3.0112766	3.13191785	3.14042008	4.5017974	-0.98005967	2.40420493	3.8622552	1.12603217
POC5	4.09868325	4.33078943	3.57088308	5.0083924	4.81711318	5.08293931	4.66331232	4.78069226	5.03414447	3.76716939	4.59512302	4.24968269	3.13633723
SPOCK1	7.19297191	5.05618141	7.09181254	7.81085212	5.89851673	6.52255786	5.923351	4.79905732	5.69240793	4.90289736	5.3651144	5.11158577	6.50137862
FAM151B	-0.48607004	0.18654237	0.20933061	1.93136638	0.17765063	-0.10098849	0.79911259	0.29835281	0.07820003	0.07820003	0.07820003	0.07820003	0.07820003
TADA1	4.09057228	4.26120374	3.87124066	4.56125306	4.80573534	5.3361809	4.46951165	4.60380919	4.69585837	4.58195825	4.88437766	4.5393291	3.94402293
GUCY1A2	1.94008898	3.53723202	1.99817228	3.8591352	4.16380142	3.32732232	4.29855809	6.17363225	4.72825093	5.19213974	4.86579783	5.00409866	5.0939413
CWF19L2	3.87353847	4.90952799	4.49533028	4.79784117	5.04881677	5.25213063	4.9519092	5.11962462	5.5777713	5.22285803	4.6487043	4.75596769	5.07391574
JMY	3.50865573	3.35959051	3.24945538	3.29338255	4.1458783	4.59197747	4.31834079	4.55705345	4.2153648	4.77715392	3.62670185	4.38497365	3.93521476
HOMER1	3.95209116	4.01942441	4.00917138	4.91893575	4.35969513	4.95756306	3.97854955	4.64714648	4.68380564	4.00998498	4.56263223	4.26764463	5.02962455
XRCC4	2.59212721	3.82911946	4.14202395	5.37976057	3.89328663	4.6311207	4.00586549	4.18138542	5.22648979	3.74789155	3.17696245	2.6659547	3.18716978
ZNF547	2.34298014	1.86886268	1.88006982	2.85548102	3.17028837	3.32775041	2.96306545	3.83715212	3.57968986	4.11972703	3.82690306	4.14177299	2.87479591
ZNF773	3.27163717	3.29579744	3.44203934	3.24000747	3.75955759	3.57692853	3.56668749	3.89955567	4.25143863	3.24044426	3.12195776	2.73697873	3.96908035
ZNF776	4.26789425	5.27163937	4.42027958	4.95671577	5.08887789	4.86782043	4.93364736	5.0407096	4.92009652	5.42190341	4.81710766	4.75295539	5.44250051
ZNF256	3.81706155	4.8485391	3.63684284	3.86544763	4.67839044	4.82657642	4.26366483	5.03823244	4.90567063	4.07773759	4.81627408	3.6093711	5.06869551
SUV39H2	3.11836791	3.52116917	3.99866471	4.74973325	3.67361643	4.18454317	4.62447029	3.68110798	4.02776085	3.92999595	3.44010963	3.83305147	3.90087822
DCLRE1C	4.84662249	5.04501776	4.40093689	5.18923336	5.08364417	4.65682481	5.2720537	5.03170967	4.88457355	3.51572946	4.98571116	4.78145724	4.60177801

OLAH	0.675617	1.93540362	0.71210663	0.53275224	0.61113372	0.86099728	0.71822225	0.80236753	2.4769315	-0.32481368	-0.32481368	-0.32481368	-0.32481368
RPP38	4.25760528	3.0831121	4.05554443	3.30599874	4.13738868	4.57737506	3.69834918	4.08568179	4.20677725	4.34212901	3.44637052	3.67215638	4.24218053
NMT2	5.02631955	5.3483716	4.32284661	4.3270141	4.93610118	5.04883982	5.01821454	5.10457698	4.9577199	5.36436216	5.12174999	5.17031174	4.46573922
ZNF837	0.70283117	1.5726781	0.78256334	-0.18402863	0.3071191	0.21405547	-0.15666963	0.55261333	-0.00079895	-0.00079895	1.6949672	-0.00079895	-0.00079895
USP12	5.21093111	6.54965664	5.40788529	6.14468944	6.64237873	6.91915162	6.7711058	6.74707699	6.2655959	5.47280458	6.6168607	6.62356572	5.41228771
CCDC50	6.2809164	7.11924125	6.33523919	6.49000199	7.27793955	7.10694107	7.11952905	7.41656259	6.85992939	6.18614924	7.11922748	7.2175407	6.48050552
CAMK4	1.60221907	1.70842335	1.51592114	1.84464842	1.65232709	1.98401306	1.86543527	1.82015569	3.21622667	3.81884709	-0.79646729	2.60879165	-0.79646729
TRIM36	4.08042847	4.38410627	4.06879614	3.5478586	6.55945945	4.87446354	4.74324	4.36322752	4.76273474	2.84083108	5.17027126	5.38039092	4.85319215
ZFP36L2	8.08252157	7.24527376	7.39159482	6.81097665	6.52458974	6.88939992	7.81028765	7.44198903	6.72067211	6.4663137	7.00585803	6.81054551	6.04540822
PAN3	4.36456083	4.94721349	3.94876891	5.07112467	4.86728044	4.62748361	4.89995078	5.17639619	4.33672784	4.76860539	4.82084423	4.55079581	5.01041319
PLEKHH2	2.42696658	3.32665586	1.93385535	1.56402407	4.14419911	2.75659251	2.65553185	3.67413549	3.7531493	2.59257098	4.07256464	2.88857479	1.24425012
PFKM	5.01580661	4.48763311	4.6496124	3.63312112	4.27363934	4.6558445	5.00902262	4.68427226	4.57650303	5.23476204	4.35553393	4.77144887	4.96456088
TMEM123	5.73605192	6.2938705	5.43056524	7.02940138	6.95201642	6.93802608	6.82428421	6.9001485	7.19223806	5.44663799	6.8076436	6.35756596	5.91573768
GRIA4	1.02746497	0.72292593	1.1508476	1.01797696	0.44532712	0.46402339	0.42750337	2.93498963	-0.4128756	-0.4128756	-0.4128756	2.6733921	-0.4128756
IGSF10	3.25713691	4.58128141	2.95768264	4.04583375	4.61698902	8.46540047	3.96283312	6.6217378	4.82801339	5.70976356	4.53245734	5.35438502	6.98170754
SPEF2	3.46942503	3.96555674	3.20063788	4.15697121	3.93757207	4.54952375	3.42740938	3.59352952	4.6932349	2.3729708	2.58845764	3.40747261	2.76856621
SPARCL1	8.77470114	9.02540175	9.29207417	8.28255748	10.0230573	10.6078985	9.83843317	10.535279	9.8520454	10.5558879	9.06956369	9.8520454	9.9270142
MBNL1	6.31967369	6.47283888	5.82384819	6.53646566	7.16772909	6.89641703	6.82653676	7.30051656	6.2327659	6.74686799	6.41014995	6.5052036	6.73240783
GPD1L	4.8326496	5.53464991	4.87125535	4.54545024	6.27894574	6.98265891	6.81259686	6.08090392	6.2539015	6.4772129	6.01700583	6.59484827	6.79517278
GJA1	8.06568608	8.01031363	7.57759671	8.42283389	8.37681463	8.97844044	8.74626415	8.72601181	8.16975252	8.18729679	8.29565282	8.68344022	8.53952324
CCNO	1.3847846	-0.01756225	1.09163886	-0.14274334	0.63852586	0.04944883	0.36554758	0.80854277	2.74845739	-0.23354711	-0.23354711	-0.23354711	3.26497649
CLEC4F	0.00586242	0.1553803	-0.07819021	0.20752226	0.13115861	0.06575411	0.11592165	0.35154241	-0.01830031	-0.01830031	-0.01830031	-0.01830031	-0.01830031
SLC30A6	3.32282085	3.80659748	3.32479342	4.92613256	4.64490726	4.25867475	4.56617085	4.52183232	4.12101901	3.35074068	4.27729898	3.46367402	2.8793066
PELO	5.32962051	5.47791742	4.8752972	5.02734027	5.38559216	5.90215704	4.81504646	5.61775704	5.12636599	4.92930252	5.95357857	5.51006522	5.03064607
RASGRP3	3.34898409	4.59841494	3.92116432	5.70981388	3.91826556	2.86867873	3.34932485	3.67804365	4.66793431	3.52990354	4.53778333	3.8397113	5.34033211
SAR1B	5.46647675	6.20927602	5.72730877	6.17184399	6.48219534	6.56289505	6.57088555	6.50203802	6.22792738	6.01389353	6.45295533	6.33424134	5.67533974
CATSPER3	2.46013911	2.41365211	2.75188767	2.4196557	2.20227051	2.63955421	3.2192726	2.86366947	1.73828699	3.14709176	1.30362429	3.59015762	3.51025046
GPR180	4.41292647	4.81660528	4.49044661	6.01033	5.43506704	5.43430787	5.42330204	5.37362702	5.32903584	4.27921117	5.06920017	5.2578088	4.34951059
TCTEX1D1	0.49165241	1.75010921	1.35588869	2.53569048	1.47288585	0.23733755	2.00185498	0.76086147	0.22170301	-0.73415313	-0.73415313	-0.73415313	-0.73415313
WDR78	4.10654335	4.41277528	4.4450175	4.69640789	4.65542856	5.36357693	4.59388904	4.30263455	5.17311134	4.75728241	4.38096552	4.2437188	3.7387949
ANKRD22	1.16622185	2.72013488	0.80404577	2.55356847	0.94240236	1.250091	1.3865547	0.62236815	2.37081816	2.9425734	-0.72220532	2.40724049	-0.72220532
FARP1	2.33990601	1.67611152	0.52661172	2.08713241	2.9630353	-0.94222429	0.84233474	1.20483065	0.13979659	0.13979659	1.9994656	0.13979659	0.13979659
IFIT5	4.76554209	5.52159462	4.69609651	5.97071016	5.57638062	6.00272734	5.47346188	5.9374919	6.0172236	5.94004704	5.70357915	5.63379517	6.05959238
SLC16A12	1.03842228	1.35632184	0.418531	0.63271029	1.18657489	1.88048187	1.33134678	3.00209855	-0.57656779	-0.57656779	3.78347636	3.3125999	4.45680214
PANK1	2.66738507	2.32105759	2.58611831	2.76657337	3.02470088	3.60968695	3.5915234	3.4045194	2.85699486	2.79144783	2.86252465	2.12321111	3.04348789
PRDM8	3.94349832	3.20778488	3.24652387	2.09633554	2.55229568	2.7665389	2.02853029	1.26911178	3.43807763	2.45843333	3.95009716	2.27866003	2.03507056
BMP3	0.96321947	1.36075526	0.96581842	0.96725521	0.96321947	1.1895156	1.32152861	1.12416686	3.53198687	-0.59328148	2.2197989	1.39592113	2.09656527
HHEX	1.85297244	3.67456505	2.67749399	4.41487248	3.67792411	3.16110754	2.77168306	3.87962601	4.48807659	4.13440743	4.67815632	3.8245521	4.05176065
UTRN	5.73845413	7.36327764	5.68397618	6.40443905	7.06667227	6.86872767	6.26100108	6.69687505	6.65401808	6.05396728	6.6059877	5.41722125	7.45211924
GRM1	0.05529276	-0.04803252	0.20267767	-0.01303697	-0.04803252	0.18546636	0.06882016	0.0889456	-0.05968529	-0.05968529	-0.05968529	-0.05968529	-0.05968529
PTPRK	5.91624561	6.02405501	5.46495764	5.74690545	6.19427087	6.70450028	6.25537504	6.07365187	6.03171279	6.19704858	5.83790183	6.51853736	6.74661609
GGPS1	4.29786723	5.08289653	4.29192123	5.3221066	5.40330037	5.27847935	5.18615322	5.00738725	5.66154475	4.82952815	5.18070807	5.54586504	4.5020148
ZNF117	4.08495905	5.46806061	4.71561168	5.60613806	4.5355432	4.90697365	4.73457362	4.95373461	5.38188813	4.02059573	4.90222541	4.59993059	4.75617405
RAB3C	0.42353358	0.86351906	0.42609287	1.07310178	1.25398934	0.80942827	1.56363308	0.8829268	2.66545567	-0.32816784	2.22202722	2.8989664	4.06646563
MARVELD2	2.56428427	2.37156169	2.21881977	2.17503014	2.33181791	2.74369241	2.63550703	2.93375101	2.27152114	2.98476625	3.28079255	1.89166745	3.63813636
RAD17	4.77744367	4.79987268	5.09056136	5.78645822	5.50324624	5.93627472	5.8718278	5.62115205	5.9334447	4.51942601	5.739739	5.86016586	4.30526328
MED21	4.91788432	5.5333794	5.02014799	5.95750764	6.31674689	6.11583446	6.15588403	6.06859081	6.58591723	5.40271113	5.98802401	6.00544897	5.29404921

PLOD2	6.02426622	6.35396207	6.4966026	9.46809553	7.41395305	7.24920531	7.52243272	7.28979587	6.71690016	6.11741022	6.42752962	6.58714364	5.71107312
STK32B	5.13918609	4.47820651	4.95990482	4.28364051	3.98259486	3.54450269	4.22583303	5.46126975	5.49790144	4.84149066	4.88294461	4.05818731	4.56715262
NRSN1	0.64573661	0.11111674	1.14647181	0.40676183	0.11111674	0.11251326	0.49375496	0.11432121	-0.13063768	2.85863942	-0.13063768	-0.13063768	-0.13063768
JAKMIP1	0.91540432	0.5515803	0.50330318	0.41648338	0.55305064	0.37706044	0.37562135	0.69571827	0.11166758	0.11166758	0.11166758	0.11166758	0.11166758
CPB1	0	0.4569423	0.00124472	0.1106261	0	0.00069205	0.01965033	0.00158797	0	0	0	0	0
SREK1IP1	5.02034863	5.38893598	4.84236547	5.43574415	5.60020971	6.07499461	5.73652749	5.66000743	5.82330441	5.07109837	5.74285306	5.64168506	5.0879888
LGI2	3.00548659	4.18317961	4.06238008	5.17002945	5.1225236	4.24546878	5.15370447	5.24284343	4.83615218	5.8337849	5.17643464	5.660337	4.55401562
CWC27	4.84682174	4.89775708	4.60703073	5.11189073	5.0118186	5.8138132	4.66758814	4.17961071	5.62390618	5.1152705	4.0936507	4.16085026	4.06921186
MR1	6.23069173	6.09012315	5.75232325	5.8024896	6.06024766	6.26107905	5.69838857	5.71374743	6.10193142	6.1718211	6.26229947	5.85637529	5.66973865
SRP19	4.7425655	5.3692431	4.74988672	5.76467946	5.42697316	5.26698902	5.632497	5.06865649	5.54893396	4.82145456	4.86470916	4.85413519	5.45925194
CENPH	2.43277	1.23377351	3.52280762	3.02756364	2.01897685	2.82255828	2.19983257	2.09872046	2.59554818	-1.23383632	2.82667215	2.65218927	2.59177439
CDYL	3.31724992	3.17642797	3.17200413	2.88659109	2.92431291	3.63434181	3.0714961	2.92064889	0	0	0	0	0
CARHSP1	5.29721869	5.29175365	5.47061924	4.39262447	5.18380646	4.45251555	5.65858407	5.04205	4.40773852	5.24768632	5.42362269	5.30457972	5.06557997
TEKT5	0.82428004	0.5246999	1.29905151	0.78641861	0.97628366	0.04346903	1.04117401	0.7803015	-0.31340721	-0.31340721	-0.31340721	-0.31340721	-0.31340721
BANK1	0.64318691	2.00094812	0.55183828	1.91542392	1.01256093	0.77355609	0.68053583	1.00717964	3.94620015	-0.49100876	-0.49100876	2.85992865	-0.49100876
TXNDC11	5.58259873	5.59893829	5.21803101	5.76054377	5.49672818	5.33790838	4.99886845	5.29727033	5.03028729	5.70493754	5.29295352	5.41053933	5.75002319
DAB2	7.9118642	7.62085894	7.89215397	8.34232553	6.96759762	5.73682261	7.39552812	6.44499098	7.0766679	6.63377463	6.57664291	6.09733648	7.45937406
BCL2L11	5.09826722	4.65264214	3.81384079	4.75618798	3.90769484	4.04150074	3.78807597	3.74398302	4.1577048	3.29210761	4.39201366	3.8980673	4.1300066
ANAPC1	3.68898265	3.85736959	3.25885402	4.03706249	4.03822781	3.81907169	3.74062479	3.46774827	3.86765967	3.4678938	2.59543789	3.35829348	4.43426479
CAST	7.48986817	7.79575592	7.04769908	7.61898365	7.91025872	7.94557446	7.79017851	8.053141	7.97399552	7.68093812	7.31570453	7.56694921	8.22692759
SCOC	-0.34032406	0.22911802	-0.27451034	0.91242599	0.8976134	1.23264	0.93098083	0.88421645	0.40493894	0.40493894	0.40493894	0.40493894	0.40493894
CLGN	0.41392688	0.41392688	0.41652063	0.45378097	0.41392688	0.41536897	0.41392688	0.9754796	3.27023172	-0.23988761	-0.23988761	-0.23988761	5.73600248
CETN3	2.20504297	3.07421587	2.53272923	3.04137187	3.65108298	4.82484765	3.75115395	3.96268293	4.20659339	2.64489215	3.97516107	3.38399145	-1.5558302
SMARCA5	2.52140979	3.41200437	3.00766797	4.10619664	3.89151041	4.6620426	4.05528617	3.83460806	4.34425703	3.94162818	3.89416599	3.56551634	0.10923501
SYCP2L	1.51088667	3.25915228	1.84938526	1.40050633	1.96650658	1.72225503	1.98864129	1.76122757	3.52092953	-0.80896755	3.48748833	2.3132113	3.75376194
BMP6	3.36506212	4.72538298	1.69553793	2.6630275	2.77975482	2.03029311	2.55620255	4.05387052	3.85422274	5.39633029	4.02386108	4.73877118	5.40400081
RASSF3	4.05684782	5.36068404	3.95300623	3.41626228	5.80077455	5.56128242	6.08778714	5.50080843	5.6347606	7.22249965	5.4334887	5.94070396	4.96150422
HNRNPU	7.04176333	7.42598513	7.47115986	7.8969839	7.79281374	7.95223726	7.93327622	7.82260614	7.82312861	7.47018708	7.50491594	7.42680193	7.35464103
RANBP2	7.10872795	7.62562004	7.27987466	7.89848315	7.92046217	8.28817367	7.86970236	7.86704044	7.87073225	7.58548505	7.64741661	7.75978061	7.63383516
AHCTF1	6.22914388	6.95482213	6.48554337	6.82267146	7.11616701	7.58314964	7.3103318	7.39415533	6.66843952	6.59359053	6.44302054	6.74529017	6.52766953
MERTK	5.17741113	4.11057009	4.88813679	5.22718653	5.15935939	4.70959858	4.20736331	4.83184456	4.75059295	5.64661301	4.32672568	5.29813274	3.99428724
TMEM87B	5.32768781	6.08366904	5.71463625	7.0665701	6.51416399	6.22427148	6.51990391	5.97613315	6.19022209	5.12308863	6.61637676	5.71963524	5.36816964
PTPRR	1.30313186	0.88021189	0.78199757	0.87068296	0.73933416	0.79529857	0.95462829	0.60053797	-0.50231918	-0.50231918	-0.50231918	-0.50231918	1.59460667
NR4A2	5.07627491	5.35138573	4.16846627	6.75289929	4.08382165	4.85431135	4.32112521	4.79990507	5.83539441	4.49467458	6.3319139	6.06070915	6.76387016
CCDC148	2.23171803	2.02526453	2.0088412	2.12499242	2.57765763	2.2999576	2.03152038	2.1649981	3.76423541	3.63823999	3.57623102	2.52595846	2.33599513
PLA2R1	6.77672009	6.46512734	6.43086199	6.94739871	6.36608475	5.854816	6.08908137	6.13092207	6.72095481	5.4649876	6.9308585	6.07804033	6.80414322
RBMS1	6.15565004	6.65591476	5.95292788	7.98345565	6.87127344	6.44884817	6.93635718	6.73421118	6.57024598	5.45665133	6.43097331	5.90101626	6.46102231
SCN3A	2.93250385	2.98100946	2.99406393	2.7554817	4.17950713	5.78048116	3.40195344	3.50157612	4.78692111	3.86444675	4.54457172	3.92327186	5.43100621
CD96	2.51474435	3.44285449	1.06717179	2.32998227	2.26871691	2.09702128	1.93884011	1.95774115	0.24112418	2.49280584	3.72974466	2.3409183	3.49977142
SLC25A27	5.3738559	5.57115593	4.47343648	4.53895254	5.57018955	5.74016426	5.78799598	5.5553071	5.18215146	4.68458959	5.4678511	4.85599982	5.13576737
FAM49B	4.54342005	5.62886992	5.16780795	6.38462253	4.80515653	4.13501166	4.82815335	4.46339037	5.41430557	4.58488138	4.75609972	4.39077585	4.91396241
ASAP1	5.32652758	5.5171279	4.79484326	6.19558888	5.74945365	4.89828715	5.30766918	5.39836719	5.57590239	4.32638003	5.76904978	5.22092421	5.76397066
TRAPPC8	4.77330036	5.75731081	4.84877563	5.93199496	5.83010026	5.74666753	5.92028215	5.8213771	5.41445587	4.86523422	5.53827368	5.77809125	5.42299751
INO80C	2.93437231	2.84441245	2.67559303	3.66300447	2.94952525	2.80400693	2.87530929	3.77020235	3.57062791	-1.23096418	3.53485547	2.87331865	3.60660858
LPCAT1	6.19621791	5.68008752	6.21528586	6.17313499	4.95891466	4.39751047	5.84302006	5.05410272	5.07922838	5.11092946	4.5070871	4.98992148	5.39160137
PLEKHG4B	0.30107281	0.38634554	0.29392507	0.3345665	0.36592542	0.78546251	0.35684879	0.43870959	-0.27079576	-0.27079576	2.90570305	-0.27079576	-0.27079576
NMRAL1	5.51944585	5.09019322	5.37195952	4.74890305	4.40884143	4.54978685	4.60279517	4.62567008	4.61463385	4.19142334	4.83472182	4.55564695	3.96659628

C16orf89	1.34226449	2.89618292	1.8131357	1.43766831	2.05197608	2.68941716	1.98615725	1.36213012	2.84380828	3.96889659	5.32662955	3.34033615	3.71403198
ING1	4.64962953	3.68245835	4.21973055	3.64628409	4.37090255	4.89397353	4.22900858	4.4602035	4.58624266	3.57961589	3.58501151	4.55104056	3.75762563
ADPRHL1	2.19346243	0.10973261	2.25712924	-0.58760174	3.4043011	4.02334831	1.6122194	3.59255682	-0.70618934	2.9612725	2.19853456	3.95156015	6.94863628
CMTM7	3.57733803	3.84639979	4.0648662	3.9164574	2.0079332	1.16399825	1.91116128	1.71107982	3.12687407	2.90247156	2.93186749	-0.99928978	3.54010182
FBXL2	5.07370503	5.20716681	4.85469672	4.16278645	5.97210113	5.41803328	5.12687487	5.6172733	5.47801735	5.34395112	5.62122557	5.87292559	5.34658515
UBP1	6.32085269	6.42348485	6.17353801	7.08812463	6.79872437	6.7992234	6.93670011	6.4856293	6.28320534	6.31888885	6.46877907	6.4477216	6.29460019
RMND5A	5.88110216	6.19631082	5.66852912	5.78704411	6.08387867	6.73499146	6.55459627	6.1344749	6.11257594	5.89592253	6.45796792	6.42678914	6.08842937
CD8A	4.12082249	1.90651253	1.49801767	2.0487567	2.57697731	1.78566518	2.54992606	1.85136718	3.24396025	2.98501424	3.62014755	3.49541351	-0.9486127
RPIA	3.71867328	3.39327685	4.59971005	3.73962177	3.95966179	4.20135731	4.18903623	3.97067849	3.5883864	2.73489669	4.15526227	3.33332666	4.22122934
PTPRD	6.76906493	5.8935438	5.34971946	5.49435205	5.58963661	6.8916329	6.6713879	7.3578588	6.84621193	6.75824176	6.1063993	6.67840007	6.84547182
CNKSR3	5.03932292	5.41121592	5.22714499	4.32722328	5.5376145	5.62868447	5.17287839	5.46405509	5.73288024	5.44605068	5.37945752	4.91995622	5.31766669
GTF2E1	4.7320657	4.39531314	4.93693299	4.99689573	4.82761616	5.47530899	4.71217583	4.43103539	5.11738842	4.70122781	5.22090188	4.40187915	4.97567003
CFDP1	4.32282107	3.63911306	4.45945506	5.22191181	4.70762492	4.81564469	5.24980291	5.03857501	5.19453864	5.4763834	4.85392537	4.21491158	4.05624113
ZDHHC7	5.72737425	5.51884923	5.51188718	5.56536725	5.35179355	4.88309417	5.73494195	5.72075973	5.42049977	5.87421249	5.21182604	5.54716166	5.53580709
C7orf31	4.09261525	3.30203977	3.26156861	3.03167741	4.2215779	4.30824899	4.10533938	3.80090696	4.04482878	4.48164706	3.88007039	3.9726001	4.06918001
JAZF1	4.75304112	5.15153109	4.83424926	5.08385648	6.01475852	5.85847523	5.69744283	5.65331245	5.60524894	4.67979521	5.89822175	5.93808142	5.40447931
CMIP	3.2980477	2.83270455	3.58720639	4.26376132	2.43644381	0.6686107	2.68732694	1.76870155	0.16274247	3.6072486	-0.51209427	2.50149022	3.29226751
SPHKAP	2.70181011	2.55753979	2.73901469	2.48905937	3.41429871	3.37370826	2.7713666	2.59922716	4.16665127	1.65653025	5.31617707	3.30295337	7.65645207
PID1	6.99281595	6.62825422	6.81323219	5.90789911	7.0016135	7.59975935	6.96400755	7.44005813	7.18958006	7.66235787	6.76567928	7.39494805	7.16294269
TRIP12	7.25051803	7.60228124	7.2398991	7.85664077	7.60494436	7.75031211	7.67447321	7.58502321	7.51373645	7.5748056	7.40493523	7.43995923	7.67901383
FBXO36	3.56162825	3.23805906	2.96610017	2.61755553	2.92721981	3.91123002	2.42806409	3.39231479	3.05426741	3.66068651	2.44163677	2.13527183	3.30461722
CEBPG	4.88840418	5.53414143	4.64128382	5.17884129	5.92716332	5.8684448	5.57048263	5.78972784	5.56121589	4.85408742	5.5567444	5.81206904	5.98017306
KCTD15	5.37122612	5.28156139	5.27755372	4.63278489	5.07390431	4.48846509	5.60851486	4.78421536	4.58488155	4.68688971	5.37630203	4.83014334	5.16775691
ZNF599	3.25528166	2.04921274	3.12290393	2.45138809	3.49104863	3.5135775	2.92099734	3.48270912	2.97351641	3.35873813	3.59433433	3.53058815	3.74533767
MCOLN2	2.8550087	1.49145448	3.4238923	2.08730002	1.15749284	1.91891635	1.34630648	1.85218919	3.31471754	-1.54424246	2.02366211	2.44456603	3.2801645
LGI4	2.69337717	2.74463671	1.69371299	1.61757719	2.98634367	3.68853229	1.9479191	3.5494841	2.98086386	4.62260836	4.202952	3.99235638	3.78276112
DDAH1	5.40075527	6.10733636	5.81924055	6.64674863	7.30765157	7.07626381	6.3770287	6.7039126	7.05944333	6.50465525	7.07421704	7.10398253	6.60202304
SREK1	5.31636862	5.7852399	5.29666532	6.51934587	5.97019781	5.97192256	6.09724195	6.36005981	5.9272317	4.91765948	5.75910548	5.82016356	5.82632218
CHD1	5.25504037	6.09016951	5.59249741	6.91500585	6.34567196	6.75404257	6.30504574	6.45114656	6.38194945	5.41187745	5.69776691	5.94633264	5.57710831
ANKFN1	0.46219422	0.25396262	0.13679259	0.16389576	0.94959641	0.13566846	0.17252311	0.49047421	-0.15499898	-0.15499898	2.62494115	-0.15499898	2.70935234
DGKE	4.67578805	4.73050254	4.06482278	4.39526987	5.39342071	5.39517011	5.25264702	5.69136309	5.19801625	4.82089251	4.82960515	4.86655117	4.59216207
HS2ST1	0.64361794	1.77852953	2.17980273	4.02624884	2.29941283	2.73428236	3.488574	2.7923198	-0.78621317	3.65617692	2.73226441	2.25328247	1.40144355
MSI2	4.44089999	4.1599495	3.79214761	3.62133453	4.18949356	4.71165156	5.06803613	4.84359425	4.88271583	4.22025447	5.03916135	4.25018632	4.33578573
CACNA2D1	4.92737227	5.48983354	5.35325256	6.76158508	6.21868967	6.51322717	6.3552641	6.45168716	6.41148917	5.47219978	6.37521916	6.75242279	5.1565714
ZUFSP	3.55750703	3.97167958	3.76695872	4.68885758	4.6112912	4.75697145	4.36915643	4.62379762	4.26188909	4.0807599	3.45817062	4.0834887	4.18523982
HS3ST3A1	0.19551469	0.47006279	0.25035014	1.25988222	0.04497004	-0.13489165	0.08523745	-0.13330967	-0.01372272	-0.01372272	2.04163775	-0.01372272	-0.01372272
GDPD1	2.80407876	2.63429311	2.02007454	3.29016064	2.90961755	3.26489766	3.35820145	3.53143186	3.11085094	2.80259993	3.86192136	3.03627823	3.12648452
NUS1	5.46605547	5.44490532	5.14655787	6.2070033	6.08732993	6.28889052	6.1197121	6.12206899	5.97257422	5.38528751	6.16602481	5.99303726	5.20408528
SEMA3D	4.40416812	3.88935286	3.78079427	4.50017203	5.27015522	6.87953882	5.97401002	7.24542618	6.24908175	7.05098069	4.14065397	6.65804045	6.08424377
PPP2R5E	5.11993806	5.75389764	5.20311962	5.97196201	6.28005838	6.00653034	6.10662205	5.99210824	6.0474048	4.64311938	5.77191997	5.62081587	5.51186793
GRAP	1.2529167	2.85786787	2.794322	3.44876356	2.75615195	1.3550246	1.2627074	1.04421626	2.94190361	2.43241314	3.73920874	2.68189617	-0.83902003
AK5	2.0267675	2.2792175	2.13905287	1.4819886	3.46337063	2.61145817	2.21914513	1.89390868	3.30793279	3.32109094	1.87594954	4.29159573	3.84580948
CABYR	2.70610368	1.69567397	2.3927537	4.14849452	3.66803128	3.18595667	3.27573803	1.57684134	3.94042992	1.37788801	3.15216439	2.66747487	2.35847733
IMPACT	4.64194361	5.4807196	4.76681939	5.82051012	5.3682968	5.77485278	5.03587716	5.56271682	5.80901594	4.68103074	6.01876874	5.54680855	5.30515083
ANKRD29	1.91839203	4.20229536	1.0196038	1.82361882	3.81848361	2.40920136	2.41026139	3.83021197	3.4445597	3.61994204	3.4946139	4.04281267	4.94558884
CHST9	0	0	0.00124472	0	0	0.00069205	0.02859145	0.07737624	0	0	0	0	0
THY1	4.45226777	5.10063584	5.25417662	7.54664518	4.41881755	3.1140963	4.03739998	3.11156079	5.91512283	4.48710276	6.29978617	5.1715465	5.50269647

DNAAF1	0.19927486	0.33084397	0.18729186	0.20671717	0.23038293	0.18613534	0.18468716	0.27926176	1.03720006	-0.14205465	-0.14205465	-0.14205465	4.14592789
C16orf74	0.06615102	0.51211518	0.24757531	0.63356617	0.39852542	-0.6050193	0.72544711	0.37124846	-0.12860934	-0.12860934	2.33560827	2.33560827	-0.12860934
TBCEL	4.86195943	5.91944669	4.89150756	5.71786794	5.55488838	5.74382896	5.70380333	5.81122024	5.59869234	5.34915398	5.51590819	5.67965475	5.77467582
JPH3	0.07692923	0.1322193	0.05064979	0.00395301	0.10828765	0.05052003	0.05802985	0.04755303	0	0	0	0	0
ANKH	7.5145123	6.79538515	7.26579011	6.40010925	7.11866019	6.8096806	7.31323399	7.47050745	7.07743873	7.58921502	7.13597471	7.08085936	7.42246177
UBASH3B	5.05587173	2.78547221	5.09485031	5.28691303	3.96069109	2.97748764	3.68790019	2.48519734	2.07526971	2.34357821	2.75779214	2.00378278	3.00100135
ROBO4	0.91152458	5.21052831	3.0807964	4.4520967	4.08046489	1.74359682	2.30002407	3.49052521	4.27148516	4.84931405	5.43431581	3.73090877	5.52253481
ROBO3	2.58334903	4.03004475	1.40810081	2.07613534	3.24250169	1.98783773	2.57400105	3.03792485	3.23294496	2.07751004	3.51569657	3.23571381	-0.92914796
TBRG1	5.81887139	6.28844054	5.41834431	5.76759361	6.28583175	6.05822683	6.01835485	6.29085887	5.84783249	5.26210992	5.84268889	5.98094565	6.01486169
NRGN	1.51404404	0.85300473	2.08609009	0.84858749	2.91013663	0.57924781	0.13226507	0.10720674	-0.02695686	2.87280794	-0.02695686	1.47280429	-0.02695686
GPR15	1.16221516	0.19556672	0.19807649	0.24307305	0.61434601	0.19696212	0.19556672	0.1987686	-0.08607231	-0.08607231	-0.08607231	-0.08607231	-0.08607231
ABI3BP	5.12718326	6.30909684	5.08018837	6.87339155	6.39355893	6.28397902	6.74419432	7.30261703	6.6070644	7.10201225	6.49973292	7.12342454	7.66344569
ANGPT1	6.2048502	6.8317361	5.17130972	5.91262571	6.01954441	6.70019761	6.50374395	6.43327435	7.31804835	6.8048837	6.5126966	6.86722075	6.55562579
PITPNC1	3.68683065	3.20519125	3.88557754	3.31070357	3.00008427	2.80537912	3.14525917	3.04651898	2.88589838	1.85808117	3.94682266	3.27153471	3.14871037
CC2D1B	5.47177891	5.30993269	5.45992355	4.60726976	5.28341138	4.9028071	5.24864982	5.67521819	4.61447895	5.61136841	5.62589513	5.13552229	5.2055871
CERS3	0.47729832	1.21649862	0.44051596	0.60584581	0.43794052	0.43937243	0.76187537	0.79507437	-0.30721349	-0.30721349	2.69147241	-0.30721349	-0.30721349
PRKCA	2.84868912	3.79401716	3.08200168	3.40687865	3.1823487	2.44185308	3.01450519	1.35943975	2.27361358	2.46294005	-0.87920063	2.47711784	2.77644505
LRRK1	5.60524939	4.87637108	5.18691472	5.0244217	4.74561684	4.46848748	4.78336229	4.68486757	4.64297248	5.38287699	4.95874049	4.32347387	4.13242242
CEP112	2.62801627	2.55063738	2.21101903	2.79064758	3.2000689	2.63100559	3.10732197	2.6915991	4.15885905	-0.541376	1.56398756	3.48838947	3.12782357
GAL3ST2	0.0705345	0.28334532	1.19059653	0.0705345	0.32263995	0.29324481	0.2993999	0.07367952	-0.05386287	-0.05386287	2.75064941	-0.05386287	-0.05386287
ABCA9	4.27331743	4.85846	2.72235444	4.01057758	5.87155353	3.60604792	5.81816771	6.1120863	5.13940777	2.5233994	5.41725978	5.05989686	5.88129735
ABCA6	3.60159726	5.07648776	1.80606037	3.53665974	5.38605513	3.21616642	5.11949217	5.15866011	4.74076584	3.69553441	5.33348788	3.95801216	5.26768707
ABCA10	2.120577	4.78182858	1.52114482	3.16148541	5.06307164	3.46622295	4.19847994	4.73078669	5.06988684	4.36313642	5.48371621	4.64347604	4.87215056
ABCA5	3.76283063	4.39255396	3.59988662	4.26148248	4.79730687	5.20046466	5.27916748	5.7414078	5.12949994	4.37358143	3.93916262	4.61776732	4.45112713
ENPP3	1.30714435	1.4832794	0.98098323	1.40739034	1.50117427	2.66823663	1.52124457	2.1457658	3.00178222	2.64068729	-0.69808801	1.29883751	3.14794012
UCHL1	6.01167509	5.90381517	6.14344773	7.0741581	4.59060593	5.90056658	5.43163152	4.49031317	6.77292185	6.35376497	6.478726	5.81777441	6.05926229
MIA3	6.73142389	6.29245727	6.47180417	6.48890194	6.46204458	7.46211651	6.66420674	6.53138657	6.59949568	5.91965963	5.96411256	6.28801912	6.77882916
DISP1	3.2719108	3.95561525	2.51681463	2.18207786	2.76719714	3.08895534	2.61641378	2.85411824	3.28276537	4.32869154	3.05390437	3.53310945	3.64200092
TNIK	4.36631315	4.34680957	4.17586828	4.65241339	3.7116659	5.25713548	3.97368964	4.05231606	4.08097914	4.21525847	3.98901018	4.46378275	5.2684869
FAM167A	1.35280751	3.25855762	1.44863185	1.55657933	1.05574661	1.05711818	1.09177122	1.25018935	2.76124031	2.69981388	-0.64230154	2.54877567	2.25665452
NEIL2	4.75553833	4.58360558	5.09215263	3.94682591	3.60282811	3.49104281	3.84998512	3.53226663	4.24752006	3.96372753	4.30893913	4.41791974	4.4355135
PGM5	6.7966874	6.9802358	6.44982535	5.15619671	7.37100792	7.49884242	7.81339164	7.63921893	6.86580454	8.03936313	7.49372474	7.70612015	7.54497021
OBSCN	5.45061246	4.97867698	4.50533551	3.64140701	4.17239451	3.66288683	4.18723427	3.96041184	4.34818259	3.78105707	4.85135663	4.17982242	7.0823981
LONRF1	4.48991088	5.12964829	4.16753494	4.48590315	5.08831223	5.34380115	5.23668955	5.30248022	4.69698559	4.41063154	4.61886602	5.45739921	4.95674413
TRIM11	3.9055728	3.97773013	4.79043603	3.45591577	3.36937684	2.97556841	3.73263456	3.85041087	2.90556417	4.03858181	3.62075242	4.14781988	2.88321713
ENAH	7.4392402	7.20833681	7.31878947	7.6314602	8.81861256	7.67762211	7.72721629	8.14127857	7.36414205	7.26383442	7.36414205	7.75282019	7.4831434
SH3RF1	6.21234714	5.39593131	5.85256915	5.75121468	6.04982504	6.38417613	5.69268293	6.02941502	5.43411777	5.88463627	5.59952078	6.18609862	5.90505444
GBP5	4.42966714	4.41984456	2.56918704	4.82394186	3.48858338	2.22049746	2.58553039	2.19988481	4.93866678	-1.71096082	3.38937605	3.29031879	4.6947845
BUB3	5.28112151	5.14248416	5.69379752	5.99156977	5.31563056	5.43530864	5.72859678	5.22507006	5.37118734	5.42904131	5.46076155	5.51336337	4.96128146
MMP21	0.27350354	0.06718998	0.06972811	0.52053112	0.80491569	0.24916468	0.23810695	0.07042804	-0.09377165	-0.09377165	-0.09377165	2.17778333	-0.09377165
FAM69A	-0.46140792	1.41483174	-0.05091389	1.45146569	1.55322601	2.74621796	1.62374988	1.46797688	0.32122497	0.32122497	0.32122497	0.32122497	1.49259644
ATP5G3	4.762676	5.3990243	4.77272885	5.22287413	4.8782408	5.33291616	4.87288889	4.77150209	5.09336852	5.10236769	5.28370889	5.42969796	4.93920651
CNTNAP3B	3.35048362	1.91072026	3.1544676	2.00866181	3.75814569	3.32500139	3.45173671	4.72741375	4.22866182	4.47598836	4.63945589	4.22496976	4.49558346
SRSF12	2.28262465	2.24497441	2.20848506	2.38682697	2.46772087	2.74388412	2.13134609	2.39473063	2.86956854	-1.61014986	-1.61014986	2.44236716	0.18225608
PDLIM3	7.92715674	7.55342252	7.31454352	6.24959481	8.35599184	7.81106258	7.46420441	7.95822508	7.33513376	8.2139041	7.75955411	8.04260726	7.30048556
SORBS2	6.78480983	8.04053993	6.39697797	6.43233886	8.13582052	8.24225054	8.18132004	6.27761114	7.75538737	8.15963997	7.76420874	7.65825249	8.45372819
LY96	4.01076291	2.19248838	2.59700181	4.32972371	3.50358745	3.65136632	4.11616014	2.18172864	3.91295003	2.51781629	1.78489846	2.89159051	3.05182417

TMSB4Y	1.49557975	1.13977902	0.38187609	0.82156946	1.85896182	1.43497927	1.36251734	2.67893336	-0.53809323	-0.53809323	-0.53809323	-0.53809323	2.82560323
CXADR	0.00469964	1.56102439	0.00124472	0.62047336	0.07953951	0.00069205	0.01481742	0.04134562	0	0	0	0	0
BTG3	4.57368245	4.35790938	4.25925465	5.0865954	4.96333201	5.80094273	4.99720708	5.36394291	4.86322161	4.64132565	4.37707937	5.32363496	4.09047452
C21orf91	4.2100863	4.42699157	3.71741278	5.06813778	4.77250372	5.16299771	4.96954969	4.75025477	4.62248266	3.9985715	5.15282681	4.63843963	4.1865194
CHODL	1.79572475	2.63807359	1.5347659	1.86048417	2.27468456	3.6424644	2.92622868	3.29365409	3.54559172	3.27784005	4.47841071	3.65272634	3.75445874
NCAM2	3.77028744	3.38492973	3.9101114	4.07894623	3.13918151	3.47024624	3.49942213	3.32066855	3.25781137	3.78260498	3.36639667	3.35163298	4.14312898
L3MBTL4	0.89723891	2.7156622	0.64542513	0.62864919	1.70950207	1.25932536	1.37042372	1.42638162	2.44192774	-0.44270195	3.42160555	-0.44270195	2.05816939
PDE1C	2.23455197	2.69456321	2.31824783	2.78488083	3.63972626	6.04195038	2.82902634	2.442891	3.05814479	2.7375526	3.85939335	3.40274014	6.53474271
RABGEF1	4.25010735	3.79757889	4.57201438	4.59419074	4.44543456	4.23381121	4.55267816	5.09257528	3.84295564	4.6595257	4.90789087	4.76540672	4.2695291
MRPL39	4.32826534	4.05519663	4.2451708	5.23014248	5.29996972	5.38161512	4.9967564	5.25436075	4.46598605	4.80211869	4.51436275	4.29266981	5.60084134
JAM2	2.92618858	0.73487791	1.65824272	1.44398566	3.54120558	1.77500802	2.00295428	2.32478733	3.56083095	5.17818912	4.22986246	3.74589593	-0.22160517
ATP5J	5.64066308	5.36141243	5.33464077	5.76317505	5.76669392	6.809427	5.64421635	5.57875664	6.0502279	6.32219406	5.28889865	5.31109826	6.37366666
GABPA	5.08102658	5.70425667	5.08814363	5.91393032	5.85841202	6.28947974	5.96342323	5.9787388	6.18048993	5.65426248	6.04077538	6.15411121	5.66985841
ADAMTS1	7.18798425	7.17243585	7.56584274	7.18016044	9.15634252	8.06026907	8.35386799	7.28992883	7.57027148	6.67318921	7.60327412	7.231789	7.93684358
ADAMTS5	1.9849184	4.62291561	3.60002745	6.10144867	5.34310007	2.64078745	2.14167323	2.95636816	3.94261353	1.15438873	4.54793172	3.94409477	4.49980298
TSEN2	4.54507013	4.3187767	4.14998491	4.18332609	4.31222999	4.39538382	4.71943179	4.26792599	3.97784996	4.68440389	3.89654826	3.7996899	4.84272494
SLFN13	3.98221084	3.19477442	4.31930892	3.98681314	4.71572288	5.20766156	4.78684734	6.27822668	4.54314914	5.72638129	4.55321473	5.3343525	4.97587221
XPC	6.46140569	6.12525036	6.28103928	5.48466825	5.57710114	5.98179501	5.72917276	5.96840141	6.17275455	6.16748916	6.32274245	6.04495247	5.65209542
FGD5	4.89390301	6.36233863	4.79881787	5.31615447	5.66943969	3.5297916	5.09882988	4.48871356	5.44297889	5.01976139	6.42482379	4.31428059	6.14454879
FLCN	5.16180922	5.35016206	5.52122699	4.58933877	4.9194645	3.89408896	5.9202216	4.73879335	5.38079223	5.5242872	5.43794382	4.88640326	4.81534838
DPH3	4.99634995	5.82933501	4.8288302	5.71315224	5.7799118	6.08611258	6.26630178	5.61209714	5.96767077	5.39804709	5.46934113	5.74950451	5.63946947
OXNAD1	2.71773636	3.14072956	2.41893807	2.89032078	2.60352077	2.92815263	3.60406124	2.62701769	2.28366066	2.47727577	3.59154852	2.98466608	3.12968353
PLCL2	1.89145436	3.21588636	1.06517822	3.26093843	2.5531982	3.48339709	2.64362591	2.64275874	2.25620827	-0.66660679	2.46783952	-0.66660679	3.19658088
CXXC1	5.7203554	4.92501693	5.84818248	4.73235718	5.0190009	4.77364971	5.18653939	5.03526391	4.95934529	5.24109869	5.025172	5.22434336	4.90705939
SKA1	0.73743692	0.62570154	3.30692936	3.07968171	0.54965727	0.44496377	1.86657732	0.55496869	0.49756413	-0.68768621	4.20535462	1.98162725	-0.68768621
PPP4R1	6.01276867	6.65750368	6.25788641	6.79205965	6.61100045	6.37631699	6.58174265	7.03666131	6.38676969	7.08610497	6.1402341	6.99272855	7.01608781
APCDD1	5.55039212	3.82130263	5.00009611	3.2136548	3.82697891	5.84026271	4.15079829	6.8431923	4.8499878	6.14568991	5.12316256	6.65614648	3.87918443
PIEZO2	1.99338744	3.94146311	2.56018212	4.15425134	2.76067823	2.68642412	2.21605523	2.71323348	3.59261234	2.74933564	2.65480536	3.65448559	2.83310548
MPPE1	5.12697609	5.56565263	5.08752174	4.39673315	4.64353798	4.85674488	5.27758691	5.19924236	4.8460701	5.54532694	5.33583565	5.23565598	5.27485577
USP43	0.31301995	0.41978126	0.40639968	0.25094472	0.24344634	0.24489246	0.24344634	0.44637953	0.00534893	0.00534893	0.00534893	0.00534893	0.00534893
RAB6B	3.74833532	3.62733841	3.59873702	3.17567125	3.96269093	5.02075477	4.1870654	4.72213534	3.73290399	4.40146099	3.92810906	4.28397878	4.75415036
EME1	0.93303044	0.4788607	2.64169793	2.6335657	0.55980027	0.4560301	0.5722859	0.48923729	2.23001309	-0.58904724	-0.58904724	-0.58904724	2.75655292
EPHB1	2.25248859	1.3367203	3.19952809	3.05740401	1.15327347	0.75054575	1.37262458	1.64437265	-0.27968959	-0.73758657	-0.73758657	2.49122506	1.3437488
ACSS1	6.1081646	5.63658295	5.93836083	4.6306287	5.00764744	5.10998604	5.35862891	5.4514164	4.63455815	5.28208474	5.37402875	5.3576144	5.75348015
ANKRD40	6.80106406	6.91551821	6.92381695	6.52996056	7.36179723	6.98980924	7.23284704	6.8238927	6.77096541	6.725648	6.9431987	6.77887246	6.82177251
ZNF18	4.03397214	4.895857	4.62403336	4.22801389	4.48286534	4.53262829	3.85386419	4.2738962	4.5355065	5.0606731	4.67798245	4.03359553	4.99112379
VOPP1	6.25485645	6.21318903	5.86621755	5.86028168	5.59843014	5.44580487	6.01013499	5.44580487	6.08617073	6.06571745	6.33487297	6.04671477	6.08593998
APOOL	5.22120545	5.1607511	5.02849578	5.23617054	6.35004984	6.20028272	6.48365627	6.19365969	6.25059665	5.80669475	5.87064095	6.05670693	6.10910334
DKK2	2.20673203	1.73078983	2.68450137	2.17891361	2.49228716	3.84520516	3.21725582	2.23807735	4.80366653	-1.16005491	3.16075837	3.85154949	3.32484061
CYP2U1	5.46725499	5.76365759	4.86334319	4.77457301	6.06327508	6.27134081	6.05318896	5.70798734	5.44876373	5.55955466	6.39804305	6.01374253	5.70593151
FBXL18	4.29806677	3.61747157	4.92763684	3.63408397	3.3401737	3.09639106	4.02497565	3.51520268	3.26598445	3.06719023	3.74464227	3.64999826	2.80166617
CNTNAP5	0	0	0.00124472	0	0	0.06813393	0.0430781	0.13305519	0	0	0	0	0
PROM2	2.33503226	0.84961972	2.18177252	1.49144917	1.40923677	1.59967681	1.88013245	1.91645919	-0.55963414	3.69358476	3.14082274	-0.55963414	2.33595204
KLF10	6.87709764	7.42004257	7.55500415	7.74912825	5.62570125	7.30639955	5.50637282	5.87010889	6.34292182	6.39001883	6.63317666	7.06526877	6.36910947
PTPRN2	2.51301027	2.37535244	1.69760969	2.0462785	2.49624289	1.97947133	1.90482601	2.03707708	1.1097499	3.26977306	3.73687808	2.78121053	2.96796085
AZIN1	5.85823418	7.25934326	6.43528842	7.2983277	7.15829954	7.08409458	7.50770393	7.28749721	7.15675704	6.23313434	7.27930899	6.97804466	6.51583212
ATP6V1C1	5.54990332	6.20156536	5.43038962	6.14253705	6.29473959	6.3830445	6.19536749	6.12034419	6.17694026	5.40733861	6.00880562	6.04675743	6.09051962

OTUD6B	3.82504521	4.02604	3.94409067	4.83417213	4.81234223	5.09400908	4.64168973	5.05080571	4.73970009	3.60309218	5.45236264	4.45589009	5.14685062
CDK19	5.19516189	4.96185672	4.32952019	5.27234047	5.3683195	5.74249599	5.7724296	5.81739446	4.99885422	4.67646413	5.2023423	4.89689582	4.86875868
GTF3C6	3.80761527	4.19175498	4.02490486	5.39908245	4.36496132	5.36058178	4.65685647	4.54171245	4.79559848	3.94117778	3.90818863	4.59854936	4.07284607
TTC39B	5.55449915	5.38810696	5.51370097	5.32138141	5.76395137	6.19693686	6.27153499	6.3082727	5.64152328	5.31986808	6.11167946	5.8284151	5.76244119
AGPAT5	1.00704231	1.93941228	1.74992991	3.36985156	1.70878509	2.32969219	1.85049854	1.12787981	0.27771046	2.58320587	2.89931396	2.0939843	-0.45920672
MMS19	5.33806968	5.62937608	5.36952896	5.71695916	5.34656423	5.00093753	5.73458323	5.82616916	5.43031112	6.00979027	5.5711731	5.20690362	5.9209559
PI4K2A	5.75637485	5.0069405	5.84864098	5.33578742	5.06671994	4.78318834	4.81583943	4.76621634	4.75506215	5.43795941	5.12539576	4.98504225	5.21894727
MARVELD1	6.02005395	6.85318052	6.36246249	6.37450758	6.91531073	5.91850442	6.59719862	6.96214224	6.34672134	6.92374714	6.82215091	7.08796442	6.53825604
ZFYVE27	5.12095401	4.95988183	5.21403831	4.69515884	4.35640493	3.94006353	4.45291809	4.6233026	3.5868488	4.78832625	5.11174455	4.88136828	3.69318754
GOLGA7B	1.26403502	1.51225676	1.37368588	0.80973574	1.04566672	1.06965751	0.91267592	0.84942326	-0.60004216	-0.60004216	3.05858191	-0.60004216	2.81837121
GPR78	0.00655074	0	0.00124472	0.17057149	0.10521826	0.00069205	0.02465071	0.14022135	0	0	0	0	0
SLC25A28	6.34911619	5.57345827	5.78525902	5.20868388	5.56543036	4.83870127	5.46897903	5.32618204	5.40722304	5.3695578	5.62343735	5.43973337	5.2227998
HSPA13	5.03327406	5.72927131	5.39267113	6.8648261	6.10159556	6.23633737	6.32080271	6.10257497	6.1577181	5.76094958	6.00358287	5.99652847	5.57173221
SAMSN1	4.24357563	4.47589101	3.86024248	6.05092507	3.67566929	2.66381864	3.56865005	2.90707093	4.25165522	2.58760997	3.32327526	2.74384253	3.11022089
USP25	-0.01272345	1.66839843	0.19833215	1.93965362	2.4875865	2.97735227	1.82692661	2.40311649	3.64800752	3.84663413	0.20290101	3.17884626	0.20290101
ZCCHC10	3.84463741	4.17004233	4.23518152	4.757629	5.24970333	5.75781979	5.42597546	5.37764841	5.6231556	3.90130273	5.35260672	4.80386115	3.39689219
C16orf87	3.41042283	3.5604709	3.76778304	3.69885162	4.33815466	4.53792453	3.9695141	4.33417608	4.14658181	3.7230726	3.71872886	3.73314381	2.32474644
MOV10	5.30262782	4.49576356	4.82940094	5.31158217	4.80657312	3.97041213	4.57103134	4.69594046	4.19995766	4.25951671	4.64284949	5.04429627	5.15206694
RHOC	6.67051162	6.46494492	7.25287525	6.53320302	6.58673005	6.57957188	5.68252363	6.41028067	6.32869052	6.8363303	6.74295304	6.67553342	6.14198682
PPM1J	3.3720566	2.83120208	2.47341067	1.88619257	2.20673462	2.02910559	1.86822428	3.34820543	2.67697541	2.43583207	3.1214049	3.42705171	1.3353711
DBI	5.27708986	4.42608244	4.58768786	4.58661183	3.81860317	5.65170013	4.35575889	4.08041309	5.81552625	5.32960959	4.28752688	4.20705417	5.18450155
SLC16A1	5.6400474	5.48019069	5.05412828	6.54154185	5.54254736	6.33084596	5.31198722	5.7114307	5.33051052	5.94112927	5.16094474	6.14037559	5.83914318
HEATR3	3.29106512	4.19504109	4.39509623	5.16453831	3.45002692	3.32609824	4.47407653	3.89984467	4.18934166	3.99130971	4.1212338	3.97580761	4.39760826
OXA1L	6.49243778	6.18333176	5.65540711	6.39296184	5.89140422	5.66715776	5.77712537	5.85995961	5.7180146	6.595427	5.98105773	6.3205655	6.30251945
SLC7A7	5.32909376	4.45315376	4.97541112	4.81753574	3.58123429	3.35178448	3.07683743	4.23139354	3.12913679	3.97270748	3.98636794	3.60720117	4.073947
MAGEC1	0.01695509	0	0.04823251	0	0	0.00069205	0.11244989	0.13772317	0	0	0	0	0
LARP1	1.7343196	1.50931155	2.17590325	1.20969851	1.62799471	0.76789032	1.75474344	1.41151107	0.96340766	0.96340766	0.96340766	0.96340766	0.96340766
CNOT8	5.27514476	5.60399477	5.3389704	6.03761187	5.50800677	6.16943341	6.23361407	6.51222988	5.61482545	5.35814899	5.48262368	5.78916322	5.46137383
GRIA1	5.05998625	6.77377246	4.96826991	3.68142423	6.90477569	6.81598475	6.06925226	6.30372241	6.63371963	6.12048072	6.84040779	6.11447443	6.57566966
LRGUK	1.44139648	2.24518854	1.35849774	2.18924283	1.97144778	1.58585169	1.67964044	1.51611021	2.29370878	2.9211783	-0.77509709	3.17413162	2.25845929
MIER3	5.21052238	5.50077805	4.98009092	5.64085519	5.67895124	6.48020144	6.04282746	5.89478278	6.00824673	5.28690388	5.97193816	5.3579274	4.65972733
NUP205	5.55644703	5.80541739	5.53738935	6.33787985	5.89687723	6.10247354	5.799721	5.62433245	5.67264482	5.87249775	5.90342869	5.88537824	6.10850636
ZKSCAN2	4.96549084	5.09874946	4.71607376	4.25797032	4.95730175	5.43121765	4.77039152	4.93321622	4.79599085	5.11075246	4.94597611	5.30228941	5.04289797
C9orf85	3.95294538	4.05391776	4.11830279	4.07274691	4.83360898	4.40899192	4.82026649	4.28459081	4.70574407	4.29042459	2.82851285	3.69326179	4.96212996
PIK3AP1	5.24776521	5.38993186	5.03010212	5.86935379	3.95903045	3.09058691	4.49719528	3.29293342	4.72037147	2.36730083	3.96246362	2.44037821	4.43313491
RBM45	3.18301272	3.63084458	3.16076237	3.93996522	3.90667886	4.37096402	4.41831927	4.00073111	3.99940805	2.80817092	4.03062694	4.15780837	3.88581297
TTN	4.34071372	5.26922973	4.59794447	4.72276846	5.33491975	4.69739488	4.42370566	4.11287457	5.323363	4.60104178	6.06279412	4.75116968	11.3338975
VSIG4	6.47160274	5.96097944	6.36619059	6.78532226	4.62770767	5.00748096	5.36407588	4.54281259	5.63699425	5.45047707	5.21685126	4.9537343	5.14422627
PDIA4	6.67255409	6.09019039	7.12164309	7.789075	5.29111003	5.57396969	6.03552926	4.5549429	5.48926301	5.37957922	6.03889506	5.26016255	5.9484281
OTOA	1.06233617	0.15860526	0.33957666	0.25662317	0.10468847	0.1060424	0.27434518	0.10779521	1.81307809	-0.141252	-0.141252	1.62943447	-0.141252
KCTD18	5.41208098	5.44283046	5.60412158	4.70619764	5.38108991	5.29438417	5.16786844	5.1243303	5.45068698	5.6685149	5.18565312	5.19658855	4.89067364
FAM126B	4.91544831	5.14032798	4.71654689	5.73475553	5.74849478	5.72726467	5.69297234	5.67514679	5.57726328	4.40749984	5.95439646	5.65529245	4.98859568
ALS2CR12	4.52634162	3.53352579	4.36123038	3.62472029	4.13020323	5.3682673	4.76266567	4.38916025	5.06047818	5.21733594	4.06634763	3.94038189	4.18979844
TMEM237	3.8613237	4.18453757	3.35623842	3.84855908	4.83311237	5.00594289	4.65545261	5.5717805	4.94352146	4.94321819	4.97460917	4.89564313	4.09279187
FZD7	4.95019636	4.74267589	4.86150224	3.53794759	5.50548191	5.14072013	4.81783117	5.38213018	4.95175762	5.27332977	5.49711824	5.77814475	4.30356217
SPAG17	0.11345799	0.44062144	0.11603749	0.57056345	0.12889672	0.11489216	0.11345799	0.1975155	-0.14504596	-0.14504596	2.39787646	-0.14504596	-0.14504596
DEPTOR	1.94271449	3.72591023	1.35555492	2.27680178	1.73484684	1.62172496	1.41117346	1.63112578	2.62002482	3.55484128	-0.57343071	2.35532301	3.20222145

FMN2	-0.08319601	0.02878889	0.06120977	-0.04701157	0.03291473	0.11004648	0.05884848	0.28285797	-0.0169604	-0.0169604	1.2194237	-0.0169604	-0.0169604
RNF20	6.64928157	6.68048436	6.51170987	6.18053358	6.31689162	7.28114831	6.46224649	6.29652977	6.73758175	6.69253876	6.03382576	6.36170601	6.89140338
PPARGC1B	0.01503706	0.77679289	0.81377193	0.65411317	0.01643637	-0.47872988	0.00990217	-0.21778013	0.06659224	0.06659224	0.06659224	0.06659224	0.06659224
ELMO1	4.00537604	4.48083139	2.86553603	4.65967251	3.83130084	2.80907471	2.99743952	4.12225945	3.87230106	3.78844937	4.45291266	3.06014464	4.51356542
SLC26A2	5.05426511	6.07610837	5.01623933	6.16845637	6.5845602	5.72574042	6.26770191	6.33564296	6.61745803	5.76045793	7.11410886	6.48906466	5.79968432
LSM11	3.47030902	4.22835918	3.18259457	3.5771119	4.44333669	3.47308832	4.22442485	4.24457552	3.40639922	4.65371675	3.78280765	3.58953337	3.08980224
MED7	3.9363709	4.03721907	3.63634233	4.11689316	4.71918359	5.01633763	4.73406408	4.42183514	5.22212823	4.77086906	4.08984613	4.35040673	4.26923687
RRAGA	6.03852677	6.2911597	5.49123715	6.11904758	6.04621347	5.7376106	5.92681006	5.90728986	5.36277477	5.99394887	5.45945375	6.10366798	6.25335847
ADCY8	0.18247212	0	0.00124472	0	0	0.00069205	0	0.00158797	0	0	0	0	0
RASA2	4.32481245	5.215507	4.33484526	5.40051464	5.45642267	5.33233464	5.49388359	5.60574061	5.45204094	4.76266106	5.242321	5.49425418	5.07642361
RMND1	3.98207957	3.46655964	4.31789847	4.67033342	3.90618132	4.50310549	4.65209243	4.80715468	4.16596348	4.56885888	4.34406543	4.23273661	4.36308674
SLA	5.39660806	5.37613611	5.65611631	5.69472607	3.99985688	2.83512682	3.93016293	3.0381928	3.89914095	3.78154015	1.60628801	2.27636239	4.29956839
TMBIM4	5.03300565	4.88796017	5.92344107	5.33612111	5.25638993	4.75125168	5.45541779	5.41138682	5.48298002	5.43459409	5.09157479	5.32297162	5.09594388
VBP1	3.86208339	4.57625032	4.35269437	5.58884391	5.10334669	5.28944753	5.11697166	5.30426343	5.53078261	4.41017145	4.87673789	4.93686124	4.81159282
RAB39B	2.95999109	3.07152321	2.59445423	2.38264112	2.91708453	3.93928245	2.83251212	2.6292557	3.0972525	3.47345724	1.66761264	3.02814763	2.2798181
CLIC2	2.94543125	5.20434209	2.81326099	4.08152058	4.42529204	4.89997408	4.61096806	4.49195001	5.47711669	3.81958018	3.84059416	4.7341929	4.71775473
AFF2	2.35828832	2.15082141	2.33879728	2.3924891	2.84956834	5.30617408	2.51679055	3.7710199	2.5059313	4.00685683	4.64670458	1.18625095	4.72597018
GRIP1	1.67667878	3.00213146	1.26460015	1.86927523	1.70380293	1.73671021	1.50862724	2.28213114	2.64392329	3.8908266	2.12382658	3.44003229	4.11039043
VPS37A	4.76183649	5.14734063	4.68411927	5.68756862	5.72617535	6.22424961	5.50346742	5.76668862	5.74731697	4.89468775	4.7827111	5.3900913	5.13496321
KIF5A	4.60129373	2.72439597	4.41388386	2.96477241	3.30381568	3.61287133	4.06140118	4.20615271	4.03795273	4.69564113	4.37620379	3.53014197	1.01975688
PSD3	5.11186988	6.01085501	5.04822731	6.37629157	4.79284919	4.94524931	4.86128761	5.59684054	5.88919305	5.53482226	5.71689644	6.01305408	6.43502921
MCU	2.72423186	2.36654147	2.25954757	3.42781321	4.81674937	3.68409476	3.37515466	4.22643716	2.92078034	4.41742518	4.4394583	4.47298041	0.94602555
GNA14	3.84905333	3.24278407	2.85394566	3.01757071	3.224586	2.19586883	3.23479099	3.65069333	2.97827967	3.82074461	3.59617076	2.42829853	4.01351098
FAM161B	5.35158277	4.69974599	5.2090988	4.3071376	4.89619142	4.86825528	4.68135939	4.6090483	5.01623381	5.2423872	5.03343039	4.79771686	5.14213559
GNAQ	6.65411398	7.17278233	6.73247526	7.26640564	7.66589667	7.4069645	7.80499756	7.36200338	7.54507893	6.64999043	7.46530871	7.30730024	6.75763086
MMP16	4.78847406	3.95734754	4.91982949	5.69011487	5.0212813	6.55563337	5.50467362	6.81597951	6.86220098	6.38192596	6.04266368	6.50842464	5.25268727
ADK	3.67654382	3.75326539	4.81191463	5.33301521	3.71133372	4.49757541	4.30285999	4.00429655	2.78163612	2.520418	3.04994508	3.7705793	3.24321602
KCNMA1	6.70984818	6.91097996	6.45578117	5.77546352	7.83802695	7.88974826	7.7168947	8.00383178	7.40091681	8.0533863	7.45469029	7.97543171	7.59760814
BATF	1.64246242	0.80283681	2.94173686	1.4873597	0.48913821	-0.02529099	0.87908976	-0.292292	-0.43633353	-0.43633353	2.58089767	-0.43633353	-0.43633353
DCK	3.49069191	4.81732146	4.07678532	5.03248454	4.77706157	4.83139552	4.87220302	4.68601418	4.77564455	3.71613475	5.2046437	4.38385861	4.2912383
ADAMTS3	5.37397669	4.51687188	4.91141809	5.50357963	3.58103714	3.88351031	3.89527013	3.95789237	4.45254841	3.04169427	4.20632202	3.54922985	3.28637311
ALX3	-0.02380941	0.25481358	-0.05205167	-0.05392725	-0.05392725	-0.05288445	-0.05392725	1.07206064	0.92471862	0.06866977	0.06866977	0.06866977	0.06866977
DPY19L4	4.66270612	5.04101386	4.71713204	5.84578267	5.70512867	5.69868738	5.86673075	5.79537592	5.68135616	4.82793003	5.70291324	5.92284172	5.05787143
DRAM2	4.69259045	5.37393057	4.54118126	6.06053633	5.45863838	5.22711988	5.54810023	5.40440347	5.6164336	4.92582441	4.89914317	5.43256248	5.49675187
C8orf37	3.96994788	3.22328247	3.3503565	4.13586547	4.13016779	4.85173644	3.71594163	3.67342404	4.30402619	3.9726375	3.83812408	3.86696582	2.37288228
PPEF2	0.40880925	0.41272266	0.44258178	0.34684412	0.68984978	0.87605067	0.61539948	0.85563974	1.62942805	-0.256339	-0.256339	-0.256339	-0.256339
ADAMTSL3	7.1205226	6.90149335	6.67994501	5.65601259	7.35911808	7.19704151	7.4495417	7.63316321	7.18598245	7.42957488	7.399805	7.15774585	7.49009768
SLC28A1	1.74034676	1.13661054	2.23664066	0.88963603	2.22509093	0.91599824	1.95862274	0.84753815	-0.70245122	3.24376315	3.579615	0.744739	1.06574611
WHAMM	5.68528616	5.19132255	5.17576378	4.23127292	5.04647584	4.8184223	5.03334011	5.1383182	4.73670684	4.88206811	4.54878027	4.85241673	4.1729834
CXCL13	0.12169867	0.85104207	-0.07473964	0.29856808	-0.07704919	-0.07576511	0.27536832	-0.07410275	-0.02953049	-0.02953049	-0.02953049	-0.02953049	-0.02953049
N6AMT1	3.80123039	4.33210647	3.38815737	3.24454668	4.62148495	5.260011	4.53873058	5.00759831	4.64746186	4.57534922	4.82230112	4.81272109	4.68436874
RWDD2B	4.67403904	4.7554313	4.777601	5.00039763	5.20511536	5.77554634	5.34630267	5.44217322	5.30277926	5.62454466	4.71925778	4.73262208	5.30432726
USP16	5.25913582	6.01303634	5.20953296	6.15145848	6.1632901	6.19013504	6.06355941	6.6670819	6.55072042	5.64347984	5.70894294	6.24287871	5.91432671
CCT8	5.61376098	6.10267747	5.98632424	7.06571349	6.95660561	6.85006976	6.98909911	7.07300255	6.55627085	6.38779127	6.4576718	6.57527394	6.3381239
BACH1	5.91529591	6.49405755	5.79987271	7.22666363	6.03222367	5.83200923	6.22558729	6.49445905	6.34200641	6.22233388	5.97756305	6.24742588	6.20083935
TSPAN7	0.14782292	0.57059677	-0.02239006	0.19683005	2.60588813	2.91002232	1.96743848	2.77844287	3.2342412	3.2920839	-0.16278755	-0.16278755	1.40347323
TIAM1	3.23416298	4.57327036	2.8545021	4.38816382	2.81075369	2.51727411	2.84410261	2.45145318	3.98302018	2.89821898	3.04195248	3.71967048	4.67611363

SCAF4	6.15792309	5.24191896	5.83719551	5.78649453	6.02074224	5.83227805	5.86877476	5.56604889	5.87594299	5.30734714	5.87360123	5.5673739	5.65207372
RPGR	3.64333746	4.28145645	3.62764661	4.4963075	5.25231514	4.29187043	4.39683298	4.51757108	4.11373484	4.23057424	3.3509612	3.25609704	4.53880267
CDK20	3.61811693	3.33093699	3.24923744	2.10159357	1.89756415	2.68729974	3.12936906	2.43475331	3.07936231	-1.19975777	3.44191259	3.49200176	3.34295751
PCGF6	2.92457133	2.98476158	3.49847394	4.62650177	3.51417878	4.07892048	4.03461125	3.99906393	4.07158023	4.34482164	3.33684789	3.56323412	3.68485716
ANKRD9	1.81102395	1.05084813	2.4772072	0.55354697	2.07556276	1.9730269	2.44283273	2.60882175	0.99253565	3.24569608	1.14442123	2.00219051	1.90783344
SFR1	2.99851086	3.2647326	4.1072351	3.80395912	3.11048574	3.49054325	3.48537188	3.95734348	4.02795134	3.0309314	3.25236906	3.7116321	3.2949986
SORCS3	0.04941141	0.06715623	0.00124472	0	0.0083578	0.00069205	0	0.00158797	0	0	0	0	0
SFXN2	2.84808819	2.73707685	2.67458358	3.28649578	3.01745903	2.05322162	2.83849983	3.65211755	2.46274688	-1.39316644	2.4831421	3.40789253	3.45397178
C14orf2	5.31330669	5.13335986	4.75994871	5.25018206	4.80161191	5.49119564	4.68632251	5.23053363	5.29629896	5.4506826	4.64946008	4.95473578	5.77207078
TDRD9	0.44302617	0.11249089	0.3264137	0.02331564	0.65482543	0.13350318	0.0011733	1.73691126	1.61312635	-0.03575126	-0.03575126	2.16425056	-0.03575126
FGF18	2.28105577	2.69384865	1.95555248	1.26414465	1.4747907	1.19620443	1.60123897	0.78202173	4.19945319	2.5826356	3.08423633	1.16955724	3.00598463
PCDH1	3.58125575	4.78065301	3.85404733	5.4541172	5.15163508	3.22973794	4.43493314	4.43052986	4.73800679	4.1528218	4.91121241	3.93204129	5.84941376
SH3RF2	3.37062204	2.45373853	3.03402633	1.36154291	1.64601169	3.38362166	2.44511653	3.21103862	3.89320638	1.95946814	1.85403907	3.07821576	3.54110073
GDF6	4.58446925	3.38427027	3.76310387	3.65117117	2.72614008	2.11345091	2.05501717	2.86987987	2.65442443	3.08874466	2.92533224	3.34959004	3.96568479
UQCRB	5.20561027	5.24443927	5.89503168	6.03009028	6.02070525	6.56700121	5.91029621	5.42264503	6.67971862	6.40779819	5.30988447	5.22734237	6.65433608
PTDSS1	5.46270646	5.13081943	4.96215974	5.76452451	4.95694917	4.92770837	4.96736923	5.07919895	4.73196553	4.8975378	4.89787857	4.90892508	6.1248328
PPP2R2B	4.68204664	4.59842378	3.85323924	3.48722114	5.30829481	5.7489227	4.78405506	5.88318041	5.02832801	5.76696215	4.92622715	5.74454159	5.63233087
RPL30	6.96871965	6.85850974	5.52942488	6.88337193	7.01931577	7.06174332	5.7666541	6.70489466	7.02771443	7.80581907	6.52446455	7.05638888	6.43044943
KCNS2	0.51157761	0.69151078	0.51417815	0.7357192	0.5963207	0.51302347	0.84776062	0.72296404	-0.32202734	3.43495262	3.07485642	3.29419737	-0.32202734
FAM122C	3.96129722	3.09140532	3.76270628	3.36247303	4.29024389	3.57057655	3.57236997	3.93772788	3.78286113	3.24559295	4.64143328	2.75155206	3.47031048
SUPV3L1	4.49943375	4.43368987	4.26174852	4.63635665	4.93682191	4.87948112	5.08602255	5.19238615	4.27714947	4.83696491	4.78623823	4.67789079	4.69232313
FAM122B	4.6247043	4.55599929	4.91747454	4.45035257	5.07267706	5.00403832	4.84783739	5.1118507	4.76719257	4.84609089	5.23038042	5.01598005	4.6728065
EEF1A1	10.0562161	10.7491502	10.3910429	10.1759479	9.88088352	9.73503736	10.3489156	9.82217364	10.0229875	10.3113425	9.83025706	10.2489539	9.7074233
FBXO43	0.43418727	0.61593409	1.02717078	1.12534197	0.27011377	0.2715266	0.76243354	0.27335564	-0.3952743	-0.3952743	-0.3952743	2.61090191	-0.3952743
HKDC1	0.13253574	0.30470935	0.37296866	0.43228064	0.324745	0.24438406	0.26747921	0.29694198	-0.09073364	-0.09073364	-0.09073364	-0.09073364	-0.09073364
HK1	6.1453227	5.97895205	6.49598081	5.70669947	5.88792803	5.62535506	5.97568364	5.54126078	0	0	0	0	0
TYSND1	4.63282623	3.97716714	4.5881255	3.155551	3.51707837	3.30264304	4.10474754	3.83713989	3.36599723	4.46911098	4.16502899	4.59572355	3.70828318
PHF6	4.14871045	4.97569802	4.68236693	5.54026933	5.31636267	5.49869546	5.21781097	5.02285604	5.57211026	4.85057064	5.43626055	5.40895953	5.26520779
CD109	5.64299552	6.0739539	6.33539019	7.05555702	6.44855998	7.03474526	5.90195563	6.97690862	6.93365627	6.25034486	6.74880102	6.98270367	6.51219596
LRFN2	0.02350381	0.21935807	0.00124472	0	0	0.00069205	0	0.00158797	0	0	0	0	0
UBE2L6	5.28813141	5.51288387	4.96605357	5.08286313	4.52342561	4.77146941	4.27411585	4.07416088	5.3400546	5.4696556	4.24501134	4.60133195	4.64211981
ZDHHC5	6.25969227	5.9277462	6.19976381	6.25969227	6.15115312	6.07012673	5.91166884	5.59717021	5.94210272	6.21526371	6.15883961	6.24324964	6.24471062
MED19	4.41560661	3.20331232	4.14298327	3.56169586	3.15933022	4.57327778	3.32287651	3.63907417	4.10854629	4.8311872	3.39621283	3.43869116	3.57116362
ZFAND3	6.77097595	6.12353132	6.00580001	5.86735848	6.380356	6.01696533	6.17639893	6.19011839	5.82833824	6.3428921	5.90349805	5.87740447	6.2996829
NPTN	5.90662402	6.01736566	5.69886266	5.88162844	6.96534473	6.96920562	6.37590968	6.63574933	6.59338922	6.93134716	6.53515258	7.15717496	6.13049051
KAT6B	5.29011489	4.87469845	4.60890921	4.18315462	5.10153983	5.48858185	5.25120493	5.05135977	5.57285194	5.17731416	4.67443936	5.4456383	4.60049622
SAMD8	2.64691706	2.94660171	2.12628985	2.9468192	3.44916447	3.55286608	3.75221354	3.78020297	3.15599402	3.47452853	1.87817438	2.43776658	3.54927524
RAB11FIP1	5.83221534	6.44605357	5.98421621	5.25255897	5.54109541	5.1212379	5.22983584	5.31251666	5.7265689	5.18843606	6.11677677	5.99876319	6.01572373
GLYATL2	0.28286477	0.28286477	0.28538156	0.28286477	0.49716441	0.28426407	1.12741322	1.1999997	2.03480224	3.51047557	-0.18260922	3.17743187	-0.18260922
UTP14A	3.69511224	2.26889432	3.82836441	3.09293882	2.00196607	3.73360784	1.88936164	1.67327988	2.61101521	2.25877607	-0.79475978	2.29789584	-0.79475978
AIFM1	4.94168121	3.62014094	5.19524914	4.57764878	4.31438066	4.48480781	4.50091319	4.29305427	4.71458345	4.6689687	3.71500562	4.18536963	5.22155123
MAPK13	4.60627058	3.40835622	4.4383621	4.40024365	3.18682532	2.85536401	3.48225881	3.356905	3.85676167	3.12575328	4.20440986	3.21919479	3.51851448
BAG4	5.68950771	5.243335	4.4101993	5.47665175	5.54403148	5.99435315	5.65312711	5.49208879	5.58878178	4.80906396	5.43347734	5.95470672	4.26969907
MS4A1	0.49569979	2.15391115	0.35645333	1.22519488	0.55107219	1.22608548	0.44070935	0.35713528	2.57639477	-0.25168844	3.35882087	-0.25168844	-0.25168844
WDYHV1	4.01873128	4.66115004	3.9527408	4.69074174	4.10303856	3.63765817	4.22668223	4.04396055	4.12103591	4.2345641	4.2553355	4.24519718	3.35548094
ATAD2	4.44267617	4.75163661	5.24759632	5.64000582	5.60905178	5.72616693	5.43593717	5.60163484	5.22199636	4.40047995	5.0250701	5.54954429	4.43460369
FBXO32	7.89011949	8.65898311	8.52879906	6.93524077	8.89978286	8.76943031	9.22252468	8.52879906	8.13578016	7.53004839	8.07531106	7.32808203	7.89124692

NSMCE2	3.08650925	3.8529023	3.55112279	4.53644071	4.02892313	4.40079121	4.55055784	4.35068191	4.52381199	3.0355442	3.47774483	4.27248244	4.29109059
ZNF689	4.80006607	4.4324783	4.48178992	3.55228987	4.4619692	4.30925641	4.13510529	4.40524047	4.43220096	4.52311507	4.55631724	5.04690463	3.79119065
PRR14	5.03028282	4.38642787	5.35395905	3.90113083	4.55851737	4.29761941	4.67283843	4.82293117	4.76877051	5.18231947	4.44645835	4.8846202	4.29213002
FBRS	4.57387168	4.03923418	4.93365284	3.66849275	4.03103445	3.41661768	4.08513015	3.7948573	3.36776654	4.67049073	3.59810719	4.65434226	4.418915
FRRS1	3.27105672	3.27074993	2.40462342	4.27544383	3.94526405	3.52464892	3.95015244	4.51520766	3.39930012	4.51708611	2.79940982	2.30579525	3.00920698
PHKG2	4.02674605	3.58782277	4.36838412	3.41079499	3.35450103	3.42386893	3.4583555	4.00082666	3.26581636	4.41430067	4.2558242	4.10932921	3.20352319
SASS6	3.16677795	3.58444369	3.38795	3.90224118	3.88340994	4.13999716	4.1967589	4.24499028	4.50617993	3.69213139	4.01201795	4.10750551	2.87647648
ITGAD	3.33392161	0.39047118	-0.00087397	0.57713555	0.04953632	-0.00087397	-0.0762859	0.0712444	-0.12675769	3.67901792	-0.12675769	-0.12675769	0.94690988
VPS8	5.27658842	5.95954227	4.96969376	6.15005882	5.73006529	5.77530407	6.04960718	5.6520371	5.98112129	5.30656322	5.91015073	5.6565085	6.20180881
GALK2	2.5627209	2.5700281	1.10205639	2.70699166	2.08864148	2.55814148	2.62518964	2.11175032	3.14543913	-0.40273974	4.48673328	3.7467442	4.16421062
B3GNT7	5.00840031	4.11312761	4.77179307	2.72102608	3.37503164	3.86439734	3.20974881	3.46857987	3.51266082	3.25499028	3.27051917	3.35310768	3.84258859
MPV17L	4.09480755	4.06964441	3.94106526	2.74356223	3.74298291	4.36834022	3.64048256	4.10657655	4.44957846	4.86551626	4.07960241	4.0547232	4.30977822
BUB1B	1.18893516	0.87508006	3.74630437	4.57924954	0.81942833	0.68309364	1.89062108	1.2576786	3.45648749	-0.97742556	-0.97742556	2.18865656	1.77580211
PDE6D	4.76292037	3.63536515	4.95437207	4.59470211	4.71316568	5.07803205	4.83352678	4.85206707	5.00880462	4.91872098	3.98649565	4.60838414	4.55144311
EIF4A2	6.86951521	7.24366923	6.37033238	7.07607736	7.398078	7.58469259	7.64501684	7.16523952	7.00318334	7.56147862	6.82417659	7.03653269	7.10280611
BRPF1	5.7009823	5.24184103	5.56394416	4.65773453	5.1181531	4.98973072	4.53442722	4.82874505	4.96717953	5.70750389	5.37166532	5.03903584	5.44228027
RPUSD3	4.67644358	3.99972526	4.95600145	3.66687943	3.47406301	3.73163612	4.19951453	3.92650981	4.05043174	4.59724241	3.91606386	3.73717156	3.89630315
TATDN2	5.22280736	5.68615179	5.67269892	5.09406755	5.06496115	4.72374099	5.45131852	5.37737526	5.00726323	5.26322038	5.68261431	5.43205355	5.24588181
GHRL	1.1254799	3.24887711	1.34110433	1.04666799	1.46321895	0.790984	0.14259368	1.04260223	0.63164642	3.17503829	3.00178235	-0.41416466	1.79178372
SEC13	6.11945857	5.93997548	6.08793281	6.89964773	5.83473643	5.63033232	5.54215403	5.70796051	6.16903164	6.48386653	6.36362847	6.1388011	6.21012306
EXOG	4.31520753	4.81095571	4.78129955	5.21362375	5.2927706	4.62539719	4.993506	4.95821865	4.95169563	4.72006034	5.14731023	4.6587032	5.06302726
NTAN1	5.72938288	5.53666729	5.36079894	5.17984155	6.00104326	5.0624434	5.05087228	5.51876418	5.43305679	5.4280429	5.15487916	4.91236397	5.25272166
NMNAT2	2.02684112	2.08470387	2.25066543	2.92446945	2.21282893	2.81826756	3.00489955	2.37726386	2.50072048	2.67132559	2.18350796	3.51425465	5.85879122
ZFYVE9	1.83640723	2.72652151	1.81783125	1.99823744	2.85320886	3.65988176	3.55382168	3.70960034	3.29279874	4.39423048	3.80266599	3.26535743	3.48833899
ATP2B2	0.23727634	0.23727634	0.46611336	0.23727634	0.28389433	0.36273508	0.2665596	0.63314843	-0.16062087	-0.16062087	-0.16062087	-0.16062087	2.47151799
SLC6A1	2.54079187	2.22333403	1.35489104	1.91213317	2.20916212	1.983729	0.94512471	3.42324027	2.76988384	3.30260563	3.50898869	-0.84025198	2.08864309
SMG1	6.75538762	7.11222967	6.59422939	7.18629497	7.35341241	7.35181506	7.46140171	7.50080805	6.80633157	6.87184837	7.15338648	6.81389278	7.50412968
FCHO2	4.72478183	4.72406517	4.01366695	5.38573498	5.23246284	5.23044094	5.2664455	5.29460496	5.7468176	5.07142123	4.40417076	4.99207002	4.93347957
RBPMS	6.51597007	5.99752818	6.00910482	5.19069601	6.91022055	6.12862564	6.83062718	6.99033064	5.66191252	6.5365948	5.42944391	6.14778378	6.16135984
TMEM171	0.02678381	0.37083099	0.00124472	0	0	0.00069205	0	0.00158797	0	0	0	0	0
TIMP4	4.24648153	2.95474713	3.88441515	3.02528721	4.65054488	6.88964104	5.12691944	6.1297493	3.10809394	4.62358195	2.70983322	5.04684678	4.73671212
SYN2	0.00859015	0	0.13581301	0	0.1823915	0.52517443	0.01867075	0.19558879	0	0	0	0	0
NRG1	0.59827178	2.19025063	0.86434775	1.63689128	0.38950769	0.21059455	0.2717892	0.16914581	0	0	0	0	0
C1orf27	4.13022022	4.70753962	4.14923522	4.87304256	4.93466758	5.35521351	5.01438194	5.30358075	5.12335699	3.91733145	4.97824738	4.60717744	4.84503711
CPT2	4.67279867	4.19819604	4.57434175	3.85423473	4.05441116	3.97961079	3.59887157	3.94657202	4.01789928	3.73124568	4.41793329	3.77669927	4.61953113
NECAP2	5.35801916	5.09485913	5.60171671	5.51557488	4.89808447	4.70297808	4.92168058	4.57007101	5.16363958	5.58910067	4.96421129	5.14198863	5.39469202
LRP8	5.76306867	3.63112863	5.53777219	4.42859211	3.77633239	4.65122646	5.25976034	3.4501048	4.41087072	3.21645574	3.7320242	4.25135469	3.50882829
CDCP2	0.05937115	0.05937115	0.27803462	0.16428581	0.3926776	0.29475381	0.05937115	0.06266043	2.46212854	-0.14276945	-0.14276945	-0.14276945	-0.14276945
PAXIP1	5.12332479	4.1791553	4.54600771	4.85961988	4.2386779	4.64198497	4.83190751	4.40463918	4.52301587	4.57275458	4.37452022	4.03211599	5.18836613
STEAP2	6.26383288	6.09674023	4.6968611	6.36136584	5.93234688	6.49046432	6.60698036	6.19050268	5.2605763	5.58617845	6.20061878	5.94166448	5.34226376
SSBP3	5.95374806	5.04332208	4.9148701	4.6628033	6.03844927	5.41232096	5.0185444	4.57728138	5.19515805	5.61433319	5.03680762	4.412102	4.59596278
CLDN12	5.52159468	4.92816461	4.72721374	5.56780556	5.87360842	6.3154927	6.00403256	5.69520939	5.5290901	4.0542494	5.68847802	6.00310155	4.73793022
MMP14	8.10844052	6.66843711	8.42188909	8.83386268	6.79497531	6.70692729	6.79911445	7.2391913	6.56588894	7.03166064	6.94223218	6.61741027	7.29065322
FZD1	5.58622638	6.28563358	5.48779507	5.09851493	6.25689775	6.33114529	6.3894437	5.79234549	5.97478963	5.94032646	6.40489373	6.26191497	5.56976664
GATAD1	5.66028547	5.74467765	5.83421107	5.54297022	6.21809472	5.79105312	6.17445837	6.09777011	6.10981041	6.26898867	6.13780933	5.9159349	5.92388693
SUSD3	2.62789865	3.75514665	2.39145626	0.69072725	1.96054787	3.36905194	2.13227367	1.02274141	3.35201577	4.88486334	3.28770398	1.71167924	2.60841942
TMED6	0.51845423	0.93400329	0.52100422	1.05038929	1.10020579	0.51987199	1.6522159	0.52170742	0.00794363	-0.45839156	-0.45839156	-0.45839156	-0.45839156

DHRS4	4.01315221	3.55800606	5.26894977	3.83170881	3.58862142	3.84989921	4.04501732	3.4774576	4.14644981	4.6978322	2.62383429	3.63609585	3.80682921
CLEC18C	2.22770863	0.78478809	1.77608666	0.81579492	1.40098577	1.15771961	1.05540109	1.90380781	-0.05088232	2.86900392	2.71650338	1.00374248	0.71690918
DDX19B	5.16672435	4.74639451	4.77828073	4.00886596	4.73685847	4.52630107	4.50917386	4.90855699	4.61942668	4.6267621	4.26344694	4.11620991	5.30932771
ST3GAL2	4.82029055	4.41417623	5.29233863	5.21445904	4.29332728	3.95548359	4.64656543	4.21704175	3.99280698	5.27488854	5.13881858	5.10225472	4.35650019
FUK	3.97998699	4.13389398	4.17309564	2.63070713	3.54028676	3.01864871	3.7023539	3.45721963	4.10625073	4.32699571	3.88640191	4.09364617	4.08576555
IL34	5.02341727	5.68854958	5.2818776	4.28714511	4.04636114	3.55786094	4.72784436	3.53474402	4.40536408	5.02590908	4.93183038	4.71708047	3.45461544
DHRS1	4.3800313	4.35988079	4.1303371	3.00780271	3.78996165	3.87363833	4.16239911	3.84354031	4.55477337	5.2268912	3.57348445	4.00570358	4.45593412
CACNA1D	0.18999322	0.13339111	0.78779468	0.3967456	0.07249978	0.12679404	1.07750295	2.12302108	3.30320825	-0.15534761	-0.15534761	2.62910073	-0.15534761
ARSE	4.82254731	1.42827979	3.54286048	2.76869011	2.33457106	2.75141194	2.89589054	2.69559616	3.09716225	4.32981027	3.59997781	3.71513048	3.3025745
KIT	3.09113083	4.45770669	2.36124037	3.5975449	4.77718763	6.14296939	6.04865349	5.11744697	4.42016966	4.16996695	-0.75624794	3.36979339	4.81641359
HYDIN	1.6500802	1.7534763	1.66469726	1.6992651	1.62436959	1.67573129	1.6121635	1.66424031	2.12018162	3.64550431	1.51607878	0.52330863	0.85435847
AASDH	3.7943298	4.53886986	3.97767844	4.94933805	4.80700745	4.90724747	4.82333609	4.57841223	4.99545859	4.29734752	4.77359977	4.81526768	4.57683965
ZNF19	3.08876656	3.50598259	3.66409859	3.08393552	3.55529177	3.23987487	3.37588806	4.29377589	4.25994069	3.18710359	3.42823586	3.46243513	3.7858459
CACNA2D3	1.46013031	2.96786098	1.50392183	1.90691648	1.92505545	4.47495503	3.40735657	2.80898864	2.14814637	3.75593111	2.46759185	2.94667787	5.46995032
RNF111	5.68504396	5.1759676	4.77562153	5.17457617	5.42793205	5.47550667	5.43775117	5.22210201	5.23253628	5.32678473	4.92090793	5.34659877	5.55251453
CCNB2	1.18094266	1.25961836	3.48005312	4.37532532	0.71842001	0.73967866	1.75153122	1.1005125	2.61150589	-0.92244476	2.9289967	0.94470621	2.98831506
FAM81A	0.13939583	0.40819274	0.32460417	0.46394675	0.66264483	0.53120465	1.03670921	0.4858602	2.00731195	-0.33202461	1.43574977	-0.33202461	-0.33202461
MYO1E	5.67587524	5.26814253	5.42390496	5.35851065	6.59081167	6.29711912	5.24658583	5.9428371	5.0802022	6.00003206	5.67791383	5.26256594	4.94054295
APPL1	6.43709987	6.96268605	5.75301174	6.92163487	6.8883814	7.0203005	7.0199465	6.72090746	6.63204795	6.47872787	6.41357344	6.32998622	6.8270906
MUM1L1	0.8132912	2.76889725	0.94932028	1.21504907	0.79842646	1.57776722	0.85829859	1.02213007	2.29817049	3.5585773	2.3363435	-0.72319854	2.96904916
AFAP1L1	3.51136045	4.82332744	4.36129467	5.65138403	4.44422947	3.10396972	3.32594085	3.75406304	4.179728	4.66337155	4.6753147	3.87852228	5.60193845
TSC22D3	6.57688075	7.30305501	6.59276254	5.44142996	7.18327542	7.55441568	7.05117308	7.18930701	6.39862662	6.4470351	6.73112345	6.66963958	6.32476938
DSCR3	5.71385293	5.70665293	5.44115892	5.52516913	5.59680404	5.33848292	5.84087276	5.91005682	5.26320329	5.67183232	5.82870645	5.64417848	5.61239814
DYRK1A	6.56389972	6.67695154	6.12217432	6.43395209	6.95559835	6.78576989	6.81258993	6.84242081	6.45816663	6.40703606	6.48965764	6.4014666	6.47674037
KCNJ6	0.48989615	0.30017599	0.20421087	0.20631915	0.23015226	0.28152189	0.22867754	0.20492744	-0.09938358	-0.09938358	-0.09938358	-0.09938358	-0.09938358
KCNJ15	0.97981541	-0.23642126	0.32752448	0.90277368	-0.23060538	-0.51770852	-0.17120809	-0.25832462	-0.13424898	-0.13424898	-0.13424898	-0.13424898	-0.13424898
ERG	8.63515269	7.06642627	7.76488938	7.15533526	7.28669414	6.61340364	7.71951763	7.54684076	7.65637354	7.52862117	7.52155065	6.86149537	7.20315726
ETS2	9.16023728	7.37803117	8.14479486	7.14662934	6.97564004	6.96949451	7.40029011	6.54258014	6.89154227	7.2312442	6.86037459	6.65718717	6.89154227
TSPAN18	1.62051135	5.3256968	2.56655089	5.59195671	4.70574668	1.93289303	3.22301511	2.9619469	4.85181506	4.99864217	5.92742872	4.56541832	6.7046705
LCA5L	1.76854083	1.99496424	1.31164035	2.18574917	1.17745282	1.56368724	1.13289785	2.00742533	1.11581575	-0.78184475	1.07560611	1.88340964	-0.78184475
SLC35B2	1.91903206	1.08107911	1.8993172	1.80514444	0.45029255	0.92571657	0.3822117	0.83242679	1.22200171	0.48942073	4.00466457	0.48942073	0.48942073
TMEM164	3.25336111	2.63107949	3.57178304	2.91257082	3.10940179	2.11826119	2.86887129	2.23203858	0.18290024	-0.54245591	2.39294414	2.96855782	2.99997755
MX1	6.36577315	6.79330507	6.29463813	7.07541732	6.17175121	6.14247846	4.65990521	4.92175002	6.94126092	5.55312716	6.70018877	4.87479593	7.2358145
CREB3L1	4.55031861	3.75076385	3.47959929	6.18057984	2.64635061	1.64749845	2.56048778	2.87379385	2.17188284	3.44797938	4.17304636	0.33351497	4.23895882
C2CD2	6.58633737	6.00622499	6.45569499	5.63905082	6.47970829	6.28033926	6.40905162	5.74398149	5.95185053	5.75413519	6.59617596	6.04676951	6.271259
TAB3	6.21425665	5.76605402	5.28077635	5.938107	6.20479424	5.89222218	5.68258185	5.93319556	5.81671339	5.47246466	5.40607707	5.47246466	5.86694369
SLC38A10	6.00280918	5.92192149	6.63106882	5.75599179	5.43663598	5.35461127	5.82261594	5.35375678	5.44571569	6.55836233	5.96275204	5.79210874	5.76599781
C9orf43	0.37687697	-0.01598767	0.76268705	0.804941	0.97316767	0.2297356	0.71745293	0.06280579	2.29339526	-0.20609081	-0.20609081	-0.20609081	-0.20609081
ZNF618	6.22181669	5.97838079	6.06496151	5.31566847	5.71728567	5.38196526	5.65000391	5.16591216	5.84152169	5.7993446	5.97105621	5.34211066	5.56986511
DGKI	4.09753621	3.75300348	3.86510028	4.98442566	6.22393553	5.55952394	5.10584808	5.19901347	5.16489053	5.62855972	5.95005438	5.15984993	4.98013193
SNX22	0.34664754	0.49619388	0.43247743	0.09389945	0.11218225	0.15389119	0.26915003	0.04763439	-0.13761995	-0.13761995	-0.13761995	-0.13761995	2.75247251
UBN2	6.01286643	6.05818441	5.64809484	6.1971133	6.49186209	6.44268154	6.38557477	6.42179751	6.18663443	6.06449936	6.17026491	6.12234111	6.08074083
BRAF	6.42555409	6.52218698	6.0889014	6.46020756	6.87833339	7.06059608	6.78492123	6.93667237	6.33623071	6.70794019	6.68956699	6.54133715	6.61392602
SLC34A2	0.4258261	0.92364642	0.35654785	1.11720126	0.37507989	0.3554143	0.35399489	0.35725187	-0.17961334	-0.17961334	2.30613191	-0.17961334	-0.17961334
ACAN	4.13120135	3.88315145	5.46118927	4.92784641	7.83715377	5.61280178	5.88956414	5.82992935	6.5766536	5.37978849	6.55017027	6.7286895	5.28564103
PSMG3	2.83002307	4.21671068	4.61274719	3.34565759	2.11400444	2.63251047	3.47004635	2.43420436	3.29745337	3.81521082	3.65365506	3.62971729	3.14269414
CABP1	0.24990764	0.30913396	0.3178209	-0.34593184	0.8896447	1.22612826	1.12641169	1.41528568	1.57811784	-0.17734883	-0.17734883	-0.17734883	-0.17734883

WDR19	6.10612522	6.30938081	5.78238751	6.78853547	6.66099481	6.48417516	6.19191566	5.91446932	6.78360281	5.95414123	7.2163349	5.98634035	6.12466788
SLC37A3	5.44484804	5.70246409	5.29098977	6.09670437	6.03171603	5.26538434	5.83814516	6.17200153	5.58477586	5.73025663	5.65669031	6.00538565	5.27659976
AP3S2	1.11278693	1.12535179	1.29473718	0.71482523	1.33796702	0.62223755	0.89058266	0.70069461	1.40873652	0.22840977	0.22840977	0.22840977	0.22840977
FMNL2	2.92524512	2.71039597	2.96033103	4.01208803	3.90504102	3.71242462	3.98513236	4.64262281	4.10863367	4.40587502	4.09826696	4.43808351	3.91163082
SPPL3	5.77459724	5.92062487	5.44413658	5.24602936	5.67020607	5.1573607	5.56739207	5.48952958	5.42404376	5.86121251	5.96542839	5.48361502	5.69122316
RAB28	4.61961653	5.51378285	4.45906536	5.22051102	4.99358135	5.62620834	5.35222157	5.2109225	5.23422038	4.17388531	5.29342785	5.10070972	4.9456282
TNFRSF14	4.21301231	4.00874717	4.65576153	3.48774354	3.46174634	2.43275695	3.37500352	2.91383437	2.36579301	2.78611164	3.10593708	3.24758251	2.76396577
PANK4	4.90823565	4.07380548	4.79743602	3.83937306	4.20583948	4.30394726	4.37893538	4.60109829	3.89493712	4.29263933	4.41956914	4.25203308	4.39768941
CIB4	0.9829746	0.64308198	0.08733789	-0.27734574	-0.14973073	1.09622648	1.22919461	-0.52990798	-0.00367843	-0.00367843	-0.00367843	-0.00367843	-0.00367843
MEGF11	0.01058708	0	0.04079605	0	0.00791055	0.03992277	0.03270389	0.10546978	0	0	0	0	0
C12orf43	4.67842727	3.81316066	5.06063891	3.72740649	4.46348104	4.28201764	4.13115241	4.39148229	4.31643827	4.23246931	4.14989925	4.5875281	4.27841429
PEX10	4.50634461	4.40492831	4.64146258	4.08384003	3.78385391	3.74527096	3.58003801	3.86116202	4.41054839	4.46438954	4.0251898	4.39131967	4.98241429
RER1	6.2202572	5.86935014	5.93595253	6.05475893	5.70094418	5.61109139	5.98613459	5.71000694	5.56958228	5.94540657	6.09908183	5.99522545	5.78443834
RADIL	2.48718478	1.52849361	2.25697948	0.66166791	1.44521964	1.22230711	1.48505198	2.37873823	2.30770554	2.61066344	-0.50981854	2.69039503	2.14650932
SKI	7.63407151	7.00413102	7.57748988	6.20485306	7.03278698	6.9071908	7.31348218	7.12091557	6.70896245	6.73016301	6.84342388	7.15175566	6.89133858
WIPI2	5.821867	5.42950659	5.65143032	5.32650797	5.21909175	5.09427025	5.12514375	5.51884003	5.44994063	5.9778745	5.59729313	5.28265814	5.73735645
LDLRAP1	5.76649382	5.36293608	6.09616133	4.85867372	5.11529447	5.54526374	5.83617202	5.77530695	5.64771355	6.10604037	6.2589982	5.48724326	5.27772501
AGAP1	2.74790572	2.26323438	2.01424433	1.17315086	2.08122133	1.53196292	2.12188029	2.36979358	2.27725333	3.58644408	-0.22354774	1.59071844	-0.22354774
KRTCAP3	1.32860222	1.05455403	0.18469657	1.00568194	0.41543422	0.55254259	0.75503624	-0.12306287	-0.16573004	-0.16573004	-0.16573004	-0.16573004	3.17284517
PAFAH2	4.20179774	4.07491067	4.87328479	3.87030585	4.06531973	4.42394378	3.79419766	3.62509782	3.8413011	4.60862349	4.67419777	3.64636385	4.99864222
EXTL1	0.16096769	0.64976646	0.64463315	-0.11946708	-0.02338568	0.91668876	0.26927732	1.17849066	-0.25510949	-0.25510949	-0.25510949	-0.25510949	-0.25510949
SLC30A2	-0.05624704	-0.05624704	-0.05378691	0.40595958	-0.0254755	-0.05487925	-0.05624704	-0.05310849	-0.03920914	-0.03920914	-0.03920914	-0.03920914	-0.03920914
TRIM63	0.75235407	0.88063212	0.433771	0.54261252	0.43117293	0.43261742	0.43117293	0.43448745	-0.0381709	-0.0381709	1.08035419	-0.0381709	7.41210023
WDR66	3.74029232	3.52312985	3.79402867	3.53780878	4.49877378	3.57306722	3.17545073	3.76814722	3.53647535	2.78474892	4.41384065	3.9553577	3.92106935
MRPL17	4.62711334	4.07761236	5.01620762	5.41045151	4.40793437	4.7992294	4.44673377	4.12564149	4.55489738	4.61907442	5.07114698	4.72418764	5.06527851
DUSP2	1.76131824	2.3383239	2.14368962	2.54350812	1.7073689	1.71112027	1.77118256	1.08001315	1.84107666	3.60762892	3.58910242	2.31207817	3.93436324
GRHL3	0.09538227	0.03172433	0.44378854	0.12944522	0.59728231	0.25372206	0.14271213	0.03497979	-0.07522668	-0.07522668	-0.07522668	3.33258866	-0.07522668
UBXN11	4.30642048	4.36813998	4.97807106	4.40152926	3.42833898	3.18235068	3.31347524	3.2813029	2.76455326	3.86584045	4.16804154	2.26631982	3.09526245
NLRP14	0.25560242	0.29329911	0.61034482	0.5150958	0.40753771	0.21302887	0.25209399	0.53735873	-0.15600151	-0.15600151	-0.15600151	3.20141581	3.29604114
PTPDC1	0.61827942	1.18710022	0.17182321	1.31489248	1.88136296	2.24513941	1.15887206	2.31558861	3.92392199	0.18828642	3.68995473	0.18828642	0.18828642
GALNT14	1.54034058	1.58753148	2.4742625	2.4664158	2.37563738	2.31085768	1.50271731	1.38982935	3.65769256	3.67113742	2.97140656	2.542605	-0.91157456
NCK1	2.08332958	2.96787492	1.79769835	3.01246074	3.09252914	3.27287095	2.6048151	3.21325447	3.72398042	4.02596147	3.24614769	1.33793605	-0.67207693
HPD	3.87235555	2.16543599	4.03291527	0.49777011	2.25396213	4.12317369	2.34102794	2.66253401	1.72765226	4.36668368	-1.00035883	4.04928941	4.19172393
RHPN1	1.73704802	1.33419827	2.63949398	1.07123446	1.26701692	0.39016768	1.36560796	1.43627599	3.11632794	-0.67836438	3.04282008	2.32756055	2.41214991
TPRG1L	6.15168676	6.03203633	5.90967056	4.99431943	6.01662848	5.69051118	5.65180402	5.44431087	5.55162595	5.81524025	5.42153074	6.27572177	5.71707804
LRRC43	0.36728707	0.16408231	0.37525807	0.07505215	0.5902293	0.08483364	0.11708514	0.07289476	0	0	0	0	0
XDH	0.1010498	0.39193621	0.27284527	0.18727578	0.07829724	0.0621394	0.17545334	0.14889582	0.06111783	0.06111783	0.06111783	0.06111783	0.06111783
XKR8	4.16997391	3.13597195	3.63860896	2.94474509	3.77219863	3.1247996	3.85970582	2.88842744	3.53704703	3.15588381	3.89608561	4.5391405	2.46921319
CNNM4	4.22317995	3.03526269	3.10760319	2.74900948	2.73265129	3.22738945	3.17658632	3.27696345	2.8240485	4.32716547	3.46101452	3.56930273	2.38194259
EYA3	5.25020522	5.58628601	5.44497953	5.62604018	6.01703342	5.59320596	6.09501929	5.50898863	5.68314953	5.31109287	5.83715947	5.61880561	5.57185173
DZIP1L	5.2256141	4.7755441	5.52614345	5.26047569	4.86484608	4.42066007	4.59750988	4.73697503	4.79536988	4.4652472	5.04723891	4.51879417	5.07158461
TMSB15A	-0.65056554	0.45755364	-0.20170138	1.36804328	-0.25812632	0.73816486	0.09329937	0.45898526	-0.09172955	-0.09172955	-0.09172955	-0.09172955	-0.09172955
FANCC	1.44922423	1.98792486	2.13604074	3.20472383	1.90247781	1.3681502	2.52573145	2.12487564	1.10634997	-0.79566411	2.56205516	0.22979023	-0.79566411
MRAS	6.23122872	6.51787235	6.26633563	5.6027523	5.98372873	5.58875839	6.4733389	5.52520227	5.72300128	6.43838362	6.35938559	5.89335673	7.1002249
WASF2	8.09417877	7.78035054	8.14361637	7.17862624	7.58838387	6.98840266	7.71092067	7.56739316	7.25809717	7.46441136	7.64674869	7.37859939	7.357866
ABHD3	3.39000809	3.32570988	2.6520543	3.86189193	3.66847123	2.52789869	4.05548372	3.97198356	3.53096106	4.71571709	1.12002402	3.16243578	3.12030757
ESYT3	3.30317642	3.25465205	3.48762562	2.25034666	2.78201629	3.35671496	3.43518018	4.17870933	3.47341078	4.06913522	3.59900838	3.20747123	3.68485516

FAIM	3.09941604	3.22024228	3.50268757	3.10217501	4.32114492	4.14492308	4.01027885	4.57679938	4.32122633	3.17487724	3.83600092	4.61719774	3.84639006
FAM46B	4.64717689	4.80380538	4.76315629	2.34836634	4.86251935	5.67656625	5.43352943	5.24964548	4.48078028	6.4278909	5.10295967	6.48363266	4.32641857
CLSTN2	1.02086232	2.14440873	0.36230986	1.36146766	3.40950328	3.14595015	2.97215601	4.34130912	3.62147268	4.09651749	3.68217292	4.15333554	4.79783876
COLEC12	6.70258799	6.56039632	6.07497575	6.41811786	5.54318061	4.68606141	5.0641778	5.23425402	6.15663914	5.06598597	6.18667881	5.70210667	6.66846981
RNF207	4.4911417	4.31108585	4.18368476	3.95138923	3.64110172	3.00503701	3.69555998	3.16001307	3.30638701	3.51894445	3.18866262	3.42421527	3.91338201
CUL4B	5.83248022	6.58366778	6.2903157	7.00749821	6.86870189	7.18409795	7.31492203	7.01885323	7.01845289	6.09520444	6.60317638	6.60317638	6.01262078
GPR153	3.4539069	3.21544077	2.97176025	3.99680183	2.60459158	1.7066126	2.84683261	2.45383251	2.06215177	3.35431068	4.03149819	2.8899168	3.27414081
SLC13A3	2.49090469	3.51209261	3.22281243	2.38136689	2.79876042	2.75062302	2.74934089	2.53236959	2.98924775	3.80381262	3.03774646	2.41065675	2.73176552
GPRASP2	5.34130815	5.29707878	5.35630741	4.49446511	4.83111104	5.03746907	4.46822447	5.1875749	5.34537822	5.31390598	5.61723467	5.28885402	5.19010033
RHBDL2	0.44828535	1.2669418	0.49383636	1.52310545	0.33879798	0.34018846	0.33879798	0.34198858	-0.47530594	-0.47530594	-0.47530594	-0.47530594	-0.47530594
AUTS2	6.76138988	5.56606467	6.53410156	5.39064889	6.58078483	5.66212036	5.8325409	6.26359174	5.67744212	5.97822947	5.65454174	5.78941517	6.06626534
SHROOM4	5.20452569	5.41237986	4.90741656	5.30357141	5.01917641	4.533471	4.47268102	5.16248127	4.6127305	4.82359896	4.50522652	4.46896064	5.59856679
HIST1H2BD	4.30949616	4.57501064	4.04929253	5.35129625	3.40761587	4.33757906	3.88414836	3.04336541	4.04131197	3.76826199	3.80761513	4.29765852	4.46643128
CDC25C	0.42391291	0.8806345	2.57296199	2.86136466	0.01573776	0.01689043	0.52378553	0.01838267	-0.44363525	-0.44363525	3.07325568	-0.44363525	-0.44363525
HIST1H4H	5.12487892	5.98772308	5.79866953	5.59301275	4.59153789	5.01393978	4.51157855	4.38682706	5.17903597	6.20726362	4.69981055	5.37661989	4.87118759
MITD1	3.89315051	3.58359773	3.62158431	4.20697048	4.50275821	4.41435277	5.13356143	4.23993183	4.9120357	3.37966614	3.88759707	4.3243415	4.53826386
EIF5B	5.87337785	6.31568172	6.32829308	6.67912146	6.73518549	7.16457116	6.09374232	6.46571488	6.97865489	6.57963944	5.97261778	6.00297244	6.68283389
RIBC1	2.06826015	0.89666395	1.08772875	0.7219043	1.74851215	2.71706432	1.1453736	1.24682492	2.80462785	3.85324103	-0.66457756	-0.66457756	3.30984026
TMSB15B	-0.0348429	0.09631192	0.79019081	0.25462978	0.2091989	0.14028397	0.22027153	0.47641072	0.08893896	0.08893896	0.08893896	0.08893896	0.08893896
KCNB1	3.26167757	3.62610381	2.33377485	2.5807403	2.92197879	4.28602366	2.79705251	3.63372506	2.33820168	4.46966664	3.28434892	3.61655961	5.23544902
TSPAN33	3.78508488	5.18546193	4.02719434	3.35170657	2.49833727	2.3325676	2.83201172	2.21242772	3.77390871	4.13971038	3.90593992	3.30956249	3.86547403
NRG2	3.01746599	1.8885448	2.00464502	1.25657253	2.16188385	2.95495022	2.06733519	1.98993606	2.3124097	-1.06765631	2.9852945	3.132952	2.56679472
AHCYL2	4.29496866	4.38651557	4.07822973	3.90727504	4.24742571	4.31290732	4.47893821	4.37830357	4.6023013	3.40672603	4.28426759	3.86659796	4.4211973
B4GALT5	6.39081846	6.0288163	6.41801523	6.50516005	5.98412222	5.96640632	6.42421772	5.20155486	5.75067007	5.32913279	5.72795808	5.25734235	6.06533899
CD1D	2.990991	3.39313264	2.54488611	4.05567663	2.02303964	1.57382122	1.90194895	1.30942779	3.18646368	-1.1528898	-1.1528898	1.50835309	2.81469274
CD1A	-0.00483047	-0.03929537	-0.03687008	0.35707191	-0.03929537	0.35201021	-0.03929537	-0.03620126	1.75316165	-0.03989814	-0.03989814	-0.03989814	-0.03989814
SPATA2	5.15899901	3.36894817	4.56141875	3.60423021	4.02483521	3.90701417	4.20235336	3.83350893	3.33636563	4.28242324	2.90065101	4.5874789	4.39447803
CD1C	0.69123599	3.42646887	0.5312635	3.74717338	0.75351586	2.0814146	1.67185454	1.46624845	4.28851668	4.07323065	-0.7558538	4.42612394	1.42879318
DNAH3	0.21259635	0.20559063	0.20819537	0.20746729	0.30968805	0.2888288	0.20559063	0.20891367	1.37236827	-0.14090364	-0.14090364	-0.14090364	2.86632285
CD1E	0.61488484	0.57140415	0.57398018	1.38087768	0.57140415	0.76474318	0.65813979	0.57469057	3.6327391	3.61878137	-0.3339879	1.41587494	-0.3339879
CPA2	0	0.47755544	0.10507228	0	0	0.00069205	0	0.09799307	0	0	0	0	0
NCF1	3.9890404	3.59992414	1.40973149	1.6508279	1.12895408	0.90620009	0.70667154	0.92191143	2.73449534	4.09950384	1.72093587	1.13353663	3.51222723
TSR2	5.96993337	6.01561567	6.17442553	5.31452102	5.56882362	5.68624745	5.37210924	5.18788766	6.10443196	6.19401332	5.96862402	5.79358487	5.84430505
PPP1R9A	3.483321	4.68320128	3.458483	2.58652289	4.35110297	5.647627	5.01874739	5.46373638	5.35574302	5.68988579	4.86323346	5.54767019	5.61672928
ZC3H18	6.06879116	5.27213737	6.37421366	5.05491946	5.68080171	5.32570697	5.46976637	5.45354085	5.28406162	5.3437138	5.52906528	5.18192962	5.37335032
ZFAND2B	4.73471861	4.37140498	4.68786797	3.12266788	4.04031204	4.31596259	3.82189754	4.66968415	4.50694906	4.37112717	3.95293438	4.35353471	3.9883171
GDPD5	4.02370197	4.91066225	4.70335179	3.81665455	3.87368358	2.93537752	2.93878607	4.18649582	3.95200311	4.16154339	5.4297195	3.76596107	3.80390078
DYNC1I1	5.73451685	5.17937661	5.09621686	4.17490976	5.24191403	6.33088324	5.56690087	5.4002103	5.95355858	6.04999472	5.20939345	5.38272801	5.8401342
PFKFB1	0.51173645	-0.02912149	0.6036844	0.35836129	1.34612852	0.54040912	0.14031743	0.4176805	-0.21840639	-0.21840639	-0.21840639	-0.21840639	-0.21840639
ALAS2	1.87246321	0.21720372	0.67432789	0.62495731	1.32484529	-0.6354088	-0.48671924	-0.42248495	0.08872106	0.08872106	0.08872106	3.12233551	0.08872106
TMED4	6.09290985	6.22708813	5.53539586	5.08497048	5.96443605	5.89879986	5.81198877	6.09342145	6.04354172	6.59083272	6.15054144	6.38912896	6.26892529
PPP1R15B	5.88192797	6.32707868	6.07032952	6.63029416	6.48622985	6.46471674	6.37899808	6.72100183	6.33962093	6.18671252	6.53112075	6.35224859	6.47005999
COPG2	3.6440108	3.40843596	3.61146004	4.53409014	3.30575457	3.96660327	3.65343926	2.83112803	3.83595394	4.52485217	4.18190135	4.55131174	3.56355236
PKD1L1	0.40167921	2.09907675	0.32241259	0.81799836	1.77589049	0.52259913	0.72787046	1.20549414	3.02708587	2.07137381	2.03197271	-0.35655013	3.6073761
ZSCAN12	4.6831799	5.26439654	4.5500008	4.87510769	5.18495251	5.633945	4.84079077	5.40222448	5.10786592	5.04380145	4.94398618	4.66235819	5.17673659
TAGLN2	6.96867082	7.23483443	7.36888786	7.63973628	6.47529533	6.22838558	6.43948653	6.07923493	6.65675384	7.09147396	7.10809655	7.10760698	6.48079627
ELK4	6.4925038	6.72493551	6.08241562	7.27447209	6.91581931	6.62796938	6.94197921	6.79774271	7.16015003	6.18601319	6.78406179	6.3809788	6.8219031

SLAMF8	5.54553117	4.10640617	4.4889754	4.98528935	2.74687106	2.079599	3.68418184	1.74625632	3.49242674	3.08736168	3.62640529	2.63357433	4.01400392
SLC45A3	3.39164136	2.54912048	3.87147627	2.2889542	3.17747981	2.39938751	3.83251876	4.09311536	3.22119784	4.33154376	4.04553445	3.54699875	2.4206733
DUSP23	2.92900154	2.69967556	3.19866332	2.49617681	2.81529901	2.52871092	1.44628621	3.2359947	3.74512455	3.92859201	3.08742482	2.23224492	0.92091888
RNF166	4.01598288	4.12181787	4.29994639	3.91278888	3.61322532	2.4849126	3.26531453	3.01951146	4.16156902	3.59701731	2.87871438	3.73011173	3.81645775
ITLN2	0.27869393	3.56708002	0.10369694	0.10175008	0.21179655	0.47214045	0.9823788	0.10423382	3.06497592	-0.20957739	3.30207536	2.05419535	-0.20957739
F11R	3.9253819	5.25172211	4.44583889	4.67287127	4.1463951	3.61043457	4.01079297	3.72034212	4.15794402	3.10802448	5.13664507	4.25050751	4.7002536
USF1	5.53164	5.5165652	5.1491195	5.5057458	4.73955829	4.95056706	4.93108149	4.34630801	5.11764782	5.01087332	5.10618127	4.96940479	4.06251129
SPATA2L	2.84838956	1.59608855	4.26859142	1.38875448	2.05103548	1.55956023	2.26604345	2.76777597	2.14836979	2.14524743	2.08486324	3.36836741	-0.90511041
NIT1	5.30931752	5.12962575	4.92406388	4.26740827	4.73263993	4.62934764	4.86681078	5.11606887	4.6878971	5.61992625	4.87098794	5.35808878	4.33915761
DEDD	6.86018467	5.65043225	5.90321054	5.58998275	5.50427334	5.5574162	5.73272133	5.95416703	5.45373527	5.60853125	5.72735318	5.85255953	5.45047962
ZNF276	4.77893299	4.32290481	5.10144417	4.41101994	4.58712597	3.36650849	4.19943486	4.61001451	4.29336494	4.98437824	5.22262637	4.20000112	4.50374843
NPM2	0.75118796	0.5902224	1.61445317	0.25404929	1.81866858	1.67435389	0.89154949	0.72404576	-0.27890861	-0.27890861	-0.27890861	-0.27890861	-0.27890861
EDA	1.25976952	1.92368162	0.5370493	0.01333266	1.73158763	1.77783632	1.88750339	1.67190068	2.81947706	-0.17417004	-0.17417004	3.64818242	-0.17417004
FGF17	0.76447152	-0.17851551	0.32673841	0.24781851	1.14410796	0.25437841	0.58876766	0.76389449	-0.07832845	-0.07832845	-0.07832845	-0.07832845	-0.07832845
CDA	1.60577846	1.49876125	1.21197798	-0.33799666	0.63048102	0.2648694	0.93517703	-0.41987491	1.43355501	-0.34965301	0.74845094	-0.34965301	3.32731081
PINK1	1.86937218	1.78734656	1.51496458	-0.75131352	1.53638695	1.47726628	1.30019567	1.78590231	0.70133714	0.70133714	3.29492273	4.03781049	0.70133714
B4GALT3	4.58947497	4.07061858	4.75229442	4.46112391	3.92245798	3.80677139	4.11789893	4.21405846	4.06016545	4.94575549	4.14529377	3.66755787	4.42174199
ADAMTS4	4.73598576	3.01453298	5.39022344	6.92045359	3.84782576	2.54863107	3.63567084	2.96978688	3.79020178	3.55989357	4.21083432	3.90531662	4.89805063
FAM160B2	5.67310867	5.10946077	5.63069086	4.63244274	5.02417968	4.22310189	4.99992094	5.10689896	4.82122654	5.20223916	5.11751987	5.49470979	4.54595429
NDUFS2	6.04419337	5.36227976	6.03939352	5.87715098	5.2004391	5.07488536	5.36227976	5.11266977	5.50236633	5.19854887	5.3451377	5.15569866	6.70669036
SLC5A11	0.39640893	0.0879868	0.45011168	0.0879868	0.35109855	0.08942132	0.13576364	0.09127846	2.41310452	-0.10431911	-0.10431911	-0.10431911	-0.10431911
FCER1G	6.01384157	5.07945579	5.98146397	6.33388819	4.39378314	4.22714501	4.88461858	3.40573093	5.0479261	5.27633012	4.47383961	3.88254218	5.27401012
TOMM40L	3.34377048	2.44533209	3.7194346	1.84282278	1.90155473	2.32504069	2.39265186	2.01386798	3.18253838	-0.48377075	2.80391952	2.99715678	3.95125891
MPZ	2.35280706	2.9472064	2.42700077	2.4380803	2.98443585	3.42023164	3.75033595	2.07998663	2.27233185	3.84105125	4.10965822	2.66105873	4.93010477
WFDC8	0	0.54075843	0.00124472	0	0	0.00069205	0	0.00158797	0	0	0	0	0
WNT9B	0.22483302	0.3385782	0.33785442	0.3940487	0.4490441	0.40123787	-0.03144431	-0.02816456	-0.07576717	-0.07576717	-0.07576717	-0.07576717	-0.07576717
CACHD1	2.98957572	3.45028015	1.38368355	1.24674425	1.67237327	1.83354117	1.83354117	2.4656811	0.68800515	3.5838793	4.10057403	1.47627389	2.846802
CDC42SE2	5.02319206	5.82602815	4.91391539	5.95158647	5.6596876	5.76712368	5.6567583	5.42941144	5.62897174	5.67128141	5.45202809	5.61331832	5.35287418
RAPGEF6	4.58822422	4.61024922	4.55846608	5.25285328	5.30504135	5.49568069	5.21277099	5.51044242	5.04300499	4.54220302	5.31289516	5.05473847	5.34596501
EPB41	1.36935953	1.51891861	0.71065928	0.61450542	1.33535895	1.11877907	1.94468182	1.23230122	2.79944659	0.43980351	0.43980351	0.43980351	0.43980351
MIS18A	3.08936388	2.60209765	4.13795685	3.7577399	2.3437589	3.70718236	3.57450564	2.37119752	3.37087228	3.08503527	2.91288894	4.03462142	3.45915964
ALG8	3.78304546	4.83821509	4.2832992	5.27747808	4.54457539	4.1729253	4.57127915	4.23852399	4.40051094	3.74213428	4.33986757	4.31015216	4.28903263
FBXW5	5.55807962	5.93882512	5.72240509	4.56905881	5.17145174	5.48804548	5.36404069	5.72556488	5.47489135	6.22522924	5.68677022	5.94639903	4.73734891
C21orf59	4.76009203	4.46418376	4.61119089	4.21055596	4.5502972	4.91504514	5.01576991	4.79248473	4.70049177	4.55668534	4.60906832	3.85755254	4.75639174
SYNJ1	5.47241224	5.14476033	5.39444152	5.56780972	5.74665054	5.86017138	6.01834222	5.66326549	5.63036426	4.91956762	5.46605147	5.37575057	5.85527024
IFNAR2	5.50035376	4.84742676	5.82400581	5.58781991	5.0395736	4.40155543	5.03816773	4.60737082	4.75241347	4.47860826	5.15791737	4.85577032	5.23249909
MRPL10	5.33774639	4.77119096	5.14829699	4.42150544	4.52325872	4.9518648	4.71522422	4.17355639	4.84498441	5.82036326	4.97793147	4.73538463	4.60594157
IFNGR2	6.61121794	6.23966666	5.62810784	7.11050967	5.44072868	4.57818731	5.66839089	5.26850979	5.62257921	5.73740733	5.42294826	5.75772283	5.64828158
GART	4.59518781	4.4449521	5.22688585	5.81810317	4.54145195	4.74746884	4.67139978	4.48532325	4.92124022	3.70344246	4.14046887	4.93530268	4.60978113
SON	6.37428328	6.95207657	6.19699473	6.60064396	6.6267906	6.28662502	6.67184059	6.66577437	6.45644905	6.67272756	6.44015922	6.50756752	6.60637892
DONSON	2.96945037	3.31321373	3.43199794	4.52059233	4.05144792	3.70440421	4.34251762	3.98709654	3.78754716	4.04614851	3.77565317	3.81025132	3.90020397
SV2A	5.655878	4.74746627	5.45174825	3.98717895	4.95266527	5.46995807	5.2239699	5.61409535	4.79512172	5.69949108	5.50283809	4.69528836	4.87397972
LAD1	0.12113022	0.19119642	0.10024494	0.13934015	0.59932572	0.2261735	0.20711734	0.28967271	-0.11445465	-0.11445465	3.6424949	-0.11445465	-0.11445465
STC1	2.61190984	4.0954372	4.77512258	6.08210734	7.16850637	3.93780034	3.36972057	3.04688096	5.52821071	3.75098307	5.63464024	4.72909229	5.15979751
TNNI1	0.09834168	0.07626881	0.07885345	0.07626881	0.12992502	0.41437783	0.10726374	0.07956621	-0.13701024	-0.13701024	0.95251644	-0.13701024	2.93690593
CSRP1	7.69267965	8.21172481	7.15430052	6.73976524	9.08263744	8.6341874	7.8377104	8.77843099	7.99091028	8.96143286	8.41508717	8.9169716	8.64959224
C1QC	6.90478897	6.65199223	5.95583438	6.91367908	5.25263921	4.84677813	5.6955427	4.4910395	5.82329685	6.43404331	5.22190215	5.21741495	5.16222199

KCNE2	3.39495932	0.96340106	2.48835146	1.36788659	2.16613005	3.33673839	2.60829764	1.76070612	2.79210412	3.16545627	-0.89705986	-0.89705986	1.21540326
ATP5G1	3.70095862	3.5339425	4.86899859	3.84336252	3.10479798	3.86636748	3.51715177	3.52861115	3.01214732	4.0774981	4.08275739	3.74302562	3.31500369
RCAN1	5.51290147	5.64528165	5.28211418	6.35249273	6.25566998	6.78433693	5.85620757	5.86856279	5.90672613	5.96722895	6.02627791	6.87860296	6.44516397
UBE2Z	6.30123075	6.13695761	6.18398982	6.37105363	6.25809969	5.53280408	6.44991779	6.04848218	5.91433098	6.54502817	6.25752346	6.27829052	5.7497137
SNF8	5.61627477	5.10958198	6.21806738	5.39744345	5.49143186	4.89183958	5.36343527	5.28866305	5.14390133	5.42270011	5.27760518	5.51775198	5.15444733
CLIC6	4.1763333	2.17168992	2.04813919	2.1336031	3.01776676	2.47931276	2.65553537	3.89872353	3.33805693	-1.36922134	3.86522813	3.41101207	4.33355184
CCDC24	2.87945424	2.14712351	3.56838724	1.29139859	1.35524343	2.26834144	2.13687443	2.10176612	3.65829319	4.12610944	2.63633669	2.90291052	-0.73738453
RUNX1	6.4880431	6.57548929	6.38276299	7.54525877	5.72476266	4.7707322	5.59328135	4.85258606	6.20537643	4.0109478	6.39904465	5.45859262	6.18639912
IGF2BP1	0.15408915	0.36489097	0.36920749	0.16192572	0.15408915	0.2276769	0.15408915	0.15739334	-0.14420007	-0.14420007	0.913563	0.87792915	-0.14420007
CBR1	5.49380013	5.21902451	5.90352305	4.81473614	4.11898647	5.07131419	4.73294013	4.54780376	5.44769347	6.08592839	4.6882188	5.06620144	5.26651298
CBR3	3.63329093	3.47697193	4.96046772	2.97002996	3.22201299	3.20353385	3.00506009	3.62166946	4.06147546	4.55217191	4.47816645	3.21755116	2.84130995
ACTC1	4.40392582	5.34379222	4.77669771	2.55916144	9.03982654	6.79709387	7.01298366	5.33396856	5.59519474	7.1218383	5.47092877	7.33429514	10.3052493
MORC3	5.84080746	6.32962918	5.695283	6.6844063	6.84162566	6.82020472	6.73792334	6.67773504	6.63472872	6.72731131	6.01280183	6.32746904	6.49442689
CHAF1B	3.23700844	1.67347764	3.54276644	3.6293512	1.954898	2.27207835	2.09331714	2.46702936	2.85760208	-1.15646124	2.86276302	2.27647459	2.51508752
CLDN14	0.77734358	0.71996072	2.03657087	0.86417824	1.21486986	0.42513974	0.42375494	0.42693251	-0.19353394	-0.19353394	-0.19353394	-0.19353394	2.98449455
HLCS	5.37789809	4.9768962	4.67898806	4.6818753	5.02576082	5.46238773	4.99543062	5.33104962	4.56885087	5.49740778	4.76642178	4.93549673	5.11188092
SCUBE1	6.89862233	2.12444898	2.23857907	2.09753601	5.24669407	2.92507786	2.83052724	2.13555423	4.20308759	1.0974307	5.31917601	3.50164924	4.12013569
ARHGAP27	3.92392135	3.5980498	4.4844853	4.12231901	3.39480181	2.26172009	3.00532159	3.46902582	3.79083534	4.27691183	2.62790886	2.22198741	4.49289128
ADPGK	5.25055314	5.47125069	5.19234331	5.91249996	4.89681392	4.23101184	5.14876521	5.17197419	4.86926447	4.88679752	4.61739924	5.28443698	5.08566745
PTMS	6.7080657	6.59300315	7.84924571	6.59866	6.71454896	6.38978145	6.58821355	6.5797119	6.71904816	7.17337616	6.55163882	6.70884289	6.95766925
PADI4	0.60597513	0.29441612	0.44690979	0.42921095	1.00734721	0.4597204	0.35477066	0.60692292	-0.27921227	-0.27921227	-0.27921227	2.12870315	1.35262865
ADIPOR1	6.75923509	6.42675291	6.03778929	6.50140419	6.29488341	6.33055059	5.78013114	5.93786158	6.68595423	6.44176011	6.34757221	6.16924185	6.26907897
CYB5R1	5.84731897	5.40602155	5.51710366	4.6854266	5.30851217	5.68050905	5.6537116	5.37132916	5.36249581	6.02676295	5.54754562	5.54567306	6.26378533
PSMD4	6.65584437	6.06105275	6.73188215	6.2384868	5.72196159	6.22662442	6.16978251	5.75556954	6.07494493	6.51353349	5.60888957	6.10545221	6.131015
ATP13A2	4.87145778	4.26766122	5.51002018	4.46751779	4.01113904	3.16091972	3.99137618	4.02672065	3.71792926	4.36685546	4.65404768	5.02107031	2.34441413
PSMB4	6.93225783	6.49591838	6.42722116	6.47666409	6.19855369	6.83972674	5.89522226	6.14001653	6.32750869	7.01100849	6.18523735	6.70456864	6.53649426
BTG2	6.88749349	7.46906272	6.16689516	6.45416107	5.8935722	6.47234579	5.59534248	5.85037179	6.81194591	5.90993902	6.54489222	6.88358463	7.00915215
HK2	6.94251593	5.45894949	6.64397892	7.2856072	4.68256697	4.32170531	4.26092177	4.49082526	4.18221734	3.99069611	4.69777458	4.31335556	4.21086762
C1R	9.01766485	8.46719816	8.31168637	8.05447456	6.98070212	6.46331249	7.85272039	7.10279247	7.11661707	6.92429711	7.43515711	6.90952102	7.81313572
ALDH4A1	2.50594286	2.60581118	3.41726339	1.61256105	2.54454476	3.81474164	2.53699718	3.70344882	3.63905077	3.69034095	2.67929913	3.53205773	3.71677509
STARD9	1.6080349	1.46071373	0.88662593	-0.61743751	2.12252494	0.86722126	2.24726067	3.31060037	0.5787805	5.18521366	0.5787805	0.5787805	0.5787805
THEM4	4.55554931	3.95129247	3.91664485	4.70939887	4.75857552	3.67279803	4.44400491	4.61190979	4.41555658	4.40295568	3.66643384	4.42374219	3.96595432
TCHH	0.92238074	0.64072916	0.9745795	0.66292925	0.64072916	0.7554463	0.64072916	0.7505828	2.31308347	3.5765454	-0.40772244	0.76797696	-0.40772244
UBR1	5.62099699	6.24851415	5.58715927	6.41231334	6.48051251	6.58374402	6.44433971	6.35094855	6.26388898	5.74546236	6.53109225	6.28114994	6.22367276
AMFR	6.95641719	6.70229458	6.77282967	5.77893483	6.72777268	5.99215162	7.19191079	6.62717307	6.41104767	7.2967027	6.73508853	7.0718926	6.74033551
MED8	5.07699055	5.0199505	5.36433006	5.99787929	4.88961352	5.35249504	5.12804647	4.92045852	5.09290997	5.51558332	5.46255796	5.19096006	4.72445243
TGM7	-0.05174386	-0.05174386	-0.04933057	-0.05174386	0.31650546	0.40432372	-0.05174386	0.68128124	-0.01390496	-0.01390496	-0.01390496	-0.01390496	-0.01390496
RGL4	0.71504249	0.30411897	0.30671657	0.33526931	0.3276323	0.30556319	0.30411897	0.3074329	-0.11563272	-0.11563272	-0.11563272	-0.11563272	-0.11563272
RSPRY1	5.56067675	5.80447555	5.586868	6.61951854	6.21920247	6.04283517	6.09966978	6.19897837	6.04099048	5.73028986	5.64290773	5.69058274	5.79457461
CCDC17	3.84289333	2.73120289	3.49955884	2.60728417	2.71631727	1.64852598	2.19292447	2.92400159	2.83198793	2.95312062	2.80930167	2.73826769	2.88702339
GPBP1L1	6.16841207	6.58496768	5.97473124	6.35579322	6.57364036	6.65643616	6.52072337	6.69720669	6.66825868	6.12555361	6.62940039	6.50035576	6.13833734
NAE1	4.7647118	4.75213279	4.79339286	5.73374712	5.5079082	5.5281461	5.74653075	5.73374712	5.29611784	4.63862765	5.09547948	5.70433134	4.44607007
TMEM69	4.38544445	3.26890684	3.60270674	4.4903527	4.07493864	4.69949183	4.16081521	4.3323141	3.87033308	4.20663162	3.97423372	4.46035857	4.63635785
ACE	2.19203153	3.17371676	3.71702196	3.69879953	2.26175636	0.30647688	2.10635791	1.98197126	3.72841382	3.28118965	3.52218899	4.06244763	4.44207259
TEPP	0.18263924	0.05531932	-0.06713686	-0.29706203	-0.00779647	0.49224208	-0.24389443	-0.01185072	1.02595801	-0.16097962	-0.16097962	-0.16097962	-0.16097962
UROC1	0.05568444	0	0.18573635	0	0	0.00069205	0.07737624	0.00158797	0	0	0	0	0
SPON2	0.14599197	2.35900835	0.07321086	3.06019195	1.05914647	0.08813029	0.00739886	0.61731102	0.30535544	2.84253955	-0.16023516	-0.16023516	3.50587506

CHCHD6	3.85097873	2.90659723	3.41161942	1.65081303	2.07950241	3.03878686	2.28825511	2.24758135	2.99615414	3.9892188	1.7450391	3.59938015	2.40612637
CTBP1	5.74438484	6.20961228	6.20234461	5.3752081	5.58567992	4.70175696	5.83782366	5.46470867	5.55119374	6.27542578	5.94149326	6.14491873	5.48024636
LRRC36	-0.01875072	-0.01875072	-0.0162657	0.0019961	0.19928813	0.13294061	0.11635828	0.57659671	-0.02100512	-0.02100512	-0.02100512	2.44727223	-0.02100512
TPPP3	2.73012286	5.73740884	1.99248651	3.7964788	5.20401144	3.73954498	2.27347591	3.3487377	4.14198194	2.2645246	4.66736584	3.80359407	5.17383096
ZDHHC1	4.78519764	3.5361065	4.67670054	2.90415954	3.60871171	2.9760906	3.34312365	3.56112499	3.42227308	4.19933031	3.69219174	3.60775846	1.55330167
ATP6V0D1	5.7753139	5.18540706	5.85718043	5.54341737	5.00339038	4.85414725	4.78930761	4.57499471	5.13270593	5.53692494	5.44772262	5.48168968	4.84816428
ZFYVE28	3.60703728	2.81457656	3.86423671	2.29585875	2.81355272	2.21920271	2.99833836	3.03429286	2.60475955	3.53690386	3.48330274	2.77176687	1.11515151
C16orf86	2.37153711	1.85782902	1.62539246	0.38580478	0.88083626	1.83182201	1.01200616	0.95427348	3.20039237	-0.50396393	3.38533478	2.39543524	3.49693437
FAM131B	3.45219867	2.36818305	2.04839883	2.05661682	2.98821632	3.24985397	3.36316129	5.03024827	-0.90588114	4.57810615	2.83787487	4.33989345	2.18846076
RGS12	2.21414369	0.89776207	2.21170551	0.56593916	1.00291008	-0.12681115	0.81880854	1.28817661	0.30213734	3.41342785	0.30213734	0.30213734	0.30213734
PSKH1	5.06737311	5.03093865	5.45259871	4.18350849	5.20551743	4.37670535	5.05964983	4.63022802	4.65269301	4.38753418	4.56491632	5.39435189	4.82317385
ZYX	5.95695628	5.18425377	6.8728484	5.32121936	6.4239018	5.5762618	5.76597643	6.48535224	5.81584033	7.00136924	5.9250772	5.77894164	5.82669402
ABR	6.73256615	6.75009497	6.85662348	5.926062	6.93651784	6.56151847	7.06282589	6.62260784	6.45658952	7.45989264	7.11510144	6.82693612	6.85382198
LYPD5	1.34440699	1.89446154	1.20368772	1.54947304	2.10092187	0.83639953	1.47078911	1.20460061	2.6706038	-0.54260071	3.79700823	1.31605049	2.80780612
CCDC117	4.50080878	5.12559225	5.22111297	5.70307569	6.38428936	6.06081974	6.1738194	6.15799844	5.58731133	4.81336001	5.61415471	5.67663361	5.33627786
ZNF230	3.9140629	4.12644419	3.71041157	4.13659059	4.80349135	5.07466949	4.20198421	3.64053397	4.80641946	4.59798177	3.8332738	4.61811328	4.10629096
CCDC107	5.63196993	5.21240406	5.82890852	4.28129314	5.44209077	6.00684576	5.1564764	5.25029523	5.45539742	6.06543061	5.26752743	5.74126795	5.17462569
ZNF222	3.20838937	3.83365449	2.96403951	3.59301241	4.39016304	4.26878234	3.23251237	3.73378391	3.487656	3.48713031	3.35542227	4.13214176	3.06973121
NPR2	6.54972974	5.86040989	6.22404764	5.27861116	5.94032568	6.13731544	6.39598578	6.32035799	5.65888565	6.40192338	6.10632533	6.23271349	5.96616059
ZNF221	4.06554696	3.56019644	4.25338075	3.6510347	4.61100602	4.83776173	4.48937574	4.0854949	4.20537516	4.46761861	2.89199249	3.1161465	3.97910947
ZNF233	1.18521624	1.62901566	1.28894635	1.62553522	2.28755021	2.25282281	1.38663006	1.27989692	3.02520135	-0.81016114	-0.81016114	1.87514295	4.5035318
ZNF235	3.7234934	4.52476085	3.86544236	3.89229108	4.69416124	4.90119152	4.18905294	4.33351743	4.46559104	4.10262307	4.46761253	4.18800873	4.08082286
GNE	5.70902938	5.25746187	5.30162699	5.20426329	5.83078888	5.78767599	5.82985925	5.71503014	5.05709148	5.23858048	5.40456438	5.6518661	5.49328485
TNFRSF13C	0.0978796	0.13425145	0.22381154	0.16755424	0	0.00069205	0.01850152	0.07443438	0	0	0	0	0
ARHGAP35	6.75122618	6.96712211	6.67834264	5.88487041	6.88336612	6.83559718	7.07181216	6.75482852	6.52825745	7.23080309	6.71538566	6.60258328	7.16329266
PTGIR	6.49866207	4.85895214	6.56771905	4.59697366	4.83413742	4.66865467	4.94849882	5.55197679	4.54888315	5.44182295	5.05878921	5.56657994	4.43815172
CALM3	7.68218379	7.85685893	8.24964421	7.68218379	7.39317576	7.57911229	7.70766394	6.94889503	7.3642834	8.12430797	7.71330525	7.88448901	7.61974826
DFFA	5.86918659	5.23619167	5.79652649	4.94686241	5.17307856	5.63702395	5.52136664	4.9750351	5.10773372	5.51222017	5.32039325	4.9591222	5.22078797
CCDC28B	2.70192991	1.37842458	2.95766505	1.82116445	1.71317015	2.27811019	2.70192991	2.79671462	3.07927031	2.60269006	2.71922934	2.2139854	2.58348773
IQCC	2.81198458	2.23128616	2.81036838	2.06872703	1.98045865	2.56691402	1.89820649	2.61863102	3.50979317	3.93256675	2.76210873	1.11088602	1.09721964
TMEM234	4.71002439	4.01460473	4.45940449	3.78115425	4.33899239	3.15074345	4.13422677	4.38701626	3.64976876	4.16328461	3.89203297	3.47503202	3.72330179
BSDC1	5.99612375	5.83951772	5.78859431	5.3888509	5.98024189	5.11859331	5.9811899	5.86240641	5.77709101	6.19315779	5.53535289	6.25150168	5.93419321
ZBTB8A	4.40249507	4.62114274	5.06093229	4.65279339	5.04793794	5.11444593	5.15104582	5.14849789	5.06199244	4.46489341	4.87650511	5.27006027	4.17546608
ATAD3B	3.4335043	3.60006022	4.48781533	3.58840794	3.74965283	2.37764833	3.97731201	3.35194959	3.90309502	3.65548332	4.17698917	3.90711409	2.95044047
SSU72	6.03098928	5.89891988	6.1746721	5.43356058	5.63382086	5.48017611	5.40780718	5.62047331	5.58624863	5.75757236	5.27801332	5.46963162	5.69065857
UBE2J2	4.78559562	4.7873117	5.01629924	4.35289392	4.29210571	3.50159026	4.9473176	4.37732904	3.68439242	5.61429444	4.49326316	4.95873915	4.03471961
ZNF362	6.11713333	5.54436959	6.42202617	4.90196493	6.11934804	5.88608116	5.61944882	6.32976313	5.80637189	6.71811395	6.25011309	6.52404324	5.7445469
FNDC5	2.58513574	3.21944022	2.9707546	1.95596129	2.77617444	5.60082252	3.0683138	5.51429547	4.07770571	4.33250552	4.21985234	4.53339843	5.81586623
CPAMD8	4.44276957	1.26679005	2.77164811	1.59216869	2.93145053	3.3798103	3.69959043	7.61835497	4.56475993	4.36691834	2.92111513	3.46836113	5.74797608
NR2F6	4.34617268	4.56576183	4.88096433	4.19187	3.64224407	3.87235522	4.05681053	3.36887155	3.34550982	5.16294546	4.1907128	5.1072372	3.12307803
ANKLE1	0.47132811	0.26593077	0.92730964	0.87779393	0.77084035	-0.00662465	0.63240001	1.36730478	0.22264211	-0.2335263	-0.2335263	-0.2335263	-0.2335263
CCDC58	2.60669893	2.56216862	2.8394013	3.55825694	3.72609684	2.09986387	3.06187113	3.84970537	2.14805243	2.41362406	2.39184582	3.47632323	-0.14085248
VMA21	5.50748915	6.05667876	5.42535934	5.97760029	6.34287728	6.24468874	6.26551104	5.88746952	6.01187214	5.92827131	6.44183728	6.108466	5.70862087
KALRN	4.90356314	5.17221672	4.9899643	3.74008562	5.6171511	5.31536073	4.55941411	5.62041136	4.83386798	5.11819602	5.48287753	5.13167125	4.68660883
CILP2	0.27694052	0.33934568	0.17306317	1.85277598	-0.11061807	-0.02874554	0.01707437	0.15776698	-0.01010464	-0.01010464	-0.01010464	-0.01010464	-0.01010464
ABCG1	2.68061293	0.92441859	0.39793745	1.70042614	1.14030365	-0.73592639	-0.07066421	0.95440686	0.27434135	4.19740786	0.27434135	1.98158188	0.27434135
TFF3	0.15657418	0.93679774	0.15910257	0.81476149	1.20433479	0.65597742	0.15657418	0.15979981	-0.07948521	-0.07948521	-0.07948521	-0.07948521	-0.07948521

TMPRSS3	0.11376361	0.77351541	0.21171912	0.30719218	0.2503663	0.21152018	0.09305434	0.09630157	-0.12502859	-0.12502859	-0.12502859	-0.12502859	-0.12502859
UBASH3A	2.15599285	0.76539676	0.66115888	1.47227751	1.0713066	0.44078545	1.15851943	0.90638396	-0.34222376	-0.34222376	4.21561095	-0.34222376	-0.34222376
SLC37A1	4.33806074	3.79945749	3.76898907	3.4121609	3.76223618	2.86568023	3.39289162	3.76836141	3.21445412	3.68285241	4.13360422	3.77591744	3.90166728
PDE9A	0.53260168	0.82356324	-0.03455525	0.367622	1.14504784	0.89266918	1.39687729	1.7063513	-0.19650559	2.80346275	-0.19650559	-0.19650559	0.84257746
WDR4	3.63828354	3.96164457	4.69001241	4.16014683	3.75237011	2.40881304	3.7204173	2.64149875	2.87400505	2.10308672	3.9399739	3.62846672	2.60899405
NDUFV3	5.61825125	5.11331374	5.72287649	5.08270265	5.13468244	5.01799098	5.61947106	4.8932188	5.78023939	5.22480433	4.94707865	5.49366364	4.76960986
PKNOX1	4.84663738	4.88729419	4.63260316	3.99424732	4.91375526	4.68746419	4.73616466	4.60859551	4.51417811	4.86120864	4.40160659	4.82082961	4.02066827
CBS	3.41897884	0.5612482	1.64108151	0.92820135	2.78155012	2.86197206	2.81609978	3.19078512	2.34641894	2.54838921	3.23369669	0.63995605	0.1191389
U2AF1	3.28516885	2.36212743	2.86684027	3.50340384	3.35549572	2.53014665	2.79624008	3.38633413	2.90716666	2.69570662	1.44676404	2.95209808	4.12227264
HSF2BP	0.66859307	0.17223223	0.82989744	0.53337968	0.30641153	-0.02496793	0.14185146	-0.0781136	2.7603173	-0.0996785	-0.0996785	-0.0996785	-0.0996785
RRP1B	5.76224042	5.84728166	6.12585001	5.46635707	5.29231709	5.53196813	5.02099591	4.7808595	5.24236663	4.91075767	5.56478131	5.14731281	5.62648064
PDXK	5.9719779	6.15766335	6.44325453	5.93632029	5.3763484	5.1270054	5.82838137	5.1022501	5.06622737	5.85464579	5.79448964	5.9130821	5.03785266
G6PD	5.49211642	4.82408202	5.78048368	4.78410206	4.59308314	4.84482289	4.88995969	4.58486421	5.06161476	5.59808802	5.14418683	4.31017331	4.89296396
CSTB	4.62401531	4.08796403	4.61004775	3.41323697	3.38065899	3.73108887	3.31872342	3.19525066	4.10449437	3.84336783	3.19739019	3.43362109	2.9047808
RRP1	4.58366855	3.9821838	5.50474267	3.76193559	3.5817896	3.56619211	3.82271141	3.46901516	4.13502337	4.47581251	3.33026318	3.84082284	3.45123303
AGPAT3	5.61449701	5.40123317	5.69966111	5.48816823	5.27894299	4.66294133	5.44226309	5.26764262	4.73973889	5.53478919	4.98955011	5.60302352	5.96185404
TRAPPC10	4.17982902	4.43031301	4.10987997	4.34758205	4.13991154	3.31493649	3.81400071	3.62512931	3.82830901	4.44645151	3.51147628	4.29442898	2.83140376
GAB3	4.85698477	4.56068393	4.22054793	5.04300432	4.38426988	4.76126173	4.81433151	4.5679944	4.62118586	4.58209018	4.59111968	4.37883973	5.50199704
C21orf33	3.90160523	4.24513576	4.47515173	2.28626545	3.52676134	3.78806442	2.90193996	2.97349683	4.22049364	1.67502174	4.19185008	3.60373435	4.27136081
ICOSLG	0.957615	1.05221511	2.20699043	0.91231018	1.84062424	0.6558231	1.14868434	1.02173538	0.15269845	3.14773301	-0.5302528	3.27937485	-0.5302528
AIRE	0.01008624	0.31142894	0.16941055	0.07937581	0.01008624	0.01152645	0.01008624	0.01339094	-0.12523112	3.37873158	-0.12523112	-0.12523112	-0.12523112
C21orf2	3.62169998	4.11069232	4.56275159	2.38693865	3.94211385	3.17493747	3.25331593	3.450189	4.20591737	3.81379843	4.16294353	4.77150273	1.99422092
LRRC3	3.83298099	3.28720344	3.85815547	2.17122476	3.91685235	3.33516094	3.5312234	4.12072507	3.40582685	4.67119038	4.11826504	4.69928824	2.57381261
ITGB2	5.9912811	5.67007832	6.30027819	6.33298848	4.50639843	4.03406178	4.81629741	4.27359053	5.51358554	5.13640957	4.45620339	3.91254083	5.7996947
FAM207A	3.46647135	1.431672	4.96848786	2.11039471	2.7999298	3.59277191	2.39791035	3.03817415	3.72758962	3.55469948	2.28550669	3.90208654	0.87111605
RALGDS	5.84745431	4.91162279	5.44219915	4.80270413	4.75733484	4.14599377	4.67977951	4.75216504	4.59414327	6.006437	5.36802618	5.30415469	4.33575628
FTCD	0.75870632	-0.28576076	1.06975084	0.15115575	-0.2083835	0.20071395	1.36625154	-0.02169552	0.16268462	0.16268462	0.16268462	0.16268462	0.16268462
LSS	5.87299002	5.84494657	6.24776455	4.67467242	5.66018142	5.46388021	5.85360543	6.30187007	5.79063022	6.39659125	6.17964455	6.29094566	5.2717595
VAV2	5.37068765	4.74782567	5.86073714	5.11587028	4.57757369	4.07432085	4.86133311	4.67136321	4.23900648	3.99873123	4.44679275	4.51040087	5.26287062
MCM3AP	6.14722127	6.00417534	6.27268148	6.08983491	6.06014542	5.37699185	6.22295414	6.12835598	5.63702691	6.42952843	6.30001377	6.223695	6.41544047
C21orf58	2.23179122	1.12127163	3.9992396	2.62843476	1.43826577	1.20477319	1.69816968	1.52007485	2.87267632	2.55848634	1.05991926	2.00145862	-0.86172435
PCNT	5.77205099	5.25098652	6.3059157	5.10538082	5.25611703	5.12816769	5.55805672	5.37500732	5.52075036	5.70163452	5.42143254	5.49592478	6.01702135
DIP2A	2.59722278	2.40352629	2.73513569	1.92268176	1.93081023	0.7215039	2.65922546	2.56054047	0.59327606	3.4448094	0.02356	3.66572366	4.3080477
S100B	3.10473417	2.17637831	2.69937965	0.87376718	2.08766988	3.84314125	2.56719832	2.99866025	3.69787207	2.93712037	4.04287412	0.09916065	2.61635268
PRMT2	6.34974363	6.40091569	6.00706891	5.48595953	6.15878582	5.92878358	6.1464529	6.14076123	6.28608647	6.41737735	6.19598316	6.13117203	6.25265617
CLDND2	0.56483017	0.66090783	1.58018231	-0.19206942	0.80883682	0.54729709	0.39638703	1.29696998	-0.21755094	-0.21755094	3.41284509	-0.21755094	1.61011325
ZNF208	4.21792716	5.48044058	4.45559231	4.43026854	5.46320438	6.25437123	5.04162643	5.21702388	5.12066974	4.04990586	4.74170234	5.07845469	4.6418056
ADAMTS13	2.36865522	0.91184889	2.91765451	1.89731004	1.50163197	1.65517468	2.58439716	2.83656832	2.70871948	3.33615477	-0.76210377	2.95040717	0.98537603
SLC2A6	1.78285085	1.24746013	2.88235185	1.18278744	1.43354125	1.08098258	1.554724	0.67564783	3.04636214	-0.41994281	-0.41994281	1.38738072	-0.41994281
ZNF761	3.78432689	5.0037427	4.13084952	5.14131506	4.78613737	4.68333958	4.5637672	4.81404515	5.00816956	4.04241838	4.21541161	4.44536379	3.78139819
C9orf116	2.0237228	0.45163712	1.59206434	0.44876127	0.51202561	1.3128354	0.74223826	1.13148286	0.31720568	-0.40407837	-0.40407837	-0.40407837	2.78951608
ZNF714	3.2658	3.34269354	4.06331453	4.48348976	3.63458319	3.73070739	3.50527242	3.49641667	3.37937952	3.27903308	4.16936337	4.00991023	3.85325318
GPSM1	5.03501621	4.01835617	5.37901079	4.21966042	4.48415417	3.18499281	4.48696407	4.46064843	4.5180234	4.11405916	4.98755594	4.88693589	4.47290742
C19orf47	4.54776063	4.35310051	5.01788809	3.13517571	4.50232929	4.22131619	4.06424088	4.47206664	4.43930518	4.74985592	4.54394036	5.49892958	2.99372633
HIPK4	0.50698187	-0.06861341	0.6484554	0.10873841	0.14609795	0.0796971	0.52932815	0.91383633	-0.06627618	-0.06627618	-0.06627618	-0.06627618	-0.06627618
TOR2A	3.149377	3.18668622	3.56353453	1.98415919	1.49448072	1.39997403	1.99073016	2.13918237	2.68903136	3.31613345	2.79521421	2.99157145	-0.97374176
ST6GALNAC	6.39665537	6.7620018	6.18605341	5.06764019	6.02619159	5.67096059	6.06178569	5.47763791	5.75445821	6.49785244	6.41018632	6.09241252	5.00086113

SHKBP1	5.42988	5.09231247	5.4868046	4.77045971	4.57463671	3.73523037	4.88037177	4.64181478	4.03027092	5.37722418	4.80094988	5.33326972	4.95406688
RDH13	3.95424032	3.73278317	3.36104335	2.52815839	2.79821004	2.53924515	3.78690624	3.38110758	3.50380774	2.86560329	3.70529044	3.64093787	3.83388182
ZER1	5.98197806	5.57090908	5.7894451	4.66250482	5.43551071	5.18454943	5.5196633	5.51295212	5.40857012	5.86131685	5.85589401	5.91784036	5.67934359
ZDHHC12	2.00608778	2.00452335	3.01816203	1.10282231	1.58535292	2.22158926	0.73697921	2.2119364	1.58895916	3.64593911	2.39377824	1.55267465	-0.54913372
PKN3	1.69923592	2.11404335	2.61352939	2.39218663	3.16239215	1.69041403	2.25631632	3.28676183	2.97613894	3.02281657	3.96610794	3.09361719	1.30580283
SPTBN4	3.21157508	2.56587639	3.1049128	1.99434167	2.27726464	2.38273847	2.2755523	2.6121513	3.39660247	2.15127505	3.31176153	3.92878033	-1.10410215
BRSK1	4.96057522	3.77549465	4.80153652	3.28881377	4.10112189	3.89120271	4.06248476	4.67167876	3.77909612	4.39265476	4.15023631	4.34517999	3.85413765
COX6B2	0.00528772	0.36057925	-0.0258457	-0.00566367	-0.02818695	-0.02688525	-0.02818695	0.35758076	0.02687809	0.02687809	0.02687809	0.02687809	0.02687809
TAOK1	6.78279402	7.37371402	6.79868596	7.71616108	7.68848879	7.87070383	7.59284301	7.66191884	7.59243469	6.9964891	7.3884689	7.23631453	7.31180756
MED27	5.1953542	4.35948869	5.03540487	5.14151194	5.24967655	5.35615029	4.75961979	4.80020107	4.89530726	4.49114104	4.63527123	5.07164135	5.19757406
DEDD2	5.22199421	4.51161582	4.66105222	3.4578428	3.90032498	3.76031242	3.93477175	4.27398788	4.35025101	4.89793074	3.15789783	4.80008316	4.25550716
SIK3	6.49946741	6.2180892	5.87458237	5.712258	5.89948964	5.51285989	6.01355933	6.04605369	5.67167455	5.60632222	6.06340099	5.88906325	5.99043901
MPZL3	1.73098987	1.19225606	1.21101372	2.18269331	2.01955136	1.55028215	1.30585172	1.29027965	3.52996335	-0.69769081	3.04423939	-0.69769081	3.76456297
NEK8	3.36240015	1.88212627	2.95451561	2.08161161	1.56981668	2.52976606	1.97616699	1.58648953	3.3384667	3.08224083	0.80944138	3.04552942	-0.9206296
TLCD1	1.55087312	0.08224591	0.90144344	2.13612536	0.70048203	0.5965386	0.6853004	0.30171997	1.28867555	3.12631666	-0.47587345	0.95964114	-0.47587345
PCSK7	5.11066115	4.75853218	5.14903323	5.27164873	4.81512184	3.70210641	4.90868631	4.57850525	4.7086212	4.79410594	5.04177462	5.04506307	4.20446366
SAFB	6.01456282	4.9830629	6.29376259	5.38313885	5.43235793	5.46586233	5.66518291	5.49354601	5.84786452	5.90001949	4.92894093	4.73965782	6.10999006
CD3G	2.36232931	2.21381902	1.02255182	1.61405358	1.20291134	1.22724482	1.68017327	1.42318155	-0.63791867	-0.63791867	2.11483979	-0.63791867	-0.63791867
S100A1	1.73428958	1.4094184	1.85345921	1.45581435	1.61386368	2.4878797	1.60155173	1.68772046	-0.23173423	4.02012761	1.05163409	1.58679535	7.66328097
CHTOP	5.51612679	5.90421337	5.32648758	6.15918221	6.14200638	5.59951609	6.05507507	6.07154065	5.58455084	6.46112426	5.73522612	5.79633739	5.75961523
CXCR5	0.02984887	0.09941896	0.07456801	0.09889575	0	0.00069205	0	0.00158797	0	0	0	0	0
ZBTB7B	5.79541207	5.62103344	6.1078388	4.84954046	5.00421234	4.82743244	5.09262602	4.82982226	4.67243192	5.2506207	5.2129284	4.89107844	4.55449678
FLAD1	4.03052946	4.0138794	4.45415115	4.38031973	3.51885691	3.45225864	3.4295229	3.57242901	2.99721826	3.93612525	4.01805345	4.61566597	3.63509202
SHC1	7.40274503	6.05191628	6.82723876	6.9224382	5.81755494	5.53950517	5.84140889	5.78243444	5.80358949	6.01773044	6.0867655	5.7040985	5.88728624
VPS11	6.56052918	5.64810654	5.86551716	5.19483823	5.4423151	5.97593981	5.60412183	5.44378611	5.25855192	5.85976275	5.42491155	5.75111508	6.2049824
NLRX1	4.40925639	3.86606478	4.52920891	3.15719438	3.93205009	3.55711944	3.18289944	3.95946076	4.18039924	3.83177585	4.07040035	4.7700062	3.78126342
ADAR	7.52563142	7.64741632	7.12560767	7.48593941	7.25081433	7.05145753	7.30953006	6.79959092	7.19968847	7.1446039	7.40631461	7.03363664	7.79100271
IL6R	1.76801994	1.5580269	1.58435796	1.35430526	0.21983761	-0.37002522	-0.24673394	-0.13530383	0.13497664	0.13497664	0.13497664	0.13497664	0.13497664
UBE2Q1	6.18602501	5.84519897	5.53353749	5.16780774	5.77240107	5.70706469	5.5872708	5.19936464	6.05523517	5.42256825	5.32217762	5.06957364	5.92794573
CHRNB2	0	0.0725262	0.00124472	0	0.02947685	0.00069205	0	0.00158797	0	0	0	0	0
CRTC2	5.90753257	4.98164542	5.77545635	4.32498702	5.09297366	4.61546352	5.02232779	4.99259645	5.00695634	5.05061161	4.16310024	4.61714003	4.54095647
ANO10	3.9090189	4.25129388	4.36665009	4.37261299	4.32098432	4.90292787	4.71167094	4.79781224	4.64808454	5.00606075	4.76113401	4.83999877	4.5039938
FDPS	5.01363858	4.84501593	5.48149828	5.11383133	4.81718801	4.86945354	5.60105804	5.00762223	5.69653396	5.26222664	5.49376328	4.64530957	5.39029127
RUSC1	5.0685114	4.90643493	5.51902029	4.01546974	5.15899078	4.498727	5.47106847	5.57198333	4.88857821	6.03602526	5.57675552	5.60429926	5.39247255
FAM189B	5.09826756	3.66975899	5.6150409	3.99663608	3.99424708	3.81021186	4.80112048	3.51264725	2.68753701	4.44524669	4.85916065	4.20974571	4.259945
PAQR6	2.09440507	2.13939542	1.37887379	2.07411229	0.97032415	0.40506591	1.36692038	0.65876021	2.5791252	3.84800208	-0.53742646	-0.53742646	-0.53742646
PMF1	4.91834713	4.03441991	4.14564559	3.64892536	3.93653889	1.93374317	3.83372083	3.67003814	4.08083581	4.60291097	3.63820566	3.96905849	2.77386079
SLC25A44	5.79849295	5.1923779	5.62659816	5.61653629	5.08648248	4.91606385	5.34200431	5.12399007	4.65876151	5.37769304	4.83541334	4.35794994	5.22855354
LMNA	6.79995152	7.26431319	8.39359979	7.1764199	7.14060351	7.04064707	7.41682741	7.55731166	7.5283107	8.11184202	8.07382366	7.91083796	7.52477989
CCR5	3.72092531	3.93136881	2.98754473	4.53823422	2.40495553	1.94162973	2.56805847	2.52860067	4.54788233	1.78698024	4.03682986	2.50938189	1.90891135
NBEAL2	5.14292463	4.68240965	4.91379836	4.24395851	4.46677781	3.45413059	4.48290157	4.57768692	4.73036958	4.96612785	5.10291522	5.28181004	4.33818993
CCDC12	4.25248091	4.33864935	5.08244442	4.02152884	3.78010474	3.74356618	3.94273635	4.07848031	4.25367575	5.25467836	3.95521059	4.04032106	4.27183422
PTH1R	4.79304831	2.73258571	2.99815266	1.99870428	3.19564486	3.36737834	3.47141509	2.58030375	1.96257442	3.85868379	2.21483778	1.57654308	1.21679367
UBQLN4	6.14231313	5.38935289	6.16433858	5.38211065	5.59293479	5.64411606	5.74053586	5.24895084	4.84116594	5.99786723	5.01767878	5.50421984	5.67624962
MYL3	3.7766623	0.35125453	0.68505733	1.30861469	2.30397262	1.69409905	1.14448748	2.42874534	0.98008036	-0.53883549	-0.53883549	3.36041813	6.28970746
PPP1R35	4.11012997	3.20730251	4.85067291	2.44213715	2.69793393	3.26293324	2.33479625	2.86015213	3.31010576	4.2439363	4.24254415	3.41078533	2.51697935
GPATCH4	4.29400812	4.08781197	5.4290413	4.55028074	3.74200882	3.48820886	3.29128753	2.64424047	3.60318439	4.22744573	4.24458147	3.30937972	4.20536185

LRRC71	-0.21424128	-0.32836343	0.08473931	0.53614923	0.16133964	0.2682602	0.27478616	-0.013731	-0.01978527	-0.01978527	-0.01978527	-0.01978527	-0.01978527
FCRL3	0.40339231	0.41013459	0.17650691	0.39462277	0.48456096	0.175356	0.38706129	0.31050795	-0.07292273	-0.07292273	2.41065625	-0.07292273	-0.07292273
AZGP1	0.4829776	1.05460862	0.48549984	0.4829776	0.65074413	2.02857455	0.4829776	0.48619539	0.14484605	-0.313455	1.80452025	0.70885849	5.85441782
FGFR4	0.06158146	-0.02658472	0.39690401	0.33970739	-0.15135665	-0.13158271	-0.1482883	-0.24776064	-0.16899067	-0.16899067	-0.16899067	-0.16899067	-0.16899067
NACC1	4.60443434	4.11364196	5.49390678	4.87281491	4.09823588	4.35391321	3.69950404	3.6600554	3.91327277	3.67198682	4.45549448	5.1283572	3.4683074
HK3	3.74234422	3.13981234	5.41333339	4.10789925	1.49710416	1.07183242	2.47156559	0.64846619	2.19872034	-1.26487597	1.91250127	0.90431051	2.21149584
LY6K	1.83297573	0.98683273	1.46858422	1.2152868	1.13090128	1.6916064	0.98453544	0.80345568	0.69898085	-0.51262646	1.93867467	-0.51262646	-0.51262646
IER2	5.78973549	5.61615066	5.98283648	5.31982234	4.48978874	4.38815509	4.65128665	4.32681254	5.08138453	5.66831076	5.54334392	5.71082422	5.53900649
ZNF394	5.87592728	5.33368141	5.49469332	4.7890701	5.13655368	5.47858346	5.29592323	5.57300851	5.58945081	6.09583115	5.32709414	5.46858846	5.78997078
CPSF4	3.94699632	3.62644411	4.06916927	2.99565207	3.10319241	2.03285992	3.12471891	3.76519137	3.97155148	4.52169862	4.10284363	3.46256837	3.00896864
LY6E	6.04380682	6.63610484	6.38662273	6.72792041	5.46837562	4.90828617	5.24096388	4.98726513	5.68564173	6.17604599	6.64046374	6.02336139	5.26853421
VPS28	5.73225571	5.24163214	6.01698195	4.55604653	4.99179469	5.2588011	4.73662627	5.15459811	5.38681523	5.93925603	5.04536341	5.50734192	4.65803911
TONSL	0.54966935	0.62253065	2.13218714	0.71063291	0.1358305	0.11583392	1.05597475	-0.09852201	1.70017136	-0.20774338	-0.20774338	0.82089225	-0.20774338
PTGER1	-0.17323404	-0.17323404	0.49538195	0.07738518	-0.17323404	-0.01165841	0.27840087	-0.03238563	-0.00761463	-0.00761463	-0.00761463	-0.00761463	-0.00761463
MUM1	5.81418055	5.67199526	6.02411021	5.07320361	5.76112173	5.2849375	6.2744623	6.23551851	5.60365398	5.99678234	6.66737593	5.89514778	5.86767684
RECQL4	1.2310955	0.85712043	3.3553705	2.06550926	0.13132984	0.5283383	1.41933889	0.49381205	-0.54517974	-0.54517974	-0.54517974	2.15278435	-0.54517974
LRRC14	4.30678561	4.61199235	5.08529532	3.02339493	4.08332432	3.63371771	4.34121797	3.73220878	3.4499789	4.72502089	4.87940475	4.50884213	3.57095685
ZNF333	5.14712136	5.06710525	4.88158262	4.75834413	5.3884301	4.97924858	5.04879056	5.61097186	5.12879274	5.32092711	5.1367365	5.19924157	5.5038107
PPP1R16A	3.94269822	3.57423005	4.42229547	2.62452543	2.82670925	2.80378091	2.84394164	3.16333127	2.69625746	4.28464329	2.74071922	2.86246653	2.48421863
ORAI2	4.64474437	4.33118406	5.05270369	4.93198209	3.93789376	3.05167912	3.68564284	3.69516341	4.15146561	2.45937615	5.13372654	4.40968135	4.50313304
ALKBH4	3.99429672	3.81189994	4.52044777	2.88218146	2.75258604	2.90613395	3.0904095	3.32421772	4.00692056	4.04546161	3.97298098	3.94569976	3.86779671
SH2B2	4.08537114	2.212758	4.21257307	1.49547256	1.91278042	1.57109474	3.30383897	2.44412571	2.46359939	3.59954601	2.87626638	2.3103025	1.04858529
SQSTM1	8.19811565	7.50298552	8.16025404	7.06430639	7.00030978	7.83580206	7.56195789	7.22040196	7.31436227	7.86256242	7.53443611	7.45934806	7.35378399
RPL8	7.72554059	7.77647971	7.41250559	7.3908808	7.03599803	7.02859966	6.64898772	6.52843978	7.46039807	7.96081365	7.54203796	7.79184762	6.54716452
MAML1	6.38719925	5.63793397	5.81147763	5.44436404	5.7468294	5.21719587	5.53047154	5.69347162	5.56298854	5.83772536	5.64008959	5.36952934	5.5435751
LRWD1	1.57392578	0.43013929	2.53257041	0.03284717	0.29807706	0.76610986	0.19313592	0.50762422	-0.0516081	-0.0516081	-0.0516081	-0.0516081	-0.0516081
FBXL13	3.27390077	3.57742375	2.50200333	3.13710168	2.79413259	2.38377037	2.77809908	2.43957366	2.73622397	4.52297334	-1.19837795	3.00548117	3.45735103
NAPEPLD	4.28979767	4.77347781	4.70565262	4.74912197	5.00476654	5.53484965	5.96385625	5.51232825	5.44732894	4.77449946	4.85289373	5.58328095	4.23460963
PSMC2	4.83571359	5.09263356	4.87730773	5.70755722	4.72484008	5.06468135	4.80195678	4.39767406	4.85289935	4.50994485	5.1379795	4.46979451	4.88009981
YDJC	2.70517236	2.47203596	4.14801802	1.47921588	2.01205835	1.83034086	2.00541211	1.97297013	3.55729023	3.14307691	2.8818949	4.23858892	-0.68640693
CCDC116	1.08170827	1.03875312	1.52007679	0.79139377	0.91116879	0.9376827	0.58299215	0.38350706	2.37703152	-0.14910747	1.60402041	2.06909886	-0.14910747
DVL3	6.52560079	6.16013099	6.47225461	6.07296661	6.47838886	5.83039071	6.16424293	6.38141984	6.16186786	6.41641092	6.26721566	6.20155956	6.59828337
AP2M1	7.46113459	6.98904839	7.46631074	6.97939925	6.66858445	6.93521682	6.92651854	6.91685088	6.71578896	7.29650487	6.8765919	7.13089921	7.26684374
ABCF3	5.59573842	5.12607177	5.68378837	4.98079667	5.12147738	4.70722046	4.92911847	4.84113829	5.15519865	5.62550611	5.52832236	5.28229296	5.37602562
PCYT1A	5.65373951	5.88996207	5.87863482	5.8562772	5.40744762	5.68055377	5.9452949	5.2418683	5.60209522	5.89028817	5.83740097	5.35620701	6.26533978
FBXO27	4.25176573	3.10596761	3.73778833	2.18099057	3.4934192	3.25319484	3.40891482	3.40241087	3.48528144	3.96197445	3.4831639	3.75415962	3.59376684
DMKN	4.0970501	4.02174697	4.06992487	3.07939014	4.46229922	4.22363812	3.26383375	3.47300989	4.53450072	4.75907966	3.42572909	3.86917952	3.67645706
U2AF1L4	3.81570275	3.17030274	4.22889563	3.04314944	2.9802437	3.12423603	2.88989594	3.02796526	3.79697981	4.04619987	3.79505074	3.61761227	2.11391948
BDH1	2.42125067	1.7119945	1.25102487	0.88508231	1.90433288	2.16486487	2.44779071	3.73864942	2.7235296	-0.91344729	1.00405961	2.71399111	4.25983125
NPHS1	0	0.15655907	0.00124472	0.01152256	0	0.00069205	0.0547842	0.00158797	0	0	0	0	0
THAP8	3.40494131	1.4186823	3.21588758	1.59532691	1.42076683	3.08874254	1.7565903	1.1914039	2.58932563	3.64446587	-0.91803581	0.73368533	-0.91803581
COX7A1	3.88610006	4.32815081	3.70540796	2.30651545	3.80050625	4.95555445	3.47493015	3.85458258	4.00099235	4.68560766	4.57342503	4.80491679	4.6304272
ZNF382	3.35627274	4.23719833	3.97462207	3.45047157	4.42159394	4.23055954	4.16764821	4.16876164	3.80833107	3.98973459	4.20910808	4.0042951	3.81412606
LRRC56	0.72254726	0.91987284	0.65031245	0.19852489	0.12551843	0.35532353	0.33479169	0.26042985	0	0	0	0	0
PLXDC1	3.25535433	4.18013391	1.29094155	4.71735971	3.04344416	1.43582616	2.22230224	1.72564125	3.76421529	3.45825508	4.32809009	4.32704197	4.84578858
PGAP3	4.86062063	4.95034985	5.51651871	4.0457255	4.21088083	4.21594374	3.91462321	4.44811319	3.51788144	5.0165401	5.15245747	5.0132288	4.63489367
IKZF3	4.56027137	5.05415622	2.69872926	3.73969256	3.39714206	2.84567442	3.46603909	2.76215158	3.89024365	4.10665474	3.9849249	1.64060107	3.84918742

GRIN2C	0.26777737	0.5664883	0.30115267	0.42864185	0.55424788	0.29835444	0.47241802	0.74695161	-0.19980882	-0.19980882	2.56915016	-0.19980882	3.10897403
FDXR	3.80014827	3.14349128	4.47792238	3.40740991	2.6381225	3.13916896	3.20897748	2.81548053	4.33207905	3.38819705	3.63187584	4.33354994	2.32997753
SAP30BP	5.79719217	5.32645486	6.41041521	5.47477798	5.57789609	5.525746	5.48074596	5.57014246	5.42030937	5.92623486	5.21900253	5.28754331	5.82935079
ACOX1	6.10281788	6.24432117	5.98672907	6.05530044	5.96288517	6.19814066	6.56261616	6.24495546	5.9513474	6.09234993	6.3657598	6.3233106	6.53790295
PRPSAP1	4.47867985	4.60363685	4.56370182	4.46558891	5.13218263	5.04334174	5.8826288	5.13887682	5.50254476	5.21581154	5.19707125	5.61719836	5.99584618
CYGB	2.23405869	3.54648429	0.48170347	2.95974193	3.14661496	0.8468287	2.49595848	2.09807502	3.85163629	4.7826208	3.43973294	2.79724123	4.59332573
SRSF2	5.86773668	6.1496855	6.16567228	6.68188697	6.02417326	5.89612178	5.97799204	6.04255379	5.79528676	6.23891557	6.03756959	6.35826951	6.03108849
ZNF577	1.62215664	0.72027847	1.17959296	0.73863321	2.31055906	2.11440068	2.43013391	2.3697477	2.33212089	3.17926195	1.62863956	-0.02088574	-0.02088574
TMEM143	3.77532459	2.14710122	3.59948017	2.60629569	2.82475471	2.91017161	3.3693244	2.32829474	3.4973534	3.18993688	1.50170318	1.74098743	3.87953608
KLHL10	1.17757027	0.86269759	0.55637894	0.47719621	1.49486095	1.70350314	1.1506747	2.25271228	-0.64299022	3.08278285	2.67017475	2.88803247	-0.64299022
ALDH16A1	3.72057642	3.23029851	4.45408185	3.1399619	2.66806804	2.41447067	2.85793722	2.55988654	1.08191688	3.76639068	2.46969866	3.5643452	3.45742647
ITGA5	7.86254993	5.4242728	8.66885921	8.16451349	7.76986071	6.32871637	6.62099372	6.20616964	5.31061788	5.96204749	6.32101951	5.50846844	6.33766063
SIGLEC11	1.48235276	1.00462525	2.52360041	1.30743691	1.14325888	1.44420972	2.41130167	1.35886701	2.00029313	2.99247481	2.85191095	0.55474759	0.29986878
ZNF385A	4.50383979	3.90287345	4.53354895	4.33436985	3.09331378	2.09659095	2.66714715	2.18821541	2.12297674	3.9834724	3.46228371	3.66344537	0.74891756
MPP3	3.78431998	4.07230195	4.44306564	4.01074728	4.76782181	4.70103681	4.80300084	5.33862354	4.81238715	4.00443332	4.94718851	4.61426805	5.1771202
CD300LG	0.69477998	1.24089392	0.67295215	0.71384037	0.93076584	1.63682049	1.12118022	1.69529456	-0.36647732	3.6565481	3.32707485	-0.36647732	3.96833564
NAGS	1.35343999	0.31084342	1.6041921	1.00276867	1.41062619	1.63853561	0.54500432	1.80229835	-0.60693167	3.83532878	-0.60693167	2.91144323	-0.60693167
LSM12	3.99631915	2.87052165	3.32977458	4.26469394	3.71082445	3.73573537	4.18699127	4.10966812	3.83621492	2.23058619	5.24598245	4.13338176	2.41240546
ASB16	2.57653848	0.58956244	3.21002755	0.30567784	2.20765791	2.05227684	1.82443094	1.25173047	2.17813247	-0.63722536	2.99043218	3.74688974	2.26779544
JOSD2	1.95941336	1.41615621	3.6662715	1.44625007	1.71281438	1.82082835	1.45226537	1.44684528	3.36028207	2.92291583	2.70752309	1.78849764	-0.34916592
SHANK1	1.79672258	1.02057862	1.22073855	1.08150124	1.02098056	1.06316524	1.07974151	2.04294539	-0.5959866	1.82349217	3.43110954	2.84037667	-0.5959866
FAM171A2	-0.08580473	-0.10542321	0.19625202	0.93860282	0.16070007	-0.10410252	0.17263946	-0.10239276	-0.11131868	-0.11131868	-0.11131868	-0.11131868	-0.11131868
DBF4B	2.53844202	-0.33407641	3.27358489	1.78600196	1.97689601	0.82716813	1.63340703	1.32447523	-0.29640925	3.35127722	1.33261275	3.5122572	1.59243431
PLCD3	4.88794547	4.71809875	5.18929617	2.89608086	4.39714464	4.51615576	5.08025971	5.31488656	5.38289996	5.63296934	5.21872736	5.44839788	4.73882082
FMNL3	6.36881788	6.98580134	6.86794212	7.53517649	6.51759732	5.12973611	5.49634241	5.38897966	6.25474996	5.17880946	7.22346336	5.13901555	6.50981939
AQP5	-0.09073915	-0.09073915	-0.08843972	-0.09073915	-0.09073915	0.4217324	0.11457355	0.83817382	-0.11887442	-0.11887442	-0.11887442	-0.11887442	-0.11887442
RACGAP1	4.29677147	3.76526538	5.40168643	5.61504769	3.8713238	4.00942548	4.5446228	3.63233637	3.39525395	3.76165301	4.26021963	3.25738803	4.19586029
LARP4	5.71248412	6.14163259	5.76200837	6.78452328	6.7730068	6.8357896	6.70932763	6.46556772	6.40071416	5.72886095	6.42908155	6.34651993	5.94873189
GRASP	3.42662527	3.60364596	2.9492636	3.11709646	4.26832431	1.93856838	4.78470238	3.75800118	4.03185421	3.42854128	3.85923347	3.69620469	4.28793237
RAVER1	4.47303689	3.80082981	5.03971438	3.83374064	3.48802028	3.43732292	3.84760003	4.01624978	3.96561934	4.9504475	4.17699889	4.48385464	3.44141797
SYCE2	1.53179149	0.55777936	1.65805379	0.74146747	0.53329526	0.49676106	1.79274431	0.60894561	-0.40096816	-0.40096816	4.07594194	2.83559685	-0.40096816
SPC24	1.22694226	-0.18344613	3.88308374	2.46261969	0.19104787	0.34169747	0.72946632	0.06684567	-0.26989988	-0.26989988	-0.26989988	0.80051544	-0.26989988
IP6K3	0.45494498	0.42933897	0.2372127	0.42041357	0.6433613	3.67250425	0.63275003	0.58896442	-0.26301382	-0.26301382	2.27994539	2.89073379	4.69347143
LEMD2	5.6626915	5.53918428	5.71137465	5.17802629	5.27891031	4.98784177	5.31555239	5.6626915	5.26274008	5.13716034	5.72423997	5.39210168	5.47800492
ALOX15	-0.11003226	4.21006673	-0.17559602	0.26745555	-0.07562968	-0.22639673	0.03821049	0.07826965	-0.08170929	-0.08170929	-0.08170929	-0.08170929	-0.08170929
TREML1	0.89051854	2.3197531	0.94675843	1.89381956	0.16396988	-0.67978298	-0.27055759	-0.48550073	-0.29077435	-0.29077435	2.88074525	-0.29077435	1.62918754
ZNF653	4.0537393	3.06979568	3.43734877	2.37255939	3.36501997	3.14584974	3.70067164	3.74715863	3.81437177	3.99416253	3.43205856	3.14540607	2.3773812
MED11	4.09550926	2.34527308	4.15845979	2.84884032	2.02114095	3.60670796	2.65924566	3.61284857	2.70480531	-0.97520455	2.96603663	3.29115309	3.85900243
CXCL16	6.023876	5.29563509	5.31475809	6.10305538	4.72454007	3.89764795	4.34285197	4.0736918	4.78414859	4.58997249	3.82128839	4.72238905	5.69453114
BCL6B	1.16149841	3.96395821	3.55015514	5.5188895	4.14723635	1.94039632	1.60070321	2.95954652	4.80754127	4.48157648	5.02662434	4.28036423	5.0619435
ASGR2	0.47664975	0.01000314	0.72364474	0.82937477	0.57697962	-0.24550525	0.0573312	-0.14400034	-0.1954293	-0.1954293	-0.1954293	-0.1954293	2.81644284
TNFSF13	5.68062707	4.57200821	5.20866019	3.72342249	3.96514726	4.42384818	3.6433884	4.21078834	4.4285261	3.77551255	3.32493219	3.74394108	4.13271184
SENP3	2.33005174	2.16298189	3.06305067	1.32865801	2.4085918	2.03410205	3.29557163	1.81776145	2.49431309	3.59567057	2.6449796	2.25658249	2.69964568
FGF11	1.51176705	1.29955221	1.24027531	1.2773854	1.23767398	1.61742042	1.45789858	1.61688165	-0.83085912	2.19426783	3.10637061	-0.83085912	3.83240215
EIF4A1	5.70940215	5.5612344	6.08518878	5.54691117	4.85345439	4.22106902	4.83428012	4.9002216	4.56812187	5.36029743	4.99757832	4.80487046	4.63261822
RPL26	6.91947508	7.69234974	7.69351808	8.02260591	7.72757241	7.00988315	7.57872288	7.27043834	7.65737393	7.5096158	6.68372295	7.17767591	7.66565282
CCDC42	0.9415536	0.21985981	0.56748776	0.56969973	1.27589961	1.21510663	0.33199037	1.82519819	1.59056203	-0.3010907	3.22654602	2.79731646	2.96246262

POLR3K	3.60589935	2.99684992	4.29577644	2.68417607	2.14113383	3.70281025	3.07034746	3.08271641	3.18359752	1.98087297	-0.19093915	3.8165329	4.21275902
SNRNP25	4.27071832	3.29586347	4.04944015	3.44052321	2.88945225	3.52286879	3.84637928	3.13544077	3.84308011	4.49496792	3.51908774	3.64011002	3.59672496
WDR90	3.47263166	3.00700187	4.51773523	2.73634836	2.72538181	1.75967194	2.97042211	3.09880103	2.88175655	2.47825691	3.63126546	3.30527461	0.24897314
JMJD8	5.9811321	5.04167945	6.18676311	4.59456729	5.35887675	5.86493768	5.09955658	5.41702855	5.50445997	6.15363295	5.61322867	6.2121618	4.70437862
CCDC78	0.5321357	0.42792147	0.66505682	0.61031184	0.2727638	0.24922109	0.33954846	0.25108564	-0.3762595	-0.3762595	2.68748275	2.56436713	-0.3762595
MSLNL	0	0.14878977	0.00124472	0	0.02253148	0.00069205	0	0.191069	0	0	0	0	0
SSTR5	2.60170914	1.53068401	1.50308143	-0.16547319	0.71472203	2.23099021	1.69054647	1.7827039	0.24775117	2.98123535	3.90534779	3.05186647	-0.42836569
SPSB3	4.66891576	4.4231489	4.55316565	2.93034398	3.9014206	3.82869293	3.8482281	4.86092486	3.56494775	4.93687819	3.94166932	4.87365933	3.85218287
CCNF	1.50389906	0.46589276	3.65727609	3.4044539	0.49583478	0.1563322	1.11725095	0.65333375	0.29182708	-0.38188099	3.273315	2.16640836	-0.38188099
TBC1D24	4.22646365	3.29394707	4.43917434	3.78714126	3.52748525	3.53809154	3.58532137	4.19563666	4.17864984	3.14956337	3.57453899	4.21652936	3.52876845
AMDHD2	4.1710671	3.49627361	4.86678087	2.6448941	3.11954491	2.41965473	3.52909002	3.49175231	3.61439107	4.57144335	3.63060637	4.2562809	2.82882768
NTN3	0	0.24103178	0.00124472	0	0	0.00069205	0.03598562	0.21382149	0	0	0	0	0
PAQR4	2.98873171	2.58005794	4.76338276	3.50947422	2.56347576	2.15562076	2.02869408	2.25564447	2.789163	3.98618344	3.71806795	2.86279688	2.99674683
FLYWCH2	3.38672968	3.57476243	4.21791686	3.51075186	2.39859833	3.79781761	1.91999664	2.49581487	1.82182612	3.94567985	2.5670872	3.80420717	2.52654045
ZNF75A	3.86006358	3.94801025	4.14302716	5.08868316	4.87725637	4.23397314	4.34829498	4.21963785	4.84551551	3.96153816	4.53684372	4.25219531	4.07374642
ADCY9	6.79752374	6.08021983	6.51450829	5.22711101	6.69115235	6.50979109	6.83068091	6.9232788	6.28745742	7.18486251	6.59597041	6.8177808	6.80890114
SHANK2	2.22063959	2.12367228	2.22225438	2.35354473	2.47320312	1.9650191	1.95984339	2.75494057	-0.45099859	2.7641386	3.72539675	2.01415531	1.80168206
CLPB	4.98938185	4.5583739	5.30103502	4.84095135	4.30206096	4.31000519	4.10290898	3.95932014	4.56935987	4.62255763	4.42310467	3.79257773	5.34078335
NEU3	4.56601349	3.4324169	4.55067266	4.217974	3.97248977	3.9807586	3.94458522	3.93437928	3.76426959	4.26738307	3.44215935	4.21357423	3.96232772
PPP1R32	3.22709751	1.96871657	3.40751576	1.55598191	3.09976628	2.15866611	2.82995184	2.99173405	2.42641037	2.58337816	3.34033142	2.3064532	3.15311408
ASRGL1	1.47260608	1.6382575	1.9982571	2.06205751	1.41056179	2.03046665	2.33018632	2.0483312	3.63996975	2.2871679	2.48803605	1.71809436	3.92419531
UBXN1	5.88500493	5.55724438	6.0255217	4.90916615	5.13003999	5.40465845	5.00287944	5.45994101	5.27112348	5.98764024	4.4569764	5.7140422	5.4116302
TTC9C	4.60266682	4.21664117	3.73197877	3.78322506	4.21222386	4.54233162	3.881496	3.94882021	4.48917904	4.70029554	4.18793361	4.15176725	4.17337355
TAF6L	3.72019068	3.15468211	3.85339078	2.50944015	3.31811134	3.02005754	3.49086428	3.91241871	3.78321574	4.13215829	3.37325517	3.91105425	3.31520614
NXF1	5.21899075	4.36788412	4.60291224	4.38511321	4.62194535	4.04027625	4.81089074	4.70497272	4.94879475	4.7169899	4.63038934	4.38929608	5.14581574
STX5	5.82617424	5.38937329	5.74403461	5.68680081	5.0662588	5.08564876	5.1300292	5.58033575	5.06988837	5.21318087	5.57209084	5.6366055	5.23229241
SLC25A45	3.89917624	3.10051599	4.51887688	2.54774487	3.56654625	2.96234857	2.88165045	3.87840613	3.01465435	4.74059435	3.88949303	4.18670421	3.30993944
RPL29	7.23104113	7.49637467	7.09132828	8.05889385	7.12816852	6.69104913	6.67263386	7.00567537	7.04531949	8.01139928	7.04287291	7.37621159	7.06057939
ITIH3	2.64645056	1.54383769	1.29553138	1.49678926	4.14628804	3.30622758	2.23516134	2.31986419	3.03183579	2.99314408	3.15876078	-1.14036423	2.5995821
SYVN1	5.09035326	4.21007882	5.0164994	3.99649668	4.25831599	3.71065414	4.07614825	4.15322024	4.23340087	5.33115292	3.91971625	4.52090778	3.11164446
ZFPL1	5.21982209	5.22159042	4.77567242	4.41387996	5.11549085	4.70759279	4.91682306	4.56372066	4.83782567	5.0258879	4.87668969	4.96440734	4.67756455
RPS6KA4	3.59177057	4.06034691	4.78436636	2.91171243	2.6436556	3.40310755	3.52113696	3.06096765	3.25817758	4.25255383	4.27278166	4.04486792	3.0553842
LRP5	2.59315731	2.44708312	2.68522581	-0.41232666	2.37889607	2.37889607	2.43528887	1.95546383	2.59915122	3.8228372	3.42094969	0.41595069	1.47542845
TPCN2	3.70623878	3.18788453	3.88778417	2.44898476	3.36481855	1.96572514	3.32673266	4.43964145	-0.86756942	3.39801983	3.46946152	3.25525632	3.53314506
CYP4A22	0.50439908	0.08084644	0.71047385	0.08084644	0.76814705	0.08223977	0.08084644	0.77727185	-0.11713164	-0.11713164	-0.11713164	2.43245496	-0.11713164
PDZK1IP1	1.80534904	3.40765724	3.67713634	2.64255852	0.32428646	2.3075708	0.88433115	-0.492796	0.80000546	-0.73128239	3.13571679	3.54609375	-0.73128239
TAL1	1.94350121	2.79638134	1.29336544	1.97655429	2.14070726	0.68765246	2.03558164	2.47210821	2.67264366	-0.78189979	-0.78189979	2.65775408	4.28546034
CMPK1	6.19500403	7.24245764	6.93107112	7.78883374	8.04338825	7.73598609	8.20075788	8.09785961	7.91881353	6.17074825	7.72221462	7.69394715	6.27686501
BEND5	5.59916242	4.37139209	4.3639703	3.51008654	3.94683431	4.68398782	4.05402993	4.53254871	4.27568484	5.34773904	4.14630667	4.15984764	3.05283531
ELAVL4	0.5036794	1.32893828	0.88693657	0.84260642	0.83083713	0.70709819	0.58709666	0.85951549	3.12618266	-0.26546407	-0.26546407	1.89280552	2.22450113
ZYG11B	4.60765078	4.50598824	4.27164651	4.68983518	4.99953688	5.38014852	5.29179871	5.42261606	5.02412003	5.37045195	5.12507049	5.13955745	5.35581667
SLC1A7	0.7261826	1.06651917	0.72869708	0.73567644	0.93214587	1.57596674	1.36934324	2.54993617	3.8044493	4.89380282	-0.22547526	4.97057469	3.95385769
C1orf123	5.71949564	5.42697694	5.03485181	5.27151967	5.66129938	6.01994589	5.25423066	5.53980939	5.40114543	6.01700603	4.90285823	5.20853836	5.69699599
MAGOH	4.8794017	4.75588874	3.39913714	4.19107486	4.25142561	4.9085559	4.86013481	3.94939213	4.79782114	4.5502092	3.20699074	4.43194686	4.10305145
ACOT11	4.05847092	3.52516551	3.71325734	2.57166623	4.10825432	3.93103982	4.87941552	4.4827262	4.46115326	3.45366445	4.23004332	5.07814107	4.44881639
PARS2	4.16973414	3.23255576	3.4089308	2.77427414	3.55424137	3.77300053	2.96707099	3.0882106	2.85553967	3.42312369	2.63039868	3.37813823	4.08012077
USP24	6.26527112	6.6953791	5.85996606	6.76506986	6.78097789	6.68267443	6.755817	6.85560524	6.21939675	5.79975568	6.48980736	6.33502239	7.18696414

NOL9	5.98208014	5.600219	5.68295424	5.98903235	5.84040224	5.69858541	5.72513049	5.74171364	5.48674762	5.56522452	5.74249196	5.59726403	5.9948478
PRKAA2	0.24957996	1.70988172	0.47952455	0.25621556	2.37414894	3.25381834	2.03521911	2.61424396	4.26128384	-0.16401899	1.80308237	3.59955043	1.36362314
KLHL21	6.12851743	5.7818939	6.38764304	4.1570064	5.4807586	5.85176795	5.98400477	5.78545738	5.24073588	6.0733498	5.59741903	6.30773466	4.63318049
ZSWIM5	4.17063339	3.06186251	3.72583299	1.93611674	3.11521919	4.09122895	3.87052803	3.34093061	3.30145635	4.27059017	3.51919842	3.73270182	2.97850566
GMEB1	5.18768927	4.40701432	4.90470628	4.5973754	4.89429717	4.39367264	4.19049376	4.82107069	3.87938266	4.75754727	4.41780051	4.62817935	5.07319257
SLC45A1	3.26338852	2.69893516	3.01933675	1.45305716	3.06278229	3.50452925	3.32697482	3.56395914	2.21498948	4.0766836	1.71664702	3.95829439	2.05404212
AK4	3.54516271	2.99539872	2.77343296	3.20012523	4.72012848	4.60846396	3.51911182	2.07193394	2.35407583	1.63767109	2.28771582	4.41504014	3.79739851
JAK1	7.40317567	7.42038003	7.02615126	7.51866548	7.31457835	7.60803788	7.3402003	7.37469662	7.28612467	7.25547267	7.25328782	7.34728416	7.61897396
RAVER2	5.38954563	5.91651382	5.06302322	5.22423544	6.70232638	6.64881052	6.16833515	6.78005788	6.31629664	5.43520551	6.21182486	6.67310469	6.77057191
CTRC	0.01032169	0.18182438	0.00124472	0	0	0.00069205	0.10255281	0.00158797	0	0	0	0	0
LZIC	4.78687152	5.68007574	4.8379576	5.36134442	5.68841909	5.6608236	5.39098349	5.19341546	5.88037079	5.50559104	5.16801821	5.42783196	5.44008399
RBP7	2.51262153	4.09549015	2.77040592	2.99942952	4.86324909	3.25734987	2.56130414	4.23899581	4.08858009	4.18473886	4.91412215	3.60487831	5.41977668
FBLIM1	8.14032253	8.04247746	8.54540941	7.22383063	8.06156461	7.9137734	8.22769462	8.42954757	7.76980429	8.63849816	8.15131683	8.44282861	8.08167098
SLC25A34	3.89533791	1.95351527	3.17916422	1.2541158	2.21402387	2.28903642	1.94193358	3.00344361	0.96026816	-0.76212687	2.50851735	3.96000342	3.5318328
AKR7A3	2.15998343	1.24103377	1.69928378	0.48959865	0.40669407	-0.15350808	0.35593485	1.69623696	-0.34029739	-0.34029739	-0.34029739	3.02976687	-0.34029739
PDPN	6.12169386	5.94734788	3.63674191	4.0641264	2.44680954	4.02212292	4.05690824	5.4590459	3.99784834	3.95174886	3.92654662	4.50219752	4.93014934
DHRS3	6.52182654	6.26001914	6.60993818	4.97058052	5.42860274	5.34987978	5.62234426	5.20277424	5.73242312	5.66042376	5.29236251	5.14361128	5.81155584
MATN1	0.56880756	0.16542578	0.39745577	0.195707	0.22827448	0.27342705	0.20461521	0.16871198	0.43995893	-0.13397724	-0.13397724	-0.13397724	-0.13397724
LAPTM5	7.25545826	6.84910642	7.09354189	7.85035762	5.7902713	4.98187503	5.67325397	5.20983999	6.22038379	5.48012553	5.37293315	5.17560824	6.19859714
SDC3	6.83351024	5.52455985	6.39374551	5.96123573	5.60870281	5.46765515	5.35612982	5.61833813	6.28642117	6.93062122	5.82951554	6.81450898	5.7753426
PEF1	6.08539236	5.94493132	6.10872152	5.01169088	5.73259765	6.10747615	5.47641766	5.16940708	5.79984017	6.82466804	6.09342401	6.51624663	4.89091271
SYNC	5.07777134	5.21096088	5.49303212	5.23305542	6.8683391	6.68301932	5.75739426	7.39466581	6.58483197	7.1886968	6.570875	7.05638219	6.55243389
RBBP4	5.53301331	6.33074949	5.97390896	6.78392573	6.50801588	6.60916878	6.87750375	6.57404909	6.42874564	6.00770633	6.44221258	6.4334053	6.22314708
KIAA1522	4.06453201	4.4461412	4.11167873	3.32372728	3.70915397	3.05476749	3.48997253	4.16062547	3.66783848	4.40989832	4.46541238	4.36526294	3.24739473
TSSK3	1.05645216	-0.2913152	1.4954903	-0.64575794	0.71120835	-0.0144149	0.87430221	1.08870243	0.20573754	0.20573754	0.20573754	0.20573754	0.20573754
TMCO4	5.15157412	4.81981245	5.16743474	3.97752435	4.9850569	5.25383142	5.00479248	4.94879842	4.38226554	5.34640337	4.81170988	4.77213881	5.25888586
UBXN10	0.24101392	1.74687388	0.14299387	0.38074424	0.20395332	0.35672854	0.28195267	0.14365958	-0.1684644	-0.1684644	-0.1684644	-0.1684644	3.49566786
CAMK2N1	4.57455947	4.54576546	4.39211427	3.70918966	5.36356463	4.84355693	5.10377302	5.76587625	4.05130843	4.35931964	4.18210537	4.45101979	4.64561834
ALPL	0.48380662	0.93743108	0.34077342	5.11034012	1.33891508	-0.00768174	1.23467748	0.19178441	0.32443052	2.69356636	2.87432133	-0.36869913	4.36619385
WNT4	0.25122979	0.52099989	0.08581253	0.21612582	0.36741538	0.084667	0.08323259	0.39364949	-0.14371429	-0.14371429	-0.14371429	-0.14371429	-0.14371429
SCNN1D	2.0501054	0.7470898	2.63195479	1.45912626	1.69426249	1.72801965	2.19489992	2.42032053	3.19024951	-0.69888323	3.44484031	2.87441599	-0.69888323
MXRA8	6.46883733	5.60816416	7.19244184	5.82654857	4.99387356	5.80442166	5.76865657	5.62756424	5.7739217	7.06029057	6.73990914	7.1858823	5.77173639
MEGF6	5.64851318	6.61512248	6.70986454	4.35549393	6.38706413	5.56311724	6.85882132	6.97325955	6.63145719	7.26972757	7.18144474	7.36738519	6.06590199
DIRAS3	5.2049096	3.39037568	4.83586572	4.14200386	2.93405455	3.96671629	3.25996573	3.26886077	4.15156135	4.55222466	2.54697406	3.83612316	2.98048192
C1orf87	1.37015683	0.76100387	0.76341928	0.76100387	1.2155728	3.3819503	1.74134281	0.76408537	2.84436959	3.97095202	-0.48499076	-0.48499076	2.05474578
NFIA	5.92658334	6.08208186	5.6567264	5.09608493	6.0384618	6.71957812	6.77001746	6.64539449	6.15792468	6.93239127	5.72209826	6.24008549	6.38123207
OMA1	4.13456245	5.56113118	4.49948606	4.92597433	5.60345348	5.48973318	5.61302896	5.25844717	5.34826225	5.4855748	5.04289348	5.18758348	5.65353057
MYSM1	5.31692132	5.93170774	5.22883856	6.45637737	6.17119568	6.12645344	6.06367967	6.5019112	5.89509134	5.15066044	5.74537744	5.70167871	5.4731843
TM2D1	4.69511354	5.15484595	4.34689242	5.06319656	5.49121114	5.18649432	5.57445337	5.75247118	5.62382057	5.39045357	5.19861808	5.34516631	4.94744513
USP1	4.74128914	5.76560492	5.21981073	6.12966863	6.09574723	6.38328166	6.10482439	6.0610275	6.46427656	5.46842541	5.86476532	6.04269218	5.09483483
FUBP1	6.46179329	6.59090911	5.92668014	7.14076462	6.64210466	7.34463868	7.05148487	6.84340929	6.44203981	6.08344436	6.29586988	6.04457266	7.12050853
NEXN	5.95330461	6.80764972	6.33092873	5.59447706	8.11326635	8.46780192	7.71038361	8.14247426	8.3452962	7.12817581	6.98065136	7.76393657	7.80984882
DNAJB4	5.15130125	6.03785053	5.61818501	6.77249619	7.66692722	7.77252912	7.23218943	7.31522928	7.52326034	6.58919886	7.22374184	7.74452573	6.60522627
LRRIQ3	0.14376414	0.43639342	0.13155637	0.16901917	0.14903387	0.57256791	0.35023294	0.13226605	-0.17086215	-0.17086215	-0.17086215	-0.17086215	-0.17086215
TYW3	5.22499284	5.21642585	5.01666912	5.47867187	5.96154075	6.26737332	5.91015006	5.72823547	5.73056915	5.96839591	5.1999923	5.6382057	5.55681398
SNX7	5.1932894	5.73439517	5.62308235	6.05914613	6.25557933	6.3374307	5.9273542	6.34901983	6.4507649	6.38896205	6.0785503	5.92656639	5.80846418
B3GALT2	1.63572557	2.34150328	1.4809772	1.68568233	2.08686429	2.79856304	1.90748405	2.55856377	2.11723899	3.25083763	2.57998008	3.58240602	4.81602682

NTNG1	0.06544745	1.61418656	0.00124472	0.24506392	0.11128265	0.04606918	0.04994155	0.12649846	0	0	0	0	0
FAM102B	5.86932981	6.23124555	5.75363833	6.27801811	5.33271272	5.13941795	6.14949505	5.51960072	5.93120761	5.70137865	5.58007285	5.77332749	5.31343723
HENMT1	2.17007213	2.52081063	2.71992402	3.73602909	2.57976147	3.40490023	2.35224545	3.44294866	3.58685992	2.77691415	1.74599717	3.00967834	0.01629582
AKNAD1	1.15298809	0.77942014	0.7820201	0.77942014	0.88450932	1.08019498	0.82381287	0.78273708	-0.53641264	-0.53641264	-0.53641264	-0.53641264	-0.53641264
C1orf52	3.82952403	4.13458795	3.71826335	4.52189761	4.83288578	4.99377695	4.46789327	4.39176173	4.56495893	2.50604643	5.03150902	4.82857307	3.33464584
WDR63	1.5833405	1.34930691	1.79726188	1.92113532	1.22954789	1.56475222	1.33745409	1.31428619	1.47347121	-0.82684144	-0.82684144	-0.82684144	2.75433002
GBP2	7.06130661	6.46430477	5.92858836	6.10385861	6.56259004	7.3164533	6.62323214	6.64763752	6.69878327	6.57726257	6.09230834	6.28661846	6.70762611
ATXN7L2	3.18015383	1.47802046	3.64314485	2.42603992	2.09125064	1.81157706	2.85351566	2.73327439	2.3914831	-1.09158272	0.54263946	2.99510695	-1.09158272
GBP4	4.62634528	5.97986274	3.52965063	4.92680765	5.10219858	4.47073176	3.97732558	3.99101026	5.47043125	4.16405302	5.67992509	4.27163354	5.66927161
ZNF326	4.32693431	4.77077246	4.16717764	5.12460938	5.14029678	5.6166387	4.98366755	5.18842	5.51776998	3.77844058	4.8696044	4.76192382	4.36919772
HFM1	0.87930716	1.03172397	1.13938195	0.96844812	1.46972582	2.18744552	1.62204165	1.61997081	1.99601257	-0.52840415	2.49682097	2.92293816	-0.52840415
GFI1	1.41381898	2.94343189	1.05704797	1.49694965	1.2054058	1.07447746	0.77166475	1.16610099	3.08021327	3.19154161	-0.56338091	1.97541882	-0.56338091
KCNT2	4.89777112	6.83938864	2.97703061	4.85491312	4.87538915	3.88101308	4.66329621	5.11421335	4.26718386	1.79761151	5.444742	3.17394289	3.62541912
AGL	4.85876066	5.86793331	4.75177726	5.61992818	6.07439158	6.29291205	6.07094959	6.01054242	4.9874733	5.98290993	5.69531198	6.17941333	6.17674912
VCAM1	6.50989149	7.57817056	6.09165909	8.72935583	5.40708166	5.06920289	4.97884269	4.68589321	7.19550996	5.02461546	7.10860133	4.93418799	6.59168386
EXTL2	4.11727981	4.65037465	4.11710324	5.41096612	4.77452011	5.12669596	5.20370329	4.75581614	5.3098924	5.02389143	4.66053158	5.50845511	4.26597267
SLC30A7	4.15241993	4.47659494	4.22180614	5.69189391	4.64937551	4.31126979	4.47013529	4.71097183	4.47097042	3.882514	4.2727666	4.84023639	4.16940595
ZNF281	4.62208539	4.95085721	4.39596769	5.72472774	5.42552503	5.53021154	5.16974861	5.09027906	5.10968736	4.6547381	4.66289793	4.93247409	4.64851963
ARPC5	5.0531159	5.98025977	5.4461456	7.12348884	6.7148601	6.33399313	6.69860108	7.00787641	6.69765486	5.1898251	6.81124854	6.89185804	5.11618776
CADM3	0.38191104	3.13735577	0.11388242	1.26086085	1.78065975	0.57801936	1.67724481	1.11763415	0	0	0	0	0
NLRP3	3.84054111	4.80217695	3.96265785	4.82524508	3.1498713	2.56901562	2.73536012	2.69228974	3.29598803	4.45159639	3.69400095	4.07302004	3.37841512
ZNF496	5.69913624	5.17382932	5.77817996	5.03545523	5.05787269	4.54133906	5.01238844	5.47377085	4.9215041	5.52435095	5.35536141	5.2917862	5.3829867
TRIM58	0.77064548	1.39655357	0.90779039	0.77494289	0.5726621	0.12918522	0.68816906	0.42177923	1.14524494	-0.62262886	-0.62262886	2.7579825	3.23179019
SLAMF9	1.6618104	-0.06686982	0.12119799	0.64797784	-0.24336258	-0.2426191	-0.24336258	-0.2416566	-0.0053942	-0.0053942	-0.0053942	-0.0053942	-0.0053942
IGSF8	6.01180868	5.35834338	5.75556772	4.05510398	5.23849312	5.38060785	5.37699375	5.96417944	5.07048148	6.34867988	5.31866079	5.98671962	4.93661017
DDR2	7.71805199	8.32274005	7.67788229	8.01868518	8.23133636	8.48317884	8.68674844	8.24839223	8.16011468	7.89098308	8.17580411	8.03466513	8.08120989
PEA15	7.59573539	7.53180911	7.30403688	7.54786058	8.31431589	8.07582692	8.12531582	8.46983347	7.7518688	8.35266807	7.78804605	7.82965021	7.86767846
PEX19	6.42107169	6.5374429	6.19819483	5.59454256	6.58717958	6.32163391	6.2815711	5.87111949	5.79903598	6.64100655	6.19579511	6.15813993	6.50209485
NCSTN	6.74314869	6.2702179	6.4984283	6.82379966	6.01510061	5.87865887	6.09803602	6.07404829	5.80073954	6.42654182	6.25973693	6.28772439	6.58489668
VANGL2	1.59352752	1.13417259	0.44065575	1.24023782	0.93830214	1.40725878	1.79514658	4.12860685	2.88464979	2.54636624	2.75495463	3.50714524	3.7132007
SLAMF6	3.11014526	3.36209898	1.12361209	2.42485048	1.42073265	1.72519806	1.96686321	0.70688864	2.49858981	-0.84045162	-0.84045162	1.82179881	3.51458364
OLFML2B	6.42829792	5.94656176	5.96734998	6.63954979	5.21231511	5.01744715	5.36568764	5.41091692	5.11231753	5.17916667	5.51617823	5.46023484	5.96288467
FCRLB	1.05341469	0.34691749	0.73066311	0.57640316	0.40711359	0.13325028	0.02818035	0.58194998	-0.28579261	-0.28579261	-0.28579261	3.6077355	1.01098942
KLHDC9	3.33989252	2.37754842	2.79091536	0.69738756	1.74426527	2.17726349	1.4183418	1.10263032	3.02192238	2.69445901	4.20293365	3.10437123	3.45704822
C1orf74	2.14593036	2.66911895	2.3276143	2.18068486	2.45175211	2.89553881	2.05925974	2.60327087	2.54229323	3.11028256	0.40850239	3.8070584	0.19738282
LMX1A	0	0.23015116	0.00124472	0	0.06767478	0.09493334	0.09115376	0.00158797	0	0	0	0	0
FLVCR1	3.60511605	4.0410911	3.27826911	4.70615437	3.87065467	3.61549926	4.61511042	4.33162659	4.00419007	3.17573907	3.83661217	3.8331662	3.30169055
FAM71A	0.12527007	0.41010014	0.49110995	0.32218126	-0.03135839	1.75727738	-0.03135839	0.62178231	1.07394046	-0.17288441	-0.17288441	-0.17288441	2.12888125
ATF3	4.12074087	5.3596087	3.58513496	5.80349819	5.2587362	7.43578382	5.14758259	4.45378491	5.31913119	3.64309539	4.95922062	5.55238608	6.30392198
RBM15	5.38763517	5.34098414	5.72745959	5.32530054	5.42463981	5.03380165	5.02691089	5.03496298	5.04882481	5.60288003	5.31373547	5.42658823	4.79972978
DENND2D	4.94415961	4.30432797	3.85172481	3.51976572	3.14198076	2.24386613	3.7486899	2.12218677	3.26078227	1.57206401	3.51199955	2.30887686	2.50661001
AXDND1	0.55364827	0.7951377	0.41524853	0.55315953	0.66270079	0.67479332	0.45318467	0.57892505	-0.2692662	-0.2692662	1.75649218	-0.2692662	-0.2692662
TDRD5	0.21547059	0.21547059	0.43127168	0.22354978	0.23663627	1.6300627	0.55884748	0.59704244	-0.19865943	-0.19865943	-0.19865943	-0.19865943	-0.19865943
IER5	4.46002851	3.98268984	5.18664239	4.56418687	3.7694575	5.16654992	4.22274794	3.87924881	4.29697618	3.99864322	4.63613249	4.59770345	4.31067928
SNED1	7.7017839	5.98129866	7.35449453	5.75110119	6.43002832	6.46686876	7.02343718	6.78049617	6.30076467	6.31947991	6.55092693	6.19007896	6.04631727
BPNT1	4.87651384	4.39098533	4.71103414	4.34709334	4.8106993	5.34544984	5.3148926	4.79153745	5.45697767	5.64842184	5.37638525	4.94161999	5.02403524
SPATA17	0.67442329	0.70509828	0.78296638	0.62752516	0.62860837	1.14342988	0.61133332	0.61464016	1.17771507	-0.3418174	2.42778457	-0.3418174	-0.3418174

C1orf115	5.42921093	5.69579167	4.73184223	3.89450525	4.73729141	4.77715063	4.81034954	6.02099694	5.41835721	5.441519	5.62491366	4.7164929	5.9968248
BROX	4.994615	5.4782835	4.98227498	6.16653016	6.08704888	6.17592364	6.10893318	6.03238497	6.25430756	5.63849578	5.64670161	5.63159807	5.14113291
ACP6	4.76783991	3.48377311	3.92941962	3.11135293	4.48971869	4.32057123	4.57776196	4.38535988	4.04254465	5.03817354	4.66823292	4.57424507	4.02014739
WDR64	0.50645058	0.76315797	0.50905444	0.50645058	0.52716252	0.60651039	0.50645058	0.50977249	-0.28301221	3.48997845	-0.28301221	-0.28301221	2.59639425
KIF26B	0.12496531	0.10195601	0.1162445	2.50147868	0.08344591	0.08519306	0.08624574	0.08193958	0	0	0	0	0
TFB2M	3.00567585	3.34777688	3.01305896	4.90482588	3.46769275	4.25182999	3.6369775	3.76165119	3.62344832	3.75256344	4.75429921	3.28383942	4.0433079
CNST	4.32216293	4.23572373	3.71270259	4.47720291	4.78742664	4.97426795	4.48473865	4.88633674	4.88027207	5.1244558	4.26098648	4.48580073	4.96146022
PPP1R21	5.4329486	5.9410419	5.12847939	5.51018144	6.22909401	5.90167526	6.07227254	6.21889779	6.0672232	6.22271498	5.64737091	6.12823956	6.25542945
KLHDC8A	0.04786178	0.04786178	0.05036995	0.59088499	0.04786178	0.04925628	0.04786178	0.05106161	-0.02924167	-0.02924167	-0.02924167	-0.02924167	-0.02924167
PM20D1	1.11013477	1.56604765	1.46286561	0.52795505	1.51757934	2.92638497	1.04884676	0.52969969	3.3455836	3.8727625	2.89974982	1.67825147	0.51846852
PKDCC	4.50615589	5.21448542	5.23733107	4.10004002	6.02437394	5.82140035	5.97183078	6.29151756	5.79901283	6.719686	6.33066861	6.71514894	5.47546653
OXER1	3.48775257	2.54478543	2.7006566	1.45097149	0.95008279	2.12119008	1.98762463	1.70574744	3.39618565	2.51505958	1.00711977	3.16590194	2.63324418
HAAO	5.34738625	4.49704315	5.74620841	4.21240253	4.37215784	5.20751904	3.96081767	4.46067436	5.40249363	5.89971458	5.02346178	5.23441814	3.25314258
B3GALNT2	5.83954884	6.08714506	5.68726872	5.17670351	6.52928059	6.09218362	6.3311319	6.64980093	6.22090651	6.30130085	6.67593584	6.97691552	6.02410265
MAPKAPK2	4.97739215	4.29381648	4.90604292	4.49237236	4.40489022	3.34826158	3.69385513	3.65521994	3.91194668	3.69724981	4.15956039	4.37240367	5.45140646
CAPN2	6.54279383	7.1671872	7.35113904	7.04159423	7.5337674	6.99337786	7.8625738	7.71904522	7.43213641	7.42333665	7.75363397	7.41216677	6.91435966
MRPL55	3.9516361	4.0645141	3.91748985	2.78367265	3.01349486	3.85412475	3.2876315	4.06184308	3.5826533	3.53268065	4.1545645	3.75039603	3.59389525
WDR26	5.92116093	6.52472528	5.84771326	6.67015693	6.60478353	6.30872168	6.6509951	6.75430185	6.62187127	6.11499756	6.43698906	6.50845329	5.88793211
REL	4.47496342	4.51426729	3.98969765	5.25046829	4.62290103	4.47403455	4.32834939	4.32388871	4.36518033	4.05801849	4.43349178	4.05375428	4.44120826
PUS10	3.10741164	3.54295783	3.26478427	4.26878718	3.68619958	4.5702847	4.1404206	3.5663974	4.07162231	3.31462761	3.42173233	3.64389512	4.45219888
PEX13	5.31835929	5.00217181	4.60195554	5.46649084	5.68398122	5.61655032	5.5865288	5.17864527	5.20081271	4.48697404	5.32577262	5.12748171	4.77592977
KIAA1841	3.8452032	3.25757511	4.37046226	4.81495707	4.1743477	4.48040271	4.82114036	4.71442369	4.66789572	3.18721489	4.87362046	4.18852234	4.61407308
TRIM17	0.0938149	0.64616382	0.09636876	0.0938149	0.13177551	0.09523481	0.0938149	0.09707303	-0.01355422	-0.01355422	3.11670746	-0.01355422	-0.01355422
RFTN2	5.77165887	5.11490382	4.79782796	5.10994401	5.65293067	6.17423236	5.90674838	5.88896596	6.32798314	5.42179162	5.84821346	5.14496774	5.12377499
DISC1	0.5377717	0.40367269	0.40760637	1.00638484	0.08423028	-0.04839582	0.4473904	0.14552625	0.23703766	0.23703766	0.23703766	0.23703766	0.23703766
LRRTM1	0.03110435	0.14785234	0.03369318	0.11785024	0.05273847	0.20908784	0.27754958	0.28122691	-0.050568	3.23006345	-0.050568	-0.050568	-0.050568
MEMO1	3.68153167	3.71275494	3.94659156	4.48092067	4.89995229	4.54287789	4.84670388	4.77835344	4.29277851	3.33167223	4.4554047	4.67154985	4.30550549
DPY30	3.86500357	4.52446421	3.93978422	4.65946138	5.00216184	5.0041117	4.49557989	4.98783407	4.964058	4.66537027	4.8167217	4.13842307	4.06858557
TYW5	3.40997302	3.86743807	3.34781829	4.44889066	4.55386732	4.4318624	4.44290766	4.50108637	4.62372307	2.07279143	3.87475082	4.2747063	4.21525536
PQLC3	4.6307611	5.30259377	4.2726161	5.29667511	5.10443075	5.4229264	5.32905781	5.1874911	5.18897305	5.75517535	4.8622198	5.51497471	4.84895642
ARL5A	5.30309497	6.24150856	5.30324498	6.68016027	6.99278906	6.95511355	7.29483041	7.49077022	7.15218186	4.85635732	7.33701087	7.11526324	4.87056093
FAM84A	3.81235898	2.11768689	3.35740183	2.00624359	2.67697142	3.67085388	2.98608092	4.32650366	3.21652968	3.92303022	2.36184126	3.55262758	2.8685166
KCNJ3	3.83358092	4.95360351	3.9339545	2.66110395	4.08329436	6.14118403	2.72748524	4.49301518	5.13246527	5.32480231	5.56734014	5.60646218	5.27885474
FRZB	7.53506454	7.66040968	7.72468794	5.41845517	8.23694041	8.46037382	7.35310862	7.19337071	8.29608991	8.44307972	7.95661335	8.34371232	7.09673178
DUSP19	4.91412043	4.54211813	4.81043168	3.93360471	5.31778139	6.11135774	5.75467431	5.23399461	5.52897513	5.00156732	5.11215904	5.73638509	4.57108056
NUP35	3.69914487	4.17410167	4.3010203	4.97598695	4.68160907	4.81428298	4.31116793	4.54876728	4.50218806	3.96878877	3.99354021	4.55579749	3.99786475
CCDC138	2.39440357	2.64233112	2.93273461	3.583941	3.19693543	2.46268595	3.65819745	2.68769502	3.5330045	-1.50540429	2.3020804	3.92018647	1.74343381
FBXO41	3.7804312	3.06285304	3.95124454	2.66992695	2.55944078	2.72693796	2.70106173	2.43776042	2.45962824	3.27349829	1.90897554	2.94593529	2.75151992
ACTG2	5.74866766	6.03941895	6.05487865	4.83427189	7.37889038	7.96113157	7.07987791	8.0858622	5.63849954	8.39852489	6.22647602	8.30944153	7.90882666
SMC6	4.08109937	4.67877554	4.50655245	5.97501093	4.98952498	5.72072033	5.27545786	5.02398268	5.94706075	3.98228853	4.12832327	5.08133554	4.50649825
VSNL1	0.65046255	0.33718164	0.63635822	0.60382559	0.33718164	0.33862586	0.33718164	0.34049554	1.82505561	-0.39323933	1.80975149	-0.39323933	5.20225543
H3F3A	5.26881687	5.19726769	4.92470917	6.2799616	5.14270545	5.58631346	5.34673941	5.34564269	5.78183758	6.05881698	5.48808028	6.09676059	5.08534901
ANKRD30BI	0	0	0.00124472	0	0	0.00069205	0.27075364	0.00158797	0	0	0	0	0
SLC16A14	3.51477677	3.09702533	2.98360317	3.179078	3.28435483	4.91611667	3.2770283	3.5484092	3.99635889	3.37204507	3.95804476	3.5888456	2.50224262
SGCB	6.6587389	6.99358912	6.26749503	6.82454166	7.64804787	7.69198846	7.23555785	7.44346877	7.60838684	7.51409662	7.4561858	7.59285152	7.32382181
SPATA18	4.6021637	5.00250672	4.85403852	4.72832104	4.9621459	5.05403077	5.40732058	4.0016596	5.35319451	4.04277913	4.99562862	4.98108611	5.10531574
NOSTRIN	1.00445155	3.63076891	1.08172459	2.84620374	4.24998809	3.38653765	3.62879948	4.77312142	5.65298578	5.16261519	5.36322407	4.96104701	6.97503334

SGPP2	-0.01346409	1.40035428	-0.13496383	0.44531532	-0.09747877	-0.13544265	-0.13604221	-0.13466645	0.01662944	0.01662944	0.01662944	0.01662944	0.01662944
INHBB	1.36709816	4.22331541	3.48735075	2.78201087	4.15944324	1.71812776	2.20964681	2.2298401	2.75723279	2.66958233	4.02612315	3.7262915	2.73105185
BBS5	4.43550615	3.67049643	4.35490327	3.60085003	3.84988977	4.01055499	3.62296341	3.38928099	3.43944916	3.58792569	4.09733201	4.40740855	1.97938448
SMARCAD1	4.84105533	5.32376892	4.80721068	6.13641215	5.75574042	5.91322655	5.85308965	5.58428807	5.44643839	4.53174656	5.85488262	5.13645324	4.83426745
HPGDS	1.52377423	3.64217511	2.31429818	2.00235296	3.66365117	2.90622395	2.45867199	2.34361061	4.35072203	4.1867802	1.62086523	2.98404969	2.7110039
PDLIM5	7.91336671	8.48197034	7.49644747	7.91625655	8.84892674	8.62976787	8.37338541	8.55579397	8.40219459	8.25996777	8.69754987	8.32997568	8.56380341
NEURL3	0.16374016	-0.1639755	-0.16142851	0.41631042	-0.07625218	0.17900473	-0.07064523	0.00543764	-0.16867084	-0.16867084	-0.16867084	-0.16867084	-0.16867084
RPRD2	7.02521684	6.21189759	6.52467964	6.12887261	6.56782899	6.85702253	6.49502582	6.17433372	6.43083533	6.34105174	6.4084714	6.04594375	6.69979807
ANKRD23	2.48238274	2.10571545	1.43219882	1.12258016	2.61569926	1.81357554	1.87341712	2.03233975	2.80238699	1.82813461	3.80360906	2.80528099	1.989145
CTSS	6.10538772	6.05926681	5.39665355	6.67709901	4.81179604	4.09310906	4.91624147	4.18127719	5.6636618	5.05111528	5.45776286	4.935481	5.78596548
MSX1	4.52078524	1.98297836	4.29140909	2.09864449	2.58654489	1.27944126	3.38904374	3.91315334	3.34476435	3.58305004	3.52535646	4.52719779	1.71276055
PACRGL	3.68154866	3.93032034	4.44818678	4.87166844	4.61514205	4.8394512	4.84896957	4.88276822	4.44785946	3.79638754	4.10799915	4.65874563	4.17912515
BNIPL	1.1269216	0.07743533	0.97935664	0.80335581	0.61650945	1.00756126	0.97081373	1.11794885	0.80785629	-0.36625139	-0.36625139	1.43563299	-0.36625139
C1QTNF7	5.26177268	6.25182143	5.19169957	3.85263792	6.34263413	7.67691014	6.77990199	7.20896715	7.56942216	7.77177592	6.96412688	7.42106395	6.92141116
TNFAIP8L2	3.33645535	3.62316143	3.9787628	4.05436882	1.65230133	1.39183158	2.32337453	0.83678254	3.12573295	2.72618943	-0.95690016	-0.95690016	2.86600068
LYSMD1	4.74404116	4.17921494	4.59804679	3.43070505	4.24164655	4.9082111	4.62398068	4.41922615	4.60605798	4.40839369	4.67889943	4.45416301	3.11020025
SCNM1	4.82913561	3.82926085	4.94867739	3.91500798	4.23015179	4.75893652	3.69200765	3.87169152	4.6705639	5.17428351	4.00684031	4.18821153	5.03517726
VPS72	5.61303714	4.81890752	5.00215966	4.66342142	4.86295845	5.46963778	4.88159796	4.68252046	4.87906924	5.69576669	5.0766666	5.2643819	4.76639165
ERCC3	5.51580998	5.20132676	5.3272723	5.16091935	5.2282958	4.78302487	5.5183963	5.58477085	5.22451881	5.30328828	5.83921678	5.6080863	5.48557184
RNF149	5.4705762	5.27651573	5.10126378	6.2082806	5.10396227	4.56011202	5.84679549	5.47763214	4.99167544	4.65432176	5.18227648	4.89055319	4.63645366
IWS1	5.35800871	5.34825964	6.23051482	5.94571697	5.42127791	5.99584138	5.38143753	5.38272466	5.90653641	5.85959112	5.27023352	5.4980603	6.01850054
BOLA3	2.002859	3.38501035	2.45518747	2.36381586	2.20660461	2.78792752	2.50519407	1.44493016	3.66768389	3.69624725	1.90136712	2.8427825	-1.18310912
CDC42EP3	6.82535878	6.98318202	6.06896958	6.15706282	7.24055415	7.1700667	6.40359645	7.3939802	6.897453	7.05344737	6.9147374	7.20975349	7.23012772
S100A11	8.45016704	6.82319304	8.32393428	8.15118886	7.09353492	7.34778306	6.76020514	6.63701139	7.72319088	7.75195165	6.97261986	6.76695005	7.38095321
DHX57	3.24631364	3.73522945	3.59534904	4.38805659	3.90538651	4.37781249	3.7423175	3.75915829	3.49540934	4.0672588	4.26771705	2.98585716	3.83705731
PGLYRP4	0.05499943	0	0.00124472	0.25919679	0.07737624	0.00069205	0	0.00158797	0	0	0	0	0
ARHGAP25	4.75451484	4.36102944	4.34792905	4.51998153	3.3985949	2.89052922	2.54365945	2.9750929	4.59109863	3.56628885	4.05368238	2.93082453	4.81262296
S100A9	6.05742086	4.20750471	5.13352065	5.80755644	2.49692346	1.50815318	4.02551152	2.40077288	3.5743413	3.24137263	2.81151452	2.3887102	5.02196684
S100A12	1.45133871	-0.37296735	-0.37052353	1.18054149	-0.03960796	-0.37160862	0.41594219	0.03082201	-0.550273	-0.550273	-0.550273	-0.550273	-0.550273
TGFA	3.0791523	3.22552832	1.94415181	2.72665574	2.34416457	2.08393761	2.47510342	1.71459301	2.47782994	2.95163138	2.13073825	3.27765939	3.15901208
TDRD10	0.56738703	2.14755481	0.03833433	0.36380316	1.842916	0.53137685	1.30233544	1.63237462	1.82220476	3.21970986	2.41731421	-0.11753836	3.45868165
CCNYL1	4.31196182	5.60589694	4.83357798	5.83246842	5.34918384	4.91189104	5.2471304	5.00942659	5.35659453	4.7629179	5.4981657	4.85800633	4.80456211
FZD5	3.58687382	2.93078634	4.32060932	3.66228714	3.31902043	4.09332816	4.23930627	4.12289074	2.7660842	3.18780408	3.27118408	2.85234576	2.90047508
DCAF16	5.78358991	5.88317376	5.60505421	5.99665238	6.42873463	6.06815184	5.83785107	6.39203593	5.80436923	6.05457533	5.99767498	5.95866243	6.36216978
C1orf189	0.56354179	2.14385644	1.44987355	0.81735372	1.93779451	-0.06583685	0.64532646	1.2271754	-0.44287918	-0.44287918	-0.44287918	2.89573889	-0.44287918
GNPDA2	4.0460807	4.3073431	4.35450582	5.20779694	5.13917683	5.63107018	5.19182115	5.27847043	5.02036416	4.87830892	5.09791763	4.91081256	3.98042331
GABRB1	1.65885651	2.42765254	1.12220955	1.14255831	2.47484628	1.12110271	1.11971676	1.12289697	3.03637777	1.5630335	1.68576391	-0.88680729	1.64489168
PAQR3	3.28399172	4.10454414	3.10385837	4.27350584	4.34125222	4.81471274	4.32754179	4.55779286	4.17457386	2.27469778	4.72711105	4.49349842	3.44169015
NIPAL1	1.34972007	1.30575389	1.51793265	1.28904986	1.3039083	1.4683873	1.56432744	1.63471727	-0.70059563	-0.70059563	-0.70059563	2.4981488	1.65010818
ANTXR2	6.6173401	6.73493959	6.40319036	6.48227845	6.30389283	7.06608495	6.16573696	5.28979444	6.22460475	5.6745672	6.7212697	5.9186514	6.71533574
HELQ	4.6085397	4.64031635	4.52955803	4.76771051	4.9629536	5.33189739	4.86074961	5.05447198	4.6444187	4.53036889	4.69137219	4.67270762	4.29773123
MRPS18C	3.54928516	4.37893614	3.10933367	4.5622645	4.42510636	4.67101894	4.5525837	4.66572942	4.71935971	4.30762384	4.47714324	3.70646724	4.80366004
CGGBP1	5.92474778	6.39681439	5.7744585	6.64449481	6.92786337	7.0912567	7.17086613	6.92113489	6.73155933	5.88683855	6.85053629	6.7411974	5.79202202
GPR155	5.18190032	5.05685613	4.22442972	5.07246496	5.36109166	5.13141318	5.10735579	4.93808599	4.93856063	4.25213834	4.95077939	5.34664508	4.37562267
DAPL1	1.35668486	0.63238616	2.84043935	0.56891776	0.23986017	3.29504931	1.44807832	2.14338072	4.71525471	3.90195766	-0.47032034	3.76104385	3.67635543
PMVK	4.77324486	4.6882351	5.26551168	4.23483461	3.98550553	4.62992663	4.2300281	4.15553638	4.49295274	4.77472003	4.80153471	5.02707226	4.18383736
PBXIP1	7.29558848	6.7752254	7.33367079	5.46605267	6.63133991	7.43410394	6.75602681	6.94910267	6.95730435	7.80322934	6.87863312	7.54535212	6.59478351

CLDN1	2.09878085	5.91192952	2.16921882	5.69434128	2.63115723	2.43440882	2.21965626	2.65131996	4.11402036	2.19797723	4.79787165	3.00401813	4.56825146
PYGO2	4.98071495	4.47265934	5.08444565	3.80325691	4.54867043	4.75475437	4.33693055	3.94119477	4.16172499	5.20926851	4.71691591	5.10342474	3.84575506
HIPK1	7.01260371	7.07853491	6.24962538	6.58137239	7.23525204	7.07585176	7.0216147	6.99156712	6.61000787	6.95963815	7.14174884	6.83571232	6.91381129
LENEP	0.88063686	-0.13414451	0.20427075	0.01716078	0.36742535	0.2645319	-0.13414451	-0.13118369	-0.05747435	-0.05747435	-0.05747435	-0.05747435	-0.05747435
DCST2	0.84732949	0.41803911	0.81211514	0.20115505	0.5466682	1.19452486	0.17302099	1.47719419	-0.35916307	-0.35916307	2.33269344	-0.35916307	-0.35916307
DCST1	0.20544594	0.03329491	0.07365352	0	0.15513323	0.07953951	0	0.06628837	0	0	0	0	0
COL6A3	7.78436295	7.76233944	8.4162287	9.82938362	7.32326727	6.51418057	7.18619752	7.31909289	7.18635913	7.6765913	7.80909135	7.5581985	8.21977345
YY1AP1	6.58051393	5.97037185	6.73627123	5.99697444	6.26245763	6.11000659	6.28485335	6.31946362	6.50811645	6.92779815	5.77260194	6.28803857	6.13307596
KBTBD8	2.21338511	2.46889754	2.37555359	3.96523377	2.97759708	3.13556484	3.41848304	2.45380654	3.88715499	-1.43051158	2.85719324	2.36649914	-1.43051158
LMOD3	0.45109479	0.45109479	0.61740241	0.60527824	0.62297935	0.81129488	0.45109479	0.45441506	1.94377662	-0.18592028	0.87054932	-0.18592028	7.36138207
POGLUT1	4.42559834	5.11825799	4.41405387	5.61606106	5.14944365	5.35843913	5.51300743	5.0780563	5.06060054	4.71368773	5.09707597	4.79872057	4.42576992
SLC22A15	4.80518663	4.22183851	3.96039872	4.58519477	4.9597565	5.28646599	4.78776348	5.12861317	4.27591466	3.44851788	4.81995268	4.99496025	4.41071476
ATP1A1	8.15057189	8.03295214	7.71662346	7.81754327	7.38987159	6.63175697	7.81754327	7.03083225	6.87470745	6.81938336	7.15898395	6.81895978	7.3077813
SLC15A2	2.84670417	3.85345911	2.60998408	3.92937872	2.98501876	3.87345773	3.84539659	2.93372793	4.13610285	-1.52199653	3.47244869	2.63426465	3.31714715
EIF4E3	5.58132506	6.02957957	4.98627848	5.60598847	6.29774663	6.07848797	5.97388899	6.11789356	6.15164223	5.87783674	6.08761644	6.00421886	5.99650576
PROK2	1.55533432	0.58365789	0.58624046	0.99315805	0.81852302	0.58509376	0.58365789	0.58695264	-0.84912661	2.81691322	-0.84912661	3.13579383	3.83823166
LRRC58	5.39466661	6.08364318	5.61727004	6.35263106	6.33162156	6.52348375	6.77069967	6.5345608	6.73166225	5.4635493	6.43859991	6.55922985	5.37237705
FSTL1	6.42775005	7.44379418	6.92460343	7.81472155	7.87525016	7.95726666	7.57275661	8.45621018	7.78119136	8.20309873	7.88801799	8.50572316	7.81872197
LMOD1	8.9832563	8.72536081	8.67357611	6.58152468	8.88122351	9.337703	9.37325946	8.89215111	8.85833504	9.32538114	8.45583247	9.25393092	8.77833244
ELF3	0.71614876	0.24498227	0.60285619	0.21862501	0.40487918	0.54861413	0.14041966	0.9318048	-0.26555518	-0.26555518	-0.26555518	-0.26555518	-0.26555518
TMEM169	1.09984926	1.60961731	0.93486499	1.27682276	0.88885594	1.11863542	0.94968964	1.33348634	2.38508871	-0.52049517	-0.52049517	3.3443075	4.21800574
IGFBP7	9.4550621	9.73909301	10.0483634	8.6847383	9.24182595	9.55893957	9.51663544	8.86663227	9.85550879	9.96424208	9.41321113	9.76878409	8.9435195
TRIM46	3.65313864	2.56671051	3.57399106	2.14043901	2.35116542	1.47296944	1.95699604	0.60237881	2.44940248	-0.74232199	3.32004704	0.90327002	-0.74232199
KRTCAP2	4.57232659	3.66476452	4.83238907	4.25071556	4.56304795	3.96198786	4.86480667	4.32056116	4.26648513	5.00324564	4.12905264	4.06987503	4.57870919
CXCR1	4.87906782	1.30879189	1.2248198	1.49362701	1.49507598	0.88617197	1.05472991	0.42547759	2.75690895	2.560051	-0.94637301	2.89007242	4.86520149
ARPC2	7.23274886	7.45599248	7.16399269	8.18813989	7.66806454	7.59066939	7.43656649	7.79296681	7.68169741	7.45980737	7.42845752	7.1738789	7.79897627
CCT3	6.65517273	6.40022838	6.60492261	6.85049322	6.28250055	6.59827824	6.25717256	5.78151932	6.31653179	6.7515329	5.99591959	6.67965695	6.19288593
TMEM79	2.58545574	1.51899449	3.31570929	2.0914271	1.59317146	2.24630086	2.92600005	1.25674312	0.10917972	2.76050457	3.21834189	2.49644962	1.09816114
SSR2	6.27501941	6.17077833	6.2863935	7.14031711	5.70081793	5.61684794	5.53864731	5.56036329	5.90434024	6.56125799	5.79046862	6.44744393	5.2178215
RNF25	5.33731613	4.16090359	5.11498676	4.66337712	4.57427629	4.72217531	4.01331814	4.69764619	5.03850015	4.90568475	3.94202833	5.08909071	5.15210908
STK36	4.84725748	4.2804859	4.51754678	4.55718976	4.16406412	3.66133156	5.10750682	4.4115794	3.95942205	4.02371186	5.27190743	4.29535338	4.48748998
ADORA1	1.91627248	1.40202633	0.84741329	0.32589774	0.67485206	0.92906806	0.73028888	0.21502313	-0.37476407	-0.37476407	3.03004366	2.27653037	-0.37476407
NEK10	1.69520953	0.78403044	2.26614111	4.77052813	1.54708194	1.41106488	2.35402628	1.58496373	2.13962124	4.14232404	2.51188478	3.49236717	3.29831583
CCDC141	3.45763113	4.37547732	3.95170664	3.28614566	4.16730983	4.06375068	3.72130286	3.01401063	4.23301621	3.6663626	5.01227915	3.39460377	6.69155781
IHH	0.18355053	0.15237481	0.15724794	0.01574934	0.16313841	0.01697596	0.64063311	1.59206701	0.09308517	0.09308517	0.09308517	0.09308517	0.09308517
EOMES	2.42859856	2.37969668	1.3328892	1.87056697	2.16544517	1.33327756	1.98972169	1.72426122	2.87198329	1.34110879	2.02414907	-1.25386654	2.56426411
CWC22	5.30168002	5.63119324	5.71151085	6.37229415	6.30926119	6.80768082	6.21249824	6.14927387	6.43326562	6.03988168	5.3853057	5.5372167	5.89465886
AZI2	4.85889379	5.43644879	4.57262251	5.92663388	5.90998647	5.92626564	5.68375919	5.7401389	5.70832888	4.48094256	5.37823573	5.9299447	4.44229187
TGFBR2	9.15398654	8.22720197	8.07972889	7.97321978	7.77365565	7.7636298	8.49788751	8.40949691	7.93510737	7.76661225	8.42802802	7.74013497	8.21321461
ANKZF1	5.39335113	5.18198295	4.81444535	4.46636967	4.86131961	4.28026419	5.18540211	5.43952725	5.27110893	4.29280962	4.97749227	4.61831555	5.26662299
HDAC11	5.20229482	4.76443599	5.12291782	3.49336492	4.56081856	4.72705635	4.57900031	4.11433824	4.41252056	5.16382347	4.90407238	4.91167356	4.01659925
TRAT1	1.13555862	0.64195451	0.81354932	1.73156274	0.82426071	1.09102371	0.7228563	0.80078997	3.27751652	-0.43271938	2.48257603	-0.43271938	-0.43271938
FBLN2	4.78893536	6.53230183	4.60646092	6.3077544	5.69682825	4.22852553	5.62679305	6.03345089	5.30834659	6.21011608	5.85097953	5.94258367	6.40643727
GLB1L	4.00346244	2.91619123	4.2271293	4.45527772	3.49210699	3.00215413	2.8932784	3.00978476	3.8334003	2.5977773	2.61307175	2.25747216	4.38579778
STT3B	6.19734439	7.0787209	6.62687968	7.53238572	7.33364081	7.43612767	7.60825899	7.34049881	7.30449536	6.56649963	7.2966445	7.31823162	6.66953821
CHCHD4	4.68044043	4.05224564	3.71045911	4.25433868	3.6144804	4.80901024	4.40187708	3.68750899	3.49683499	4.68313099	3.47479231	3.48100121	4.43468841
NFASC	4.40355037	4.35200515	3.12810861	2.33979492	3.22907654	3.73601958	2.67633329	1.02646346	2.96375409	3.14678031	2.92713071	3.27942681	4.52136935

FCRL1	0.41107748	0.67608861	0.2047755	0.49842688	0.11183655	0.07912121	0.07770213	0.08095835	-0.09061616	-0.09061616	-0.09061616	-0.09061616	-0.09061616
SERPINI1	3.95851166	3.90503275	4.51139031	4.29927087	4.93092526	6.31530087	5.12123011	4.59641949	6.01603867	5.69929948	5.12390814	4.91487748	5.29242431
CLASP2	6.01433659	7.1500365	5.98044475	6.53979404	7.44660952	7.37132781	7.17585024	7.22340047	6.97345221	7.20846782	7.19756226	7.17910074	7.34641021
SUCLG1	4.99092794	5.85861871	4.77912787	5.80765403	5.32534154	6.03799003	5.83177534	5.8953886	5.47489937	6.12365141	4.86232882	5.73075671	6.79990816
NUAK2	2.68405643	2.83571608	2.2720805	3.12530658	1.78545682	1.28712549	1.14098919	0.94043698	2.42464111	1.49646638	0.54616193	1.05919343	3.70744439
SPTA1	0.39328984	0.49118475	0.39589141	0.66498748	0.43177365	0.46813175	0.39328984	0.60063584	0.47165647	-0.2750349	3.1707732	-0.2750349	-0.2750349
PRKCI	5.16954965	6.09189898	5.34996262	5.94102177	6.23175453	6.03016255	6.18927291	6.31830496	5.91089531	5.09451738	5.92824904	5.72397079	5.39551264
MNDA	4.42596918	4.07788866	4.85611153	6.75183424	3.39643547	3.42454981	3.57768327	2.74039822	5.97307018	3.65533467	2.97096925	4.10857779	5.18637312
PYHIN1	2.34128228	1.7440892	1.06086581	1.89304434	2.3678246	2.09143646	1.38569076	1.30337611	1.16309268	2.47002142	4.21976564	-0.72450201	3.55590232
IFI16	7.02494647	6.44215157	6.49977644	8.16136415	6.52100548	6.04224013	6.37471377	5.73321852	6.89459177	5.86666199	6.04105163	5.48307826	6.5633674
AIM2	1.66672624	1.657454	1.02024431	2.75062677	1.46121287	0.9480049	0.68526736	1.00209421	2.4932641	-0.89140449	-0.89140449	2.20525984	0.59126374
EFHB	1.35772816	0.43074709	0.90487211	1.36700378	0.43074709	0.8368171	0.43074709	0.62670808	-0.21656818	-0.21656818	2.66142942	-0.21656818	-0.21656818
EIF5A2	4.75705344	5.35996749	4.46173899	5.24143987	5.98185272	5.79500461	5.59236796	5.61403116	5.62356869	4.73081103	5.48871566	5.38412753	4.95480044
RPL22L1	3.49841324	3.23508887	3.83390179	5.21217055	3.53363638	3.41089869	2.91270393	3.87062922	3.05846021	2.25857155	3.7259207	3.12796776	3.23550801
PPM1L	5.50718453	4.19714571	4.24068747	4.09664849	4.07421131	5.10800938	4.73593821	3.76381481	3.30869141	4.47502605	4.49382062	3.83107472	4.06641743
ICA1L	1.24899635	1.06286921	1.45404838	1.09299644	1.60086298	1.56903497	1.37743815	1.84606506	0	0	0	0	0
CTLA4	0.99416987	1.50728746	0.95797482	1.04606235	1.6865017	0.95682688	0.95538946	0.95868778	3.22455176	-0.35594357	-0.35594357	-0.35594357	-0.35594357
ICOS	1.18826665	1.14953023	0.81457445	1.13869084	1.29946647	1.15092875	0.93480505	1.02120393	3.77468854	-0.36910747	-0.36910747	-0.36910747	-0.36910747
RYBP	5.82218447	5.747135	4.99064136	6.17076662	6.12532301	6.39949737	6.14909895	6.15241841	5.83879331	5.07073921	5.65119335	5.83340806	5.54750677
PPP4R2	2.93804951	4.05975883	3.44037865	5.12689551	4.83800976	4.91432446	5.02020102	5.11389905	4.45389277	4.54616138	5.01522259	4.81008514	4.12264029
CD200R1	1.99279167	2.73331749	1.56494723	3.29245795	2.12867751	2.07611901	2.25302019	1.46632577	2.59514563	3.01817643	1.84540143	1.29945302	2.57873187
GTPBP8	4.4032906	4.73983782	4.30007959	4.68203272	4.32503827	5.29050859	4.87702055	4.77761131	4.62065624	4.34181351	4.95305254	4.43556257	4.1674292
SPICE1	4.28546458	4.68343548	3.89046482	5.7581742	4.62542058	5.62160434	5.309295	4.96768695	4.9851053	3.7500919	4.78384349	3.60780328	4.84502656
CADPS	0.40145286	0.55021878	0.37919856	0.66646204	0.44718601	0.61242056	0.8605626	0.37991523	-0.20715492	-0.20715492	-0.20715492	-0.20715492	6.71331895
NKX6-1	0	0	0.00124472	0	0.06177155	1.15223838	0.02382665	0.06125387	0	0	0	0	0
CDS1	0.99168376	1.69758186	1.13418865	1.38142977	1.12954822	1.98368629	1.35113257	1.20273245	2.45358374	-0.67920285	3.10825765	2.49377167	1.18670352
WDFY3	7.42226105	7.76603152	6.91376891	7.40792129	7.7330671	7.86309673	7.81743268	7.74708916	7.50103079	7.57320743	7.7786781	7.74228406	7.82855967
COX18	4.20152555	4.0276803	4.79583513	4.74834664	4.26502648	4.09799765	4.02592143	4.42991112	4.45966224	4.12254551	4.29185748	4.13096157	3.90061006
PTPN13	5.10201987	6.258182	4.61899747	6.02990755	5.46448621	5.6592819	5.53157521	5.77585633	6.14706618	5.59162741	5.72809667	5.62758712	6.01567682
ALB	0.0553172	0.28079366	0.3094958	0.38213433	0.29911179	0.05675818	0.27770155	0.05862367	-0.02218742	-0.02218742	-0.02218742	-0.02218742	-0.02218742
C4orf36	3.21043631	2.58234137	2.71956079	1.89117965	2.62608158	2.8921842	2.18201457	1.76911743	2.34177054	-0.64901522	2.50911934	3.37168357	3.22827699
THOC7	4.5624193	5.23525579	5.37699735	5.59280915	5.71726308	6.08090479	5.49680536	5.07699868	5.98417936	5.78076183	5.04096964	4.96706133	5.94287256
ATXN7	5.89151419	5.55104101	5.14507579	5.7103807	6.13917441	5.61505948	5.85325522	6.37582947	5.56395968	5.85122907	5.85259918	5.79404383	6.0866227
PSMD6	4.89609535	5.54097607	4.8591244	5.89782623	5.71074845	6.04226821	5.62608334	5.38855417	5.88226939	5.58420185	5.25415102	5.61796489	5.56952714
PRICKLE2	6.5348712	6.1286627	6.26661619	6.35495073	5.94847947	6.11681834	6.37901137	5.71455888	5.99703133	5.81871661	6.31897071	5.74439618	5.90379636
ADAMTS9	4.52606512	5.10507788	5.43840842	6.40457002	5.85246093	6.23104228	5.58354694	6.89996364	4.51303381	5.45381331	5.36459595	6.00819363	6.16649935
PPM1K	5.67572937	5.98634689	5.72565596	5.24570066	6.68075866	7.12472684	7.02291319	7.20546812	6.91047911	6.31665815	6.70947791	6.39901228	7.34322693
GMPS	5.08801082	5.56429336	5.34452035	5.94164484	6.10837536	6.25948972	5.94393578	6.02347177	5.73373639	5.29905984	5.71196869	5.71224943	5.84996483
TIPARP	4.39171487	4.70522488	3.80845767	5.30820106	4.55597468	5.81076982	5.08313665	4.29121633	4.23369389	4.76179001	4.35333086	3.76316543	5.40837627
CCNL1	5.7554188	6.3691393	5.02566828	7.16188699	6.13120792	6.35029187	6.10369479	6.25761093	6.07513936	4.90219577	6.2676159	5.92586037	5.99557407
PTX3	4.73491105	1.7993663	6.16474697	6.57261475	2.21710472	1.91793678	2.6682637	2.21642618	2.69634262	0.77217154	2.66336689	1.5516166	2.50225734
HESX1	1.27735308	1.5086113	0.90226172	0.78667191	1.00599497	0.57963947	0.74457275	1.69172044	2.61449964	1.82650065	1.33814538	2.06378199	-0.66888563
SLMAP	4.00935196	4.62471719	3.92318673	3.93128666	5.76973772	5.91916872	5.05136406	6.01964891	4.49034149	5.1655878	5.09933808	6.05568887	4.5719009
RPL9	4.01978939	6.75026825	6.95328299	6.87206363	4.70794239	3.87602492	4.56807225	6.31789024	7.04960566	4.00728495	6.43045786	7.50448273	6.47777053
RPP14	4.90380645	4.76823651	4.41868031	4.57843458	4.74226041	4.78891804	5.21504835	4.50481476	4.46019137	4.53543994	4.37620701	4.63305286	4.54650181
ABHD6	4.81068285	4.05895222	3.97730721	3.4031042	3.53675364	3.47327417	3.99950518	3.90495723	3.71810621	1.87556432	4.61875576	3.33165117	4.45569621
DNASE1L3	2.49534111	3.81708716	0.24400196	1.01644918	2.82512917	1.88850939	1.3841993	1.75352592	5.15307417	-0.56531022	4.98853553	3.70885051	4.6948688

C3orf67	0.33203287	0.29662298	0.35626803	1.1675766	0.30851401	0.37205184	0.59303889	0.28926169	-0.23885257	-0.23885257	1.96630851	-0.23885257	-0.23885257
RBM47	5.0763383	5.00938378	5.20565989	5.25497209	3.45486274	2.66539843	3.74408003	2.89840592	4.06712077	1.26974164	2.68894987	3.06505723	3.86866157
APBB2	6.29191849	6.56447021	6.19866044	6.25547884	7.32787315	7.25508572	6.70401749	7.38053833	6.72989342	7.04609725	7.24186954	7.38893019	7.23757324
IL17RE	3.71870993	4.18582068	3.67468336	2.42080912	3.41341412	3.47609691	3.17442826	3.0623393	3.52957409	3.20499887	4.23232528	2.79152358	3.67704106
IL17RC	4.83585844	4.44503506	4.76831162	3.2217915	3.61769925	3.67863815	4.27060304	4.52401356	3.68078611	4.27861771	4.66978028	4.77610212	4.27105295
CRELD1	6.01280524	5.59239177	5.58501076	4.64343392	6.20157451	6.21941099	5.80792183	5.22024647	5.43242502	6.24985333	5.97006116	5.86050128	5.24430976
PRRT3	3.30960725	2.57508778	3.36914881	1.96654939	2.00284373	2.87162947	2.40482597	2.60912419	2.02157574	3.49361577	3.95856397	3.02645006	3.08507929
PCOLCE2	5.3207371	6.18051463	5.45785082	5.70519346	5.24712275	4.12684307	4.97109058	6.33548407	6.12701405	6.52171251	5.12452095	6.44301596	5.96945726
U2SURP	5.75661431	6.50830077	5.9680007	7.00333078	6.9228723	7.00987436	6.74198698	6.82704966	6.79206881	5.90709272	6.47362274	6.66477316	6.12234475
MTMR14	4.95075174	5.08908288	4.8062983	4.68726135	4.46092547	3.95872015	4.56141042	4.95568042	4.40082074	4.23826259	4.99946493	4.44156416	5.10681516
TTC14	4.60510265	5.82345195	4.99773271	6.08456307	6.12660515	6.57374294	6.23428055	6.4244071	6.42750467	4.22031228	6.46903154	6.01463202	4.71419462
CXCL3	2.60109634	1.08867563	1.92593644	2.77463741	0.40286239	0.40421205	0.40286239	0.40595932	-1.07612282	1.5365385	2.55561165	0.16019714	-1.07612282
CXCL5	5.88694135	2.6764411	3.23610357	1.49079306	1.29782112	1.17850025	1.22315433	1.18019523	-1.21492296	-1.21492296	-1.21492296	-1.21492296	-1.21492296
PPBP	4.55460908	0.19428949	1.12776144	2.23919431	2.11149111	0.1955863	1.21944251	0.94257125	-1.31489859	-1.31489859	3.08550921	0.41495258	-1.31489859
PF4	1.93921681	-0.38336448	2.28245477	0.28376962	0.52892811	-0.38218741	-0.38336448	-0.38066357	0.81738686	-0.26709914	4.66722058	-0.26709914	-0.26709914
MTHFD2L	2.74228368	3.48482254	2.81294962	4.34454706	4.0955227	4.45697107	4.03362463	3.80052825	4.13196363	4.35856431	4.09277542	2.8562046	3.75158909
CXCL1	4.20464447	4.7982543	2.49323122	4.8865917	0.98724365	1.65841353	1.58646645	1.54050745	2.64993869	1.23338693	2.32313532	-0.38721373	2.97847751
RCHY1	4.03623935	4.99441968	4.33828762	5.22205143	5.19318016	5.30166462	5.26245772	5.32154893	4.59144048	4.82825842	4.97245762	5.37663654	4.69322921
PLSCR2	1.37731329	0.96927501	0.98024669	1.94560833	0.90225213	0.85381626	0.71199352	1.20737018	-0.50598163	-0.50598163	3.19696755	2.24977076	1.99845731
CCDC158	2.74202946	2.82907381	2.02248938	2.10449004	2.80625827	3.43306081	2.2693342	3.55870404	3.90200076	-1.05310819	3.57304138	2.59169588	2.27064618
CPA3	2.05761263	5.32270316	0.9451346	2.55783412	4.07641495	3.37525266	4.46975922	1.56986256	4.00513301	2.80172898	1.46060363	-0.34669759	3.51268005
GYG1	6.45363297	6.61732675	5.74716366	6.088689	6.51734897	6.7030954	6.84088933	6.44431138	6.18930627	6.78869059	6.47659052	6.91641278	6.45703434
HPS3	5.24785139	5.24591755	4.89954636	5.73533555	5.77752675	5.82350953	6.00881176	5.74735919	5.71442439	5.32694399	5.4855242	5.87307258	5.69109943
TM4SF18	1.4726577	4.48941589	1.75934612	4.51571355	3.02695489	3.13445493	2.56196217	3.08539637	4.15366385	3.79969346	4.76960645	4.23286092	5.47612613
TOPBP1	5.08474851	5.29804129	5.09665577	6.1369261	5.41354145	5.48033601	5.55413926	5.48701697	5.55723854	5.2287844	5.50731435	5.45861608	5.67216151
RYK	5.3254684	5.51003941	4.74414646	5.29556387	5.57797832	5.75084984	5.68920342	5.80760302	5.50872533	5.5382692	5.79883943	5.72270728	5.08144144
SNRK	5.44339961	6.17450569	4.74646111	6.08776181	6.2494671	5.95891692	5.72722858	6.04234608	6.04419071	5.08528159	6.19959061	6.06426014	6.404894
TCF23	0.04387545	0.49377875	0.00124472	0.04519482	0.02115212	0.00069205	0.06532363	0.06489215	0	0	0	0	0
DNAJC5G	1.26730797	-0.0031771	-0.00073025	0.07920875	0.59391033	0.52186817	0.29687565	1.12885746	-0.1502589	-0.1502589	-0.1502589	-0.1502589	-0.1502589
UCN	0.91815212	0.12822489	0.54856174	-0.58119123	0.7947	0.16951717	0.48902275	-0.5852431	0.03959079	0.03959079	0.03959079	0.03959079	0.03959079
ZNF513	4.34428416	3.82944817	4.13449857	3.60930896	3.44341719	3.44341719	2.46521546	3.30819478	2.79451566	3.47236559	2.18387173	4.02267865	2.98208405
SLC4A1AP	5.05275783	5.00220533	5.64190195	5.39940313	5.2699044	5.74259871	5.65955031	5.01383402	5.86027479	5.58916507	5.26205467	5.04589341	5.5403708
PLB1	4.0233997	4.74598786	4.63285673	4.08968227	3.92053108	3.14313569	3.32144189	3.44692886	4.08529519	4.78302744	3.27091775	4.49794132	4.18841379
SPDYA	0.9317303	0.71411734	2.36134845	1.12043857	3.25470031	2.77522759	3.30846406	3.87264637	2.46815314	-0.67425609	3.33478642	1.46051999	-0.67425609
KIAA1143	5.6381439	5.77076269	5.23759802	5.48674481	5.8145986	6.29052997	5.66204133	5.58947787	6.18019814	5.86055172	5.78864322	5.92974495	5.15792216
KIF15	1.27069299	1.58399322	3.26508125	4.39496252	1.39701225	1.39701225	2.13297697	1.43687929	3.3272992	3.0077594	-1.00258285	3.28102887	2.76305939
WDR43	4.18204148	4.70529765	4.01764684	5.72899976	4.9919854	5.40095121	4.58927787	4.48597111	4.96956774	3.76826542	4.01667078	4.24944948	5.2835731
ZDHHC3	6.03530844	5.81863814	5.78986237	5.69276913	6.1058823	5.84058404	5.75082151	6.23605405	5.67614974	5.67090533	6.04193477	5.81520453	6.32509395
CDCP1	5.56284884	3.37699785	5.19398598	5.03719135	2.29721649	2.7924212	2.62947287	1.63816195	3.52127823	1.22595687	2.23995054	2.47095352	3.32279901
CLEC3B	3.43086532	5.17040734	2.63935428	2.2549408	3.00453881	3.72028518	2.92823445	3.44586993	4.38302379	5.09066982	3.75204994	4.58926888	4.55713053
LZTFL1	4.50039034	4.9066843	4.14575492	5.3499167	5.20908285	5.61957449	5.40369071	4.86898664	5.59181573	4.22326234	5.0394654	5.30402499	3.85655858
FYCO1	7.4356667	7.38968438	7.33878788	5.9843397	7.21813794	7.38281248	7.39771292	7.12231133	6.98108352	7.86192248	7.41607677	6.98108352	7.6454912
CCR1	5.28343591	5.07372076	5.25848084	5.68768743	3.19333699	2.92517954	3.42717861	2.30427888	4.19642529	3.15156855	2.33798408	2.48397824	4.45170799
LRRC2	0.71283522	1.12713663	0.70460112	0.95858361	1.09396606	0.79394949	0.86593106	2.09028263	-0.40419334	-0.40419334	3.17952137	-0.40419334	6.28830707
FBXO40	1.28254473	1.25953457	1.26213856	1.25953457	1.32004378	1.44247376	1.25953457	1.6480999	3.30241949	2.83941129	-0.62857429	-0.62857429	8.2290359
DTX3L	5.93863309	6.45686087	6.10956807	6.83992036	5.92231701	5.16649775	6.05646892	5.07746221	5.73896873	4.97403294	5.93689071	4.96005342	6.24745016
ZNF148	5.9567411	6.42455603	5.81496675	6.51960221	6.72750046	7.13253579	6.88123007	6.68185362	7.24635755	6.46648573	6.81479726	6.88338566	6.22102336

NMNAT3	4.04781955	3.8915638	3.18238209	2.4636815	3.32785602	3.78236679	3.52124198	4.3666658	4.08825066	4.52279594	4.00454185	3.77538495	3.65924261
ZMYM6	4.91155086	4.93603757	5.30461483	5.46554179	5.50798536	5.49803218	5.47088804	5.66419248	5.38313434	5.21599741	5.29714923	4.87200703	5.17901989
TPRA1	3.98018351	3.63281913	3.76403158	2.44147299	2.96958701	2.27903118	3.62159871	2.86238734	2.38239763	4.06416497	4.15515157	4.38046188	2.49638532
YEATS2	6.63811741	5.81720325	6.03610892	6.63639565	6.05104144	5.71831469	6.06585456	5.80151036	5.86644878	6.06864783	6.10763479	5.55771291	6.27817788
GRIK3	1.11303728	1.3710166	1.10804416	1.10545096	1.30007833	1.63324009	1.80912581	1.77131648	2.10452696	3.49467888	4.31950563	-0.67760326	4.58192694
ZC3H12A	4.64732673	4.554589	3.99016978	3.97102808	2.64277038	2.92114663	2.57372455	2.74900874	3.70078206	1.85944333	1.16231982	5.0236888	2.50096511
MEAF6	5.59856164	5.64786514	5.39867118	5.2800378	5.74426136	6.12143584	5.28798891	5.64821982	5.47039183	5.77136322	5.2037499	5.68222843	5.24427788
SNIP1	4.97699131	4.95184973	4.68119313	4.40219686	4.96217	5.31626772	4.71236205	5.26185257	4.88603086	4.72522962	5.07205397	4.86050065	5.20999756
DNALI1	5.13448849	3.9665435	3.83924893	3.98394995	4.01937917	4.42146135	3.99659476	3.9310259	4.81631161	3.9078064	4.28298707	3.36211565	4.17270359
POLR2H	5.16905458	4.66797812	5.58232725	4.92023426	4.79663186	3.93639007	5.58568165	4.8638437	4.21420475	4.37933184	3.88349279	4.73348758	4.53551289
KLF15	5.1259262	4.87970156	4.80756504	1.89601924	5.12005144	5.66532871	5.39815225	6.01495304	4.64800495	6.00375658	3.80134267	5.74304091	4.64510263
CAMK2N2	0	0	0.51653791	0.26910702	0	0.00069205	0.26920785	0.16564332	0	0	0	0	0
LIPH	2.29372101	2.51303076	1.50920674	1.58514004	1.67844539	2.51822488	2.80892113	2.49432016	3.50893399	2.34077217	3.92377343	-1.00796288	1.57181207
TMEM41A	4.76473814	4.77795075	4.58948601	4.16802773	4.54489297	4.2991337	4.54811144	4.39122938	4.204828	4.28016	4.42099776	4.1963095	4.63434719
RPN1	7.02904847	7.05072523	6.97053604	8.16347561	6.28986072	6.79479918	6.74494589	5.99877213	6.51903708	6.95442189	6.7566477	6.83428241	6.98621658
SENP2	4.73217106	5.16584919	4.53245615	4.86019724	5.20964352	5.06453878	5.47204796	5.33850673	5.35024698	5.45363219	4.9045558	4.9856107	5.19374948
HEYL	6.81591814	7.45139788	7.88322959	6.21965493	6.99850719	6.93502588	5.89136689	6.59853604	6.37929048	7.08409714	7.09714929	6.56637359	6.60198817
IFT122	6.05227441	5.28839934	5.90769791	5.44642672	5.38889369	4.65632861	5.55978734	6.01786596	5.31939252	5.14971742	5.287773	5.09727531	5.87883463
RFC4	3.7805778	5.09122747	4.16013739	5.03969845	4.46639908	4.28776315	4.52334522	3.59941958	3.89587006	3.81412747	4.04432292	3.87600281	4.47390418
RPL39L	0.26350898	-0.36503525	1.18571723	0.61733096	0.87408494	0.9902929	0.47372947	0.43888738	0.34604965	0.34604965	2.01293613	0.34604965	0.34604965
BAP1	6.17140306	6.07696601	6.0604972	5.41461977	5.51632478	5.24292846	5.50887319	5.53030896	5.57145617	5.91445984	6.09554351	5.86180315	5.50691035
TKT	6.80859881	6.01499065	6.47326459	6.07735337	5.78809311	6.05829099	6.82247934	5.52633296	5.76600118	6.39067289	5.78340186	5.17622977	5.75118731
PRKCD	5.71875517	5.29160778	5.97888853	5.1339914	4.88356032	4.94994934	4.90892512	4.76879166	5.40767391	5.63579803	5.29581572	4.72959038	5.29153952
RFT1	4.94759906	5.09105023	5.02629704	4.77740686	4.74211462	4.23100832	4.66693706	4.50135783	4.58923472	4.49394085	4.51384232	5.13593292	5.24308142
SFMBT1	3.99587373	4.64214634	4.72201742	4.58026124	3.89275152	4.66460994	4.84046481	4.80623662	4.34926153	3.19564823	4.5574088	4.72706808	5.0116816
GNL3	4.45047657	4.49226343	4.38250606	6.07274096	4.98308031	4.50726088	3.8056024	4.41045694	5.12657742	4.69008114	4.97233143	4.8290062	5.33571058
PBRM1	6.1053443	6.5225809	6.27944336	6.88929252	6.84813731	6.89535122	6.76974829	6.74100146	6.58919907	6.42419883	6.78212074	6.49239142	6.82210828
FAM208A	6.10075764	6.88644649	6.22162464	7.20035471	7.12556614	7.15732076	7.14504559	7.12178876	7.02423098	6.56399864	6.68458825	6.60007163	6.57819372
ARHGEF3	5.63811327	5.87453471	5.22652158	5.53740164	4.60234763	4.07464214	4.76282957	4.408261	5.01856375	4.43768835	4.78635128	4.14287395	5.36784699
SLBP	4.42696033	4.89494881	5.30319413	5.61847818	5.14582457	5.26345883	5.12618819	4.97423227	4.94310685	4.0705805	4.40992716	4.98444042	4.62052618
LRPAP1	6.25503227	5.79349464	6.31203152	5.21565854	5.4810123	6.22450569	5.49747004	5.73403117	6.26184847	6.42896461	6.06822596	5.82519022	5.90014035
ZDHHC19	0.40171417	0.01746836	0.02003066	0.28128921	0.08956253	0.01889296	0.36300396	0.02073725	-0.07454162	-0.07454162	-0.07454162	-0.07454162	-0.07454162
UBXN7	5.82359657	5.86701824	5.96321808	6.27910499	6.23797744	6.27550964	6.48338662	6.44556337	6.14366305	5.98058487	5.78104476	6.10988775	5.97865575
RNF168	5.92412368	5.81837884	6.0095198	5.43915837	5.79567058	6.08116229	5.64391519	6.37134319	5.8689296	5.71995995	5.30564984	5.66469776	6.1121195
PIGX	4.36129624	3.70940222	4.78720098	5.05694964	4.85877941	4.8536911	5.05123016	4.47265284	4.79814698	4.0652988	3.71155759	4.51960815	3.45506665
ABLIM2	3.89363884	4.11649393	2.56238942	1.73837932	3.6217112	4.01807967	4.21057448	3.90050807	3.69922709	3.48824987	4.40281321	4.21074502	3.76638344
C1orf50	3.26328853	3.44466454	3.43300554	3.40040599	3.13743635	3.21486932	3.99060542	4.27483871	3.33539801	3.83146947	2.28212315	3.81855265	2.99133308
ERMAP	5.07689303	4.9949568	4.82701824	5.13794904	4.65836725	3.92342788	4.72665881	4.70133261	4.3155421	5.11719196	5.35121271	5.13562955	4.64211904
ZNF691	4.85054068	3.36266006	4.51068703	3.87608279	4.06140901	4.37210734	4.03214651	3.73128945	4.3616428	4.65105923	3.67531965	3.61153576	3.86052475
AIMP1	4.6534303	5.01120893	5.29555304	5.50743651	5.59885986	6.03541128	5.41443665	5.81883502	5.96586577	5.18973933	5.44744035	5.46933416	4.91549164
SGMS2	2.66014897	4.50774756	3.12203027	4.76697541	4.65546987	4.4194053	4.36251922	4.75133149	3.53513402	3.67230969	4.169474	4.52811241	4.79408208
METAP1	2.59093805	3.13807416	2.8774043	3.50567642	4.39983994	4.48347246	4.76394247	4.20741581	4.27981005	3.23703752	4.09577794	4.28854178	2.66385344
DNAJB14	5.51335249	6.30305852	5.55791954	6.57095437	6.56805844	6.78384213	6.73478221	6.65719483	6.84866518	5.86034474	6.58056612	6.67211015	5.5109326
H2AFZ	5.05332904	5.16807302	5.93131535	6.41803317	5.31140049	4.81849813	5.46480484	4.66176118	5.55232662	5.61832107	4.49059111	5.46790225	5.08566053
EMCN	1.31448452	4.69169224	1.95918517	4.9693801	4.3809092	3.24481782	3.86631685	4.29250001	5.6974957	4.07113588	6.2844154	5.06683019	6.60920148
SLC9B1	0.50985256	-0.67935683	-0.16575775	0.36357352	0.48799427	1.1417714	0.18033849	0.69999374	-0.04637891	-0.04637891	-0.04637891	3.10964215	-0.04637891
SLC9B2	4.46750144	4.84589187	4.40325212	5.56794518	4.81688415	4.70568159	5.20199338	4.57450753	4.986546	4.47600194	4.72574509	4.52113967	4.50831057

BDH2	5.22812605	6.03191628	4.86166753	5.46289126	5.5769888	5.43488518	5.30205965	5.73798543	5.80227172	5.98669671	5.53525597	5.78570239	5.37044909
PGRMC2	5.75134164	6.00561955	5.53184396	6.27008189	6.51898991	6.49373001	6.57938133	6.40118687	6.33544171	5.8654261	6.49217402	6.51326175	5.55219401
CDC25A	1.46317795	1.05286443	2.84519091	2.58506549	0.8888446	1.22935624	1.83165506	1.00905315	2.67400489	3.02836505	0.23812593	3.59914113	-0.82557871
ZNF589	2.86644093	1.21973658	1.15204882	0.42175077	1.68054847	1.93015437	2.23493192	1.59564572	-0.19975255	4.45487603	-0.19975255	-0.19975255	3.45129428
FBXW12	-0.07511605	-0.10527974	0.03293887	-0.10527974	-0.06309955	-0.10401872	0.0289285	0.543769	-0.03162127	-0.03162127	-0.03162127	-0.03162127	-0.03162127
PLXNB1	5.86609229	5.98637541	6.12962831	4.16136698	6.3836756	6.01405446	6.53049557	6.64177454	5.36019547	6.8560669	5.89605376	6.22392322	5.99854995
CCDC51	3.76226956	3.85474868	4.32221403	3.13548881	3.0948299	4.24336494	3.14551054	2.65551394	3.8967262	4.5375849	3.4793295	3.91118475	3.87143265
ATRIP	3.16148218	2.69278871	3.21784041	2.88960329	3.19463014	3.47258702	3.30985052	3.39145872	2.03670633	3.64380137	3.00695093	3.99435644	3.17465674
SHISA5	6.59744364	6.40032626	6.81509532	6.25095469	6.15542708	5.89354821	5.75881241	5.71106547	5.78774808	6.57599496	6.36236596	6.17030442	5.66709228
SPRY1	6.54352539	6.55853193	5.13697472	6.18272138	6.22328038	6.38128536	5.60892115	5.85204721	6.44664132	5.03153938	6.46393531	5.58852116	6.36476095
BSN	3.00094765	2.17789841	3.16777906	2.29172766	2.50188033	2.78569376	2.47152741	2.3886586	2.06564245	2.40826524	3.45110203	2.98321287	3.23829448
APEH	5.92226714	6.13084946	6.22647452	5.41997174	5.47765623	5.35900058	5.63327581	5.39643917	5.22873096	6.02113178	5.63520463	5.53768091	5.97070398
INTU	4.1386482	4.12160044	4.14571054	4.30049841	4.92620576	4.97681118	4.67440872	4.46453341	5.01081488	3.9917122	4.83031075	4.51307779	4.00345966
RNF123	4.67901464	4.12873637	4.85741316	3.66295212	4.36797459	3.81510041	4.55878243	4.370394	3.63504343	3.73074408	4.15758472	4.68817263	4.67711859
HSPA4L	4.51471406	4.44185626	4.26884811	4.39507465	5.04828846	5.85086183	5.37462291	4.95516061	5.12306505	4.92543926	4.71357239	5.29641935	4.79380777
MFSD8	4.03785021	4.45199935	3.80766262	4.59202924	4.92563634	4.72749963	4.89882642	5.13539609	4.39214325	4.76774866	4.94442681	4.86255971	3.82912365
MON1A	4.43192685	3.56061189	4.75035898	3.62696461	3.60062404	3.93387418	3.82161821	3.3470438	3.86046032	4.1261296	3.77531196	4.38656056	3.43247067
MST1R	3.23767361	3.61155454	2.02391041	2.11458606	1.48839497	2.16421782	2.27208135	2.50310719	1.67225547	-0.8393213	3.57372619	3.20699046	2.638961
RAD54L2	6.00814912	5.79751651	6.20807284	5.51898133	5.80416739	5.41779031	5.63301165	5.76632542	5.4894384	5.4479805	5.95452853	5.76770527	5.58807244
TEX264	4.46007663	4.52572338	4.59566293	3.64051902	3.35927117	3.39693671	3.34410674	3.23656691	4.04124428	4.60150313	4.22030129	3.86350069	4.04347022
GRM2	0.68076972	0.45878824	0.54615926	0.49834236	0.26896812	0.2828879	0.15678433	0.40717141	-0.05462349	-0.05462349	-0.05462349	1.05021368	2.55931764
DUSP7	5.51425789	5.92361991	6.76811232	5.27893086	6.10005935	6.08376347	5.81425472	5.58510835	5.3252292	5.78050819	5.67387718	5.5200832	5.39432141
POC1A	2.34584544	0.62248445	4.48888965	2.69449534	1.00617918	1.29086023	2.38865451	1.45756041	2.13749843	-0.89959869	-0.89959869	2.42869755	2.75639054
PPM1M	5.44066202	5.87334324	6.05502395	5.09958931	5.10101123	4.75088237	5.40065272	5.11362915	5.32368236	5.79361039	5.51651355	5.96201622	5.65036514
WDR82	6.58388531	6.61043984	6.29875778	6.77914952	6.45939202	6.12493524	6.91226665	6.54947537	6.11078582	6.84334644	6.54869121	6.45871129	6.9844374
C4orf3	6.18007944	7.34234984	6.22935014	6.84824887	7.4036764	7.49594903	7.12216974	7.12388693	7.32713953	6.47536647	7.35211381	7.21825948	6.1918611
PRSS12	0.9917484	0.97754369	1.07171766	0.99886661	1.50500984	1.23992313	1.18561217	1.59398583	0.91003432	1.83412807	2.74387617	2.50379452	0.56103483
HMGB2	5.1666176	3.90585292	5.67691499	5.22787937	4.2674328	5.63569249	4.21520605	4.85582623	5.62858871	5.68412534	4.76212909	4.10814478	5.37984424
SAP30	3.55517512	2.6322447	4.34088538	3.668524	4.32047854	4.43611187	4.8150834	4.47570182	4.52705772	4.10259861	2.55543947	3.83309976	3.12709752
SCRG1	4.59687248	4.37922431	5.44389058	5.53583446	5.87908752	6.82574243	6.35962786	5.44675122	6.02428814	7.18198955	4.51289677	6.63020942	6.18306277
HAND2	5.9661678	5.38498784	5.43750138	3.44498533	5.70239972	4.79846918	5.80395151	5.74970683	5.74903112	6.45036046	5.93540708	5.76381114	6.32827848
MAD2L1	1.82132296	1.47749616	2.98654393	4.34677602	2.28500346	2.49754205	2.60108986	1.95527825	3.23566228	-1.30306938	2.56117598	2.83539868	1.8295277
ANXA5	7.71059376	8.38007882	8.09049167	8.95846251	8.66175474	8.71244466	8.67231296	8.79413277	8.4169299	8.33250212	8.58826826	8.58001209	8.79566962
TMEM155	0.5115403	0.3929294	0.76682169	0.21829603	0.26299483	0.21973312	0.42822778	0.22159358	3.41894786	-0.16836109	2.75120691	-0.16836109	-0.16836109
MAP9	4.60709009	5.00193624	4.25372654	5.14180109	5.31598289	6.1668855	5.45479831	5.3464126	6.00162291	4.63365759	4.36344255	5.46220099	4.66030507
GUCY1A3	7.11610487	7.91810725	6.00497112	7.93365932	6.95407825	7.70846622	7.59571386	7.25141172	7.83591506	7.46200348	7.93979035	7.73635049	7.62770405
FBXO8	4.80184023	4.83704913	4.70819048	4.92351997	5.26663435	5.91979197	5.31360464	5.36219307	5.80527377	5.39498631	5.66486541	5.32963916	5.24573605
CEP44	4.30637094	4.72395725	4.63565256	4.7477107	5.0258743	5.27297648	5.32926231	5.54029521	5.29801208	4.41783851	5.22628381	5.4799814	4.42061752
HPGD	2.01875434	2.98707698	1.44923382	3.17526284	2.0040282	1.98212388	2.92356073	1.5653691	2.30367837	2.52974428	3.4742981	3.06203294	-0.98179296
TMEM144	4.19217801	4.11392947	3.87224206	4.4796275	5.62054956	5.47516069	5.57833426	5.13692284	5.298916	4.52438692	5.65803069	5.08760337	5.36418424
FAM198B	6.80900649	7.53991604	6.86226318	7.52461286	8.38536215	8.37916679	8.08725454	8.30963623	8.20974943	8.27787657	8.16514766	8.33239876	7.84131742
NPY1R	4.11202399	3.87576338	3.87768029	3.58342541	6.53841035	7.31822794	6.34097967	6.43960828	6.399712	4.99239266	5.86322948	6.83574545	5.39782451
NPY5R	1.53095243	1.76649947	2.04813181	1.62827911	3.17724084	4.39264911	3.00853707	2.98063662	4.15467666	3.56032232	3.69293346	4.33640155	0.40302016
NAA15	4.81215139	5.95097264	5.35016934	6.91693699	6.4461136	6.53881212	6.08211967	6.21305233	6.15488968	5.34459801	5.84739687	5.64251153	4.93020816
IL15	3.63985295	3.93195724	3.05393202	3.50264567	3.77077252	3.22975311	4.40104953	4.04880844	3.9989451	3.39550393	3.66415525	3.75811457	3.13753111
FAM160A1	5.23566143	4.66206934	3.98916789	3.47436806	5.26511872	5.46629584	4.73671229	4.80950001	4.46982924	3.73531526	5.36930649	4.07077163	5.43293115
ARFIP1	2.14360105	3.16631394	2.15458971	3.81673518	3.79612646	4.46844466	4.05005159	4.08323655	3.4087609	3.29607562	3.77292591	2.80227554	3.4920564

HHIP	0.32842044	1.22871063	0.42894573	0.56249714	0.45387011	0.71678149	0.40441891	1.46332422	1.08668321	-0.14404634	3.25123883	-0.14404634	-0.14404634
ANAPC10	3.77525499	4.17724873	3.1153049	4.4720968	4.47085535	5.01140304	4.40322909	4.38775671	5.20537574	4.02632591	4.06584086	4.2007577	2.09039334
ABCE1	4.95746479	5.94877404	5.26082811	7.00712623	6.28498573	6.48638922	5.82559092	5.80408051	5.94323678	4.63933138	5.81323718	5.48615644	5.08834941
OTUD4	5.88244646	6.28263665	5.87992017	6.78045153	6.82894221	6.78472651	6.93086467	6.63202537	6.40243206	5.8242519	6.06284408	6.03528849	5.99482862
LSM6	2.26827912	2.60769982	2.17625497	3.35403327	3.7489887	3.39040207	3.208423	3.7118173	4.03393723	2.99238762	2.88556051	2.59612192	2.19612244
TMEM184C	4.47564807	5.56342235	4.45427504	5.23028286	5.22356369	5.64541206	5.80237543	5.0582608	5.40755259	4.5881395	5.27052531	4.80347397	5.0702596
ITGA2	5.50345568	4.50000951	6.49481057	6.95738539	3.90871331	3.98777021	4.54239265	3.87639013	3.9838754	3.36710615	4.9452047	3.98188676	3.77887774
MOCS2	5.14525506	5.60218855	5.25158876	5.436619	5.86912789	6.14505346	5.60236247	5.768702	6.11222369	5.72326987	5.53700596	5.90364726	5.51916287
SLC45A2	-0.20438036	0.24307607	-0.15132574	0.52011875	0.14746059	0.02172421	0.22417948	0.03381043	0.00268368	0.00268368	0.00268368	0.00268368	1.7231964
EDIL3	7.40322941	8.37155668	7.6931124	8.39231415	8.24556734	8.12393657	8.70636515	8.5448696	8.57423276	8.16021563	8.33996484	8.59081336	8.02167594
TMEM161B	3.69559651	4.40491807	3.78313295	4.81886266	4.62533114	4.83993877	4.76419764	4.72599321	4.8712148	4.19531331	5.12165576	4.53042777	3.40911153
ELOVL7	1.38147261	2.50273329	1.3688865	2.06812497	3.80847644	2.84168249	4.08730838	3.52578488	2.40737627	2.58030867	5.04688004	3.67238164	3.81064319
NDUFAF2	4.35798577	3.24574134	3.27625747	4.54218312	3.50586141	4.4427813	3.11571263	2.74785802	4.79585638	4.04655162	3.49254565	3.70245076	3.23421133
ZNF474	0.63447297	0.43475048	0.73894886	0.72297335	1.01034955	0.71214078	0.5233616	0.42915136	-0.25234501	-0.25234501	-0.25234501	1.78170399	3.19954669
LMBRD2	4.34470176	4.87859833	4.36593049	5.11212605	5.34957006	5.92597237	5.60658692	5.45686306	5.57184818	4.34373285	5.29833646	4.96991028	4.58063847
RANBP3L	4.4683434	4.4148854	3.73304729	3.77506978	5.15974462	5.98283865	6.06362565	5.56744025	5.60097257	4.23131919	5.71027114	5.15971308	4.22312629
NIPBL	2.38641588	3.09871567	2.03258876	3.54072729	3.73915056	4.3130854	3.47402549	3.79324222	4.16090851	3.78191194	3.54365539	3.1915324	0.79670498
RNF180	5.71187891	6.02687808	5.18565824	5.15678786	5.99432378	6.66647418	5.97952601	6.21901076	6.51996692	6.15315106	5.96632945	6.12095672	5.72210763
SLC25A46	4.90933068	5.54318351	4.9907828	6.18145242	6.05423852	6.15977368	6.15279849	5.97928858	5.86442139	4.51696712	5.98959435	5.72559018	5.14817954
STARD4	2.75109085	3.46506129	3.24699308	4.84221519	4.62078929	4.47924505	5.26612196	5.06480677	4.22132957	2.48842143	5.14845124	4.39260687	2.69167034
PGGT1B	4.41049832	5.15466904	4.23556356	5.48677478	5.35695671	5.6894343	5.34567451	5.43085551	5.5785372	4.94378742	5.33880615	5.45529761	4.00404493
F2RL2	3.52772062	2.61473699	2.60381147	3.666866	3.70223113	2.74942569	2.4588522	2.98355536	4.02453111	1.09230871	4.01486264	4.10887946	4.13766585
CCDC112	3.9729195	3.86011734	3.80457623	4.12006317	3.5944601	5.52725905	3.73069659	4.34783336	5.43619497	4.06751077	4.12553377	2.78440682	3.9834437
ANKRD33B	4.61929958	4.47858981	4.85559091	3.21938275	4.20856262	5.04284719	4.57327497	5.68854652	4.47954322	4.23537266	4.83063096	3.47499598	4.74198519
CMBL	5.33276952	5.44387849	5.26316547	4.21034703	4.68170085	5.25346074	5.73490425	4.45208998	5.35636339	5.52960797	5.08913735	5.61276567	5.63540397
C5orf63	2.70319271	2.87858646	2.55294208	3.25468113	3.43175104	4.54612819	3.09679888	3.38755145	2.1697501	4.27408955	3.75860143	3.18095432	3.54831272
PRRC1	6.27631921	6.37770556	6.01113602	7.26541196	6.33250825	6.43257127	6.29581504	6.34412566	6.54738369	6.34279461	6.81970645	6.63807217	6.30175159
F2RL1	3.10235158	2.17751683	4.07209515	4.81757232	1.48880501	1.39268657	1.13105675	2.49049077	-1.28089192	2.77870251	2.95341901	3.08690426	3.97793538
AGGF1	5.09759555	5.82989	5.59448781	6.12816957	6.06742337	6.56249876	5.92799705	6.04454358	6.04850651	5.35381127	5.66445196	5.87864938	4.94129415
WDR41	5.15397279	5.5825533	5.1758016	6.14412276	6.1757591	5.99627466	6.05927965	6.34490493	5.92748404	5.94607193	6.02945527	5.99521344	5.3136076
NDUFS4	4.94302855	5.25467571	4.40943504	5.71475049	5.29952893	5.61448131	5.41614166	5.25714804	5.36810798	4.68882491	5.28649038	5.70503739	5.52250121
SCGB3A2	1.66863385	1.37316646	1.63529093	1.89131314	6.05931388	1.67871558	1.37316646	1.37551399	3.10815245	2.37412093	-0.95756103	2.73435702	1.53845829
SPINK1	2.7777322	1.63723061	2.83147738	2.4387978	5.6962629	2.53087712	2.05590813	3.08209205	2.58865326	-1.34916459	3.3570195	1.91597144	2.61206771
HTR4	4.88928471	1.95107164	2.61526614	2.23317994	2.76092845	2.0223539	2.71055199	5.1634607	3.87179482	3.46587267	2.61267616	3.30025304	2.9812417
ESM1	3.54693913	5.08030296	4.46175775	4.6196099	3.04636973	3.45238166	2.14690422	1.76751927	3.43136243	1.62313216	1.92560012	-0.27067172	2.79822815
GRPEL2	4.31787666	5.15367788	4.48442707	5.99163909	5.02170534	4.96195306	5.10707794	4.63611757	5.28416615	3.90850574	4.99244642	5.13735853	4.36458985
ARSK	4.17124607	3.77522865	4.16724407	3.98624869	4.41233505	5.36083807	4.88701093	4.66606307	4.44149896	4.36603581	3.59339005	4.46880204	4.43863066
RHOBTB3	5.73678178	5.21013055	5.04555271	6.97171795	5.20722669	4.54479549	4.91943727	4.99311794	4.7895726	4.70289788	4.57800555	4.62728137	5.49530438
GPX8	5.93075599	5.44373028	5.93985379	7.31749299	5.75439941	6.28568548	5.73913806	5.68379976	7.08161422	5.67457779	6.94292664	5.89296845	5.84664344
TIGD6	4.69637491	4.11624795	4.28706916	4.05799229	4.00771087	4.26480891	3.78079362	3.66861268	4.20976815	3.890678	4.01133933	3.29425573	4.81645433
SERINC5	5.07021537	5.15368398	5.32217732	5.99614222	4.72810875	4.48052014	4.90786091	5.08601793	4.83734182	4.698855	5.28996227	4.96060299	5.58623699
ENPP6	0.66671023	0.36491336	0.62221449	0.40791248	2.00075514	2.27244026	1.83447691	0.77966191	-0.26445677	3.64235656	2.9114525	2.87892136	-0.26445677
CAGE1	0.00987259	0	0.09173861	0.10014539	0.04540318	0.09966655	0.0978796	0.33191658	0	0	0	0	0
CASP3	4.87113768	5.08771416	4.82806307	6.08089773	5.124588	5.3818375	5.21828503	6.07283951	5.03967519	4.78557323	4.87032811	5.04374515	4.37324871
ERAP1	4.85325343	5.23635269	4.51615397	5.90288428	5.01446809	5.29817089	4.89709451	4.25947959	5.00563199	5.09746708	5.33591063	4.9250238	5.22051584
ERAP2	5.39740093	2.34656909	5.20234267	6.01351228	4.80158462	2.11903077	5.91502753	4.98327086	3.32492762	5.70774772	3.38448749	2.26711423	5.66347813
CMYA5	4.0558855	3.99593187	4.43774177	3.81665998	4.49876082	6.36987083	4.53267436	5.73585424	5.15584396	4.86945275	4.34950683	5.26023398	8.96896969

EGFLAM	1.75546081	1.08456288	1.33464179	2.20530701	1.16387545	1.48693067	1.03252325	0.62145694	0	0	0	0	0
CARTPT	7.18086655	-0.15529511	6.14993445	2.28482637	4.45572169	6.35612163	3.52136089	2.3478872	1.68152725	1.31491463	2.71257317	1.78252564	5.85099372
RICTOR	5.86093267	6.45513109	5.68697989	6.71733541	6.90843487	6.83450284	6.75999091	7.22174182	6.41862359	6.04980473	6.66168516	7.19594652	6.60631828
PAPD4	5.27797374	5.95620726	5.31354624	6.41992522	6.27194551	6.107834	6.32011197	6.35541028	6.37191167	6.21794861	6.27797732	6.02628898	5.94897163
EBF1	6.81439467	6.07466491	5.77354756	5.6689846	6.36401132	7.04078225	6.5226183	6.42054142	6.17024181	6.90845864	6.63114062	6.48349247	6.08682911
ANKRA2	4.48163324	4.99828451	4.19076037	5.02670514	5.13442574	5.31391821	5.06698634	5.19191434	4.76700726	4.09627625	5.04287891	4.22867122	4.70973641
UBLCP1	4.2899397	5.10463833	4.88664745	5.63597179	5.63959567	5.91797948	5.58050883	5.76111616	5.44982803	4.83617435	5.70888154	5.49553125	4.5963686
UTP15	4.23944515	4.41152766	3.96985769	5.28219586	5.17699335	5.67511629	4.81319117	4.88457132	4.69992254	3.36920584	5.11500191	5.05603508	4.33848854
TLR3	3.50378936	4.40170403	3.38105068	4.47890902	3.90809328	3.91591888	3.36354377	3.85115624	4.73502492	3.68991419	4.11980025	3.38721843	3.61136538
KLKB1	1.6858228	0.97250372	1.53181622	1.6227292	1.82165868	2.54078057	3.31289072	1.78991931	3.39601354	-0.74291164	2.75373311	-0.74291164	3.67191257
NSA2	5.04287158	5.26799635	5.06234627	5.69899493	5.66635388	6.22442977	5.1268395	5.10325983	5.99703755	5.87432651	4.43832154	5.66593401	5.46456993
GFM2	4.93450453	5.0715148	4.87404516	5.77477646	5.55085035	5.84758527	5.41862679	5.22969821	5.7170624	5.58502779	5.06136635	5.36385966	6.1066585
CCDC127	4.65338331	4.00043159	4.09958383	3.40431045	4.0890645	4.58369899	4.08292625	4.46981918	4.15969635	4.64214127	4.50551305	4.6252133	3.74300752
ACSL6	1.61226077	1.67628666	1.7688355	1.71864259	1.60619868	2.38683065	1.82992731	1.96309792	-0.85770212	-0.85770212	3.27177841	2.87919067	3.73114038
SHROOM1	3.35915443	2.13465311	3.6263777	3.18260144	3.38346175	1.94122584	3.46299356	3.29952383	3.23739721	4.75769827	4.24990102	2.91552032	3.76108605
GDF9	1.41013519	0.77673606	1.27705061	1.38136877	1.68102685	2.38646565	1.75534922	1.88729498	2.77969966	-0.67809645	2.64986835	3.77049911	-0.67809645
UQCRQ	4.38992236	5.18867862	4.78094668	4.31291584	3.93867005	5.01471134	3.85917307	4.00289931	4.7525172	4.55589164	4.82925164	4.32026459	4.86173231
LEAP2	0.66740287	1.43205371	0.51804035	-0.06109184	0.78075448	-0.01523635	0.55546985	0.94277557	-0.12424981	-0.12424981	3.87528276	-0.12424981	-0.12424981
SLC35A1	3.79513528	4.30045087	4.04385849	4.09742541	4.77781976	5.36815063	3.98687139	5.00090332	5.13122211	4.28410975	4.09791983	4.7126493	2.8471963
GRIK2	0.00594016	2.53964118	1.59716464	2.54393509	1.61270228	0.5799723	0.25624076	0.00978619	3.36809805	-0.31293103	-0.31293103	2.35375909	3.33277719
TXLNB	4.57595952	3.71774183	4.24555183	4.26395318	3.95676284	5.72022812	3.33453453	3.49123608	4.36325366	4.22586201	2.64195699	3.83704579	6.27381467
CITED2	6.44379349	5.56336912	5.81041239	5.85116694	6.74660453	6.56798256	6.11174943	5.31088097	5.3835018	5.33075172	6.11568369	5.35896216	5.39222915
T	0	0.10109519	0.00124472	0.1173386	0	0.00069205	0	0.00158797	0	0	0	0	0
DCBLD1	4.3889863	4.57789262	4.59905768	5.55009364	4.34810085	4.66179971	4.58782071	4.63257044	4.47124335	3.92738001	4.28088625	5.06854767	4.45363285
SFXN1	4.54586226	4.22363365	4.37832406	5.6644439	4.26544167	4.29064048	4.69043385	4.72424943	3.87551784	4.10671119	4.52390387	4.17721054	4.97591777
SAMD3	2.65311429	2.35796451	1.48486956	2.09619496	2.15534694	2.2179592	2.42968178	1.57195333	2.90490337	2.76454321	3.59013263	-1.11688541	2.83983824
TMEM200A	4.87342774	3.77016686	3.44566135	4.61071547	3.43934075	3.18479721	3.01808843	2.67981981	4.43893831	1.45931324	4.03151724	3.71027254	1.34066917
DACT2	0.07163574	2.35325058	-0.04122773	0.01711853	0.07253334	-0.0419674	0.23261663	0.40602422	1.34652044	3.66157666	0.07055617	0.07055617	0.07055617
PDSS2	3.94033457	4.68642326	4.21664375	4.3966717	4.32301691	4.59985644	4.49948973	4.04906669	4.45059342	3.13705214	4.14571697	3.76222555	4.40906961
STXBP5	5.51060132	5.92929521	5.03954247	6.44356638	5.85533356	6.06989409	5.93141176	5.98366732	5.93823846	4.98209954	6.1178723	5.76699963	5.47618944
IL31RA	1.69964704	0.99584987	1.72505585	0.8365245	4.17475765	3.22918062	5.44963145	6.33273661	2.97385111	5.72552106	3.48970644	5.90465756	5.16660852
ANKRD55	0.21287724	0	0.00124472	0	0.10373337	0.00069205	0	0.06376223	0	0	0	0	0
RAET1E	0.83063662	3.34526379	0.96625879	1.26940374	1.61801843	1.10461186	0.94051902	1.7051033	2.64425469	-0.75696073	3.93478609	2.6788414	3.65712023
PI16	-0.08576959	7.05831902	-0.04659912	1.52704616	5.65673512	2.66999407	4.74441609	4.01286196	6.31182918	7.25091183	6.54386216	7.36819778	6.01109689
TBX20	5.43598504	5.53396545	5.01605238	4.64632922	3.88350208	2.78182121	2.95502509	4.48450267	3.23672243	3.87710441	4.58873459	5.46061188	6.08682021
DAGLB	4.67819308	4.76668378	4.46637635	4.33704373	3.92588261	3.10798358	4.40163538	3.97720995	3.00340394	4.21615807	4.22018388	4.26762334	4.25015325
KIAA0895	4.55467773	4.06679215	3.60513875	3.80222403	4.04693759	4.67797136	4.76594274	4.08953266	3.52908132	3.85492139	4.25538974	3.94072246	3.7240878
STK17A	5.39543711	5.92338661	6.02948141	6.63826161	6.92737926	6.42609226	6.76696409	6.58298903	6.58973015	6.0634735	6.86924027	6.73707204	5.26636909
TRA2A	5.31966268	5.41972506	5.08019103	6.85314522	6.56185746	6.41919823	6.87411829	6.5202939	6.12516873	5.49114681	5.99397465	6.10262294	6.32688147
GALNT10	4.70380381	5.14923981	4.63632577	5.41299347	4.47818355	3.95327351	3.93772287	3.84482588	4.91446439	4.62273185	3.85512436	3.43546598	5.0882257
SAP30L	5.78148601	6.14367084	5.4134506	5.05487018	5.85874898	5.74033811	5.92301362	5.94011274	5.69120145	6.17676157	6.14113079	5.57866763	5.94411175
RPS14	7.26562419	7.87600203	7.16379944	7.84248722	6.99726459	7.0271662	6.98576196	7.02248045	7.12894864	7.65439754	7.07881781	7.64346003	6.96011076
HCN1	0.30805009	0.60146502	0.47753306	0.30805009	0.30805009	0.30949589	0.38456478	0.31136763	-0.16540788	-0.16540788	2.93658019	-0.16540788	4.25803485
MYOZ3	4.53123861	3.15081818	5.01795862	3.41577361	3.52023192	3.33891545	4.57737516	3.11567097	3.33579186	4.64038239	3.2759824	3.55006921	3.85208498
COG5	5.45618809	5.87847222	5.10105747	6.31952934	6.22365313	6.2550128	5.87167344	6.25039874	6.49143528	5.72225172	6.47441729	6.16814374	6.11694885
GPR85	2.39814864	2.57802161	1.99321401	2.89325663	2.24400367	2.21163178	1.99237719	1.90339384	2.77066061	-1.08088711	2.84090758	1.87465372	2.59589253
SLU7	5.86327654	6.09151803	5.8356997	5.77018153	5.95145498	6.73824069	6.08886048	6.13906852	6.29546644	6.47936761	5.54972716	5.93504926	6.09316524

RP9	4.46548302	3.18498887	3.73498025	4.16822911	4.08410815	4.33517055	4.61252182	3.73879381	4.15626191	3.54952302	3.98969083	3.6061206	3.72162525
PTTG1	3.19112345	3.26357627	5.38211235	5.22003467	2.29857617	2.60582621	2.79131477	2.66936785	2.86254	1.7984448	2.60375895	2.37653899	1.73285727
CAMLG	4.93665632	4.97560234	4.26267292	4.90454221	5.0503137	5.43606747	5.43699946	5.31701833	5.11642365	5.81567579	5.27100321	5.08358303	4.53190809
BMPER	5.04881531	2.41538094	4.00127054	3.07498917	2.42061642	2.53532482	2.50978504	1.76007768	-0.88974245	1.13106995	3.01712719	2.61827467	3.98039719
RELL2	1.58518067	0.90532907	1.90756097	1.40932412	0.88189814	0.21330825	1.45578496	0.82066025	0.59166542	-0.43771711	-0.43771711	-0.43771711	-0.43771711
KCNK5	1.07171109	2.9158017	1.47041431	0.99244403	1.36867087	1.24647887	1.03956489	0.88596246	-0.39334351	4.01690023	3.95574263	-0.39334351	3.61899277
KIF6	0.88542605	1.15848563	0.78929119	0.97480648	1.10343016	1.0479198	1.26456514	1.10481294	3.25444237	2.63169111	3.21480168	-0.43350399	1.42252317
ZNF12	5.18751062	5.70374304	5.35086696	5.97154937	5.9574687	6.58813834	5.92127786	5.97763606	5.62713878	5.61592359	5.58579102	5.34960793	5.32278974
SLC29A4	1.18310451	0.8122611	0.76812338	0.79070696	0.55133917	0.78994126	0.56182591	0.51866108	-0.48477246	3.18843988	-0.48477246	-0.48477246	2.68991531
STEAP1	4.78411263	2.66967612	4.31217323	6.7066262	3.09637959	2.86997284	4.20057383	3.7552104	2.99226151	1.77740126	2.7348898	2.91918084	1.74343224
CDCA7L	4.96930425	5.57400664	5.13204645	4.89471218	4.61709405	3.96427666	4.44154888	5.43712399	5.42643073	5.57183194	5.02045956	4.83242616	5.94011837
MIOS	4.6093057	5.62810325	4.63973646	5.77829349	5.3425053	5.52052158	5.5693457	5.0845663	5.0380917	5.10481448	4.77893324	5.01461839	5.1232637
KIAA1324L	5.22614649	5.28986629	4.15141273	4.34482832	5.20958368	5.87478234	5.0876688	5.86060107	5.44946821	5.3625927	5.54595549	4.99546154	5.25766985
USP49	4.64946543	3.73518146	3.86768523	4.48907379	4.58866533	4.13257982	4.14368425	4.31574275	4.32460099	3.94938732	3.68488781	3.70807847	3.6404574
SYTL3	3.59810402	3.01284389	2.08741402	3.32728048	1.75020071	2.43551978	1.92533567	1.93812968	2.59097391	-1.04216799	3.98878261	-1.04216799	4.20261413
IQUB	2.26718522	2.76342367	2.60948445	2.59917036	2.53918893	3.56831468	3.17413907	2.71004497	3.03835832	2.46073053	3.71422971	2.01765503	-0.12219932
HEY1	1.7988002	2.11843906	2.11999015	2.83160418	3.22737244	3.09994943	3.65997696	3.78628094	3.08269974	4.02571799	4.03925834	2.51200573	3.11120881
ZNF704	7.01812988	7.63428655	6.77727451	6.26823472	7.75590919	7.51922628	7.71460981	8.06407092	7.84961482	7.6440612	7.80490078	7.64886706	7.61731438
FABP5	2.40853156	0.58091946	3.55019708	3.62338181	0.80215618	-0.01348111	0.34174542	2.21801477	3.31769091	1.76974486	1.50635094	-0.80414196	0.41382977
TAGAP	3.9677926	4.67318239	2.81432919	4.24841248	2.98561188	2.29789226	2.24879113	2.37691622	4.36464047	4.19431859	3.7094512	3.22738608	4.34656239
COL1A2	8.39262535	9.02585614	10.095977	10.9302797	8.74494997	8.64006042	9.45870217	9.84519202	9.37349991	9.80643669	9.89322091	10.300723	9.62146179
FNDC1	3.70683769	3.76150786	5.56329641	6.12736889	3.90004008	3.17632823	4.6057879	5.92846645	5.1968926	7.01565403	5.04029425	7.12404835	5.50109701
CHMP4C	4.94827414	4.60942942	3.27168841	2.65228621	5.15069253	5.20240873	4.85139121	4.921217	4.86745631	4.12641259	4.44245221	4.10974318	4.27269235
SLC13A4	1.35919499	1.53136557	1.62868125	0.98152447	2.25450615	1.58528459	1.14866928	2.2841065	0.429855	-0.49409547	2.47988875	-0.49409547	3.99713968
PGAM2	3.70264143	2.4684719	4.28100108	1.401477	3.65547371	4.55721737	2.22266129	2.10095369	2.4003491	-1.83846082	3.45368962	2.43491572	7.26626297
BRI3	5.09117998	5.05988237	4.86302271	3.89084637	4.85780917	3.97708563	4.56124098	4.86136841	4.74586504	4.70558639	4.58780802	5.18430396	4.1838732
LMTK2	5.84656782	5.65340439	5.66461389	4.87592385	5.9003683	5.88660867	5.7275483	6.02021056	5.68048576	5.96564279	5.68620226	5.52529995	6.07268356
CTSB	8.77241807	7.82033458	8.65884153	8.92368284	7.29011128	7.26435518	7.94441617	7.25081033	7.6763591	7.81720378	7.81187115	7.63390037	8.18102207
SOX17	1.58730115	1.38079436	1.42441823	2.5922007	1.2072043	0.99771216	2.13717199	1.48564264	-1.13443592	2.39065376	3.87015911	2.73337371	3.54288686
DLC1	6.04165682	6.66934185	6.07201669	6.59769437	7.467014	6.57388489	6.67308503	6.64810763	6.56167967	6.37045172	7.25988164	7.14555984	7.3868378
ADCY1	3.37300555	3.47504232	2.49968703	3.04069656	3.03222813	2.57506651	3.19256469	3.49478485	3.64812482	3.41440549	5.34603799	3.89740547	4.56648437
C8orf48	2.99605384	3.8923883	3.91568556	4.13009823	4.01193731	4.58249263	3.40685207	3.6755477	4.36107143	3.30598216	2.59170545	3.69521267	4.12759104
HNF4G	0.80772448	0.54564943	0.64768867	0.55276353	0.56689956	0.7764746	0.54564943	0.54896929	2.65154992	3.02607751	-0.33167938	-0.33167938	2.36503851
PEX2	4.88638829	5.33351521	4.95537939	5.47515983	5.0047046	5.62571808	5.53967686	5.27436921	5.33480125	5.24990686	5.77254246	5.41512742	5.42598971
RAD21	6.03656241	6.61816614	6.11597944	6.90314471	6.70984676	6.881425	6.78613294	6.64038718	6.91494819	6.30933354	6.67652048	6.73105854	6.3574062
MED30	3.16047144	2.28027946	3.06613088	2.17017186	2.00874875	3.77802882	2.95528045	3.93022869	4.41163662	3.00265071	2.75985355	4.12705331	2.16482353
TNFRSF11B	6.53282562	7.22419904	6.64439826	5.77935122	7.92554455	7.92878726	7.09537348	8.37029546	8.70047939	6.81790762	8.11548023	7.21679194	7.76856934
PHKG1	5.92136218	3.3744805	4.69667038	2.83232222	2.92469679	3.03666049	3.55553622	3.03813652	3.37814199	3.35702156	3.63940717	2.40240379	3.14021599
CSMD3	0.04535569	0.23209996	0.16368179	0.04535569	0.04535569	0.04679529	0.12889386	0.048659	-0.02191752	-0.02191752	-0.02191752	-0.02191752	-0.02191752
ORC5	3.70392125	4.70287274	3.93627951	5.06428844	4.4883539	4.52953056	4.62967358	4.90081959	4.66855791	4.37162979	3.76699249	4.45300195	4.60482975
OSGIN2	5.0790183	5.7267409	4.78022148	5.33671946	5.90334513	6.44840784	6.01027778	6.05298573	6.56714195	4.46242865	6.15276011	5.58967572	5.00284898
DEFB1	-0.27413433	0.48935547	-0.27269639	-0.27413433	0.61477234	-0.27333486	-0.27413433	0.6765963	0.01049672	0.01049672	0.01049672	0.01049672	0.01049672
SUN1	5.67793819	5.95616525	5.64149939	4.91536427	6.27454963	5.78807418	6.75261983	6.76937617	6.08288993	6.74198729	6.28015594	6.57267084	6.69204763
OXR1	4.67741368	5.63900311	4.92473717	5.69987557	6.04126731	6.15741117	6.08649032	5.9588484	6.57362082	5.46593385	6.00781911	5.41989362	5.33691688
TMEM74	3.47444524	3.94917874	3.037007	2.76906956	3.55798123	4.87814372	4.4757376	4.15089776	4.47340904	3.99825609	4.53315066	4.73642139	3.13157143
GPR146	2.86954341	3.07805188	1.40290796	1.93737925	2.38943822	2.10499655	1.7504739	3.01908663	3.07817303	3.29816784	3.13575985	1.13135212	2.56991075
TMEM184A	-0.01176809	-0.01176809	0.1836339	0.13344342	-0.01176809	-0.01034764	-0.01176809	-0.00850873	-0.05205667	-0.05205667	-0.05205667	-0.05205667	-0.05205667

NOS3	0.86654988	3.65287956	2.3856305	3.16968082	3.40427278	1.07178113	2.38193081	2.32099378	2.75063366	4.49939367	4.90842807	3.02196298	5.04273707
MICALL2	4.10700185	4.04564969	5.0566504	3.58732796	3.93784472	3.20492525	4.50120859	4.06254655	3.71598712	3.98919534	4.55565274	3.25820083	4.4463665
CA3	2.37851589	1.53916106	0.84605227	1.50482875	2.69403913	3.99245503	2.58415504	2.87446483	0.04179742	3.43954944	0.89452433	4.06321731	3.11963337
INTS1	5.83640537	5.75982496	6.47473638	5.0572948	5.39852804	5.12101524	5.60361149	5.57729712	5.33684191	6.3307192	5.91628997	6.2433833	5.35158836
CDK5	3.827779	2.88761723	4.30586681	3.72395599	2.51206026	3.40883124	3.01746005	2.61293501	3.32528582	3.79046663	3.03058326	2.65232757	3.56814539
SLC4A2	5.54022921	5.5437226	6.33935835	5.07968218	5.45370198	4.73552923	6.04874702	5.48328118	5.1596637	5.77294499	6.15812621	5.95007894	5.25454738
FASTK	4.41345418	4.59659775	5.11781539	4.66890458	3.97837299	3.53996504	4.45039795	4.17636115	3.85951701	4.1688967	5.09050762	5.81231021	3.75833223
TMUB1	4.76748977	3.53138471	5.23650469	3.7999517	4.05978528	3.6545691	3.89421453	3.49241266	4.127505	4.55853772	4.5631589	4.83670186	3.18685444
PHAX	5.6907202	4.93876995	6.20404231	5.70503423	5.56377675	5.98927502	5.51042993	5.20034258	6.05886686	5.48273223	5.52227381	5.3935184	5.47204093
ALDH7A1	5.73980172	5.16357351	5.57239837	5.43011418	5.17230207	6.02676265	5.92146841	5.98597654	5.94960335	6.25682398	6.01302197	5.83977733	6.02755254
FOXK1	3.94557947	3.98230466	4.60901204	2.84063629	3.5527621	2.95366915	3.81953548	3.57453606	2.7249938	4.72072949	3.24706847	3.64706027	2.43171424
COX6C	5.8787488	6.34490732	5.52653555	5.71949112	5.92740022	5.85712464	6.15712702	5.47431899	5.98841379	5.92398787	5.57662776	5.62068586	6.24535828
OSR2	4.83331695	3.27163761	3.01584546	2.46195345	2.76598879	3.50869197	3.67404272	4.23355452	4.66986656	5.21175534	4.08771045	3.58182177	4.1279189
YWHAZ	7.10498235	7.92790309	7.46125794	8.38520169	8.30445804	8.24604925	8.26285355	8.27089218	8.30996863	7.95799126	8.19718295	8.40361349	7.9155084
BAALC	0.14390812	0.68643304	0.65953762	0.62672119	0.39133677	0.2397612	0.37450398	0.25508066	0	0	0	0	0
FZD6	4.98533516	5.73829499	5.50591286	5.66610014	6.19255373	6.33345346	6.45171333	6.59094901	6.333418	5.82315869	6.26415495	6.53181266	6.29037024
CTHRC1	3.07510153	3.68659943	4.5041681	6.93961614	4.59056583	4.46606428	4.30316083	5.30382876	6.02891033	5.2411291	6.00539177	5.10797969	5.53640611
SLC25A32	4.78142261	5.32367371	4.43260367	5.64679736	5.78262406	5.92542481	5.86159201	5.81515902	5.5794355	4.92355631	5.64732147	5.23501488	5.15641462
DCAF13	3.90632357	3.91325262	4.67261333	5.8864573	4.63401796	5.08816857	5.19706933	4.90917528	4.9571564	4.67222363	4.37517887	4.70035846	4.12977664
TP53INP1	5.58582122	5.71334125	4.97259505	6.13148534	5.31929237	5.6126445	5.58463231	6.31707384	5.91642519	5.14807893	5.98110682	5.76297274	5.44617785
INTS8	5.33633427	5.42401539	5.06821044	6.08726348	5.87529271	5.98482401	6.13623715	6.04478796	5.72792871	5.24949238	5.91161467	5.73957764	5.13702165
FREM1	5.1238288	5.9858457	5.60940233	3.77448216	5.70712172	7.44050791	6.32807062	6.79370942	6.68451701	6.72857821	6.58928069	7.05612767	6.49665977
GEM	6.16674935	7.52748544	6.53075737	5.28301768	8.50041514	8.37012326	7.52748544	8.51439646	8.2611765	8.4243697	8.20890719	8.76644942	7.8830526
PDP1	6.00899911	6.52265457	5.61580274	5.69244389	6.52082809	6.46990053	6.33236056	6.64919745	6.72044898	6.4783282	6.69704961	6.85427998	6.34673717
TMEM67	4.36508976	4.33060832	4.32708052	5.26604253	4.75407976	4.74895204	5.47350047	4.79580537	4.48408321	3.46128954	4.6431925	3.85478928	4.74993327
C9orf24	1.39751152	0.41655859	0.66854783	-0.04482681	0.73279805	1.59439961	0.37154722	1.50050125	-0.18106598	-0.18106598	2.65995811	-0.18106598	2.3383061
SNAPC3	5.4527096	5.672416	5.49435615	6.07170815	6.42493866	6.3858805	6.59192846	6.29348631	6.36053114	6.08893591	6.1797911	6.57673192	5.72872602
NUDT2	4.77002875	4.96453263	4.73044306	4.42205326	4.31307824	4.04587948	3.01005848	3.74577298	4.40376144	5.07771798	4.37279768	3.32573774	3.0799732
TMEM65	3.68489699	4.40090098	4.18888973	4.90311577	4.67918488	4.52492018	5.13233909	5.19142673	4.92325815	3.4354229	5.29411742	5.3076183	4.59740289
PSIP1	5.88625401	6.10509013	6.54137917	6.43750246	6.96865166	7.28297431	7.26062208	7.12554483	7.39951351	6.94578259	6.119105	6.28924806	6.98847047
UBAP1	4.79823885	3.19513481	4.12984709	3.44027654	3.83418204	3.91128515	3.59417558	3.13547349	3.36694021	4.12418388	1.02699371	3.30583964	-0.58890301
DIRAS2	0	0.12207218	0.00124472	0	0.00987259	0.00069205	0	0.11183175	0	0	0	0	0
SYK	5.2571605	5.71313653	5.20390018	5.55296627	4.19061971	3.58260278	4.32594568	3.71550222	5.09401384	4.75171277	4.72178882	4.30507504	5.45574885
NIPSNAP3B	3.75990889	4.81007607	4.04898305	4.02221878	4.64672947	4.7944385	4.74753913	5.41641162	4.77198376	3.82156669	5.24113925	4.85628973	3.85549644
ABCA1	7.70971153	6.20326242	6.60534298	7.28943272	5.7601567	5.10230912	4.8102814	6.07560249	5.20581769	5.12711357	5.32290957	5.08989118	6.06032787
NFIL3	5.12458736	4.52765661	4.68474818	5.99637482	5.32407744	6.75768271	5.47086052	5.40300298	4.70402702	4.48685364	4.09433362	4.43404097	4.57061104
LETM2	3.01653007	2.66303545	2.75599939	3.12814252	2.19395813	2.53193782	2.49611647	2.4371206	3.49353862	2.59700927	4.21122791	2.90060585	2.25935245
METTL2B	5.10473177	4.69153556	4.90263205	4.64520719	5.02734391	4.54906192	5.35586524	4.91220144	5.21116442	4.22062963	4.92112803	5.31572305	4.44469674
FXN	4.60524171	3.49491205	4.44086907	4.13520715	3.49715704	3.66588074	4.04085417	3.27102975	3.36788669	3.9611262	3.76238662	4.12236738	4.29561548
ZMAT4	0.70187695	0.41818999	0.42076544	0.41818999	0.55360683	1.14247584	0.41818999	0.42147566	-0.19377504	3.76282501	2.2757764	3.01212305	-0.19377504
TMEM71	2.61169983	3.39933219	1.47546726	4.2847159	2.71937082	2.22424018	2.03195152	2.43085351	2.53956069	2.69204702	3.65580475	3.4636408	4.2887565
MAMDC2	3.09506225	5.94425833	3.38527269	3.43221234	4.78505392	5.427909	5.15586985	5.45205313	5.01653199	5.72664965	6.11370583	5.6482232	6.12231202
CPA6	0	0.14390812	0.00124472	0.1131285	0	0.00069205	0	0.30481363	0	0	0	0	0
C8orf34	2.56059786	0.73296755	2.64716125	0.75487648	3.95910135	4.70764398	3.84415039	2.12572295	3.21829509	3.92183856	2.49534772	2.46222197	2.45487282
TMC1	0	0.15011969	0.00124472	0.46978721	0.07682702	0.00069205	0	0.14425478	0	0	0	0	0
ALDH1A1	6.26072175	6.22144443	4.13763841	4.46110882	5.15013081	8.26066308	6.07535851	5.78128439	6.83181956	6.13356349	6.27247971	6.35975206	6.42037091
KDM1B	5.64543687	5.40977917	5.34236458	5.49089069	4.76629665	5.45163978	5.32099216	4.71341471	5.18673225	4.84981924	5.04856109	4.72814929	5.26834945

HGSNAT	4.07081842	3.93965595	3.63206187	4.0835745	3.98379579	2.29366933	3.67689268	4.5198959	3.75930895	3.40547323	4.58092275	3.29685009	5.17111238
RASEF	0.59477596	0.59477596	0.59726282	0.59874647	0.59477596	0.81711052	0.79639078	2.40862397	3.48999055	3.46796252	-0.35256004	4.37979916	-0.35256004
GKAP1	4.28745257	3.71992606	4.27240264	3.7360949	4.89139623	5.30056701	5.10258513	4.70021817	5.50732802	4.34564987	4.95722777	4.85224124	3.53689292
KIF27	3.07540356	3.40961232	3.18838496	3.11769963	3.59198474	4.08430286	3.05668153	3.48830184	3.15551809	4.27583737	0.54870162	2.91054588	2.05952054
C9orf64	4.63170758	4.29363094	5.24366214	4.29103511	4.22701935	4.60318625	4.24649818	4.20245104	4.98210875	4.44104745	4.52781096	4.68905578	4.66283915
HNRNPK	5.91611151	6.29186889	5.46758646	6.47178808	6.05724955	6.71317659	6.09154303	6.16290469	6.42727044	5.90310525	6.29114381	6.34794015	5.63040302
SVEP1	5.86554688	7.40884476	5.12334337	5.65560223	6.60685598	5.74517399	5.96096311	7.68429466	6.82936751	6.57213748	7.02669164	6.55030643	7.58257835
TRPV6	0.00940045	0.07876584	0.06767478	0.02245113	0.01920028	0.00069205	0	0.00158797	0	0	0	0	0
ANKS6	5.28168322	4.80775486	4.9369812	4.41926796	4.85129048	4.67762574	5.15504203	5.52014403	4.69516188	5.35802692	5.39457616	5.08176015	5.13929288
FBP1	3.73174261	4.14154977	3.59361845	3.26307895	1.62429948	2.06434151	1.41359891	2.64846367	3.53469694	2.50236197	1.0948852	3.10395323	3.1595432
ZHX1	5.66361661	6.24743104	5.09047585	6.25047294	6.37445288	6.79394557	6.42917292	6.55629485	6.75986432	6.26384545	6.23874534	6.50177184	5.89233134
CYBB	5.6562742	6.90087527	5.88536817	7.34122933	5.46877216	4.95480631	6.01199854	4.18157731	6.66086111	5.84238492	5.90860087	5.63158242	6.83584293
DYNLT3	5.32726689	5.72110673	4.8436537	5.92555553	6.47606226	7.224731	6.35420063	6.46134229	6.35089434	5.23039731	6.29806105	6.44373579	4.67969324
MID1IP1	6.06940059	5.12207198	6.27040923	4.99890918	4.79591912	5.26085628	4.45799246	4.79550379	3.99764182	5.12944317	4.5776254	5.32369133	4.35664281
KIAA1958	4.8852242	4.29183095	3.98377389	3.77123018	4.52219975	4.57408193	4.59845979	4.5143618	4.32917458	3.61943087	4.71819791	4.73342711	4.47554546
PTCHD1	0.15266862	0.06959096	0.25868066	0.26616572	0.10876836	1.05091023	0.04910246	0.15786664	0.03563558	0.03563558	0.03563558	2.72799577	0.03563558
RNF183	0.08010171	0.31204827	0.07302908	0	0	0.00069205	0	0.00158797	0	0	0	0	0
ASB11	0.5798637	1.00401201	0.39081292	0.38822689	0.38822689	0.38966469	0.38822689	0.79827613	-0.26047923	2.7133135	-0.26047923	-0.26047923	5.55462331
PCDH19	2.20714281	1.75609877	1.90733352	1.92548036	2.90921379	2.55421446	2.49249617	2.6760689	3.86336664	3.82524778	3.90309498	3.06876602	3.47282035
PIGA	4.00861139	4.9595941	3.52259948	5.20488103	4.54366165	4.88671187	4.32673572	4.96695369	4.60020915	3.67007941	3.90440894	4.59616719	4.47821623
STRBP	3.66933636	4.53667025	3.56153496	3.97070309	4.3992041	4.69469847	4.34471168	4.31294747	4.36048728	3.85873427	3.76120132	4.23104145	4.41637934
GAPVD1	2.3010544	2.62872083	2.33258626	2.67123658	3.16609842	2.81718943	3.02366221	3.10592094	3.53649843	-0.2075585	2.93590245	3.649743	2.88236644
WNK2	1.239309	0.9888222	1.18918826	1.00726507	1.86926596	1.63071429	1.83536309	2.2400994	2.64487878	3.40891119	0.96877478	0.9747241	6.69958867
ATP7A	2.29838821	2.90270066	2.57238507	3.09522506	3.35521718	4.0830388	3.26431912	3.46848797	3.06951966	2.27242273	3.50063773	3.87695193	3.63066603
ZNF367	3.01115488	2.39730285	5.46913944	4.70225193	3.26596165	3.53749148	4.10446049	3.31892551	3.94698465	1.70791461	3.89532024	3.84006794	1.39485993
NLGN4Y	3.96212411	2.72910802	2.27813704	2.85823092	2.25262372	3.25831376	1.53488978	1.58233493	-0.82616039	-0.82616039	-0.82616039	-0.82616039	2.4299652
HDX	4.27547467	4.23587563	4.06509772	4.79326736	5.1235531	5.66377214	5.23703987	4.96457411	5.15903786	4.36478485	5.13741855	4.0319957	4.66231264
NDUFB6	2.69748956	2.34939111	3.06189184	3.83110982	3.72266939	4.56019823	3.97823852	2.56452349	4.25625732	2.93915542	3.18396288	4.70477097	2.58410815
AQP7	0.81700252	2.08112078	0.84802498	0.8696053	1.80674677	1.38525946	1.53992822	1.52895187	-0.5711237	-0.5711237	2.57410112	4.30415011	5.00784793
NOL6	4.72327793	4.81677238	5.18800932	4.83649978	4.24230357	3.57205251	4.27157249	4.11063921	4.29791057	4.29332174	4.97567812	4.51533033	4.46057399
AQP3	3.10099953	3.44251827	2.08359409	1.9356297	1.68099979	0.54716221	0.98437504	0.55221738	1.47115619	-0.73138952	1.18541468	4.37745546	1.86412978
VCP	6.93860442	7.06613932	7.36041455	7.11783398	6.55203827	6.50521485	7.06613932	6.44384591	6.69837029	7.27359021	6.95897993	7.11408366	7.20150513
PIGO	4.74436257	4.18014657	4.89944527	4.11287723	4.14952147	3.55059374	3.79285249	4.15851479	3.41493634	4.97592959	4.30859736	4.80384064	3.31863171
STOML2	5.90790787	6.15308875	5.66817513	6.00739257	5.06486284	5.28874375	5.22852719	4.82055315	4.45113807	5.76309276	4.38913299	5.33847182	5.45000749
BRWD3	5.37596948	5.60764458	4.87446443	5.78594757	6.00621208	6.00716568	6.00871057	6.07385844	5.33426875	5.18568481	5.64338669	5.4450473	5.32549346
SLITRK5	2.14124502	2.54943482	1.94776174	1.83708707	1.9421444	3.40201697	2.11027962	3.48688732	3.82923532	3.61774302	2.49851539	3.56039475	3.45790687
MELK	0.69294359	1.08442142	3.20372513	4.77388665	0.3087538	0.55113849	1.51454673	0.73634361	1.4148997	3.42467075	1.9591609	-0.77311902	-0.77311902
OTUD1	4.36030672	4.64814487	3.93476425	4.5050412	4.8524075	4.75826776	5.15415801	5.23260427	5.14076746	5.16888781	5.21703876	4.98644459	4.96728018
ARHGAP12	5.36594466	6.25596247	5.16201151	6.1186024	6.25967147	6.58091347	6.38107586	6.56492718	6.24583286	6.39975066	5.73011606	6.65289275	5.99166978
FAT3	2.37916213	3.16088723	0.05288532	-0.37795237	1.68010185	2.72295785	2.15064833	2.42121967	0.20530376	0.20530376	0.20530376	0.20530376	2.99804165
HECTD2	4.85028704	5.71294944	5.04117755	6.25352324	6.23072896	6.01652393	6.34095448	6.38061848	5.71503274	5.57037149	6.0512526	5.91508592	5.27147177
FBXO33	4.93680829	4.80724322	4.40335309	4.53781448	5.05645233	5.38081677	5.10788161	5.22916592	4.45666583	5.16947061	4.84582785	5.2034579	4.81023995
CLDN2	0.11996819	0.06877083	0.07136341	0.47089324	0.06877083	0.1903611	0.06877083	0.07207835	-0.24532727	-0.24532727	-0.24532727	-0.24532727	-0.24532727
LRFN5	0.60053482	0.25090059	0.76081916	1.09773674	1.19536665	1.56876535	0.86128853	2.33235543	0.16823143	2.77832584	-0.51575483	2.68149169	1.1196287
SPTSSA	4.15120158	4.3262107	4.37656439	5.42106097	5.25887434	5.65641344	5.50770555	5.17347125	5.47965929	5.094544	5.91507074	5.61140451	4.22775679
WRN	4.55020567	5.63618022	4.82729662	5.79580473	5.81950751	5.79976764	5.63125301	6.05040209	5.80176565	4.1682779	5.78804881	5.71648507	5.40387308
CFL2	5.40535748	6.36875224	5.40189266	6.21112656	7.3595389	7.2839843	6.97952559	7.39229251	6.84465128	6.77255529	6.87586056	7.45545197	6.97868452

SUGT1	5.13184959	5.77067018	5.40392626	5.6286174	6.2772371	6.27353991	6.10373697	6.1273177	5.89989752	5.48175736	5.73810598	5.67067174	5.50076108
GTF2A1	6.14956765	6.07503933	5.76833809	6.42836115	6.62608979	6.98752242	6.85312306	6.72316592	6.39316835	6.12258372	6.22445655	6.30617459	5.83843776
ZCCHC24	8.02187723	6.58987034	7.25690829	6.19698323	6.34688698	5.96469246	6.61059349	6.69295762	6.26156861	6.27384305	6.48591773	6.10392278	6.14454848
PGM2L1	5.56762492	5.69450301	5.5050273	5.8042386	7.0380283	5.9432806	5.8319877	6.39906678	6.23706743	4.61364938	6.46562056	5.78847684	5.83052704
PHYHIPL	1.27112762	1.41949273	1.40989868	1.35965194	2.04528508	2.47230466	2.5262175	2.96088674	0.77737057	2.65037166	2.11061124	3.155775	3.68257896
SLC16A9	4.10654815	3.32179401	4.05475147	3.64031046	3.09900291	4.57444238	4.24690462	3.18700997	4.32520839	3.63879861	2.71658964	3.66974513	3.5916407
FOLR2	6.14904867	5.94880099	4.99895976	5.98081271	4.21192333	4.36455228	4.69649391	3.46459667	5.95870982	5.56091337	3.76375501	4.98049368	5.71298694
INPPL1	6.2126354	5.76798235	6.29298879	5.91665678	6.0229677	5.3720096	6.18970278	6.11378245	5.77579831	6.02835968	6.16549532	6.20355614	6.26576935
GJB2	0.15161236	0.15161236	0.15381437	2.43918474	0.55720043	0.29083451	0.3371519	0.1544216	2.03311009	4.21639354	-0.16840816	1.40745232	-0.16840816
CRYL1	5.49586649	5.46456008	4.89870033	4.6409502	5.06018512	4.69387771	4.93226486	5.26800687	4.74812192	5.53265371	4.66195669	5.15009489	5.72020166
REEP3	6.11467157	6.45377147	6.08349343	6.55020494	7.25760052	7.32684652	6.99165727	7.30898001	7.22041024	6.31929216	7.39131549	6.98819605	6.41656446
HEPACAM	0.03170292	0.61586754	0.03418644	0.03170292	0.2216515	0.48914376	0.16569602	0.03487131	0.04228793	0.04228793	0.04228793	0.04228793	0.04228793
SKA3	1.49394697	1.15686876	3.36043376	4.23625089	0.97408954	0.98690196	1.18976978	0.98107992	-0.00080904	2.49711618	2.17105523	3.13605029	-1.0847255
PCF11	6.19574149	6.50321679	5.99409065	6.46888703	6.62132344	7.17413108	6.58340616	6.44755744	6.69910593	6.56131461	6.3414101	6.12759485	6.6518765
PKNOX2	6.15192956	3.56993987	5.52101233	5.5463905	3.06565395	3.47299893	3.4985531	3.44313478	3.04060474	2.77190049	3.82519993	2.52635189	3.96255445
LRR1	0.54188882	-0.025561	2.46707432	2.80072904	1.46015085	1.7050364	1.43140028	0.90936975	2.6704881	3.00713641	2.89207169	1.97144116	-0.4398607
RPL36AL	1.57550292	0.74041658	1.67328705	1.25475796	0.87441701	1.61621809	0.20800474	0.26246945	0.81686384	0.81686384	0.81686384	2.94509518	0.81686384
DNAAF2	3.82887959	2.95513107	4.18624512	3.93540604	3.96664653	4.49707537	4.2296821	3.92989358	4.30315811	2.84246568	3.99916896	4.02143195	2.91866659
C10orf10	9.93377311	7.35421335	9.65738799	8.03872009	6.5968449	5.2121929	6.55020108	4.42699092	5.01215417	5.28209208	5.54070415	5.04114237	5.64117564
MAGEC3	2.62363223	-0.04956605	1.94309949	0.38948306	2.02654854	0.59236636	1.97942638	2.61540717	-0.25021805	4.2149775	-0.25021805	2.21690558	1.8979543
ZNF22	4.66035911	5.54684661	4.51653326	5.61933327	5.37075074	5.55511033	5.01205972	5.33672153	5.64368198	4.51657105	5.18292388	4.9773305	5.2810952
KLHDC2	5.34973013	5.29325159	5.08941724	5.06243919	5.68333911	5.66302472	5.49080759	5.94994563	5.32090822	5.58048076	5.53580963	5.4261941	5.99271753
EML5	2.94779862	3.66206361	3.30933988	2.86552901	3.50871086	4.66249987	3.91163251	3.97745377	3.38674664	4.47253603	2.74737578	4.20546554	3.97160614
NEMF	5.58309864	5.943124	5.60229658	6.37516716	6.37769541	6.49406286	6.23228857	6.32556729	6.79048981	6.1530825	5.67896688	5.88151699	6.1136476
RPUSD4	5.21620718	4.99582248	4.85201822	4.16700127	4.28231368	4.26425292	4.91214406	4.52786692	4.78341711	5.44451771	4.0621115	4.83430676	4.53183852
ARF6	6.75332657	6.58809182	6.69123257	7.90288288	7.12904465	6.602332	6.7611265	6.96621386	7.10861462	5.98221304	6.94227324	6.65462594	6.27208172
TTC8	4.97757809	4.54220287	4.1046039	4.42518378	4.74989946	5.27519039	4.72663467	4.83215974	4.98150296	5.21134938	4.88841602	5.49722244	4.39620255
TMEM63C	0.25557156	-0.12507064	0.16338358	0.48324507	-0.10735483	0.00951132	-0.03969458	-0.12190374	-0.08434375	-0.08434375	-0.08434375	-0.08434375	-0.08434375
NOXRED1	2.005504	1.37081911	0.97954564	2.23523023	2.24551113	1.61972753	1.66822302	3.46733096	1.34579717	1.92777586	-0.83304826	3.36459154	2.04878487
AKR1E2	4.23278962	3.87941258	4.36956856	2.7202153	4.56011783	4.07479771	3.31860987	4.9359211	4.45731285	4.13697738	4.42578897	4.31016365	5.33453041
KBTBD6	4.7103367	4.27251189	4.7974548	4.25777426	4.59293938	5.43002418	4.80606123	4.31014968	5.01904738	4.72170013	4.77959228	4.76219931	4.1957194
FAAH2	0.92674869	2.76858818	0.60595002	0.73054157	0.68695591	1.54416508	1.16349756	1.00574472	1.68598677	-0.47146407	3.04961221	0.94241594	-0.47146407
NUDT5	5.20339622	4.83718274	4.38696231	5.8039149	5.42289337	4.82875807	5.21935065	4.9955885	4.96983967	3.70082305	4.29363023	4.57749167	5.03593823
DACT1	6.2713095	6.99182036	5.83443115	5.70837475	6.49003001	5.77431551	5.11002631	6.13837387	6.364304	6.82093014	6.71649338	6.92971642	6.24529794
OXGR1	0.29743139	0.9388659	0.27987361	0.44850038	0.27529789	0.12956264	0.34751433	0.13141096	-0.12910946	-0.12910946	-0.12910946	3.84149175	-0.12910946
BEND7	4.49432254	4.67121291	4.30421379	3.72553111	5.18554966	4.91148483	5.25669982	5.46846118	4.72452153	4.23463211	5.05479949	4.2228017	5.32780625
ATP5C1	5.75336772	6.69181002	5.93808564	7.15596522	6.6164545	6.56209816	6.68556786	6.59353857	6.62089553	6.52872748	6.3299496	6.47803462	7.42085656
PRPF18	4.17331926	3.00889277	4.04192832	4.38347583	3.86027571	4.51966555	4.21038065	3.74136398	4.3032687	4.32695798	3.07731185	3.17756391	4.45657152
TAF3	5.8720047	5.26309967	5.35814083	4.59048845	5.34110774	5.85668907	5.16611253	5.00947687	5.53411069	5.20570048	4.43685647	4.82407514	5.3701155
VSTM4	6.93422081	6.63715524	6.57619978	5.35199883	6.59581023	6.87750413	6.90144396	6.91355182	6.48156088	7.46545247	6.3356803	7.26340452	6.83590543
VDAC2	5.8834328	6.48662442	5.84550232	6.63772417	6.58713092	6.82037885	6.90128612	6.98019627	6.35883202	6.97707593	6.04914792	6.53322063	7.0937238
COMTD1	1.88639257	2.93366928	2.76998151	0.9786954	2.01573695	1.85734659	1.27163585	1.53719907	3.60357817	3.60237033	2.33033826	3.07332429	-0.49916471
SLC18A2	0.53638392	1.07403821	0.28066175	1.05093253	1.33396839	0.50157497	2.12376954	0.47270669	2.18497114	-0.48934117	-0.48934117	-0.48934117	3.16678756
PDZD8	5.57062269	5.94172134	5.47210056	5.83371077	6.43494989	6.61960758	6.13622508	6.59739593	6.02791883	6.13535876	5.95337185	5.9474399	6.2226703
ZNF503	5.75213599	4.92556944	5.47786125	3.8415619	4.39869801	5.168774	4.87349641	5.23301175	4.66780115	6.01781473	4.57612533	5.6302333	4.89845561
QSOX2	5.02753465	3.64812162	5.4168586	4.73107273	4.1816108	4.01605782	4.72015332	3.92169328	3.68817895	4.13744366	3.56777601	4.35265086	3.459445
FAM204A	5.08712109	5.23119852	5.23704234	5.40136985	5.53179342	5.64188537	5.66439029	5.67346957	5.63409121	5.39512531	5.59164161	5.67039994	5.25008092

NSD1	6.52071481	6.75688651	6.43422921	6.75688651	6.44225818	6.87932196	6.89174168	6.50811192	6.27607001	6.76947533	6.45640273	6.52962195	7.09525214
PRDX3	4.78886617	5.56301962	4.8495703	5.99085893	5.67918274	5.42541941	6.1461391	5.87946118	5.53470383	4.89329168	5.55814055	6.1336843	4.79102459
ENOX2	4.46021072	4.76977601	4.1445305	3.76007818	5.16697294	4.9553957	5.19225345	5.62250059	4.91373411	5.48695031	4.99505675	5.38058757	5.45994741
GHITM	6.73353888	6.89423753	6.5539787	6.91082187	7.1298276	7.55916578	7.27142076	6.5837026	7.36770673	6.94440512	6.83763537	6.68837231	7.29779598
SNAPC4	4.32473049	3.78363896	4.67172307	3.74050579	3.52988801	3.2303892	3.83382661	3.66110265	3.37434132	4.20198403	3.69388462	4.02043247	3.50596444
PMPCA	5.17225217	4.15481111	5.13698159	4.98152694	4.79555849	4.46355306	4.80778765	4.94169155	4.55983323	5.01540102	5.45233649	5.01042079	4.9345555
SDCCAG3	4.94218236	4.41306273	5.30387472	4.51158622	4.22192855	3.66846168	4.82030961	4.30006269	4.20270631	4.21055818	3.71494738	3.98217825	4.22858079
FRMD7	1.68070184	1.93936775	1.70622049	1.37341674	2.82558529	3.96101655	2.56920439	2.41152656	4.52745195	4.10282644	4.22669851	5.81846466	4.07886739
AK8	0.81342843	1.20047148	1.417773	1.14281106	0.55719477	1.59612788	0.19345826	1.85849915	1.41936709	-0.47130365	1.25970824	-0.47130365	-0.47130365
TSC1	6.48168929	6.01530779	6.05091884	5.65510535	6.12673341	6.03017449	6.31745284	6.51953157	6.18295038	5.95261844	5.97235138	6.16513985	6.81665571
HPRT1	4.35039879	4.29444761	4.31885153	5.87857827	5.10928395	5.02328598	5.35659134	4.34049459	4.51176452	4.56792171	4.92070093	4.47595351	3.80693859
FAM69B	0.7567994	2.6512294	1.04474842	1.51798037	1.60021647	1.30525858	1.1621457	2.58746439	4.25288912	3.10994882	3.83595929	1.7502396	3.25750164
ZMYND19	4.41991427	3.28516178	4.4798933	4.06426994	4.06773209	2.80192263	3.50715562	4.01424832	2.81956083	5.0059658	4.40179695	3.9687047	3.25988558
STOX1	0.68243858	0.89931774	1.02578387	0.7241424	1.40154116	1.84870752	1.07820525	1.43243984	1.657397	3.44965948	3.67617907	0.81762188	-0.4774185
RET	1.06613404	0.76303577	0.81603796	0.73997394	0.78702394	1.41058102	0.95656597	0.58503346	1.06181794	4.20714367	2.3851561	-0.43035749	-0.43035749
DDX21	5.77501703	6.43510027	6.384434	8.13725274	6.85147507	6.79362881	6.54842976	6.2193093	6.31274069	5.85850753	6.24664481	6.13420457	6.56649333
BMS1	5.68261769	5.71649803	6.12112563	5.67688804	5.89414749	6.33335131	5.53300805	5.52194256	6.01216571	5.35485731	5.5737314	5.1537865	6.10778782
STK32C	2.841607	3.04161921	4.01026141	1.87447925	1.88859388	1.33429582	2.30393481	2.94979901	1.91724449	3.39105695	2.67876603	2.87179091	3.54698613
FUNDC2	6.16039791	6.21304125	5.67853735	5.90612312	6.27836178	6.16210297	5.57296302	5.49172035	5.86464814	6.08910201	5.33344306	5.90598661	6.24181753
METTL17	4.59445732	4.59257139	4.80934099	4.60240379	5.00284166	4.1211611	5.265945	5.15405888	4.1197139	4.02558667	5.54695883	4.91153897	4.91770914
NDRG2	6.355687	6.08755857	5.44985209	4.7585057	6.21129964	6.05705338	6.66785567	6.56394692	6.03452078	6.82167012	6.00006041	6.19165857	6.55602732
ARHGEF40	5.81988307	5.71708366	6.71716396	5.84214206	5.44950886	5.11490991	5.5762539	5.81402521	5.6458818	6.56730499	6.38777	6.20356462	5.7660931
ZNF219	4.99514811	5.08569633	5.26271537	3.25523161	5.1091893	4.14943434	5.00149227	5.23524606	4.76991548	5.47589983	5.51646231	5.14952009	4.2193061
CASP7	4.72552342	5.99703273	4.56921102	4.81743415	4.3176296	4.98608763	4.39946833	4.63407643	5.09853843	4.47984924	4.92972532	3.98470995	4.746208
PPP1R36	1.43095632	2.17424473	1.21613684	0.81364206	1.4381511	1.69642706	2.45553698	2.17333735	2.88031347	3.21728468	2.56769038	-0.65535728	2.06635817
BTNL9	-0.99301135	1.20414666	-0.25364022	-0.11037548	2.27518555	1.2339058	0.586223	1.81967319	0.46679243	0.46679243	0.46679243	0.46679243	0.46679243
VWA2	0	0.21553524	0.00124472	0.04755303	0.08816589	0.00069205	0	0.39845569	0	0	0	0	0
METTL3	4.76966878	5.16911189	4.39399773	5.0906466	5.26015039	4.96673614	5.41511174	5.42857219	5.0912566	5.23016253	4.95037093	5.6177368	4.96265
SALL2	6.00515987	5.30046931	5.48823674	4.28761244	5.0284093	5.44348574	5.42310168	5.02257133	4.83957238	5.7553138	5.27573967	5.19278451	4.7179848
TRUB1	4.10030342	5.01922912	4.08249626	5.26050739	4.99244042	5.52500542	5.21887685	4.93009711	5.49852624	5.16383519	5.70137782	5.22719775	4.73738814
ZFYVE1	5.50393198	5.08988755	5.4265953	4.8180922	4.99941234	5.04635097	5.32108423	5.34256056	5.09759037	5.2306028	5.30858135	5.3407733	5.09572677
C10orf82	0.12539702	0.12539702	0.70440372	0.12539702	0.42869547	0.83413596	0.77884252	0.28976431	-0.10084649	2.96068255	3.39487195	-0.10084649	-0.10084649
HSPA12A	4.3727175	4.77726852	4.70688883	4.63141959	4.75211353	5.7937586	5.55960537	4.95340173	5.07360083	6.12483792	4.50864613	5.86260506	5.15228718
FRAT1	2.31907667	1.76731421	2.60690496	1.28653637	1.56894915	3.0329138	2.39580732	1.87190959	-1.0106377	3.43526259	2.36306525	2.95343933	2.41110079
UBTD1	4.31614925	3.99153022	5.31510775	4.3320532	3.27018542	3.67734525	3.07237165	3.27291883	3.52645017	3.83528027	4.10634117	4.45918011	1.45212692
ANKRD2	0.30340444	-0.04173042	0.86980318	-0.04173042	0.05890231	1.76714227	0.81017464	0.58459563	3.73069618	-0.00543331	-0.00543331	-0.00543331	2.53457704
E2F7	1.3408977	1.10007335	2.98339918	3.46662234	0.94234828	0.86127766	1.47188126	1.1565924	2.35929824	2.99253389	0.21724838	-0.84075559	-0.84075559
ARHGAP42	3.48833541	3.8739333	3.21439053	5.36402867	3.93910588	4.31368255	4.14232634	4.17758979	4.54618019	3.83885082	4.17006314	3.52368053	4.25179861
ISCA2	3.41877749	3.3205152	2.76333967	3.05231788	2.97013499	3.86543167	3.37556106	3.21454968	3.62241089	3.46793547	2.02051571	2.93686214	3.37795653
OTOGL	1.98934249	1.88681081	2.15648616	2.37170869	2.032153	2.50475514	2.13098485	2.23656286	2.29276035	1.95409592	-1.07437078	2.57574207	5.50313445
PACSIN3	5.17968467	3.78474577	5.15730038	3.533397	4.55783647	5.09013932	4.90992103	5.3430092	4.32314515	6.05355641	4.59549523	5.26837922	5.35001687
TTC7B	4.92326838	4.19288606	4.6054091	4.18488778	4.90538843	4.52197998	4.61106138	4.90322735	4.61793596	4.50191197	5.07629729	4.80620907	4.49080568
SLC39A13	4.95557118	4.86834649	5.76939296	4.63070575	5.4193366	4.9110679	4.67810829	5.40839818	4.99808489	5.36149436	5.68664273	5.83507132	4.91288391
PSMC3	5.83795089	6.1203112	6.39031638	5.69000661	5.04022592	5.84227798	5.22384939	5.10466667	6.25868804	6.56858891	5.68743837	5.83939866	5.79550398
RAPSN	0.66912936	1.02127546	1.27462074	-0.27598043	0.20388384	0.40893351	0.36420234	0.84230825	-0.10816072	-0.10816072	-0.10816072	-0.10816072	2.98416235
AGBL2	1.06235097	2.28502845	2.1313632	1.35087939	1.34714273	1.24223098	1.40136415	1.20145312	1.06372492	3.26009243	-0.89350165	1.27048034	-0.89350165
TC2N	6.44529143	7.14742712	6.60158999	6.75205985	8.47438438	8.41410648	8.12818835	8.64528209	6.65666308	7.00476777	7.20482442	8.12075402	6.40440844

CPSF2	5.24930449	5.48012831	5.90945443	6.02113309	6.12591167	6.52594802	6.20397162	5.87034357	5.87394542	5.44060128	6.0168041	5.59538519	5.85017301
MOAP1	5.67379649	5.84560533	5.51195099	4.24620126	5.56513906	5.7812732	5.51753063	5.56759839	5.53288897	6.35809081	5.65001226	5.93074832	6.01859435
IFI27L1	3.51786937	2.2776682	3.25342186	4.20542103	2.33718475	2.80651296	3.11040678	3.29934879	3.23526524	4.11872201	2.6731139	0.12942678	1.45794132
IFI27	1.00038213	4.98517946	1.62284937	3.41885061	3.25292213	1.65580589	2.55721814	2.01368819	4.49819002	3.59871897	3.43963563	2.46970577	4.89956807
CLMN	3.99923338	3.65714994	4.2201049	4.08166278	5.0601992	4.78045373	3.89259168	4.72868371	3.38659719	3.48806442	4.79696677	3.15806137	5.26277518
PDZRN4	6.00092365	4.95615942	4.39582502	3.33546779	4.79393547	6.18777804	4.12772569	4.35944165	4.9211341	5.96721527	5.03560035	4.91814262	5.81304787
CCDC38	-0.13366349	-0.13366349	0.27047429	0.26029831	0.18349213	-0.00297032	-0.13366349	1.06393503	-0.03588515	3.23534866	-0.03588515	-0.03588515	-0.03588515
NELL1	0.57239672	0.90939996	0.3763499	1.0123016	0.40125009	0.57933764	0.37376663	0.37706228	4.15165533	-0.10293313	-0.10293313	-0.10293313	-0.10293313
PTER	4.51601637	5.12331789	3.49980481	4.52820424	4.75200762	5.05979647	4.66389295	4.99577809	4.85145327	4.18228414	4.75359878	4.58812863	4.11970851
C1QL3	1.91832412	1.88779461	1.89038912	1.88779461	2.10543503	2.6131025	1.88779461	1.8911046	4.84169386	4.31461992	3.61996963	3.16117229	2.02532511
CACNB2	4.80951005	4.23061267	3.98141705	3.09130342	4.78720831	4.96012602	4.96860633	5.43902635	3.91173129	4.67995391	3.56381244	3.95584246	5.35867858
ARL5B	4.41960149	5.36976315	4.72444919	6.55859812	5.86304758	6.36616912	5.91553342	6.0715691	5.90249139	4.45030688	6.07732248	5.7685003	4.6696856
TAF1D	4.75127338	4.89222051	4.81905597	6.4547659	5.58347401	5.59047985	5.81139977	5.85797198	5.77115503	4.46505999	4.68906711	5.46491989	4.18071456
ABTB2	4.1089801	3.516168	4.81319725	2.30748439	3.38619989	4.0209921	3.43219441	3.73505489	3.33326961	3.88746662	3.95110132	3.44703458	4.31542715
AMOTL1	7.07231447	7.41145936	6.8653209	7.05012359	7.32435489	7.6554023	7.38692873	7.20759302	6.81284339	7.45189833	7.11227878	7.21575292	7.42408607
HTRA1	8.65944202	8.48765929	8.32820907	8.46507309	8.04340319	8.00623398	8.08163509	7.67216343	8.3778394	8.69075094	8.94037857	8.5670707	8.48035532
LIPC	-0.2207979	0.0655781	-0.13256288	0.57544994	0.46222939	0.0862904	0.07206442	-0.08169547	0.04685179	0.04685179	0.04685179	0.04685179	0.04685179
CEP57	4.90008759	5.34683923	4.70818273	5.72844984	5.56578307	5.79984369	5.83065612	5.33455692	5.88742232	5.05173362	4.73599483	5.49613521	4.99175262
TCP11L2	5.26133598	6.09687685	4.94405413	5.47099001	5.71760223	5.13880421	6.32884235	6.32746403	5.39864518	5.52252904	5.98691906	5.87010157	6.53179419
SPRED1	5.45865047	6.5455129	6.09056021	7.16320101	6.39582882	6.17989961	6.71557973	6.30529053	6.25047908	5.62062977	6.23205347	6.07227951	5.88521266
GPR176	5.88309859	6.71306776	5.41589913	5.71983842	6.89733415	5.35454335	6.34106656	7.00000219	6.30613094	6.3830682	6.49486132	6.40012277	6.48397411
JAM3	4.7777886	4.98997226	4.15030512	3.84971511	4.92135844	5.24943298	5.30950318	4.65859234	4.55164776	4.73929226	3.90942489	4.75399784	4.37584263
IL25	0	0.33116356	0.00124472	0	0	0.21120536	0	0.00158797	0	0	0	0	0
CMTM5	2.15916025	1.25830279	2.17294519	1.14835473	1.30428735	3.30977703	1.48560435	1.5417775	0.68811453	3.96689535	3.33574201	3.70117826	-0.8139901
ADAMTS15	4.47609191	5.88802432	4.06479515	4.04004912	3.34827082	4.09633925	4.03918881	3.08395071	2.50420642	2.6869871	4.345624	4.50562413	4.12636304
GPT2	4.45710361	3.10679072	4.95549035	4.03215075	3.15849456	3.44628033	3.61385366	3.3003061	2.90265168	4.10985273	4.47104704	3.5355619	3.82232396
AMN	0.07832395	-0.15500243	-0.15255986	-0.15500243	-0.12021059	-0.1536444	-0.15500243	0.35466932	-0.11429442	-0.11429442	-0.11429442	-0.11429442	-0.11429442
RAB8B	5.54771318	6.13652018	5.32439779	7.30802852	6.47478135	6.79343866	6.93750252	6.60884319	6.22714178	5.50995895	5.75602018	5.90168562	6.05917804
IKBIP	3.85825387	4.16299789	4.12812732	6.34297884	4.88082037	4.57314337	4.86899525	4.30298122	4.96300404	3.6415187	4.98465219	4.53342068	3.53217614
RPUSD2	4.00257062	4.34037106	3.56650414	2.56939933	3.06847021	3.80186952	2.89362872	2.85616695	3.28408443	3.93228281	0.97824546	3.97697552	4.0362178
HIF1AN	6.57361276	5.89292204	6.29910494	5.65832499	6.2308565	5.93080378	6.6473394	6.06847906	5.98093904	6.28560515	6.38209434	6.17724908	6.15948143
NDUFB8	4.67270211	4.80674503	4.10466513	4.07415535	5.01562992	4.2890062	4.77446421	5.25280974	4.11705305	5.38836301	4.57913335	4.99675494	4.47805916
ZFYVE19	4.63090168	3.72064971	4.93730739	3.83091012	3.79048872	3.68934877	4.36105049	3.50767542	4.18147963	4.69397255	4.37606575	3.30984619	3.30746773
SPINT1	2.02616031	2.48790844	2.55613416	1.84894138	0.19467127	0.71811806	0.69955	0.86819204	-0.91273183	3.67405957	-0.91273183	2.63711431	2.42083283
FBN1	8.62119498	8.29844629	8.80554684	9.30927303	7.90413645	7.60994644	8.00347841	8.39627157	7.98033814	7.90197397	8.36719237	8.08770357	8.35975222
AVPR1A	2.44361896	3.86603448	2.46222136	3.29739295	4.60298106	3.92272473	2.58369547	2.71704649	3.19718462	3.6887312	5.47958634	3.97157142	4.57990828
DEPDC4	1.05130415	1.05748809	1.36304422	2.00446515	1.4213206	1.54476107	1.28831423	1.58301517	2.50213878	-0.70049681	2.14057445	1.182867	-0.70049681
BRD7	5.62414615	5.60942039	5.46559859	5.36216163	6.01837702	6.57513976	5.66975814	5.80940489	6.369316	6.39999113	5.55133244	5.65107991	6.0950307
CKB	2.28189927	2.92061729	2.23236201	1.56080753	4.49114042	5.44777677	4.8176153	4.99104757	3.88140388	6.18530895	4.60622306	5.04823163	5.77892463
TRMT61A	3.91813715	3.31373926	4.6817255	1.64116933	3.41042804	3.09955814	3.30356038	3.20498108	3.73719858	3.05410191	4.26096022	4.0966401	-1.06359965
BTRC	1.74775905	1.92627173	0.50769117	1.95079188	1.88011663	1.5103802	1.87881422	1.58519914	1.02307248	3.60405242	-0.00512929	3.50785806	-0.00512929
POLL	5.12419598	4.49871516	5.1122578	3.92384664	4.2883691	3.85724306	4.34815947	4.93950296	4.66230528	5.51108343	5.15399786	5.16200985	4.52287082
BAG5	5.59370324	6.12883701	5.76339268	5.51386975	6.13711636	6.35917607	6.12769047	6.0785739	6.13604924	5.75505118	5.85945062	6.17725021	5.67179897
DPCD	3.58469412	2.92471529	3.79929208	2.95648731	2.73438571	2.868075	2.65500642	2.80502422	3.25439568	3.25439568	-1.01248009	2.53956757	3.1847448
LARP6	6.17352015	4.90681687	6.04134556	4.32413574	5.22759806	5.58652396	5.38630265	6.10449853	4.94588242	5.40536499	4.68175811	5.18428989	4.67986473
API5	6.02001221	6.7143854	6.23233474	6.9757873	6.93700333	7.25165179	7.00099279	6.8009724	6.9251961	6.54113376	6.57482655	6.55865025	6.55076481
ASPG	0.27091986	0.09663686	0.19355619	-0.03848716	0.05167839	-0.04172791	0.09162674	0.5927181	0.08567899	0.08567899	0.08567899	0.08567899	0.08567899

ZNF319	4.62018725	3.79126231	4.80519125	3.01419351	3.52962474	3.64696999	3.73865281	3.54455708	3.88342718	3.41026171	3.72057964	4.2393492	3.65802225
HPS6	4.46363556	4.14241489	5.10558277	3.64170674	3.2939	4.00164225	3.63684439	3.85549997	3.16563305	4.41761309	3.8596172	4.95584372	2.13382663
SENP8	0.76824438	0.12544757	0.38047273	0.61549132	0.43668252	1.85998754	-0.07037817	1.18638377	-0.15896167	-0.15896167	-0.15896167	-0.15896167	2.59009668
NOLC1	6.19796893	6.00207219	6.41004106	6.16928914	5.85595398	6.58674778	5.53174933	5.06776342	5.81239343	5.31070753	5.68519782	5.33667908	5.49978782
ALKBH3	4.72900749	4.66337489	4.35683368	4.22947713	4.86901264	4.87832759	3.83233204	4.51012924	4.90380414	5.38438988	3.81755879	3.64586076	5.03351917
COPS2	5.24715236	5.99314524	5.69241592	6.88509744	6.58558631	6.8600386	6.88422175	6.51451356	6.77524021	5.87886593	5.81184703	6.4960601	4.89093555
GABRB3	2.29325634	2.5067699	1.87762336	1.86965625	1.7911398	3.85262913	2.15110408	2.17009496	3.344272	-1.11644372	1.39549682	3.04865797	4.97325954
SGPL1	5.35896682	5.38720922	5.91462521	6.15338862	5.11200269	4.77789026	5.13615989	5.11684132	5.06179885	4.89595858	5.2614489	5.04859075	5.50453218
FRS2	5.43569526	5.89995816	5.25924723	6.29248579	6.27049506	6.60489982	6.06347528	6.30183039	6.34192121	5.68652231	6.27557651	6.27297444	5.38032089
CCT2	5.69266054	5.84852092	5.80923468	6.72987051	5.74577847	6.31327398	5.82892491	5.35404087	6.12616636	5.76032213	5.76943021	5.56649474	5.44650469
PCBD1	5.76884562	5.69966434	5.58673804	5.31965593	5.83328084	5.80813764	5.78328741	4.7689342	5.57011681	5.87605273	5.90588726	5.73158588	5.68504914
ARIH1	3.59924351	4.18253745	3.28167808	4.10132806	4.28959129	4.50716341	4.48171057	4.22817615	4.39628771	4.04673898	4.40203192	4.00039219	3.53682387
C16orf71	0.10407396	0.27826301	0.62570181	0.46439961	0.49241809	1.2866861	0.92015482	0.81063931	-0.30186935	2.91774832	2.56961419	-0.30186935	0.86412726
CLMP	8.5516513	5.99825293	8.70152882	7.73203496	6.68862983	7.30344834	7.23803306	6.8632225	6.46948683	7.11129729	6.28869571	6.45242194	6.53084547
SCN3B	2.2946238	1.91211167	1.63986495	2.09779689	1.78767294	1.54700072	1.85705929	2.02766464	-1.24247788	3.96776669	2.83095616	3.3598664	3.49570508
COX11	4.29053855	4.17739041	4.18859891	5.13754358	5.41696525	5.06965663	5.53744362	5.1421672	5.45903231	4.87780618	5.19467805	5.31472997	1.5698928
ZNF202	4.3746695	4.92957426	4.81993447	4.40965195	3.97017066	4.78761887	4.44254913	4.65069427	4.73863767	3.75909466	4.31879514	5.07267301	4.85477449
STXBP4	4.39245711	4.30112157	4.24293378	4.57568242	4.93421498	5.82786359	5.20752616	4.75293907	4.21555692	3.8189225	5.1314769	4.89468349	4.35245477
CYYR1	1.48401275	4.1656365	1.5875838	4.34577068	5.03565405	3.73997764	3.77044929	4.7045793	5.20720265	3.74074056	5.68729587	3.89556203	5.96970973
CUL5	5.09785061	6.00792639	5.50848661	6.16467851	6.07778706	6.42870906	6.35642842	6.31134332	6.57767465	5.77713923	5.6726737	5.82151035	5.95812011
C2	1.31708755	2.34959781	1.18229058	0.97210336	-0.40224514	-0.56319453	-0.12948528	-0.41420362	0.03445377	0.03445377	4.19148709	0.03445377	0.03445377
PLEKHF1	4.88858471	4.04652484	4.64102861	2.6763533	3.3201096	4.31804801	4.03192554	4.51122398	4.34102615	4.57891775	4.48211449	5.10502589	2.88147984
TMEM100	1.30798075	3.32393764	1.8083162	1.2218173	2.45171153	2.03947983	2.70737759	3.60867495	3.69433334	3.74779351	4.35649482	4.36660007	4.07581343
ANAPC16	5.88044488	6.42953318	6.09983804	5.38854674	6.11780354	6.36045076	6.48021341	6.00317008	6.30743876	6.38481781	5.99357972	6.24454866	5.84418091
SMPD1	5.50567906	5.31503676	6.01146909	5.87338961	5.17141882	5.25419533	4.83043829	4.90554963	5.03648094	5.24713188	5.70149498	5.98563153	5.42957227
APBB1	5.45233232	5.21044255	5.47488319	4.03813637	5.50948181	4.8938192	5.63265811	5.61067232	5.06572186	6.20578845	5.33428109	6.10758257	4.68852389
SYNPO2L	0.57045363	0.72982926	0.84359385	0.54232515	0.54109151	0.67466007	0.69203858	0.85920073	1.40802998	3.56506225	-0.30539306	3.13944829	8.27720775
NUDT13	2.18921592	1.94501933	2.11748212	2.67763451	2.03550928	2.38205818	3.1209934	3.40866005	3.71166086	3.33541172	3.04499437	1.60170002	2.86949341
C11orf65	0.58672	0.56181586	1.06210779	1.16117513	0.89762395	1.39341797	0.91170483	1.11055749	1.89534339	2.99204099	-0.65267999	2.58401304	-0.65267999
TRIM44	6.31173194	7.08194339	6.24826329	6.73101627	6.98126232	6.74619737	6.74357779	6.82081034	6.66142019	6.53269099	6.48878561	6.58378885	6.63999208
ILK	7.33150077	7.71208286	7.35072956	6.80633346	8.33416882	8.22790323	7.99114633	8.59511961	7.60186059	8.4699581	8.30878445	8.43181554	8.25694541
TAF10	4.99113859	3.35666943	3.80570013	4.46456655	4.39115009	4.31662546	3.58817985	3.98702781	4.25233293	4.67872533	3.56998544	3.12884273	4.85646545
TPP1	7.22110877	6.23651432	6.86815936	7.26992044	6.25775241	6.31403108	6.54123756	5.68443483	6.49461963	6.42958547	6.10217395	6.00916147	6.8250174
DCHS1	6.98706819	6.21927874	6.70584472	5.41492004	6.49601428	6.11583416	6.60284133	7.03545342	5.66273066	7.26152884	6.56300414	7.18087794	6.53435088
NETO1	0.18934002	0.13228489	0.25662755	0.14730713	0.16497689	0.13372315	0.13228489	0.51016343	-0.08419687	-0.08419687	-0.08419687	-0.08419687	1.63592934
CYB5A	3.94639498	4.51130835	3.36981252	3.14974963	3.88233332	4.18787895	3.98001123	4.04597688	3.82390821	4.26870519	3.80270501	3.26681695	3.63047776
USP54	5.9873855	4.67239327	4.72152548	4.76090695	4.85733851	4.7506223	5.40598277	5.99715049	4.36134501	5.05294992	4.53361463	4.58320639	5.04951835
RAG1	2.74248856	2.73704452	2.55511688	2.73620449	3.24919496	3.8010525	3.13210878	3.28442236	2.95231598	2.89378426	2.83549879	2.52950228	3.06767441
C11orf74	3.50513894	3.6297098	4.01740686	4.46242546	4.71347262	5.29550067	4.18727533	4.62952404	4.79379769	4.2101812	4.0444125	4.48522845	4.83685847
WDR88	0.35343905	0.3206418	0.62240044	0.0498632	0.0498632	0.23939561	0.0498632	0.05311984	-0.0882432	-0.0882432	2.23077868	-0.0882432	-0.0882432
OR2D2	0.31018164	0.03756142	0.64990787	1.98500303	0.94262559	-0.12564395	0.13156928	-0.12398843	-0.17216875	2.90760471	-0.17216875	-0.17216875	-0.17216875
ATP9B	5.16235968	4.91771336	4.65268911	4.64195709	4.90093109	4.24156545	4.81774211	5.02170576	4.74466206	5.12914755	4.19506269	4.53028342	4.79064283
PPFIBP2	5.98984543	6.46688243	5.79185199	4.90552895	5.95684889	5.40170313	5.89363891	5.57842809	6.12315824	6.529909	6.49001424	5.9365175	6.42222494
CYB5R2	3.95279295	3.34004737	3.8878787	3.26764517	3.87369671	4.06209498	3.22705627	3.9892903	3.52144425	3.44664385	3.06000591	3.88441119	3.93001672
SERPINB7	0.19648886	1.00412249	0.19895841	1.09563557	0.19648886	0.1978619	0.25523235	0.19963943	0.26986166	-0.19158639	2.48988771	-0.19158639	0.85048545
KIAA0355	2.52823839	1.92864355	2.05749741	1.01493787	3.09466267	1.86587862	2.3168005	3.437009	3.35720822	3.43696832	2.49605086	0.0213432	0.0213432
SERPINB8	5.54185182	5.02341733	4.70888016	5.98535222	5.19175757	4.9812424	4.26898891	5.83482565	5.2973627	3.8396037	4.6347049	3.81349867	4.78963011

TUB	0.9520882	1.32219549	0.23749338	0.4754466	0.87248246	0.12811527	0.61746799	1.03922109	0.49637714	0.49637714	0.49637714	0.49637714	0.49637714
RIC3	4.06563793	4.52473207	3.665593	2.86985749	4.47596496	5.04590334	4.69540793	5.2768502	4.43825064	4.93370873	5.01548056	4.64345496	5.00713382
IDH3A	6.21296048	5.28195698	5.51661341	5.49459567	6.02886814	6.23992846	5.72306557	5.53042989	5.60209407	5.57102722	5.89765169	4.79879222	6.43108174
WDR72	1.12031393	1.48647439	1.2126652	1.10484943	1.23628541	1.48894783	1.38557767	1.10507865	-0.64438003	-0.64438003	-0.64438003	3.36532803	2.87596814
CRABP1	0.31552412	0.31552412	0.31780649	0.31552412	0.31552412	0.91687984	2.42641356	0.3184359	1.51870228	3.61520756	1.83676022	-0.22583237	1.50820558
PLD4	1.91385891	1.73325287	1.61736527	1.41356604	1.01816698	1.17469408	1.34956296	1.38381442	2.95242527	3.13582196	-0.67156876	4.07848909	-0.67156876
ZMAT1	4.48854017	5.42233468	4.21207184	5.09865042	5.44420737	6.14868278	5.3515459	5.57655854	6.10696598	5.5240266	5.81326947	5.2977732	4.87487498
XRRA1	5.52113683	5.25406855	5.06583639	3.66309942	5.25339427	5.81589511	5.99459425	5.86374782	5.58047267	5.44372412	5.1815459	5.32957912	5.5185302
TRIM66	5.37156333	4.74889092	5.03008174	4.64497624	4.49439255	4.14469004	5.01039974	4.83265015	4.97243663	4.40436533	5.54950594	4.83426259	5.01028823
RNF169	6.36124976	6.08517351	5.69931108	5.82720412	6.19850115	6.26091617	6.29634272	6.30552111	5.8429742	6.38566655	5.88027783	5.91480161	5.51706445
RPL27A	6.1670768	6.99937739	6.44649977	6.19265367	6.17140834	7.24215058	6.02318063	5.99269397	7.33517021	7.33517021	6.46656398	5.93970313	6.82327643
ST5	5.78728908	5.4552625	6.21470411	5.75372056	5.34785181	5.37277965	5.53482111	6.16453766	5.68705278	6.62629183	5.77033799	5.95456694	6.21362551
CDYL2	2.82426293	3.42355141	3.6304744	3.45761097	3.63550113	3.35155063	3.8123786	3.30977381	4.0382382	2.74346513	4.45568193	1.7099276	2.20521299
TMEM130	6.19331315	7.39509183	6.26343246	4.3678447	6.56891592	5.95292558	4.61318515	3.59437942	6.72542099	4.99282043	6.83494366	6.39123518	5.97509746
PRTG	4.18425814	3.12343487	3.64771707	3.96752597	3.84163286	5.155715	3.97155927	4.14789223	4.05039764	2.40698178	4.59994878	4.52046886	4.47234102
CENPN	3.29017198	3.13413056	4.60541569	5.22075382	3.51873587	3.72631317	3.90013864	3.62300152	3.73393753	3.09799316	3.55941548	2.50277958	3.97598653
AKIP1	4.07326342	4.91262269	3.89383018	3.77109968	3.87417048	4.52501342	4.69120013	3.98764007	3.61720446	4.59359161	4.22349276	4.39746114	4.69412033
ATMIN	6.17629402	5.89472785	5.64012342	6.43451025	6.21732055	6.46923696	6.13336353	6.03880859	6.1294249	5.75715903	5.97261127	6.12067849	6.54097863
TMEM41B	4.62953733	4.29726881	4.98680782	5.38860448	5.28522256	4.84943315	5.58672543	6.09713913	5.2472614	3.58437405	5.60249279	5.12047669	3.82948574
LEO1	4.51348549	3.85252391	5.1296836	5.36702776	4.03576374	5.42493055	4.57487217	3.78709884	5.01919694	5.13847477	3.76844125	3.59019179	4.80394428
ZNF143	5.52290904	5.08324668	4.34095591	4.80680774	5.49372027	5.59804219	5.18257873	5.5745751	5.31605284	5.19794116	4.8219736	4.88507139	4.73026773
TMX3	4.82679632	5.72717598	5.41347848	6.48605235	6.03465605	6.13445358	6.38276585	6.19577118	6.11599548	4.71935061	6.00485414	6.08503083	5.29941084
MFAP4	8.11540405	8.58110078	8.73624302	6.73474841	8.7148172	8.99630226	8.28925148	7.78732734	8.20220619	9.30788946	8.531504	9.47467451	8.04880307
WEE1	4.92550821	5.43261508	5.06916097	5.59353188	6.38882575	6.23311189	5.92366274	6.55238026	5.63014889	6.02952393	5.62589064	6.31800269	6.68102674
MAPK7	4.99213159	4.19111775	5.20396753	4.11849864	4.75244225	3.94158649	4.34832288	4.77658289	4.41059166	4.95207486	4.49524092	4.87442239	3.75866534
PRKCB	3.65222275	3.63646228	3.97627047	3.46456334	2.45282009	2.75342495	4.67870315	3.14161345	2.9632871	4.73042792	3.024121	2.82194945	3.71970814
NDST2	4.19492431	3.85042883	4.43272345	4.24097955	4.57001284	4.20882126	4.65037108	4.80743904	3.78626042	3.35550222	4.70774764	4.12598334	4.27772269
MCM7	5.02191359	3.89168557	5.13259836	4.31324262	2.9218784	2.96100805	3.54818213	3.13353647	4.60820367	4.03758771	0.03890573	3.79169626	4.28827698
CLEC3A	1.4751951	1.49461393	1.77914859	0.99631092	2.42610512	1.56022665	2.88278474	1.34599429	4.54931348	-0.78337196	5.2427383	4.92981512	-0.78337196
CCDC68	2.56310829	3.74067253	2.06383303	2.26861713	5.1404045	3.15829937	3.16011404	3.45906967	4.34859173	2.18989052	3.94728903	-1.43912265	5.04762278
CLEC4E	2.80915249	1.51401518	2.15233417	2.99267055	1.64659018	3.37468073	1.66386729	1.80422476	-0.13381427	2.57075367	1.30214029	0.58904851	3.72464875
ZNF3	5.60775678	5.40349691	5.46180769	5.04435706	5.05567597	4.8796189	4.86831786	4.90213657	4.94037665	5.13258863	5.12453127	4.85864611	5.02353684
CLEC4D	0.18502464	0.74133099	0.56117015	1.21358394	0.18502464	0.62658335	0.5091616	0.18828738	3.34388945	-0.36362596	2.99186296	-0.36362596	-0.36362596
ZSCAN21	4.70718694	4.25161618	4.03237597	3.88466737	4.69278116	4.85169373	4.28912881	4.6973584	4.11547435	4.9867692	4.58632308	4.84969594	5.09551596
RIMKLB	4.16947578	4.7044018	4.15520399	4.28599348	5.47622609	5.41915798	5.30264602	5.71879796	5.50371477	5.23485794	5.24976316	5.5614081	5.59252149
A2ML1	0.42608853	0.38020964	0.52013352	0.38020964	0.46260903	0.91456596	0.41262734	0.47065816	-0.1679629	-0.1679629	2.25069742	3.37880335	-0.1679629
BEAN1	2.28703339	0.95442162	0.33834044	-0.07091713	0.94429309	0.0655549	0.74913332	1.63464924	1.28565309	3.90206227	-0.24041091	-0.24041091	-0.24041091
TK2	5.90891207	5.95859519	5.57465385	5.41724176	5.39290473	4.94799449	5.31743901	5.43260648	5.79799842	5.52758413	6.12625764	5.69677883	5.7487538
TMED3	4.92555999	4.94967224	5.12266562	5.62083743	4.3142953	4.43834704	4.4881427	4.46520361	5.04628636	5.47389608	5.13650127	5.23854223	4.42110446
SLC38A8	0.28251938	-0.09408184	0.10636866	0.15028583	-0.09408184	-0.09280819	-0.09408184	0.81191381	-0.01037051	-0.01037051	-0.01037051	2.24281766	-0.01037051
SEC11C	4.51476813	4.151151	4.72147291	4.09430225	4.40523552	4.98122201	4.13206635	3.89594191	5.26785228	4.65877305	4.23189324	3.63486734	4.85597212
GALR1	0.02725148	0.22200682	0.02984249	0.03184186	0.05451813	0.02869205	0.09804744	0.1795978	-0.06421037	-0.06421037	-0.06421037	-0.06421037	-0.06421037
TMEM135	3.62832296	4.42212237	3.44548061	4.6584053	4.03774488	4.78015611	4.56710675	5.08199465	5.06117358	5.32722915	5.26557445	4.75962879	4.24865226
IQCD	2.02020362	1.76151028	2.18049205	1.47972287	1.7736721	2.02629768	1.60525369	1.23711236	2.66260372	-0.89549905	0.93878133	-0.89549905	3.72421406
NDEL1	6.81220244	6.07262392	6.46558172	6.69490786	6.81705546	6.46422058	6.72546302	6.75398731	6.58792744	6.65819006	6.45171965	6.69668705	6.83079837
CENPV	3.16699613	4.04695641	3.43708733	2.70728971	3.69706055	3.64398184	2.81701521	3.56497255	3.65580248	3.92161166	3.15208576	3.32404886	4.28116073
RRAD	4.41474398	4.70531432	4.84258779	2.69445671	3.89821931	5.66009221	4.600408	2.86768976	3.14495196	5.76831402	3.36474503	4.67727787	5.08353656

FAM96B	3.63434736	4.20157721	3.38223759	2.85967283	2.4168112	4.40147326	2.88297905	3.22259667	3.57686821	2.60895441	2.99090337	4.14287042	3.01192665
HSP90B1	0.39655733	1.85649183	1.33262749	3.76381537	2.1027902	2.18190522	2.54329934	1.37820737	1.15650483	5.03257063	1.15650483	1.15650483	1.15650483
MC4R	0.01509032	0.67821093	0.01746599	0.01509032	0.01509032	0.01641116	0.01509032	0.01812112	-0.0221739	-0.0221739	-0.0221739	-0.0221739	-0.0221739
BLCAP	5.07430479	5.14193437	4.89878542	4.95295129	4.90814701	3.86071674	4.92089353	5.15616446	5.18011306	5.42281287	4.86205968	5.58578821	4.91662194
CHRFAM7A	2.48553736	1.50523104	2.76094782	2.09065985	2.32625458	2.45334904	2.571211	2.73515218	3.48262274	4.15255383	3.4636533	-1.02598813	2.66788973
ATF7IP2	3.99704957	4.80492475	4.32949268	4.77396883	5.14799102	5.04044881	4.7381919	4.51288255	5.23591474	4.7726712	4.37741128	5.08144674	4.41214665
MMP10	0.91266962	0.44816303	1.23202915	0.80469383	0.44816303	0.44960116	0.80615878	0.45146297	-0.67564501	-0.67564501	-0.67564501	-0.67564501	-0.67564501
TMPRSS5	2.97783411	1.22446129	1.81480699	0.74205286	2.54307654	0.98064787	2.20167124	2.26459756	1.28624493	3.42568011	-0.49401236	2.72435859	-0.49401236
COG1	5.63877805	5.6047563	5.05939233	5.11386003	5.60951553	5.00154224	4.90718926	5.5838876	5.56890254	5.76898508	5.51421437	5.57662476	5.35873473
PLEKHA7	3.65307502	3.92892046	4.56241809	2.84773415	4.45656846	3.68740325	4.58000632	4.81558308	4.0286732	3.43649028	4.12663066	3.58779862	5.24221925
ZNF606	3.87911006	4.3379226	4.23353837	4.00181726	4.80426546	4.57783317	4.50523696	4.84492222	4.28081221	3.90026348	4.63718608	4.48835681	4.06576977
ZCCHC18	2.33434775	2.71490199	2.33917489	1.37661533	2.79507084	2.70193586	2.12287296	3.00713134	4.00070424	4.31684424	1.46736697	4.10833454	1.78757172
B2M	8.94041457	9.79822608	8.66960154	10.210248	9.5493224	8.81491262	9.11351508	8.91246295	9.22205837	8.6792318	9.05188595	8.84217909	9.31375964
ZNF592	6.31716211	5.80141405	6.19623317	5.25398632	5.94922939	5.62737014	5.99338038	6.07790154	5.51203764	6.61072456	6.1621179	6.39376755	5.70569929
CASC4	6.16677348	6.81202544	5.9541204	6.99401346	6.95871289	6.98072679	6.99401346	7.15943665	7.6383882	7.25444132	6.71502581	6.81372826	6.83868505
HTR3A	0	0.13710693	0.15528848	0	0	0.00069205	0	0.00158797	0	0	0	0	0
NNMT	7.58844121	7.57148888	8.62848772	8.13817968	7.1856792	5.93327441	7.56072469	6.87901742	6.56933719	6.47541051	6.71852316	6.63585605	6.7944969
ACSM1	1.826923	2.6690742	2.07879984	1.68243228	1.42820108	3.7744653	1.68837424	1.41932674	2.36104074	-0.831615	1.08850314	2.65911447	2.38751685
AP1G1	5.97763936	6.08228549	5.94401251	6.6732941	6.51019994	6.40929054	6.50257844	6.54096161	5.99037952	6.15833498	6.42958294	6.50313905	6.20329559
SLFN5	6.54341373	6.54011129	5.23766073	6.67539516	6.13411104	6.02900455	5.44030722	5.78324999	5.8434464	5.52397447	5.45970747	4.69470773	6.45971885
CATSPER2	3.54443858	2.91411025	3.31897966	2.05743286	3.47000042	2.57572985	3.04691946	4.00787781	0.70519342	3.3753591	2.34290393	3.64562303	4.23743459
C16orf45	6.4430329	5.82713087	6.3580464	5.34538005	6.09259597	6.38523443	6.09877924	5.79478601	5.92826066	6.76521418	5.9572189	6.0280757	5.7143671
SAAL1	3.47425825	3.17832896	3.77776613	3.91227755	3.91529336	4.7044354	4.10357172	4.8770695	4.28896269	4.16563	4.18000756	4.40599668	4.0774796
YPEL4	3.30506526	1.35848265	1.88993857	0.8196543	1.1977358	1.08874571	1.79741277	2.02028171	-0.95800124	3.17562808	1.68777825	2.44561605	0.11425156
PPIB	6.96641834	6.76423927	7.12614861	7.76268908	6.26929628	7.09271806	6.23400052	6.50920008	7.26580155	7.08139568	6.84434267	6.82816724	6.8657185
LDHC	0.21903477	-0.04686114	0.39425571	0.03437766	0.00573794	-0.04546117	0.46562428	-0.04364875	-0.0774345	-0.0774345	-0.0774345	-0.0774345	-0.0774345
FAM96A	3.85405392	4.08138543	4.19445788	5.64377153	4.44565736	4.1852687	4.63567221	4.11303023	4.60128243	3.74773064	4.96603714	3.55715423	3.239111
FAM111A	4.62823342	5.36827298	4.60493158	5.90381801	5.67889631	5.23454045	5.60594539	5.54033016	5.96916316	5.36667546	5.61592292	5.04111578	5.1334749
KIF7	5.3094995	4.14773845	5.45033629	4.03745603	4.19763204	4.21337833	4.60914021	4.53786223	4.62926048	4.94915967	4.62865273	4.88052596	4.24891172
LDHD	2.30516489	2.04617352	1.85991321	0.10805541	2.11899408	2.48551973	1.31559487	0.68609259	1.73915021	-0.45176247	-0.45176247	2.00602609	4.11435826
PLIN1	3.24849392	4.70139732	2.61629104	1.94994716	2.68147244	4.24310136	2.88041012	2.33706587	2.23705335	3.403661	4.41640943	3.32893815	4.35646149
PEX11A	4.86992476	4.14653011	3.84068	3.51577341	3.60567456	4.54684779	4.54014127	4.3203071	4.28204567	4.83812143	4.05737523	4.05298165	4.02987888
TMEM170A	3.73045745	3.4112736	4.07465463	3.94512785	4.26820406	3.96063926	4.85177506	4.41565488	3.70682152	3.5898657	4.31738	3.89669967	3.82207244
MESP1	0.14215104	-0.08143472	0.16008655	0.38756351	-0.03710705	-0.08002284	0.2474925	-0.07819501	-0.16232561	-0.16232561	-0.16232561	2.21130131	-0.16232561
ANPEP	5.38104998	4.09920118	6.47968803	5.81231793	3.2819744	2.20726506	2.70629873	3.03723121	2.32109285	2.56381726	3.53370889	1.43021063	4.52130747
SCNN1G	-0.09216262	0.37752822	-0.09076477	-0.09216262	-0.09216262	0.20936573	-0.09216262	-0.0903793	-0.01417991	-0.01417991	-0.01417991	-0.01417991	-0.01417991
RBPMS2	3.62658711	2.05198508	3.3338497	2.06819851	4.41742322	1.5710993	4.20699569	4.24017219	3.74358058	3.28745578	4.88644204	3.91266226	5.96240833
NAV2	1.81413946	1.29057328	2.94460736	0.62041573	3.45719736	2.50164397	2.48778505	3.77063954	3.23248716	3.80648373	4.48199239	0.71566825	3.9469759
ANKDD1A	5.17308644	4.70699488	4.96572491	3.96573293	4.81847806	4.53610625	5.39077177	4.44336596	5.1159745	4.55272607	4.50212939	5.14799831	4.09866785
C18orf54	2.98258404	3.57221719	3.27941714	4.85522062	3.82383855	4.62866113	4.42919062	4.36562256	2.88806882	2.60373958	3.55188914	3.94649864	3.31544614
DCTN5	5.54072913	5.79778288	5.39031265	6.22898666	5.65975483	5.91118517	6.17597793	5.53827594	5.65120386	5.80415615	6.02704588	6.33170462	5.54788093
TERF2IP	6.06336672	6.34327486	6.42900501	6.22013293	6.72524197	7.04386699	6.6354262	6.79646954	6.67575279	6.75428368	6.05124945	6.31872484	7.08818459
PLK1	2.60909874	0.87164018	4.50918763	4.16888829	1.76233571	1.48636011	2.2557682	1.84573625	2.2062115	2.33760885	2.28159282	2.41712712	-1.09436526
CLPX	5.19457483	5.69342335	5.29146158	5.51853733	5.28790329	5.74559907	5.32100225	5.56161802	5.54417093	5.52206334	5.29220858	5.29098792	5.42989144
GPR182	0.19883378	0.57234992	0.20143338	0.39550697	0.40769772	0.20027912	0.29179683	0.42556672	-0.0929979	-0.0929979	3.05368456	-0.0929979	-0.0929979
ZBTB39	4.91431594	4.4873399	4.64114134	4.09290018	4.82987181	4.52856841	4.58658008	4.72434956	4.35777256	4.33600468	4.80978198	4.4983673	4.52346525
MYO1A	0.57616799	0.57223522	0.53871328	0.45553753	1.01802473	0.5884042	0.49729234	0.51661417	0.9365895	-0.27766379	-0.27766379	-0.27766379	-0.27766379

NAB2	5.12505465	4.88821833	5.72657388	4.42438292	4.41433224	5.28871765	4.85719734	4.81392437	4.9541384	5.44192046	4.80913261	4.69993585	4.48408218
VPS39	6.50851603	6.32089787	5.87434765	5.93659585	6.29095385	6.16499132	6.31680845	6.37643078	5.76870052	6.31142589	6.08058724	6.21787879	6.65010059
STAT6	7.87321897	7.05386784	7.60429898	6.33922328	6.8120265	6.7639473	7.6943348	6.95190967	6.58044738	7.53003512	6.98793095	7.18941909	6.93038592
PATL1	6.08178232	5.53217974	5.57435301	6.44527578	6.08008682	5.23025442	5.72266339	5.4804109	5.87342995	5.61482035	5.20922984	5.38293693	5.72318682
ELFN2	0.3100327	0.46699917	0.31263595	0.3100327	0.3100327	0.38377159	0.3100327	0.31335383	0.05369502	2.97446094	0.05369502	0.05369502	0.05369502
STX3	3.40512822	2.07612268	2.68618202	2.79896742	3.40885383	3.05537092	3.77831225	3.85659674	4.2992786	-0.55634495	3.79129242	3.35238622	3.82011
MRPL16	5.37275187	5.088446	5.21382575	5.39489455	5.09221311	5.2260581	4.76312574	4.5802392	5.0238413	4.49184595	4.57888114	5.1698648	5.38919023
PIP4K2C	5.5827483	5.61706472	5.2378938	4.27888083	5.81007123	5.78666875	5.67508865	5.52717718	5.41494935	5.80816342	5.38776177	5.28701897	5.84580772
MTMR10	5.63479527	6.09586988	5.19656509	6.1660529	5.9891763	5.90022461	5.93974641	6.08648009	5.73439051	5.1415679	5.94458943	5.62468294	5.68774382
YWHAB	6.80900268	7.50157994	7.15416985	7.64301456	7.45384391	7.39207682	7.74552439	7.1643395	7.47134418	7.19902311	7.30504082	7.2662688	7.54426683
C15orf48	1.57652573	0.70659329	1.32686127	1.74922307	0.07222587	-0.31157065	-0.13353724	-0.30982541	-0.7411297	-0.7411297	0.42477592	-0.7411297	-0.7411297
SCG5	2.94595136	3.1136121	4.09869638	3.73921387	2.77061488	3.5896099	2.02576323	2.95153355	2.79404863	2.79266856	3.40410381	2.65776058	2.99216197
GREM1	0.28670969	0.55270576	0.26305119	1.47756761	0.26566566	0.17614589	0.53255704	0.45439817	0.28393239	2.59031847	2.76486743	1.22471032	-0.17506475
TSC22D4	5.31767658	4.23735786	5.59635428	4.07695333	3.98100778	3.74769088	3.88859689	3.96499573	3.38706402	5.1557303	4.02940769	4.14654408	2.24526851
MS4A6E	0.0307332	-0.21846652	1.48821408	1.21361541	-0.21846652	-0.21756392	-0.21846652	-0.21639542	0.01297499	0.01297499	0.01297499	0.01297499	0.01297499
MS4A7	5.09690855	5.75342454	4.99486587	6.20434948	5.03263038	3.73067854	5.1841562	3.97692384	5.08410882	3.72535367	5.18586606	4.64321813	5.17315478
MS4A14	1.80747581	2.34843002	2.4327286	4.32121885	2.68999307	1.5340556	2.53192162	2.34843002	2.76132533	2.4319211	2.76882947	2.37015767	4.19463418
DIS3L	5.71870916	5.44037596	5.29998924	4.90532384	5.14774842	5.67742174	5.71642644	5.32651599	5.85621126	6.00603877	5.70958897	5.52284371	5.75019508
CCNDBP1	5.36270439	5.70002516	5.40404452	5.16631864	6.23436574	5.92853593	5.90200776	6.29653715	6.15651121	6.32940633	6.17128621	5.92963309	5.73391163
EPB42	0.1414815	0.8471654	0.06970439	0.0343095	0.07802153	-0.02388534	0.00899227	0.35842872	-0.04984511	-0.04984511	-0.04984511	-0.04984511	-0.04984511
SMAD3	7.1158716	6.1358989	6.77066341	6.63483525	6.05311553	5.75304942	6.26035807	6.53467321	5.80942349	6.36462041	6.33231922	6.2872623	6.04876539
MAP1A	6.38965917	5.96267222	7.13357248	6.4340437	6.40660672	6.57912556	6.53642566	5.92601106	6.89166829	7.23388408	6.72571014	6.38224507	7.05754355
RCCD1	4.27573942	2.78635762	4.48171823	3.52695958	3.30582778	3.27933788	3.86245025	3.32118162	3.98734168	4.58096714	3.66294508	4.36305167	2.83119589
AKTIP	5.43012217	5.7264738	5.08318088	5.16975686	6.18824094	6.12472648	5.95686077	5.94037072	5.9184379	6.01382894	6.26317864	5.91377492	5.59326234
MAPRE2	7.046123	6.91491919	6.35677533	6.52129411	7.29935114	7.1340122	6.50640101	6.27684662	6.73416775	6.32333436	6.78282113	6.46818977	7.20288662
MARS	5.60561119	5.5040209	5.76779756	6.08995747	5.66041835	5.0827749	5.75920503	5.40215305	4.95370677	5.67441146	5.64055633	5.26644832	5.7011218
MBD6	5.23385104	4.29361279	5.28278858	4.09237675	4.72469741	4.00659794	4.5562746	4.64785055	4.24489137	4.94542346	4.76415172	4.40307557	4.818572
CNPY4	5.17331652	4.36705197	5.26004013	4.94180404	4.21201405	4.68853818	4.7233406	4.05870925	5.05614835	4.91401303	3.91697671	4.93150254	4.86205686
PDIA3	7.00847704	7.44218335	7.56884009	7.88894639	7.23888041	7.90713159	7.16676143	7.09743109	7.63373313	7.46994713	7.46402585	7.1031509	7.73272833
NUDT21	5.75669454	6.83063777	6.23791518	7.25908985	6.95523093	7.18804502	6.84857153	6.96288835	6.99376352	6.21710398	6.78151238	6.93970407	6.08645634
NKX3-1	4.36151699	4.0264069	3.22482565	3.20639974	3.8384623	3.89865071	3.3880599	3.77056904	3.28005314	3.13507997	4.50537817	3.31636966	1.50989417
SGSM1	5.91719852	4.84326051	5.33160644	3.32974342	4.94123845	4.61082435	5.27888842	4.1315106	4.25168592	4.23369745	4.96221262	4.26366719	5.19609969
DUSP18	4.73365347	3.80237283	4.64501205	3.9626573	3.65831522	3.58512621	4.02357977	3.63575539	3.75420833	4.23702395	3.9227171	3.56235137	3.75121738
TEF	2.69879809	3.37277375	2.4112172	-0.4435385	0.75561006	1.89602299	1.49058451	1.03632958	1.73383631	3.93256652	3.7754621	3.97502387	0.18403062
MEI1	0.6788611	1.10087668	0.50671173	0.58419697	0.8329804	0.08576568	0.32112545	0.46391297	0	0	0	0	0
PBX3	4.60628517	5.40710787	4.58063228	5.25916097	4.75738271	4.78899716	4.7792525	4.12082427	5.2380797	4.42355688	4.83904639	4.3800985	5.28839964
GNGT2	1.46970234	0.98049273	1.31409827	1.87327134	1.35553355	-0.32610682	0.88614457	0.50115783	1.30920218	3.39434732	3.97524104	-0.48279739	1.69009291
PHB	5.83545093	5.61057213	6.22749839	5.47199683	5.4600423	5.76247723	5.12358336	4.90814305	5.49543627	6.26241761	5.41507244	5.68816963	5.04289496
SNRPD1	5.3137346	5.56960934	6.1503055	6.14719069	5.38675113	5.38063678	5.54270766	4.85725998	5.41437016	5.29995184	5.17920045	4.7118441	5.72733837
TTC16	0.71524044	0.21289451	0.21548089	0.43982763	0.38222835	0.35944835	0.40856777	0.30373355	2.33433176	-0.15768225	1.5537765	-0.15768225	-0.15768225
SAMD14	4.01671908	1.8794469	3.30542003	1.61444632	3.02125403	3.44538228	2.34770393	3.10867235	1.24458505	3.79685239	3.34880737	3.80623226	-1.1240609
PIP5KL1	0.70647843	1.49053749	2.0114345	0.4755126	0.91522444	0.8490424	2.04122001	1.12000896	-0.41143751	2.56158441	-0.41143751	-0.41143751	-0.41143751
TMEM92	2.61664971	1.17235094	1.40568251	2.35747497	2.09952231	2.1568701	1.42753441	1.11224204	0.0370577	-0.89347924	2.1653226	-0.89347924	2.49426907
FAM102A	5.03987155	5.06118609	5.10029936	4.18338072	3.92631453	3.48605759	4.33191442	4.43096724	4.36582821	5.03274422	4.59793934	4.68349356	4.51224621
ACSF2	3.11272471	2.4105061	2.27866748	1.48316265	2.08825374	1.01887436	1.86897387	1.83238005	2.91137055	3.21707709	3.82053155	-0.08778243	-0.08778243
GOLGA2	6.59591494	6.54042985	6.85862067	6.12529234	6.17201769	6.04843807	5.96963431	6.1850734	6.79590465	6.82797627	6.71076879	6.5344477	6.59275468
TRUB2	4.3564785	3.80965903	5.02521659	4.07999406	3.75936434	3.94825237	3.82931243	3.67337257	3.84822111	4.34999129	4.16106919	3.6909634	4.65296535

COQ4	4.25635634	3.86515023	4.60536122	4.26298406	3.7831594	2.71825361	3.45057726	3.62700564	3.44436606	4.41843319	4.73762434	3.85222799	3.74234668
SLC27A4	1.33182325	0.56991134	2.18520266	0.34155445	0.57761724	0.4107709	0.93048125	0.62153056	0.35054333	0.35054333	0.35054333	2.54221175	0.35054333
URM1	5.16039228	4.94009815	6.07415513	5.5379021	4.77154992	4.71314446	4.55976622	4.66668668	4.88951901	5.36635032	4.0273636	4.71163143	4.29289954
CERCAM	5.30358122	5.83731409	5.81955841	6.79725871	4.88871411	4.66355497	4.98103309	5.54975531	5.22581044	5.57803499	6.35512066	6.0439116	5.42615341
DOLPP1	3.72601416	3.72274764	4.23159919	3.25541079	3.2416841	3.8284611	2.99337881	2.49890508	3.33606874	4.55902918	2.2753533	3.74477763	2.89656911
ENDOG	3.60183788	3.04639614	3.42237167	1.83174902	2.9615509	3.15340172	3.53922648	2.75404066	2.42406514	4.34904581	3.17183299	3.09751317	3.00404119
PRRX2	3.57778226	1.07973346	3.07199264	1.19524923	2.39399993	2.66769069	1.17475478	1.31946373	2.05431314	3.93041298	0.10061001	4.61795459	0.10061001
C15orf39	5.87829952	5.27845862	6.6253257	5.06886922	4.47153597	4.44718297	4.61911825	4.66381181	4.32316492	5.136155	5.09084101	4.7056236	4.88442473
ISLR2	0.23860229	0.35999752	0.33285766	0.39062685	0.65021787	0.39610945	0.5383886	0.459934	1.25374511	-0.26808626	-0.26808626	-0.26808626	3.84846207
SP2	5.93780959	5.26645701	5.72004281	4.73219452	5.4097348	4.96711103	4.81480106	5.10006233	4.89974952	5.77656868	5.33146033	5.20134996	5.23238641
PRR15L	0	1.1898305	0.00124472	0.1111395	0	0.00069205	0	0.00158797	0	0	0	0	0
COQ7	4.97725312	5.42279267	5.03338548	4.36588464	5.5423452	5.40937959	4.90608801	5.42472227	5.53402085	5.37273475	4.76296501	5.32835265	4.69533385
GPRC5B	4.28322658	4.10857975	3.79512631	4.04442529	5.43705443	4.7062913	4.73399655	7.02359606	4.99370278	5.92349395	5.22981827	6.1319498	6.19021627
CRK	6.54932452	6.58982167	5.95254956	6.87634681	6.50080077	6.55717936	6.84194509	6.31735606	6.07881435	6.65529543	6.22950809	6.34433018	6.59915711
FBXO22	3.78476348	3.75156401	4.02312763	4.54223764	4.45801017	4.78436284	4.76999288	4.37352151	4.37664184	3.5446013	4.16010172	4.34758278	4.11123311
TBC1D2B	6.46371181	6.81341976	6.22523976	6.47864858	6.05051069	5.89088511	6.18215634	5.62265449	6.29042061	6.42361633	6.88023835	6.05092873	6.59996005
NOD2	3.7256281	3.05076065	4.05152852	4.24154043	2.29466805	3.16276966	2.72676164	2.18881469	3.38873559	1.73887506	3.69797227	2.38727841	2.75427485
SNX20	2.99556113	3.30830815	2.48389658	3.05123778	1.49443863	0.68546486	0.97662545	0.24180831	3.16619189	2.87209648	1.24885652	2.00431366	3.30576892
LOXHD1	0.63907008	1.19253389	0.45443226	1.91739062	0.59592407	0.45331926	0.59396276	0.61839722	-0.37940121	-0.37940121	-0.37940121	2.28166738	2.88937524
KATNAL2	1.91844451	2.47322882	1.55128353	2.69411399	1.33979723	2.63865202	2.92217431	1.90463801	2.95696485	3.43516372	3.0371541	-0.87580778	0.1670478
HDHD2	4.82453007	4.96229561	4.37839783	5.6674028	5.5307973	5.41724421	5.41103691	5.91493164	5.34966993	4.56108125	5.09213644	4.9444379	5.41416014
ZNF91	5.19766632	5.34976703	4.796671	5.5120856	5.76383124	6.27381218	5.74735498	5.71735585	6.47835388	4.97868718	5.82611986	5.09472833	5.56032166
IGF2	1.80216807	6.21541079	3.21199352	5.30044024	5.54769349	3.31713009	2.99452094	4.56042797	6.00948753	6.09083635	6.18450276	5.28973345	6.98843462
RNF214	5.89493986	5.546172	5.74676269	4.99231733	5.80117697	5.59603727	5.84244117	5.39737473	5.63719164	5.68931936	5.3382455	5.22707913	5.80967237
CDK12	6.34351013	5.88691447	6.1284615	6.10570271	6.50486722	6.02727558	6.3318721	6.18960599	6.12615617	5.85621652	5.8900836	6.14827287	6.3872494
DPEP2	3.63189361	1.70698999	1.83703519	3.42626118	1.211897	-0.10974033	0.12539574	1.05934812	2.495128	3.55263015	3.46307623	1.47106883	-0.59402485
POP5	3.48006864	3.1319561	4.07734335	3.41283553	4.08145186	4.21817261	3.63762808	3.69549878	4.75586836	4.19050971	2.54049322	4.26543121	3.49056728
ENGASE	4.36374332	4.7174213	5.04745404	3.98875306	4.67887129	3.1672629	5.03305985	4.53355921	3.91477806	2.99314562	4.51427798	4.23947596	4.17029005
ATP5L	5.49072247	5.15034999	5.28676878	5.79751902	5.91144942	6.57956508	5.50482714	5.0940252	6.34042222	6.48041819	5.47035031	5.19036468	6.35432878
CD3D	3.50658141	3.12261419	0.56858011	1.90410448	2.14846914	0.50933892	0.77087452	0.40419082	2.71332756	-0.41978114	1.7031144	3.31994406	2.33685618
TBC1D16	6.3600348	5.52754311	6.55948175	5.38090446	5.43510476	4.96729177	5.53345258	5.62797471	4.85597095	5.16406375	5.41617721	5.37634465	5.21582862
MYO5B	4.22825248	5.00891803	5.60366592	3.71117426	5.85198976	6.12146904	4.02618288	5.24523195	5.88302612	6.17089773	5.29457067	5.98111201	5.36208388
ART5	0.56743451	0.15072668	-0.12150754	-0.15596145	-0.22620108	0.06060234	-0.22620108	-0.13025184	-0.03688913	-0.03688913	-0.03688913	-0.03688913	-0.03688913
ACAA2	5.38708299	5.71621108	5.31025435	5.53758886	4.81267517	5.72356834	4.84743155	4.95394031	5.51777238	5.71208164	4.82765899	5.4969779	6.11784349
STIM1	2.93697346	1.96189219	2.37769937	1.3861233	2.85552314	2.91413781	2.63180279	2.55032115	3.33098389	4.26181677	3.80559555	4.35366473	1.94187294
RRM1	5.28339166	5.00475592	5.62654616	6.56267248	5.40679314	5.81205756	5.69092855	5.67237321	5.3722845	5.44578171	5.34541383	5.19795867	5.51788034
TRIM68	4.83120312	4.12749061	4.41604656	3.74863191	4.17634897	4.45517473	4.35207642	3.91560481	4.07509005	4.66443861	4.23575598	4.10641403	4.16034539
OR51I1	0.38813831	-0.37951085	0.74594997	-0.37951085	0.29528977	1.39625221	-0.01789515	-0.18763288	0.00712583	0.00712583	0.00712583	0.00712583	0.00712583
FN3K	5.01171336	4.53275472	5.04806112	2.66552045	4.98762567	4.16665609	4.67455273	4.63548989	4.02677162	5.32436881	4.91573485	4.86295134	3.67301773
PRRT2	4.51197895	3.35298279	4.70705752	2.12118001	4.13785616	3.51365944	4.63854635	4.71776276	4.7540474	4.87047082	4.64447499	4.37176588	2.88005541
ZNF23	4.07135789	4.06089897	3.92189612	4.13073831	4.63036823	4.57761648	4.32539819	4.24366695	4.87831237	4.56804342	3.94050459	4.46069768	3.96896072
IRGQ	6.03594818	4.80202045	6.26223635	5.1417596	5.3777038	5.16138502	5.72582455	5.34874365	5.14547321	5.71485615	5.67950616	5.73711181	5.81144315
ZNF226	4.51889572	4.9619819	4.29442143	4.81284845	5.1504912	5.25075496	4.88430544	5.1252844	5.12498613	5.2459252	5.08523175	5.37186332	4.7297331
ZNF180	3.73306576	3.75790622	3.9371682	3.96702693	4.56419671	5.04768273	4.89186741	4.44914917	4.6584208	4.25282138	4.87750299	4.21453931	4.46917688
PPP2R3B	1.59383333	0.55914117	2.46986314	0.7327202	1.04953307	0.31773544	0.79994254	1.23832534	0.52330609	2.59825578	1.16845238	2.94952791	2.34549787
ZNF668	3.41507559	3.81532259	3.84003981	2.790686	2.21239145	2.58446547	2.80247856	3.06569099	3.21834804	2.16976487	3.81092703	3.98249154	3.04639981
ZNF646	5.55970263	4.65329734	5.4426935	4.26832804	4.67625018	4.48794171	4.5640794	4.62654755	4.50470618	4.88268997	4.8284217	4.73086124	5.29300558

VKORC1	4.37057008	4.09966328	4.68515519	4.25811116	4.54505648	3.94744425	4.14246021	4.8220569	4.78772267	5.50065056	4.5038741	4.84431655	3.69180774
GNG8	1.3274748	-1.14144366	1.23200295	-0.15796271	1.87554057	0.03335019	0.56805529	1.24597131	0.10412861	0.10412861	3.32419664	0.10412861	0.10412861
LPO	0.63072634	-0.04865419	-0.04636167	0.1299768	-0.04865419	-0.04737958	-0.04865419	0.05040714	-0.03239854	-0.03239854	-0.03239854	-0.03239854	-0.03239854
CA4	1.68650997	1.32993922	0.76088809	0.75843182	2.54979108	3.47084026	0.89661555	2.07745951	3.69172754	4.54562286	4.13055028	1.10977183	5.94399724
SMG8	4.83525116	4.64285805	4.18729344	3.85919741	4.16053959	4.94994015	4.3455705	4.21384326	4.22172203	5.03857968	3.95674292	4.54182356	4.50320685
TPM4	8.72585386	9.32206193	9.10221107	10.3954943	9.91102439	9.2030508	9.67529755	9.40223219	9.15078734	8.91476427	9.24605824	8.79957561	9.30297149
RAB8A	6.1550142	5.63436125	6.20439209	6.1084113	5.63885987	5.90387015	5.37444314	5.29946592	5.92384754	6.09910444	5.40395185	5.70147103	6.44236774
GPX4	7.11479542	6.78108277	6.68776923	5.98531075	6.13514622	6.36055397	5.66770148	6.73229773	6.94108746	7.43057561	6.85190436	7.17469318	6.48038726
MIDN	6.5456836	6.09696923	6.66529872	5.50017962	4.54912888	4.70266425	4.33539063	4.62770429	4.05850239	5.32177652	4.92225124	5.08563722	4.71577104
FAM129C	0.22735017	0.71654768	0.49384651	0.33018995	0.3215807	0.27784981	0.10890674	0.07901726	-0.19633727	-0.19633727	-0.19633727	-0.19633727	-0.19633727
KLHL26	4.0378954	3.3635921	4.5107476	1.57022247	2.79439453	3.31032713	3.69621947	3.76657662	3.59749054	5.09444318	3.41901879	4.26055388	-1.21461872
GATAD2A	4.25332754	3.5249041	4.76482782	3.65575489	3.43911715	3.01813496	3.15010739	2.85805372	3.71348531	3.78389299	2.90473285	3.54620599	0.71766955
MVD	4.0153325	4.13903057	4.91050481	3.24729097	3.49303123	3.17156743	4.2052889	4.33121007	4.11883169	4.10494479	5.00249866	4.22396398	3.42063871
CDT1	1.27026375	1.1706808	3.68718291	2.64165922	0.23027978	0.20784189	1.46000182	0.54707827	-0.73956782	-0.73956782	2.36111469	0.8729409	-0.73956782
TRAPPC2L	4.87322381	4.68969496	4.64820635	3.88397418	3.88561463	4.29756678	3.82003952	4.37906906	3.97085383	4.380327	4.78833303	4.74456499	4.39104011
ANKRD11	4.89007054	4.14785404	5.31107981	4.01248521	4.32728125	4.17939791	4.15658062	4.37306977	4.02725464	3.6645139	4.41079558	4.47437309	4.60943225
SGK494	3.70082506	2.54817603	3.73320844	3.72516254	3.37705574	2.51997607	3.49117037	3.02295307	2.96502483	1.99875662	3.48909073	3.15475113	4.06344437
PROCA1	1.30831585	0.74584146	1.86429463	0.77121585	0.81800912	1.10341421	1.62642384	0.22521476	3.60140503	-0.36987319	-0.36987319	-0.36987319	0.66353157
RPL13	6.95137106	7.30428407	7.0230733	6.21621409	5.74845567	6.1560511	6.00145957	6.13116803	6.7187986	7.55672847	6.66000592	7.06647086	5.91609563
ZNF641	5.28931472	5.83364623	5.71369907	5.7553227	5.86711234	6.19716101	6.03067211	5.9764223	5.76361727	5.71774586	6.42817023	5.50380449	5.90683304
CACNB3	5.03994844	4.27506179	4.71314185	3.50264634	4.42551204	4.04811631	4.84788895	4.67447423	4.32706852	4.8934019	4.19146589	4.65063858	3.95258901
DHRS13	4.87098623	2.67182364	3.0974313	2.09676955	1.93979978	1.29959225	2.58336041	2.24739147	-0.24722239	3.7216862	-1.16857158	2.48044286	-1.16857158
TP53I13	3.56561205	3.0999019	4.84766068	3.47393517	3.30306379	2.91484602	3.15235699	3.38083873	3.8101398	3.25565528	4.03472036	4.40697439	1.50588106
CORO6	3.77372375	2.22068171	3.52560347	1.54960985	3.8627607	3.89582067	4.13577023	5.00339971	3.46672351	4.75576252	4.63025569	4.41583549	5.38406165
RHEBL1	0.50473119	0.83406726	0.31425616	0.19164276	0.36800318	-0.19561652	0.85836275	1.06892492	-0.25257451	-0.25257451	-0.25257451	-0.25257451	-0.25257451
TUBA1A	7.57400398	7.69926286	8.39234677	8.68266965	7.39832195	6.956911	7.74011938	6.59921622	7.37382018	8.0148244	7.64558078	8.19322108	8.20563805
TUBA1C	6.80316094	6.42028038	8.11351926	8.08553169	6.32655128	5.94781542	6.34529868	6.01551418	6.17905831	5.96605157	6.43526921	5.57073973	6.80111544
ZNF610	3.26831413	2.22974696	3.46510944	2.9054632	4.18780937	4.07015691	3.46291554	3.31848164	3.6270269	3.96675453	4.16889443	3.88972954	3.25841705
ZNF528	4.64720499	5.06251202	4.63762575	4.80713142	5.7885737	4.63340786	4.95616892	5.49682498	5.55194614	4.81185732	5.20593192	5.02413574	4.96081324
ZNF701	4.71592634	4.89545139	4.92729454	4.43856871	5.13471945	5.03234775	5.20537174	4.86423857	4.87585223	4.1258198	5.15536457	4.63514674	4.78199113
SERTAD3	4.2464132	4.64344747	4.29601437	3.88198491	3.67625267	4.49008628	3.3320606	3.74436726	4.08341794	4.91886045	4.32854481	4.37916152	3.58434548
NCKAP5L	4.77777461	4.38589406	5.38079111	4.51994059	4.23307183	3.72348994	4.55332923	4.71770824	4.43956173	4.62336153	4.68304369	4.50854542	3.7383813
RAB4B	2.13769562	0.95161481	1.94818468	1.46107814	2.80829766	1.97734463	2.23921161	2.84624735	1.09875215	2.47180526	3.15133254	2.04738578	1.56551462
AQP2	0	0.20571006	0.00124472	0	0	0.00069205	0.05587777	0.11662256	0	0	0	0	0
GPD1	1.40917455	2.48289824	1.87532657	0.81375243	1.57461639	3.63493149	1.3664941	2.17219303	2.33820564	3.13098663	3.04634792	3.57118059	3.43124967
CYP2S1	2.66906535	2.02316616	4.3181654	3.22062307	1.40641609	1.38089871	1.06586275	1.09856747	2.94035677	3.2074967	1.98878936	1.13142267	3.58421178
AXL	5.53045386	5.3958087	6.02967558	5.31492731	5.28156193	5.03074639	5.47498347	5.94996767	4.82522076	5.82235851	5.71101359	5.64809403	4.64213319
NFKBID	3.42850751	1.55940557	4.67264204	3.21237796	2.94100204	1.5446155	2.16321882	1.7667887	3.33636992	-0.90546056	3.02682537	3.03519999	2.83571713
TMC4	0.17902056	-0.05974484	0.55191136	0.38737927	1.39421501	0.86152565	0.21401102	1.45530119	1.71886569	3.36999239	-0.50368387	-0.50368387	-0.50368387
LAIR1	5.3993343	5.26733607	5.43858694	5.6520748	3.55905876	3.34187046	4.01467769	3.22984657	4.41206075	3.54754648	3.71568398	4.08474552	4.56401057
TTYH1	0.23163947	0.23163947	0.72467862	0.28188501	0.36868646	0.23307791	0.31757811	0.49941855	-0.14092764	-0.14092764	-0.14092764	-0.14092764	3.34434022
LENG8	6.8602384	6.66893757	6.96626931	5.97228004	6.92047115	5.49504774	6.53592105	6.83727305	6.41064553	6.71630443	7.24442565	6.61925897	6.5048857
CDC42EP5	2.30476155	3.63654523	4.08140053	2.85871963	1.87099121	1.84133764	2.09610052	2.88774533	2.80299516	3.18781892	3.50132309	4.31098636	-0.60796704
LAIR2	0.46820821	-0.29447653	0.40685288	0.55752287	0.70805511	-0.29327075	-0.29447653	-0.29170974	0.04158873	0.04158873	0.04158873	0.04158873	0.04158873
ZNF526	4.96378773	4.32260993	5.22461727	4.01438199	4.47276613	4.27005202	4.34597016	4.52615784	4.18237618	4.81060384	4.90056565	4.73141123	4.37414717
TRAPPC9	4.59550189	4.49154115	4.62069911	3.88875112	4.09994504	3.57616312	3.99017126	4.43975474	4.16239233	5.05032447	3.69491232	4.38985957	4.98806668
KIR3DL1	0	0.25457323	0.00124472	0	0.03231374	0.00069205	0	0.00158797	0	0	0	0	0

ZNF146	5.25017857	5.83667635	5.68191797	6.44185876	6.15331865	6.7283709	5.96385739	6.14274634	6.69651688	5.46508724	5.63361258	5.91633684	5.89518204
ZNF283	3.80817907	3.86353745	3.7712761	4.56562799	4.81396092	4.90459548	4.59048267	4.92788855	5.05516729	3.43732681	4.56203722	4.40454747	3.28310205
PPP1R14A	6.24827766	7.2245801	7.13128476	4.43431865	7.80451386	8.15667407	6.81903209	7.70612303	7.40613507	8.19361816	7.27677394	7.80153291	6.20735978
SPINT2	5.94349679	6.20269421	6.03510044	4.10644717	6.17899646	6.1529331	5.6572405	6.34432144	6.36201437	6.96687248	6.43981572	6.59585053	5.82352906
C19orf33	0.41212962	-0.262015	1.13703159	0.37929439	0.87810159	0.58779633	2.19790118	0.72085487	-0.25038146	2.82583056	-0.25038146	1.18849426	-0.25038146
YIF1B	1.03603466	1.34842863	1.55912857	1.71163287	1.53864379	-0.70684459	0.27494805	1.21953738	0.43968723	4.26928154	0.43968723	0.43968723	0.43968723
PSCA	0.99207717	0.60863765	0.40315708	0.80935608	0.04771269	1.59662224	0.50712866	-0.03393222	-0.37636732	3.12240728	-0.37636732	3.20547424	-0.37636732
DAPK3	5.25346804	4.9974764	5.47906968	4.26629865	5.70045746	4.96692111	4.72075916	6.04910773	5.52605372	6.10499473	5.64633628	6.10070259	4.49471547
EEF2	8.9826867	9.49870774	9.49870774	9.11885292	8.63491153	7.89666438	9.00688604	8.63491153	8.88189242	9.44546257	9.12997093	9.35102772	8.8329088
CHAF1A	3.94822954	3.60443292	5.2537614	3.77564582	3.13893963	3.46837781	3.6563668	3.60567843	3.85751282	3.74422936	3.93097676	3.92935504	4.31786583
UBXN6	4.39076966	4.26409336	4.97154741	3.1554261	3.65977304	3.54860799	3.5946252	3.98426025	4.28575054	4.44409712	5.2168579	5.03922926	2.60827841
PLIN4	1.83327147	3.88652083	1.61968475	1.99821205	2.04360677	4.35217331	2.67873387	3.94107054	3.61564713	4.84277609	4.65872767	4.58841928	5.37396262
SEMA6B	2.99987335	3.58498048	2.34160666	4.18107153	2.97499389	1.11956828	2.81817924	1.92667157	2.51235477	3.02800092	1.16097616	2.67880379	3.714373
ZNF444	4.48799763	3.94747526	4.75071129	3.12442474	3.79256373	3.61762883	4.15370958	3.91443783	2.81593083	4.45626544	3.99257243	4.72582331	3.75363756
NXN	3.85350578	4.28293598	4.08928639	4.57264468	5.11732508	3.95783385	4.85230387	5.80229131	4.51226247	5.92009119	4.59243586	5.19819184	5.37106674
FAM57A	4.61089683	2.68595204	4.05860918	5.00698333	3.71131506	3.23936452	2.9358004	3.01834444	3.54162416	4.2326207	3.72425688	2.55860128	2.03667888
GLOD4	5.70283529	5.85029156	5.3427841	5.60143871	5.56158035	5.43754373	5.68017078	4.94647813	5.73454223	6.20278016	5.398884	5.83533874	5.71879054
MFSD3	2.05839043	0.6336192	2.77787923	1.06397086	0.81499889	1.1145006	1.20614965	0.8848146	-0.5038556	4.2024693	3.09313506	3.00968714	2.05262316
GPT	0.82150773	1.26826532	2.10058942	-0.25461225	0.08684574	0.44547326	1.34721314	-0.39703929	-0.06614202	-0.06614202	-0.06614202	2.38782484	2.51165416
KIFC2	3.70005819	2.89661037	3.7437751	1.9820781	3.29037839	2.45827528	3.25459539	3.90811631	1.99277088	2.19677858	3.05154265	3.95464918	1.77337787
SLC43A2	4.94523506	3.81860032	5.71534975	4.90852881	2.47843298	3.02973108	2.58627017	2.80278107	3.28136062	2.91625621	3.02910072	3.48936377	2.5676469
RILP	3.99646778	2.99926778	4.22698032	2.41214966	2.44784409	2.86149523	2.70274331	3.40661357	-0.65819248	4.39441068	3.63637403	3.23628453	4.14483798
SERPINF2	1.47999828	0.68053483	0.90736666	1.60171965	2.44506398	0.52965699	2.35315544	1.2767504	2.63479915	-0.49444192	-0.49444192	1.69645219	-0.49444192
WDR81	4.59736113	4.88841513	4.87434968	4.46952318	3.9929259	3.3045673	3.94462716	3.94257452	4.13546411	4.45267982	5.19783212	4.50939219	3.61751258
SRR	0.92109838	0.5104508	1.23232351	1.8014029	0.91083728	1.93530154	0.75738124	1.74716641	-0.06807874	-0.06807874	-0.06807874	3.56191857	-0.06807874
TSR1	4.97807921	5.10479969	5.34117395	5.29335582	4.88703983	5.01151475	4.70797619	4.82937095	4.57554553	4.47386875	4.93855157	4.87654041	4.95128468
TRPV3	0.66576762	1.17058139	0.92144978	1.15543944	0.7156726	0.78726898	1.32653774	0.89467685	-0.35042197	-0.35042197	3.13320794	-0.35042197	3.15427501
HSD11B1L	2.28363664	1.7320238	2.1433869	1.7464338	1.23461612	2.13316548	1.79293993	1.09047728	2.58715037	-0.70189119	1.68656536	2.93291623	2.69336429
CYB5D2	5.21045175	4.79843805	4.26559899	3.92109047	4.75432247	4.60911043	4.4673181	4.46438143	4.74330481	4.76678637	4.76426906	5.12677983	3.82706518
C19orf48	2.85198116	2.77731919	4.70453656	4.17619797	1.81512208	2.22333621	2.24631908	2.41777981	3.63371789	3.92134186	3.8854941	3.53993515	2.35937898
KLK5	-0.02471903	0.39151272	-0.02229302	0.26805775	-0.02471903	0.47845551	-0.02471903	-0.02162401	0.00379053	0.00379053	0.00379053	0.00379053	0.00379053
KLK11	-0.1311156	2.18080106	-0.13002119	0.37051833	-0.1311156	-0.13050712	-0.1311156	-0.12971939	-0.01478305	-0.01478305	2.91602519	-0.01478305	-0.01478305
ZNF83	5.58896234	5.7075272	5.88684113	5.94712354	6.4548722	6.04723978	6.52306112	6.85249855	6.58254808	6.39130216	6.39815931	6.00364823	5.79571719
KRT80	0.9957352	0.80152683	0.99693182	1.55268783	1.21401339	1.07510202	1.04692792	0.82293819	-0.38157589	-0.38157589	3.32274459	1.05463738	3.11322032
OTUB1	5.16511158	4.54822493	5.32415952	4.33325205	4.65523069	5.22089239	4.0651299	4.94734178	4.91835093	5.15987155	4.77252972	4.85906623	4.27927531
RCOR2	3.18951293	1.02555734	3.73557879	2.00816545	2.00870926	4.15748724	2.10468409	2.40043739	3.61181903	4.2041054	1.3866783	4.34041107	3.71911754
ANGPTL4	8.47458365	3.10029968	6.56266335	6.9964727	2.18903978	0.99356317	1.27796439	0.80875248	2.11602985	-1.60477438	-1.60477438	2.12423223	1.742248
CD320	4.43279878	3.85625174	4.97845883	3.77215083	3.20876495	3.35177806	3.62471806	2.91267096	3.30459249	3.36520871	5.00121889	3.85491825	3.49857825
SPRYD3	6.435255	6.99811592	6.82377686	5.36703601	6.82096935	6.62184357	6.28624254	6.61690346	6.24420177	6.96331132	6.47868054	7.01416628	6.13128123
IGFBP6	3.88898761	6.20788775	5.17677741	4.08197704	5.01074049	7.20775054	4.93832362	6.21785831	6.10577897	6.51313572	6.33585419	6.37919395	5.97307178
SOAT2	0.5022904	1.34131664	0.46277577	-0.16123043	1.0861329	2.14316619	0.35613611	1.37351882	0.02736967	0.02736967	2.6751333	0.02736967	0.02736967
ZNF558	4.86333927	5.05275476	4.60720021	4.67351789	4.94850638	5.24051321	5.02835676	4.80698973	4.60696887	5.0295163	4.82090494	4.38975934	4.72356092
NDUFV1	5.54014288	5.43318176	6.16227116	4.90921114	5.06847565	5.17647323	5.50614786	5.75648129	5.42641855	6.08891344	5.60081795	6.05521991	6.30086575
CDK2AP2	5.27103078	4.85603818	5.60784614	4.36419153	4.83798014	5.5637671	4.57079997	4.4893548	5.5188943	5.05233045	4.98561231	4.58085516	5.033998
NUDT8	1.73684911	2.00715143	2.82127612	-0.11446154	0.9410265	1.5432388	1.51696349	2.04774595	3.16443404	-0.51396806	3.37172067	2.71963457	3.69971079
PRDX2	6.17037793	5.8508647	5.98687216	5.35858261	5.79809639	6.55179737	5.28082177	5.56069682	5.59490513	6.309082	5.69245531	6.01049277	6.15270244
ZNF232	3.71648545	2.99687834	3.97889991	3.22117903	3.70120993	3.74617062	3.37043321	4.65619722	4.09983154	3.26187199	3.07933506	4.30374322	2.20272193

MIS12	4.35650061	4.87469522	3.82379594	4.78330775	4.70947498	5.06492665	4.70875423	4.71491058	4.58133817	3.96087698	4.78687532	4.81806936	4.43573734
CD300C	1.48663291	2.13366044	3.24592792	2.38855616	-0.24231889	-0.19517072	0.63152635	-0.09742756	-0.41334195	-0.41334195	-0.41334195	2.53526814	1.28053888
CD300A	4.64909507	3.39164083	4.66268929	4.19704526	1.71795119	1.52281839	1.79586514	1.38132278	2.9035524	2.18723183	0.89618272	3.3347848	3.63890594
ATP5H	5.76953361	5.2277338	5.78555372	5.42378986	5.11925933	5.88280474	4.78097488	5.17630609	5.31379194	5.38972307	4.67861564	5.44930965	6.09523321
TMEM88	0.92350945	2.40396349	0.53018307	1.60427878	3.23191774	1.72628614	1.9048494	0.53338097	3.87679589	3.18986269	4.02180853	2.93033284	2.85966719
EVPL	-0.05079683	0.74808169	-0.04848962	-0.04160237	0.06230907	0.05463893	0.12921477	-0.04785337	-0.06853286	-0.06853286	-0.06853286	-0.06853286	-0.06853286
SRP68	6.99348088	5.91789641	6.2514516	6.20517473	5.97819405	6.00004334	6.36345969	6.0209596	6.01790918	5.98169524	5.88271711	5.92481123	6.54111597
MGAT5B	-0.04904852	-0.06263061	0.20560849	-0.0060955	0.76882131	0.1327812	0.70788814	0.26336643	0.07753069	0.07753069	0.07753069	0.07753069	0.07753069
TMC8	4.02510479	3.18347034	4.32671037	3.30238888	2.15318285	1.21198483	2.35640133	1.11026272	2.89790274	3.82378333	4.17065366	-1.01672756	3.43249525
TK1	1.92750469	0.83243395	4.64486841	4.51542052	0.82426745	0.53926797	1.60578337	0.76538431	0.03391873	2.53656075	3.55616974	2.6909165	1.82906731
TMEM68	3.35207451	3.43279066	3.09168873	3.98378511	4.02535308	4.20555069	4.41067889	3.8227918	3.7984423	3.83342281	3.38473771	4.22620032	3.24718823
CYP7A1	0.3666596	0.30437336	0.30696419	0.39657724	0.41537131	0.30581383	0.30437336	0.75258082	2.34558016	-0.1649407	-0.1649407	-0.1649407	-0.1649407
GSDMA	-0.00608284	2.21001331	0.57993953	1.77803397	-0.00608284	1.15379772	0.19801327	0.43298403	1.82398302	-0.19848241	-0.19848241	3.11418766	-0.19848241
TMEM99	2.82130319	2.91071993	3.51651077	2.16174602	2.77041464	3.52072021	2.98097662	2.48997655	2.46838878	3.6581209	3.45963684	4.10829552	2.0447471
GHDC	4.96617683	4.33829167	4.76179181	3.59618323	3.64237681	4.04501058	3.1155903	3.87719912	3.77156301	4.85504577	4.53679713	4.89023181	3.99538435
SOST	5.00130084	3.05456082	4.40381574	2.78125508	7.13194848	7.45233129	5.97443058	4.18716578	6.80256536	7.23329389	3.49322415	4.9644575	4.63873981
ZNF598	4.54191923	4.60643882	4.90282468	3.75984361	3.82548019	3.65046079	3.8888004	3.55192883	3.5275966	3.75360944	4.01458379	4.45484065	3.10167743
RAB26	0.52649285	0.26159388	0.43438231	0.19136503	-0.19222084	0.11342862	0.2773935	-0.18901147	0.01673682	0.01673682	0.01673682	0.01673682	0.01673682
MLST8	4.1952593	3.76338225	4.86006638	3.33758778	3.45629845	3.38128605	3.39933353	3.69205043	4.20047546	4.71473662	4.24082973	4.60708331	2.4639047
E4F1	4.44169032	3.76157111	5.02366273	3.49831794	3.94499911	3.74063238	4.32030989	4.23237451	4.22899989	3.79671338	4.47089042	5.09772758	4.11061184
DNASE1L2	0.22501167	-0.25972352	0.64087618	-0.20175563	-0.21733645	0.69167182	0.90643997	0.5293806	0.00263087	0.00263087	3.26322153	0.00263087	0.00263087
ECI1	4.57686473	3.7872933	4.8047204	3.02224983	3.5288365	3.95661622	4.16016511	3.84891395	3.5846738	5.28206788	3.97010018	4.34212261	3.98458312
CASKIN1	0.10272823	0.23575056	0.21154102	0.04262864	0.15277006	0.04005435	0.03860884	0.40491506	-0.46980122	-0.46980122	-0.46980122	4.0010938	3.37142717
ABCA3	4.92962627	5.2027037	5.02416795	3.4049504	5.02937036	4.99386024	5.26553203	5.7100533	5.20758425	5.86093673	5.5474534	5.92645677	5.0823887
KCTD5	4.33582061	3.88813259	4.84373928	4.83922093	4.04876824	3.89401444	3.67989616	3.63359374	3.88716184	4.49554334	4.24033665	4.23654518	4.59859144
SRRM2	8.4905769	7.82722255	8.37124553	7.7838426	8.3785702	7.60088673	8.41423544	8.47825703	7.97139798	8.29163228	8.39317764	7.9438547	8.54872688
ZNF597	3.58920345	3.81009023	3.18907692	4.14165723	4.64770236	4.60443485	3.82731695	4.06417887	3.82396711	3.86501014	3.66703135	3.84124256	4.31114866
NLRC3	3.03182993	3.10986263	1.80295456	2.03665372	2.22167997	1.57317771	2.09791525	1.69764638	2.72060283	2.21711621	3.93535311	-0.98878563	3.8832214
SDHAF2	4.69090919	4.34908666	4.89674476	4.55241698	4.82329274	4.13277746	5.30436307	4.86392852	4.58827971	3.82884412	4.57494662	4.05396266	5.36133036
DDB1	7.58463997	7.23429132	7.2301154	7.22581464	7.00090584	7.08914673	6.68872476	6.63888984	6.63721936	7.53721297	7.00215245	7.24976054	7.64519948
VPS37C	5.07902458	5.04637504	5.37049499	4.00988272	4.84641446	4.18252818	4.51270479	4.63612383	4.23945338	4.05644601	5.0700398	4.48232175	4.40819137
VWCE	2.13670289	0.53578878	2.54460826	1.69426643	1.69212695	0.66113206	1.81146428	1.72787551	3.47667061	3.02203377	1.96331921	2.83272728	3.27395021
RAB3IL1	4.84364573	4.33069855	5.10729477	3.73200568	4.13726105	3.08038106	4.16764179	3.70230398	4.34793961	4.444858	3.9778719	3.0031283	3.00774208
BEST1	4.21616107	3.11626638	3.96392377	3.6369189	2.74837075	2.38939843	2.52726064	2.52533976	3.21982896	2.07853676	2.12119599	1.73961422	2.58455541
FTH1	10.3871958	8.66643528	10.8308791	10.1443134	8.43366139	8.20987127	8.78645332	8.23626275	9.21209451	8.72506419	8.31104141	7.66359862	8.995237
BSCL2	2.04625546	0.02964223	1.87527784	1.24796415	0.81425035	0.59190019	1.14203724	-0.72309704	0.96147279	3.3645502	-0.10174663	2.6341447	-0.10174663
POLR2G	4.61832524	4.90893794	4.74238326	4.96303147	4.58375686	4.4841695	4.57921116	4.02848593	4.67020883	4.86623999	4.72960333	4.80783937	3.84847518
SLC3A2	6.06930577	5.53887797	6.64879062	5.70522721	4.59081967	5.16321882	4.8791578	5.12249676	4.63367345	5.01061688	4.96655497	5.03684102	4.68930792
HRASLS5	1.62682908	4.24589898	1.61231622	1.83430765	1.58329959	2.11250035	1.5229736	1.43392372	3.4955287	2.44469427	2.2059983	2.90757671	2.6382278
C11orf84	4.08926122	4.00595352	5.29229895	3.95552468	3.66269071	3.5947118	3.90694548	4.10474643	3.94974119	4.83480672	3.13932331	4.46790406	3.93181635
ATG16L2	4.99942056	3.99954223	4.63853187	4.37515559	4.49408951	3.33275558	4.76369095	4.0833465	3.83105955	5.21631217	4.6882305	4.51018202	4.03085643
C2CD3	5.49740262	5.30546784	5.31181238	5.6252016	5.54372158	5.193795	5.47100888	4.87028929	5.22514893	4.44954913	5.71941519	5.2420611	5.68516702
TRANK1	6.82629672	6.52853205	6.11441654	5.57985727	5.44364994	5.65632584	6.73542645	5.79914878	6.15341629	5.50487014	6.51359763	5.75431329	6.57480614
TTC21A	3.55396444	3.27181301	3.65864826	3.23104311	3.17576595	2.50849925	3.52778584	3.67693772	2.69827842	2.684079	3.72129099	3.61006495	3.92865456
RPSA	6.67080637	7.07499669	6.92652571	7.66566871	6.38122205	5.98441914	7.08754347	6.51282353	6.54662858	7.06077428	6.2599884	6.69406652	6.59346681
ENTPD3	0.35148883	0.59741062	0.49511855	1.05336571	1.08877198	1.52042944	0.66898103	0.48165552	2.04705404	-0.21808064	2.68959258	2.71904894	-0.21808064
CTNNB1	7.53869774	7.80259705	7.26836576	8.00086866	7.34634382	7.26328786	7.64845909	7.41095433	7.44206006	7.62805443	7.78527935	7.56302861	7.45024231

ULK4	3.83714787	4.31077841	3.24653987	4.07318364	4.52738222	3.68511315	3.46382502	4.102642	3.54025907	3.94424931	4.43835899	3.76488162	4.1557378
FADD	4.74251288	3.73663165	5.34273407	4.25571868	4.09162196	3.75064124	4.45975145	3.7668981	3.95275083	3.56311862	4.56388599	4.36915304	3.22695923
LTBP3	8.08612906	7.41564186	8.40359691	6.40993157	7.51569458	7.22708103	7.50025612	7.91831959	7.00793342	8.33806937	7.64060146	8.24372204	7.14564718
NAALADL1	4.42540806	3.8898783	3.82124019	2.43288857	3.04343192	3.5271791	3.06296108	3.50549759	3.11974989	3.74772857	4.42439291	4.21219642	2.57533357
SAC3D1	2.77410486	1.7290463	3.96743801	1.30035292	2.27101991	1.99546123	2.30219316	1.78267607	2.93574823	3.89569915	2.3751772	2.45126473	-0.82962457
BATF2	3.42233082	2.1146368	2.5803159	1.29286817	2.08991836	2.14399669	1.35460571	0.61365275	2.29520586	2.39699375	0.1890166	0.95560183	1.8622077
SF1	6.88284697	6.74636937	6.63092909	6.34026654	6.8906568	6.1247528	6.76444314	6.82834707	6.47719375	7.09001812	6.4804716	6.73573467	6.7867557
MAP4K2	4.09094142	3.49182732	4.11215635	2.54626442	3.0772364	3.29879711	3.37777714	3.45952938	3.3091931	3.55478475	1.24331641	4.3955083	3.27665425
CCDC88B	1.89742417	1.22168372	2.3077886	1.06274589	0.29856864	0.16927446	0.42637134	0.1644116	-0.12862999	-0.12862999	-0.12862999	3.11571934	-0.12862999
SCARA3	8.48328047	6.63521914	7.44777476	6.23794386	6.34059176	6.33307906	6.9256829	6.86153214	7.57230396	7.01524558	7.86319113	6.90901055	6.85526842
PBK	0.05231491	-0.21064944	2.39359832	3.22246399	-0.26886723	-0.26770994	0.73884628	-0.26621172	3.20780646	-0.55229694	-0.55229694	-0.55229694	-0.55229694
SCARA5	1.0279056	6.67566084	0.58956869	0.8440741	5.89940133	3.13415006	5.49722075	5.14310836	5.47873356	6.43331142	4.45066912	6.32828639	6.50951944
PNOC	0.22723462	0.02766878	0.03019001	0.59184537	0.15319269	0.26622685	0.02766878	0.03088529	0.01936524	0.01936524	0.01936524	0.01936524	0.01936524
COPS6	6.54484295	6.37202194	6.32044119	6.05683332	5.99648018	5.99797801	6.18547888	5.96911866	6.4316809	6.45306062	5.92492238	6.39404212	6.15208784
PAFAH1B2	6.3793901	6.7951315	6.20472396	7.12448232	6.87320879	6.5464592	6.90330303	6.80752003	6.55769908	6.21378478	6.72857529	6.41020797	6.37655755
ANKS3	4.62839129	4.26688403	4.91084058	3.97619215	4.4595375	3.69130127	4.52170369	4.93358693	4.36999073	4.74167845	5.13642829	4.94151751	4.6298092
NUDT16L1	3.12164453	2.30145419	3.74476638	1.7001948	1.96899227	2.31430969	2.41911627	2.18070845	3.29673234	4.05876032	4.1995968	4.07368514	2.8144187
KIAA1586	4.43170535	4.52480058	4.29398504	5.42961542	5.40248722	5.58483811	5.4443669	5.65838089	5.67914511	4.75461227	4.94426665	5.28268	4.32108538
RAB4A	5.19971196	5.17446779	4.58363478	4.66702394	5.38538541	5.6587581	5.44479689	5.52388815	5.45786941	5.68884528	5.30643457	5.47223826	5.62896491
SETD5	7.2345791	7.18700705	6.47025733	7.43416576	7.52289723	6.86888319	7.60432055	7.46834429	6.7642769	6.79267497	7.21244048	6.98389346	7.33614293
VASN	5.35040329	5.2675355	6.22735332	3.86954196	4.97929518	6.07081422	3.8470111	5.9082757	4.76653178	6.50027354	5.6135688	6.40913399	5.23732185
FAM83B	0.31971794	0.31971794	0.32231517	0.62996745	0.31971794	0.32116196	0.31971794	0.5594585	1.48840901	-0.22144164	-0.22144164	-0.22144164	4.81137104
THAP9	3.18064456	3.4221901	3.19366657	3.77656999	3.98959834	4.49820697	3.96155891	4.39264054	3.95469214	4.34846903	4.16076331	3.76797087	4.01608424
OR2C1	0.07251637	-0.20770331	-0.00857854	0.12543423	0.39045843	0.17932946	-0.09970094	0.57976939	-0.01076525	-0.01076525	-0.01076525	-0.01076525	-0.01076525
RNF187	5.58708986	5.2661402	5.89696638	4.98810903	4.79203007	5.23022126	4.93351961	5.05480161	5.21557751	5.55649359	5.49781884	5.39185492	5.61377325
HOOK3	6.25135296	6.71967687	6.49814335	7.15034898	7.25239414	7.51195181	7.29117817	7.4257939	7.01687005	6.81649576	6.94922962	7.10785413	6.55846926
MAPK1IP1L	6.42435396	5.971137	6.55275765	7.2229355	7.17910068	6.69042874	6.87838411	6.74818351	6.49092219	5.89527728	6.45050567	6.5540293	6.14336385
DDIT4	5.09847184	6.14025017	6.54028836	7.37240432	5.04880521	5.38275296	3.98037929	4.9560784	4.35786565	2.68488537	3.86273241	4.52053552	4.5753629
RBPJ	6.29649453	6.8015409	5.85838619	7.2686896	6.86207069	6.89441498	6.78960235	6.79652379	6.85232064	5.93579023	6.419662	6.5835976	6.68630989
LMBRD1	4.42017452	4.8857489	4.42734738	5.20281473	5.66814219	5.84768811	5.33418003	5.72328971	5.65861314	4.93717167	4.90538643	5.51682187	4.61050722
ZCCHC4	4.10076447	4.89701084	4.04966594	4.6205557	5.03795844	4.92337867	4.70112223	4.80801006	4.55652881	4.60040607	4.46585538	5.06685982	4.18072256
PTGDR	1.03364893	2.50271183	0.68245522	2.28118854	1.30711481	1.35614491	1.9190932	0.72441642	2.74136017	-0.50473943	-0.50473943	3.00964095	4.82684257
TTC39C	0.32159454	0.70109905	0.47322385	0.89968035	0.4917338	0.05781409	0.43161375	0.68669075	0.43357951	0.43357951	0.43357951	0.43357951	0.43357951
GLYCTK	3.9751793	3.06053145	3.74131099	4.01632573	2.3273571	1.31577184	3.10225703	3.38519508	0.03567241	-1.01611624	3.98392014	3.57214592	3.62885698
GNG4	0.01389063	0.11474547	0.09013554	0.00217	0.0200415	0.04429785	0.06237785	0.00158797	0	0	0	0	0
UBTD2	4.4004513	5.04925267	4.63874114	5.60104498	5.31670898	5.2639453	5.52781489	5.42877858	5.37096576	3.89465343	5.53741463	5.22241227	3.9283357
NKIRAS2	5.20754388	4.36677705	5.41013641	4.70982088	4.80430426	4.68017258	4.54144514	4.97070397	5.36900021	5.14270852	4.78256378	4.82739603	4.98080744
DNAJC7	5.55259762	5.63919672	6.09626041	6.21358423	5.98979064	4.94717749	5.88474083	6.07522552	6.08609355	5.52114255	5.65093468	5.33650504	5.94170985
IRF2BP2	6.86784632	7.14066748	6.46760872	6.67369786	7.01445641	7.78064741	7.55158943	6.8052934	6.99148055	7.05539826	7.0053618	7.0548693	6.77001125
NT5DC2	4.71050308	4.12898692	5.30159414	6.06687384	4.16898137	3.48335904	4.53355887	4.40905187	4.76629302	4.77616079	4.47692168	4.85790804	3.97201325
KIF5C	0.78062474	-0.55077703	-0.1951972	-0.16788251	0.38418608	1.64253474	1.03997276	1.61561352	0.19476327	0.19476327	0.19476327	0.19476327	0.19476327
MGAT2	5.10885658	5.28723095	5.82330242	6.70146937	4.76773286	5.09583435	4.97235638	4.80815913	5.42145666	3.4444597	5.51665258	5.72387309	4.14115377
BMI1	5.30403247	5.98221586	5.46938066	6.1105774	6.32623103	6.53231427	6.87315116	6.4455613	6.53629516	5.51306571	6.53629516	6.18171046	5.73964998
THAP11	4.57981527	5.36734144	4.98561796	3.66963571	4.13793788	4.44492816	4.77946936	4.40430825	3.95297208	5.03591166	4.31526328	5.34878679	4.69518759
MMADHC	4.80948365	5.70870636	5.51129724	6.06905278	5.88555122	5.89356228	6.20455908	6.1739164	5.94243615	4.74048994	5.67255361	6.0811252	5.09335819
PDHB	4.94939099	5.76513605	4.62263585	5.34873976	5.48998788	5.23424244	5.27069581	5.27247234	5.48564685	5.01476914	5.32725682	5.677184	5.82505133
PXK	6.01645194	5.0074563	4.76792324	5.27087592	5.24648778	4.90940959	5.21080936	5.19655031	5.08893957	4.3111775	5.6299992	4.68381833	5.18740102

HIST1H1E	5.51445604	5.77413232	5.83278272	5.61085436	4.72563595	6.32912017	4.29739998	4.89379494	5.53254731	6.94740049	4.89529263	5.90357243	5.3406528
PCMTD1	5.42067519	6.62234004	5.70095218	6.77846764	6.98055048	7.32885728	7.29434529	7.45259969	7.38649329	6.6889504	6.99234216	7.26469671	5.85340665
KCTD6	3.4054123	4.34609059	3.51178278	3.53846673	4.10376383	4.335299	4.43136098	4.37458481	4.161336	3.419484	4.60193962	4.22497035	4.55313583
ACOX2	4.02627316	4.02585238	3.30050608	2.34327859	3.25576066	3.1730594	3.07165888	2.6749477	3.99761831	4.20811247	4.34378667	4.31843222	3.38920962
FAM107A	4.03690823	4.29063186	2.85066487	2.02296515	5.27320401	4.77272519	5.32136664	4.29628182	4.90551714	4.97610058	5.34076397	4.82356275	6.78173723
IRF2	6.5971851	6.06864443	5.6909154	6.00637432	6.11558136	5.70803878	5.65713848	5.03723237	5.58177984	5.65214141	5.65075594	4.90419062	6.03312999
MOBP	0.63971541	0.63649365	0.58615161	0.51563986	0.48972723	0.45476456	0.48503288	0.45663844	-0.23590928	-0.23590928	2.31680875	-0.23590928	-0.23590928
CX3CR1	2.12755007	6.21960225	2.68170574	6.03267304	3.80292899	1.59266239	2.8859832	2.06076274	5.89446466	5.94221108	4.1175444	4.96823891	5.83985979
XIRP1	0.50576407	0.54184702	0.88335201	0.68537481	0.51988278	0.55242083	0.47074693	0.47406667	0.36195593	-0.10046243	-0.10046243	-0.10046243	7.97108234
DEGS2	0.11897115	0.13838238	0.01061377	0.07604349	0.21438929	0.2326344	0.00802047	0.01132891	-0.08864	-0.08864	-0.08864	-0.08864	-0.08864
SCN11A	0.83035803	0.81593129	0.84935633	1.50441279	1.10333358	0.89578565	1.05808097	4.26466773	3.61074484	-0.61167766	1.7146211	-0.61167766	2.83905314
ARF4	5.33065556	6.34414514	5.90442602	7.97644826	7.1222858	7.06461469	6.99285431	6.85718526	7.19462048	6.10163319	7.55716633	7.17286654	5.76921322
FILIP1L	6.85929826	6.86321194	6.5791554	6.22391773	9.08481583	9.04014832	7.75569699	8.94663149	8.79834012	8.69345687	7.28788328	8.75237125	8.67527212
MFSD2A	0.4389055	0.60340745	0.59555733	1.05523568	-0.38492497	-0.97695257	-0.16806832	-0.9910119	1.04353295	-0.07645704	-0.07645704	-0.07645704	-0.07645704
DTYMK	3.51688412	2.63455952	4.79292053	4.25557768	2.95400057	2.60332166	3.42549232	3.02604191	3.20830135	3.63221499	4.14108977	4.12059561	3.20830135
TAP1	5.0509613	5.4772693	4.87048433	5.4374495	4.8679853	3.86375494	4.88795979	4.37449082	4.51253398	4.31705828	5.43961935	4.38121939	4.68752109
ING5	5.20716046	4.94300659	5.28950024	4.24834495	4.94645322	4.4069013	5.34621476	5.32863963	5.1000991	5.20485338	4.78349117	4.696473	4.74681547
ATG4B	5.27255002	4.85032148	5.47343066	4.82519243	5.22791245	4.70455631	5.45321246	5.5971081	5.12912212	5.05796474	5.50069665	4.94996106	5.01554254
BDKRB2	0	0.92521423	0.05555544	0.49044557	0.53232825	0.07946101	0.4413917	0.26977666	0	0	0	0	0
MLKL	5.45906076	5.16704439	5.24385418	5.69316797	4.35733328	3.81220139	4.9666905	4.22582118	4.68136279	4.2605626	4.44811777	3.19830994	4.94323067
RFWD3	5.1889405	4.58046574	5.52740144	5.76250373	5.02459157	5.11478708	4.84297922	4.6990867	4.63614779	4.54387961	4.81531103	5.06838581	5.20363734
MTNR1A	0.03836696	0.63580143	0.04086654	0.03836696	0.03836696	0.03975669	0.39290888	0.04155584	-0.00490099	-0.00490099	-0.00490099	-0.00490099	-0.00490099
RHOH	2.75102301	3.11073962	1.40221297	2.69544832	1.97585341	1.43331658	2.21305063	1.81168594	2.5586753	-1.11158557	1.59842012	0.92768503	2.62316945
KLHL30	4.34896314	3.98243044	4.85611657	1.64005031	4.35710148	4.03830446	4.32879301	4.92753042	4.49672822	5.11145834	5.34679311	5.68589145	2.36002576
COG7	5.48144281	5.33074112	5.42793447	5.17937922	5.02509323	4.4889764	4.99332009	4.80958051	4.81626992	5.30727839	5.19465002	4.74146987	5.45730561
CDC40	5.04067043	5.23618519	5.20378311	5.85997803	5.53232738	5.76147544	5.86164475	5.82250736	5.79137031	5.53954802	5.53072792	4.98113955	5.85829127
STIP1	6.4710595	5.68122321	6.13041789	6.04814453	6.19591176	6.38643544	5.73472519	5.11153909	6.1254344	6.18294379	5.66947585	6.06398124	6.03845977
SCNN1B	0.46511023	0.26933897	0.27178663	0.3136457	1.77636382	1.29788876	0.67104119	1.75265609	-0.33917291	-0.33917291	2.86388392	-0.33917291	2.92607841
HR	3.08407746	1.24482247	1.23731657	1.08831896	1.05996863	2.02106927	2.20292987	1.68779918	0.06647777	2.5428602	3.80664057	3.6151627	-0.6567615
TXNDC2	1.35350848	0.60985408	1.16140951	0.64637162	0.86964091	1.76595766	1.09207677	2.17531413	1.98313046	-0.51625719	-0.51625719	0.50517238	-0.51625719
RAB31	3.64029211	4.62677176	3.45977689	5.34042103	5.17111695	4.68989752	4.8833137	5.11449639	3.85782788	3.70101129	4.67374127	4.64983672	4.68790607
REEP4	3.1050653	1.93463224	4.45890668	3.83237548	1.67448339	1.56358184	1.90909749	2.12296373	3.27616335	2.73819633	1.96302787	3.18448173	3.42596846
TNXB	5.76356669	7.39871552	5.80666478	4.39792179	5.88127503	5.14878334	6.45394762	6.31162469	5.92326137	7.14212961	6.77540207	6.58090832	6.6035447
LGI3	0.15732551	0.12523617	0.32292914	0.12523617	0.31914401	1.39856109	0.18399916	0.38870115	-0.20640825	-0.20640825	-0.20640825	-0.20640825	2.6213169
BMP1	5.4651253	4.81520388	5.7646577	6.41010562	4.81698019	4.03433994	5.00969958	5.38826254	4.28631787	5.5701199	5.55057495	4.83529248	4.38815108
ATXN2L	6.16451742	5.6833199	6.27311657	5.75920913	5.95522946	5.27122249	5.94116637	5.91202063	5.59224634	5.82085198	6.36304811	5.60985593	5.68779811
PHYHIP	5.35808131	5.24856268	4.80365263	1.88165705	2.14690945	1.98613039	2.84776151	1.75567653	2.15422131	-1.40648853	2.83839841	2.50922253	2.74986935
CCDC110	2.58020513	3.46768386	1.98120141	3.59463363	3.99995967	5.41162746	3.29933325	3.2348011	4.98570809	-1.63797685	0.12284477	4.12755479	3.49417329
POLR3D	5.0493853	4.88573989	5.08745405	5.39681632	5.05830001	4.44823477	4.34337847	4.79543884	4.83679895	4.92684131	4.38877328	4.85815842	5.01599647
FEN1	3.55024006	2.86056294	4.72036331	4.52242255	2.40173493	2.87054601	2.76619198	2.65519382	3.49059017	3.3990169	2.19832494	3.33955017	3.26467845
HEXIM2	2.83544269	1.22683861	3.92424437	1.59029159	1.45011881	2.71648446	1.82974412	1.75161579	3.42391121	3.36760718	1.53044593	2.84501862	2.72755071
FNTA	4.93700716	5.607524	5.05586185	5.82515201	5.68307993	5.6506053	5.5440002	5.67643729	6.02354682	4.75540389	5.11864467	5.57929833	5.18434446
SERINC2	2.00244251	3.1273967	4.92495337	1.04777562	3.06701586	1.28255435	2.64052817	3.23795344	-0.46101626	3.3702152	2.41080024	3.63781218	2.56105036
COL3A1	5.98595937	6.51478184	7.76118985	9.30767968	6.42692583	6.25804092	6.77152183	8.06082682	5.99992359	6.81361418	6.56362405	7.82006646	6.430062
GFRA2	3.70597012	2.38367294	1.98674261	1.61442205	2.10153735	1.59616303	2.96074625	2.6197256	1.10571503	3.49519534	3.67661258	4.01138113	-0.0025896
ING2	4.35881251	2.30990887	3.39124942	3.60099363	3.42709324	4.75036678	3.30636067	3.62625397	4.6392539	3.92343408	4.31134535	3.55759191	3.10023704
CDKN2AIP	4.99015754	5.28825661	4.28667588	4.64573285	4.82360311	5.47780231	5.10625038	5.11105574	5.21230443	5.42857561	4.383596	4.81747409	4.87968768

SNRNP48	4.56511722	4.49288815	4.54011083	5.35602988	5.57410313	5.43826182	5.89100488	5.59532708	5.23103701	4.36782875	4.93840037	5.32766441	4.25246803
TMEM223	4.51985303	2.84537427	3.89094584	3.23449575	3.45672931	3.30335086	3.37544272	3.50051664	4.00232983	1.63158834	2.85613335	4.49341706	4.17534917
SLC20A2	5.7986037	5.94976863	5.58512957	5.25897286	6.31633744	6.63009857	6.05992529	6.1663665	5.29107265	6.0042656	5.50249513	6.08257877	6.43871852
DYNLRB2	1.10033954	0.80569624	0.20757422	0.44454807	0.38118754	1.34562971	0.52398339	0.45543446	2.18665075	-0.3075824	-0.3075824	-0.3075824	-0.3075824
TMUB2	5.33758398	4.54799095	5.32623441	4.87438319	4.76635661	4.66771818	5.06810053	4.62219635	4.83369707	5.32938143	5.05565754	4.78540428	4.33357554
STAT3	8.3770492	7.86025657	7.8094756	7.38753115	7.54862892	7.16961099	7.88327452	7.17452815	6.91539563	7.32394534	7.14167772	7.28255587	7.35300076
ZSWIM1	4.99470459	3.38953661	4.49434976	4.09276468	4.01022234	4.2942718	3.63495621	3.71529647	3.89197749	4.6703597	4.31078918	2.52310417	3.60510999
ADAM9	6.89416127	7.18027659	7.24767902	8.22320602	7.03002715	7.24973062	7.39165108	6.93365499	7.14145813	7.29086383	6.99368718	7.3063848	7.27629138
GDNF	0.71619014	0.69801252	0.80837348	1.09802458	0.72131135	0.69945707	0.73761852	0.95967923	2.34229667	2.33808595	1.86115099	-0.32660093	2.09728338
AXIN2	5.03098761	4.57704343	5.52901775	3.42306432	4.87927003	5.26621141	4.83497704	5.87170142	3.67270708	4.80338899	3.76102788	4.93360283	4.51224696
NDUFS5	5.90532367	4.25711241	5.50130072	5.14554344	4.78328634	6.31837929	4.49855215	4.64916675	6.02736183	5.62079942	4.83598536	4.77892686	6.13968915
VWA3B	0.337118	0.14268846	0.2769087	0.18700529	1.05849175	0.81476868	0.41289695	0.79213137	-0.10965542	-0.10965542	-0.10965542	-0.10965542	-0.10965542
ZNF30	2.9261833	3.88769182	2.82457629	2.71169108	2.8525929	4.07249632	3.55877576	3.37557301	3.33523498	3.72766959	3.84888077	2.9649203	3.28134364
FAM84B	3.42514418	5.21932263	3.37145061	3.22953999	3.83816732	3.71495307	4.22092545	3.48160383	4.7127463	3.31327074	4.75242427	4.84970729	4.92062929
KCTD19	-0.18256253	0.37660467	-0.0427961	-0.18256253	-0.18256253	-0.09330608	-0.18256253	0.35718101	-0.14411057	-0.14411057	1.97348723	-0.14411057	-0.14411057
SLC16A4	5.22291052	5.11745667	5.25463769	5.50570355	5.86805343	6.30046994	6.13778673	6.01861619	5.76286576	5.8086136	5.71598981	5.86387906	4.76950388
IL7R	4.35257532	3.62724234	2.78421191	5.12541311	3.25130693	3.07875378	2.61161215	2.86603421	4.15749677	-2.09099463	4.55753865	1.65993264	3.94518035
TMEM208	3.83537342	3.17069851	4.3848733	3.65629375	3.88850054	3.493037	3.03607932	3.27825139	3.81266271	3.72774129	3.13923707	3.97587259	2.26244342
LRP1B	1.31232177	1.2586727	1.15146255	1.19662475	1.51162949	1.28358874	1.41572911	2.23595161	3.94168043	3.44669705	2.53592468	2.99222477	2.82323223
AHCYL1	6.94461015	6.99871032	6.47818644	6.8422849	7.07086238	7.01353602	6.97122597	6.90059039	6.72584669	6.90505428	6.74386392	6.79346809	7.06822371
DNAJC21	5.33203395	5.50446057	5.77471677	5.49581255	5.93181705	6.23198865	6.18173858	5.94320563	6.18085857	6.20820803	5.83547017	5.63221577	5.49459846
PKIG	6.46153849	6.74741572	6.670698	5.88645002	5.96390907	6.40062964	5.79172357	5.42615668	6.10631371	6.20015964	6.44931128	6.06268341	6.31966907
NPNT	7.26677182	7.66748076	7.1033638	6.54233121	8.41566009	8.54157469	8.28110636	8.44716241	7.52729349	7.98005874	7.69570434	8.05374348	7.97036495
FAM178B	2.56493397	1.95439746	1.55564046	0.54670689	0.98786719	1.59887132	1.13949795	2.22144494	2.60499322	3.82351262	-0.62052542	0.89780404	-0.62052542
SEMA4C	6.02401835	5.82645155	5.75130769	4.62288132	5.13123549	5.06821127	5.2569561	5.32062427	5.14149747	5.83341682	5.74877954	5.54450002	5.53018633
CNNM3	5.12782358	4.78722901	5.00759161	3.59786857	4.44601069	4.04181863	4.69751436	5.17135232	3.64769348	4.35018481	5.43436435	4.75687166	4.69169489
GSTM4	4.67491075	4.61282041	4.20347475	3.10278145	3.81604426	4.79006253	4.29769145	3.57532047	5.01342321	5.22094714	3.685368	3.94156751	4.634793
TET2	6.24982073	6.01364111	5.62501093	6.75215167	6.30150882	6.47906488	6.03082774	6.60482774	6.42745168	6.49609927	6.20810368	6.54475199	6.53880491
CXXC4	3.70772398	3.01737051	3.37697301	2.90756825	3.72362193	5.45100678	3.91778817	3.97664101	5.07689273	4.72727586	4.06948095	4.68410791	3.7042882
TCTN2	5.55572504	4.35630068	4.8027878	4.36237793	4.70617572	4.33898003	4.71456988	3.94036072	3.82219409	4.9030696	4.9895832	4.09716831	4.353783
SHOX2	4.86616527	3.17347065	4.7442372	2.99597862	2.7836518	3.42682539	2.33774947	1.98141296	4.14447385	4.34994161	4.25003304	1.7414082	3.49750854
PPIP5K1	4.54320981	4.47317635	4.2502274	3.64691883	4.1415114	3.88465737	3.83411079	3.82713137	3.98879285	3.73239786	3.38675929	4.05410938	4.63960913
TSPAN5	4.59221214	3.91703113	4.62102578	5.09411588	4.05140634	3.80106921	3.82190366	3.63503672	3.17925024	4.23548585	4.81753286	3.9545723	3.93238519
ABHD15	4.08254755	4.79528634	4.44394308	4.16143358	3.37586414	3.50053956	4.23653322	4.01448837	4.44982337	4.2068067	3.83934637	4.34550184	4.01896737
ZBTB5	5.8591347	5.40773771	5.12863505	4.90409542	5.2630448	5.71336335	5.45140515	5.54043063	4.9277738	5.33401993	5.73613171	5.70994109	4.82732437
CHTF8	5.65930753	5.97741743	5.38938021	5.32297428	5.12168817	5.36857536	5.2017508	4.87883734	5.47524395	6.14760694	5.5477629	5.57409178	5.50878107
ADAL	3.88850771	4.42546232	3.97022015	3.73517698	3.94547139	5.00099926	3.60656178	4.24252444	4.53260672	4.00242712	4.13846081	4.42184431	5.25571177
LCMT2	4.15806171	4.11430512	4.40432579	3.84593485	4.28517791	4.87616301	3.96930213	4.69607086	3.24027664	4.30885726	4.25616668	4.82570012	4.36520581
SNTB2	4.04304258	4.51662944	4.01358022	4.81524274	4.87837602	4.51995691	5.19211777	4.91667849	4.55762083	4.85714207	4.57137166	4.8032033	4.93237913
IL12A	0.242233	0.19948608	0.66276972	0.20630236	0.03934887	0.04073347	0.03934887	0.48135857	-0.08985286	-0.08985286	-0.08985286	-0.08985286	-0.08985286
ZNF507	5.9647675	6.11601252	5.54866914	5.86182487	6.4612095	6.57299214	6.33477262	6.55336855	6.15289711	6.4333743	6.63473067	6.23097216	6.72389838
STX18	5.36034218	4.49333308	5.04437096	5.21694225	4.95633957	4.70296054	4.54979188	5.27387467	5.08193455	4.78809723	4.87506616	4.52601973	5.72848072
ZBTB49	4.0602973	4.1606183	3.19936117	3.1111493	4.4638985	4.04969696	3.65580077	3.63980186	3.75146711	4.19659391	4.03261809	3.25646348	4.06896936
GFM1	4.98398163	5.34168333	4.87113468	6.20648536	5.65280776	5.81368892	5.23199515	5.26239029	5.93673126	5.56370489	5.33517458	5.22755567	5.83633335
DDX19A	5.28877618	4.68609292	5.32461443	4.85797685	5.08239867	4.56201471	5.31380069	4.89969681	4.64870381	5.45501056	4.56047162	4.87977265	4.76955384
ATOH8	5.226806	4.8237472	4.66704349	2.44110123	5.27761808	4.98136538	5.89203119	4.8315013	4.70332331	5.58332837	4.24924221	5.11192141	4.16198233
ANKRD49	4.35704028	4.18127696	4.21932911	4.90559448	4.95359782	5.37539283	4.94473938	5.10259655	5.45580575	4.02161777	4.83071777	5.02773338	4.34412056

USP39	2.66298615	2.49701101	1.91783534	1.85119351	2.32066059	2.76360175	2.33107223	1.91276694	0.07965197	3.48782047	1.57642676	3.30005755	2.58409755
TNIP2	5.98461362	5.28810521	5.95787839	4.86456259	4.72770404	4.27181028	4.4388354	4.44500026	4.96352912	4.93424858	4.39492737	4.61166256	4.06379557
C2orf68	5.56909287	5.27230104	4.86282113	5.2284469	5.58992896	5.23812558	5.2751202	5.9069388	5.40784584	5.74018618	5.42994199	5.70935425	5.85103264
TMEM150A	4.44435476	3.88289883	4.51309829	3.13357384	3.63557045	3.34126913	3.15129405	3.58123013	3.457003	4.05356138	4.2949984	4.2161588	2.69271374
RNF181	5.52361352	5.11191795	5.99090172	5.55697085	4.74570569	6.06099615	4.84060938	5.20429497	5.26842713	5.86714307	4.83189086	4.92974054	5.04324462
VAMP5	4.8092737	4.48288239	4.86116421	5.0266549	3.42473412	4.99423058	3.840664	4.31362816	3.69046265	5.64456136	4.77267187	4.13202948	4.18287026
LRRC28	2.95284904	2.49057862	1.92416143	2.54220945	3.07967858	2.40755705	3.25282864	2.88501868	3.87998777	-0.72934198	3.11789735	2.7135404	2.58012498
MAT2A	6.19752469	7.41136712	6.12104094	7.68747302	7.65757472	7.16382656	7.53705031	7.1855416	6.68896647	7.2687789	7.12105816	7.32798184	7.35626368
ENHO	0.18809602	0.09134005	0.49473232	0.22426988	0	0.25096991	0	0.1988613	0	0	0	0	0
ZNF608	6.47421671	5.46635859	5.67866759	5.08421587	4.54572096	5.63402114	4.59913545	4.71908799	5.13789133	5.02478329	5.1424366	3.76436922	5.60110472
INPP5D	4.94886345	5.21160916	4.68852056	5.27620692	4.35439757	3.15958429	3.99222399	3.57316878	4.98230539	3.29501058	4.54005269	4.06894186	5.38213966
LETM1	5.39686039	5.2741938	5.97693484	5.41697245	4.99040343	4.68630329	5.22071629	4.82363528	4.75708702	5.10013989	5.23705194	5.25924014	5.31785605
CTRB2	0.20818919	0.03786665	0.28477292	0.03786665	0.03786665	0.80428973	0.03786665	0.04107176	-0.1123352	-0.1123352	-0.1123352	2.89438035	-0.1123352
TMEM129	4.30477569	4.94335858	5.01520633	3.52365679	4.07783571	3.75811009	4.02952747	3.75574751	4.07899065	4.95838967	4.24385901	5.18421392	3.76076888
PPIC	5.56508743	5.30371235	6.23183072	6.48994829	5.11229929	5.19584274	5.77025165	5.76647782	6.05284764	6.29796176	5.8430761	6.5001877	5.70591596
SPRY3	1.16041296	0.60761029	0.48328245	1.01798936	1.33802193	1.95035244	1.30824411	2.03148381	0.44728257	3.21259268	1.46164782	1.82339108	0.87425693
CEP120	5.22632887	5.6196208	4.72537664	5.54966038	5.79169841	5.96155984	5.68019874	5.9524905	6.41847863	5.74599443	5.96354216	5.20744351	5.10243566
STXBP6	1.30025942	3.20200169	1.61434414	2.47327766	3.08986246	2.02693758	1.90904198	1.42388228	2.74275512	3.36319979	3.04533957	-0.98040335	3.63654936
MFF	4.95786297	5.34724433	4.72839889	5.60338342	5.66249561	5.99350134	5.72003647	5.77497149	5.38809888	5.64154557	5.57061252	5.57559388	6.04057731
GRM5	1.55129462	1.91916008	1.19650836	1.15605698	1.53630388	3.00616812	1.80931444	2.34297921	2.5951988	-0.69479507	4.16820217	2.58362334	1.98097443
LGALS9	4.9262356	4.51802064	5.50445273	5.03126867	3.60125892	3.10853056	3.48993508	3.29872326	4.20898078	3.42044581	4.5792221	3.10883317	4.26792263
JMJD7-PLA2	3.1716046	2.41616348	3.23873915	1.85416296	2.41367051	2.00649297	2.95145855	2.48598424	2.21270549	3.33144877	0.96151905	2.45637245	3.33144877
CPLX1	-0.28865039	1.52749619	0.36514503	0.40594312	1.13266485	0.17053816	0.37171542	0.30418441	0.30351012	0.30351012	0.30351012	0.30351012	0.30351012
SIGLEC7	3.2216607	0.23623535	3.50960396	3.16369722	0.10638111	0.33147825	0.37394656	-0.21531433	-0.41196388	2.99777534	-0.41196388	2.84368106	-0.41196388
E2F6	3.62677616	3.84082307	3.92291321	4.0374846	4.42180727	3.91859975	4.39618813	4.23383653	4.54613384	3.98305145	4.40613322	4.05530714	3.91622669
FEM1B	6.38583113	6.5176449	6.14856082	6.73121328	7.04163963	7.25405951	6.89595307	7.36547363	6.68882271	6.48763345	6.65680607	6.85508456	6.40684131
COMMD8	2.86117634	3.43616884	3.53747353	4.38374095	3.59411012	4.38798852	4.28334639	4.42442146	4.85949319	2.83519567	3.69091138	3.96814385	4.11233851
ATP5I	4.40503837	2.83327646	4.78613202	2.90202839	3.65361318	4.75204558	3.33520791	4.29293503	3.99529875	5.02488529	2.94386103	3.6509964	5.0224512
UQCRFS1	5.30505823	5.45042176	5.79297883	5.16788819	5.13901041	5.55096295	5.44894919	5.19338465	4.9400311	4.81335628	5.33857322	5.64816008	6.2857174
MFSD7	3.74114673	2.90804523	4.00036686	2.54758071	2.67888751	1.916674	3.28324494	3.27508027	1.54113597	2.97200726	2.99873895	3.62979153	2.47420363
COL4A3	6.06045477	4.92577178	5.40791464	4.71934411	5.61151036	5.74777799	6.18763676	7.02664692	5.74280898	5.80986918	6.23401424	5.81722677	5.93959633
MAP2K1	3.96382109	3.48151114	3.95559696	3.27573831	3.94169835	3.27510417	3.56544689	3.53823583	1.45546294	3.87189059	2.50519474	3.59861917	4.61027875
KLK7	0.06128469	1.63787384	0.06351562	0.44855739	0.06128469	0.06252506	0.06128469	0.06413084	-0.12977278	-0.12977278	0.9384503	1.50351858	-0.12977278
HNRNPH1	7.07047214	7.48655613	6.91776848	7.99747081	7.70339864	7.56142013	8.04001839	7.73290326	7.65049745	7.34775067	7.34371293	7.21226611	7.47156077
IRS1	6.18181365	4.92717523	5.81373965	4.9955222	5.3285897	5.73855201	5.38365815	5.69092216	5.15920774	5.58726005	5.57927506	5.4082373	5.31921966
MECP2	6.99445427	6.75871441	6.56694052	5.99633507	6.81633824	6.67320522	6.68515863	6.69969926	6.32653767	6.95402651	6.74140042	6.71963152	6.6032325
UPF3A	5.3873671	5.32852205	5.13756972	5.23118713	5.66408154	4.90708953	5.76115048	5.94426146	5.95290658	5.34147083	5.50208546	5.33609813	5.49485543
ROR2	1.15251484	3.5678748	-0.12028484	3.97780595	0.45871348	-0.20087074	1.19995342	1.63054562	3.12489269	3.0687919	4.44467237	3.53674839	4.36109348
AR	6.67497335	6.08703942	6.22133822	5.32596617	6.32614755	7.13710074	6.63127867	6.78540829	6.95128516	7.13979698	6.81890137	6.72613366	6.60104055
DHRSX	0.96733124	0.72020323	0.82013832	0.75022343	0.67577978	0.28145861	0.87214734	0.65298429	0.65307504	0.65307504	0.65307504	0.65307504	0.65307504
C8orf46	1.25467458	2.59005209	2.57911631	0.95089783	2.87865557	4.01191739	3.08519132	3.99960258	3.84785778	4.5798724	3.27944983	3.63649166	4.17721442
HSPBAP1	4.17132795	4.61053461	3.57231616	5.18161944	4.63271834	4.01415697	4.71930445	4.37532645	4.0651115	3.64830244	3.98773102	3.73841422	5.15543701
ASMTL	4.00030617	4.24249022	4.57897821	2.63576825	3.10771432	3.57720827	3.33679865	3.58506327	2.71144577	3.3125585	4.02448743	2.90869492	3.17558463
SLC25A6	2.27812203	2.15282862	2.08696189	1.60185557	1.23051236	0.45135758	0.83134041	1.16783659	1.47030888	3.52117909	0.99407354	0.99407354	0.99407354
CHST14	5.04009182	4.18620864	5.1468484	3.68887612	3.86956202	3.86720714	3.99004287	4.05346647	4.86137873	5.40231559	4.863569	5.05069779	3.78729625
PARM1	5.45094405	5.41197132	4.56076461	4.62155074	6.1756257	6.47231143	5.81806789	6.07676528	5.79354652	6.33877044	5.48650909	5.37274041	6.02054297
CSNK1G1	5.30676073	5.20079183	4.92268598	5.91834264	5.2703428	4.96305703	5.3025442	5.01890407	5.0042181	4.60087702	5.00183384	5.01460961	5.10618851

FAM110B	3.57509844	2.90109094	2.1992871	2.30386794	1.63597964	2.26333856	2.08258294	2.23202259	1.68464604	2.3804122	1.13436459	3.95161524	3.22698522
ARMC4	0.68831222	1.54477468	1.27142886	0.48973531	0.46087228	0.8675698	0.33857264	0.11506185	2.58528308	-0.42340476	-0.42340476	2.76577196	2.47382479
AFAP1L2	3.05220733	4.68416617	3.03554095	3.40650289	5.31083471	4.55278421	4.44291655	4.34286853	5.25774052	4.31012124	5.76590302	2.96280675	4.7177894
ZNF354A	3.65122259	4.12895417	4.16771578	4.61445942	4.97327509	5.25046253	4.38366467	4.9705108	4.90314565	4.04484104	3.82914121	4.74377693	3.32856502
ATF5	3.73797919	3.61702688	5.07870243	3.43742467	2.46667513	2.84486671	2.15954515	2.81322966	2.80357507	3.74590166	3.8881115	3.78754565	-1.2037529
UBE2V2	5.34344826	5.78322903	5.41471221	6.15826052	6.06212875	6.55847366	6.12869581	5.8510976	6.32663976	5.79587142	5.75995912	6.13855125	5.51466633
ZBTB43	5.19073807	5.47929822	5.17360304	4.99141244	5.43828074	5.28012672	4.91435867	5.34288062	4.89422274	4.88506726	5.03101171	4.80879048	5.4729373
CPT1C	4.49708348	1.71602471	2.61969145	2.95125558	2.13843562	1.56194797	2.91315846	3.43389785	2.95167655	-1.00814265	2.59351792	2.47491207	3.28641742
PCSK9	0.26727675	0.61616329	0.35357285	0.31511549	0.18388391	0.29136626	0.69790448	0.73091142	2.52111347	-0.05880466	2.73345709	3.63048301	-0.05880466
XPO6	6.23549001	6.3121616	6.37205234	6.60965334	6.11295781	5.19114833	6.22627778	5.9979576	5.76308178	5.66672559	6.58978303	5.86701508	6.17491212
GSG1L	5.89804882	4.41025969	5.58169129	3.52656306	6.77882247	6.62860695	5.44750131	5.50944019	6.37852164	6.51096569	6.57291888	6.72291771	6.1359841
MN1	5.63377711	5.4279374	5.52303682	3.66735885	5.52267647	6.731672	5.79930422	5.7970025	6.14745124	6.97857722	6.25510599	6.49963448	5.36097541
APEX2	4.75775065	3.8841716	4.64544097	4.2382825	3.80863125	3.78328833	3.65195936	3.58662839	3.79503248	4.71634973	3.5724694	3.77003187	3.47669879
NSMCE1	5.43173641	5.66071066	5.83146771	4.8633661	4.76327382	5.54786702	4.91454829	4.74970283	5.31879779	4.9831266	5.07722587	4.89207271	5.43274742
CCDC126	3.57767614	3.42639086	3.5157036	4.27054978	4.25807224	4.90115344	4.99439927	4.9894553	4.08020763	3.76730038	4.81729594	3.94614758	2.10052136
IL13	0.24906666	0.24409524	0.09570777	0	0.22576338	0.17884396	0	0.00158797	0	0	0	0	0
RAB3B	0.33624106	0.32673077	0.40999312	0.43104249	0.34755485	0.42792642	0.60603655	0.9175721	0.43668746	2.19591877	3.4738688	3.17943318	0.8054866
CD2BP2	6.04503805	6.07608434	6.55576433	5.35491651	5.53370458	5.61971233	5.6136921	5.46519519	5.6136682	6.22983195	5.73811133	5.80780817	5.32464832
RSPO1	1.92840136	4.74109005	1.90006188	0.94033938	2.05926947	1.17064435	0.84333151	1.10011077	3.48885272	-0.73826913	3.87197609	4.14666846	3.97817067
RGS14	4.16320066	3.20417345	4.37626396	2.49765837	1.96968777	2.33441718	2.75296666	1.66915993	3.6313272	3.04654246	2.28388742	2.49113202	0.84113595
TBC1D10B	5.09072587	4.84825473	5.6340129	4.95619695	4.73706556	3.8645514	4.95087628	4.89916088	4.51508827	5.38868962	4.33056217	5.34772233	4.9990602
LMAN2	6.10291752	5.66607409	6.52300038	5.95353777	5.10607495	5.57573044	5.47974921	5.00067117	5.90907666	6.34673801	5.93636532	5.46952501	5.50636381
RAB24	4.05399022	4.02199699	3.82862056	3.48495028	3.38487787	3.73312576	3.62385914	3.82548844	4.06120427	3.33898436	3.46621142	3.88785275	3.54977954
PRELID1	5.92305256	6.11672185	6.03783483	6.23036364	5.18000107	5.6641081	4.97114218	5.09290384	5.53027515	5.98476845	5.42999559	5.53694731	5.31392787
THBS3	5.81785651	5.45129011	5.91908901	5.4552015	5.30663459	4.42454484	5.73434608	5.57710093	5.52453587	5.69962246	6.13818174	5.90901004	6.14817031
CA5B	4.9782561	4.89932386	4.23099751	3.76176104	5.12319907	4.92040819	4.44970883	5.53380415	5.274836	5.23072318	5.73423219	5.15459818	5.04344905
SLC50A1	5.6085524	4.64296208	4.98439583	5.32042423	4.11003239	3.7610015	4.76128456	3.77736304	3.93945655	4.89858921	4.54522869	3.7993726	4.80924946
EFNA1	4.32864067	3.8704883	3.34101895	4.00800082	3.4491936	2.51460667	3.80889885	3.65477581	3.40542225	2.39790784	3.93184707	2.36873782	4.51544618
CXCL10	2.09058725	3.73418219	1.449077	5.74598095	2.53066158	1.843911	1.71961078	0.91935588	3.88255329	3.44205469	3.50059243	2.89370112	3.94769157
SH3TC2	1.81239882	2.60468089	2.32682691	2.16388725	2.49604361	1.93163572	1.95326727	2.30993401	1.46578979	1.66639332	2.03577928	1.62727738	1.7847299
CXCL11	0.37474211	0.73979524	0.91588572	1.27900394	0.37474211	0.37617094	0.37474211	0.3780207	4.62413754	-0.44045422	3.89790423	1.08039933	-0.44045422
ZRSR2	4.96182709	3.313207	4.88710012	4.10523295	4.21803752	4.30314392	4.2159996	4.43618637	5.12908077	4.78036345	4.85032565	4.1711525	3.89691804
NMD3	5.62867722	6.27734189	5.7950394	7.05914863	6.49010546	6.4475487	6.69318553	6.5253389	6.73274001	6.37912161	6.72606797	6.84117843	6.50765951
ADRB2	3.19077297	4.62387264	3.58937254	3.38447328	3.15374883	2.96357538	2.54733679	2.8153909	2.96321364	3.71283767	4.3142632	2.76114274	4.70893844
B3GALNT1	4.66447446	5.94114731	4.88296832	5.59127499	4.58055526	4.57692144	4.27158282	4.98665528	5.86990627	4.89818727	5.78050477	5.02108211	5.53786654
GPRIN1	0.47457283	0.1898663	1.04367578	1.26074701	0.21595731	0.95770374	0.68023489	0.0606897	-0.23225356	-0.23225356	-0.23225356	-0.23225356	3.22118043
HSPB3	1.74477087	2.04766266	1.2821601	0.54735492	1.20787662	2.48235133	0.54735492	0.84444903	3.00030653	-0.31992886	-0.31992886	3.13453719	6.90333234
KCNAB1	2.3207694	4.20781067	2.59204939	1.34307738	4.75134312	6.09160849	4.43104025	4.9087194	3.42665245	4.32002753	5.05462207	5.15761301	3.86435989
MRPL1	3.38308852	4.77715293	3.98853365	5.08436116	4.66167406	4.91519135	4.5492439	4.5853539	4.49509127	4.06632428	4.05924482	4.56656152	4.41331389
SHE	2.32701757	4.40181157	2.06995795	3.83001277	4.97069777	2.64220645	4.51318323	4.26691387	5.68655171	4.88223097	5.61612068	4.74498732	5.92509245
PGM2	5.37886223	5.50964955	5.06474297	6.21557089	4.93034483	5.4303559	5.34943531	5.30036889	5.39183141	5.09578502	5.43290974	4.58740381	5.64084539
STK32A	5.37442569	5.21391435	5.2890409	4.29833627	4.73415903	5.87102198	5.15027039	5.07867941	5.50772598	5.00144717	5.16051336	5.03856475	4.91581267
IL1RAPL1	0	0	0.05378513	0.02429514	0.01147455	0.09259121	0.04156584	0.27773961	0	0	0	0	0
P2RY12	0.07352738	2.78156158	1.13496083	0.85529316	2.08973744	2.34440539	2.30027049	2.51943327	4.02922727	2.76371252	-0.69826976	4.18751137	2.43172344
C22orf15	0.08158432	-0.03535573	1.25057333	0.18621289	0.31110768	0.37395582	0.59725073	0.20153876	-0.16233976	-0.16233976	-0.16233976	-0.16233976	-0.16233976
KIAA1024	2.9599748	2.51526428	3.04914561	4.14913163	3.11041839	3.09922514	3.19232024	3.73574775	2.87700934	3.38575256	3.86894561	2.14717084	3.79168716
SLC33A1	5.04260816	5.16132567	5.13107994	5.50987382	5.45976637	5.76959278	5.56475129	5.21475051	5.50923522	5.54956579	5.79882186	5.22348985	5.49469205

SNUPN	4.51252895	4.16108858	4.96240347	4.12618613	3.69443314	4.67042417	4.43950791	4.12542608	3.38797452	3.8355419	4.44020909	4.20733575	4.20569142
CRADD	4.00779354	2.90285703	3.56584149	2.45825278	3.43198121	3.52386372	4.19446694	2.69526407	2.89314444	4.16264445	3.06797899	3.66347255	3.69607136
SIN3A	6.85893745	6.0205551	6.21557803	5.78692257	6.33563863	6.46725527	6.11903346	6.10473355	5.15340551	6.13191431	5.55470258	5.831357	6.40714991
ARL13B	2.15781036	2.20154784	2.0161364	3.42713249	3.67380887	3.98458673	2.30476402	2.7740233	-0.29967801	2.80344006	2.94847138	3.85295641	4.13528475
RNASE2	2.60321109	0.89063256	2.15372394	3.24785509	-0.15296102	0.89409216	0.54595612	2.08155821	2.9415114	3.02255415	-0.78832799	-0.78832799	-0.78832799
PTK2	5.76560739	6.17622655	5.75563468	6.4484766	6.80123331	6.50616774	6.5399581	6.7944592	6.42238615	6.43504096	6.75368636	6.89142572	6.40383671
PTAFR	4.57463396	4.03587462	4.21631165	4.77008504	3.01923542	2.6402998	2.98181869	2.28992503	4.60143572	3.54599917	1.85340435	2.84863609	3.76070727
PTPN9	5.59354255	5.32699925	5.62616531	5.09337808	5.35862306	5.28560844	5.15954404	5.63929913	4.88611153	5.41219421	5.323362	5.42262387	5.84932955
RNASE6	4.29234366	4.89326508	3.73744551	5.16750244	4.38128782	4.23836494	3.67765793	2.7388539	4.16176863	3.76704011	4.03059389	2.73488589	4.53472996
NPR1	5.68221749	6.45362145	6.11792112	4.13708624	6.78202493	7.30700311	6.25252352	7.13526629	6.29824222	7.57248493	6.80578868	7.23143024	6.19776937
KCNK9	1.49641061	-0.12590319	-0.01766024	-0.06463313	0.94073372	1.70348633	0.89048256	1.55414466	-0.38166904	3.82390781	-0.38166904	3.62639916	-0.38166904
SCN9A	2.53619324	4.72443286	2.80448396	3.67782079	3.39019801	3.92544977	4.01152673	2.70044354	3.84140756	3.903123	4.15882449	4.70091605	3.8695619
RASSF6	0.41433292	0.6304919	0.37844151	0.37970575	0.37583928	0.37728608	0.37583928	0.37915912	2.31155242	-0.17692647	-0.17692647	-0.17692647	-0.17692647
COL22A1	0.68494627	0.8786327	1.43679796	1.64869191	0.60343553	0.69105914	0.57296132	0.80549388	2.29366125	2.79879174	2.0500535	-0.50071002	-0.50071002
SDC2	6.28076054	6.82133398	6.51180292	7.43600617	7.72934436	7.61466165	7.41051266	7.45180131	7.35416022	6.78444719	7.53876241	7.58279547	6.41139592
CD52	2.87725253	0.34560227	2.68115851	2.05407544	0.45585625	-0.69224064	-0.22576844	0.26781706	-0.44867485	-0.44867485	-0.44867485	2.62681213	-0.44867485
MMGT1	5.50913364	6.18216033	5.5064739	6.34212801	6.73979721	6.78987387	6.75726235	6.56887745	6.33969125	5.8065097	6.42247146	6.23762072	5.19775731
TM2D2	4.77173635	4.89463997	5.06847415	5.83122015	4.85896395	5.18407524	5.11219428	4.72347188	5.29163591	5.05833501	5.21183431	4.8286681	5.03808949
HTRA4	3.90344362	0.98888883	1.28395015	0.90777871	0.97503805	0.60079039	0.98566145	0.01212853	3.29681797	-0.34525343	-0.34525343	-0.34525343	0.79887612
PLEKHA2	5.61538689	6.15765038	5.44235245	5.49434684	5.3989391	5.60477682	5.98870259	5.15299893	5.20429597	5.61916416	5.59482859	5.48384845	6.25069214
CLIC4	8.26031921	8.59244979	8.45472557	9.53511542	9.69614945	10.0729198	9.82004506	9.95722254	8.93757919	8.30615094	9.00276165	8.81438214	8.24649998
SLC38A11	0.48030667	0.9566593	0.48287166	0.69508092	1.14424837	1.54114602	1.01151161	0.99433545	-0.39690803	-0.39690803	2.70316398	-0.39690803	3.24470294
GPR183	3.43187219	4.0015259	2.92340787	5.69505746	3.38435853	3.70012611	2.725069	3.50325581	4.19017849	3.2325639	4.43298386	3.22899306	3.62003045
CCDC8	5.42481308	4.41924468	6.12930795	4.29285485	4.79820403	5.17526412	4.5825803	5.07971095	5.41066686	6.35000641	5.3158854	5.82959679	4.82753822
METTL15	3.03747082	3.72039322	2.43729166	3.97823706	3.80010679	3.95894235	3.78367502	3.59013333	4.10013161	3.19721626	3.2311197	4.78861322	3.11884287
ZEB2	7.27003406	7.59877919	6.92397731	8.22648556	7.00467275	7.1561936	7.23095756	6.93090499	7.41681173	7.40086218	7.33196512	6.90938003	7.62874942
PCBP1	7.7258712	7.16590302	7.02956245	6.90828585	7.00013944	6.69514383	7.15514208	6.35761864	6.64524575	7.29914589	6.73179146	6.73581713	6.84855437
HINT1	4.70144372	5.54126043	5.32869528	6.05403965	5.62413429	5.87545446	5.58749648	5.70387857	6.06804332	5.75258657	5.17274602	6.07313986	4.93624471
DTWD2	3.93182547	4.75660461	3.91543457	3.8040251	4.45090886	5.0850014	4.51184202	4.0740004	5.00956892	4.83644203	4.57370306	5.01220273	4.50706644
CLIC3	0.71973866	2.77040531	1.3075368	0.47173867	0.77040625	0.9551321	1.29145009	1.90137682	3.44987279	3.18919784	1.23696034	1.55533569	-0.29179843
INO80E	5.48943055	4.97854721	5.57758151	4.44795639	5.09039207	4.77792614	4.76028972	4.90890325	5.22877115	5.43361866	4.77747181	5.27117899	5.19037791
BNC1	-0.05024282	4.54898803	-0.02726399	1.47835462	-0.08187309	-0.08105381	-0.04869979	0.36701491	-0.22168658	-0.22168658	4.26734745	1.66752097	-0.22168658
DFFB	4.55433051	3.07863166	4.27373941	3.03999769	3.22468802	3.00583423	3.58907462	3.56566711	3.32260423	4.26954436	3.19949648	3.92517355	3.98509689
NFU1	4.6147257	4.93548729	4.50729887	5.15408139	5.47739829	5.49629049	5.48936958	5.31402721	5.72120353	5.39942902	4.71528678	4.59509675	5.42225983
ANTXR1	8.41131955	9.11384973	7.93495149	7.89763067	8.7268623	8.0601403	8.75545188	8.01644785	8.55764914	8.51913895	9.04605603	8.65588988	8.19839643
CKAP2L	0.52132903	0.66923925	2.94810006	3.50715118	0.5017755	0.70005803	1.31784113	0.64208026	3.61650141	-0.79658371	0.28736972	-0.79658371	3.39560741
C15orf40	4.01720547	3.9993022	4.38511147	3.39580594	4.29487579	3.88120582	4.05204394	4.09868298	4.40877599	4.13871152	4.02937413	3.5597665	3.25066124
FAM103A1	4.76979729	5.14253778	4.08931785	4.35112324	4.53544941	5.87339213	4.0443663	4.38808131	4.37804266	4.49560492	4.07131758	4.31246563	4.27143832
APLF	4.41720907	5.2438666	4.39265846	4.76873015	5.22696872	5.91104186	4.89411127	4.85459721	5.57279189	5.30879856	4.94037691	5.03726484	4.21745524
BOLA2B	0.97589628	1.37109421	1.29406988	2.05444939	0.87653504	0.99754788	0.7128876	0.9471574	0.42974936	3.50911839	0.75491696	0.41702815	0.68995882
HIC2	4.03464555	2.87518657	3.72876371	2.4759692	3.0115467	3.23191458	3.17450384	3.61176552	3.71636626	1.08745812	4.49323178	2.86470126	4.07903817
LUZP1	6.04089867	6.22924335	6.02177357	6.6191676	6.06828785	6.55109294	6.03733359	6.05859657	6.41454653	6.48687335	6.19520912	6.2508411	6.55624954
HEXDC	4.28562569	3.66079104	4.04773575	2.72382787	3.50803253	2.81194866	3.7006306	3.82105038	3.40720284	3.63798579	4.12964373	4.04917724	2.60415208
BUB1	1.89369095	1.39112533	5.05545326	5.62275013	1.25769974	1.47658621	2.89170029	1.21042177	3.991942	2.96973763	3.24930939	3.53733452	-1.27958098
SPNS1	3.40100419	3.22325466	4.78881304	3.55217775	3.5246223	3.15468516	3.44648644	3.52297138	3.23335647	5.12164151	3.75075127	4.3480848	2.93025743
LRRC45	2.74292284	1.41082852	4.42925962	2.08314305	1.94592148	2.1596774	2.86112328	2.78513545	1.73954979	3.74932727	2.21637976	-1.13147263	3.08497539
CHRNA5	1.03530672	0.64842514	1.23076455	1.5534517	0.84363576	1.25909186	1.50970389	0.75421007	2.19672788	-0.80058908	-0.80058908	2.6506945	-0.80058908

AGPAT2	4.7178068	4.34504791	5.06422488	3.62650183	3.78533671	3.68018679	3.51121404	3.68266216	3.68784737	4.64771955	4.75233206	4.28255568	3.75396222
ASPSCR1	3.80861377	3.56059651	4.60607391	3.20829445	3.76276094	3.00917522	3.89756937	4.48570467	2.82442372	4.46792766	4.24028637	4.18514656	3.12133857
FASN	6.01876816	5.35237968	6.61756503	5.63094136	5.39014609	5.38183385	5.59949431	5.36212646	4.75200385	5.17692568	5.88802829	5.63982555	4.56199631
CNBP	8.08150554	8.50673125	7.86039536	8.10442562	8.49691909	8.84157653	8.57523981	8.33918015	8.41870983	8.0034703	8.02657168	8.46610643	7.66817303
MT1E	7.05574941	6.56601674	6.11483313	6.19200958	4.01195154	7.36223644	5.315036	4.558517	4.07248723	4.71431777	4.48579221	4.97991115	4.77996931
DUS1L	5.16068329	4.59216989	5.53293526	4.67651807	4.66512495	4.25371426	4.55641956	4.85423985	4.67830362	4.67429741	5.07603084	5.14059656	4.49087999
GPS1	5.57241985	5.28452603	5.53511166	4.68718113	4.51997902	4.95139441	4.62771174	4.65443862	5.019223	5.9215496	5.31015224	5.75475557	4.96372846
RFNG	4.7941862	4.00131505	4.95333735	3.95796094	4.17851165	3.34853158	4.24122778	3.72484434	4.28508888	5.10207813	4.36976051	4.22540941	2.87147813
DCXR	4.48643758	4.59050547	4.5911451	3.55780948	3.25494721	4.61079867	3.60553234	4.79009226	4.36705549	4.65325237	4.01854879	4.33587586	4.9228114
ZNF32	5.50919134	5.80469638	5.64753306	4.95328372	5.32708512	5.58636917	5.37055774	4.90050365	5.64961786	5.48042956	4.90080531	5.48042956	5.49610124
LDB2	3.52933225	5.32783809	1.61899004	5.96293675	5.56476723	3.70846782	5.04923761	5.59994742	5.91030018	5.33804802	6.56264057	4.68260366	6.79066089
RAC3	-0.25746306	0.20101542	0.48666686	1.1538393	-0.21575406	-0.03458491	-0.4890546	0.46382591	-0.05258976	-0.05258976	-0.05258976	-0.05258976	-0.05258976
NRG4	-0.13741325	1.5731863	-0.1356378	0.16861804	-0.11996168	-0.13642612	0.08627174	0.32437434	0.12304125	0.12304125	0.12304125	0.12304125	0.12304125
LIMS1	6.14159203	6.05270253	6.47071945	7.31293495	6.64639263	6.63980294	6.76603128	7.10895996	6.76916442	5.87325739	6.76668259	6.8809633	6.2533956
NLGN1	3.98469801	3.55713802	3.0247324	2.82710064	4.02960773	5.26999234	4.47216086	4.92787218	4.46196926	4.51593338	2.60193681	4.52625603	4.08520068
TAPT1	5.21335801	5.20235707	4.86505589	5.05922281	5.98636429	5.68840973	5.95373305	6.37284821	5.78479747	4.91113698	5.56816045	6.27519961	5.13385678
UGP2	6.58952599	7.32330245	6.94321373	7.4913615	7.55956364	7.61796009	7.9392028	7.57399981	7.68898068	7.34228404	7.45241774	7.75571481	7.06015524
LINGO1	0.25594361	0.88876426	0.95075677	1.64688842	0.52781311	1.11288079	0.64864086	1.07973486	0.49358337	3.27192112	2.80877638	3.40550849	4.80872174
HNRNPF	7.35528855	7.06542777	7.12144767	7.3199379	6.45415364	6.60956954	6.75755026	5.88067917	6.73480354	6.53594199	6.26288527	6.4965292	6.73540667
BTD	5.03854132	4.92908566	4.4466477	4.41856556	4.21052608	4.24472403	5.42459899	4.28213106	5.21694841	4.94018136	5.25553018	4.52432052	4.92571976
CSGALNACT	6.09987661	6.36472896	6.17755954	7.19058569	6.53116725	6.67411336	6.52657724	6.87274095	6.51762286	6.02327849	6.44391604	6.71559362	6.06061622
PCDH7	7.91384059	7.99142206	7.73112479	6.80656446	8.54595341	9.08997703	8.87219825	8.87673738	8.57935215	8.71822585	8.36767973	8.97533941	8.42087478
ROBO1	6.63020439	5.78627789	5.96490586	6.47972469	5.57015288	6.57849465	5.83566491	6.04276434	6.20602106	6.56482443	5.9258017	6.37929268	6.57641491
ONECUT1	0.04759325	-0.0602641	0.42956356	-0.0602641	-0.01388368	-0.05893631	0.08873721	0.07617303	-0.0244532	-0.0244532	-0.0244532	-0.0244532	-0.0244532
AVEN	4.53071945	4.80687137	4.38508249	4.48061797	5.34679649	4.64147725	5.20806165	5.99257655	4.77763795	5.48109071	5.5024981	4.78139925	5.5407245
P2RY1	2.54741496	2.99123659	3.14482662	3.15297379	2.71249963	4.50713009	3.67010252	3.28539805	2.61015314	3.68289352	2.3974349	3.82734846	0.99916914
CTNND2	0.34970619	0.52304811	0.35230392	0.38273173	0.34970619	0.48629488	0.39801706	0.76208213	-0.1735484	-0.1735484	-0.1735484	3.13949696	4.84421319
TRIM56	5.54450198	5.72751017	5.74319003	4.38555761	5.63205848	5.16589018	5.34325662	5.35043654	5.31670049	5.96843885	4.72502799	5.09100631	4.74308617
WNT10B	0.03398258	1.17393951	-0.12129667	-0.34591552	0.80044134	-0.10190807	-0.03648224	-0.3429829	-0.17735385	-0.17735385	-0.17735385	-0.17735385	-0.17735385
CALML6	0.48249954	-0.49131248	0.96158438	0.24507046	0.1767982	0.48576915	-0.1247486	0.24507046	-0.03278248	-0.03278248	-0.03278248	-0.03278248	-0.03278248
REPS2	3.67092379	2.96470385	3.55666428	3.25142164	3.87641365	3.98546806	3.71439021	4.210132	4.01921163	3.60874355	3.80909609	2.97815269	4.18674123
MUC3A	0.00177258	0.18296136	-0.00268467	0.19178689	0.64776169	0.15781681	0.25257658	0.0734353	0.97036622	-0.07211339	-0.07211339	-0.07211339	-0.07211339
SYAP1	5.36053078	4.99177847	5.47540397	5.17629804	5.45864208	6.23754427	5.48388541	5.74376588	5.90829787	6.00248963	5.8110049	6.02854671	5.09677961
ITGAM	4.87387758	5.0889956	4.99323093	5.41361818	3.71820894	3.0615711	3.86155038	2.99585143	4.45349895	4.56241264	3.52492733	3.08848031	5.50488842
TPST1	4.64956372	4.45856332	4.78075021	5.91961897	4.823011	4.78932793	5.06843817	5.09229523	5.33498957	5.71911398	4.77618368	4.98754836	4.87929816
TOR1AIP2	6.32090238	6.60132863	6.51098389	6.92579629	6.52832469	6.78347825	6.88137889	6.52832469	6.66473326	6.25368877	6.80376063	6.48680682	6.39658388
TM4SF1	9.06176128	8.75465111	8.06259698	8.6198587	7.01831016	6.83687173	7.69853048	7.01527937	7.59892069	6.32133106	7.31436046	6.55639928	6.74324087
OTUD3	4.74779763	4.38585866	4.28540464	4.65707164	5.03613356	5.12656387	4.89728724	5.05033812	3.84037068	4.85743269	4.68171028	4.85506726	4.63154911
OTUD7A	2.72784861	1.87883104	1.57708511	1.48207048	2.39955541	2.53223146	2.54448974	2.77017793	0.92765981	4.08293076	2.58204041	2.52360449	-1.01487002
GUSB	5.50912475	5.06831841	5.59426622	5.13739324	4.77057301	4.60898834	4.66571077	4.77584612	5.1356917	5.29643937	5.3573039	5.14569994	4.93810961
BRD3	6.09916975	5.70390077	5.94704495	5.17443384	5.46169328	5.65658364	5.60920377	5.39526214	5.60155148	5.7513571	5.78688337	5.51933536	5.89456912
KLF13	6.45719321	6.87106805	6.38322371	5.82836259	6.19518386	6.56787388	6.16418171	5.92634221	6.05560311	6.6277566	6.2723895	6.52440161	6.61105083
FRMPD4	0.01659164	0.28545255	0.01913303	0.04031926	0.03726898	0.08767222	0.01659164	0.01983386	-0.00851474	-0.00851474	-0.00851474	1.25061268	-0.00851474
ZFPM2	5.03533068	5.18556943	4.33469261	4.73422997	4.59676437	5.46830822	4.91089657	5.47688595	5.19522778	5.36956865	4.39492301	5.37317917	5.69717998
ZNF764	4.08853291	3.35773099	3.88594284	2.52639668	3.09279862	2.98391138	3.06225894	3.146952	2.31788857	2.3515216	3.86353322	3.99955145	2.49730523
HSFY2	0.08483364	0.04463856	0.00124472	0.0663649	0	0.27260743	0.15465283	0.38921435	0	0	0	0	0
ZNF747	4.81466909	4.31260509	4.74876413	3.814864	4.14918695	3.91808441	4.31260509	4.31863741	4.42003889	4.69774271	4.25832186	4.64817203	4.1536127

ZNF768	5.56028627	5.21023409	5.46514948	3.93789127	4.95258643	4.75144045	4.25855103	4.60840373	4.47520748	5.68128192	4.69161521	5.26607958	4.50081462
TAS1R3	0.16494078	0.61799436	0.53063261	0.16587892	0.10315897	0.01799138	0.37849664	0.77230718	-0.15056589	3.07606656	2.41718463	-0.15056589	-0.15056589
TMEM42	3.33880444	2.4535406	2.94964994	2.70021568	3.09694517	2.83353491	3.07316522	2.73888393	3.30875871	3.70749249	3.12588914	3.06816118	2.2727602
MAP3K2	6.10895949	6.77917608	6.20683715	7.10484819	7.09856057	7.20693849	7.22342887	7.09734428	6.86475799	6.21594923	7.16455237	6.82424881	6.44638988
PUSL1	2.08786441	2.24678592	2.94553348	1.05298925	0.59811876	1.60772492	1.3063521	0.80363301	2.27655941	2.96136625	3.05262273	2.90062243	-0.52852549
SF3B5	5.75479396	4.73342824	5.66070305	5.48302794	4.56589219	5.32056657	4.71094356	4.81335898	4.80524219	5.70836069	4.57558354	5.01370716	4.55909733
ZNF35	4.74031315	5.20675401	4.80448038	5.244919	4.56111898	4.93749046	4.5355464	4.63851726	4.2633918	5.63205089	4.8229623	4.61203122	4.73894575
TIGD4	0.99796469	0.84686666	1.01970936	1.07908922	1.5115	1.67199698	1.33576736	1.5156205	3.09688308	-0.61103509	3.87885676	3.43726568	2.81619732
IFFO2	5.78726157	4.60860903	5.61404241	4.66389188	5.39917755	5.26678946	5.2858404	5.1133154	4.5377056	5.44385812	5.05532009	4.78542475	5.23505663
NLGN2	6.24668987	4.57828956	5.96231397	4.88354497	5.06313089	4.52592297	5.18499236	5.22308481	4.41657388	6.08156734	4.95862607	5.08004116	4.83613765
MYO7B	0.46032972	0.44218661	1.65429473	0.44125339	0.29723623	0.25981325	0.32061681	0.44653722	-0.09313735	-0.09313735	1.69542403	3.40117603	-0.09313735
CHD3	7.3615691	6.04617573	6.60876313	6.80062839	6.385001	6.51401459	6.58054852	6.60531177	6.4485818	6.84247544	6.60904799	6.08491144	6.83268512
TMEM154	2.98474793	3.71373672	2.05467891	3.46956347	2.3766147	2.18237351	2.49018565	2.41060093	2.85447302	4.10402077	-1.4469232	2.21284773	3.82161093
MYRIP	1.31430674	2.91449858	1.37360466	1.26466623	3.51056759	2.38118003	2.38838584	3.54640545	4.87452131	3.66076072	5.38374642	3.4845647	4.80727462
ALCAM	6.55907176	7.36402713	7.3438796	8.13828384	8.16664398	8.08343243	8.48977521	8.11956286	8.52369702	7.12449544	8.00090043	7.44924502	6.86813297
YWHAG	6.51587419	7.09819327	6.60803454	7.35941112	6.92946449	7.41888977	7.33960048	6.7958881	6.89098461	6.73774128	7.27272456	7.19961261	6.80333704
UBE2E3	4.34339888	4.83807507	4.38032531	5.21765766	4.89265359	5.13660915	4.9562369	5.44036388	5.15152641	4.30170785	4.98092511	5.02383693	5.08507669
CNTROB	5.17623909	4.73776432	5.69168388	4.98976906	4.51923682	4.19940519	4.83436329	4.64340471	4.51275319	4.99862342	4.81235537	5.09117489	4.89077083
TRAPPC1	5.17258596	6.16959704	5.69275138	5.97578865	5.22533001	5.14528405	5.19700191	4.77861385	5.15919365	5.87910546	5.6396709	5.78686081	5.13972891
ZPLD1	0.75671308	0.57710468	0.65160699	0.22326373	0.14277532	0.12052035	0.1588649	0.12234234	-0.16845559	-0.16845559	-0.16845559	-0.16845559	-0.16845559
KCNAB3	0.90616631	0.9032244	0.64511047	1.17069734	0.94426622	0.20110252	0.05013012	0.6729257	-0.19983842	3.20963559	-0.19983842	-0.19983842	-0.19983842
FAM153A	0.66355166	3.04214079	0.52091103	0.71382479	0.15274631	0.72963145	0.3041727	0.68617145	-0.35379696	-0.35379696	-0.35379696	-0.35379696	3.51677777
TMEM192	5.33007928	4.94974601	4.77309948	4.41036695	5.17915579	5.51656044	5.34183367	5.19432656	5.42304818	4.66843607	5.42604009	5.14667918	5.32070584
ZNF778	4.80035999	4.35853679	4.69733633	3.89189906	4.48726927	4.77066687	4.61287977	4.63469832	4.2989977	4.46767066	4.21117038	4.15076898	4.75786133
NIPA1	5.05422228	5.29331689	4.78995666	4.71208951	5.46209114	5.60994171	5.74258615	5.42105473	5.70255788	4.7004367	5.92053075	4.91867041	4.86099122
FOXD4	0.61489462	0.90658903	0.98516866	0.04498344	0.168184	1.67631169	1.2208809	1.53639323	-0.33617804	3.25083941	-0.33617804	1.39877713	1.76754303
UBE2E1	4.19479944	4.49871612	3.41417603	5.30586926	4.716348	4.98987601	4.76591081	4.81646192	5.21258212	4.88485582	4.34009572	4.72014613	3.93594932
HNRNPA3	2.37920533	3.70885116	2.89075915	4.65710445	4.53500032	4.30476441	4.71662647	4.28154257	4.21062915	4.68859985	2.63352177	4.30645707	0.37394845
SIK2	4.90436968	4.11620206	4.08483001	3.64987368	3.87967269	4.06580654	4.00994165	5.48873601	3.73629468	3.41693187	2.42858557	3.42523888	3.81509394
RNF150	5.27248287	5.62021563	4.89119593	4.84553515	4.89514666	6.10486586	5.784318	5.03601586	5.33976338	5.7527377	5.02985192	5.83321897	5.93903817
CCDC144A	2.71961906	2.47500838	2.59041254	2.4722843	2.35102942	2.90869125	2.42133254	2.65728069	2.96221503	1.98049047	2.37310548	-1.32529879	2.21172716
CHRNB1	3.07340496	3.66171506	3.75389071	2.44115925	2.6100973	3.68747432	3.02013852	3.17082713	3.65521854	3.72999525	3.43017733	2.9275767	4.04304065
GYPA	0	0.03069756	0.10728323	0.02195633	0.1650363	0.25430215	0.02364429	0.23522873	0	0	0	0	0
USP38	4.4172192	5.24937514	4.74601994	5.98341009	5.52798373	5.70707075	5.6301492	5.5108644	5.51417478	4.93427269	5.34285905	5.59884006	4.92757999
SLC16A5	2.78405717	4.0580734	2.89340564	3.05142544	2.22603826	1.11228846	1.88983829	2.36074569	2.50561246	2.22475559	1.28656367	2.60100663	3.86610449
NANP	3.68951405	3.57644517	4.32937678	4.82630762	4.37587823	4.4782115	4.48842717	4.49179487	4.75768772	4.45521122	4.60488822	3.85224141	4.68070345
ANKK1	0.89549383	0.61728243	0.68109361	0.27501853	0.65224388	0.68081155	0.53971252	0.59271357	-0.21180025	-0.21180025	-0.21180025	2.66454165	-0.21180025
ADRA1B	3.37509069	3.72133619	3.43223097	2.1871331	4.84449397	4.94394692	4.64464144	5.60155814	3.78953897	4.78474491	3.74542838	5.00214395	4.84681617
PWWP2A	5.72555563	5.73854659	5.25880911	5.60055264	5.99867066	5.94790613	6.19611729	5.69042576	5.53719803	5.16320398	5.80733107	5.32016255	5.59278019
USP50	0.64152871	1.68150896	0.96539116	0.02933058	0.33073766	0.07936026	-0.15197788	0.08434487	2.35893374	-0.22549952	-0.22549952	-0.22549952	-0.22549952
USP47	6.05229018	6.81724004	6.34004966	6.93196853	7.00684165	7.2005506	6.96792707	7.02383498	6.78168195	6.71900322	6.47309313	6.53982739	7.13185877
PDCD6IP	6.56089099	6.86613106	6.42889432	7.34434379	7.36291547	7.41802474	7.24651839	7.29178979	6.88212689	6.52577374	7.28883828	7.03980176	6.83320076
ZNF212	4.50684702	3.60637255	4.3592787	3.365938	3.69490993	3.59407776	4.15869569	4.01864152	4.04215287	4.39843687	4.36812974	4.00266377	3.47820456
FAM161A	3.31004489	2.98313071	3.55935334	3.31904321	3.5889037	4.40060765	4.19100311	3.78930798	4.43998409	3.83668314	3.7877095	3.74658338	2.50958015
ZNF282	5.40859366	4.23632072	5.62691972	4.37148809	4.77932106	4.09502133	4.52235283	4.86334124	4.13475922	5.12779486	4.85576419	5.55957551	4.64598196
GLB1	5.20401589	5.03678228	4.70910042	5.54770931	4.73581117	4.42111978	4.62099372	4.50827891	4.53104084	5.41542258	4.82496096	4.55954544	4.68212187
CRTAP	8.46739049	8.91097898	8.21234602	8.03720122	8.675553	8.06860881	8.64702359	8.37617748	8.56704369	8.61571483	8.64783328	8.6610214	8.12845035

HSPB2	2.54533083	2.47491141	3.46293764	2.6191934	2.71987948	3.65940875	2.79416483	3.2657292	3.87263358	3.86595167	3.19884958	4.42435956	1.73868806
SLN	0.130543	0.66754535	0.13307663	0.54149737	0.52341069	1.66113033	0.774955	0.13377532	-0.40608865	2.08362576	1.82884546	3.04825442	5.93444007
CMTM8	0.18813448	0.24571935	0.11612103	0.14878049	0.05080935	0.80156143	-0.0417216	1.02807344	0.05920121	0.05920121	0.05920121	0.05920121	0.05920121
GABARAP	5.80878217	5.66237302	5.35424831	5.66029136	5.66946844	6.1685627	5.88404445	5.6449485	5.95771872	6.58004596	5.91824097	6.06680602	5.80358872
STX8	5.2154237	5.47027054	5.27376907	5.04280502	5.38491554	6.09736655	4.95737314	4.87199009	5.38736638	5.24626109	4.53844296	5.07688984	5.44604529
CDK1	0.43120569	1.30279719	4.15203233	5.5630579	0.96932465	1.15330755	3.27658504	1.43705641	3.96861046	-0.99505281	1.64454557	2.7682286	2.58577431
UBB	7.49232252	7.23138713	7.46278267	7.58263594	6.66901651	7.79947243	6.54806602	6.71498236	6.9538857	7.56141351	6.9422437	7.66704326	7.02134846
NFRKB	5.99590877	5.58348232	5.22191833	4.86065067	5.22493328	5.01574448	5.64804236	5.57891447	5.12580619	5.62576832	5.30146168	5.57879235	5.52107645
FABP4	2.0678186	4.91716837	4.26335035	7.2223368	3.89158187	4.85122065	1.80957728	3.7419279	5.51178211	5.13977455	5.75896474	4.68846739	6.75803796
PRDM10	5.19452272	3.94299287	4.78911869	4.48330111	4.78767265	4.59794872	5.16009707	4.76330875	4.80694787	3.60350212	5.22883398	4.65420473	5.0409021
B3GNT2	2.68182628	2.17857806	0.95827387	2.77281717	2.60339304	3.17994647	2.43660905	2.297175	2.83145634	-0.46521786	3.53057232	3.2795007	3.24654139
FOS	6.22205497	8.36933364	5.82481372	7.75517992	6.23676319	8.16147278	4.29092828	5.21507547	7.23420134	7.39370244	7.15060738	7.96103707	8.32367852
TMED10	6.93398749	7.57793978	7.12116365	8.01542191	7.71786229	7.48126581	7.68526971	7.36073258	7.70363573	7.091551	7.92810764	7.51983219	7.13213057
SETMAR	5.59660814	5.02551539	5.25399361	4.75958738	5.16195596	5.71173346	5.35378805	5.34457499	5.28398964	5.51620337	5.14771307	5.26962941	5.86904805
SMAD1	2.18460139	1.97511816	1.31603411	3.22381449	2.16963196	2.24861275	1.43504092	1.51003346	1.72250434	3.30367628	2.395176	3.03412491	3.112445
EMX2	0.32114241	0.69348903	0.32369977	0.41603696	0.35630954	1.1656839	0.59909868	0.64362303	-0.14182632	2.32719791	-0.14182632	-0.14182632	-0.14182632
CST1	-0.03177274	0.93911941	0.51024303	0.27628297	0.42779811	-0.03040149	-0.03177274	-0.02862626	-0.07628029	-0.07628029	-0.07628029	-0.07628029	-0.07628029
SP7	0.08387943	-0.03932981	-0.03738276	0.46550566	0.0836662	-0.03824728	-0.03932981	-0.03684583	0.00990261	0.00990261	0.00990261	0.00990261	0.00990261
SEMA3E	2.51755418	3.01816939	2.26419813	2.14124053	3.02879766	5.86536352	2.67512188	2.68202169	4.35277888	1.74674351	2.36412819	3.58417648	4.58124546
LRRN2	0.61712094	1.99676329	0.86604728	1.94870492	0.81167721	0.99344585	0.73138793	1.62090474	2.95300861	3.62186284	-0.52795533	4.07621004	3.53531648
SLC30A1	4.54389579	5.14660606	5.09062146	5.9869182	4.77356947	5.70047246	5.50660163	4.56627234	5.11909891	4.50786764	5.1239355	5.16385754	4.44300947
DCLK2	4.8145392	5.16004489	4.46770964	4.40039365	5.19266556	4.39323691	5.18386837	5.90677375	5.29136544	5.68950695	4.83818419	5.91363689	5.67070067
ZNF804A	0.47049568	0.11126734	0.31152018	0.14386782	-0.00968775	-0.00828681	-0.00968775	-0.00647315	-0.03911261	-0.03911261	-0.03911261	-0.03911261	-0.03911261
GPRC5C	5.54301332	4.35250151	5.79180031	3.22523959	4.72625507	5.39626736	4.87833985	4.46226471	4.40789886	5.65649259	5.28181095	4.89083746	2.82943728
TMEM182	2.99094649	2.55193701	2.526641	3.43514706	2.6912938	4.12832093	3.36566332	3.04039318	2.92676668	1.90456564	2.92676668	3.58107396	5.48666897
KRT8	4.28108055	6.56571786	4.62224809	4.99737043	5.34613029	5.24402021	4.28023909	3.73861057	5.08209223	4.43506541	5.40246249	5.41381809	5.55187523
ADORA2B	0.97648579	-0.10957714	1.39097536	0.9265866	-0.04613278	0.20202838	0.29951999	0.71051579	-0.77406051	-0.77406051	-0.77406051	-0.77406051	-0.77406051
SDR9C7	0.06880938	0.0253136	0.02777941	0.0253136	0.10270817	0.34615918	0.59656693	0.49337701	-0.05315349	-0.05315349	-0.05315349	1.78375677	-0.05315349
MGMT	4.29635484	3.88924869	4.77675586	3.81648102	3.63636599	3.52775392	3.82293636	4.56976375	4.16172614	4.26660241	4.90411715	4.52153179	4.36439243
METTL7B	1.77652637	1.23022082	4.0342923	2.88816567	0.97398617	1.31717781	1.09551187	0.86062621	2.17871302	-0.65876931	-0.65876931	-0.65876931	5.5275296
KRT86	0.01221828	2.13949118	0.2232102	0.06144233	1.05167736	-0.49522235	-0.39411441	-0.49397281	0.06233763	0.06233763	0.06233763	0.06233763	0.06233763
HARS	5.92204025	5.21154799	5.47388529	5.51060392	5.15948644	5.27861833	5.03065076	5.53949715	5.09151153	5.62268948	5.65651327	5.77310482	5.06000835
NFXL1	5.0882686	4.57480846	5.06255273	5.33679755	4.70143187	4.93535374	5.07965138	5.2795335	5.26852561	4.21942173	5.42351507	5.31265829	4.69035912
DENND5B	5.63666618	5.54855898	5.12566443	5.07023774	5.83891317	6.07701595	5.91224456	5.91719021	5.91156198	6.12215282	5.75792911	5.58814393	6.19341372
CD14	6.42157796	6.01433972	6.32316853	6.43802296	4.44749729	4.07607244	5.14588524	3.87786358	5.54248646	5.59843687	3.9241242	4.6025466	4.86504757
DNAJC18	5.1357103	4.64093764	4.81874358	4.18580122	5.36952835	5.5538174	5.36270197	5.25816112	4.88258225	5.88936356	5.29771908	5.25551514	5.15269087
SPATA24	2.72967079	1.48336395	1.49045119	2.0573675	1.79385583	2.94177801	2.19544608	2.52759902	2.65062244	3.12020453	0.85208379	2.00382967	2.31080628
RALGAPB	4.80378678	5.19415126	4.6424174	5.40093643	5.5640207	5.45556247	5.55467903	5.55752002	4.45816284	4.90079684	4.71011896	5.05522943	5.49444621
MZB1	2.54625556	2.86070498	0.19263269	1.8485595	0.35145027	0.700353	0.73822774	0.76298578	3.28095704	3.43108648	-0.37330362	1.48414511	2.74898317
NPAS2	3.79656245	2.50672558	4.22637454	4.28322211	4.52041787	4.62071226	4.26861457	4.8676628	4.15798646	2.82298891	3.19789201	3.53663832	4.96884377
LONRF2	5.85704476	5.74915218	5.50222998	4.01340806	5.40977491	6.36044392	5.83452622	5.78362781	5.85151922	6.49825001	6.26296533	6.26763673	6.32054345
NUDT9	5.57809553	5.25386948	5.39676544	5.23407155	5.3733988	5.90305895	5.51708635	4.66807313	5.24213708	5.24498571	5.2370836	5.23430705	5.42912615
HSD17B13	0.68629888	1.05892671	0.89480398	1.17583011	1.17861132	1.31340947	1.16313336	1.8681423	0.67856215	3.28431777	-0.50653759	-0.50653759	-0.50653759
PA2G4	6.79241206	6.20016651	6.72184841	6.02136346	6.34996357	6.66261293	5.94467737	5.47000399	6.22012606	6.34549253	6.04327305	5.95281933	6.39226594
ELOVL6	3.50489845	3.07926891	3.7364026	4.86126787	2.84498366	4.88526411	3.57431502	3.01047733	3.73955713	2.70850927	3.74919632	3.5357953	2.87092563
PFKFB3	4.06838441	1.27857048	3.82090961	1.99748935	1.13308558	1.41965336	3.02117126	2.72975232	0.67101622	4.02233338	3.5461935	4.25841079	0.67101622
TMC7	1.53599082	1.73744708	1.85010234	1.81935167	2.76297535	1.55717236	1.66124993	3.42529733	1.84766094	4.4576389	3.26319069	2.47719713	1.12498843

ARL6IP1	5.34472569	6.43260982	6.3657499	7.31491224	6.61295458	6.7089322	6.80286105	6.67847822	6.85257849	6.07873747	6.73953577	6.41830283	6.15734156
SERPINB9	5.04711949	5.25282964	4.79400594	6.03704824	5.12058028	4.38213495	4.50264042	4.72401021	5.23061982	5.13846656	4.93982924	4.75551863	5.28361177
SMAGP	2.78067666	2.40225229	3.36037139	4.79822535	3.09183568	2.30273187	2.2376319	2.528878	2.24282481	-1.26869813	2.9039118	2.42382189	4.37143333
IRX1	2.56130988	0.53422714	1.62086062	0.3074217	1.53243003	2.26081612	0.60102552	2.04341944	3.18659722	3.88810407	1.71104176	3.59405004	1.20652436
CDH2	6.69389576	7.60229494	6.09150709	6.1981464	6.61584019	6.64130811	6.18381523	7.54210987	7.10691702	7.04822659	7.23405475	7.34047634	7.63520593
IRX2	1.69770019	1.13446973	1.5153183	1.05122784	1.04450899	1.14820728	1.00517656	1.49861287	0.09442278	-0.58133315	2.4041748	3.25086226	1.79367882
EMB	4.1679987	6.38865449	3.92983693	6.30860736	5.31135671	4.91974247	4.9352149	4.09759666	5.61850978	2.75846092	6.07533606	4.69068804	4.57550088
SIX2	3.23795095	2.16546873	3.60257478	2.61070758	2.30864656	4.0437137	2.71640205	3.87108067	3.3908895	4.12711493	2.15017449	4.17286892	-1.02837115
DLGAP1	3.80394944	3.1716011	4.56706696	2.63373745	4.87403884	3.75661549	3.23284646	4.35677415	4.54331803	4.01366239	4.08320484	4.14653705	2.00902916
STAT2	6.4807981	6.47774944	6.45552009	6.86074497	6.23695274	5.56738983	6.62250834	6.21126991	6.18748111	5.8554393	6.86723328	6.24483925	6.57480317
NUDCD2	3.07474842	3.64863657	3.58926158	3.65694172	4.28774581	4.39295119	4.00966232	4.2035679	3.96571438	3.19463477	4.25030485	4.00973235	2.3076913
IRF2BP1	4.96506448	4.18261507	5.11742935	2.88375171	3.59262302	3.92952873	4.00336978	4.31012536	4.16635874	5.39614704	3.73189271	5.03898664	3.10727798
HSPA4	5.71870764	6.13965421	6.149348	6.43119825	6.96028716	6.97243789	6.44796073	6.80738052	6.81852516	6.47403646	6.60235692	6.7964177	6.47713577
FOXA3	0	0	0.13249543	0.04325717	0.08510027	0.36689483	0	0.00158797	0	0	0	0	0
SLC26A5	0.65528265	0.29129921	0.29385035	0.29129921	0.48765624	0.80851162	1.21371726	1.27674425	1.71245339	-0.4255835	4.50681424	2.09391744	2.90295389
COMMD5	4.49650102	4.57401369	5.09357042	3.76631699	3.71691272	3.4816105	3.91186901	4.36111183	3.92099198	4.0872371	4.02752715	4.45592052	2.97161099
SGCD	5.52736606	5.98880354	5.29761871	5.86301365	7.82783183	7.39118481	6.6769287	7.78284471	6.81213208	7.23010359	6.81378639	7.44920159	7.14331252
GTSF1	0.70405136	1.23592407	0.6444425	0.45286975	0.67477402	0.45429894	0.45286975	0.45614917	1.56354138	-0.25182659	-0.25182659	-0.25182659	-0.25182659
ZNF16	4.33886969	4.62270867	4.48293655	3.91409514	3.93208382	4.25817196	3.66063572	3.27173806	4.47227131	3.5794557	4.03157394	4.07814865	4.11270958
ARMC10	4.56069153	4.82801116	4.44803503	4.60741256	5.36229252	4.72015454	5.33928521	5.34799344	5.07593202	4.40606091	4.79647997	4.72924471	3.93596172
RNF34	5.30245823	5.58035288	5.31306975	5.46400617	5.22322257	5.52696762	4.97842666	5.19629388	4.84281223	5.25879531	5.50294898	5.44547868	5.54702234
ACYP2	3.02157021	3.04716679	2.17735772	2.61471111	3.98166569	3.32447885	3.69071029	3.23668779	3.51343361	3.3954495	4.27224313	1.28280826	2.62123392
TRABD	4.56806309	4.63801665	5.31135814	4.1005817	3.44650732	3.24518105	3.62590849	3.70080205	3.06046209	2.66351162	5.0414185	4.22748305	4.17002621
TMEM133	2.8705736	2.73240555	2.37055513	4.66512833	3.00905498	3.34778118	3.41908641	3.18994716	2.94549696	2.34172253	4.13739117	3.80736663	2.72918965
ATF7	6.97599019	6.28182581	6.28329774	6.35547819	6.96468616	6.6236081	7.00740619	6.67706169	6.13700227	6.7157408	6.76082346	6.16671145	6.97457378
SOCS6	5.16572683	5.82054346	4.73864056	5.27786812	5.71148817	5.85770417	5.86145801	5.83861271	5.60594489	4.37138714	5.74530164	5.8473181	5.16652711
ZNF296	0.35899744	1.07043941	1.34705649	0.20559779	0.03362212	0.05562234	0.15340031	-0.25195137	-0.12199388	-0.12199388	-0.12199388	-0.12199388	-0.12199388
HOXB9	0	0.18453263	0.00124472	0	0	0.00069205	0	0.00158797	0	0	0	0	0
TTLL6	-0.03631485	-0.04678045	0.05942225	-0.04322446	0.02884661	0.02058403	-0.02227829	0.58202013	0.79211703	-0.00680428	-0.00680428	-0.00680428	-0.00680428
POLH	5.34585969	4.76760875	5.42539991	5.75824849	4.78238497	5.07649071	5.59052897	5.09532063	5.39673058	5.25934482	5.92903619	5.56633762	5.62205722
SYT9	0.76914867	0.93967074	1.8209289	0.5976418	0.69611972	0.67609153	0.8156327	1.80231005	0.05991849	-0.39856928	1.91602913	3.13896652	-0.39856928
KCNS3	4.92175527	4.9325778	4.65436787	3.79463198	4.7372797	4.95134675	5.29990931	5.14938599	4.72401679	4.77552974	4.82744211	4.87576299	4.95366588
KIF5B	6.95568481	7.65660076	7.18991613	7.58427968	7.95583236	8.340875	7.76012176	7.97831213	8.2149358	7.96678637	7.54986428	8.08617744	7.63959067
GPR37	2.80364191	3.71551873	3.81787794	2.51367477	3.24457747	2.76958337	2.84947005	2.40174557	3.38629325	3.50320916	-1.37807174	2.80254089	2.99517933
AKAP13	1.29059487	1.70146476	1.12069458	1.71578081	1.34689662	1.22572405	1.6562737	2.023439	1.94181811	0.9025826	4.3635125	0.9025826	0.9025826
CDCA4	3.47507837	1.6905655	4.8270115	3.96084495	2.60002059	2.37781455	3.32661584	3.00146422	2.7517111	3.96715259	1.52044718	3.85685979	2.55077183
CHCHD7	5.40201324	6.07929388	5.21827667	5.53619116	5.4416204	5.77177222	5.19495502	5.22650303	5.77764456	5.75434066	5.08388938	4.67348402	6.11131203
HTRA3	1.86003155	6.13982099	2.6737074	3.07542461	3.09138005	1.81938649	3.03465214	3.74166749	3.71592264	6.17124015	0.48867482	6.16774238	3.9374649
FOXN2	5.14964824	5.81908552	4.9476633	5.98169536	5.99258493	6.40589115	6.00230864	5.98653078	5.96775953	5.31577403	5.98297512	5.86038878	5.16169356
FSHR	-0.0009895	1.05484047	-0.11586809	-0.03079719	-0.11806649	-0.11684421	-0.11806649	0.28090631	2.77412996	-0.06167765	-0.06167765	-0.06167765	-0.06167765
USP32	5.63405571	5.90107748	5.24532561	6.01773401	6.05160922	6.20304506	5.83035909	5.86259079	5.8869918	5.65631582	5.57918733	5.81308	6.16576737
CEL	0.958774	0.43077305	1.19370696	-0.36764813	1.14740672	0.17635125	0.78509342	1.64557435	-0.14548652	-0.14548652	-0.14548652	0.88758891	-0.14548652
PPM1D	4.7564182	4.37844054	4.68891527	4.8832172	5.22024428	5.23711388	5.1585125	5.26285311	4.87336382	5.19247111	4.81964989	4.64317031	5.3532872
GPR27	1.0655814	0.28566916	0.94430442	1.07876931	0.79628709	0.43654909	0.66416339	1.53743675	2.89902217	3.44018533	-0.35366107	3.6480462	-0.35366107
KBTBD2	5.26001436	5.62175602	5.31207829	6.3070536	5.97869041	5.95572999	5.93394897	5.84604736	5.90496526	5.31529504	6.03744657	5.44677187	5.918966
TRIAP1	2.69229884	3.86306619	4.31535215	5.09275002	3.47117507	3.78636155	3.61306814	3.73009294	4.31246073	4.4025515	2.597172	4.05386141	2.87711806
LSM3	5.16775669	5.10380931	4.74799606	5.61303146	5.3007238	6.19611066	5.09635744	5.25432618	5.90170036	5.44413658	5.07522035	5.1356113	5.35763165

KIAA0232	6.12553316	6.86594586	6.27974777	6.24669669	7.09744451	7.60270924	6.76521804	6.99679658	6.75005379	6.88936365	6.7576176	6.9137005	7.13073194
MTSS1	4.93042917	5.39064668	5.55441941	5.43795675	4.95434112	3.65501838	4.17612467	3.97769923	4.38313599	4.9460134	5.20447643	3.81494569	5.36426251
TMEM43	6.99837397	7.32907232	7.00978819	6.56552861	7.59563408	7.61537144	7.56838602	7.73552938	7.24183973	7.89780235	7.45154297	7.85159518	7.00192591
RNF139	4.69189539	5.48204925	4.8443668	5.81792435	5.88892874	5.96467878	5.79572969	5.74725716	5.61084762	4.93498783	5.72815714	5.97537037	4.69255292
RPS9	6.97407286	7.13504403	7.18815032	6.71768686	6.12533693	5.91083305	6.16237274	6.46505105	6.80968697	7.27360997	6.46929274	7.18753675	6.12041234
CYTL1	6.49894804	5.04103881	6.43245762	5.92032046	6.61837849	6.1864971	6.69672342	10.1154418	8.95936883	6.63109251	8.03400027	7.40556515	7.0622582
TSEN34	4.77376004	4.55142708	4.63801127	3.25502483	3.59133103	4.03662457	3.99809863	4.00639053	3.7436146	4.73052086	4.76697126	4.08196773	2.71889303
GSTA4	4.17808962	4.63191496	4.1543859	5.26064137	4.64480244	5.13602683	4.3207864	5.07716643	5.01511041	4.5204578	4.83611551	4.9102188	3.92721697
NDUFA3	3.94330468	3.55962832	4.73993343	1.77603477	3.79986866	4.09324111	3.06607257	3.19315785	3.46717924	4.34439691	3.70538466	3.34617597	4.62693857
OSCAR	1.46764028	2.30573773	2.75315173	1.98943235	0.69244931	0.82195014	-0.14176161	0.66139534	2.37047244	2.87322319	-0.53204375	1.28506223	2.68372448
PAQR8	4.92012739	3.891483	4.13062084	4.40404059	3.48110502	3.78854186	3.76055767	3.9651011	4.56165184	2.58750216	4.10500619	3.71116122	3.42823362
NUDT6	2.1636946	2.29399979	2.78880988	2.99474742	2.97390867	3.19976912	2.7437075	2.17142495	2.98326025	-1.21333906	0.92530694	1.99533359	3.35713108
TANC2	4.29211914	2.95342818	3.36592107	3.93151984	3.7912229	2.35132642	3.69899726	4.00097672	2.23695283	1.57377258	4.28164459	4.14836314	2.58765925
PKHD1	0.21515242	0.21515242	0.2177582	0.29968462	0.23684402	0.3041249	0.24257441	0.21847678	1.05764379	-0.12677987	2.61526131	-0.12677987	-0.12677987
DNAJC24	4.41784284	4.71915085	4.75153334	5.47377118	5.24402151	5.34286313	5.32352581	4.98967877	5.70386098	4.62582619	5.04313112	5.01115002	4.22394414
ZNF160	5.61072798	5.61714546	5.25378729	5.63613885	6.15343352	6.03177291	5.60840939	6.26780766	6.04116649	6.27399957	5.48903878	5.84001533	6.03758591
ZNF415	4.31141214	4.04716245	4.81043328	3.74336325	5.10533047	5.02480942	4.69562813	5.05731597	5.17981458	5.45818536	4.12446335	5.14273597	4.84297062
CEACAM3	1.93801043	0.30535984	0.20975001	0.16873598	0.31309556	-0.15883529	-0.16006419	-0.00942113	-0.06048928	-0.06048928	-0.06048928	-0.06048928	3.50476309
DCDC1	0.01081336	0.79125263	-0.03150685	0.1531876	0.0532914	0.15586211	-0.1213083	0.01993904	-0.06179572	-0.06179572	-0.06179572	-0.06179572	-0.06179572
HAS2	3.07746524	2.87757706	4.61429415	6.89534613	1.98142232	2.09702332	2.32159309	2.01183019	2.33203852	0.69793624	-0.78560687	2.14050106	3.03403585
PDGFD	6.51903436	7.00034766	5.62819689	6.49236572	7.0727425	8.26747957	7.01838523	8.0226654	7.44763269	6.29901057	7.38356014	8.03883078	6.91545517
S1PR1	6.11264931	5.41748259	5.05951688	6.03394861	5.62247869	3.87041783	5.13113318	4.63503123	5.2480308	5.0602469	5.84352499	4.25244302	6.04343729
HS6ST2	0.42979797	0.78657285	0.52908869	0.63590206	0.58219023	0.52790089	0.42979797	0.43311366	-0.26795844	-0.26795844	-0.26795844	-0.26795844	2.50203156
PYGO1	5.11672453	4.95055062	4.90127042	5.26381188	5.68092841	5.97632504	5.94626888	5.65185354	5.76179242	5.25200412	5.92838645	5.82133037	4.98657568
LRRC8E	-0.03587163	0.36938004	-0.03354985	0.9437382	0.02424233	0.13312195	-0.03587163	0.20071142	0.13667104	0.13667104	0.13667104	0.13667104	0.13667104
PKIA	-0.34659211	0.13899316	-0.03884652	0.55956782	-0.04546267	0.36532845	-0.06039225	-0.0972448	-0.09447469	-0.09447469	-0.09447469	-0.09447469	-0.09447469
XKR6	2.22948693	2.20760495	2.63384263	1.90809304	2.44796691	2.69356167	2.4141841	2.91119854	-0.25840822	-0.83506989	-0.83506989	2.38242947	2.88117768
TSNARE1	4.76430768	4.24000187	4.95456219	2.92705393	4.01780883	3.65180183	4.26221708	4.44778034	4.40113256	5.24624427	4.66513622	5.04129279	3.67226692
FPR2	3.50680095	0.92666568	2.79454933	3.02367443	0.80557386	0.62190474	1.1894796	0.62358674	-1.20569445	-1.20569445	2.43807869	-1.20569445	3.78476998
FPR1	5.2976735	4.54004748	5.09648994	5.48172081	3.00266826	2.16220103	3.39210104	1.7655918	2.81540863	4.25513965	0.88475297	3.76319165	3.61556816
FEZ2	4.441513	5.37433212	4.34379688	5.66464027	5.4521476	5.46714674	5.33327141	5.19876581	5.70165299	5.22053586	5.6821229	5.07128278	4.71337059
SOX7	1.780288	3.28592548	3.02096977	3.30838126	3.08089864	2.27045297	2.14797943	2.94084353	3.7196192	3.49243805	4.54643881	2.61385241	5.22052719
C11orf24	5.60674247	5.54577789	5.51833342	6.61387719	5.57898923	5.4578477	4.92745883	5.24652215	5.73917927	6.34137156	5.50531237	6.14242234	5.86383391
MTM1	5.02144334	4.64687496	3.91833619	4.91727383	4.84977476	5.43503857	5.07446084	5.07316123	5.48328158	5.33508742	4.45727568	4.39963435	5.17997505
TRMT61B	3.8823486	4.85132218	4.07629782	4.56597274	4.55308916	4.67117445	5.1944245	4.26037998	4.48279497	4.71716721	4.94004983	4.52171393	5.23040281
INSR	6.18382603	6.44948981	6.69569473	5.86338807	7.34242169	6.95826332	7.28757857	7.20633762	6.57062572	6.92424194	6.69988299	7.05530563	7.24604578
MFN1	5.39439022	5.81029354	5.3738166	6.14825609	6.42804836	6.31081423	6.3827063	6.67903294	6.33614547	5.84571517	6.16478276	6.53711903	6.64224103
GIMAP8	3.51046463	5.77280134	3.86390466	5.0422314	5.08449995	3.43618858	4.45548581	3.70633972	5.29808932	4.47988838	5.33832174	4.36243652	6.14444501
HSFX1	-0.0245741	-0.0245741	1.65533836	0.00039273	-0.0245741	0.92537183	0.68732055	-0.02167407	0.43659799	2.68834338	3.14713099	-0.08628285	-0.08628285
KCNMB3	2.72034728	2.49077765	2.43778569	2.89424273	3.50215112	3.32477388	3.61300936	3.94242124	2.8553314	-1.3791607	3.62903284	2.42023027	2.68460401
ATP6V0E2	4.26530592	4.82672291	4.93047444	2.63856796	4.61051845	4.66037796	4.07363934	3.78885754	4.17524231	4.81738938	5.26462088	5.39817542	3.45748266
PRKCE	4.13727776	3.04199988	3.48120709	4.01998216	2.51751741	3.00950474	3.62879252	3.54982359	2.50013324	3.58958103	3.94414437	3.39373068	4.37522499
JAGN1	4.93527557	4.38879764	4.49024704	4.48220093	4.73352069	4.17941347	4.58329089	3.8212032	3.52542079	4.2655631	4.19836898	4.74626964	3.43565294
TADA3	6.68506175	6.23827798	6.6153307	5.9000456	5.85897441	5.97551687	5.78601942	5.8414533	6.02204028	6.74854875	5.97098308	6.10759013	6.09844691
SOCS5	5.51374274	6.39632467	5.14127301	6.55217675	6.19935649	6.37323445	6.1501308	6.2329737	6.52412478	5.75336816	6.28387577	6.36370653	5.84414178
C1GALT1C1	4.41616873	5.25950945	5.13203084	5.98774225	5.78146311	6.08568465	5.83528645	6.08262687	5.89978008	4.68530696	5.71587492	5.62648049	4.74211571
C9orf16	3.01117724	2.22885077	4.76240576	3.14685929	2.50877913	2.8561636	3.35227372	3.68445781	3.28972004	3.42522948	3.39416822	4.61622914	0.47672308

MORN4	4.18416118	4.06913373	3.29034557	2.90326047	3.55922162	3.89727735	3.681219	3.59873395	3.69734507	3.97757339	3.85753913	3.65799964	3.71598333
ZNF672	5.19783339	4.51977104	4.89958737	4.18629328	4.38829278	4.24852165	3.90566999	4.18149482	3.6365492	4.87413214	4.40397822	4.65856401	4.53241922
ZNF692	4.86366535	4.04792226	4.78897142	3.87340991	4.29650998	3.66983838	4.70788116	4.19769362	3.95314488	4.28546304	5.04861298	4.38797701	4.97671998
NAIF1	4.11885358	2.74549676	3.9545469	2.84643584	3.14315625	3.58671493	3.23529703	3.44719547	3.11006924	3.43389923	3.53012326	3.70617916	2.57471051
RBKS	2.25272651	1.59011194	2.2449097	2.3312002	1.15471305	3.03635212	1.52804193	1.5806328	0.57636283	2.97260316	3.28364591	3.01118982	3.07546713
GRIK1	0.17219042	0.17219042	0.17476986	0.35993825	0.62158984	0.36655553	0.17219042	0.17548118	3.39415917	-0.12074237	-0.12074237	-0.12074237	-0.12074237
TMEM126A	3.22923595	3.64005826	3.73914047	4.68321421	4.16505556	4.86555646	3.88747819	4.33655553	3.74668951	3.64899228	3.3100847	3.95449334	3.49265335
TMEM126B	4.17214317	5.22621252	3.89741672	5.06020655	5.43984181	5.12871854	5.35123598	5.58157742	5.12391952	4.6389492	4.34038556	4.9015604	3.76935776
TRIM8	6.95714233	6.21844287	6.61408379	5.80240271	5.59604839	5.46984348	5.85468671	5.85765444	5.95554586	6.32389526	6.01419798	5.88357906	5.76809305
NETO2	1.95803229	1.68897618	2.37280707	3.82828864	1.92072596	2.04374635	2.01715167	2.05002856	2.06142612	-1.5572211	2.24162541	-1.5572211	3.10838261
CDC42BPG	0.38858968	0.82239494	0.57034841	0.62603975	0.69512758	0.21201552	0.42930092	0.97797919	0.79004749	-0.16047885	-0.16047885	3.74000103	-0.16047885
SCAND1	3.63403926	3.67586635	4.42858326	1.94885601	2.90632206	2.42993956	1.7009263	3.01829287	2.76146049	4.65758059	0.92747337	4.20320661	2.9056641
JUNB	7.96191687	7.00503604	8.10503831	6.88697866	5.48439755	6.39461304	5.59911602	5.76448114	6.49463975	6.84894179	6.03672725	6.98069835	6.38206596
TMEM37	4.42067454	5.22563411	3.76537518	4.29050555	2.63137283	3.11155586	3.01708142	2.62232593	3.75793735	5.12955598	3.18810575	4.30093755	3.51088183
LRG1	1.49556481	0.50951585	0.19042066	0.79663081	0.42627931	0.40025733	0.35132212	0.19112344	-0.36697013	-0.36697013	-0.36697013	-0.36697013	-0.36697013
SHCBP1	1.78864089	1.50152708	4.85313822	4.64563216	1.54297457	1.10678368	2.2138584	1.41590993	3.23454966	-1.13148192	3.8086381	2.73969275	2.68323948
SOSTDC1	0.27028995	0.27028995	0.27280245	0.27028995	0.27028995	0.27168686	0.65153501	1.46168365	2.03993427	-0.10700186	3.52642529	-0.10700186	-0.10700186
NPTX1	0.65351206	0.49976156	0.50236728	0.5042884	0.54129035	0.64820749	0.59554674	0.50308585	-0.16172478	2.11817236	0.53097914	-0.69114278	-0.69114278
FAM98B	1.03791943	2.4925296	0.81457213	2.7932125	3.59446465	3.85838485	2.94613601	2.81537965	4.37163473	-0.56198295	4.06098739	-0.56198295	3.58769755
ZNF439	3.51437714	4.30969805	3.77576257	3.42477932	3.88620171	4.17696659	4.42028524	4.88594531	4.84363572	3.75528361	4.28750065	4.34697045	4.24913158
GAA	6.29458536	5.38402173	5.69535652	5.35482992	4.7722593	4.7242556	4.75429368	4.81094614	4.70578881	5.6663084	5.70475014	5.78099232	4.78640877
CANT1	6.15243979	4.96675152	6.28215439	6.16401085	5.0381447	4.99222752	5.74114185	4.83916965	4.86536186	5.90597138	5.36422608	5.3613013	4.49036954
KCNK3	4.87456259	3.09166912	5.11719294	3.08298708	3.32967001	5.27370732	4.16380686	3.36250199	2.03723498	4.88898154	2.39853824	4.18110195	3.2728608
ZDHHC16	4.94385348	4.67688114	4.80070991	5.25775873	5.25950891	3.78956781	5.20631459	5.24075942	4.46593176	4.99525782	5.13747997	4.58789784	4.10634518
CHST11	3.760324	2.96932251	3.61741924	3.74282324	2.70589513	3.78832042	3.06606948	2.52603852	3.50432932	3.39899214	4.53897034	2.7146087	3.44734527
EXOSC1	4.12481975	3.73328111	4.01743773	4.13098729	4.3972298	4.56776445	4.38073301	4.00785409	4.72180099	4.83186179	3.85977777	3.47351372	4.1408709
PGAM1	6.12064542	5.86415106	5.16317649	5.83975597	5.46858224	5.94052345	5.30543262	5.0637773	5.32633704	5.71527642	5.4808766	5.7545802	5.71703259
CHD7	4.5884039	4.70067808	4.16755194	5.18664657	4.0534359	3.74953353	3.66328971	3.94736469	3.45675064	4.11580409	3.8981512	3.77160884	5.56001152
ESCO2	0.95377218	0.91435377	2.10104551	2.9794279	0.78303472	1.04848761	1.3989042	0.76532917	-0.71142716	-0.71142716	-0.71142716	-0.71142716	2.79317098
KRT19	3.17517443	6.24134827	3.92914042	4.44051953	3.08337839	3.42690321	3.7153373	2.80704062	3.78602778	3.80711618	4.65673126	3.5932791	4.80899219
KRT15	0.08086486	0.26687225	0.0727715	0.11688726	0.07017501	0.07161862	0.07017501	0.28067753	-0.20947532	-0.20947532	-0.20947532	-0.20947532	-0.20947532
CLCN5	1.10258554	1.35092487	1.06933835	2.36440514	1.3429073	2.12175598	1.67156354	1.66700698	1.76423376	-0.35962135	1.24531943	2.73335053	-0.35962135
TPPP	4.65164362	5.15495557	4.24352929	3.08628315	4.82159763	5.19473364	4.53672126	4.40313538	3.89111211	4.86533981	4.79926424	4.49250411	5.16700048
KCND3	4.14127537	3.80946205	3.1877051	2.44094814	3.74974996	4.0287412	4.17445446	3.49628339	3.81567529	4.16419106	2.84984442	3.59170962	2.99043434
APLN	6.82737772	4.61361435	5.04014708	5.11323116	4.96249459	3.83823884	2.09028361	1.60254333	1.98884911	3.91655653	0.67350086	2.41566242	2.33629168
PDE7B	0.00554581	2.62256228	-0.13129642	1.4236817	0.62527913	0.20837295	-0.0032887	0.67344069	-0.05911731	-0.05911731	-0.05911731	-0.05911731	1.06630998
MRPL36	3.60399556	3.54746857	3.68580252	3.9094061	3.13781267	3.68429983	2.04882526	3.60012004	3.67345773	4.09214619	2.94508157	2.81641488	3.64897646
ZNF581	3.48112202	3.71875149	3.53804451	2.71889148	2.6362719	1.38987095	3.52108829	2.97262493	2.46047287	3.44656893	3.64961271	2.92371269	3.71727633
NAT1	2.54978621	2.61237725	3.10945829	3.97838614	2.92104025	3.75539177	3.24496749	3.75904482	4.11687797	3.92901587	3.83385306	3.05617625	3.65643427
KSR2	0.12952524	0.11411636	0.22835949	0.11411636	0.11411636	0.24138953	0.15953228	0.23407755	0.84336082	-0.08278658	-0.08278658	-0.08278658	-0.08278658
ZNF524	4.05372801	2.36141696	3.8202712	1.90703898	3.117504	2.43329932	3.31655388	3.10512838	1.94031581	3.81014497	1.50978249	4.19415946	3.20562568
MCC	4.9205363	5.37070303	4.79518405	6.09408137	4.59379896	4.88453871	5.14235581	4.93722887	4.83848327	4.7965857	5.60327848	5.43902851	6.03940299
ZBTB26	3.36597658	3.35826687	3.32251111	4.09675714	4.31446737	5.01774142	4.26638633	4.55335723	4.67891216	2.70875719	4.70032017	5.25742495	3.36902278
DSEL	5.78821119	6.91498619	6.33979558	7.16788694	7.29886937	7.15657428	7.72580479	7.38139132	7.53823192	6.94423001	7.90493338	7.5868188	7.13381921
POLR1C	4.92678894	4.52396413	5.06965516	5.09312707	4.45648098	4.2210652	4.01682619	3.59517819	3.92953466	4.36918957	3.72824283	3.77855303	4.53290053
ASXL1	6.35583919	6.28284002	6.05906652	6.00463429	6.15683952	5.36563574	6.33381815	6.38218948	5.60590073	6.14839479	6.04281474	6.02898313	6.25040563
ZNF562	5.10727523	5.78185304	5.84949588	5.63102644	5.68218746	5.26676969	5.25733174	5.55438776	5.57757561	5.76460824	5.4639361	5.60010641	5.64168341

ZNF318	6.30781814	5.87462236	5.70171277	5.78779313	5.64776751	5.62563259	6.27994752	5.94042498	5.54509078	5.88286457	5.49366125	5.64462828	6.29511868
ZNF561	5.28180668	5.82268443	5.32734552	5.76796638	6.01329333	6.16093129	6.0012159	5.8056117	5.84063839	6.39265226	5.79657929	5.94109557	5.71588477
WIPF2	4.25055965	3.61552715	3.72698683	2.83225259	3.7629551	3.07863928	3.21601023	3.14563068	3.7994327	3.70327763	3.78445765	3.95311156	3.60870791
HOPX	1.39704162	1.62428785	1.26448114	1.43497623	1.34270754	1.36823841	1.61618779	1.00412904	1.71640072	3.20224327	1.33346682	-0.78096681	-0.78096681
LRRC8C	0.40357865	2.31795808	0.5028999	3.61655626	2.47084376	1.67135295	2.12070566	2.00031577	0.69167869	4.21385333	3.43177672	2.75288126	-0.49781766
RSL1D1	6.31016667	6.04998005	6.05056871	6.50707761	5.99218897	6.42980414	5.59732244	5.30757341	6.7115477	6.44516192	5.35443801	5.57843989	6.09745787
LRRC8D	3.96360125	4.27439336	3.91333895	5.21775506	3.52346595	4.02202418	3.96602053	3.79757805	4.31965103	4.72039622	3.89468459	3.52553733	4.21980368
OR1L8	0.64284965	0.93076377	0.55877869	0.44760256	0.34747107	1.04055896	0.3614726	0.81796524	-0.29548782	-0.29548782	1.65742862	-0.29548782	-0.29548782
PPID	4.89460235	5.12605981	5.76814656	4.51958373	4.88662147	5.13975127	5.72821808	3.79153535	5.38061097	5.19491774	4.56655652	4.59405242	4.86725374
COL24A1	4.16387831	3.33057441	2.318894	4.54302393	2.54890232	3.31923588	3.46747348	2.17933342	4.52073179	3.29372154	-1.05273036	3.00298768	3.20629352
ETFDH	5.03485469	5.04705654	4.52040746	5.01746781	5.18373604	5.3979359	4.92064973	5.67298716	4.92141264	4.86772122	4.94778834	4.60528478	6.19613684
RXFP1	1.3468429	1.45694825	1.51815548	2.17462239	4.73055727	2.47782767	1.59091282	1.41152158	1.84287949	-1.01368648	2.54025443	-1.01368648	2.22375717
LPAR3	1.66938228	1.21491923	1.33272624	1.25129657	1.21491923	1.33322481	1.31279499	1.62059257	-0.65093791	-0.65093791	2.54144009	2.71523606	5.42010261
PTGER4	5.28490341	4.58707974	3.40315302	4.41648295	3.11150593	2.61697416	3.23580804	2.42229422	4.02455101	3.47405192	4.06320772	4.55977667	3.4691032
TBCA	5.15051527	5.3753911	4.5209708	5.0817914	5.1611255	5.97844905	5.02598884	5.3574978	5.34421512	5.41644579	4.60377983	4.41171347	5.60421854
MAP6	5.68609766	5.39099194	5.5286022	4.36561293	5.36861248	5.35806274	5.15752478	4.98940832	5.3572754	5.51782256	5.2600669	5.47760981	4.85302305
ECEL1	0.68702929	0.51032285	0.51291158	0.51032285	1.12675236	0.51176215	0.55542983	0.69967379	3.80140362	-0.21457885	-0.21457885	2.41852274	2.80991364
BCL2L1	6.7544097	6.72379456	6.96924011	5.40556474	6.44366191	6.78010643	6.36175632	6.43746241	6.11107064	6.04386763	6.3260571	5.91360638	6.21423683
PLRG1	5.53516717	5.76014014	5.39079533	6.34610163	6.2058335	6.57679504	6.33895174	6.17906771	6.15258337	6.01783408	6.2761707	6.3291958	5.90434851
ZNF584	4.56051835	3.80794396	3.90983791	2.97459161	4.26149282	3.79624431	3.75095979	4.27400177	3.68573824	3.61384606	4.04580619	2.80235685	4.38399966
DSCAM	0.19368046	0.33296526	0.31810489	0.3304265	0.23534484	0.18738004	0.2502417	0.18925231	0.00105669	0.00105669	0.00105669	0.00105669	0.00105669
NMUR1	1.02272004	1.3217282	0.49408148	1.17058069	0.82228158	1.14473789	0.96096591	1.37749074	2.7183779	-0.38511575	4.08395914	-0.38511575	-0.38511575
CLSTN1	6.91134085	6.60292346	7.159622	6.05862004	6.41955415	6.80137316	6.83202935	7.0120163	6.61561909	7.47967662	6.84316156	7.15375986	6.78989377
CXXC5	6.29746702	5.52892665	6.32066006	5.53160403	5.27388744	5.59324131	5.56534732	5.05602567	5.62513892	6.03844856	5.8569708	4.98101581	5.10241212
ZNF274	5.52610744	4.52244823	5.41935157	4.2937335	5.04085241	4.84138109	4.62590378	4.73136182	4.38181427	5.10594379	4.01672904	4.80506487	4.81478163
PIK3CD	3.46889242	3.24603205	3.32043195	2.74644633	2.74323894	2.09388164	2.56475025	2.75380848	0	0	0	0	0
PTCRA	0.63329502	-0.13794033	0.27222926	0.59629812	-0.13794033	0.34463382	0.00549483	-0.1347726	-0.04345415	-0.04345415	-0.04345415	-0.04345415	-0.04345415
SLC25A33	1.96310011	1.71115271	3.20854172	2.22231521	2.11426779	2.72927914	3.36804044	2.44522795	2.93521893	-1.13901237	2.77639546	3.39816213	3.04464378
ENC1	3.3477008	4.09357135	4.29259377	6.05511499	3.7091254	2.9311443	2.85538287	2.73687762	2.32749887	3.07395806	4.41852406	4.28233797	4.48790355
SPSB1	7.14129676	6.89706518	6.73070459	5.92375831	5.88610795	6.74445869	5.61109766	5.72536718	6.12944333	6.68698674	6.19205271	6.2142464	5.03901039
P2RY6	4.30556296	3.80636857	4.2801627	4.49026399	2.75097999	2.7213872	2.28647274	1.659965	2.21251788	2.04558877	3.2652762	2.17133661	1.55344334
BPTF	7.19333562	6.95326745	6.6624868	6.88893124	7.32698078	7.58190654	7.18456519	7.36424886	7.48389196	7.61473355	7.00881781	6.90703977	7.61016206
S100Z	-0.10096753	-0.32084531	0.20692013	0.90525169	0.15203852	-0.31975096	0.21910347	-0.31833423	0.87714652	-0.06730952	-0.06730952	-0.06730952	-0.06730952
ZIK1	3.98816143	4.01112346	3.95490184	3.36341812	3.98659161	3.99415394	3.69161991	4.0336301	3.26227497	3.57416322	4.28952288	3.68573924	4.07351312
GPR82	0.80985841	1.33650437	0.99708873	1.47282426	1.6474561	1.6810667	0.80985841	0.92114258	2.76806111	2.67768443	-0.51658309	-0.51658309	-0.51658309
GPR34	4.53501023	5.35394742	3.76920668	6.27835429	5.48514292	4.67321384	4.57146206	4.51048136	5.47263811	4.69661167	4.0313646	4.78996331	4.54380683
PLEKHG5	4.59185594	4.44224405	4.64362057	3.20753901	3.77598148	3.64674952	4.4562098	4.24218422	3.88987274	3.77381837	4.96900677	5.28597914	3.85238123
ATF7IP	6.64005486	7.07745671	6.31788167	7.19346847	7.09052706	7.32317124	7.28306744	7.05559207	7.24212952	6.20824369	7.32219708	6.91633931	6.89025672
RGS19	3.17480447	2.84360876	4.80898505	3.39777162	2.28300101	2.24929289	2.04143567	2.12136057	2.93649805	4.00882752	3.42426889	0.89267242	3.44484753
TCEA2	5.28124292	4.57684876	5.55376557	3.53406362	4.59541909	4.78524604	4.56484237	4.38197003	4.54650203	5.21613116	4.72480724	5.30374851	4.45409274
ANO5	4.02515679	4.98158312	4.6838868	4.42233919	5.61331975	5.96422539	5.78086087	6.03914405	5.66227999	5.40853096	5.91437497	6.42891659	6.46550143
HDAC3	5.53993412	5.69464379	5.6964204	5.35722167	5.17180684	4.58195421	5.45740605	5.41114787	5.37596227	5.56115344	5.2015219	5.29719917	6.10313155
GPHN	3.91160691	3.4169723	2.69563842	2.98768442	2.69969802	3.62976132	3.38113944	3.14366029	3.39383083	2.46532105	-0.84700219	2.9054442	3.82877439
VAT1L	4.13006086	6.12316683	5.60936064	5.02372959	4.17530582	4.39584824	7.97862282	6.95378451	7.05039618	6.84716072	6.03934712	7.32858061	6.49763552
TMEM51	0.6515714	0.32186487	1.88057048	0.89359902	0.46922343	1.35907175	0.87444333	-0.24203167	0.08232392	0.08232392	0.08232392	0.08232392	2.87583151
CAMTA1	4.50774702	4.64579384	4.14064947	3.93864597	4.95936079	5.28009399	4.72804749	5.25445143	4.92596106	5.51945364	4.33835012	4.30731454	4.29118909
LGALS4	0.75687433	0.15576958	0.99399774	0.02577488	-0.17396413	0.48373258	0.4823889	0.00747132	0.91238214	-0.04097101	-0.04097101	-0.04097101	-0.04097101

LRRC34	2.0228311	2.73936419	2.25778431	2.49394728	2.70510684	3.40176896	2.85163742	3.16812079	1.04490842	2.50942164	4.11068707	2.75153036	-0.34168792
SPATA5L1	4.43299724	3.23878261	4.06778309	4.32364613	3.93496043	4.38893843	4.36747675	4.0419213	4.31687193	3.81636841	3.08672711	4.50442873	4.29874043
GATM	4.07518947	5.03899847	2.84721854	3.35422558	5.15722927	4.72568216	4.51794212	5.12782066	4.48046649	4.96635536	3.96390864	5.52565534	5.75711013
RASGRP4	3.32488412	3.37874409	3.59495838	2.99355297	1.28602151	1.52591583	1.89686826	1.4065524	3.05524591	3.32383673	3.04809058	3.79763606	3.23883697
NHLH1	0.23536725	-0.031872	0.34402546	0.19278971	0.52589033	0.31156401	0.01752097	0.0964553	-0.04417065	-0.04417065	-0.04417065	-0.04417065	-0.04417065
SLFNL1	2.23106444	1.3549252	2.03138935	1.03719187	2.00924412	1.29117965	2.72963604	1.86775465	2.35113374	-0.73351525	2.68500044	2.97110716	2.31570226
BCL2	5.6946619	6.24842875	5.87266471	5.24235563	6.55356672	6.11217843	6.48863367	6.41743398	6.14318231	6.29982411	6.75145315	6.21628177	6.25834748
KNDC1	2.68389339	2.61215158	1.34354456	0.98269914	1.65835496	0.83607335	0.7621904	1.01484629	-0.62394774	-0.62394774	2.98890654	2.59914231	1.0791944
METTL18	2.84679329	4.53540851	3.38209924	3.72464109	3.74944324	4.89443774	3.53673205	3.88357785	4.3071502	4.03003226	3.79927566	3.92322743	2.78876368
COL8A2	5.26991482	6.18832386	5.70657445	4.14407761	5.91594804	5.61148638	5.57463117	4.72250659	5.84940539	5.66808517	5.88729018	6.10821511	5.02324769
PWWP2B	3.48476537	2.85290452	4.16827755	2.28516979	2.03952043	2.20280551	2.74256796	2.6399989	2.84945229	3.22320245	2.90650098	3.26922555	2.8704644
ZNF540	0.32130778	0.36201589	-0.0902467	-0.53613077	0.87629094	1.21016035	0.89500037	0.7468023	0.28224083	0.28224083	0.28224083	0.28224083	0.28224083
ANGPTL7	4.4819133	3.15293877	3.6446337	1.53170075	3.54954755	5.89951604	3.05685204	7.64625432	4.9586615	5.16965947	4.12907371	4.59909581	5.4813344
FBXL14	5.52448732	4.1683997	5.15044328	3.38770296	3.44909491	3.23963369	4.02797293	3.20819609	4.28061981	-1.24248616	4.90347882	4.61889077	-1.24248616
EXOSC10	6.15081243	5.67853635	5.6295864	6.04927327	6.1961158	5.90564368	6.03887143	6.18440784	6.02348247	6.27262815	6.14241689	5.93529761	6.22297128
ZNF570	3.98307427	4.30947011	3.9743401	4.04709902	4.51580046	5.15045816	4.52501227	4.63137935	4.64123496	4.47287954	3.4201368	4.55249381	4.26404738
NINJ2	2.17156898	1.06345261	2.90937317	1.90633467	1.3144908	2.85050667	0.587068	-0.06411201	0.53823929	2.66198023	2.87476092	1.89113563	1.37347152
MLLT3	3.72892255	3.78139128	3.01596627	3.91186427	3.69480826	4.88251145	4.81742569	4.2382847	4.79868413	3.83782706	2.8913996	3.08354061	3.85781193
FAM90A1	0.71509958	0.67219288	0.45373969	0.90986386	-0.098303	0.42304058	-0.098303	-0.09520152	-0.20001988	-0.20001988	1.64294328	-0.20001988	-0.20001988
RRM2	2.58487645	1.84085038	4.68102351	5.67167043	1.48397773	1.9537349	2.66025958	1.85102063	1.8964358	-1.38411845	2.10242638	1.61729072	4.39212762
RPS21	5.16668303	6.53626361	5.47792434	5.5112709	4.93436868	5.07186894	5.12186583	5.32468221	5.3351009	5.78793003	5.27851493	5.97678078	4.91727643
C3AR1	5.2691765	5.67686865	4.91074516	5.7411726	3.75806937	3.91472797	4.58749199	3.58045426	4.84641657	5.22163036	3.095548	4.04517656	5.01505661
PTEN	5.62566781	6.45186769	5.58167818	6.82247567	6.8172747	6.76172563	6.93444845	6.7287472	6.23197665	6.24995725	6.01957677	5.84037859	5.85082206
RPS7	6.73540687	7.53238843	6.53889334	7.37507823	6.7505843	6.35320891	6.73484649	6.58102246	7.01502635	7.46252117	6.37889141	7.14247118	6.76519162
PRND	0.24778096	0.93800854	0.24997133	3.08564016	0.29776447	0.24899878	0.24778096	0.25057536	2.7874463	-0.30610415	3.60995747	1.31541152	-0.30610415
RNASEH1	5.22455733	5.05948868	5.06885877	4.44686229	5.09335691	5.13908356	4.09196943	4.99500009	4.64711807	4.77327822	4.43475221	4.95499185	5.40562905
PRNP	7.79880587	7.80615891	7.01546277	7.34435075	8.0708526	8.1101533	7.70165389	7.95372601	7.82321338	7.94330245	7.54091154	7.85105306	8.02855016
KLF17	2.97168904	1.55554594	3.13456509	2.50669936	2.50095012	1.83734479	3.30615625	1.55870873	1.97748393	-1.09090149	3.25367455	0.99358697	1.40790301
ADRA1D	1.74643963	0.13492945	1.32976571	0.73568769	2.49356714	4.88920402	4.86336234	4.05972058	2.55113361	5.54287792	3.25520281	4.0868753	2.35983997
FRMD5	1.26336906	1.22971885	1.58192294	2.23827625	1.43519413	1.8117603	1.62806577	1.52325262	1.85063672	-0.73523166	4.48832732	1.8626774	4.79924907
CYP4F11	2.76209409	2.2165939	2.76604572	2.0881511	4.19833908	4.68138871	2.46395231	2.88020415	3.75310105	4.24059235	2.11702499	3.53736088	2.1719318
TLN2	2.42501367	1.30158893	2.82261701	0.98226155	2.87209428	3.02062492	3.73189716	3.98435624	-0.09864934	4.7046275	3.14675456	2.69805457	0.94803476
FBXW10	0.79019988	0.60133308	0.71508717	1.10826058	0.68699773	1.16509439	1.24444548	1.40532824	3.60575249	-0.43087951	2.25657156	0.73040678	-0.43087951
ZNF217	6.25911321	6.22184674	5.80273753	6.68775881	5.67549769	5.23971172	4.91849241	5.22621478	5.60669353	4.33481429	5.57763183	5.10204165	5.84183763
SCG2	6.72280345	4.53028381	5.58156763	4.61463121	5.3232373	4.01081094	3.61959437	3.07065583	6.23908773	3.41999985	7.2893149	5.37131969	5.72843276
ATPAF2	4.32275376	3.99425643	4.474482	3.69017388	3.21321676	3.10959573	4.4197842	3.94758067	4.09824318	3.99344126	4.21144494	4.24823662	3.09615812
CYP4F22	0.09698729	0.27040318	0.00124472	0.02214044	0.13249543	0.11721571	0.09181162	0.00158797	0	0	0	0	0
PPIH	4.34036964	4.73447105	5.29145014	4.9810969	4.0248981	4.20881677	3.68529133	4.38457291	4.88198159	3.89374191	4.34970818	4.53844129	4.64820621
ZNF57	2.78147164	2.54893719	3.11009541	2.51160268	2.61704245	4.11551025	2.67713056	3.6484085	2.48501297	3.43728819	3.49642382	2.87408787	2.8833751
C20orf196	2.29197045	3.57196144	1.40070088	2.74852706	1.36047633	2.14553261	2.23244606	1.95412021	-0.7475735	3.2472885	3.39015728	1.63813628	3.99047672
JMJD1C	5.75250645	6.37928109	5.47446669	6.80152422	6.35877913	6.52035643	6.13101699	6.32838429	6.61073957	5.77697438	5.95561107	6.08754872	6.52960199
SYNPO	9.50662383	9.43228238	9.51911821	8.23487893	9.08550005	8.21955161	9.25398026	8.43162018	8.73244166	9.00290049	9.28566842	8.74254139	8.61759912
MAL	1.03484921	1.03585564	0.93514627	1.36062897	1.53959266	3.23003559	2.15300824	2.2710562	2.15882289	3.9507722	3.12576075	1.42046056	2.65766034
ZNF554	4.94973032	4.51163191	4.41337269	3.70163649	4.19898565	3.91844068	4.55279391	4.74778545	3.26270341	4.25439377	4.80342045	4.21745562	5.11126849
RAB33B	4.95137851	5.00478329	4.49778499	4.63391384	5.64146174	5.8872331	5.50479664	6.0075654	5.57230258	5.62110907	5.99214508	6.02253511	4.10356927
THOP1	4.25508047	3.5142804	5.0269001	3.64646248	3.71219397	3.18038105	3.4380293	3.34646943	2.82284263	3.54792215	4.14889476	3.90890621	2.89523604
ANKRD20A4	0.17279069	0.17279069	0.1753412	0.19745457	0.43156365	0.90266322	0.20993228	0.4135914	3.20193763	-0.13559587	-0.13559587	2.96774628	-0.13559587

GAP43	4.03014237	3.49483171	5.34539485	5.21591023	2.56837383	2.60356558	3.29673526	2.28060029	4.55724022	5.01616286	2.22625215	4.54866203	3.70529167
EPHX4	0.71517591	0.83122859	1.12819991	1.60404532	0.80257117	1.13582425	1.46821261	0.75069376	1.13033329	-0.44815631	-0.44815631	-0.44815631	-0.44815631
LAMB2	8.07004913	7.84675671	7.674473	7.35974331	8.08486279	7.81535173	8.28692814	8.46942857	7.46125071	8.22223205	8.20542238	8.27729091	8.29662921
USP19	5.84238874	5.41607201	6.04527963	4.89115785	5.3051144	5.14281954	5.51912622	5.39723971	5.08075024	5.94994326	5.37379649	6.04913925	5.14156563
QARS	4.99143031	5.36093484	4.98367735	5.30131376	4.40189966	4.17846742	4.77175786	4.62935066	5.26406546	5.36130799	5.25388456	5.50179197	5.0491966
ORMDL3	5.51205832	5.59988813	6.05980777	5.55619882	5.40013061	5.08493363	5.52623227	5.58270875	5.96618767	6.38180596	5.98533158	5.63458989	5.8780559
SERF1A	1.20874002	1.03780147	1.41922821	0.80543599	0.95824476	1.35647991	1.33094481	1.42697864	0.41500759	2.25832967	0.88713544	1.20334632	0.67661139
KLF11	5.40901339	6.22531063	5.61580054	5.08520666	4.62187079	5.18655603	4.91522117	5.33055969	5.05518753	5.4961852	5.63488589	6.217485	5.88714123
LRRC15	1.03706214	1.72372201	1.20274939	3.87695763	1.04778353	1.092169	1.01614032	1.09469672	-0.12429464	-0.78753565	4.71017518	2.7172783	3.82540674
SMN1	3.43929365	4.40529536	3.63089553	4.10644065	3.47067998	5.16801458	3.84123297	4.6354208	4.20082541	3.19314953	3.61468536	3.89647217	4.43741523
EIF2AK3	5.11949119	5.72346204	5.0600588	6.3861161	5.99629986	6.16622442	6.05271685	6.27929321	5.63438083	4.6631655	5.56902457	5.85002755	6.31052279
MOB3A	5.8950191	5.49576356	6.00472324	5.76541914	5.39206576	4.86266681	5.38865047	5.04102665	5.14220639	5.02645102	5.56838858	5.48894994	5.35642197
KRCC1	4.27945794	3.78435416	4.60991529	5.31772288	5.31249212	5.67549524	5.38299295	5.12319909	6.15612299	5.42478562	5.15879209	4.7977526	4.6732884
NME6	0.60295254	1.69275264	1.6321372	1.24297743	1.34912022	0.58424242	0.81041947	0.62439924	0.41752391	0.41752391	0.41752391	0.41752391	1.77284908
CYCS	4.82999692	4.8791659	5.01476471	6.60885308	5.83420285	5.82342621	6.03932012	5.47805121	6.12591619	4.4840555	5.9535165	5.92486193	6.30414725
CD8B	2.45351003	1.73399585	0.39517498	1.45136496	0.55891071	0.19729405	0.61142617	0.75661936	2.59531457	-0.35624952	-0.35624952	-0.35624952	-0.35624952
SLFN12	4.02311355	5.25527635	4.67324904	5.56827436	4.97555139	5.12973769	5.20934562	5.57153635	5.5498728	5.45024543	5.8569409	5.45957257	4.87635277
CALB2	0.11875408	5.23394664	0.02355465	2.7775735	0.15561472	0.02293215	0.02215267	0.26620097	-0.42487852	3.68648773	2.33007413	2.15606748	3.03983723
CCL11	0.27994008	0.51598761	0.17871876	1.7304837	0.17631162	0.17764996	0.17631162	0.17938257	-0.08747993	-0.08747993	3.74342877	-0.08747993	3.32441032
FRMD3	4.40317701	5.08275237	4.40867359	4.13555472	4.75065915	5.41935095	5.7051035	5.06535064	6.18162197	4.8471643	5.53631241	4.81535196	4.99136293
SNTB1	5.65562532	4.90312995	4.59515103	4.75573338	5.0823388	5.60333054	4.19884021	5.09116812	5.57015958	4.344053	5.52656888	5.36954736	4.91232864
MTBP	2.1893066	3.17953319	3.11671798	4.44551428	3.39379068	3.63910777	3.63992199	3.77783167	4.36627835	3.0272482	3.09982585	4.25439528	2.25030696
MRPL13	3.41100582	3.43935831	2.95579931	4.47690984	4.00049601	4.76809272	4.18888356	4.16287723	4.18969063	2.91235745	3.66693156	4.11031604	4.13544165
MALT1	2.23171554	2.55190497	1.84857331	3.64118648	3.11523145	3.02539989	3.24027482	2.9022313	0.86898911	2.0120924	3.14957952	2.60912826	3.41577296
ISG20	4.18065108	3.4642611	2.96277158	3.66987966	3.28680743	2.48540841	2.66055946	2.32107391	2.99232824	1.30646103	4.05284457	2.77678066	2.0126835
MBOAT1	5.02073468	4.10567526	3.40315345	4.24838418	3.80967704	3.46616027	4.38241606	3.88097731	4.66138612	2.95714912	4.47168785	3.38282172	5.38629069
ID4	6.6654171	7.28210434	6.16937802	5.61625029	7.83574189	7.01078501	8.4440314	7.26383296	8.25515775	7.05209711	7.9042178	8.18644208	6.0492315
CXCR6	2.33817028	2.52382128	1.12663415	1.76974802	2.40981878	1.43613819	1.6533177	1.12732667	0.42531166	-0.64272564	3.54879369	-0.64272564	1.95078205
CEBPB	4.76474131	5.38523698	5.72286292	3.69368165	4.13973293	5.61898994	4.41405637	4.46749021	4.26838024	4.66870684	4.09767545	4.73718436	3.75945274
AZU1	0.32896836	0	0.34343733	0.01688172	0	0.00069205	0	0.13710693	0	0	0	0	0
TPSAB1	1.14265438	1.98831808	-0.10661898	1.48739576	2.65533687	2.2750821	3.59561555	0.23045167	-0.54717613	-0.54717613	-0.54717613	3.37934042	0.81024866
PAIP1	5.48219269	5.94909278	5.59503806	6.00622147	6.23479731	6.68810512	6.01852971	6.04057454	6.45202582	6.18405006	5.72874861	6.00361815	6.25517476
CLEC7A	4.43417752	4.32777619	4.39057582	5.38197598	3.32948638	2.80165782	3.94423918	3.38163624	4.17122619	2.88987494	3.89717019	2.47551352	4.85141545
C5orf34	1.74924664	1.67532316	1.80309511	2.72500637	2.46987428	3.29552103	1.66452995	2.19302145	2.94759875	3.09591462	2.58651175	2.51316884	-0.89902344
C1QTNF4	0.41958336	0.04758741	-0.19725751	-0.32661782	0.85117696	0.49608137	-0.27409223	0.34532001	-0.05715174	-0.05715174	-0.05715174	-0.05715174	-0.05715174
NEGR1	4.83351565	5.42034459	4.87720487	4.57154875	6.29744734	6.42247789	6.35235084	6.91050845	6.4280353	5.72555951	6.20518772	6.76376652	6.19784117
ZNF131	5.51460757	4.95233972	5.04482912	5.43966025	5.22738391	5.83982415	5.42650056	5.36958896	5.73517268	4.61866861	5.17586789	4.95228284	5.28669685
MACROD2	0.18925389	0.73625037	0.30368269	0.42150121	0.74603378	1.2686403	1.61006626	1.75106172	2.64462462	-0.18362144	1.12426963	3.27384043	2.55590781
DPAGT1	4.92919166	4.56593601	4.57022059	4.81162034	4.09113866	4.26688974	4.40706142	3.7263969	3.44908969	4.10388008	4.07249122	4.18243891	4.28731944
BSG	7.25646262	7.28252445	7.9739995	6.96984275	6.71187893	7.20861164	6.96150976	7.01040512	6.91709305	7.74042459	7.54796991	7.58943396	7.88976728
HINFP	4.74766894	3.94216138	4.41557785	3.71534041	4.2810802	3.5602212	4.66999215	4.62489226	3.99971255	4.38877675	4.04795528	3.37854709	3.41464758
CERS6	2.59401132	3.49792483	2.75841628	4.43797735	3.80966336	4.10450284	3.42470984	2.89892709	3.5254588	3.67500791	2.08719309	2.91504966	4.62729515
SPTLC3	2.80566886	3.20701236	3.00898837	2.93039183	2.47679108	4.04860983	3.56743119	3.32015855	3.29593038	4.05527966	3.80295946	2.91491044	1.29382833
TP53RK	4.19808936	4.62841812	4.78546941	4.99135811	5.13877896	5.18025474	4.83817518	4.41372088	5.09040501	4.92058276	4.92946757	4.93385744	4.81884256
B3GALT1	0.16186851	0	0.00124472	0.00910427	0.15567011	0.60672663	0.27306054	0.26260321	0	0	0	0	0
CLEC12A	0.74637947	3.30925272	1.22433599	4.37140184	2.28337764	1.38343543	1.99309651	1.3187368	3.58673266	-1.01431387	3.68402227	3.59155917	3.23542533
BPGM	4.56766764	5.12979156	4.16917493	4.90859066	4.67029937	5.41918888	4.51306848	4.26079637	5.17435591	4.30178756	4.87097763	4.68500128	4.04212245

POP7	4.7319588	3.30540473	3.70140391	3.12349732	3.69989262	4.75945366	2.68687223	3.15239084	3.36919329	4.12507247	3.71268469	4.38753117	2.79808559
ALG14	3.21892486	2.82133551	3.34197344	3.76950806	3.46383408	3.498452	3.34528608	3.46122056	3.8799492	4.18408458	3.47890176	3.10964938	2.42504013
SUCLG2	6.09506136	6.17238157	5.32899713	5.91836839	5.72472185	6.27157768	6.14448571	6.26859016	6.02253122	6.23497746	5.78053933	6.41664448	5.91743212
STARD5	3.64196185	3.4882108	2.77921229	2.67162856	2.91561968	2.61027979	2.97626567	3.29890165	3.41437062	2.58776958	3.87162611	3.39094816	3.5772907
CSDC2	5.05520385	4.70538658	5.56847991	2.7036967	4.79597859	5.05423011	5.30998535	4.636687	4.02385749	6.02201897	4.69765083	5.45983047	4.75133443
RCAN2	7.27485931	7.87151718	6.88990451	5.95678549	9.07814527	8.86998084	8.42765796	8.51485611	8.23561514	8.57177264	8.70211167	8.50377197	8.44664545
IL16	4.25839854	5.57847151	3.70352851	3.83118652	3.7345841	3.30063165	4.01743868	5.2285026	4.80671943	4.07488768	5.27469069	5.2300677	5.17688678
ABCG4	0.7503765	0.05828028	0.28297666	0.53550345	1.01072972	0.87784207	1.26642104	1.22424124	-0.2929736	2.14743482	2.10783112	-0.2929736	-0.2929736
GNB2	6.48105374	6.43734476	6.81200033	6.18897894	5.84569689	4.96730796	5.86186761	5.92615226	5.64065176	6.85066889	6.2366928	6.55817537	5.55494771
PDZD3	0.68478894	2.78856327	0.56540357	0.37685627	0.53941107	0.37818937	0.37685627	0.73127449	-0.24924604	3.65541728	3.49623262	-0.24924604	-0.24924604
C2CD2L	4.04052583	3.63505596	4.73401501	3.60457387	3.27081005	3.54224077	3.67822896	3.65248061	4.21596785	4.2791163	4.40385666	3.59527388	4.02193357
ARNT2	1.09242941	1.14176451	0.79099906	0.18598657	0.26121205	0.00564615	0.72462772	-0.13780757	0.35819418	0.35819418	0.35819418	0.35819418	0.35819418
GNG12	6.97550546	7.37393266	7.17440158	7.77788487	7.94860878	8.12486147	7.94587643	8.18506589	7.92464096	7.87250367	7.81823263	8.11725284	6.87676623
PRSS27	1.06187051	0.23249088	1.09509042	0.09756662	0.46811131	0.38747919	-0.22562698	0.24923088	-0.13960517	-0.13960517	-0.13960517	-0.13960517	-0.13960517
MYOZ2	7.04381283	4.11213338	5.8730949	4.40643299	6.7486675	7.30846799	5.8078461	6.85076252	5.43579789	6.061514	5.8300254	6.43767454	8.22846663
SYNPO2	6.60452146	6.38947576	6.16276857	5.09727504	7.11730847	7.54585493	6.89605196	7.13652454	6.51695991	7.66197828	6.80270475	7.17658603	7.2746388
CLP1	4.38220941	3.85865076	4.02950794	3.026085	3.28929957	3.77326699	3.63401221	3.84681251	3.58739941	4.4440768	3.48951758	3.8776155	3.79986547
TTC36	0.50185597	2.19840693	0.62829364	0.16785328	0.3320138	0.43779383	0.3909383	1.27712739	2.75089882	3.26168559	2.32271664	-0.19801143	-0.19801143
RSPH9	0.95512644	0.29539201	0.46960281	0.916391	0.71972587	0.41101835	0.99283597	0.73335673	-0.3278404	-0.3278404	-0.3278404	-0.3278404	-0.3278404
GTPBP2	6.25015109	5.27609341	6.04076692	5.78290659	5.75997252	4.64545895	5.58753339	6.0064224	5.14998484	5.18584678	5.28037145	5.28507803	5.60445373
FGGY	4.65684359	4.620034	4.44864606	4.13464211	3.72729477	4.3015965	3.90292049	4.78267266	4.76733503	4.90057284	5.18223999	3.68205967	4.7504736
IL17D	1.40359248	0.46855004	0.02922892	-0.45641775	0.53099608	0.48789432	0.51393428	2.89080073	0.0809386	0.0809386	0.0809386	3.93946713	0.0809386
TCEAL1	5.31575027	5.94808993	5.23655996	4.8701047	6.51147765	7.34878698	6.20172746	6.92279302	5.95631698	6.54231606	5.24751528	6.39974678	5.3413055
ZNF24	6.34930842	6.89516071	6.37766615	6.95948637	7.06618293	7.37269298	7.08637256	6.82150339	6.76984193	6.39450428	6.80603045	6.7587193	6.57411241
MANEA	3.94096404	4.53975781	4.22578758	4.72762126	5.06029628	5.75193005	5.12400293	5.4474	5.21891361	4.02634723	5.03583454	5.36068923	4.49997041
AFF1	4.36411367	4.53816516	3.51220536	4.09752668	4.37329267	3.96579344	3.63603017	3.69002192	3.41566127	4.3918193	2.58044115	3.09235709	3.96183624
ACOT12	0.97229819	1.02152005	0.70762025	0.42455093	0.51390516	0.64302791	0.65418637	0.35267106	2.20275872	-0.08541673	3.24997519	-0.08541673	-0.08541673
FIBP	5.12637165	4.97818025	6.05005867	5.67673462	4.58615466	5.03314109	4.55628795	5.04571632	5.03593988	5.57623398	5.31506023	4.8421373	5.50388164
CARNS1	3.33168727	5.6770974	3.1062246	1.93867368	4.51508306	3.90474292	4.12667673	3.51456716	4.59978253	4.55628519	4.62665241	4.68931532	3.73544383
BANP	5.00067263	4.05726659	4.76797384	4.55865238	4.4600813	4.1464712	4.99363409	4.23465209	4.17513547	5.00161205	4.59926373	4.72057014	4.73034053
PPP1CA	5.19557994	5.21640318	5.9384661	5.55979533	4.20921438	4.56459559	4.45837363	4.49808041	5.10615384	4.68364195	3.79789889	3.94659101	4.89021578
HCFC1	6.85949284	5.89902628	7.2450108	5.7063625	5.87606634	5.62006187	6.05447797	5.70612738	5.51707149	6.79404965	6.43955121	6.492146	5.63166025
CTSW	1.94800111	1.02214979	1.61552946	0.71648649	1.61891169	-0.00214175	-0.2135693	0.21454273	2.4712324	-0.14637881	-0.14637881	-0.14637881	3.6297606
NIPAL4	0.88933093	0.86882107	1.60133423	1.03335441	1.86099148	0.87024984	0.86882107	1.2224057	1.20571399	-0.64153372	2.66163145	2.77516971	-0.64153372
SNTG2	0.21146129	0.565494	-0.02659325	0.50172672	3.39234769	0.99962872	2.35165776	2.15880638	1.79543028	-0.09901036	1.90217309	-0.09901036	3.33219631
PDE3A	5.03599381	5.4733702	4.41249874	5.91695215	4.59633978	5.27384451	4.74790901	4.70472599	5.12941123	5.37028178	4.82547978	5.06666554	5.23421152
RASGRP1	2.4274654	3.24486805	1.40824363	2.46121183	2.32459944	1.78895156	2.51776401	2.40676208	3.08811351	-0.97724496	3.43349713	3.53912833	3.74502085
KLHL6	4.58950456	3.71775988	3.68717276	4.41334421	3.09583351	2.54754993	3.19070612	2.60175555	2.90889988	2.06758447	3.08088499	2.5733752	4.02795373
CHCHD1	1.85428615	1.99455152	3.22733064	3.00382147	3.89329957	3.61230348	2.68182283	2.76541494	3.37434151	1.91161097	4.04804995	3.82009645	2.52612006
MRPL52	3.5078681	2.46109257	3.84932844	3.67532745	3.12861762	3.84932844	4.15946387	3.22963214	2.25472582	3.75613038	0.46278967	3.76963461	3.46556539
SMPDL3A	4.12432934	2.89674986	2.72958933	4.79171072	2.9564047	3.46089258	2.70546553	3.18519677	3.30338377	3.40047532	2.43469625	2.45134415	3.54177493
RND1	3.16172719	1.4318021	2.00920258	3.06729615	1.48450727	2.36644102	1.68791114	1.56552741	-1.21309721	-1.21309721	-1.21309721	2.19066685	2.07196988
RAD9A	4.16092719	3.20746435	4.37032374	2.96024466	3.33174562	2.57301275	3.63849182	3.58775877	3.44949312	1.62996421	3.84485354	3.67232151	2.55187343
EFEMP2	6.55424679	6.51979936	6.69044299	6.12088089	6.40000797	5.50374882	6.13469918	6.60955558	6.52884241	6.90394291	6.39171346	6.80926832	6.41703443
TMEM134	3.91141992	2.48032364	4.16756564	2.25435907	2.75942188	2.60239643	2.7342924	3.39347958	3.22174544	2.86976918	3.52838094	3.58745426	2.87542572
ZMAT3	5.92102518	6.82202833	5.62434415	7.17824532	6.88075688	6.82606909	7.22066447	5.95982596	7.01328055	5.44473978	7.30379715	6.7719139	6.10811077
ZFAND4	4.19098091	4.50428776	3.94251035	4.53902462	4.88107323	5.01092587	4.77214051	5.12882373	4.98008646	4.72517054	4.33849208	4.68386279	3.92006352

THEMIS	3.08020136	2.65174379	1.31115932	2.93978116	2.28912617	1.43862627	1.75189149	1.97513028	3.69545091	-0.79868465	5.05738453	-0.79868465	2.12058292
ZNF738	3.53084174	2.29592644	3.99984851	3.42051498	3.45560985	3.36287593	3.89414498	3.85171645	3.71325843	2.15378101	4.05527519	3.49002948	1.88648725
SLFN11	5.669239	6.21105694	5.56181936	7.19968872	5.83936836	5.64988545	6.0828478	5.52895279	5.80546506	5.45510134	6.14274232	5.34495955	5.76632971
FAM71D	0.19991825	-0.23130493	0.55076512	0.04003697	0.01150056	0.37867274	0.49260262	0.41532857	0.00522792	0.00522792	0.00522792	0.00522792	1.11388582
CCL19	6.10556286	4.43231874	1.68148845	4.57557179	3.21217256	2.90284619	2.44609442	0.89693509	4.90303132	1.91509233	5.13113667	3.22470823	4.65671533
CORO1B	5.4083302	5.1141966	5.96596188	4.34879999	4.6463485	4.90610808	4.72611921	4.62801108	4.98096727	5.99655752	4.96215579	5.6022131	4.66354764
FUT10	5.15844577	4.70426121	4.97304855	4.81511768	5.060208	5.27552073	5.19365443	5.98530671	5.35115347	5.34266892	5.65950645	5.47597392	5.53827529
LRRC20	3.85098829	3.10504922	4.24368311	2.45248303	2.68170593	3.18380766	3.18947808	2.52593644	3.63307143	3.20663376	3.64868062	3.48302605	3.983277
MUS81	5.03978822	4.38127762	5.23848022	3.87520419	5.09509898	4.6955814	4.78996075	4.80861167	4.53247703	5.51296584	5.19206396	4.73507347	4.70209056
PURG	4.296791	4.52314747	3.62504682	3.22948873	4.70178655	4.82659036	4.66716234	5.38954786	5.51745068	4.27232911	4.82406274	5.54847639	3.46017161
TMEM217	2.42592992	1.0925014	1.69334459	2.55317482	1.62991727	1.57504114	1.8989359	1.72080827	2.14401628	-0.81959344	2.18145953	3.12239111	3.03255555
ZNF596	3.26418488	3.8945086	3.94258697	4.11562912	3.40865318	3.99190268	3.30745841	3.700144	3.55688024	3.81765153	4.12009966	4.34825787	3.03340965
CFL1	6.51023825	6.87019736	7.57642389	7.57642389	6.65591796	6.925748	6.13421842	6.31084003	7.24644365	7.74425412	7.21220395	6.85803936	6.76618076
TMCC1	6.03152514	5.59325256	5.4966823	5.35183412	5.979926	6.10326946	5.75451791	5.80353291	5.54207652	5.67345795	5.79023069	5.69122609	6.09214835
NAA16	3.94281147	4.1002585	3.74215942	4.86328473	4.88729171	4.99305809	4.95597927	4.96766011	4.34654872	4.50708578	3.92188197	4.40134191	3.74005007
FAM192A	6.03410658	6.31930307	5.94325668	5.96062046	5.95726857	5.97960834	5.96160729	6.15735669	6.11958779	5.93456037	6.04764655	5.97144678	6.3510615
RAB43	2.28833589	2.3938638	2.27042463	1.43183641	1.99319991	1.7076399	1.80278349	2.3342516	0.49677135	3.93463635	0.60607848	3.27160892	2.78954101
RAB37	0.60863011	0.29934315	0.020416	0.08022392	-0.09348808	0.65042976	-0.03981814	0.12580499	0.0606365	0.0606365	0.0606365	0.0606365	0.0606365
DCP2	5.07861375	5.66536463	5.03959191	6.44853821	5.72144488	5.80314305	6.02665956	5.80414218	5.72703965	5.13461156	5.83943639	5.25028093	5.17334654
SNX32	1.56574885	0.37975137	0.61922989	0.14325893	0.48528054	0.99482313	0.658802	-0.08762827	-0.20255059	-0.20255059	-0.20255059	3.32335485	-0.20255059
RPL38	6.24081865	5.91178857	6.22060105	6.16803065	5.70572171	6.6399973	5.08539406	5.42057878	6.91263655	7.13839526	6.00415216	5.77211222	5.98373528
CYP7B1	3.26419014	4.41322769	2.30647846	4.94595402	2.98623644	3.920189	3.55886205	3.09447724	4.02739254	3.50233981	4.48708012	3.42963104	4.62785856
RARG	5.07993735	5.018118	5.26798922	4.08847631	4.62127555	4.2541946	4.90888608	4.93023127	4.92891015	5.25292703	5.22077408	4.73771781	5.00085492
CES4A	3.55743967	1.98380276	3.24516619	2.60567427	3.28747505	2.41794148	2.98162987	4.09417534	3.40469375	2.42259466	2.98163849	2.16670242	4.38376676
CES3	0.37114227	0.94466562	0.44150309	0.02020625	0.86910644	0.48136938	0.45042822	0.70050454	-0.14883306	-0.14883306	-0.14883306	2.06923748	2.29348932
SSH3	5.27772069	4.85404512	5.25495192	3.28947277	4.6944391	4.89526963	4.48135066	5.06053454	4.47453817	5.4469421	4.37663866	4.66440547	4.11690153
CES2	6.19161178	5.83047834	6.26182071	5.25037113	5.30578135	5.4598176	5.04848727	5.17162119	6.07823605	6.3284249	5.06493053	5.41983443	6.19138872
PDP2	5.21136148	5.38204805	4.76946653	4.13821875	4.81278818	5.75769023	5.32271469	5.16022967	4.75273232	4.12759702	5.75742026	4.40457645	4.55300459
SP3	6.15088389	6.67794665	5.82707543	6.96607104	6.85594615	7.0435655	6.67274611	6.92234538	6.97212151	6.62832246	6.9071218	6.81318113	6.23360229
DMXL1	4.7083235	5.57622362	4.93846662	5.76063069	5.78517609	5.89513236	5.84023039	5.78298108	5.57824758	5.38410928	5.81320657	5.37651214	5.92874044
METAP1D	3.31012552	3.44035325	2.76997759	3.5273199	3.6432619	2.88692487	4.79779105	2.89034762	2.89321493	3.16896903	4.5572941	3.35010691	3.86005992
ZNF621	6.13500142	6.42251295	5.93600523	6.29875616	6.3313078	5.79997669	6.17832597	6.35629099	6.04338827	6.42975725	6.42749593	5.93561886	6.05905634
EGFL7	0.84487854	4.45082147	3.42903053	3.94101526	4.76472686	1.22757671	3.68640945	3.26046786	3.86553088	5.02274668	4.23151802	4.37864733	4.90612241
NADSYN1	4.76383395	4.42588007	4.27796508	3.96884864	4.32890949	3.4608531	4.78417333	4.34059765	4.00860839	3.92465291	3.8005285	4.77306628	3.99136511
DHCR7	3.9373842	4.824806	3.62608005	3.67452031	3.8939717	3.67306281	3.56111694	3.56631845	3.25744664	4.09149244	3.98207653	4.07878173	2.93700493
NBEA	4.95646226	5.38649318	4.30757234	4.78318524	5.8305341	5.53244261	5.42598323	5.69552444	5.41857999	5.61464919	5.29434375	5.73581945	5.81271105
RNASEH2C	5.31373086	4.91670698	5.21674494	4.08273336	5.17137107	4.73218639	5.54260775	5.75485455	4.98717651	5.86452491	5.40111885	5.71180998	4.47610683
MYEOV	0.40570164	0.25164724	0.47018633	0.03000089	0.51920178	0.01103434	0.3294402	0.01287958	2.7258761	-0.09355909	-0.09355909	-0.09355909	-0.09355909
ANKRD13D	4.4793535	4.61969709	5.08417272	4.17846509	3.92689569	2.8635346	4.18337065	4.10188855	4.43230068	4.33160196	4.42783357	4.51036389	3.81927975
MRGPRF	4.04908363	4.87551455	4.38467989	1.6338613	5.24322927	5.13014378	4.99811879	5.96585554	5.39537779	6.73209889	5.82842564	6.47382199	4.49772714
MYD88	5.93190531	5.61580403	5.70966577	5.83602692	4.66276178	4.51672533	4.67617651	4.16187649	5.33208778	5.1473351	4.46802117	4.50901195	5.26794719
MRGPRD	0.38589749	0	0.30884502	0	0.08744593	0.15011969	0.08266354	0.12404746	0	0	0	0	0
OXSR1	5.83074822	6.1188983	5.74912874	6.36874281	6.49359909	6.16199436	6.33239924	6.49464606	5.87372528	5.68625655	5.81840247	6.17312074	6.28797832
SLC22A13	1.5971725	-0.42376609	1.05364364	0.50285647	0.42692004	0.03743433	1.38361676	1.36124764	1.62629223	-0.10263278	2.91423897	-0.10263278	-0.10263278
PHF8	6.20826286	5.52420204	5.9972128	5.22149204	5.73816063	5.4463437	5.593387	6.12489595	5.41601254	5.59750661	5.49814835	5.27381106	5.95794067
LCLAT1	4.86431729	5.63748724	5.20522076	5.43419755	5.85075558	6.46215554	5.94337893	5.58172467	5.35280127	4.87331612	5.52023545	5.61736065	5.14396219
ADH6	0.41461602	0.30591557	0.56209495	0.31603427	0.3333952	0.5118986	0.73267697	0.53769842	-0.2522057	-0.2522057	-0.2522057	-0.2522057	-0.2522057

KAT5	5.72546527	5.68154512	5.86861316	4.95413313	5.51355342	5.81191889	5.23730055	5.47164475	5.55423374	6.25449425	5.22534345	5.83025242	5.46078368
SH3RF3	3.85682026	3.21982002	4.08351204	1.49009233	3.16081025	3.2718826	2.97759597	2.52427216	1.91787498	2.12500875	2.64775133	4.29662261	-0.66273196
GXYLT2	4.70581331	5.26515485	5.07636602	5.95635197	5.64150946	4.35762659	4.7112127	5.5758376	5.51743	5.89579704	5.76077998	5.76955977	5.05077838
HPSE2	4.64688589	3.02047797	4.44058076	2.68711512	5.33680852	7.87829171	6.01949944	6.07480123	3.69303844	3.26799466	7.61601328	4.78968041	5.41875477
DCAKD	5.48891645	4.3489447	5.14127363	4.21136245	4.43376441	5.28706644	4.33637809	4.29886543	4.56589533	5.25883541	5.01447388	4.80336149	3.68569236
ARPP21	3.8972452	4.94435258	5.63041988	2.55865759	5.30371681	3.91790631	4.20832941	3.50918643	4.99476852	3.93948669	5.06196803	4.67364859	3.24798486
TADA2B	5.74836711	5.5339026	5.58642656	4.58482993	5.20471714	5.20606362	5.28863376	5.10496996	4.87848458	5.96078675	5.34345907	5.2324231	5.57124522
CCDC96	1.66071043	0.44970852	1.75051004	0.43065867	0.87655941	1.11861089	0.51078264	0.88347033	0.62876441	-0.40481947	-0.40481947	3.57973776	-0.40481947
RELA	6.74555157	5.64618817	6.32992944	5.53493732	6.12949542	5.09761565	5.87257023	5.49128553	5.92959501	5.94684525	5.43679997	5.46595242	5.34294215
EVC2	5.60383206	4.63057323	5.3366519	4.67315711	5.35102856	4.96345024	5.03554485	5.5841557	4.89312506	4.70109505	5.4258516	4.44722578	5.31372046
ZNF680	4.35202531	5.35267661	4.52394481	5.55597849	5.4491837	6.4110178	6.11924374	5.83977462	6.21940694	3.53778016	5.7840083	5.7863433	4.47920056
BNC2	5.17695085	5.8604456	4.79089657	6.57461908	4.62172839	5.46926635	5.06486534	5.21421986	5.54044844	6.24190691	5.50314308	5.24940208	6.43212287
HPSE	3.21216903	2.98111471	3.48183988	4.42565237	2.59101867	3.89628288	3.49605493	3.92433825	3.13162063	1.13062385	2.16870484	3.87973856	3.53510515
COQ2	3.74104646	3.60923077	4.02786385	4.83674904	3.04610318	3.35371317	3.79822345	3.26759863	3.13116759	4.55348958	2.88136368	4.13241286	4.32687512
HSPA6	5.10607617	3.88537613	4.08849728	5.09690361	3.0552891	2.48756743	3.03940854	2.66534134	2.70162869	1.24161045	3.42020688	3.09926294	-0.16062914
TRMT112	5.06254413	4.99735187	5.39213639	4.92611842	4.63939981	5.54894754	4.57902838	4.05259915	5.2448847	5.76890421	4.96566395	5.63213529	4.12399956
LRRN3	3.23309183	2.72991336	2.55306206	3.41000397	2.60137889	2.63330165	1.95132794	2.74825594	4.22064214	-1.43409472	4.10422777	2.21324112	4.33000306
KDM2A	6.9248141	6.64416104	6.72524462	6.77056908	6.37039003	5.87379747	6.62857564	6.30500748	6.05787994	6.42825109	6.41677279	6.16127598	6.44375355
ADCK5	3.05061417	1.33112517	2.99668524	1.00859226	2.06305898	1.82318668	1.39579977	2.55866078	2.49210014	-0.6795768	3.41246559	4.01156181	1.68113563
NOC3L	3.65472101	5.03258112	4.12989423	5.69330038	5.25339418	5.6095926	4.92869436	5.51169903	5.1748273	3.05861119	4.77206964	4.40826385	4.29270264
ESRRA	4.16524875	3.66367112	4.84701682	3.89757684	3.22886174	2.49581342	3.33474699	2.83364999	3.89692566	4.43359876	0.52758703	3.02042301	4.17138609
RHOD	4.6363324	4.38880445	5.33397401	3.25539375	3.88943221	3.54009229	3.75105023	3.49096856	4.12320709	4.34881973	4.38496778	5.04083543	2.73626683
COMMD1	4.65785062	5.12842686	4.17533563	4.81350199	4.91255765	5.01016972	4.48681646	4.30880532	4.25424687	4.65986627	4.02729975	4.76138419	4.63765403
RAPH1	6.75218095	7.30052161	6.97709753	7.25092156	7.75481823	6.47422858	7.00518447	7.24268306	6.82999042	6.39834547	7.11651005	6.72854618	7.13271986
MTX1	2.71064261	2.50425311	3.73900328	2.35010481	2.2965913	3.24655318	2.82597747	3.28237945	3.06610176	2.6128397	2.4595966	2.78271136	3.51651534
ADCY5	6.7751752	6.96500058	6.92332359	4.67184167	6.93517693	6.8556568	7.20055452	6.75886313	6.35469733	7.72648727	6.99089027	7.24729186	7.16318055
PARP14	6.16432039	6.80552749	6.25665643	7.74042289	6.82475847	6.4839863	6.69927515	6.61055637	6.84107703	6.02562545	6.90507014	6.12322856	7.01052816
CYSLTR1	2.41401664	4.99244929	3.38805744	5.10861455	3.36902337	3.07068882	3.51574015	2.87983117	3.91454904	4.49023014	4.30904963	3.33579939	5.05017034
PARP15	0.54068877	1.96236288	-0.11596902	1.24548146	1.05867967	-0.53403142	-0.09053574	-0.16676908	0.21490771	0.21490771	0.21490771	0.21490771	0.21490771
CKS1B	3.97692291	3.96802396	3.27047231	3.60490563	1.42354385	2.68264148	2.64796577	1.64422904	1.08571777	3.72208289	2.92037562	3.4936803	-0.82604415
ABCD2	1.77241357	1.90658193	1.59528518	1.72368037	1.6328051	3.18394162	2.01027601	2.27604645	2.16832176	3.60769809	3.706515	3.52540619	4.0705607
AHSA2	5.91899156	5.80879767	5.845828	6.10784215	6.31072333	5.6055787	6.17133925	6.81282235	5.7336745	5.80183227	6.24650153	5.70320895	6.50839968
ABLIM3	6.17156248	5.80718309	6.47397396	5.42334971	5.99696438	6.00263773	4.98466821	5.52211742	6.3564545	5.32039724	6.25558624	5.22241506	6.5439695
VANGL1	4.90513853	4.71182821	5.29040239	4.69323982	5.35593092	5.19125017	5.13126039	5.6285693	5.26053389	5.40408003	5.5873733	5.55046112	5.42456327
GLRX	9.0073743	4.958129	6.89989661	6.61937489	4.59582353	4.10420347	5.07583176	4.43046101	3.98961315	3.75484916	4.03616304	2.85338453	4.94998514
IQCB1	4.49225877	4.95332017	4.12766913	5.03098199	5.17654997	5.45198595	4.94276316	5.09296965	5.3761613	4.78185937	4.33235279	4.52458203	4.56971633
SYT12	2.66834963	4.47746262	4.18097818	2.31251345	3.8254142	2.3516344	1.35407855	1.93439439	3.51306679	-1.30055624	5.06074961	2.84592573	3.53317818
GOLGB1	7.09270659	7.8240591	7.36444036	7.80253308	7.76340286	8.33159494	7.49949344	7.65741463	8.23422207	7.65718457	7.0378527	7.44207326	7.80598967
LIPM	0.02718094	0.7677984	0.02952057	0.06545435	0.02718094	0.02848174	0.02718094	0.03016576	0.00077192	0.00077192	0.00077192	0.00077192	0.00077192
ZNF483	5.24711688	6.62897319	5.53480112	4.80426573	6.13382538	6.39228775	6.31463957	5.94889339	6.20576764	6.33891027	6.27009308	6.58017128	6.03427456
PLAC8L1	1.09439312	0.19930937	1.74794827	1.93899362	2.43146965	2.27326143	2.56166267	1.03295614	-0.63460222	3.02523338	3.68598159	-0.63460222	2.88721635
GPR137	5.03415406	4.08768798	5.32628521	3.795097	3.75835073	3.92782255	4.10049253	3.60377843	3.8456719	4.07876635	4.06809284	4.48647078	2.97095138
SNCG	5.6123401	6.83632859	6.12250232	5.22786637	5.3954481	5.99722474	3.97856651	3.04008942	5.01069229	4.11216547	4.67169508	3.65187462	4.3739788
MMRN2	4.41170465	5.99675374	5.09989905	5.98014878	5.56083676	4.05805957	5.23498249	4.91267508	4.99609029	4.73241301	5.97938452	4.406343	6.18584684
MZT2A	3.30069399	2.69487824	3.81609942	2.70310503	2.0693273	3.12544566	2.67518242	2.8850754	3.36122847	-1.07415478	3.17789095	2.84953276	3.72072655
TNKS	5.17101986	5.47695389	4.58170257	5.46263764	5.3599472	5.42595638	5.34060557	5.34163733	4.76329122	4.55331585	5.35326037	5.15947799	5.48003775
ZNF449	4.4207134	5.0471517	4.66182184	5.76924429	5.60472226	5.49010044	4.879751	5.69668651	5.50699204	4.81890353	4.76986285	5.47588811	4.85848262

PPP1R3B	7.20900146	6.89217999	7.00284774	6.05532664	7.44676215	7.1827918	7.35013494	7.31575431	6.95397812	6.94049833	7.20613972	7.24501576	6.78439575
STOX2	3.78252747	4.1658054	3.70031766	3.37913981	4.53982057	5.77177389	4.30451899	5.35844793	4.75754002	5.23287382	4.68402074	4.77849234	4.81095918
MAP3K11	4.70918727	4.9197951	5.23708855	4.24376598	4.21415086	3.19102172	4.23177914	4.03759194	4.34071084	4.51984633	4.87620547	4.69300968	2.63413455
TRIB1	5.97000385	5.49267667	5.50442179	6.26067486	4.33336938	4.61124726	3.83466161	4.52521795	4.43648825	3.72914277	4.1161012	4.48612831	5.77789739
KCNK7	0.79503182	-0.09171544	0.65349553	0.48124937	0.59272995	0.2403057	0.59685273	0.77646715	-0.06645794	3.56756192	-0.06645794	-0.06645794	1.77663942
SFT2D3	3.92822393	4.13427326	3.98413782	3.72026827	3.92983401	4.10172067	3.95445227	4.66563087	4.26976338	3.77076934	4.66646876	4.17208098	3.36233319
C1QB	7.29391539	5.85208431	5.44856466	6.04075379	4.87664756	5.81717469	4.72131888	4.06485566	6.29416031	6.11427254	4.03125433	4.23804114	5.87767386
C1QA	6.17445962	6.82479407	5.07090198	6.26366682	4.67228293	4.6158846	5.08421778	3.6301772	5.49510738	4.97710212	3.78833978	5.77579815	5.4023868
NDNF	2.85045316	2.3817071	1.91213856	1.59828783	3.75888871	3.41877321	2.81169877	3.09535343	2.8770569	-1.12780755	2.76008928	3.26167905	4.94995481
OLR1	1.77428717	2.79863954	3.15170902	7.25849277	0.60771819	0.63552135	0.92425461	1.25970089	-0.0136313	-1.67267406	-0.17230362	0.96993451	2.18874918
DAG1	6.71322464	7.01876591	6.96179707	5.63342956	6.78893126	7.01795737	6.70484371	7.01403839	6.35042235	7.3223064	6.80199646	7.03704639	6.81701357
DAB1	0.50063886	0.88396978	0.68472413	0.85691061	0.9549825	1.16361527	0.54300235	0.91112662	2.47340898	-0.32178485	-0.32178485	2.33904747	2.72613096
ARV1	3.86289534	4.47822097	3.66152683	4.21658855	4.64351789	4.80905559	4.23454791	4.23066223	4.50989184	4.61019705	4.49754178	4.53439714	4.19250313
NAA20	4.64820126	4.80140616	4.58745431	5.48939771	4.68876005	4.88713525	4.90170832	4.78337561	5.38375335	4.85110361	5.07450896	4.30212568	5.08263152
CCDC36	4.25509437	3.22903642	4.32781091	3.34629015	4.66638868	3.77298341	2.92947741	4.31631036	4.12666543	3.44134737	4.44221219	3.22663587	2.6121744
SAA1	5.27643523	1.5761561	1.94627653	3.04234391	1.26970385	4.49527559	3.91855519	1.34952828	3.48224219	-2.11614789	4.69946127	1.28911739	3.58930073
MINOS1	1.15496282	0.53971196	0.53177118	0.28875453	1.04460174	0.98660884	1.57689384	1.31618355	0.20409174	0.20409174	1.58942396	0.20409174	0.20409174
EHBP1L1	7.1201608	6.87883657	7.70101034	6.28318779	7.13379846	6.47524626	7.10678233	7.08414104	6.9496049	7.39990966	7.26490052	6.9984229	7.27892163
THAP2	2.83998275	3.02376712	2.98496936	3.02520787	3.52132599	4.12550841	4.24041139	3.68348228	4.36561062	3.42764368	3.90070572	3.00262232	2.69539572
RNF26	5.19097466	5.0939875	5.52189522	4.86850439	4.66548198	4.56254959	4.58543728	4.35385704	4.10561824	5.34175218	5.04252665	5.11135427	4.90850098
PPP1R14B	5.04036691	3.34928154	6.02689389	4.49143294	4.24739343	4.8217461	3.60349916	3.67451509	5.04513473	5.29708547	3.7103824	3.59757711	4.30251339
SSSCA1	3.71042456	3.55936538	3.86091435	3.19690184	3.06701689	3.73138778	3.36315236	2.54840903	3.07967233	3.46591492	2.57646371	3.93323023	1.19839564
SMARCC1	6.93264445	6.79149183	7.11184517	7.35846696	6.52408385	6.41887179	7.1237811	6.38944541	6.4450367	6.26074765	6.62837414	6.40295842	6.39468917
ZNF417	4.88631093	5.04164972	4.88249962	5.44467001	5.67905603	5.16675925	5.24664484	5.27781062	5.06875352	5.47300095	5.33638958	5.48303287	5.25582777
PTPRM	4.06051705	4.04685411	3.5539613	3.97268234	4.82169202	4.87689272	4.33651222	5.01111054	4.21412651	4.41270325	4.98436648	4.95758318	4.64903372
FKBP2	5.19573282	4.28507693	6.13610426	4.5161378	4.46055402	4.86896797	4.53402007	4.07920275	4.76509341	4.57543988	4.84396878	3.67323424	4.85373697
VEGFB	5.77769651	5.93465947	5.87089377	4.25431463	5.66143002	5.30716079	5.42155472	5.77476395	5.91367129	6.61835959	5.66360976	6.23100319	5.5355159
PEAK1	6.80969311	7.04003321	6.47460103	7.32658736	7.51793247	6.71940704	6.76362292	7.13155239	7.101116	6.43659823	7.33678763	6.91488259	7.68380731
TNFRSF10D	6.56840612	4.952122	6.09268382	5.60663911	4.45629352	4.35228709	5.06310064	4.35786656	3.95787486	4.07328571	4.4134878	3.88707619	4.15981362
MST1	3.13807806	2.7560381	2.82005424	2.23359005	1.71546917	1.81359826	2.32408978	2.07954063	2.61446192	-1.18454208	3.53344375	2.02505106	2.09207309
TNFRSF10C	4.65311622	3.07253916	3.83095368	3.80042372	3.55518363	3.05995227	3.07185201	2.29782109	3.81446846	3.30023579	4.07544404	2.09852594	4.14399893
GMPPB	3.65018434	3.92274393	4.69863482	3.80331988	3.22966482	3.25141974	3.25922017	3.21265523	3.08030817	3.94809669	4.30506802	3.78155782	2.20531892
MOB1B	4.71369382	5.7062176	5.19939802	6.10261146	5.94825518	6.03908396	6.06711689	5.89695671	5.96106451	4.74739361	6.40474377	5.97947371	4.95945148
ZNF622	5.53884228	5.28253464	5.90616525	5.12371084	4.37866752	5.40788988	4.80454639	4.57192496	5.4898469	5.29398672	5.16169755	5.00330186	4.21171284
CSPG4	7.00169498	7.1567931	8.27483487	6.26093093	7.85674302	7.21349811	7.48788567	7.26897824	6.74691826	7.8756532	7.71755907	7.92977685	6.89040503
SNX33	6.85187	6.04950597	6.16874918	4.90845028	6.0296132	6.12340586	6.22476346	6.30621877	5.59132984	6.35326004	6.1162656	6.00323005	5.5564372
CHD2	5.05446897	5.07953953	4.57655387	5.22790705	5.17782363	4.59359429	4.46044598	5.01783684	5.20349191	5.33046231	5.27419125	4.62114809	4.9789562
XCR1	1.03914979	0.18889378	0.48352295	0.95538133	0.2269809	0.19029496	0.18889378	0.19210892	-0.2215279	-0.2215279	3.6847259	-0.2215279	-0.2215279
CCDC106	3.76000969	3.33082665	4.36538733	2.58872569	2.8825982	3.27569079	3.16427187	3.02160259	2.67519222	3.86858791	2.27299021	3.41573717	3.27765235
SULT1B1	1.40936511	1.8178595	1.76120187	2.75848713	1.766005	1.52201868	2.40861595	1.81832067	2.97095432	-1.02254608	-1.02254608	3.40256997	1.12897734
NUDT4	3.53010023	3.67796431	3.11190636	3.86007686	4.48890193	4.84856068	4.68521125	4.79469468	3.2409699	3.993601	4.02533024	5.24548643	3.352631
PC	3.96853198	2.60370105	4.37879874	2.68185894	2.69189825	2.58649183	2.82210153	1.99980824	3.64894945	2.65454735	4.04551434	3.66300336	2.24631955
SCAI	3.97187957	4.28997308	4.09517749	4.36824597	4.98650961	4.9237189	5.04693381	5.24995732	4.88465181	3.38254888	5.17025626	5.15831988	4.46006473
NMNAT1	2.95905548	2.37097373	3.52999576	2.9695797	2.26404347	2.66715051	2.51445268	2.51648094	1.33621146	3.82712525	2.66880039	2.63611229	2.82946257
LRFN4	1.68470536	2.97824845	4.22478777	1.69050109	1.76384081	2.02909649	1.09350523	1.4097314	2.84239308	3.65399895	-0.48787156	4.41918244	1.87263686
SLC19A1	2.61601255	0.61832985	3.52989615	2.3199786	0.5639836	0.50188669	1.57262194	0.49813491	3.01939564	-0.60982194	2.34385383	2.44210961	3.27384089
HSPB7	7.71010234	7.85661698	7.83935225	5.83101265	8.76051914	8.71734171	8.32729315	8.72629787	7.22932753	8.6784688	7.86731063	8.29250994	8.66299285

RCE1	3.79812989	2.52153392	4.09783763	2.6656965	2.8474636	2.51614319	2.84846386	2.32353591	2.19851376	3.59035838	3.23964926	3.53504532	3.63676282
UQCRH	5.15251897	4.77638705	5.05546432	6.10544625	5.15525669	4.98645677	4.97722836	5.04172874	5.66159327	6.19561391	4.5601428	4.88230257	6.0635305
TAS1R1	0.77304203	0.05891006	0.75757333	0.62161491	0.49188689	0.06033618	0.23007046	0.25073516	-0.38593832	-0.38593832	-0.38593832	-0.38593832	-0.38593832
EIF1AX	4.76543808	5.40242928	5.14843179	6.47854782	6.39602612	6.77397571	6.51918617	6.66114948	6.51106864	5.02859496	6.69817396	6.69033334	3.95254372
PSMD1	5.72022716	6.3138076	5.76316431	6.85399203	6.25609659	6.49083081	6.2877264	5.89481774	6.27081125	6.61850537	6.41055395	6.46437302	6.45107674
MUC13	0.13330449	0.13330449	0.34105412	0.17521303	0.45054753	0.62179103	0.24420976	0.45908146	2.52072085	-0.143332	-0.143332	1.25477472	-0.143332
SUSD5	8.54559827	8.62275805	8.92527035	6.93999751	9.15335375	9.28593156	8.73417502	9.11273293	9.05162236	8.84322974	9.27172572	9.29008138	8.38726357
HEG1	6.35985235	8.13696457	6.19456328	7.63258009	6.78307718	6.41718188	7.18673556	6.97854241	7.18923346	7.25184836	7.61662198	7.25618839	8.36563624
WFIKKN2	0.55260803	0.53822472	0.7619358	0.18260093	0.20272867	0.52921528	0.26866243	0.23427244	3.31184732	-0.20746223	-0.20746223	3.07683751	-0.20746223
C11orf80	2.12633636	3.2611776	3.34844893	2.57742463	3.47027932	1.37305914	3.67940623	3.03889609	2.99244437	3.34078228	3.58495237	2.80227677	-0.76851807
TOMM20	6.1711937	7.05667039	6.48481574	7.24759417	7.4647033	7.36032221	7.32252747	7.33310852	7.12534723	7.05399815	7.55863379	7.36728339	6.90546815
AGFG1	5.78946828	6.21188156	5.4912669	6.68682759	6.29885061	6.22461983	6.42413833	6.65365102	5.82717154	5.47540338	6.13447983	6.11333211	5.37725985
STAT5B	6.93215941	6.64598092	6.47328002	5.62819424	6.5406832	6.08507692	6.56318838	6.5946605	6.08011867	6.47020456	6.59923748	6.70357737	6.71239045
CD7	2.08332555	3.30380607	0.639317	0.75899936	0.78786039	0.16820447	0.5727412	0.38314414	3.00369968	-0.35262463	-0.35262463	-0.35262463	-0.35262463
CNP	6.13153796	5.77542268	6.2423031	5.38674538	5.23475768	5.23079457	5.33506026	5.33506026	5.53754691	6.1657601	5.4851923	5.70959728	5.6727351
JUP	6.01812508	5.39797351	6.11298767	4.73870412	4.93182755	6.37743987	5.23930245	4.98252539	5.64911529	6.2207132	6.30562699	5.74099209	5.97342486
HAP1	-0.00382719	0.15186948	0.27199803	-0.01390027	0.10142429	-0.01247512	0.26913374	-0.01063012	-0.11184378	-0.11184378	-0.11184378	-0.11184378	-0.11184378
TDRD12	0.29059781	0.05516989	0.27538883	0.05516989	0.64484746	0.8645114	0.44690014	0.3020467	-0.07936276	-0.07936276	3.12824312	-0.07936276	-0.07936276
EIF1	7.14154933	7.84211369	6.74752159	7.68185811	7.77219644	7.50469666	7.72835631	7.70753936	7.45963722	7.49098606	7.32380717	7.45302224	7.80436021
ENDOV	2.12136279	0.44949029	1.97125449	1.24072633	1.55622261	0.17896991	1.65797102	1.88006436	0.1156132	0.1156132	1.99276531	1.75162923	3.2627113
RNF213	7.47005228	7.50795488	7.49169576	7.69323227	7.13090219	6.47618785	7.54271424	6.83285798	7.06941711	6.8590494	7.82972205	6.68068612	7.80231937
TIGD3	0.48911916	0.26583006	0.18258487	0.01609623	0.28455214	0.01752871	0.26050382	0.01938319	0.49712253	-0.17042867	-0.17042867	-0.17042867	-0.17042867
PLK3	5.19284507	4.91018894	5.58129135	4.90974818	4.13309242	3.88422157	4.25433202	4.7707801	4.89822652	4.41201421	5.3417101	4.95095739	4.46599747
NET1	8.34673039	7.78648679	7.68039636	7.31210322	7.95194072	8.95522153	8.39717547	7.95378645	8.76321365	8.284519	8.41057961	7.93437051	7.56646891
DPY19L1	4.83312195	4.70541256	4.76656846	5.77910909	5.18447669	5.61886164	5.58077214	5.69031121	5.12188675	5.32428022	5.05947195	5.27089799	4.87661875
PHOSPHO1	1.31652846	0.03269275	0.85500928	0.05423089	0.64187558	0.03401917	0.43352033	0.03573636	-0.22380268	-0.22380268	-0.22380268	-0.22380268	1.5102904
ZNF791	5.51406444	5.62720953	5.64032994	5.48716979	6.0115537	6.15814592	5.93289737	6.03667712	6.41895814	5.9438825	5.61236289	5.86230337	6.03522158
PHC3	7.39460827	7.42342414	7.03198759	7.15492382	7.84408256	7.83979821	7.81263439	7.79375469	7.52909879	7.28336294	7.64694639	7.46922176	7.26117718
GPR160	0.36403918	0.24715851	-0.04082325	2.20818458	1.03494178	0.48714773	-0.15020599	0.18745357	-0.30044781	-0.30044781	-0.30044781	2.53574165	-0.30044781
CBX2	0.30390623	1.2482638	1.0652163	1.10583276	0.92404624	0.91109895	0.64287816	0.57940875	3.02206549	3.2230104	2.31460087	1.9643481	-0.46325095
SPTBN2	2.09726759	2.29238635	2.26290146	2.19444588	1.7771057	1.84498035	2.07981235	1.89677826	2.51960101	2.03536945	2.15835512	2.5611507	3.1274968
GOLIM4	6.06379244	5.71561985	6.35771506	6.55429615	6.03315649	6.71610892	5.86574554	6.28025652	6.96663085	6.68339029	6.14729821	6.38875353	6.65087733
RBM4B	4.44038981	3.62035889	3.55287357	4.26250997	4.39141092	4.65838386	4.39740189	4.05750181	4.18056992	4.70851689	4.05144663	4.11041384	4.3933781
USMG5	4.78627151	5.5169094	4.8345856	5.80721339	5.83458491	5.96027567	5.69904687	5.64585811	6.07460744	5.91271573	5.59369545	5.79176514	5.93990873
HOXB2	3.65493127	4.18029599	4.34382673	4.0782102	3.35682206	5.38165967	3.11198867	3.06742203	4.21557768	3.57413355	4.88373959	2.78271618	4.61922857
C1QTNF1	8.45629516	7.20812794	7.42147165	6.19037028	6.06055324	7.30753305	6.5188271	5.17441643	5.47339348	6.40258228	5.7786837	5.67818585	5.089639
SLCO4C1	1.05400273	1.45625094	1.16603642	1.55151725	1.50969852	1.71679958	1.15036536	1.97099272	3.45622392	-0.81211157	2.77006377	4.18741063	1.65554683
RBM4	5.36404261	5.92009208	5.44651	5.74327872	5.89605043	5.26623298	6.10503942	6.14639388	5.20488049	5.74294074	5.76967715	5.6538505	5.3172404
XXYLT1	3.8322481	3.88596004	4.24266014	3.88596004	3.60285415	3.18350214	3.01697173	3.82953729	3.94105672	4.87705162	4.30701863	4.43103274	4.4544298
UBXN2A	1.75308809	3.06936916	2.3392745	2.1262977	2.90863705	2.44879891	3.26044428	3.20186813	3.60934972	3.38635342	2.53037909	3.49824616	3.33293156
TCAP	2.1850491	1.87565968	2.61775946	1.18781375	1.75693836	3.10835347	1.47638517	0.74417613	2.048937	3.80890848	0.58281971	4.18394923	7.2268664
CCS	4.88985761	4.3832755	5.01140386	4.28670621	4.39328071	3.90864037	4.1633877	3.92121289	4.30890674	4.93679899	4.49339549	4.25432189	4.68309883
CEP19	2.90236343	3.35679324	2.59036101	2.80485172	2.24420624	3.43167139	3.34565219	2.16439419	3.82266154	2.40111792	3.64569641	3.01910697	1.91123714
KLHL15	4.55668447	4.73987186	4.38504981	5.00991728	4.77035228	5.68542133	4.98975259	5.38422221	4.66918328	4.26007026	4.17979379	5.64200204	4.93878968
GNG5	4.73306481	3.78566829	5.27725294	5.23304349	4.72852403	4.9135129	4.05496998	4.33800595	5.73890285	4.4631716	4.74936084	3.88333017	5.54403596
SLC25A30	5.67322627	5.68393259	5.06261179	5.28471254	5.75419981	6.22885769	6.11653575	5.32118294	5.93862661	5.34983998	6.08589016	5.16120878	6.03025937
CD34	6.27379788	6.65402673	4.63601015	5.04017483	6.55122233	6.15833174	5.9519969	6.98426153	6.63299833	6.62943081	6.57566014	6.1360191	7.09917712

CTSF	6.8129686	6.77727056	5.8173099	5.22146377	5.5100307	6.37847882	5.95893706	6.19869327	6.29435857	6.75454609	5.95180498	6.74806597	6.56276877
MSRB3	5.43386133	6.20115345	5.11532593	5.25834542	6.50978938	6.33160346	6.51255439	6.65138498	5.98313066	6.46261413	6.13964532	6.42677811	5.76500711
LEMD3	5.18527305	4.93970326	4.94903289	5.48442856	5.61724169	6.03643782	5.9196638	5.94018639	5.43659504	5.61072617	5.14418223	5.66050995	4.40016439
C16orf91	3.5476735	3.72008872	4.81569228	2.90223238	2.71322117	3.79846526	2.93273634	2.75913255	4.05081712	4.34884131	3.5930514	4.37330024	2.48551685
TLR10	0.79391172	1.4582896	0.72238058	1.36125797	0.9210681	0.73516386	0.55486035	0.80613667	-0.50855991	-0.50855991	-0.50855991	-0.50855991	-0.50855991
TLR1	4.11117971	5.49122311	4.31260603	6.45890169	4.41621814	4.3486255	4.37124268	4.35298473	5.45128258	2.80360495	4.70120612	4.26600762	5.06818537
TLR6	3.91571636	3.94934114	3.58839928	4.65903521	2.85389695	3.20424879	3.57734939	3.03984686	4.50140693	3.48305553	2.85342013	3.16627189	3.98839852
FAM174A	4.61181902	3.8857252	3.89402608	4.2570425	4.35813944	5.38220549	4.65336339	4.65280535	4.58372381	4.26961321	4.44620247	4.5090471	3.41961913
RGMB	0.69318192	1.00093788	0.34952009	0.07082106	0.68508932	1.24183234	0.69735005	1.08138516	0.52725578	0.52725578	0.52725578	0.52725578	0.52725578
FAM53A	0.59813613	0.05568759	2.4088385	0.53238964	0.14924583	1.09609047	1.16728498	1.09047498	-0.21580357	-0.21580357	-0.21580357	2.56463393	-0.21580357
CYB561D1	4.83712401	4.92128511	5.08356295	3.95068701	4.40469565	4.5162025	5.24641252	4.63829846	4.26350034	4.67965055	4.96858992	4.90993139	4.42636418
ZDHHC24	2.91929761	1.19761044	2.6676681	1.31899615	1.76572318	1.09218161	1.94238616	1.99395196	2.08064539	4.48097823	3.19701285	-0.4107652	-0.4107652
SELP	1.56350569	3.83603481	3.10865557	1.91247808	2.29004262	0.99371112	3.16647796	2.44668138	4.56686736	2.98828904	4.93714444	3.9695122	5.78282337
CTU2	3.66245842	1.72107715	4.74668055	3.37285127	3.53205916	2.94785401	3.17511354	2.55938929	3.37994949	4.16638491	-1.09161103	4.09331517	1.84188601
MGA	5.19815185	5.52854707	5.238266	5.70600799	5.9003876	6.11866772	5.93720846	5.92194376	6.20338381	6.2841066	5.43855191	5.9555118	5.33571386
C12orf66	4.07238597	4.230139	4.23553196	3.99693864	3.69495991	3.6107445	3.65305307	3.55248226	4.25310555	3.08190486	3.67555927	3.72535407	4.23758422
SNX31	1.50221121	1.14326266	1.21316266	0.84735831	1.60873884	2.40313599	1.81510848	1.34162512	3.34000728	-0.4667694	-0.4667694	2.2636422	3.39631397
PIGG	5.29630183	5.4696704	5.38079957	5.11348512	5.4493449	4.37108122	5.41074302	5.46387281	4.88845763	4.42732849	5.72937152	5.33989685	4.66189775
PRPF8	8.17289343	7.77597676	7.45045788	7.4022016	7.67972816	7.71950719	7.5765201	7.58337345	7.39611748	7.87200899	7.56697573	7.7176932	7.75087508
ADCY6	5.45173518	5.99585855	6.14585846	4.87555744	5.51835914	5.19190327	6.18073713	5.66222866	5.19394606	5.44987346	6.37431382	5.56022321	5.98405923
REP15	3.07096715	1.88307071	3.21693269	2.93952554	3.02679943	2.9355453	4.28412879	3.60408212	2.08504063	3.73538974	3.06562578	3.07443824	3.30186888
PITPNA	6.71798981	5.96470352	6.06296496	5.84818715	6.00466461	5.78584179	6.21872943	5.87161563	6.0214948	6.17126344	5.74557217	5.55633412	6.46446591
DDX23	6.56157346	5.97147272	6.58036561	5.64123763	5.63904936	6.10193592	5.92255873	5.77908011	5.80755148	6.17155366	5.49416759	5.88489558	5.8127312
ZNF80	0.58709193	0.31482902	0.31741834	0.31482902	0.31482902	0.31626865	0.62637372	0.31813239	-0.03939022	-0.03939022	1.87070503	-0.03939022	1.64436028
ZNHIT2	2.12902526	1.34999379	3.83019511	1.00740683	2.06097175	1.5139733	1.44572194	1.20916141	-0.62773911	3.8516917	3.16773076	-0.62773911	-0.62773911
ZBTB4	7.47602938	7.30634848	7.24781615	6.20160543	7.27375689	6.965568	7.09526165	6.94335329	6.86599621	7.60138123	7.21046139	7.04579456	7.06976355
TNK1	3.32176328	3.12037493	1.86431948	1.18196076	2.65892224	2.33450418	1.9767373	2.72773018	2.93168684	3.87298716	1.71579646	2.51484847	2.86550901
ZHX3	5.97130322	6.21168615	5.99444822	5.29382561	6.58265199	6.36239255	6.37604473	6.72448022	5.72772327	5.54408326	6.587142	6.14152934	6.29986823
PHLDA3	4.59673554	5.04899255	4.88997349	3.53403981	4.06673892	3.85345174	5.04263718	3.20748694	3.80183861	5.04004425	4.72112369	5.17975789	2.87711721
SLC16A11	0.18826976	0.13348806	0.00124472	0.05160707	0.14712815	0.16030696	0.09013554	0.37704162	0	0	0	0	0
SLC16A13	1.10965189	0.17500364	1.12166935	0.96095806	0.84615562	-0.36239158	0.46060084	-0.29090306	-0.11869261	-0.11869261	-0.11869261	-0.11869261	-0.11869261
GLIS1	1.96983071	0.22377593	0.75130507	1.354333	1.0942482	1.10093788	2.23787139	1.4562489	-0.40674734	-0.40674734	3.40699641	2.35838299	1.53344424
PODN	3.20276655	5.44412149	2.6166754	2.29040133	5.54029609	2.78086049	3.56655997	4.01787247	4.32278133	5.61146456	5.05803527	4.78160466	4.45611083
C11orf45	3.27248718	3.76414044	3.8495429	3.26311878	2.66717401	3.93823212	3.16287089	2.77980184	3.77711939	4.24112541	3.73551863	4.15415465	2.72541005
EXO1	1.10199479	1.22414535	2.21302843	3.87838642	0.91406412	1.36608276	1.71981506	1.02793349	0.33118031	-0.85795687	0.20590807	0.8696505	2.85384614
RALGAPA1	6.10056757	6.46264288	6.01147402	6.46737876	6.91390117	6.93076162	6.75409974	7.05092921	6.60812582	6.48945058	6.76158732	6.75664055	6.44621049
LIG4	4.1017366	4.50318287	4.22875251	5.33120681	5.37110667	5.50074329	5.02344497	4.95499812	5.57496426	4.82112122	4.60487874	4.44096809	4.61915121
GTF2IRD2B	5.21861207	4.39617876	4.45105652	3.44260828	3.84667843	4.38130645	4.26860216	4.81468202	3.9988138	5.03417923	4.57318243	4.41525901	5.21707717
ABRA	1.58024689	1.5095584	1.72516263	1.5095584	2.16176248	2.33157256	2.11354951	2.24199862	3.84591539	-0.9964107	3.62679662	2.57393837	4.31670364
ATP2A2	8.21587621	7.9707749	7.99057428	7.69518039	8.44824688	8.69888836	8.91531649	8.75789982	7.61778016	8.27857844	8.10835292	8.35892503	9.28566352
ZWILCH	3.28170093	4.03984134	4.07282069	5.33902472	3.78737948	4.05865346	4.17566366	4.0767109	4.40407558	3.97611566	4.7263647	4.58774365	3.92560175
RPL4	8.52419025	8.82546315	7.98797933	8.69347539	8.08895768	8.16701476	8.12172521	7.97382338	8.44830193	8.78445067	8.25562411	8.60868859	8.24131238
SNAPC5	4.13679481	4.29180758	4.9800549	3.95595935	4.54551943	4.53973171	4.07725988	4.47851344	4.45069298	4.34852265	4.65526732	4.37068886	4.49646113
STARD6	0.18280692	-0.00478694	0.20648015	0.03798826	0.11326622	0.22241991	-0.00478694	0.71271836	-0.05784521	-0.05784521	-0.05784521	-0.05784521	-0.05784521
VWC2L	0.30089401	0.13087096	0.13347105	0.13588637	0.1767355	0.13231657	0.16613517	0.22644924	0.11516319	0.11516319	0.11516319	0.11516319	0.11516319
C12orf76	3.75541332	3.79259233	3.45616612	3.34328349	3.81364149	3.9516103	4.55677837	3.64105456	3.64327397	4.10725196	4.12754693	4.29622279	2.75953077
ZCCHC12	0.92599884	1.3579511	0.68441	0.88519574	0.72926113	0.96142246	0.68182374	0.6851232	2.73051476	3.66466834	-0.20570102	2.69664017	-0.20570102

CNTNAP2	0.00355681	0.33226683	0.00124472	0.05190119	0	0.07289476	0	0.00158797	0	0	0	0	0
GALNTL6	1.35426207	0.78620167	0.78852956	0.90421866	1.55242127	3.3102828	1.88765402	0.78917152	3.33225951	3.88673945	3.65206834	1.65622131	-0.5483145
BBS1	4.56547566	4.45449231	5.50141562	4.4256696	5.0630305	4.27636724	4.95929287	4.9858395	4.60766607	5.13119113	4.86355211	4.86268749	4.39208711
DENND4A	5.38883769	6.36425439	5.60629018	6.62680111	6.32906742	6.66441379	6.60901254	6.48019748	5.87883183	5.44006192	5.96742348	6.03072422	6.36836801
SLC26A9	0.77919888	1.0232113	0.68269472	1.19931316	0.62644298	1.35657056	0.62749335	0.66776343	2.70425042	-0.17353785	2.37536188	-0.17353785	5.28215362
PELI3	5.27339625	4.75475699	4.73349101	2.99154601	4.40082264	4.29013539	4.41974335	4.21523593	4.46527594	4.56512935	5.19667254	4.76599493	2.34429438
TMEM81	2.12194955	0.74756095	2.08931567	3.02521812	1.67803109	2.37678752	3.62186775	2.54383711	0.67839666	-0.82935671	2.11734938	0.79442269	3.04514198
MRPL11	4.55859983	4.38651571	5.2433062	4.21929518	4.04724861	4.70175467	4.39804907	4.04004215	5.02508201	4.68127548	4.03151881	4.14902011	5.0045451
IL20RB	3.17824496	2.14512616	2.61748685	2.13554781	2.12901399	2.4316422	2.13554781	2.28355747	-0.95373695	3.27408461	-0.95373695	2.86792112	3.51579418
AKIRIN1	5.51222786	6.3899333	5.07453398	5.98841399	6.00866214	5.92450095	5.7926986	5.31930031	5.72333922	5.1482855	5.98486565	5.88282871	5.83086068
NPAS4	0.02698116	-0.10333251	0.25622347	-0.08324067	2.64242631	1.36308371	0.90972237	1.83588314	2.86203322	-0.30019009	2.93425471	2.59992021	-0.30019009
MSL2	5.88522958	5.89603086	5.7882192	5.78365526	6.28428419	6.60585239	6.3063106	6.30200519	6.12404626	5.67106713	6.14697159	6.08207955	6.12934442
ZNF497	0.7516909	0.21650255	0.31618745	0.12724426	0.27054682	0.35343633	0.57872535	0.12102794	-0.25529613	-0.25529613	-0.25529613	-0.25529613	-0.25529613
TRAM1L1	1.86953505	2.01334947	1.46911459	2.52604126	2.53713976	3.73341284	2.22345593	2.64718385	2.26891863	3.26530283	3.05476481	3.4522305	2.37788471
CMKLR1	6.9402416	7.51316247	7.2488029	6.98315441	6.52659089	6.39923104	6.09601813	5.44809236	6.54802071	6.79487559	7.59276746	6.33494019	6.61890008
ANGEL2	4.6677423	5.22425175	4.51059083	5.12386523	5.27885169	5.26339581	5.42502662	5.53868041	5.02541037	4.57037566	5.54450957	5.22338814	4.78627195
UGT8	0.50416786	0.3761634	0.49946882	0.31951581	0.45456597	1.60477384	1.13839582	0.75187311	-0.20410706	3.5422996	-0.20410706	-0.20410706	-0.20410706
KY	2.00994757	2.20367262	3.64747969	3.35039668	1.42655032	2.9052358	1.56901957	0.85042338	3.42840447	2.28231369	3.05282074	3.58493005	3.98012154
IQCK	3.77475053	3.98696106	3.45902528	4.48237899	3.69657964	3.58427351	3.13147895	3.87906777	3.41840118	2.60899348	3.87548598	3.39564987	4.26858499
SLCO2A1	0.52332498	3.34898398	0.71723465	2.62569628	4.00129834	2.90615102	4.99984969	3.9460945	4.14520777	5.62749034	5.8864196	3.05208292	6.56825641
ZNF266	4.77164082	5.5974068	5.08478149	4.52598461	5.26888049	5.07136138	5.42073572	5.89593492	5.07046029	5.69235192	5.50262812	5.78014195	5.30710942
SLC29A2	1.57096896	1.14847707	-0.14280505	-0.13294387	2.67211327	0.83233351	2.59640108	1.75367751	2.74876708	-0.53716244	-0.53716244	2.0436021	-0.53716244
BRSK2	0.56074877	0.04016258	0.25565002	0.04016258	0.68690758	0.21240663	0.34560131	0.8166743	-0.29836342	-0.29836342	-0.29836342	-0.29836342	-0.29836342
TMEM167A	5.13279045	6.30082438	5.35996892	7.18519531	6.54476785	6.60859053	6.64243602	6.65896953	6.73680887	5.52361191	6.95260853	6.31528224	5.465182
LEP	2.88257784	1.71377334	2.1822468	1.69194031	1.34263984	1.74469097	1.29586193	1.36406618	2.90324599	2.78817722	-0.91856637	3.81618724	2.79633633
SH3PXD2B	6.1306977	5.6348321	6.24010104	6.82187149	5.97625801	5.28220725	5.45527707	5.67987501	5.498723	5.36472653	6.24186093	5.57146391	5.8419919
LARP7	4.74506024	5.25996467	5.306475	5.90216111	5.40584351	6.32671683	5.3790433	5.19283229	6.51852197	6.04675169	5.07151599	4.95532013	5.60529324
FGFBP3	1.99067831	2.4398672	1.67511758	1.40744215	2.54563342	1.51682202	2.09410411	1.94522316	2.92928921	-0.79921553	2.63509888	3.35428179	2.53481452
NR1D2	5.52207367	7.2440994	5.72024462	5.66949273	6.42068102	7.11103905	7.36986523	7.3985935	7.04590785	7.0019359	7.32453767	7.76311052	6.98153469
PABPC5	4.2573725	3.45400289	3.38410351	2.9686735	3.74488377	4.91325723	4.10561797	4.05487895	4.24561645	4.69433787	4.30582333	4.61101733	2.73272843
BRMS1	4.65676482	3.69688763	5.31981486	3.77975282	4.03784953	4.13063893	3.63317905	4.04505477	3.88181275	4.49018006	4.42059126	4.34426366	4.23026419
RPL15	8.22278216	8.52957494	7.40117367	8.45727666	8.51625127	8.42549524	8.16059828	8.28013105	8.53406457	8.6256351	8.52407246	8.65839989	8.05188432
HRAS	3.51948949	3.11577882	4.60810161	3.09042255	3.06259633	2.96652967	2.95468755	3.3555765	0	0	0	0	0
WDR49	1.9420816	3.11801671	2.35498668	2.00433068	2.56505305	3.26616265	2.6294249	2.49047309	3.43957683	4.23847523	3.69006727	2.54991049	3.57902468
SRP72	5.90220976	6.10615336	5.93266056	7.40249385	6.591885	6.59863322	6.49493731	6.39564669	6.51901464	5.99747521	5.65491139	6.03182925	6.09489253
PCP2	-0.08563335	0.95940079	-0.08390106	-0.08563335	-0.08563335	-0.08467022	-0.08563335	-0.08342335	-0.06043194	-0.06043194	-0.06043194	-0.06043194	-0.06043194
RIN1	2.59589158	2.32695053	3.60092665	2.62679037	1.40530001	1.40674851	1.89301098	1.30561249	2.34666833	3.0133746	1.93360264	2.59132176	-0.89520807
THAP6	4.72493434	4.66940048	4.58879053	5.1966216	5.19613197	5.71808376	4.8797235	5.24987663	5.68804545	4.80452989	5.62955464	4.75913504	4.20915821
CEP135	4.00656484	4.56221508	4.3828294	4.95455998	5.03508145	4.8384288	4.61225522	4.20174047	4.48171176	3.98350948	4.46035596	4.17177559	3.75490969
FZD4	7.22843013	7.71232973	6.92920774	6.85575552	7.19091345	6.76625059	8.18403464	7.41185087	7.54595407	7.51968984	7.95815021	7.14858869	7.59138703
CD248	4.1961595	4.79857621	3.53764092	6.21761218	3.97938429	3.72114801	3.76410792	3.42466075	3.77420859	4.400101	4.75954423	4.99740702	4.53024234
BTC	2.72258423	3.79178748	2.78040609	2.55915046	4.86738839	6.15310163	4.9420982	4.71372053	5.25651696	5.77298776	4.94515514	5.91042651	3.68520897
PDZK1	1.12762821	0.71214082	1.07327197	0.9408926	1.22024145	1.39562492	2.38481534	1.57870304	-0.48239187	-0.48239187	2.82396757	-0.48239187	2.62968024
PDE12	4.64592161	4.72987008	5.3379269	4.94845514	5.17505896	4.93224893	5.12955868	4.94131523	4.95794699	5.1863217	5.14003374	5.4362731	3.8947405
GLMN	2.59168577	3.64098018	3.18429822	4.74472632	4.07198304	4.16254003	4.50108134	4.34532205	4.39629804	2.41972842	4.8999808	3.9523022	2.63591963
DNAH12	0.6237186	0.66049917	0.77277577	0.91096796	0.75871408	0.96862452	0.79217374	0.58907617	-0.41171535	2.0099689	-0.41171535	-0.41171535	2.56053746
YIF1A	4.98179783	5.22640842	5.09261736	5.5418476	4.9839192	4.50883824	4.26058414	4.68236756	4.65881594	5.47356178	4.77225559	5.80994007	4.49630377

CNIH2	0.82235016	0.49953682	0.30672267	-0.33004299	0.52213809	0.60704705	0.40030753	0.12547367	2.44362678	-0.17637708	-0.17637708	1.83874389	-0.17637708
NLRP6	1.15699366	0.29280696	0.63425693	0.48422869	0.40331034	0.41898216	0.29280696	0.29607798	-0.30679752	-0.30679752	-0.30679752	-0.30679752	-0.30679752
NDUFA11	4.91345083	5.08613153	5.30096509	4.21614125	4.83765479	3.89368019	4.77137041	5.19827436	5.28194107	5.8344507	4.62200929	4.73891604	5.00142425
RSRC1	4.34484631	4.33154986	4.22459778	4.67611399	4.78331091	5.40148983	4.14385221	4.54738458	5.41867145	5.00324509	3.62707287	4.20112272	4.20893396
RAB1B	7.09643723	6.88514555	7.44686711	6.63590959	6.70610654	6.36266197	6.4358746	6.36433472	6.37017364	7.22306289	6.45866635	6.97094155	6.78050695
PTDSS2	3.62884141	3.14225671	3.76948476	2.69957247	2.84066924	2.38682444	3.2364103	2.78707741	0	0	0	0	0
C19orf70	3.22714607	4.36764692	4.1375313	2.58013166	3.27970571	3.79175516	2.91897009	3.21540651	2.90681813	4.18070071	3.02439574	3.69102386	0.65758339
C3orf33	3.32366586	2.59897558	2.62270869	1.95136637	3.20653802	3.20383764	3.70914563	2.99551851	3.40777347	1.46367934	2.94906997	2.18253594	3.01488011
SEZ6L2	2.34237981	2.62128019	1.87143757	3.35428647	0.83084479	0.64197036	1.16701569	1.37679106	3.47270844	2.50703221	2.81604219	3.36783114	-0.68007045
ASPHD1	0.34472071	1.57467934	0.77245755	0.85910277	-0.19031498	-0.26688632	-0.2681898	-0.26519884	0.35112439	-0.36959079	0.77798277	-0.36959079	-0.36959079
KCTD13	4.0066845	2.77830352	4.5852364	3.41421202	3.34249487	3.3250297	3.62474233	3.94531511	3.90441011	3.82652121	4.73412737	2.20404311	3.70812069
P2RY14	0.52453793	3.28834256	0.71658515	1.83580178	2.60699233	3.47644267	1.71255229	2.74531112	4.48956043	4.3582351	3.95955811	4.43229252	4.42025206
AMZ1	3.2821779	1.503963	3.14637573	1.61463973	2.89826157	1.23347378	1.5086973	1.86557215	-0.91876589	-0.91876589	2.22459929	2.69939919	2.65829177
GPR171	1.75221749	1.88652675	1.4604325	2.04671923	1.23672401	1.99761391	1.43606786	2.21623651	3.67518212	-0.76316618	-0.76316618	-0.76316618	2.14348493
FUT1	1.36253953	1.81143377	1.66645743	1.7506269	1.94187667	1.27651774	2.08509041	1.8210431	2.42474613	3.56045668	3.73701009	3.33703687	2.75023163
DHX36	5.21863747	5.64594432	5.46906956	6.1942578	6.2784817	6.21964751	6.25121918	6.41470872	6.07847446	5.1976256	6.06482036	5.88266397	5.2738274
FBXW8	4.26312563	3.94648558	3.96699932	4.52633027	4.07517104	3.50422379	3.88150986	4.11009922	4.58148579	4.09867357	4.05063891	4.30098718	4.56768419
KLC2	5.22246096	4.67622911	5.40207114	4.120911	5.22951042	4.87263598	4.93284988	4.63955347	4.83983304	5.07884124	4.89199981	5.33292691	4.82553117
SLC22A1	1.86117257	1.0745952	1.74741768	0.81972645	0.99924054	2.01211863	2.18845047	2.38928309	2.83622709	3.0332005	-0.58020511	2.43662442	2.97881777
CTBP2	6.06183778	6.20338917	6.08118944	5.71739972	6.38715529	6.2903878	6.05478736	6.3600172	6.34643749	6.39135454	6.18364177	5.74785908	6.10228731
CHST2	2.0211125	2.65681875	3.08806071	3.4031767	2.06828244	1.62685959	1.75453026	1.66156583	3.07431521	1.78247238	-1.23312586	-0.18452914	2.83943422
ZDHHC14	3.74916677	3.2009173	2.2267319	0.96578022	2.75254884	2.81280766	2.25168736	1.39554677	3.85665592	3.71870976	3.55954246	2.91554432	2.94724245
ATR	1.71273021	3.01705725	1.78785784	4.5336982	3.57868227	3.17078801	3.45398687	3.1685786	3.19248041	-0.71830025	3.3962904	3.91663113	2.61577689
UBE2C	1.32773759	0.76665514	3.7546556	3.84212193	-0.22012326	0.02869204	0.301656	0.49906174	2.7447059	2.97439763	-0.51684489	1.69886926	1.31088307
GK5	5.39071056	6.08785293	5.68396293	5.8275742	6.41596574	5.69476509	6.62982166	6.89113223	6.34756304	5.86901484	6.28128953	5.75167538	6.17778097
VCPIP1	5.78381353	6.27489882	5.79607728	6.20899559	6.46559505	6.4016321	6.28779454	6.3501456	6.30981464	6.3038153	6.33441007	6.45732561	6.43495515
RTP1	0.68599166	0.15230066	0.87148611	0.15230066	0.15230066	0.65659053	0.37633581	0.15555485	2.743835	-0.15395199	-0.15395199	-0.15395199	-0.15395199
DES	4.97017224	7.53961527	5.09743349	3.32989796	6.12052811	7.49776535	6.94966472	7.22274371	6.1633326	7.11734203	6.31078729	7.73751061	9.44898453
PDIK1L	3.39961384	4.02098432	3.89233175	4.4308292	4.33870185	5.39909312	4.79869176	4.9562443	5.12513632	3.96217182	5.02634626	4.73442991	3.96941706
SPSB4	0.30341371	0.45783667	0.35076464	0.42406474	0.60072416	1.66610597	0.24364856	1.47589026	2.46575438	-0.22221845	-0.22221845	3.82479158	1.31093997
TRAF6	6.05928338	6.08636957	5.30384702	5.69424348	6.44399614	6.33945662	6.13342209	6.26324388	5.70768612	5.44480002	5.91564868	5.75947373	6.38680149
ZNF654	1.46842631	2.72682975	1.4478765	3.39603226	3.59336631	4.15996664	3.82950537	3.71381356	3.29875143	-0.87405007	4.0244672	4.22706886	0.75802092
MRPS22	5.24206445	4.88991812	5.0355801	5.51553844	4.89456157	5.56824389	5.16105498	4.70654101	5.00168978	5.66924695	5.06728178	4.96700146	5.53014668
PACS1	6.81723397	6.39196929	6.92716095	6.20014967	6.39036956	5.54736844	6.39109828	6.16835652	6.02799448	6.7649687	6.73875718	6.47216474	6.59280623
MARCKSL1	3.51799027	4.34613383	3.64979572	5.7847417	3.37068823	1.6717244	2.97061467	1.92760784	2.48735676	3.68911188	3.86016584	3.70557096	4.59065957
SH3BP5L	5.08176721	5.05668271	5.40370509	4.84234968	4.90401047	4.5398958	4.91676903	4.65694916	4.82192617	5.12360029	4.76209287	5.10110144	4.78894182
YPEL2	4.73601809	5.15834392	5.04730944	4.20077936	5.27619691	5.15926202	5.2370343	5.74248139	5.88446051	6.28845337	4.68003111	4.59322003	4.73633126
CADM2	0.246861	0.08528053	0.08784126	0.08528053	0.18311025	0.27025753	0.4951276	0.57966774	-0.07402699	-0.07402699	-0.07402699	-0.07402699	-0.07402699
ABO	0.42817901	1.45485997	0.58344973	0.52333134	2.11997828	0.54011818	1.40309883	1.27166044	-0.22430261	-0.22430261	1.40630508	-0.22430261	3.85334833
PSMD2	6.95834754	6.64754848	7.03963073	6.94505123	6.29375342	6.34350928	6.60132607	6.15142007	6.61185962	6.62280543	6.78134852	6.67575541	7.08444076
FAM182B	3.15838932	1.53749824	2.37708582	1.06273663	1.26672853	1.55688227	1.79075201	2.77744385	2.20675512	-0.35499113	1.03604823	2.47694455	2.34608565
PPM1E	0.0045177	0	0.10877766	0.10080427	0	0.00069205	0.01463262	0.0546554	0	0	0	0	0
FAM131A	4.55228095	4.3742091	5.2706077	3.79421985	4.40281972	4.17849226	4.94063307	5.10275837	4.34037425	4.64059608	4.22261564	4.9521123	3.98545815
CSRP2	5.6836904	5.44876306	5.00050463	5.00066652	6.33010382	7.08584863	6.14682331	6.01273057	5.49316198	5.98791742	5.17896959	5.40287271	6.61246666
PARL	4.68264906	4.45840384	4.98406137	5.46048991	5.00273232	5.06751379	4.94216273	5.42406639	4.73595073	4.14662099	4.88751759	5.18055285	4.91232222
DDIT3	5.7166429	5.22579953	5.53018574	4.48109898	4.12017706	5.21738658	5.18606547	4.82839886	4.72893535	5.51321089	4.73793543	4.98280562	5.04033192
PCCA	3.81263998	4.13518099	3.30679774	4.30078625	3.87430598	4.19468656	4.67485961	4.1990197	4.12961643	3.76276069	3.48552998	4.21587453	4.6059145

DCTN2	6.106448	5.7658087	6.19931221	5.35350598	6.00133092	5.82747058	5.71997165	5.92535582	6.30850599	6.23721563	5.68004929	6.12608798	6.24556271
ZNF408	3.8764328	2.61553557	4.37988732	2.3808181	3.11946809	3.42411246	3.43059734	3.37966079	2.8212407	4.04355549	3.49888956	4.36414784	3.36163049
CTDSP2	7.34743487	7.20019263	7.11365552	6.9640083	6.86931406	6.54994206	7.57941075	7.02321347	6.74904795	6.99109548	6.94585466	7.12409799	6.68871342
CKAP5	5.36747529	5.34406189	5.588627	5.88942366	5.50338038	6.23212695	5.59104718	5.38238728	6.00770461	5.61499175	5.19717361	5.54122415	6.25254956
ARHGAP1	7.34338419	6.63073022	7.82744158	6.72053825	7.00009094	6.92822368	7.23055194	7.10270047	6.68687623	7.72927972	7.44711579	7.48705484	7.20149788
MED16	5.38199553	4.62380728	5.4480967	4.37663999	3.73206099	3.68116235	4.45126807	4.74879534	3.95553442	5.64534805	5.10376747	4.92284708	3.75234496
[truncated: 1,214,771 more chars]
